# Epigenome-wide association study of bronchopulmonary dysplasia in preterm infants: results from the discovery-BPD program

**DOI:** 10.1186/s13148-022-01272-0

**Published:** 2022-04-28

**Authors:** Xuting Wang, Hye-Youn Cho, Michelle R. Campbell, Vijayalakshmi Panduri, Silvina Coviello, Mauricio T. Caballero, Deepa Sambandan, Steven R. Kleeberger, Fernando P. Polack, Gaston Ofman, Douglas A. Bell

**Affiliations:** 1 Immunity, Inflammation and Disease Laboratory, National Institute of Environmental Health Sciences, National Institutes of Health, Building 101, MD C3-03, PO Box 12233, 111 TW Alexander Dr., Research Triangle Park, NC 27709 USA; 2 Epigenetics and Stem Cell Biology Laboratory, National Institute of Environmental Health Sciences, National Institutes of Health, Research Triangle Park, NC 27709 USA; 3 Fundación INFANT, Buenos Aires, Argentina; 4 Consejo Nacional de Investigaciones Científicas y Técnicas (CONICET), Buenos Aires, Argentina; 5 Department of Pediatrics, Vanderbilt University Medical Center, Nashville, TN 37232 USA; 6 Section of Neonatal-Perinatal Medicine, Center for Pregnancy and Newborn Research, University of Oklahoma Health Sciences Center, Oklahoma City, OK 73104 USA; 7 Present Address: The Golden LEAF Biomanufacturing Training and Education Center, North Carolina State University, Raleigh, NC 27606 USA

**Keywords:** Preterm infant, Cord blood, DNA methylation, Epigenome-wide association study, Gestational age, Stochastic epimutation, Nucleated red blood cell, cDNA microarray, Bronchopulmonary dysplasia, Lung

## Abstract

**Background:**

Bronchopulmonary dysplasia (BPD) is a lung disease in premature infants caused by therapeutic oxygen supplemental and characterized by impaired pulmonary development which persists into later life. While advances in neonatal care have improved survival rates of premature infants, cases of BPD have been increasing with limited therapeutic options for prevention and treatment. This study was designed to explore the relationship between gestational age (GA), birth weight, and estimated blood cell-type composition in premature infants and to elucidate early epigenetic biomarkers associated with BPD.

**Methods:**

Cord blood DNA from preterm neonates that went on to develop BPD (*n* = 14) or not (non-BPD, *n* = 93) was applied to Illumina 450 K methylation arrays. Blood cell-type compositions were estimated using DNA methylation profiles. Multivariable robust regression analysis elucidated CpGs associated with BPD risk. cDNA microarray analysis of cord blood RNA identified differentially expressed genes in neonates who later developed BPD.

**Results:**

The development of BPD and the need for oxygen supplementation were strongly associated with GA (BPD, *p* < 1.0E−04; O_2_ supplementation, *p* < 1.0E−09) and birth weight (BPD, *p* < 1.0E−02; O_2_ supplementation, *p* < 1.0E−07). The estimated nucleated red blood cell (NRBC) percent was negatively associated with birth weight and GA, positively associated with hypomethylation of the tobacco smoke exposure biomarker cg05575921, and high-NRBC blood samples displayed a hypomethylation profile. Epigenome-wide association study (EWAS) identified 38 (Bonferroni) and 275 (false discovery rate 1%) differentially methylated CpGs associated with BPD. BPD-associated CpGs in cord blood were enriched for lung maturation and hematopoiesis pathways. Stochastic epigenetic mutation burden at birth was significantly elevated among those who developed BPD (adjusted *p* = 0.02). Transcriptome changes in cord blood cells reflected cell cycle, development, and pulmonary disorder events in BPD.

**Conclusions:**

While results must be interpreted with caution because of the small size of this study, NRBC content strongly impacted DNA methylation profiles in preterm cord blood and EWAS analysis revealed potential insights into biological pathways involved in BPD pathogenesis.

**Graphical Abstract:**

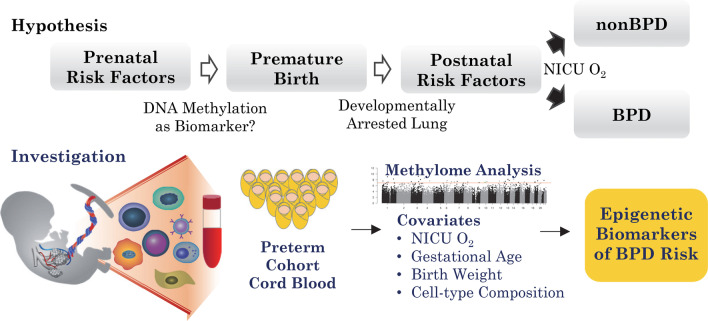

**Supplementary Information:**

The online version contains supplementary material available at 10.1186/s13148-022-01272-0.

## Background

Lungs of preterm infants born at 24–36 weeks of gestational age (GA) are undergoing late canalicular and saccular phase development including widening of distal airways to prepare for the subsequent formation of alveoli, differentiation of type 1 and 2 alveolar epithelial cells, and thinning of the air–blood barrier [1, 2]. Premature infants born during this GA frequently require oxygen (O_2_) supplementation in the neonatal intensive care unit (NICU) for an extended time. Bronchopulmonary dysplasia (BPD) is a leading cause of chronic respiratory morbidity among survivors of preterm birth with the greatest risk for those born at 23–30 weeks GA [3, 4]. BPD is diagnosed if an infant has a need for oxygen therapy to maintain blood oxygen level at 36 weeks postmenstrual age (PMA) for babies born before 32 weeks GA or at 56 postnatal days of age for babies born at or after 32 weeks GA [4]. The epidemiology of BPD continues to demonstrate that birth weight and GA are the most predictive risk factors for developing BPD, and the frequency of BPD has been approximately 40% in surviving infants born at ≤ 28 weeks GA and about 30% in infants with birth weight < 1000 g during the 20 years from 1993 to 2012 [5].

While knowledge about O_2_-induced BPD pathogenesis has significantly improved, the mechanisms that lead to the disease are not fully understood. Genetics, prenatal conditions, postnatal factors, and the interaction with O_2_ therapy are thought to drive disease development. Advances in supportive therapy including antenatal/postnatal corticosteroids and surfactant replacement have improved survival rate and greatly reduced the respiratory distress syndrome and prominent pathology including disruption of immature lung structures (e.g., diffuse airway damage, smooth-muscle hypertrophy), neutrophilic inflammation, and parenchymal fibrosis, sometimes referred to as ‘old’ BPD [6], in the patients. This ‘old’ BPD was observed in preterm infants who were exposed to aggressive mechanical ventilation and high concentration of O_2_ and has largely disappeared [6].

However, many extremely preterm and very low birth weight neonates who received O_2_ therapy had no apparent lung damage but displayed a new pattern of lung developmental disorder (‘new’ BPD) [7]. These ‘new’ BPD patients showed less, or were free, of the ‘old’ BPD pathology but displayed altered lung growth (i.e., arrested alveolarization with larger, simplified alveoli, disturbed pulmonary microvascular development) and an increase in elastic tissue proportional to the severity and duration of the respiratory disease before death [3, 7–9]. Development of the ‘new’ BPD is thought to be attributed to the prenatal exposure factors (e.g., chorioamnionitis, maternal smoking) and/or genetic/heritability factors critical for surfactant synthesis, vascular development, and inflammatory regulation [10–13]. The ‘new’ BPD lacks a definitive cure, and the persistent lung impairment leads to long-term pulmonary outcomes including functional abnormalities and increased risk for adverse respiratory symptoms (e.g., airway diseases, exercise intolerance) in BPD survivors [14–17].

During the last decade, genome-wide association (GWAS) analysis in different ethnic groups has revealed several potential genetic risk factors [13, 18–20] and transcriptomic studies have looked for biomarkers of susceptibility and early signs of pathology [21–25]. In addition, epigenome-wide DNA methylation studies have reported associations of epigenetic profiles with GA at birth (cord bloods) [26–31], with prematurity complications at discharge [32], and with BPD (autopsy lung tissues) [33]. Experimental studies have revealed DNA methylome-associated genes in BPD pathology [33–35]. These studies suggest that methylation profiles in cord blood might prove useful for detecting acquired risk factors, susceptibilities or nascent disease. However, prospectively determining the methylomic landscape of BPD pathogenesis in cord blood at birth is challenging because the developing fetal hematopoietic system is highly dynamic, with cell-type composition and DNA methylation strongly correlated with GA and/or birth weight [36].

Using DNA methylation analysis of cord blood DNA, we investigated association of GA and birth weight with the estimated distribution of cord blood cell types, particularly nucleated red blood cells (NRBCs), in a pilot-size cohort of preterm infants with or without BPD. We describe changes in methylation-based estimates of blood cell-type composition in relation to GA and birth weight. After adjusting for covariates (GA, birth weight, cell-type proportions, etc.), we identify differentially methylated CpGs and genes associated with the BPD epigenome. Furthermore, we also determine stochastic epigenetic mutation (SEM) load relative to BPD and examine methylation-based estimates of epigenetic GA (EGA) in BPD.

## Results

### Demographics and association with clinical outcomes

**Fig. 1:**
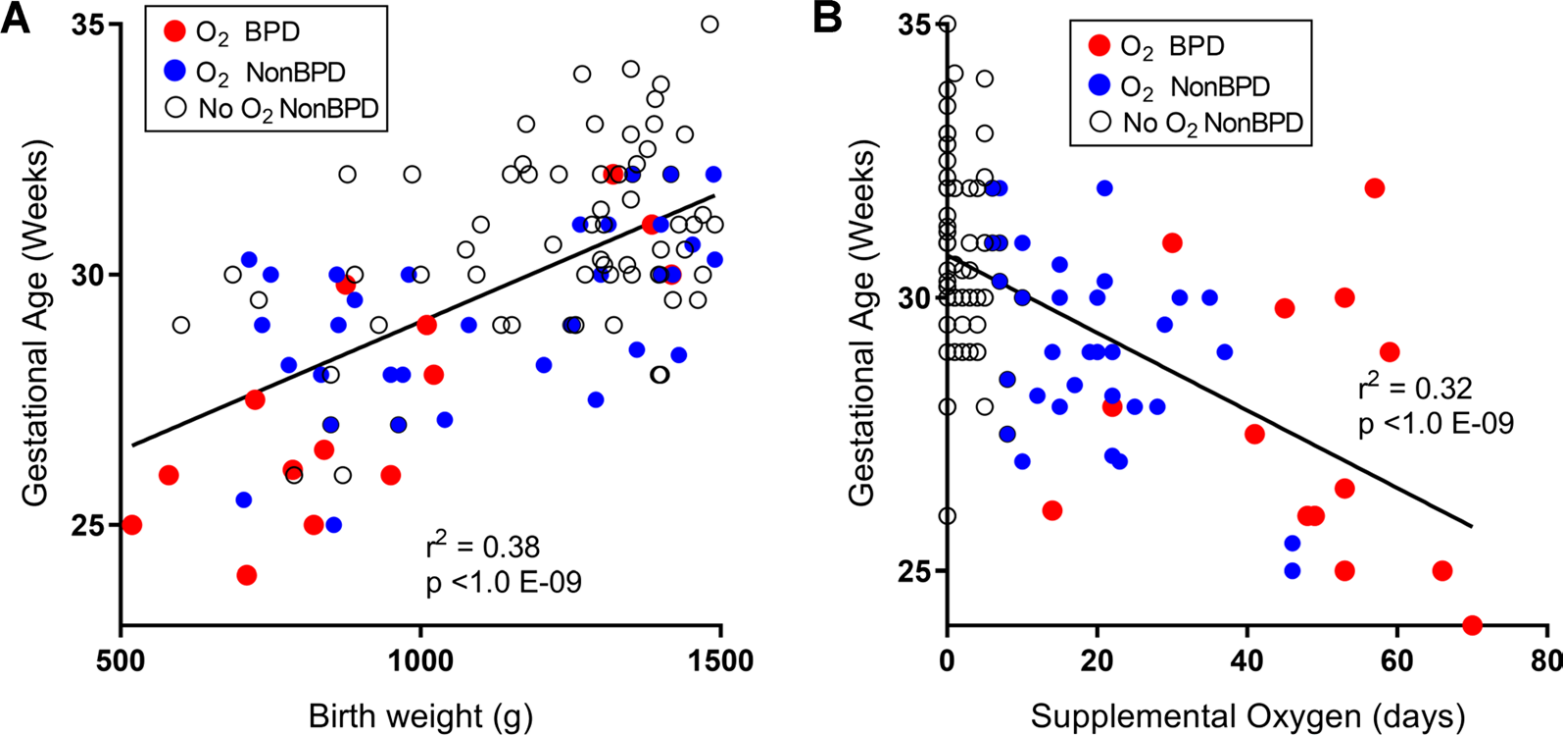
Association between birth weight, gestational age and oxygen supplementation in preterm neonates. **A** There was a significant correlation between gestational age and birth weight among all preterm infants (*n* = 107). BPD neonates had significantly lower birth weight and gestation age compared to non-BPD. **B** An inverse association was observed between days of supplemental oxygen (O_2_) therapy in newborn intensive care unit (NICU) and gestational age in preterm infants. Infants with BPD had more days of O_2_ supplementation regardless of gestational age compared to non-BPD. Red circles = BPD (*n* = 14), blue circles = O_2_ supplementation ≥ 5 days and non-BPD (*n* = 41). Open circles indicate preterm non-BPD neonates with 1–4 days (*n* = 21) or with zero (*n* = 31) supplementation of O_2_

After applying exclusion criteria, a total of 107 premature infants with methylation data were included in the study. Diagnosis of BPD was made for 14 preterm infants (13%) based on the NIH consensus definition of BPD (see Methods) and O_2_ needs at either 36 weeks PMA or at 28–56 days of postnatal life based on GA [4]. Table 1 and Additional file 1: Table S1 present the maternal characteristics, fetal complications, and outcomes of the prematurely born neonates diagnosed as BPD or non-BPD. BPD was diagnosed for 22.5% of males and 7.5% of females. Infants with BPD were born at significantly lower mean birth weight (925.9 g vs 1187.9 g) and GA (27.6 weeks vs 30.3 weeks) compared to non-BPD infants (Table 1). Birth weight was significantly (*r*^2^ = 0.38, *p* < 1.0E−09) associated with GA (Fig. 1A). Supplemental O_2_ therapy was given to stabilize premature infants’ breathing pattern, blood O_2_ levels, and to support sufficient growth and weight gain. We observed a significant negative correlation (*r*^2^ = 0.32, *p* < 1.0E−09) between GA of infants and their cumulative days of O_2_ supplementation in the NICU (Fig. 1B). Low birth weight infants showed a similar association with longer O_2_ supplement (*r*^2^ = 0.23, *p* < 1.0E−07, not shown); however, it was notable that ten neonates with birth weight < 1000 g did not require supplemental O_2_ to maintain blood O_2_ levels. In agreement with many other studies [5, 10, 37], it is evident from these data that extremely low birth weight, low GA infants are more likely to need supplemental O_2_ and to develop BPD.

**Table 1:** Characteristics of the Buenos Aires, Argentina, preterm infant cohort

Characteristics	Infants with BPD	Infants without BPD	*p* value
Neonate characteristics			
Sample size	14^a^	93	
Birth weight (g)	925.9 ± 75.6	1187.9 ± 25.2	0.003
Gestation age (weeks)	27.6 ± 0.7	30.3 ± 0.2	0.0000157
Sex	Male 9	Male 31	0.0372
	Female 5	Female 62	
Cumulative NICU O_2_ (Days)	47.1 ± 5.2	7.8 ± 1.1	< 0.001
Delivery room surfactant	1/12 (8%)	4/86 (5%)	0.302
Maternal characteristics			
Maternal race			0.0693
European-Latin	11	68	
Criollos	0	14	
European-other	0	5	
Jewish	1	3	
Arab-Middle Eastern	1	0	
African Caribbean	0	1	
Native American	1	0	
Asian	0	1	
Unknown or refused	0	1	
Maternal age (year)	32.2 ± 1.9	34.1 ± 0.5	0.211
Maternal smoking history^b^	7/14 (50%)	25/93 (27%)	0.0795
Maternal antenatal steroid	13/13 (100%)	82/90 (91%)	0.333
Maternal preeclampsia	3/14 (21%)	25/93 (27%)	1
IUGR-reason for delivery	5/12 (42%)	33/90 (37%)	0.321
BMI	33.6 ± 1.8	34.1 ± 5.2	0.604
Education^c^	9/4/0/1	63/20/5/5	0.849
Employed first trimester	10/13 (77%)	69/88 (78%)	0.886
Alcohol during pregnancy	0	1/88 (1%)	0.331
Chorioamnionitis	2/12 (17%)	6/85 (7%)	0.238
Asthma	0	0	1
Other respiratory disease	0	0	1
Fetal complications			
Fetal IUGR	5/12 (42%)	38/88 (43%)	0.460
Fetal oligohydramnios	3/12 (25%)	11/88 (13%)	0.155

Samples recruited from the Discovery-Bronchopulmonary Dysplasia Program (D-BPD) cohort in Buenos Aires, Argentina [80]. Mean ± S.E.M. presented

*NICU* newborn intensive care unit, *O*_*2*_ oxygen supplement, *IUGR* intrauterine growth restriction. Detailed cohort characteristics are in Additional file 1: Table S1

^a^Two neonates were diagnosed with BPD upon death at 14 and 22 days of life

^b^Self-reported former and/or current smoker

^c^Higher/secondary/primary/NA

### Methylation-based estimation of cord blood cell-type composition

**Fig. 2:**
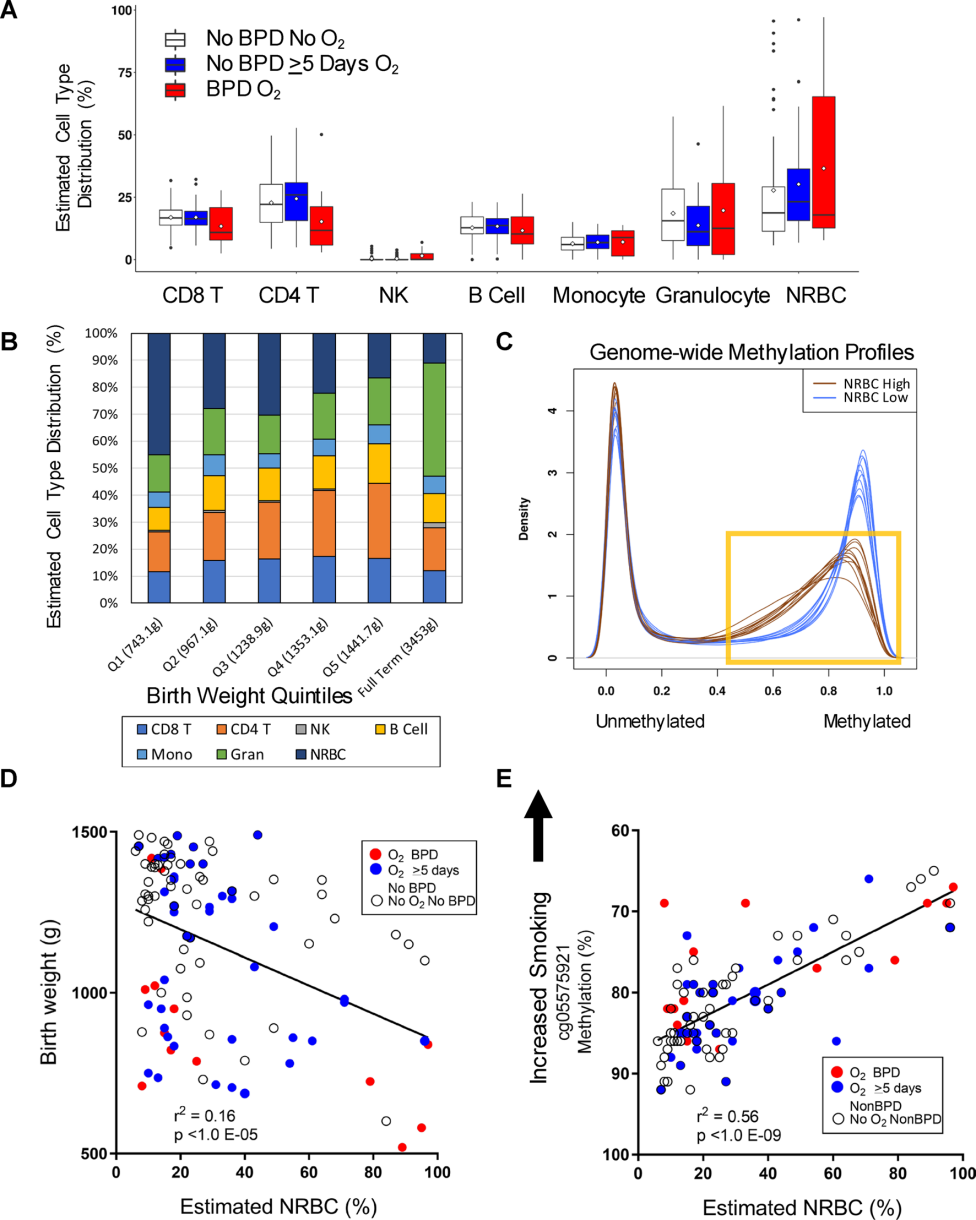
DNA methylation-based prediction of cord blood cell-type composition. **A** Box and whisker plot shows percent distribution of 7 blood cell types in preterm infants with or without bronchopulmonary dysplasia (BPD). Mean indicated by open dot within box, median, 25th and 75th percentile presented. **B** Variation in percent cell-type distribution by birth weight quintile and comparison with that in full-term infants. **C** Density plot of genome-wide methylation distribution comparing profiles of the 10 highest (brown) and 10 lowest (blue) nucleated red blood cell (NRBC) samples. Yellow box highlights demethylation in high NRBC samples. **D** Correlation of estimated percent NRBC with birth weight in preterm infants. **E** Correlation of aryl-hydrocarbon receptor repressor (*AHRR*) smoking biomarker (cg05575921) with estimated NRBC and distribution among BPD groups. *CD4T* CD4^+^ T cells, *CD8T* CD8^+^T cells, *NK* natural killer cells, *Mono* monocyte, *Gran* granulocyte

The methylation-based estimated percentages of CD8^+^ T cells, CD4^+^ T cells, B cells, natural killer cells, monocytes, granulocytes, or NRBCs in cord blood DNA did not differ significantly between BPD and all non-BPD infants. We also observed no cell-type differences relative to the non-BPD infants who did not require supplemental O_2_ (Fig. 2A, Additional file 1: Table S2). Figure 2B displays cord blood cell-type composition relative to birth weight quintiles among all infants regardless of disease or O_2_ status [mean weight for quintiles (mean GA, weeks), Q1: 743.1 g (27.3), Q2: 967.1 g (29.1), Q3: 1238.9 g (30.7), Q4:1353.1 g (31.2), Q5: 1441.7 g (30.8) and full term, 3453 g (39.4)]. Estimated cell-type distributions in preterm infant < 35 weeks (all infants in this study) show gradual shifts relative to birth weight but even the largest preterm infants have more NRBCs and fewer granulocyte than full-term infants (mean birth weight = 3453 g) (Fig. 2B far right bar, estimated cell-type composition for full term infants extracted from our recent study, Bergens et al. [38]). Among all preterm infants, there was a significant positive correlation between birth weight and CD4^+^ T cell percentage (*r*^2^ = 0.183, *p* = 4.3E−06) suggesting a linear relation with hematopoiesis and fetus size. GA was also correlated with CD4^+^ T cell percent (*r*^2^ = 0.14, *p* = 7.4E−05) but not as highly.

NRBCs, immature red blood cells with nuclei containing genomic DNA, occur at substantial frequencies in cord blood. Our analysis estimated high average NRBC levels in the smallest, lowest GA infants (Fig. 2B). The estimated proportion of cord blood DNA contributed by NRBCs ranged from 5.7 to 97.2% (mean = 29.9%, SD = 24.1%) and a relatively large number (n = 18) of preterm neonates had > 50% NRBC DNA in their blood (Fig. 2B, Additional file 1: Table S2). The mean of 29.9% is similar to the reference mean value of 25% for infants born at less than 30 weeks GA [57]. We thus assessed if NRBC proportions affected DNA methylation levels by comparing genome-wide profiles from high and low NRBC cord blood samples. Figure 2C shows overall DNA methylation density profiles (~ 450,000 CpGs) from the 10 highest (brown) and the 10 lowest (blue) NRBC cord blood samples. The distribution of β-values (estimate of methylation level) was markedly dissociated between the low- and high-NRBC samples. Moreover, a reduction in fully methylated CpGs was found in the high-NRBC bloods indicating demethylation relative to the expected methylation profiles for low NRBC bloods. Using univariate regression, there was a significant (*r*^2^ = 0.160, *p* = 1.9E−05) negative correlation between estimated NRBC percent and birth weight (Fig. 2D) and a nominally significant correlation with GA (*p* = 0.027). Importantly, while four of the preterm neonates among the very low birth weight and very high NRBCs developed BPD (Fig. 2D), a large proportion of the high NRBC bloods were among the larger neonates, suggesting other factors in the etiology of NRBCs. DNA methylation levels of less than 75% at aryl-hydrocarbon receptor repressor (*AHRR*) cg05575921, the top-ranked epigenetic biomarker of smoking, are suggestive of prenatal tobacco smoke exposure [38, 39], and frequency of NRBCs in cord blood has been correlated with cigarette smoking [40, 41]. The blood samples with high NRBCs in our cohort were significantly correlated with lower percent methylation level of cg05575921 (Fig. 2E), which suggested recent maternal exposure to tobacco smoke in these preterm babies. The genome-wide demethylation of NRBCs (shown in Fig. 2C) did not contribute substantially to this result (see Additional file 2: Figure S1).

NRBC estimation is based on a reference set of 100 NRBC-specific CpGs, and the estimates are used as covariates in EWAS to adjust for any difference in NRBCs across the disease groups. Because NRBCs have highly divergent methylation profiles, we had a concern that adjustment in multivariable regression analysis might not fully compensate for differences in NRBC levels among cord blood samples, and thus, we assessed if additional CpGs were NRBC-specific relative to the reference NRBC set. Comparing reference NRBC methylation profiles to preterm cord blood methylation profiles identified 3647 CpGs at genome-wide significance level (Additional file 1: Table S3).

### Epigenome-wide association study (EWAS) of BPD in preterm infants

**Fig. 3:**
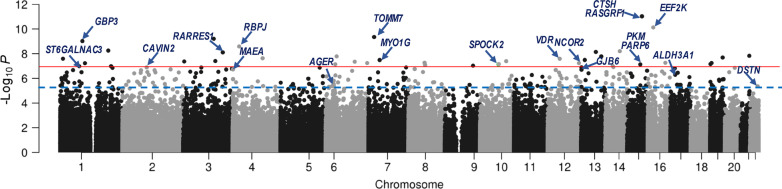
Epigenome-wide association study (EWAS) for bronchopulmonary dysplasia (BPD) risk in preterm infants. A Manhattan plot of robust linear regression model-based cord blood CpGs associated with BPD risk in Argentina preterm infant cohort (*n* = 107). Thirty-eight CpGs were significant following Bonferroni cutoff (*p* < 1.04E−07, red line) and 275 CpGs were significant at false discovery rate < 0.01 (blue line). Representative gene names annotated to the differentially methylated CpGs are labeled and depicted by arrows. *AGER* advanced glycosylation end-product-specific receptor, *ALDH3A1* aldehyde dehydrogenase 3 family member A1, *CAVIN2* Caveolae-associated protein 2, *CTSH* cathepsin H, *DSTN* destrin, *EEF2K* eukaryotic elongation factor 2 kinase, *GBP3* guanylate-binding protein 3, *GJB6* gap junction protein beta 6, *MAEA* macrophage erythroblast attacher, *MYO1G* myosin IG, *NCOR2* nuclear receptor corepressor 2, *RARRES1* retinoic acid receptor responder 1, *PARP6* protein mono-ADP-ribosyltransferase, *PKM* pyruvate kinase, *RBPJ* recombination signal-binding protein for immunoglobulin kappa J region, *SPOCK2* SPARC (osteonectin), Cwcv and Kazal-like domains proteoglycan 2, *ST6GALNAC3* alpha-N-acetylgalactosaminide alpha-2,6-sialyltransferase 3, *TOMM7* translocase of outer mitochondrial membrane 7, *VDR* vitamin D (1,25- dihydroxy vitamin D3) receptor

Robust linear regression analysis with adjustment for covariates (GA, birth weight, smoking, ethnicity/race, seven cell-type percentages, hospital at birth, and percentile of days in which O_2_ supplementation used in NICU) identified 38 CpGs associated with BPD at genome-wide (Bonferroni, *p* < 1.12E−07), and 275 CpG sites at false discovery rate (FDR) < 1% (Fig. 3), among which 64% (176/275) CpGs were differentially hypomethylated in BPD than in non-BPD (Table 2 and Additional file 1: Table S4). At FDR 5% (Additional file 1: Table S4), 1581 CpG sites annotated to a total of 2164 genes were differentially methylated between BPD and non-BPD (874/1581 loci were hypomethylated in BPD). The 275 FDR 1% differentially methylated loci were annotated to a total of 386 nearby genes (Table 2 and Additional file 1: Table S4). Winsorizing the DNA methylation dataset to reduce the potential impact of outliers had little effect on the detection of the differentially methylated CpGs (Additional file 1: Table S5). Of the 275 BPD CpGs at 1%FDR, only 6 CpGs overlapped with the NRBC-specific epigenome list (Additional file 1: Table S3).

**Table 2:** Representative CpGs significantly associated with bronchopulmonary dysplasia (BPD) risk

CpG	*P*	BPD Meth (%)	Non- BPD Meth (%)	dMeth (%)	Chr	hg19 position	Associated genes	Gene description
cg23328237	9.26E−12	84.96	85.35	− 0.39	15	79,252,815	*CTSH* *RASGRF1*	PRO-cathepsin H precursor Ras-specific guanine nucleotide-releasing factor 1
cg08139238	7.41E−11	77.50	81.95	− 4.45	16	22,219,550	*EEF2K*	Eukaryotic elongation factor 2 kinase
cg14448850	4.46E−10	88.63	90.12	− 1.49	7	22,860,191	*TOMM7*	mitochondrial import receptor subunit TOM7 homolog
cg22707878	6.17E−10	81.68	83.73	− 2.05	3	122,604,848	*LINC02035* *SLC49A4*	Long intergenic non-protein coding RNA 2035 Solute carrier family 49 member 4
cg23540651	9.46E−10	81.90	84.71	− 2.81	1	89,490,034	*GBP3*	Guanylate-binding protein 3 isoform 4
cg26968498	2.58E−09	87.07	89.35	− 2.28	4	26,174,157	*RBPJ*	Recombining binding protein suppressor of hairless
cg00013369	5.54E−09	84.61	85.93	− 1.32	1	192,857,594	*LINC01032*	Long intergenic non-protein coding RNA 1032
cg19815139	6.24E−09	6.15	5.61	0.53	14	77,924,143	*AHSA1* *VIPAS39*	Activator of 90 kDa heat shock protein ATPase homolog 1 Spermatogenesis-defective protein 39 homolog
cg05577945	7.20E−09	5.23	4.54	0.69	13	78,271,874	*SLAIN1* *MIR3665*	SLAIN motif-containing protein 1 microRNA 3665
cg16708174	7.81E−09	76.80	78.42	− 1.62	3	158,430,962	*RARRES1*	Retinoic acid receptor responder protein 1 precursor
cg06342416	1.48E−08	15.94	16.12	− 0.18	21	45,773,569	*TRPM2*	Transient receptor potential cation channel subfamily M member 2
cg26371345	1.64E−08	16.82	12.84	3.98	6	44,090,748	*MRPL14* *TMEM63B*	39S ribosomal protein L14, mitochondrial CSC1-like protein 2
cg22148297	1.65E−08	14.39	11.05	3.34	13	99,910,587	*GPR18* *UBAC2*	N-arachidonyl glycine receptor Ubiquitin-associated domain-containing protein 2
cg24025538	2.03E−08	85.77	86.95	− 1.18	19	50,364,499	*PTOV1-AS2* *PTOV1* *PNKP*	PTOV1 antisense RNA 2 Prostate tumor-overexpressed gene 1 protein isoform 2 Bifunctional polynucleotide phosphatase/kinase
cg09757859	2.31E−08	85.54	85.85	− 0.32	4	119,948,505	*MYOZ2* *SYNPO2*	Myozenin-2 synaptopodin-2
cg09551145	2.52E−08	11.26	11.05	0.21	1	11,790,591	*AGTRAP* *DRAXIN*	Type-1 angiotensin II receptor-associated protein draxin precursor
cg22833603	2.54E−08	84.94	87.24	− 2.31	12	48,304,627	*VDR*	Vitamin D3 receptor
cg20084577	3.20E−08	80.69	83.32	− 2.62	13	33,780,913	*STARD13*	stAR-related lipid transfer protein 13
cg06787669	3.22E−08	6.58	5.39	1.19	7	45,018,789	*MYO1G* *SNHG15*	Unconventional myosin-Ig small nucleolar RNA host gene 15
cg18067163	3.93E−08	76.98	78.17	− 1.20	3	128,584,482	*LOC653712*	Intraflagellar transport 122 homolog pseudogene
cg24252723	4.04E−08	3.39	2.82	0.57	10	104,678,150	*CNNM2*	Metal transporter CNNM2
cg07733260	4.27E−08	83.54	84.26	− 0.72	3	4,355,431	*SETMAR*	Histone-lysine N-methyltransferase SETMAR
cg16176600	4.46E−08	70.13	71.84	− 1.70	6	116,381,609	*FRK*	Uncharacterized protein LOC583550
cg21845457	5.35E−08	9.77	7.40	2.37	8	65,499,091	*BHLHE22* *CYP7B1*	Class E basic helix-loop-helix protein 22 Cytochrome P450 7B1 isoform 1
cg06733215	5.44E−08	79.48	81.87	− 2.39	16	70,317,506	*AARS1*	Alanine–tRNA ligase, cytoplasmic
cg20238368	5.63E−08	85.00	85.56	− 0.56	19	5,795,577	*DUS3L*	tRNA-dihydrouridine(47) synthase [NAD(P)( +)]-like
cg17270257	5.70E−08	6.92	5.75	1.17	6	166,074,870	*PDE10A*	cAMP and cAMP-inhibited cGMP 3',5'-cyclic phosphodiesterase 10A
cg24847366	5.72E−08	75.54	84.10	− 8.56	12	125,034,283	*NCOR2*	Nuclear receptor corepressor 2
cg22228748	5.82E−08	86.93	88.21	− 1.28	1	99,385,800	*PLPPR5*	Phospholipid phosphatase-related protein type 5
cg10355458	6.88E−08	91.32	89.35	1.97	19	1,578,206	*MEX3D* *MBD3*	RNA-binding protein MEX3D isoform 1 Methyl-CpG-binding domain protein 3
cg02370222	7.17E−08	88.61	87.82	0.79	6	38,580,256	*BTBD9*	BTB/POZ domain-containing protein 9
cg17958658	7.25E−08	5.81	4.47	1.34	10	73,847,963	*SPOCK2*	SPARC (osteonectin), Cwcv, and Kazal-like domains proteoglycan 2
cg20064830	7.39E−08	83.30	86.31	− 3.00	15	72,535,923	*PKM* *PARP6*	Pyruvate kinase PKM Protein mono-ADP-ribosyltransferase PARP6
cg07866137	8.21E−08	6.24	5.32	0.92	8	67,579,786	*C8orf44* *VCPIP1* *C8orf44-SGK3*	Chromosome 8 open reading frame 44 Deubiquitinating protein VCIP135 Serine/threonine-protein kinase Sgk3
cg13854960	9.35E−08	75.92	78.23	− 2.31	9	112,965,949	*C9orf152*	Uncharacterized protein C9orf152
cg08221288	9.96E−08	73.06	79.53	− 6.47	14	50,081,280	*RPL36AL* *LRR1*	60S ribosomal protein L36a Leucine-rich repeat protein 1
cg16290101	1.04E−07	85.73	87.33	− 1.60	1	204,010,929	*LINC00303*	Long intergenic non-protein coding RNA 303
cg07108118	1.04E−07	77.09	80.24	− 3.15	1	76,543,815	*ST6GALNAC3*	Alpha-N-acetylgalactosaminide alpha-2,6-sialyltransferase 3
*cg18363035	1.27E−07	36.55	30.83	5.73	13	20,805,385	*GJB6*	Gap junction beta-6 protein
*cg04141129	2.09E−07	69.78	77.35	− 7.57	12	32,530,696	*FGD4* *BICD1*	FYVE, RhoGEF and PH domain-containing protein 4 Protein bicaudal D homolog 1 isoform 7
*cg03369382	3.37E−07	84.29	85.63	− 1.35	7	139,427,635	*HIPK2*	Homeodomain-interacting protein kinase 2
*cg14227558	1.13E−06	27.56	17.94	9.63	2	213,983,851	*IKZF2*	Zinc finger protein Helios

A total of 275 CpGs were significantly different between BPD (*n* = 14) and non-BPD (*n* = 93) infants at FDR = 1%. Shown are 39 passing Bonferroni correction (*p* < 1.04E−07)- and four selected (*) among FDR CpGs are shown. Robust linear regression *p* value adjusted by GA, sex, birth weight, 7 cell types, percentage cumulative neonate intensive care unit oxygen days and hospital

*Meth* mean methylation value (%), *dMeth* methylation difference in BPD relative to non-BPD, *Chr* chromosome, *hg19* human genome assembly GRCh37

Full list of the significant CpGs differentially methylated between two groups at FDR 5% (1581 CpGs) is in Additional file 1: Table S4

The most significant CpG associated with BPD in the current study was cg23328237 in the 3’ untranslated region of *RASGRF1* (encoding Ras protein-specific guanine nucleotide releasing factor 1) and upstream of *CTSH* (encoding cathepsin H). *RASGRF1*, *CTSH* and two additional genes that were strongly associated with BPD, *NCOR2* (encoding nuclear receptor corepressor 2; cg24847366) and *SPOCK2* (encoding SPARC (osteonectin), Cwcv, and Kazal-like domains proteoglycan 2; cg17958658), have previously been reported to be related to BPD pathogenesis or preterm GA [13, 18, 26, 27, 33, 42].

### BPD association with EGA acceleration

EGA is a concept introduced by Horvath [43] in which age, or in this case GA, is estimated by a model using DNA methylation data and is compared with actual recorded GA. EGA acceleration in newborns has been associated with a set of adverse conditions [28, 44]. We calculated EGA using two published methods [26, 28]. Comparing recorded GA with calculated EGA, we found both Bohlin et al. [26] and Knight et al. [28] methods overestimated GA for both non-BPD and BPD infants in our study (Additional file 2: Figure S2A). EGA acceleration was calculated as the residual of the linear regression of EGA on GA for each sample. Then, we carried out a multivariable linear regression analysis to test if EGA acceleration was associated with BPD status. Additional file 2: Figure S2B displays violin plots of the results of the different models and significance levels without or with adjustment for all EWAS covariates. The Bohlin model produced an EGA acceleration result suggesting a more mature value for BPD infants (adjusted *p* = 0.033).

### BPD associated with increased stochastic epimutations

**Fig. 4:**
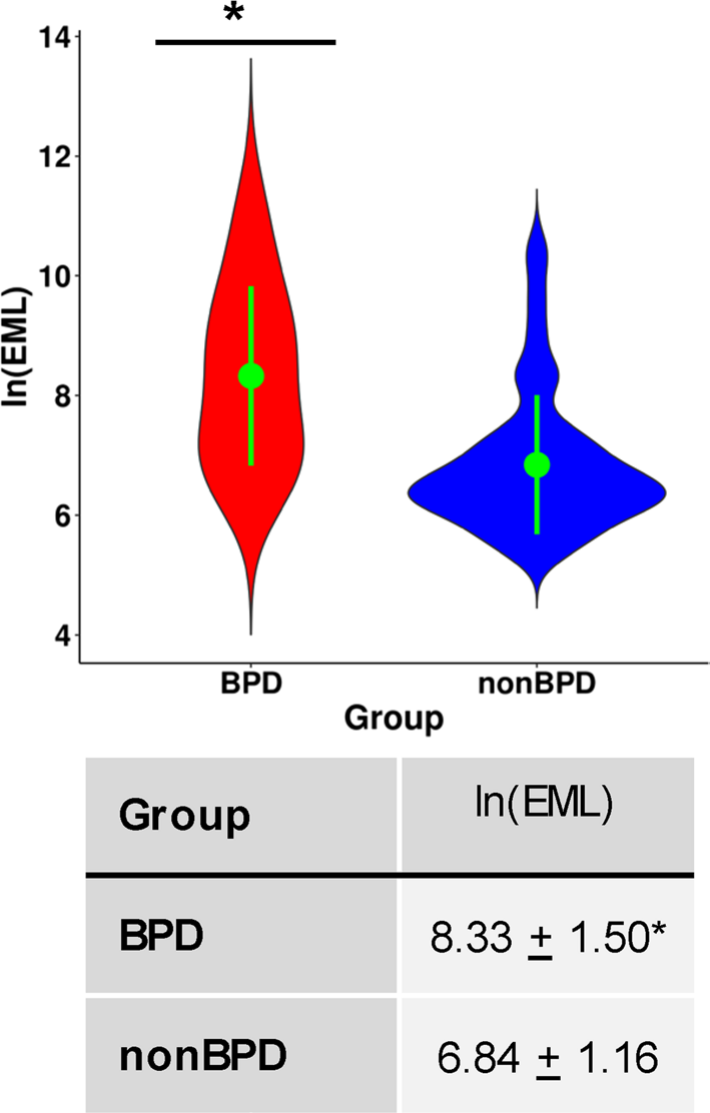
Epigenetic mutation load (EML) in preterm infants with or without bronchopulmonary dysplasia (BPD). EML was significantly higher in BPD samples than in non-BPD samples. EML was calculated as the natural log (ln) of total number of stochastic epigenetic mutations (SEMs) per individual. In violin-plot, green dot and bar show mean and standard deviation, respectively. *Statistics of linear regression on EML; *r* = 0.39, *p* = 4.18 × 10^–5^ (*p* adjusted = 0.02). Statistics of biweight midcorrelation (bicor); *r* = 0.32, *p* = 6.56 × 10^–4^

SEMs are outlier methylation values observed at a CpG relative to other samples in a dataset and are thought to represent an alteration of epigenetic maintenance [42, 45]. SEMs have previously been linked to preterm birth [42], cancer risks [46], and aging [45], and they may be a mediator between environmental exposures and adverse health outcomes. Epigenetic mutation load (EML) values were calculated as the natural logarithm of total number of SEMs per individual [42, 45]. Among non-BPD samples, SEMs were highly variable, with a range of 191 to a maximum of 75,588. First, the association between EML and BPD status or other covariates was examined by linear regression as well as biweight midcorrelation (bicor), a median-based measurement of correlation that is robust to outliers [47]. In these unadjusted analyses, EMLs were significantly higher among BPD neonates (*p* = 4.18E−05 for linear regression and *p* = 6.56E−04 for bicor, Fig. 4). Additionally, in univariate analyses EMLs were associated with birth weight, cg05575921 methylation, some cell-type percentages and cumulative days of O_2_ (Additional file 1: Table S6). We further performed a robust linear regression of EMLs on BPD status with adjustment for all EWAS covariates and observed a strongly attenuated but significant association between EMLs and BPD (*p* adjusted = 0.02). In order to explore what the source of SEMs might be relative to other outcomes we compared the list of multiply occurring SEM CpGs (CpGs occurring 3 or more times among SEMs) with the list of CpGs associated with the NRBC reference set (3647 CpGs at Bonferroni significance). The overlap was highly significant, with 30% (1095/3647) of SEM CpGs found to be among NRBC-associated CpGs (*p* < 2.2E−16).

### Pathway analyses for genes annotated in BPD epigenome

**Fig. 5:**
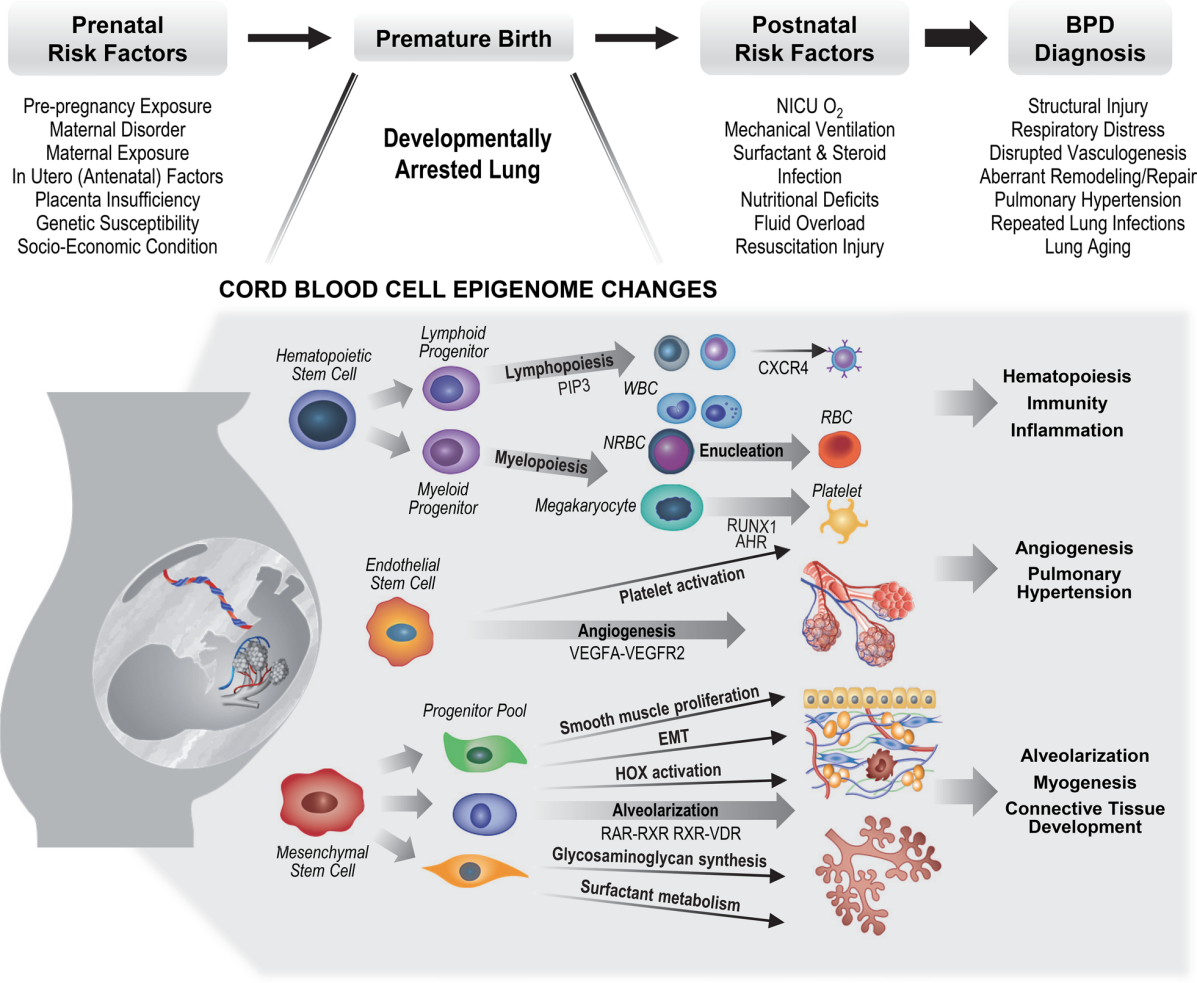
Biological functions and pathways predicted by epigenome changes in bronchopulmonary (BPD) cord blood. Pathway analyses done by Ingenuity Pathway analysis (IPA), ToppGene Suite, David Functional Annotation, and Reactome Pathway Database determined enriched biological functions and signal transduction pathways for the annotated 386 genes to 275 CpG loci associated with BPD risk. *CXCR4* C-X-C motif chemokine receptor 4, *HOX* homeobox, *NRBC* nucleated red blood cell, *PIP3* phosphoinositide 3, *RAR* retinoic acid receptor, *RXR* retinoid X receptor, *RUNX1* runt-related transcription factor 1, *VDR* vitamin D receptor, *VEGF* vascular endothelial growth factor, *WBC* white blood cell

To gain insights into the relationship between differentially methylated CpGs and BPD pathogenesis, CpGs were annotated to the nearest genes and pathway analysis tools were applied to elucidate gene ontology, biological process, diseases, and canonical pathways of these annotated genes cutoff at both FDR 1% and FDR 5%. Importantly, many enriched functions and pathways were related to lung development (Table 3 and Additional file 1: Table S7A, Fig. 5). These included retinoic acid receptor (RAR)/retinoid X receptor (RXR) signaling (e.g., *NR0B2*, *NCOR2*, *VDR*, *RARRES1*), androgen receptor signaling (e.g., *PTEN*, *BRCA1*, *DSTN*, *RB1*, *FOXP1*), cell proliferation and extracellular matrix (ECM) events including BPD-associated epithelial-mesenchymal transition (EMT) [48] (e.g., *RB1*, *RBPJ*, *FOXP1*, *AVDR*, *CHRM5*, *COL21A1*, *VEPH1*), and lung surfactant and glycosaminoglycan metabolism and alveolarization (e.g., *CTSH*, *AGER*, *FOXP1*, *CAVIN2*, *SPOCK2*, *CHST14*, *HS6ST1*, *ARRB1*, *DSTN*). In addition, hematological system development and vascular disorder related processes, which are also critical to lung maturation, were associated with the BPD epigenome (Table 3 and Additional file 1: Table S7A, Fig. 5). These included angiogenesis and vascular endothelial growth factor (VEGF) signaling (e.g., *BAD*, *PLCB3*, *CDC42*, *ITGA2B*, *THBS1*, *LCK*, *EDN1*, *ANGPT2*, *EEF2K*), platelet activation (e.g., *THBS1*, *SLC39A5*, *EDN1*, *AMOTL1*, *CCL17*, *CDC42*, *AGER*, *CAVIN2*), aryl hydrocarbon receptor signaling (e.g., *ALDH16A1*, *ALDH3A1*, *H2AC4*, *AGO2*, *THBS1*, *KIF1B*), leukopoiesis (e.g., *NR0B2*, *PTEN*, *PDPK1*, *BRCA1*, *CCL17*, *CDC42*, *CXCL11*, *LCK*, *CYTH3*, *FLOT2*) and RBC enucleation and maturation (e.g., *HIPK2*, *RB1*, *MAEA*, *RHAG*). Other genes were related to BPD-associated outcomes such as retinopathy of prematurity (ROP, e.g., *GBP3*, *FJX1*, *HIPK2*) and hearing loss (e.g., *GJB6*, *TMEM63B*) and mitochondrial energy metabolism (e.g., *TOMM7*, *SLC25A26*, *SLC25A33*, *PDK1*, *PKM*, *MDH1*). Enriched functions and pathways of the annotated genes from FDR 5% cutoff further expanded focal adhesion (e.g., multiple cadherin genes) and actin cytoskeleton organization (e.g., *ABL2*, *CUL3*, *GAS7*, *SHROOM3*, *DBNL*, *RND3*) signaling pathways that are critical in cellular morphogenesis and movement particularly during development (Additional file 1: Table S7A). Table 3 and Additional file 1: Table S4 also include the results from the GOmeth analysis on Gene Ontology (GO, Additional file 1: Table S7B) and Kyoto Encyclopedia of Genes and Genomes (KEGG, Additional file 1: Table S7C) databases to enrich the pathways with consideration of the different numbers of CpG sites per gene and the CpGs annotated with multiple genes. Overall, these results suggest multiple cord blood cell genes that were differentially methylated in BPD may play roles in critical cellular and molecular processes related to BPD pathogenesis.

**Table 3:** Predicted functions and pathways of genes related with bronchopulmonary dysplasia (BPD) epigenome

Functions and pathways	p-adj*	Selected related genes
Development and lung alveolarization		
Retinoic acid/retinoid X receptor/vitamin D receptor signaling	1.70E−02	*ARID5A NCOA1 NCOR2 NR0B2 RARRES1 RARRES2 VDR*
Androgen receptor signaling	8.30E−03	*BRCA1 CDC42 DSTN FHL2 FOXP1 HSD17B3 NCOA1 NCOR2 NR0B2 PTEN RB1*
Lung surfactant metabolism, alveolar epithelial development, glycosaminoglycan metabolism	2.90E−02	*AGER AHSA1 ARRB1 CAVIN2 CHST14CTSH FNDC3B FOXP1 HS6ST1 MIR449A MIR326 NCOR2 RB1 RBPJ SPOCK2*
In utero/embryo growing	2.10E−02	*AGER AGO2 AKAP13 ARRB1 CDH5 CTNND1 EDN1 NCOA1 NCOR2 NF1 NKX3-2 PBX2 PDPK1 PTEN RARRES2 RB1 VDR*
Hematological system development and vascular disorders		
Platelet activation and coagulation	9.91E−03	*AGER AMOTL1 ARRB1 CAVIN2 CDC42 CCL17 DGKA EDN1 GNG2 H3C2 ITGA2B LCK PDK1 PDPK1 PTEN SLC39A5 THBS1*
Angiogenesis and vascular permeability IL-8- and CXCR2-mediated signaling, VEGF/VEGFR2 pathway	1.44E−02	*ANGPT2 ARRB1 CAVIN2 CCN1 CDH5 CDC42 CTNND1 DOCK2 EDN1 EEF2K EPS15 GNG2 ITGA2B LCK PDK1 PDPK1 PLCB3 SLC39A5 SYNPO2 THBS1*
Hematopoiesis Leukopoiesis, CXCR4-mediated signaling, PIP3 signaling in B lymphocytes	8.30E−03	*AGER BAD BRCA1 AREB1 CCL17 CCN1 CDC42 CXCL11* *CYTH3 DAPP1 FLOT2 FLT3LG GNG2 IKZF2 LCK NF1NR0B2 PDPK1 PLCB3 PTEN UBQLN1*
Enucleation of erythroid precursor cells	1.44E−02	*HIPK2 RB1 MAEA RHAG*
Megakaryocyte differentiation Aryl hydrocarbon receptor signaling, RUNX1 regulation	2.09E−02	*AGO2 ALDH16A1 ALDH3A1 CDC42 DOCK2 GNG2 H2AC4 H3C2 ITGA2B KIF1B LCK NCOR2 NEDD8 NR0B2 PSMB8 RB1 THBS1*
Cell proliferation and extracellular matrix		
Smooth muscle proliferation, muscle morphology and size, skeletal system development	1.40E−02	*AGER AGTRAP AKAP13 ANGPT2 CHRM5 CLASP1 EDN1 FHL2 FOXP1 KEL LDB3 MYOZ2 NCOA1 NF1 PDPK1 PTEN RARRES2 RASGRF1 RB-SKI RB1 RBPJ TERC THBS1 TNFSF9 TRPM2 VDR ZBTB16*
Epithelial-mesenchymal transition, extracellular matrix organization	3.46E−02	*BANP BRCA1 BTBD7 CCN1 CDC42 CLASP1 COL21A1 FNDC3B ITGA2B LAMB3 MFAP3L MIR193B NCOA1 PDPK1 PHLDB2 PTEN RB1 RBPJ SLC25A33 THBS1 UBQLN1 VEPH1*
Mitochondrial energy metabolism Response to decreased O_2_ level, glucose deprivation	1.13−E02	*AGER AGTRAP ALDH3A1 ANGPT2 EDN1 EEF2K GHSR HIPK2 MDH1 MRPL14 NF1 PDK1 PKLR PKM PSMB8 PTEN RBPJ RHAG SLC25A26 SLC25A33 TERC THBS1 TOMM7 UBQLN1*
Retinal disorder, inner ear development	4.66−E02	*CAVIN2 CCN1 FJX1 GBP3 GJB6 HIPK2 LCK PTEN RB1 RBPJ TMEM63B*
Immune and inflammatory responses	1.27−E02	*AGER AGTRAP ANGPT2 ARRB1 CCN1 CCNT1 CDC42 CDH5 CXCL11 CYP7B1 DOCK2 EDN1 FLT3LG GPR18 KAT8 LCK MYO1G PDK1 PLCB3 PNKP PSMB8 PTEN RARRES2 RB1 TERC TNFSF9 TOP1 TRPM2 UBQLN1 VDR*
Production of reactive oxygen species	1.81−E02	*AGER AGTRAP ANGPT2 BRCA1 CCN1 CDC42 CDH5 DOCK2 EDN1 LCK PDK1 PLCB3 PNKP PSMB8 PTEN RARRES2 RB1 RRBPJ SLC25A33 TOP1 TRPM2 UBQLN1 VDR*

Analyses were done using Ingenuity Pathway Analysis (IPA), ToppGene Suite, David Functional Annotation, and Reactome Pathway Database tools

*Significance between the number of genes differentially methylated in BPD versus non-BPD and the total number of genes in annotated gene ontology, functions, or pathways (the lowest adjusted *p* value of similar annotations from multiple pathway analysis tools). Analyzed with 385 genes associated with 275 CpGs (FDR 1%) significantly varied between BPD (*n* = 14) and non-BPD (*n* = 93). Pathways analyzed with 2164 genes annotated to 1581 CpGs (FDR 5%) are listed in Additional file 1: Table S7A

### cDNA microarray profiles associated with BPD in preterm infant cord blood cells

A small number of cord blood samples (BPD *n* = 6, non-BPD *n* = 16) were available for genome-wide gene expression analysis using Illumina HT12 arrays. A total of 273 cord blood genes were significantly altered in BPD infants (273 at FDR < 5%, with 16 genes at Bonferroni) compared to non-BPD controls (Table 4 and Additional file 1: Table S8). In BPD patient RNA samples, more genes displayed downregulation (*n* = 216/273) over non-BPD expression level. Based on pathway enrichment analysis (Table 4), genes altered in BPD blood cells were mainly enriched in cell cycle regulation and arrest (e.g., *PPP2CA*, *RBL2*, *CENPU*, *CCNY*, *CDK6*) and pulmonary disorder and developmental disorders (e.g., *AGER*, *ITGA6*, *CA4*, *BACH2*, *IGFBP2*, *BMPR2*, *SKI*, *DAB2*). In addition, genes associated with hematological disease (e.g., *PDE7A*, *LMAN1*, *F13A1*, *OLR1*) and mitochondrial biogenesis and redox (e.g., *NOS3*, *ALDH5A1*, *PRXL2A*, *SLC25A13*, *CPT2*) were also significantly altered in BPD patients (Table 4).

**Table 4:** Representative cord blood genes altered in bronchopulmonary dysplasia (BPD)

Functions and Pathways	*p-adj*	dEx	Gene symbol	Gene description
Cell cycle regulation/arrest, gene expression	0.0000138287972305428	− 0.61	CDC14B*	Cell division cycle 14B
	0.018196732932063	− 0.12	*PTPA*	Protein phosphatase 2 phosphatase activator
	0.018196732932063	− 0.19	*PRDM2*	PR/SET domain 2
	0.0245160911780221	− 0.37	*RBL2*	RB transcriptional corepressor-like 2
	0.0270275178681041	− 0.73	*PPP2CA*	Protein phosphatase 2 catalytic subunit alpha
	0.0305102740202983	− 0.56	*CENPU*	Centromere protein U
	0.0363416990685133	− 0.29	*SOX6*	SRY-box transcription factor 6
	0.036719398944197	− 0.85	*CCNY*	Cyclin Y
	0.0490173110568865	− 0.58	*CDK6*	Cyclin-dependent kinase 6
Morbidity/mortality, developmental disorder, skeletal and muscular system development/ functions, connective tissue disorders	0.00953646339176695	− 0.61	*IGFBP2*	Insulin-like growth factor-binding protein 2
	0.0112129962231962	− 0.45	*HBZ*	Hemoglobin subunit zeta
	0.0139799604731326	− 0.13	*CASP4*	Caspase 4
	0.04143444	− 0.77	*ALDH5A1*	Aldehyde dehydrogenase 5 family member A1
	0.0442867038269876	− 1.00	*ALOX12*	Arachidonate 12-lipoxygenase, 12S type
	0.0000349238050329156	− 0.54	SSB*	Small RNA-binding exonuclease protection factor La
	0.0141776721977405	− 0.93	*COPS8*	COP9 signalosome subunit 8
	0.0171815019940262	− 0.78	*GNPNAT1*	glucosamine-phosphate N-acetyltransferase 1
	0.0193484434496371	0.18	*SDC1*	Syndecan 1
	0.0194007739744968	− 0.96	*BMPR2*	Bone morphogenetic protein receptor type 2
	0.0210884700904177	− 0.73	*MYCN*	MYCN proto-oncogene, bHLH transcription factor
	0.0349205290474328	1.16	*VSIG10*	V-set and immunoglobulin domain containing 10
	0.0369693192354005	− 1.58	*MIB1*	MIB E3 ubiquitin protein ligase 1
	0.0428609710686184	0.21	*SKI*	SKI proto-oncogene
	0.043587	− 0.29	*SMARCC1*	SWI/SNF related, matrix-associated, actin-dependent regulator of chromatin subfamily c member 1
	0.0435831755630875	0.23	*DAB2*	DAB adaptor protein 2
	0.0465058827027208	− 1.06	*UBA3*	Ubiquitin-like modifier activating enzyme 3
Respiratory disease	0.00309322755884207	− 0.69	ITGA6*	Integrin subunit alpha 6
	0.00579452607644582	− 0.81	*FUBP1*	Far upstream element-binding protein 1
	0.00817443536604729	0.51	*AGER*	Advanced glycosylation end-product-specific receptor
	0.0133236306671656	− 1.27	*ATMIN*	ATM interactor
	0.0352336910664588	0.74	*CA4*	Carbonic anhydrase 4
	0.0418477880132438	− 1.51	*BACH2*	BTB domain and CNC homolog 2
Hematological disease	0.009801833424076	− 0.92	*PDE7A*	Phosphodiesterase 7A
	0.0166001613836398	− 0.78	*LMAN1*	Lectin, mannose-binding 1
	0.0326065857959076	− 0.72	*MCFD2*	Multiple coagulation factor deficiency 2, ER cargo receptor complex subunit
	0.0222521938060266	− 0.76	*F13A1*	Coagulation factor XIII A chain
Mitochondrial biogenesis, free radical scavenging	0.00155622393947997	1.68	OLR1*	Oxidized low density lipoprotein receptor 1
	0.00157141424422093	− 0.04	NOS3*	Nitric oxide synthase 3
	0.00342586086803695	− 1.39	*NADK2*	NAD kinase 2, mitochondrial
	0.00420515005042535	0.70	*ADCY6*	Adenylate cyclase 6
	0.0145472161400915	− 1.36	*OPA1*	OPA1 mitochondrial dynamin-like GTPase
	0.0145472161400915	− 0.43	*PANK2*	Pantothenate kinase 2
	0.0194007739744968	− 0.51	*PRXL2A*	Peroxiredoxin-like 2A
	0.0194007739744968	− 0.35	*SLC25A13*	solute carrier family 25, member 13
	0.0241780091500456	− 0.72	*ACSL3*	Acyl-CoA synthetase long-chain family member 3
	0.0337481033423519	− 0.71	*CPT2*	Carnitine palmitoyltransferase 2
	0.0400303820036726	0.11	*MFN1*	Mitofusin 1
	0.0428609710686184	− 0.49	*NDUFS1*	NADH:ubiquinone oxidoreductase core subunit S1

A total of 273 genes were significant varied (*Bonferroni and/or false discovery rate < 0.05) between BPD (*n* = 6) and non-BPD (*n* = 16) cord blood cells as determined by cDNA microarray analysis (Illumina HumanHT-12 WG-DASL V4.0 R2 expression beadchip). Ingenuity, Reactome, and ToppGene pathway analyses tools used to determine enriched functional categories and pathways. dEx = expression difference (Log2) in BPD relative to non-BPD. Full list of the differentially expressed genes are in Additional file 1: Table S8

Five differentially expressed genes, advanced glycosylation end product-specific receptor (*AGER*), ceroid-lipofuscinosis, neuronal 8 (*CLN8*), family with sequence similarity 50 member B (*FAM50B*), SKI proto-oncogene (*SKI*), transmembrane p24 trafficking protein 7 (*TMED7*), were among those annotated to differentially methylated loci (Table 2 and Additional file 1: Table S4) suggesting epigenetic change might mediate their expression. Pathway analyses determined that among genes mapped to differentially methylated CpGs (Table 2 and Additional file 1: Table S4) were a number of potential upstream transcriptome regulators such as caveolae-associated protein 2 (CAVIN2; *p* value of overlap = 0.0137, downstream target—*NOS3*), zinc finger CCCH-type containing 12C (ZC3H12C; *p* = 0.03, downstream targets—*GPR34* and *PLA2G7*), and retinoblastoma protein 1 (RB1; *p* = 0.127, downstream targets—*ATP1B1*, *CASP4*, *CLIC4*, *GMFB*, *MCM8*, *OLR1*, *OPA1*, *RBL2*, *RMI1*, *SDHC*). Thus, upstream or indirect regulation effects of altered methylation may impact transcriptomics in BPD.

### Differential expressions of BPD cord blood epigenome markers in murine lungs

We determined if genes associated with BPD cord blood epigenome are changed in murine lungs during lung developmental process and in a mouse model of BPD. During saccular stage of lung development (embryonic day E17.5-postnatal day PND4), mRNAs and proteins of mouse SPOCK2 and AGER were highly increased with peaks toward the saccular-to-alveolar transition time at PND4 and thereafter (Additional file 2: Figure S3; Additional file 3: Figure S3B), indicating their roles in alveolar development [49, 50]. Mouse CTSH expression was higher at early saccular stage with mRNA peaks at E17.5–E18.5 and protein peaks at and before E17.5 (Additional file 2: Figure S3; Additional file 3: Figure S3B), supporting its contribution to lung branching, surfactant production and secretion at saccular stage [51, 52]. Hyperoxia exposure upregulated both message and protein levels of SPOCK2, CTSH, and AGER in newborn mouse lungs (Additional file 2: Figure S3; Additional file 3: Figure S3B).

## Discussion

In the current study, we report a number of new observations regarding the epigenetics of preterm GA and birth weight relative to cord blood cell-type composition, including NRBCs, and describe an EWAS analysis comparing a small group of BPD neonates to non-BPD neonates. NRBCs typically compose less than 10% of non-pathologic cells in full term cord blood and are rapidly cleared from the bloodstream after birth [53, 54]. Numerous reports have observed that higher NRBC levels were associated with prenatal complications such as placental dysfunction, intrauterine hypoxia, preeclampsia, asphyxia, and maternal obesity and diabetes in term and preterm infants [54–58] as well as later risk of unfavorable outcomes [59]. Using a methylation-based model to estimate NRBCs, we observed that lower birth weight and GA were associated with high NRBC levels, but considerable variation was observed, with some of the larger and older infants displaying very high NRBC content. NRBC proportions were, however, not associated with BPD. It has been suggested that fetal oxidative stress or hyperoxic stress caused by maternal smoking might be a driver for NRBC formation. A significant correlation between newborn venous NRBC count and the number of cigarettes smoked per day of mothers has been reported [40]. The present study provides support for this hypothesis as we observed a significant correlation between higher cord blood NRBCs and demethylation of *AHRR* cg05575921 (an established biomarker of maternal smoking) [60]. However, in a previous epigenetic study of full-term births we did not observe a significant relationship between maternal smoking and estimated NRBC percent or actual counts [38].

High levels of NRBCs in cord blood can potentially confound DNA methylation studies because they have an unusual genome-wide methylation profile caused by genome-wide DNA demethylation during enucleation [61–63]. For NRBC enucleation and maturation, histone acetylation status-dependent nuclear and chromatin condensation is known to be essential [64]. Consistent with these notions, we found genome-wide demethylation in high-NRBC cord bloods compared to low-NRBC cord bloods (shown in Fig. 2C). Comparing NRBC reference CpGs to cord blood profiles of the non-BPD individuals, we found 3647 significantly associated CpGs (Bonferroni). The present BPD-EWAS used estimated NRBC percentage as a covariate and only a small number of BPD CpGs at FDR1% (6 of 275 CpGs) overlapped with the NRBC-EWAS-associated CpGs (3647 CpGs).

GA together with birth weight are the most important predictors for neonate morbidity and mortality. Many recent studies indicate the association of cord blood DNA methylation profiles with GA at birth [26–31]. In non-BPD samples, we conducted EWAS with covariate adjustment on GA (378 CpGs at Bonferroni) and on birth weight (3 CpGs at Bonferroni) (Additional file 1: Table S9). No CpGs among the EWAS analyses of GA and birth weight were in common with the BPD Bonferroni EWAS CpGs. However, at FDR 1% we found 5 CpGs (cg02236679, cg11791427, cg16762386, cg17514088, cg19595760) overlapped between EWASs on BPD and GA. In addition, we found 225 CpGs overlapped between the present EWAS on GA and two recently published EWAS on GA [26, 27].

Considering the importance of GA in neonatal health outcomes, cord blood DNA methylation has been incorporated into predictive GA models [26, 28], and the discrepancy between GA estimated from DNA methylation (epigenetic maturity, EGA) and clinically recorded (chronological GA, determined by ultrasound or last menstrual period) is termed EGA acceleration [43]. Several studies have reported that lower GA acceleration values (i.e., epigenetically less mature than their clinical GA) were associated with maternal factors such as gestational diabetes in a previous pregnancy, Sjögren’s syndrome, and maternal Medicaid (vs private insurance), as well as postnatal surfactant or steroid use, longer days of assisted ventilation, lower birth weight, and BPD development [28, 44, 65]. More mature or accelerated EGA has been correlated with maternal age of over 40 years, previous pregnancy, preeclampsia, or maternal steroid treatments [28, 44, 65]. In the current study, we measured EGA using models developed by Bohlin [26] and Knight [28]. In each case the EGA models overestimated GA and indicated accelerated EGA (more mature) in neonates that went on to develop BPD, a result opposite of a previous report in which infants with accelerated EGA were less likely to develop BPD [65]. While the present study is limited by size, the determination of EGA acceleration and its relationship to developmental and perinatal factors and adverse respiratory outcomes would appear to need more study.

Epigenetic drift that leads to stochastic epimutation is a recent concept defined as outlier methylation levels at a genomic position relative to the interquartile range of methylation values determined for all samples in a dataset. It has been proposed that SEMs reflect loss of epigenetic regulation and may be involved in aging and carcinogenesis [66]. Spada et al. [42] observed in a small study that total SEM burden (both hypomethylated and hypermethylated SEM) was significantly greater in preterm cord blood samples relative to full-term, and proposed weak maintenance of epigenetic state in preterm blood might be related to risk of disease in later life. Epimutation load levels (SEM load per individual) in the current study were strongly associated with a number of covariates in univariate analysis including birth weight, several cell types, and days of oxygen therapy, all covariates that were strongly associated with altered methylation profiles. Indeed, in both unadjusted (*p* = 5.36E−09) and adjusted (*p* = 1.89E−06) regression analysis, NRBC estimates were strongly associated with EMLs. Adjusting for all covariates in multivariable regression on BPD status, we observed a strongly attenuated level of significance (*p* = 0.03) for difference in EML among BPD infants relative to non-BPD infants suggesting a very large impact of cell-type composition. Spada et al. [42] reported that SEMs in cord blood occurred at CpG sites, and genes (*NCOR2*, *PLCH1*, *FOXK1*, and *IGF2BP1*) that were in common with those annotated to EWAS CpGs on preterm birth; however, these analyses were not adjusted for NRBC percentages. While the biological significance and source of stochastic epimutation in preterm cord blood are largely unknown, our finding that a very large proportion of CpGs (30%) that we observe as SEMs were among the CpGs observed as NRBC Bonferroni CpGs suggests that determination of SEMs may be strongly confounded by cell-type composition.

It is unknown if epigenetic profiles in cord blood cells represent or mirror those in the neonatal lung developing BPD; however, the study of Merid et al. [27] reported that 78 CpGs overlapped between GA EWAS analyses in cord blood and fetal lung tissues, suggesting this may be the case. The EWAS result in the present work revealed 275 CpGs significantly associated with BPD risk at 1% FDR. Examining the genes annotated to these CpGs, we found potentially important signal transduction pathways and biological functions related to BPD risk, including lung development-associated pathways and functions.

The lung is an active organ for platelet activation and a pool for hematopoietic progenitor cells that can migrate to and repopulate the bone marrow and contribute to hematopoietic lineages in blood [67]. One of the hematopoietic pathways associated with the BPD epigenome was angiogenesis and platelet activation (Fig. 5), and genes associated with runt-related transcription factor 1 (RUNX1), AHR and VEGF receptor pathways were predicted to play key roles. Regarding genes associated with lung development, *CTSH* was annotated to the most significant CpG associated with BPD (cg23328237) and was reported to be differentially methylated and expressed in BPD lungs compared to control lungs [33]. CTSH plays a role in surfactant production [51] and bone morphogenetic protein 4 (BMP4)-mediated lung branching [52]. The same CpG is annotated to *RASGRF1*, a gene that has been associated with BPD in GWAS [18]. We found several annotated genes that are critical to lung glycosaminoglycan metabolism and contribute to alveolarization and hematopoiesis [68]. *SPOCK2*, the other GWAS-determined BPD susceptibility gene, was upregulated in a rat lung model of BPD [13] and use of lung-specific *SPOCK2* overexpression mice demonstrated its deleterious role in BPD development [49]. In addition, *AGER*, a specific alveolar type 1 cell differentiation marker [50], and *HS6ST1* encoding heparin sulfate 6-O-sufotransferase 1 [69] involve in alveolar development. BPD epigenome-annotated genes were also associated with androgen receptor signaling which delayed alveolar maturation and increased respiratory morbidity in preterm male infants [70], suggesting a molecular basis of male susceptibility of newborn pulmonary morbidity and possibly BPD [71]. Vitamin A regulates lung growth (e.g., branching, proximal–distal patterning, alveolar septation, surfactant production) and supports the immunity and repair of injured respiratory epithelium [72, 73]. Among the RAR-RXR and RXR-vitamin D receptor (VDR) signaling genes enriched, *NCOR2* is a transcriptional corepressor in various developmental signaling [74] and has been a commonly determined gene in multiple EWAS for GA prediction [26, 27] as well as in epigenetic mutation CpG in preterm birth [42]. It is also known to affect later life lung function in chronic respiratory conditions [75]. BPD has also been associated with vitamin D receptor (*Fok1*) polymorphisms [76] and with lower levels of 25-hydroxyvitamin D in preterm infants [77, 78]. Overall, pathway analyses indicated that epigenome changes in cord blood immune and progenitor cells may in part anticipate the lung pathogenesis in BPD patients.

There are several limitations to this study, the most obvious being the small number of BPD patients included in the analysis and this restricts interpretation to the generation of new hypotheses. The logistics of obtaining cord blood samples from extremely low gestational age neonates are challenging and contributed to limiting this study to a pilot scale rather than a full-size investigation. Some studies of preterm chronic lung disease have observed that inflammatory conditions such as chorioamnionitis are associated with changes in cell-type counts, which might be indicators of susceptibility to BPD [79]. Study size limitation and availability of data did not allow investigation of this question. At the time of the enrollment of patients, it was not known that leukocyte cell counts, NRBC counts and cell-type proportions would be important covariates in an EWAS and some potentially useful data was not abstracted from original medical records. The inclusion of estimated cell-type proportions as covariates has been the standard approach to reduce the possibility of confounding by leukocyte cell-type variability. The highly dynamic nature of hematopoiesis in the developing fetus, including the presence of DNA from NRBCs in cord blood, make DNA methylation studies in preterm neonates a challenge. The present study is one of only a very small number of published epigenetic studies of preterm birth and BPD, and this epigenetic exploration of cell-types in relationship to GA, birth weight, and BPD provides a unique view of the developing neonate.

## Conclusions

Although limited by small sample size, the current investigation provides an exploratory basis for examining potential cord blood DNA methylation biomarkers of BPD risk in preterm infants and offers descriptive comparisons between methylation profiles and various preterm phenotype variations. Further studies are needed to determine if the CpGs identified here will prove to be clinically relevant. A future project using epigenomic and transcriptomic profiling of preterm infant blood in the early weeks of life will examine the persistence of the results described here.

## Methods

### Study cohort

The Discovery-Bronchopulmonary Dysplasia Program (D-BPD) cohort is described in detail elsewhere [80]. Briefly, 378 preterm infants under 1500 g of birth weight in Buenos Aires, Argentina, were recruited within 13 days of life and followed prospectively in the NICU until discharged or 44 weeks of corrected GA. Inclusion criteria were: born alive at any of the four participating hospitals at ≤ 35 weeks’ gestation; with very low birth weight (< 1500 g at birth); and free of cyanotic heart disease, congenital anomalies of the respiratory tract (i.e.: tracheoesophageal fistula, pulmonary hypoplasia, diaphragmatic hernia), ocular malformations, congenital immune suppression, or severe malformations affecting breathing or vision (i.e. anencephaly). Infants who died prior to completion of all the first maternal questionnaire were excluded from participation. A total of 107 patients (14 BPD, 93 non-BPD) who satisfied all study inclusion criteria provided a cord blood sample and had a successful methylation array. BPD was diagnosed for infants who received at least 28 days of O_2_ (> 21%) supplementation therapy and need for O_2_ (≥ 30%) and/or positive pressure (1) at 36 weeks of PMA or at discharge (whichever comes first) if born < 32 weeks GA or (2) at 28–56 days postnatal age or at discharge (whichever comes first) if born ≥ 32 weeks GA [4]. However, two infants receiving 14 or 22 days of O_2_ at the time of their death were diagnosed with severe BPD. The study was approved by the local Institutional Review Board (IRB) and the NIEHS (08-E-N159). Parents provided written informed consent.

### Genomic DNA and total RNA extraction from cord blood

Umbilical cord blood samples were collected at birth in PAXgene reagent (Qiagen Inc., Valencia, CA) and snap frozen at − 80 °C. Samples were processed with PAXgene Blood miRNA Kit (PreAnalytix/Qiagen) following the manufacturer’s procedure. Briefly, blood specimens were incubated at room temperature for 2 h to lyse RBCs and centrifuged (3500*g*, 15 min) to acquire cell pellets. The pellets were washed and treated with proteinase K at 55 °C (800 rpm, 15 min), and isopropanol was added to the soluble fractions of the supernatants prepared from the Shredder spin columns. For RNA isolation, the isopropanol precipitants were added into the PAXgene RNA spin columns and processed for DNase treatment followed by RNA extraction procedures as indicated in the manufacturer’s brochure. For DNA isolation, the isopropanol precipitants prepared with the PAXgene miRNA Kit were loaded into the DNeasy Mini spin columns (DNeasy Blood and Tissue Kit, Qiagen) and followed the manufacturer’s procedure. DNAs and RNAs were quantified using Qubit (Thermo Fisher, Waltham, MA) and stored at − 70 °C until used.

### DNA methylation microarray analysis

Aliquots (250 ng) of genomic DNA from BPD (*n* = 14) and non-BPD (*n* = 93) cord blood cells were bisulfite-converted using a Zymo EZ DNA Methylation (batch 1, 8 BPD, 43 non-BPD) and EZ-96 DNA Methylation MagPrep (batch 2, 6 BPD, 60 non-BPD) kits (Zymo Research, Irvine, CA) which use identical reagents following the manufacturer’s instructions. Briefly, all samples were bisulfite converted in a thermocycler with the following conditions: 16 cycles of 95 °C for 30 s followed by a 50 °C hold for 60 min. Cleanup of converted product was then wither done on a column or with magnetic beads using the same kit reagents. Bisulfite-converted DNAs were applied to HumanMethylation450 BeadChip (Illumina, San Diego, CA) which covers over 480,000 CpG sites in human genome for genome-wide DNA methylation array analysis. The raw IDAT files of methylation arrays were read into R with the minfi package [81], and the data were preprocessed with background correction and dye-bias normalization using the preprocessNoob method [82]. The combat function in sva package [83] was used to do batch (‘Sample_Plate’) correction on methylation array data. Prior to normalization, DNA methylation data were filtered based on the following quality control criteria, exclusion of: arrays having more than 5% failed probes (1 array); all CpG probes on the X and Y chromosomes; and any probes containing single-nucleotide polymorphism with a minor allele frequency ≥ 1% (in EUR population of the 1000 Genomes Project) within 5 nucleotides to the CpG site. We also removed 43,254 probes reported to hybridize to one or more non-target sites in the genome [84]. There were 447,246 CpG probes remaining after exclusions. Differentially methylated probes were identified by a robust linear regression analysis of M values (log ratio of beta values) on disease status (BPD vs non-BPD) with adjustment for infant sex, GA (weeks), birth weight (g), ancestry, maternal smoking status, seven estimated blood cell-type proportions, hospital at birth, and days in which oxygen supplementation was used in newborn intensive care unit. Days of O_2_ supplementation were transformed to a percentile using empirical percentile transformation method (R function ‘percentize’ in heatmaply package, https://www.rdocumentation.org/packages/heatmaply/versions/1.2.1/topics/percentize). In order to minimize the impact of outliers on the differential methylation results, a Winsorized methylation (https://www.rdocumentation.org/packages/DescTools/versions/0.99.44/topics/Winsorize) dataset was created and robust linear regression with adjustment was repeated. DNA methylation array data are deposited in Gene Expression Omnibus (GEO, accession number: GSE188944).

### Methylation-based cord blood cell-type prediction

Percentages of seven blood cell types (CD4^+^ T cells, CD8^+^ T cells, B cells, monocytes, granulocytes, natural killer cells, and NRBCs) were estimated using the reference DNA methylation profiles (R package ‘FlowSorted.CordBlood.450 k’ [85]) and Houseman deconvolution algorithm [86]. To identify additional CpGs associated with NRBCs, we assessed the association between NRBC reference CpGs and cord blood methylation profiles in non-BPD neonates.

### SEM calculation

The calculation of SEM was carried out as in a previously published and validated approach [42, 45]. An individual CpG having a methylation level three times the interquartile range above the third quartile or below the first quartile was identified as a SEM. Toward this end, we calculated the interquartile range (IQR) for each of the 447,246 probes. Then, SEMs were identified based on extreme methylation levels. Finally, we summed across the count of all SEMs per sample and defined the total number of SEMs of each study participant as EML. EML was not normally distributed; therefore, we used the natural log of the number of SEMs for all statistical analyses.

### EGA estimation and EGA acceleration

We calculated DNA methylation-based GA using two different prediction methods [26, 28] and Horvath’s method for EGA acceleration [43]. The difference between the residual of the linear regression of methylation-based GA and clinically determined gestation age is referred to as EGA acceleration. Positive EGA acceleration was defined as a greater (or older) DNA methylation GA than clinical GA; negative EGA acceleration was defined as a lower (or younger) DNA methylation GA than clinical GA.

### cDNA microarray analysis

Total RNA isolated from cord blood of individuals available at the time of the initial study in 2012 (6 BPD, 16 non-BPD) was amplified, labeled, and fragmented according to the manufacturer’s protocol (NuGEN Technologies, Inc., Redwood City, CA) and applied to Illumina HumanHT-12 WG-DASL V4.0 R2 Gene Expression BeadChip targeting > 47,000 transcripts (Illumina, San Diego, CA) in the NIEHS Microarray core facility. Differentially expressed genes were detected using log2-transformed expression fold-change estimates with respect to the Robust Multichip Average-corrected fluorescence log-intensity levels (log_2_FC). Probe-wise log_2_FC values were tested across statistical groups through a resolution-weighted ANOVA. Significance level was accepted at *p* < 0.05 adjusted for multiple comparisons. Microarray data are deposited in GEO (accession number: GSE188949).

### Pathway analyses

Enriched biological processes, functions and diseases, and canonical pathways for the genes associated with the differentially methylated sites (DNA methylation array) or the differentially expressed genes (cDNA microarray) were analyzed using ToppGene Suite (https://toppgene.cchmc.org), Ingenuity Pathway Analysis (IPA, Qiagen Inc., Valencia, CA), David Bioinformatics Resources (https://david.ncifcrf.gov), and Reactome Pathway Database (https://reactome.org). R package missMethyl performed GOmeth [87, 88] on GO and KEGG databases to take into account the different number of CpG probes per gene and multiple gene-annotated CpGs in the pathway analysis.

### Developmental mouse studies

Gene and protein expressions were determined in total RNAs and proteins isolated from lungs of CD-1 mice (Charles River, Wilmington, MA) harvested at E13.5, 15,0.5 and 17.5, and PND 0, 1, 4, 14, and 42, or after exposure to air or hyperoxia during PND 1–4 as previously described in detail [89]. All animal use was approved by the NIEHS Animal Care and Use Committee. Total lung proteins were prepared from right lung homogenates (*n* = 3/group) in RIPA buffer including PMSF (10 μg/ml) and protease/phosphatase inhibitor cocktail (Sigma-Aldrich, St. Louis, MO). Proteins were quantified and 80 μg of pooled proteins were separated on 10–20% Tris–HCl SDS-PAGE gels (Bio-Rad Laboratories, Hercules, CA) and analyzed by Western blotting using mouse-specific antibodies against SPOCK2 (R&D Systems Inc., Minneapolis, MN), CTSH (LSBio, Seattle, WA), AGER (Santa Cruz Biotechnology Inc, Dallas, TX) and β-ACTIN (Santa Cruz Biotechnology) (Additional file 2: Figure S3; Additional file 3: Figure S3B). The assay was done duplicates. An aliquot of total RNA isolated from mouse lungs (*n* = 3/group) was reverse transcribed into cDNAs, and cDNA (40 ng) was subjected to quantitative PCR in 20 μl reaction containing 0.5 μmol of commercially available (Real Time Primers, LLC, Elkins Park, PA) cDNA primers for *Spock2*, *Ctsh*, and *Ager* using an CFX Connect Realtime System (Bio-Rad) as previously described [89]. The relative quantification of target gene expression was calculated using the comparative quantification cycle (*C*_q_) method by subtracting fluorescence detected *C*_q_ of 18 s rRNA (5′-tacctggttgatcctgccag-3′, 5′-ccgtcggcatgtattagctc-3′) from that of target gene in the same sample (Δ*C*_T_).

### Other statistical analyses

Association between neonatal or maternal characteristics and clinical outcomes, GA and birth weight, and GA and supplemental O_2_ days were analyzed by linear regression analyses (GraphPad Prism 9, GraphPad Software, San Diego, CA). One-way ANOVA or two-way ANOVA were used to evaluate the relationship between developmental age or neonatal exposure on mouse gene expressions determined by qRT-PCR. Student–Newman–Keuls test was used for a posteriori comparison of means (*p* < 0.05). Statistical analyses of qRT-PCR data were performed using SigmaPlot 14.0 program (Systat Software, San Jose, CA).

## Supplementary Information

**Additional file 1**. **Table S1.** Cohort demographics by bronchopulmonary dysplasia (BPD) status (*n* = 107). **Table S2.** Distribution of cord blood cell types and methylation status of a tobacco smoke epigenetic biomarker in neonates with or without bronchopulmonary dysplasia (BPD). **Table S3**. Differentially methylated CpG sites associated with nucleated red blood cell (NRBC) counts. **Table S4.** Differentially methylated CpG sites associated with bronchopulmonary dysplasia (BPD) risk and their annotated gene information. **Table S5.** The number of probes identified by differential methylation analysis. **Table S6.** Association of stochastic epimutation load (EML) with bronchopulmonary dysplasia (BPD) and other covariates. **Table S7A.** Biological functions and pathways of the genes annotated to the differentially methylated CpG sites (FDR 5%) associated with bronchopulmonary dysplasia (BPD) risk. **Table S7B.** GOmeth enriched GO terms on CpGs associated with BPD risk. **Table S7C.** GOmeth enriched KEGG pathways on CpGs associated with BPD risk. **Table S8.** cDNA microarray determined differentially expressed genes in cord blood cells of infants with bronchopulmonary dysplasia (BPD). **Table S9**. DNA CpG methylation sites associated with gestational age (GA) or birth weight (BW).

**Additional file 2**. **Figure S1.** DNA methylation of aryl-hydrocarbon receptor repressor (*AHRR*) CpG (cg23953254) shows minimal association NRBC or smoking. **Figure S2.** Epigenetic estimation of gestational age (GA) and GA acceleration in bronchopulmonary dysplasia (BPD). **Figure S3.** Expression of bronchopulmonary dysplasia (BPD) epigenome-associated genes in mouse lung tissues.

**Additional file 3**. Western Blot Raw Images

## Data Availability

The datasets analyzed during the current study are available in the Gene Expression Omnibus repository (https://www.ncbi.nlm.nih.gov/gds, GSE188944 and GSE188949). The other datasets generated or analyzed during the current study are included in this article and its supplementary information files or available from the corresponding author on reasonable request.

## Abbreviations

AGER: Advanced glycosylation end product-specific receptor
AHRR: Aryl hydrocarbon receptor repressor
BNP4: Bone morphogenetic protein 4
BPD: Bronchopulmonary dysplasia
CAVIN2: Caveolae-associated protein 2
CLN8: Ceroid-lipofuscinosis, neuronal 8
CTSH: Cathepsin H
E: Embryonic day
ECM: Extracellular matrix
EGA: Epigenetic gestational age
EML: Epigenetic mutation load
EMT: Epithelial-mesenchymal transition
FAM50B: Family with sequence similarity 50 member B
FDR: False discovery rate
GA: Gestational age
GEO: Gene Expression Omnibus
GO: Gene Ontology
GWAS: Genome-wide association study
IPA: Ingenuity Pathway Analysis
IQR: Interquartile range
KEGG: Kyoto Encyclopedia of Genes and Genomes
MAEA: Macrophage erythroblast attacher
NCOR2: Nuclear receptor corepressor 2
NICU: Neonatal intensive care unit
NIEHS: National Institute of Environmental Health Sciences
NIH: National Institutes of Health
NRBC: Nucleated red blood cell
O_2_: Oxygen
PMA: Postmenstrual age
PND: Postnatal days
RAR: Retinoic acid receptor
RASGRF1: Ras protein-specific guanine nucleotide releasing factor 1
RB1: Retinoblastoma protein 1
RXR: Retinoic acid (RA)/retinoid X receptor
SEM: Stochastic epigenetic mutation
SKI: SKI proto-oncogene
SPOCK2: SPARC (osteonectin), Cwcv, and Kazal-like domains proteoglycan 2
TMED7: Transmembrane p24 trafficking protein 7
VDR: Vitamin D receptor
VEGF: Vascular endothelial growth factor
ZC3H12C: Zinc finger CCCH-type containing 12C
