# Supplementary material for: Epigenome-wide association study of bronchopulmonary dysplasia in preterm infants: results from the discovery-BPD program
Source: Clin Epigenetics. 2022 Apr 28;14:57. doi: 10.1186/s13148-022-01272-0 (PMC9052529; doi:10.1186/s13148-022-01272-0)
Supplement: Supplementary file 1 — Additional file 1. Table S1. Cohort demographics by bronchopulmonary dysplasia (BPD) status (n = 107). Table S2. Distribution of cord blood cell types and methylation status of a tobacco smoke epigenetic biomarker in neonates with or without bronchopulmonary dysplasia (BPD). Table S3. Differentially methylated CpG sites associated with nucleated red blood cell (NRBC) counts. Table S4. Differentially methylated CpG sites associated with bronchopulmonary dysplasia (BPD) risk and their annotated gene information. Table S5. The number of probes identified by differential methylation analysis. Table S6. Association of stochastic epimutation load (EML) with bronchopulmonary dysplasia (BPD) and other covariates. Table S7A. Biological functions and pathways of the genes annotated to the differentially methylated CpG sites (FDR 5%) associated with bronchopulmonary dysplasia (BPD) risk. Table S7B. GOmeth enriched GO terms on CpGs associated with BPD risk. Table S7C. GOmeth enriched KEGG pathways on CpGs associated with BPD risk. Table S8. cDNA microarray determined differentially expressed genes in cord blood cells of infants with bronchopulmonary dysplasia (BPD). Table S9. DNA CpG methylation sites associated with gestational age (GA) or birth weight (BW). [file 13148_2022_1272_MOESM1_ESM.pdf]

Table S1. Cohort demographics by bronchopulmonary dysplasia (BPD) status (n=107).

Characteristics	BPD	NonBPD
**Neonate characteristics**		
Sample size	14 [a]	93
Birth weight (g)	925.9 ± 79.7	1187.9 ± 25.2
Gestation age (Weeks)	27.6 ± 0.7	30.3 ± 0.2
Gender	Male 9<br>Female 5	Male 31<br>Female 62
Cumulative NICU O_2 (Days)	47.1 ± 5.2	7.8 ± 1.1
Delivery room resuscitation O_2 only	0	27/93 (29%)
Delivery room surfactant	1/12 (8%)	4/86 (5%)
Respiratory distress diagnosis for NICU	9/12 (75%)	41/86 (48%)
Multiple birth	2/14 (14%)	28/93 (30%)
**Maternal characteristics and pregnancy complications**		
Maternal ace		
European-Latin	11	68
Criollos	0	14
European-other	0	5
Jewish	1	3
Arab-Middle Eastern	1	0
African Caribbean	0	1
Native American	1	0
Asian	0	1
Unknown or Refused	0	1
Maternal age (Year)	32.2 ± 1.9	34.1 ± 0.5
Cesarean section	10/12 (83%)	84/87 (97%)
Maternal smoking history [b]	7/14 (50%)	24/93 (27%)
Maternal antenatal steroid	13/13 (100%)	82/90 (91%)
Maternal pre-eclampsia	3/14 (21%)	25/93 (27%)
IUGR-reason for delivery	5/12 (42%)	33/90 (37%)
BMI	33.6 ± 1.8	34.1 ± 5.2
Education [c]	9/4/0/1	63/20/5/5
Employed first trimester	10/13 (77%)	69/88 (78%)
Alcohol use during pregnancy	0	1/88 (1%)
Chorioamnionitis	2/12 (17%)	6/85 (7%)
Asthma	0	0
Other respiratory disease	0	0
Gestational diabetes	0	1/90 (1%)
Previous preterm pregnancy	9/13 (69%)	50/90 (56%)
Assisted reproduction	2/13 (15%)	27/90 (30%)
**Fetal complications**		
Fetal IUGR	5/12 (42%)	38/88 (43%)
Fetal oligohydramnios	3/12 (25%)	11/88 (13%)
Fetal decelerations	1/12 (8%)	1/88 (1%)
Fetal bradycardia	1/12 (8%)	1/88 (1%)

Samples recruited from the Discovery-Bronchopulmonary Dysplasia Program (D-BPD) cohort in Buenos Aires, Argentina. Mean ± S.E.M. presented. NICU= newborn intensive care unit. O_2= oxygen supplement. IUGR= intrauterine growth restriction. BMI= body mass index. [a] One neonate with BPD deceased at 14 days of life. [b] Self-reported former and/or current smoker. [c] Higher/Secondary/Primary/NA.

Table S2. Distribution of cord blood cell types and methylation status of a tobacco smoke epigenetic biomarker in neonates with or without bronchopulmonary dysplasia (BPD).

Sample_Name	Sample_Group	Sex	Birth weight (g)	GA (weeks)	CD8T (%)	CD4T (%)	NK (%)	B cell (%)	Mono (%)	Gran (%)	NRBC (%)	*cg05575921 methylation (%)	Hospital code	Cumulative Days O2
1007_1	**BPD**	M	839	26.5	2.47	2.89	0.07	0.00	0.00	0.00	97.16	66.72	1	53
1009_2	**BPD**	M	1418	30	22.58	27.34	0.00	24.28	10.91	13.81	10.87	82.11	1	53
1012_1	**BPD**	M	1321	32	27.80	19.46	6.90	11.86	4.57	10.50	22.48	83.81	1	57
1013_1	**BPD**	M	1010	29	21.75	14.03	0.00	26.40	7.03	24.11	9.29	82.28	1	59
1014_1	**BPD**	F	787	26.1	8.97	6.58	2.60	14.24	11.85	40.05	24.79	87.28	1	14
1037_1	**BPD**	M	822	25	13.32	16.72	0.07	12.25	12.94	32.68	17.32	75.26	1	66
2002_1	**BPD**	F	1385	31	22.37	50.13	0.00	8.66	1.75	8.07	14.30	80.57	2	30
2003_2	**BPD**	M	724	27.5	12.16	9.43	0.00	5.08	0.00	0.00	78.82	76.08	2	41
2012_1	**BPD**	M	1022	28	18.21	27.11	0.00	18.18	13.83	19.27	12.14	84.28	2	22
2014_1	**BPD**	M	580	26	6.71	2.94	1.75	1.71	0.75	0.00	94.83	69.09	2	49
2017_1	**BPD**	F	519	25	7.43	5.60	0.00	5.83	1.35	0.00	89.07	69.16	2	53
3013_1	**BPD**	F	950	26	5.04	6.46	3.74	7.56	11.65	54.30	18.46	86.96	3	48
3017_1	**BPD**	M	875	29.8	8.95	21.79	5.40	18.39	10.66	11.27	14.62	86.42	3	45
4005_1	**BPD**	F	710	24	9.66	3.00	0.95	8.70	11.35	61.60	7.74	68.93	4	70
1001_1	**nonBPD**	M	1397	28	11.03	10.82	0.71	10.05	14.71	44.79	15.67	84.39	1	0
1002_1	**nonBPD**	F	1152	29	9.50	8.08	0.50	10.43	5.16	14.20	60.09	70.52	1	1
1003_1	**nonBPD**	F	1455	31	16.01	29.28	0.00	17.45	7.86	24.58	6.77	91.99	1	5
1004_1	**nonBPD**	F	1205	28.2	15.39	18.84	1.55	14.65	4.98	5.60	49.22	74.67	1	12
1005_1	**nonBPD**	F	1230	32	13.72	12.45	1.20	4.31	2.98	2.74	68.17	74.66	1	1
1006_1	**nonBPD**	F	1360	28.5	26.03	28.36	0.00	13.61	8.29	16.11	17.77	84.75	1	8
1009_1	**nonBPD**	F	1352	30	12.55	23.52	0.00	12.06	3.91	4.46	49.17	72.66	1	1
1010_1	**nonBPD**	F	1075	30.5	18.73	23.03	0.00	20.48	9.68	17.38	20.21	83.14	1	0
1011_1	**nonBPD**	F	714	30.3	19.94	32.90	0.00	14.27	3.94	2.00	31.26	76.80	1	21
1015_1	**nonBPD**	M	1253	29	19.28	30.45	0.00	16.66	5.28	4.70	28.54	86.49	1	22
1016_1	**nonBPD**	F	878	32	15.06	8.31	2.83	2.51	7.34	57.32	7.84	91.44	1	0
1017_1	**nonBPD**	F	1391	33.5	13.59	20.56	0.00	12.06	4.95	42.76	10.59	84.85	1	0
1018_1	**nonBPD**	F	687	30	21.12	38.06	0.00	2.14	0.14	2.87	39.62	81.64	1	5
1019_1	**nonBPD**	F	855	25	14.44	15.40	0.00	9.79	9.38	21.40	36.35	80.94	1	46
1020_1	**nonBPD**	F	1417	32	17.50	39.74	0.00	13.09	9.84	12.07	12.94	88.60	1	7
1021_1	**nonBPD**	F	1269	34	19.66	13.60	2.90	13.70	9.65	28.25	17.57	86.43	1	5
1022_1	**nonBPD**	F	1134	29	16.98	17.46	0.00	12.19	7.76	30.28	20.71	87.17	1	3
1023_1	**nonBPD**	M	1093	30	19.86	30.48	0.00	16.75	5.42	7.92	25.61	87.08	1	2
1024_1	**nonBPD**	F	789	26	12.79	8.41	0.00	13.40	12.15	22.82	39.74	81.40	1	0
1025_1	**nonBPD**	F	870	26	22.01	20.15	0.00	16.23	5.18	12.25	28.58	84.63	1	0
1026_1	**nonBPD**	F	1306	30.2	15.00	31.39	0.00	11.89	8.80	28.15	8.93	87.35	1	0
1027_1	**nonBPD**	M	1344	30.2	15.70	36.08	0.00	13.69	7.56	20.76	9.72	85.56	1	0
1028_1	**nonBPD**	M	850	28	5.62	4.89	0.00	0.25	0.00	0.00	96.08	72.21	1	5
1029_1	**nonBPD**	M	736	29	19.32	32.99	0.00	11.89	13.45	13.89	12.96	84.94	1	19
1029_2	**nonBPD**	M	863	29	23.17	29.96	0.00	12.02	12.09	12.03	15.64	85.25	1	14
1030_1	**nonBPD**	F	1453	30.6	18.67	15.63	0.00	16.24	7.56	22.58	24.29	84.71	1	15
1030_2	**nonBPD**	F	1221	30.6	23.42	28.86	0.00	17.06	5.35	18.89	9.90	84.92	1	1
1031_1	**nonBPD**	F	1100	31	4.76	4.34	0.00	0.00	1.13	0.00	95.58	68.51	1	0
1032_1	**nonBPD**	F	1285	31	19.82	27.36	0.00	21.15	11.04	16.35	7.80	87.60	1	3
1033_1	**nonBPD**	F	1353	32	15.10	31.92	0.00	22.99	8.91	8.18	17.61	87.07	1	21

Sample_Name	Sample_Group	Sex	Birth weight (g)	GA (weeks)	CD8T (%)	CD4T (%)	NK (%)	B cell (%)	Mono (%)	Gran (%)	NRBC (%)	*cg05575921 methylation (%)	Hospital code	Cumulative Days O2
1034_2	**nonBPD**	M	1389	33	26.60	29.22	0.00	12.10	6.17	17.86	11.72	77.44	1	0
1035_1	**nonBPD**	F	1176	33	16.52	29.22	0.00	12.25	5.17	19.06	21.96	83.81	1	5
1038_1	**nonBPD**	M	1316	30	11.08	12.68	0.00	15.10	12.13	18.33	35.90	81.24	1	5
1039_1	**nonBPD**	F	1488	32	15.13	19.93	0.00	19.07	9.51	23.26	19.09	80.47	1	6
1040_1	**nonBPD**	F	1313	31	16.06	41.85	0.00	16.46	7.14	8.46	15.43	79.32	1	10
2003_1	**nonBPD**	F	1292	27.5	17.98	26.50	0.00	19.34	5.63	4.26	36.06	79.68	2	8
2004_1	**nonBPD**	F	930	29	18.78	18.35	0.00	22.66	12.08	13.79	21.86	83.79	2	0
2005_1	**nonBPD**	F	1397	30	31.68	35.61	0.00	19.99	1.14	5.42	13.14	86.34	2	1
2005_2	**nonBPD**	F	890	30	27.46	11.70	0.46	11.89	3.74	4.07	49.34	76.00	2	0
2006_1	**nonBPD**	F	1305	31	20.98	19.20	0.32	2.36	0.00	0.00	64.49	75.90	2	0
2008_1	**nonBPD**	F	986	32	16.60	34.42	0.00	18.34	3.58	10.47	21.66	82.09	2	0
2009_1	**nonBPD**	F	1290	33	19.06	39.68	0.00	14.02	5.24	18.19	10.12	85.39	2	0
2011_1	**nonBPD**	M	1400	28	10.11	9.41	0.63	12.52	15.06	48.60	11.56	85.69	2	0
2013_1	**nonBPD**	F	1482	35	22.76	20.51	0.00	10.60	8.06	32.21	10.75	85.52	2	0
2015_1	**nonBPD**	F	1266	31	13.92	29.89	0.00	15.90	5.82	11.11	28.59	80.81	2	7
2016_1	**nonBPD**	M	1400	30.5	15.32	30.07	0.00	20.31	9.34	21.28	9.19	91.22	2	2
2018_1	**nonBPD**	F	1274	30	8.61	17.87	4.42	7.83	9.59	33.12	24.85	87.88	2	3
2019_1	**nonBPD**	M	963	27	16.36	18.72	0.00	19.69	10.36	28.67	10.43	88.47	2	23
2020_1	**nonBPD**	M	1180	32	5.94	10.25	0.00	3.31	0.29	0.00	86.52	66.43	2	1
2021_1	**nonBPD**	F	834	28	14.75	25.45	0.00	21.47	10.80	13.70	18.30	85.79	2	25
2021_2	**nonBPD**	M	950	28	18.33	31.40	0.07	18.82	10.64	11.54	13.84	84.54	2	28
2022_1	**nonBPD**	M	1420	29.5	15.33	36.24	0.00	19.05	6.03	10.55	17.21	84.54	2	0
2022_2	**nonBPD**	M	1462	29.5	18.19	38.32	0.00	20.33	5.65	6.75	16.20	92.37	2	4
2023_1	**nonBPD**	M	1378	32.5	17.28	27.48	0.00	13.50	12.25	22.22	15.25	83.10	2	0
2024_1	**nonBPD**	F	601	29	9.19	8.06	0.00	3.19	0.02	0.00	83.60	67.29	2	2
2025_1	**nonBPD**	F	1000	30	17.16	19.04	0.00	17.45	13.22	24.01	13.96	79.62	2	4
2026_1	**nonBPD**	F	1258	29	13.89	23.48	0.00	17.72	6.11	34.33	8.94	91.04	2	4
2026_2	**nonBPD**	F	1322	29	15.44	25.73	0.00	19.71	5.97	17.95	22.16	88.40	2	4
2027_1	**nonBPD**	F	850	27	11.15	9.26	1.13	9.09	4.92	11.11	61.34	85.96	2	10
3001_1	**nonBPD**	M	860	30	25.05	17.94	0.21	8.24	2.36	0.29	55.00	77.48	3	31
3002_1	**nonBPD**	F	705	25.5	5.72	11.64	0.00	10.68	10.53	30.90	36.26	79.70	3	46
3003_1	**nonBPD**	M	1430	28.4	20.85	17.06	0.00	10.04	10.22	28.26	17.29	79.42	3	17
3004_1	**nonBPD**	F	1490	30.3	13.57	26.19	0.00	12.89	2.72	5.62	44.18	79.61	3	7
3005_1	**nonBPD**	F	1400	33.8	17.92	25.86	0.00	23.11	5.21	12.45	20.26	80.19	3	0
3006_1	**nonBPD**	M	1300	31.3	19.54	25.18	0.00	12.68	8.15	32.38	9.61	81.88	3	0
3007_1	**nonBPD**	M	1440	30.5	16.72	22.59	0.00	12.88	10.79	13.69	30.03	76.66	3	3
3008_1	**nonBPD**	M	1350	34.1	14.95	14.28	0.00	6.15	1.72	4.38	64.01	73.33	3	1
3009_1	**nonBPD**	F	1430	31	11.97	37.47	0.00	17.83	7.53	14.58	14.70	85.11	3	0
3009_3	**nonBPD**	F	1490	31	14.82	49.69	0.00	13.23	3.91	14.79	7.18	88.98	3	0
3010_1	**nonBPD**	F	1170	32.2	30.24	25.94	1.82	21.43	3.05	3.38	22.98	79.52	3	5
3010_2	**nonBPD**	F	1360	32.2	28.73	25.00	2.65	14.54	2.24	5.87	25.62	79.24	3	0
3011_1	**nonBPD**	F	890	29.5	18.03	22.25	1.14	14.52	14.29	24.13	14.52	83.06	3	29
3011_2	**nonBPD**	F	730	29.5	25.36	15.11	5.38	13.49	9.49	14.27	27.24	84.56	3	2
3014_1	**nonBPD**	F	1350	32.8	15.18	31.32	0.00	16.53	5.41	21.18	16.66	83.40	3	0
3015_1	**nonBPD**	F	1400	31	9.96	23.16	0.00	14.97	7.87	24.89	23.22	78.65	3	6

Sample_Name	Sample_Group	Sex	Birth weight (g)	GA (weeks)	CD8T (%)	CD4T (%)	NK (%)	B cell (%)	Mono (%)	Gran (%)	NRBC (%)	*cg05575921 methylation (%)	Hospital code	Cumulative Days O2
3016_1	**nonBPD**	M	1470	31.2	19.16	43.01	0.00	5.86	0.00	5.26	28.86	77.62	3	0
3018_1	**nonBPD**	M	1440	32.8	16.72	21.30	0.00	12.23	5.42	41.14	5.69	85.70	3	0
3019_1	**nonBPD**	F	1470	30	19.85	35.83	0.00	11.08	9.58	11.85	14.93	83.23	3	0
4002_1	**nonBPD**	M	1080	29	15.73	22.38	0.00	11.51	6.81	11.16	42.59	76.21	4	37
4003_1	**nonBPD**	F	1420	30	19.37	39.12	0.00	15.15	5.03	10.63	14.56	83.36	4	10
4003_2	**nonBPD**	F	1400	30	19.87	28.06	2.34	12.33	4.40	8.58	27.13	91.12	4	10
4006_1	**nonBPD**	M	1330	32	21.82	16.82	3.61	10.77	6.09	28.58	16.81	75.62	4	4
4006_2	**nonBPD**	F	1300	32	16.70	21.65	0.00	16.14	8.46	29.31	11.74	78.83	4	3
4007_1	**nonBPD**	M	1350	31.5	14.36	12.32	0.00	7.48	6.86	39.36	27.12	78.75	4	0
4008_1	**nonBPD**	M	1300	30.3	25.25	15.21	0.00	6.51	2.02	11.58	43.17	73.23	4	0
4009_1	**nonBPD**	M	980	30	9.47	9.26	3.87	5.38	4.06	3.54	71.36	76.59	4	15
4010_1	**nonBPD**	M	1300	30	18.48	33.26	0.00	10.36	4.47	5.83	32.87	68.97	4	35
4010_2	**nonBPD**	F	750	30	13.72	52.79	0.00	19.02	0.54	7.14	10.15	88.08	4	20
4011_1	**nonBPD**	M	970	28	13.61	12.10	0.94	4.86	4.12	0.32	71.24	65.83	4	15
4012_1	**nonBPD**	F	1040	27.1	13.85	10.07	0.00	7.58	12.14	46.35	15.44	72.95	4	22
4013_1	**nonBPD**	F	1150	32	5.61	4.81	0.00	1.99	2.21	1.15	90.76	64.98	4	0
4014_1	**nonBPD**	F	1250	29	32.16	30.83	0.46	14.85	3.16	10.06	17.88	86.24	4	20
4015_1	**nonBPD**	F	780	28.2	12.48	11.09	0.00	7.94	5.59	18.00	53.96	71.93	4	22

*a tobacco smoke exposure CpG biomarker on AHRR (aryl-hydrocarbon receptor repressor)

CD8T= CD8+T cell; CD4T= CD4+ T cell; NK= natural killer cell, Mono= monocyte; Gran= granulocyte; NRBC=nucleated red blood cell.

Table S3. Differentially methylated CpG sites associated with cord blood nucleated red blood cell (NRBC) counts.

Illumina HumanMethylation 450 BeadChip analysis on cord blood DNA from nonBPD infants (n=93).

Bonferroni (BF) correction p< 1.12E-07. t=moderated t-statistic value

Chr=chromosome. Pos.hg19=position on Genome Reference Consortium Human Build 37 (GRCh37, hg19). dist2TSS=distance to transcription start site.

Probe	p_adj	q_val	t	Chr	Pos.hg19	locations	dist2TSS	gene.Illumina	gene.nearest
cg11923627	2.79E-31	1.25E-25	-18.3634	18	55470401	5'UTR	146	*ATP8B1*	*ATP8B1*
cg11067736	6.88E-24	1.54E-18	-14.0727	8	1.01E+08	Body;Body	54690	*SPAG1*	*SPAG1*
cg04512050	1.11E-23	1.66E-18	-13.9599	6	31620972	TSS1500;TSS1500;TSS1500;TSS1500;TSS1500;TSS1500;Body;Body;TSS5000	-495	*BAG6;APOM*	*BAG6*
cg06679265	2.11E-19	2.24E-14	-11.7124	12	57040204	TSS1500;TSS5000;TSS1500	-436	*ATP5F1B;SNORD59B;SNORD59A*	*ATP5F1B*
cg13335447	2.50E-19	2.24E-14	-11.674	12	57853735	TES5000;5'UTR;TSS200;TSS200	168	*INHBE;GLI1*	*GLI1*
cg19317265	5.43E-19	3.82E-14	-11.5026	18	12702832	Body;TSS200;TSS200;TSS200;TSS1500	-56	*PSMG2;CEP76*	*CEP76*
cg13687825	5.98E-19	3.82E-14	-11.4809	8	1.01E+08	Body;Body	54782	*SPAG1*	*SPAG1*
cg15021640	4.11E-18	2.05E-13	11.05682	22	49697802		239312		*MIR3667*
cg13380511	4.99E-18	2.05E-13	-11.0145	11	65837996	TES5000;1stExon	254	*SF3B2;PACS1*	*PACS1*
cg10763598	5.35E-18	2.05E-13	-10.9992	19	19431466	TSS200;TSS200	-148	*SUGP1;MAU2*	*SUGP1*
cg08271318	5.46E-18	2.05E-13	-10.9947	19	44809193	TSS200	-15	*ZNF235*	*ZNF235*
cg07295974	5.49E-18	2.05E-13	-10.9936	16	85045150	TSS200;TSS200	-9	*ZDHHC7*	*ZDHHC7*
cg08917174	1.48E-17	5.08E-13	-10.7771	2	2.17E+08	5'UTR	556	*MARCHF4*	*MARCHF4*
cg10970776	2.16E-17	6.90E-13	-10.6943	11	61100608	TSS200;TSS200;TSS200;TSS200;TSS200;TSS200;TSS200	-31	*DDB1;TKFC*	*DDB1*
cg15653194	4.23E-17	1.26E-12	-10.5479	7	2559473	Body;Body;5'UTR;5'UTR	66	*LFNG*	*LFNG*
cg22604213	5.58E-17	1.56E-12	-10.4878	4	1.05E+08	1stExon;Body;Body	679	*CXXC4;CXXC4-AS1*	*CXXC4-AS1*
cg22939524	1.38E-16	3.64E-12	10.29086	7	1.39E+08	Body;Body	-69812	*KIAA1549*	*ATP6V0A4*
cg12837896	2.77E-16	6.59E-12	-10.1409	17	21415289		39652		*LINC02693*
cg26400491	2.80E-16	6.59E-12	10.13826	8	1.45E+08	Body;Body;TES5000	3297	*SCRIB;MIR937*	*MIR937*
cg10203375	3.37E-16	7.54E-12	-10.0981	1	2.11E+08	Body;Body;TSS1500	312	*KCNH1;KCNH1-IT1*	*KCNH1*
cg26808979	5.70E-16	1.21E-11	-9.98467	11	16759946	Body;TSS1500	78	*SOX6;C11orf58*	*SOX6*
cg27500918	1.03E-15	2.10E-11	-9.85632	16	2933308	Body;5'UTR;5'UTR	93	*FLYWCH2*	*FLYWCH2*
cg13600149	1.94E-15	3.77E-11	10.49424	9	98079129	Body;Body;Body	407	*FANCC*	*FANCC*
cg01926858	2.25E-15	4.19E-11	-9.68893	14	53619558	1stExon;1stExon;1stExon;TSS1500	483	*DDHD1;LOC101927620*	*DDHD1*
cg27013478	5.02E-15	8.78E-11	-9.51591	16	2961939	TSS200;TSS200;TSS200	-40	*FLYWCH1*	*FLYWCH1*
cg07091346	5.11E-15	8.78E-11	9.512194	11	888685	Body;Body;Body;Body;Body	22125	*CHID1*	*CHID1*
cg11869233	5.39E-15	8.92E-11	-9.50073	2	48009809	TSS1500;TSS1500;TSS1500;TSS1500	-411	*MSH6*	*MSH6*
cg00490450	6.43E-15	9.74E-11	-9.46257	3	1.39E+08	TSS200;TSS200;Body;Body	37	*COPB2;LOC100507291*	*LOC100507291*
cg11791427	6.47E-15	9.74E-11	-9.46139	7	73704194	Body;Body	458	*CLIP2*	*CLIP2*
cg06421238	6.54E-15	9.74E-11	9.459116	17	5993785	Body	19852	*WSCD1*	*WSCD1*
cg26712763	7.51E-15	1.08E-10	-9.42928	17	21030248	5'UTR	8	*DHRS7B*	*DHRS7B*
cg06782035	7.70E-15	1.08E-10	-9.42384	5	16179135	Body;TSS1500	765	*MARCHF11;LOC401176*	*MARCHF11*
cg13682223	8.18E-15	1.11E-10	-9.41087	3	50336418	TSS200;Body;Body;Body;Body;TSS200;Body;Body;TES5000;TES5000;TES5000;TES5000;TES5000;TES5000	-127	*HYAL3;NAA80;HYAL1*	*NAA80*
cg04724176	9.24E-15	1.21E-10	9.384757	2	1.29E+08		110809		*HS6ST1*
cg23118188	9.55E-15	1.22E-10	9.377578	11	1.34E+08		-26840		*IGSF9B*
cg16588967	1.25E-14	1.54E-10	9.319656	6	43128935	3'UTR;3'UTR;3'UTR;3'UTR;Body;Body;3'UTR	-10071	*PTK7*	*SRF*
cg19529957	1.27E-14	1.54E-10	9.315533	7	4198590	Body;Body	-24121	*SDK1*	*LOC105375131*
cg13348574	1.33E-14	1.56E-10	9.306762	17	207478	TSS5000;TSS5000;TSS5000;TSS5000	-4845	*RPH3AL*	*RPH3AL*
cg13175480	1.56E-14	1.79E-10	-9.2724	15	83680325	1stExon;1stExon;1stExon;1stExon;Body;1stExon;Body;TES5000;TES5000	66	*C15orf40;BTBD1*	*C15orf40*
cg09112623	1.74E-14	1.94E-10	-9.24891	6	33756905	TSS5000;5'UTR;5'UTR;5'UTR	2	*LEMD2*	*LEMD2*
cg12451869	3.08E-14	3.36E-10	-9.12624	2	1.03E+08		-60276		*IL1RL1*
cg05370752	5.38E-14	5.73E-10	9.005991	21	40756954	Body;Body;Body;Body;Body;Body;Body;Body;Body;Body;TSS5000;TSS5000	-2734	*GET1;WRB-SH3BGR*	*GET1*
cg07023902	5.75E-14	5.98E-10	-8.99193	2	74682183	Body;TSS200;TSS5000	-30	*INO80B-WBP1;INO80B;WBP1*	*INO80B*
cg09201264	6.27E-14	6.28E-10	8.973439	20	3748382	Body;5'UTR;5'UTR;5'UTR	18	*C20orf27*	*C20orf27*
cg02103891	6.40E-14	6.28E-10	8.968996	5	1.72E+08	Body;Body	56808	*SH3PXD2B*	*LOC100288254*
cg22160662	6.46E-14	6.28E-10	8.966943	3	66908896		-120136		*LOC105377143*
cg03634947	6.68E-14	6.36E-10	8.959698	20	33547679	TSS5000;TSS5000;TSS5000;Body	-3849	*GSS;MYH7B*	*GSS*
cg12057156	7.23E-14	6.73E-10	8.942787	7	162052		12335		*LOC100507642*
cg17798633	7.63E-14	6.96E-10	8.931109	17	19363769		49225		*RNF112*
cg05295584	8.61E-14	7.70E-10	8.905187	1	1.09E+08	TSS200;Body;TSS200;TSS200	-18	*AKNAD1;SPATA42*	*AKNAD1*
cg16565692	9.50E-14	8.33E-10	8.884121	5	1.31E+08	TSS1500	-1360	*CSF2*	*CSF2*
cg04798574	9.99E-14	8.59E-10	-8.87321	3	1.49E+08	TSS200;TSS200	-129	*HPS3*	*HPS3*
cg01257309	1.32E-13	1.12E-09	8.812985	13	51418614	TSS1500;Body;TSS1500	-539	*DLEU7;DLEU7-AS1*	*DLEU7*
cg00503298	1.51E-13	1.25E-09	8.784307	1	47263713	TSS1500;TSS1500;TSS1500;TSS1500;TSS1500;TSS1500	-1004	*CYP4B1*	*CYP4B1*
cg15854394	1.75E-13	1.42E-09	8.752952	8	22769830	Body;Body	15597	*PEBP4*	*PEBP4*
cg21544437	2.31E-13	1.85E-09	-8.69267	2	1.14E+08	TES5000	4219	*FOXD4L1*	*FOXD4L1*

Probe	p_adj	q_val	t	Chr	Pos.hg19	locations	dist2TSS	gene.Illumina	gene.nearest
cg14192081	3.05E-13	2.36E-09	8.63337	14	62519784	Body;Body;Body;Body;Body;Body;Body;Body	-64290	*SYT16*	*LINC00643*
cg17457701	3.06E-13	2.36E-09	8.632431	15	86313860	Body;TSS200	-51	*KLHL25;MIR1276*	*MIR1276*
cg04066147	3.43E-13	2.60E-09	8.608003	7	77854601	Body;Body	-121957	*MAGI2*	*RPL13AP17*
cg23152572	3.57E-13	2.65E-09	8.599635	10	13328293	Body;Body;Body;Body;Body;Body	13453	*PHYH*	*PHYH*
cg13407144	3.62E-13	2.65E-09	8.596615	10	33468337	3'UTR;3'UTR;3'UTR;Body;3'UTR	-62602	*NRP1*	*IATPR*
cg19170021	4.00E-13	2.88E-09	8.575321	17	79077169	Body;Body;Body;Body	22004	*BAIAP2*	*MIR657*
cg17144164	4.39E-13	3.10E-09	8.55497	17	78896700	Body;Body	48313	*RPTOR*	*LOC400627*
cg25717351	4.44E-13	3.10E-09	8.552526	17	744675	Body;Body	22676	*NXN*	*NXN*
cg09190824	5.30E-13	3.62E-09	8.51439	4	77662419	Body	156878	*SHROOM3*	*SOWAHB*
cg18043280	5.35E-13	3.62E-09	8.512628	17	40996886	TES5000;TES5000;TES5000;TES5000;TES5000;1stExon;1stExon	290	*PSME3;AOC2*	*AOC2*
cg10012920	6.12E-13	4.09E-09	8.483584	11	1.23E+08		-6598		*GRAMD1B*
cg00678709	6.41E-13	4.21E-09	8.473707	3	14584745	TSS5000	-2896	*GRIP2*	*GRIP2*
cg11492063	7.57E-13	4.90E-09	8.437968	17	5990345	Body	16412	*WSCD1*	*WSCD1*
cg05592114	8.89E-13	5.68E-09	8.403236	12	7244104	Body;Body;TES5000;TES5000;TES5000	385	*C1R;C1RL*	*C1R*
cg11978634	9.06E-13	5.70E-09	8.399293	13	43971194	Body;Body;Body;Body;Body;Body;Body;Body;Body;Body;Body;Body	-51793	*ENOX1*	*ENOX1-AS2*
cg17250993	1.00E-12	6.22E-09	8.377643	4	24958390	Body	23436	*CCDC149*	*CCDC149*
cg11942622	1.11E-12	6.81E-09	8.355168	15	79298659	TSS1500;Body;Body	-657	*RASGRF1*	*RASGRF1*
cg12789935	1.19E-12	7.17E-09	8.341264	2	2.42E+08	TSS1500;TSS1500;Body	-573	*MAB21L4*	*MAB21L4*
cg25329415	1.40E-12	8.29E-09	8.305402	6	28076851		-15482		*ZSCAN16*
cg26062048	1.41E-12	8.29E-09	-8.30405	5	1.53E+08	5'UTR;Body;Body;Body;TSS200;TSS200;TSS200	58	*FAM114A2;MFAP3*	*FAM114A2*
cg16432441	1.56E-12	9.07E-09	8.281934	10	72531006	3'UTR;3'UTR;3'UTR;3'UTR;Body;Body;Body;Body	11323	*TBATA*	*TBATA*
cg16944964	1.75E-12	1.01E-08	-8.25691	2	2.28E+08	TSS1500;Body;Body;Body;Body;Body;Body;Body;TSS5000;TSS5000;TSS5000	386	*MFF-DT;MFF*	*MFF*
cg05877280	1.82E-12	1.03E-08	8.248907	15	76475969	Body;TSS5000	-2142	*TMEM266;LOC101929439*	*LOC101929439*
cg15386350	2.02E-12	1.13E-08	8.226686	1	11591766	Body	52555	*DISP3*	*DISP3*
cg00146249	2.10E-12	1.16E-08	8.217571	17	79042364	Body;Body;Body;Body	33414	*BAIAP2*	*BAIAP2*
cg04735960	2.15E-12	1.17E-08	8.21296	13	21006388	Body;Body;TES5000	1597	*CRYL1;MIR4499*	*MIR4499*
cg27078600	2.28E-12	1.23E-08	8.200053	6	1.68E+08	TSS5000;1stExon;Body	270	*FRMD1*	*FRMD1*
cg08447380	2.37E-12	1.26E-08	8.192101	8	8185703	Body;Body;Body	58051	*PRAG1*	*PRAG1*
cg14994521	2.40E-12	1.26E-08	8.189131	4	1.89E+08		97428		*TRIML1*
cg18745239	2.43E-12	1.27E-08	8.186154	10	44064597	TSS1500;Body;Body;Body;Body;Body;Body;Body;Body;Body;Body	-690	*ZNF239*	*ZNF239*
cg04687371	2.53E-12	1.30E-08	8.177971	8	1916285	TES5000;TES5000;TSS5000	-3277	*LOC101928058;KBTBD11-OT1*	*KBTBD11-OT1*
cg22474130	2.58E-12	1.31E-08	8.173503	6	32017048	Body;Body;TSS5000	-3143	*TNXB*	*TNXB*
cg24154024	2.63E-12	1.32E-08	8.169386	1	2.29E+08	TES5000;Body;Body	34409	*OBSCN*	*MIR6742*
cg16431233	2.77E-12	1.36E-08	8.158316	17	61518244	TSS1500;TSS200;Body;Body	-56	*CYB561*	*CYB561*
cg22556768	2.78E-12	1.36E-08	8.157594	5	66586585		-93958		*CD180*
cg06373804	2.80E-12	1.36E-08	8.155687	2	2.43E+08	Body	-24500	*LINC01237*	*LINC01238*
cg26683025	3.11E-12	1.50E-08	8.133056	11	65560420	Body;TSS5000	-2081	*OVOL1;OVOL1-AS1*	*OVOL1-AS1*
cg01287788	3.25E-12	1.54E-08	8.124172	2	86362935	Body;TSS200	-57	*PTCD3;SNORD94*	*SNORD94*
cg01301117	3.34E-12	1.57E-08	8.117686	7	1953969	Body;Body;Body;Body;Body;Body	26238	*MAD1L1*	*MAD1L1*
cg21915765	3.44E-12	1.60E-08	-8.11141	19	18682480	TES5000;TES5000;TES5000;TSS200;TSS200;TSS200;TSS200;TSS200;TSS200;TSS200;TSS1500	-59	*KXD1;UBA52*	*UBA52*
cg15570395	3.88E-12	1.79E-08	8.085826	4	1.85E+08	Body	9978	*ENPP6*	*ENPP6*
cg03033447	4.08E-12	1.86E-08	8.07495	1	2.28E+08	Body;Body;Body	-72339	*OBSCN*	*OBSCN-AS1*
cg19294448	4.47E-12	2.02E-08	8.054992	17	6900799	Body;Body	1432	*ALOX12-AS1;ALOX12*	*ALOX12*
cg12647283	4.66E-12	2.09E-08	8.045908	10	1.27E+08	Body;Body;Body;Body;Body;Body;Body	55950	*CTBP2*	*CTBP2*
cg02485348	5.08E-12	2.25E-08	8.02743	1	2.45E+08		73787		*DESI2*
cg02725290	5.60E-12	2.43E-08	-8.01964	6	10837710	Body;Body;Body	1062	*MAK*	*MAK*
cg05235171	5.61E-12	2.43E-08	8.006088	11	1.18E+08	TSS1500;Body;Body;Body;Body;Body;Body;Body	-426	*SMIM35;TMPRSS4*	*SMIM35*
cg06799163	5.76E-12	2.48E-08	8.000067	5	1.38E+08	TSS5000;TSS5000;TSS5000;Body;TSS5000;TSS5000;Body;Body;Body;TSS5000;TSS5000;TSS5000;TSS5000	2570	*CDC25C;LOC100128966;FAM53C*	*LOC100128966*
cg03283048	6.07E-12	2.59E-08	-7.98876	16	2933047	TSS200;TSS200;TSS200	-148	*FLYWCH2*	*FLYWCH2*
cg17257469	6.56E-12	2.77E-08	7.972138	7	1.38E+08	Body;Body;Body	13900	*AKR1D1*	*AKR1D1*
cg16465129	6.75E-12	2.82E-08	7.965829	5	1.31E+08	TSS1500	-1332	*CSF2*	*CSF2*
cg01782709	6.99E-12	2.89E-08	7.958516	6	32918306	Body	2568	*HLA-DMA*	*HLA-DMA*
cg19826045	7.04E-12	2.89E-08	7.95694	17	61561602	Body;TSS1500;TSS1500	-604	*ACE*	*ACE*
cg16097532	7.91E-12	3.22E-08	7.931596	14	1.05E+08	Body;Body	20678	*AHNAK2*	*AHNAK2*
cg24768137	8.02E-12	3.22E-08	7.928684	2	2.43E+08		24028		*NEU4*
cg26014570	8.05E-12	3.22E-08	7.927744	12	1.07E+08	TSS5000	-2340	*NUAK1*	*NUAK1*
cg04605084	8.54E-12	3.35E-08	-7.91499	1	91487590	TSS1500;5'UTR;5'UTR	2	*ZNF644*	*ZNF644*
cg26360004	8.59E-12	3.35E-08	7.913721	1	9376755	Body	23803	*SPSB1*	*SPSB1*
cg14645926	8.62E-12	3.35E-08	7.912886	8	1.04E+08	TSS5000;TSS5000;TSS5000;TSS5000;Body;Body;Body;Body;Body	4473	*LOC105369147;FZD6*	*FZD6*
cg22084460	8.84E-12	3.37E-08	7.907556	19	13348050	Body;Body;Body;Body;Body	86826	*CACNA1A*	*IER2*

Probe	p_adj	q_val	t	Chr	Pos.hg19	locations	dist2TSS	gene.Illumina	gene.nearest
cg06912227	8.85E-12	3.37E-08	7.907238	16	4053409	Body	-52964	*ADCY9*	*LINC02861*
cg06746774	8.89E-12	3.37E-08	7.906325	1	33216950	Body;TSS5000	-2649	*KIAA1522*	*KIAA1522*
cg03469578	9.24E-12	3.47E-08	7.897874	4	3735986		-32125		*ADRA2C*
cg09284102	9.64E-12	3.59E-08	7.88875	16	66116829		-283764		*CDH5*
cg08031769	9.79E-12	3.62E-08	7.885444	3	41042969		-198026		*CTNNB1*
cg03186514	9.91E-12	3.63E-08	7.882749	2	1.1E+08	Body	112398	*SH3RF3*	*SEPTIN10*
cg16806210	1.01E-11	3.66E-08	7.878473	11	1.18E+08	TES5000;TES5000;Body;Body;Body;Body;Body;5'UTR;5'UTR;1stExon;TSS5000;TSS5000;TSS5000;TSS5000;TSS5000;TSS5000;TSS5000;TSS5000;TSS5000;TSS5000	130	*KMT2A;LOC101929089;TTC36;TMEM25*	*TTC36*
cg20832559	1.01E-11	3.66E-08	7.877648	3	97594786	Body	53932	*CRYBG3*	*CRYBG3*
cg04340895	1.03E-11	3.67E-08	7.875209	4	47463168	Body;Body	2551	*COMMD8*	*COMMD8*
cg03383006	1.08E-11	3.85E-08	7.863342	17	42992924	TSS200;TSS200;TSS200;TSS200	-4	*GFAP*	*GFAP*
cg21620356	1.16E-11	4.07E-08	7.849404	2	1.98E+08	Body;Body;Body;Body;Body;Body	-36761	*ANKRD44*	*ANKRD44-AS1*
cg21746991	1.17E-11	4.07E-08	7.846545	8	1.44E+08	Body	65938	*ADGRB1*	*ADGRB1*
cg07518837	1.18E-11	4.07E-08	-7.84521	8	1.01E+08	Body;Body	54516	*SPAG1*	*SPAG1*
cg07814886	1.18E-11	4.07E-08	7.844255	7	36086032		-48887		*LOC101928618*
cg22286732	1.24E-11	4.23E-08	7.834443	13	1.14E+08	TES5000;TES5000	78659	*ATP11A*	*MCF2L-AS1*
cg11228504	1.31E-11	4.41E-08	7.822882	12	1.12E+08	Body;Body;Body;Body;Body	35264	*ATXN2*	*ATXN2*
cg07847273	1.31E-11	4.41E-08	7.821964	3	43195345		-47774		*POMGNT2*
cg21217270	1.34E-11	4.47E-08	7.817523	1	25238271	Body;Body;Body	7636	*RUNX3*	*MIR6731*
cg11084729	1.36E-11	4.51E-08	7.813887	15	1.02E+08		113192		*CHSY1*
cg25497487	1.39E-11	4.57E-08	7.80976	22	25615355	TSS1500	-250	*CRYBB2*	*CRYBB2*
cg02626129	1.48E-11	4.85E-08	7.795241	13	31480646	Body;5'UTR	239	*TEX26-AS1;MEDAG*	*MEDAG*
cg02732134	1.61E-11	5.17E-08	7.777453	10	1.02E+08	Body	37322	*DNMBP*	*DNMBP*
cg11746924	1.61E-11	5.17E-08	7.777083	17	62007912	Body;Body;Body;Body	1779	*CD79B*	*CD79B*
cg25355645	1.62E-11	5.17E-08	7.776297	19	42873746	Body;Body	18464	*MEGF8*	*MIR8077*
cg25770267	1.68E-11	5.31E-08	7.768029	1	7596685	Body;Body;Body;Body;Body	-133104	*CAMTA1*	*CAMTA1*
cg14596967	1.69E-11	5.31E-08	7.767508	11	1.14E+08	Body;Body;Body;Body;Body	23012	*ZBTB16*	*ZBTB16*
cg09336628	1.78E-11	5.58E-08	7.755283	7	1.43E+08	Body;Body	8354	*CLCN1*	*CLCN1*
cg26510500	1.88E-11	5.84E-08	7.744098	2	74778482	TSS5000;Body;Body;Body;TSS5000;TSS5000;TSS5000;TSS5000;TSS5000	-1829	*LOXL3;DOK1*	*LOXL3*
cg06328100	2.08E-11	6.42E-08	7.721862	6	33638806	Body	-27098	*ITPR3*	*MIR3934*
cg16272981	2.15E-11	6.54E-08	7.714786	5	1489889	Body	21082	*LPCAT1*	*MIR6075*
cg01854776	2.16E-11	6.54E-08	7.714305	2	2.36E+08	Body;Body;Body;Body;Body	14349	*SH3BP4*	*SH3BP4*
cg09176893	2.16E-11	6.54E-08	7.713443	4	1.01E+08	Body	1240	*C4orf54*	*C4orf54*
cg21881330	2.19E-11	6.58E-08	7.71078	14	92938068	Body;Body;Body	-42056	*SLC24A4*	*RIN3*
cg04868072	2.31E-11	6.88E-08	7.699471	16	85968253		16301		*MIR6774*
cg05998826	2.37E-11	7.01E-08	7.694102	3	31494410		-80006		*STT3B*
cg08714895	2.45E-11	7.20E-08	7.686735	1	17996919	Body;Body;Body;Body;Body;Body;Body	52112	*ARHGEF10L*	*ARHGEF10L*
cg10096321	2.48E-11	7.24E-08	7.684101	11	1.14E+08	1stExon;1stExon;1stExon;1stExon;1stExon	362	*ZBTB16*	*ZBTB16*
cg14131889	2.52E-11	7.32E-08	7.680198	17	39913822	Body;Body;Body;Body;Body;Body;Body	-22924	*JUP*	*HAP1*
cg19305424	2.57E-11	7.41E-08	7.675691	13	1.11E+08	TSS5000	-3542	*RAB20*	*RAB20*
cg27388680	2.58E-11	7.41E-08	7.674901	19	6857265	3'UTR;3'UTR;3'UTR	-30324	*VAV1*	*ADGRE1*
cg02735962	2.61E-11	7.42E-08	7.672363	10	14740875	Body;Body	76021	*FAM107B*	*FAM107B*
cg04267851	2.62E-11	7.42E-08	7.671639	20	377134	Body;Body;Body;Body;Body;Body	-11559	*TRIB3*	*RBCK1*
cg23024326	2.65E-11	7.46E-08	7.669092	6	36761843	Body;TES5000	-36657	*CPNE5*	*CPNE5*
cg14862445	2.79E-11	7.80E-08	-7.65822	6	31633163	TSS5000;TSS1500;Body;Body;Body;Body;TSS1500;TSS1500	244	*C6orf47;GPANK1;CSNK2B*	*GPANK1*
cg00176101	2.86E-11	7.94E-08	-7.65303	13	1.15E+08	Body;Body;Body;Body;Body;Body;Body;Body;Body;Body;Body;Body;Body;Body;Body;Body;Body;Body;Body;Body;Body;Body;Body;Body;Body;TSS1500	-217	*UPF3A*	*UPF3A*
cg12462858	2.97E-11	8.20E-08	7.644552	1	16810776	Body	8420	*CROCCP3*	*CROCCP3*
cg06172235	3.01E-11	8.25E-08	7.641991	6	1.68E+08	TSS5000;Body	138	*FRMD1*	*FRMD1*
cg20379919	3.05E-11	8.32E-08	7.638821	3	1.84E+08	5'UTR;5'UTR;5'UTR;5'UTR;5'UTR;1stExon;5'UTR;5'UTR;5'UTR;Body;TSS5000;TSS5000;TSS5000;TSS5000;TSS5000	55	*THPO;CHRD*	*THPO*
cg05134426	3.08E-11	8.35E-08	7.636586	5	1.24E+08	1stExon	326	*ZNF608*	*ZNF608*
cg25827666	3.19E-11	8.54E-08	7.629395	1	1.57E+08	Body;Body;Body;Body;Body;5'UTR	17	*SH2D2A;NTRK1*	*NTRK1*
cg07899272	3.19E-11	8.54E-08	7.629264	2	75049872		-11381		*HK2*
cg05573829	3.23E-11	8.59E-08	7.626579	5	1.32E+08		-9095		*SHROOM1*
cg14363146	3.33E-11	8.80E-08	-7.61998	9	6704178		-16684		*KDM4C*
cg24013105	3.44E-11	9.05E-08	7.61275	8	1.38E+08		412453		*LINC02055*
cg08649013	3.48E-11	9.11E-08	7.609992	4	10042198	TSS1500	-326	*SLC2A9*	*SLC2A9*
cg08380311	3.66E-11	9.52E-08	-7.60664	19	3435252	Body;Body;Body;Body;Body	45271	*NFIC*	*SMIM24*
cg00825597	3.70E-11	9.58E-08	7.59656	10	89116372	Body;TSS1500	-1104	*LINC00863;NUTM2D*	*NUTM2D*
cg15488660	3.75E-11	9.64E-08	7.593876	2	2.43E+08	TES5000;TES5000	-20623	*THAP4*	*BOK-AS1*
cg12010750	3.81E-11	9.70E-08	7.590502	11	17803160	TES5000;Body	45666	*KCNC1*	*KCNC1*

Probe	p_adj	q_val	t	Chr	Pos.hg19	locations	dist2TSS	gene.Illumina	gene.nearest
cg06205922	3.84E-11	9.70E-08	7.588962	1	1473141	TES5000;TES5000;TES5000;Body;TES5000	2863	*ATAD3A;TMEM240;SSU72*	*TMEM240*
cg00231754	3.84E-11	9.70E-08	7.58885	2	38176859	Body;Body;Body;Body;TES5000;TSS1500;TSS1500	-727	*RMDN2;RMDN2-AS1*	*RMDN2*
cg26463800	3.92E-11	9.85E-08	7.584224	17	72300792	Body;Body;Body;Body	-21572	*DNAI2*	*KIF19*
cg02472906	3.99E-11	9.98E-08	7.58016	16	87938341	Body;Body;Body;Body	31794	*CA5A*	*CA5A*
cg01009920	4.21E-11	1.05E-07	7.568725	8	8153650		67559		*FAM86B3P*
cg16452248	4.24E-11	1.05E-07	7.566963	16	613526	1stExon;TSS5000;TSS5000	3133	*PRR35;NHLRC4*	*PRR35*
cg09695261	4.28E-11	1.05E-07	7.565215	17	79304600		-21552		*LINC00482*
cg08858220	4.30E-11	1.05E-07	7.563867	6	36805317	Body;Body	1903	*CPNE5*	*CPNE5*
cg02250259	4.34E-11	1.06E-07	7.56196	7	27273366		-9054		*EVX1*
cg20943723	4.43E-11	1.07E-07	7.557743	3	1.55E+08	Body;Body	-9327	*PLCH1*	*PLCH1*
cg23580024	4.48E-11	1.08E-07	7.555307	10	93811989	Body;Body	128463	*CPEB3*	*BTAF1*
cg08137852	4.53E-11	1.08E-07	7.552805	11	1856025	Body;Body;Body;Body;TSS5000;TSS5000	369	*SYT8;TNNI2*	*SYT8*
cg24860020	4.60E-11	1.09E-07	7.549257	8	1.23E+08		-135860		*HAS2-AS1*
cg15889768	4.63E-11	1.10E-07	7.547976	17	42619247		-15604		*FZD2*
cg19033943	4.99E-11	1.17E-07	7.531787	7	1140782	Body;Body;Body;Body;Body	13034	*C7orf50*	*GPER1*
cg00437011	5.13E-11	1.20E-07	7.525647	6	1.71E+08	Body	5571	*LOC154449*	*LOC154449*
cg13131263	5.16E-11	1.20E-07	7.524312	1	9324393	Body;Body	24488	*H6PD*	*H6PD*
cg07131274	5.17E-11	1.20E-07	7.523813	2	3270259	Body;Body;Body	111330	*EIPR1*	*EIPR1*
cg02340615	5.48E-11	1.26E-07	7.511377	12	1.05E+08	Body	7502	*TXNRD1*	*TXNRD1*
cg09144398	5.57E-11	1.28E-07	7.507659	11	1.19E+08	TSS200;TSS200	-126	*TREH*	*TREH*
cg08389497	5.87E-11	1.34E-07	7.496231	10	73820491	3'UTR;3'UTR	27818	*SPOCK2*	*SPOCK2*
cg13726814	5.92E-11	1.34E-07	7.49429	2	1.2E+08	Body;TSS5000;TSS5000	-3347	*SCTR;LOC107105282*	*LOC107105282*
cg08492875	5.98E-11	1.35E-07	7.491989	2	1.35E+08	Body;Body	194429	*MGAT5*	*MGAT5*
cg11614093	6.03E-11	1.35E-07	7.490461	6	1.55E+08		-5124		*TIAM2*
cg19951269	6.10E-11	1.36E-07	7.487753	11	94783903		-16137		*SRSF8*
cg16764236	6.41E-11	1.43E-07	7.477052	17	32611290	TSS1500	-1470	*CCL11*	*CCL11*
cg07905199	6.52E-11	1.44E-07	7.473332	17	67507798	Body;Body	9396	*MAP2K6*	*MAP2K6*
cg12044293	6.57E-11	1.45E-07	7.471598	17	78896669	Body;Body	48344	*RPTOR*	*LOC400627*
cg16233472	6.65E-11	1.45E-07	7.468944	11	65562093	Body;TSS5000	-3754	*OVOL1;OVOL1-AS1*	*OVOL1-AS1*
cg06187344	6.66E-11	1.45E-07	7.468699	10	3151576	Body;Body;Body;Body;Body;Body;Body;Body;Body;Body;Body	4776	*PFKP*	*PFKP*
cg16841189	6.70E-11	1.46E-07	7.467168	8	1799411	Body;Body;Body	27320	*ARHGEF10*	*ARHGEF10*
cg27604721	6.95E-11	1.50E-07	7.459221	11	2722258	Body;Body;TSS1500	-1030	*KCNQ1;KCNQ1OT1*	*KCNQ1OT1*
cg13802527	7.01E-11	1.51E-07	7.457356	9	1.15E+08	TSS1500;TSS1500;TSS1500;TSS1500;TSS1500;TSS1500;TSS1500;TSS1500;TSS1500;TSS1500	-642	*INIP*	*INIP*
cg12082793	7.04E-11	1.51E-07	7.456481	1	20218923	Body	10036	*OTUD3*	*OTUD3*
cg14330078	7.11E-11	1.52E-07	7.454213	3	1.88E+08	Body;TSS200	-199	*LPP*	*LPP*
cg12850636	7.15E-11	1.52E-07	7.453205	19	3727602	Body;Body	5868	*TJP3*	*TJP3*
cg02891082	7.29E-11	1.54E-07	7.448939	11	68118671	Body;Body	38613	*LRP5*	*LRP5*
cg27092975	7.46E-11	1.57E-07	7.443812	11	70805455	Body	-86982	*SHANK2*	*MIR3664*
cg16374972	7.49E-11	1.57E-07	7.442996	21	44811965		-29736		*LINC01679*
cg21491340	7.62E-11	1.58E-07	7.439223	19	18092572	Body	-19360	*KCNN1*	*ARRDC2*
cg22332100	7.65E-11	1.58E-07	7.438463	1	2000508	Body;TSS5000;TSS5000;TSS5000;TSS5000;TSS5000;TSS5000;TSS5000	-4392	*PRKCZ*	*PRKCZ*
cg00904483	7.84E-11	1.62E-07	7.433098	10	1.31E+08	Body	89369	*MGMT*	*MGMT*
cg11219178	8.27E-11	1.69E-07	7.421265	11	3120949	Body;Body;Body	-42296	*OSBPL5*	*CARS1*
cg20432475	8.27E-11	1.69E-07	7.42124	1	15763737	TSS1500	-1201	*CTRC*	*CTRC*
cg26932889	8.45E-11	1.72E-07	7.416618	1	54135470	Body;Body	64407	*GLIS1*	*GLIS1*
cg13960678	8.56E-11	1.73E-07	7.413867	3	1.22E+08	Body;Body	-63346	*CASR*	*CSTA*
cg26337259	8.57E-11	1.73E-07	7.413627	17	62770948	Body;TES5000	-5928	*ARHGAP27P1-BPTFP1-KPNA2P3;ARHGAP27P1*	*MIR6080*
cg02605387	8.64E-11	1.73E-07	7.411804	6	32062252	Body;Body	14863	*TNXB*	*TNXB*
cg10510707	8.67E-11	1.73E-07	7.41111	7	563858	TSS5000;TSS5000;Body	3831	*PDGFA;HRAT92*	*HRAT92*
cg01131289	8.77E-11	1.74E-07	7.408507	22	25755786	Body;Body	2738	*LRP5L*	*LRP5L*
cg19827089	8.81E-11	1.74E-07	7.407524	4	1.44E+08	TSS5000;TSS5000	-3276	*GAB1*	*GAB1*
cg07831351	8.87E-11	1.75E-07	-7.40596	20	3229402	TES5000	-9566	*C20orf194*	*SLC4A11*
cg11454547	8.91E-11	1.75E-07	7.405043	2	11293378	TES5000;TES5000;TES5000;TSS5000;TSS5000;TSS5000;TSS5000;TSS5000	-2119	*C2orf50;SLC66A3*	*SLC66A3*
cg07232063	9.01E-11	1.76E-07	7.4025	1	86953050	Body	18441	*CLCA1*	*CLCA1*
cg09774179	9.15E-11	1.77E-07	7.399257	6	36652043	Body;1stExon;1stExon;1stExon;1stExon	5553	*CDKN1A*	*CDKN1A*
cg03585419	9.16E-11	1.77E-07	-7.39901	19	54369681	Body;Body;Body;TSS1500;TSS1500;TSS1500;TSS1500;TSS1500;TSS5000;TSS5000;TSS5000;TSS5000	57	*MYADM*	*MYADM*
cg24429708	9.16E-11	1.77E-07	7.398967	10	640061	Body	-34442	*DIP2C*	*LOC101930421*
cg03205258	9.21E-11	1.77E-07	-7.39782	22	19929274	TSS5000;1stExon;1stExon;Body;1stExon;TSS200;TSS200	-20	*TXNRD2;COMT*	*COMT*
cg20567067	9.54E-11	1.82E-07	7.390071	11	72394353	TES5000;TES5000;TES5000;TES5000;TES5000	-8887	*ARAP1*	*PDE2A*

Probe	p_adj	q_val	t	Chr	Pos.hg19	locations	dist2TSS	gene.Illumina	gene.nearest
cg15895782	1.00E-10	1.91E-07	7.379405	1	2.15E+08	Body	74720	*PTPN14*	*PTPN14*
cg00545229	1.03E-10	1.96E-07	7.37291	4	15429531	Body;Body;Body;TSS200	7	*C1QTNF7-AS1;C1QTNF7*	*C1QTNF7-AS1*
cg02957094	1.05E-10	1.97E-07	7.369963	1	1.78E+08		-12527		*CRYZL2P*
cg09235385	1.05E-10	1.97E-07	7.368944	13	1.15E+08	Body;Body;Body	17979	*RASA3*	*RASA3*
cg07150777	1.07E-10	2.00E-07	7.365587	2	2.4E+08	Body	-5643	*HDAC4*	*MIR4440*
cg23046231	1.08E-10	2.01E-07	7.363698	2	2.01E+08	Body;Body;Body;Body;Body;Body;Body	32209	*SPATS2L*	*KCTD18*
cg10503473	1.08E-10	2.01E-07	7.362523	16	67290849	Body;Body;Body;Body;Body;Body;Body;Body;Body;Body	7968	*SLC9A5*	*SLC9A5*
cg07036035	1.13E-10	2.09E-07	7.353288	14	96133072	Body;Body	14639	*TCL6*	*TCL6*
cg22218588	1.15E-10	2.12E-07	7.349251	6	1.56E+08	Body;Body;TES5000	9994	*TIAM2;TFB1M*	*TIAM2*
cg02712545	1.16E-10	2.12E-07	7.347637	15	99398084	Body;Body	70430	*IGF1R*	*MIR4714*
cg14527639	1.18E-10	2.15E-07	7.3439	14	79359526	Body;Body;Body;Body;Body;Body;Body;Body	-306798	*NRXN3*	*LOC105370586*
cg01428849	1.19E-10	2.15E-07	7.342554	10	1.05E+08	Body;Body	26898	*SH3PXD2A*	*SH3PXD2A*
cg20823730	1.19E-10	2.15E-07	7.342016	8	790768	Body;Body	42458	*DLGAP2*	*LOC105377777*
cg14855150	1.19E-10	2.15E-07	7.341043	1	64608517	3'UTR;Body	-29043	*ROR1*	*ROR1-AS1*
cg08118412	1.20E-10	2.16E-07	7.339213	3	64113418	Body;Body	24273	*PRICKLE2*	*PRICKLE2-AS2*
cg06301297	1.23E-10	2.20E-07	7.334876	11	68043180	TSS5000;TSS5000	-3739	*C11orf24*	*C11orf24*
cg12137439	1.26E-10	2.25E-07	7.328856	21	44983258	Body;TSS5000	-1804	*HSF2BP;H2BS1*	*H2BS1*
cg06749592	1.29E-10	2.30E-07	-7.32324	2	2.33E+08	Body;TSS200;TSS200	-90	*EFHD1*	*EFHD1*
cg27054514	1.30E-10	2.31E-07	7.321551	1	2252995	Body	31105	*MORN1*	*LOC100129534*
cg20255933	1.31E-10	2.31E-07	7.320402	12	20094250		-73368		*LINC02398*
cg08050663	1.33E-10	2.33E-07	7.317777	14	77725701	3'UTR	-6859	*TMEM63C*	*MIR1260A*
cg10720966	1.35E-10	2.36E-07	7.314064	7	1.02E+08	Body;Body;Body;Body;Body;Body;Body	-64569	*CUX1*	*SH2B2*
cg25736198	1.39E-10	2.42E-07	7.30776	12	72345225	Body	12601	*TPH2*	*TPH2*
cg05413061	1.40E-10	2.43E-07	7.305682	3	16225307	Body;Body	9121	*GALNT15*	*GALNT15*
cg22572125	1.42E-10	2.45E-07	7.303433	7	56296973		-112863		*NUPR2*
cg04016527	1.45E-10	2.49E-07	7.298441	17	36707775	Body	54430	*SRCIN1*	*SRCIN1*
cg14180747	1.50E-10	2.56E-07	7.291571	9	37647717	Body;Body;TSS5000	-3233	*FRMPD1*	*FRMPD1*
cg04704247	1.50E-10	2.56E-07	7.291353	4	1672920	Body;Body;Body	12798	*FAM53A*	*FAM53A*
cg16022049	1.52E-10	2.58E-07	7.288213	12	13359372	Body	9713	*EMP1*	*EMP1*
cg13170179	1.52E-10	2.58E-07	7.287638	14	69790294	Body;Body	63420	*GALNT16*	*GALNT16*
cg09011038	1.53E-10	2.58E-07	7.286818	16	4385355	Body;Body;TES5000;TES5000	3131	*GLIS2;PAM16;CORO7-PAM16*	*GLIS2*
cg11334499	1.55E-10	2.60E-07	7.284268	10	1.33E+08	Body	106227	*TCERG1L*	*TCERG1L-AS1*
cg20129782	1.57E-10	2.62E-07	7.281651	20	30135362	Body;Body;Body	25704	*HM13*	*HM13-AS1*
cg00819818	1.58E-10	2.63E-07	7.279743	17	38512762	3'UTR;3'UTR;3'UTR;3'UTR;TES5000	8227	*RARA;GJD3*	*GJD3*
cg21903221	1.60E-10	2.66E-07	7.277244	14	1.06E+08	Body;Body;Body	-12893	*PACS2*	*TEX22*
cg08808258	1.61E-10	2.67E-07	7.275265	22	24671137	Body;Body;Body;Body	4339	*SPECC1L;SPECC1L-ADORA2A*	*SPECC1L*
cg19301303	1.62E-10	2.67E-07	7.274613	4	1.2E+08	Body;Body	37487	*SEC24D*	*SEC24D*
cg04168854	1.64E-10	2.69E-07	7.272022	10	1.05E+08	TSS200	-105	*RPEL1*	*RPEL1*
cg23694114	1.64E-10	2.69E-07	-7.27086	17	15848221	TSS200	-9	*ADORA2B*	*ADORA2B*
cg18033335	1.65E-10	2.69E-07	7.270047	19	11034897	TES5000;TES5000;TES5000;Body;Body;Body;TSS5000	4418	*CARM1;YIPF2;TIMM29*	*YIPF2*
cg24362829	1.66E-10	2.69E-07	7.2693	1	1.78E+08	TES5000;TES5000	-26636	*RASAL2*	*TEX35*
cg24738325	1.66E-10	2.70E-07	7.268253	1	9322665	Body;Body	22760	*H6PD*	*H6PD*
cg14244251	1.68E-10	2.71E-07	7.266589	9	1.32E+08	TSS5000;TSS5000;TSS5000;TSS5000;TSS5000;TSS5000;TSS5000;TSS5000;TSS5000;TSS5000;TSS5000;TSS5000;TSS5000;TSS5000	-3348	*SH3GLB2;MIGA2*	*MIGA2*
cg08036346	1.71E-10	2.74E-07	7.262537	15	68098991	Body;Body;Body	-18855	*MAP2K5*	*SKOR1*
cg12654045	1.71E-10	2.74E-07	7.262022	1	17559606	Body	-15986	*PADI1*	*PADI3*
cg05046097	1.73E-10	2.77E-07	7.259399	10	88717926	TSS1500;Body;TSS1500	-429	*MMRN2;SNCG*	*SNCG*
cg12290217	1.76E-10	2.81E-07	7.255605	15	99563030	Body	5098	*LUNAR1*	*LUNAR1*
cg08621168	1.80E-10	2.84E-07	7.251242	8	56919462	Body;Body	66998	*LYN*	*SNORD54*
cg25788513	1.80E-10	2.84E-07	7.250386	10	93314761	Body	56456	*HECTD2-AS1*	*HECTD2-AS1*
cg13509765	1.81E-10	2.84E-07	7.250266	9	1.34E+08	Body;Body	23968	*ABL1*	*ABL1*
cg16791528	1.83E-10	2.86E-07	7.247919	2	79347532	TSS200	-81	*REG1A*	*REG1A*
cg10960375	1.84E-10	2.87E-07	7.246626	3	42694144	TES5000;TES5000;TES5000;TES5000;Body;TSS1500	-1044	*NKTR;LOC101928323;ZBTB47*	*ZBTB47*
cg17046586	1.87E-10	2.92E-07	7.242185	12	50665774	Body;Body	11529	*LIMA1*	*LIMA1*
cg01168833	1.90E-10	2.95E-07	7.239424	12	1.06E+08		-141539		*CASC18*
cg26456942	1.91E-10	2.96E-07	7.237465	2	2.42E+08	TSS5000;TSS5000;TSS5000;TSS5000;TSS5000;TSS5000;TSS5000;TSS5000;TSS5000	-2569	*STK25*	*STK25*
cg25314706	1.93E-10	2.98E-07	7.235511	2	1.28E+08	Body	-25552	*MYO7B*	*LOC105373609*
cg00215182	1.94E-10	2.98E-07	7.234185	1	22979495	TES5000;TES5000;TES5000;TES5000;TSS1500;TSS1500	-233	*C1QC;C1QB*	*C1QB*
cg09797390	1.94E-10	2.98E-07	7.234007	1	1.12E+08	TES5000;TES5000;TES5000;TES5000;TES5000;TES5000;TES5000	18922	*RAP1A;INKA2*	*INKA2*
cg07442105	1.97E-10	3.00E-07	7.231432	16	66962501	TSS5000;TSS5000;TES5000;TES5000;TES5000	-3062	*RRAD;CIAO2B*	*RRAD*

Probe	p_adj	q_val	t	Chr	Pos.hg19	locations	dist2TSS	gene.Illumina	gene.nearest
cg16124977	2.02E-10	3.05E-07	7.226036	17	80371295	Body;Body;Body;TSS5000;TSS5000;TSS5000;TSS5000	-4927	*OGFOD3;HEXD*	*HEXD*
cg26619035	2.02E-10	3.05E-07	7.225711	7	87936923	TSS1500;TSS1500;TSS1500	-714	*STEAP4*	*STEAP4*
cg12464898	2.02E-10	3.05E-07	7.225345	8	73451462	Body	2003	*KCNB2*	*KCNB2*
cg17580363	2.03E-10	3.05E-07	7.224872	11	6478177	Body;Body;Body;Body	-15960	*TRIM3*	*HPX*
cg15675123	2.04E-10	3.07E-07	7.223182	5	12623177	Body	48209	*LINC01194*	*LINC01194*
cg17468459	2.09E-10	3.12E-07	7.218421	1	92208474	Body;Body;Body;Body	143234	*TGFBR3*	*TGFBR3*
cg00601162	2.09E-10	3.12E-07	7.217863	22	29655504	3'UTR;3'UTR;TES5000;TES5000;TES5000	8466	*EMID1;RHBDD3*	*RHBDD3*
cg24303306	2.10E-10	3.12E-07	7.216804	3	1.26E+08	3'UTR;Body	15938	*CHST13;C3orf22*	*C3orf22*
cg02087342	2.11E-10	3.13E-07	7.216051	17	7579047	TSS1500;TSS1500;TSS1500;TSS1500;TSS1500;TSS1500;Body;Body;Body;Body;Body;Body;Body;Body;Body	-236	*TP53*	*TP53*
cg11360098	2.15E-10	3.17E-07	7.212312	2	95613040	Body	47	*LOC442028*	*LOC442028*
cg26849077	2.16E-10	3.18E-07	7.210469	15	65914281	Body;5'UTR;TSS5000;TSS5000	6	*SLC24A1*	*SLC24A1*
cg04246521	2.18E-10	3.19E-07	7.20909	13	96086275	Body;1stExon	423	*CLDN10*	*CLDN10*
cg18421360	2.26E-10	3.30E-07	7.200877	5	90415181	Body;Body	195038	*ADGRV1*	*LUCAT1*
cg07072281	2.29E-10	3.33E-07	7.198321	11	2713432	Body;Body;Body	7796	*KCNQ1;KCNQ1OT1*	*KCNQ1OT1*
cg03513571	2.32E-10	3.37E-07	7.194906	2	3691618	Body;Body;Body;Body;Body;Body;Body;Body;Body;Body;Body	-14171	*COLEC11*	*ALLC*
cg04414912	2.33E-10	3.38E-07	7.193899	11	1.31E+08	TSS1500;TSS1500;TSS1500;TSS1500;TSS1500;TSS1500;TSS1500	-1479	*NTM*	*NTM*
cg14037769	2.35E-10	3.39E-07	7.192652	1	9895799		-11215		*CLSTN1*
cg04847174	2.36E-10	3.39E-07	7.191651	5	1.34E+08	Body	-9727	*C5orf66*	*C5orf66-AS1*
cg25305174	2.38E-10	3.41E-07	7.189436	7	44279680	Body;Body;Body;Body;Body;Body;Body;Body;Body	39104	*CAMK2B*	*YKT6*
cg25333056	2.42E-10	3.45E-07	7.185722	10	1.35E+08	Body;Body;Body;Body	-15258	*TUBGCP2*	*ZNF511*
cg03194383	2.42E-10	3.45E-07	7.185624	3	14600098		-18249		*GRIP2*
cg07967632	2.44E-10	3.46E-07	7.184443	15	1.02E+08	Body	141570	*LRRK1*	*LRRK1*
cg24631518	2.47E-10	3.49E-07	7.181539	10	73104270	Body;Body;Body;Body;Body	25255	*SLC29A3*	*SLC29A3*
cg13609939	2.47E-10	3.49E-07	7.181015	10	1.26E+08	Body;Body;Body;Body;Body;Body;Body;Body;Body;Body	2332	*OAT*	*OAT*
cg20435464	2.50E-10	3.51E-07	7.1791	19	40711928	Body	12378	*MAP3K10*	*TTC9B*
cg22396632	2.51E-10	3.52E-07	7.178046	1	3079212	Body;Body	34674	*PRDM16*	*MIR4251*
cg08770935	2.54E-10	3.55E-07	7.175195	17	17255562	TES5000	48876	*NT5M*	*NT5M*
cg14599857	2.55E-10	3.56E-07	7.1741	1	2010959	Body;Body;Body;Body;Body;Body;Body;Body	5535	*PRKCZ*	*PRKCZ*
cg16260424	2.63E-10	3.66E-07	7.167425	1	1.45E+08		-16390		*PDE4DIP*
cg05370068	2.65E-10	3.66E-07	7.16619	6	35184382	Body;Body	2650	*SCUBE3*	*SCUBE3*
cg08995449	2.66E-10	3.66E-07	7.164967	8	1.02E+08		-67828		*PABPC1*
cg12044654	2.67E-10	3.66E-07	7.164711	8	1.4E+08	Body	35942	*COL22A1*	*COL22A1*
cg26237651	2.67E-10	3.66E-07	7.164213	2	2.2E+08	TES5000;TSS5000	-1804	*PRKAG3;MIR9500*	*MIR9500*
cg08367573	2.72E-10	3.72E-07	7.160395	3	1.95E+08		-49440		*LINC01972*
cg20989899	2.73E-10	3.73E-07	7.159213	18	77921368	Body;Body;Body	15562	*PARD6G-AS1;PARD6G*	*PARD6G-AS1*
cg00108938	2.76E-10	3.74E-07	7.156648	7	1.58E+08	Body;Body;Body;Body;Body	-14024	*PTPRN2*	*LOC100506585*
cg05277146	2.78E-10	3.74E-07	7.155599	2	59212893	Body	-231949	*LINC01122*	*LINC01793*
cg18973735	2.78E-10	3.74E-07	7.155139	15	43822758	3'UTR;TES5000	12953	*MAP1A;PPIP5K1*	*MAP1A*
cg17821649	2.79E-10	3.74E-07	7.15496	1	1403857	Body;TSS5000	-3287	*ATAD3C;ATAD3B*	*ATAD3B*
cg04064828	2.79E-10	3.74E-07	7.154805	10	1.34E+08	TES5000;TES5000;TES5000;TES5000;TES5000;TES5000;Body	2300	*JAKMIP3;DPYSL4*	*DPYSL4*
cg15161251	2.79E-10	3.74E-07	7.154292	19	1305252	TES5000	19381	*EFNA2*	*EFNA2*
cg22855764	2.85E-10	3.80E-07	7.150286	2	1.6E+08	Body;Body;Body;Body;Body;Body;Body	30247	*TANC1*	*TANC1*
cg26921987	2.88E-10	3.83E-07	7.14781	11	1.2E+08	Body;Body;Body	51541	*NECTIN1*	*NECTIN1*
cg04912501	2.88E-10	3.83E-07	7.147382	6	1.67E+08	Body;Body;Body	4130	*RPS6KA2*	*RPS6KA2*
cg12676289	2.89E-10	3.83E-07	7.146515	5	9523352	Body	11908	*SEMA5A*	*SEMA5A-AS1*
cg23197955	2.93E-10	3.87E-07	7.1435	19	8921541	3'UTR;3'UTR	11198	*ZNF558*	*ZNF558*
cg07792978	2.94E-10	3.87E-07	7.142783	16	56626178	TES5000	2739	*MT3*	*MT3*
cg16309460	2.95E-10	3.87E-07	7.142256	3	9403346	TSS1500;TSS1500	-1391	*THUMPD3*	*THUMPD3*
cg15286784	3.00E-10	3.91E-07	-7.13883	20	47895175	TSS1500;Body;Body;Body;Body;TSS200;TSS1500;TSS5000;TSS5000	-3	*ZNFX1;ZFAS1;SNORD12C;SNORD12B;SNORD12*	*ZFAS1*
cg07555102	3.00E-10	3.91E-07	7.138825	1	10696066	TES5000	160637	*CASZ1*	*CASZ1*
cg22077553	3.05E-10	3.97E-07	7.13481	1	1.72E+08	1stExon	96	*MYOC*	*MYOC*
cg14510567	3.10E-10	4.02E-07	7.131527	5	646835	Body;Body	34381	*CEP72*	*CEP72*
cg04259358	3.12E-10	4.04E-07	7.129801	10	1.2E+08		-215881		*CASC2*
cg12612213	3.13E-10	4.04E-07	7.129155	3	1.84E+08	Body	6331	*EPHB3*	*EPHB3*
cg19761682	3.16E-10	4.06E-07	7.127425	10	1.24E+08	Body;Body;Body;Body	-29548	*DMBT1*	*LOC112577516*
cg17279942	3.19E-10	4.08E-07	7.124962	12	1.11E+08	Body	27795	*CUX2*	*CUX2*
cg03159004	3.20E-10	4.08E-07	7.124714	19	46445221	Body	-27185	*NOVA2*	*NANOS2*
cg16881843	3.24E-10	4.12E-07	7.121885	6	42691042	TSS1500;TSS5000	-707	*PRPH2;ATP6V0CP3*	*PRPH2*

Probe	p_adj	q_val	t	Chr	Pos.hg19	locations	dist2TSS	gene.Illumina	gene.nearest
cg06601579	3.25E-10	4.13E-07	7.12106	7	142966	TES5000	6472	*LOC102723672*	*LOC102723672*
cg22877230	3.29E-10	4.17E-07	7.118221	2	2.4E+08	Body;Body	1475	*HDAC4;MGC16025*	*MGC16025*
cg13941555	3.35E-10	4.23E-07	7.114187	1	2565265	TSS1500	-810	*MMEL1*	*MMEL1*
cg00430287	3.36E-10	4.23E-07	7.11383	6	1.16E+08	TSS1500	-822	*FRK*	*FRK*
cg04896013	3.41E-10	4.28E-07	7.110431	15	89760469	Body	4341	*RLBP1*	*RLBP1*
cg19251737	3.41E-10	4.28E-07	7.110195	1	9795376	Body;Body;Body	43852	*CLSTN1*	*PIK3CD*
cg00085732	3.43E-10	4.28E-07	7.109264	17	26800820	1stExon;1stExon;5'UTR;5'UTR	159	*SLC13A2*	*SLC13A2*
cg16854200	3.46E-10	4.31E-07	7.107415	12	5603261	Body;TSS200	-36	*NTF3*	*NTF3*
cg08815359	3.50E-10	4.34E-07	7.104916	3	11606853	TES5000;Body;Body;Body;Body;Body;Body	3419	*ATG7;VGLL4*	*VGLL4*
cg11034708	3.57E-10	4.41E-07	7.100593	11	66436639	TES5000;Body;Body	8719	*RBM4;RBM4B*	*RBM4B*
cg16776231	3.57E-10	4.41E-07	-7.10052	7	1.34E+08	TSS200;TSS200;TSS200	-51	*LRGUK*	*LRGUK*
cg10538486	3.60E-10	4.42E-07	7.098484	15	57810509	Body;Body	-73592	*CGNL1*	*MYZAP*
cg01407576	3.60E-10	4.42E-07	7.098383	7	55515199		79374		*VOPP1*
cg26509870	3.60E-10	4.42E-07	7.09825	8	22076166	TES5000;TES5000;TES5000;TES5000;TES5000	13446	*PHYHIP*	*PHYHIP*
cg19466605	3.61E-10	4.42E-07	7.097621	12	57488961	3'UTR;3'UTR;TES5000;TES5000;TES5000;TES5000;TES5000;TES5000	6065	*NAB2;STAT6*	*NAB2*
cg12736760	3.64E-10	4.43E-07	7.096321	15	99330290	Body;Body;TES5000	2636	*IGF1R;MIR4714*	*MIR4714*
cg13312968	3.66E-10	4.43E-07	7.095001	10	1.14E+08	Body	6576	*GUCY2GP*	*GUCY2GP*
cg25796963	3.66E-10	4.43E-07	7.094661	3	52827657	TES5000;TES5000;TES5000;TES5000;TSS1500	-1126	*ITIH1;ITIH3*	*ITIH3*
cg22097941	3.67E-10	4.43E-07	7.094484	6	32711028	Body	1861	*HLA-DQA2*	*HLA-DQA2*
cg04258189	3.68E-10	4.44E-07	7.093467	11	1.25E+08		-33451		*PKNOX2*
cg26372765	3.69E-10	4.44E-07	7.092953	10	1.32E+08	Body	-23997	*EBF3*	*MIR4297*
cg05253226	3.71E-10	4.45E-07	7.091879	8	1.45E+08	1stExon	11792	*EPPK1*	*EPPK1*
cg25562925	3.74E-10	4.47E-07	7.0899	2	25110049	Body;Body	33057	*ADCY3*	*ADCY3*
cg07992779	3.75E-10	4.47E-07	7.089471	19	15254683		-18086		*ILVBL*
cg10883542	3.80E-10	4.51E-07	7.086689	10	1.19E+08	Body	-7493	*HSPA12A*	*MIR9851*
cg12740857	3.80E-10	4.51E-07	7.086487	20	44237356	Body	-21021	*WFDC9*	*WFDC10A*
cg07811262	3.83E-10	4.52E-07	7.084983	16	50871285		30703		*LINC02168*
cg00132888	3.83E-10	4.52E-07	7.084755	13	26599785	3'UTR;3'UTR	25413	*ATP8A2*	*SHISA2*
cg25112747	3.84E-10	4.52E-07	7.084346	20	60798751	TSS5000;TSS5000	-3417	*HRH3;LOC105369209*	*HRH3*
cg02158038	3.88E-10	4.56E-07	7.081823	3	1.94E+08	Body;Body;Body;Body	14935	*TMEM44*	*TMEM44-AS1*
cg03032497	3.91E-10	4.58E-07	-7.08873	14	61108227	TES5000;TES5000	2294	*SALRNA1;SIX1*	*SALRNA1*
cg13908977	4.07E-10	4.75E-07	-7.07138	16	11945749	TSS1500	-350	*RSL1D1*	*RSL1D1*
cg23528142	4.13E-10	4.80E-07	7.067964	14	1.01E+08	TSS200	-68	*LINC00523*	*LINC00523*
cg18118249	4.14E-10	4.80E-07	7.067675	11	75873027		44519		*WNT11*
cg06644719	4.15E-10	4.80E-07	7.06727	2	2.35E+08		185031		*LINC01891*
cg07575519	4.16E-10	4.80E-07	7.066652	5	16743265	Body	-126098	*MYO10*	*RETREG1*
cg13544966	4.17E-10	4.80E-07	7.066141	10	640085	Body	-34418	*DIP2C*	*LOC101930421*
cg12544392	4.20E-10	4.83E-07	7.064263	19	42544587	Body;Body	25370	*GRIK5*	*GRIK5*
cg20825472	4.23E-10	4.85E-07	7.062828	6	1.49E+08	Body	78667	*UST*	*UST-AS1*
cg14333520	4.24E-10	4.85E-07	7.062202	4	8109007	Body;Body;Body;Body;Body;Body;Body	-35292	*ABLIM2*	*ABLIM2*
cg09582256	4.25E-10	4.85E-07	7.061781	17	78515367	TSS5000;TSS5000	-3270	*RPTOR*	*RPTOR*
cg27021406	4.31E-10	4.91E-07	7.058716	19	19224387	TES5000;TES5000	24900	*SLC25A42*	*TMEM161A*
cg02577788	4.34E-10	4.92E-07	7.057153	10	22770717		-43859		*LOC100499489*
cg03649574	4.36E-10	4.92E-07	7.056176	1	1.58E+08	3'UTR;3'UTR	43915	*KIRREL1*	*LINC01704*
cg26094842	4.37E-10	4.92E-07	7.055506	10	1.14E+08	Body;Body;Body;Body;Body;Body;Body;Body	83019	*VTI1A*	*MIR4295*
cg03605066	4.38E-10	4.92E-07	7.055439	5	1786804		13147		*MRPL36*
cg17881042	4.38E-10	4.92E-07	7.055313	17	78192008	Body;Body;Body;Body;TSS5000;TSS5000;TSS5000;TSS5000	2124	*SGSH;SLC26A11*	*SGSH*
cg20775254	4.39E-10	4.93E-07	7.054464	2	95940705	Body;Body;Body;Body	509	*PROM2*	*PROM2*
cg00161955	4.46E-10	4.99E-07	7.051229	8	82608057	TSS1500	-764	*SLC10A5*	*SLC10A5*
cg00757822	4.49E-10	5.01E-07	7.049739	3	1.55E+08	Body;Body;Body;Body;Body;Body	89210	*PLCH1*	*PLCH1*
cg08215453	4.50E-10	5.01E-07	7.049195	14	41623629		199714		*LINC02315*
cg18852305	4.52E-10	5.01E-07	7.048366	17	4475219		-11347		*GGT6*
cg19282928	4.54E-10	5.02E-07	7.04744	2	2.42E+08	3'UTR;3'UTR;3'UTR	5680	*GPR35*	*GPR35*
cg27060831	4.56E-10	5.03E-07	7.046159	16	48248004	Body;Body;Body;Body;Body	18178	*ABCC11*	*ABCC11*
cg13185030	4.56E-10	5.03E-07	-7.04615	14	55595949	TSS200;TSS200	-20	*LGALS3*	*LGALS3*
cg02358190	4.61E-10	5.06E-07	7.043951	5	66187002	Body;Body;Body;Body	62402	*MAST4*	*MAST4*
cg16392754	4.63E-10	5.07E-07	7.042982	5	1.72E+08	TSS5000;TSS5000;Body;Body;Body;Body	2284	*LOC100268168;RPL26L1*	*RPL26L1*
cg19854900	4.64E-10	5.07E-07	7.042634	7	4265529	Body;Body	-91060	*SDK1*	*LOC105375131*
cg08258667	4.75E-10	5.18E-07	7.037486	1	2.04E+08	Body	-33672	*SOX13*	*ERLNC1*

Probe	p_adj	q_val	t	Chr	Pos.hg19	locations	dist2TSS	gene.Illumina	gene.nearest
cg04839219	4.80E-10	5.23E-07	7.034855	2	2.35E+08	TES5000;TES5000;TES5000;TES5000;TES5000;TES5000;TES5000;TES5000;TES5000;TES5000;TSS1500	-345	*UGT1A8;UGT1A10;UGT1A9;UGT1A7;UGT1A6;UGT1A5;UGT1A4;UGT1A3;UGT1A1;MROH2A*	*MROH2A*
cg18721888	4.83E-10	5.24E-07	7.033773	6	31627126	TES5000;TES5000;TES5000;Body;TES5000;TES5000;TES5000;TES5000;TES5000	1429	*APOM;C6orf47;GPANK1*	*C6orf47*
cg11925872	4.84E-10	5.25E-07	7.032973	11	1.14E+08	Body;Body;Body;Body;Body	70213	*ZBTB16*	*ZBTB16*
cg24968629	4.88E-10	5.27E-07	7.031438	22	46770644	Body	39034	*CELSR1*	*TRMU*
cg20754324	4.95E-10	5.33E-07	7.028272	22	38381626	Body;Body;Body;TSS1500;TSS5000	-1064	*POLR2F;SOX10;MIR4534*	*SOX10*
cg14812313	4.98E-10	5.35E-07	7.026883	6	32797268	Body;Body	9279	*TAP2*	*TAP2*
cg00316223	5.01E-10	5.37E-07	7.025482	2	2.42E+08	Body	20845	*ANO7*	*ANO7*
cg12385811	5.01E-10	5.37E-07	7.02529	5	1.78E+08	Body	-37846	*COL23A1*	*PHYKPL*
cg12712908	5.03E-10	5.37E-07	7.024716	8	21966204	TES5000;TES5000;TES5000;TES5000;TES5000;TES5000;TES5000;TES5000;Body	728	*FAM160B2;NUDT18*	*NUDT18*
cg19992474	5.12E-10	5.45E-07	7.020879	7	1.55E+08		-103674		*INSIG1*
cg25040937	5.15E-10	5.46E-07	7.019539	10	1.13E+08		458966		*GPAM*
cg07148025	5.15E-10	5.46E-07	7.019421	5	1.16E+08		-10706		*LINC02214*
cg14578366	5.26E-10	5.57E-07	7.014584	13	76109220	Body;Body;Body;Body;Body	2788	*COMMD6*	*COMMD6*
cg06966242	5.29E-10	5.58E-07	7.013661	1	4832432	Body;Body	117764	*AJAP1*	*AJAP1*
cg16908901	5.35E-10	5.62E-07	7.011049	1	94054835	TSS5000;Body;Body;Body;Body;TSS5000	-2689	*BCAR3;BCAR3-AS1*	*BCAR3-AS1*
cg14615946	5.35E-10	5.62E-07	7.010826	17	74466087	3'UTR;3'UTR;Body;TES5000;TES5000	2403	*AANAT;RHBDF2*	*AANAT*
cg14044874	5.40E-10	5.65E-07	7.008982	3	14545362	Body	36487	*GRIP2*	*GRIP2*
cg26164234	5.43E-10	5.67E-07	7.007845	10	98946778	TSS1500;Body	-1062	*SLIT1;ARHGAP19-SLIT1*	*SLIT1*
cg01804370	5.45E-10	5.68E-07	7.006835	7	1.49E+08	Body;Body	-15902	*ZNF282*	*ZNF212*
cg23886978	5.53E-10	5.75E-07	7.003741	8	1272628	Body	-21801	*DLGAP2*	*LOC286083*
cg21173532	5.56E-10	5.77E-07	7.002474	1	7701604	Body;Body;Body;Body;Body	-28185	*CAMTA1*	*CAMTA1*
cg27653399	5.59E-10	5.78E-07	7.001402	14	24546521	Body;Body;TES5000;TES5000;TES5000;TES5000	5751	*CPNE6;NRL*	*CPNE6*
cg11515702	5.61E-10	5.80E-07	7.000327	11	67789123	Body;Body;Body;1stExon;Body	-9024	*ALDH3B1*	*NDUFS8*
cg27470307	5.65E-10	5.81E-07	6.998787	11	61106785	Body;Body;Body;Body;Body;Body	6086	*TKFC*	*TKFC*
cg02957433	5.65E-10	5.81E-07	6.998723	14	1.05E+08	Body;Body	5111	*PLD4*	*PLD4*
cg09614629	5.68E-10	5.82E-07	6.997851	7	1.58E+08	Body;Body;Body;Body;Body	337781	*PTPRN2*	*LOC100506585*
cg11276053	5.73E-10	5.87E-07	6.995689	19	46307734	Body	10832	*RSPH6A*	*RSPH6A*
cg06598544	5.81E-10	5.93E-07	6.992899	20	61472147	3'UTR;TES5000;TSS5000	-3770	*COL9A3;TCFL5;DPH3P1*	*DPH3P1*
cg20420873	5.82E-10	5.93E-07	6.992189	13	97639223	TSS5000;Body;Body;Body;Body;Body	-2797	*LINC00359;OXGR1*	*LINC00359*
cg02923490	5.91E-10	6.00E-07	6.989143	6	37872019	Body	84517	*ZFAND3*	*ZFAND3*
cg24704405	5.96E-10	6.05E-07	6.986986	2	30914938		115374		*CAPN13*
cg03844762	5.98E-10	6.05E-07	6.986415	15	89066817	Body;Body;Body;Body;Body;Body;Body;Body;Body;Body;Body	22789	*DET1*	*DET1*
cg03514843	6.01E-10	6.07E-07	6.985136	2	27301195	TSS1500	-287	*EMILIN1*	*EMILIN1*
cg09919727	6.04E-10	6.08E-07	6.984321	14	1.05E+08	TSS1500	-859	*LINC00638*	*LINC00638*
cg07728485	6.05E-10	6.08E-07	6.983776	5	1279582	Body;Body;Body;Body	15601	*TERT*	*TERT*
cg14421371	6.13E-10	6.15E-07	6.980744	9	1.37E+08	Body;Body	-55154	*VAV2*	*SARDH*
cg07826522	6.16E-10	6.15E-07	6.979704	5	1.35E+08	Body	35615	*C5orf66*	*C5orf66-AS2*
cg00969271	6.19E-10	6.15E-07	6.978883	9	1.17E+08	TES5000;Body;TES5000	984	*ORM1;ORM2;AKNA*	*ORM2*
cg12504163	6.19E-10	6.15E-07	6.978693	7	1.48E+08		36194		*C7orf33*
cg26041493	6.21E-10	6.15E-07	6.978169	11	66306861	Body;3'UTR	6658	*ZDHHC24*	*ZDHHC24*
cg16973107	6.21E-10	6.15E-07	6.978124	12	57828180	TSS5000;TSS5000;TSS5000;TSS5000;TSS5000;TSS5000;TSS5000;TSS5000;TSS5000;TSS5000;TSS1500	-386	*R3HDM2;INHBC*	*INHBC*
cg07259382	6.28E-10	6.21E-07	6.975477	4	1.65E+08	TSS5000;Body	-1542	*MARCHF1*	*MARCHF1*
cg18564167	6.29E-10	6.21E-07	6.975176	6	53720312	Body	60510	*LRRC1*	*LRRC1*
cg27479455	6.43E-10	6.33E-07	6.970252	19	7529920	Body;Body;Body;Body	24001	*ARHGEF18*	*PEX11G*
cg02710090	6.44E-10	6.33E-07	6.969901	1	2.07E+08	TSS1500;Body;TSS1500;TSS1500	-706	*YOD1;PFKFB2*	*YOD1*
cg17090954	6.47E-10	6.34E-07	6.968954	8	1364458	Body	-113631	*DLGAP2*	*LOC286083*
cg26146855	6.48E-10	6.34E-07	6.968638	7	1660603	TES5000	6498	*TFAMP1*	*TFAMP1*
cg17919821	6.50E-10	6.35E-07	6.967794	3	8713619		-19849		*SSUH2*
cg02817007	6.51E-10	6.35E-07	6.967466	3	50379334	TSS1500;TSS5000;TSS5000;TSS1500;TSS1500;TES5000;Body;Body	-967	*RASSF1;RASSF1-AS1;ZMYND10*	*RASSF1*
cg25062951	6.54E-10	6.36E-07	6.966629	7	817576	Body;Body	-37617	*DNAAF5*	*SUN1*
cg17621966	6.57E-10	6.37E-07	6.965615	20	33147743	3'UTR;3'UTR;TES5000	1222	*MAP1LC3A;PIGU*	*MAP1LC3A*
cg18494672	6.59E-10	6.38E-07	6.964933	5	54133279		-93193		*LOC102467080*
cg27405799	6.70E-10	6.47E-07	6.961131	10	22514531		-15619		*EBLN1*
cg10438893	6.77E-10	6.53E-07	6.958868	19	1924761	3'UTR;3'UTR;3'UTR	-16409	*SCAMP4*	*CSNK1G2*
cg20841156	6.81E-10	6.55E-07	6.957444	12	1.21E+08	TES5000;Body;Body;Body;Body	13685	*PXN-AS1;PXN*	*PXN-AS1*
cg05757007	6.84E-10	6.57E-07	6.956475	6	1.01E+08	Body	-142004	*ASCC3*	*SIM1*
cg24542821	6.90E-10	6.61E-07	6.954597	2	2.2E+08	TES5000	13831	*WNT10A*	*WNT10A*
cg22542373	6.93E-10	6.62E-07	6.953752	4	38192482		70908		*TBC1D1*

Probe	p_adj	q_val	t	Chr	Pos.hg19	locations	dist2TSS	gene.Illumina	gene.nearest
cg04762890	7.01E-10	6.68E-07	6.951304	16	58077795	Body	18138	*MMP15*	*MMP15*
cg27617132	7.04E-10	6.70E-07	6.950238	10	1.35E+08	Body;Body	49093	*INPP5A*	*INPP5A*
cg20745568	7.08E-10	6.71E-07	6.949108	17	40321545	Body	11615	*KCNH4*	*KCNH4*
cg12510201	7.08E-10	6.71E-07	6.949063	10	1.18E+08	3'UTR;3'UTR;3'UTR;3'UTR;3'UTR;3'UTR	136412	*GFRA1*	*SNORD158*
cg05788437	7.24E-10	6.84E-07	-6.94392	3	1.97E+08	TSS1500;Body;1stExon;TSS1500	127	*RUBCN;FYTTD1*	*FYTTD1*
cg18089146	7.25E-10	6.84E-07	6.943695	5	60472989	Body	-14694	*SMIM15-AS1*	*SMIM15*
cg04909854	7.29E-10	6.84E-07	6.942498	1	1.7E+08	TSS1500	-595	*LINC01142*	*LINC01142*
cg04486673	7.30E-10	6.84E-07	6.942324	17	66191540	TSS5000	-3260	*ARHGAP27P2*	*ARHGAP27P2*
cg12658387	7.30E-10	6.84E-07	6.94206	17	81033543	TSS5000	-3941	*METRNL*	*METRNL*
cg13444966	7.31E-10	6.84E-07	6.941826	11	2705357	Body;Body;Body	15871	*KCNQ1;KCNQ1OT1*	*KCNQ1OT1*
cg01731204	7.33E-10	6.84E-07	6.941325	1	31194554	Body;Body	1878	*MATN1;MATN1-AS1*	*MATN1*
cg10024799	7.40E-10	6.89E-07	6.939264	12	2641381	Body;Body;Body;Body;Body;Body;Body;Body;Body;Body;Body;Body;Body;Body;Body;Body;Body;Body;Body;Body;Body;Body;Body	140005	*CACNA1C*	*CACNA1C-AS2*
cg07558734	7.41E-10	6.89E-07	6.938843	15	45827540	Body;TSS5000	-1908	*HMGN2P46;SNORA41B*	*SNORA41B*
cg16646726	7.54E-10	6.99E-07	6.935062	7	22641501		38546		*LOC100506178*
cg08312198	7.58E-10	7.02E-07	6.933715	2	2.36E+08	Body;Body;Body;Body;Body;Body	24974	*SH3BP4*	*SH3BP4*
cg03390183	7.62E-10	7.05E-07	6.932546	3	36779043	1stExon	2309	*DCLK3*	*DCLK3*
cg17303922	7.73E-10	7.13E-07	6.929449	7	44117899	Body;Body;Body;Body;Body;Body;Body;TSS5000	4230	*POLM;MIR6838*	*POLM*
cg13008301	7.84E-10	7.22E-07	-6.92634	20	54978749	Body;Body;Body	-8342	*CSTF1*	*CASS4*
cg05005332	7.87E-10	7.23E-07	6.925405	9	98979142		-29602		*HSD17B3-AS1*
cg22011888	7.89E-10	7.23E-07	6.924953	11	63856712	Body	-14649	*MACROD1*	*FLRT1*
cg14810242	7.95E-10	7.27E-07	6.92325	2	1.02E+08	Body;Body;Body;Body	4566	*RFX8*	*RFX8*
cg26706961	8.05E-10	7.35E-07	6.920451	2	1.75E+08		-20994		*LINC01960*
cg26343889	8.10E-10	7.37E-07	6.919077	15	68848244	Body	-23087	*CORO2B*	*CORO2B*
cg01708964	8.11E-10	7.37E-07	6.918813	7	44181791	TSS1500;TES5000;TES5000;TES5000;TES5000;TES5000;TES5000;TES5000	-860	*MYL7;GCK*	*MYL7*
cg18184053	8.14E-10	7.38E-07	6.91809	10	5882739		-27227		*GDI2*
cg19003397	8.30E-10	7.51E-07	6.913798	8	82432003		11622		*FABP12*
cg14074428	8.34E-10	7.54E-07	6.912605	5	1.54E+08		-32982		*MIR3141*
cg07129978	8.36E-10	7.54E-07	6.912116	17	39791049	Body	5402	*KRT42P*	*KRT42P*
cg17001401	8.46E-10	7.60E-07	6.90947	1	1.54E+08	Body;Body	-46805	*IL6R*	*IL6R-AS1*
cg07099121	8.46E-10	7.60E-07	6.909435	11	71146096	3'UTR;3'UTR	-11696	*DHCR7*	*FLJ42102*
cg09283977	8.48E-10	7.60E-07	6.908838	2	2.4E+08	Body	34733	*HDAC4*	*MGC16025*
cg15010109	8.53E-10	7.63E-07	6.907572	6	1.35E+08		53663		*ALDH8A1*
cg14173736	8.63E-10	7.69E-07	6.90503	9	1.36E+08	TES5000	-7954	*CEL*	*CELP*
cg10811945	8.64E-10	7.69E-07	6.904914	1	1.57E+08	Body;Body;Body;Body;Body;TSS1500	-628	*SH2D2A;NTRK1*	*NTRK1*
cg01674405	8.65E-10	7.69E-07	6.904618	9	1.39E+08		-18376		*UBAC1*
cg04632997	8.70E-10	7.72E-07	6.90328	20	60904163	Body;TES5000	3450	*LAMA5;MIR4758*	*MIR4758*
cg08648138	8.72E-10	7.72E-07	6.902724	1	92247732	Body;Body;Body;Body	103976	*TGFBR3*	*TGFBR3*
cg05531689	8.73E-10	7.72E-07	6.902538	2	26729139	Body;Body	-28147	*OTOF*	*OTOF*
cg27420946	8.81E-10	7.77E-07	6.90039	15	32404311	Body;Body;Body	81594	*CHRNA7*	*CHRNA7*
cg03749915	8.84E-10	7.78E-07	6.899741	7	1.59E+08	Body;TES5000;TES5000;TES5000;TES5000	16132	*LINC00689;VIPR2*	*LINC00689*
cg16874347	8.85E-10	7.78E-07	6.899464	2	86450216	Body;Body;Body;Body;Body;Body	23637	*REEP1*	*MRPL35*
cg12726658	8.91E-10	7.81E-07	6.897992	11	75927166		-9620		*WNT11*
cg04208996	8.95E-10	7.83E-07	6.897037	8	1.44E+08		-11837		*MAFA-AS1*
cg07017532	9.01E-10	7.87E-07	6.895533	1	12211767		-15232		*MIR7846*
cg02433807	9.04E-10	7.88E-07	6.894753	16	11234924	Body	115115	*CLEC16A*	*SOCS1*
cg09900893	9.08E-10	7.90E-07	6.893836	1	26865376	Body;TSS5000	-4226	*RPS6KA1*	*RPS6KA1*
cg20841588	9.17E-10	7.97E-07	6.891472	2	1.77E+08	TSS1500	-801	*MIR1246*	*MIR1246*
cg11177550	9.19E-10	7.97E-07	6.891038	1	20470903	Body;Body	5088	*PLA2G2F*	*PLA2G2F*
cg03708680	9.25E-10	8.00E-07	6.889703	10	1.35E+08		49988		*LINC01168*
cg08568447	9.45E-10	8.16E-07	6.884915	17	7328520	TES5000;TSS1500;TSS5000;TSS5000	-414	*SPEM1;SPEM2;SPEM3*	*SPEM2*
cg06898293	9.48E-10	8.16E-07	6.884262	7	1.15E+08	Body	31692	*LINC01393*	*LINC01393*
cg01275566	9.48E-10	8.16E-07	6.884127	10	79684776	Body;TSS5000	1737	*DLG5;DLG5-AS1*	*DLG5*
cg08651187	9.51E-10	8.16E-07	6.883508	1	59726071		-36413		*FGGY*
cg23915701	9.56E-10	8.18E-07	6.882335	1	2.35E+08		45850		*LOC101927851*
cg20032789	9.56E-10	8.18E-07	6.882333	10	30336565	5'UTR;1stExon;1stExon;5'UTR	11950	*JCAD*	*JCAD*
cg19006220	9.61E-10	8.20E-07	6.881216	2	4050719		28969		*LOC105373394*
cg09559163	9.67E-10	8.24E-07	6.879765	10	1.3E+08		214647		*LINC01163*
cg03049693	9.75E-10	8.29E-07	6.877966	11	89465190		21724		*TRIM77*
cg05764121	9.77E-10	8.29E-07	6.877602	6	5082456	Body;Body;Body;TSS5000	-3118	*LYRM4-AS1;LYRM4;PPP1R3G*	*PPP1R3G*

Probe	p_adj	q_val	t	Chr	Pos.hg19	locations	dist2TSS	gene.Illumina	gene.nearest
cg04527220	9.85E-10	8.35E-07	6.875611	14	1.06E+08	5'UTR;5'UTR;Body;Body	9806	*GPR132*	*GPR132*
cg20249839	1.01E-09	8.52E-07	6.870614	19	49658532	TES5000;TES5000;5'UTR;TSS5000;TSS5000;TSS5000;TSS5000;TSS5000;TSS5000	121	*PPFIA3;HRC;TRPM4*	*HRC*
cg19260189	1.02E-09	8.59E-07	6.8683	12	1.07E+08	Body;Body;5'UTR	76	*LOC100287944;RFX4*	*RFX4*
cg03648690	1.02E-09	8.60E-07	6.867766	13	99383591	Body	21335	*SLC15A1*	*SLC15A1*
cg03475821	1.03E-09	8.63E-07	6.866494	14	1.05E+08	Body	583	*LINC00638*	*LINC00638*
cg25675673	1.03E-09	8.68E-07	6.86477	6	88617150	Body	122573	*LOC101928911*	*LOC101928911*
cg24009650	1.04E-09	8.69E-07	6.864029	12	48722169	TSS1500	-593	*H1-7*	*H1-7*
cg15857627	1.04E-09	8.69E-07	6.863828	12	1.31E+08	Body;Body;Body;Body;Body	26015	*RIMBP2*	*RIMBP2*
cg18568657	1.04E-09	8.70E-07	6.863148	3	48517026	Body;Body;Body;Body;Body;TSS5000;TSS5000	-2459	*SHISA5*	*SHISA5*
cg03399655	1.05E-09	8.73E-07	6.861805	3	1.13E+08	TES5000;TES5000;Body;Body;Body	20113	*CD200R1L-AS1;CD200R1L*	*CD200R1L-AS1*
cg01698177	1.06E-09	8.78E-07	6.860252	16	19870092	TES5000;TES5000;TES5000;TES5000;TES5000;3'UTR;TES5000	26078	*IQCK;GPRC5B*	*GPRC5B*
cg06275635	1.06E-09	8.84E-07	6.858362	1	1.53E+08	TSS1500	-800	*PGLYRP3*	*PGLYRP3*
cg12783693	1.07E-09	8.86E-07	6.857316	14	66532279		-420809		*CCDC196*
cg22724639	1.07E-09	8.86E-07	6.857003	8	12670236	TSS1500;TSS1500;Body	-1249	*LOC340357;LINC00681*	*LOC340357*
cg16905004	1.07E-09	8.86E-07	6.856551	6	32317028	Body;Body;Body;Body;Body;Body	22652	*TSBP1-AS1;TSBP1*	*TSBP1*
cg01972919	1.09E-09	8.95E-07	6.853661	4	736924	Body;Body	37304	*PCGF3*	*PCGF3*
cg21906911	1.09E-09	8.95E-07	6.85342	17	18169035	3'UTR;3'UTR;3'UTR	4612	*MIEF2*	*MIEF2*
cg04400620	1.09E-09	8.95E-07	6.852682	16	30385284	TSS5000;Body;Body;TSS1500;TES5000;TES5000;TSS5000;TSS5000	-838	*TBC1D10B;MYLPF;SEPTIN1;ZNF48*	*MYLPF*
cg01657380	1.09E-09	8.95E-07	6.852637	12	53901523	TES5000;TES5000;TES5000;TSS1500;TSS1500;Body;Body;Body;TES5000;TES5000;TES5000;TES5000;TES5000;TES5000;TES5000	-255	*TARBP2;NPFF;ATF7-NPFF;ATF7*	*NPFF*
cg05491376	1.09E-09	8.95E-07	6.852329	17	7641552	Body;Body	-16085	*DNAH2*	*RPL29P2*
cg16184737	1.10E-09	8.97E-07	6.851021	7	2679971	Body	8352	*TTYH3*	*TTYH3*
cg13551098	1.10E-09	8.97E-07	6.850832	14	1.01E+08	TSS5000;TSS5000;TSS5000;TSS5000;TSS5000;TSS5000;TSS5000;TSS5000;TSS5000;TSS5000;TSS5000;TSS5000;TSS5000;TSS5000;TSS5000	-2974	*MEG3*	*MEG3*
cg19677203	1.10E-09	8.97E-07	-6.85045	6	31783322	TSS1500;5'UTR	3	*HSPA1L;HSPA1A*	*HSPA1A*
cg10690277	1.11E-09	9.04E-07	6.848437	7	55113199	Body;Body;Body;Body;Body;Body;Body;Body	26475	*EGFR*	*EGFR*
cg05825053	1.12E-09	9.07E-07	6.84725	16	11697522		-16200		*LITAF*
cg22064390	1.12E-09	9.07E-07	6.846871	7	2615547	Body;Body;Body;Body;Body	16935	*IQCE*	*IQCE*
cg05781049	1.12E-09	9.07E-07	6.846367	10	44374390	Body	19532	*LINC00840*	*LINC00840*
cg24771111	1.13E-09	9.10E-07	6.845066	5	73893040		-42807		*HEXB*
cg27373765	1.13E-09	9.10E-07	6.844615	5	1.77E+08	TSS5000;Body;Body;Body;Body	-4810	*DBN1*	*DBN1*
cg14436098	1.13E-09	9.10E-07	6.844515	4	4690634	Body	24582	*STX18-AS1*	*SNORD162*
cg17811167	1.14E-09	9.13E-07	-6.84349	17	36861499	TES5000;TSS5000;TSS200	-26	*LOC105371763;MIR4734;MLLT6*	*MLLT6*
cg24746106	1.14E-09	9.15E-07	6.842441	13	1.14E+08	Body;Body;Body;Body;Body;Body;Body;Body	9330	*MCF2L*	*MCF2L*
cg07449447	1.15E-09	9.20E-07	6.840374	14	55228808	Body;Body;Body	7258	*SAMD4A*	*SAMD4A*
cg03765822	1.15E-09	9.20E-07	6.840362	12	69519871		-113477		*CPSF6*
cg10300728	1.16E-09	9.20E-07	6.840169	13	1.13E+08		24701		*SOX1-OT*
cg05008825	1.16E-09	9.22E-07	6.83912	7	94045725	Body	21519	*COL1A2*	*COL1A2*
cg18483750	1.17E-09	9.28E-07	6.837324	5	78406928	TSS1500	-703	*BHMT*	*BHMT*
cg09814480	1.17E-09	9.29E-07	6.836811	7	1.35E+08	TSS1500	-545	*STRA8*	*STRA8*
cg25407543	1.18E-09	9.30E-07	6.836006	8	21945413	TSS1500;TSS1500;TSS1500;TSS1500;TSS1500;TSS1500;TSS1500;TSS1500	-1247	*FAM160B2*	*FAM160B2*
cg07291317	1.18E-09	9.32E-07	6.835263	5	16701194	Body	-84027	*MYO10*	*RETREG1*
cg07001334	1.19E-09	9.35E-07	6.834217	10	1.13E+08	Body	101040	*RBM20*	*RBM20*
cg07949863	1.19E-09	9.38E-07	6.832977	20	17715916	Body;Body;Body	35324	*BANF2*	*BANF2*
cg08550490	1.20E-09	9.38E-07	6.832552	1	2.04E+08	Body	24400	*PLEKHA6*	*PLEKHA6*
cg20323436	1.20E-09	9.38E-07	6.832261	12	97144035	Body;Body	-156999	*CFAP54*	*NEDD1*
cg17412102	1.20E-09	9.39E-07	6.831606	1	2119531	TES5000;TES5000;TES5000;TES5000;TES5000;TES5000;TES5000;TES5000;TES5000;TSS5000;Body;Body;Body;Body;Body;TES5000;TES500 0;TES5000;TES5000;TES5000	-4217	*PRKCZ;PRKCZ-AS1;FAAP20*	*PRKCZ-AS1*
cg04341461	1.21E-09	9.42E-07	6.830642	1	2410006	Body;Body;Body	2253	*PLCH2*	*PLCH2*
cg01469715	1.22E-09	9.48E-07	6.828787	16	27900716	Body;Body;Body;TSS1500	-1238	*GSG1L*	*GSG1L*
cg03249590	1.23E-09	9.56E-07	-6.82648	12	57040199	TSS1500;TSS5000;TSS1500	-431	*ATP5F1B;SNORD59B;SNORD59A*	*ATP5F1B*
cg00549018	1.23E-09	9.56E-07	6.826182	19	48968137	3'UTR;TSS5000;TSS5000	-4327	*KCNJ14;CYTH2*	*CYTH2*
cg09644157	1.23E-09	9.56E-07	6.825798	12	1.25E+08	Body;Body;Body	-74424	*NCOR2*	*MIR6880*
cg23678322	1.24E-09	9.56E-07	6.824767	15	41801319	Body;Body;Body	4706	*LTK*	*LTK*
cg06993420	1.24E-09	9.56E-07	6.824581	6	1.69E+08		169095		*LOC105378146*
cg16213787	1.24E-09	9.56E-07	6.824478	2	1.73E+08		129663		*DLX2-DT*
cg15923688	1.25E-09	9.58E-07	6.823059	17	79623755	TSS5000;TSS200;Body;TSS200;Body	-183	*PDE6G*	*PDE6G*
cg02775369	1.25E-09	9.58E-07	6.823034	16	56316221	Body;Body	36790	*GNAO1*	*MIR3935*
cg26251854	1.25E-09	9.58E-07	6.822368	1	2.29E+08		-154734		*RAB4A*
cg13958625	1.25E-09	9.58E-07	6.822365	20	49123093	TSS5000;TSS5000	-3764	*PTPN1*	*PTPN1*
cg03131736	1.26E-09	9.58E-07	6.821637	19	50985397	Body;Body	-5292	*EMC10*	*FAM71E1*

Probe	p_adj	q_val	t	Chr	Pos.hg19	locations	dist2TSS	gene.Illumina	gene.nearest
cg09657256	1.26E-09	9.58E-07	6.82123	17	42153683	3'UTR;3'UTR;Body;Body;TES5000;TES5000	5405	*G6PC3;HDAC5*	*G6PC3*
cg15986469	1.26E-09	9.58E-07	6.821177	4	1192053	Body;Body	10697	*SPON2;LOC100130872*	*SPON2*
cg23536555	1.26E-09	9.58E-07	6.821067	12	1.02E+08	Body;Body;Body;Body;Body;Body;Body;Body;Body;Body	-31533	*MYBPC1*	*CHPT1*
cg26348616	1.27E-09	9.66E-07	6.818836	20	39661470	Body	4009	*TOP1*	*TOP1*
cg07489488	1.28E-09	9.70E-07	6.817494	10	400897	Body	174964	*DIP2C*	*ZMYND11*
cg26226702	1.30E-09	9.82E-07	6.81379	14	1.06E+08	Body;Body;Body	-39853	*PACS2*	*BRF1*
cg13484848	1.30E-09	9.82E-07	6.813662	17	79131053	Body	-8253	*AATK*	*PVALEF*
cg03605666	1.30E-09	9.82E-07	6.813645	19	3383594	Body;Body;Body;Body;Body	17014	*NFIC*	*NFIC*
cg15573998	1.31E-09	9.86E-07	6.811873	2	51228125	Body;Body;Body;Body;Body;Body;Body;Body;Body;Body;Body;Body;Body;Body;Body;Body;Body;Body;Body	31145	*NRXN1*	*NRXN1*
cg16684958	1.31E-09	9.86E-07	6.811666	7	75615977	3'UTR;3'UTR;TES5000;TES5000;TES5000;TES5000	7936	*POR;TMEM120A*	*TMEM120A*
cg08735172	1.31E-09	9.86E-07	6.811652	11	45722809		-9483		*MIR7154*
cg16715363	1.32E-09	9.87E-07	6.810989	11	71711087	TES5000;TES5000;TES5000;TES5000;Body;Body;Body;Body;Body;Body;Body;TES5000;TES5000;TES5000	115	*RNF121;IL18BP;NUMA1*	*IL18BP*
cg13273097	1.32E-09	9.88E-07	-6.81014	3	1.53E+08	TSS1500	-701	*RAP2B*	*RAP2B*
cg18454774	1.32E-09	9.88E-07	6.810002	10	3378983	Body	18097	*LOC105376360*	*LOC105376360*
cg18574298	1.34E-09	9.98E-07	6.806733	21	47015398	Body	1831	*LINC01694*	*LINC01694*
cg11328697	1.34E-09	9.98E-07	6.806501	17	39971602	TSS5000;Body	2347	*P3H4;FKBP10*	*FKBP10*
cg02637309	1.35E-09	9.98E-07	6.806311	11	1.3E+08	Body	27543	*ST14*	*ST14*
cg13691901	1.35E-09	9.98E-07	6.806018	7	2011787	Body;Body;Body;Body;Body	-31580	*MAD1L1*	*MAD1L1*
cg16086782	1.35E-09	9.98E-07	6.805859	11	70741332	Body	-22859	*SHANK2*	*MIR3664*
cg25811386	1.35E-09	9.98E-07	6.80557	12	49315068	Body;Body;TES5000;TES5000;TES5000	3703	*CCDC65;FKBP11*	*FKBP11*
cg14102880	1.36E-09	1.01E-06	6.803642	17	915634	Body;Body;Body;Body;Body;Body;Body	10165	*ABR*	*MIR3183*
cg15223066	1.37E-09	1.01E-06	6.801707	10	33270717		-23559		*ITGB1*
cg00243187	1.38E-09	1.01E-06	6.801109	11	1.29E+08		-8946		*KCNJ1*
cg13464321	1.38E-09	1.01E-06	6.800639	9	1.36E+08	Body;Body	23166	*ADAMTSL2*	*ADAMTSL2*
cg04014002	1.39E-09	1.02E-06	6.799396	1	33206468	TSS1500	-962	*KIAA1522*	*KIAA1522*
cg13156411	1.39E-09	1.02E-06	6.798957	3	46919454	Body;TSS5000	244	*PTH1R*	*PTH1R*
cg08198228	1.39E-09	1.02E-06	6.798345	2	1.1E+08	Body	-30539	*SH3RF3*	*MIR4265*
cg19044706	1.40E-09	1.02E-06	6.797432	2	2.42E+08	Body;Body;Body	1767	*MAB21L4*	*MAB21L4*
cg17667592	1.40E-09	1.02E-06	6.796822	11	1.23E+08	3'UTR;3'UTR;3'UTR;3'UTR;3'UTR;3'UTR;3'UTR;3'UTR;TES5000;TES5000	29315	*GRAMD1B;SCN3B*	*SCN3B*
cg12640180	1.41E-09	1.02E-06	6.796553	8	1.44E+08		32736		*LY6H*
cg13299743	1.41E-09	1.02E-06	6.796264	6	1523751		-82014		*FOXCUT*
cg11590213	1.42E-09	1.03E-06	6.794714	5	66331682	Body;Body;Body;Body;Body	31200	*MAST4*	*MAST4*
cg02444226	1.42E-09	1.03E-06	6.794668	2	66545288		40172		*MIR4778*
cg04367958	1.42E-09	1.03E-06	6.793881	16	11371350	TSS5000;TSS1500;TSS1500;TSS1500;TSS1500;TSS1500;TSS1500;TES5000	-1013	*PRM3;PRM2;PRM1*	*PRM2*
cg26786924	1.43E-09	1.03E-06	6.793487	2	2.19E+08	Body;TSS1500;TES5000	-247	*PNKD;CATIP-AS2*	*PNKD*
cg06535849	1.43E-09	1.03E-06	6.793109	16	81925084	Body	112189	*PLCG2*	*PLCG2*
cg17286712	1.44E-09	1.04E-06	6.790783	3	1.94E+08		33972		*LINC02038*
cg08530632	1.44E-09	1.04E-06	6.790521	1	89037170	Body	-112656	*PKN2-AS1*	*PKN2*
cg04913653	1.45E-09	1.04E-06	6.790365	3	21790232	Body	2470	*ZNF385D*	*ZNF385D*
cg23256630	1.45E-09	1.04E-06	-6.79559	19	45681987	TSS1500;TSS1500;TSS1500;TSS1500;TSS200	-54	*TRAPPC6A;BLOC1S3*	*BLOC1S3*
cg06270545	1.45E-09	1.04E-06	6.789434	15	65385417	3'UTR	13280	*UBAP1L*	*UBAP1L*
cg23894219	1.46E-09	1.04E-06	6.788566	1	1356565	5'UTR;5'UTR;5'UTR;TSS5000	91	*ANKRD65;TMEM88B*	*ANKRD65*
cg10904854	1.46E-09	1.04E-06	6.788483	3	1.86E+08		64489		*NMRAL2P*
cg00636675	1.46E-09	1.04E-06	6.788163	7	1.57E+08		46644		*MIR153-2*
cg11784742	1.46E-09	1.04E-06	6.787986	10	88722838	3'UTR;3'UTR	4483	*SNCG*	*SNCG*
cg04879750	1.47E-09	1.04E-06	6.786268	12	1.31E+08		34650		*LINC02419*
cg18707417	1.50E-09	1.06E-06	6.78138	3	46937363	Body;Body	13625	*PTH1R*	*PTH1R*
cg03284113	1.51E-09	1.06E-06	6.78128	19	1235093	Body	2911	*CBARP*	*CBARP*
cg21152351	1.51E-09	1.06E-06	6.781038	6	16057773		-71543		*MYLIP*
cg27295042	1.51E-09	1.06E-06	6.78048	1	10738569	Body;Body	118134	*CASZ1*	*CASZ1*
cg26718357	1.52E-09	1.07E-06	6.779639	8	22554232	TSS5000;TSS5000;TSS5000	-3239	*EGR3*	*EGR3*
cg01420369	1.52E-09	1.07E-06	6.779509	12	6304565	TSS5000;TSS5000	-4546	*CD9*	*CD9*
cg15578176	1.53E-09	1.07E-06	6.778345	1	1201631	Body;Body;Body;Body	7603	*UBE2J2*	*UBE2J2*
cg17729668	1.53E-09	1.07E-06	6.777502	13	30727927	TSS1500	-716	*LINC00384*	*LINC00384*
cg20480899	1.53E-09	1.07E-06	6.77701	12	11548867	TSS1500;TSS1500	-366	*PRB1;PRB2*	*PRB2*
cg02367918	1.54E-09	1.07E-06	6.776607	19	19289281	Body;Body;Body;Body;TES5000	-8209	*BORCS8-MEF2B;BORCS8*	*MEF2B*
cg16366073	1.54E-09	1.08E-06	6.775765	21	32185576	Body	16	*KRTAP8-1*	*KRTAP8-1*
cg00454856	1.55E-09	1.08E-06	6.774349	16	58030811	Body;TSS5000;TSS5000;TSS5000;TSS5000;TSS5000	-2695	*ZNF319;USB1*	*USB1*
cg03008241	1.56E-09	1.09E-06	6.772677	8	1.45E+08	Body;Body;Body;Body;Body;Body;Body;Body	21531	*PLEC*	*PLEC*
cg03679716	1.58E-09	1.09E-06	6.771038	16	87379916	Body;Body	28802	*FBXO31*	*LOC101928659*

Probe	p_adj	q_val	t	Chr	Pos.hg19	locations	dist2TSS	gene.Illumina	gene.nearest
cg12299478	1.58E-09	1.09E-06	6.771017	10	1.12E+08		-23498		*LOC105378479*
cg24986469	1.58E-09	1.09E-06	6.770967	14	1.03E+08	3'UTR;3'UTR;3'UTR;3'UTR	-14144	*TRAF3*	*AMN*
cg05900058	1.58E-09	1.09E-06	6.770959	17	7690396	Body	32759	*DNAH2*	*RPL29P2*
cg15879620	1.59E-09	1.10E-06	6.768956	12	45032094	Body;Body;Body;Body;Body	237350	*NELL2*	*NELL2*
cg26549601	1.59E-09	1.10E-06	6.768846	10	1.35E+08	Body;Body	39291	*INPP5A*	*NKX6-2*
cg07727170	1.61E-09	1.10E-06	6.767012	15	70458214		-67958		*TLE3*
cg25402979	1.62E-09	1.11E-06	6.764952	12	58068659		-19078		*OS9*
cg02434736	1.62E-09	1.11E-06	6.764783	17	67463366	Body	18307	*MAP2K6*	*LOC105371880*
cg10213114	1.63E-09	1.11E-06	6.763778	7	98885577	Body	14654	*MYH16*	*MYH16*
cg02426611	1.63E-09	1.11E-06	6.763514	16	87681276	Body;Body	45163	*JPH3*	*JPH3*
cg15618758	1.63E-09	1.11E-06	6.763157	6	1.57E+08	Body;Body;Body;Body	-67969	*ARID1B*	*MIR4466*
cg15246825	1.64E-09	1.12E-06	6.762189	6	30956796	3'UTR;Body	5302	*MUC21*	*MUC21*
cg25948075	1.65E-09	1.12E-06	6.761162	3	1.26E+08	Body;Body	45373	*TXNRD3*	*TXNRD3*
cg08518101	1.65E-09	1.12E-06	6.761134	16	83786701	Body;Body;Body;Body	50880	*CDH13*	*LOC102724163*
cg08878651	1.65E-09	1.12E-06	6.761089	17	30066757		119224		*COPRS*
cg02935562	1.65E-09	1.12E-06	6.761033	16	19834374	Body;Body;Body;Body;Body	61796	*IQCK*	*GPRC5B*
cg15506060	1.65E-09	1.12E-06	-6.76076	19	42432556	5'UTR;TSS1500	317	*ERFL;LOC100505585*	*ERFL*
cg25052847	1.66E-09	1.12E-06	6.759699	2	1.62E+08		-226037		*TANK*
cg11673964	1.67E-09	1.12E-06	6.758724	8	1.46E+08	Body;Body;Body	23407	*ARHGAP39*	*ARHGAP39*
cg00500540	1.67E-09	1.12E-06	6.757844	16	56394104	TES5000;TES5000;TES5000;TES5000	64797	*GNAO1;AMFR*	*AMFR*
cg15210427	1.69E-09	1.13E-06	6.755685	20	23548991	1stExon	130	*CST9L*	*CST9L*
cg22421804	1.69E-09	1.13E-06	6.755492	5	54100067		-59981		*LOC102467080*
cg11584499	1.71E-09	1.14E-06	6.753203	1	38322941	1stExon;TES5000;TES5000;TES5000;TES5000;TES5000;TES5000;TES5000;TES5000;TES5000;TES5000;TES5000;TES5000;TES5000;TES5000	2323	*MTF1;INPP5B*	*MTF1*
cg07671695	1.71E-09	1.14E-06	6.752926	16	67421302	TES5000;TES5000;TES5000;TES5000	6099	*LRRC36;TPPP3*	*TPPP3*
cg04182114	1.72E-09	1.15E-06	6.751785	15	63116019	Body;TSS200	-136	*TLN2;MIR190A*	*MIR190A*
cg07444005	1.72E-09	1.15E-06	6.751445	13	21678461		-36191		*SAP18*
cg14242024	1.72E-09	1.15E-06	6.751297	9	1.39E+08	Body	20287	*NACC2*	*NACC2*
cg01502320	1.72E-09	1.15E-06	6.751053	17	16875558	TSS200;TES5000	-126	*TNFRSF13B;LOC284191*	*TNFRSF13B*
cg14006905	1.73E-09	1.15E-06	6.749655	9	36736771		86825		*MIR4475*
cg03436967	1.74E-09	1.15E-06	6.749187	7	1.47E+08	Body	-131839	*CNTNAP2*	*MIR548F4*
cg20543183	1.75E-09	1.16E-06	6.747591	6	31648146	1stExon	4	*LY6G5C*	*LY6G5C*
cg20108693	1.76E-09	1.16E-06	6.746951	8	1.44E+08	5'UTR	15	*SLURP1*	*SLURP1*
cg07594209	1.77E-09	1.17E-06	-6.74511	5	17218278	TSS1500;Body;Body	539	*BASP1-AS1;BASP1*	*BASP1*
cg22054328	1.78E-09	1.17E-06	6.744126	4	1.85E+08	Body;Body	103760	*STOX2*	*STOX2*
cg01821113	1.78E-09	1.17E-06	6.743656	2	2.18E+08	Body	130076	*DIRC3*	*DIRC3-AS1*
cg23485639	1.79E-09	1.18E-06	6.742315	6	28045615	TES5000;TSS5000	-2730	*OR1F12;ZNF165*	*ZNF165*
cg00860354	1.79E-09	1.18E-06	6.742297	16	88442765		-6601		*ZNF469*
cg07093639	1.79E-09	1.18E-06	6.742082	7	1.57E+08		51816		*MIR153-2*
cg02280200	1.81E-09	1.18E-06	6.739665	12	286620	3'UTR	-26190	*IQSEC3*	*LOC101929384*
cg04453552	1.82E-09	1.18E-06	6.739527	16	57394387	Body	1693	*CCL22*	*CCL22*
cg26242064	1.82E-09	1.18E-06	6.739448	10	1.12E+08		-62707		*SMNDC1*
cg06942727	1.82E-09	1.18E-06	6.739305	2	60322100		288071		*MIR4432HG*
cg05760947	1.82E-09	1.18E-06	6.738705	1	2.28E+08		-24748		*ZNF678*
cg09815377	1.83E-09	1.19E-06	6.737984	6	30309236	Body;Body;Body;Body;Body;Body;TSS5000;TSS5000;TSS5000	-3696	*TRIM39;TRIM39-RPP21;RPP21*	*RPP21*
cg25692451	1.85E-09	1.20E-06	6.735667	4	78035411		-38269		*CCNI*
cg19642727	1.85E-09	1.20E-06	6.735455	1	853876	Body;Body	1196	*LINC02593*	*LINC02593*
cg16426149	1.85E-09	1.20E-06	6.73525	17	80983536	Body;Body;Body	-18663	*B3GNTL1*	*B3GNTL1*
cg02157454	1.85E-09	1.20E-06	6.734767	6	32036404	Body;Body	-22499	*TNXB*	*TNXB*
cg09255332	1.86E-09	1.20E-06	6.734095	10	1.32E+08	Body	-11672	*EBF3*	*MIR4297*
cg05364747	1.86E-09	1.20E-06	6.733968	1	2.29E+08	Body;Body;Body	82166	*OBSCN*	*MIR6742*
cg14031465	1.87E-09	1.20E-06	6.732857	8	1.44E+08	TSS5000	-1518	*LY6D*	*LY6D*
cg05221654	1.87E-09	1.20E-06	6.732811	16	32487825		-186523		*LOC390705*
cg03729670	1.88E-09	1.20E-06	6.73168	7	600505	Body;Body;Body;Body;Body;Body;TSS1500	-1089	*PRKAR1B;PRKAR1B-AS2*	*PRKAR1B-AS2*
cg18147543	1.88E-09	1.20E-06	6.731277	1	16276294	Body;Body;Body;Body;Body;Body	26333	*ZBTB17*	*ZBTB17*
cg22453911	1.88E-09	1.20E-06	6.731238	19	2354290	3'UTR;3'UTR	25608	*SPPL2B*	*SPPL2B*
cg19038027	1.89E-09	1.21E-06	6.730251	10	1.34E+08		43575		*LOC107984282*
cg24302133	1.90E-09	1.21E-06	6.728873	1	1.82E+08	3'UTR;3'UTR;3'UTR	260674	*CACNA1E*	*ZNF648*
cg24923818	1.91E-09	1.21E-06	6.72852	19	14580273	Body;Body;TES5000	5893	*PKN1;PTGER1*	*PTGER1*
cg05184395	1.91E-09	1.21E-06	6.728216	6	1.69E+08		171904		*LOC105378146*

Probe	p_adj	q_val	t	Chr	Pos.hg19	locations	dist2TSS	gene.Illumina	gene.nearest
cg00775172	1.91E-09	1.21E-06	6.728202	22	37601268	3'UTR;3'UTR	5461	*SSTR3*	*SSTR3*
cg14365123	1.91E-09	1.21E-06	6.727754	1	2938246	5'UTR	216	*ACTRT2*	*ACTRT2*
cg19450046	1.91E-09	1.21E-06	6.727622	21	45712938	Body	-6997	*AIRE*	*PFKL*
cg26016241	1.92E-09	1.21E-06	6.726964	19	17571656	1stExon	79	*NXNL1*	*NXNL1*
cg11374884	1.93E-09	1.22E-06	6.725764	7	1.28E+08	Body;Body;Body;Body;Body;Body;Body;Body;Body;Body	18354	*CCDC136*	*CCDC136*
cg10457735	1.94E-09	1.22E-06	6.724619	21	37437505	TSS5000;TSS5000;TSS5000;TSS5000;TSS5000;TES5000;TSS5000;TSS5000	-4681	*SETD4;LOC100133286;CBR1*	*SETD4*
cg11971461	1.94E-09	1.22E-06	6.724389	4	1.43E+08	Body;Body;Body	65458	*IL15*	*IL15*
cg27278802	1.95E-09	1.23E-06	6.723375	11	66359430	Body;TSS1500	1104	*CCDC87;CCS*	*CCDC87*
cg14151616	1.97E-09	1.23E-06	-6.72175	19	10426492	Body;TES5000;TES5000	199	*FDX2;RAVER1*	*FDX2*
cg15861089	1.98E-09	1.24E-06	6.720512	12	52695561	Body	-10243	*KRT86*	*KRT81*
cg14824301	1.98E-09	1.24E-06	6.720454	11	61515894	TES5000;TSS5000	-4213	*DAGLA;MYRF*	*MYRF*
cg26555126	1.99E-09	1.24E-06	6.719451	6	33998729	Body;Body;Body;Body;Body;Body	-30901	*GRM4*	*MIR1275*
cg01002508	1.99E-09	1.24E-06	6.718811	1	2.46E+08	Body;Body	-529137	*SMYD3*	*KIF26B-AS1*
cg13169491	1.99E-09	1.24E-06	6.718479	7	1.28E+08	Body;Body;Body;Body;Body	-10472	*FAM71F1*	*CALU*
cg08245147	2.03E-09	1.27E-06	6.714036	7	1.51E+08	TES5000;TES5000;TES5000;TES5000;TES5000;TES5000;TES5000	-28622	*NUB1;WDR86*	*WDR86-AS1*
cg20102878	2.04E-09	1.27E-06	6.713598	10	1.19E+08	Body	10113	*SLC18A2*	*SLC18A2*
cg16334849	2.04E-09	1.27E-06	6.713046	8	36636869		-5022		*KCNU1*
cg09878269	2.05E-09	1.27E-06	6.712749	13	1.14E+08	TSS1500;TSS1500	-437	*PROZ*	*PROZ*
cg25793425	2.05E-09	1.27E-06	6.712438	19	6007842	Body;Body;Body	29429	*LOC100128568;RFX2*	*LOC100128568*
cg21006325	2.08E-09	1.28E-06	6.709132	21	44480624	Body;Body;Body;Body;Body	7890	*CBS*	*CBS*
cg15502607	2.09E-09	1.29E-06	6.708479	1	1990986	Body	9137	*PRKCZ*	*PRKCZ*
cg24053265	2.10E-09	1.29E-06	6.707188	22	49498271		235690		*LINC01310*
cg12837296	2.10E-09	1.29E-06	6.707149	17	42426483	Body;TES5000	4030	*GRN;FAM171A2*	*GRN*
cg06286026	2.10E-09	1.29E-06	6.707141	4	3013267	Body;Body;Body;Body;Body	47970	*GRK4*	*GRK4*
cg26166433	2.10E-09	1.29E-06	6.706775	15	78937416	TSS5000;TSS5000	-3864	*CHRNB4*	*CHRNB4*
cg11236641	2.11E-09	1.29E-06	6.70621	1	3348815	Body;Body	-22413	*PRDM16*	*ARHGEF16*
cg24540066	2.12E-09	1.29E-06	6.705324	1	5891521		31280		*MIR4689*
cg15285494	2.12E-09	1.29E-06	6.705187	4	1.88E+08		-166605		*FAT1*
cg16660494	2.12E-09	1.29E-06	6.705066	5	57073212		121777		*LINC02225*
cg20905460	2.12E-09	1.29E-06	6.704775	7	1018498	TSS5000;Body;TSS5000	-3313	*COX19;C7orf50;CYP2W1*	*COX19*
cg19069039	2.13E-09	1.30E-06	6.703742	12	52238665	TSS5000	-3341	*FIGNL2-DT*	*FIGNL2-DT*
cg08050907	2.15E-09	1.31E-06	6.701275	3	1.12E+08	TSS1500;TSS1500;TSS5000	-856	*TMPRSS7*	*TMPRSS7*
cg04311964	2.16E-09	1.31E-06	6.701046	8	1.44E+08	5'UTR	8	*LYPD2*	*LYPD2*
cg16021847	2.17E-09	1.31E-06	6.700112	19	55689649	Body;Body;TES5000;TES5000	1067	*SYT5;PTPRH*	*SYT5*
cg06493289	2.17E-09	1.32E-06	6.699347	11	1.14E+08	1stExon;1stExon;1stExon;1stExon;1stExon	423	*ZBTB16*	*ZBTB16*
cg20401945	2.18E-09	1.32E-06	6.698567	16	29912460	TSS5000;TSS5000;TSS5000;TSS5000;TSS5000;TSS5000;1stExon	652	*SEZ6L2;ASPHD1*	*ASPHD1*
cg04120487	2.18E-09	1.32E-06	6.698389	14	24524822	TES5000;TES5000;TES5000;TES5000;TES5000;TES5000;TES5000;TES5000;Body	3605	*DHRS4L1;CARMIL3*	*CARMIL3*
cg01804500	2.18E-09	1.32E-06	6.698116	1	9324932	3'UTR;3'UTR	25027	*H6PD*	*H6PD*
cg14314458	2.19E-09	1.32E-06	6.697418	4	963830	Body	3527	*DGKQ*	*DGKQ*
cg04041000	2.19E-09	1.32E-06	6.697332	3	46919066	TSS200;TSS5000	-144	*PTH1R*	*PTH1R*
cg06399569	2.20E-09	1.32E-06	6.696208	11	1.13E+08	3'UTR	12504	*ANKK1*	*ANKK1*
cg00228118	2.21E-09	1.32E-06	6.695611	9	1.1E+08		-226572		*KLF4*
cg26070487	2.22E-09	1.32E-06	6.695024	10	47086508	Body;Body	2975	*NPY4R*	*NPY4R*
cg06804411	2.22E-09	1.32E-06	6.694851	1	1.53E+08	TSS1500	-1251	*LELP1*	*LELP1*
cg25513073	2.22E-09	1.32E-06	6.694762	11	2481641	Body;TSS1500	-1042	*KCNQ1*	*KCNQ1*
cg15374027	2.22E-09	1.32E-06	6.694505	17	15415649	Body;Body;Body	-41499	*TVP23C-CDRT4;TVP23C*	*CDRT3*
cg13355078	2.22E-09	1.32E-06	6.694473	6	2636363	TSS5000;Body;Body	-1526	*LINC01600;LINC02521*	*LINC01600*
cg04663194	2.23E-09	1.32E-06	6.693939	11	66315239	TSS5000;TSS5000;Body;Body	866	*ZDHHC24;ACTN3*	*ACTN3*
cg14301000	2.23E-09	1.32E-06	6.693711	9	1.37E+08	1stExon	288	*DBH*	*DBH*
cg12513686	2.23E-09	1.32E-06	6.693698	10	709592	Body;3'UTR	13618	*DIP2C;PRR26*	*PRR26*
cg01809540	2.25E-09	1.33E-06	6.691918	11	77793764	TSS5000;TSS5000;TSS5000;TSS5000;TSS5000;TSS5000	-2856	*NDUFC2-KCTD14;NDUFC2*	*NDUFC2*
cg12598054	2.25E-09	1.33E-06	6.691862	4	7437156	Body;TSS1500	-499	*SORCS2;PSAPL1*	*PSAPL1*
cg01417432	2.25E-09	1.33E-06	6.691514	10	63913731		104616		*ARID5B*
cg17357600	2.25E-09	1.33E-06	6.691371	20	44096016	TSS5000	-2376	*WFDC2*	*WFDC2*
cg07912003	2.25E-09	1.33E-06	6.691269	2	75797838	TSS1500	-993	*EVA1A*	*EVA1A*
cg24286200	2.25E-09	1.33E-06	6.691102	7	1.39E+08	TSS5000;TSS5000;Body;Body;Body;Body;Body	3228	*HIPK2;TBXAS1*	*TBXAS1*
cg03605542	2.26E-09	1.33E-06	6.690521	5	59929339	Body;Body	66654	*DEPDC1B*	*DEPDC1B*
cg16251986	2.26E-09	1.33E-06	6.690123	15	1.02E+08	Body;Body;Body	-5125	*PCSK6*	*PCSK6-AS1*
cg14120827	2.27E-09	1.33E-06	6.689852	9	88973574	TSS5000;TSS5000;TSS5000;TSS5000	-4249	*TUT7*	*TUT7*
cg00189989	2.27E-09	1.33E-06	6.689546	22	19513176	TSS1500;TSS1500;TSS1500;TSS1500	-316	*CLDN5*	*CLDN5*

Probe	p_adj	q_val	t	Chr	Pos.hg19	locations	dist2TSS	gene.Illumina	gene.nearest
cg15654892	2.27E-09	1.33E-06	6.68929	8	60834013		359946		*CA8*
cg23978866	2.28E-09	1.33E-06	6.688568	2	47230407	Body;Body;Body;Body	-61413	*TTC7A*	*MCFD2*
cg13662644	2.29E-09	1.33E-06	6.687751	19	7677237	Body;Body	-5087	*CAMSAP3*	*MIR6792*
cg15516226	2.30E-09	1.34E-06	6.686707	5	1.8E+08	Body;Body;TES5000	254	*BTNL9;MIR8089*	*BTNL9*
cg20988960	2.30E-09	1.34E-06	6.686511	1	3191815	Body;Body	147277	*PRDM16*	*MIR4251*
cg03859929	2.31E-09	1.34E-06	6.685458	11	67291247	TSS1500;TSS1500	-330	*CABP2*	*CABP2*
cg22250586	2.33E-09	1.35E-06	6.683558	13	33704014	Body;Body;Body;Body;Body;Body	56214	*STARD13*	*STARD13*
cg01064758	2.36E-09	1.36E-06	6.681315	8	1.34E+08	Body;TSS1500;TSS1500;TSS1500;TSS1500	-807	*TG;SLA*	*SLA*
cg02400611	2.36E-09	1.37E-06	6.680792	3	1.95E+08		-32042		*XXYLT1-AS1*
cg06714360	2.36E-09	1.37E-06	6.680606	10	1.34E+08	Body	-22446	*INPP5A*	*INPP5A*
cg00808579	2.37E-09	1.37E-06	6.680214	1	40768026	Body	14932	*COL9A2*	*COL9A2*
cg18465319	2.37E-09	1.37E-06	6.679547	3	1.16E+08	Body;Body	39227	*LSAMP*	*LSAMP-AS1*
cg08115618	2.38E-09	1.37E-06	6.679448	8	1.35E+08		28754		*LOC105375773*
cg01904151	2.38E-09	1.37E-06	6.679159	3	1.56E+08		52248		*TIPARP*
cg23650353	2.38E-09	1.37E-06	6.678523	4	1.49E+08	3'UTR	289898	*ARHGAP10*	*MIR4799*
cg05264881	2.39E-09	1.37E-06	6.678267	1	15375148	Body;Body;Body;Body;Body;Body;Body;Body	102734	*KAZN*	*KAZN*
cg15318957	2.41E-09	1.38E-06	6.676489	6	32044104	Body;Body	-30199	*TNXB*	*TNXB*
cg26431815	2.41E-09	1.38E-06	6.676084	8	668053	Body;Body;Body	13171	*ERICH1*	*ERICH1*
cg01580176	2.42E-09	1.38E-06	6.675685	14	24839226	Body;Body;Body;Body;Body;Body;Body;Body;1stExon	939	*NFATC4*	*NFATC4*
cg11158902	2.42E-09	1.38E-06	6.6756	4	6536914	Body;Body	20428	*PPP2R2C*	*PPP2R2C*
cg11757945	2.42E-09	1.38E-06	6.675392	1	54602356	Body;TES5000	16498	*CDCP2*	*CDCP2*
cg11645098	2.43E-09	1.38E-06	6.674296	17	43198124	Body	-6323	*PLCD3*	*MIR6784*
cg27129144	2.46E-09	1.39E-06	6.671829	17	78848533	Body;Body	-69101	*RPTOR*	*LOC101928855*
cg04688645	2.46E-09	1.39E-06	6.671807	3	99772843	Body;Body;Body	60514	*CMSS1;FILIP1L*	*FILIP1L*
cg10754341	2.46E-09	1.39E-06	6.671807	2	1.14E+08		-32585		*IL37*
cg02628907	2.47E-09	1.40E-06	6.670878	10	43562544		-9930		*RET*
cg26963259	2.47E-09	1.40E-06	6.670659	11	1299151	Body;Body;Body;Body;Body	21317	*TOLLIP*	*MIR6744*
cg16098955	2.48E-09	1.40E-06	6.669857	2	55172992	Body	64416	*EML6*	*RTN4*
cg12084925	2.49E-09	1.40E-06	6.669281	6	33130696	3'UTR;3'UTR;3'UTR	19596	*COL11A2*	*HCG24*
cg17155330	2.49E-09	1.40E-06	6.668923	13	1.11E+08	3'UTR;3'UTR;Body;Body;3'UTR;3'UTR;TES5000;TES5000;TES5000;TES5000	22978	*NAXD;CARS2*	*NAXD*
cg23884924	2.49E-09	1.40E-06	6.668594	19	18882253	Body;Body	19861	*CRTC1*	*COMP*
cg25544935	2.51E-09	1.41E-06	6.666799	6	1.69E+08	Body;Body	184288	*SMOC2*	*SMOC2*
cg25377744	2.52E-09	1.41E-06	6.666212	13	47063673		-22762		*LINC01198*
cg05605194	2.52E-09	1.41E-06	6.666158	2	7739870		178479		*LOC100506274*
cg18331061	2.53E-09	1.41E-06	6.665222	13	51818035	Body;Body;Body	21502	*FAM124A*	*FAM124A*
cg07429804	2.53E-09	1.41E-06	6.665205	17	39666961	TES5000	-5097	*KRT15*	*KRT13*
cg20383521	2.53E-09	1.41E-06	6.665119	1	1.52E+08	3'UTR;3'UTR;3'UTR	-29701	*TUFT1*	*SNX27*
cg18192987	2.54E-09	1.42E-06	6.66415	2	1.45E+08	Body;Body	-22706	*ZEB2*	*ZEB2-AS1*
cg27471755	2.55E-09	1.42E-06	6.66374	19	35842445	TES5000;TES5000;TES5000;TES5000;TES5000;5'UTR	1	*CD22;FFAR1*	*FFAR1*
cg22259242	2.55E-09	1.42E-06	6.663629	11	2021243	TES5000;TSS5000;TSS5000;Body;TSS5000	1453	*HOTS;H19;MIR675*	*H19*
cg03094083	2.55E-09	1.42E-06	6.663194	17	26864469	3'UTR;3'UTR	13511	*FOXN1*	*FOXN1*
cg04293770	2.55E-09	1.42E-06	6.663193	5	1.48E+08		111436		*MIR584*
cg22981153	2.56E-09	1.42E-06	6.66256	8	1.42E+08	5'UTR;5'UTR	278	*PTP4A3*	*PTP4A3*
cg11479380	2.57E-09	1.42E-06	6.661417	7	1.16E+08	TSS1500;Body;Body	-1316	*TFEC*	*TFEC*
cg24245305	2.58E-09	1.42E-06	6.661252	7	43351320	Body;Body	160827	*HECW1*	*MIR3943*
cg10547843	2.61E-09	1.44E-06	6.658655	7	99601692	Body;Body	6380	*LOC105375423*	*LOC105375423*
cg04930829	2.62E-09	1.44E-06	6.657351	6	41409982		-60199		*LINC01276*
cg15622527	2.63E-09	1.44E-06	6.656969	10	1749494	Body	30225	*ADARB2*	*ADARB2*
cg03214715	2.63E-09	1.44E-06	6.656747	12	57873176	TSS5000;TSS5000;TSS5000;TSS5000;TSS5000;TSS5000;TSS5000;TSS5000;1stExon;1stExon;1stExon	214	*ARHGAP9*	*ARHGAP9*
cg23520574	2.63E-09	1.44E-06	6.656614	11	4928572	TSS200	-27	*OR51A7*	*OR51A7*
cg06859833	2.63E-09	1.44E-06	6.656321	3	1.67E+08		-55811		*LOC105374194*
cg15305112	2.64E-09	1.44E-06	6.656162	2	2.36E+08	Body;Body;Body;Body;Body	-7716	*SH3BP4*	*SH3BP4*
cg23870378	2.64E-09	1.44E-06	6.655659	14	1.01E+08	TSS1500;TSS1500;TSS1500;TSS1500;TSS1500;TSS1500;TSS1500;TSS1500;TSS1500;TSS1500;TSS1500;TSS1500;TSS1500;TSS1500;TSS1500	-1376	*MEG3*	*MEG3*
cg11398309	2.64E-09	1.44E-06	6.655466	2	1.02E+08		-87922		*MAP4K4*
cg18048370	2.65E-09	1.44E-06	6.655217	10	1.35E+08	Body	30987	*CFAP46*	*CFAP46*
cg16366373	2.65E-09	1.44E-06	6.655091	1	35501245	TSS5000	-3699	*ZMYM6*	*ZMYM6*
cg25477456	2.66E-09	1.45E-06	6.654193	18	32552858	TSS5000	-4033	*MAPRE2*	*MAPRE2*
cg13690873	2.66E-09	1.45E-06	6.653657	1	36587439	Body;Body	3384	*COL8A2*	*COL8A2*
cg27143703	2.68E-09	1.45E-06	6.652721	17	77961719	Body	-37067	*TBC1D16*	*TBC1D16*

Probe	p_adj	q_val	t	Chr	Pos.hg19	locations	dist2TSS	gene.Illumina	gene.nearest
cg12640930	2.69E-09	1.46E-06	6.65147	1	1.75E+08	Body	14204	*TNN*	*TNN*
cg01323840	2.70E-09	1.46E-06	6.651082	16	12901108	TSS5000;TSS5000	-3364	*CPPED1*	*CPPED1*
cg23458186	2.71E-09	1.47E-06	6.649713	19	3935938	Body;Body;Body	2838	*NMRK2*	*NMRK2*
cg08498406	2.72E-09	1.47E-06	6.649272	8	27306846	Body;Body;Body;Body	15960	*PTK2B*	*MIR6842*
cg15995714	2.73E-09	1.48E-06	6.647982	10	1.34E+08	Body;Body	11742	*PWWP2B*	*PWWP2B*
cg24195796	2.75E-09	1.48E-06	6.646589	15	29362669	Body;Body;Body;Body;Body;Body;Body;Body;Body;Body;TSS200;TSS200	-191	*APBA2*	*APBA2*
cg23244540	2.76E-09	1.49E-06	6.645802	2	1.28E+08		118655		*GYPC*
cg21918848	2.77E-09	1.49E-06	6.645387	17	3776939	Body;Body;Body	17098	*CAMKK1*	*CAMKK1*
cg05855039	2.78E-09	1.49E-06	-6.65419	14	85996751	Body;Body;Body;5'UTR;5'UTR;TSS1500;TSS5000;TSS5000	264	*FLRT2*	*FLRT2*
cg12970662	2.83E-09	1.52E-06	6.640372	3	46873759	Body;Body	1554	*PRSS42P*	*PRSS42P*
cg05338384	2.83E-09	1.52E-06	6.640048	7	1.57E+08		-10523		*NOM1*
cg08788246	2.83E-09	1.52E-06	6.639884	22	20758901	Body;Body;Body;Body;Body	10461	*ZNF74*	*ZNF74*
cg26461880	2.84E-09	1.52E-06	6.63961	2	2.43E+08	Body	-13320	*LINC01237*	*LOC285097*
cg00804179	2.86E-09	1.53E-06	6.637722	3	13083974	Body	30955	*IQSEC1*	*IQSEC1*
cg25565264	2.87E-09	1.53E-06	6.637299	5	1.57E+08	Body	3147	*LSM11*	*LSM11*
cg07746960	2.88E-09	1.53E-06	6.636571	19	42546662	Body;Body	23295	*GRIK5*	*GRIK5*
cg11356247	2.88E-09	1.54E-06	6.635963	7	1.58E+08	Body;Body;Body;Body;Body	97071	*PTPRN2*	*LOC100506585*
cg10702755	2.90E-09	1.54E-06	6.634429	8	528084		-32303		*TDRP*
cg03065614	2.91E-09	1.55E-06	6.633775	6	44096542	TSS1500;TSS5000;TSS5000;TSS1500;TSS1500;TSS1500;Body;Body	1252	*MRPL14;TMEM63B*	*TMEM63B*
cg27213024	2.92E-09	1.55E-06	6.632808	6	1.44E+08		-15989		*PHACTR2*
cg05168655	2.93E-09	1.55E-06	6.632621	6	1.67E+08	Body;Body;Body;Body	-15320	*RPS6KA2*	*RPS6KA2*
cg05375811	2.93E-09	1.55E-06	6.632181	12	1.32E+08	Body;Body	-27500	*ADGRD1*	*ADGRD1-AS1*
cg25884442	2.93E-09	1.55E-06	6.63211	12	6178606	Body	-29257	*VWF*	*SNORA120*
cg25931491	2.94E-09	1.55E-06	6.631944	11	1422468	Body;Body;Body;Body	-9231	*BRSK2*	*BRSK2*
cg14018797	2.94E-09	1.55E-06	6.631656	7	1849782		34107		*MIR4655*
cg26896748	2.94E-09	1.55E-06	6.631641	11	2861064	Body;Body;TES5000	21735	*KCNQ1;KCNQ1-AS1*	*KCNQ1-AS1*
cg16694664	2.95E-09	1.55E-06	6.630801	7	1.29E+08	TSS5000;TES5000;TES5000	292	*MIR182;MIR96;MIR183*	*MIR96*
cg01173944	2.95E-09	1.55E-06	6.630787	14	67986361	TSS5000;Body;TSS5000;TSS5000;TSS5000;Body	-4159	*TMEM229B*	*TMEM229B*
cg05345160	2.97E-09	1.56E-06	6.629554	6	1.45E+08		-57374		*UTRN*
cg27276918	2.97E-09	1.56E-06	6.629415	8	1.45E+08	TES5000;TES5000;TES5000;TES5000;TES5000;TES5000;TES5000;TES5000	29426	*PLEC*	*PLEC*
cg14473516	2.98E-09	1.56E-06	6.628977	2	309026		-7257		*LINC01865*
cg16782184	2.98E-09	1.56E-06	6.628472	14	24555085	Body;Body;Body;Body	-8434	*NRL*	*PCK2*
cg26673975	2.99E-09	1.56E-06	6.627685	16	66420974	Body	20381	*CDH5*	*CDH5*
cg18109186	3.00E-09	1.56E-06	6.627369	3	1.27E+08	Body	23595	*PLXNA1*	*PLXNA1*
cg10632215	3.02E-09	1.57E-06	6.625881	22	47097566	Body	36583	*CERK*	*CERK*
cg26453360	3.03E-09	1.58E-06	6.625034	11	46741373	Body;Body	611	*F2*	*F2*
cg01975706	3.07E-09	1.60E-06	6.621572	21	27015734	Body;Body;Body;Body	4141	*JAM2*	*JAM2*
cg17331904	3.08E-09	1.60E-06	6.621273	7	23749977	Body;Body;5'UTR;5'UTR;Body	140	*STK31*	*STK31*
cg19275832	3.08E-09	1.60E-06	6.620873	1	1.18E+08		117496		*GDAP2*
cg11793646	3.09E-09	1.60E-06	6.620497	21	44087129	Body;Body;Body;Body;Body;Body;Body;Body;Body;Body;Body;Body;Body;Body;Body;Body;Body;Body;Body;Body;Body	13268	*PDE9A*	*PDE9A*
cg11569478	3.09E-09	1.60E-06	6.62036	4	1.2E+08	Body;Body;Body;Body;Body	100692	*SYNPO2*	*SYNPO2*
cg19251719	3.10E-09	1.60E-06	6.619704	1	2.03E+08	TSS1500;Body	-339	*MYOG;MYOPARR*	*MYOG*
cg04654153	3.12E-09	1.61E-06	-6.61837	2	1.77E+08	TES5000;TSS5000	-1864	*HOXD13;HOXD12*	*HOXD12*
cg16875711	3.13E-09	1.61E-06	6.617552	18	51798822	Body;Body;Body;Body;Body;Body;Body;Body;Body;Body;Body;Body;Body;Body;Body	2879	*POLI*	*POLI*
cg04213213	3.14E-09	1.62E-06	6.617029	3	16831943		-94504		*PLCL2*
cg27403117	3.14E-09	1.62E-06	6.616703	16	70698053	TES5000;TES5000;TES5000;Body	21903	*IL34;MTSS2*	*MTSS2*
cg05238976	3.14E-09	1.62E-06	6.616561	10	44140134	Body;Body;Body;Body;Body;Body;Body;Body;Body;TSS1500	815	*ZNF32-AS3;ZNF32;ZNF32-AS1;ZNF32-AS2*	*ZNF32-AS1*
cg16420340	3.15E-09	1.62E-06	6.616442	12	31083391	Body;Body;Body	3554	*TSPAN11*	*TSPAN11*
cg01278797	3.17E-09	1.62E-06	6.615091	16	2339405	Body	14785	*ABCA3*	*MIR4717*
cg15922246	3.17E-09	1.62E-06	6.614854	7	1.51E+08	TSS1500;TSS1500;TSS1500;Body;Body;TSS5000;TSS5000;TSS5000;TSS5000;TSS5000;TSS5000;TSS5000	-833	*FASTK;TMUB1;AGAP3*	*FASTK*
cg06107104	3.18E-09	1.63E-06	6.61395	17	73617762	Body	-11751	*MYO15B*	*SMIM5*
cg06639691	3.19E-09	1.63E-06	6.613012	10	1.19E+08	TSS200	-180	*KCNK18*	*KCNK18*
cg21162779	3.20E-09	1.63E-06	6.612889	1	2564998	TSS1500	-543	*MMEL1*	*MMEL1*
cg13271206	3.20E-09	1.63E-06	6.612623	11	75109830	TSS1500;TSS1500;TSS1500;TSS1500;TSS5000	-704	*RPS3;SNORD15A*	*RPS3*
cg07050194	3.23E-09	1.64E-06	6.610769	14	34233812	Body;Body;Body;Body	55670	*NPAS3*	*SNORA89*
cg18207011	3.23E-09	1.64E-06	6.610503	14	68861095	Body;Body;Body;Body;Body;Body;Body;Body;Body;Body;Body;Body	234409	*RAD51B*	*LOC100996664*
cg03516551	3.26E-09	1.66E-06	6.608591	7	2764919	Body;TES5000;TES5000;TES5000;TES5000	37084	*AMZ1;GNA12*	*AMZ1*
cg10943877	3.27E-09	1.66E-06	6.608054	5	1.45E+08	Body	11206	*GRXCR2*	*GRXCR2*
cg13143743	3.27E-09	1.66E-06	6.607908	3	1.78E+08	Body	36042	*LINC02015*	*LINC02015*

Probe	p_adj	q_val	t	Chr	Pos.hg19	locations	dist2TSS	gene.Illumina	gene.nearest
cg13862169	3.27E-09	1.66E-06	6.60767	1	2.01E+08	TSS5000	-3469	*TMEM9*	*TMEM9*
cg26671873	3.28E-09	1.66E-06	6.607013	4	1.88E+08		-18613		*LINC02374*
cg05323683	3.32E-09	1.68E-06	6.604384	8	95168291	Body;Body	52492	*CDH17*	*CDH17*
cg06739525	3.32E-09	1.68E-06	6.604147	2	45417905	Body	64175	*LINC01121*	*LINC01121*
cg00411697	3.33E-09	1.68E-06	6.603809	11	1.17E+08	TSS5000;TSS5000;TSS1500;TSS200;TSS200	-185	*ZPR1;APOA5*	*APOA5*
cg17524821	3.33E-09	1.68E-06	6.603606	19	15535460	Body;Body;Body;Body	-5661	*WIZ*	*AKAP8L*
cg16926712	3.35E-09	1.69E-06	6.602237	8	28657193	Body;Body;Body;Body;Body	90239	*INTS9*	*INTS9*
cg23296684	3.36E-09	1.69E-06	6.602	10	93108887	Body	-60104	*HECTD2-AS1*	*HECTD2*
cg14422903	3.37E-09	1.69E-06	6.601089	9	36742062		81534		*MIR4475*
cg11301967	3.38E-09	1.70E-06	6.600256	5	1.33E+08	Body;TSS5000	3133	*FSTL4;MIR1289-2*	*MIR1289-2*
cg00040322	3.39E-09	1.70E-06	6.599911	2	11240641	Body	31661	*FLJ33534*	*FLJ33534*
cg04682087	3.40E-09	1.70E-06	6.598799	2	875946		-11834		*LINC01115*
cg26797894	3.42E-09	1.71E-06	6.597673	2	2.11E+08		-7341		*KANSL1L*
cg00901675	3.43E-09	1.71E-06	6.59713	13	30728236	TSS1500	-1025	*LINC00384*	*LINC00384*
cg18778299	3.45E-09	1.72E-06	6.59605	3	1.34E+08	TSS1500;TSS5000	-1163	*C3orf36;SLCO2A1*	*C3orf36*
cg03930004	3.46E-09	1.72E-06	6.594929	7	1.51E+08	TSS1500;TSS1500;TSS1500;Body;Body;TSS5000;TSS5000;TSS5000;TSS5000;TSS5000;TSS5000;TSS5000	-821	*FASTK;TMUB1;AGAP3*	*FASTK*
cg19669199	3.46E-09	1.72E-06	6.594778	19	7584241	1stExon;TSS5000	3253	*ZNF358;MCOLN1*	*ZNF358*
cg13859183	3.47E-09	1.72E-06	6.594626	13	67196386	Body;Body;Body;Body	-202914	*PCDH9*	*PCDH9-AS2*
cg13194638	3.48E-09	1.73E-06	6.593875	17	37725898		38275		*NEUROD2*
cg04533623	3.48E-09	1.73E-06	6.593541	9	1.4E+08	TSS5000;TSS5000	-4250	*AGPAT2*	*AGPAT2*
cg21232935	3.49E-09	1.73E-06	6.593286	7	51113700	Body;Body;Body;Body;Body	-252550	*COBL*	*GRB10*
cg04574834	3.49E-09	1.73E-06	6.592909	10	99891730	TSS5000;TSS5000;TSS5000;TSS5000;TSS5000;TSS5000;TSS5000;TSS5000;TSS5000;TSS5000;TSS5000;TSS5000;TSS5000	-2650	*R3HCC1L*	*R3HCC1L*
cg20862496	3.52E-09	1.74E-06	6.591404	8	1.44E+08	Body	90	*SLURP1*	*SLURP1*
cg24478926	3.52E-09	1.74E-06	6.591028	11	69237550	TSS5000	-2872	*LOC102724265*	*LOC102724265*
cg06294856	3.52E-09	1.74E-06	6.591019	9	18776340	Body	203037	*ADAMTSL1*	*MIR3152*
cg14269525	3.53E-09	1.74E-06	6.590493	14	98214031	TSS5000	15137	*LINC02312*	*LINC02312*
cg03956353	3.53E-09	1.74E-06	6.590354	1	1.1E+08	TSS200;TSS200;TSS200;TSS200	-97	*EPS8L3*	*EPS8L3*
cg22849635	3.55E-09	1.74E-06	6.589542	17	7910804	Body	4872	*GUCY2D*	*GUCY2D*
cg06521145	3.55E-09	1.74E-06	6.589129	19	2048650	Body;Body	2593	*MKNK2*	*MKNK2*
cg19007167	3.56E-09	1.74E-06	6.588737	2	2.43E+08	Body;Body;Body;Body;Body	2860	*NEU4*	*NEU4*
cg26736154	3.57E-09	1.74E-06	6.588121	15	37020592	Body;Body;Body;Body;Body;Body;Body;Body;Body;Body	90115	*C15orf41*	*CSNK1A1P1*
cg22433647	3.57E-09	1.74E-06	6.588048	1	18021329	Body;Body;Body;Body;Body;Body;Body	-60498	*ARHGEF10L*	*ACTL8*
cg20747012	3.57E-09	1.74E-06	6.587926	7	1.15E+08	Body;Body	11536	*MDFIC*	*MDFIC*
cg03419232	3.57E-09	1.74E-06	6.587926	6	1.58E+08	Body;Body	54570	*SYNJ2*	*SYNJ2*
cg12235469	3.61E-09	1.76E-06	6.585559	11	12746796	Body	50829	*TEAD1*	*TEAD1*
cg24375627	3.62E-09	1.76E-06	6.584823	1	1.54E+08	TSS1500;TSS5000	-817	*S100A6;S100A5*	*S100A6*
cg23892392	3.63E-09	1.77E-06	6.584432	4	1172078	Body	-5079	*SPON2*	*SPON2*
cg21539981	3.65E-09	1.77E-06	6.5832	8	58106094		-24740		*LINC01606*
cg14433037	3.65E-09	1.77E-06	6.583059	7	824325	Body;Body	-30868	*DNAAF5*	*SUN1*
cg27649253	3.65E-09	1.77E-06	6.582914	19	5245891	Body;Body;Body;Body	94932	*PTPRS*	*PTPRS*
cg22388471	3.65E-09	1.77E-06	6.582844	6	52363701	TSS5000;TSS5000;TSS5000;3'UTR	77896	*EFHC1;TRAM2*	*EFHC1*
cg25616787	3.66E-09	1.77E-06	6.582396	1	9409349	Body	56397	*SPSB1*	*SPSB1*
cg18256432	3.69E-09	1.78E-06	6.580851	14	74823368	Body	8195	*VRTN*	*VRTN*
cg22511877	3.72E-09	1.80E-06	6.57867	2	1942942	Body;Body;Body;Body;Body;Body;Body;Body;Body;Body	-194655	*MYT1L*	*PXDN*
cg05404847	3.74E-09	1.80E-06	6.57779	19	42931316	Body;5'UTR;Body;TSS5000;TSS5000	224	*LIPE-AS1;LIPE;CXCL17*	*LIPE*
cg16510460	3.74E-09	1.80E-06	6.577502	3	1.84E+08	Body	4942	*LOC107986163*	*LOC107986163*
cg09075327	3.75E-09	1.80E-06	6.577273	8	18708915	Body;Body	35652	*PSD3*	*PSD3*
cg00866863	3.75E-09	1.80E-06	6.576821	14	94453019		-10103		*ASB2*
cg16999602	3.76E-09	1.80E-06	6.576646	1	53608163	5'UTR;5'UTR;5'UTR;Body;5'UTR	147	*SLC1A7*	*SLC1A7*
cg01779679	3.77E-09	1.81E-06	6.575866	16	87857222		11044		*MIR6775*
cg24474279	3.78E-09	1.81E-06	6.575242	1	1.74E+08	3'UTR;Body	-6899	*ANKRD45*	*SLC9C2*
cg02178151	3.81E-09	1.82E-06	6.573631	14	25423086	Body;Body;Body;Body;Body;Body;Body	95120	*STXBP6*	*STXBP6*
cg18572214	3.82E-09	1.83E-06	6.572708	14	94423192	1stExon;Body	201	*ASB2*	*ASB2*
cg12494049	3.83E-09	1.83E-06	6.572399	10	44702818		-85379		*C10orf142*
cg14793844	3.83E-09	1.83E-06	6.572385	2	1.01E+08	Body;Body	205165	*AFF3*	*AFF3*
cg08895618	3.85E-09	1.84E-06	6.571092	5	1.5E+08	Body;TSS1500	-684	*NDST1*	*NDST1*
cg21896720	3.88E-09	1.85E-06	6.569189	4	4673378	Body	7326	*STX18-AS1*	*SNORD162*
cg23108559	3.91E-09	1.86E-06	6.567473	14	55491009	Body;Body;TSS5000;TSS5000	2808	*WDHD1;SOCS4*	*WDHD1*
cg14516350	3.91E-09	1.86E-06	6.567429	6	30166781	1stExon;1stExon	14402	*TRIM26*	*TRIM26*

Probe	p_adj	q_val	t	Chr	Pos.hg19	locations	dist2TSS	gene.Illumina	gene.nearest
cg12000333	3.92E-09	1.86E-06	6.567311	17	14891737		-42554		*CDRT7*
cg08644365	3.93E-09	1.87E-06	6.566331	1	2.03E+08	Body;Body;Body;Body	23341	*ADORA1*	*ADORA1*
cg07469546	3.96E-09	1.88E-06	6.564666	16	86927990		-163399		*LINC02181*
cg17673769	3.98E-09	1.88E-06	6.563639	2	46707773	Body	1070	*TMEM247*	*TMEM247*
cg02026049	3.98E-09	1.88E-06	6.563539	17	79049229	Body;Body;Body;Body	40279	*BAIAP2*	*BAIAP2*
cg10089457	4.03E-09	1.90E-06	6.560614	17	73499284	TES5000;TES5000;TES5000;TES5000;TES5000;TES5000;Body;Body	4656	*TMEM94;MIR6785;CASKIN2*	*MIR6785*
cg22259126	4.04E-09	1.90E-06	6.560097	6	32020750	Body;Body	-6845	*TNXB*	*TNXB*
cg19747745	4.05E-09	1.90E-06	6.55984	21	44817305		29706		*SIK1*
cg00246969	4.05E-09	1.90E-06	6.559763	13	99159656	Body;Body;Body	14723	*STK24*	*STK24*
cg10097045	4.06E-09	1.90E-06	6.559383	1	55151051	Body;Body;Body;Body;Body;Body;Body	-30443	*MROH7;MROH7-TTC4*	*TTC4*
cg08392325	4.06E-09	1.90E-06	6.559307	2	1.22E+08	Body	14688	*GLI2*	*GLI2*
cg21838952	4.06E-09	1.90E-06	6.559138	15	64197566	TES5000	-22906	*DAPK2*	*LOC101928988*
cg00613113	4.06E-09	1.90E-06	6.558985	10	80720100	Body;Body;Body	107105	*ZMIZ1-AS1*	*ZMIZ1-AS1*
cg25064458	4.06E-09	1.90E-06	6.558963	1	64086533	Body;Body;TSS5000	-2353	*PGM1*	*PGM1*
cg19705386	4.07E-09	1.90E-06	6.558811	2	1.76E+08	TES5000;TES5000;TES5000;TES5000;TES5000;TES5000	-31391	*CHN1*	*CHRNA1*
cg25203481	4.09E-09	1.91E-06	-6.55759	11	2891082	TSS200	-180	*KCNQ1DN*	*KCNQ1DN*
cg08223760	4.11E-09	1.91E-06	6.556551	1	43291209	Body;TSS200	-39	*ERMAP*	*ERMAP*
cg05008843	4.11E-09	1.91E-06	6.556371	14	52622696		-86919		*NID2*
cg07521668	4.12E-09	1.91E-06	6.556099	11	63799381	Body	46057	*MACROD1*	*OTUB1*
cg12679749	4.12E-09	1.91E-06	6.555967	4	81101251		-5172		*PRDM8*
cg09145900	4.12E-09	1.91E-06	6.555922	6	1.68E+08	TSS1500	-1494	*HGC6.3*	*HGC6.3*
cg00355799	4.13E-09	1.92E-06	6.555541	1	2.44E+08	Body	28509	*LINC02774*	*LINC02774*
cg25412641	4.14E-09	1.92E-06	6.55499	6	1.58E+08	Body;Body	-73043	*ZDHHC14*	*MIR3692*
cg13675957	4.16E-09	1.93E-06	6.553432	17	39672463	Body;TES5000	1006	*KRT15;MIR6510*	*MIR6510*
cg06556929	4.19E-09	1.94E-06	6.552284	8	1.42E+08	Body	9217	*GPR20*	*GPR20*
cg09689177	4.20E-09	1.94E-06	6.551768	5	55959580		-57497		*C5orf67*
cg23175130	4.20E-09	1.94E-06	6.551573	10	1.21E+08	Body	29025	*GRK5*	*GRK5*
cg01566831	4.21E-09	1.94E-06	6.550782	17	80320602	3'UTR	3480	*TEX19*	*TEX19*
cg08374552	4.22E-09	1.95E-06	6.55051	5	1.11E+08	Body	85579	*STARD4-AS1*	*NREP*
cg03193299	4.23E-09	1.95E-06	6.549702	4	1167239	TSS1500;TSS1500;Body	-240	*SPON2*	*SPON2*
cg19786808	4.24E-09	1.95E-06	6.549355	17	71431877	Body	-77944	*SDK2*	*LOC101928251*
cg24853340	4.26E-09	1.95E-06	6.548496	13	1.14E+08	TES5000;TES5000	76658	*ATP11A*	*MCF2L-AS1*
cg02575193	4.26E-09	1.95E-06	6.548369	11	6642908	TSS5000;3'UTR	-2255	*TPP1;DCHS1*	*TPP1*
cg24227837	4.26E-09	1.95E-06	6.548283	13	26620952	Body	4246	*SHISA2*	*SHISA2*
cg26219674	4.26E-09	1.95E-06	6.548135	1	1.55E+08	Body;Body;Body;Body;Body	-80140	*KCNN3*	*ADAR*
cg21139713	4.28E-09	1.96E-06	6.547488	1	27675587	Body;Body	7075	*SYTL1*	*SYTL1*
cg22811478	4.28E-09	1.96E-06	6.547147	7	1.12E+08	Body;Body	57063	*DOCK4*	*DOCK4-AS1*
cg24317122	4.28E-09	1.96E-06	6.547036	11	1705432	Body	1427	*FAM99B*	*FAM99B*
cg14418963	4.31E-09	1.96E-06	6.545941	10	1.31E+08	Body	185258	*MGMT*	*MGMT*
cg13889750	4.33E-09	1.97E-06	6.544849	10	1.02E+08		-35263		*CPN1*
cg07133741	4.33E-09	1.97E-06	6.544596	20	55101981	TSS1500;TES5000	-1000	*GCNT7;FAM209A*	*GCNT7*
cg09761846	4.35E-09	1.98E-06	6.543663	11	57117162	Body	11337	*P2RX3*	*P2RX3*
cg08128925	4.35E-09	1.98E-06	6.543553	19	11141458	Body;Body;Body;Body;Body;Body;Body	46631	*SMARCA4*	*SMARCA4*
cg03055021	4.37E-09	1.98E-06	6.542673	1	15664770	Body;Body;Body	5609	*FHAD1;FHAD1-AS1*	*FHAD1-AS1*
cg14788994	4.37E-09	1.98E-06	6.542581	17	8274570	Body;Body	288	*KRBA2*	*KRBA2*
cg08106792	4.37E-09	1.98E-06	6.542512	1	1.54E+08	TSS1500;TES5000	-856	*S100A6;S100A5*	*S100A6*
cg03376089	4.41E-09	2.00E-06	6.540496	17	17839409	Body;Body;Body;Body;Body;Body;Body;Body;Body	36305	*TOM1L2*	*TOM1L2*
cg21748138	4.41E-09	2.00E-06	6.540396	2	1.74E+08		31952		*CDCA7*
cg09231295	4.42E-09	2.00E-06	6.539953	1	58549612	Body;Body	166563	*DAB1*	*DAB1*
cg25940827	4.43E-09	2.00E-06	6.539511	1	22182100	Body;Body	43343	*HSPG2*	*LDLRAD2*
cg01765882	4.43E-09	2.00E-06	6.539354	12	1.32E+08	Body	44479	*LINC01257*	*LINC01257*
cg17793286	4.44E-09	2.00E-06	6.538904	17	18011879	TES5000;TES5000;Body;TSS200	-190	*DRG2;LOC105371566;MYO15A*	*MYO15A*
cg21896394	4.46E-09	2.01E-06	6.538053	8	1.29E+08	Body	38079	*PVT1*	*MIR1207*
cg10835872	4.46E-09	2.01E-06	6.537951	19	13920075	Body;Body	13819	*ZSWIM4*	*ZSWIM4*
cg20151452	4.52E-09	2.03E-06	6.534945	11	86290281	Body;Body;Body;Body;Body;Body;Body;Body	92959	*ME3*	*ME3*
cg24539060	4.52E-09	2.03E-06	6.534898	17	76570762	Body	2716	*DNAH17*	*DNAH17*
cg26982892	4.52E-09	2.03E-06	6.534832	7	1.12E+08		29046		*LSMEM1*
cg27087650	4.55E-09	2.04E-06	6.533377	19	45255796	Body	3832	*BCL3*	*BCL3*
cg01509809	4.55E-09	2.04E-06	6.533265	17	47284233	Body;Body;Body;Body;TSS5000;TSS5000	2510	*GNGT2;ABI3*	*GNGT2*
cg00317577	4.56E-09	2.04E-06	6.533054	15	83239908	Body;Body;Body;Body;Body;Body;Body;Body;Body;Body;Body;Body;Body;Body;Body;Body;Body	608	*CPEB1*	*CPEB1*

Probe	p_adj	q_val	t	Chr	Pos.hg19	locations	dist2TSS	gene.Illumina	gene.nearest
cg17216266	4.56E-09	2.04E-06	6.5328	1	2.19E+08	Body	85266	*LYPLAL1-DT*	*LYPLAL1-DT*
cg24134767	4.57E-09	2.04E-06	6.532277	11	1.14E+08	TSS200;TSS200;TSS200;TSS5000	-158	*HTR3A*	*HTR3A*
cg24171294	4.58E-09	2.04E-06	6.532081	10	6205688	Body;Body;Body;Body	-11463	*PFKFB3*	*MIR3155B*
cg06960356	4.58E-09	2.04E-06	6.532034	11	2596416	Body;Body	113733	*KCNQ1*	*KCNQ1*
cg14413862	4.60E-09	2.05E-06	6.530909	1	2.48E+08	5'UTR;5'UTR;5'UTR;5'UTR;5'UTR;5'UTR	716	*NLRP3*	*NLRP3*
cg05889222	4.62E-09	2.05E-06	6.529932	17	80617384	Body	8799	*RAB40B*	*MIR4525*
cg16309319	4.63E-09	2.06E-06	6.529469	11	6191393	Body	163	*OR52B2*	*OR52B2*
cg09100014	4.66E-09	2.06E-06	6.527976	16	85937496	Body;Body;Body	876	*IRF8*	*IRF8*
cg15164733	4.66E-09	2.06E-06	6.527966	11	64827224	TSS1500	-1199	*NAALADL1*	*NAALADL1*
cg27327268	4.66E-09	2.06E-06	6.527896	10	1.18E+08	Body;Body;Body;Body;TES5000;TES5000	826	*C10orf82;HSPA12A*	*C10orf82*
cg00299105	4.67E-09	2.06E-06	6.527849	11	1.13E+08	Body	-14683	*TTC12*	*ANKK1*
cg07795964	4.67E-09	2.06E-06	6.527724	4	2066320	3'UTR	5267	*NAT8L*	*NAT8L*
cg25799473	4.68E-09	2.06E-06	6.52731	19	57881548	Body;TES5000	6630	*ZNF547;TRAPPC2B*	*TRAPPC2B*
cg06780673	4.75E-09	2.09E-06	6.523955	10	89367706		-51928		*PAPSS2*
cg19360675	4.77E-09	2.10E-06	-6.52303	17	53046073	TSS200;TSS200;TSS200;TSS200;5'UTR;5'UTR;5'UTR;TSS200	-52	*COX11;STXBP4*	*STXBP4*
cg00103908	4.80E-09	2.11E-06	6.521302	1	3184106	Body;Body	139568	*PRDM16*	*MIR4251*
cg07194832	4.82E-09	2.11E-06	6.52074	7	97978655	Body	51747	*BAIAP2L1*	*BAIAP2L1*
cg00788354	4.82E-09	2.11E-06	6.52061	6	1.68E+08	TSS5000;TES5000	-2186	*LINC01558;LOC441179*	*LINC01558*
cg18253910	4.82E-09	2.11E-06	6.520486	22	30230951	Body;Body;Body;Body;Body;Body;Body;Body;Body;Body;Body;Body;Body;Body;Body;Body;Body;Body;Body;Body;Body;Body;Body;Body;Body;Body;Body;Body;Body;Body;Body;Body;Body	3312	*ASCC2*	*ASCC2*
cg09419983	4.82E-09	2.11E-06	-6.52044	3	1.49E+08	TSS200;TSS200	-151	*HPS3*	*HPS3*
cg20826165	4.82E-09	2.11E-06	6.520303	12	64491841	Body;Body	124235	*SRGAP1*	*C12orf66*
cg00134340	4.83E-09	2.11E-06	6.520163	10	47966590		-189352		*CTSLP2*
cg09712066	4.85E-09	2.12E-06	6.518886	5	59783629	Body;Body;Body;Body;Body;TSS200;TSS200	90	*PDE4D;PART1*	*PART1*
cg22386774	4.86E-09	2.12E-06	6.518453	2	2.09E+08	TSS1500;TES5000	-1377	*CRYGA;C2orf80*	*CRYGA*
cg08054217	4.87E-09	2.12E-06	6.518356	20	33444618	Body;Body	16041	*GGT7*	*GGT7*
cg13534740	4.90E-09	2.13E-06	6.516699	17	79167630	Body;Body;Body;Body	-27609	*CEP131*	*AATK*
cg02291619	4.94E-09	2.15E-06	6.515187	5	1.42E+08	Body;Body;Body;Body	44739	*ARHGAP26*	*ARHGAP26-AS1*
cg26773567	4.98E-09	2.16E-06	6.513216	10	82173535	Body;Body;Body;Body;TSS200;TSS200	-39	*PRXL2A*	*PRXL2A*
cg02398789	4.98E-09	2.16E-06	6.513105	20	61453507	Body	5106	*COL9A3*	*COL9A3*
cg01545079	4.99E-09	2.16E-06	6.512748	14	24541377	TES5000;Body;Body	607	*CARMIL3;CPNE6*	*CPNE6*
cg20064693	5.01E-09	2.17E-06	6.5119	1	2.29E+08	Body;Body;Body	2951	*TRIM17*	*TRIM17*
cg00493358	5.05E-09	2.19E-06	6.510081	16	88980724	Body;Body	-25720	*CBFA2T3*	*LOC100129697*
cg26440309	5.07E-09	2.19E-06	6.50926	12	52403511	TSS5000;Body;TSS1500	-764	*GRASP-AS1;GRASP*	*GRASP*
cg00864551	5.07E-09	2.19E-06	6.508973	7	1.52E+08	Body;Body;3'UTR	3082	*PRKAG2;LOC644090*	*LOC644090*
cg21802919	5.09E-09	2.20E-06	6.508307	17	1850983	Body	-70026	*RTN4RL1*	*LOC105371485*
cg17501210	5.11E-09	2.20E-06	6.507481	6	1.67E+08	Body;Body;Body;Body	-12074	*RPS6KA2*	*RPS6KA2*
cg11860005	5.12E-09	2.21E-06	6.506861	22	32149125	TSS5000;TSS1500;TSS1500;TSS1500;TSS1500;TSS1500;TSS1500;TSS1500;TSS1500;TSS1500;TSS1500;TSS1500;TSS1500;TSS1500;TSS1500;TSS1500;TSS1500;TSS1500;TSS1500	-828	*PRR14L;DEPDC5*	*DEPDC5*
cg02276614	5.14E-09	2.21E-06	6.506222	19	55837381	TSS1500;TSS1500;TSS1500;TSS1500	-672	*TMEM150B*	*TMEM150B*
cg08052428	5.15E-09	2.22E-06	6.505526	9	1.36E+08	1stExon;1stExon;Body;Body;Body	206	*RALGDS*	*RALGDS*
cg23931796	5.17E-09	2.22E-06	6.504633	21	43491319	Body;Body;TSS200;TSS200	-106	*UMODL1*	*UMODL1*
cg00349700	5.21E-09	2.24E-06	6.502835	1	1.55E+08	Body;Body	-21219	*TDRD10*	*UBE2Q1-AS1*
cg15428904	5.25E-09	2.25E-06	6.501422	20	57394097	TSS1500;TSS1500;Body	-729	*MIR296;MIR298;GNAS-AS1*	*MIR298*
cg10037068	5.25E-09	2.25E-06	6.501328	2	1.76E+08	TSS1500;Body	-739	*WIPF1*	*WIPF1*
cg15082394	5.25E-09	2.25E-06	6.501032	6	33264525	Body;1stExon;TES5000;TES5000	2194	*RGL2;TAPBP*	*RGL2*
cg04198042	5.31E-09	2.27E-06	6.498873	17	48169370	TES5000;TSS5000;TSS5000;TSS5000;TSS5000	-2730	*ITGA3;PDK2*	*PDK2*
cg21240640	5.33E-09	2.28E-06	6.497824	2	27799388	5'UTR	0	*C2orf16*	*C2orf16*
cg06286521	5.34E-09	2.28E-06	6.497612	10	77909928	Body;Body;Body	367410	*LRMDA*	*LRMDA*
cg13446911	5.34E-09	2.28E-06	6.497482	9	96077700	Body;Body;TES5000	30885	*WNK2;C9orf129*	*C9orf129*
cg20122849	5.35E-09	2.28E-06	6.497146	19	18084724	1stExon	22614	*KCNN1*	*KCNN1*
cg25055932	5.36E-09	2.28E-06	6.496583	4	1.38E+08	Body	-380840	*LINC02511*	*LINC02510*
cg00858415	5.37E-09	2.28E-06	6.496259	2	2.32E+08	TSS5000	-3375	*NMUR1*	*NMUR1*
cg13732058	5.38E-09	2.28E-06	6.495822	4	28829667		8464		*MIR4275*
cg03571301	5.38E-09	2.28E-06	6.495686	17	80019566	TES5000;TES5000;TES5000;TES5000;TES5000;TES5000;TES5000;TES5000;TES5000;Body	4114	*GPS1;DUS1L*	*DUS1L*
cg12077664	5.38E-09	2.28E-06	6.495582	12	1.25E+08		-93288		*NCOR2*
cg09110634	5.38E-09	2.28E-06	6.495541	2	2.33E+08	TSS1500	-1437	*MIR1471*	*MIR1471*
cg03555203	5.38E-09	2.28E-06	6.495524	19	31770188	Body;Body;Body	70154	*TSHZ3*	*TSHZ3*
cg03344433	5.39E-09	2.28E-06	6.495308	5	84126213		-445515		*EDIL3*
cg16210324	5.40E-09	2.28E-06	6.494829	6	1.62E+08	Body;Body;Body	-31447	*PRKN*	*LOC105378098*
cg19784054	5.41E-09	2.28E-06	6.494267	15	81666847	Body;TSS1500	-293	*TMC3-AS1;TMC3*	*TMC3*

Probe	p_adj	q_val	t	Chr	Pos.hg19	locations	dist2TSS	gene.Illumina	gene.nearest
cg01938514	5.42E-09	2.28E-06	6.494134	16	18166238		263479		*NPIPA8*
cg07122319	5.44E-09	2.29E-06	6.493206	1	55227533	Body	2663	*PARS2*	*PARS2*
cg11032634	5.48E-09	2.31E-06	-6.49149	22	19929254	TSS5000;1stExon;1stExon;Body;1stExon;TSS200;TSS200	-40	*TXNRD2;COMT*	*COMT*
cg26013796	5.49E-09	2.31E-06	6.491341	12	46948457	Body;Body;Body;Body;Body	170568	*LOC100288798*	*LOC100288798*
cg00974578	5.51E-09	2.31E-06	6.490444	6	1.44E+08		-12831		*PHACTR2*
cg13927938	5.52E-09	2.32E-06	6.489937	1	2.04E+08	TSS200	-110	*PIK3C2B*	*PIK3C2B*
cg11771002	5.53E-09	2.32E-06	6.48952	5	1.13E+08	Body;Body	42004	*MCC*	*MCC*
cg22912101	5.53E-09	2.32E-06	6.489433	19	11918238	3'UTR	-6849	*ZNF491*	*ZNF440*
cg14522150	5.57E-09	2.33E-06	6.487728	10	50616478		-12343		*DRGX*
cg04280548	5.61E-09	2.35E-06	6.486103	11	1.2E+08	Body;Body	6789	*TRIM29*	*TRIM29*
cg19698416	5.62E-09	2.35E-06	6.486048	15	73996268	Body;Body;Body;Body	19158	*CD276*	*CD276*
cg27664018	5.63E-09	2.35E-06	6.485657	16	57405979	TSS1500;TSS1500	-422	*CX3CL1*	*CX3CL1*
cg02743895	5.64E-09	2.35E-06	6.485042	17	77394015	Body;Body;Body	84665	*RBFOX3*	*RBFOX3*
cg13324448	5.66E-09	2.36E-06	6.484338	3	40752056		185606		*ZNF621*
cg01296230	5.68E-09	2.36E-06	6.483658	2	12894736		17244		*MIR3125*
cg04409427	5.68E-09	2.36E-06	6.483632	21	35736023	TSS1500	-299	*KCNE2*	*KCNE2*
cg14305383	5.68E-09	2.36E-06	6.483557	9	1.32E+08	TES5000;TES5000;TES5000;TES5000;TES5000;TES5000;TES5000;TES5000;TES5000;TES5000;Body;Body;3'UTR	4214	*NTMT1;ASB6*	*ASB6*
cg26632171	5.68E-09	2.36E-06	6.483323	2	17997117	TSS1500	-609	*MSGN1*	*MSGN1*
cg18607580	5.70E-09	2.36E-06	6.482493	10	13542696	Body;Body;Body;Body	2280	*BEND7*	*BEND7*
cg27306680	5.72E-09	2.37E-06	6.481924	2	2.18E+08		-18379		*IGFBP5*
cg13166781	5.73E-09	2.37E-06	6.481476	7	45115954	Body;3'UTR;3'UTR;3'UTR;3'UTR;3'UTR;3'UTR;TES5000	12614	*CCM2;NACAD*	*NACAD*
cg05431171	5.73E-09	2.37E-06	6.481317	6	35461405	Body;TES5000;TES5000	3451	*TEAD3;TULP1*	*TEAD3*
cg25768250	5.76E-09	2.38E-06	6.480309	1	2117733	TES5000;TES5000;TES5000;TES5000;TES5000;TES5000;TES5000;TES5000;TES5000;TSS5000;Body;Body;Body;Body;Body;TES5000;TES500 0;TES5000;TES5000	-2419	*PRKCZ;PRKCZ-AS1;FAAP20*	*PRKCZ-AS1*
cg18471029	5.80E-09	2.39E-06	6.478929	6	1.37E+08	Body;TSS5000	-3703	*MAP3K5;MAP3K5-AS1*	*MAP3K5-AS1*
cg08156016	5.80E-09	2.39E-06	6.478866	15	62990470	Body	50961	*TLN2*	*TLN2*
cg21178618	5.82E-09	2.40E-06	6.478127	14	1.01E+08	TSS5000	-3921	*MIR379*	*MIR379*
cg02196805	5.83E-09	2.40E-06	6.477702	5	1.31E+08	1stExon	156	*CSF2*	*CSF2*
cg03684886	5.83E-09	2.40E-06	6.477683	7	1.39E+08	TSS1500;TSS1500	-1168	*KIAA1549*	*KIAA1549*
cg10598353	5.86E-09	2.41E-06	-6.47629	6	31783466	TSS1500;5'UTR	147	*HSPA1L;HSPA1A*	*HSPA1A*
cg07316556	5.86E-09	2.41E-06	6.476281	2	1.01E+08		-53017		*NPAS2*
cg11805450	5.88E-09	2.41E-06	-6.47581	11	1.23E+08	Body;Body;Body;Body;Body;Body;Body;Body	16872	*GRAMD1B*	*GRAMD1B*
cg20601276	5.88E-09	2.41E-06	6.475787	20	36148133	Body;Body;Body;Body;Body;Body;Body;TSS5000;TSS5000;TSS5000	572	*BLCAP;NNAT*	*BLCAP*
cg13672547	5.89E-09	2.41E-06	6.475201	6	3562789		-105996		*SLC22A23*
cg16566595	5.91E-09	2.41E-06	6.474466	18	30090866	TSS1500	-759	*WBP11P1*	*WBP11P1*
cg18053030	5.91E-09	2.41E-06	6.474424	6	47097679		-87611		*ADGRF1*
cg08808817	5.92E-09	2.41E-06	6.474285	17	72690539	Body;3'UTR;3'UTR;3'UTR;3'UTR;3'UTR;3'UTR;3'UTR;Body	18523	*RAB37;CD300LF*	*CD300LF*
cg10896230	5.92E-09	2.41E-06	6.474235	8	11188675	TES5000;1stExon	181	*MTMR9;SLC35G5*	*SLC35G5*
cg23689441	5.93E-09	2.42E-06	6.473594	1	1248347	TES5000;TES5000;TES5000;Body;Body;Body;Body;Body;TSS1500	-401	*PUSL1;INTS11;MIR6727*	*MIR6727*
cg10702457	5.94E-09	2.42E-06	6.473529	4	88753179	Body;TSS1500;TSS1500;TSS1500;TSS1500;TSS5000	-941	*MEPE*	*MEPE*
cg14374781	5.94E-09	2.42E-06	6.473271	1	17739956	Body;Body	25076	*RCC2*	*RCC2*
cg04970014	5.95E-09	2.42E-06	6.473043	22	49043384	Body;Body	71233	*TAFA5*	*TAFA5*
cg14248981	5.97E-09	2.42E-06	6.472168	6	31937066	Body;TES5000;TES5000;TES5000;TSS5000;TSS5000;TSS5000	-1885	*SKIV2L;DXO;STK19*	*STK19*
cg02271490	5.99E-09	2.43E-06	6.471579	7	1.39E+08	Body;Body	-68964	*KIAA1549*	*ATP6V0A4*
cg16396153	5.99E-09	2.43E-06	6.471396	14	1.05E+08	Body;Body	17639	*AHNAK2*	*AHNAK2*
cg17602885	6.01E-09	2.43E-06	6.470582	1	9804627	Body;Body;Body	53103	*CLSTN1*	*PIK3CD*
cg21384114	6.06E-09	2.45E-06	6.468895	17	80320037	1stExon	2915	*TEX19*	*TEX19*
cg24838864	6.08E-09	2.46E-06	6.468115	19	11598009	Body	-6148	*ZNF653*	*ELAVL3*
cg05642993	6.10E-09	2.46E-06	6.46742	4	1.19E+08		67269		*LINC02264*
cg21728844	6.10E-09	2.46E-06	6.467251	2	2.34E+08	Body;Body	9195	*NGEF*	*SNORC*
cg00240378	6.15E-09	2.47E-06	6.465562	2	8850020		27835		*ID2*
cg15549029	6.15E-09	2.47E-06	6.465539	5	1.38E+08	TSS5000	-3689	*GFRA3*	*GFRA3*
cg09359750	6.15E-09	2.47E-06	6.465506	3	58708188		-55687		*FAM3D*
cg27045264	6.15E-09	2.47E-06	6.465349	16	31441954	TES5000;TES5000;TSS5000;TES5000;TES5000	-2273	*ITGAD;COX6A2;ZNF843*	*COX6A2*
cg26886231	6.16E-09	2.47E-06	6.465173	17	78936889	Body;Body;TES5000	8124	*RPTOR;LOC400627*	*LOC400627*
cg07783183	6.16E-09	2.47E-06	6.465119	11	75852355	Body	65191	*UVRAG*	*WNT11*
cg12117975	6.17E-09	2.47E-06	6.464682	15	95577957		179366		*LOC440311*
cg06127781	6.20E-09	2.48E-06	6.463675	16	15240413		8446		*MIR3180-4*
cg21767191	6.20E-09	2.48E-06	6.463591	3	17082105	Body;Body	107418	*PLCL2*	*MIR3714*
cg02973377	6.21E-09	2.48E-06	6.463234	19	15342548	Body;Body	711	*EPHX3*	*EPHX3*

Probe	p_adj	q_val	t	Chr	Pos.hg19	locations	dist2TSS	gene.Illumina	gene.nearest
cg07453911	6.24E-09	2.49E-06	6.462387	19	19645610	Body;Body;TSS5000	-3463	*YJEFN3;CILP2*	*CILP2*
cg02261294	6.24E-09	2.49E-06	6.462382	5	1.78E+08	Body;Body;Body;Body;Body;Body;Body	4548	*HNRNPAB;PHYKPL*	*HNRNPAB*
cg14157417	6.24E-09	2.49E-06	6.462345	7	1.58E+08	Body;Body;Body;Body;Body	60020	*PTPRN2*	*MIR595*
cg17810966	6.26E-09	2.49E-06	6.461534	4	95460771	Body;Body;Body;Body;Body;Body;Body;Body	87679	*PDLIM5*	*PDLIM5*
cg11876177	6.27E-09	2.49E-06	6.4613	6	25874121	Body;Body	350	*SLC17A3*	*SLC17A3*
cg20839145	6.28E-09	2.49E-06	6.460907	3	1.24E+08	Body;Body;Body;Body;Body	75082	*MYLK*	*MYLK*
cg01590076	6.28E-09	2.49E-06	6.460801	8	70246362		114117		*LINC01603*
cg11374169	6.29E-09	2.50E-06	6.460491	2	2.08E+08		-96425		*CREB1*
cg21368479	6.30E-09	2.50E-06	6.459966	6	1.49E+08		-61221		*LOC105378047*
cg26815611	6.31E-09	2.50E-06	6.459861	19	13919905	Body;Body	13649	*ZSWIM4*	*ZSWIM4*
cg10973789	6.34E-09	2.50E-06	6.458739	11	1.16E+08		71726		*LOC101928985*
cg13256308	6.34E-09	2.50E-06	6.458734	14	93106286	Body;Body	63708	*RIN3*	*RIN3*
cg00491335	6.34E-09	2.50E-06	6.458678	2	2.25E+08	TSS5000;TSS5000;TSS5000	-3355	*AP1S3*	*AP1S3*
cg05740739	6.36E-09	2.51E-06	6.457807	2	2.41E+08	TSS1500	-1162	*OR6B3*	*OR6B3*
cg11971918	6.37E-09	2.51E-06	6.457484	11	1.11E+08	Body;Body;Body;Body	15831	*LAYN*	*LAYN*
cg11332681	6.39E-09	2.52E-06	6.456819	1	2.23E+08	Body;Body	-18045	*DISP1*	*DISP1*
cg24030037	6.42E-09	2.53E-06	6.45583	8	27457282	Body;Body;Body	10986	*CLU*	*MIR6843*
cg03474702	6.43E-09	2.53E-06	6.455498	18	12030535	3'UTR	49030	*IMPA2*	*IMPA2*
cg09534489	6.43E-09	2.53E-06	6.455498	16	1609908	Body;TES5000;TES5000	-20619	*IFT140;TMEM204*	*LOC105371046*
cg23468144	6.43E-09	2.53E-06	6.455293	19	1322527		-32430		*PWWP3A*
cg00906476	6.44E-09	2.53E-06	6.4551	5	1.39E+08	Body;Body;Body;Body;Body;Body;Body;Body;Body	7061	*CXXC5*	*CXXC5*
cg22379463	6.47E-09	2.53E-06	6.454239	3	45260330		7449		*TMEM158*
cg14932952	6.47E-09	2.53E-06	6.454037	3	1.88E+08	Body;5'UTR;5'UTR	90	*LPP*	*LPP*
cg16682721	6.48E-09	2.53E-06	6.453573	11	71931577	Body;Body;Body;Body;TSS5000	3740	*FOLR2;INPPL1*	*FOLR2*
cg10163220	6.49E-09	2.53E-06	6.453386	12	1.05E+08		-74640		*CHST11*
cg01803856	6.49E-09	2.53E-06	6.453312	17	650796	TES5000;TES5000;TES5000;TES5000;Body;TSS5000	4708	*TLCD3A;GEMIN4;DBIL5P*	*GEMIN4*
cg13485481	6.49E-09	2.53E-06	6.45321	7	2186344	Body;Body;Body;Body;Body	-57657	*MAD1L1*	*SNORA114*
cg25827873	6.52E-09	2.54E-06	6.45242	8	593655	Body	87569	*ERICH1*	*ERICH1*
cg06807993	6.52E-09	2.54E-06	6.452279	17	79099785	Body;Body;TSS1500;TES5000;TSS200	-36	*AATK;MIR657;MIR3065;MIR338*	*MIR338*
cg14989351	6.53E-09	2.54E-06	6.452024	14	83282545		173570		*LINC02301*
cg01995099	6.53E-09	2.54E-06	6.451993	22	42394048	3'UTR;3'UTR;3'UTR;TSS1500	-743	*SEPTIN3;WBP2NL*	*WBP2NL*
cg21465236	6.53E-09	2.54E-06	6.451926	2	1933212	Body;Body;Body;Body;Body;Body;Body;Body;Body;Body	-184925	*MYT1L*	*PXDN*
cg00945260	6.56E-09	2.54E-06	6.450983	16	1104925		-17830		*SSTR5*
cg14497545	6.56E-09	2.54E-06	6.450955	4	1.41E+08	Body	167503	*MAML3*	*MGST2*
cg19835040	6.56E-09	2.54E-06	6.45079	3	46538742	TSS1500	-723	*RTP3*	*RTP3*
cg00762830	6.56E-09	2.54E-06	6.450777	16	88990161	Body;Body	-16283	*CBFA2T3*	*LOC100129697*
cg18063824	6.60E-09	2.55E-06	6.44964	4	54075042	Body	157186	*SCFD2*	*SCFD2*
cg12412384	6.61E-09	2.55E-06	6.449395	12	12673025	Body	42772	*DUSP16*	*DUSP16*
cg17750946	6.62E-09	2.55E-06	6.448915	10	1.35E+08	Body;Body	40014	*INPP5A*	*NKX6-2*
cg05245416	6.62E-09	2.55E-06	6.448888	2	1.05E+08	Body	36206	*LINC01102*	*LINC01102*
cg23325999	6.63E-09	2.56E-06	6.448479	19	5217431	Body;Body;Body;Body	123392	*PTPRS*	*PTPRS*
cg26191586	6.68E-09	2.57E-06	6.446981	1	53794760	TSS1500;TSS1500;TSS1500;TSS1500;Body	856	*LRP8;LINC01771*	*LINC01771*
cg13856573	6.69E-09	2.58E-06	6.446477	11	62475078	TSS1500;TSS1500;5'UTR;Body;Body;Body;TSS200	-69	*BSCL2;HNRNPUL2-BSCL2;GNG3*	*GNG3*
cg08926040	6.71E-09	2.58E-06	6.445821	11	8638598	3'UTR	-22823	*TRIM66*	*STK33*
cg10891959	6.74E-09	2.59E-06	6.444695	13	81432486		-160039		*LINC00377*
cg17044090	6.75E-09	2.59E-06	6.444443	1	2.48E+08		-28572		*NLRP3*
cg19596509	6.76E-09	2.59E-06	6.443997	3	1.84E+08	TSS1500;TSS5000	-1366	*LINC01840;EPHB3*	*LINC01840*
cg17095916	6.78E-09	2.60E-06	6.443528	12	56226873	TSS5000;Body;Body;Body;Body;TES5000;TES5000;TES5000	-3453	*DNAJC14;TMEM198B;MMP19*	*DNAJC14*
cg16298761	6.78E-09	2.60E-06	6.44342	8	1.45E+08	Body	7181	*WDR97*	*WDR97*
cg06908450	6.79E-09	2.60E-06	6.443123	17	79921870	TSS5000	-2807	*NOTUM*	*NOTUM*
cg06093201	6.80E-09	2.60E-06	6.44288	16	1405095	TSS5000;Body	3164	*TSR3;GNPTG*	*GNPTG*
cg23393549	6.80E-09	2.60E-06	6.442742	5	54058686		-18600		*LOC102467080*
cg01591973	6.81E-09	2.60E-06	6.442595	19	37583283	Body;Body;Body;Body;Body;Body;Body;Body;Body;Body;Body	13949	*ZNF420*	*ZNF420*
cg21661864	6.83E-09	2.60E-06	6.441917	2	2.41E+08		-84746		*LOC150935*
cg05785100	6.86E-09	2.61E-06	6.44099	6	1.69E+08	Body;Body	79374	*SMOC2*	*SMOC2*
cg20685981	6.91E-09	2.63E-06	6.439253	19	42871187	Body;Body	15905	*MEGF8*	*MIR8077*
cg11390378	6.92E-09	2.63E-06	6.438845	4	1.48E+08		16183		*POU4F2*
cg26506947	6.92E-09	2.63E-06	6.438714	16	25228688	Body	421	*AQP8*	*AQP8*
cg14760438	6.93E-09	2.63E-06	6.438493	6	33409448	Body;Body;TES5000	3341	*SYNGAP1;MIR5004*	*MIR5004*
cg01624504	6.93E-09	2.63E-06	6.438405	13	25643386		-26889		*PABPC3*

Probe	p_adj	q_val	t	Chr	Pos.hg19	locations	dist2TSS	gene.Illumina	gene.nearest
cg02634641	6.94E-09	2.63E-06	6.438223	1	16369395	TSS1500	-881	*CLCNKB*	*CLCNKB*
cg18674466	6.94E-09	2.63E-06	6.438168	3	9400729	TSS5000;TSS5000	-4008	*THUMPD3*	*THUMPD3*
cg09298818	6.96E-09	2.63E-06	6.437572	12	1.11E+08	TSS1500;TSS1500	-850	*PPP1CC*	*PPP1CC*
cg09416203	6.96E-09	2.63E-06	6.437436	7	94928249	TES5000;TES5000;TES5000;TES5000;TES5000;3'UTR	25595	*PPP1R9A;PON1*	*PON1*
cg19902593	7.02E-09	2.65E-06	6.435505	1	56038708		347395		*MIR4422*
cg14570531	7.03E-09	2.65E-06	6.435262	6	32168751	Body;Body;Body	-5451	*NOTCH4*	*GPSM3*
cg08200856	7.05E-09	2.66E-06	6.434626	8	1.44E+08	TES5000;Body	9476	*ZFP41*	*ZFP41*
cg00402366	7.07E-09	2.66E-06	-6.43416	1	74663750	5'UTR;5'UTR;TSS200;TSS200;TSS200;TSS200;TSS200	110	*LRRIQ3;FPGT;FPGT-TNNI3K*	*LRRIQ3*
cg23033024	7.12E-09	2.68E-06	6.432538	20	23472484	1stExon;1stExon	731	*CST8*	*CST8*
cg13968099	7.13E-09	2.68E-06	-6.43209	5	1.63E+08	5'UTR;5'UTR;Body;5'UTR;5'UTR;5'UTR;5'UTR;Body;Body;Body;Body;Body;Body;5'UTR;5'UTR	168	*CCNG1*	*CCNG1*
cg14336112	7.15E-09	2.69E-06	6.431466	15	77645762	Body	66684	*PEAK1*	*PEAK1*
cg26785882	7.17E-09	2.69E-06	6.430698	3	1.78E+08		-14064		*LINC02015*
cg13379822	7.19E-09	2.69E-06	6.43027	12	54816219	Body;Body;TSS5000;Body	2651	*LOC102724050;ITGA5*	*LOC102724050*
cg00933415	7.19E-09	2.69E-06	6.43025	6	33135453	Body;Body;Body	24353	*COL11A2*	*HCG24*
cg09430586	7.19E-09	2.69E-06	6.43007	19	54242124	TES5000;TES5000;TSS1500;TSS5000;TSS5000	-462	*MIR518D;MIR516B1;MIR518A2;MIR517C;MIR 520H*	*MIR518A2*
cg09433131	7.20E-09	2.70E-06	6.429786	8	73480179	1stExon	30720	*KCNB2*	*KCNB2*
cg19817126	7.21E-09	2.70E-06	6.429612	10	93357526	Body	13691	*HECTD2-AS1*	*HECTD2-AS1*
cg01827999	7.22E-09	2.70E-06	6.429354	17	17791914	Body;Body;Body;Body;Body;Body;Body;Body;Body	-51589	*TOM1L2*	*SREBF1*
cg06234941	7.25E-09	2.71E-06	6.428299	3	1.94E+08	Body;Body;Body;Body	20072	*TMEM44*	*TMEM44-AS1*
cg17944981	7.26E-09	2.71E-06	6.427934	13	94461196	Body	49530	*GPC6*	*GPC6-AS2*
cg03037969	7.27E-09	2.71E-06	6.427743	20	1277814	Body;Body	16503	*SNPH*	*SDCBP2*
cg25974167	7.29E-09	2.71E-06	6.427226	3	1.28E+08	Body	-20579	*EEFSEC*	*RUVBL1*
cg14437072	7.30E-09	2.71E-06	6.426911	8	1.44E+08	Body	10085	*RHPN1*	*RHPN1*
cg08355099	7.32E-09	2.72E-06	6.426097	1	2.04E+08	Body	-41704	*PLEKHA6*	*GOLT1A*
cg16095411	7.33E-09	2.72E-06	6.425916	17	55762444	TES5000;TES5000	60216	*MSI2*	*CCDC182*
cg19539519	7.34E-09	2.73E-06	6.425414	17	26553687	Body	99	*PYY2*	*PYY2*
cg02424569	7.36E-09	2.73E-06	6.425039	3	14851410	Body	1511	*LINC02011*	*LINC02011*
cg22143209	7.36E-09	2.73E-06	6.424789	7	4254977	Body;Body	-80508	*SDK1*	*LOC105375131*
cg11582369	7.38E-09	2.73E-06	6.424439	1	43788427	3'UTR;3'UTR	-15061	*TIE1*	*MPL*
cg11295935	7.38E-09	2.73E-06	6.424254	3	87018446	Body;Body;Body	21853	*VGLL3*	*VGLL3*
cg00880872	7.39E-09	2.73E-06	6.424001	9	1.25E+08		-5372		*MIR4478*
cg16811443	7.39E-09	2.73E-06	6.423998	17	20517129	Body	19435	*LINC02088*	*LINC02088*
cg14101380	7.39E-09	2.73E-06	6.423894	11	70718575	Body;TSS200	-102	*SHANK2;MIR3664*	*MIR3664*
cg24150157	7.41E-09	2.73E-06	6.423252	19	51891210	5'UTR;5'UTR;TES5000;TES5000	18	*LIM2;C19orf84*	*LIM2*
cg12848315	7.49E-09	2.76E-06	6.420959	14	76479761	Body;Body;Body;Body;Body	27656	*IFT43*	*IFT43*
cg20847325	7.49E-09	2.76E-06	6.420887	1	64148960		60074		*PGM1*
cg18833133	7.50E-09	2.76E-06	6.420708	8	1.2E+08	Body;Body;Body;Body;Body	-15847	*SAMD12*	*SAMD12*
cg20784098	7.54E-09	2.77E-06	6.419349	1	32157059	Body	12559	*COL16A1*	*COL16A1*
cg17329004	7.55E-09	2.77E-06	6.419236	19	49714934	3'UTR;3'UTR;3'UTR;3'UTR;3'UTR;3'UTR	53886	*TRPM4*	*TRPM4*
cg07347049	7.56E-09	2.77E-06	6.41888	13	1.14E+08	TSS5000;Body;Body	3993	*MCF2L-AS1;MCF2L*	*MCF2L*
cg18249908	7.57E-09	2.78E-06	6.418416	8	22864325	Body;Body;Body	7234	*RHOBTB2*	*RHOBTB2*
cg00394718	7.58E-09	2.78E-06	6.418356	8	1.21E+08	Body	709	*ENPP2*	*ENPP2*
cg09466667	7.60E-09	2.78E-06	6.417665	3	42140775	Body;Body;Body;Body;Body;Body	7927	*TRAK1*	*TRAK1*
cg10841540	7.61E-09	2.78E-06	6.417279	14	59907007		25091		*GPR135*
cg06704466	7.62E-09	2.78E-06	6.417011	2	2.37E+08	Body;Body	66772	*AGAP1*	*GBX2*
cg14588828	7.63E-09	2.78E-06	6.416627	9	1.17E+08	Body;Body;Body	16383	*AKNA*	*ORM2*
cg02660541	7.64E-09	2.78E-06	6.416483	16	3193410	TES5000;TES5000;TES5000;TSS1500	-881	*ZNF213;CASP16P*	*CASP16P*
cg27117439	7.64E-09	2.78E-06	6.416392	2	33151557	Body;Body;TSS1500	-636	*LINC00486*	*LINC00486*
cg21706038	7.65E-09	2.78E-06	6.416313	2	3317693	Body;Body;Body	63896	*EIPR1*	*EIPR1*
cg17191518	7.68E-09	2.79E-06	6.415345	15	52468850	Body;Body;TSS5000	3272	*GNB5;CERNA1*	*GNB5*
cg25540579	7.68E-09	2.79E-06	-6.41533	19	1653193	TSS1500;TSS1500;TSS1500;TSS1500	-579	*TCF3*	*TCF3*
cg15021658	7.70E-09	2.80E-06	6.414673	11	61249826	Body;Body	1235	*PPP1R32*	*PPP1R32*
cg24060938	7.70E-09	2.80E-06	6.414617	7	1.3E+08	Body	497	*CPA2*	*CPA2*
cg02434866	7.71E-09	2.80E-06	6.414343	16	88964786	Body;Body	14864	*CBFA2T3*	*LOC101927793*
cg15894581	7.74E-09	2.80E-06	6.41354	15	63889406	TES5000;TES5000;TES5000;TES5000;Body;TSS200;TSS200;TSS200;TSS200	-179	*USP3;USP3-AS1;FBXL22*	*FBXL22*
cg01895989	7.74E-09	2.80E-06	6.413391	16	58166238	TSS5000;TSS5000	-2909	*CFAP20*	*CFAP20*
cg02857384	7.75E-09	2.80E-06	6.413253	16	89332300	TES5000;TES5000;TES5000	-30235	*ANKRD11*	*LOC105371414*
cg14091531	7.77E-09	2.81E-06	6.412536	1	13881461		-28790		*PDPN*
cg13274775	7.77E-09	2.81E-06	6.412518	6	74078997	Body	518	*OOEP*	*OOEP*

Probe	p_adj	q_val	t	Chr	Pos.hg19	locations	dist2TSS	gene.Illumina	gene.nearest
cg13810766	7.78E-09	2.81E-06	6.412468	7	1.52E+08	Body	-30495	*PRKAG2*	*PRKAG2*
cg22838399	7.78E-09	2.81E-06	6.41238	5	1.07E+08	Body	93853	*EFNA5*	*EFNA5*
cg06136617	7.78E-09	2.81E-06	6.412249	16	4027502	Body	-27057	*ADCY9*	*LINC02861*
cg07492281	7.80E-09	2.81E-06	6.411873	7	966947	TSS200;Body;Body;Body	-95	*ADAP1*	*ADAP1*
cg09529165	7.81E-09	2.81E-06	6.411372	17	45808345	TSS5000	-2256	*TBX21*	*TBX21*
cg21211321	7.82E-09	2.81E-06	6.41112	11	1.34E+08	Body	10449	*IGSF9B*	*IGSF9B*
cg21113603	7.84E-09	2.81E-06	6.410751	19	34306891	TES5000;TES5000;TES5000	19117	*KCTD15*	*KCTD15*
cg03445173	7.86E-09	2.82E-06	6.409971	2	17998337	1stExon	611	*MSGN1*	*MSGN1*
cg13748657	7.87E-09	2.82E-06	6.409887	17	80973520	Body;Body;Body	-8647	*B3GNTL1*	*B3GNTL1*
cg24615251	7.87E-09	2.82E-06	6.40975	17	32683481	TSS200;TES5000	-17	*CCL13;CCL1*	*CCL13*
cg19478343	7.91E-09	2.83E-06	6.408503	20	49620679	Body	18965	*KCNG1*	*KCNG1*
cg00339012	7.92E-09	2.83E-06	6.408214	2	5865442		32927		*SOX11*
cg23208881	7.95E-09	2.84E-06	6.407506	15	91411495	TSS1500;TSS5000;TSS5000	-322	*FURIN*	*FURIN*
cg20638429	7.96E-09	2.84E-06	6.407248	22	24979642	TSS200;TES5000	-75	*GGT1;LRRC75B*	*GGT1*
cg14508832	7.98E-09	2.85E-06	6.406724	1	2404894	Body;TSS5000;TSS5000	-2833	*PLCH2*	*PLCH2*
cg06905155	8.02E-09	2.86E-06	6.40543	2	2.41E+08	TES5000	39393	*LOC150935*	*LOC150935*
cg05708396	8.03E-09	2.86E-06	6.405241	7	47531340	Body	-26939	*TNS3*	*SNORD151*
cg03072483	8.06E-09	2.87E-06	6.40427	17	78327711	Body;Body	-61255	*RNF213;RNF213-AS1*	*ENDOV*
cg13789881	8.08E-09	2.87E-06	6.403809	2	86094755	5'UTR;Body;Body;Body;Body;Body;Body;Body;Body;Body;Body	38	*ST3GAL5*	*ST3GAL5*
cg11393040	8.09E-09	2.88E-06	6.403371	1	47907054	TSS5000;TES5000	3339	*LINC01389;FOXD2*	*FOXD2*
cg18589874	8.11E-09	2.88E-06	6.403017	11	47608310	TES5000;5'UTR;5'UTR;5'UTR;5'UTR;TES5000	108	*NDUFS3;FAM180B;C1QTNF4*	*FAM180B*
cg05338567	8.12E-09	2.88E-06	6.402795	6	31859187	Body;Body;Body;Body;Body	5579	*EHMT2*	*EHMT2*
cg01118569	8.13E-09	2.88E-06	6.402387	5	1.24E+08		-138107		*ZNF608*
cg11344073	8.15E-09	2.89E-06	6.401726	2	37376067	5'UTR;TSS1500;5'UTR	-1102	*EIF2AK2*	*EIF2AK2*
cg00629117	8.16E-09	2.89E-06	6.401574	16	1844932	TSS1500;TSS1500;TSS200	-23	*IGFALS*	*IGFALS*
cg24048650	8.17E-09	2.89E-06	6.401339	21	47828142	Body;Body	-50661	*PCNT*	*DIP2A*
cg26176014	8.18E-09	2.89E-06	6.40102	17	80491645	Body	14038	*FOXK2*	*FOXK2*
cg24492694	8.18E-09	2.89E-06	6.401009	11	2595605	Body;Body	112922	*KCNQ1*	*KCNQ1*
cg13580755	8.19E-09	2.89E-06	6.400647	6	1.67E+08	Body;Body;Body;Body;Body	-13088	*RPS6KA2*	*RPS6KA2-IT1*
cg25029562	8.20E-09	2.89E-06	6.400327	4	25886418		-21230		*SEL1L3*
cg04366300	8.21E-09	2.89E-06	6.400232	11	1.25E+08	Body	53030	*PKNOX2*	*PKNOX2*
cg09001591	8.22E-09	2.89E-06	6.39996	2	29236577	Body;Body;Body	32414	*TOGARAM2*	*TOGARAM2*
cg07890827	8.22E-09	2.89E-06	6.399862	10	1.32E+08	Body;TES5000	5016	*EBF3;MIR4297*	*MIR4297*
cg19618984	8.23E-09	2.89E-06	6.399733	18	77337219	TSS5000	-2062	*LOC284240*	*LOC284240*
cg00449344	8.23E-09	2.89E-06	6.399514	12	658824	Body	36828	*B4GALNT3*	*LOC105369595*
cg13724780	8.24E-09	2.89E-06	-6.39944	5	1.8E+08	TSS1500;TSS1500;TSS1500;TSS1500;Body;Body;Body;Body;Body;Body;Body;TSS1500;5'UTR;TSS1500;TSS1500;TSS1500;Body	52	*MGAT1*	*MGAT1*
cg27106356	8.24E-09	2.89E-06	6.399428	16	11363968	TSS1500;TES5000	-786	*TNP2;PRM3*	*TNP2*
cg27395226	8.24E-09	2.89E-06	6.399384	7	1.05E+08	Body;Body;Body;Body;Body;Body;Body	35046	*SRPK2*	*SRPK2*
cg07215518	8.26E-09	2.89E-06	6.398755	13	22534365		-82066		*LINC00424*
cg06259339	8.28E-09	2.90E-06	6.398338	6	1.49E+08	Body;TSS200	-143	*UST;UST-AS1*	*UST-AS1*
cg06336926	8.28E-09	2.90E-06	6.398147	6	22571177	3'UTR	1383	*HDGFL1*	*HDGFL1*
cg09452257	8.31E-09	2.91E-06	6.397304	16	2321599	TSS5000;TSS5000;TSS5000;TSS5000;TSS5000;TSS5000;Body;TES5000;TSS200;TSS5000;TES5000	-148	*RNPS1;LOC106660606;MIR3677;MIR940;MIR4717;ABCA3*	*MIR940*
cg06085038	8.38E-09	2.93E-06	6.395577	17	45946982		-13919		*SP6*
cg02697979	8.41E-09	2.93E-06	6.394738	11	34265361	Body	114196	*ABTB2*	*ABTB2*
cg24627325	8.41E-09	2.93E-06	6.394711	3	1.02E+08	TSS1500	-222	*LOC101929411*	*LOC101929411*
cg13463109	8.42E-09	2.93E-06	6.394401	22	42522669	TES5000;Body;Body	4143	*NDUFA6-DT;CYP2D6*	*CYP2D6*
cg18671115	8.44E-09	2.94E-06	6.393876	6	33154189	Body;Body;Body;TES5000	6055	*COL11A2*	*COL11A2*
cg22884541	8.47E-09	2.95E-06	6.39311	7	97841864	TES5000;1stExon;TES5000	299	*LMTK2;BHLHA15;TECPR1*	*BHLHA15*
cg23218877	8.49E-09	2.95E-06	6.392578	10	35536010	5'UTR	58	*CCNY*	*CCNY*
cg24680570	8.52E-09	2.96E-06	6.391752	1	2.33E+08		-75484		*SIPA1L2*
cg14215786	8.53E-09	2.96E-06	6.391532	14	1.01E+08	Body;Body;Body;Body;Body;Body	14808	*WDR25*	*WDR25*
cg09055992	8.54E-09	2.96E-06	6.391366	8	1626697	Body	-56867	*DLGAP2*	*DLGAP2-AS1*
cg04049637	8.54E-09	2.96E-06	6.391302	7	56296570		-112460		*NUPR2*
cg15744869	8.55E-09	2.96E-06	6.390857	10	96219442	Body	57145	*TBC1D12*	*TBC1D12*
cg15676916	8.59E-09	2.97E-06	6.389912	10	1.29E+08	Body;Body	-156254	*DOCK1*	*NPS*
cg01699581	8.59E-09	2.97E-06	6.389841	11	1.3E+08		37244		*ADAMTS15*
cg08822689	8.59E-09	2.97E-06	6.389802	16	79021949	Body;Body	612685	*WWOX*	*MAF*
cg20603162	8.60E-09	2.97E-06	6.389568	4	1.23E+08	Body;Body;Body	25559	*ADAD1*	*ADAD1*

Probe	p_adj	q_val	t	Chr	Pos.hg19	locations	dist2TSS	gene.Illumina	gene.nearest
cg20077007	8.64E-09	2.98E-06	6.388524	8	713183	Body;Body	24636	*DLGAP2*	*LOC401442*
cg11448270	8.66E-09	2.98E-06	6.387991	6	1.16E+08	Body;TSS1500	-1436	*FRK;TPI1P3*	*TPI1P3*
cg09182900	8.69E-09	2.99E-06	6.38737	6	43909955	TES5000	51191	*LINC01512*	*LINC01512*
cg25342675	8.75E-09	3.00E-06	6.385732	5	63465753	Body;Body;Body;Body	4084	*RNF180*	*RNF180*
cg03480308	8.75E-09	3.00E-06	6.385693	22	50493212	Body;TES5000;TES5000	3444	*TTLL8;MLC1*	*TTLL8*
cg18718410	8.75E-09	3.00E-06	6.385674	19	15285943	Body;TES5000	4218	*NOTCH3;MIR6795*	*MIR6795*
cg22378583	8.76E-09	3.00E-06	6.385509	6	1.43E+08		-6217		*HIVEP2*
cg10618882	8.76E-09	3.00E-06	6.385497	19	18509358	TSS1500	-926	*LRRC25*	*LRRC25*
cg20525663	8.77E-09	3.01E-06	6.385228	4	9482233		35974		*DEFB131A*
cg04132000	8.78E-09	3.01E-06	6.385041	1	2802609		-85176		*TTC34*
cg12197168	8.79E-09	3.01E-06	6.384562	5	83585467	Body;Body	95218	*EDIL3*	*EDIL3*
cg20671578	8.81E-09	3.01E-06	6.384179	11	70332529	Body;Body;Body	87895	*SHANK2*	*CTTN*
cg15736726	8.82E-09	3.01E-06	6.383963	3	1.59E+08	TSS1500;TSS1500;TSS1500;TSS1500;TSS1500	-847	*IQCJ;IQCJ-SCHIP1*	*IQCJ*
cg10050318	8.90E-09	3.04E-06	6.381823	14	77291879	TSS1500	-871	*LRRC74A*	*LRRC74A*
cg07533617	8.90E-09	3.04E-06	6.381759	11	1.18E+08	Body;Body;Body	43005	*DSCAML1*	*DSCAML1*
cg04592706	8.93E-09	3.04E-06	6.381231	1	94586592	1stExon	112	*ABCA4*	*ABCA4*
cg06942978	8.93E-09	3.04E-06	6.381172	5	1266595	Body;Body;Body;Body	28588	*TERT*	*TERT*
cg12182971	8.95E-09	3.05E-06	6.380534	6	1.66E+08	Body;Body	-44625	*LINC00473*	*LINC00602*
cg10577713	8.95E-09	3.05E-06	6.380515	1	2.04E+08	Body	-20468	*PIK3C2B*	*PPP1R15B*
cg09108073	8.97E-09	3.05E-06	6.380138	16	65077778	Body;Body;Body	78188	*CDH11*	*CDH11*
cg22601877	8.98E-09	3.05E-06	6.379789	8	1.45E+08	Body;Body;Body;Body;Body;Body;Body;Body	21689	*PLEC*	*PLEC*
cg27557525	8.99E-09	3.05E-06	6.379689	2	73215621	Body;Body;Body;Body;Body;Body;Body;Body;Body;Body;Body;Body;Body;Body;Body;Body;Body;Body;Body;Body;Body	70680	*SFXN5*	*SFXN5*
cg19240938	9.03E-09	3.06E-06	6.378676	11	2942036	Body;Body;Body;Body	8614	*SLC22A18*	*PHLDA2*
cg25426044	9.12E-09	3.09E-06	6.376368	8	95254202		20345		*GEM*
cg08971667	9.12E-09	3.09E-06	6.376208	7	27201728	TES5000;TES5000	3428	*HOXA9;HOXA10-HOXA9*	*HOXA9*
cg11902408	9.17E-09	3.10E-06	6.37507	3	14859299	TSS1500;TSS1500	-1169	*FGD5*	*FGD5*
cg03531810	9.21E-09	3.11E-06	6.374179	8	1.44E+08	Body	651	*LY6D*	*LY6D*
cg18454510	9.22E-09	3.11E-06	6.373863	6	40363184	Body	17109	*LRFN2*	*TDRG1*
cg05959989	9.23E-09	3.11E-06	6.373603	11	68517164	Body;Body;TES5000	1824	*TESMIN;CPT1A*	*TESMIN*
cg08705882	9.23E-09	3.11E-06	6.373524	10	94337576	TSS5000;TSS5000;TSS5000;TSS5000;TSS5000;TSS5000	-3724	*IDE*	*IDE*
cg03847541	9.24E-09	3.11E-06	6.373452	1	55752514		61201		*MIR4422*
cg01209118	9.25E-09	3.11E-06	6.373163	6	1.08E+08	TSS5000;Body;Body	-1674	*SCML4*	*SCML4*
cg00504285	9.25E-09	3.11E-06	6.373082	22	20019887	Body;Body;Body;Body;Body;Body;Body;Body;Body;Body;Body;Body;Body;Body;Body;Body;Body;Body;Body;Body;Body;Body;Body;Body;Body;Body;Body;Body;Body;Body;Body;Body;Body;Body;Body;Body;Body;Body;Body;TSS1500	-774	*TANGO2;MIR185*	*MIR185*
cg19754180	9.28E-09	3.11E-06	6.372481	5	1362700	TES5000	-17486	*LINC01511*	*CLPTM1L*
cg02447659	9.28E-09	3.11E-06	6.372311	2	45537078		-54998		*LINC01121*
cg10469297	9.28E-09	3.11E-06	6.372305	11	63871243	Body;TSS200	-118	*MACROD1;FLRT1*	*FLRT1*
cg13985252	9.29E-09	3.11E-06	6.37218	19	43918371	Body;TSS1500	-670	*TEX101*	*TEX101*
cg10614856	9.29E-09	3.11E-06	6.372139	19	50025115	Body;Body	-6144	*FCGRT*	*RCN3*
cg02214441	9.29E-09	3.11E-06	6.372033	22	31536996	TSS1500	-502	*PLA2G3*	*PLA2G3*
cg23573510	9.30E-09	3.11E-06	6.371832	6	33716103	TSS1500;TSS1500	-1341	*IP6K3*	*IP6K3*
cg09763347	9.30E-09	3.11E-06	6.371827	8	1.34E+08	TSS1500	-284	*HPYR1*	*HPYR1*
cg18892790	9.31E-09	3.11E-06	6.371616	22	25819979		-24074		*CRYBB2P1*
cg14209870	9.31E-09	3.11E-06	6.37156	7	1.41E+08	TSS1500	-622	*TAS2R5*	*TAS2R5*
cg14837598	9.33E-09	3.11E-06	6.371276	8	21916635	Body;Body;Body;Body;Body;Body;Body;Body;Body;Body;Body;Body;Body;Body;Body;Body;Body;Body;Body;Body;Body;Body;5'UTR;5'UTR;5'UTR;5'UTR;5'UTR;5'UTR	45	*DMTN*	*DMTN*
cg07560932	9.33E-09	3.11E-06	6.371254	20	61598002	Body;Body	13942	*SLC17A9*	*SLC17A9*
cg15618336	9.34E-09	3.11E-06	6.370974	12	52182759	Body;Body;Body;Body	42942	*SCN8A*	*FIGNL2*
cg03660788	9.36E-09	3.12E-06	6.370498	5	1.16E+08	Body;Body;TSS1500	-279	*SEMA6A;SEMA6A-AS1*	*SEMA6A-AS1*
cg21600380	9.37E-09	3.12E-06	6.370186	8	1.44E+08		-19693		*LY6D*
cg18431572	9.37E-09	3.12E-06	6.370087	2	85329830		-30684		*TCF7L1*
cg14201424	9.43E-09	3.13E-06	-6.36884	4	1.83E+08	Body;Body	2271	*TENM3-AS1*	*TENM3-AS1*
cg10142863	9.44E-09	3.14E-06	6.368414	8	43405739		258155		*POTEA*
cg06477487	9.51E-09	3.15E-06	6.366853	6	33286558	TSS5000;TSS5000;TSS5000;TSS1500;TSS1500;Body;Body;Body;Body;TES5000	-839	*TAPBP;ZBTB22;DAXX;SMIM40*	*ZBTB22*
cg24343430	9.51E-09	3.15E-06	6.366762	8	1.17E+08		-119814		*LINC00536*
cg24349804	9.51E-09	3.15E-06	6.366714	17	79400831	Body	17383	*BAHCC1*	*MIR3186*
cg17599241	9.52E-09	3.15E-06	6.366627	5	82858676	Body;Body;Body;Body;TSS1500	-543	*VCAN;VCAN-AS1*	*VCAN-AS1*
cg22450927	9.63E-09	3.18E-06	6.364037	1	11713238	Body;TSS1500;TSS1500;TSS5000;TSS5000;TSS5000;TSS5000;TSS5000;TSS5000	-1225	*FBXO2;FBXO44*	*FBXO44*
cg09820557	9.65E-09	3.19E-06	6.363469	17	8249349	3'UTR;3'UTR	6192	*ODF4*	*ODF4*
cg08774694	9.66E-09	3.19E-06	6.363377	16	75287019	TSS5000;TSS1500;TSS1500;Body;Body;Body;Body;Body	-1497	*BCAR1*	*BCAR1*

Probe	p_adj	q_val	t	Chr	Pos.hg19	locations	dist2TSS	gene.Illumina	gene.nearest
cg18641937	9.66E-09	3.19E-06	6.363326	1	1.61E+08	TES5000;Body	210	*LOC101928372;ITLN2*	*ITLN2*
cg18113181	9.70E-09	3.20E-06	6.362312	3	66004205	Body;Body;Body;Body;Body;Body	20388	*MAGI1*	*MAGI1*
cg05218653	9.70E-09	3.20E-06	6.362231	16	2334964	Body	10344	*ABCA3*	*MIR4717*
cg27459437	9.74E-09	3.21E-06	6.36145	20	37464594	1stExon;1stExon	30255	*PPP1R16B*	*PPP1R16B*
cg07189342	9.77E-09	3.21E-06	6.360788	4	89619420	Body;Body;TSS1500	-440	*HERC3;NAP1L5*	*NAP1L5*
cg10455413	9.78E-09	3.22E-06	6.360412	2	2.36E+08	Body;Body;Body	-26039	*AGAP1*	*TNRC17*
cg18165630	9.80E-09	3.22E-06	6.360095	8	43061532	TES5000;TES5000;TES5000;TES5000	65926	*HGSNAT*	*HGSNAT*
cg27582180	9.82E-09	3.22E-06	6.35955	5	35852286	TSS5000;TSS5000	-4706	*IL7R*	*IL7R*
cg03727169	9.84E-09	3.23E-06	6.358973	10	31418969		-57842		*LINC02664*
cg19886978	9.93E-09	3.26E-06	6.35694	15	63967183	Body	-74157	*HERC1*	*USP3-AS1*
cg26636379	9.99E-09	3.27E-06	6.355575	11	1.29E+08	Body;TSS200;Body;Body	-174	*TP53AIP1*	*TP53AIP1*
cg11755933	1.00E-08	3.28E-06	6.355205	11	67266128	Body;Body	6706	*PITPNM1*	*PITPNM1*
cg05491762	1.00E-08	3.28E-06	6.355095	10	99328970	Body;TSS5000;TSS5000;TSS5000;TSS5000;TSS5000	-3227	*UBTD1;ANKRD2*	*ANKRD2*
cg00507823	1.01E-08	3.29E-06	6.353875	16	71894108	TES5000;Body;Body;Body;Body;3'UTR;Body;Body	14230	*ATXN1L;ZNF821*	*ATXN1L*
cg06086073	1.01E-08	3.29E-06	6.353756	6	28549832	Body;Body	5243	*ZBED9*	*ZBED9*
cg26332285	1.01E-08	3.30E-06	6.353209	10	1.35E+08	Body;TSS200	-3	*ADGRA1*	*ADGRA1*
cg02255892	1.01E-08	3.30E-06	6.353068	17	42908915	TSS1500;TSS1500	-736	*GJC1*	*GJC1*
cg06793849	1.01E-08	3.30E-06	6.35276	5	1.68E+08	Body;Body;Body;Body	22032	*TENM2*	*MIR12125*
cg08338368	1.01E-08	3.30E-06	6.352382	12	68620926	TSS1500	-1336	*IL26*	*IL26*
cg12247164	1.01E-08	3.30E-06	6.352317	8	10005590	Body;Body;Body;Body	52430	*MSRA*	*MSRA*
cg19113840	1.01E-08	3.30E-06	6.352302	10	99375205	TES5000;TES5000;3'UTR;3'UTR	18052	*HOGA1;MORN4*	*MORN4*
cg08436467	1.02E-08	3.30E-06	6.351747	20	3024166	TES5000;TES5000;TES5000;TSS200;TSS200;TSS200;TSS200;TSS5000	-101	*PTPRA;GNRH2;MRPS26*	*GNRH2*
cg08846327	1.02E-08	3.32E-06	6.350118	5	14359614	Body;Body	-104477	*TRIO*	*SNORD170*
cg04626868	1.03E-08	3.32E-06	6.34968	12	23999034	Body;Body;Body;Body;Body	103570	*SOX5*	*SOX5*
cg18778658	1.03E-08	3.32E-06	6.349638	6	31854450	Body;Body;Body;Body;Body	-7627	*EHMT2*	*SLC44A4*
cg05124770	1.03E-08	3.32E-06	6.349238	22	24578799	TES5000;TES5000;TES5000;Body	1345	*CABIN1;SUSD2*	*SUSD2*
cg08219991	1.03E-08	3.32E-06	6.349228	14	1E+08	Body	15612	*CCDC85C*	*CCDC85C*
cg02314394	1.03E-08	3.32E-06	6.349187	5	646139	Body;Body	33685	*CEP72*	*CEP72*
cg11708784	1.03E-08	3.32E-06	6.349179	1	26012082	Body;Body	68124	*MAN1C1*	*MAN1C1*
cg21308760	1.03E-08	3.32E-06	6.348905	7	1.42E+08	3'UTR;3'UTR	11185	*CLEC5A*	*OR9A4*
cg26495867	1.03E-08	3.32E-06	6.348597	5	1.32E+08	TES5000	14353	*IRF1-AS1*	*IRF1*
cg05422096	1.03E-08	3.32E-06	6.348582	17	54230560	TSS1500;TSS1500;TSS1500	-275	*ANKFN1*	*ANKFN1*
cg10399899	1.03E-08	3.32E-06	6.348505	20	62705081	TES5000;TES5000;3'UTR;3'UTR;TES5000	3282	*TCEA2;RGS19;MIR6813*	*MIR6813*
cg18616385	1.03E-08	3.32E-06	6.348239	5	76377018	Body;Body;TSS1500;TES5000	-622	*ZBED3;SNORA47*	*SNORA47*
cg01512113	1.03E-08	3.32E-06	6.347707	1	1680232	TSS5000;TSS5000;TES5000;TES5000;TES5000;TES5000;TES5000;TES5000	-2794	*SLC35E2A;NADK*	*SLC35E2A*
cg05188488	1.03E-08	3.32E-06	6.347683	11	36670361	Body;Body;Body;Body;Body;Body;Body;Body	-50532	*C11orf74*	*RAG2*
cg07118811	1.03E-08	3.32E-06	6.347679	9	1.32E+08	TSS1500	-1108	*C9orf106*	*C9orf106*
cg01962471	1.03E-08	3.32E-06	6.347668	6	1.58E+08	Body;Body	-73227	*ZDHHC14*	*MIR3692*
cg12517318	1.04E-08	3.32E-06	6.347533	4	529948	Body;Body;Body;Body;Body;Body;Body;Body;Body;Body;Body;Body;Body;Body;Body;Body;Body;Body;Body;Body	36950	*PIGG*	*PIGG*
cg08951891	1.04E-08	3.32E-06	6.347466	11	67222784	TSS5000;Body;TSS200;TSS200	-40	*GPR152;CABP4*	*CABP4*
cg04247530	1.04E-08	3.33E-06	6.34702	8	1.45E+08	Body;Body;Body;Body;Body;Body;Body;Body	16410	*PLEC*	*PLEC*
cg00635382	1.04E-08	3.33E-06	6.346507	8	1.44E+08	TSS1500	-734	*SLURP1*	*SLURP1*
cg13734833	1.04E-08	3.33E-06	6.346375	11	68779817	Body;Body;TSS200	-4	*MRGPRF;MRGPRF-AS1*	*MRGPRF-AS1*
cg08956101	1.04E-08	3.34E-06	6.345944	7	14543945	Body;Body;Body;Body;Body;Body;Body;Body;Body;Body;Body;Body;Body;Body;Body;Body;Body;Body;Body	337130	*DGKB*	*DGKB*
cg20788149	1.04E-08	3.34E-06	6.345445	1	2.03E+08	TES5000;TES5000;TES5000;TES5000;TES5000;TES5000;TES5000;TES5000;TES5000;TES5000;TES5000;TES5000;TES5000;TES5000;TES5000;TES5000;TES5000;TES5000;Body;Body	2741	*TMEM183A;PPFIA4*	*PPFIA4*
cg18181787	1.05E-08	3.34E-06	6.345242	17	32627486		14726		*CCL11*
cg24744721	1.05E-08	3.34E-06	6.345107	17	78896969	Body;Body	48044	*RPTOR*	*LOC400627*
cg00777926	1.05E-08	3.34E-06	6.344683	13	20807375	TSS5000;TSS1500;TSS1500;TSS1500;TSS1500;TSS1500;TSS1500	-841	*GJB6*	*GJB6*
cg00382919	1.05E-08	3.35E-06	6.343823	12	68659823		-12439		*IL22*
cg07473985	1.05E-08	3.36E-06	6.343302	20	23584367	Body	2243	*CST9*	*CST9*
cg20333904	1.06E-08	3.37E-06	6.34241	2	2.41E+08	TES5000	39612	*LOC150935*	*LOC150935*
cg04549418	1.06E-08	3.37E-06	6.34232	1	94102054	Body;Body;Body	-22400	*BCAR3*	*BCAR3*
cg13329242	1.06E-08	3.37E-06	6.342048	11	1.17E+08	Body;Body	184190	*DSCAML1*	*CEP164*
cg09531376	1.06E-08	3.37E-06	6.342003	11	1.33E+08	Body;Body	168323	*OPCML*	*OPCML*
cg07343122	1.06E-08	3.37E-06	6.341794	3	46000900	Body	15945	*FYCO1*	*CXCR6*
cg00794381	1.06E-08	3.37E-06	6.341665	1	1.71E+08	3'UTR;3'UTR;3'UTR;3'UTR	27742	*FMO1*	*FMO1*
cg01260070	1.06E-08	3.37E-06	6.341251	22	44602827	3'UTR;3'UTR	26023	*PARVG*	*PARVG*
cg06241915	1.07E-08	3.38E-06	6.340601	8	95106865		113918		*CDH17*
cg16975888	1.08E-08	3.40E-06	6.338992	4	83722528	TSS5000;TSS5000	-2518	*SCD5*	*SCD5*

Probe	p_adj	q_val	t	Chr	Pos.hg19	locations	dist2TSS	gene.Illumina	gene.nearest
cg01389283	1.08E-08	3.41E-06	6.33851	11	5539372	TSS1500;TES5000	-1443	*UBQLNL;OLFM5P*	*UBQLNL*
cg16413428	1.08E-08	3.41E-06	6.338291	4	6114627	5'UTR;5'UTR;5'UTR;5'UTR	81719	*JAKMIP1*	*JAKMIP1*
cg05753629	1.08E-08	3.41E-06	6.33823	1	43535050		-78524		*FAM183A*
cg02525975	1.09E-08	3.43E-06	6.336617	6	1.1E+08	Body	-14796	*AK9*	*ZBTB24*
cg23092788	1.09E-08	3.43E-06	6.336161	4	949558	Body;Body;Body;Body;Body;Body;Body;TES5000	17799	*TMEM175;DGKQ*	*DGKQ*
cg25819219	1.09E-08	3.43E-06	6.336147	3	73112133	Body;Body;Body;Body;3'UTR	1324	*PPP4R2;EBLN2*	*EBLN2*
cg18557145	1.09E-08	3.43E-06	6.335765	9	35618083	Body	327	*CD72*	*CD72*
cg25143794	1.09E-08	3.44E-06	6.335339	11	1.23E+08	Body;Body;Body;Body	-15803	*GRAMD1B*	*GRAMD1B*
cg17627813	1.09E-08	3.44E-06	6.335084	11	1.3E+08	TSS1500;TSS1500;TSS1500;TSS1500;Body;Body;Body;Body;Body;Body;Body;Body;Body;Body	-1039	*PRDM10*	*PRDM10*
cg05813544	1.09E-08	3.44E-06	6.334815	14	1.05E+08	Body;Body	24433	*CEP170B*	*MIR12121*
cg21367783	1.10E-08	3.44E-06	6.334748	7	4775278	Body	-39975	*FOXK1*	*AP5Z1*
cg11524128	1.10E-08	3.44E-06	6.334722	2	1.28E+08	Body;Body;Body	12618	*CYP27C1*	*CYP27C1*
cg20681184	1.10E-08	3.44E-06	6.334663	1	9750813	Body;TSS5000;TSS5000;TSS5000;Body;TSS1500	-711	*PIK3CD;PIK3CD-AS2*	*PIK3CD*
cg02976539	1.10E-08	3.45E-06	6.333659	17	72757302	Body	12536	*SLC9A3R1*	*SLC9A3R1*
cg13751265	1.10E-08	3.45E-06	6.333042	17	72947651	TES5000;TES5000;3'UTR	15659	*OTOP3;HID1*	*OTOP3*
cg05886789	1.10E-08	3.45E-06	6.332968	10	20122964	Body;Body	17604	*PLXDC2*	*PLXDC2*
cg10507754	1.11E-08	3.47E-06	6.331266	15	99504358	3'UTR;3'UTR	44527	*IGF1R*	*PGPEP1L*
cg18278817	1.11E-08	3.47E-06	6.331192	7	1.59E+08	TSS5000;Body;Body;Body	-4108	*VIPR2*	*VIPR2*
cg06788856	1.11E-08	3.47E-06	6.331117	17	1821969		88671		*RPA1*
cg04494844	1.11E-08	3.47E-06	6.331049	7	70257926	3'UTR;3'UTR	-339203	*AUTS2*	*GALNT17*
cg15356320	1.11E-08	3.47E-06	6.330828	2	1.28E+08	Body;Body;Body;Body;Body;Body;Body;Body;Body;Body;Body;Body;Body;Body;Body;Body	21641	*BIN1*	*BIN1*
cg04217539	1.11E-08	3.47E-06	6.330711	10	1.32E+08	Body;TES5000	1499	*EBF3;MIR4297*	*MIR4297*
cg07701909	1.12E-08	3.48E-06	6.330353	1	1.16E+08		-85381		*VANGL1*
cg08758352	1.12E-08	3.49E-06	6.32927	17	46205147	TES5000	20201	*SNX11*	*SNX11*
cg01644385	1.12E-08	3.50E-06	6.328871	21	45930044	Body;Body;Body	4118	*TSPEAR;TSPEAR-AS1*	*TSPEAR-AS1*
cg26226487	1.13E-08	3.50E-06	6.328649	4	80329486	TSS200	-104	*GK2*	*GK2*
cg22870661	1.13E-08	3.50E-06	6.327963	3	57144290	Body;5'UTR	-30994	*IL17RD*	*ARHGEF3*
cg07522190	1.13E-08	3.50E-06	6.327776	16	28925877	Body	10682	*RABEP2*	*RABEP2*
cg01169396	1.13E-08	3.50E-06	6.327715	5	1.79E+08		-38003		*TBC1D9B*
cg24285942	1.13E-08	3.51E-06	6.327469	11	64361020	Body;Body;Body;Body	2329	*SLC22A12*	*SLC22A12*
cg06073141	1.13E-08	3.51E-06	6.327124	3	1.9E+08	5'UTR	102	*CLDN16*	*CLDN16*
cg06883868	1.14E-08	3.51E-06	6.326527	18	13643516	Body;Body;Body;Body;Body;Body	1760	*LDLRAD4*	*LDLRAD4*
cg14996490	1.14E-08	3.51E-06	6.326439	6	32171796	Body;Body;Body	-8496	*NOTCH4*	*GPSM3*
cg02733444	1.14E-08	3.52E-06	6.325922	10	1.35E+08	Body;Body	41360	*INPP5A*	*INPP5A*
cg07415530	1.14E-08	3.52E-06	6.325898	18	51823343	3'UTR;3'UTR;3'UTR;3'UTR;Body;Body;3'UTR;3'UTR;3'UTR;3'UTR;3'UTR;3'UTR;3'UTR;3'UTR;3'UTR	27400	*POLI*	*POLI*
cg10357368	1.14E-08	3.53E-06	6.325181	13	1.09E+08		133073		*TNFSF13B*
cg20258934	1.15E-08	3.54E-06	6.323985	7	4119142	Body	-50174	*SDK1*	*SDK1*
cg07798386	1.15E-08	3.55E-06	6.323623	19	12874033	TES5000;3'UTR;3'UTR	11429	*BEST2;HOOK2*	*BEST2*
cg06942124	1.16E-08	3.56E-06	6.322566	1	66899771		79554		*PDE4B*
cg23209962	1.16E-08	3.58E-06	6.321184	5	73163484	Body;Body;Body	54114	*ARHGEF28*	*ARHGEF28*
cg03263716	1.16E-08	3.58E-06	6.320826	11	64435066	Body;Body	-24049	*NRXN2*	*NRXN2*
cg24354958	1.17E-08	3.58E-06	6.32056	8	49954840		-12054		*PPDPFL*
cg03404726	1.17E-08	3.58E-06	6.320416	10	76818754	TSS1500	-413	*DUPD1*	*DUPD1*
cg12342451	1.17E-08	3.59E-06	6.319885	10	86001509	TSS1500;TSS5000;TSS5000;TSS5000	-207	*LRIT1;RGR*	*LRIT1*
cg19140548	1.17E-08	3.59E-06	6.319857	10	1.06E+08	Body	45870	*SH3PXD2A*	*SH3PXD2A-AS1*
cg01130922	1.17E-08	3.59E-06	6.319692	7	552776	Body;Body	6705	*PDGFA*	*PDGFA*
cg12614113	1.17E-08	3.59E-06	6.319408	10	79133935	Body;Body;Body;Body;Body;Body;Body;Body;Body;Body;Body;Body;Body	23306	*KCNMA1;KCNMA1-AS3*	*KCNMA1-AS3*
cg05719052	1.17E-08	3.59E-06	6.31899	17	3561497	Body;Body;TES5000;TES5000;TES5000	10375	*CTNS;TAX1BP3;P2RX5-TAX1BP3*	*TAX1BP3*
cg06371044	1.17E-08	3.59E-06	6.318939	8	1.45E+08	TSS5000;TSS5000	-1748	*NRBP2;MIR6845*	*NRBP2*
cg02485236	1.17E-08	3.59E-06	6.31881	16	67067224	Body;Body;Body;Body;Body;Body	4173	*CBFB*	*CBFB*
cg08205236	1.18E-08	3.59E-06	6.318621	11	68776740	Body;Body;TSS5000	-3081	*MRGPRF;MRGPRF-AS1*	*MRGPRF-AS1*
cg26091981	1.18E-08	3.60E-06	6.318217	2	1.37E+08	TSS200;TES5000	-112	*LCT;MCM6*	*LCT*
cg08033838	1.18E-08	3.60E-06	6.317959	14	78378994	Body;Body;Body;Body;Body;Body;Body;Body;Body;Body;Body	112543	*ADCK1*	*ADCK1*
cg07170302	1.18E-08	3.60E-06	6.317574	11	1.18E+08	TSS200;Body;Body;Body	-34	*FXYD2;FXYD6-FXYD2*	*FXYD2*
cg21079719	1.18E-08	3.60E-06	6.31719	15	98568785	TES5000;TES5000;TES5000	22518	*LINC02251*	*LINC02251*
cg04768315	1.18E-08	3.61E-06	6.316947	2	1898518	Body;Body;Body;Body;Body;Body;Body;Body;Body;Body	-150231	*MYT1L*	*PXDN*
cg17151621	1.19E-08	3.61E-06	6.316461	3	1.86E+08	TSS1500;TSS1500;TSS1500;TSS1500	-781	*AHSG*	*AHSG*
cg19911494	1.19E-08	3.61E-06	6.316252	5	9169758	Body	-115751	*SEMA5A*	*MIR4636*
cg14455403	1.19E-08	3.62E-06	6.31519	3	8596772	Body;TES5000;Body;Body	-16695	*LMCD1*	*LINC00312*
cg09130091	1.19E-08	3.62E-06	6.315158	6	31838613	Body;Body;Body	6835	*SLC44A4*	*SLC44A4*

Probe	p_adj	q_val	t	Chr	Pos.hg19	locations	dist2TSS	gene.Illumina	gene.nearest
cg16056219	1.19E-08	3.62E-06	6.315023	14	75043777	Body	35163	*LTBP2*	*LTBP2*
cg14222990	1.20E-08	3.63E-06	6.314559	9	1.4E+08	Body;Body	26078	*PNPLA7*	*PNPLA7*
cg10800250	1.20E-08	3.63E-06	6.314506	8	10470790	Body	41863	*RP1L1*	*RP1L1*
cg25691026	1.20E-08	3.63E-06	6.314161	19	8670586	1stExon	5014	*ADAMTS10*	*ADAMTS10*
cg26493247	1.20E-08	3.63E-06	6.314109	16	14029535	Body	15525	*ERCC4*	*ERCC4*
cg10682957	1.20E-08	3.63E-06	6.314006	11	1.18E+08		-8340		*FXYD6*
cg14168269	1.20E-08	3.63E-06	6.313999	9	1.32E+08		-45569		*IER5L*
cg15545316	1.20E-08	3.63E-06	6.313824	1	42618970	TSS200	-110	*GUCA2B*	*GUCA2B*
cg13300744	1.21E-08	3.64E-06	6.312868	12	1.12E+08	Body;TSS1500	-1143	*CUX2*	*CUX2*
cg25433648	1.21E-08	3.64E-06	6.312762	1	1.54E+08	TSS5000;TSS5000;5'UTR;TES5000;TES5000;TES5000;TES5000;TES5000	19	*S100A16;S100A14;S100A13*	*S100A14*
cg23944586	1.21E-08	3.64E-06	6.312453	11	19598794	Body;TES5000	1938	*NAV2;MIR4486*	*MIR4486*
cg20490175	1.21E-08	3.64E-06	6.312325	12	2797745	Body;Body;Body;Body;Body;Body;Body;Body;Body;Body;Body;Body;Body;Body;Body;Body;Body;Body;Body;Body;Body;Body;Body;Body	2578	*CACNA1C;CACNA1C-AS1*	*CACNA1C-AS1*
cg17680303	1.21E-08	3.64E-06	6.312155	4	47183921	Body	150338	*GABRB1*	*GABRB1*
cg09973663	1.21E-08	3.65E-06	6.311642	2	1.92E+08	Body;Body;Body;Body;Body	5039	*MYO1B*	*MYO1B*
cg16270472	1.21E-08	3.65E-06	6.311577	12	2885744	Body	-18391	*ITFG2-AS1*	*FKBP4*
cg14410198	1.22E-08	3.66E-06	6.310734	9	37424897	Body	2205	*GRHPR*	*GRHPR*
cg07163991	1.22E-08	3.66E-06	6.310565	2	1.28E+08	Body	-21603	*MYO7B*	*GPR17*
cg15367378	1.23E-08	3.68E-06	6.309029	21	38366369	TSS5000;TSS5000;TSS5000	-3858	*HLCS*	*HLCS*
cg11935831	1.23E-08	3.68E-06	6.30897	17	80137075	Body;Body;Body	33643	*CCDC57*	*CCDC57*
cg27548752	1.23E-08	3.68E-06	6.308776	19	47263614	TES5000;TES5000	-13907	*FKRP*	*STRN4*
cg25836545	1.24E-08	3.70E-06	6.307217	15	45426386	TSS5000;TSS5000;TSS5000;TSS5000;Body;Body	4195	*DUOXA1;DUOX1*	*DUOX1*
cg25561792	1.24E-08	3.71E-06	6.306296	17	62050205	5'UTR	73	*SCN4A*	*SCN4A*
cg15695435	1.24E-08	3.72E-06	6.305935	12	1.22E+08	Body;Body	-27310	*KDM2B*	*MIR7107*
cg22246344	1.24E-08	3.72E-06	6.305825	2	45183790		-14773		*SIX3-AS1*
cg14034270	1.25E-08	3.72E-06	6.305387	14	1.01E+08	TSS1500;TSS1500;TSS1500;TSS1500;TSS1500;TSS1500;TSS1500;TSS1500;TSS1500;TSS1500;TSS1500;TSS1500;TSS1500;TSS1500;TSS1500	-1344	*MEG3*	*MEG3*
cg01347702	1.25E-08	3.72E-06	6.305214	6	31845672	TSS1500;Body;Body;TES5000;TES5000;TES5000;TES5000;TES5000	-224	*SLC44A4;EHMT2*	*SLC44A4*
cg18889973	1.25E-08	3.72E-06	6.30515	7	1.58E+08	Body;Body;Body;Body;Body	310985	*PTPRN2*	*LOC100506585*
cg25644015	1.25E-08	3.73E-06	6.304481	6	31848099	TSS5000;TSS1500;TSS1500;Body;Body;Body;Body;Body	-1276	*SLC44A4;EHMT2*	*SLC44A4*
cg09226319	1.25E-08	3.73E-06	6.304096	4	74698986	TSS5000	-3408	*CXCL6*	*CXCL6*
cg06440665	1.26E-08	3.73E-06	6.303678	1	32133300	Body	-22823	*COL16A1*	*PEF1*
cg17881037	1.26E-08	3.73E-06	6.303596	7	1.53E+08		258035		*ACTR3B*
cg16192029	1.26E-08	3.74E-06	6.303	7	1.18E+08	TSS200	-83	*ANKRD7*	*ANKRD7*
cg06713968	1.26E-08	3.75E-06	6.302629	2	47089970	TES5000	34968	*LINC01119*	*LINC01119*
cg05922453	1.26E-08	3.75E-06	6.302481	16	50425414	Body	3630	*LINC02178*	*LINC02178*
cg18522715	1.26E-08	3.75E-06	6.301989	7	1.58E+08	Body;Body;Body;Body;Body	26547	*PTPRN2*	*MIR595*
cg18237398	1.27E-08	3.75E-06	6.301911	1	22645936		53205		*MIR4418*
cg06520450	1.27E-08	3.75E-06	6.301778	3	1.9E+08	Body;Body;Body;Body;Body;Body;TSS200;TSS200;TSS200;TSS200;TSS200;TSS200;TSS200	-51	*TP63*	*TP63*
cg01026196	1.27E-08	3.75E-06	6.301498	9	1.34E+08	Body;Body	15544	*FIBCD1*	*FIBCD1*
cg26494252	1.27E-08	3.75E-06	6.301449	19	15083916	TSS200;Body;Body;Body	-186	*SLC1A6*	*SLC1A6*
cg26886909	1.27E-08	3.75E-06	6.301128	1	1.12E+08	Body;Body;Body;Body;Body;Body;Body;Body	26279	*CHIA*	*CHIA*
cg26367275	1.27E-08	3.75E-06	6.301049	1	11596950	3'UTR	57739	*DISP3*	*DISP3*
cg01187498	1.27E-08	3.76E-06	6.300298	5	1.8E+08		-7613		*RASGEF1C*
cg08754294	1.27E-08	3.76E-06	6.300282	17	55175274	Body;Body;Body;Body;Body;Body;Body;Body	2425	*AKAP1*	*AKAP1*
cg03478916	1.28E-08	3.76E-06	6.299912	8	22421029	Body;TSS5000	-2212	*SORBS3*	*SORBS3*
cg22563937	1.28E-08	3.77E-06	6.299623	13	1.08E+08		324267		*SNORD31B*
cg12583367	1.28E-08	3.77E-06	6.298943	4	4412229	Body;Body;Body;Body	23540	*NSG1*	*NSG1*
cg21582785	1.28E-08	3.77E-06	6.298851	7	3982778	Body	-186538	*SDK1*	*SDK1*
cg01214573	1.29E-08	3.78E-06	6.298149	15	49257931	TSS5000	-1815	*SHC4*	*SHC4*
cg09354317	1.29E-08	3.78E-06	6.297921	3	52869307	TSS5000;TSS5000;TSS1500;Body;TES5000	-272	*ITIH4;MUSTN1;STIMATE-MUSTN1;STIMATE*	*MUSTN1*
cg23531317	1.29E-08	3.78E-06	6.297799	15	41193043	Body	-5963	*VPS18*	*LOC105370943*
cg06177446	1.29E-08	3.78E-06	6.297674	20	1093802	TSS200;TSS200;TES5000	-69	*PSMF1;LOC105372493*	*PSMF1*
cg05362892	1.29E-08	3.79E-06	6.297329	11	68583422	Body;Body	25962	*CPT1A*	*CPT1A*
cg19628225	1.29E-08	3.79E-06	6.297117	1	20005822	Body;TES5000;TES5000;TES5000;TES5000	14455	*HTR6;TMCO4*	*HTR6*
cg07533384	1.29E-08	3.79E-06	6.296954	13	1.13E+08	Body;Body	98756	*TUBGCP3*	*TUBGCP3*
cg10427868	1.29E-08	3.79E-06	6.296839	10	80936798	Body	108076	*ZMIZ1*	*ZMIZ1*
cg27243405	1.30E-08	3.79E-06	6.296483	6	1.39E+08	Body	34130	*NHSL1*	*NHSL1*
cg02662873	1.30E-08	3.79E-06	6.296257	6	1.69E+08	Body;Body	88328	*SMOC2*	*SMOC2*
cg21778654	1.30E-08	3.80E-06	6.295307	2	37997383		59471		*LINC00211*

Probe	p_adj	q_val	t	Chr	Pos.hg19	locations	dist2TSS	gene.Illumina	gene.nearest
cg26405841	1.30E-08	3.80E-06	6.295231	14	96715970		-6576		*BDKRB1*
cg07925867	1.30E-08	3.80E-06	6.295064	16	66953432	TSS1500;TSS1500;TSS1500;TSS1500;TES5000;TES5000	-644	*CDH16;RRAD*	*CDH16*
cg12470367	1.30E-08	3.80E-06	6.294908	6	30137056	Body	5734	*TRIM15*	*TRIM15*
cg26239816	1.31E-08	3.80E-06	6.294836	22	25615317	TSS1500	-288	*CRYBB2*	*CRYBB2*
cg24531009	1.31E-08	3.80E-06	6.29471	7	99987853	Body;Body;Body	16744	*PILRA*	*PILRA*
cg08009345	1.31E-08	3.80E-06	6.294694	11	57790202	TSS1500	-1150	*OR9Q1*	*OR9Q1*
cg03390207	1.31E-08	3.80E-06	6.294491	17	27948752	TSS5000;TSS5000;TSS1500;TES5000;TES5000	-311	*CORO6;SSH2*	*CORO6*
cg11468199	1.31E-08	3.80E-06	6.29442	5	1501109	Body	9862	*LPCAT1*	*MIR6075*
cg00329472	1.31E-08	3.82E-06	6.293244	8	23144184	TSS1500;TSS1500;TSS1500	-1420	*R3HCC1*	*R3HCC1*
cg10965117	1.32E-08	3.83E-06	6.292561	13	1.12E+08		-76111		*LINC02337*
cg09608716	1.32E-08	3.83E-06	6.292381	16	88455742	Body	6376	*ZNF469*	*ZNF469*
cg01439119	1.32E-08	3.84E-06	6.291555	12	1.07E+08	3'UTR	-11035	*TCP11L2*	*POLR3B*
cg26730024	1.33E-08	3.84E-06	6.291145	11	36448470	Body;Body;Body;Body	25924	*PRR5L*	*PRR5L*
cg01728951	1.33E-08	3.84E-06	6.290995	17	46228066	Body;Body	5807	*SKAP1*	*MIR1203*
cg08794084	1.33E-08	3.85E-06	6.290232	1	2.29E+08	Body;Body;Body	75430	*OBSCN*	*MIR6742*
cg08440418	1.34E-08	3.87E-06	6.28924	16	88132436	TES5000	2155	*LOC400553*	*LOC400553*
cg05348888	1.34E-08	3.87E-06	6.288911	1	2937278	TSS1500	-752	*ACTRT2*	*ACTRT2*
cg11700630	1.34E-08	3.88E-06	6.288352	17	73855804	TSS5000;TSS5000;TSS5000	-3287	*WBP2*	*WBP2*
cg10206380	1.35E-08	3.88E-06	6.287772	16	57672032	Body;Body;Body;Body;Body;Body;Body;Body;Body;Body;Body;Body;Body;Body;Body;Body;Body;Body;Body;Body;Body;Body;Body;Body;Body;Body;Body;TSS1500;TSS1500	-1371	*ADGRG1*	*ADGRG1*
cg09369294	1.35E-08	3.88E-06	6.28765	17	3483541	Body;Body;Body;Body	12630	*TRPV1*	*TRPV1*
cg07283630	1.35E-08	3.89E-06	6.287098	15	48667436		43043		*DUT*
cg12107580	1.35E-08	3.90E-06	6.286636	8	1.42E+08	Body;TSS200	-50	*PTP4A3*	*PTP4A3*
cg04308167	1.36E-08	3.90E-06	6.286268	17	19440767	Body	3601	*SLC47A1*	*SLC47A1*
cg24465224	1.36E-08	3.90E-06	6.285871	8	1.45E+08	TES5000;TES5000;TES5000;TES5000;TES5000;TES5000;TES5000;TES5000	29166	*PLEC*	*PLEC*
cg04345694	1.36E-08	3.91E-06	6.285359	8	8439596		-119850		*CLDN23*
cg10214132	1.36E-08	3.91E-06	6.285339	19	3814131	Body	-12334	*ZFR2*	*MATK*
cg25220751	1.36E-08	3.91E-06	6.28523	6	4655948		45150		*KU-MEL-3*
cg08592104	1.36E-08	3.91E-06	6.285012	11	8761869	Body;Body;Body	-28455	*DENND2B*	*LOC102724784*
cg27567922	1.36E-08	3.91E-06	6.2849	3	1.95E+08	Body;Body;Body	32788	*MUC4*	*MUC20*
cg11630135	1.36E-08	3.91E-06	6.284644	3	13679166	Body;Body;Body	-13054	*FBLN2*	*LINC00620*
cg02294870	1.37E-08	3.91E-06	6.284563	17	41479663	TES5000	3303	*ARL4D*	*ARL4D*
cg23922724	1.37E-08	3.91E-06	6.284322	11	76474735		-18621		*TSKU*
cg26812481	1.37E-08	3.91E-06	6.284075	1	25976496	Body;Body	32538	*MAN1C1*	*MAN1C1*
cg03395993	1.37E-08	3.92E-06	6.283387	1	2109923	Body;Body;Body;Body;Body;Body;Body;Body;Body;TES5000	5391	*PRKCZ;PRKCZ-AS1*	*PRKCZ-AS1*
cg00474017	1.38E-08	3.93E-06	6.282654	13	1.14E+08	TES5000;TES5000	-5727	*ADPRHL1*	*LOC101928841*
cg09635874	1.38E-08	3.93E-06	6.282559	13	98952518	Body;Body	91741	*FARP1*	*MIR3170*
cg08458055	1.38E-08	3.95E-06	6.281396	13	1.11E+08	Body;Body;Body;Body	33800	*CARS2*	*NAXD*
cg07768378	1.39E-08	3.95E-06	6.281129	5	1.12E+08	Body;Body	67215	*MCC*	*DCP2*
cg10881615	1.39E-08	3.96E-06	6.280015	2	69100119	TSS5000	-1590	*BMP10*	*BMP10*
cg18126802	1.39E-08	3.96E-06	6.279959	1	1.72E+08	Body;Body;Body;Body;Body	-68757	*DNM3*	*C1orf105*
cg26758810	1.39E-08	3.96E-06	6.279945	6	1.49E+08		54258		*SASH1*
cg15261887	1.39E-08	3.96E-06	6.279809	17	17064703	Body;Body;Body	44944	*MPRIP*	*PLD6*
cg19075717	1.40E-08	3.97E-06	6.279351	17	48670940	Body;Body;Body;Body;Body;Body;Body;Body;Body;Body;Body;Body;Body;Body;Body;Body;Body;Body;Body;Body;Body;Body;Body;Body;Body;Body;Body;Body;Body;Body;Body;Body;Body	-31471	*CACNA1G*	*CACNA1G-AS1*
cg26784112	1.40E-08	3.97E-06	6.279035	11	69300874	Body;Body;Body;Body	546	*LINC01488*	*LINC01488*
cg09216666	1.40E-08	3.98E-06	6.278454	1	8436291	Body;Body;Body	47601	*RERE*	*RERE*
cg26665507	1.40E-08	3.98E-06	6.278352	19	3596441	TES5000;Body;Body	10432	*GIPC3;TBXA2R*	*TBXA2R*
cg00079388	1.40E-08	3.98E-06	6.278336	2	1.87E+08	Body	4333	*ZC3H15*	*ZC3H15*
cg00269996	1.40E-08	3.98E-06	6.278227	6	42979961	3'UTR;3'UTR;3'UTR;3'UTR;3'UTR;3'UTR;3'UTR;3'UTR;TSS5000;TSS5000	1723	*PPP2R5D;MEA1;KLHDC3*	*MEA1*
cg20713525	1.41E-08	3.98E-06	6.277789	5	34044727	Body;TSS1500;TSS1500;Body	-1409	*C1QTNF3-AMACR;C1QTNF3*	*C1QTNF3*
cg00696464	1.41E-08	3.98E-06	6.277634	14	24002201	Body;Body;Body	18773	*THTPA;ZFHX2*	*ZFHX2*
cg06496478	1.42E-08	4.00E-06	6.276404	17	44821280	Body;Body	74778	*NSF*	*WNT3*
cg08603449	1.42E-08	4.00E-06	6.27615	8	1.06E+08	Body;Body	-12622	*LRP12*	*MIR548A3*
cg08067023	1.43E-08	4.03E-06	6.27443	16	72118960	3'UTR;3'UTR;3'UTR;3'UTR;3'UTR	8559	*TXNL4B*	*TXNL4B*
cg08690459	1.43E-08	4.04E-06	6.273937	11	47279586	Body;Body;Body;Body;5'UTR;5'UTR	40	*NR1H3*	*NR1H3*
cg02612270	1.43E-08	4.04E-06	6.273861	16	1015103	Body;Body;Body;Body;Body;Body;Body	5881	*LMF1*	*LMF1*
cg26232558	1.43E-08	4.04E-06	6.273606	17	3195808	Body	68	*OR3A1*	*OR3A1*
cg19670885	1.43E-08	4.04E-06	6.273528	4	3345495	Body;Body	-26429	*RGS12*	*RGS12*
cg13303682	1.43E-08	4.04E-06	6.273283	5	56778129	Body	509	*ACTBL2*	*ACTBL2*
cg00868860	1.44E-08	4.04E-06	6.273191	12	55041948	Body;Body	201	*DCD*	*DCD*

Probe	p_adj	q_val	t	Chr	Pos.hg19	locations	dist2TSS	gene.Illumina	gene.nearest
cg00689330	1.44E-08	4.04E-06	6.272841	13	50024281	Body;Body;Body;Body;Body	5872	*SETDB2;SETDB2-PHF11*	*SETDB2-PHF11*
cg27394794	1.44E-08	4.05E-06	6.272168	10	52600370	Body;Body;Body;Body;Body;Body;Body;Body	45017	*A1CF*	*A1CF*
cg17826530	1.44E-08	4.05E-06	6.271788	1	59280290	Body;Body;Body	29468	*LINC01135*	*LINC01135*
cg04147929	1.44E-08	4.05E-06	6.271664	1	2.24E+08	TSS1500	-1301	*CAPN8*	*CAPN8*
cg27361520	1.45E-08	4.07E-06	6.270902	2	1.03E+08	Body	2922	*IL18RAP*	*IL18RAP*
cg23419907	1.46E-08	4.08E-06	6.26992	2	2.2E+08	TES5000;TES5000;TES5000;TES5000;TES5000;TES5000	4603	*DNAJB2;PTPRN;MIR153-1*	*MIR153-1*
cg09400152	1.46E-08	4.09E-06	6.269205	15	83226580	Body;Body;Body;Body;Body;Body;Body;Body;Body;Body;Body;Body;Body;Body;Body;Body;Body	13936	*CPEB1*	*CPEB1*
cg14118523	1.46E-08	4.09E-06	6.269037	2	30629374		-40737		*LCLAT1*
cg23982568	1.47E-08	4.10E-06	6.268419	8	6118526		145543		*LOC100287015*
cg06034255	1.47E-08	4.10E-06	6.268319	10	1.26E+08	Body;Body;Body;Body	44964	*CHST15*	*CHST15*
cg22529396	1.47E-08	4.11E-06	6.267745	2	1.28E+08	TSS200	-191	*MYO7B*	*MYO7B*
cg06643056	1.48E-08	4.12E-06	6.26676	5	1.71E+08	Body	29033	*FGF18*	*FGF18*
cg12430457	1.48E-08	4.12E-06	6.266585	12	79439547	Body;Body;Body;5'UTR	76	*SYT1*	*SYT1*
cg15262728	1.48E-08	4.13E-06	6.266057	2	2.32E+08	Body;Body;Body;Body;Body	4284	*ITM2C*	*ITM2C*
cg13407062	1.49E-08	4.14E-06	6.265406	8	1.44E+08	Body;Body;Body	5619	*TOP1MT*	*TOP1MT*
cg15466222	1.49E-08	4.14E-06	6.264936	6	1.39E+08	TSS1500;TSS5000;TSS5000	-1492	*HEBP2*	*HEBP2*
cg18538751	1.49E-08	4.15E-06	6.264746	2	2.34E+08	TSS1500;Body	-1183	*NGEF;LOC101928881*	*NGEF*
cg00490431	1.49E-08	4.15E-06	6.264375	17	48201664	Body;Body	5860	*SAMD14*	*SAMD14*
cg07297777	1.49E-08	4.15E-06	6.264319	12	56097482	Body;Body;Body;Body;Body	4206	*ITGA7*	*ITGA7*
cg26116525	1.50E-08	4.16E-06	6.26365	11	62371596	TSS5000;TSS5000;Body;Body;Body	-2259	*MTA2;EML3*	*MTA2*
cg19473392	1.50E-08	4.16E-06	6.263207	6	35170850		-10882		*SCUBE3*
cg06014083	1.51E-08	4.19E-06	6.261572	17	81052928	TES5000;TES5000	13907	*METRNL*	*METRNL*
cg18191138	1.52E-08	4.20E-06	6.26067	5	1487638	Body	23333	*LPCAT1*	*MIR6075*
cg18648055	1.52E-08	4.21E-06	6.260287	12	32403282	Body;Body;Body;Body;Body;Body;Body;Body	143502	*BICD1*	*BICD1*
cg09998078	1.52E-08	4.21E-06	6.260217	14	94448493		-5577		*ASB2*
cg06884875	1.52E-08	4.22E-06	6.259516	11	46313051	Body	13839	*CREB3L1*	*CREB3L1*
cg00664454	1.53E-08	4.22E-06	6.259159	16	57149847	Body	23353	*CPNE2*	*CPNE2*
cg01875118	1.54E-08	4.26E-06	6.257064	14	1.05E+08	Body	1341	*LINC00638*	*LINC00638*
cg02914790	1.54E-08	4.26E-06	6.257016	3	1.23E+08	TSS200	-4	*LINC02035*	*LINC02035*
cg08440945	1.54E-08	4.26E-06	6.256615	11	65983863	Body	-40901	*PACS1*	*KLC2*
cg00161450	1.55E-08	4.27E-06	6.255921	2	63849938		33654		*MDH1*
cg26017506	1.55E-08	4.27E-06	6.255708	3	57312779	Body;1stExon;TES5000	201	*ASB14;LOC105377102*	*ASB14*
cg20147164	1.55E-08	4.27E-06	6.255467	2	1879019	Body;Body;Body;Body;Body;Body;Body;Body;Body;Body	-130732	*MYT1L*	*PXDN*
cg20491573	1.55E-08	4.28E-06	6.255291	12	1.31E+08	Body;Body;Body;Body;Body	6785	*RIMBP2*	*RIMBP2*
cg06839777	1.56E-08	4.28E-06	-6.2549	6	1.23E+08	TSS200;TSS200;TSS200	-19	*HSF2*	*HSF2*
cg21369919	1.56E-08	4.29E-06	6.254094	10	21318882	Body;Body	-132351	*NEBL*	*NEBL*
cg05753930	1.56E-08	4.29E-06	6.25398	11	2678450	Body;Body;Body	42778	*KCNQ1;KCNQ1OT1*	*KCNQ1OT1*
cg01180831	1.56E-08	4.29E-06	6.253752	14	89361469		70475		*TTC8*
cg06500476	1.57E-08	4.31E-06	6.252869	4	37584954	Body;TSS1500	-900	*C4orf19*	*C4orf19*
cg26598388	1.57E-08	4.31E-06	6.252584	17	16284909	Body;Body;Body;Body;Body;5'UTR	131	*UBB*	*UBB*
cg17312277	1.57E-08	4.31E-06	6.252525	13	31306018	Body;TSS5000	-3626	*ALOX5AP*	*ALOX5AP*
cg18516609	1.57E-08	4.31E-06	6.252424	10	31747241	Body;Body;Body;Body;Body;Body;Body;Body;Body;Body;Body;Body;Body;Body;Body;Body;Body;Body;Body;Body;Body;Body;Body;Body;Body;Body;Body;Body;Body;Body;Body;Body;Body;Body;Body;Body;Body;Body;Body;Body;Body	-58557	*ZEB1*	*LOC100505502*
cg00851125	1.57E-08	4.31E-06	6.252205	16	539845	Body;Body;Body	14922	*RAB11FIP3*	*RAB11FIP3*
cg06595653	1.58E-08	4.31E-06	6.251918	12	1.31E+08	Body	8774	*FZD10-AS1*	*FZD10-AS1*
cg10192483	1.58E-08	4.31E-06	6.251845	7	76632239	Body	22101	*DTX2P1-UPK3BP1-PMS2P11*	*DTX2P1-UPK3BP1-PMS2P11*
cg13775629	1.58E-08	4.31E-06	6.251632	10	72360448	1stExon;1stExon	2049	*PRF1*	*PRF1*
cg11540815	1.58E-08	4.31E-06	6.251511	15	83368245	Body;Body;Body;Body	10382	*AP3B2*	*AP3B2*
cg14112601	1.58E-08	4.31E-06	6.251361	6	10520874	TSS1500	-693	*GCNT2*	*GCNT2*
cg24615853	1.58E-08	4.31E-06	6.25131	12	30978660		30046		*LINC00941*
cg07653695	1.58E-08	4.32E-06	6.250832	2	1.02E+08		-84314		*RFX8*
cg13779777	1.59E-08	4.32E-06	6.250455	17	79435897	TES5000	-17683	*BAHCC1*	*MIR3186*
cg09859596	1.59E-08	4.32E-06	6.250311	1	21868624	Body;Body;Body;Body;Body	-9138	*ALPL*	*ALPL*
cg19768143	1.59E-08	4.33E-06	6.24991	14	59666747	Body	11363	*DAAM1*	*DAAM1*
cg06773963	1.59E-08	4.34E-06	6.249139	1	9416303	1stExon	63351	*SPSB1*	*SPSB1*
cg01761729	1.60E-08	4.34E-06	6.249006	2	1.28E+08	Body;Body;Body;Body	2517	*LIMS2*	*LIMS2*
cg00523067	1.60E-08	4.34E-06	6.248914	6	1.54E+08	3'UTR;3'UTR;3'UTR;3'UTR;3'UTR;3'UTR;Body;Body;Body;3'UTR;3'UTR	29393	*OPRM1*	*OPRM1*
cg23248351	1.61E-08	4.36E-06	6.247648	8	1.09E+08	TSS5000;TSS5000	-1554	*ANGPT1*	*ANGPT1*
cg06798749	1.61E-08	4.36E-06	6.247448	11	62360527	TSS5000;TSS5000;TES5000;TES5000	-1532	*TUT1;MTA2*	*TUT1*
cg17787990	1.61E-08	4.36E-06	6.247399	17	65632761	Body;Body	-81187	*PITPNC1*	*NOL11*

Probe	p_adj	q_val	t	Chr	Pos.hg19	locations	dist2TSS	gene.Illumina	gene.nearest
cg01910494	1.62E-08	4.39E-06	6.245486	16	89139320		-20939		*ACSF3*
cg23338388	1.63E-08	4.41E-06	6.244279	2	1.1E+08	Body	-45578	*SH3RF3*	*MIR4265*
cg11741115	1.63E-08	4.41E-06	6.244099	1	1.75E+08	TSS1500	-565	*TNN*	*TNN*
cg14653877	1.64E-08	4.43E-06	6.243308	17	74912348	Body;Body;Body	43620	*MGAT5B*	*MGAT5B*
cg21659425	1.64E-08	4.43E-06	6.242918	3	1.26E+08	Body;Body	697	*C3orf22*	*C3orf22*
cg00464796	1.64E-08	4.43E-06	6.242786	15	53852614	Body;Body	-19314	*WDR72*	*WDR72*
cg06112087	1.65E-08	4.44E-06	6.241988	11	18740452	Body;Body	7283	*IGSF22*	*IGSF22*
cg03434029	1.65E-08	4.45E-06	6.241417	1	45274048	TES5000;TSS1500;TSS200	-105	*PLK3;TCTEX1D4;BTBD19*	*BTBD19*
cg03028769	1.65E-08	4.45E-06	6.241198	10	73421791	Body;Body;Body	57744	*CDH23*	*C10orf105*
cg13924015	1.65E-08	4.45E-06	6.241183	9	1.08E+08		-95767		*SLC44A1*
cg10091891	1.66E-08	4.46E-06	6.240506	4	13417329	Body;Body;Body	68635	*RAB28*	*RAB28*
cg08103144	1.66E-08	4.46E-06	6.240278	5	1.5E+08	Body;Body;1stExon;1stExon	8765	*SYNPO*	*SYNPO*
cg03118021	1.66E-08	4.46E-06	6.24018	6	1838155	Body;Body	228006	*GMDS*	*FOXC1*
cg14032734	1.66E-08	4.46E-06	6.240088	9	1.34E+08	3'UTR;3'UTR;3'UTR;3'UTR;3'UTR;3'UTR;3'UTR;3'UTR;3'UTR;3'UTR;3'UTR;3'UTR;3'UTR;Body;Body;Body;Body;Body;Body;Body;Body;Body;Body;TES5000;TES5000;TES5000;TES5000;TES5000;TES5000;TES5000	7956	*POMT1;UCK1*	*UCK1*
cg09954729	1.66E-08	4.46E-06	6.24007	16	2334596	Body	9976	*ABCA3*	*MIR4717*
cg05452391	1.66E-08	4.46E-06	6.239855	5	1.32E+08	Body	34786	*IRF1-AS1*	*IRF1-AS1*
cg16553338	1.66E-08	4.46E-06	6.239788	16	2242886	Body	3720	*CASKIN1*	*CASKIN1*
cg22958018	1.66E-08	4.46E-06	6.239768	7	86795287	Body;Body;Body;Body;Body	13418	*DMTF1*	*DMTF1*
cg02824817	1.66E-08	4.46E-06	6.239689	4	1.7E+08		-73202		*SH3RF1*
cg15273575	1.66E-08	4.46E-06	6.239643	6	1.45E+08	Body	364013	*UTRN*	*SNORA98*
cg07821739	1.67E-08	4.46E-06	6.239134	1	26347541	TSS1500	-724	*EXTL1*	*EXTL1*
cg25848990	1.67E-08	4.47E-06	6.23875	3	11607161	TES5000;Body;Body;Body;Body;Body;Body	3111	*ATG7;VGLL4*	*VGLL4*
cg05516617	1.67E-08	4.47E-06	6.238529	7	27221689	TSS5000;TSS5000;3'UTR;TSS5000	-1809	*HOXA10-HOXA9;HOXA10;HOXA11;HOXA11-AS*	*HOXA10-HOXA9*
cg02886715	1.67E-08	4.47E-06	6.238237	6	44691838		-172220		*LOC105375075*
cg13879195	1.67E-08	4.47E-06	6.238049	3	28886575		269807		*LINC00693*
cg00593212	1.68E-08	4.49E-06	6.237066	8	18250927	Body	2136	*NAT2*	*NAT2*
cg04723679	1.68E-08	4.49E-06	6.236661	15	62548919		-7925		*GOLGA2P11*
cg00622010	1.69E-08	4.50E-06	6.236047	7	30951086	TSS1500;TSS1500	-222	*AQP1*	*AQP1*
cg15310349	1.70E-08	4.52E-06	6.235031	8	74358148	TES5000;Body;Body;Body;Body	25840	*STAU2-AS1;STAU2*	*STAU2-AS1*
cg11426800	1.70E-08	4.52E-06	6.235027	2	23710000	Body	101951	*KLHL29*	*KLHL29*
cg12471789	1.70E-08	4.52E-06	6.234931	1	1.51E+08	Body;Body;Body;Body;TES5000	5260	*ADAMTSL4;ADAMTSL4-AS1*	*MIR4257*
cg26882115	1.70E-08	4.52E-06	6.234634	1	1.54E+08	TSS5000;TSS5000;TSS5000;TSS5000;Body;1stExon;TSS5000;TSS5000;TSS5000;TSS5000	2139	*S100A13;S100A1;CHTOP*	*S100A1*
cg12071775	1.70E-08	4.52E-06	6.234486	14	52591786		-56009		*NID2*
cg12552438	1.70E-08	4.52E-06	6.234304	12	13288571		-31970		*GSG1*
cg25714526	1.71E-08	4.53E-06	6.233846	13	24616628	Body	62688	*SPATA13*	*SPATA13*
cg18036653	1.71E-08	4.53E-06	6.233599	17	39484787		-12825		*KRTAP17-1*
cg06535498	1.71E-08	4.53E-06	6.233585	6	32185171	Body;Body;Body	6673	*NOTCH4*	*NOTCH4*
cg14379905	1.71E-08	4.53E-06	-6.23336	6	52149891	TSS1500;TSS1500;TSS1500;TSS1500;TSS1500;TSS1500;TSS1500;TSS1500;TSS1500;TSS1500;TSS1500;TSS1500;TSS1500	-351	*MCM3*	*MCM3*
cg20187049	1.71E-08	4.53E-06	6.233076	3	28982939		339491		*RBMS3-AS3*
cg23257697	1.71E-08	4.53E-06	6.232939	2	75784752	Body;Body;Body;Body	3313	*EVA1A*	*EVA1A*
cg11445542	1.71E-08	4.54E-06	6.232598	15	75943684	Body;Body	2337	*SNX33*	*SNX33*
cg26794997	1.72E-08	4.54E-06	6.232501	18	76189257		-483574		*LINC01029*
cg13403232	1.72E-08	4.54E-06	6.232031	10	98802990	Body	-59522	*SLIT1*	*SLIT1-AS1*
cg01297896	1.72E-08	4.55E-06	6.231419	8	1.43E+08		-111497		*MROH5*
cg01830256	1.72E-08	4.55E-06	6.231295	14	1.06E+08	3'UTR;3'UTR;3'UTR;TSS5000	-2934	*PACS2;TEX22*	*TEX22*
cg03446502	1.73E-08	4.55E-06	6.230856	2	2.32E+08	TSS1500	-1017	*NMUR1*	*NMUR1*
cg22879111	1.73E-08	4.55E-06	6.230738	5	1.5E+08	Body;Body	36306	*NDST1*	*NDST1*
cg07551054	1.73E-08	4.55E-06	6.230687	8	1.41E+08	Body;Body;Body	-28834	*TRAPPC9*	*KCNK9*
cg09206774	1.73E-08	4.55E-06	6.230469	10	3581233	Body	-36203	*LOC105376360*	*LINC02669*
cg13485703	1.73E-08	4.55E-06	6.23039	17	21730179	Body	579	*UBBP4*	*UBBP4*
cg24027780	1.73E-08	4.55E-06	6.230259	7	1.5E+08	Body	5352	*LOC728743*	*LOC728743*
cg05897481	1.73E-08	4.55E-06	6.230193	15	77366214	TSS5000;TSS5000;TSS5000	-2763	*TSPAN3*	*TSPAN3*
cg23273465	1.73E-08	4.55E-06	6.230113	2	36588933	Body	6013	*CRIM1*	*CRIM1*
cg26349474	1.74E-08	4.57E-06	6.229352	17	46705637	TSS5000;TES5000	-1917	*HOXB9;MIR196A1*	*HOXB9*
cg20608699	1.74E-08	4.57E-06	6.228958	15	43559486	TSS1500;TSS1500	-360	*TGM5*	*TGM5*
cg14114322	1.74E-08	4.57E-06	6.228647	6	7269249		44183		*SSR1*
cg01720795	1.74E-08	4.57E-06	6.228617	3	1.88E+08	Body	-7728	*LPP*	*LPP-AS2*
cg24435704	1.75E-08	4.57E-06	6.228569	7	1.4E+08	TSS1500;TSS1500	-1022	*PARP12*	*PARP12*

Probe	p_adj	q_val	t	Chr	Pos.hg19	locations	dist2TSS	gene.Illumina	gene.nearest
cg14454015	1.75E-08	4.58E-06	6.22819	9	88971417	TSS5000;TSS5000;TSS5000;TSS5000	-2092	*TUT7*	*TUT7*
cg14020278	1.76E-08	4.59E-06	6.22726	9	1.4E+08	Body;Body;Body;TES5000	1990	*RABL6;MIR4292*	*MIR4292*
cg04975966	1.76E-08	4.59E-06	6.22717	1	1.11E+08	Body	3321	*ALX3*	*ALX3*
cg10355623	1.76E-08	4.59E-06	6.22699	2	2.33E+08	TES5000	8814	*ALPG*	*ALPG*
cg00566187	1.76E-08	4.61E-06	6.22626	1	2.3E+08	Body;Body	47372	*GALNT2*	*GALNT2*
cg24331786	1.76E-08	4.61E-06	6.226133	17	76895244	Body;Body;Body	4039	*TIMP2;CEP295NL*	*CEP295NL*
cg14558431	1.76E-08	4.61E-06	-6.22599	9	89763370	TSS200	-188	*LINC02872*	*LINC02872*
cg08651894	1.77E-08	4.61E-06	6.225807	1	1.55E+08	5'UTR;Body;Body;Body;Body	117	*KCNN3*	*KCNN3*
cg12354318	1.77E-08	4.61E-06	6.225686	10	14582415	TSS5000;Body;Body;Body;Body;Body;Body;Body;Body;Body;Body;Body;Body;Body;Body;Body	-1874	*FAM107B*	*FAM107B*
cg03731898	1.77E-08	4.61E-06	6.225541	2	2.08E+08	TSS200	-160	*CPO*	*CPO*
cg09997638	1.77E-08	4.61E-06	-6.22538	19	17530742	TES5000;TES5000;Body;TSS200	-113	*BISPR;MVB12A*	*MVB12A*
cg05218346	1.77E-08	4.61E-06	6.22516	14	70041283	5'UTR	305	*CCDC177*	*CCDC177*
cg11867016	1.77E-08	4.61E-06	6.224912	4	1.3E+08		173563		*LINC02615*
cg02833614	1.78E-08	4.61E-06	6.224606	10	1.35E+08	Body;Body	41379	*INPP5A*	*INPP5A*
cg06594578	1.78E-08	4.61E-06	6.224557	12	49719373	TSS1500;TES5000;TES5000;Body	-1014	*LOC101927267;TROAP*	*LOC101927267*
cg13800802	1.78E-08	4.61E-06	6.2245	9	98619666	Body	-18306	*LINC00476*	*ERCC6L2*
cg04121415	1.78E-08	4.62E-06	6.223984	3	38325566	Body	1782	*SLC22A14*	*SLC22A14*
cg04573653	1.79E-08	4.64E-06	6.222641	7	28072450	Body	-147625	*JAZF1*	*JAZF1-AS1*
cg05536132	1.80E-08	4.66E-06	6.221742	10	76335449	Body;Body;Body;Body;Body;Body	-46367	*ADK*	*LOC102723439*
cg19246549	1.80E-08	4.67E-06	6.221055	1	1.55E+08	Body;Body;Body;Body;Body	89827	*KCNN3*	*KCNN3*
cg21046119	1.81E-08	4.67E-06	6.220798	2	1.34E+08	Body;Body	51359	*NCKAP5*	*NCKAP5-AS2*
cg03343942	1.81E-08	4.68E-06	6.220179	12	56625109	TES5000;1stExon;1stExon	609	*NABP2;SLC39A5*	*SLC39A5*
cg18404706	1.82E-08	4.69E-06	6.219546	19	18761564	Body;Body;Body;Body;Body;Body	6117	*KLHL26*	*KLHL26*
cg07167632	1.82E-08	4.69E-06	6.219335	11	67269440	Body;Body;TES5000;TES5000;TES5000	3394	*PITPNM1;CDK2AP2*	*PITPNM1*
cg07974025	1.82E-08	4.69E-06	6.219258	1	3150935	Body;Body	106397	*PRDM16*	*MIR4251*
cg08472341	1.82E-08	4.69E-06	6.219251	5	17670475		-14517		*H3Y1*
cg09029959	1.82E-08	4.69E-06	6.219226	4	1.64E+08		108856		*MIR4454*
cg02603003	1.82E-08	4.69E-06	6.218912	17	10535221	Body	25405	*MYH3*	*MYH3*
cg04844423	1.82E-08	4.69E-06	6.2189	1	48188069		274988		*TRABD2B*
cg19026547	1.82E-08	4.69E-06	6.218736	22	42688838		-8905		*TCF20*
cg04276316	1.82E-08	4.69E-06	6.218723	1	2572242	TES5000	-7787	*TTC34*	*MMEL1*
cg15477209	1.82E-08	4.69E-06	6.218684	6	1408659		-18013		*MIR6720*
cg05035024	1.82E-08	4.69E-06	6.218408	17	7912378	Body	6446	*GUCY2D*	*GUCY2D*
cg15998505	1.83E-08	4.69E-06	6.218241	1	1.53E+08		28766		*S100A6*
cg24582338	1.83E-08	4.69E-06	6.218061	6	1.07E+08		47616		*PRDM1*
cg02250028	1.83E-08	4.70E-06	6.21763	15	75775352	Body	-27196	*PTPN9*	*SIN3A*
cg23733608	1.83E-08	4.70E-06	6.217428	21	35261572	3'UTR;3'UTR	26536	*ITSN1*	*ATP5PO*
cg27265778	1.83E-08	4.70E-06	6.217335	1	1.58E+08		-6220		*LINC01704*
cg05358575	1.84E-08	4.71E-06	6.216803	2	26764961	Body;Body	16663	*OTOF*	*OTOF*
cg02296890	1.84E-08	4.71E-06	6.216474	5	73495785		108365		*LINC01335*
cg15007123	1.84E-08	4.71E-06	6.216167	12	1.12E+08	3'UTR;3'UTR;3'UTR	8265	*PHETA1*	*PHETA1*
cg26035615	1.84E-08	4.72E-06	6.215914	6	30038254	TES5000;3'UTR;3'UTR	3280	*PPP1R11;RNF39*	*PPP1R11*
cg22367466	1.85E-08	4.72E-06	6.215671	12	6840532	3'UTR;3'UTR;3'UTR;3'UTR	7080	*COPS7A*	*COPS7A*
cg25486749	1.85E-08	4.72E-06	6.215659	10	1.33E+08	Body	49251	*TCERG1L*	*TCERG1L-AS1*
cg14811011	1.85E-08	4.72E-06	6.215357	17	73055792	Body;Body;Body	12473	*KCTD2*	*KCTD2*
cg18394881	1.85E-08	4.73E-06	6.21496	1	3512777	Body	15295	*MEGF6*	*MEGF6*
cg13496041	1.85E-08	4.73E-06	6.21463	3	14494726	Body;Body;Body	50621	*SLC6A6*	*SLC6A6*
cg13782388	1.86E-08	4.74E-06	6.213868	3	1.27E+08	Body;Body	-92686	*CHCHD6*	*PLXNA1*
cg13043400	1.86E-08	4.74E-06	6.213863	7	1530760	Body	13265	*INTS1*	*INTS1*
cg05335473	1.87E-08	4.75E-06	6.213152	1	84040080	TES5000	128344	*LINC01725*	*LINC01712*
cg05318454	1.87E-08	4.75E-06	6.21303	19	46117805	Body;Body;Body;Body;Body;Body;Body;Body;Body;Body;Body;Body	-12335	*EML2*	*GPR4*
cg18593210	1.87E-08	4.75E-06	6.212994	17	81021770		-12084		*B3GNTL1*
cg21222007	1.87E-08	4.75E-06	6.212748	9	38055200	Body	14024	*SHB*	*SHB*
cg07973479	1.87E-08	4.76E-06	6.212425	13	25945255	TSS1500	-856	*ATP8A2*	*ATP8A2*
cg15616998	1.87E-08	4.76E-06	6.212277	1	40435396	3'UTR;3'UTR;3'UTR;3'UTR;3'UTR;3'UTR;3'UTR;Body	14580	*MFSD2A*	*MFSD2A*
cg04479041	1.88E-08	4.77E-06	6.211513	5	10838874		-77528		*DAP*
cg08043210	1.88E-08	4.77E-06	6.211257	12	1.04E+08	Body;Body;TSS5000	3661	*GLT8D2;HCFC2*	*GLT8D2*
cg25365006	1.89E-08	4.78E-06	6.210813	3	1.26E+08	TSS1500;TES5000;TES5000;TES5000;TES5000;TSS5000	-361	*SLC41A3;ALDH1L1;ALDH1L1-AS1*	*SLC41A3*
cg23286295	1.89E-08	4.78E-06	6.210794	19	48674931	TSS1500;TSS1500;TSS1500;TSS1500;TSS1500;TSS1500;TSS1500;TSS1500;TSS1500;TSS1500;TSS1500;Body	995	*LIG1;ZSWIM9*	*ZSWIM9*
cg11843948	1.89E-08	4.78E-06	6.210296	7	5410861	Body	52316	*TNRC18*	*TNRC18*

Probe	p_adj	q_val	t	Chr	Pos.hg19	locations	dist2TSS	gene.Illumina	gene.nearest
cg01446100	1.89E-08	4.78E-06	6.21029	2	2.4E+08	Body	-14113	*HDAC4*	*MIR2467*
cg13666659	1.90E-08	4.79E-06	6.209688	17	26487538	Body	-66050	*NLK*	*PYY2*
cg26151839	1.90E-08	4.80E-06	6.209116	6	1.3E+08		-17371		*L3MBTL3*
cg12193249	1.90E-08	4.80E-06	6.209057	19	440701	Body	20295	*SHC2*	*SHC2*
cg18180846	1.90E-08	4.80E-06	6.208792	10	1.03E+08	TSS5000;Body	1505	*LBX1;LBX1-AS1*	*LBX1-AS1*
cg25604819	1.90E-08	4.80E-06	6.208749	3	82857026		821734		*LINC02008*
cg21248405	1.90E-08	4.80E-06	6.20873	6	30156394	Body;Body	24789	*TRIM26*	*TRIM26*
cg13844287	1.91E-08	4.80E-06	6.208341	1	28476989	Body;Body;Body;Body	26212	*PTAFR*	*PTAFR*
cg18760317	1.91E-08	4.80E-06	6.208338	7	1.18E+08	1stExon	170	*ANKRD7*	*ANKRD7*
cg00033371	1.91E-08	4.80E-06	6.208328	19	4956786	Body;Body;Body;Body;Body	-12337	*UHRF1*	*KDM4B*
cg23746148	1.91E-08	4.80E-06	6.208297	6	1.35E+08	TSS1500;TSS1500;TSS1500	-562	*ALDH8A1*	*ALDH8A1*
cg15405429	1.91E-08	4.81E-06	6.207737	17	76556857	Body	16621	*DNAH17*	*DNAH17*
cg05482119	1.91E-08	4.81E-06	6.207413	3	49191777	Body	57	*LAMB2P1*	*LAMB2P1*
cg26558992	1.92E-08	4.82E-06	6.206883	1	1.47E+08	Body;Body	848	*GJA5*	*GJA5*
cg02308712	1.92E-08	4.82E-06	6.206801	12	52598204	TES5000;TES5000;TES5000;TES5000;TES5000;TES5000;TES5000	6410	*LINC02874*	*LINC02874*
cg01350169	1.92E-08	4.82E-06	6.206529	10	98862384	Body;TSS200	-128	*SLIT1;SLIT1-AS1*	*SLIT1-AS1*
cg20530613	1.93E-08	4.84E-06	6.205549	3	99440673	Body;Body	83236	*COL8A1*	*COL8A1*
cg11630148	1.93E-08	4.84E-06	6.205405	10	1.34E+08	Body	54090	*INPP5A*	*INPP5A*
cg06438114	1.94E-08	4.86E-06	6.20449	1	27954305	TSS1500;Body;TSS5000	-1197	*FGR*	*FGR*
cg13728308	1.95E-08	4.87E-06	6.203623	14	34532891		-112611		*EGLN3*
cg06888836	1.95E-08	4.87E-06	6.203534	1	2074268	Body;Body;Body;Body;Body;Body;Body;Body;Body	38114	*PRKCZ*	*PRKCZ*
cg03656688	1.95E-08	4.87E-06	6.203505	7	1.34E+08	Body;Body;Body	4040	*LRGUK*	*LRGUK*
cg13392848	1.95E-08	4.87E-06	6.203297	6	1.7E+08		17338		*LINC01615*
cg06678137	1.95E-08	4.87E-06	6.203019	11	495007	TES5000;TES5000;TES5000;TES5000;TES5000;Body;Body;Body;Body;Body;Body;Body;Body	11761	*PTDSS2;RNH1*	*RNH1*
cg05308819	1.96E-08	4.88E-06	6.202425	1	1.56E+08		-9805		*ARHGEF2*
cg00958815	1.96E-08	4.88E-06	6.202288	6	31736112	TES5000;TES5000;TSS5000;Body	-2747	*MSH5-SAPCD1;SAPCD1;SAPCD1-AS1;VWA7*	*SAPCD1-AS1*
cg07444554	1.97E-08	4.90E-06	6.201215	5	1.39E+08	Body;Body;Body;Body;Body	77379	*NRG2*	*PSD2*
cg11185950	1.97E-08	4.91E-06	6.200981	5	73482658		121492		*LINC01335*
cg07364781	1.97E-08	4.91E-06	6.200427	12	26221695	Body;Body;Body;Body	16199	*RASSF8*	*RASSF8*
cg00095396	1.98E-08	4.92E-06	6.200267	16	87722219	Body;Body	77373	*JPH3*	*KLHDC4*
cg11411127	1.98E-08	4.92E-06	6.19985	21	35821909	Body;Body;Body;Body;Body;Body;Body;Body	6198	*KCNE1*	*KCNE1*
cg09527561	1.98E-08	4.93E-06	6.199552	2	1.75E+08	Body;Body	48826	*WIPF1*	*WIPF1*
cg27605104	1.98E-08	4.93E-06	6.199283	16	85349644		-9713		*MIR5093*
cg13686648	1.99E-08	4.93E-06	6.198969	13	34256302		-5371		*STARD13*
cg11403706	1.99E-08	4.93E-06	6.198775	22	49042600	Body;Body	70449	*TAFA5*	*TAFA5*
cg02478172	1.99E-08	4.94E-06	6.198165	7	1.57E+08	Body;Body;Body;Body;Body	-100968	*PTPRN2*	*MIR153-2*
cg09325506	1.99E-08	4.94E-06	-6.19811	2	2.43E+08	TSS5000	-4446	*LINC01880*	*LINC01880*
cg23705113	2.00E-08	4.94E-06	6.197915	11	77774867	Body;Body;Body;TSS200;TES5000;TES5000;TES5000	-39	*NDUFC2-KCTD14;THRSP;NDUFC2*	*THRSP*
cg07348300	2.00E-08	4.95E-06	6.197326	1	37949342	3'UTR;3'UTR;3'UTR;TES5000	3512	*ZC3H12A;MIR6732*	*MIR6732*
cg14330206	2.01E-08	4.97E-06	6.196324	17	76490717	Body;Body	9726	*DNAH17;DNAH17-AS1*	*DNAH17-AS1*
cg08041140	2.01E-08	4.98E-06	6.195789	15	69383275	Body	10086	*EWSAT1*	*EWSAT1*
cg11958675	2.02E-08	5.00E-06	6.194933	10	35618813	Body	-6623	*CCNY*	*CCNY*
cg09240001	2.02E-08	5.00E-06	6.194717	6	27292364	TSS200	-175	*VN1R10P*	*VN1R10P*
cg02423030	2.02E-08	5.00E-06	6.194709	19	12968505	Body	5432	*MAST1*	*MIR6794*
cg24395504	2.02E-08	5.00E-06	6.19461	6	1.55E+08		-38090		*CNKSR3*
cg06790053	2.03E-08	5.00E-06	6.194329	1	2.47E+08		-13465		*LINC01341*
cg25350703	2.03E-08	5.01E-06	6.193745	10	88329610		-48061		*WAPL*
cg07888300	2.03E-08	5.01E-06	6.193586	16	57879672		35124		*LOC388282*
cg11758345	2.04E-08	5.02E-06	6.193238	17	79304498		-21450		*LINC00482*
cg00232903	2.04E-08	5.02E-06	6.193129	15	49779212	Body;Body;3'UTR	63774	*FAM227B;FGF7*	*FGF7*
cg05216221	2.04E-08	5.02E-06	6.192827	11	1.11E+08	TSS1500;TSS1500;TSS200;TSS200;5'UTR;Body;Body;Body;Body;Body	22	*COLCA1;COLCA2*	*COLCA1*
cg10736955	2.04E-08	5.02E-06	6.192652	6	33150216	Body;Body;Body;TES5000	10028	*COL11A2*	*COL11A2*
cg16711450	2.04E-08	5.02E-06	6.192567	11	2183505	TSS1500;TSS1500;TSS1500;TSS1500;TSS1500;TSS1500;TES5000;TES5000;TES5000	-1066	*INS-IGF2;INS;TH*	*INS*
cg13348519	2.04E-08	5.02E-06	6.192515	6	42691466	TSS1500;TSS5000	-1131	*PRPH2;ATP6V0CP3*	*PRPH2*
cg24545495	2.04E-08	5.02E-06	6.192376	1	6149049	Body;Body;Body;Body;Body;Body	43102	*KCNAB2*	*KCNAB2*
cg09516509	2.04E-08	5.02E-06	6.192335	20	43849998	TSS200	-15	*SEMG2*	*SEMG2*
cg00540593	2.05E-08	5.03E-06	6.191459	20	61977814	3'UTR;Body;3'UTR	-13525	*CHRNA4*	*LOC100130587*
cg17818873	2.06E-08	5.04E-06	6.190755	1	1.54E+08	TSS5000;TSS5000;Body;Body;Body;Body;Body;Body;TES5000	2839	*SLC39A1;CREB3L4;JTB*	*CREB3L4*
cg12008118	2.06E-08	5.04E-06	6.190742	10	88428429	TES5000;TES5000;5'UTR;5'UTR;5'UTR;5'UTR;5'UTR;5'UTR;5'UTR;5'UTR;5'UTR;5'UTR;5'UTR;5'UTR	109	*OPN4;LDB3*	*LDB3*

Probe	p_adj	q_val	t	Chr	Pos.hg19	locations	dist2TSS	gene.Illumina	gene.nearest
cg23202058	2.06E-08	5.04E-06	6.190552	4	1.85E+08	TSS1500	-622	*ENPP6*	*ENPP6*
cg13508741	2.06E-08	5.05E-06	6.190147	11	32012497		50367		*LOC100506675*
cg16569813	2.07E-08	5.05E-06	6.18992	3	58235849	Body;Body	12331	*ABHD6*	*ABHD6*
cg20944177	2.07E-08	5.05E-06	6.189821	13	30062797	Body;Body;Body;Body;TSS1500	-910	*MTUS2;MTUS2-AS1*	*MTUS2-AS1*
cg19764295	2.07E-08	5.05E-06	6.189719	7	1.35E+08	Body;Body;Body;Body;Body	19193	*CALD1*	*CALD1*
cg04581073	2.07E-08	5.05E-06	6.189645	6	37776593		-10909		*ZFAND3*
cg07087779	2.07E-08	5.05E-06	6.18955	16	2194837	TSS5000;TSS5000	-3307	*RAB26*	*RAB26*
cg10785537	2.07E-08	5.06E-06	6.18918	2	74603226	TSS1500;TSS1500;Body;Body;Body;Body;Body	-1364	*DCTN1*	*DCTN1*
cg12867037	2.08E-08	5.06E-06	6.188633	2	1.77E+08	TES5000	-5121	*LOC401021*	*HOXD-AS2*
cg14260004	2.08E-08	5.06E-06	6.188601	1	2.33E+08		-41556		*SIPA1L2*
cg08258494	2.09E-08	5.08E-06	6.187671	1	2.4E+08	Body;Body	75828	*CHRM3*	*CHRM3-AS1*
cg24140200	2.09E-08	5.09E-06	6.187233	10	63422254	TSS1500;TSS1500;TSS1500;TSS1500;TSS1500;TSS1500	-432	*CABCOCO1*	*CABCOCO1*
cg20252015	2.09E-08	5.09E-06	6.186994	1	64100814	Body;Body;Body	11928	*PGM1*	*PGM1*
cg02604524	2.11E-08	5.13E-06	-6.18522	3	1.29E+08	Body;Body;TSS1500;1stExon	211	*KIAA1257;EFCC1*	*KIAA1257*
cg26628100	2.11E-08	5.13E-06	6.184866	16	1615017	Body	-15510	*IFT140*	*LOC105371046*
cg12697833	2.12E-08	5.14E-06	6.184048	11	1.33E+08	Body;Body	-271770	*OPCML*	*OPCML*
cg11283404	2.12E-08	5.14E-06	6.183925	8	42163265	Body;Body;Body;Body;Body;Body	-32746	*IKBKB*	*POLB*
cg11126205	2.12E-08	5.14E-06	6.183868	12	89158050		-183800		*KITLG*
cg20504417	2.12E-08	5.14E-06	6.183721	8	1.03E+08		-23453		*UBR5*
cg05692585	2.12E-08	5.14E-06	6.183599	2	2.32E+08	Body;TSS200	99	*SPATA3-AS1;SPATA3*	*SPATA3-AS1*
cg18737136	2.12E-08	5.14E-06	6.183551	2	2.25E+08	1stExon;Body;1stExon;Body	29754	*SERPINE2*	*SERPINE2*
cg10149582	2.13E-08	5.14E-06	6.183436	14	23846456	TES5000;TES5000;5'UTR;5'UTR;5'UTR;5'UTR;5'UTR;TES5000	202	*IL25;CMTM5;MYH6*	*CMTM5*
cg09466190	2.13E-08	5.15E-06	6.182965	4	1.2E+08		-5910		*SEC24D*
cg19014245	2.13E-08	5.15E-06	6.182781	7	64333138		-30481		*ZNF273*
cg07751753	2.14E-08	5.16E-06	6.182371	2	1.1E+08	Body	24133	*SH3RF3*	*MIR4266*
cg11617965	2.15E-08	5.18E-06	6.181354	7	47962164	Body	25924	*PKD1L1*	*PKD1L1*
cg05887749	2.15E-08	5.18E-06	6.181202	16	88097594	Body;Body;Body;Body;Body;Body;Body	36997	*BANP*	*LOC400553*
cg16474647	2.15E-08	5.19E-06	6.180731	6	31625159	TSS5000;TSS5000;TSS5000;TSS5000;TSS5000;TSS5000;Body;Body;Body;TES5000;TES5000;TES5000;TES5000;TES5000;TES5000	1489	*BAG6;APOM;C6orf47;GPANK1*	*APOM*
cg02961385	2.15E-08	5.19E-06	6.18046	19	18872019	Body;Body	30095	*CRTC1*	*COMP*
cg07071809	2.15E-08	5.19E-06	6.180304	7	1585771	TES5000;3'UTR	10070	*MAFK;TMEM184A*	*TMEM184A*
cg04053571	2.16E-08	5.19E-06	6.180225	12	1.33E+08	Body;TSS5000	-4206	*GALNT9;LOC100130238*	*LOC100130238*
cg09308502	2.16E-08	5.19E-06	6.180209	11	1.19E+08		-6251		*CCDC84*
cg09304624	2.16E-08	5.19E-06	6.180169	8	75735733	TSS1500;TSS1500	-1035	*PI15*	*PI15*
cg05081514	2.16E-08	5.19E-06	6.179973	8	1.43E+08	Body;Body;Body;Body;Body;Body;Body	71414	*TSNARE1*	*TSNARE1*
cg22837726	2.16E-08	5.19E-06	-6.17994	19	7565953	1stExon	3520	*TEX45*	*TEX45*
cg18741586	2.17E-08	5.20E-06	6.179177	3	52868999	TSS5000;TSS5000;5'UTR;Body;TES5000	36	*ITIH4;MUSTN1;STIMATE-MUSTN1;STIMATE*	*MUSTN1*
cg01535312	2.17E-08	5.20E-06	6.178976	6	39903199	TSS1500;TSS1500;TSS1500;TSS1500;TSS1500;TSS1500;TSS1500	-998	*MOCS1*	*MOCS1*
cg24133771	2.17E-08	5.20E-06	6.178947	17	80769004	Body	28894	*TBCD*	*ZNF750*
cg06840723	2.17E-08	5.20E-06	6.178716	15	52484492	TSS1500;Body;TES5000	-903	*GNB5;CERNA1;MYO5C*	*GNB5*
cg03406011	2.17E-08	5.21E-06	6.178519	3	1.29E+08	TSS1500;Body;TSS5000;Body	-861	*KIAA1257;EFCC1*	*KIAA1257*
cg12043732	2.18E-08	5.21E-06	6.177986	20	61284223	Body	10369	*SLCO4A1*	*SLCO4A1*
cg07987051	2.18E-08	5.22E-06	6.17784	16	5080405	Body;TSS5000	-3297	*NAGPA;NAGPA-AS1*	*NAGPA-AS1*
cg02269496	2.18E-08	5.22E-06	6.177408	13	1.13E+08		74614		*SPACA7*
cg27577993	2.19E-08	5.22E-06	6.177109	7	1.51E+08	Body;Body	-21395	*PRKAG2*	*PRKAG2*
cg00650672	2.19E-08	5.22E-06	6.177046	3	65764475	Body;Body;Body;Body;Body;Body	-115015	*MAGI1*	*MAGI1-AS1*
cg10648623	2.19E-08	5.22E-06	6.177006	7	89866283	3'UTR;3'UTR;3'UTR;3'UTR;3'UTR;3'UTR	-8204	*STEAP2*	*CFAP69*
cg17917368	2.19E-08	5.23E-06	6.176636	3	1.28E+08	TES5000	7959	*LINC01565*	*LINC01565*
cg15106420	2.20E-08	5.24E-06	6.175908	5	1.09E+08	Body	17171	*MAN2A1*	*MAN2A1*
cg06924081	2.20E-08	5.24E-06	6.175696	15	69791774		46618		*RPLP1*
cg25790955	2.20E-08	5.25E-06	6.175481	11	73202336	Body;Body;Body	106821	*FAM168A*	*FAM168A*
cg00629700	2.20E-08	5.25E-06	6.175118	17	30190328	TSS5000;TSS5000;3'UTR	-4065	*COPRS;UTP6*	*COPRS*
cg25135145	2.20E-08	5.25E-06	6.175111	3	16233024	Body;Body	16838	*GALNT15*	*GALNT15*
cg22191973	2.20E-08	5.25E-06	6.175094	18	55292558	TSS5000	-3490	*NARS1*	*NARS1*
cg18309674	2.21E-08	5.25E-06	6.174979	14	1.05E+08		-8442		*CDCA4*
cg14737905	2.21E-08	5.26E-06	6.174127	2	1.28E+08	Body;TSS5000	-4984	*PROC;MIR4783*	*MIR4783*
cg05997793	2.22E-08	5.28E-06	6.173435	3	37901338	Body;Body;TSS5000;TSS5000	1933	*ITGA9-AS1;CTDSPL*	*ITGA9-AS1*
cg05348188	2.22E-08	5.28E-06	6.173314	21	45877268	TSS5000;1stExon	1876	*LRRC3-DT;LRRC3*	*LRRC3*
cg07899634	2.22E-08	5.28E-06	6.173178	6	33234620	Body;Body;Body;Body	5033	*VPS52*	*VPS52*

Probe	p_adj	q_val	t	Chr	Pos.hg19	locations	dist2TSS	gene.Illumina	gene.nearest
cg00495305	2.22E-08	5.28E-06	6.173054	3	43659797	Body;Body;Body;Body;Body;Body;Body;Body;Body;Body;Body;Body	3621	*ANO10*	*ANO10*
cg21230284	2.23E-08	5.28E-06	6.172892	4	1660919	Body;Body;Body	24799	*FAM53A*	*FAM53A*
cg02041108	2.23E-08	5.29E-06	6.172348	5	35946185	TES5000	7283	*UGT3A1*	*LOC100506406*
cg11523960	2.24E-08	5.30E-06	6.171758	5	88004111	Body	-17253	*MEF2C-AS2*	*LINC00461*
cg16194160	2.24E-08	5.30E-06	6.171633	8	1.45E+08	Body;Body;Body	3454	*PARP10*	*PARP10*
cg07539121	2.24E-08	5.30E-06	6.171337	5	55606711		-77554		*ANKRD55*
cg00598956	2.24E-08	5.30E-06	6.171298	4	77409957	Body	53576	*SHROOM3*	*SHROOM3*
cg04813711	2.24E-08	5.30E-06	6.171061	16	57597905	Body;Body;Body	21351	*ADGRG5*	*ADGRG5*
cg01030178	2.25E-08	5.31E-06	6.170757	8	67037892	TSS1500;TSS1500;TSS1500;TSS1500	-1471	*TRIM55*	*TRIM55*
cg21898299	2.25E-08	5.31E-06	6.170682	15	68924220	Body;Body;Body;TSS200	-106	*CORO2B*	*CORO2B*
cg26893743	2.25E-08	5.31E-06	6.17053	5	1.54E+08	Body	22813	*GALNT10*	*MIR1294*
cg19692314	2.25E-08	5.31E-06	6.170146	1	45042285	Body;Body;Body	31121	*RNF220*	*MIR5584*
cg25651608	2.25E-08	5.31E-06	6.169978	3	42073868		-54823		*TRAK1*
cg03994274	2.26E-08	5.32E-06	6.169585	8	1.03E+08	Body;Body	65788	*GRHL2*	*GRHL2*
cg04398275	2.26E-08	5.32E-06	6.169331	12	1.24E+08	Body;Body	14573	*SBNO1*	*MIR8072*
cg04093821	2.26E-08	5.32E-06	6.169254	2	38247975	TES5000;Body;TES5000;TES5000;Body;TES5000;Body	15509	*RMDN2;RMDN2-AS1*	*RMDN2-AS1*
cg06411137	2.26E-08	5.33E-06	6.169034	11	20390803	Body;Body;Body;Body;TES5000	5117	*HTATIP2*	*HTATIP2*
cg20987283	2.27E-08	5.33E-06	6.168549	8	1.36E+08	Body	1137	*LOC101927845*	*LOC101927845*
cg16100530	2.27E-08	5.33E-06	6.168328	15	84146864	Body;Body;Body;Body;Body;Body;Body;Body;Body;Body;Body;Body;Body	-12501	*SH3GL3*	*SH3GL3*
cg12072770	2.27E-08	5.33E-06	6.168263	10	1995014		61528		*LINC00700*
cg25208740	2.27E-08	5.34E-06	6.168001	15	42780090	Body;Body;Body;Body	3249	*ZNF106*	*ZNF106*
cg12523691	2.27E-08	5.34E-06	6.167934	4	1.7E+08	Body;Body;Body	-35564	*PALLD*	*PALLD*
cg22158175	2.28E-08	5.35E-06	6.16738	6	32809475	TSS5000;TSS5000;Body;Body;TSS5000;TSS5000;TSS5000;TSS5000;TES5000;TES5000	2349	*TAP2;PSMB8;PSMB8-AS1;TAP1*	*PSMB8*
cg05036120	2.28E-08	5.35E-06	6.167292	1	2.13E+08	3'UTR;3'UTR;3'UTR;3'UTR;3'UTR;3'UTR;TSS5000	-4532	*ATF3;FAM71A*	*FAM71A*
cg19751824	2.28E-08	5.35E-06	6.167076	11	1.25E+08	Body	-30174	*FEZ1*	*LOC403312*
cg20181262	2.28E-08	5.35E-06	6.167008	12	1.32E+08	Body;Body	-28981	*SFSWAP*	*MMP17*
cg19350970	2.28E-08	5.35E-06	6.166955	2	2.38E+08		44617		*COPS8*
cg04226002	2.29E-08	5.35E-06	6.166708	11	1.14E+08	Body;Body;Body;Body;Body	19530	*ZBTB16*	*ZBTB16*
cg01584939	2.29E-08	5.35E-06	6.166424	22	37254322	Body;TSS5000;TSS5000	-2707	*NCF4-AS1;NCF4*	*NCF4*
cg14122749	2.29E-08	5.36E-06	6.165941	12	64355151	Body;Body	116672	*SRGAP1*	*SRGAP1*
cg06736674	2.29E-08	5.36E-06	6.165934	6	1.67E+08	Body	646	*LOC729681*	*LOC729681*
cg05790630	2.30E-08	5.36E-06	6.165684	3	64963168	Body	-289492	*ADAMTS9-AS2*	*ADAMTS9*
cg10840135	2.30E-08	5.36E-06	6.16556	22	42526619	1stExon;1stExon	193	*CYP2D6*	*CYP2D6*
cg05834191	2.30E-08	5.36E-06	6.165549	14	1.06E+08	Body;Body	-20592	*MTA1*	*CRIP2*
cg08732428	2.31E-08	5.38E-06	6.164503	1	55243019	TES5000	-12823	*TTC22*	*PARS2*
cg00044466	2.31E-08	5.38E-06	6.164054	16	15238525		10334		*MIR3180-4*
cg04104695	2.31E-08	5.38E-06	6.164039	5	1.39E+08	Body;Body;Body;Body;Body;Body;Body;Body;Body;Body;Body;Body;Body;Body	3722	*CXXC5*	*CXXC5*
cg26187724	2.31E-08	5.38E-06	6.163994	6	3697798		54448		*PXDC1*
cg09817985	2.32E-08	5.39E-06	6.163831	20	25054507	Body;Body;Body;Body;TES5000;TES5000	8508	*VSX1*	*VSX1*
cg21609154	2.32E-08	5.39E-06	6.163705	13	1.14E+08	TSS5000;Body;Body	1118	*MCF2L-AS1;MCF2L*	*MCF2L*
cg09970511	2.32E-08	5.39E-06	6.163521	6	56823640	TSS5000;Body;Body	3736	*DST;BEND6*	*BEND6*
cg07850832	2.32E-08	5.39E-06	6.163355	5	37378050	TSS1500;TSS1500;TSS1500	-1369	*WDR70*	*WDR70*
cg25050479	2.32E-08	5.39E-06	6.163247	11	45718671		-5345		*MIR7154*
cg04988367	2.33E-08	5.40E-06	6.16277	5	10933006		-171660		*DAP*
cg14328415	2.33E-08	5.40E-06	6.162577	9	1.4E+08	Body	590	*LCN12*	*LCN12*
cg00310792	2.33E-08	5.40E-06	6.16242	4	1.48E+08	1stExon;Body;Body;Body	3217	*TTC29*	*TTC29*
cg12852139	2.33E-08	5.41E-06	6.161941	5	16794882	Body	141515	*MYO10*	*MYO10*
cg04158698	2.34E-08	5.41E-06	6.161893	1	2.02E+08		34010		*RPS10P7*
cg13315130	2.35E-08	5.44E-06	6.160456	22	21045582	Body	1740	*POM121L4P*	*POM121L4P*
cg08453263	2.35E-08	5.44E-06	6.160078	11	16838675	Body;Body;Body	78482	*PLEKHA7*	*C11orf58*
cg15318814	2.36E-08	5.45E-06	6.159575	2	98351769	Body;Body	901	*ZAP70*	*ZAP70*
cg16720847	2.37E-08	5.48E-06	6.158383	12	42854129	Body;Body;Body;Body	23288	*PRICKLE1*	*PRICKLE1*
cg13893221	2.37E-08	5.48E-06	6.158134	15	85199995	TSS5000;TSS5000;TSS5000;TSS5000;TSS5000;Body;Body	1435	*WDR73;NMB*	*NMB*
cg20216030	2.38E-08	5.49E-06	6.157504	10	1.34E+08	Body	10653	*BNIP3*	*BNIP3*
cg13296809	2.39E-08	5.50E-06	6.157025	11	69709095		-74911		*FGF3*
cg04522821	2.39E-08	5.50E-06	6.156863	19	54041329	Body;Body;Body;TSS200;TSS200;TSS200;TSS200;TSS200;TSS1500	-69	*ZNF331*	*ZNF331*
cg00672802	2.39E-08	5.50E-06	6.156832	1	2.26E+08		25596		*LINC01703*
cg24829772	2.39E-08	5.50E-06	6.156829	8	18577488	Body;Body;Body	88889	*PSD3*	*PSD3*
cg10397819	2.39E-08	5.50E-06	6.156431	14	75447512		-22111		*EIF2B2*
cg01553613	2.40E-08	5.52E-06	6.155777	14	77734687	Body;TES5000	2127	*NGB;MIR1260A*	*MIR1260A*

Probe	p_adj	q_val	t	Chr	Pos.hg19	locations	dist2TSS	gene.Illumina	gene.nearest
cg18909596	2.40E-08	5.52E-06	6.155715	8	1.41E+08	Body;Body;Body	-28960	*TRAPPC9*	*KCNK9*
cg01131388	2.40E-08	5.52E-06	6.155423	3	1.58E+08		275441		*SHOX2*
cg05625955	2.41E-08	5.52E-06	6.155082	6	1.71E+08	TES5000	10663	*LOC154449*	*LOC154449*
cg24381587	2.41E-08	5.52E-06	6.15508	1	1.59E+08	Body	160	*OR6N1*	*OR6N1*
cg10932916	2.41E-08	5.53E-06	6.15446	5	1.66E+08		828284		*LINC01947*
cg24716420	2.41E-08	5.53E-06	6.154387	4	7802369	Body;Body;Body;Body	46553	*AFAP1*	*AFAP1-AS1*
cg19955284	2.41E-08	5.53E-06	6.154349	6	31833747	TSS5000;Body;Body;Body	-3149	*NEU1;SLC44A4*	*NEU1*
cg22136329	2.41E-08	5.53E-06	6.154208	7	27296033		-9185		*EVX1-AS*
cg25246281	2.42E-08	5.53E-06	6.153923	17	27188748	TES5000;TES5000;TES5000;TES5000;TSS1500;TES5000;TSS200;Body	0	*ERAL1;MIR451A;MIR451B;MIR144;MIR4732*	*MIR4732*
cg18893868	2.42E-08	5.53E-06	6.153888	12	1.31E+08	Body;Body;Body;Body;Body;Body;Body;Body;Body	132284	*RIMBP2*	*PIWIL1*
cg13683507	2.43E-08	5.55E-06	-6.15305	10	1463108	Body	-105716	*ADARB2*	*ADARB2-AS1*
cg05614408	2.43E-08	5.56E-06	6.152609	5	94901279	Body	10475	*ARSK*	*ARSK*
cg23947050	2.43E-08	5.56E-06	6.152308	13	26046175	Body;Body	3264	*ATP8A2*	*ATP8A2*
cg26656387	2.44E-08	5.56E-06	6.152233	13	74356270	Body	217890	*KLF12*	*LINC00392*
cg19102120	2.44E-08	5.56E-06	6.152138	5	1.18E+08		-178135		*MIR1244-2*
cg22514764	2.44E-08	5.56E-06	6.152123	1	2.44E+08	Body;Body;Body;Body;Body;Body	128361	*SDCCAG8*	*MIR4677*
cg20899625	2.44E-08	5.56E-06	6.152072	3	1.27E+08	Body	13695	*PLXNA1*	*PLXNA1*
cg22802461	2.44E-08	5.57E-06	6.151486	19	35981531	TSS200;TSS200	-165	*KRTDAP*	*KRTDAP*
cg20775508	2.45E-08	5.57E-06	6.151302	20	44996230	TSS5000;TSS5000;TSS5000;TSS5000;TSS5000;TSS5000;TSS5000;Body;Body;Body	-3133	*SLC35C2;ELMO2*	*SLC35C2*
cg05780864	2.45E-08	5.57E-06	6.151053	8	1.41E+08	Body;Body	82581	*KCNK9*	*KCNK9*
cg03805019	2.45E-08	5.58E-06	6.15052	20	62464090	TSS1500	-1473	*ZBTB46*	*ZBTB46*
cg26161024	2.46E-08	5.59E-06	6.150381	1	27693840	TSS1500;TSS1500;TES5000;TES5000	-388	*MAP3K6;FCN3*	*MAP3K6*
cg13256598	2.46E-08	5.60E-06	6.149748	4	5624571	Body;Body	85715	*EVC2*	*EVC2*
cg01572468	2.47E-08	5.60E-06	6.149127	11	75225065	Body;Body	11553	*GDPD5*	*GDPD5*
cg20152382	2.47E-08	5.60E-06	6.149024	17	64783099	Body;TSS200	-90	*PRKCA;MIR634*	*MIR634*
cg26674132	2.47E-08	5.60E-06	6.148946	19	9434637	5'UTR;5'UTR;5'UTR;5'UTR;5'UTR;5'UTR;TSS1500;TSS1500;TSS1500;TSS1500	190	*ZNF559;ZNF559-ZNF177*	*ZNF559*
cg14780276	2.47E-08	5.60E-06	6.148888	4	1.49E+08	Body	39455	*ARHGAP10*	*MIR4799*
cg20902353	2.47E-08	5.60E-06	6.148887	14	94425114	TSS5000;Body	-1721	*ASB2*	*ASB2*
cg27623914	2.47E-08	5.60E-06	6.148607	17	21355856		76188		*KCNJ12*
cg05465059	2.47E-08	5.60E-06	6.148549	11	45672555	Body	14617	*CHST1*	*CHST1*
cg08061360	2.48E-08	5.60E-06	6.148493	19	51917747	Body;Body;Body;Body;Body;Body;Body;TSS1500;TSS1500	-1230	*SIGLEC10;LOC100129083*	*LOC100129083*
cg15369608	2.48E-08	5.60E-06	6.14842	2	2.4E+08	Body	15390	*HDAC4*	*MIR4440*
cg11031372	2.48E-08	5.61E-06	6.148109	17	79173614	Body;Body;Body;Body	23151	*CEP131*	*CEP131*
cg23192479	2.49E-08	5.62E-06	6.147531	2	1.1E+08	Body	-21625	*SH3RF3*	*MIR4266*
cg17543112	2.49E-08	5.62E-06	6.147316	5	1.32E+08	Body;Body	947	*PDLIM4*	*PDLIM4*
cg06024642	2.49E-08	5.62E-06	6.147192	17	4510776	Body;Body;TSS5000	-2967	*SMTNL2;LINC01996*	*LINC01996*
cg06504039	2.50E-08	5.64E-06	6.146426	6	32978379	TSS1500	-1011	*HLA-DOA*	*HLA-DOA*
cg00998379	2.50E-08	5.64E-06	6.146311	1	53074332	3'UTR	6290	*GPX7*	*GPX7*
cg00016968	2.50E-08	5.64E-06	6.146132	1	1.13E+08	TSS1500;TSS1500;TSS1500;TES5000	-423	*RHOC;PPM1J*	*RHOC*
cg19808587	2.50E-08	5.64E-06	6.146052	1	28520874	TSS1500;TSS1500	-427	*PTAFR*	*PTAFR*
cg13427810	2.50E-08	5.64E-06	6.145973	9	1.17E+08	Body	18923	*COL27A1*	*MIR455*
cg12586179	2.51E-08	5.65E-06	6.145413	17	80349858	3'UTR;3'UTR;Body	17706	*OGFOD3*	*UTS2R*
cg27252367	2.51E-08	5.65E-06	6.145284	11	71313287		-19366		*KRTAP5-11*
cg20749005	2.51E-08	5.65E-06	6.14493	8	1.41E+08	Body;Body;Body	160627	*TRAPPC9*	*PEG13*
cg26354398	2.52E-08	5.65E-06	6.144803	9	1.16E+08	Body;TSS5000;TSS5000	-1943	*RGS3*	*RGS3*
cg27607136	2.52E-08	5.65E-06	6.144605	11	1857442	Body;Body;Body;Body;TSS5000;TSS5000;TSS5000	1786	*SYT8;TNNI2*	*SYT8*
cg19833679	2.52E-08	5.65E-06	6.144479	22	41636941	TSS200;TES5000;TES5000;TES5000	-6	*CHADL;RANGAP1*	*CHADL*
cg09950164	2.52E-08	5.65E-06	6.144476	11	65984109	Body	-40655	*PACS1*	*KLC2*
cg20227165	2.52E-08	5.65E-06	6.144437	11	45115819	Body	256	*PRDM11*	*PRDM11*
cg26353161	2.52E-08	5.65E-06	6.144309	7	28331380		-7559		*CREB5*
cg25981875	2.53E-08	5.66E-06	6.143942	6	31860696	Body;Body;Body;Body;Body;TSS5000	4070	*EHMT2;C2*	*EHMT2*
cg14891651	2.53E-08	5.67E-06	6.143503	11	3690320	TES5000;Body;Body;Body	2294	*ART1;CHRNA10*	*CHRNA10*
cg20259594	2.54E-08	5.68E-06	6.142877	3	15920154	TES5000	4877	*MIR563*	*MIR563*
cg16989443	2.54E-08	5.68E-06	6.142779	1	1849195	TES5000;TES5000;3'UTR;TES5000	1523	*CALML6;TMEM52;CFAP74*	*TMEM52*
cg08684854	2.54E-08	5.68E-06	6.142697	16	30955244	Body;Body	-5144	*FBXL19*	*ORAI3*
cg15161225	2.54E-08	5.69E-06	6.142187	22	36013678	TSS1500;TSS200;Body;Body	-123	*MB*	*MB*
cg11598561	2.55E-08	5.69E-06	6.142013	3	1.28E+08	3'UTR;TSS1500	-1114	*DNAJB8;DNAJB8-AS1*	*DNAJB8-AS1*
cg09578614	2.55E-08	5.69E-06	6.141976	16	68405406	1stExon	60390	*SMPD3*	*PRMT7*
cg21504353	2.56E-08	5.71E-06	6.141012	22	24577288	TES5000;TES5000;TES5000;TSS200	-166	*CABIN1;SUSD2*	*SUSD2*

Probe	p_adj	q_val	t	Chr	Pos.hg19	locations	dist2TSS	gene.Illumina	gene.nearest
cg23210309	2.56E-08	5.71E-06	6.140926	10	70232583	TSS1500;TSS1500	-705	*DNA2*	*DNA2*
cg09585195	2.56E-08	5.71E-06	6.140907	16	2016875	TES5000;TSS5000;TSS5000;TSS5000;TES5000;TES5000;5'UTR;5'UTR	19	*NDUFB10;RPS2;SNORA10;SNORA64;SNHG9;SNORA78;RNF151*	*RNF151*
cg25786651	2.56E-08	5.71E-06	6.140729	7	1.51E+08	TSS200;TSS200;Body;TES5000	-8	*ASB10;IQCA1L*	*ASB10*
cg16173775	2.56E-08	5.71E-06	6.140601	1	32673381	TES5000;TES5000;Body;Body;TSS1500	-1313	*CCDC28B;IQCC;DCDC2B*	*DCDC2B*
cg27074221	2.56E-08	5.71E-06	6.1406	14	1.02E+08	TES5000;TES5000;TES5000;TSS200	-11	*MIR494;MIR1193;MIR543;MIR495*	*MIR495*
cg22588275	2.59E-08	5.77E-06	6.13801	3	1.68E+08	Body	23468	*EGFEM1P*	*EGFEM1P*
cg02671333	2.59E-08	5.77E-06	6.138002	19	39806102	TSS200	-126	*LRFN1*	*LRFN1*
cg14020326	2.59E-08	5.77E-06	6.1377	14	1.06E+08	Body;Body;Body	-39618	*PACS2*	*BRF1*
cg06885284	2.60E-08	5.78E-06	6.137189	8	20010728	Body;Body;Body;Body	29919	*SLC18A1*	*SLC18A1*
cg23040531	2.60E-08	5.78E-06	6.136991	10	1.32E+08	Body;TES5000	4909	*EBF3;MIR4297*	*MIR4297*
cg22759234	2.60E-08	5.78E-06	6.136958	17	62975582	TSS5000	-3879	*AMZ2P1*	*AMZ2P1*
cg26158528	2.60E-08	5.78E-06	6.136925	1	28607681	3'UTR	21653	*SESN2*	*SESN2*
cg21052025	2.61E-08	5.78E-06	6.136756	11	69001490		-60131		*MYEOV*
cg09293816	2.61E-08	5.78E-06	6.136436	19	50015532	TSS1500;TSS1500	-385	*FCGRT*	*FCGRT*
cg25498385	2.61E-08	5.78E-06	6.136331	13	21288714	Body	11233	*IL17D*	*IL17D*
cg12845177	2.61E-08	5.78E-06	6.136132	19	8650523	Body;Body	6557	*ADAMTS10*	*ADAMTS10*
cg14467840	2.61E-08	5.78E-06	6.136044	1	1.54E+08	TSS1500;TSS1500;TSS1500;TSS1500;Body;5'UTR	63	*S100A13;S100A1*	*S100A1*
cg02133510	2.61E-08	5.78E-06	6.136005	6	32044959	Body;Body	-31054	*TNXB*	*TNXB*
cg23232719	2.62E-08	5.79E-06	6.135823	14	1.04E+08	Body	-31988	*TDRD9*	*RD3L*
cg04165188	2.62E-08	5.79E-06	6.135458	8	840173	Body;Body	-6947	*DLGAP2*	*LOC105377777*
cg19611163	2.62E-08	5.80E-06	6.135104	3	1.23E+08	Body;Body;Body;Body;Body;Body	2721	*MYLK;MYLK-AS2*	*MYLK-AS2*
cg06070644	2.63E-08	5.80E-06	6.13494	4	25677759	Body;Body;Body	19674	*SLC34A2*	*SLC34A2*
cg17883035	2.64E-08	5.83E-06	6.133506	19	45261808	Body;TSS200	-105	*BCL3;MIR8085*	*MIR8085*
cg23742669	2.64E-08	5.83E-06	6.133337	18	13642414	Body;Body;Body;Body;Body;5'UTR	658	*LDLRAD4*	*LDLRAD4*
cg04657715	2.65E-08	5.83E-06	6.133217	3	99904527	TES5000;TSS200;TSS200	-140	*CMSS1;TMEM30CP*	*TMEM30CP*
cg09386620	2.65E-08	5.83E-06	6.133163	2	2.18E+08	Body	84350	*DIRC3*	*DIRC3-AS1*
cg25012649	2.65E-08	5.83E-06	6.133034	8	485766	Body;Body	9079	*TDRP*	*TDRP*
cg09629753	2.65E-08	5.83E-06	6.132914	16	29323721	Body	10114	*SNX29P2*	*SNX29P2*
cg05628390	2.65E-08	5.83E-06	6.132848	1	2259411	Body	24689	*MORN1*	*LOC100129534*
cg26313421	2.66E-08	5.85E-06	6.13217	2	47465189	Body;Body	-61114	*EPCAM-DT*	*CALM2*
cg21127729	2.66E-08	5.85E-06	6.131952	7	1.28E+08		34981		*MIR593*
cg10912307	2.66E-08	5.85E-06	6.131736	22	50895738	Body;Body	17726	*SBF1*	*SBF1*
cg11855842	2.67E-08	5.86E-06	6.131461	1	23807492	Body;Body	3180	*ASAP3*	*ASAP3*
cg04981940	2.67E-08	5.86E-06	6.131361	1	1398348	Body	-8796	*ATAD3C*	*ATAD3B*
cg01287505	2.68E-08	5.88E-06	6.130317	17	56607190	Body;TSS5000;TSS1500;TSS1500;Body;Body;Body;Body;Body;TSS1500;TSS1500;Body;Body	-362	*SEPTIN4-AS1;SEPTIN4*	*SEPTIN4*
cg16068383	2.68E-08	5.88E-06	6.130247	15	56201496	Body;Body;Body;Body;Body;Body;Body	7833	*NEDD4*	*NEDD4*
cg01589587	2.68E-08	5.88E-06	6.130047	14	76002440	Body	13629	*BATF*	*BATF*
cg15369784	2.68E-08	5.88E-06	6.130029	6	36906565		-9473		*PI16*
cg04125962	2.68E-08	5.88E-06	6.12985	16	27476250	Body;Body	-11536	*GTF3C1*	*IL21R-AS1*
cg24482544	2.69E-08	5.88E-06	6.129815	1	1.6E+08	Body	1102	*VANGL2*	*VANGL2*
cg08861541	2.69E-08	5.88E-06	6.129676	11	2610149	Body;Body	111079	*KCNQ1*	*KCNQ1OT1*
cg19210342	2.69E-08	5.88E-06	6.12958	10	23640730		-7004		*C10orf67*
cg01894322	2.70E-08	5.90E-06	6.128494	17	37789575	Body;Body;Body;TSS5000;TSS5000;TSS5000	-3757	*PPP1R1B;STARD3*	*STARD3*
cg14210001	2.70E-08	5.90E-06	6.128309	12	1.25E+08	Body;Body	143732	*ZNF664-RFLNA*	*ZNF664-RFLNA*
cg09452911	2.70E-08	5.90E-06	6.128227	2	69473156	3'UTR	141203	*ANTXR1*	*GFPT1*
cg08393972	2.72E-08	5.93E-06	6.127095	4	2372832	Body;Body;Body;Body	-6291	*ZFYVE28*	*ZFYVE28*
cg04580068	2.72E-08	5.93E-06	6.126864	3	23590052	Body;Body	-257393	*UBE2E2*	*UBE2E1*
cg01624768	2.73E-08	5.94E-06	6.126441	16	85465657		-125726		*MIR5093*
cg10779202	2.73E-08	5.94E-06	6.126212	7	92626636		120656		*SAMD9*
cg18304839	2.73E-08	5.94E-06	6.126162	8	1246922	Body;Body	3905	*DLGAP2;LOC286083*	*LOC286083*
cg15485378	2.73E-08	5.95E-06	6.125816	3	52828680	TES5000;TES5000;TES5000;TES5000;TSS200	-103	*ITIH1;ITIH3*	*ITIH3*
cg11090131	2.73E-08	5.95E-06	6.125771	16	354263	Body;Body;Body	21100	*AXIN1*	*PDIA2*
cg00653847	2.74E-08	5.95E-06	6.125513	10	1.02E+08		-6777		*SCD*
cg09317036	2.75E-08	5.97E-06	6.124721	8	1.45E+08	TSS200;TES5000;TSS5000	-9	*GSDMD;LOC100310756*	*GSDMD*
cg00472879	2.76E-08	5.99E-06	6.123722	10	97181896	Body;Body;Body;Body;Body;Body;Body;Body;Body;Body;Body;Body	19041	*SORBS1*	*SORBS1*
cg02901002	2.76E-08	5.99E-06	6.123638	7	42001667	3'UTR	-258979	*GLI3*	*INHBA*
cg24332389	2.76E-08	5.99E-06	6.123618	1	6558085	TSS1500;TSS1500;Body	-581	*PLEKHG5*	*PLEKHG5*
cg09558069	2.76E-08	5.99E-06	6.123373	1	1.54E+08	TSS5000;Body	-2429	*S100A6;S100A5*	*S100A6*
cg00459816	2.77E-08	6.01E-06	6.122695	17	37862113	Body;Body;Body;Body;Body;Body	5883	*ERBB2*	*ERBB2*

Probe	p_adj	q_val	t	Chr	Pos.hg19	locations	dist2TSS	gene.Illumina	gene.nearest
cg10251744	2.77E-08	6.01E-06	6.122552	13	74258128	TES5000	119748	*KLF12*	*LINC00392*
cg00130707	2.78E-08	6.01E-06	6.122141	16	20303127		35708		*GP2*
cg23586659	2.78E-08	6.01E-06	6.122105	1	2.26E+08	TES5000;TES5000	-55433	*ENAH*	*LBR*
cg08634826	2.78E-08	6.02E-06	6.121929	11	71193572	Body	9024	*NADSYN1*	*MIR6754*
cg18815565	2.78E-08	6.02E-06	6.121802	20	17540008	TSS1500;TSS1500;TSS1500;Body	-530	*BFSP1*	*BFSP1*
cg09854814	2.78E-08	6.02E-06	6.121587	1	1.54E+08	TSS5000;TSS5000;TSS5000	-2659	*ILF2;NPR1*	*ILF2*
cg06427957	2.79E-08	6.04E-06	6.120754	5	81601203	5'UTR;Body;Body;5'UTR;5'UTR	25923	*ATP6AP1L*	*ATP6AP1L*
cg10276506	2.79E-08	6.04E-06	6.120628	6	31690170	TES5000;TES5000;TSS1500;TSS1500;TSS1500;TSS1500;TSS1500;TSS1500;TSS1500;TES5000;TES5000;TES5000	-647	*LY6G6F-LY6G6D;LY6G6D;LY6G6C;MPIG6B;DDAH2*	*LY6G6C*
cg07917177	2.80E-08	6.04E-06	6.120608	20	62811729	Body	15925	*MYT1*	*MYT1*
cg06809123	2.81E-08	6.06E-06	6.119627	5	65497964	TES5000	7310	*LINC02065*	*LINC02065*
cg16284000	2.81E-08	6.06E-06	6.119333	8	21638355	Body;Body;Body	8032	*GFRA2*	*GFRA2*
cg13355449	2.81E-08	6.06E-06	6.119333	6	46923414	TSS1500	-738	*ADGRF5*	*ADGRF5*
cg21767295	2.82E-08	6.07E-06	6.118742	15	45819129	TSS5000;TSS5000;Body	-4176	*SLC30A4;HMGN2P46*	*SLC30A4*
cg02096194	2.82E-08	6.07E-06	6.118675	3	51997492	TSS1500;Body;Body;Body;Body	-584	*PCBP4*	*PCBP4*
cg03736685	2.82E-08	6.07E-06	6.118624	16	55546074	Body	3091	*LPCAT2*	*LPCAT2*
cg03564009	2.83E-08	6.08E-06	6.118121	13	1.11E+08	Body	30728	*COL4A1*	*LOC105370361*
cg04573153	2.83E-08	6.08E-06	6.118094	3	42944225	TSS5000;TSS5000	-3408	*ZNF662*	*ZNF662*
cg05576828	2.83E-08	6.08E-06	6.118012	8	29194717	Body;Body	11605	*DUSP4*	*DUSP4*
cg20260028	2.83E-08	6.08E-06	6.117922	15	31358057	Body;Body;Body;TSS1500;TES5000	-713	*TRPM1;MIR211*	*MIR211*
cg02669055	2.83E-08	6.08E-06	6.117811	8	39848333	Body	55860	*IDO2*	*IDO2*
cg01118026	2.83E-08	6.08E-06	6.117622	16	88896814	Body;Body;Body	-18478	*GALNS*	*APRT*
cg25144574	2.83E-08	6.08E-06	6.117378	5	36655019	Body;Body;Body;Body	48312	*SLC1A3*	*SLC1A3*
cg00593773	2.84E-08	6.09E-06	6.117067	18	77336201	TSS5000	-3080	*LOC284240*	*LOC284240*
cg12903047	2.84E-08	6.09E-06	6.116919	11	68928793	Body;TES5000;Body	5391	*LOC338694;SMIM38*	*SMIM38*
cg25014937	2.84E-08	6.09E-06	6.116743	13	66583686		-43125		*MIR548X2*
cg18802022	2.84E-08	6.09E-06	6.116653	10	1E+08	Body	-7767	*PYROXD2*	*MIR1287*
cg25523261	2.85E-08	6.09E-06	6.11649	2	38118971		-15965		*LINC00211*
cg00271096	2.85E-08	6.10E-06	6.116174	21	32410480	3'UTR	315	*KRTAP19-8*	*KRTAP19-8*
cg01148276	2.85E-08	6.10E-06	6.115861	15	68608583	Body	38501	*ITGA11*	*FEM1B*
cg02544317	2.86E-08	6.11E-06	6.11559	8	57024065	TES5000	2476	*MOS*	*MOS*
cg03166258	2.86E-08	6.11E-06	6.115375	2	1.33E+08		-23306		*ANKRD30BL*
cg22182781	2.86E-08	6.11E-06	6.115324	11	66436559	TES5000;Body;Body	8799	*RBM4;RBM4B*	*RBM4B*
cg02829076	2.86E-08	6.11E-06	6.115114	11	1.02E+08	TSS5000;TSS5000;TSS5000	-2780	*CFAP300*	*CFAP300*
cg08247776	2.86E-08	6.11E-06	6.11507	16	48422428	TSS5000	-3104	*SIAH1*	*SIAH1*
cg09167705	2.88E-08	6.13E-06	6.114026	1	33235619	Body;Body;Body;Body	4392	*KIAA1522*	*KIAA1522*
cg20323874	2.88E-08	6.14E-06	6.113772	14	1E+08		-18945		*EML1*
cg14329508	2.88E-08	6.14E-06	6.113508	20	18168289	3'UTR;Body	42547	*KAT14*	*KAT14*
cg17744368	2.88E-08	6.14E-06	6.113435	19	33369969	3'UTR	-9296	*CEP89*	*SLC7A9*
cg14283649	2.89E-08	6.14E-06	6.113248	9	36843876	Body;Body;Body;Body;Body;Body;Body;Body;Body;Body;Body;Body;Body	-20280	*PAX5*	*MIR4475*
cg02929681	2.89E-08	6.14E-06	6.11303	9	95479924	Body;Body	47191	*BICD2*	*BICD2*
cg09890930	2.89E-08	6.14E-06	6.113023	12	56149396	3'UTR;Body;Body;TES5000	12491	*GDF11;SARNP*	*GDF11*
cg25513712	2.89E-08	6.14E-06	6.11292	8	23750098		-37779		*STC1*
cg21712732	2.89E-08	6.15E-06	6.112659	7	1.58E+08	Body;Body;Body;Body;Body	89402	*PTPRN2*	*MIR595*
cg18830687	2.90E-08	6.15E-06	6.112274	17	66298463	Body;Body;Body;Body;Body;Body;Body;Body;Body;Body;Body;Body;Body	10188	*ARSG*	*ARSG*
cg00153306	2.90E-08	6.15E-06	6.112216	3	46741492	Body;Body;TSS1500	-1330	*TMIE*	*TMIE*
cg10437415	2.90E-08	6.15E-06	6.112113	7	1.02E+08	Body;Body;Body;Body;Body;Body;Body	99346	*CUX1*	*CUX1*
cg17271030	2.90E-08	6.15E-06	6.112073	10	1.35E+08		10109		*ADGRA1-AS1*
cg12266049	2.90E-08	6.15E-06	6.112067	11	69461334	Body	5411	*CCND1*	*CCND1*
cg13512537	2.90E-08	6.15E-06	6.112047	8	22265999	Body;Body;Body;Body;Body;Body;Body;Body;Body;Body	15651	*SLC39A14*	*SLC39A14*
cg27604897	2.91E-08	6.16E-06	6.11154	17	48266770	Body	12230	*COL1A1*	*COL1A1*
cg06576318	2.91E-08	6.16E-06	6.111272	8	1.34E+08	3'UTR;3'UTR;3'UTR;3'UTR	46519	*NDRG1*	*CCN4*
cg10004481	2.91E-08	6.16E-06	6.111093	14	92893598	Body;Body;Body	-86526	*SLC24A4*	*RIN3*
cg23845400	2.91E-08	6.16E-06	6.110977	6	36343668	Body;Body;Body;Body;1stExon;Body;1stExon;Body	11778	*ETV7*	*ETV7*
cg10382148	2.92E-08	6.17E-06	6.110682	1	2.1E+08	Body;Body;TES5000;Body;TES5000;3'UTR;TES5000;TES5000	3722	*TRAF3IP3;C1orf74;IRF6*	*C1orf74*
cg15190834	2.92E-08	6.18E-06	6.110296	7	34066196	Body;Body	121046	*BMPER*	*BMPER*
cg18179621	2.92E-08	6.18E-06	6.110183	3	1.4E+08	Body	38002	*CLSTN2*	*CLSTN2*
cg03066081	2.93E-08	6.18E-06	6.110022	17	77784036		-13160		*CBX8*
cg00222684	2.93E-08	6.18E-06	6.109938	3	36902435	Body;Body	-38444	*TRANK1*	*LINC02033*
cg13082816	2.93E-08	6.18E-06	6.109609	1	27702055	TSS1500;TSS1500;TES5000;TES5000	-712	*FCN3;CD164L2*	*FCN3*

Probe	p_adj	q_val	t	Chr	Pos.hg19	locations	dist2TSS	gene.Illumina	gene.nearest
cg24953213	2.93E-08	6.18E-06	6.109593	5	172140	Body	-19469	*PLEKHG4B*	*LRRC14B*
cg17964305	2.93E-08	6.18E-06	6.109474	8	9947360	Body;Body	-5800	*MSRA*	*MSRA*
cg06269204	2.93E-08	6.18E-06	6.109432	7	1.08E+08	Body;Body;Body;Body;TSS5000;TSS5000;Body;Body;Body;Body;Body;Body;Body;Body;Body;Body;Body;Body;Body;Body;Body;Body;Body;Body;Body;Body;Body;Body;Body;Body;Body;Body;Body;Body;Body;Body;Body;Body;Body;Body;Body;Body;Body;Body;Body;Body;Body;Body;Body;Body;Body;Body;Body;Body;Body;Body;Body;Body;Body;Body;Body;Body;Body;Body;Body;Body;Body;Body;Body;Body;Body;Body;Body	-2077	*NRCAM*	*NRCAM*
cg04234543	2.94E-08	6.18E-06	6.10911	1	1.57E+08	3'UTR	8589	*NES*	*NES*
cg25669088	2.94E-08	6.19E-06	6.108961	8	786658	Body;Body	46568	*DLGAP2*	*LOC105377777*
cg10622644	2.94E-08	6.19E-06	6.108669	1	12538455	Body;Body	-28844	*VPS13D*	*SNORA59A*
cg02876129	2.95E-08	6.21E-06	6.108016	14	75394075	TSS5000;TSS5000;TSS5000;TSS5000;TSS5000;TSS5000;TSS5000;TSS5000;TSS5000;TSS5000	-4112	*RPS6KL1*	*RPS6KL1*
cg13087299	2.95E-08	6.21E-06	6.107815	6	1.68E+08	TSS5000;TSS5000	-4802	*UNC93A*	*UNC93A*
cg20452711	2.96E-08	6.21E-06	6.107621	1	10718221	Body;Body	138482	*CASZ1*	*CASZ1*
cg20905984	2.96E-08	6.22E-06	6.107169	6	30023112	Body;Body;Body;Body	3397	*ZNRD1ASP*	*ZNRD1ASP*
cg05031695	2.96E-08	6.22E-06	6.107134	4	15470372	TSS1500;TSS1500;TSS1500	-1116	*CC2D2A*	*CC2D2A*
cg09279679	2.97E-08	6.22E-06	6.106826	2	421661		70994		*LINC01874*
cg03405473	2.97E-08	6.22E-06	6.10653	5	1.5E+08	Body	1542	*ARSI*	*ARSI*
cg27062924	2.97E-08	6.22E-06	6.10649	7	95157323	Body;Body	42069	*ASB4*	*ASB4*
cg08626004	2.97E-08	6.22E-06	6.106483	19	2513687	3'UTR	37563	*GNG7*	*GADD45B*
cg09706243	2.99E-08	6.25E-06	6.105471	11	67121634	Body;TSS1500;TSS1500;TSS1500;TSS1500;TSS1500	-567	*LOC100130987;POLD4*	*POLD4*
cg02518899	2.99E-08	6.26E-06	6.104784	17	39742965	1stExon	182	*KRT14*	*KRT14*
cg14352983	3.00E-08	6.27E-06	6.104558	18	6414976	TSS200;TSS200;TSS200;TSS200;Body;TSS200;TSS200;TSS200;TSS200	-58	*L3MBTL4*	*L3MBTL4*
cg10083071	3.00E-08	6.27E-06	6.104479	6	1.51E+08	Body;Body;Body;Body;Body;Body;Body;Body;Body;Body	19523	*MTHFD1L*	*MTHFD1L*
cg23978062	3.01E-08	6.28E-06	6.103854	1	26357956	Body	9691	*EXTL1*	*EXTL1*
cg24664672	3.01E-08	6.29E-06	6.103196	7	44794445	Body;Body;TSS1500	-1341	*ZMIZ2*	*ZMIZ2*
cg12793706	3.02E-08	6.29E-06	6.103157	17	39638276	TSS1500;TES5000	-884	*KRT35;KRT36*	*KRT35*
cg07130639	3.02E-08	6.29E-06	6.103063	16	89145892		-14367		*ACSF3*
cg23701012	3.02E-08	6.29E-06	6.102978	17	49004644		-59305		*TOB1*
cg20611334	3.02E-08	6.30E-06	6.102747	1	33209453	Body	2023	*KIAA1522*	*KIAA1522*
cg26976112	3.03E-08	6.30E-06	6.102337	5	1.7E+08	Body	1882	*LINC01187*	*LINC01187*
cg20536794	3.03E-08	6.31E-06	6.102134	5	1.54E+08		53774		*MIR3141*
cg20248691	3.04E-08	6.32E-06	6.10155	11	532330	3'UTR;3'UTR;3'UTR;3'UTR	3237	*HRAS*	*HRAS*
cg01956386	3.04E-08	6.32E-06	6.101338	4	5056767	Body;Body;Body	3561	*STK32B*	*STK32B*
cg22588445	3.04E-08	6.33E-06	6.101041	7	1.57E+08	Body;Body;Body;Body;Body	-56051	*PTPRN2*	*MIR153-2*
cg00640315	3.04E-08	6.33E-06	6.100912	22	19644159		-57866		*SEPTIN5*
cg20557069	3.05E-08	6.33E-06	6.100726	21	47646604	Body;Body;Body;Body;TSS5000;TSS5000;TSS5000;TSS5000	2084	*LSS;MCM3AP-AS1*	*LSS*
cg05655953	3.05E-08	6.33E-06	6.100507	10	1.24E+08	Body;Body;Body;Body	61355	*TACC2*	*TACC2*
cg24553773	3.06E-08	6.34E-06	6.099984	1	53746271	Body;Body;Body;Body	41990	*LRP8*	*LOC100507564*
cg14879279	3.06E-08	6.34E-06	6.099952	11	68427020		-24991		*GAL*
cg08136042	3.06E-08	6.34E-06	6.099811	12	52213077	3'UTR	12624	*FIGNL2*	*FIGNL2*
cg06847237	3.06E-08	6.35E-06	6.099479	16	55094729		129619		*IRX5*
cg01523600	3.06E-08	6.35E-06	6.09942	8	22959713	Body;TSS1500	-613	*LOC254896;TNFRSF10C*	*TNFRSF10C*
cg06931637	3.07E-08	6.35E-06	6.09926	16	90125163	Body	-10972	*PRDM7*	*URAHP*
cg10895168	3.07E-08	6.35E-06	6.099114	8	67373171	Body	28433	*ADHFE1*	*ADHFE1*
cg27190943	3.07E-08	6.35E-06	6.099108	10	79965132		-43249		*LINC00856*
cg02180633	3.07E-08	6.35E-06	6.098879	4	680114	TES5000;TES5000;Body;Body;Body	2826	*MYL5;SLC49A3*	*SLC49A3*
cg19094333	3.08E-08	6.36E-06	6.098623	7	93697091		-63424		*BET1*
cg00423398	3.08E-08	6.36E-06	6.098501	3	38038969	Body;Body;Body;Body;Body	3341	*VILL*	*VILL*
cg22113065	3.08E-08	6.37E-06	6.098084	5	74672346	Body;Body;Body	39302	*CERT1*	*HMGCR*
cg22354030	3.09E-08	6.37E-06	6.097832	8	1938554	Body	16712	*KBTBD11*	*KBTBD11*
cg08462108	3.10E-08	6.39E-06	6.096911	11	70680470	Body	-28424	*SHANK2*	*SHANK2-AS3*
cg18501945	3.10E-08	6.40E-06	6.096556	8	42292924	Body;Body;Body	43527	*SLC20A2*	*VDAC3*
cg01215211	3.10E-08	6.40E-06	6.096508	2	1.74E+08	Body;TSS5000	-2151	*RAPGEF4-AS1;RAPGEF4*	*RAPGEF4*
cg17453961	3.11E-08	6.40E-06	6.096314	6	1.6E+08		-40175		*FNDC1*
cg24181216	3.11E-08	6.41E-06	6.095755	14	21991414	Body;Body;Body;Body;Body;Body	3066	*SALL2*	*SALL2*
cg08330031	3.12E-08	6.42E-06	6.095251	10	1.05E+08	Body;3'UTR;3'UTR	-5723	*BORCS7-ASMT;BORCS7*	*AS3MT*
cg23756143	3.12E-08	6.43E-06	6.095032	11	66643501	Body;Body;Body	18967	*PC*	*LRFN4*
cg05233128	3.13E-08	6.44E-06	6.094524	19	3659669	Body;Body;Body	-32856	*PIP5K1C*	*CACTIN*
cg07147573	3.13E-08	6.44E-06	6.094471	3	42918806	TSS1500	-1435	*CYP8B1*	*CYP8B1*
cg25397111	3.14E-08	6.45E-06	6.093711	17	2592953	TES5000;3'UTR;3'UTR;3'UTR;TES5000	3260	*PAFAH1B1;CLUH;MIR6776*	*MIR6776*
cg14311251	3.14E-08	6.46E-06	6.093577	13	1.11E+08	Body;Body	46386	*COL4A1*	*COL4A1*
cg03510477	3.15E-08	6.46E-06	6.09345	6	1.69E+08		-44373		*DACT2*

Probe	p_adj	q_val	t	Chr	Pos.hg19	locations	dist2TSS	gene.Illumina	gene.nearest
cg05037360	3.15E-08	6.46E-06	6.093165	1	1.16E+08	TSS200	-153	*CASQ2*	*CASQ2*
cg06714705	3.15E-08	6.46E-06	6.093091	11	7506940	Body;Body;5'UTR;TSS200;TSS200	-56	*OLFML1*	*OLFML1*
cg23437810	3.15E-08	6.46E-06	6.092845	21	46816585		-8494		*COL18A1*
cg16911872	3.16E-08	6.46E-06	6.092608	7	1.53E+08		134339		*ACTR3B*
cg14088119	3.16E-08	6.46E-06	6.092593	5	1.6E+08	TSS1500;TSS1500	-355	*LINC02159*	*LINC02159*
cg08756885	3.16E-08	6.47E-06	6.092418	6	14178192		60190		*CD83*
cg21660452	3.16E-08	6.47E-06	6.092116	11	64397807	Body;Body;Body	13210	*NRXN2*	*NRXN2*
cg02128981	3.17E-08	6.47E-06	6.091969	11	58904901	Body	-5317	*FAM111A-DT*	*FAM111A*
cg02029665	3.17E-08	6.47E-06	6.091957	22	46441799	TSS1500	-1051	*LINC00899*	*LINC00899*
cg27466235	3.17E-08	6.48E-06	6.091443	4	77625095	Body	128392	*SHROOM3*	*MIR548AH*
cg03494692	3.17E-08	6.48E-06	6.091372	16	51498587		-297842		*LINC01571*
cg11338178	3.19E-08	6.50E-06	6.090554	3	72646997		66403		*LOC105377162*
cg13116478	3.19E-08	6.50E-06	6.090433	1	9217203	Body	-5367	*MIR34AHG*	*MIR34A*
cg00682158	3.19E-08	6.50E-06	6.090237	6	1.6E+08	Body;Body	18363	*PNLDC1*	*PNLDC1*
cg26700702	3.20E-08	6.52E-06	6.089651	16	1960348	TES5000	8068	*HS3ST6*	*HS3ST6*
cg03141815	3.20E-08	6.52E-06	6.089649	7	1.02E+08	Body;Body;Body;Body;Body;Body;Body	219524	*CUX1*	*CUX1*
cg11752320	3.20E-08	6.52E-06	6.089304	17	60741166	Body	36136	*MRC2*	*MRC2*
cg04428290	3.21E-08	6.53E-06	6.089085	16	88883363	Body;Body;Body	-5027	*GALNS*	*APRT*
cg03037620	3.21E-08	6.53E-06	6.088816	10	79699612		13043		*DLG5-AS1*
cg01386335	3.21E-08	6.53E-06	6.088632	7	17704951		-106418		*LOC101927630*
cg20633203	3.22E-08	6.53E-06	6.088357	8	8239143	1stExon;1stExon;Body	4611	*PRAG1*	*PRAG1*
cg22039287	3.22E-08	6.53E-06	6.088293	18	40696075	TSS1500;TSS1500	-418	*RIT2*	*RIT2*
cg08835301	3.22E-08	6.53E-06	6.088276	7	30585918	TES5000	31477	*GARS-DT*	*GARS-DT*
cg20220737	3.22E-08	6.53E-06	6.088254	8	1.33E+08	Body	9415	*OC90*	*OC90*
cg06041759	3.22E-08	6.54E-06	6.087898	11	528924	TES5000;TES5000;TES5000;TES5000	6643	*HRAS*	*HRAS*
cg04614459	3.22E-08	6.54E-06	6.087833	10	1.04E+08		23519		*TRIM8*
cg11060393	3.22E-08	6.54E-06	6.087686	20	50380560	Body	4369	*ATP9A*	*ATP9A*
cg15061647	3.23E-08	6.54E-06	6.087665	7	1.58E+08	Body;Body;Body;Body;Body	76296	*PTPRN2*	*MIR595*
cg23530031	3.23E-08	6.54E-06	6.087502	11	7541099	Body;Body;Body;Body;Body;Body;Body	5870	*PPFIBP2*	*PPFIBP2*
cg07917546	3.23E-08	6.54E-06	6.087432	16	86496223		46243		*FENDRR*
cg02225988	3.23E-08	6.54E-06	6.087337	8	1.41E+08	Body;Body;Body	-36978	*TRAPPC9*	*PEG13*
cg04773926	3.23E-08	6.54E-06	6.087077	7	2697363	Body	-21792	*TTYH3*	*AMZ1*
cg16767170	3.23E-08	6.54E-06	6.087015	8	87351999	TSS5000	-2776	*WWP1*	*WWP1*
cg00792251	3.24E-08	6.55E-06	6.086632	1	2.24E+08	TSS1500	-389	*CAPN2*	*CAPN2*
cg02414710	3.24E-08	6.55E-06	6.086404	1	2.33E+08	1stExon	1212	*SIPA1L2*	*SIPA1L2*
cg10848291	3.25E-08	6.55E-06	6.086251	6	73334702	Body;Body;Body;Body;Body	3132	*KCNQ5*	*KCNQ5*
cg26960224	3.25E-08	6.55E-06	6.086245	14	24509703	Body;Body;Body;Body;Body;Body;Body;Body	4050	*DHRS4L1*	*DHRS4L1*
cg24181174	3.25E-08	6.55E-06	6.085974	10	1.24E+08	Body;Body;Body;Body;Body	-22283	*TACC2*	*TACC2*
cg22222644	3.25E-08	6.55E-06	6.085742	19	51883242	3'UTR;3'UTR	-7334	*LIM2*	*NKG7*
cg05919312	3.25E-08	6.55E-06	6.085712	11	7694163	TSS200;Body;Body;Body	-41	*CYB5R2*	*CYB5R2*
cg13577778	3.25E-08	6.55E-06	6.085685	14	69481517		35119		*ACTN1-AS1*
cg06321213	3.25E-08	6.55E-06	6.085567	16	86009621		57669		*MIR6774*
cg24524895	3.25E-08	6.55E-06	6.085563	15	90229590	Body;Body;Body;TSS5000;TSS5000;TSS5000	4395	*PEX11A;WDR93*	*PEX11A*
cg23677712	3.26E-08	6.55E-06	6.085317	2	9617768	TES5000;TES5000;TES5000;TES5000;TES5000;Body;Body;Body;Body;Body	2585	*CPSF3;IAH1*	*IAH1*
cg08383930	3.26E-08	6.55E-06	6.085299	2	2.07E+08		162927		*INO80D*
cg03344747	3.26E-08	6.55E-06	6.085245	13	43989172	Body;Body;Body;Body;Body;Body;Body;Body;Body;Body;Body	-33815	*ENOX1*	*ENOX1-AS2*
cg22664152	3.26E-08	6.56E-06	6.084971	19	56726303	TES5000;TES5000;TES5000;TES5000	9021	*ZSCAN5A*	*ZSCAN5C*
cg23941077	3.27E-08	6.56E-06	6.084762	2	2.2E+08	TSS5000;Body;Body;Body	3560	*RNF25;STK36*	*STK36*
cg12374353	3.27E-08	6.57E-06	6.084403	3	1.55E+08	Body	8516	*MME*	*MME*
cg19732795	3.27E-08	6.57E-06	6.084318	2	71160396	3'UTR;TSS5000	-2633	*VAX2;ATP6V1B1*	*ATP6V1B1*
cg11043918	3.28E-08	6.57E-06	6.083932	8	81191306		37683		*MIR5708*
cg04132510	3.28E-08	6.58E-06	6.083509	1	3044741	Body;Body;TES5000	203	*PRDM16;MIR4251*	*MIR4251*
cg27175302	3.29E-08	6.59E-06	6.083205	4	71586685	Body;TSS1500;TSS1500;TSS1500	-995	*RUFY3*	*RUFY3*
cg02072002	3.29E-08	6.59E-06	6.08318	16	4736795	Body;3'UTR;3'UTR;Body;Body	-6898	*MGRN1*	*NUDT16L1*
cg26899811	3.30E-08	6.60E-06	6.082625	1	2.03E+08	3'UTR;3'UTR;3'UTR	-17947	*KDM5B*	*SYT2*
cg00065385	3.30E-08	6.60E-06	6.0826	9	1.12E+08	TSS1500	-1150	*ACTL7A*	*ACTL7A*
cg00697970	3.31E-08	6.63E-06	6.081475	14	1.04E+08	TSS1500	-1233	*TDRD9*	*TDRD9*
cg04115136	3.31E-08	6.63E-06	6.081395	1	32232407	TSS5000;TSS5000;TSS5000	-2466	*ADGRB2*	*ADGRB2*
cg15821214	3.32E-08	6.63E-06	6.081096	2	95939815	TSS1500;TSS1500;TSS1500;TSS1500	-381	*PROM2*	*PROM2*
cg20250908	3.32E-08	6.63E-06	6.081049	11	65032183	Body	2751	*POLA2*	*POLA2*

Probe	p_adj	q_val	t	Chr	Pos.hg19	locations	dist2TSS	gene.Illumina	gene.nearest
cg17779676	3.32E-08	6.63E-06	6.080839	17	55982124	TSS1500;Body	-1374	*CUEDC1*	*CUEDC1*
cg21130124	3.32E-08	6.63E-06	6.080807	10	5542328	TSS1500	-795	*CALML5*	*CALML5*
cg17972058	3.32E-08	6.63E-06	6.080793	1	1.6E+08	TSS1500;TSS1500	-431	*SLAMF8*	*SLAMF8*
cg03083680	3.34E-08	6.66E-06	6.07955	9	1.39E+08	Body;Body;Body	11906	*PMPCA*	*PMPCA*
cg18480033	3.34E-08	6.66E-06	6.079356	2	2.43E+08	Body	-9976	*GAL3ST2*	*NEU4*
cg10952649	3.34E-08	6.66E-06	6.079347	3	1.41E+08	1stExon	425	*GRK7*	*GRK7*
cg17415650	3.35E-08	6.67E-06	6.078914	12	1.13E+08	3'UTR	-5754	*OAS1*	*OAS3*
cg26279783	3.35E-08	6.67E-06	6.078791	6	33143356	Body;Body;Body	16888	*COL11A2*	*COL11A2*
cg22728186	3.36E-08	6.67E-06	6.078495	1	10712967	Body;Body	143736	*CASZ1*	*CASZ1*
cg18705098	3.36E-08	6.67E-06	6.078469	10	1.35E+08	Body	19826	*CFAP46*	*CFAP46*
cg18051668	3.36E-08	6.67E-06	6.078354	17	55126941	TSS5000	4103	*RNF126P1*	*RNF126P1*
cg04710179	3.36E-08	6.67E-06	6.078268	4	37891376	TSS1500;TSS1500	-1328	*TBC1D1*	*TBC1D1*
cg01546472	3.37E-08	6.69E-06	6.077598	4	1.84E+08	3'UTR	115438	*TENM3*	*DCTD*
cg01108815	3.38E-08	6.70E-06	6.077078	7	1.4E+08		28395		*KDM7A-DT*
cg27468270	3.38E-08	6.70E-06	6.077044	2	642539		34165		*TMEM18*
cg02565132	3.38E-08	6.70E-06	6.076819	2	1.1E+08	Body	46203	*EDAR*	*EDAR*
cg13325066	3.38E-08	6.70E-06	6.076789	7	2499932		16085		*GRIFIN*
cg07117596	3.38E-08	6.71E-06	6.076613	1	2.33E+08	Body	31697	*MAP3K21*	*MAP3K21*
cg10071113	3.39E-08	6.71E-06	6.076377	2	3328376	Body;Body;Body	53213	*EIPR1*	*EIPR1*
cg24359048	3.39E-08	6.71E-06	6.076281	1	59650472		-37993		*HSD52*
cg12547943	3.39E-08	6.71E-06	6.075973	1	18700971	Body	-106452	*IGSF21*	*KLHDC7A*
cg03196440	3.40E-08	6.72E-06	6.07578	20	62583946	TSS1500;TSS1500;TSS1500;Body;Body;Body;Body;Body;Body;Body;Body;Body;Body;Body;Body;Body;Body;Body;Body;TSS1500;TSS5000	-790	*UCKL1;UCKL1-AS1;ZNF512B*	*UCKL1-AS1*
cg02340395	3.41E-08	6.74E-06	6.074841	22	24586447	TSS5000	8993	*SUSD2*	*SUSD2*
cg25007511	3.41E-08	6.74E-06	6.074596	11	1.09E+08		-298411		*C11orf87*
cg10732094	3.41E-08	6.74E-06	6.074558	19	45957466	TSS5000;TSS5000;TSS5000;TSS5000	-2661	*ERCC1*	*ERCC1*
cg13131859	3.42E-08	6.76E-06	6.073891	4	4323012	Body;Body;Body	31035	*ZBTB49*	*ZBTB49*
cg19404567	3.43E-08	6.76E-06	6.073818	6	33049494	TSS1500;TSS1500;Body	-969	*HLA-DPA1;HLA-DPB1*	*HLA-DPA1*
cg18438345	3.43E-08	6.76E-06	6.073764	6	41009313	TSS5000;TSS1500;TSS1500	-892	*UNC5CL;TSPO2*	*TSPO2*
cg00908824	3.44E-08	6.78E-06	6.072973	6	33238058	5'UTR;5'UTR;5'UTR;Body;TSS5000	1595	*VPS52;RPS18*	*VPS52*
cg27177415	3.44E-08	6.78E-06	6.072912	17	79412681	Body	5533	*BAHCC1*	*MIR3186*
cg18132405	3.44E-08	6.78E-06	6.072854	3	1.9E+08	TSS5000;TSS200;Body	-78	*P3H2;P3H2-AS1*	*P3H2*
cg07656641	3.44E-08	6.78E-06	6.072674	22	50018876	Body;Body;Body	32291	*C22orf34*	*C22orf34*
cg03788230	3.44E-08	6.78E-06	6.072514	10	1.26E+08	Body;Body;Body;Body	35510	*CHST15*	*CHST15*
cg12360084	3.45E-08	6.78E-06	6.072328	22	21321273	TSS5000;Body;Body;TSS1500;TSS1500	-269	*LINC01637;AIFM3*	*AIFM3*
cg11857626	3.45E-08	6.78E-06	6.071938	3	51909628	Body;TSS200	-11	*IQCF5-AS1;IQCF5*	*IQCF5*
cg12178147	3.45E-08	6.78E-06	6.071916	12	1.1E+08	Body	22538	*ACACB*	*ACACB*
cg05083202	3.45E-08	6.78E-06	6.071883	21	40146149	TSS1500	-748	*LINC00114*	*LINC00114*
cg21533830	3.45E-08	6.78E-06	6.071871	11	1.17E+08	Body	102913	*DSCAML1*	*CEP164*
cg10734246	3.46E-08	6.79E-06	6.071624	11	1.23E+08	Body;Body;Body;Body;Body;Body;Body;Body	46966	*GRAMD1B*	*SCN3B*
cg06744481	3.47E-08	6.81E-06	6.070689	1	5817321		105480		*MIR4689*
cg05920345	3.47E-08	6.81E-06	6.070527	6	44282149	TSS1500	-1065	*AARS2*	*AARS2*
cg26808779	3.48E-08	6.81E-06	6.070387	11	1.19E+08		112157		*NECTIN1*
cg00374015	3.48E-08	6.81E-06	6.070276	3	1.27E+08	Body	29790	*PLXNA1*	*PLXNA1*
cg22948684	3.48E-08	6.81E-06	6.070247	1	15222078	Body;Body;Body	-28546	*KAZN*	*KAZN*
cg22816131	3.48E-08	6.82E-06	6.069916	5	76132547	TSS5000	-13291	*F2RL1*	*S100Z*
cg27248571	3.49E-08	6.83E-06	6.069673	6	31685542	Body;TSS5000;TSS5000;Body;TSS5000	2410	*LY6G6F-LY6G6D;LY6G6E;LY6G6D;LY6G6C*	*LY6G6D*
cg03777083	3.49E-08	6.83E-06	6.069558	17	40932199	TSS5000;TSS1500;TSS1500	-428	*VPS25;WNK4*	*WNK4*
cg15123159	3.50E-08	6.84E-06	6.068868	6	43045686	TSS5000;TSS5000;TSS5000;TSS5000;TSS5000;Body;Body;Body;Body;Body;Body;Body	1135	*KLC4;PTK7*	*PTK7*
cg18575863	3.50E-08	6.84E-06	6.068848	6	1.09E+08	Body;Body	-29158	*ARMC2*	*ARMC2-AS1*
cg08445039	3.50E-08	6.84E-06	6.068736	7	32997659	Body;Body	608	*FKBP9*	*FKBP9*
cg16904520	3.51E-08	6.85E-06	6.068368	2	2.31E+08		-11691		*DNER*
cg05151739	3.51E-08	6.85E-06	6.068338	1	6527809	TSS5000;TSS5000;TSS5000;TSS5000;TSS5000;TSS5000;Body;Body;Body;Body;Body;Body;Body;Body	-1554	*TNFRSF25;PLEKHG5*	*TNFRSF25*
cg14241609	3.52E-08	6.86E-06	6.067733	11	1.29E+08	1stExon;1stExon	-5700	*KCNJ5*	*C11orf45*
cg24554789	3.52E-08	6.86E-06	6.067707	1	32046825	Body;Body;Body	3080	*TINAGL1*	*TINAGL1*
cg08750547	3.53E-08	6.87E-06	6.067166	15	1.02E+08	Body	127492	*LRRK1*	*LRRK1*
cg05132782	3.54E-08	6.90E-06	6.066083	7	1.5E+08	TSS5000	-4349	*LINC00996*	*LINC00996*
cg09070415	3.55E-08	6.90E-06	6.065835	1	32041052	TSS1500;TSS1500;TSS5000	-1033	*TINAGL1*	*TINAGL1*
cg20086597	3.55E-08	6.90E-06	6.065834	1	2.43E+08	TSS1500	-271	*LINC01347*	*LINC01347*
cg13829638	3.55E-08	6.90E-06	6.065728	9	1.28E+08	TSS5000;TSS5000;TSS5000;TSS5000;Body;TSS5000	3877	*WDR38;RPL35;ARPC5L*	*RPL35*

Probe	p_adj	q_val	t	Chr	Pos.hg19	locations	dist2TSS	gene.Illumina	gene.nearest
cg11364290	3.55E-08	6.90E-06	6.06564	7	1.28E+08	TSS1500	-321	*MIR129-1*	*MIR129-1*
cg16277885	3.55E-08	6.90E-06	6.065424	7	899935	TES5000;Body;Body;Body;Body;Body;Body;Body;Body;Body;Body;Body;Body;Body;Body;Body;Body;Body;Body;Body;Body;Body;Body;Body;Body;Body;Body;Body;Body;Body;Body;Body;Body;Body;Body;Body;Body;Body;Body;Body;Body;Body;Body;Body;Body;Body;Body;Body;Body;Body;Body;Body;Body;Body;Body;Body;Body;Body;Body;Body;Body;Body;Body;Body;Body;Body;Body;Body;Body;Body	-16255	*SUN1*	*GET4*
cg18943655	3.55E-08	6.90E-06	6.065401	1	32209578	Body;Body;Body	14758	*ADGRB2*	*MIR4254*
cg04632683	3.55E-08	6.91E-06	6.065237	19	54620122	TSS5000;TSS1500;Body	989	*TFPT;PRPF31*	*PRPF31*
cg21937244	3.56E-08	6.91E-06	6.064978	14	1.03E+08	Body	17420	*CDC42BPB*	*AMN*
cg24510285	3.56E-08	6.91E-06	6.064808	12	1948648	Body;TES5000;TES5000;TES5000	18918	*CACNA2D4;LRTM2*	*LRTM2*
cg14184130	3.56E-08	6.92E-06	6.064615	9	38437270		-12819		*IGFBPL1*
cg17981101	3.57E-08	6.92E-06	6.064548	7	1.01E+08	TES5000;TSS5000;TSS5000;TSS200;TSS200	-122	*MUC12;MUC12-AS1;MUC17*	*MUC17*
cg16266893	3.57E-08	6.92E-06	6.064278	1	39451509		-5406		*AKIRIN1*
cg20820557	3.57E-08	6.93E-06	6.064012	6	53453217		-15144		*LOC101927136*
cg23245711	3.58E-08	6.93E-06	6.06388	3	48413678	TSS200;TSS200;TSS200	-30	*FBXW12*	*FBXW12*
cg21988972	3.58E-08	6.93E-06	6.063558	2	1.01E+08	TSS1500	-571	*AFF3*	*AFF3*
cg02711147	3.59E-08	6.94E-06	6.063107	10	1.31E+08	TSS5000	-4775	*MGMT*	*MGMT*
cg14829953	3.60E-08	6.96E-06	6.062443	4	1.54E+08	Body;TSS5000	-1677	*LOC729870;FHDC1*	*FHDC1*
cg03107179	3.60E-08	6.96E-06	6.062384	6	1.67E+08	Body;Body;Body	-24637	*RPS6KA2*	*RPS6KA2*
cg01566282	3.60E-08	6.96E-06	6.062374	19	53794975	TSS1500	-288	*BIRC8*	*BIRC8*
cg03260021	3.60E-08	6.96E-06	6.062015	12	1.28E+08		-30344		*LOC105370068*
cg04559293	3.61E-08	6.97E-06	6.061687	12	39727030	Body;Body;Body;Body	109892	*KIF21A*	*KIF21A*
cg02016467	3.62E-08	6.98E-06	6.061311	16	801549		-9184		*MSLN*
cg17279410	3.62E-08	6.98E-06	6.061271	18	59998729	Body;Body;Body;Body;Body	6187	*TNFRSF11A*	*TNFRSF11A*
cg26162385	3.62E-08	6.98E-06	6.061225	1	37271723	Body	228393	*GRIK3*	*GRIK3*
cg08415731	3.62E-08	6.98E-06	6.060913	6	18273503		-8742		*DEK*
cg25320749	3.63E-08	6.99E-06	6.060411	6	28494732	Body;Body;TSS1500	944	*GPX5*	*GPX5*
cg26364080	3.64E-08	7.01E-06	6.059929	2	2.32E+08	Body;Body;Body;Body;Body;Body;Body;Body;Body;Body	57970	*ARMC9*	*ARMC9*
cg14467690	3.64E-08	7.01E-06	6.059596	9	1.35E+08	TES5000	83263	*NTNG2*	*NTNG2*
cg26306056	3.65E-08	7.02E-06	6.05909	6	32972989	3'UTR	4379	*HLA-DOA*	*HLA-DOA*
cg05673892	3.65E-08	7.02E-06	6.059043	3	38033934	TSS1500;TSS5000;TSS5000;TSS5000;TSS5000	-1143	*VILL*	*VILL*
cg02937055	3.65E-08	7.02E-06	6.058931	3	1.71E+08	Body;Body	38648	*PLD1*	*PLD1*
cg02282892	3.66E-08	7.03E-06	6.058777	5	34042976	Body;1stExon;Body;Body	342	*C1QTNF3-AMACR;C1QTNF3*	*C1QTNF3*
cg03789372	3.66E-08	7.03E-06	6.058527	8	27125756		-9835		*STMN4*
cg16760008	3.66E-08	7.03E-06	6.058466	17	77708928	Body	4224	*ENPP7*	*ENPP7*
cg14608550	3.66E-08	7.03E-06	6.05843	17	75398014	Body;Body;Body;Body;Body;Body;TSS5000;TSS5000	-3144	*SEPTIN9;MIR4316*	*SEPTIN9*
cg10222534	3.67E-08	7.03E-06	6.05803	19	48958894	3'UTR;5'UTR	64	*GRWD1;KCNJ14*	*KCNJ14*
cg01226614	3.67E-08	7.03E-06	6.058017	13	44947593	TSS200;TSS1500;TSS1500	-192	*SERP2*	*SERP2*
cg05707492	3.67E-08	7.04E-06	6.057623	17	78833484	Body;Body	-54052	*RPTOR*	*LOC101928855*
cg16072340	3.68E-08	7.04E-06	6.057539	8	1.04E+08	Body;Body;Body	-39638	*BAALC*	*BAALC-AS1*
cg23158862	3.68E-08	7.05E-06	6.05732	15	25984561	Body	109411	*ATP10A*	*MIR4715*
cg26059839	3.68E-08	7.05E-06	6.057273	1	1988681	Body	6832	*PRKCZ*	*PRKCZ*
cg12515402	3.69E-08	7.05E-06	6.056946	8	1.45E+08	Body;TES5000	1944	*NRBP2;MIR6845*	*MIR6845*
cg00660765	3.69E-08	7.05E-06	6.056828	3	69371349	Body	63890	*FRMD4B*	*FRMD4B*
cg03328935	3.69E-08	7.05E-06	6.056682	1	2.02E+08	Body;TES5000	-33664	*NAV1;IPO9-AS1*	*MIR5191*
cg09699373	3.69E-08	7.05E-06	6.056587	16	84822712		-30878		*CRISPLD2*
cg15073112	3.69E-08	7.05E-06	6.056572	1	86989797		-22963		*CLCA4*
cg15627072	3.70E-08	7.06E-06	6.056284	1	2432621	Body;Body;Body	24868	*PLCH2*	*PLCH2*
cg20505372	3.70E-08	7.06E-06	6.056228	15	73916210	Body;Body;Body;Body	9520	*NPTN*	*NPTN*
cg23107916	3.70E-08	7.07E-06	6.05571	13	1.13E+08	TES5000;TES5000	8163	*LINC00354*	*LINC00354*
cg26065909	3.72E-08	7.09E-06	6.054842	1	1.51E+08	5'UTR;5'UTR;5'UTR	8	*SELENBP1*	*SELENBP1*
cg02362409	3.72E-08	7.09E-06	6.054633	4	76823713	TSS200	-108	*PPEF2*	*PPEF2*
cg21417459	3.72E-08	7.09E-06	6.054481	1	2.03E+08		-8539		*LINC01353*
cg03281369	3.73E-08	7.09E-06	6.05427	19	40266546	TSS1500	-687	*LEUTX*	*LEUTX*
cg23737263	3.73E-08	7.09E-06	6.054254	2	43328424	Body	1159	*LINC02580*	*LINC02580*
cg22930986	3.73E-08	7.09E-06	6.054235	3	59171831		-136073		*C3orf67*
cg15583738	3.73E-08	7.09E-06	6.054193	8	2176944		152276		*MIR7160*
cg04609841	3.73E-08	7.09E-06	6.054079	7	4012372	Body	-156944	*SDK1*	*SDK1*
cg21038780	3.73E-08	7.09E-06	6.054075	5	1.68E+08	Body;Body;Body	62215	*WWC1*	*WWC1*
cg24438135	3.73E-08	7.09E-06	6.054033	6	1.69E+08	Body;Body	170541	*SMOC2*	*SMOC2*
cg18692273	3.73E-08	7.09E-06	6.054029	1	2.01E+08	TSS1500;TSS1500;TSS1500;TSS1500;TSS1500;TSS1500;TSS1500;TES5000	-675	*TNNT2;LAD1*	*TNNT2*
cg24493568	3.74E-08	7.10E-06	6.053329	17	36709027	Body	53178	*SRCIN1*	*SRCIN1*

Probe	p_adj	q_val	t	Chr	Pos.hg19	locations	dist2TSS	gene.Illumina	gene.nearest
cg07723431	3.74E-08	7.10E-06	6.053311	19	2044540	Body;Body	6703	*MKNK2*	*MKNK2*
cg27209679	3.74E-08	7.10E-06	6.053242	6	1.43E+08	Body	46257	*HIVEP2*	*HIVEP2*
cg17247026	3.76E-08	7.11E-06	6.052601	10	1.35E+08	TSS5000;TSS5000	-3537	*CALY*	*CALY*
cg00636737	3.76E-08	7.11E-06	6.052597	20	62695569	Body;Body	1019	*TCEA2*	*TCEA2*
cg22855393	3.76E-08	7.11E-06	6.052354	8	74361427	Body;Body;Body;Body	29119	*STAU2*	*STAU2-AS1*
cg12385643	3.76E-08	7.11E-06	6.05235	2	2.35E+08	Body;Body;Body;Body;TSS200;TSS1500	-166	*UGT1A8;UGT1A10;UGT1A9;UGT1A7;UGT1A6*	*UGT1A6*
cg22600610	3.76E-08	7.11E-06	6.052344	1	21300625	Body;Body;Body;Body	14300	*EIF4G3*	*MIR1256*
cg11988128	3.76E-08	7.11E-06	6.052245	17	76449562	Body	-31429	*DNAH17*	*DNAH17-AS1*
cg10334509	3.76E-08	7.11E-06	6.052238	1	44573252		-11240		*KLF17*
cg05864627	3.76E-08	7.11E-06	6.052216	15	25323793	Body;TES5000;TES5000;TES5000;TSS1500;TSS1500;TSS5000;TSS5000;TSS5000	-410	*SNHG14;SNORD116-10;SNORD116-11;SNORD116-12;SNORD116-13;SNORD116-14;SNORD116-15;SNORD116-16;SNORD116-17*	*SNORD116-13*
cg19088141	3.76E-08	7.11E-06	6.052067	1	1.13E+08	Body;Body;Body	4565	*WNT2B*	*WNT2B*
cg22354363	3.77E-08	7.12E-06	6.051742	11	69514015	3'UTR	4775	*FGF19*	*FGF19*
cg08298584	3.77E-08	7.12E-06	6.051549	16	989477	Body;Body;Body;Body;Body;Body;Body;TES5000;TES5000	18445	*LMF1;LMF1-AS1*	*LMF1-AS1*
cg16279544	3.78E-08	7.13E-06	6.051223	10	1.21E+08		-12694		*BAG3*
cg02385411	3.78E-08	7.13E-06	6.0512	17	7674264	Body	16627	*DNAH2*	*RPL29P2*
cg02589568	3.78E-08	7.13E-06	6.051087	5	1.29E+08	Body	132943	*CHSY3*	*CHSY3*
cg07949217	3.79E-08	7.14E-06	6.050591	9	1.24E+08	Body	-7082	*DAB2IP*	*DAB2IP*
cg08066306	3.80E-08	7.15E-06	6.050081	3	1.08E+08	Body	40307	*LINC00636*	*LINC00636*
cg20303331	3.80E-08	7.15E-06	6.050007	17	36945575	Body	10583	*PIP4K2B*	*PIP4K2B*
cg11080731	3.80E-08	7.15E-06	6.049944	12	94939916		-15648		*MIR5700*
cg20152300	3.80E-08	7.16E-06	6.049604	6	32015389	Body;Body;TSS1500	-1484	*TNXB*	*TNXB*
cg10740594	3.81E-08	7.16E-06	6.049328	10	1.03E+08		-28693		*SLF2*
cg18274791	3.81E-08	7.16E-06	6.049213	5	484843	Body;Body;TES5000	11493	*SLC9A3;SLC9A3-AS1*	*SLC9A3-AS1*
cg13992493	3.81E-08	7.16E-06	6.049121	6	1.69E+08		250470		*SMOC2*
cg13927401	3.81E-08	7.16E-06	6.049054	7	99918120	Body;TES5000	12796	*SPDYE3;PMS2P1*	*SPDYE3*
cg17826043	3.82E-08	7.17E-06	6.048582	19	42299635	TSS1500;TSS1500;TSS1500	-883	*CEACAM3*	*CEACAM3*
cg21120413	3.82E-08	7.17E-06	6.048541	5	1.78E+08	Body	71274	*COL23A1*	*COL23A1*
cg18817444	3.82E-08	7.17E-06	6.048539	17	39306458	TSS1500	-381	*KRTAP4-5*	*KRTAP4-5*
cg11283729	3.83E-08	7.18E-06	6.048048	6	1.08E+08	Body	116252	*SOBP*	*SOBP*
cg10951736	3.83E-08	7.18E-06	6.048024	1	1.67E+08	Body	32436	*DUSP27*	*DUSP27*
cg26835568	3.83E-08	7.18E-06	6.047953	6	1.64E+08	Body;Body;Body;TSS1500	-667	*PACRG;PACRG-AS3*	*PACRG-AS3*
cg13298209	3.85E-08	7.20E-06	6.047082	7	6568476	Body	22591	*GRID2IP*	*GRID2IP*
cg06249421	3.85E-08	7.20E-06	6.04701	17	46567806		40567		*HOXB1*
cg02394247	3.85E-08	7.20E-06	6.046962	1	2836643		-101387		*ACTRT2*
cg05194636	3.85E-08	7.21E-06	6.046686	11	12098045		-34077		*MICAL2*
cg26369180	3.86E-08	7.21E-06	6.04647	1	1246414	TSS5000;Body;Body;Body;TES5000;TES5000;TES5000;TES5000;TES5000;TES5000	1532	*ACAP3;PUSL1;INTS11;MIR6727*	*MIR6727*
cg24475275	3.86E-08	7.21E-06	6.046386	6	30644278	TSS5000;TSS5000;TSS5000;3'UTR;3'UTR	-3448	*DHX16;PPP1R18*	*DHX16*
cg01315407	3.86E-08	7.21E-06	6.046109	4	1.22E+08	TSS1500;Body;Body	-701	*TNIP3*	*TNIP3*
cg14394265	3.86E-08	7.21E-06	6.046108	4	1.88E+08	Body	7139	*FAT1*	*FAT1*
cg07804260	3.87E-08	7.22E-06	6.045679	11	2397977	Body;Body;TSS1500	-569	*CD81-AS1;CD81*	*CD81*
cg07311566	3.87E-08	7.22E-06	6.045559	3	58632000	Body	-18663	*FAM3D*	*FAM107A*
cg17786902	3.87E-08	7.22E-06	6.045421	1	19740722	Body;Body;Body;Body;Body	70067	*CAPZB*	*CAPZB*
cg25052223	3.87E-08	7.22E-06	6.045366	10	16993958	Body	-134505	*CUBN*	*RSU1*
cg13443800	3.91E-08	7.28E-06	6.043348	9	1.3E+08		-5180		*STXBP1*
cg22040874	3.91E-08	7.28E-06	6.04329	1	4230824		-221784		*LINC01345*
cg04490024	3.92E-08	7.29E-06	6.042925	8	1.44E+08	TES5000	4724	*LY6L*	*LY6L*
cg24202922	3.92E-08	7.29E-06	6.04291	11	77296743	TSS5000;TSS5000	-3936	*AQP11*	*AQP11*
cg16270633	3.92E-08	7.29E-06	6.042775	15	91256830	Body;TSS5000;TSS5000;TSS5000;TSS5000	3541	*CRTC3-AS1;BLM*	*CRTC3-AS1*
cg16999495	3.92E-08	7.29E-06	6.042541	10	85995762	Body	5540	*LRIT1*	*LRIT1*
cg13968679	3.93E-08	7.30E-06	6.042381	10	1.05E+08	Body;TES5000	3621	*CALHM3;NEURL1-AS1*	*CALHM3*
cg04770020	3.93E-08	7.30E-06	6.042168	2	1544321	Body;Body;Body;Body;Body;Body	126142	*TPO*	*TPO*
cg25737411	3.94E-08	7.31E-06	6.041715	17	7286288	1stExon;1stExon	1881	*TNK1*	*TNK1*
cg05482956	3.95E-08	7.32E-06	6.041192	11	16635016	Body	125008	*SOX6*	*SOX6*
cg09319797	3.95E-08	7.33E-06	6.040828	16	12061715	TSS5000;3'UTR	2649	*NPIPB2;TNFRSF17*	*TNFRSF17*
cg03962200	3.95E-08	7.33E-06	6.040662	22	31518707	TSS1500;TSS1500;TSS1500;TSS1500;TSS1500;TSS1500	-201	*INPP5J*	*INPP5J*
cg13930220	3.95E-08	7.33E-06	6.040633	13	1.07E+08		48098		*LINC00443*

Probe	p_adj	q_val	t	Chr	Pos.hg19	locations	dist2TSS	gene.Illumina	gene.nearest
cg15518542	3.96E-08	7.34E-06	6.040468	10	1444784	Body	-124040	*ADARB2*	*ADARB2-AS1*
cg14321708	3.98E-08	7.37E-06	6.039325	9	1.37E+08	Body;Body	9819	*WDR5*	*RNU6ATAC*
cg18485070	3.98E-08	7.37E-06	6.039225	2	1.32E+08	TSS1500	-1278	*NOC2LP2*	*NOC2LP2*
cg10945695	3.98E-08	7.37E-06	6.03922	5	32871131		-76417		*LINC02120*
cg17434901	3.98E-08	7.37E-06	6.038978	11	1.34E+08	TSS5000	11702	*LINC02731*	*LINC02731*
cg25003658	3.99E-08	7.38E-06	6.03851	5	1.18E+08	Body;Body	-21759	*DTWD2*	*MIR1244-2*
cg17077641	3.99E-08	7.38E-06	6.038507	17	13499920	Body	5326	*HS3ST3A1*	*HS3ST3A1*
cg04784014	4.00E-08	7.38E-06	6.038276	7	5521290	Body;3'UTR;3'UTR;3'UTR	14258	*FBXL18*	*MIR589*
cg08649439	4.00E-08	7.38E-06	6.038248	7	4761868	Body	39943	*FOXK1*	*FOXK1*
cg06025671	4.00E-08	7.39E-06	6.037904	8	1.42E+08		-26191		*DENND3*
cg13424284	4.00E-08	7.39E-06	6.037865	1	26624129	Body;Body;Body	9066	*UBXN11*	*UBXN11*
cg19979750	4.01E-08	7.39E-06	6.037712	12	1.23E+08	Body	27991	*VPS37B*	*VPS37B*
cg26520908	4.01E-08	7.39E-06	6.037553	1	3191876	Body;Body	147338	*PRDM16*	*MIR4251*
cg11579646	4.01E-08	7.39E-06	6.037425	6	1.7E+08		-85629		*LOC102724511*
cg02500075	4.01E-08	7.39E-06	6.037385	3	1.79E+08		-17464		*ACTL6A*
cg20748397	4.02E-08	7.40E-06	6.036933	17	18062972	Body	-24169	*MYO15A*	*ALKBH5*
cg07283859	4.02E-08	7.41E-06	6.036612	5	14160853	Body;Body	17403	*TRIO*	*TRIO*
cg06817802	4.03E-08	7.42E-06	6.036161	5	1291319	Body;Body;Body;Body	3864	*TERT*	*TERT*
cg07450393	4.03E-08	7.42E-06	6.036118	17	78898262	Body;Body	46751	*RPTOR*	*LOC400627*
cg06373817	4.04E-08	7.43E-06	6.035593	15	93123126		-7633		*LINC00930*
cg03816507	4.04E-08	7.43E-06	6.035565	4	1217392	Body;Body	13485	*CTBP1*	*CTBP1-AS*
cg09301143	4.05E-08	7.44E-06	6.035081	2	95955715	Body;Body;3'UTR;Body	-7379	*PROM2*	*KCNIP3*
cg12367543	4.06E-08	7.45E-06	6.034849	22	48602194		-67981		*MIR3201*
cg17559785	4.06E-08	7.45E-06	6.03479	12	1.08E+08	Body;Body;Body;Body	31945	*BTBD11*	*BTBD11*
cg01395690	4.07E-08	7.48E-06	6.033803	19	50478330	3'UTR;Body;TSS5000;TSS5000	5410	*SIGLEC16;VRK3*	*SIGLEC16*
cg18877200	4.08E-08	7.48E-06	6.033652	6	29720927	TSS5000;TSS5000;TSS5000	-2002	*HLA-F-AS1;IFITM4P*	*IFITM4P*
cg22111818	4.08E-08	7.48E-06	6.033393	15	93588137	3'UTR;3'UTR;3'UTR;3'UTR;3'UTR;3'UTR	28248	*RGMA*	*RGMA*
cg01252713	4.08E-08	7.48E-06	6.033381	2	2.36E+08		-41722		*LINC01173*
cg01952788	4.09E-08	7.49E-06	6.033099	15	40663180	3'UTR	-11741	*DISP2*	*KNSTRN*
cg05621516	4.09E-08	7.49E-06	6.032954	6	99728990	3'UTR;3'UTR;Body;3'UTR;3'UTR;3'UTR;Body	68560	*FAXC*	*FAXC*
cg14776050	4.10E-08	7.50E-06	6.032326	11	74978433	Body;Body	26615	*ARRB1*	*TPBGL*
cg25663741	4.10E-08	7.50E-06	6.032087	15	49686764	Body	-28674	*FAM227B*	*FGF7*
cg10059178	4.10E-08	7.50E-06	6.032073	8	1.45E+08	1stExon	8961	*EPPK1*	*EPPK1*
cg06848073	4.10E-08	7.50E-06	6.032067	1	11713540	Body;TSS1500;TSS1500;TSS1500;TSS1500;TSS1500;TSS1500;TSS1500;TSS1500	-923	*FBXO2;FBXO44*	*FBXO44*
cg17023911	4.11E-08	7.51E-06	6.031676	22	49786029		151085		*MIR3667*
cg10962490	4.12E-08	7.51E-06	6.031405	1	54072456	Body;Body	127421	*GLIS1*	*GLIS1*
cg04324276	4.12E-08	7.51E-06	6.031392	17	17817462	Body;Body;Body;Body;Body;Body;Body;Body;Body	58252	*TOM1L2*	*TOM1L2*
cg25193782	4.12E-08	7.51E-06	6.031348	17	47976494		-51115		*TAC4*
cg09694272	4.12E-08	7.51E-06	6.031293	13	1E+08	TSS5000;TSS5000	-4034	*CLYBL*	*CLYBL*
cg13759446	4.13E-08	7.53E-06	6.030809	11	1.03E+08	Body;Body;Body	1013	*WTAPP1;MMP1*	*MMP1*
cg00359365	4.13E-08	7.53E-06	6.030637	1	26880547	Body;Body;Body;TSS1500	-485	*RPS6KA1;MIR1976*	*MIR1976*
cg22584718	4.13E-08	7.53E-06	6.030358	12	97512826		211583		*NEDD1*
cg02948259	4.14E-08	7.53E-06	6.030308	22	24988020	Body;Body	1020	*GGT1;LRRC75B*	*LRRC75B*
cg22442352	4.14E-08	7.54E-06	6.029923	5	1.49E+08	3'UTR;3'UTR;3'UTR	75859	*PPARGC1B*	*PPARGC1B*
cg08095452	4.14E-08	7.54E-06	6.029845	17	55958800	Body;Body	21950	*CUEDC1*	*CUEDC1*
cg27189723	4.15E-08	7.55E-06	6.029396	7	1.34E+08	Body;Body;Body	6207	*AKR1B1*	*AKR1B1*
cg16330786	4.16E-08	7.56E-06	6.029121	2	75326898	Body;Body	8960	*TACR1*	*MIR5000*
cg05687719	4.17E-08	7.57E-06	6.028632	16	86265226	Body	35440	*LINC01081*	*LINC01082*
cg07122009	4.17E-08	7.57E-06	6.028438	16	1611802	Body	-18725	*IFT140*	*LOC105371046*
cg25798827	4.17E-08	7.57E-06	6.028415	6	89788126	TSS5000	-2343	*PNRC1*	*PNRC1*
cg02531548	4.17E-08	7.58E-06	6.02823	2	8234915	Body	-117970	*LINC00299*	*LINC00298*
cg23588952	4.19E-08	7.60E-06	6.027154	8	2024300	Body;TSS1500	-368	*MYOM2;MIR7160*	*MIR7160*
cg12804910	4.19E-08	7.60E-06	6.027127	12	1.33E+08	Body;TSS5000	4784	*DDX51;NOC4L*	*DDX51*
cg23480479	4.19E-08	7.60E-06	6.027111	5	38665490	Body	54885	*LIFR-AS1*	*LINC01265*
cg12115087	4.20E-08	7.60E-06	6.026897	7	1.33E+08	Body	243992	*EXOC4*	*MIR6133*
cg03499581	4.20E-08	7.60E-06	6.026869	15	78384868	TSS200	-58	*SH2D7*	*SH2D7*
cg24159142	4.20E-08	7.60E-06	6.026825	20	50310568	Body	74361	*ATP9A*	*ATP9A*
cg02392813	4.20E-08	7.60E-06	6.026792	5	80594700	Body;Body;Body;TSS5000;TSS5000;TSS5000	2688	*CKMT2-AS1;ZCCHC9*	*CKMT2-AS1*
cg00329447	4.20E-08	7.60E-06	6.026697	8	1.45E+08	TSS5000;TSS200;Body;Body	-168	*PLEC*	*PLEC*
cg14210790	4.20E-08	7.60E-06	6.026567	15	70877277	Body	1668	*LINC02204*	*LINC02204*

Probe	p_adj	q_val	t	Chr	Pos.hg19	locations	dist2TSS	gene.Illumina	gene.nearest
cg07973507	4.21E-08	7.60E-06	6.026414	14	1.06E+08		8071		*MIR8071-1*
cg03525254	4.21E-08	7.60E-06	6.026382	1	1.1E+08	Body;Body;Body;Body;TSS5000;TSS5000;TSS5000;TSS5000;TSS5000	-3746	*ATXN7L2;CYB561D1*	*CYB561D1*
cg01963976	4.21E-08	7.60E-06	6.02629	14	21498561	TSS5000;TSS5000;TSS5000;TSS5000;TSS5000;Body;TSS5000;TSS5000;TSS5000;TSS5000;TSS5000;TSS5000;TSS5000;TSS5000;Body;TES5000	186	*NDRG2;TPPP2;RNASE13*	*TPPP2*
cg14412490	4.22E-08	7.63E-06	6.025538	11	77880406	Body;Body;TES5000	19266	*KCTD21-AS1;KCTD21*	*KCTD21*
cg05824174	4.22E-08	7.63E-06	6.025431	12	3142845	Body;Body;Body	-43675	*TEAD4*	*TSPAN9*
cg15687653	4.24E-08	7.64E-06	6.024819	1	6271667	Body	5489	*RNF207*	*RNF207*
cg09759523	4.24E-08	7.66E-06	6.024323	10	7621857	Body;Body;Body	39779	*ITIH5*	*ITIH5*
cg19566879	4.25E-08	7.66E-06	6.024267	6	16334128	Body;Body;Body	95311	*ATXN1*	*GMPR*
cg27047255	4.25E-08	7.66E-06	6.023929	1	2.04E+08	Body	34303	*PIK3C2B*	*PIK3C2B*
cg16306644	4.25E-08	7.66E-06	6.023926	7	1006943	3'UTR	8242	*COX19*	*COX19*
cg00709423	4.25E-08	7.66E-06	6.023919	7	1.51E+08	Body;Body;Body;Body;Body;Body;Body;Body	5110	*AGAP3*	*AGAP3*
cg21153004	4.25E-08	7.66E-06	6.023817	15	65207109	Body	3009	*ANKDD1A*	*ANKDD1A*
cg13713066	4.26E-08	7.67E-06	6.023295	7	1.29E+08	TSS1500;TES5000;TES5000	-325	*MIR182;MIR96;MIR183*	*MIR182*
cg10758165	4.26E-08	7.67E-06	6.02329	15	65392263	Body	6434	*UBAP1L*	*UBAP1L*
cg07317047	4.27E-08	7.67E-06	6.023055	3	69994982	Body;Body;Body;Body;Body;Body;Body;Body;Body;Body;Body;Body	9232	*MITF*	*MITF*
cg24734792	4.27E-08	7.67E-06	6.022944	10	64580360	TSS5000;TSS5000;TSS5000;TSS5000;TSS1500	-1433	*EGR2*	*EGR2*
cg24612498	4.27E-08	7.67E-06	6.022911	3	8572798	Body;Body;Body;Body	29290	*LMCD1*	*LMCD1*
cg07397503	4.27E-08	7.67E-06	6.022873	13	23471764	Body;Body	556	*LINC00621;BASP1P1*	*BASP1P1*
cg07185068	4.28E-08	7.69E-06	6.022174	3	53801915	Body;Body;Body	78531	*CACNA1D*	*CHDH*
cg05885676	4.31E-08	7.73E-06	6.020965	14	93104601	Body;Body	62023	*RIN3*	*RIN3*
cg08573869	4.31E-08	7.74E-06	6.020507	8	1.45E+08	Body;Body;Body;TES5000;TES5000;TES5000;TES5000;TES5000;TES5000;TES5000;TES5000;TES5000;TES5000;TES5000	44	*SCRIB;MIR937;PUF60*	*MIR937*
cg09883947	4.32E-08	7.74E-06	6.020357	3	33477985	Body;Body;Body	3885	*UBP1*	*UBP1*
cg13741304	4.32E-08	7.74E-06	6.020224	9	1.41E+08	Body;Body	1168	*ARRDC1*	*ARRDC1*
cg03835755	4.32E-08	7.74E-06	6.020042	2	1.92E+08	Body;Body;Body;TSS5000;TSS5000	-4284	*NAB1*	*NAB1*
cg18912541	4.32E-08	7.74E-06	6.020019	14	69596496	Body;Body;Body;Body	22820	*DCAF5*	*DCAF5*
cg25837277	4.33E-08	7.74E-06	6.019954	19	46999366	Body;TSS1500	-242	*PPP5D1;PNMA8B*	*PNMA8B*
cg04142643	4.33E-08	7.75E-06	6.019611	17	75478263	Body;Body;Body;Body;Body;Body;Body;1stExon;1stExon;1stExon;Body	6939	*SEPTIN9*	*SEPTIN9*
cg09088050	4.34E-08	7.76E-06	6.019311	11	8090206	Body;Body;Body	8622	*TUB;TUB-AS1*	*TUB-AS1*
cg14228177	4.35E-08	7.78E-06	6.018475	7	1606526	TES5000;TES5000;TSS5000;TSS5000;TSS5000	3142	*PSMG3;PSMG3-AS1*	*PSMG3*
cg27329908	4.36E-08	7.79E-06	6.018289	3	38671866	Body;Body;Body;Body;Body;Body;Body	19297	*SCN5A*	*SCN5A*
cg03078819	4.37E-08	7.80E-06	6.017764	7	1.4E+08	3'UTR;3'UTR;Body;3'UTR	-38969	*DENND2A*	*MKRN1*
cg08272591	4.37E-08	7.80E-06	6.017719	8	28576644	Body;Body;Body	17627	*EXTL3*	*EXTL3*
cg02083492	4.38E-08	7.82E-06	6.017102	7	98031846	TSS1500	-1444	*BAIAP2L1*	*BAIAP2L1*
cg06340836	4.39E-08	7.83E-06	6.016651	16	88765676	Body;Body;Body	4313	*RNF166*	*RNF166*
cg07172339	4.39E-08	7.83E-06	6.016507	1	1.79E+08	Body;Body;Body;Body;Body;Body;Body;Body	27546	*ABL2*	*ABL2*
cg00264994	4.40E-08	7.84E-06	6.016165	17	47991806		-54747		*DLX4*
cg20569070	4.40E-08	7.84E-06	6.015999	2	2.33E+08	3'UTR;3'UTR	19256	*PDE6D*	*MIR1244-1*
cg14517133	4.40E-08	7.84E-06	6.01597	6	42664420	TES5000;TES5000;3'UTR	25915	*UBR2;PRPH2*	*PRPH2*
cg02055353	4.40E-08	7.84E-06	6.015938	11	1.26E+08	TES5000;TES5000;TES5000;TES5000;TES5000;TES5000;TES5000;TES5000;TES5000;TES5000	11042	*ST3GAL4*	*ST3GAL4*
cg18447587	4.40E-08	7.84E-06	6.015785	2	64627858		-53437		*LGALSL*
cg24265719	4.41E-08	7.84E-06	6.015693	8	1651101	3'UTR	60399	*DLGAP2*	*LOC101927752*
cg15864104	4.41E-08	7.84E-06	6.015482	18	55254508	TSS1500;TSS1500;TSS1500;TSS1500	-574	*FECH*	*FECH*
cg20396782	4.41E-08	7.84E-06	6.015426	17	27330221	Body;Body;Body	2830	*SEZ6*	*SEZ6*
cg05056653	4.41E-08	7.84E-06	6.015377	17	81023856		-13628		*METRNL*
cg20522761	4.42E-08	7.86E-06	6.014744	8	1.44E+08	Body	13240	*ADGRB1*	*ADGRB1*
cg09168501	4.43E-08	7.86E-06	6.014575	1	1.7E+08	TSS1500	-944	*METTL11B*	*METTL11B*
cg01041717	4.43E-08	7.87E-06	6.0143	13	1.07E+08		-19849		*LINC00460*
cg10497481	4.43E-08	7.87E-06	6.014262	7	36008153		-126766		*LOC101928618*
cg00348891	4.43E-08	7.87E-06	6.014217	13	46251914	TES5000	23405	*LINC01055*	*LINC01055*
cg05689208	4.44E-08	7.87E-06	6.014062	8	49496391	Body	32265	*LOC101929268*	*LOC101929268*
cg10265952	4.44E-08	7.87E-06	6.013923	6	5043901	TES5000;TES5000;Body;TES5000;TES5000	13696	*LYRM4-AS1*	*LYRM4-AS1*
cg13634653	4.44E-08	7.87E-06	6.013828	15	78552612	TSS5000;TSS5000	-3874	*DNAJA4*	*DNAJA4*
cg02683408	4.44E-08	7.87E-06	6.01367	17	79487159		-7334		*ACTG1*
cg03830401	4.45E-08	7.87E-06	6.013531	10	70812585		-34773		*SRGN*
cg27542224	4.46E-08	7.89E-06	6.012837	6	1.7E+08	Body;Body;Body;Body	211768	*WDR27*	*WDR27*
cg13803632	4.46E-08	7.90E-06	6.012647	1	81968855	Body;Body;Body	54532	*ADGRL2*	*LOC101927434*
cg25483191	4.47E-08	7.90E-06	6.012446	8	67050040	Body;Body;Body;Body	10677	*TRIM55*	*TRIM55*
cg21924070	4.47E-08	7.90E-06	6.012345	3	1.41E+08	Body	18265	*SPSB4*	*SPSB4*
cg24960118	4.47E-08	7.90E-06	6.012315	6	51815507	Body;Body	136904	*PKHD1*	*PKHD1*

Probe	p_adj	q_val	t	Chr	Pos.hg19	locations	dist2TSS	gene.Illumina	gene.nearest
cg09607229	4.47E-08	7.90E-06	6.012228	3	44619924	Body;Body;Body;Body	-6531	*ZKSCAN7;ZKSCAN7-AS1*	*ZNF660*
cg08695416	4.47E-08	7.90E-06	6.012216	2	43309970		19613		*LINC02580*
cg07619672	4.48E-08	7.90E-06	6.012015	15	64338028	Body;Body	656	*DAPK2*	*DAPK2*
cg19562108	4.49E-08	7.93E-06	6.01105	17	28420331	Body	-23493	*EFCAB5*	*NSRP1*
cg13052876	4.50E-08	7.94E-06	6.010728	20	35972924	TSS200;TSS5000	-163	*SRC*	*SRC*
cg05257528	4.50E-08	7.94E-06	6.010676	19	846179	Body	5181	*PRTN3*	*PRTN3*
cg17876831	4.50E-08	7.94E-06	6.010539	17	18918410	Body;Body;Body;Body;Body	-10333	*SLC5A10*	*FAM83G*
cg06956052	4.51E-08	7.94E-06	6.010399	1	1.6E+08	TSS200	-191	*ATP1A4*	*ATP1A4*
cg03900565	4.51E-08	7.94E-06	6.010021	1	2.03E+08	Body;Body	-21107	*PPFIA4*	*MYOPARR*
cg05642426	4.52E-08	7.94E-06	6.009987	8	22492984	Body;Body;Body;TES5000	6738	*BIN3;BIN3-IT1*	*BIN3-IT1*
cg01502208	4.52E-08	7.94E-06	6.00994	2	54855588	Body;Body	70058	*SPTBN1*	*SPTBN1*
cg25152348	4.52E-08	7.94E-06	-6.00987	22	50946712	TSS1500;TSS1500;5'UTR;5'UTR;5'UTR	60	*LMF2;NCAPH2*	*NCAPH2*
cg22790973	4.52E-08	7.94E-06	-6.00984	12	51566990	TSS200;TSS200;TSS200	-72	*TFCP2*	*TFCP2*
cg07365452	4.53E-08	7.96E-06	6.009325	3	42265749	3'UTR;Body;3'UTR;3'UTR;3'UTR;3'UTR;3'UTR	39690	*TRAK1*	*CCK*
cg16772207	4.54E-08	7.98E-06	6.008697	20	62795807	5'UTR	3	*MYT1*	*MYT1*
cg01728830	4.55E-08	7.98E-06	6.008478	11	1.18E+08	5'UTR;Body;Body;Body;Body;Body;Body;Body	13	*SMIM35;TMPRSS4*	*SMIM35*
cg00122854	4.55E-08	7.98E-06	6.008289	2	2.19E+08	TSS1500	-1165	*RUFY4*	*RUFY4*
cg05347158	4.55E-08	7.98E-06	6.008139	1	32148832	Body	20786	*COL16A1*	*COL16A1*
cg12458932	4.55E-08	7.98E-06	6.008087	2	2.25E+08		-33833		*SERPINE2*
cg00294940	4.55E-08	7.98E-06	6.008027	1	48687524	TSS1500;TSS1500	-863	*SLC5A9*	*SLC5A9*
cg13699245	4.55E-08	7.98E-06	6.008019	9	1.37E+08		-48266		*COL5A1*
cg10809622	4.56E-08	7.98E-06	6.007873	6	1.68E+08		-13895		*CCR6*
cg00384632	4.56E-08	7.98E-06	6.007799	7	2194791	Body;Body;Body;Body;Body	-66104	*MAD1L1*	*SNORA114*
cg15261605	4.56E-08	7.99E-06	6.007635	10	73234421	Body;Body;Body;Body;Body	37256	*CDH23*	*CDH23-AS1*
cg01806238	4.57E-08	8.00E-06	6.007051	17	56597682	TSS5000;Body;3'UTR;3'UTR;3'UTR;3'UTR;3'UTR;3'UTR;3'UTR;3'UTR;Body;Body;Body;3'UTR	485	*MTMR4;SEPTIN4-AS1;SEPTIN4*	*SEPTIN4-AS1*
cg22672830	4.58E-08	8.00E-06	6.006905	6	31829093	Body;TES5000;TES5000;TES5000	1505	*NEU1;SLC44A4*	*NEU1*
cg17276118	4.58E-08	8.00E-06	6.006728	11	92083801	Body;TSS1500	-1460	*FAT3*	*FAT3*
cg05798126	4.58E-08	8.00E-06	6.006656	6	3089496	Body;Body;Body;Body;Body;Body;Body	20769	*RIPK1*	*RIPK1*
cg02302670	4.58E-08	8.00E-06	6.006621	8	21904079	Body;Body;TSS5000;TSS5000	-2362	*FGF17;DMTN*	*DMTN*
cg09424896	4.59E-08	8.02E-06	6.006226	19	16284351	TSS200;TSS200	-15	*CIB3*	*CIB3*
cg17794788	4.60E-08	8.03E-06	6.00557	2	2.39E+08	TSS1500;TSS1500	-856	*RAMP1*	*RAMP1*
cg16156434	4.61E-08	8.04E-06	6.005344	10	1.34E+08	Body	-28394	*INPP5A*	*INPP5A*
cg13768360	4.62E-08	8.06E-06	6.004647	20	61312704		-14731		*SLCO4A1-AS1*
cg00978848	4.62E-08	8.06E-06	6.004527	2	2.36E+08		88994		*LINC01173*
cg26086527	4.63E-08	8.07E-06	6.004103	14	34272019	3'UTR;3'UTR;3'UTR;3'UTR	93877	*NPAS3*	*SNORA89*
cg10060191	4.63E-08	8.07E-06	6.0041	16	66282209		-118384		*CDH5*
cg16372825	4.64E-08	8.07E-06	6.003937	16	87823925		-11171		*LOC102724467*
cg00959298	4.64E-08	8.07E-06	6.003867	17	71825500	TSS1500	-824	*LINC00469*	*LINC00469*
cg21730858	4.66E-08	8.10E-06	6.002842	6	1.39E+08	Body;Body	14311	*NHSL1*	*NHSL1*
cg23925540	4.66E-08	8.11E-06	6.002637	12	50962271	Body	63504	*DIP2B*	*DIP2B*
cg10100556	4.67E-08	8.11E-06	6.002441	16	89657887	Body;Body	15722	*CPNE7*	*CPNE7*
cg03213396	4.67E-08	8.12E-06	6.00213	19	53760449	TSS5000;TSS5000;TSS1500	-1095	*ZNF677;VN1R2*	*VN1R2*
cg23545258	4.68E-08	8.13E-06	6.001807	14	50394636		34837		*ARF6*
cg14323608	4.68E-08	8.13E-06	6.001748	19	13873961	3'UTR;3'UTR;3'UTR;3'UTR;3'UTR;3'UTR;3'UTR;3'UTR;TSS1500;TSS1500;TSS1500;TSS1500	-1374	*CCDC130;MRI1*	*MRI1*
cg27573606	4.69E-08	8.14E-06	6.001083	1	838379		16693		*LINC02593*
cg13912358	4.69E-08	8.14E-06	6.001076	3	14505367	Body;Body;Body	61262	*SLC6A6*	*SLC6A6*
cg06325565	4.70E-08	8.15E-06	6.000859	11	63889842	Body;TES5000	18481	*MACROD1;FLRT1*	*FLRT1*
cg01987202	4.70E-08	8.16E-06	6.000477	15	40633194	TSS200	-26	*CCDC9B*	*CCDC9B*
cg14355321	4.71E-08	8.16E-06	6.000385	8	1.24E+08	Body;Body	83273	*ZHX2*	*DERL1*
cg01019188	4.71E-08	8.16E-06	6.000319	7	14809526	Body;Body;Body;Body;Body;Body;Body;Body;Body;Body;Body;Body;Body;Body;Body;Body;Body;Body;Body	71549	*DGKB*	*DGKB*
cg19556337	4.71E-08	8.16E-06	6.000086	11	511660	TSS5000;TSS5000;TSS5000;TSS5000;TSS5000;TSS5000;TSS5000;TSS5000	-4418	*RNH1*	*RNH1*
cg06995828	4.71E-08	8.16E-06	6.000005	2	84160861		-356944		*FUNDC2P2*
cg19968179	4.72E-08	8.17E-06	5.99977	7	33847134		-97392		*BMPER*
cg19039367	4.72E-08	8.17E-06	5.999654	6	30012436	Body;Body	14073	*ZNRD1ASP*	*ZNRD1ASP*
cg18665513	4.72E-08	8.17E-06	5.99961	4	8087448	Body;Body;Body;Body;Body;Body;Body	-13733	*ABLIM2*	*ABLIM2*
cg26907405	4.73E-08	8.17E-06	5.999448	15	93609551	Body;Body;Body;Body;Body;Body	6834	*RGMA*	*RGMA*
cg16555595	4.74E-08	8.18E-06	5.998975	13	1.14E+08	Body;Body;Body;Body;Body;Body;Body;Body	-37206	*TMCO3*	*TFDP1*
cg27146356	4.74E-08	8.18E-06	5.99897	16	56719841	TES5000	3460	*MT1X*	*MT1X*
cg04995849	4.74E-08	8.18E-06	5.998851	1	1.15E+08		-29528		*SIKE1*
cg09962925	4.74E-08	8.18E-06	5.99873	16	86228639	TSS1500	-1147	*LINC01082*	*LINC01082*

Probe	p_adj	q_val	t	Chr	Pos.hg19	locations	dist2TSS	gene.Illumina	gene.nearest
cg00493363	4.74E-08	8.18E-06	5.998723	7	1.07E+08		-65375		*PRKAR2B*
cg10987960	4.74E-08	8.18E-06	5.998684	3	1.57E+08	TSS5000;TSS1500;TSS1500;TSS1500;TSS1500	-1007	*VEPH1*	*VEPH1*
cg13625620	4.75E-08	8.18E-06	5.998501	8	10273691	Body;Body;Body;Body	-58383	*MSRA*	*LINCR-0001*
cg21992507	4.75E-08	8.18E-06	5.998476	11	6789940	Body	248	*OR2AG2*	*OR2AG2*
cg03646542	4.76E-08	8.19E-06	5.997923	5	1.72E+08	Body;Body;Body	7890	*NEURL1B*	*NEURL1B*
cg11832804	4.76E-08	8.19E-06	5.997776	5	1279449	Body;Body;Body;Body	15734	*TERT*	*TERT*
cg16094073	4.76E-08	8.19E-06	5.997724	1	2.21E+08		-55062		*RAB3GAP2*
cg05041871	4.76E-08	8.19E-06	5.997625	2	1732234	Body	16053	*PXDN*	*PXDN*
cg08435240	4.76E-08	8.19E-06	5.997579	21	39643733	Body;Body;Body;Body;Body;Body;Body;TSS1500	-608	*KCNJ15*	*KCNJ15*
cg06164804	4.77E-08	8.19E-06	5.997397	2	2.4E+08	Body	-36767	*HDAC4*	*MIR4441*
cg15470261	4.77E-08	8.19E-06	5.997387	19	1698699		-46085		*TCF3*
cg05014245	4.77E-08	8.19E-06	5.997386	10	82172262	Body;Body;Body;Body;TSS1500;TSS1500	-1312	*PRXL2A*	*PRXL2A*
cg12401058	4.77E-08	8.19E-06	5.99738	13	23423130		49190		*BASP1P1*
cg18391058	4.77E-08	8.19E-06	5.997354	1	6207067	Body	33314	*CHD5*	*CHD5*
cg11553153	4.77E-08	8.19E-06	5.997327	15	34348521	Body;Body	-17116	*CHRM5*	*AVEN*
cg15821544	4.78E-08	8.20E-06	5.996774	1	43473840		49121		*SLC2A1-AS1*
cg12398117	4.78E-08	8.21E-06	5.996595	5	76474556	TSS5000;TSS5000	-1572	*PDE8B*	*PDE8B*
cg27526892	4.79E-08	8.22E-06	5.996191	1	3372482	Body	1254	*ARHGEF16*	*ARHGEF16*
cg26842720	4.80E-08	8.23E-06	5.995797	8	62212734	1stExon	12206	*CLVS1*	*CLVS1*
cg07698783	4.80E-08	8.23E-06	5.99575	11	47395915	Body;Body	4183	*SPI1*	*SPI1*
cg07373589	4.81E-08	8.24E-06	5.995244	1	6189304	Body	51077	*CHD5*	*CHD5*
cg08880875	4.82E-08	8.25E-06	5.994813	2	74873273	Body;Body;Body;Body	1891	*M1AP*	*M1AP*
cg09040752	4.82E-08	8.25E-06	5.9948	17	41003155	TES5000;TES5000;TSS200;TSS200;TSS200;TSS5000	-45	*AOC2;AOC3*	*AOC3*
cg02155184	4.82E-08	8.25E-06	5.994702	1	1.1E+08	3'UTR;TSS5000	-3995	*GPR61;GNAI3*	*GNAI3*
cg02508229	4.83E-08	8.25E-06	5.994551	1	27336989	Body	2352	*TENT5B*	*TENT5B*
cg00543840	4.83E-08	8.25E-06	5.994421	1	22213376	Body;Body	50427	*HSPG2*	*HSPG2*
cg24445724	4.83E-08	8.26E-06	5.99419	1	1.55E+08	TES5000;Body;Body;Body;Body	2813	*MIR92B;THBS3*	*MIR92B*
cg23323468	4.84E-08	8.26E-06	5.994049	2	2.29E+08		-15037		*C2orf83*
cg16806051	4.84E-08	8.26E-06	5.994024	6	1.69E+08		-57298		*DACT2*
cg03096286	4.84E-08	8.26E-06	5.993873	2	2.19E+08	Body;TSS5000;TSS5000	-1768	*CATIP-AS2;CATIP*	*CATIP*
cg01076675	4.85E-08	8.27E-06	5.993404	19	7977612	3'UTR;3'UTR;3'UTR;TSS5000	-3419	*MAP2K7;TGFBR3L*	*TGFBR3L*
cg09068492	4.86E-08	8.29E-06	5.992971	11	14993658	Body;Body;Body;Body	174	*CALCA*	*CALCA*
cg18327319	4.87E-08	8.30E-06	5.992386	1	11795409	TSS1500;TSS1500;TSS1500;TSS1500;TSS1500	-732	*AGTRAP*	*AGTRAP*
cg00278329	4.88E-08	8.32E-06	5.991858	16	31440531	TES5000;TES5000;TSS1500	-850	*ITGAD;COX6A2*	*COX6A2*
cg12790106	4.89E-08	8.33E-06	5.991464	15	63105204	Body	-10951	*TLN2*	*MIR190A*
cg12936992	4.90E-08	8.34E-06	5.991166	1	92002689		36025		*CDC7*
cg10447827	4.90E-08	8.34E-06	5.990967	8	1.42E+08	Body;Body;Body;Body	39789	*DENND3*	*DENND3*
cg25389250	4.90E-08	8.34E-06	5.990851	8	1814835	Body;Body;Body	42744	*ARHGEF10*	*ARHGEF10*
cg02335700	4.91E-08	8.34E-06	5.990814	12	1.1E+08		-11606		*TRPV4*
cg12601353	4.91E-08	8.35E-06	5.990517	12	15687708	Body;Body	-11836	*PTPRO*	*PTPRO*
cg21008530	4.92E-08	8.35E-06	5.990331	7	88229994		-159019		*ZNF804B*
cg06330587	4.92E-08	8.35E-06	5.990279	7	1.49E+08	Body	15083	*SSPO*	*SSPO*
cg05198969	4.92E-08	8.35E-06	5.990167	5	1.42E+08	TSS1500;TSS1500;TSS1500;TSS1500;TSS1500;TSS1500;TSS200;Body;Body;Body;Body;Body;Body;Body;Body;Body;Body;Body;Body;Body;Body;Body;Body	-68	*FGF1*	*FGF1*
cg09372449	4.92E-08	8.35E-06	5.990082	16	2760410	TES5000;TES5000;TES5000	9815	*KCTD5;PRSS27*	*PRSS27*
cg08922109	4.92E-08	8.35E-06	5.989996	7	1.41E+08	TSS200	-82	*TAS2R3*	*TAS2R3*
cg22659681	4.92E-08	8.35E-06	5.989996	17	7318433	Body	-5209	*NLGN2*	*SPEM1*
cg00903932	4.92E-08	8.35E-06	5.989967	1	1.01E+08		73430		*VCAM1*
cg06841192	4.93E-08	8.35E-06	5.989803	16	58518580	Body;Body;Body;Body	-15466	*NDRG4*	*NDRG4*
cg10797737	4.93E-08	8.35E-06	5.989669	14	1.06E+08	3'UTR;3'UTR;3'UTR;3'UTR;3'UTR	7794	*TEDC1*	*TEDC1*
cg14074366	4.94E-08	8.36E-06	5.989204	9	91014994	Body	11636	*SPIN1*	*SPIN1*
cg02699885	4.94E-08	8.36E-06	5.989184	16	87492801	Body	33829	*ZCCHC14*	*ZCCHC14*
cg22994520	4.94E-08	8.36E-06	5.989132	2	54630332		-53131		*SPTBN1*
cg15730967	4.94E-08	8.36E-06	5.989101	3	12856795	Body;Body	18617	*CAND2*	*CAND2*
cg08616111	4.94E-08	8.36E-06	5.989029	19	13112808	Body;Body;Body	6520	*NFIX*	*NFIX*
cg16716752	4.96E-08	8.38E-06	5.988357	6	33157811	Body;Body;Body;Body;TES5000;TES5000;TES5000	2433	*COL11A2;RXRB*	*COL11A2*
cg07092135	4.96E-08	8.38E-06	5.988289	4	1.25E+08	Body;Body	58445	*LINC01091*	*LINC01091*
cg25137715	4.97E-08	8.39E-06	5.987795	2	8301539	Body	167010	*LINC00299*	*LINC00299*
cg02250353	4.97E-08	8.40E-06	5.987593	15	43533238	Body;Body	-19915	*TGM5*	*EPB42*
cg13337655	4.98E-08	8.40E-06	5.98743	17	48335371		-13395		*TMEM92*

Probe	p_adj	q_val	t	Chr	Pos.hg19	locations	dist2TSS	gene.Illumina	gene.nearest
cg17046756	4.98E-08	8.40E-06	5.987391	11	1855851	Body;Body;Body;Body;TSS5000;TSS5000	195	*SYT8;TNNI2*	*SYT8*
cg26081974	4.99E-08	8.41E-06	5.987058	13	1.14E+08	Body	2811	*LOC101928841*	*LOC101928841*
cg03722908	4.99E-08	8.41E-06	5.986728	6	1.65E+08		477386		*MEAT6*
cg25660265	4.99E-08	8.41E-06	5.986701	4	4785832	Body	22346	*LOC101928279*	*LOC101928279*
cg19993573	5.00E-08	8.41E-06	5.986614	8	1495289	Body	74541	*DLGAP2*	*DLGAP2-AS1*
cg06053862	5.00E-08	8.42E-06	5.986243	11	68028857	3'UTR;3'UTR	10584	*C11orf24*	*C11orf24*
cg11215013	5.01E-08	8.44E-06	5.985862	16	89777115	Body;TSS1500	-1148	*VPS9D1;VPS9D1-AS1*	*VPS9D1-AS1*
cg03669198	5.02E-08	8.44E-06	5.985523	6	33374377	Body;TSS5000;TSS5000;TSS5000	-4413	*KIFC1;PHF1*	*PHF1*
cg07359183	5.02E-08	8.44E-06	5.985443	1	2.08E+08	Body	59711	*PLXNA2*	*PLXNA2*
cg06360527	5.03E-08	8.46E-06	5.984939	17	7491548	TSS5000;TSS5000;TSS5000;TSS5000;TES5000;TES5000;TES5000;3'UTR;TES5000	1864	*LOC100996842;MPDU1;SOX15;FXR2*	*SOX15*
cg08970446	5.04E-08	8.46E-06	5.98468	1	53608262	5'UTR;5'UTR;5'UTR;Body;5'UTR	48	*SLC1A7*	*SLC1A7*
cg26937943	5.04E-08	8.46E-06	5.984635	18	8800248	Body	82870	*MTCL1*	*MTCL1*
cg19410265	5.04E-08	8.46E-06	5.984591	22	18897735	Body;TES5000;TES5000	3903	*DGCR6;PRODH*	*DGCR6*
cg14492061	5.04E-08	8.46E-06	5.984539	3	63637129	TSS1500;TSS1500	-1221	*SNTN*	*SNTN*
cg16582836	5.04E-08	8.46E-06	5.984491	1	6952297	TES5000;TES5000;TES5000;Body;Body;Body;Body;Body;TES5000	106784	*CAMTA1*	*CAMTA1*
cg11525548	5.04E-08	8.46E-06	5.98444	1	7611041	Body;Body;Body;Body;Body	-118748	*CAMTA1*	*CAMTA1*
cg17209280	5.06E-08	8.48E-06	5.983646	3	16834977		-91470		*PLCL2*
cg14973055	5.06E-08	8.48E-06	5.983595	17	72306038	Body;Body;Body;Body	-16326	*DNAI2*	*KIF19*
cg08644340	5.06E-08	8.48E-06	5.983546	5	72585503		-48229		*LINC02230*
cg11514462	5.06E-08	8.48E-06	5.983501	11	1.21E+08	Body	27919	*TECTA*	*TECTA*
cg11413763	5.07E-08	8.49E-06	5.983294	1	9365494	Body	12542	*SPSB1*	*SPSB1*
cg23141614	5.08E-08	8.50E-06	5.982817	6	1.67E+08	Body;Body;Body	-95693	*RPS6KA2*	*RPS6KA2*
cg22377480	5.08E-08	8.50E-06	5.9827	3	35934217		148250		*MIR128-2*
cg09946870	5.08E-08	8.50E-06	5.982528	8	1.42E+08	Body;Body;Body;Body	49073	*DENND3*	*SLC45A4*
cg07304551	5.09E-08	8.50E-06	5.982427	16	85618571		-26443		*GSE1*
cg02452209	5.10E-08	8.53E-06	5.981589	7	1.58E+08	Body;Body;Body;Body;Body	264604	*PTPRN2*	*MIR595*
cg17016369	5.11E-08	8.53E-06	5.98155	2	1.98E+08		53539		*SF3B1*
cg18857216	5.11E-08	8.53E-06	-5.98153	1	11021217	Body;Body	5527	*C1orf127*	*C1orf127*
cg16871520	5.13E-08	8.56E-06	5.98064	3	52442033	Body;TSS5000;TSS5000;TSS5000;TSS5000	1991	*BAP1;PHF7*	*BAP1*
cg00798342	5.14E-08	8.57E-06	5.980202	12	1.11E+08	TSS5000;TSS5000;TSS5000	-2217	*HVCN1*	*HVCN1*
cg02523263	5.14E-08	8.57E-06	5.980111	12	64687265	Body;Body	-71189	*C12orf56*	*C12orf66*
cg27478879	5.14E-08	8.57E-06	5.980014	9	1.39E+08	TES5000;Body	-7551	*GPSM1*	*GPSM1*
cg11567627	5.15E-08	8.58E-06	5.979646	5	1.19E+08		-46899		*FAM170A*
cg16977304	5.15E-08	8.59E-06	5.979362	13	20134785	Body	929	*TPTE2*	*TPTE2*
cg14070323	5.16E-08	8.59E-06	5.979317	14	1.01E+08	TSS1500;TSS1500;TSS1500;TSS1500;TSS1500;TSS1500;TSS1500;TSS1500;TSS1500;TSS1500;TSS1500;TSS1500;TSS1500;TSS1500;TSS1500	-588	*MEG3*	*MEG3*
cg00391247	5.16E-08	8.59E-06	5.979033	12	31378024		-23855		*OVOS2*
cg27182133	5.16E-08	8.59E-06	5.978974	1	10658147	Body	123141	*PEX14*	*PEX14*
cg16618389	5.16E-08	8.59E-06	5.978893	12	46865385	Body;Body;Body;Body;Body	87496	*LOC100288798*	*LOC100288798*
cg25770411	5.18E-08	8.61E-06	5.978243	7	73946901	Body;Body;Body	-63393	*GTF2IRD1*	*MIR10525*
cg02387639	5.18E-08	8.61E-06	5.978164	3	1.79E+08	Body	13466	*USP13*	*USP13*
cg16811875	5.18E-08	8.62E-06	5.978012	7	1.32E+08	1stExon;1stExon;1stExon	68406	*PLXNA4*	*PLXNA4*
cg00765127	5.20E-08	8.64E-06	5.977205	8	22959168	Body;TSS1500	-1158	*LOC254896;TNFRSF10C*	*TNFRSF10C*
cg11920592	5.20E-08	8.64E-06	5.977155	6	1.12E+08	Body;Body	-45426	*FYN*	*FYN*
cg19275091	5.21E-08	8.65E-06	5.976811	1	1.2E+08		-10053		*LINC01780*
cg24101387	5.21E-08	8.65E-06	5.976806	6	2510586		-124147		*LINC02521*
cg23499929	5.21E-08	8.65E-06	5.976653	17	79097152	Body;Body;TES5000;TSS5000;TES5000	2021	*AATK;MIR657;MIR3065;MIR338*	*MIR657*
cg11204562	5.21E-08	8.65E-06	5.976644	10	1.16E+08	TSS1500;TSS1500;TSS1500	-287	*PLEKHS1*	*PLEKHS1*
cg10600536	5.22E-08	8.66E-06	5.976262	11	47608204	TES5000;5'UTR;5'UTR;5'UTR;5'UTR;TES5000	2	*NDUFS3;FAM180B;C1QTNF4*	*FAM180B*
cg11929693	5.23E-08	8.66E-06	5.976157	5	1.35E+08	Body;Body	8278	*C5orf66;C5orf66-AS2*	*C5orf66-AS2*
cg16598668	5.23E-08	8.66E-06	5.976147	7	82159556		-86284		*CACNA2D1*
cg06250841	5.23E-08	8.66E-06	5.975893	8	58138048	Body	7214	*LINC01606*	*LINC01606*
cg08290287	5.24E-08	8.68E-06	5.975367	19	42744095	Body	2659	*GSK3A*	*GSK3A*
cg06406822	5.24E-08	8.68E-06	5.975321	5	1.76E+08	3'UTR	9347	*RNF44*	*RNF44*
cg11154727	5.25E-08	8.68E-06	5.975079	5	85189638		-388623		*NBPF22P*
cg15445319	5.25E-08	8.68E-06	5.975006	17	72997680	Body	-11084	*CDR2L*	*MRPL58*
cg07518851	5.26E-08	8.69E-06	5.974725	10	1.35E+08	3'UTR	-5274	*KNDC1*	*UTF1*
cg25324021	5.26E-08	8.69E-06	5.974682	13	27261076	3'UTR;3'UTR	73847	*WASF3*	*GPR12*
cg25682188	5.26E-08	8.69E-06	5.974533	2	75278194	Body;3'UTR	-39744	*TACR1*	*MIR5000*
cg23199797	5.26E-08	8.69E-06	5.974443	3	1.22E+08	Body;Body	-63252	*CASR*	*CSTA*

Probe	p_adj	q_val	t	Chr	Pos.hg19	locations	dist2TSS	gene.Illumina	gene.nearest
cg19046730	5.27E-08	8.69E-06	5.974423	10	81943774	Body;Body;Body;Body;Body;Body	20473	*ANXA11*	*ANXA11*
cg08822523	5.27E-08	8.69E-06	5.974401	4	91156290	Body;5'UTR	109	*CCSER1*	*CCSER1*
cg24360909	5.27E-08	8.69E-06	5.974332	15	49189538	Body	19208	*SHC4*	*EID1*
cg15242048	5.28E-08	8.70E-06	5.9738	2	2.34E+08	Body;Body	4239	*NGEF*	*NGEF*
cg09834343	5.28E-08	8.70E-06	5.973795	15	32942910	Body;Body;Body	9097	*ARHGAP11A-SCG5;SCG5*	*SCG5*
cg07379741	5.28E-08	8.70E-06	5.973684	17	7311030	TSS5000;TSS5000;TSS200	-133	*TMEM256-PLSCR3;TMEM256;NLGN2*	*NLGN2*
cg09995105	5.29E-08	8.70E-06	5.973439	1	1.86E+08	Body	3589	*HMCN1*	*HMCN1*
cg08106205	5.29E-08	8.70E-06	5.973425	1	2062664	Body;Body;Body;Body;Body;Body;Body;Body;Body	26510	*PRKCZ*	*PRKCZ*
cg05410536	5.29E-08	8.70E-06	5.973373	14	69754550	Body;Body	27676	*GALNT16*	*GALNT16*
cg12636499	5.29E-08	8.71E-06	5.973166	10	1.05E+08	Body;Body	29374	*SH3PXD2A*	*SH3PXD2A*
cg02280348	5.30E-08	8.71E-06	5.973105	14	22011087		-5832		*SALL2*
cg25155149	5.30E-08	8.71E-06	5.972861	10	1.02E+08	TSS5000;TSS5000;TSS5000;TSS5000;TSS5000;TSS5000;3'UTR;3'UTR	-1668	*BLOC1S2;PKD2L1*	*BLOC1S2*
cg14152608	5.32E-08	8.73E-06	5.972018	10	3159711	Body;Body;Body;Body;Body;Body;Body;Body;Body;Body	12911	*PFKP*	*PFKP*
cg25143743	5.32E-08	8.73E-06	5.972016	5	10725546	Body;Body	35800	*DAP*	*DAP*
cg12178225	5.32E-08	8.73E-06	5.971999	6	30165961	Body;Body	15222	*TRIM26*	*TRIM26*
cg03180404	5.32E-08	8.73E-06	5.971968	11	1.27E+08		-29413		*LOC101929473*
cg16429208	5.33E-08	8.73E-06	5.971788	1	2.48E+08	Body;Body;Body;Body;Body;Body;Body;TES5000	1104	*GCSAML;GCSAML-AS1;OR2C3*	*GCSAML-AS1*
cg00936728	5.33E-08	8.73E-06	5.971736	1	1.6E+08	Body;5'UTR	45	*FCRL6*	*FCRL6*
cg12136716	5.33E-08	8.73E-06	5.971712	3	57722951		-20031		*SLMAP*
cg00972950	5.34E-08	8.75E-06	5.971212	4	1684791	Body;Body;Body	927	*FAM53A*	*FAM53A*
cg07416364	5.34E-08	8.75E-06	5.971146	2	2.01E+08		-17932		*FTCDNL1*
cg24194730	5.35E-08	8.76E-06	5.970859	11	47436603	Body;Body;Body;Body;TES5000	6463	*SLC39A13;PSMC3*	*SLC39A13*
cg00094943	5.35E-08	8.76E-06	5.97055	7	1.5E+08	Body	-9780	*ZNF775*	*LOC728743*
cg14824983	5.36E-08	8.77E-06	5.970368	3	50304424	Body;TSS1500;TSS1500;TSS5000;TSS5000	379	*SEMA3B-AS1;SEMA3B*	*SEMA3B-AS1*
cg22044669	5.36E-08	8.77E-06	5.970104	22	32369286	Body	2477	*LINC02558*	*LINC02558*
cg04246526	5.37E-08	8.77E-06	5.970043	3	43033200	Body	12398	*GASK1A*	*GASK1A*
cg24995503	5.37E-08	8.77E-06	5.970008	14	1.03E+08		-12685		*RCOR1*
cg02924954	5.37E-08	8.77E-06	5.96999	10	1262895	Body	57188	*ADARB2*	*LINC00200*
cg24742092	5.38E-08	8.78E-06	5.969487	1	20251280	TSS1500	-1170	*PLA2G2E*	*PLA2G2E*
cg11804604	5.38E-08	8.78E-06	5.969428	1	1.57E+08	TES5000;3'UTR;3'UTR;TES5000	1180	*LRRC71;ARHGEF11;MIR765*	*MIR765*
cg25184717	5.38E-08	8.78E-06	5.969416	17	79354866	Body;Body	4059	*LOC100130370*	*LOC100130370*
cg26635367	5.38E-08	8.78E-06	5.969288	4	1.66E+08	Body;Body;Body;TSS200;TSS200	-51	*KLHL2*	*KLHL2*
cg18410168	5.40E-08	8.80E-06	5.968698	19	35843282	1stExon	838	*FFAR1*	*FFAR1*
cg17243636	5.40E-08	8.80E-06	5.968569	10	1.34E+08		-40341		*BNIP3*
cg17313222	5.41E-08	8.82E-06	5.968086	8	96213626		14976		*LINC01298*
cg07594007	5.42E-08	8.83E-06	5.96775	10	1.12E+08	TSS200	-50	*LOC105378479*	*LOC105378479*
cg13904508	5.42E-08	8.83E-06	5.96763	1	1.12E+08	Body;Body;Body;Body;Body;Body;Body;Body;Body;Body;Body;Body;Body;Body;Body;Body;Body;Body;Body;Body;Body;TSS5000;TSS5000;TSS5000;TSS5000;TSS5000;TSS5000	-2686	*DRAM2;CEPT1*	*CEPT1*
cg22334548	5.42E-08	8.83E-06	5.967606	19	49971665	Body;Body	-5806	*ALDH16A1*	*FLT3LG*
cg15035324	5.42E-08	8.83E-06	5.967542	2	46269605	Body	-254940	*PRKCE*	*EPAS1*
cg24347822	5.43E-08	8.83E-06	5.967364	2	2.37E+08	Body;Body;Body	-294118	*AGAP1*	*TNRC17*
cg15546282	5.43E-08	8.83E-06	5.967272	1	1903425	Body	31800	*CFAP74*	*CFAP74*
cg08329561	5.44E-08	8.84E-06	5.966903	10	79997038		-11343		*LINC00856*
cg27646075	5.45E-08	8.86E-06	5.966367	11	1.18E+08	TSS1500	-862	*SCN2B*	*SCN2B*
cg23613162	5.46E-08	8.87E-06	5.965915	1	27891416	Body;Body	38589	*AHDC1*	*AHDC1*
cg24875415	5.46E-08	8.87E-06	5.965898	20	23669470	1stExon	192	*CST4*	*CST4*
cg20359147	5.47E-08	8.89E-06	5.965411	6	226092		-20608		*LOC285766*
cg15714692	5.48E-08	8.89E-06	5.965332	11	57519659	Body;TSS1500	-406	*TMX2-CTNND1;BTBD18*	*BTBD18*
cg20540969	5.48E-08	8.89E-06	5.965042	2	2.36E+08	Body;Body;Body;Body;Body;Body	26043	*SH3BP4*	*SH3BP4*
cg09412782	5.49E-08	8.90E-06	5.96485	15	45721589	TSS1500;TSS1500;TSS5000	-1137	*C15orf48;MIR147B*	*C15orf48*
cg07255158	5.49E-08	8.90E-06	5.964672	13	1.12E+08		73530		*LINC02337*
cg15161827	5.49E-08	8.90E-06	5.964638	1	16318994		-11736		*SRARP*
cg20342907	5.50E-08	8.90E-06	5.964508	11	1.18E+08	Body;Body;Body;Body	10551	*TMPRSS13*	*TMPRSS13*
cg21687300	5.50E-08	8.91E-06	5.964322	1	52284363	Body;Body;Body	17711	*NRDC*	*MIR761*
cg03283124	5.51E-08	8.91E-06	5.963993	13	67802272	1stExon;1stExon;1stExon;1stExon;Body	2196	*PCDH9*	*PCDH9*
cg07025157	5.51E-08	8.92E-06	5.963739	4	1.28E+08		-491354		*INTU*
cg03033996	5.52E-08	8.92E-06	5.963647	4	8071486	Body;Body;Body;Body;Body;Body;Body;Body	2229	*ABLIM2*	*ABLIM2*
cg03968976	5.53E-08	8.94E-06	5.963084	11	64273016		-50396		*SLC22A11*
cg17217194	5.54E-08	8.96E-06	5.96252	4	3870523		86632		*FAM86EP*

Probe	p_adj	q_val	t	Chr	Pos.hg19	locations	dist2TSS	gene.Illumina	gene.nearest
cg20467339	5.54E-08	8.96E-06	5.962463	19	54237666	TES5000;TES5000;TSS1500;TSS5000;TSS5000	-464	*MIR518E;MIR518A1;MIR518D;MIR516B1;MIR518A2*	*MIR518D*
cg14978637	5.56E-08	8.98E-06	5.961887	1	41950817	TSS1500;TSS1500;TSS1500	-463	*EDN2*	*EDN2*
cg04100337	5.56E-08	8.98E-06	5.961715	17	71336777	Body	-28637	*SDK2*	*CDC42EP4*
cg03696393	5.57E-08	8.98E-06	5.961454	13	1.07E+08	TSS1500;TSS1500	-1420	*LINC00460*	*LINC00460*
cg05067156	5.57E-08	8.98E-06	5.961439	16	88591451	Body;Body	6010	*ZFPM1;LOC100128882*	*LOC100128882*
cg09758386	5.57E-08	8.98E-06	5.961357	16	2528110	TES5000;Body;Body	2983	*NTN3;TBC1D24*	*TBC1D24*
cg09933215	5.57E-08	8.98E-06	5.961304	1	31360239	Body	21366	*SDC3*	*SDC3*
cg20463292	5.58E-08	8.99E-06	5.961091	1	1.61E+08	TES5000;TES5000;TES5000;TES5000;TES5000;TES5000	4083	*TSTD1;USF1*	*TSTD1*
cg24778849	5.58E-08	8.99E-06	5.961014	6	42424712	TSS5000;TSS5000	-4750	*TRERF1*	*TRERF1*
cg07795016	5.58E-08	8.99E-06	5.960977	7	1.51E+08	Body;TES5000;TES5000	15265	*NOS3;ATG9B*	*ATG9B*
cg20387706	5.58E-08	8.99E-06	5.960797	2	1.02E+08		-5740		*RFX8*
cg04845625	5.59E-08	9.00E-06	5.960438	1	2.02E+08	Body;Body;Body	23629	*LGR6*	*UBE2T*
cg11765913	5.60E-08	9.01E-06	5.959911	19	7926080	Body;Body	13195	*EVI5L*	*PRR36*
cg23600311	5.61E-08	9.01E-06	5.95988	4	7646859	Body	-108957	*SORCS2*	*AFAP1-AS1*
cg11732321	5.61E-08	9.01E-06	5.959874	8	99841169	TSS5000;TSS5000;Body	-3253	*STK3*	*STK3*
cg20166049	5.61E-08	9.01E-06	5.959719	19	45721076	Body	16393	*EXOC3L2*	*EXOC3L2*
cg00239742	5.61E-08	9.01E-06	5.959694	8	1339852	Body	-89025	*DLGAP2*	*LOC286083*
cg04604420	5.62E-08	9.03E-06	5.959248	7	1E+08	TSS1500	-1208	*POP7*	*POP7*
cg19109439	5.62E-08	9.03E-06	5.959112	6	32025657	Body;Body	-11752	*TNXB*	*TNXB*
cg05903223	5.63E-08	9.03E-06	5.95885	7	35836570	Body;Body;TSS5000;TSS5000;TSS5000;TSS5000	3665	*SEPTIN7-AS1;SEPTIN7*	*SEPTIN7-AS1*
cg07486252	5.63E-08	9.03E-06	5.958837	4	1.14E+08	Body;Body;Body;Body;Body;Body;Body;Body;Body;Body;Body;Body;Body;Body;Body;Body;Body;Body;Body;Body;Body;Body;Body;Body;Body;Body;Body;Body;Body;Body;Body;Body;Body;Body;Body;Body;Body;Body;Body;Body;Body;Body;Body;Body;TSS200;TSS200;TSS200;TSS200;TSS200	-80	*ANK2*	*ANK2*
cg14023020	5.64E-08	9.05E-06	-5.9583	8	1972405		-20755		*MYOM2*
cg07250771	5.66E-08	9.07E-06	5.957803	12	70289007	Body	70001	*MYRFL*	*MYRFL*
cg23386212	5.66E-08	9.07E-06	5.957613	14	62041363	TSS5000;TSS5000;Body	-3992	*LINC01303;FLJ22447*	*LINC01303*
cg04251828	5.66E-08	9.07E-06	5.957514	16	75252744	TSS200;TSS200	-141	*CTRB1*	*CTRB1*
cg16673412	5.67E-08	9.08E-06	5.957268	6	32062885	Body;Body	14230	*TNXB*	*TNXB*
cg25096368	5.68E-08	9.09E-06	5.956966	12	54150616	TSS5000;Body	202	*CISTR*	*CISTR*
cg11253785	5.68E-08	9.09E-06	5.95677	19	46806117	Body;Body;Body;TSS1500	-758	*HIF3A*	*HIF3A*
cg23690312	5.68E-08	9.09E-06	5.95669	19	22030534	Body;Body;Body;Body;Body	4333	*ZNF43*	*ZNF43*
cg10713715	5.69E-08	9.09E-06	5.956612	11	63533656	Body	2591	*C11orf95*	*C11orf95*
cg21219252	5.69E-08	9.09E-06	5.956472	9	1.36E+08	TES5000;TSS5000;TSS5000;TSS5000	3846	*LOC105376306;GTF3C5*	*LOC105376306*
cg25383568	5.69E-08	9.09E-06	5.956338	19	39217721	Body;Body;TES5000	17393	*ACTN4;CAPN12*	*CAPN12*
cg11208869	5.69E-08	9.09E-06	5.956303	5	1.54E+08	Body	-59602	*GALNT10*	*MIR1294*
cg10635350	5.70E-08	9.09E-06	5.956162	4	56708175	TES5000;TES5000	-11638	*EXOC1L*	*EXOC1*
cg25819103	5.70E-08	9.09E-06	5.956153	11	63906050	Body	27535	*MACROD1*	*MACROD1*
cg23870181	5.70E-08	9.10E-06	5.955891	6	32713044	Body;TES5000	3877	*HLA-DQA2;MIR3135B*	*HLA-DQA2*
cg04925437	5.72E-08	9.12E-06	5.955301	6	1.45E+08	Body	439196	*UTRN*	*SNORA98*
cg19735250	5.74E-08	9.14E-06	5.954555	17	43318610	Body	-6681	*FMNL1*	*MAP3K14-AS1*
cg05297692	5.74E-08	9.14E-06	5.954539	16	16228264	Body	89064	*ABCC1*	*ABCC6*
cg01927741	5.74E-08	9.15E-06	5.954287	9	1.4E+08	TSS5000;Body;TSS1500;TSS1500;TSS1500;TSS1500;TSS1500;TSS1500;TSS5000	-596	*FAM166A;STPG3-AS1;STPG3;NELFB*	*STPG3*
cg23615262	5.74E-08	9.15E-06	5.954242	1	1.67E+08		-42659		*POGK*
cg23881299	5.75E-08	9.15E-06	5.954179	10	1.21E+08	Body	-20493	*GRK5*	*MIR4681*
cg16178907	5.76E-08	9.16E-06	5.953723	12	1.25E+08	Body;Body;TSS1500	-1069	*ZNF664-RFLNA;RFLNA*	*RFLNA*
cg14480594	5.76E-08	9.16E-06	5.953648	9	1.12E+08	1stExon;TES5000;TES5000;TES5000	747	*ACTL7A;ELP1*	*ACTL7A*
cg06559575	5.76E-08	9.16E-06	5.953631	12	53490352	TSS1500	-1098	*IGFBP6*	*IGFBP6*
cg17895903	5.77E-08	9.17E-06	5.95326	7	1.29E+08	Body;Body;Body;TSS1500	-425	*NRF1*	*NRF1*
cg02981103	5.77E-08	9.18E-06	5.953024	14	24049380	TSS1500;TSS1500	-1381	*JPH4*	*JPH4*
cg26975787	5.79E-08	9.20E-06	5.952372	12	2613778	Body;Body;Body;Body;Body;Body;Body;Body;Body;Body;Body;Body;Body;Body;Body;Body;Body;Body;Body;Body;Body;Body;Body	167608	*CACNA1C*	*CACNA1C-AS2*
cg20539816	5.80E-08	9.21E-06	5.9519	17	5988249	Body	14316	*WSCD1*	*WSCD1*
cg17657322	5.81E-08	9.22E-06	5.951621	13	1.13E+08	Body	-30646	*SOX1-OT*	*SOX1*
cg19270286	5.81E-08	9.22E-06	5.951564	1	3562445	Body	4222	*WRAP73*	*WRAP73*
cg12234455	5.83E-08	9.24E-06	5.950968	19	49220235	5'UTR;Body;TSS200;TES5000	-21	*MAMSTR;RASIP1*	*MAMSTR*
cg22382058	5.83E-08	9.25E-06	5.950782	19	3841025	Body	28011	*ZFR2*	*ZFR2*
cg03399210	5.84E-08	9.25E-06	5.950573	8	37616064	TES5000;TES5000;TSS5000;TSS5000;TSS5000;TSS5000;TSS5000	-3999	*ERLIN2;PLPBP*	*PLPBP*
cg06005177	5.84E-08	9.26E-06	5.950201	10	1.35E+08	TSS5000;TSS5000;TSS5000;TSS5000;TSS5000;TSS5000;TSS5000;TSS5000	-2101	*ECHS1;PAOX*	*PAOX*
cg11717552	5.85E-08	9.27E-06	5.95005	14	23311678	Body	5885	*MMP14*	*MMP14*
cg25987871	5.86E-08	9.27E-06	5.949738	8	1.41E+08	Body;Body;Body	-120904	*TRAPPC9*	*PEG13*

Probe	p_adj	q_val	t	Chr	Pos.hg19	locations	dist2TSS	gene.Illumina	gene.nearest
cg12644929	5.86E-08	9.28E-06	5.949491	4	1.89E+08		-62703		*ZFP42*
cg02944637	5.87E-08	9.28E-06	5.949381	16	19733345	TSS5000;TSS5000;TSS5000;TSS5000;TSS5000;TSS5000;TSS5000;TSS5000;TSS5000;TSS5000;TSS5000;TSS5000;Body;Body;Body;Body;Body	-3796	*KNOP1;IQCK*	*KNOP1*
cg10276400	5.87E-08	9.28E-06	5.949252	16	17481251	Body	83566	*XYLT1*	*XYLT1*
cg01945840	5.87E-08	9.28E-06	5.949208	22	36013675	TSS1500;TSS200;Body;Body	-120	*MB*	*MB*
cg02549170	5.87E-08	9.28E-06	5.949082	4	1.86E+08	Body;Body;Body;Body;Body	20691	*PDLIM3*	*PDLIM3*
cg18965322	5.88E-08	9.28E-06	5.948989	2	99735582	Body;Body;Body;Body;Body;Body	22061	*TSGA10*	*TSGA10*
cg09514588	5.88E-08	9.29E-06	5.94886	10	1.19E+08	Body;TES5000	523	*HSPA12A;MIR9851*	*MIR9851*
cg18097503	5.88E-08	9.29E-06	5.948699	2	38442463		84217		*CYP1B1-AS1*
cg27168737	5.90E-08	9.31E-06	5.948055	1	2.38E+08	Body;Body	4718	*LOC100130331;ZP4*	*ZP4*
cg05716724	5.90E-08	9.31E-06	5.947849	8	56362167	Body;Body	5714	*XKR4;SBF1P1*	*SBF1P1*
cg01939381	5.91E-08	9.31E-06	5.947788	6	1.69E+08		103245		*LOC105378146*
cg00337558	5.91E-08	9.31E-06	5.947778	11	1.25E+08	Body	109777	*PKNOX2*	*PKNOX2*
cg08573687	5.91E-08	9.32E-06	5.94757	11	2193041	5'UTR;5'UTR;5'UTR;TSS1500	4	*TH;MIR4686*	*TH*
cg19185641	5.92E-08	9.33E-06	5.94729	6	16347197	Body;Body;Body	108380	*ATXN1*	*GMPR*
cg09921371	5.92E-08	9.33E-06	5.947113	2	1670073	Body	78214	*PXDN*	*PXDN*
cg22677498	5.92E-08	9.33E-06	5.947079	1	8004329	TSS5000	-3430	*TNFRSF9*	*TNFRSF9*
cg06513819	5.93E-08	9.33E-06	5.946949	4	13300507		185457		*RAB28*
cg04766779	5.96E-08	9.39E-06	5.945478	17	30596481	Body;Body;Body;Body;Body	3287	*RHBDL3*	*RHBDL3*
cg16320910	5.97E-08	9.39E-06	5.94539	20	23614541	Body;3'UTR	4051	*CST3*	*CST3*
cg13968809	5.98E-08	9.40E-06	5.945013	9	98787613	TSS5000	-3520	*LINC00092*	*LINC00092*
cg13771227	5.99E-08	9.41E-06	5.944515	9	1.34E+08	Body;Body;Body;Body	4073	*AIF1L*	*AIF1L*
cg06403617	5.99E-08	9.41E-06	5.944486	10	88140970		-14418		*GRID1*
cg00160302	5.99E-08	9.41E-06	5.944385	8	53323549	TSS1500;TSS1500	-1110	*ST18*	*ST18*
cg17850539	5.99E-08	9.41E-06	5.94435	1	2.04E+08	3'UTR;Body	9208	*ATP2B4*	*LINC00260*
cg25238480	6.00E-08	9.41E-06	5.944263	11	11335633	Body;Body	39164	*GALNT18*	*CSNK2A3*
cg09070760	6.01E-08	9.42E-06	5.943868	17	5412948	Body;TES5000;TES5000;TES5000;TES5000	-8622	*NLRP1*	*LOC728392*
cg24186249	6.01E-08	9.42E-06	5.943797	17	46092692		-17555		*MIR10226*
cg22723960	6.02E-08	9.43E-06	5.943491	1	1134018	TES5000;TES5000;TES5000;TES5000;TES5000	7954	*TTLL10;TNFRSF18*	*TNFRSF18*
cg02615561	6.02E-08	9.44E-06	5.943211	5	1.38E+08		-26194		*CDC23*
cg01297349	6.02E-08	9.44E-06	5.94319	12	2049039	TSS5000;TES5000	-3297	*LINC00940;DCP1B*	*LINC00940*
cg11863863	6.03E-08	9.44E-06	5.943102	17	75706410		18231		*LINC01987*
cg02256198	6.03E-08	9.44E-06	5.943016	4	81306753	Body;Body	49880	*CFAP299*	*CFAP299*
cg09548420	6.04E-08	9.45E-06	5.942614	8	1.45E+08	TES5000;TES5000;TES5000;TES5000;TES5000;TES5000;TES5000;TES5000;TES5000;TES5000;TES5000;TES5000;Body	92	*ZNF707;BREA2*	*BREA2*
cg17933148	6.04E-08	9.45E-06	5.942598	7	20122468	Body	57581	*LOC101927668*	*LOC101927668*
cg00802237	6.04E-08	9.45E-06	5.942534	14	94858066	TSS5000;TSS1500;TSS1500;TSS1500;TSS1500;TSS1500;TSS1500;TSS1500;TSS1500;TSS1500;TSS1500	-1094	*SERPINA1*	*SERPINA1*
cg04794202	6.05E-08	9.45E-06	5.942342	16	75469775	TSS5000	-2374	*CFDP1*	*CFDP1*
cg16261114	6.05E-08	9.45E-06	5.942308	15	99551997	TSS1500;TSS5000	-976	*PGPEP1L*	*PGPEP1L*
cg16647364	6.05E-08	9.45E-06	5.942188	7	1.49E+08	Body	16653	*SSPO*	*SSPO*
cg14633455	6.05E-08	9.45E-06	-5.94211	9	1.16E+08	TSS200;TSS200;TSS200	-154	*ZFP37*	*ZFP37*
cg11572080	6.05E-08	9.46E-06	5.941993	6	1.12E+08	TES5000;TES5000;Body;Body;Body	1923	*TRAF3IP2-AS1;TRAF3IP2*	*TRAF3IP2*
cg14697605	6.06E-08	9.46E-06	5.941788	8	22240140	Body;Body;Body;Body;Body;Body;Body;Body	-10208	*SLC39A14*	*SLC39A14*
cg05909343	6.08E-08	9.48E-06	5.941213	10	1.33E+08	Body	58497	*TCERG1L*	*TCERG1L*
cg24827784	6.08E-08	9.48E-06	5.941082	11	1.19E+08	TES5000;Body;TES5000	1146	*CBL;MCAM;MIR6756*	*MIR6756*
cg15726896	6.09E-08	9.50E-06	5.940459	10	35090668	Body;Body;Body;Body;Body;Body;Body;Body;Body;Body;Body	13556	*PARD3*	*PARD3*
cg20025115	6.10E-08	9.52E-06	5.940083	18	77560833		-62834		*KCNG2*
cg13562353	6.11E-08	9.52E-06	5.939854	9	34662773	TES5000;TES5000;TSS200;TES5000;TES5000;TES5000	-119	*IL11RA;CCL27;LOC730098*	*CCL27*
cg22549984	6.11E-08	9.52E-06	5.939832	10	395367	Body	169434	*DIP2C*	*ZMYND11*
cg07139794	6.12E-08	9.53E-06	5.939413	6	33449735		27383		*ZBTB9*
cg02203881	6.13E-08	9.54E-06	5.939231	15	42386909	TSS200	-149	*PLA2G4D*	*PLA2G4D*
cg23547656	6.13E-08	9.54E-06	5.939185	11	48161861	Body	43528	*PTPRJ*	*MIR3161*
cg10085305	6.14E-08	9.54E-06	5.93887	17	77704942	1stExon	238	*ENPP7*	*ENPP7*
cg10138630	6.14E-08	9.54E-06	5.938862	17	74024966	TSS5000;TSS5000	-1579	*EVPL*	*EVPL*
cg18305790	6.14E-08	9.54E-06	5.938833	3	48453562	Body;Body	17310	*PLXNB1*	*PLXNB1*
cg10980293	6.14E-08	9.54E-06	5.938782	7	1.51E+08	TSS1500;TSS1500;TSS200;TES5000	-148	*ASB10;IQCA1L*	*ASB10*
cg17223218	6.14E-08	9.54E-06	5.938717	1	1.83E+08	Body	16186	*LAMC1*	*LAMC1*
cg04105350	6.14E-08	9.54E-06	5.938707	1	2111868	Body;Body;Body;Body;Body;Body;Body;Body;Body;TES5000;TES5000;TES5000;TES5000;TES5000;TES5000	3446	*PRKCZ;PRKCZ-AS1;FAAP20*	*PRKCZ-AS1*
cg17314180	6.15E-08	9.54E-06	5.938451	2	2.09E+08	Body;TSS200	-171	*LOC100507443;CRYGB*	*CRYGB*
cg17605604	6.16E-08	9.55E-06	5.938121	5	1.03E+08	3'UTR;3'UTR;3'UTR	17775	*C5orf30*	*C5orf30*

Probe	p_adj	q_val	t	Chr	Pos.hg19	locations	dist2TSS	gene.Illumina	gene.nearest
cg07670676	6.16E-08	9.56E-06	5.937868	10	1E+08	Body;TES5000;TES5000;TES5000;TES5000;TES5000;TES5000;TES5000;TES5000;TES5000;TES5000;TES5000;TES5000;TES5000;TES5000	3250	*PYROXD2;HPS1*	*PYROXD2*
cg12178316	6.17E-08	9.56E-06	5.937735	15	22944125	Body;Body;Body;Body;Body;Body;Body;Body;Body	-11629	*CYFIP1*	*CYFIP1*
cg19640585	6.18E-08	9.58E-06	5.937354	6	1.44E+08	Body;Body;Body;Body	75516	*PHACTR2*	*PHACTR2*
cg22156852	6.18E-08	9.58E-06	5.937086	1	1.13E+08	Body	6847	*CTTNBP2NL*	*CTTNBP2NL*
cg21249563	6.19E-08	9.59E-06	5.936838	2	29297717	TSS1500	-328	*PCARE*	*PCARE*
cg27019059	6.20E-08	9.60E-06	5.936495	3	1.34E+08		-76031		*LINC02000*
cg22916215	6.20E-08	9.60E-06	5.936342	14	1E+08	Body	14623	*CCDC85C*	*CCDC85C*
cg16998016	6.21E-08	9.62E-06	5.935977	7	4051915	Body	-117401	*SDK1*	*SDK1*
cg16693369	6.22E-08	9.63E-06	5.935561	4	1.56E+08		-13579		*FGG*
cg17873456	6.23E-08	9.63E-06	5.935478	13	1.07E+08		55647		*EFNB2*
cg00507477	6.23E-08	9.63E-06	5.935244	15	90292483	TES5000	2057	*MESP1*	*MESP1*
cg17221945	6.23E-08	9.63E-06	5.935211	1	39953603	TES5000;TSS5000	-3704	*MACF1;BMP8A*	*BMP8A*
cg27073349	6.23E-08	9.63E-06	5.935188	4	3640481		39101		*LINC02171*
cg05126581	6.24E-08	9.64E-06	5.935004	1	2.16E+08	Body	-100987	*USH2A*	*LOC102723833*
cg08251765	6.24E-08	9.64E-06	5.934924	8	1.36E+08	Body;Body;Body;Body;Body;Body;Body	31767	*ZFAT*	*ZFAT-AS1*
cg25353436	6.25E-08	9.64E-06	5.934701	10	10839523	Body;Body;Body;Body;Body;Body;TSS5000;Body;Body;TSS1500;TSS1500;TSS1500	722	*CELF2;SFTA1P*	*CELF2*
cg27054948	6.28E-08	9.69E-06	5.93345	8	18537539	Body;Body;Body	128838	*PSD3*	*PSD3*
cg12150538	6.29E-08	9.69E-06	5.933216	12	92886824		26370		*LINC02397*
cg19120695	6.29E-08	9.69E-06	5.933211	4	7528947	Body	67193	*SORCS2*	*MIR4274*
cg14171369	6.29E-08	9.70E-06	5.933009	14	75447688		-21935		*EIF2B2*
cg20227326	6.30E-08	9.71E-06	5.932608	8	25227128	Body	-8100	*DOCK5*	*LOC105379331*
cg25196881	6.31E-08	9.72E-06	5.932423	15	39780412		-92867		*THBS1*
cg23330006	6.32E-08	9.73E-06	5.932094	12	1.07E+08	Body	26098	*NUAK1*	*NUAK1*
cg00866295	6.32E-08	9.73E-06	5.931871	1	2.04E+08	Body;Body;Body;Body	-38232	*ATP2B4*	*SNORA77*
cg21828629	6.33E-08	9.73E-06	5.931799	1	2.06E+08	TSS1500;TSS1500	-834	*SLC26A9*	*SLC26A9*
cg23051970	6.33E-08	9.73E-06	5.931747	17	75284193	Body;Body;5'UTR	221	*SEPTIN9*	*SEPTIN9*
cg02941286	6.33E-08	9.73E-06	5.93173	4	778336	TSS5000;TSS5000;TES5000	-2700	*LOC100129917;CPLX1*	*LOC100129917*
cg11337160	6.35E-08	9.76E-06	5.931013	22	36014182	TSS1500;TSS1500;Body;Body	-627	*MB*	*MB*
cg08552167	6.35E-08	9.76E-06	5.930767	10	1.35E+08	Body;Body	39542	*INPP5A*	*NKX6-2*
cg21492137	6.36E-08	9.76E-06	5.930723	14	1.02E+08	TES5000;TES5000;TES5000;TES5000;TSS200;TSS1500;TSS5000;TSS5000	-152	*MIR134;MIR668;MIR485;MIR323B;MIR154;MIR496;MIR377;MIR541*	*MIR154*
cg09681025	6.36E-08	9.76E-06	5.930689	2	1.84E+08	TSS5000;TSS5000;TSS5000;TSS5000;TSS5000	-3358	*DNAJC10*	*DNAJC10*
cg13188915	6.36E-08	9.76E-06	5.930586	12	25622615		83602		*LMNTD1*
cg14744919	6.36E-08	9.76E-06	5.930448	8	17269638	Body	1222	*MTMR7*	*MTMR7*
cg22685502	6.37E-08	9.76E-06	5.930179	3	72496851	TSS1500	-1077	*RYBP*	*RYBP*
cg08928038	6.37E-08	9.76E-06	5.930147	17	39868610	5'UTR	28	*GAST*	*GAST*
cg14447703	6.37E-08	9.76E-06	5.930138	6	30846517	TSS5000	-3872	*DDR1*	*DDR1*
cg08306084	6.37E-08	9.76E-06	5.930123	6	33248546	TES5000;TES5000;Body;Body	3647	*RPS18;B3GALT4;WDR46*	*B3GALT4*
cg21023831	6.37E-08	9.76E-06	5.930103	2	2.19E+08	Body;Body;Body;TSS5000	-4433	*TNS1;MIR6809*	*MIR6809*
cg01949837	6.38E-08	9.77E-06	5.929755	8	1.33E+08	Body;Body	-26325	*KCNQ3*	*HHLA1*
cg06513139	6.38E-08	9.77E-06	5.929723	19	4455923	TSS1500;Body	-625	*UBXN6*	*UBXN6*
cg19961522	6.39E-08	9.77E-06	5.929563	20	30407388	1stExon	230	*MYLK2*	*MYLK2*
cg10414006	6.39E-08	9.77E-06	5.929487	16	34984121	Body	3199	*LINC02167*	*LINC02167*
cg18220799	6.41E-08	9.79E-06	5.928906	18	44099340	Body;TSS1500;Body;TSS1500;Body	-291	*LOXHD1*	*LOXHD1*
cg01792145	6.41E-08	9.79E-06	5.928879	1	1.13E+08	Body;Body;TSS1500	-492	*SLC16A1;AKR7A2P1*	*AKR7A2P1*
cg06738548	6.41E-08	9.79E-06	5.92883	15	94019180		269886		*LOC105370980*
cg03784197	6.43E-08	9.81E-06	5.928195	16	88471457	Body	22091	*ZNF469*	*ZNF469*
cg13108289	6.43E-08	9.81E-06	5.928054	14	1.05E+08	TES5000;TES5000;Body;Body	17768	*PLD4;AHNAK2*	*PLD4*
cg10986946	6.43E-08	9.81E-06	5.928024	11	12001622	Body;Body;Body;Body	28564	*DKK3*	*DKK3*
cg22331294	6.43E-08	9.81E-06	5.927994	3	45698299	Body	32075	*LIMD1*	*LIMD1-AS1*
cg26182418	6.46E-08	9.86E-06	5.926814	14	89715336	Body;Body	168241	*FOXN3*	*FOXN3*
cg21757633	6.47E-08	9.86E-06	5.926669	22	50847567	Body;Body;Body;Body;Body;Body;Body;Body;Body;Body;Body;Body;Body;TES5000	1697	*PPP6R2;MIR12114*	*MIR12114*
cg03455107	6.48E-08	9.87E-06	5.926386	17	54509207	Body;Body;Body	-161852	*ANKFN1*	*NOG*
cg13374432	6.48E-08	9.87E-06	5.926353	15	86192745	Body;Body;Body	29565	*AKAP13*	*AKAP13*
cg10860364	6.49E-08	9.88E-06	5.926027	17	27936184	Body	8951	*ANKRD13B*	*CORO6*
cg20642630	6.49E-08	9.88E-06	5.925917	8	22846006	Body	1243	*RHOBTB2*	*RHOBTB2*
cg10860802	6.50E-08	9.89E-06	5.925638	7	1.55E+08	Body;Body;Body;Body;Body;Body;Body;Body;Body;Body	-87624	*DPP6*	*PAXIP1-AS2*
cg23225572	6.50E-08	9.89E-06	5.925474	1	1.62E+08	Body;Body;Body	3210	*NOS1AP*	*NOS1AP*
cg13947528	6.51E-08	9.90E-06	5.925222	9	1.37E+08	Body;Body	-122055	*VAV2*	*SARDH*

Probe	p_adj	q_val	t	Chr	Pos.hg19	locations	dist2TSS	gene.Illumina	gene.nearest
cg22957339	6.52E-08	9.91E-06	5.924903	15	31246272	Body	37559	*MTMR10*	*MTMR10*
cg07805345	6.52E-08	9.91E-06	5.924777	3	46922486	Body;TSS1500	-1252	*PTH1R*	*PTH1R*
cg17498476	6.53E-08	9.91E-06	5.924577	7	2052420	Body;Body;Body;Body;Body	-72213	*MAD1L1*	*MAD1L1*
cg18617770	6.53E-08	9.91E-06	5.924551	12	1.33E+08		-30797		*FBRSL1*
cg02345148	6.54E-08	9.93E-06	5.924039	2	46678772		22444		*LINC02583*
cg12145085	6.55E-08	9.94E-06	5.923825	14	73289895	Body;Body;Body;Body	69462	*DPF3*	*DPF3*
cg24710938	6.55E-08	9.94E-06	5.923772	13	36788861	TSS200;Body;TSS200	-109	*SOHLH2;CCDC169-SOHLH2*	*SOHLH2*
cg26424967	6.56E-08	9.94E-06	5.923487	1	31197383	TSS1500;Body	-951	*MATN1;MATN1-AS1*	*MATN1*
cg26859186	6.56E-08	9.94E-06	5.92347	7	1.57E+08	Body;Body;Body;Body;Body	15406	*PTPRN2*	*MIR153-2*
cg20321706	6.56E-08	9.94E-06	5.923438	12	1.33E+08	Body	-6102	*GALNT9*	*LOC100130238*
cg07042007	6.56E-08	9.94E-06	5.923405	4	2303638	Body;Body;Body	-39802	*ZFYVE28*	*MXD4*
cg11841288	6.58E-08	9.98E-06	5.922515	1	53842221		48317		*LINC01771*
cg21959757	6.59E-08	9.99E-06	5.922183	5	1.15E+08		-17389		*LINCADL*
cg23049178	6.60E-08	9.99E-06	5.922079	3	72939830	Body;Body	2634	*GXYLT2*	*GXYLT2*
cg14559176	6.60E-08	9.99E-06	5.921944	9	1.39E+08		-19680		*UBAC1*
cg00219792	6.60E-08	9.99E-06	5.921792	12	83053315		-27369		*TMTC2*
cg25599641	6.62E-08	1.00E-05	5.921274	22	42456139	3'UTR;3'UTR;3'UTR	10677	*NAGA*	*NAGA*
cg00646185	6.62E-08	1.00E-05	5.921118	15	26234833	Body	87327	*LINC02346*	*LINC02346*
cg16884601	6.63E-08	1.00E-05	5.920787	11	65712968	TSS200	-146	*TSGA10IP*	*TSGA10IP*
cg13859639	6.64E-08	1.00E-05	5.92065	11	2846716	Body;Body	36083	*KCNQ1*	*KCNQ1-AS1*
cg10077060	6.64E-08	1.00E-05	5.920552	12	76177845		247711		*PHLDA1*
cg01739737	6.65E-08	1.00E-05	5.920018	14	1.02E+08	Body	48324	*DYNC1H1*	*DYNC1H1*
cg19969694	6.66E-08	1.01E-05	5.9197	15	41185800	TSS1500	-847	*VPS18*	*VPS18*
cg17808746	6.67E-08	1.01E-05	5.919563	2	74571836	TSS1500	-1293	*SLC4A5*	*SLC4A5*
cg01567656	6.67E-08	1.01E-05	5.91947	11	76895869	Body;Body;Body	56578	*MYO7A*	*MYO7A*
cg25966817	6.67E-08	1.01E-05	5.919452	5	60170058	3'UTR;3'UTR;3'UTR	-29957	*ERCC8*	*ELOVL7*
cg02600349	6.68E-08	1.01E-05	5.91928	22	39051817	Body;Body;TSS1500;TSS1500	580	*FAM227A;CBY1*	*FAM227A*
cg03699459	6.68E-08	1.01E-05	5.918999	10	4864636	TSS5000;TSS5000;TSS5000;TSS5000;TSS5000;TSS5000	-3732	*AKR1E2*	*AKR1E2*
cg26456255	6.69E-08	1.01E-05	5.918868	19	38698718	3'UTR;TES5000;TES5000;TES5000;TES5000;TES5000	16172	*SIPA1L3;DPF1*	*DPF1*
cg00020419	6.69E-08	1.01E-05	5.918704	6	43273548	TES5000;TES5000;3'UTR;3'UTR	3016	*SLC22A7;CRIP3*	*CRIP3*
cg20997656	6.69E-08	1.01E-05	5.918636	6	1.59E+08	Body;Body	66982	*SYNJ2*	*SYNJ2*
cg20059312	6.70E-08	1.01E-05	5.918353	2	2.34E+08	5'UTR;Body	109	*NGEF;LOC101928881*	*NGEF*
cg11902961	6.71E-08	1.01E-05	5.91797	17	4835341	TSS1500	-230	*GP1BA*	*GP1BA*
cg22136051	6.71E-08	1.01E-05	5.917952	7	6192053	Body;Body	19715	*USP42*	*CYTH3*
cg04441333	6.72E-08	1.01E-05	5.917626	7	1.58E+08	Body;Body;Body;Body;Body	-134087	*PTPRN2*	*LOC100506585*
cg03837963	6.73E-08	1.01E-05	5.917538	16	15666930	Body;Body	38022	*BMERB1*	*MIR6506*
cg04909420	6.75E-08	1.01E-05	5.916774	6	1.6E+08		-22281		*IGF2R*
cg05208483	6.75E-08	1.01E-05	5.916574	15	75975041	Body	30148	*CSPG4*	*CSPG4*
cg21377881	6.76E-08	1.01E-05	5.916402	22	40064566	Body;Body	97809	*CACNA1I*	*CACNA1I*
cg21263471	6.76E-08	1.01E-05	5.916366	17	56399584	Body;Body;Body;TSS5000	-3226	*TSPOAP1;TSPOAP1-AS1*	*TSPOAP1-AS1*
cg08769634	6.77E-08	1.01E-05	5.91614	16	71657362	TSS5000;TSS5000;TSS5000	-2724	*MARVELD3*	*MARVELD3*
cg16759954	6.77E-08	1.01E-05	5.9161	17	6461153	TSS1500;TSS1500	-1278	*PITPNM3*	*PITPNM3*
cg08063745	6.77E-08	1.01E-05	5.916056	20	44690652	TES5000;TES5000;3'UTR;3'UTR;3'UTR;3'UTR;3'UTR	27949	*SLC12A5;NCOA5*	*NCOA5*
cg11586930	6.77E-08	1.01E-05	5.915949	1	59314514	Body;Body;Body	63692	*LINC01135*	*LINC01135*
cg03072740	6.77E-08	1.01E-05	5.915901	12	56030437	TSS1500	-238	*OR10P1*	*OR10P1*
cg23023126	6.78E-08	1.02E-05	5.915727	19	308808	Body;Body	-17405	*MIER2*	*PLPP2*
cg08722720	6.78E-08	1.02E-05	5.915518	15	34331557	TSS200;Body;Body	-152	*AVEN;CHRM5*	*AVEN*
cg07315051	6.79E-08	1.02E-05	5.915505	2	2.4E+08	Body	51594	*HDAC4*	*MGC16025*
cg11851915	6.79E-08	1.02E-05	5.915499	1	44060116	Body;Body;Body;Body;Body;Body	-55703	*PTPRF*	*KDM4A*
cg14078070	6.79E-08	1.02E-05	5.915222	6	1.67E+08	Body;Body;Body	-10779	*RPS6KA2*	*RPS6KA2*
cg09778734	6.81E-08	1.02E-05	5.914803	12	1.11E+08	5'UTR;Body;Body	111	*C12orf76*	*C12orf76*
cg00488734	6.81E-08	1.02E-05	5.914575	16	58076165	Body	16508	*MMP15*	*MMP15*
cg26588612	6.81E-08	1.02E-05	5.914497	6	1.49E+08		-31126		*SASH1*
cg01548249	6.82E-08	1.02E-05	5.914477	19	7582188	Body	1200	*ZNF358*	*ZNF358*
cg24844387	6.82E-08	1.02E-05	5.914404	6	20215399	TSS5000;TSS5000	-2699	*MBOAT1*	*MBOAT1*
cg00976532	6.82E-08	1.02E-05	5.914322	16	2636476	Body;Body;Body	-16908	*PDPK1*	*LOC652276*
cg23065576	6.83E-08	1.02E-05	5.913812	20	25207247	3'UTR;3'UTR	-21473	*ENTPD6*	*PYGB*
cg27228272	6.84E-08	1.02E-05	5.913785	2	1.28E+08		67303		*BIN1*
cg18234542	6.84E-08	1.02E-05	5.913661	14	78553121		-83594		*NRXN3*

Probe	p_adj	q_val	t	Chr	Pos.hg19	locations	dist2TSS	gene.Illumina	gene.nearest
cg02701826	6.84E-08	1.02E-05	5.913638	19	15903794	TSS1500	-1064	*OR10H5*	*OR10H5*
cg21550098	6.84E-08	1.02E-05	5.913522	4	1.66E+08	TSS1500	-1476	*TRIM61*	*TRIM61*
cg21558893	6.85E-08	1.02E-05	5.913399	15	75950332	3'UTR;3'UTR	8985	*SNX33*	*SNX33*
cg07126979	6.85E-08	1.02E-05	5.913344	3	1.27E+08	Body	10852	*PODXL2*	*PODXL2*
cg17433546	6.86E-08	1.02E-05	5.912904	12	1.07E+08	Body;Body;Body;Body;Body;Body	2239	*TMEM263*	*TMEM263*
cg20424065	6.87E-08	1.02E-05	5.912508	6	1.52E+08	Body;Body;Body	10517	*AKAP12*	*AKAP12*
cg08193821	6.89E-08	1.03E-05	5.911856	6	5412087	Body;Body	46221	*FARS2*	*LOC101927972*
cg23369632	6.90E-08	1.03E-05	5.911773	6	56438637	Body;Body;Body;Body	69050	*DST*	*DST*
cg20413432	6.91E-08	1.03E-05	5.911393	17	45892150	Body	7010	*OSBPL7*	*OSBPL7*
cg05452618	6.92E-08	1.03E-05	5.910945	5	1271848	Body;Body;Body;Body	23335	*TERT*	*TERT*
cg08087047	6.93E-08	1.03E-05	5.910642	17	72461209	TSS1500;TSS5000;TSS5000;TSS5000	-1302	*CD300A*	*CD300A*
cg26145461	6.94E-08	1.03E-05	5.910263	6	2510637		-124096		*LINC02521*
cg04772457	6.95E-08	1.03E-05	5.910093	20	61822507	TES5000	12656	*YTHDF1*	*MIR124-3*
cg06632797	6.95E-08	1.03E-05	5.909877	11	68968215		-29185		*LOC338694*
cg21484910	6.95E-08	1.03E-05	5.909873	2	40097661		-47112		*SLC8A1-AS1*
cg04357398	6.96E-08	1.03E-05	5.909476	6	32365960	Body;TES5000;Body	7674	*TSBP1-AS1;HCG23;BTNL2*	*HCG23*
cg05211790	6.96E-08	1.03E-05	5.909459	5	34683189	Body;Body;Body;TSS1500;TSS1500;TSS5000	-1422	*RAI14*	*RAI14*
cg17013888	6.97E-08	1.03E-05	5.909151	2	1.53E+08	Body	2826	*FMNL2*	*FMNL2*
cg09414264	6.98E-08	1.03E-05	5.908947	11	3113709	Body;Body;Body	-35056	*OSBPL5*	*CARS1*
cg06143346	6.98E-08	1.03E-05	5.908816	3	62320945	3'UTR;3'UTR;3'UTR	16249	*C3orf14*	*C3orf14*
cg01800500	7.00E-08	1.04E-05	5.908196	14	95417572		180594		*GSC-DT*
cg11845785	7.01E-08	1.04E-05	5.907998	2	1.05E+08	Body;Body	8420	*LINC01102;LINC01103*	*LINC01103*
cg19732653	7.01E-08	1.04E-05	5.907958	1	2.46E+08	Body	-450979	*KIF26B*	*KIF26B-AS1*
cg24951140	7.01E-08	1.04E-05	5.907938	8	29156846		-36259		*KIF13B*
cg26651810	7.03E-08	1.04E-05	5.907325	16	53851122	Body;Body;Body;Body;Body;Body;Body;Body;Body;Body;Body;Body;Body	113055	*FTO*	*FTO*
cg25987842	7.03E-08	1.04E-05	5.907282	17	21326491	TES5000	46823	*KCNJ12*	*KCNJ12*
cg18994049	7.05E-08	1.04E-05	5.90674	11	69813825		96205		*LINC02753*
cg13373361	7.07E-08	1.04E-05	5.905985	7	98885594	Body	14671	*MYH16*	*MYH16*
cg17074213	7.07E-08	1.04E-05	-5.90583	1	92351695	5'UTR;5'UTR;Body;Body	13	*TGFBR3*	*TGFBR3*
cg25026926	7.08E-08	1.04E-05	5.905724	17	48266268	Body	12732	*COL1A1*	*COL1A1*
cg09031013	7.09E-08	1.05E-05	5.905264	21	35835170	TSS5000;TSS5000;Body;Body;Body;TSS5000;Body	-2934	*KCNE1*	*KCNE1*
cg21759500	7.11E-08	1.05E-05	5.904601	3	1.08E+08	Body	58074	*MYH15*	*MYH15*
cg19677739	7.11E-08	1.05E-05	5.904516	1	1.61E+08	TES5000;TES5000;Body;TES5000	2692	*NDUFS2;FCER1G;APOA2*	*FCER1G*
cg22822219	7.12E-08	1.05E-05	5.904388	14	24105017	Body;TSS1500;TSS1500	-555	*DHRS2*	*DHRS2*
cg11145643	7.13E-08	1.05E-05	5.903997	14	1.01E+08	Body;Body;Body;Body;TSS5000;TSS5000;TSS5000;TSS5000;TSS5000;TSS5000	1866	*WARS1;WDR25*	*WARS1*
cg14578500	7.14E-08	1.05E-05	5.903789	17	43191793	Body;Body	8	*PLCD3;MIR6784*	*MIR6784*
cg02577092	7.14E-08	1.05E-05	5.903718	5	73778483	Body	54318	*LINC01331*	*LINC01331*
cg04421632	7.14E-08	1.05E-05	5.903638	19	15618618	TSS1500	-717	*CYP4F22*	*CYP4F22*
cg21299491	7.14E-08	1.05E-05	5.903499	1	3057366	Body;Body	12828	*PRDM16*	*MIR4251*
cg18522038	7.16E-08	1.05E-05	5.903064	17	30568596		-24254		*RHBDL3*
cg23230478	7.16E-08	1.05E-05	5.90304	1	6360620	Body;Body;Body;Body	-39462	*ACOT7*	*GPR153*
cg00292947	7.16E-08	1.05E-05	5.902852	15	78499137	Body;Body	27770	*ACSBG1*	*ACSBG1*
cg06668976	7.17E-08	1.05E-05	5.902733	5	1.71E+08		-83184		*SMIM23*
cg02800747	7.17E-08	1.05E-05	5.902542	17	77047094	TES5000;TES5000;TES5000;TES5000;TES5000	16714	*C1QTNF1*	*C1QTNF1*
cg10948036	7.19E-08	1.06E-05	5.902005	10	1.35E+08	Body;Body	11128	*ADGRA1*	*ADGRA1*
cg09123026	7.19E-08	1.06E-05	5.901868	17	74480528	Body;Body	3463	*RHBDF2*	*RHBDF2*
cg05269713	7.21E-08	1.06E-05	5.901375	17	48353633	Body;Body	1807	*TMEM92*	*TMEM92*
cg00227651	7.21E-08	1.06E-05	5.901328	12	1.28E+08		-55705		*LINC02393*
cg04791129	7.22E-08	1.06E-05	5.900967	12	53490632	TSS1500	-818	*IGFBP6*	*IGFBP6*
cg14351526	7.23E-08	1.06E-05	5.900629	6	72086737	TSS200	-3	*MIR30C2*	*MIR30C2*
cg10165842	7.23E-08	1.06E-05	5.90061	11	1155095	Body	3516	*MUC5AC*	*MUC5AC*
cg01773858	7.26E-08	1.06E-05	5.899776	11	73076892	Body	-10551	*ARHGEF17*	*RELT*
cg14790552	7.27E-08	1.07E-05	5.899443	6	30866999	Body;Body;Body;Body;Body;Body;Body;3'UTR;Body	8340	*DDR1*	*MIR4640*
cg05016066	7.27E-08	1.07E-05	5.899403	16	66377143		-23450		*CDH5*
cg12036817	7.28E-08	1.07E-05	5.899135	11	405846	TES5000;TES5000;3'UTR;3'UTR;3'UTR	9153	*PKP3;SIGIRR*	*SIGIRR*
cg17527348	7.28E-08	1.07E-05	5.899127	22	22285359	Body	-7249	*PPM1F*	*PPM1F-AS1*
cg09596025	7.28E-08	1.07E-05	5.899037	16	84090260	Body	-14498	*MBTPS1*	*SLC38A8*
cg16263801	7.30E-08	1.07E-05	5.898592	11	2444866	TSS1500	-591	*TRPM5*	*TRPM5*
cg13487183	7.31E-08	1.07E-05	5.89815	13	78428203	Body	35132	*EDNRB-AS1*	*EDNRB-AS1*
cg08834602	7.31E-08	1.07E-05	5.898041	19	44220122	TSS200	-105	*IRGC*	*IRGC*

Probe	p_adj	q_val	t	Chr	Pos.hg19	locations	dist2TSS	gene.Illumina	gene.nearest
cg08028035	7.32E-08	1.07E-05	5.897807	3	42252958	Body;Body;3'UTR;Body;Body;Body;3'UTR;Body;Body;3'UTR	51274	*TRAK1*	*TRAK1*
cg10041069	7.32E-08	1.07E-05	5.897798	10	1560628	Body	-8196	*ADARB2*	*ADARB2-AS1*
cg15068428	7.32E-08	1.07E-05	5.897743	11	6473744	Body;Body;Body;Body	-11527	*TRIM3*	*HPX*
cg11161150	7.33E-08	1.07E-05	5.89767	5	98119892	Body;Body;Body;Body;Body	10526	*RGMB*	*RGMB*
cg05880452	7.33E-08	1.07E-05	5.897621	2	1.53E+08	TSS1500	-656	*FMNL2*	*FMNL2*
cg07751803	7.34E-08	1.07E-05	5.897137	19	47258810	1stExon;1stExon	-9103	*FKRP*	*STRN4*
cg01628245	7.35E-08	1.07E-05	5.896997	2	97464763	Body;TES5000	749	*CNNM4;MIR3127*	*MIR3127*
cg12255062	7.35E-08	1.07E-05	5.896869	6	32138332	TES5000;TES5000;TES5000;Body;Body;Body;Body;Body;TSS1500	-439	*PPT2-EGFL8;EGFL8;AGPAT1;MIR6721*	*MIR6721*
cg21452808	7.35E-08	1.07E-05	5.896769	1	25900135	TES5000	30039	*LDLRAP1*	*LDLRAP1*
cg09741713	7.36E-08	1.07E-05	5.896675	12	62471915	Body	114705	*TAFA2*	*TAFA2*
cg10855945	7.36E-08	1.07E-05	5.896621	6	1.66E+08	Body;Body	-28811	*PDE10A*	*C6orf118*
cg21546868	7.36E-08	1.07E-05	5.896607	14	1.06E+08	TES5000;TES5000	13215	*JAG2*	*MIR6765*
cg11340785	7.37E-08	1.07E-05	5.896261	5	61374735		-227253		*KIF2A*
cg02647493	7.37E-08	1.07E-05	5.89615	6	32019042	Body;Body	-5137	*TNXB*	*TNXB*
cg07993372	7.39E-08	1.07E-05	5.89568	16	427084	Body	4894	*PGAP6*	*PGAP6*
cg13002506	7.40E-08	1.08E-05	5.895459	11	76998821	Body	13890	*GDPD4*	*GDPD4*
cg04576048	7.41E-08	1.08E-05	5.895023	1	2.06E+08	3'UTR;Body	-15267	*PM20D1*	*SLC41A1*
cg09841666	7.41E-08	1.08E-05	5.894957	20	58478713	Body	29979	*SYCP2*	*SYCP2*
cg15507787	7.41E-08	1.08E-05	5.894877	10	93802554	TES5000;TES5000	119028	*CPEB3*	*BTAF1*
cg04103326	7.42E-08	1.08E-05	5.89474	6	34489623	Body;Body	6975	*PACSIN1*	*PACSIN1*
cg21655267	7.43E-08	1.08E-05	5.894406	2	1.29E+08		-7028		*HS6ST1*
cg07048527	7.43E-08	1.08E-05	5.894278	17	46700449	Body	3271	*HOXB9*	*HOXB9*
cg00703885	7.44E-08	1.08E-05	5.894014	2	1.28E+08	TSS1500	-887	*LIMS2*	*LIMS2*
cg26879644	7.44E-08	1.08E-05	5.89399	15	92238013		-158924		*SLCO3A1*
cg13296093	7.44E-08	1.08E-05	5.893917	6	31868740	TSS5000;TSS5000;TSS5000;TSS5000;TSS5000;Body;Body;TSS200	-35	*EHMT2;C2;ZBTB12*	*C2*
cg11931798	7.45E-08	1.08E-05	5.8937	12	20246350	Body	34485	*LINC02398*	*LINC02468*
cg12870830	7.46E-08	1.08E-05	5.893407	11	1.19E+08	Body;TSS5000;Body	2078	*MFRP;C1QTNF5*	*MFRP*
cg16896008	7.47E-08	1.08E-05	5.893249	6	24206177	Body;Body	79735	*DCDC2*	*NRSN1*
cg13263323	7.48E-08	1.08E-05	5.892772	15	86062960	Body;Body	-100220	*AKAP13*	*AKAP13*
cg08204369	7.49E-08	1.08E-05	5.892619	17	73150733	TSS1500;TSS1500;TSS200;TSS200;TSS200;TSS200;TSS200	-126	*JPT1*	*JPT1*
cg12303582	7.49E-08	1.08E-05	5.89237	10	3169516	Body;Body;Body;Body;Body;Body;Body;Body;Body;Body;Body	-6779	*PFKP*	*SNORD142*
cg07730159	7.50E-08	1.09E-05	5.892212	12	29299659	TSS5000	-2551	*FAR2*	*FAR2*
cg13193392	7.51E-08	1.09E-05	5.891915	13	60055857		-530994		*DIAPH3-AS1*
cg10276116	7.51E-08	1.09E-05	5.891899	6	1.55E+08	Body;Body;Body;Body	116095	*OPRM1;IPCEF1*	*IPCEF1*
cg12103547	7.51E-08	1.09E-05	5.891862	6	1.69E+08	Body;Body	209093	*SMOC2*	*SMOC2*
cg27410165	7.51E-08	1.09E-05	5.891751	11	18978169		-21620		*MRGPRX1*
cg00066828	7.52E-08	1.09E-05	5.891552	17	1837612	TES5000	-83397	*RTN4RL1*	*LOC105371485*
cg16929041	7.52E-08	1.09E-05	5.891537	17	26648951	Body	2726	*TMEM97*	*TMEM97*
cg16899036	7.52E-08	1.09E-05	5.89147	19	19052705	TSS5000;TSS5000;TSS5000;TSS5000;TSS1500;Body;Body	-679	*HOMER3;HOMER3-AS1*	*HOMER3*
cg23034420	7.54E-08	1.09E-05	5.89103	21	43434317	TSS5000;TSS5000;TSS5000;TSS5000;TSS5000;Body	-3848	*ZBTB21;ZNF295-AS1*	*ZBTB21*
cg22274234	7.55E-08	1.09E-05	5.890713	12	1.1E+08	TES5000;3'UTR;3'UTR;3'UTR;Body;Body	26247	*MYO1H;KCTD10*	*KCTD10*
cg14723566	7.55E-08	1.09E-05	5.890607	15	80711027	Body	14304	*ARNT2*	*ARNT2*
cg18152154	7.57E-08	1.09E-05	5.88995	1	46639632	Body;Body;TSS1500;TES5000	941	*PIK3R3;LOC110117498-PIK3R3;TSPAN1;P3R3URF*	*PIK3R3*
cg14252164	7.57E-08	1.09E-05	5.889943	6	44019598	Body	22791	*LOC101929705*	*LOC101929705*
cg11767711	7.59E-08	1.09E-05	5.889292	19	36358783	TES5000;TES5000;TES5000;TES5000;TES5000;TSS1500;TSS1500	-563	*KIRREL2;APLP1*	*APLP1*
cg00359511	7.60E-08	1.09E-05	5.88919	16	28509413	Body;TES5000	3450	*APOBR;IL27*	*APOBR*
cg02202315	7.60E-08	1.09E-05	5.889015	2	98357867	TES5000;TES5000	6999	*ZAP70*	*ZAP70*
cg03766174	7.62E-08	1.10E-05	5.888418	16	81547907	Body;Body	18949	*CMIP*	*CMIP*
cg08686879	7.63E-08	1.10E-05	5.888305	5	1.31E+08	1stExon	153	*CSF2*	*CSF2*
cg04851348	7.63E-08	1.10E-05	5.888156	1	1.63E+08	Body	12490	*CCDC190*	*CCDC190*
cg02504734	7.63E-08	1.10E-05	5.888137	16	70955865	Body	-120804	*HYDIN*	*VAC14*
cg09855212	7.63E-08	1.10E-05	5.888042	11	85427572	Body;Body;Body;Body;Body;Body;Body;Body;Body;Body;Body;Body;Body;Body;Body;Body;Body;Body;Body;Body;Body	2565	*SYTL2*	*SYTL2*
cg24987745	7.64E-08	1.10E-05	5.888002	2	17819167	Body;Body;Body;Body;Body	97205	*VSNL1*	*VSNL1*
cg09037726	7.64E-08	1.10E-05	5.887973	4	76995173	Body;TSS1500;TSS1500	-674	*ART3*	*ART3*
cg20356136	7.64E-08	1.10E-05	5.887862	4	7802238	Body;Body;Body;Body	46422	*AFAP1*	*AFAP1-AS1*
cg00955867	7.64E-08	1.10E-05	5.887738	1	1.6E+08	5'UTR	178	*NHLH1*	*NHLH1*
cg09232918	7.65E-08	1.10E-05	5.887683	16	53120404	Body	31502	*CHD9*	*CHD9*
cg23046566	7.65E-08	1.10E-05	5.887591	6	1.7E+08		-76836		*LOC102724511*

Probe	p_adj	q_val	t	Chr	Pos.hg19	locations	dist2TSS	gene.Illumina	gene.nearest
cg12672050	7.65E-08	1.10E-05	5.887455	2	2.43E+08	Body;Body	8136	*ATG4B*	*ATG4B*
cg04842038	7.66E-08	1.10E-05	5.887386	8	1307402	Body	-56575	*DLGAP2*	*LOC286083*
cg23428922	7.66E-08	1.10E-05	5.887347	12	56040601		9926		*OR10P1*
cg20915763	7.67E-08	1.10E-05	5.886814	15	85198577	TSS1500;TSS1500;TSS1500;TSS1500;TSS1500;3'UTR;Body	-1003	*WDR73;NMB*	*WDR73*
cg21646084	7.68E-08	1.10E-05	5.886769	2	16802843	Body	44259	*FAM49A*	*FAM49A*
cg03726791	7.68E-08	1.10E-05	5.886738	2	2.43E+08	Body;Body	27168	*THAP4*	*THAP4*
cg08848643	7.68E-08	1.10E-05	5.886536	16	50745102	Body;Body;Body;Body	11812	*NOD2*	*NOD2*
cg05883713	7.69E-08	1.10E-05	5.886403	14	23963992		-16976		*THTPA*
cg10034588	7.70E-08	1.10E-05	5.886038	2	2.32E+08	Body	3101	*NMUR1*	*NMUR1*
cg01139342	7.70E-08	1.10E-05	5.885972	16	85520338		-124676		*GSE1*
cg04381004	7.71E-08	1.10E-05	5.885849	2	1.66E+08	Body	-49905	*CSRNP3*	*CSRNP3*
cg09683413	7.71E-08	1.10E-05	5.885751	2	16400723		210175		*GACAT3*
cg24277586	7.71E-08	1.10E-05	5.885696	3	52099561	TSS1500	-433	*LINC00696*	*LINC00696*
cg06182031	7.72E-08	1.10E-05	5.885533	3	1.29E+08	3'UTR;3'UTR;TES5000	2972	*H1-8;PLXND1*	*H1-8*
cg12064201	7.72E-08	1.10E-05	5.88552	15	1.02E+08	Body	146445	*LRRK1*	*LRRK1*
cg13446351	7.72E-08	1.10E-05	5.885493	1	26536844		-14966		*ZPLD2P*
cg07725967	7.74E-08	1.10E-05	5.88497	4	7663352	Body	-92464	*SORCS2*	*AFAP1-AS1*
cg03898802	7.75E-08	1.11E-05	5.884653	21	37617652	Body;Body	-74834	*DOP1B*	*MORC3*
cg03152913	7.76E-08	1.11E-05	5.884244	2	1.79E+08	TSS5000;TSS1500;TSS1500;TSS1500	-1451	*PDE11A;RBM45*	*RBM45*
cg21671386	7.77E-08	1.11E-05	5.884079	11	1.13E+08	3'UTR;3'UTR	22070	*DRD2*	*ANKK1*
cg00369677	7.77E-08	1.11E-05	5.884079	2	2.37E+08	Body;Body	66981	*AGAP1*	*GBX2*
cg24555562	7.77E-08	1.11E-05	5.884027	2	2.43E+08	Body	3583	*LINC01237*	*LINC01237*
cg17959824	7.79E-08	1.11E-05	5.883411	11	70391706	Body;Body;Body	-85492	*SHANK2*	*SHANK2-AS1*
cg19503826	7.80E-08	1.11E-05	5.883138	19	56187810	TES5000;TES5000;Body;Body;Body;TSS200	-180	*U2AF2;EPN1*	*EPN1*
cg14416316	7.80E-08	1.11E-05	5.883071	13	1.11E+08	Body	14943	*COL4A2*	*COL4A2-AS1*
cg26475742	7.81E-08	1.11E-05	5.882771	7	1.51E+08	Body;Body	1769	*AOC1*	*AOC1*
cg11801490	7.82E-08	1.11E-05	5.882312	14	63922365	Body;Body;Body;Body;Body;Body;Body	52591	*PPP2R5E*	*PPP2R5E*
cg00113117	7.83E-08	1.11E-05	5.882217	1	56946754		66120		*LINC01767*
cg17489690	7.84E-08	1.11E-05	5.881995	1	3136333	Body;Body	91795	*PRDM16*	*MIR4251*
cg01455069	7.84E-08	1.11E-05	5.881952	11	62382430	TSS5000;TSS5000;TSS5000;3'UTR;TES5000;TES5000;TES5000;TES5000;TES5000	-2183	*EML3;ROM1;B3GAT3*	*EML3*
cg17262868	7.84E-08	1.11E-05	5.881866	17	1127152	Body;TES5000	5864	*ABR*	*ABR*
cg02369450	7.85E-08	1.12E-05	5.88152	5	1950212		62768		*CTD-2194D22.4*
cg14758740	7.85E-08	1.12E-05	5.881425	17	941872	Body;Body;Body;Body;Body;Body	-6801	*ABR*	*ABR*
cg20496526	7.86E-08	1.12E-05	5.881318	14	92335333	TSS1500;TES5000	-1453	*TC2N;FBLN5*	*TC2N*
cg18270610	7.86E-08	1.12E-05	5.881304	11	1.34E+08	TSS1500;TSS1500	-758	*SPATA19*	*SPATA19*
cg05617027	7.87E-08	1.12E-05	5.880939	4	40437086	Body;Body;Body;Body	67050	*RBM47*	*MIR4802*
cg15685971	7.88E-08	1.12E-05	5.880742	8	1.46E+08	TES5000;TES5000;TES5000;TES5000;TES5000;TSS1500	-396	*PPP1R16A;GPT*	*GPT*
cg01234748	7.88E-08	1.12E-05	5.880633	11	2005425	Body	5725	*MRPL23-AS1*	*MRPL23-AS1*
cg16643228	7.91E-08	1.12E-05	5.879918	17	18535909	TES5000	-6979	*TBC1D28*	*CCDC144B*
cg14855378	7.91E-08	1.12E-05	5.879894	5	1.5E+08	TSS1500	-866	*ARSI*	*ARSI*
cg00534540	7.91E-08	1.12E-05	5.879803	7	1.58E+08	Body;Body;Body;Body;Body	-60487	*PTPRN2*	*LOC100506585*
cg04988978	7.91E-08	1.12E-05	5.87976	17	56359578	TSS1500	-1282	*MPO*	*MPO*
cg19442470	7.91E-08	1.12E-05	5.879717	8	27470225	Body;TSS1500;TSS1500;TSS5000	-957	*CLU;MIR6843*	*CLU*
cg21768014	7.91E-08	1.12E-05	5.87968	8	2060983	Body	36315	*MYOM2*	*MIR7160*
cg18741958	7.91E-08	1.12E-05	5.879654	3	1.25E+08	Body;Body	16459	*SLC12A8*	*SLC12A8*
cg27396346	7.91E-08	1.12E-05	5.879639	15	99334874	Body;Body	7220	*IGF1R*	*MIR4714*
cg01066588	7.92E-08	1.12E-05	5.879455	16	84655553	TSS5000	-3869	*COTL1*	*COTL1*
cg06064098	7.92E-08	1.12E-05	5.879381	13	1.14E+08	Body	1795	*ATP4B*	*ATP4B*
cg03181618	7.94E-08	1.12E-05	5.878846	2	1.6E+08	Body;Body;Body;Body;Body;Body;Body;Body;Body;Body	29871	*WDSUB1*	*WDSUB1*
cg15001714	7.95E-08	1.12E-05	5.87862	1	48459813	Body	3244	*TRABD2B*	*TRABD2B*
cg13344757	7.95E-08	1.12E-05	5.878474	17	26904381	TSS1500;TES5000	-468	*ALDOC;SPAG5*	*ALDOC*
cg04027302	7.97E-08	1.12E-05	5.878146	1	2.37E+08	Body;TES5000;TSS1500;TSS1500;TSS5000	-1214	*LGALS8;LGALS8-AS1*	*LGALS8*
cg13965908	7.97E-08	1.12E-05	5.877996	11	70129690	Body;Body;Body;TSS1500	-370	*PPFIA1;MIR548K*	*MIR548K*
cg08379828	7.97E-08	1.12E-05	5.877969	19	4228543	TES5000;TSS1500	-976	*ANKRD24;EBI3*	*EBI3*
cg08657939	8.00E-08	1.13E-05	5.877026	11	69295007		-5321		*LINC01488*
cg15033969	8.01E-08	1.13E-05	5.876868	11	57105814	TSS5000;TSS200	-11	*SSRP1;P2RX3*	*P2RX3*
cg18997701	8.01E-08	1.13E-05	5.87676	14	1.02E+08	Body;Body;Body;Body;Body;Body;Body;Body;Body;Body	-72887	*PPP2R5C*	*DYNC1H1*
cg06127256	8.01E-08	1.13E-05	5.876752	16	73206588		-28242		*ZFHX3*
cg24190139	8.02E-08	1.13E-05	5.876705	3	48951829	TES5000;TSS5000	-4445	*ARIH2OS;ARIH2*	*ARIH2*

Probe	p_adj	q_val	t	Chr	Pos.hg19	locations	dist2TSS	gene.Illumina	gene.nearest
cg13071306	8.02E-08	1.13E-05	5.876642	1	6314113	Body	7045	*GPR153*	*GPR153*
cg26793019	8.02E-08	1.13E-05	5.876517	8	2795929	3'UTR	-209938	*CSMD1*	*LOC101927815*
cg02855045	8.02E-08	1.13E-05	5.876453	3	72777245		-63845		*LOC105377162*
cg04515311	8.02E-08	1.13E-05	5.876437	16	4822815	TES5000;TES5000	-5652	*SEPTIN12*	*ZNF500*
cg27270968	8.03E-08	1.13E-05	5.876301	16	48846874		-202783		*N4BP1*
cg26199552	8.03E-08	1.13E-05	5.876149	11	70368372	Body;Body;Body	-108826	*SHANK2*	*SHANK2-AS1*
cg04957757	8.04E-08	1.13E-05	5.876041	16	70463218	Body	9785	*ST3GAL2*	*ST3GAL2*
cg12277870	8.05E-08	1.13E-05	5.875632	1	2.08E+08		-53156		*CD34*
cg03599257	8.05E-08	1.13E-05	5.875604	1	28283712	TES5000;Body;Body;TSS5000	-2771	*SMPDL3B;XKR8*	*XKR8*
cg17716358	8.06E-08	1.13E-05	5.875474	10	82268218	Body;Body;Body;Body;Body;Body;Body;Body;Body	27480	*TSPAN14*	*LOC101929574*
cg00163510	8.06E-08	1.13E-05	5.875331	1	2.15E+08	Body	1993	*PTPN14*	*PTPN14*
cg06518327	8.06E-08	1.13E-05	5.875306	7	1.57E+08	Body	2239	*LOC101927914*	*LOC101927914*
cg11813064	8.07E-08	1.13E-05	5.874979	20	62003032	TES5000	-10284	*LOC100130587*	*CHRNA4*
cg26282205	8.08E-08	1.13E-05	5.874949	10	75602367	Body;Body;Body;Body;Body;Body;Body;Body;Body;Body;Body;Body;Body;Body;Body;Body;Body;Body;Body;Body;Body;Body;Body;Body;Body;Body;Body;Body;Body;Body;Body;Body;Body;Body;Body;Body;Body;Body;Body;Body;Body;Body;Body;Body;Body;Body;Body;Body;Body;Body;Body;Body;Body;Body;Body;Body	-30789	*CAMK2G*	*NDST2*
cg01374918	8.08E-08	1.13E-05	5.874791	4	7754366	TSS1500	-1450	*AFAP1-AS1*	*AFAP1-AS1*
cg25571414	8.10E-08	1.13E-05	5.874296	4	25659572	Body;Body;Body	1487	*SLC34A2*	*SLC34A2*
cg16772023	8.11E-08	1.14E-05	5.873976	10	1308866	Body	103159	*ADARB2*	*LINC00200*
cg14312860	8.11E-08	1.14E-05	5.873965	17	79105559	Body;Body;TES5000	189	*AATK;MIR1250*	*AATK*
cg07037963	8.12E-08	1.14E-05	5.873551	6	43265846	TSS200;TSS200	-151	*SLC22A7*	*SLC22A7*
cg11834106	8.13E-08	1.14E-05	5.873511	6	31747010	TSS5000;Body	-1905	*VWA7;VARS1*	*VWA7*
cg21052104	8.13E-08	1.14E-05	5.873403	5	1.79E+08	Body;Body	59826	*ADAMTS2*	*ADAMTS2*
cg14205362	8.14E-08	1.14E-05	5.873116	19	2389655	TSS200	-113	*TMPRSS9*	*TMPRSS9*
cg12476443	8.14E-08	1.14E-05	5.872968	2	26624228	TSS1500	-559	*DRC1*	*DRC1*
cg17978274	8.15E-08	1.14E-05	5.872926	15	93013676	TES5000;TES5000;Body;TSS1500;TSS1500	1100	*ST8SIA2;LOC104613533;C15orf32*	*LOC104613533*
cg21958642	8.17E-08	1.14E-05	5.872169	7	22592804		-10151		*LOC100506178*
cg10758022	8.18E-08	1.14E-05	5.871928	7	1.31E+08	TSS200;TES5000;TES5000;TES5000;TES5000	-23	*MIR29A;MIR29B1;LINC-PINT*	*MIR29A*
cg16616937	8.18E-08	1.14E-05	5.871898	12	57866267	TES5000;TES5000;TES5000;3'UTR;3'UTR;3'UTR;3'UTR;3'UTR;3'UTR;3'UTR;3'UTR;3'UTR;3'UTR	5383	*GLI1;ARHGAP9*	*ARHGAP9*
cg07947531	8.19E-08	1.14E-05	5.871794	1	32252266	TES5000;TES5000;TES5000	-22325	*SPOCD1*	*ADGRB2*
cg11330282	8.19E-08	1.14E-05	5.871589	5	1.49E+08	Body;Body	7872	*CARMN*	*CARMN*
cg03649855	8.20E-08	1.14E-05	5.871521	6	45576388		186377		*RUNX2*
cg18601578	8.20E-08	1.14E-05	5.871466	19	58180234	TSS200	-68	*ZSCAN4*	*ZSCAN4*
cg16474118	8.20E-08	1.14E-05	5.871419	2	12760191		-96806		*TRIB2*
cg16142349	8.21E-08	1.14E-05	5.871236	6	1.67E+08	Body;Body;Body;Body;Body	11912	*RPS6KA2*	*MIR1913*
cg24188437	8.21E-08	1.14E-05	5.871073	5	1.8E+08	TSS200	-74	*OR2Y1*	*OR2Y1*
cg04579608	8.21E-08	1.14E-05	5.871061	7	1.29E+08	TSS200;TES5000;TES5000	-103	*MIR182;MIR96;MIR183*	*MIR182*
cg04508216	8.22E-08	1.15E-05	5.870696	1	11107452	TSS200;TSS200	-156	*MASP2*	*MASP2*
cg00562312	8.23E-08	1.15E-05	5.870504	5	1.79E+08	Body;Body	19463	*ADAMTS2*	*ADAMTS2*
cg20370783	8.25E-08	1.15E-05	5.869903	2	1.01E+08	Body	12219	*NPAS2*	*NPAS2*
cg21385480	8.26E-08	1.15E-05	5.869715	3	66454763	Body	96082	*LRIG1*	*LRIG1*
cg16996571	8.26E-08	1.15E-05	5.869682	14	91691129	Body;Body;Body;Body;Body;Body;Body;Body;Body;Body;Body	19919	*DGLUCY*	*GPR68*
cg26573807	8.27E-08	1.15E-05	5.869527	1	19654245	Body;Body;Body;Body;Body	15426	*SLC66A1*	*SLC66A1*
cg10623290	8.27E-08	1.15E-05	5.869462	1	1912185	Body	23040	*CFAP74*	*CFAP74*
cg13097090	8.28E-08	1.15E-05	5.869196	14	74815158	TSS200	-15	*VRTN*	*VRTN*
cg23375617	8.28E-08	1.15E-05	5.869096	10	54299632		-69339		*LNCAROD*
cg08448074	8.29E-08	1.15E-05	5.868961	17	80268958	TES5000	6526	*CD7*	*CD7*
cg03221619	8.29E-08	1.15E-05	5.868813	19	7767348	TSS5000;TSS1500;TSS1500	-316	*FCER2*	*FCER2*
cg13748092	8.30E-08	1.15E-05	5.868626	15	58024200		25289		*POLR2M*
cg25099516	8.30E-08	1.15E-05	5.868565	1	48288813	Body	174244	*TRABD2B*	*TRABD2B*
cg20418191	8.31E-08	1.15E-05	5.868329	12	50185413	3'UTR;3'UTR	36783	*NCKAP5L*	*NCKAP5L*
cg07400453	8.32E-08	1.15E-05	5.867976	11	1.26E+08	Body;Body	11269	*FAM118B*	*SRPRA*
cg02797569	8.33E-08	1.15E-05	5.867792	7	1E+08	TES5000;Body;TES5000;TES5000;5'UTR	24	*FBXO24;PCOLCE-AS1;PCOLCE*	*PCOLCE*
cg00360077	8.33E-08	1.15E-05	5.867769	15	80889593	3'UTR	16150	*ARNT2*	*MIR5572*
cg00993677	8.34E-08	1.15E-05	5.867495	2	2.4E+08		59360		*MIR4440*
cg08033127	8.34E-08	1.15E-05	5.867408	1	55232442	TSS5000	-2246	*PARS2*	*PARS2*
cg03963250	8.34E-08	1.15E-05	5.867364	10	44198369		-54040		*ZNF32*
cg17826786	8.34E-08	1.15E-05	5.867357	2	3322489	Body;Body;Body	59100	*EIPR1*	*EIPR1*
cg13461190	8.37E-08	1.16E-05	5.8665	9	1.33E+08	Body	9310	*NCS1*	*NCS1*
cg19536075	8.38E-08	1.16E-05	5.866437	8	1.43E+08	Body;Body;Body;Body;Body;Body;Body	9892	*TSNARE1*	*TSNARE1*
cg26576047	8.38E-08	1.16E-05	5.866366	7	1.32E+08	Body	-71141	*PLXNA4*	*LOC101928807*

Probe	p_adj	q_val	t	Chr	Pos.hg19	locations	dist2TSS	gene.Illumina	gene.nearest
cg10333415	8.38E-08	1.16E-05	5.866294	3	1.95E+08	Body;Body	34147	*XXYLT1*	*XXYLT1*
cg24522478	8.39E-08	1.16E-05	5.866008	11	2177498	Body;Body;TES5000;TES5000;TES5000;TES5000	4941	*INS-IGF2;INS*	*INS-IGF2*
cg16362014	8.39E-08	1.16E-05	5.865967	5	1.18E+08	Body;Body	2359	*DTWD2*	*DTWD2*
cg11582558	8.39E-08	1.16E-05	5.865958	8	1.02E+08	TSS1500;TSS1500;TSS1500;TSS1500	-782	*ANKRD46*	*ANKRD46*
cg26535547	8.40E-08	1.16E-05	5.865877	17	42151680	Body;Body;Body;Body;TES5000;TES5000	3402	*G6PC3;HDAC5*	*G6PC3*
cg14358492	8.40E-08	1.16E-05	5.865811	9	91454488		-93668		*MIR4289*
cg08970020	8.40E-08	1.16E-05	5.865772	2	64216731	Body;Body	29831	*VPS54*	*VPS54*
cg06581825	8.40E-08	1.16E-05	5.865704	2	1.63E+08	Body;Body;Body;Body;Body;Body;Body;Body;Body;Body;Body;Body;Body;Body;Body;Body;Body;Body;Body	92361	*SLC4A10*	*DPP4*
cg14230719	8.41E-08	1.16E-05	5.865482	9	1.23E+08		-15541		*MEGF9*
cg02299946	8.42E-08	1.16E-05	5.865289	10	1.24E+08	Body	3402	*HTRA1*	*HTRA1*
cg07464250	8.42E-08	1.16E-05	5.865235	5	1.19E+08	TES5000;TES5000;TES5000;TES5000;TES5000	46668	*TNFAIP8*	*TNFAIP8*
cg23293467	8.42E-08	1.16E-05	5.865165	8	6762877		20721		*DEFA6*
cg23833425	8.43E-08	1.16E-05	5.865049	6	1.42E+08	Body;Body;Body	8241	*VTA1*	*VTA1*
cg08955099	8.43E-08	1.16E-05	5.865049	2	2.13E+08	Body;Body	395111	*ERBB4*	*MIR548F2*
cg00910616	8.43E-08	1.16E-05	5.865009	1	41245733	TSS5000;TSS5000	-3725	*KCNQ4*	*KCNQ4*
cg11229160	8.44E-08	1.16E-05	5.864729	6	5510800	Body;Body	-52492	*FARS2*	*LOC101927972*
cg27543823	8.44E-08	1.16E-05	5.864675	16	63650892		1505074		*CDH11*
cg25384949	8.45E-08	1.16E-05	5.864254	6	32190320	Body;Body;Body	1524	*NOTCH4*	*NOTCH4*
cg02249030	8.46E-08	1.16E-05	5.864219	6	99961954	5'UTR;5'UTR;Body;Body;Body;Body;Body;Body;Body;Body;Body;Body;Body;Body;Body;Body;Body;Body	1298	*USP45*	*USP45*
cg22550943	8.46E-08	1.16E-05	5.864157	7	2122577	Body;Body;Body;Body;Body	6110	*MAD1L1*	*SNORA114*
cg21033494	8.47E-08	1.16E-05	5.863886	10	51550114	Body;Body	562	*MSMB*	*MSMB*
cg21784143	8.47E-08	1.16E-05	5.863882	17	20460588		-22448		*CDRT15L2*
cg00917181	8.47E-08	1.16E-05	5.863821	17	76925901	TSS5000	-4432	*TIMP2*	*TIMP2*
cg23271998	8.48E-08	1.16E-05	5.86366	2	3260576	Body;Body;Body	121013	*EIPR1*	*EIPR1*
cg19802542	8.48E-08	1.16E-05	5.863561	3	46786238	Body	7	*PRSS45P*	*PRSS45P*
cg20256567	8.48E-08	1.16E-05	5.863522	10	1.27E+08	Body;Body;Body;Body;Body;Body;Body	31705	*CTBP2*	*CTBP2*
cg14962616	8.49E-08	1.16E-05	5.863396	19	39437613	Body;Body;Body	5714	*FBXO17*	*FBXO17*
cg23639734	8.49E-08	1.16E-05	5.863273	4	24796753	TSS1500	-441	*SOD3*	*SOD3*
cg13401014	8.50E-08	1.16E-05	5.863067	11	1.29E+08	TSS5000;Body;TES5000;TSS5000;TSS5000;TSS5000	-2427	*LOC101929538;FLI1;SENCR*	*LOC101929538*
cg03709271	8.50E-08	1.16E-05	5.862996	10	1.35E+08		40625		*LINC01168*
cg22188571	8.51E-08	1.17E-05	5.862608	6	34074955	TSS1500;Body;Body;Body;Body;Body	-1211	*GRM4*	*GRM4*
cg08104780	8.52E-08	1.17E-05	5.862562	16	2848117	TSS5000;TSS1500	-353	*SNORA3C;PRSS41*	*PRSS41*
cg27492725	8.52E-08	1.17E-05	5.862517	2	1.83E+08		-21899		*PDE1A*
cg00069014	8.53E-08	1.17E-05	5.862256	1	14156840		80931		*PRDM2*
cg05824594	8.54E-08	1.17E-05	5.86177	12	2734503	Body;Body;Body;Body;Body;Body;Body;Body;Body;Body;Body;Body;Body;Body;Body;Body;Body;Body;Body;Body;Body;Body;Body	46883	*CACNA1C*	*CACNA1C-AS2*
cg06909503	8.56E-08	1.17E-05	5.8614	1	1.65E+08	Body;Body;Body;Body;Body	18651	*PBX1*	*PBX1-AS1*
cg25140751	8.56E-08	1.17E-05	5.861335	15	78419875	Body;Body;Body;Body;Body	3912	*CIB2*	*CIB2*
cg07198170	8.57E-08	1.17E-05	5.861147	7	1581217	3'UTR;TES5000	-6701	*MAFK;TMEM184A*	*LOC100128653*
cg20039983	8.57E-08	1.17E-05	5.861101	14	23516460	TES5000;3'UTR;3'UTR	5040	*PSMB11;CDH24*	*PSMB11*
cg26500457	8.57E-08	1.17E-05	5.860978	11	1.23E+08	Body;5'UTR	42	*CRTAM*	*CRTAM*
cg18220380	8.57E-08	1.17E-05	5.86097	8	1.42E+08	Body;Body;Body;Body	46760	*DENND3*	*DENND3*
cg09155692	8.58E-08	1.17E-05	5.860815	4	1.85E+08	Body	4078	*STOX2*	*STOX2*
cg03680528	8.58E-08	1.17E-05	5.860811	18	72999716	1stExon;Body	76233	*TSHZ1*	*TSHZ1*
cg13034825	8.58E-08	1.17E-05	5.860679	3	1.5E+08		-16632		*SIAH2*
cg22659852	8.59E-08	1.17E-05	5.860559	6	1.52E+08	Body;Body;Body;Body	24602	*SYNE1*	*SYNE1*
cg20934731	8.59E-08	1.17E-05	5.860548	8	4439787	Body	412649	*CSMD1*	*CSMD1*
cg11387897	8.60E-08	1.17E-05	5.860337	1	1.84E+08	Body;Body;Body	92674	*COLGALT2*	*COLGALT2*
cg22489883	8.60E-08	1.17E-05	5.860203	1	2537547	Body	19332	*MMEL1*	*PRXL2B*
cg00401797	8.61E-08	1.17E-05	5.859925	1	3315193	Body;Body	-56035	*PRDM16*	*ARHGEF16*
cg04944931	8.63E-08	1.18E-05	5.85941	10	76996462	TSS1500	-747	*COMTD1*	*COMTD1*
cg13063207	8.63E-08	1.18E-05	5.859348	8	1442292	Body	127538	*DLGAP2*	*DLGAP2-AS1*
cg21440339	8.64E-08	1.18E-05	5.859213	2	2.01E+08	TSS5000;TSS5000;TSS5000;TSS5000	-3895	*KCTD18*	*KCTD18*
cg03405834	8.64E-08	1.18E-05	5.859191	16	677895	3'UTR;3'UTR;3'UTR;3'UTR;3'UTR;TSS5000	-3089	*RAB40C;WFIKKN1*	*WFIKKN1*
cg03760226	8.65E-08	1.18E-05	5.859012	1	1390794	Body	5726	*ATAD3C*	*ATAD3C*
cg13429270	8.65E-08	1.18E-05	5.858855	18	10727599	Body	32048	*PIEZO2*	*MIR6788*
cg00355377	8.67E-08	1.18E-05	5.858485	10	50336460	Body;TES5000	5605	*FAM170B-AS1;FAM170B*	*FAM170B*
cg14898646	8.67E-08	1.18E-05	5.858385	7	820298	Body;Body	-34895	*DNAAF5*	*SUN1*
cg12407978	8.67E-08	1.18E-05	5.858385	11	10590159	Body;Body;Body;Body;5'UTR;TES5000;TES5000;TES5000;TES5000;TES5000;TES5000	53	*MRVI1-AS1;LYVE1;MRVI1*	*LYVE1*
cg02049938	8.68E-08	1.18E-05	5.85809	8	21994573	TES5000;TES5000;TES5000	4847	*REEP4*	*REEP4*

Probe	p_adj	q_val	t	Chr	Pos.hg19	locations	dist2TSS	gene.Illumina	gene.nearest
cg23239637	8.68E-08	1.18E-05	5.85804	2	1507832	Body;Body;Body;Body;Body;Body	89653	*TPO*	*TPO*
cg14487577	8.69E-08	1.18E-05	5.857951	6	52012390	TSS5000;Body;TSS1500	-1330	*MIR206;LINCMD1;MIR133B*	*MIR133B*
cg22463035	8.69E-08	1.18E-05	5.857785	12	1.31E+08	Body;Body;Body;Body;Body	12839	*RIMBP2*	*RIMBP2*
cg05141924	8.70E-08	1.18E-05	5.857589	2	28669866		-49060		*PLB1*
cg26639479	8.70E-08	1.18E-05	5.857496	16	30363681	3'UTR;3'UTR;TES5000	2574	*CD2BP2;TBC1D10B*	*CD2BP2*
cg21640603	8.71E-08	1.18E-05	5.857425	19	11310131	TSS5000;TSS5000;TSS5000;3'UTR;3'UTR;TSS5000	-1910	*KANK2;DOCK6;LOC105372273*	*KANK2*
cg01092528	8.71E-08	1.18E-05	5.857303	4	76861103	Body;Body;Body	1063	*NAAA*	*NAAA*
cg25302704	8.71E-08	1.18E-05	5.857293	8	27470629	Body;TSS5000;TSS1500;TSS5000	-1361	*CLU;MIR6843*	*CLU*
cg02056325	8.71E-08	1.18E-05	5.857201	11	11087328		207565		*ZBED5-AS1*
cg15365949	8.72E-08	1.18E-05	5.857089	3	15141111	TSS1500;TSS1500;TSS1500	-447	*RBSN*	*RBSN*
cg24586376	8.73E-08	1.18E-05	5.856779	7	955551	Body;Body;Body;Body;Body	4975	*ADAP1*	*ADAP1*
cg17016922	8.74E-08	1.18E-05	5.856483	5	1.41E+08		50402		*PCDH1*
cg00426425	8.75E-08	1.18E-05	5.856201	16	87677927	Body;Body	41814	*JPH3*	*JPH3*
cg14233878	8.76E-08	1.18E-05	5.856049	9	1.4E+08	Body	-6752	*HSPC324*	*EGFL7*
cg03919942	8.76E-08	1.18E-05	5.855861	3	1.23E+08	TES5000;TES5000;Body;Body;Body;Body;Body	-13469	*MYLK-AS1;MYLK*	*MYLK*
cg02722625	8.77E-08	1.18E-05	5.855777	3	52726624	Body;Body;Body;TES5000;TES5000;TES5000;TES5000;TSS200;TES5000;TES5000;TES5000;TES5000;TES5000	-127	*GNL3;SNORD136;SNORD19;SNORD19B;SNORD19C;SNORD69;GLT8D1*	*SNORD69*
cg11138192	8.77E-08	1.18E-05	5.855747	22	39965842	TSS1500;TSS1500	-915	*CACNA1I*	*CACNA1I*
cg23636072	8.78E-08	1.18E-05	5.855505	11	1216443	Body	-27852	*MUC5AC*	*MUC5B*
cg07297322	8.78E-08	1.18E-05	5.855414	16	74661825	Body;Body;Body;Body;Body;Body;Body;Body;Body	-20813	*RFWD3*	*GLG1*
cg03652564	8.78E-08	1.18E-05	5.855347	1	1.65E+08	Body;Body;Body;Body;Body	90807	*PBX1*	*PBX1*
cg06569904	8.78E-08	1.18E-05	5.85534	3	42452635	TSS1500;TSS1500	-533	*LYZL4*	*LYZL4*
cg24071464	8.79E-08	1.18E-05	5.855239	2	97195532	Body	2511	*LOC105373496*	*LOC105373496*
cg04756168	8.79E-08	1.18E-05	5.855226	11	1.17E+08	TSS1500	-1033	*APOC3*	*APOC3*
cg19754709	8.79E-08	1.18E-05	5.855119	16	2334346	Body	9726	*ABCA3*	*MIR4717*
cg21383284	8.79E-08	1.18E-05	5.855106	2	20826833	Body	24025	*HS1BP3*	*HS1BP3*
cg14321058	8.80E-08	1.18E-05	5.854996	11	1436177	Body;Body;Body;Body;Body;Body	3796	*BRSK2*	*BRSK2*
cg10767312	8.80E-08	1.18E-05	5.854956	13	78236645	Body	293	*LOC100129307*	*LOC100129307*
cg19076895	8.80E-08	1.18E-05	5.854915	6	7633299		42869		*SNRNP48*
cg11753771	8.81E-08	1.18E-05	5.854665	1	2.15E+08	1stExon	86875	*PTPN14*	*PTPN14*
cg22506757	8.81E-08	1.18E-05	5.854661	1	5919703	TES5000;TES5000;TES5000;TES5000;TES5000	3098	*MIR4689;NPHP4*	*MIR4689*
cg14873515	8.81E-08	1.18E-05	5.854648	5	1.5E+08	Body;TSS1500	-231	*NDST1*	*NDST1*
cg09477124	8.81E-08	1.18E-05	5.854647	3	10379281	Body;Body;Body;Body;Body	7369	*ATP2B2*	*MIR378B*
cg25031824	8.81E-08	1.18E-05	5.854514	17	3819363	Body	396	*P2RX1*	*P2RX1*
cg11414267	8.84E-08	1.19E-05	5.853935	10	99694717	Body;Body	-59063	*CRTAC1*	*MIR3085*
cg03038672	8.84E-08	1.19E-05	5.85386	16	3062056	TSS1500;TES5000	-867	*CLDN9;CLDN6*	*CLDN9*
cg27011898	8.84E-08	1.19E-05	5.853729	2	1.33E+08	TSS5000	-4819	*FAM201B*	*FAM201B*
cg26004664	8.85E-08	1.19E-05	5.85356	4	1.84E+08		44028		*DCTD*
cg06121207	8.85E-08	1.19E-05	5.853462	16	3494155	TSS1500;Body;Body	476	*ZNF597;NAA60*	*NAA60*
cg02370023	8.86E-08	1.19E-05	5.853219	17	80319923	5'UTR	2801	*TEX19*	*TEX19*
cg03590257	8.88E-08	1.19E-05	5.852915	4	17805193	Body;Body;Body;Body;Body;Body;Body;Body;Body;Body	7187	*DCAF16*	*DCAF16*
cg19707326	8.89E-08	1.19E-05	5.852449	14	24787611	TES5000;TES5000;3'UTR;3'UTR;3'UTR	5299	*LTB4R;ADCY4*	*LTB4R*
cg21323028	8.89E-08	1.19E-05	5.852427	2	1.71E+08	Body	102668	*UBR3*	*UBR3*
cg06850158	8.90E-08	1.19E-05	5.852356	4	74567419		-38866		*CXCL8*
cg13487655	8.90E-08	1.19E-05	5.852265	14	70497701	3'UTR;3'UTR	49233	*SMOC1*	*SLC8A3*
cg04506911	8.90E-08	1.19E-05	5.852216	10	72937119		-35207		*UNC5B*
cg19910568	8.91E-08	1.19E-05	5.852019	10	22048295	Body	225135	*DNAJC1*	*MLLT10*
cg19523255	8.91E-08	1.19E-05	5.851992	4	82439860		-46799		*RASGEF1B*
cg12078872	8.91E-08	1.19E-05	5.851922	6	1.11E+08	Body;Body;Body;Body;Body;Body;Body;Body;Body	15149	*DDO*	*DDO*
cg17221993	8.92E-08	1.19E-05	5.851797	10	1234983	Body	29276	*ADARB2*	*LINC00200*
cg20919143	8.92E-08	1.19E-05	5.851789	14	1.06E+08	Body;Body;1stExon;1stExon;Body	-23316	*BRF1*	*BRF1*
cg02335370	8.92E-08	1.19E-05	5.851787	8	33579138		-121514		*DUSP26*
cg22127299	8.92E-08	1.19E-05	5.851608	6	27988718		-52375		*OR1F12*
cg16106080	8.93E-08	1.19E-05	5.85157	7	1.4E+08	3'UTR	22160	*RAB19*	*RAB19*
cg21847267	8.93E-08	1.19E-05	5.851413	7	1.43E+08	Body;Body	16083	*CLCN1*	*FAM131B*
cg08571475	8.93E-08	1.19E-05	5.851411	6	11494909		-43072		*TMEM170B*
cg08549026	8.93E-08	1.19E-05	5.851375	1	1.68E+08	Body;Body;Body	-34761	*RCSD1*	*MPZL1*
cg02014020	8.93E-08	1.19E-05	5.851363	1	1115461	Body;Body;1stExon	401	*TTLL10*	*TTLL10*
cg19849323	8.93E-08	1.19E-05	5.851347	3	72286803		86396		*LINC00870*
cg00417963	8.94E-08	1.19E-05	5.851135	7	1529251	Body	14774	*INTS1*	*INTS1*

Probe	p_adj	q_val	t	Chr	Pos.hg19	locations	dist2TSS	gene.Illumina	gene.nearest
cg21339555	8.96E-08	1.19E-05	5.850807	15	62536691	Body	4303	*GOLGA2P11*	*GOLGA2P11*
cg13405222	8.98E-08	1.20E-05	5.85023	6	20811065	Body	276378	*CDKAL1*	*CDKAL1*
cg04423464	8.98E-08	1.20E-05	5.850102	19	18779588	TES5000;Body;Body;Body;Body;1stExon	-14901	*KLHL26*	*CRTC1*
cg18794473	8.99E-08	1.20E-05	5.849866	8	77617694	1stExon	-22184	*ZFHX4*	*ZFHX4-AS1*
cg00049475	9.00E-08	1.20E-05	5.849636	10	29923736	5'UTR;Body;Body;Body	164	*SVIL*	*SVIL*
cg22782105	9.00E-08	1.20E-05	5.849604	10	63731624	Body	70167	*ARID5B*	*ARID5B*
cg09941453	9.01E-08	1.20E-05	5.849382	8	99812867	Body;Body;Body	25032	*STK3*	*STK3*
cg18423737	9.01E-08	1.20E-05	5.849334	18	46500072		-22991		*SMAD7*
cg06648812	9.01E-08	1.20E-05	5.849263	4	619162	TSS200;TSS200	-200	*PDE6B*	*PDE6B*
cg00160002	9.02E-08	1.20E-05	5.849125	2	2.37E+08	Body;Body	44450	*AGAP1*	*GBX2*
cg08708790	9.04E-08	1.20E-05	5.848614	16	22176873	5'UTR	3022	*SDR42E2*	*SDR42E2*
cg06448332	9.05E-08	1.20E-05	5.848464	16	1493343	Body;TES5000;TES5000	1147	*CCDC154;CLCN7*	*CCDC154*
cg11838639	9.05E-08	1.20E-05	5.848463	10	3512917	Body;Body	32113	*LOC105376360;LINC02669*	*LINC02669*
cg03433404	9.05E-08	1.20E-05	5.848393	16	57794955	TES5000;Body;Body;Body;Body;Body;Body;Body;Body;Body;Body	11309	*KATNB1;KIFC3*	*MIR6772*
cg12958836	9.05E-08	1.20E-05	-5.8483	12	8185024	TSS1500	-287	*FOXJ2*	*FOXJ2*
cg17049320	9.05E-08	1.20E-05	5.848287	8	1.08E+08	TSS1500	-262	*ABRA*	*ABRA*
cg21339084	9.06E-08	1.20E-05	5.848135	2	1.28E+08	Body;Body;TSS1500;Body	-266	*LIMS2*	*LIMS2*
cg11113753	9.09E-08	1.21E-05	5.84729	2	44065383	Body;TSS1500;TSS1500	595	*ABCG5;ABCG8*	*ABCG5*
cg09096159	9.09E-08	1.21E-05	5.847255	22	46509557	Body;Body;TES5000;TES5000;TSS200	-8	*MIRLET7BHG;MIRLET7A3;MIR4763;MIRLET7B*	*MIRLET7B*
cg14945317	9.11E-08	1.21E-05	5.846767	6	32363933	Body;TES5000;Body	5647	*TSBP1-AS1;HCG23;BTNL2*	*HCG23*
cg14251805	9.12E-08	1.21E-05	5.846638	9	1.29E+08	Body	-49619	*MVB12B*	*NRON*
cg07792815	9.12E-08	1.21E-05	5.846606	16	19474834	Body;Body;Body;Body;Body	7052	*TMC5*	*TMC5*
cg07132038	9.12E-08	1.21E-05	5.846508	7	1.52E+08	TSS1500;Body	-1477	*PRKAG2;PRKAG2-AS1*	*PRKAG2*
cg16822777	9.13E-08	1.21E-05	5.846414	7	98649896	Body;Body;Body	39109	*SMURF1*	*LOC101927550*
cg27295571	9.13E-08	1.21E-05	5.846321	20	30008142	TES5000	-7501	*DEFB122*	*DEFB121*
cg15665090	9.13E-08	1.21E-05	5.846285	6	31778371	TSS5000;Body;TSS5000	-3628	*LSM2;HSPA1L;HSPA1A*	*LSM2*
cg12165943	9.16E-08	1.21E-05	5.845623	15	93130167		-14674		*LINC00930*
cg06230513	9.16E-08	1.21E-05	5.845605	13	76069242		-12937		*TBC1D4*
cg05888433	9.16E-08	1.21E-05	5.845495	14	20711344	1stExon	394	*OR11H4*	*OR11H4*
cg12144870	9.18E-08	1.21E-05	5.845057	18	29525892	TSS5000	-2801	*TRAPPC8*	*TRAPPC8*
cg19026125	9.18E-08	1.21E-05	5.845056	10	461847	Body	-212656	*DIP2C*	*LOC101930421*
cg14258951	9.19E-08	1.21E-05	5.844869	2	2E+08	Body;Body;Body	185582	*SATB2*	*SATB2*
cg17423813	9.19E-08	1.21E-05	5.844799	10	1.21E+08	Body	26717	*GRK5*	*GRK5*
cg17293838	9.20E-08	1.22E-05	5.844448	2	1797700	Body;Body;Body;Body;Body;Body;Body;Body;Body;Body	-49413	*MYT1L*	*PXDN*
cg00425177	9.21E-08	1.22E-05	5.844302	10	3058239		50886		*LOC101927824*
cg22104371	9.22E-08	1.22E-05	5.844059	7	1787371	3'UTR	-5425	*ELFN1*	*ELFN1-AS1*
cg19772651	9.22E-08	1.22E-05	5.844031	12	57351191	1stExon;5'UTR	1967	*RDH16*	*RDH16*
cg12736037	9.22E-08	1.22E-05	5.844004	6	1.64E+08		-202274		*LOC102724152*
cg06342870	9.23E-08	1.22E-05	5.843866	11	17455488	Body;Body;Body;Body;Body;Body	42904	*ABCC8*	*ABCC8*
cg23282025	9.24E-08	1.22E-05	5.843549	2	1.7E+08	TSS5000	-2435	*PPIG*	*PPIG*
cg23423230	9.24E-08	1.22E-05	5.843454	21	43327978	Body;Body	18834	*C2CD2*	*C2CD2*
cg11856561	9.25E-08	1.22E-05	5.843306	6	1.35E+08	Body	7101	*SGK1*	*SGK1*
cg24945092	9.26E-08	1.22E-05	5.84303	17	11872861	3'UTR;3'UTR	27828	*DNAH9*	*ZNF18*
cg17608529	9.26E-08	1.22E-05	5.843029	10	436626	Body	210693	*DIP2C*	*ZMYND11*
cg17788227	9.27E-08	1.22E-05	5.842686	17	7347145	Body;3'UTR;3'UTR;TSS1500	-1234	*FGF11;CHRNB1*	*CHRNB1*
cg02495250	9.28E-08	1.22E-05	5.842447	16	82735595	Body;Body;Body;Body;Body;Body	13060	*CDH13*	*MIR8058*
cg08373477	9.29E-08	1.22E-05	5.842195	4	1957896	Body;Body;Body;Body	-18466	*NSD2*	*SCARNA22*
cg09762115	9.30E-08	1.22E-05	5.842094	2	86095308	TSS1500;Body;Body;Body;Body;Body;Body;Body;Body;Body;Body	-515	*ST3GAL5*	*ST3GAL5*
cg08619669	9.30E-08	1.22E-05	5.842084	19	49493463	Body;Body;Body;TSS5000;TSS5000;TSS5000;TSS5000	3103	*GYS1;RUVBL2*	*GYS1*
cg02579668	9.30E-08	1.22E-05	5.84192	1	28928325	TES5000;TES5000	9319	*TAF12*	*RAB42*
cg02343803	9.30E-08	1.22E-05	5.841861	19	58300127		19084		*ZNF586*
cg17460183	9.31E-08	1.22E-05	5.841783	10	48370158	Body;Body;Body;Body;Body;Body	15090	*ZNF488*	*ZNF488*
cg00405043	9.33E-08	1.22E-05	5.841326	16	963814	Body;Body;Body;Body;Body;Body;Body	-7218	*LMF1*	*LMF1-AS1*
cg26794871	9.33E-08	1.23E-05	5.841173	20	44934712	Body	2425	*CDH22*	*CDH22*
cg25352924	9.34E-08	1.23E-05	5.841028	7	2083583	Body;Body;Body;Body;Body	45104	*MAD1L1*	*SNORA114*
cg02717506	9.35E-08	1.23E-05	5.840718	3	56390366	Body;Body	112129	*ERC2*	*ERC2*
cg10459202	9.36E-08	1.23E-05	5.840567	4	3377595	Body;Body;Body	5671	*RGS12*	*RGS12*
cg01541650	9.37E-08	1.23E-05	5.840209	6	1.58E+08	Body;Body;Body;Body;Body	148408	*ARID1B*	*ARID1B*
cg08382035	9.37E-08	1.23E-05	5.840191	5	1.39E+08	Body;Body;Body;Body;Body	56551	*NRG2*	*PSD2*

Probe	p_adj	q_val	t	Chr	Pos.hg19	locations	dist2TSS	gene.Illumina	gene.nearest
cg03726437	9.38E-08	1.23E-05	5.840091	17	3474461	Body;Body;Body;Body	-13355	*TRPV1*	*TRPV3*
cg12386808	9.38E-08	1.23E-05	5.84008	3	44762171	Body;Body;Body;Body;Body	8020	*ZNF502*	*ZNF502*
cg21946195	9.38E-08	1.23E-05	5.839947	2	86012225	Body;TSS1500	-1425	*ATOH8;MIR6071*	*MIR6071*
cg05862007	9.39E-08	1.23E-05	5.839684	2	48950153	Body;Body	32710	*STON1-GTF2A1L;LHCGR*	*LHCGR*
cg00829041	9.40E-08	1.23E-05	5.839537	2	2.19E+08	Body	1572	*DIRC3*	*DIRC3*
cg16748816	9.40E-08	1.23E-05	5.839445	11	1.24E+08	TSS1500	-1366	*OR10G9*	*OR10G9*
cg00217225	9.40E-08	1.23E-05	5.839405	4	1.53E+08		-45161		*FBXW7-AS1*
cg01694527	9.41E-08	1.23E-05	5.839317	8	1.46E+08	Body	15701	*ARHGAP39*	*ARHGAP39*
cg26946836	9.41E-08	1.23E-05	5.8393	2	71064086	TSS1500	-1140	*CD207*	*CD207*
cg10616306	9.41E-08	1.23E-05	-5.83921	5	87564718	TSS200;TSS200;TSS200;TSS200;TSS200;TSS200;TSS200;TSS200;TSS200;Body;Body;TSS200;TSS200	20	*TMEM161B;TMEM161B-AS1*	*TMEM161B-AS1*
cg21922577	9.45E-08	1.23E-05	5.83827	5	1.49E+08	Body	9753	*CSF1R*	*CSF1R*
cg10158715	9.47E-08	1.24E-05	5.83775	15	83382987	TSS5000;TSS5000;TSS5000;TSS5000;TSS1500	-242	*AP3B2;LOC338963*	*LOC338963*
cg27625815	9.47E-08	1.24E-05	5.837658	8	8195300	Body;Body;Body	48454	*PRAG1*	*PRAG1*
cg11828251	9.48E-08	1.24E-05	5.837586	1	1.11E+08	Body	22549	*SLC6A17*	*SLC6A17*
cg22878469	9.48E-08	1.24E-05	5.837548	5	235427	Body;Body;Body	16993	*SDHA*	*SDHA*
cg22971153	9.49E-08	1.24E-05	5.837351	2	1.14E+08	TES5000;TES5000;TSS1500;TSS5000	-244	*IL36RN;IL1F10*	*IL1F10*
cg08013274	9.51E-08	1.24E-05	5.836756	20	3709877	TSS5000;TSS5000;TSS5000	-3454	*HSPA12B*	*HSPA12B*
cg01567754	9.52E-08	1.24E-05	5.836641	14	1.03E+08	3'UTR;TES5000;TES5000;TES5000	10558	*TECPR2;ANKRD9*	*ANKRD9*
cg10287483	9.52E-08	1.24E-05	5.836495	17	74300440	Body;Body	3321	*QRICH2*	*QRICH2*
cg16435782	9.55E-08	1.24E-05	5.83578	21	39087399	Body	201361	*KCNJ6*	*KCNJ6*
cg15324331	9.55E-08	1.24E-05	5.835664	10	30085133		-59268		*SVIL*
cg03940520	9.56E-08	1.24E-05	5.835604	17	47925010	Body;Body;Body;Body;Body;TES5000	369	*TAC4;FLJ45513*	*TAC4*
cg12116027	9.56E-08	1.25E-05	5.835428	12	58130410	Body;Body	1619	*AGAP2*	*AGAP2*
cg21860675	9.57E-08	1.25E-05	5.835404	3	71586357	Body;Body;Body;Body;Body;Body;Body;Body;Body;Body;TES5000	4883	*FOXP1;MIR1284*	*MIR1284*
cg11643099	9.57E-08	1.25E-05	5.835246	1	26485229	TSS1500	-340	*FAM110D*	*FAM110D*
cg27515201	9.57E-08	1.25E-05	5.835206	1	33363113	TES5000;Body;Body;Body;Body;Body	3877	*HPCA;TMEM54*	*TMEM54*
cg01371004	9.58E-08	1.25E-05	5.835066	2	1.21E+08	Body	-114050	*PTPN4*	*EPB41L5*
cg10755016	9.58E-08	1.25E-05	5.835023	2	23607015	TSS1500	-1034	*KLHL29*	*KLHL29*
cg12274407	9.58E-08	1.25E-05	5.834992	1	1074226	Body	1830	*LINC01342*	*LINC01342*
cg21915998	9.59E-08	1.25E-05	5.834864	2	67060872		-70695		*LINC01799*
cg10251973	9.59E-08	1.25E-05	5.834708	14	93419016	Body;Body;Body;Body	29522	*ITPK1*	*CHGA*
cg24152353	9.59E-08	1.25E-05	5.834685	1	6149107	Body;Body;Body;Body;Body;Body	43160	*KCNAB2*	*KCNAB2*
cg17865718	9.60E-08	1.25E-05	5.834664	3	1.04E+08		-683593		*MIR548AB*
cg05405275	9.60E-08	1.25E-05	5.834511	7	1.51E+08		-24591		*AOC1*
cg19816367	9.61E-08	1.25E-05	5.834337	1	3419426	Body	48198	*MEGF6*	*ARHGEF16*
cg22828707	9.62E-08	1.25E-05	5.834114	14	79950202	Body;Body;Body;Body;Body;Body;Body;Body;Body;Body;Body;Body	204480	*NRXN3*	*NRXN3*
cg24710435	9.62E-08	1.25E-05	5.834038	10	14543847		30866		*FAM107B*
cg22145559	9.63E-08	1.25E-05	5.833904	20	57583009	TSS1500	-700	*CTSZ*	*CTSZ*
cg13793214	9.63E-08	1.25E-05	5.833767	9	95379929	TES5000;3'UTR;TES5000;Body;TSS1500	-402	*CENPP;IPPK;LOC100128361*	*LOC100128361*
cg02145319	9.64E-08	1.25E-05	5.833651	16	339492	TES5000;Body;Body;Body	6329	*PDIA2;AXIN1*	*PDIA2*
cg10763141	9.64E-08	1.25E-05	5.83357	13	28589808	Body;Body	-26978	*FLT3*	*URAD*
cg18558108	9.64E-08	1.25E-05	5.833489	8	1.31E+08	Body;Body;Body;Body;Body	16810	*ASAP1*	*ASAP1*
cg19387862	9.65E-08	1.25E-05	5.833307	19	50931875	3'UTR;3'UTR;3'UTR;3'UTR;TSS5000	-4273	*SPIB;MYBPC2*	*MYBPC2*
cg09368832	9.65E-08	1.25E-05	5.833269	17	9146934	3'UTR	-64499	*NTN1*	*LOC101928266*
cg24315508	9.67E-08	1.25E-05	5.832842	3	43234682		-87111		*POMGNT2*
cg18068923	9.68E-08	1.25E-05	5.832691	3	14386950	TES5000	7118	*LINC01267*	*LINC01267*
cg24004139	9.68E-08	1.25E-05	5.832653	1	26670703	1stExon	9918	*CRYBG2*	*CRYBG2*
cg07126091	9.68E-08	1.25E-05	5.832577	8	1.41E+08	Body	98205	*KCNK9*	*KCNK9*
cg11422092	9.68E-08	1.25E-05	5.832572	12	83744367		663434		*TMTC2*
cg05860371	9.68E-08	1.25E-05	5.832554	4	1.53E+08		-90613		*LINC02273*
cg09868359	9.68E-08	1.25E-05	5.832516	21	43230669	Body;Body;Body;Body;Body;Body;Body	-43457	*PRDM15*	*RIPK4*
cg07614623	9.69E-08	1.25E-05	5.832484	5	56600264		90364		*GPBP1*
cg11518883	9.69E-08	1.25E-05	5.832364	1	6758433	Body	3443	*DNAJC11*	*DNAJC11*
cg18533365	9.70E-08	1.25E-05	5.832205	10	1.34E+08	Body	70043	*INPP5A*	*INPP5A*
cg04181542	9.72E-08	1.25E-05	5.831775	6	1.01E+08		-142276		*MCHR2*
cg05528931	9.72E-08	1.25E-05	5.83162	3	16539054	Body	16152	*RFTN1*	*RFTN1*
cg01361261	9.73E-08	1.25E-05	5.831409	10	436604	Body	210671	*DIP2C*	*ZMYND11*
cg19300655	9.73E-08	1.25E-05	5.83137	1	1.84E+08	Body;Body;Body;TSS5000;TSS5000	-3833	*RGL1*	*RGL1*
cg08903422	9.73E-08	1.25E-05	5.831307	17	7328532	TES5000;TSS1500;TSS5000;TSS5000	-402	*SPEM1;SPEM2;SPEM3*	*SPEM2*
cg26359389	9.74E-08	1.25E-05	5.831237	2	2.4E+08	Body	6960	*HDAC4*	*MIR4440*

Probe	p_adj	q_val	t	Chr	Pos.hg19	locations	dist2TSS	gene.Illumina	gene.nearest
cg07233483	9.75E-08	1.26E-05	5.830841	5	1.54E+08	3'UTR;Body	26731	*GALNT10;SAP30L-AS1*	*SAP30L-AS1*
cg00093829	9.76E-08	1.26E-05	5.830739	10	6247101	Body;Body;Body;Body;Body;Body;Body;Body	2207	*PFKFB3*	*PFKFB3*
cg23877683	9.76E-08	1.26E-05	5.830723	10	88122364	Body	4188	*GRID1*	*GRID1*
cg12492670	9.76E-08	1.26E-05	5.830713	11	50231261		26372		*LOC441601*
cg04365255	9.77E-08	1.26E-05	5.830442	6	1.23E+08	Body	4213	*FABP7*	*FABP7*
cg22763136	9.78E-08	1.26E-05	5.830331	10	73395000	Body;Body;Body;Body	84535	*CDH23*	*C10orf105*
cg02337695	9.79E-08	1.26E-05	5.829929	19	49585219	TSS5000;TSS5000	-3488	*SNRNP70*	*SNRNP70*
cg20887522	9.80E-08	1.26E-05	5.829833	10	30918800	TSS200	-118	*LYZL2*	*LYZL2*
cg04977343	9.80E-08	1.26E-05	5.82975	6	56777045	Body	42583	*DST*	*DST*
cg02264779	9.82E-08	1.26E-05	5.829247	22	24817310	TES5000;TES5000;Body;TES5000;TES5000;TSS5000	-2254	*SPECC1L;SPECC1L-ADORA2A;ADORA2A*	*ADORA2A*
cg18753337	9.83E-08	1.26E-05	5.829074	15	80206998	Body;Body;Body	-8114	*ST20-MTHFS;ST20*	*ST20-AS1*
cg17282592	9.84E-08	1.26E-05	5.828681	10	6797081		17738		*LINP1*
cg12952132	9.86E-08	1.27E-05	5.828284	19	55417647	Body;Body;Body;Body;Body	142	*NCR1*	*NCR1*
cg19599165	9.87E-08	1.27E-05	5.828059	2	1.2E+08	3'UTR;TES5000	6098	*TMEM37;SCTR*	*TMEM37*
cg17925308	9.88E-08	1.27E-05	5.827756	14	69344852	Body;Body;Body	-81892	*ACTN1*	*ZFP36L1*
cg11724750	9.89E-08	1.27E-05	5.827506	6	1.64E+08	Body;Body;Body	54211	*PACRG*	*PACRG-AS3*
cg02520816	9.90E-08	1.27E-05	5.82726	16	4056403	Body	-55958	*ADCY9*	*LINC02861*
cg15370309	9.92E-08	1.27E-05	5.826956	17	9141550	Body	-59115	*NTN1*	*LOC101928266*
cg04470324	9.92E-08	1.27E-05	5.826949	5	1.33E+08	Body	175695	*FSTL4*	*MIR1289-2*
cg24557027	9.93E-08	1.27E-05	5.826772	4	95335239		-37768		*PDLIM5*
cg09977980	9.93E-08	1.27E-05	5.826732	17	18283678	Body	2587	*EVPLL*	*EVPLL*
cg05892673	9.95E-08	1.27E-05	5.826214	17	43335632	Body;Body;Body;Body;TSS5000;TSS5000;TES5000	-3814	*MAP3K14-AS1;SPATA32;MAP3K14*	*MAP3K14-AS1*
cg02702692	9.95E-08	1.27E-05	5.826207	11	71724504	Body;Body;Body;TSS1500	-832	*NUMA1;LOC100128494*	*LOC100128494*
cg14542378	9.97E-08	1.28E-05	5.825661	4	1.85E+08	Body;Body	105089	*STOX2*	*STOX2*
cg20984614	9.99E-08	1.28E-05	5.825148	8	1.45E+08	Body;Body;Body;Body;Body;Body;Body;Body;Body;Body;Body;Body;TSS5000	-4976	*ZNF707;BREA2*	*BREA2*
cg06519044	1.00E-07	1.28E-05	5.825013	17	79237297	Body;Body	23584	*SLC38A10*	*NDUFAF8*
cg24655428	1.00E-07	1.28E-05	5.824585	15	74467047	Body;Body;TES5000;TES5000;TES5000;TES5000;TES5000;TES5000;TES5000	160	*ISLR;STRA6*	*ISLR*
cg00436254	1.00E-07	1.28E-05	5.82451	2	1.28E+08	Body;Body;Body;Body;Body;Body;Body;Body;Body;Body;Body;Body;Body;Body	2116	*BIN1*	*BIN1*
cg14170172	1.00E-07	1.28E-05	5.824272	21	45675982	Body;Body;TES5000	5287	*DNMT3L;DNMT3L-AS1*	*DNMT3L-AS1*
cg15035476	1.00E-07	1.28E-05	5.824162	19	46457107	Body	20016	*NOVA2*	*NOVA2*
cg04578792	1.00E-07	1.28E-05	5.824036	3	1.28E+08		-30168		*DNAJB8-AS1*
cg08007639	1.00E-07	1.28E-05	5.823991	16	2999034	3'UTR;3'UTR	-15241	*FLYWCH1*	*KREMEN2*
cg09434667	1.01E-07	1.28E-05	5.823745	15	67078890	TES5000;TES5000	84317	*SMAD6*	*SMAD6*
cg02332902	1.01E-07	1.28E-05	5.823651	7	1103177	Body;Body;Body;Body;Body;TES5000;TES5000;TES5000	8172	*C7orf50;GPR146*	*GPR146*
cg18908677	1.01E-07	1.29E-05	5.823199	10	1.21E+08	Body	21232	*BAG3*	*BAG3*
cg03147430	1.01E-07	1.29E-05	5.823056	16	81929395	Body	115680	*PLCG2*	*SDR42E1*
cg03788952	1.01E-07	1.29E-05	5.822804	18	55474222	TSS5000	-3675	*ATP8B1*	*ATP8B1*
cg24691014	1.01E-07	1.29E-05	5.822129	8	1.12E+08		331904		*LINC01608*
cg07571379	1.01E-07	1.29E-05	5.822109	11	1.35E+08	Body	25589	*LINC02714*	*LINC02714*
cg00902427	1.01E-07	1.29E-05	5.822087	22	41074991	TSS200	-190	*MCHR1*	*MCHR1*
cg12903574	1.02E-07	1.29E-05	5.821514	15	37326903	Body;Body;Body;Body;Body;Body;Body;Body;Body	-61801	*MEIS2*	*MIR8063*
cg03873049	1.02E-07	1.29E-05	5.821439	5	1.5E+08	Body;Body;Body;Body;Body	44142	*CAMK2A*	*CAMK2A*
cg12556802	1.02E-07	1.29E-05	5.821358	4	1.52E+08	Body;Body;Body	-24016	*SH3D19*	*SH3D19*
cg00391945	1.02E-07	1.29E-05	5.821019	6	68598790		-166452		*LOC102723883*
cg07280155	1.02E-07	1.29E-05	5.820833	8	41346222	TSS5000;TSS5000;TSS5000;TSS5000;TSS5000;TSS5000;TSS5000;TSS5000	-1692	*GOLGA7*	*GOLGA7*
cg11949175	1.02E-07	1.29E-05	5.820795	2	1.36E+08	Body;Body;Body	76153	*ZRANB3*	*SNORA40B*
cg20934799	1.02E-07	1.30E-05	5.820258	11	36433850	Body;Body;Body;Body	11304	*PRR5L*	*PRR5L*
cg24336769	1.02E-07	1.30E-05	5.820111	6	38305513	Body;Body;Body;Body	-143858	*BTBD9*	*BTBD9-AS1*
cg27076046	1.02E-07	1.30E-05	5.820062	21	45683528	TSS5000;TSS5000	-1748	*DNMT3L*	*DNMT3L*
cg01079795	1.02E-07	1.30E-05	5.819976	1	1.11E+08		-90811		*CD53*
cg18973586	1.02E-07	1.30E-05	5.819831	4	963968	Body	3389	*DGKQ*	*DGKQ*
cg17471193	1.02E-07	1.30E-05	5.819776	6	32056667	Body;Body	20448	*TNXB*	*TNXB*
cg16500987	1.02E-07	1.30E-05	5.8194	6	1.67E+08	Body;Body;Body	-64022	*RPS6KA2*	*RPS6KA2*
cg13639244	1.02E-07	1.30E-05	5.819267	17	17717006	TES5000;Body;Body;Body;TSS200;TES5000	-147	*RAI1;SREBF1;MIR6777;MIR33B*	*MIR6777*
cg02802750	1.03E-07	1.30E-05	5.819144	17	10543522	Body	17104	*MYH3*	*MYH3*
cg26894297	1.03E-07	1.30E-05	5.819062	1	23754550	TSS5000;TES5000;TES5000	-3309	*TCEA3;ASAP3*	*TCEA3*
cg08967456	1.03E-07	1.30E-05	5.818909	16	3114948	TES5000;TSS1500;TSS1500;TSS1500;TSS1500;TSS1500;TSS1500;TSS1500;TSS1500;TSS1500;TSS1500;TSS1500;TSS1500;TSS1500;TSS1500 ;TSS1500;TSS1500;TSS1500;TSS1500	-455	*MMP25;IL32*	*IL32*
cg18375902	1.03E-07	1.30E-05	5.818743	4	7764949	Body;3'UTR;3'UTR;3'UTR;3'UTR	9133	*AFAP1-AS1;AFAP1*	*AFAP1-AS1*
cg15381259	1.03E-07	1.30E-05	5.818444	7	26525682	Body;Body;Body;Body;Body;Body	52762	*LOC441204*	*KIAA0087*

Probe	p_adj	q_val	t	Chr	Pos.hg19	locations	dist2TSS	gene.Illumina	gene.nearest
cg08324925	1.03E-07	1.30E-05	5.818292	5	1.31E+08	TSS1500	-322	*CSF2*	*CSF2*
cg08684959	1.03E-07	1.30E-05	5.818047	11	70031709	Body;Body	-17559	*ANO1*	*FADD*
cg14057644	1.03E-07	1.30E-05	5.817988	11	1.28E+08	TES5000;TES5000;TES5000;TES5000	64540	*ETS1*	*ETS1*
cg24601424	1.03E-07	1.30E-05	5.817933	2	1.93E+08		-8354		*CAVIN2*
cg15745944	1.03E-07	1.30E-05	5.817794	17	39861969		-6613		*GAST*
cg07972026	1.03E-07	1.30E-05	5.817639	6	1636961	Body;Body	26812	*GMDS*	*FOXC1*
cg23485716	1.03E-07	1.31E-05	5.817555	10	390268	Body	164335	*DIP2C*	*ZMYND11*
cg24364282	1.03E-07	1.31E-05	5.817402	6	1.14E+08		-51764		*MARCKS*
cg02167986	1.03E-07	1.31E-05	5.817172	14	68201122	3'UTR	32520	*RDH12*	*RDH12*
cg07382129	1.03E-07	1.31E-05	5.817132	15	1E+08	Body;Body;Body;Body;Body;Body;Body;Body;Body;Body;Body;Body;Body;Body;Body;Body;Body;Body;Body;Body	24456	*MEF2A*	*LYSMD4*
cg02401639	1.03E-07	1.31E-05	5.816991	12	4405549	Body	-20199	*CCND2*	*CCND2-AS1*
cg24057701	1.04E-07	1.31E-05	5.816931	13	1.12E+08	Body	30573	*LINC02337*	*LINC02337*
cg03466779	1.04E-07	1.31E-05	5.816891	4	7666141	Body	-89675	*SORCS2*	*AFAP1-AS1*
cg27523644	1.04E-07	1.31E-05	5.816611	10	1.31E+08		-51260		*MGMT*
cg02975187	1.04E-07	1.31E-05	5.816541	22	18512423	Body	273	*LINC01634*	*LINC01634*
cg26500398	1.04E-07	1.31E-05	5.816431	8	22974616	3'UTR	14290	*TNFRSF10C*	*TNFRSF10C*
cg10262188	1.04E-07	1.31E-05	5.816203	10	490478	Body	-184025	*DIP2C*	*LOC101930421*
cg09652142	1.04E-07	1.31E-05	5.816196	3	47932918	Body;Body	41874	*MAP4*	*MIR1226*
cg21533544	1.04E-07	1.31E-05	5.81619	4	1.85E+08	3'UTR	-6064	*IRF2*	*LINC02362*
cg19476368	1.04E-07	1.31E-05	5.815828	11	1.22E+08		207000		*MIR125B1*
cg03552937	1.04E-07	1.31E-05	5.815665	7	44795713	Body;Body;TSS200	-73	*ZMIZ2*	*ZMIZ2*
cg03785426	1.04E-07	1.31E-05	5.815662	12	1.24E+08	Body;Body;TES5000;TES5000;TES5000;TES5000;TES5000;TES5000	36636	*DNAH10;CCDC92*	*CCDC92*
cg26311677	1.04E-07	1.31E-05	5.815248	3	1.68E+08	Body	-126692	*EGFEM1P*	*MIR551B*
cg02365577	1.04E-07	1.31E-05	5.815173	19	38486137	Body	88290	*SIPA1L3*	*SIPA1L3*
cg25422089	1.04E-07	1.31E-05	5.81515	17	72210927	TES5000;TES5000;TSS1500;Body;TSS1500	-841	*RPL38;MGC16275;TTYH2*	*TTYH2*
cg13554033	1.04E-07	1.31E-05	5.814985	14	99951125	TSS5000;TSS5000;Body	3380	*SETD3;CCNK*	*CCNK*
cg16572910	1.04E-07	1.31E-05	5.814951	1	43880657	Body;Body	25088	*SZT2*	*SZT2*
cg10406414	1.05E-07	1.31E-05	5.814681	8	49587029	Body	54067	*LOC101929268*	*LOC101929217*
cg00215019	1.05E-07	1.32E-05	5.813896	10	50311040	Body;3'UTR	12527	*VSTM4*	*VSTM4*
cg23324442	1.05E-07	1.32E-05	5.813869	14	1.02E+08	TSS1500	-944	*LINC00239*	*LINC00239*
cg20663939	1.05E-07	1.32E-05	5.813861	15	72526917	TSS5000;TSS5000;TSS5000;TSS5000;TSS5000;TSS5000;TSS5000;TSS5000	-2952	*PKM*	*PKM*
cg22553828	1.05E-07	1.32E-05	5.813842	5	1.74E+08	TES5000	2307	*MIR4634*	*MIR4634*
cg14862385	1.05E-07	1.32E-05	5.813833	5	58321624	Body;Body;Body;Body;Body;Body;Body;Body;Body;Body;Body;Body;Body;Body	13679	*PDE4D*	*PDE4D*
cg10304865	1.05E-07	1.32E-05	5.813789	6	41371102		67757		*NCR2*
cg26007962	1.05E-07	1.32E-05	5.813761	1	973887	Body;Body;Body	4515	*AGRN*	*AGRN*
cg06369573	1.05E-07	1.32E-05	5.81361	19	19755321	TSS1500;TSS1500;TSS1500;TES5000	-855	*GMIP;ATP13A1*	*GMIP*
cg14049634	1.05E-07	1.32E-05	5.813491	9	1.16E+08	Body;Body;Body;Body;Body;Body	-10151	*RGS3*	*RGS3*
cg19016444	1.05E-07	1.32E-05	5.81332	17	17061977	Body;Body;Body	47670	*MPRIP*	*PLD6*
cg23217463	1.05E-07	1.32E-05	5.813187	17	46676973	Body;Body;Body;Body;Body;Body;Body;Body;Body;Body	3654	*HOXB-AS3;HOXB6*	*HOXB-AS3*
cg09569989	1.05E-07	1.32E-05	5.812993	4	38053419	Body;Body	-58382	*TBC1D1*	*TBC1D1*
cg02883621	1.05E-07	1.32E-05	5.812892	4	6694923	TSS5000;TSS1500	-681	*LINC002481;S100P*	*S100P*
cg17695382	1.05E-07	1.32E-05	-5.8127	2	61697821	1stExon	401	*USP34*	*USP34*
cg16314923	1.05E-07	1.32E-05	5.812545	12	7918357	Body	546	*NANOGNB*	*NANOGNB*
cg08616951	1.06E-07	1.32E-05	5.812397	11	394545	Body;Body	337	*PKP3*	*PKP3*
cg18255764	1.06E-07	1.32E-05	5.812295	2	26405871	Body;Body	2287	*GAREM2*	*GAREM2*
cg22454053	1.06E-07	1.32E-05	5.812152	1	79801791		-329376		*ADGRL4*
cg02834447	1.06E-07	1.32E-05	5.812102	2	2.38E+08		198762		*LOC93463*
cg03795245	1.06E-07	1.32E-05	5.811871	12	6929671	3'UTR;3'UTR;3'UTR;3'UTR;3'UTR;TSS1500;TSS1500	-1315	*CD4;GPR162*	*GPR162*
cg17082959	1.06E-07	1.32E-05	5.811674	1	2.09E+08		-117023		*LINC01696*
cg07244783	1.06E-07	1.32E-05	5.811436	4	4432211	Body;Body;Body;Body;Body	31488	*STX18*	*STX18*
cg13528199	1.06E-07	1.32E-05	5.811402	10	1.35E+08		50607		*LINC01168*
cg03070534	1.06E-07	1.32E-05	5.81137	13	1.15E+08		-32339		*RASA3*
cg04294388	1.06E-07	1.32E-05	5.811002	20	44780839		33887		*CD40*
cg25105990	1.06E-07	1.33E-05	5.810533	1	28921512	3'UTR;3'UTR	2506	*RAB42*	*RAB42*
cg06496010	1.07E-07	1.33E-05	5.810249	2	97201196	TSS5000;TSS1500;TSS1500;TSS1500;TSS1500;TSS1500;TSS1500;TSS5000	-1292	*LOC105373496;ARID5A*	*ARID5A*
cg04903829	1.07E-07	1.33E-05	5.810005	16	2027629	Body;TES5000;TES5000;TES5000;TES5000	3841	*TBL3;NOXO1*	*NOXO1*
cg14632918	1.07E-07	1.33E-05	5.809902	7	1.58E+08	Body;Body;Body;Body;Body	311023	*PTPRN2*	*LOC100506585*
cg05077865	1.07E-07	1.33E-05	5.809902	20	2637470	Body;Body;Body;TES5000;TES5000;TES5000;TES5000;TES5000;TSS200;TES5000;TES5000;TES5000;TES5000;TES5000	-114	*NOP56;MIR1292;SNORD110;SNORA51;SNORD86;SNORD56;SNORD57;IDH3B*	*SNORD57*
cg09258200	1.07E-07	1.33E-05	5.809397	4	77669688	Body	149609	*SHROOM3*	*SOWAHB*

Probe	p_adj	q_val	t	Chr	Pos.hg19	locations	dist2TSS	gene.Illumina	gene.nearest
cg21686379	1.07E-07	1.33E-05	-5.80905	20	61448079	TES5000;TSS1500	-322	*OGFR;COL9A3*	*COL9A3*
cg11909975	1.07E-07	1.33E-05	5.808889	2	1.28E+08		-13017		*MAP3K2*
cg16706374	1.07E-07	1.33E-05	5.808777	14	1.01E+08	Body;5'UTR	3975	*SLC25A47*	*SLC25A47*
cg04366880	1.07E-07	1.34E-05	5.80856	10	93431680		-38852		*PPP1R3C*
cg06897790	1.07E-07	1.34E-05	-5.80831	5	87564715	TSS200;TSS200;TSS200;TSS200;TSS200;TSS200;TSS200;TSS200;TSS200;Body;Body;TSS200;TSS200	17	*TMEM161B;TMEM161B-AS1*	*TMEM161B-AS1*
cg13438549	1.08E-07	1.34E-05	5.807913	17	48633206	3'UTR;3'UTR;3'UTR;TES5000;TSS5000	-4869	*SPATA20;CACNA1G-AS1;CACNA1G*	*CACNA1G*
cg00313498	1.08E-07	1.34E-05	5.807768	4	7787944	Body;Body;Body;Body	32128	*AFAP1*	*AFAP1-AS1*
cg19335195	1.08E-07	1.34E-05	5.807145	7	1.52E+08	TES5000	58995	*KMT2C*	*GALNT11*
cg04628181	1.08E-07	1.34E-05	5.806962	13	25250623	TSS5000;TSS5000	-3922	*ATP12A*	*ATP12A*
cg05682965	1.08E-07	1.35E-05	5.806382	19	15292631	Body;TSS5000	-2470	*NOTCH3;MIR6795*	*MIR6795*
cg10044629	1.08E-07	1.35E-05	5.806356	11	1097602	Body	22728	*MUC2*	*MUC2*
cg00908271	1.09E-07	1.35E-05	5.805817	16	81254010	TSS200;TSS200	-11	*PKD1L2*	*PKD1L2*
cg11025750	1.09E-07	1.35E-05	5.805634	2	45618637	Body	-136557	*SRBD1*	*LINC01121*
cg04154583	1.09E-07	1.35E-05	5.805456	22	44889756	3'UTR	4357	*RTL6*	*RTL6*
cg11935041	1.09E-07	1.35E-05	5.805398	5	1.51E+08		-28885		*FAT2*
cg26721890	1.09E-07	1.35E-05	5.80521	5	1.38E+08	Body	629	*GFRA3*	*GFRA3*
cg24339194	1.09E-07	1.35E-05	5.80475	19	43911682	Body	-7359	*TEX101*	*TEX101*
cg07011538	1.09E-07	1.35E-05	5.804735	2	47497026	Body;Body	-65427	*EPCAM-DT*	*BCYRN1*
cg26914630	1.09E-07	1.35E-05	5.804629	16	88455751	Body	6385	*ZNF469*	*ZNF469*
cg12890671	1.09E-07	1.35E-05	5.804569	8	1.45E+08	TSS5000	-3269	*OPLAH*	*OPLAH*
cg18680035	1.09E-07	1.35E-05	5.804512	19	6818326	Body;Body;Body	45608	*VAV1*	*VAV1*
cg09603381	1.09E-07	1.35E-05	5.804337	10	49954066	Body;Body	60559	*WDFY4*	*WDFY4*
cg18222440	1.10E-07	1.36E-05	5.803619	1	6366658	Body;Body;Body;Body	-45500	*ACOT7*	*GPR153*
cg19589494	1.10E-07	1.36E-05	5.803579	11	9648370		-37253		*SWAP70*
cg26744084	1.10E-07	1.36E-05	5.803569	6	29581182	Body;Body;Body;Body	14823	*GABBR1*	*GABBR1*
cg17724366	1.10E-07	1.36E-05	5.80355	19	52249171	3'UTR;3'UTR	5960	*FPR1*	*FPR1*
cg07058918	1.10E-07	1.36E-05	5.8035	2	2.33E+08	TSS1500	-376	*ALPI*	*ALPI*
cg25932761	1.10E-07	1.36E-05	5.803275	12	1.12E+08	Body;Body	19031	*CUX2*	*MIR6760*
cg02818160	1.10E-07	1.36E-05	5.803262	2	15804728		-26177		*LINC01804*
cg15655295	1.10E-07	1.36E-05	5.803137	19	30367842		-46721		*URI1*
cg07669776	1.10E-07	1.36E-05	5.802955	13	1.11E+08	Body	16061	*COL4A2*	*COL4A2*
cg02941085	1.10E-07	1.36E-05	5.802938	7	1.57E+08	Body;Body;Body;Body;Body	-46173	*PTPRN2*	*MIR153-2*
cg22540431	1.10E-07	1.36E-05	5.802548	6	42290322	Body;Body	-104723	*TRERF1*	*MRPS10*
cg01147055	1.10E-07	1.36E-05	5.80252	13	1.15E+08	Body;Body;Body	5268	*RASA3*	*RASA3*
cg23329275	1.10E-07	1.36E-05	5.802484	5	14508132	Body;Body	44041	*TRIO*	*SNORD170*
cg25673112	1.10E-07	1.36E-05	5.801736	10	1.27E+08	Body;Body;Body;Body;Body;Body;Body	22948	*CTBP2*	*CTBP2*
cg10778636	1.10E-07	1.36E-05	5.801698	1	20878361	TSS1500	-704	*FAM43B*	*FAM43B*
cg09371620	1.10E-07	1.36E-05	5.80169	12	46319990	Body	64367	*SCAF11*	*SCAF11*
cg08303483	1.11E-07	1.36E-05	5.801504	10	50506293	Body;TSS1500	645	*C10orf71-AS1;C10orf71*	*C10orf71-AS1*
cg23518497	1.11E-07	1.36E-05	5.801299	12	60298928		215778		*SLC16A7*
cg07463197	1.11E-07	1.36E-05	5.801235	5	1.46E+08		-36491		*TCERG1*
cg00550529	1.11E-07	1.36E-05	5.801167	12	1.04E+08	TSS1500	-1380	*STAB2*	*STAB2*
cg06258549	1.11E-07	1.36E-05	5.801143	1	3274631	Body;Body	-96597	*PRDM16*	*ARHGEF16*
cg14285539	1.11E-07	1.36E-05	5.801055	9	1.27E+08	Body;Body;Body;Body	-9506	*NEK6*	*LOC613206*
cg21295295	1.11E-07	1.36E-05	5.800999	1	1.56E+08	TSS5000;TSS5000;TSS5000;TSS5000;5'UTR;Body;Body	2333	*SMG5;TMEM79*	*TMEM79*
cg24516799	1.11E-07	1.36E-05	5.800932	17	1409928	Body;Body;Body	9978	*INPP5K*	*INPP5K*
cg23239690	1.11E-07	1.36E-05	5.800573	10	1.32E+08	Body	135823	*MGMT*	*MIR4297*
cg07928065	1.11E-07	1.37E-05	5.800447	17	9694739	5'UTR;5'UTR	170	*DHRS7C*	*DHRS7C*
cg22522939	1.11E-07	1.37E-05	5.800093	8	1.44E+08		30785		*LY6H*
cg25195987	1.11E-07	1.37E-05	5.80008	7	31125925	Body;Body;Body;Body	33850	*ADCYAP1R1*	*ADCYAP1R1*
cg13710586	1.11E-07	1.37E-05	5.799527	14	36274049	Body;Body;Body;Body;Body;Body;Body;Body;Body;Body;Body	4452	*RALGAPA1*	*RALGAPA1*
cg15236035	1.12E-07	1.37E-05	5.799486	17	48658011	Body;Body;Body;Body;Body;Body;Body;Body;Body;Body;Body;Body;Body;Body;Body;Body;Body;Body;Body;Body;Body;Body;Body;Body;Body;Body;Body;Body;Body;Body;Body;Body;Body	-18542	*CACNA1G*	*CACNA1G-AS1*
cg02217616	1.12E-07	1.37E-05	5.799334	11	79079450	Body	33703	*TENM4*	*MIR708*
cg13547132	1.12E-07	1.37E-05	5.799218	1	2835258		-102772		*ACTRT2*
cg18415147	1.12E-07	1.37E-05	5.799202	14	1.06E+08		-39596		*ELK2AP*
cg24960565	1.12E-07	1.37E-05	5.799194	3	1.17E+08		124320		*LINC00901*
cg13571576	1.12E-07	1.37E-05	5.798937	15	1.02E+08	Body	128410	*LRRK1*	*LRRK1*

Table S4. Differentially methylated CpG sites associated with bronchopulmonary dysplasia (BPD) risk and their annotated gene information.

Illumina HumanMethylation 450 BeadChip analysis on cord blood DNA from infants (n=107).

Bonferroni (BF) correction < 1.08E-07, false discovery rate (FDR) < 0.05. M.BPD=mean methlation in BPD samples. M.nonBPD=mean methlation in non-BPD samples. dMeth=methylation difference.

Chr=chromosome. Pos.hg19=position on Genome Reference Consortium Human Build 37 (GRCh37, hg19). dist2TSS=distance to transcription start site.

#cpg	p_adj	q_val	Meth.BPD	Meth.nonBPD	dMeth	significance	Chr	pos.hg19	locations	dist2TSS	gene.nearest	gene.Illumina
cg23328237	9.26E-12	4.14E-06	0.849641642	0.853523317	-0.003881675	BF|FDR0.01	15	79252815	3'UTR;3'UTR;3'UTR	-15375	***CTSH***	***RASGRF1***
cg08139238	7.41E-11	1.66E-05	0.774967052	0.819469637	-0.044502585	BF|FDR0.01	16	22219550	Body	1959	***EEF2K***	***EEF2K***
cg14448850	4.46E-10	6.64E-05	0.886345564	0.901223039	-0.014877475	BF|FDR0.01	7	22860191	Body	2280	***TOMM7***	***TOMM7***
cg22707878	6.17E-10	6.90E-05	0.816786549	0.837319708	-0.020533159	BF|FDR0.01	3	122604848	TES5000;TSS1500	-511	***LINC02035***	***DIRC2;LINC02035;SLC49A4***
cg23540651	9.46E-10	8.46E-05	0.819033795	0.847103698	-0.028069903	BF|FDR0.01	1	89490034	TSS1500;TSS1500;TSS1500;TSS1500;TSS1500	-1485	***GBP3***	***GBP3***
cg26968498	2.58E-09	0.000191968	0.870714636	0.893481327	-0.022766691	BF|FDR0.01	4	26174157	Body	9081	***RBPJ***	***RBPJ***
cg00013369	5.54E-09	0.000348642	0.846119428	0.859335114	-0.013215687	BF|FDR0.01	1	192857594	Body;Body;Body;Body	-12479	***RN7SKP126***	***RP11-139E24.1;LINC01032***
cg19815139	6.24E-09	0.000348642	0.061461172	0.056136464	0.005324708	BF|FDR0.01	14	77924143	TSS200;TSS1500;TSS200;TSS200;TSS200;TSS200;TSS200	-76	***AHSA1***	***VIPAS39;AHSA1***
cg05577945	7.20E-09	0.000349426	0.052325138	0.045412247	0.006912892	BF|FDR0.01	13	78271874	TES5000;TSS200;TSS1500;TSS1500	-114	***SLAIN1***	***MIR3665;SLAIN1***
cg16708174	7.81E-09	0.000349426	0.768023451	0.784226033	-0.016202583	BF|FDR0.01	3	158430962	Body;Body	19313	***RARRES1***	***RARRES1***
cg06342416	1.48E-08	0.000567015	0.1594111	0.161170593	-0.001759493	BF|FDR0.01	21	45773569	5'UTR;5'UTR;5'UTR;Body	86	***TRPM2***	***TRPM2***
cg26371345	1.64E-08	0.000567015	0.16819043	0.128373094	0.039817336	BF|FDR0.01	6	44090748	TSS5000;Body;Body;Body;Body;Body;TSS5000;Body	3256	***MRPL14***	***TMEM63B;MRPL14***
cg22148297	1.65E-08	0.000567015	0.14387923	0.110502046	0.033377184	BF|FDR0.01	13	99910587	Body;Body;5'UTR;Body;5'UTR	95	***GPR18***	***UBAC2;GPR18***
cg24025538	2.03E-08	0.000648594	0.857670733	0.869462908	-0.011792175	BF|FDR0.01	19	50364499	3'UTR;TES5000;TSS5000;TES5000;TES5000;TES5000;TES5000;TES5000;TES5000;TES5000;TES5000	-1953	***PTOV1-AS2***	***PNKP;PTOV1;PTOV1-AS2***
cg09757859	2.31E-08	0.000667306	0.855357252	0.858543903	-0.003186651	BF|FDR0.01	4	119948505	Body;Body;Body;Body;Body	-108433	***MYOZ2***	***SYNPO2***
cg09551145	2.52E-08	0.000667306	0.112575594	0.110505544	0.00207005	BF|FDR0.01	1	11790591	TES5000	-5549	***AGTRAP***	***DRAXIN***
cg22833603	2.54E-08	0.000667306	0.849351644	0.872421165	-0.023069521	BF|FDR0.01	12	48304627	Body;TES5000	5723	***RP11-89H19.2***	***VDR;RP11-89H19.2***
cg20084577	3.20E-08	0.000757184	0.806925671	0.833170019	-0.026244348	BF|FDR0.01	13	33780913	TSS1500;Body;Body;TSS1500	-726	***STARD13***	***STARD13***
cg06787669	3.22E-08	0.000757184	0.065759787	0.053851569	0.011908218	BF|FDR0.01	7	45018789	TES5000;TSS200;TES5000;TES5000;TES5000;TES5000	-85	***MYO1G***	***SNHG15;MYO1G***
cg18067163	3.93E-08	0.000859377	0.769764803	0.781723609	-0.011958806	BF|FDR0.01	3	128584482	Body	5902	***LOC653712***	***LOC653712***
cg24252723	4.04E-08	0.000859377	0.033903109	0.028243164	0.005659945	BF|FDR0.01	10	104678150	5'UTR;5'UTR;5'UTR	76	***CNNM2***	***CNNM2;LOC107984265;LOC107984265***
cg07733260	4.27E-08	0.000866953	0.835412569	0.842587423	-0.007174854	BF|FDR0.01	3	4355431	Body;Body;Body;Body;Body;Body	10444	***SETMAR***	***SETMAR***
cg16176600	4.46E-08	0.000866953	0.701349752	0.718366877	-0.017017125	BF|FDR0.01	6	116381609	5'UTR	312	***FRK***	***FRK***
cg21845457	5.35E-08	0.00089824	0.097663129	0.074003127	0.023660002	BF|FDR0.01	8	65499091	TES5000;TES5000;TES5000;TSS5000	-4642	***LOC401463***	***CYP7B1;BHLHE22;LOC401463***
cg06733215	5.44E-08	0.00089824	0.794824222	0.818732536	-0.023908314	BF|FDR0.01	16	70317506	Body	5906	***AARS;AARS1***	***AARS;AARS1***
cg20238368	5.63E-08	0.00089824	0.850007668	0.855635447	-0.005627779	BF|FDR0.01	19	5795577	TSS5000;TSS5000	-4328	***DUS3L***	***DUS3L***
cg17270257	5.70E-08	0.00089824	0.069156038	0.057486848	0.011669191	BF|FDR0.01	6	166074870	Body;Body	718	***PDE10A***	***PDE10A***
cg24847366	5.72E-08	0.00089824	0.755362096	0.841000177	-0.085638081	BF|FDR0.01	12	125034283	Body;Body;Body	17727	***NCOR2***	***NCOR2***
cg22228748	5.82E-08	0.00089824	0.869282605	0.882075434	-0.012792829	BF|FDR0.01	1	99385800	Body;Body	-84031	***LOC100129620***	***PLPPR5***
cg10355458	6.88E-08	0.00100191	0.913246896	0.893515548	0.019731348	BF|FDR0.01	19	1578206	Body;Body	-10149	***MEX3D***	***MBD3***
cg02370222	7.17E-08	0.00100191	0.886140393	0.878210489	0.007929904	BF|FDR0.01	6	38580256	Body;Body	-16413	***BTBD9***	***BTBD9***
cg17958658	7.25E-08	0.00100191	0.0581027	0.044723185	0.013379515	BF|FDR0.01	10	73847963	1stExon;1stExon;1stExon	568	***SPOCK2***	***SPOCK2***
cg20064830	7.39E-08	0.00100191	0.833022019	0.863067158	-0.030045139	BF|FDR0.01	15	72535923	Body;Body;Body;Body;Body;Body;Body;Body;Body;Body;Body;Body;Body;Body;Body;Body;Body;Body;Body;Body;Body;Body;Body;Body;Body;Body	-11958	***PKM***	***PARP6***
cg07866137	8.21E-08	0.001080061	0.062374613	0.053195468	0.009179145	BF|FDR0.01	8	67579786	TSS1500;5'UTR;5'UTR	0	***C8orf44-SGK3***	***VCPIP1;C8orf44-SGK3;C8orf44***
cg13854960	9.35E-08	0.001194835	0.759201931	0.782268082	-0.023066151	BF|FDR0.01	9	112965949	Body	4464	***C9orf152***	***C9orf152***
cg08221288	9.96E-08	0.001227981	0.730600981	0.795337521	-0.06473654	BF|FDR0.01	14	50081280	3'UTR;Body;Body;TES5000;3'UTR	6123	***RPL36AL***	***LRR1;RPL36AL***
cg16290101	1.04E-07	0.001227981	0.857330556	0.873307924	-0.015977368	BF|FDR0.01	1	204010929	TSS1500	-537	***LINC00303***	***LINC00303***
cg07108118	1.04E-07	0.001227981	0.77091274	0.802408247	-0.031495508	BF|FDR0.01	1	76543815	Body;Body;Body;Body;Body;Body;Body;Body;Body;Body	3447	***ST6GALNAC3***	***ST6GALNAC3***
cg09261791	1.21E-07	0.001358289	0.825329224	0.861178834	-0.03584961	FDR0.01	14	52347968	Body;Body;Body	20947	***GNG2***	***GNG2***
cg18363035	1.27E-07	0.001358289	0.36558342	0.308288736	0.057294684	FDR0.01	13	20805385	TSS200;Body;Body;Body	-13	***GJB6***	***GJB6***
cg19954537	1.29E-07	0.001358289	0.100066187	0.086085533	0.013980654	FDR0.01	2	97204401	Body;Body;Body;Body;Body;Body;Body	786	***ARID5A***	***ARID5A***
cg04657062	1.30E-07	0.001358289	0.834396831	0.820842334	0.013554497	FDR0.01	7	150819920	Body;Body;Body;3'UTR;3'UTR;Body;Body;3'UTR	8156	***AGAP3***	***AGAP3***
cg24113274	1.31E-07	0.001358289	0.771514249	0.809878603	-0.038364354	FDR0.01	3	72487146	Body	8628	***RYBP***	***RYBP***
cg08953461	1.34E-07	0.001358289	0.050931663	0.039172678	0.011758986	FDR0.01	5	157158882	Body;Body;5'UTR;Body	62	***THG1L***	***THG1L***
cg19709585	1.40E-07	0.001358289	0.89081106	0.912829465	-0.022018405	FDR0.01	1	209825849	TSS200;TSS1500;TSS200	-29	***LAMB3***	***LAMB3***
cg26348616	1.40E-07	0.001358289	0.736807855	0.806068329	-0.069260474	FDR0.01	20	39661470	Body	4013	***TOP1***	***TOP1***
cg20885063	1.54E-07	0.001466117	0.677624042	0.678698695	-0.001074653	FDR0.01	17	17939419	Body;TSS5000	3061	***ATPAF2***	***ATPAF2;GID4***
cg11338339	1.66E-07	0.001544867	0.855904739	0.863492534	-0.007587794	FDR0.01	3	194823909	Body;Body	8593	***XXYLT1-AS1***	***XXYLT1***
cg12563372	1.73E-07	0.001580564	0.098255679	0.066828966	0.031426713	FDR0.01	8	25908503		-5590	***EBF2***	
cg01931028	1.85E-07	0.001619819	0.829038644	0.894248506	-0.065209862	FDR0.01	4	1287888	Body;Body;Body;Body;Body	4250	***MAEA***	***MAEA***
cg19699264	1.85E-07	0.001619819	0.889851723	0.895489589	-0.005637866	FDR0.01	2	192701019	Body	10987	***CAVIN2***	***CAVIN2***
cg19244312	1.91E-07	0.00163971	0.891212636	0.895567273	-0.004354637	FDR0.01	3	171964315	Body;Body	201931	***GHSR***	***FNDC3B***
cg01446515	1.96E-07	0.001654222	0.159520637	0.126188496	0.033332141	FDR0.01	13	20805443	TSS200;Body;Body;Body	-71	***GJB6***	***GJB6***
cg04141129	2.09E-07	0.001729469	0.697754124	0.773500119	-0.075745995	FDR0.01	12	32530696	3'UTR;3'UTR;3'UTR;3'UTR;Body;3'UTR	-108209	***FGD4***	***BICD1***

#cpg	p_adj	q_val	Meth.BPD	Meth.nonBPD	dMeth	significance	Chr	pos.hg19	locations	dist2TSS	gene.nearest	gene.Illumina
cg03761216	2.14E-07	0.001737673	0.73575898	0.760535094	-0.024776114	FDR0.01	1	51986130	TSS1500	-1135	***EPS15***	***EPS15***
cg11823159	2.18E-07	0.001737673	0.747280821	0.795251891	-0.04797107	FDR0.01	2	128611859	Body	3870	***POLR2D***	***POLR2D***
cg26529864	2.22E-07	0.001740417	0.897249355	0.914070341	-0.016820986	FDR0.01	2	74375903	TSS1500;Body;TES5000;Body;Body;TES5000;TES5000;TES5000;TSS1500	796	***BOLA3-AS1***	***BOLA3;BOLA3-AS1;MOB1A***
cg17246318	2.40E-07	0.00184746	0.891838918	0.907330983	-0.015492065	FDR0.01	15	99795399	Body;Body;Body;TSS5000;Body;Body;Body;Body;Body;Body;Body;Body;Body;Body;Body;TSS5000;Body;TSS5000	-2330	***HSP90B2P***	***LRRC28;TTC23;HSP90B2P***
cg26252958	2.47E-07	0.001871909	0.717909068	0.732076429	-0.014167361	FDR0.01	6	105847870	Body	3129	***PREP***	***PREP***
cg19546781	2.77E-07	0.00201811	0.804702803	0.810545497	-0.005842694	FDR0.01	16	57438715	5'UTR	37	***CCL17***	***CCL17***
cg27614038	2.87E-07	0.00201811	0.026994005	0.021931757	0.005062248	FDR0.01	3	28390080	TSS1500;TSS1500;Body;TSS1500;Body;Body;Body;TSS1500	538	***AZI2***	***ZCWPW2;AZI2***
cg15081033	2.93E-07	0.00201811	0.811693381	0.844327279	-0.032633898	FDR0.01	3	140805567	Body	35324	***SPSB4***	***SPSB4***
cg02900980	3.00E-07	0.00201811	0.034188387	0.030382747	0.00380564	FDR0.01	3	71633249	TSS1500;TSS1500;TSS1500;TSS1500;TSS1500;TSS200;TSS200;TSS1500;TSS200	-109	***FOXP1***	***FOXP1***
cg26369642	3.05E-07	0.00201811	0.827804466	0.844078133	-0.016273667	FDR0.01	8	58987333	Body	80221	***FAM110B***	***FAM110B***
cg02518895	3.05E-07	0.00201811	0.821776034	0.829695584	-0.00791955	FDR0.01	2	99918603	TSS1500	-964	***LYG1***	***LYG1***
cg10001646	3.10E-07	0.00201811	0.768730129	0.764253167	0.004476962	FDR0.01	14	24683737	Body;Body;TES5000;Body;Body;Body;TSS1500;Body;Body	-701	***CHMP4A***	***MDP1;NEDD8;NEDD8-MDP1;CHMP4A***
cg12632411	3.14E-07	0.00201811	0.908106177	0.920870153	-0.012763976	FDR0.01	20	17595355	Body;Body;Body	44757	***DSTN***	***RRBP1***
cg21245975	3.15E-07	0.00201811	0.073983909	0.073386803	0.000597106	FDR0.01	1	85725523	TSS200;TSS200	-168	***C1orf52***	***C1orf52***
cg16576694	3.21E-07	0.00201811	0.910700623	0.93265643	-0.021955807	FDR0.01	14	106071815	TSS5000;TSS5000;TSS200;TSS5000	-121	***MIR8071-1***	***IGHE;RP11-731F5.1***
cg09936218	3.23E-07	0.00201811	0.807181584	0.824840507	-0.017658923	FDR0.01	7	38331871	TSS200	-192	***TARP***	***TRGV11***
cg03181382	3.26E-07	0.00201811	0.86208283	0.865241337	-0.003158507	FDR0.01	2	131839563	Body;Body;Body;Body;Body;Body;Body	10968	***FAM168B***	***FAM168B***
cg19305527	3.29E-07	0.00201811	0.714835202	0.782380855	-0.067545653	FDR0.01	2	225434838	Body;TSS1500;Body	-296	***CUL3***	***CUL3***
cg16762386	3.32E-07	0.00201811	0.860804793	0.857264094	0.003540699	FDR0.01	1	27240514	5'UTR	53	***NR0B2***	***NR0B2***
cg03369382	3.37E-07	0.00201811	0.842932223	0.856394029	-0.013461806	FDR0.01	7	139427635	Body;Body	50058	***HIPK2***	***HIPK2***
cg23018181	3.38E-07	0.00201811	0.817920756	0.828183634	-0.010262878	FDR0.01	12	45581910	Body;TSS1500	-658	***RNY5***	***PLEKHA8P1;RNY5***
cg23352695	3.46E-07	0.002036745	0.204678659	0.158206569	0.04647209	FDR0.01	17	29648811	TSS200;Body;TSS200;Body	-44	***EVI2A***	***EVI2A;NF1***
cg03526652	3.53E-07	0.002051141	0.757732757	0.798520693	-0.040787936	FDR0.01	2	72977657	Body;Body;Body;Body;Body;Body;Body;Body	75514	***EXOC6B***	***EXOC6B***
cg10513984	3.74E-07	0.002144603	0.043619646	0.03921046	0.004409186	FDR0.01	3	51534217	TSS200;TSS200;TSS200;TSS200;TSS200;TSS200;TSS200	-199	***DCAF1***	***DCAF1***
cg08770358	3.82E-07	0.002162732	0.758664451	0.780711997	-0.022047546	FDR0.01	13	48876684	Body;TSS1500	1113	***RB1-DT***	***RB1-DT;RB1***
cg04437845	3.87E-07	0.002165436	0.827924504	0.838719963	-0.010795459	FDR0.01	2	47291703	Body;Body;Body;Body	90814	***STPG4***	***TTC7A***
cg16109381	3.93E-07	0.002170237	0.767501708	0.79341722	-0.025915512	FDR0.01	15	34339800	Body;Body	-8497	***AVEN***	***CHRM5***
cg04173154	4.06E-07	0.002215326	0.85959834	0.858760801	0.000837539	FDR0.01	16	66257231		-78480	***RNA5SP428***	***CDH5***
cg04933530	4.43E-07	0.002386597	0.731371916	0.740000288	-0.008628373	FDR0.01	14	77419754		-6226	***LINC01629***	
cg22889620	4.48E-07	0.002386597	0.875180059	0.865486346	0.009693713	FDR0.01	14	75639473	Body	3876	***TMED10***	***TMED10***
cg18254364	4.96E-07	0.002611754	0.790508922	0.793965816	-0.003456893	FDR0.01	14	24741821	TSS1500;TSS1500	-988	***RABGGTA***	***RABGGTA***
cg23392390	5.05E-07	0.002624127	0.842210242	0.829304435	0.012905807	FDR0.01	17	19649191	Body;Body;TSS1500;TSS1500	-266	***ALDH3A1***	***ALDH3A1***
cg27223827	5.17E-07	0.002660043	0.649855996	0.645608609	0.004247387	FDR0.01	2	106054920	5'UTR;5'UTR	310	***FHL2***	***FHL2***
cg10420310	5.35E-07	0.002691048	0.101983501	0.088404862	0.013578639	FDR0.01	16	14397576	Body;Body;TSS1500	-247	***MIR193B***	***MIR193BHG;MIR193B***
cg14818960	5.36E-07	0.002691048	0.769424048	0.806925207	-0.037501158	FDR0.01	11	71903119	Body;Body;TSS200;Body	-53	***FOLR1***	***FOLR1***
cg03552327	5.69E-07	0.002812426	0.033188577	0.028730392	0.004458184	FDR0.01	12	50222455	Body;TSS1500;Body;Body	130	***BCDIN3D-AS1***	***BCDIN3D-AS1;NCKAP5L***
cg20716812	5.72E-07	0.002812426	0.798219599	0.822630086	-0.024410487	FDR0.01	6	39267536	Body;Body	14700	***KCNK17***	***KCNK17***
cg20566475	6.15E-07	0.002977973	0.648780126	0.633247014	0.015533112	FDR0.01	10	126278272	Body;Body;Body	-114324	***FAM53B-AS1***	***LHPP***
cg08775835	6.19E-07	0.002977973	0.875641512	0.910431462	-0.03478995	FDR0.01	16	31147044	TES5000;TES5000;5'UTR;TES5000;TES5000;TES5000	39	***PRSS8***	***KAT8;PRSS36;PRSS8***
cg19595760	6.40E-07	0.00304295	0.427161673	0.426762813	0.00039886	FDR0.01	1	25948511	Body;Body	4553	***MAN1C1***	***MAN1C1***
cg14837165	6.55E-07	0.003083743	0.051194805	0.044708692	0.006486113	FDR0.01	15	40763995	TES5000;1stExon;TES5000;TES5000	836	***CHST14***	***BAHD1;CHST14***
cg02856606	6.67E-07	0.003095258	0.029839903	0.025535771	0.004304132	FDR0.01	1	19282682	1stExon	144	***IFFO2***	***IFFO2***
cg25399998	6.71E-07	0.003095258	0.838611573	0.842977483	-0.004365909	FDR0.01	5	170217126	Body;Body	6404	***GABRP***	***GABRP***
cg02193425	6.91E-07	0.00315514	0.857919013	0.868742385	-0.010823372	FDR0.01	6	3848280	TSS1500	-1319	***FAM50B***	***FAM50B***
cg16465882	7.12E-07	0.003218271	0.916106235	0.936009727	-0.019903491	FDR0.01	12	113633320	TES5000;TES5000;3'UTR;3'UTR	9990	***RITA1***	***RITA1;IQCD***
cg22935609	7.32E-07	0.003253162	0.8445875	0.848692401	-0.0041049	FDR0.01	6	55989097	Body;Body;Body;Body;Body;Body	-32638	***COL21A1***	***COL21A1***
cg16246188	7.35E-07	0.003253162	0.923863664	0.911182823	0.01268084	FDR0.01	11	113947203	Body;Body;Body;Body;Body	13271	***ZBTB16***	***ZBTB16***
cg19554281	7.55E-07	0.003312249	0.834625787	0.847668055	-0.013042268	FDR0.01	15	39726997		-7601	***RP11-624L4.1***	***THBS1***
cg05315276	7.79E-07	0.003321243	0.761736538	0.808744443	-0.047007906	FDR0.01	14	52981335	Body;Body	37966	***TXNDC16***	***TXNDC16***
cg00247020	7.79E-07	0.003321243	0.050948135	0.044432349	0.006515786	FDR0.01	3	47844655	5'UTR;5'UTR;Body;5'UTR	257	***DHX30***	***DHX30***
cg17514088	7.85E-07	0.003321243	0.849505753	0.853537513	-0.00403176	FDR0.01	17	76503074	Body;TES5000	22083	***DNAH17-AS1***	***DNAH17;DNAH17-AS1***
cg00705661	7.87E-07	0.003321243	0.968182546	0.964322698	0.003859849	FDR0.01	14	93706609	3'UTR;3'UTR	-33150	***GON7***	***BTBD7;UBR7***
cg07274880	8.24E-07	0.003446125	0.615539098	0.650348158	-0.03480906	FDR0.01	12	7373811	TES5000	30854	***PEX5***	***PEX5***
cg16695253	8.50E-07	0.003520522	0.774049216	0.798731632	-0.024682416	FDR0.01	16	88079762	Body;Body;Body;Body;Body;Body;Body	54829	***LOC400553***	***BANP***
cg25838060	8.61E-07	0.003532107	0.886672729	0.929242334	-0.042569605	FDR0.01	7	139932729		-8940	***RNU1-58P***	***KDM7A-DT***
cg24267275	8.73E-07	0.00354871	0.770780396	0.788121407	-0.017341011	FDR0.01	2	88958370		30682	***AC062029.1***	***EIF2AK3-DT***
cg12562418	9.12E-07	0.003674933	0.902494733	0.91525046	-0.012755727	FDR0.01	7	150039392	TES5000;TES5000;TES5000;TSS1500;TES5000;TES5000	-629	***RARRES2***	***LRRC61;RARRES2***
cg09136597	9.30E-07	0.003713745	0.813500966	0.83759702	-0.024096054	FDR0.01	1	5946381	Body;Body;Body;Body	-23580	***MIR4689***	***NPHP4***

#cpg	p_adj	q_val	Meth.BPD	Meth.nonBPD	dMeth	significance	Chr	pos.hg19	locations	dist2TSS	gene.nearest	gene.Illumina
cg00422311	9.45E-07	0.003739985	0.771652317	0.787471637	-0.015819319	FDR0.01	2	161395263		-44945	***RBMS1***	
cg17147466	9.56E-07	0.003751192	0.083171787	0.079508409	0.003663377	FDR0.01	15	93353422	TSS1500;TSS1500;TSS5000;TES5000;TSS1500	-308	***FAM174B***	***FAM174B;CTD-2313J17.1;CTD-2313J17.6;ASB9P1***
cg21406409	9.70E-07	0.003764842	0.78311666	0.790835653	-0.007718992	FDR0.01	1	10413700	Body;Body;Body	-45348	***PGD***	***KIF1B***
cg26333751	9.83E-07	0.003764842	0.195260478	0.157202504	0.038057974	FDR0.01	12	56323079	TSS5000;TSS5000;TSS5000;TSS1500;TSS5000;TSS5000;TSS5000;TSS5000;TSS5000;TSS5000;TSS5000;TSS5000;TSS5000;TSS5000	-1382	***PYM1***	***DGKA;PYM1***
cg15659537	9.85E-07	0.003764842	0.837735136	0.853534858	-0.015799722	FDR0.01	12	75712028	Body;Body;Body;Body;Body;Body;Body;Body;Body;Body;Body;Body;Body;Body;Body;Body;Body;Body;Body	12084	***CAPS2***	***CAPS2***
cg05605603	1.01E-06	0.003829318	0.828849109	0.834214238	-0.00536513	FDR0.01	11	35602594	Body	4264	***FJX1***	***RP5-945I17.2***
cg19554797	1.05E-06	0.003928101	0.819576297	0.828778532	-0.009202235	FDR0.01	5	111494267	Body;TES5000;TES5000;TSS5000;TSS5000	-1955	***EPB41L4A-AS1***	***EPB41L4A;EPB41L4A-AS1;SNORA13***
cg22849526	1.06E-06	0.00395918	0.814687745	0.817942725	-0.003254981	FDR0.01	7	6199437	TES5000;Body;TES5000	54858	***USP42***	***CYTH3;USP42***
cg03998655	1.09E-06	0.004024315	0.790200195	0.825924497	-0.035724303	FDR0.01	16	1424607	Body;Body;Body	5077	***UNKL***	***UNKL***
cg20967329	1.12E-06	0.004089622	0.802587832	0.837391314	-0.034803481	FDR0.01	1	211558942	Body	2846	***LINC00467***	***LINC00467***
cg14227558	1.13E-06	0.004089622	0.275649649	0.17939172	0.096257929	FDR0.01	2	213983851	Body;Body	31207	***IKZF2***	***IKZF2***
cg05470179	1.15E-06	0.004089622	0.681249146	0.69153822	-0.010289074	FDR0.01	4	1578163	Body	3817	***FAM53A***	***RP11-1398P2.1***
cg15711521	1.15E-06	0.004089622	0.776833387	0.767132045	0.009701342	FDR0.01	3	167767619	Body;Body	46094	***GOLIM4***	***GOLIM4***
cg26103369	1.15E-06	0.004089622	0.719340862	0.785463061	-0.066122199	FDR0.01	14	75153307	Body	26500	***AREL1***	***AREL1***
cg01624238	1.16E-06	0.004096274	0.505764132	0.580180162	-0.07441603	FDR0.01	19	54269320	TES5000;TES5000	3723	***MIR519A2***	***MIR519A2;MIR516A2***
cg15816742	1.20E-06	0.004192655	0.854715431	0.892810412	-0.038094981	FDR0.01	19	50643548	TSS1500;TSS1500;Body	29	***SNAR-D***	***SNAR-B1;SNAR-B2;SNAR-D***
cg09499787	1.22E-06	0.004227694	0.165239406	0.110760552	0.054478855	FDR0.01	10	118900492	TSS5000;TSS5000	-2680	***VAX1***	***VAX1***
cg02060627	1.27E-06	0.004322378	0.668457966	0.669305346	-0.00084738	FDR0.01	1	22378412	TSS1500;TSS1500;TSS1500	-707	***CDC42***	***CDC42***
cg17314180	1.29E-06	0.004322378	0.821827959	0.854127668	-0.03229971	FDR0.01	2	209011053	TSS200;Body	-176	***CRYGB***	***CRYGB;LOC100507443***
cg19279529	1.29E-06	0.004322378	0.042158013	0.035289289	0.006868724	FDR0.01	19	35760554	Body;TES5000;TES5000;TES5000;Body;Body;TES5000;TES5000	674	***USF2***	***USF2;LSR***
cg13907754	1.29E-06	0.004322378	0.82592421	0.851751309	-0.025827099	FDR0.01	6	90117574	Body	4421	***RRAGD***	***RRAGD***
cg26873132	1.30E-06	0.004322378	0.810633156	0.841817922	-0.031184766	FDR0.01	11	9191490	Body;Body;Body;Body;Body	75581	***KRT8P41***	***DENND5A***
cg12710648	1.30E-06	0.004322378	0.067736628	0.055308957	0.012427671	FDR0.01	1	7308091	Body;Body;Body;Body;Body	141723	***LOC102725193***	***CAMTA1;CAMTA1-AS2***
cg11277768	1.33E-06	0.004368711	0.824735531	0.822816102	0.001919428	FDR0.01	7	141490056	5'UTR	40	***TAS2R5***	***TAS2R5***
cg20713968	1.37E-06	0.004460195	0.692196265	0.75150995	-0.059313684	FDR0.01	8	1737564	TES5000	-11726	***MIR3674***	***CLN8***
cg05325785	1.38E-06	0.004477573	0.747690274	0.806365038	-0.058674765	FDR0.01	14	103827144		19838	***EIF5***	***SNORA28***
cg15390960	1.39E-06	0.004477573	0.061220808	0.049445298	0.011775511	FDR0.01	13	36921096	TSS1500;Body;TSS1500;TSS200;Body;Body	-162	***SPART***	***SPART;SPART-AS1***
cg21166985	1.49E-06	0.004772251	0.919549492	0.929589031	-0.010039539	FDR0.01	12	121882267	TSS200;Body;Body	-112	***MIR7107***	***MIR7107;KDM2B***
cg09597192	1.53E-06	0.004843124	0.772595688	0.778767617	-0.006171929	FDR0.01	6	32141591	Body;Body;TSS5000;TSS5000;TSS5000	2325	***AGPAT1***	***AGPAT1;RNF5;MIR6721;RNF5P1***
cg08279008	1.56E-06	0.004909744	0.108129896	0.094091986	0.014037909	FDR0.01	2	86263224	Body	70054	***POLR1A***	***POLR1A***
cg07816786	1.61E-06	0.005047488	0.049381346	0.044146921	0.005234425	FDR0.01	19	37063800	TSS200;Body;Body;Body;Body;Body;TSS200;TSS200;TSS200;Body	-171	***ZNF529-AS1***	***ZNF529-AS1;ZNF529***
cg01713086	1.67E-06	0.005177151	0.805823755	0.847790461	-0.041966706	FDR0.01	8	28268413	Body;TES5000	-5551	***ZNF395***	***FBXO16;RP11-181B11.1;RNU6-178P***
cg22838882	1.68E-06	0.005177151	0.811381999	0.827749657	-0.016367658	FDR0.01	3	138952118	Body	246	***PISRT1***	***PISRT1***
cg15777769	1.69E-06	0.005177151	0.721003275	0.757919081	-0.036915806	FDR0.01	7	38423649	3'UTR;3'UTR	42472	***TRG-AS1***	***AMPH***
cg26153483	1.73E-06	0.005260702	0.806345173	0.843636659	-0.037291486	FDR0.01	2	24838522	Body;Body;Body;Body;Body;Body;Body	31177	***NCOA1***	***NCOA1***
cg21614638	1.74E-06	0.005262489	0.145556358	0.123009735	0.022546623	FDR0.01	4	100737821	TSS200;TSS200	-159	***DAPP1***	***DAPP1***
cg17891928	1.75E-06	0.005262489	0.76119509	0.805093229	-0.043898138	FDR0.01	1	76968506	Body;Body;Body;Body;Body;Body;Body;Body;Body;Body	276446	***LINC02567***	***ST6GALNAC3***
cg03247301	1.82E-06	0.005435716	0.808600085	0.810873163	-0.002273078	FDR0.01	2	59058823	Body	310936	***LINC01122***	***LINC01122***
cg25596783	1.86E-06	0.00551758	0.812701201	0.852180105	-0.039478904	FDR0.01	13	80715583		-66891	***RP11-470M1.2***	***LINC01080***
cg05159290	1.90E-06	0.005562404	0.873808399	0.856676864	0.017131536	FDR0.01	12	130934389	Body;Body;Body;Body;Body;Body;Body;Body;Body	111957	***PIWIL1***	***RIMBP2***
cg15161251	1.90E-06	0.005562404	0.872827264	0.868241184	0.00458608	FDR0.01	19	1305252	TES5000	19100	***EFNA2***	***EFNA2***
cg17003376	1.92E-06	0.005581672	0.836220022	0.862571787	-0.026351765	FDR0.01	11	123613212	TSS1500;TSS1500;TSS1500	-821	***ZNF202***	***ZNF202***
cg06087554	1.99E-06	0.005753761	0.786969135	0.802846367	-0.015877231	FDR0.01	16	31151917	Body;TSS5000;Body;Body	-4834	***PRSS8***	***PRSS36;PRSS8***
cg22161983	2.03E-06	0.005767103	0.030349631	0.025048948	0.005300683	FDR0.01	8	103818636	TSS1500	-413	***GASAL1***	***GASAL1***
cg17687789	2.03E-06	0.005767103	0.710520231	0.781789198	-0.071268966	FDR0.01	3	40543613	TSS5000;TSS5000;TSS5000	-3916	***ZNF620***	***ZNF620***
cg21155609	2.05E-06	0.005767103	0.848479666	0.875453085	-0.026973418	FDR0.01	1	32713275	TSS5000;TSS5000;1stExon	458	***FAM167B***	***LCK;FAM167B***
cg13968233	2.05E-06	0.005767103	0.82821547	0.837953712	-0.009738242	FDR0.01	1	197564458	Body;Body;Body;Body	180165	***DENND1B***	***DENND1B***
cg17805540	2.06E-06	0.005767103	0.075184944	0.056855848	0.018329096	FDR0.01	4	13543730	Body;TES5000	2384	***NKX3-2***	***NKX3-2;LINC01096***
cg19862344	2.10E-06	0.005818586	0.7177455	0.768322836	-0.050577336	FDR0.01	6	49605181	TSS1500	-594	***RHAG***	***RHAG***
cg16016960	2.11E-06	0.005818586	0.749545112	0.804139231	-0.054594119	FDR0.01	8	98883226	Body;Body;Body	1978	***MATN2***	***MATN2***
cg02516419	2.15E-06	0.005891034	0.820725848	0.837976092	-0.017250244	FDR0.01	12	47771422		-13370	***MIR4494***	
cg10937973	2.20E-06	0.005991085	0.738065338	0.73871401	-0.000648672	FDR0.01	2	122313888	Body;Body;Body;Body	25433	***RNU4ATAC***	***CLASP1***
cg02327812	2.22E-06	0.006004287	0.931571129	0.9533669	-0.021795771	FDR0.01	5	1218507	Body	-6962	***SLC6A18***	***SLC6A19***
cg19364141	2.23E-06	0.006006323	0.877484819	0.890283284	-0.012798465	FDR0.01	2	63846679	Body;Body	13736	***MDH1***	***WDPCP***
cg17891251	2.34E-06	0.0062616	0.803984204	0.791812032	0.012172172	FDR0.01	1	57286265	TSS1500	-896	***FYB2***	***FYB2***
cg20257096	2.35E-06	0.0062616	0.026755356	0.022709775	0.004045581	FDR0.01	14	21851954	TES5000;TES5000;Body	471	***SUPT16H***	***CHD8;SUPT16H***

#cpg	p_adj	q_val	Meth.BPD	Meth.nonBPD	dMeth	significance	Chr	pos.hg19	locations	dist2TSS	gene.nearest	gene.Illumina
cg12476579	2.38E-06	0.00629329	0.634192869	0.648530101	-0.014337232	FDR0.01	5	169172387	Body;Body	108109	***DOCK2***	***DOCK2***
cg21075870	2.42E-06	0.006343737	0.039378771	0.03663084	0.00274793	FDR0.01	1	155715476	TES5000;TES5000;TES5000;TSS200	-82	***MSTO2P***	***GON4L;MSTO2P***
cg16529676	2.43E-06	0.006343737	0.681891086	0.677260321	0.004630765	FDR0.01	3	169470001		12847	***TERC***	
cg01124843	2.50E-06	0.00643152	0.039686256	0.02536062	0.014325636	FDR0.01	16	2653626	TES5000;TES5000;TES5000;Body	242	***LOC652276***	***PDPK1;LOC652276***
cg18783781	2.50E-06	0.00643152	0.62089146	0.6681062	-0.04721474	FDR0.01	1	9599067	TSS1500	-460	***SLC25A33***	***SLC25A33***
cg20960181	2.51E-06	0.00643152	0.753409642	0.785389013	-0.031979371	FDR0.01	1	86094825		30012	***CCN1***	***RP11-290M5.2***
cg21075986	2.53E-06	0.00643152	0.741776658	0.73820119	0.003575468	FDR0.01	14	106666649	TES5000;TES5000;TES5000	1446	***IGHV3-20***	***AB019440.50;AB019440.1;IGHV3-20;LINC00226***
cg10580335	2.55E-06	0.00643152	0.726171977	0.764849527	-0.03867755	FDR0.01	3	125803859	Body;TSS1500;TSS1500;TSS1500	-725	***SLC41A3***	***SLC41A3***
cg04467611	2.55E-06	0.00643152	0.059469702	0.053458189	0.006011512	FDR0.01	11	64808285	TES5000;TES5000;TSS200;TES5000;TES5000	-90	***SAC3D1***	***NAALADL1;SNX15;SAC3D1;ARL2-SNX15;ARL2-SNX15***
cg09314766	2.62E-06	0.006575964	0.86050728	0.848855959	0.01165132	FDR0.01	8	39695697	1stExon;1stExon;1stExon	111	***ADAM2***	***ADAM2***
cg21954542	2.70E-06	0.006749622	0.792519875	0.812630377	-0.020110502	FDR0.01	17	78113146	Body	7781	***EIF4A3***	***EIF4A3***
cg15625675	2.80E-06	0.006867134	0.86313908	0.882634305	-0.019495225	FDR0.01	5	114957464	TES5000;TES5000;Body;Body;Body	4412	***TMED7***	***LOC101927100;TMED7;TMED7-TICAM2***
cg00764217	2.81E-06	0.006867134	0.783875999	0.803114557	-0.019238558	FDR0.01	7	116660240	TSS200;Body;Body	-24	***MIR6132***	***MIR6132;ST7***
cg05064181	2.83E-06	0.006867134	0.764126908	0.789853637	-0.025726729	FDR0.01	10	116445398	Body;TSS1500;TSS1500;Body;TSS1500;Body;TSS1500	-636	***ABLIM1***	***ABLIM1***
cg03467047	2.86E-06	0.006867134	0.938118246	0.952587785	-0.01446954	FDR0.01	3	118864763	5'UTR;5'UTR;5'UTR;TSS1500	152	***IGSF11***	***IGSF11;C3orf30;TEX55***
cg14605274	2.86E-06	0.006867134	0.742095486	0.787791884	-0.045696398	FDR0.01	2	169750924	TSS5000	-3980	***SPC25***	***SPC25***
cg07282201	2.87E-06	0.006867134	0.162974122	0.155594933	0.007379189	FDR0.01	11	67177296	3'UTR;3'UTR;Body	5850	***TBC1D10C***	***TBC1D10C***
cg25658010	2.88E-06	0.006867134	0.795186822	0.807238243	-0.012051421	FDR0.01	10	112455412	Body	51258	***RBM20***	***RBM20***
cg08321684	2.90E-06	0.006867134	0.731768836	0.753826106	-0.022057269	FDR0.01	6	32150404	Body;TES5000;Body;TSS5000;Body;Body;Body;Body;Body;TES5000;TES5000;Body;Body;Body;TES5000	1695	***AGER***	***AGER;MIR6833;AGPAT1;RNF5;PBX2;RNF5P1***
cg11791427	2.90E-06	0.006867134	0.045910927	0.056679469	-0.010768542	FDR0.01	7	73704194	Body;Body	390	***CLIP2***	***CLIP2***
cg01829002	2.90E-06	0.006867134	0.041320558	0.031697456	0.009623102	FDR0.01	5	50259133	TES5000	3709	***CTD-2089N3.1***	***CTD-2089N3.1;LINC02106***
cg20955688	2.98E-06	0.007013842	0.167106555	0.163767229	0.003339326	FDR0.01	8	133772889	5'UTR;5'UTR;TSS200	25	***TMEM71***	***TMEM71***
cg22966986	3.04E-06	0.007107394	0.070020989	0.05705242	0.012968568	FDR0.01	14	48143852	5'UTR	305	***MDGA2***	***MDGA2***
cg00301033	3.13E-06	0.007290695	0.77248214	0.761414933	0.011067207	FDR0.01	11	43390036	Body;Body	9602	***TTC17***	***TTC17***
cg23753021	3.18E-06	0.007364289	0.06344214	0.057877094	0.005565046	FDR0.01	10	89623142	TSS200;TSS200;Body;TSS200	-52	***PTEN***	***PTEN;KLLN***
cg20569893	3.26E-06	0.00745532	0.240391089	0.221484477	0.018906612	FDR0.01	17	27220101	TES5000;Body;Body	4614	***FLOT2***	***DHRS13;FLOT2***
cg15491950	3.27E-06	0.00745532	0.757662769	0.793520196	-0.035857427	FDR0.01	3	132004600	TSS1500	-346	***ACP3***	***CPNE4***
cg13488877	3.29E-06	0.00745532	0.735626459	0.751301619	-0.01567516	FDR0.01	12	45581327	Body;TSS200	-75	***RNY5***	***PLEKHA8P1;RNY5***
cg05894719	3.30E-06	0.00745532	0.730467657	0.746998081	-0.016530425	FDR0.01	11	1989899	Body;Body	13093	***SNORD131***	***MRPL23***
cg13112525	3.30E-06	0.00745532	0.882568289	0.892766791	-0.010198502	FDR0.01	2	70187740	TES5000;3'UTR	1657	***ASPRV1***	***PCBP1-AS1;ASPRV1***
cg01816186	3.34E-06	0.007462409	0.785644519	0.782452919	0.0031916	FDR0.01	2	15310191	Body;Body	391281	***NBAS***	***NBAS***
cg23693293	3.36E-06	0.007462409	0.782684432	0.813317587	-0.030633156	FDR0.01	17	41194642	TES5000;TES5000;TES5000;TES5000;TES5000;TES5000	17385	***RND2***	***BRCA1***
cg15451045	3.36E-06	0.007462409	0.697982003	0.697851571	0.000130432	FDR0.01	19	6529614	TSS1500	-1395	***TNFSF9***	***TNFSF9***
cg14176638	3.37E-06	0.007462409	0.854267082	0.869239061	-0.014971979	FDR0.01	9	99061672	Body	2762	***HSD17B3***	***HSD17B3***
cg27488674	3.39E-06	0.007476521	0.732684248	0.780115023	-0.047430775	FDR0.01	17	34134865	TSS5000;TSS5000	-1593	***TAF15***	***TAF15***
cg07005513	3.41E-06	0.007481926	0.056883299	0.052893175	0.003990124	FDR0.01	11	61595956	Body;Body;1stExon	452	***FADS2***	***FADS2***
cg00386007	3.48E-06	0.007596351	0.860831598	0.867715262	-0.006883664	FDR0.01	10	50143337	Body	-21057	***LRRC18***	***WDFY4***
cg07410597	3.57E-06	0.007732029	0.789912542	0.802975164	-0.013062622	FDR0.01	3	66404129	Body;Body;Body;Body;Body;Body	132702	***SLC25A26***	***SLC25A26***
cg01303385	3.58E-06	0.007732029	0.864045165	0.871234569	-0.007189404	FDR0.01	3	111451470	Body;Body;5'UTR	144	***PHLDB2***	***PLCXD2;PHLDB2***
cg15851800	3.69E-06	0.007941349	0.050277575	0.043617166	0.006660409	FDR0.01	5	126853405	5'UTR;5'UTR	105	***PRRC1***	***PRRC1***
cg12099546	3.71E-06	0.007941349	0.710581101	0.742266725	-0.031685623	FDR0.01	15	72763849	Body;TSS5000	2731	***TMEM202-AS1***	***TMEM202-AS1;ARIH1***
cg02753404	3.81E-06	0.008098519	0.854628179	0.856657242	-0.002029063	FDR0.01	15	95258951		115709	***RP11-57P19.1***	***LOC440311***
cg27036740	3.83E-06	0.008098519	0.737263565	0.75648518	-0.019221615	FDR0.01	12	49086943	Body;3'UTR	-10908	***KANSL2***	***CCNT1***
cg01949002	3.84E-06	0.008098519	0.56727849	0.61008426	-0.04280577	FDR0.01	1	2203589	Body	43456	***SKI***	***SKI***
cg02236679	3.95E-06	0.008205457	0.803245182	0.818862477	-0.015617295	FDR0.01	16	78782477	Body;Body	649168	***WWOX***	***WWOX***
cg24768137	3.95E-06	0.008205457	0.917806021	0.922407336	-0.004601315	FDR0.01	2	242776518		-10013	***AC114730.2***	***NEU4***
cg26477169	3.96E-06	0.008205457	0.69360289	0.760109267	-0.066506377	FDR0.01	17	75196864	Body;Body;Body;Body;Body;Body;Body	15570	***SEC14L1***	***SEC14L1***
cg18688062	3.96E-06	0.008205457	0.705647026	0.708273519	-0.002626494	FDR0.01	6	31146471	Body;Body;Body;Body;Body;Body;Body;Body;Body;Body;Body;Body;Body	7623	***PSORS1C3***	***PSORS1C3***
cg20735050	4.05E-06	0.008341987	0.809123962	0.831331074	-0.022207112	FDR0.01	11	94521117	Body;Body	19610	***AMOTL1***	***AMOTL1***
cg10803923	4.11E-06	0.008434914	0.745204055	0.762712585	-0.01750853	FDR0.01	12	56637310	Body	12810	***SLC39A5***	***ANKRD52***
cg16523801	4.15E-06	0.008453334	0.85285081	0.855492102	-0.002641291	FDR0.01	16	56448618	Body;Body;Body	10289	***AMFR***	***AMFR***
cg10416963	4.16E-06	0.008453334	0.062008538	0.044048964	0.017959574	FDR0.01	4	121844401	TSS1500;TSS1500;TSS1500	-380	***PRDM5***	***PRDM5***
cg13304609	4.18E-06	0.008453334	0.775895282	0.822630498	-0.046735216	FDR0.01	1	79085162	TSS1500	-904	***IFI44L***	***IFI44L***
cg00642752	4.20E-06	0.008453334	0.815074875	0.802636281	0.012438594	FDR0.01	6	26036687	TSS5000;TSS5000	-2891	***HIST1H2AB***	***HIST1H2AB;HIST1H3B;H3C2;H2AC4***
cg12621315	4.22E-06	0.00846499	0.215564494	0.208053829	0.007510664	FDR0.01	2	129080120	TSS5000	-3949	***HS6ST1***	***HS6ST1***
cg26454662	4.25E-06	0.008495615	0.829229226	0.804805957	0.02442327	FDR0.01	11	47470803	TSS200;TSS200	-73	***RAPSN***	***RAPSN***

#cpg	p_adj	q_val	Meth.BPD	Meth.nonBPD	dMeth	significance	Chr	pos.hg19	locations	dist2TSS	gene.nearest	gene.Illumina
cg19708418	4.30E-06	0.008526654	0.766598696	0.804756014	-0.038157318	FDR0.01	15	101820662	TES5000;TES5000;TES5000;TES5000;TSS5000;TSS5000	-2937	***SELENOS***	***SNRPA1;SELENOS***
cg23435915	4.32E-06	0.008526654	0.855289428	0.842580657	0.012708771	FDR0.01	5	43044640	Body;TSS5000;Body	2405	***LOC153684***	***LOC648987;ANXA2R;LOC153684***
cg13165140	4.33E-06	0.008526654	0.126557179	0.117150711	0.009406467	FDR0.01	6	32813522	TSS1500;Body;Body;Body;Body;TSS5000;Body;Body	-810	***PSMB8***	***PSMB8;TAP1;PSMB8-AS1***
cg17157894	4.35E-06	0.008526654	0.202803293	0.168211317	0.034591976	FDR0.01	22	47187343	Body;Body;Body;Body;Body	17520	***TBC1D22A***	***TBC1D22A***
cg19886853	4.46E-06	0.008719852	0.038194743	0.043561569	-0.005366826	FDR0.01	1	8277746	TES5000	9015	***LINC01714***	***LINC01714***
cg02914087	4.49E-06	0.00872835	0.042654151	0.038668445	0.003985706	FDR0.01	19	12267676	TSS200;TSS200;TSS200	-130	***ZNF625-ZNF20***	***ZNF625;ZNF625-ZNF20***
cg13581922	4.63E-06	0.008925341	0.119496022	0.112305055	0.007190968	FDR0.01	12	124938116	Body;Body;Body	113894	***NCOR2***	***NCOR2***
cg17067226	4.63E-06	0.008925341	0.804424131	0.83040551	-0.025981379	FDR0.01	3	157211395	Body;Body;Body;Body;Body;Body	1900	***VEPH1***	***VEPH1***
cg27651452	4.65E-06	0.00893177	0.85972354	0.840475692	0.019247848	FDR0.01	11	64035061	TES5000;3'UTR;Body;TES5000;3'UTR	16067	***PLCB3***	***BAD;PLCB3***
cg18210128	4.70E-06	0.008968692	0.752796929	0.803076028	-0.050279099	FDR0.01	12	92396747	Body;Body;Body;Body;Body	138742	***LINC01619***	***LINC01619***
cg26571889	4.73E-06	0.008968692	0.700490658	0.71775651	-0.017265852	FDR0.01	4	170950517	TSS5000;TES5000;TSS5000	-2936	***MFAP3L***	***MFAP3L***
cg00728848	4.81E-06	0.008968692	0.74065143	0.739714813	0.000936617	FDR0.01	6	13483832	Body;Body;Body	2583	***GFOD1***	***GFOD1***
cg06564523	4.82E-06	0.008968692	0.026148597	0.02409085	0.002057747	FDR0.01	12	115174628	Body;TSS1500	-880	***TBX3***	***RP4-601P9.2;SNORA27***
cg02659532	4.85E-06	0.008968692	0.827367037	0.81436177	0.013005268	FDR0.01	11	1991875	Body;Body	15069	***MRPL23***	***MRPL23;MRPL23-AS1***
cg18145090	4.85E-06	0.008968692	0.770150281	0.746425041	0.02372524	FDR0.01	13	20805094	5'UTR;Body;Body;Body	278	***GJB6***	***GJB6***
cg04915974	4.87E-06	0.008968692	0.78230376	0.775386836	0.006916924	FDR0.01	16	29205675	Body;Body	-10278	***RP11-426C22.1***	***RP11-426C22.12;RP11-426C22.5;SNX29P2***
cg24736010	4.88E-06	0.008968692	0.866157761	0.878525494	-0.012367733	FDR0.01	11	109941265	Body	-22821	***ZC3H12C***	***RDX***
cg05460571	4.89E-06	0.008968692	0.597118343	0.60175227	-0.004633927	FDR0.01	8	8630364	Body;Body	8581	***CLDN23***	***RP11-211C9.1***
cg24305797	4.89E-06	0.008968692	0.04969008	0.044077239	0.005612842	FDR0.01	16	9185438	TSS200	-98	***C16orf72***	***C16orf72***
cg24176760	4.89E-06	0.008968692	0.892291679	0.908803574	-0.016511895	FDR0.01	4	76958061	Body;TSS1500;TSS1500	-711	***CXCL11***	***ART3;CXCL11***
cg26233832	4.95E-06	0.009036629	0.663982276	0.708997596	-0.045015321	FDR0.01	7	142660973	TSS1500	-1470	***KEL***	***KEL***
cg06633463	4.97E-06	0.009036629	0.708005529	0.773567454	-0.065561926	FDR0.01	11	123616411	TSS5000;TSS5000;TSS5000	-4020	***ZNF202***	***ZNF202***
cg27261597	5.11E-06	0.009226385	0.797807106	0.806043447	-0.00823634	FDR0.01	1	209604378	TSS1500;Body;Body;Body;Body;Body	-1099	***MIR205***	***MIR205;MIR205HG***
cg01464484	5.14E-06	0.009226385	0.633048241	0.663974865	-0.030926624	FDR0.01	11	67089352	Body	4043	***LOC100130987***	***LOC100130987***
cg04232674	5.14E-06	0.009226385	0.047954074	0.043780142	0.004173933	FDR0.01	1	150601743	Body;Body;TSS200;TSS200;TSS200;Body;Body;Body	-134	***ENSA***	***ENSA***
cg08769781	5.22E-06	0.009310682	0.043520242	0.039615398	0.003904844	FDR0.01	8	23315473	TSS1500;TSS1500	-229	***ENTPD4***	***ENTPD4***
cg12190521	5.26E-06	0.009310682	0.742749549	0.743779487	-0.001029938	FDR0.01	7	1220036		19616	***LOC101927021***	
cg08595592	5.27E-06	0.009310682	0.694592643	0.77528363	-0.080690987	FDR0.01	7	16780162		-12997	***TSPAN13***	
cg15433309	5.27E-06	0.009310682	0.041404412	0.036575103	0.004829309	FDR0.01	5	54468913	TSS5000;5'UTR;TSS5000;TSS1500;5'UTR;5'UTR	92	***CDC20B***	***MIR449B;CDC20B;MIR449A;MIR449C***
cg05355301	5.29E-06	0.009310682	0.688443784	0.68771894	0.000724844	FDR0.01	17	39143239	Body	148	***KRT40***	***KRT40***
cg25053018	5.32E-06	0.009328577	0.738076325	0.771567338	-0.033491013	FDR0.01	2	173477995	Body	57299	***PDK1***	***PDK1***
cg02280309	5.37E-06	0.009382265	0.638008696	0.638313728	-0.000305032	FDR0.01	1	155270960	TSS200;Body	-168	***PKLR***	***PKLR***
cg23033014	5.45E-06	0.009469826	0.859659898	0.886062424	-0.026402526	FDR0.01	10	43849970		12737	***FXYD4***	***RNU6ATAC11P***
cg16233074	5.50E-06	0.009469826	0.862889368	0.857969291	0.004920077	FDR0.01	17	42445042	TSS5000;TES5000	-3807	***FAM171A2***	***FAM171A2;ITGA2B***
cg11516463	5.50E-06	0.009469826	0.912265346	0.915838843	-0.003573497	FDR0.01	5	53811272	TSS5000;TSS5000;TSS5000	-2316	***SNX18***	***SNX18***
cg22708853	5.51E-06	0.009469826	0.040089369	0.034774996	0.005314372	FDR0.01	19	49977670	Body;TES5000;TES5000;Body;TSS200;Body;Body	-147	***FLT3LG***	***FLT3LG;ALDH16A1***
cg12746120	5.53E-06	0.009477857	0.760959498	0.786393939	-0.025434441	FDR0.01	13	110398188		17560	***RP11-313L9.1***	***LINC00676***
cg10677324	5.58E-06	0.009507091	0.799922557	0.824362055	-0.024439498	FDR0.01	3	49837654	TES5000;TSS1500;TSS5000;TSS5000	-386	***CDHR4***	***UBA7;CDHR4;INKA1***
cg12074090	5.60E-06	0.009507091	0.781262093	0.786307916	-0.005045823	FDR0.01	8	6360436	3'UTR;3'UTR;Body;3'UTR;Body;Body;Body	60348	***ANGPT2***	***ANGPT2;MCPH1***
cg04739306	5.61E-06	0.009507091	0.097987025	0.115698169	-0.017711144	FDR0.01	7	100230781	Body;Body	492	***TFR2***	***TFR2***
cg26750548	5.77E-06	0.009678275	0.982997149	0.985305291	-0.002308141	FDR0.01	10	88441152	Body;Body;Body;Body;Body;Body	12727	***LDB3***	***LDB3***
cg03891302	5.78E-06	0.009678275	0.848664622	0.859693767	-0.011029145	FDR0.01	6	13248958	Body;Body;Body;Body;Body;Body;Body;Body;Body	34716	***LOC100130357***	***PHACTR1***
cg21221712	5.78E-06	0.009678275	0.685227086	0.766196189	-0.080969103	FDR0.01	15	86132153	Body;Body	-31027	***AKAP13***	***AKAP13***
cg08015382	5.90E-06	0.009826631	0.659532716	0.680271128	-0.020738412	FDR0.01	6	12365899		41114	***EDN1***	***RN7SKP293***
cg16127573	5.91E-06	0.009826631	0.750402115	0.788256555	-0.03785444	FDR0.01	11	57532299	Body;Body;Body;Body;Body;Body;Body;Body;Body;Body;Body;Body;Body;Body;Body;Body;Body;Body;Body;Body;Body;Body;Body	3066	***CTNND1***	***TMX2-CTNND1;CTNND1***
cg16558348	5.95E-06	0.009849216	0.034444096	0.031375464	0.003068632	FDR0.01	9	86322296	Body;TSS1500;TSS1500;Body	-453	***LOC105376114***	***UBQLN1;LOC105376114***
cg12041266	5.97E-06	0.009849216	0.693674108	0.730558194	-0.036884086	FDR0.01	11	75050147	Body;Body;TSS5000	-3917	***MIR326***	***ARRB1;MIR326***
cg07586391	6.03E-06	0.0098948	0.946664875	0.94502574	0.001639135	FDR0.01	2	3063651	Body	66147	***LINC01250***	***LINC01250***
cg12979234	6.04E-06	0.0098948	0.756773398	0.804803103	-0.048029705	FDR0.01	7	44083518	Body;Body;TSS1500;TSS1500;TSS1500;TSS1500;TSS1500;TSS1500;TSS5000;TES5000	-720	***DBNL***	***LINC00957;DBNL;RASA4CP***
cg26229591	6.06E-06	0.009897599	0.057010807	0.052270311	0.004740495	FDR0.01	5	174905345	TSS200;TSS200;TSS200;TSS200;TSS200;TSS200;TSS200	-155	***SFXN1***	***SFXN1***
cg02911270	6.09E-06	0.009905278	0.824346988	0.832090705	-0.007743717	FDR0.01	8	141629513	Body;Body	16132	***AGO2***	***AGO2***
cg17591741	6.18E-06	0.01002045	0.778513023	0.811413759	-0.032900736	FDR0.05	1	206316396	TSS1500;TSS1500;TSS1500	-1062	***CTSE***	***CTSE***
cg27307975	6.21E-06	0.010022003	0.754323063	0.762839578	-0.008516514	FDR0.05	15	58797839	Body	-6905	***LIPC-AS1***	***LIPC***
cg15876825	6.30E-06	0.010098391	0.848495131	0.886169313	-0.037674182	FDR0.05	3	11651881	Body;Body;Body	-5826	***VGLL4***	***VGLL4***
cg23013664	6.30E-06	0.010098391	0.784666337	0.812294409	-0.027628073	FDR0.05	10	71582505	Body;Body;Body;Body;Body;Body;Body	20862	***COL13A1***	***COL13A1***
cg14436861	6.33E-06	0.010116723	0.777705976	0.825470326	-0.04776435	FDR0.05	11	9610239	3'UTR;3'UTR	14006	***WEE1***	***WEE1***
cg09111484	6.37E-06	0.01013778	0.801413198	0.830565365	-0.029152167	FDR0.05	12	80749651	Body	-88474	***PTPRQ***	***OTOGL***
cg17214295	6.42E-06	0.010185348	0.260353764	0.236137222	0.024216543	FDR0.05	2	383401		-5010	***AC105393.1***	

#cpg	p_adj	q_val	Meth.BPD	Meth.nonBPD	dMeth	significance	Chr	pos.hg19	locations	dist2TSS	gene.nearest	gene.Illumina
cg11391732	6.49E-06	0.010259175	0.854489073	0.860425355	-0.005936282	FDR0.05	5	53751919	1stExon	489	***HSPB3***	***HSPB3***
cg06200607	6.52E-06	0.010267407	0.871072822	0.883298207	-0.012225385	FDR0.05	2	29298362	TSS1500	-1235	***PCARE***	***PCARE***
cg04604962	6.69E-06	0.010495251	0.866440338	0.877798674	-0.011358336	FDR0.05	1	233430241	Body;Body	1218	***PCNX2***	***PCNX2***
cg19230709	6.78E-06	0.010567054	0.9731956	0.969090486	0.004105114	FDR0.05	19	4933046	Body;Body;Body;Body;TES5000;Body	348	***MIR4747***	***UHRF1;MIR4747***
cg20601276	6.78E-06	0.010567054	0.777861952	0.768224718	0.009637234	FDR0.05	20	36148133	Body;Body;Body;TSS1500;Body;Body;Body;TSS1500;Body;TSS1500	572	***BLCAP***	***BLCAP;NNAT***
cg16235843	6.88E-06	0.010681355	0.846560069	0.84615683	0.00040324	FDR0.05	11	129899734		26902	***NCRNA00167***	
cg15806103	6.95E-06	0.010757439	0.917517169	0.910143587	0.007373582	FDR0.05	6	74079722	TSS1500	-207	***OOEP***	***OOEP***
cg22113065	6.98E-06	0.010763388	0.775773688	0.822962479	-0.047188791	FDR0.05	5	74672346	Body;Body;Body	39354	***HMGCR***	***COL4A3BP***
cg16509192	7.06E-06	0.010846251	0.8976944	0.902091469	-0.004397069	FDR0.05	3	122107024	TSS5000;Body;TSS5000	4002	***FAM162A***	***CCDC58;FAM162A***
cg12258179	7.11E-06	0.010852319	0.932519695	0.955137849	-0.022618154	FDR0.05	19	49232187	Body	-9211	***MAMSTR***	***RASIP1***
cg01655150	7.11E-06	0.010852319	0.048698205	0.044640496	0.004057709	FDR0.05	3	126195255	TES5000;TSS1500;TES5000;TSS1500;TSS1500	-493	***ZXDC***	***UROC1;ZXDC***
cg21317856	7.28E-06	0.011045599	0.907847185	0.892089285	0.0157579	FDR0.05	19	1918891	Body;Body;Body	13679	***ADAT3***	***SCAMP4***
cg21651000	7.30E-06	0.011045599	0.271407159	0.268735514	0.002671645	FDR0.05	8	71129472	Body;Body;Body;Body;Body;Body	-145910	***PRDM14***	***NCOA2***
cg18733486	7.32E-06	0.011045599	0.030515439	0.025487331	0.005028109	FDR0.05	5	31532181	5'UTR;TSS200;TSS200;5'UTR	101	***DROSHA***	***DROSHA;C5orf22***
cg11047382	7.33E-06	0.011045599	0.855262215	0.85789434	-0.002632125	FDR0.05	8	1812614	Body;Body;Body	40473	***ARHGEF10***	***ARHGEF10***
cg09976893	7.41E-06	0.011098097	0.703895878	0.72587789	-0.021982012	FDR0.05	8	117753093	Body	14969	***EIF3H***	***EIF3H***
cg24301247	7.45E-06	0.011098097	0.850331895	0.861291087	-0.010959192	FDR0.05	12	129295649	Body	12892	***SLC15A4***	***SLC15A4***
cg19618807	7.46E-06	0.011098097	0.858128866	0.85504453	0.003084336	FDR0.05	12	14217297		-82406	***GRIN2B***	
cg05041511	7.49E-06	0.011098097	0.760133705	0.777800293	-0.017666588	FDR0.05	7	137671105	Body;Body	15742	***CREB3L2***	***CREB3L2***
cg00079056	7.51E-06	0.011098097	0.841953772	0.853218998	-0.011265226	FDR0.05	9	33239641	TSS1500	-520	***SPINK4***	***SPINK4***
cg04570551	7.52E-06	0.011098097	0.738383783	0.713332692	0.025051091	FDR0.05	13	113917124	Body;Body;Body;Body;TSS200;Body;Body;Body;Body;Body	-109	***MIR8075***	***CUL4A;MIR8075***
cg25110043	7.55E-06	0.011107903	0.037185791	0.03449412	0.002691672	FDR0.05	12	56511951	TES5000;TES5000;TES5000;TSS1500;TSS200;TSS200	-52	***ZC3H10***	***RPL41;PA2G4;ZC3H10***
cg16729016	7.70E-06	0.011218245	0.729120785	0.76988643	-0.040765646	FDR0.05	1	213298061	Body;Body;Body;Body;Body;Body;Body;Body;Body;Body;Body;Body;Body;Body;Body;Body;Body;5'UTR;Body;Body;Body;Body;Body;Body;Body;Body;Body;Body;Body;5'UTR;Body;Body;Body;Body;Body	73487	***RPS6KC1***	***RPS6KC1***
cg24977891	7.70E-06	0.011218245	0.740030388	0.808056027	-0.068025639	FDR0.05	2	201733454	TES5000;TSS5000;TSS5000;TES5000;TSS5000;TSS5000	-3987	***CLK1***	***PPIL3;CLK1***
cg18144742	7.71E-06	0.011218245	0.288594791	0.310412651	-0.02181786	FDR0.05	14	107252125		7089	***MIR5195***	
cg20575173	7.73E-06	0.011218245	0.56449437	0.594530024	-0.030035654	FDR0.05	3	101228308	Body;Body;Body;Body;Body;Body	3777	***SENP7***	***SENP7***
cg07089276	7.86E-06	0.01138	0.833311565	0.833542924	-0.000231359	FDR0.05	3	100207343	TSS5000;TSS5000	-4119	***TMEM45A***	***TMEM45A***
cg16365445	7.98E-06	0.011507691	0.872676067	0.878662875	-0.005986809	FDR0.05	10	33230686	Body;Body	-6200	***ITGB1***	***ITGB1***
cg06176929	8.02E-06	0.011526732	0.940282871	0.956175921	-0.01589305	FDR0.05	7	158331066	Body;Body;Body;Body;Body	3402	***PTPRN2***	***PTPRN2***
cg26341453	8.06E-06	0.011526732	0.742524585	0.750999002	-0.008474417	FDR0.05	1	28593732	Body	7770	***SESN2***	***SESN2***
cg12491368	8.07E-06	0.011526732	0.817008374	0.824059732	-0.007051358	FDR0.05	13	32523340	Body	-82096	***FRY***	***EEF1DP3***
cg08471739	8.16E-06	0.011620785	0.177690507	0.174348262	0.003342246	FDR0.05	3	129312902	Body	12680	***PLXND1***	***PLXND1***
cg17675088	8.38E-06	0.011858688	0.774447866	0.780809638	-0.006361771	FDR0.05	5	93013224	Body;Body;Body;Body	-56730	***MIR2277***	***FAM172A***
cg07622687	8.38E-06	0.011858688	0.825996214	0.828861162	-0.002864948	FDR0.05	17	65503465	Body;Body	-35764	***MIR548D2***	***PITPNC1***
cg13899143	8.41E-06	0.011858688	0.84162096	0.851754464	-0.010133504	FDR0.05	7	11315835	Body;Body;Body;Body;Body;Body	23096	***AC004538.3***	***AC004160.1***
cg08678256	8.46E-06	0.011897961	0.13239515	0.107525617	0.024869533	FDR0.05	16	30196023	Body;Body;TSS5000	1293	***CORO1A***	***CORO1A;LOC606724***
cg17095737	8.49E-06	0.011897961	0.922211657	0.931579536	-0.009367879	FDR0.05	15	70654556		40642	***RP11-543G18.1***	
cg21672401	8.52E-06	0.011912396	0.753764181	0.807956682	-0.054192502	FDR0.05	12	43499608		-49509	***LINC02461***	
cg06757356	8.58E-06	0.011959125	0.785469138	0.803032179	-0.017563041	FDR0.05	13	60712449	Body;Body;Body;Body;Body	-6382	***DIAPH3-AS2***	***DIAPH3***
cg19096799	8.72E-06	0.012111629	0.021017335	0.018684257	0.002333078	FDR0.05	5	131347112	Body;Body;Body;Body;Body;Body	243	***ACSL6***	***ACSL6***
cg12289801	8.80E-06	0.012189969	0.662684386	0.672551585	-0.009867199	FDR0.05	19	19106904	Body;Body;Body;Body;Body;Body;Body;Body;Body	37476	***SUGP2***	***SUGP2***
cg13990856	8.93E-06	0.012320218	0.847746737	0.848695151	-0.000948414	FDR0.05	10	49940677	Body	47160	***WDFY4***	***WDFY4***
cg27104413	9.04E-06	0.01243436	0.712742682	0.76485448	-0.052111798	FDR0.05	3	101044447	3'UTR;3'UTR;3'UTR;3'UTR;3'UTR	-5028	***IMPG2***	***SENP7***
cg10878542	9.14E-06	0.012543021	0.737397257	0.79122888	-0.053831623	FDR0.05	14	55591912	TSS5000;TSS5000	-4022	***LGALS3***	***LGALS3***
cg09289439	9.17E-06	0.012543021	0.744572263	0.761419786	-0.016847522	FDR0.05	7	73994764	Body;Body;Body	-77226	***GTF2I***	***GTF2IRD1***
cg10947001	9.22E-06	0.012543021	0.778599247	0.823295435	-0.044696187	FDR0.05	4	139593196		-6473	***RP11-173E2.2***	
cg00957325	9.23E-06	0.012543021	0.891101537	0.88754759	0.003553947	FDR0.05	6	36812260	TSS5000	-4482	***CPNE5***	***CPNE5***
cg25968247	9.25E-06	0.012543021	0.885766257	0.879326419	0.006439837	FDR0.05	3	57581113	TSS5000;Body;TSS5000;Body	2102	***ARF4***	***ARF4-AS1;ARF4;PDE12***
cg01243513	9.29E-06	0.012556114	0.664445648	0.686360581	-0.021914932	FDR0.05	12	58003151	3'UTR;TSS5000;TSS5000;TSS5000;3'UTR;TSS1500;3'UTR	-811	***ARHGEF25***	***DTX3;ARHGEF25***
cg20472746	9.38E-06	0.012572613	0.371470612	0.318423363	0.053047249	FDR0.05	13	42039334	Body	7793	***RGCC***	***RGCC***
cg21451816	9.45E-06	0.012572613	0.40689669	0.39788926	0.009007429	FDR0.05	7	1468848	Body;Body;Body	8399	***MICALL2***	***MICALL2***
cg05097455	9.48E-06	0.012572613	0.041567396	0.03678747	0.004779926	FDR0.05	3	47324909	TSS1500;TSS1500;Body;TSS1500	-572	***KIF9***	***KIF9;KLHL18***
cg15134174	9.49E-06	0.012572613	0.574480918	0.610577796	-0.036096877	FDR0.05	4	170022074	Body	-90606	***CBR4***	***SH3RF1***
cg14947411	9.56E-06	0.012572613	0.946885422	0.961622965	-0.014737543	FDR0.05	17	80660948	TSS5000	-4350	***RAB40B***	***RAB40B***
cg15526035	9.57E-06	0.012572613	0.055866969	0.064075918	-0.008208949	FDR0.05	14	54686833		-176733	***CDKN3***	
cg12127863	9.57E-06	0.012572613	0.741752521	0.759142312	-0.017389792	FDR0.05	13	100252695		-6222	***CLYBL***	
cg19975917	9.57E-06	0.012572613	0.880904291	0.880139634	0.000764657	FDR0.05	3	188231905	Body;Body;Body	54549	***LPP-AS1***	***LPP***

#cpg	p_adj	q_val	Meth.BPD	Meth.nonBPD	dMeth	significance	Chr	pos.hg19	locations	dist2TSS	gene.nearest	gene.Illumina
cg14441153	9.58E-06	0.012572613	0.063252379	0.054437645	0.008814734	FDR0.05	9	4763200		-21157	***AK3***	
cg22215508	9.61E-06	0.012572613	0.774693671	0.782853523	-0.008159852	FDR0.05	4	26024198		-48177	***RP11-263J14.1***	
cg18418962	9.61E-06	0.012572613	0.036397623	0.031728657	0.004668966	FDR0.05	18	2571633	TSS200;5'UTR;TSS200	124	***NDC80***	***METTL4;NDC80***
cg23471393	9.67E-06	0.012613596	0.70731021	0.717331169	-0.010020959	FDR0.05	17	79797038	TSS5000;TES5000	-4112	***PPP1R27***	***PPP1R27;P4HB***
cg18464559	9.75E-06	0.01266512	0.023819	0.018557733	0.005261266	FDR0.05	1	63989278	TSS1500;TSS1500;TSS1500;5'UTR;TSS1500;TSS1500;TSS1500	307	***EFCAB7***	***ITGB3BP;EFCAB7***
cg16023337	9.81E-06	0.01266512	0.866283174	0.865046324	0.001236849	FDR0.05	17	39662206	TSS1500;TSS1500	-341	***KRT13***	***KRT13***
cg01806520	9.81E-06	0.01266512	0.026677849	0.022749702	0.003928146	FDR0.05	8	93115504	TSS200;TSS200;TSS5000	-50	***RUNX1T1***	***RUNX1T1***
cg22874989	9.83E-06	0.01266512	0.6926614	0.66074852	0.03191288	FDR0.05	15	29277898	Body;Body;Body;Body;Body;Body;Body;Body;Body	-9817	***APBA2***	***APBA2***
cg24035299	1.00E-05	0.012825943	0.804915315	0.851751365	-0.04683605	FDR0.05	1	65883252	TES5000;TES5000;TSS5000;TSS5000;TSS5000;TSS5000;TES5000;TSS5000;TSS5000	-2878	***LEPROT***	***DNAJC6;LEPR;LEPROT***
cg13580783	1.00E-05	0.012825943	0.765928377	0.751085516	0.014842861	FDR0.05	1	24128567	3'UTR;TSS5000;3'UTR;TSS1500;TSS5000	-1273	***GALE***	***HMGCL;GALE***
cg17068675	1.01E-05	0.012825943	0.879015375	0.868731451	0.010283924	FDR0.05	6	29759197	TSS1500;Body	-485	***HLA-V***	***HLA-V;HCG4***
cg21141329	1.01E-05	0.012825943	0.119721736	0.102858226	0.01686351	FDR0.05	17	48290459	TSS5000	-1595	***LINC01969***	***LINC01969***
cg02312904	1.04E-05	0.013152273	0.85663948	0.847201557	0.009437923	FDR0.05	17	59234870	Body;Body;Body;Body;Body;Body;Body;Body	210195	***LOC101927855***	***BCAS3***
cg15698795	1.04E-05	0.013178357	0.203599631	0.190031704	0.013567927	FDR0.05	11	67177103	3'UTR;Body;Body	5657	***TBC1D10C***	***TBC1D10C***
cg08069370	1.05E-05	0.013220141	0.169308247	0.197498665	-0.028190419	FDR0.05	15	64387884	TSS5000;TSS5000;TSS200;TSS5000;TSS200;TSS200;TSS5000	-198	***SNX1***	***CIAO2A;SNX1***
cg17220545	1.05E-05	0.013220141	0.829008366	0.84587453	-0.016866164	FDR0.05	7	157085556		-10176	***RP5-1142J19.1***	
cg09929187	1.05E-05	0.013220141	0.834112065	0.818706117	0.015405948	FDR0.05	12	92501570	Body;Body;Body;Body;Body	33919	***LINC01619***	***LINC01619***
cg09872523	1.06E-05	0.013303843	0.819566223	0.831078407	-0.011512184	FDR0.05	10	121001024	Body	33828	***GRK5***	***GRK5***
cg19734228	1.07E-05	0.013373298	0.062714334	0.05758992	0.005124414	FDR0.05	9	90113593	Body;Body;Body;Body	144	***DAPK1***	***DAPK1***
cg01934638	1.08E-05	0.013400015	0.783954949	0.831006674	-0.047051725	FDR0.05	1	3720744		-7676	***LRRC47***	
cg13521891	1.08E-05	0.013400015	0.04652963	0.041329127	0.005200503	FDR0.05	12	95466445	Body;Body;TES5000;Body	959	***NR2C1***	***NR2C1;FGD6***
cg16942644	1.08E-05	0.013400015	0.871350193	0.862139002	0.009211191	FDR0.05	21	46867867	Body	-7556	***COL18A1***	***COL18A1***
cg17864040	1.08E-05	0.013400015	0.040509045	0.035579485	0.00492956	FDR0.05	1	78148524	Body;TSS200	-181	***ZZZ3***	***ZZZ3***
cg06439889	1.09E-05	0.013400015	0.663962013	0.661425749	0.002536264	FDR0.05	6	72077399	Body;Body;Body	7691	***RP3-331H24.7***	***RP3-331H24.5;LINC00472***
cg19850275	1.09E-05	0.013420463	0.729321645	0.77549928	-0.046177635	FDR0.05	10	75654257		-14677	***PLAU***	
cg07148919	1.11E-05	0.013593611	0.877942885	0.884957853	-0.007014968	FDR0.05	5	1466943	Body	-21400	***SLC6A3***	***LPCAT1***
cg17698643	1.12E-05	0.013633033	0.05251851	0.045194982	0.007323528	FDR0.05	16	15982631	TSS200;TSS200;TSS200;TSS200;TSS200;TSS200;TSS200;TSS200;TSS200	-125	***FOPNL***	***FOPNL***
cg19611418	1.13E-05	0.013633033	0.086295872	0.075388026	0.010907846	FDR0.05	2	131851282	TSS1500;TSS1500;TSS1500;TSS1500;TSS1500;TSS1500;TSS1500	-249	***FAM168B***	***FAM168B***
cg26023268	1.13E-05	0.013633033	0.737747966	0.721173488	0.016574478	FDR0.05	17	71258215	TSS200	-196	***CPSF4L***	***CPSF4L***
cg04208329	1.13E-05	0.013633033	0.905352141	0.908413543	-0.003061402	FDR0.05	14	75445376		-22889	***PGF***	
cg00611192	1.13E-05	0.013633033	0.609365431	0.623062155	-0.013696724	FDR0.05	1	249151633	Body;Body;Body;5'UTR;Body	1492	***ZNF692***	***ZNF692***
cg17017514	1.13E-05	0.013633033	0.889730474	0.885300679	0.004429795	FDR0.05	1	156679733	TSS5000;TSS5000	-4125	***CRABP2***	***CRABP2***
cg16126186	1.14E-05	0.013738924	0.866548463	0.885045586	-0.018497124	FDR0.05	18	22053986	Body;Body;Body	13394	***HRH4***	***HRH4***
cg20122476	1.15E-05	0.013738924	0.096685527	0.076524089	0.020161438	FDR0.05	17	1465162	Body	948	***PITPNA***	***PITPNA***
cg11791710	1.17E-05	0.014039218	0.708240601	0.738091991	-0.029851391	FDR0.05	10	129172535	Body;Body	-175077	***NPS***	***DOCK1***
cg01626326	1.18E-05	0.014053632	0.055872831	0.048621147	0.007251683	FDR0.05	2	144694809	Body	176	***LOC101928386***	***LOC101928386***
cg15878685	1.18E-05	0.014074962	0.699017683	0.760497721	-0.061480037	FDR0.05	4	69818856	TSS1500;TSS1500	-1347	***UGT2A3***	***UGT2A3***
cg11794814	1.19E-05	0.014074962	0.02748378	0.02513746	0.00234632	FDR0.05	19	40791331	TSS200;TSS200;TSS200;TSS5000	-29	***AKT2***	***AKT2;MIR641***
cg16219968	1.20E-05	0.014186654	0.031112533	0.028039593	0.00307294	FDR0.05	12	40500016	TSS1500	-355	***SLC2A13***	***SLC2A13***
cg13430960	1.21E-05	0.01428378	0.816553554	0.818014326	-0.001460772	FDR0.05	3	37926307	Body;Body	22639	***CTDSPL***	***CTDSPL***
cg06373817	1.22E-05	0.01428378	0.793959549	0.809762654	-0.015803105	FDR0.05	15	93123126	TSS1500	-932	***RP11-386M24.3***	***RP11-386M24.3***
cg07493198	1.22E-05	0.01428378	0.038514878	0.03487233	0.003642548	FDR0.05	6	107781013	TSS1500	-234	***PDSS2***	***PDSS2***
cg24516147	1.22E-05	0.01428378	0.043099658	0.034803684	0.008295974	FDR0.05	6	130339725	TSS1500;TSS1500;5'UTR;5'UTR	212	***L3MBTL3***	***L3MBTL3***
cg10457079	1.22E-05	0.01428378	0.86266072	0.853241243	0.009419476	FDR0.05	16	12298857	Body	228267	***SNX29***	***SNX29***
cg10081621	1.23E-05	0.014305959	0.855191694	0.866965085	-0.011773391	FDR0.05	15	94808566	Body;Body;Body;Body	-32928	***MCTP2***	***MCTP2***
cg21230400	1.24E-05	0.014335482	0.78854722	0.785644143	0.002903077	FDR0.05	9	112405522	Body	2455	***PALM2***	***PALM2***
cg27213024	1.24E-05	0.014335482	0.847711049	0.871318133	-0.023607084	FDR0.05	6	143913327	Body	-15989	***PHACTR2***	***PHACTR2***
cg08769073	1.24E-05	0.014335482	0.661073346	0.681596938	-0.020523592	FDR0.05	11	12214804	Body;Body;Body;Body;Body;Body;Body;Body;Body;Body;Body;Body;Body;Body;Body;Body;Body;Body;Body;Body;Body	29575	***MIR6124***	***MICAL2***
cg25368824	1.24E-05	0.014335482	0.68024656	0.738746868	-0.058500308	FDR0.05	5	132009352	TSS1500;TSS1500;TSS1500	-325	***IL4***	***IL4***
cg24355182	1.25E-05	0.01439277	0.872045851	0.86929155	0.002754301	FDR0.05	21	45842649	Body;Body;TSS5000;Body;Body;Body	-2762	***TRPM2***	***TRPM2;TRPM2-AS***
cg12973904	1.27E-05	0.014538502	0.64628761	0.715811274	-0.069523665	FDR0.05	7	149457885	TES5000;TES5000	12683	***ZNF467***	***ZNF467***
cg13922494	1.28E-05	0.014640801	0.829026364	0.842788435	-0.013762071	FDR0.05	5	106890760	Body	115836	***EFNA5***	***EFNA5***
cg14906455	1.29E-05	0.014652916	0.054282097	0.042701319	0.011580778	FDR0.05	17	2628088	Body	12486	***LOC105371592***	***CCDC92B***
cg14746369	1.29E-05	0.014652916	0.052991145	0.049089	0.003902146	FDR0.05	6	33290934	TSS200;TSS200;TSS200;TSS200	-141	***DAXX***	***DAXX***
cg04067066	1.29E-05	0.014652916	0.362238069	0.339432928	0.022805141	FDR0.05	2	219756890	Body	11636	***WNT10A***	***WNT10A***
cg08112479	1.30E-05	0.014702498	0.037145132	0.032915169	0.004229963	FDR0.05	6	37665626	5'UTR	140	***MDGA1***	***MDGA1***
cg25224369	1.31E-05	0.014751456	0.592548411	0.602295514	-0.009747103	FDR0.05	3	12918528	TES5000	8182	***LINC02022***	***LINC02022***

#cpg	p_adj	q_val	Meth.BPD	Meth.nonBPD	dMeth	significance	Chr	pos.hg19	locations	dist2TSS	gene.nearest	gene.Illumina
cg10443146	1.31E-05	0.014751456	0.816563029	0.813894652	0.002668377	FDR0.05	2	170779769	Body	95752	***UBR3***	***UBR3***
cg12018521	1.32E-05	0.014797776	0.73712254	0.78824025	-0.05111771	FDR0.05	7	115858134	Body;TSS5000	-4870	***TES***	***TES***
cg09725286	1.34E-05	0.014951454	0.790135092	0.813009617	-0.022874525	FDR0.05	8	134419335		68842	***ST3GAL1***	
cg18799217	1.34E-05	0.014951454	0.059531933	0.051395356	0.008136576	FDR0.05	5	87985589	TSS5000;Body;Body;Body;TSS5000;TSS5000;TSS5000;TSS5000	1269	***LINC00461***	***LINC00461;MEF2C-AS2***
cg04294331	1.34E-05	0.014978557	0.749537964	0.825534792	-0.075996828	FDR0.05	6	32133978	TES5000;TES5000;Body;TES5000;TES5000;Body;TES5000;1stExon;TES5000	1597	***EGFL8***	***AGPAT1;PPT2-EGFL8;PPT2;EGFL8;MIR6721***
cg11549417	1.35E-05	0.015001879	0.143764646	0.145457535	-0.001692888	FDR0.05	13	114814643	Body;Body;Body	28811	***RASA3***	***RASA3***
cg27292977	1.36E-05	0.015008693	0.787328047	0.802403523	-0.015075477	FDR0.05	14	92305602	Body;TSS5000;TSS5000;TSS5000	-2732	***TC2N***	***TC2N***
cg05005073	1.36E-05	0.015008693	0.683083292	0.742520835	-0.059437543	FDR0.05	1	39513326		21337	***NDUFS5***	
cg24375626	1.36E-05	0.015008693	0.846192261	0.833750212	0.012442049	FDR0.05	1	182098716	TSS1500;Body	-916	***GS1-122H1.1***	***GS1-122H1.1;GS1-122H1.2***
cg24046475	1.36E-05	0.015008693	0.120085815	0.116477064	0.003608751	FDR0.05	8	86371819	TSS5000;Body;Body;Body;Body;TSS5000	3464	***CA3-AS1***	***CA2;CA3-AS1***
cg01943585	1.37E-05	0.015055126	0.852720211	0.878306742	-0.025586531	FDR0.05	1	112981693	Body	22762	***MIR4256***	***CTTNBP2NL***
cg04865220	1.37E-05	0.015055126	0.844590028	0.854783183	-0.010193155	FDR0.05	15	73886532	Body;Body;Body;Body	-24648	***NPTN-IT1***	***NPTN***
cg13844820	1.38E-05	0.015055126	0.826132362	0.837915585	-0.011783223	FDR0.05	1	50574156	TSS1500;TSS1500;Body;TSS1500;TSS1500;TSS1500;Body;TSS1500;TSS1500;TSS1500;TSS1500;TSS1500;TSS1500;Body;Body	-435	***ELAVL4***	***ELAVL4***
cg07810726	1.38E-05	0.015070221	0.037274599	0.032179908	0.005094691	FDR0.05	5	158524649	Body;5'UTR;Body;Body;5'UTR;Body;5'UTR;Body;5'UTR;Body;Body;TSS5000;Body;Body;Body;Body	315	***EBF1***	***EBF1;LINC02202***
cg03636488	1.38E-05	0.015070221	0.319877908	0.266983557	0.052894351	FDR0.05	12	132551127	Body	-17700	***EP400P1***	***EP400***
cg24685109	1.41E-05	0.015259825	0.646847428	0.665047786	-0.018200359	FDR0.05	15	43477090	3'UTR;3'UTR;3'UTR;3'UTR;3'UTR;3'UTR;3'UTR;Body;Body;TSS1500;TSS1500;3'UTR;3'UTR;3'UTR;3'UTR;TSS1500;Body;TSS1500;3'UTR;3'UTR;3'UTR;3'UTR;3'UTR;3'UTR;3'UTR;3'UTR;3'UTR;3'UTR;3'UTR;3'UTR;3'UTR;3'UTR;3'UTR;3'UTR;3'UTR;TSS1500;3'UTR;Body;3'UTR;3'UTR	-375	***CCNDBP1***	***TMEM62;CCNDBP1***
cg16706324	1.42E-05	0.015317866	0.847082159	0.854526885	-0.007444726	FDR0.05	11	5685914	3'UTR;Body;3'UTR	20425	***TRIM5***	***TRIM5***
cg03403539	1.42E-05	0.015317866	0.033517753	0.02896741	0.004550343	FDR0.05	7	23221679	5'UTR	234	***NUPL2***	***NUPL2***
cg09253696	1.43E-05	0.015337734	0.319684932	0.429840272	-0.11015534	FDR0.05	17	73873529	Body	1127	***TRIM47***	***TRIM47***
cg15417654	1.44E-05	0.015337734	0.661150866	0.671051795	-0.009900929	FDR0.05	3	187959138	Body;Body;Body	15946	***LPP***	***LPP***
cg16384957	1.44E-05	0.015337734	0.872602201	0.908491817	-0.035889616	FDR0.05	11	1050848	Body	1071	***LOC101927503***	***LOC101927503***
cg04128776	1.44E-05	0.015337734	0.938436399	0.946130075	-0.007693677	FDR0.05	11	3435091	TES5000	-21946	***OR7E12P***	***TSSC2***
cg09396068	1.44E-05	0.015337734	0.97681095	0.984625267	-0.007814317	FDR0.05	15	22940794	Body;Body;Body;Body;Body;Body;Body;5'UTR;Body	-14960	***CYFIP1***	***CYFIP1***
cg16570980	1.45E-05	0.015337734	0.826406742	0.867222713	-0.040815971	FDR0.05	16	438561	Body	6321	***LOC100134368***	***LOC100134368***
cg09970511	1.45E-05	0.015337734	0.875001329	0.886477155	-0.011475825	FDR0.05	6	56823640	TSS5000;Body;Body	3868	***BEND6***	***DST;BEND6***
cg04368716	1.45E-05	0.015337734	0.035355456	0.031233397	0.004122059	FDR0.05	14	104028314	Body;TSS1500;TSS1500;TSS1500;TSS1500;TSS1500;TSS1500;1stExon;Body	341	***BAG5***	***BAG5;APOPT1***
cg06818435	1.45E-05	0.015337734	0.678800716	0.681071952	-0.002271236	FDR0.05	1	33279680	Body;TSS5000;TSS5000	-3362	***S100PBP***	***YARS;S100PBP***
cg27119329	1.45E-05	0.01534517	0.594762025	0.591798477	0.002963548	FDR0.05	15	79055434	Body	11056	***LOC646938***	***ADAMTS7***
cg09717809	1.46E-05	0.015389826	0.932238558	0.946389594	-0.014151036	FDR0.05	2	74375930	TSS1500;Body;TES5000;Body;Body;TES5000;TES5000;TES5000;TSS1500	823	***BOLA3-AS1***	***BOLA3;BOLA3-AS1;MOB1A***
cg20586676	1.49E-05	0.015650562	0.033094697	0.029173862	0.003920835	FDR0.05	17	36714386	Body	47797	***SRCIN1***	***SRCIN1***
cg18036143	1.50E-05	0.015678451	0.770770349	0.781258891	-0.010488542	FDR0.05	12	125329150	Body;Body	19369	***SCARB1***	***SCARB1***
cg02036826	1.50E-05	0.015678451	0.782361507	0.80996078	-0.027599273	FDR0.05	1	40721495	TES5000;TSS5000	-2226	***ZMPSTE24***	***TMCO2;ZMPSTE24***
cg04411052	1.51E-05	0.015711733	0.865938108	0.897513357	-0.031575249	FDR0.05	14	72219061		22153	***SIPA1L1***	
cg12332454	1.52E-05	0.015711733	0.042541432	0.036822042	0.00571939	FDR0.05	11	19138563	TSS200;TSS200	-128	***ZDHHC13***	***ZDHHC13***
cg23309527	1.52E-05	0.015711733	0.861351727	0.875216512	-0.013864784	FDR0.05	14	52771169		-9846	***PTGER2***	
cg12205429	1.52E-05	0.015711733	0.947104373	0.940539581	0.006564792	FDR0.05	7	2328896	Body	25214	***SNX8***	***SNX8***
cg14821245	1.52E-05	0.015711733	0.755236704	0.766398524	-0.01116182	FDR0.05	2	80253010	Body;Body;Body;Body	-159301	***MIR8080***	***CTNNA2***
cg11835209	1.53E-05	0.015739653	0.735656536	0.708638485	0.027018051	FDR0.05	5	4512534	Body;Body	160	***RP11-445O3.1***	***RP11-445O3.2;RP11-445O3.1***
cg24636999	1.53E-05	0.015781132	0.736930075	0.782051198	-0.045121123	FDR0.05	6	38751903	Body;Body	68787	***DNAH8***	***DNAH8***
cg11516031	1.55E-05	0.01594567	0.687285167	0.689639747	-0.00235458	FDR0.05	4	15776675	TSS5000;TSS5000	-3212	***CD38***	***CD38***
cg18945794	1.56E-05	0.01594567	0.973886847	0.977642789	-0.003755942	FDR0.05	4	3319669	1stExon;1stExon	3796	***RGS12***	***RGS12***
cg06902929	1.57E-05	0.015974738	0.729830327	0.729575827	0.0002545	FDR0.05	6	32123258	TSS1500;Body;Body;TSS5000;Body;TSS1500;TSS1500;TSS1500;Body;TSS5000	-1116	***LOC100507547***	***LOC100507547;PPT2-EGFL8;PPT2;PRRT1***
cg04402088	1.57E-05	0.015974738	0.96682973	0.973702729	-0.006872999	FDR0.05	13	114764394	Body;Body;Body	-20907	***C13orf46***	***RASA3***
cg10636959	1.58E-05	0.016058373	0.821551929	0.819661595	0.001890334	FDR0.05	6	30162942	Body;Body	18329	***TRIM26***	***TRIM26***
cg01566552	1.59E-05	0.016089624	0.720704984	0.699767746	0.020937238	FDR0.05	19	18977814	3'UTR;3'UTR;TES5000;TES5000	29139	***GDF1***	***UPF1;GDF1;CERS1***
cg23484392	1.60E-05	0.016181958	0.726250521	0.741083568	-0.014833047	FDR0.05	18	66465334	Body;5'UTR	18	***CCDC102B***	***CCDC102B***
cg03580195	1.61E-05	0.016253531	0.939260955	0.927610811	0.011650144	FDR0.05	19	50155693	Body	10312	***SCAF1***	***SCAF1***
cg25765104	1.62E-05	0.016253531	0.085218782	0.068381013	0.016837769	FDR0.05	1	167601275	Body;Body;Body;Body	1802	***RCSD1***	***RCSD1***
cg11676189	1.62E-05	0.016253531	0.164429874	0.13362709	0.030802785	FDR0.05	2	69875470	TSS5000;Body	-4493	***AAK1***	***AAK1;ANXA4***
cg14190534	1.62E-05	0.016253531	0.892533141	0.900163469	-0.007630328	FDR0.05	14	90848416	TES5000;TSS1500	875	***AL512791.1***	***AL512791.1;RP11-471B22.3***
cg16781439	1.64E-05	0.01643748	0.017328232	0.018433247	-0.001105016	FDR0.05	22	29469035	TSS200;TSS200	-30	***KREMEN1***	***KREMEN1***
cg24067150	1.66E-05	0.016567765	0.745526859	0.800176116	-0.054649257	FDR0.05	6	30956397	Body;3'UTR;3'UTR;3'UTR	4913	***MUC21***	***MUC21***
cg12492345	1.66E-05	0.016567765	0.023260863	0.020362874	0.002897989	FDR0.05	16	54971007	TES5000;TES5000	5897	***IRX5***	***IRX5***

#cpg	p_adj	q_val	Meth.BPD	Meth.nonBPD	dMeth	significance	Chr	pos.hg19	locations	dist2TSS	gene.nearest	gene.Illumina
cg16978168	1.68E-05	0.016653326	0.690418576	0.70701872	-0.016600144	FDR0.05	6	159227784	Body;Body	-11226	EZR-AS1	EZR
cg12971660	1.69E-05	0.016786805	0.040513448	0.03722743	0.003286018	FDR0.05	2	173421442	Body;Body;Body;Body	746	PDK1	PDK1
cg08862210	1.70E-05	0.016786805	0.865727773	0.862877738	0.002850035	FDR0.05	6	29364963	1stExon	548	OR12D2	OR12D2
cg27482571	1.70E-05	0.016786805	0.708218393	0.773951427	-0.065733033	FDR0.05	12	42479554	3'UTR;3'UTR	59119	GXYLT1	GXYLT1
cg19012958	1.72E-05	0.016909957	0.791224539	0.805821127	-0.014596588	FDR0.05	7	150479452		14368	TMEM176B	
cg13276631	1.73E-05	0.016965178	0.741900858	0.772910145	-0.031009287	FDR0.05	3	46872239	Body;Body	3074	PRSS42	PRSS42
cg11634875	1.73E-05	0.016993129	0.774627635	0.817153795	-0.04252616	FDR0.05	6	31922863	Body;TES5000;TSS5000;TES5000	1854	MIR1236	NELFE;CFB;SKIV2L;MIR1236
cg11688548	1.75E-05	0.017074954	0.822651812	0.888982192	-0.06633038	FDR0.05	12	1489532	Body;Body;Body;Body;Body;Body	-120124	LINC00942	ERC1
cg04895895	1.75E-05	0.017074954	0.704916937	0.779472944	-0.074556006	FDR0.05	1	231005895	TSS1500;TSS5000;TSS5000	-560	C1orf198	C1orf198;LOC101927604
cg10601625	1.77E-05	0.017273484	0.205793216	0.207754805	-0.00196159	FDR0.05	8	41570622	Body;Body;Body;Body;Body	-47818	ANK1	ANK1
cg00857326	1.81E-05	0.017572353	0.746452977	0.741576067	0.00487691	FDR0.05	8	144920813	Body;TSS1500	-825	MIR6845	NRBP2;MIR6845
cg22973812	1.82E-05	0.017700163	0.761693049	0.754343933	0.007349116	FDR0.05	11	65786500	Body	7039	CST6	CATSPER1
cg12203394	1.84E-05	0.017740349	0.062540215	0.052783358	0.009756856	FDR0.05	1	27248618	Body	406	NUDC	NUDC
cg14508973	1.84E-05	0.017740349	0.921152621	0.920693373	0.000459248	FDR0.05	9	96209198	TSS5000;TSS5000;TSS5000;Body;Body;Body;Body;Body;Body;Body;Body;3'UTR;TSS5000;Body;Body;3'UTR	4695	FAM120AOS	FAM120A;FAM120AOS
cg13962347	1.86E-05	0.017902948	0.270116522	0.22166492	0.048451602	FDR0.05	6	5174647	Body;Body;Body;Body;Body;Body;Body;Body	-26091	MIR3691	LYRM4;LYRM4-AS1
cg02010829	1.87E-05	0.018008935	0.754088322	0.739307988	0.014780333	FDR0.05	16	16090604	Body	47171	ABCC1	ABCC1
cg02474195	1.89E-05	0.018158306	0.744455933	0.769760858	-0.025304926	FDR0.05	3	43391449	Body;3'UTR;3'UTR;3'UTR	2005	SNRK-AS1	SNRK-AS1;SNRK
cg04203742	1.90E-05	0.018181154	0.851582519	0.851710877	-0.000128358	FDR0.05	10	2371007		-13738	RP11-311B18.1	
cg12163532	1.91E-05	0.018277009	0.728584964	0.773178729	-0.044593766	FDR0.05	8	101428643	TSS5000	-1956	KB-173C10.2	KB-173C10.2
cg08275047	1.92E-05	0.018277009	0.051991784	0.046258789	0.005732995	FDR0.05	8	26428926	Body	-6413	DPYSL2	DPYSL2
cg01547051	1.92E-05	0.018277009	0.045163883	0.041331988	0.003831896	FDR0.05	7	150777654	TES5000;TES5000;TES5000;TES5000;Body;Body;TES5000;Body;TES5000	316	FASTK	TMUB1;SLC4A2;FASTK
cg26386653	1.94E-05	0.018391892	0.778730128	0.780594181	-0.001864053	FDR0.05	3	14815452	TES5000;TES5000;TES5000	37469	LINC02011	C3orf20
cg22194305	1.96E-05	0.018597274	0.072828562	0.066507219	0.006321343	FDR0.05	4	2264032	TSS1500	-293	MXD4	MXD4
cg02852327	1.99E-05	0.01873598	0.063512638	0.057951282	0.005561356	FDR0.05	7	121950975	TES5000	-6410	FEZF1	FEZF1-AS1
cg26352718	1.99E-05	0.01873598	0.807223729	0.802601303	0.004622427	FDR0.05	7	36024391		-44829	AC083864.3	
cg06269115	1.99E-05	0.01873598	0.023805491	0.019219938	0.004585552	FDR0.05	11	68451849	TSS200	-93	GAL	GAL
cg17511416	1.99E-05	0.01873598	0.053704351	0.045807726	0.007896624	FDR0.05	9	15553286	5'UTR;5'UTR	282	CCDC171	CCDC171
cg08455184	2.00E-05	0.01875707	0.776344117	0.827800676	-0.051456559	FDR0.05	19	12998927	TSS5000;TSS5000;TSS5000;TSS1500;TSS5000	-910	KLF1	GCDH;KLF1
cg13997124	2.00E-05	0.01875707	0.038159409	0.033518375	0.004641033	FDR0.05	6	28304194	TSS1500;TSS200;TSS200;Body;TSS1500;TSS200;TSS200;Body	-42	ZSCAN31	ZSCAN31
cg02710119	2.03E-05	0.018925038	0.939087282	0.933069882	0.006017399	FDR0.05	21	45078694	Body;TSS1500	680	HSF2BP	HSF2BP;RRP1B
cg21888330	2.04E-05	0.019006324	0.798485435	0.829233585	-0.03074815	FDR0.05	13	42546577	Body	11273	VWA8-AS1	VWA8-AS1
cg18090197	2.05E-05	0.019085656	0.215030132	0.227058887	-0.012028755	FDR0.05	7	99769602	TSS5000;Body	-3229	GAL3ST4	GAL3ST4;GPC2
cg09820180	2.08E-05	0.019307797	0.684778891	0.745751299	-0.060972408	FDR0.05	6	140168822	Body	-7164	FILNC1	FILNC1
cg02704103	2.09E-05	0.01938436	0.865181844	0.884374708	-0.019192864	FDR0.05	3	72846991	Body	50607	SHQ1	SHQ1
cg09587172	2.12E-05	0.019484728	0.030721112	0.026431024	0.004290088	FDR0.05	1	205600246	Body;Body	1754	ELK4	ELK4
cg13883576	2.12E-05	0.019484728	0.822503239	0.856946743	-0.034443504	FDR0.05	12	13152467	Body;Body;TSS1500	776	HEBP1	HEBP1;GPRC5D-AS1;HTR7P1
cg05579861	2.14E-05	0.019484728	0.747641839	0.743837027	0.003804813	FDR0.05	7	157631040	Body;Body;Body;Body;Body	-16236	LOC100506585	PTPRN2
cg07588006	2.14E-05	0.019484728	0.837570235	0.857241463	-0.019671228	FDR0.05	4	175752696	TSS5000;TSS200;TSS5000	-182	LOC101928551	GLRA3;LOC101928551
cg27647623	2.14E-05	0.019484728	0.854583293	0.866729359	-0.012146066	FDR0.05	10	131650678	Body	-9040	MIR4297	EBF3
cg02225096	2.14E-05	0.019484728	0.030827729	0.026191088	0.004636642	FDR0.05	10	46168403	TSS200;TSS1500;TSS200;TSS200;TSS1500	-142	ZFAND4	ZFAND4
cg07403228	2.15E-05	0.019484728	0.846527865	0.84863808	-0.002110215	FDR0.05	17	61792641	Body;Body;Body;Body;Body;Body;Body;Body;Body;Body;Body;Body;Body	14944	LOC729683	STRADA
cg16517358	2.15E-05	0.019484728	0.81023309	0.810448721	-0.000215631	FDR0.05	11	46164796	Body	19673	RP11-702F3.3	RP11-702F3.3
cg20056324	2.15E-05	0.019484728	0.029705538	0.024289813	0.005415725	FDR0.05	12	55413610	TSS200	-118	NEUROD4	NEUROD4
cg14776998	2.15E-05	0.019484728	0.754691507	0.671763787	0.08292772	FDR0.05	3	32863439	Body	3930	TRIM71	TRIM71
cg05521261	2.15E-05	0.019484728	0.88945535	0.892756548	-0.003301198	FDR0.05	10	6208594	Body;Body;Body;Body	-14369	MIR3155B	PFKFB3
cg27144883	2.16E-05	0.019484728	0.888772338	0.898161788	-0.00938945	FDR0.05	17	13696080	Body;TSS1500;Body;Body	-213	COX10-AS1	COX10-AS1
cg07296772	2.17E-05	0.019484728	0.872355988	0.856073432	0.016282556	FDR0.05	14	24683528	Body;Body;TES5000;Body;Body;Body;TSS1500;Body;Body	-492	CHMP4A	MDP1;NEDD8;NEDD8-MDP1;CHMP4A
cg20555305	2.17E-05	0.019484728	0.670955726	0.665886815	0.005068911	FDR0.05	8	145626588	Body;TSS1500	-849	MIR6849	CPSF1;MIR6849
cg05616858	2.17E-05	0.019484728	0.751812894	0.745020164	0.00679273	FDR0.05	17	37843591	Body;TSS1500;TSS1500;Body;Body;Body;Body;Body;TSS1500	732	PGAP3	PGAP3;ERBB2
cg21985690	2.19E-05	0.019619264	0.217249991	0.178892644	0.038357347	FDR0.05	5	79330669	Body;TSS1500;Body;TSS1500	-452	THBS4	THBS4
cg22772747	2.19E-05	0.019619264	0.863272221	0.875326175	-0.012053954	FDR0.05	7	27164285	Body;TES5000;Body	2354	HOXA3	HOXA3;HOXA4;HOXA-AS2
cg18315963	2.21E-05	0.019710076	0.091753291	0.077327096	0.014426195	FDR0.05	4	30723455	1stExon;1stExon;1stExon;1stExon	1419	PCDH7	PCDH7
cg01969683	2.24E-05	0.019921077	0.832221532	0.856987124	-0.024765593	FDR0.05	8	23341028	Body	10683	CTC-756D1.3	AC104561.1
cg14601053	2.24E-05	0.019921077	0.736418617	0.748970536	-0.012551918	FDR0.05	9	130271989	Body;Body	57456	LRSAM1	FAM129B
cg13511752	2.25E-05	0.019942619	0.198519527	0.198320754	0.000198773	FDR0.05	10	74094697	3'UTR;Body;Body;Body;Body	19980	DNAJB12	DNAJB12
cg26357596	2.25E-05	0.019960893	0.132765303	0.12062211	0.012143193	FDR0.05	5	54397592	TSS1500	-881	GZMA	GZMA
cg18034577	2.26E-05	0.020018427	0.817308735	0.832272315	-0.01496358	FDR0.05	15	102345708	TSS1500	-214	OR4F6	OR4F6
cg24496563	2.27E-05	0.020039833	0.741002812	0.792635956	-0.051633144	FDR0.05	1	67842041	Body;Body;Body;Body;Body;Body;Body	54082	SERBP1	IL12RB2

#cpg	p_adj	q_val	Meth.BPD	Meth.nonBPD	dMeth	significance	Chr	pos.hg19	locations	dist2TSS	gene.nearest	gene.Illumina
cg04661457	2.28E-05	0.020039833	0.50185814	0.514041391	-0.012183251	FDR0.05	12	92531553	Body;Body;Body;TES5000;Body;Body	3936	***LINC01619***	***LINC01619;BTG1***
cg01149896	2.28E-05	0.020039833	0.059059764	0.053887862	0.005171901	FDR0.05	4	37828114	TSS200	-167	***PGM2***	***PGM2***
cg17978995	2.31E-05	0.020265529	0.818904732	0.815383775	0.003520956	FDR0.05	6	32021154	Body;Body	-7249	***TNXB***	***TNXB***
cg07438401	2.33E-05	0.020319988	0.822383042	0.845340589	-0.022957547	FDR0.05	3	67698057	Body;Body	6981	***SUCLG2***	***SUCLG2***
cg19362874	2.34E-05	0.020319988	0.875297315	0.878457916	-0.003160601	FDR0.05	6	3053558	TSS5000	-2565	***RP1-40E16.2***	***RP1-40E16.2***
cg05661968	2.34E-05	0.020319988	0.844479929	0.870714632	-0.026234702	FDR0.05	1	200808193	Body;Body;Body	-33889	***GPR25***	***CAMSAP2***
cg09616857	2.34E-05	0.020319988	0.847741444	0.851855113	-0.004113668	FDR0.05	3	169122917	Body;Body;Body;Body	-42737	***LOC105374205***	***MECOM***
cg02133716	2.34E-05	0.020319988	0.710707581	0.720130783	-0.009423202	FDR0.05	8	128981622	Body	8744	***MIR1205***	***PVT1***
cg04026747	2.35E-05	0.020319988	0.756693221	0.799971959	-0.043278738	FDR0.05	1	1197551	Body;Body;Body;Body	11683	***UBE2J2***	***UBE2J2***
cg07258149	2.35E-05	0.020319988	0.807944225	0.812549699	-0.004605474	FDR0.05	2	109940114	Body	-10033	***MIR4266***	***SH3RF3***
cg04934519	2.36E-05	0.020319988	0.778184954	0.795684205	-0.017499251	FDR0.05	1	185303447	Body	724	***GS1-279B7.1***	***GS1-279B7.1***
cg19400179	2.36E-05	0.020319988	0.559719502	0.582750033	-0.023030531	FDR0.05	3	108321607	Body	-13116	***CIP2A***	***DZIP3***
cg18254123	2.37E-05	0.020395745	0.259004715	0.229732491	0.029272224	FDR0.05	15	80544661	TSS200	-58	***CTXND1***	***CTXND1***
cg24379122	2.38E-05	0.020395745	0.800881401	0.827211384	-0.026329983	FDR0.05	1	43290402	TSS1500;Body	-846	***ERMAP***	***ERMAP***
cg15675643	2.38E-05	0.020395745	0.754735296	0.810631688	-0.055896392	FDR0.05	2	38711146	Body	31736	***LINC02613***	***LINC02613***
cg14780115	2.40E-05	0.020561645	0.859041936	0.860354237	-0.0013123	FDR0.05	6	31660861	Body;Body;Body;Body	8007	***MIR4646***	***ABHD16A***
cg18931760	2.41E-05	0.020587765	0.80269805	0.827257989	-0.024559939	FDR0.05	11	32608616	Body;Body	3304	***EIF3M***	***EIF3M***
cg13753183	2.42E-05	0.020619003	0.721977539	0.727727788	-0.005750249	FDR0.05	9	32989852	1stExon;Body;1stExon;Body;1stExon;Body;Body;Body;Body;Body;1stExon;Body	11722	***APTX***	***APTX***
cg15770553	2.43E-05	0.020629632	0.523625801	0.501992605	0.021633197	FDR0.05	2	383369		-5042	***AC105393.1***	
cg13090587	2.43E-05	0.020657095	0.791861685	0.817822263	-0.025960579	FDR0.05	4	91570154	Body;Body	413973	***CCSER1***	***CCSER1***
cg11270815	2.46E-05	0.020787201	0.055694058	0.049927732	0.005766326	FDR0.05	19	9546371	TSS200;TSS200	-117	***ZNF266***	***ZNF266***
cg25978167	2.46E-05	0.020787201	0.039631734	0.033660099	0.005971635	FDR0.05	4	57301353	Body;TSS1500;TSS1500;TSS1500;Body	449	***PPAT***	***PPAT;PAICS***
cg16918801	2.47E-05	0.020805407	0.051986417	0.040511944	0.011474473	FDR0.05	2	18742235	TES5000;TES5000;TSS1500;TES5000;Body;TES5000;Body;TES5000	-276	***RDH14***	***NT5C1B;RDH14;NT5C1B-RDH14***
cg11964338	2.48E-05	0.020848503	0.869141692	0.877568917	-0.008427225	FDR0.05	19	52847185	Body;Body;Body;TSS1500	-1496	***ZNF610***	***ZNF610***
cg01999622	2.48E-05	0.020848503	0.836182049	0.830948971	0.005233078	FDR0.05	7	157694573	Body;Body;Body;Body;Body	47297	***LOC100506585***	***PTPRN2***
cg18347238	2.49E-05	0.020848503	0.066012806	0.058002323	0.008010483	FDR0.05	16	69345951	TES5000;Body	665	***VPS4A***	***SNTB2;VPS4A***
cg02720691	2.49E-05	0.020848503	0.835455851	0.841906972	-0.006451121	FDR0.05	4	39366468	Body;Body;Body;Body	1533	***RFC1***	***RFC1***
cg05681670	2.50E-05	0.020857829	0.864344384	0.869822706	-0.005478322	FDR0.05	16	2740814	Body	8320	***KCTD5***	***KCTD5***
cg27540878	2.50E-05	0.020857829	0.7890026	0.794231129	-0.00522853	FDR0.05	1	120312730	TSS1500;TSS1500	-1175	***HMGCS2***	***HMGCS2***
cg10225362	2.51E-05	0.020857829	0.790158135	0.807740159	-0.017582024	FDR0.05	20	44743722	TSS5000;TSS5000;TSS5000;TSS5000;TSS5000;TSS5000;TSS5000;TSS5000	-3170	***CD40***	***CD40***
cg06735218	2.51E-05	0.020857829	0.831576595	0.851020841	-0.019444246	FDR0.05	11	4664117	TSS1500;TES5000	-1038	***OR51E1***	***OR51E1;OR51D1***
cg21104971	2.51E-05	0.020857829	0.762960853	0.795418837	-0.032457985	FDR0.05	4	24647197		-14281	***RP11-496D24.2***	
cg17196805	2.52E-05	0.02087535	0.793781029	0.818462054	-0.024681025	FDR0.05	17	37796442	Body;Body;Body;TES5000;TES5000;TES5000	3110	***STARD3***	***STARD3;PPP1R1B***
cg18011064	2.54E-05	0.020968209	0.833703743	0.835219585	-0.001515842	FDR0.05	11	394289	Body;5'UTR	107	***PKP3***	***PKP3***
cg09726469	2.55E-05	0.020968209	0.070193504	0.060254713	0.009938791	FDR0.05	1	150552014	TSS5000;5'UTR;5'UTR;5'UTR	200	***MCL1***	***ADAMTSL4-AS1;MCL1***
cg15678817	2.55E-05	0.020968209	0.142659702	0.128198205	0.014461497	FDR0.05	11	64621386	3'UTR;3'UTR;3'UTR	-9261	***CDC42BPG***	***EHD1***
cg27016366	2.56E-05	0.020968209	0.746496337	0.800192356	-0.053696019	FDR0.05	8	41604672	Body;Body;Body;Body;Body	50468	***ANK1***	***ANK1***
cg26293512	2.57E-05	0.020968209	0.54753497	0.577427912	-0.029892942	FDR0.05	16	58010433	1stExon;1stExon	95	***TEPP***	***TEPP***
cg12297030	2.57E-05	0.020968209	0.020229283	0.018669275	0.001560008	FDR0.05	17	43662878		-7108	***RP11-707O23.1***	
cg16905907	2.58E-05	0.020968209	0.850629512	0.862814499	-0.012184987	FDR0.05	8	93088292	Body;Body;Body;5'UTR;5'UTR;5'UTR;5'UTR;Body;5'UTR;5'UTR;5'UTR;5'UTR;Body	-13101	***RUNX1T1***	***RUNX1T1***
cg14640682	2.58E-05	0.020968209	0.042010902	0.038112704	0.003898198	FDR0.05	19	49496473	5'UTR;5'UTR;TSS1500;Body;TSS1500;TSS1500;TSS1500	137	***GYS1***	***GYS1;RUVBL2***
cg24682760	2.58E-05	0.020968209	0.90803624	0.89621107	0.011825169	FDR0.05	11	97588992		60529	***RNA5SP347***	
cg25406657	2.58E-05	0.020968209	0.753665092	0.790800785	-0.037135693	FDR0.05	15	63342033	Body;Body;TSS1500;Body;Body;Body;Body;Body;Body;Body;Body;Body;Body;Body;Body;Body;Body;Body;Body;Body	-447	***TPM1-AS***	***TPM1;TPM1-AS***
cg00592315	2.58E-05	0.020968209	0.837059049	0.867698049	-0.030639	FDR0.05	14	94384611	TSS1500;Body;TSS1500	-628	***FAM181A***	***FAM181A;FAM181A-AS1***
cg18805734	2.61E-05	0.021052446	0.657848105	0.681494041	-0.023645936	FDR0.05	12	31453901	Body;Body;Body	-23348	***FLJ13224***	***SINHCAF***
cg25958582	2.61E-05	0.021052446	0.623201582	0.638186192	-0.01498461	FDR0.05	3	50163269	Body	-24848	***RBM5-AS1***	***SEMA3F-AS1***
cg13831570	2.61E-05	0.021052446	0.866383576	0.860537648	0.005845928	FDR0.05	17	39020053	Body	3409	***KRT12***	***KRT12***
cg06448090	2.62E-05	0.021052446	0.035314477	0.031361427	0.00395305	FDR0.05	8	13424135	Body;TSS1500;Body	-216	***C8orf48***	***DLC1;C8orf48***
cg20170777	2.64E-05	0.021052446	0.727413958	0.724265	0.003148957	FDR0.05	17	13972099	Body;TSS1500	-619	***COX10***	***COX10-AS1;COX10***
cg25332717	2.64E-05	0.021052446	0.805646173	0.806179575	-0.000533402	FDR0.05	2	106020669	TSS5000;TSS5000;Body;TSS5000;TSS5000;Body;TSS5000	-4545	***FHL2***	***FHL2***
cg15203271	2.64E-05	0.021052446	0.935222325	0.9292253	0.005997025	FDR0.05	4	1203168	TES5000;TSS1500;TSS1500;TES5000;TSS1500	-418	***LOC100130872***	***CTBP1;CTBP1-AS;SPON2;LOC100130872***
cg03205495	2.64E-05	0.021052446	0.056926379	0.050368064	0.006558315	FDR0.05	19	14640249	TSS200;TSS200;TSS200;TSS200;TSS200;TSS200	-105	***MIR639***	***MIR639;DNAJB1;TECR***
cg10247878	2.64E-05	0.021052446	0.919258842	0.916100864	0.003157978	FDR0.05	20	44522910	Body;TSS5000;TSS5000;TES5000;TES5000;TES5000;Body;Body;TES5000;Body	-2984	***NEURL2***	***CTSA;NEURL2;PLTP***
cg09327847	2.65E-05	0.021052446	0.675780472	0.689977868	-0.014197396	FDR0.05	16	24087793	Body;Body	-126643	***MIR1273H***	***PRKCB***
cg19944675	2.65E-05	0.021052446	0.021598211	0.019829874	0.001768337	FDR0.05	2	118771867	TSS200	-128	***CCDC93***	***CCDC93***
cg05992340	2.66E-05	0.021052446	0.881754769	0.881692356	6.24E-05	FDR0.05	5	58882243	Body;Body;Body;5'UTR;5'UTR;Body;Body;5'UTR;Body;Body;Body;Body;Body	81	***PDE4D***	***PDE4D***

#cpg	p_adj	q_val	Meth.BPD	Meth.nonBPD	dMeth	significance	Chr	pos.hg19	locations	dist2TSS	gene.nearest	gene.Illumina
cg04225089	2.66E-05	0.021052446	0.095355623	0.105624625	-0.010269003	FDR0.05	17	73874465	1stExon	191	***TRIM47***	***TRIM47***
cg07712541	2.66E-05	0.021052446	0.445117413	0.404313118	0.040804295	FDR0.05	10	88072732	Body	-48187	***MIR346***	***GRID1***
cg11008701	2.67E-05	0.021052446	0.865358244	0.866329151	-0.000970907	FDR0.05	7	157938562	Body;Body;Body;Body;Body	291286	***LOC100506585***	***PTPRN2***
cg17100200	2.68E-05	0.021052446	0.852153696	0.843362996	0.008790701	FDR0.05	1	42618313	TSS1500	-778	***GUCA2B***	***GUCA2B***
cg00447324	2.68E-05	0.021052446	0.808906531	0.855981887	-0.047075356	FDR0.05	17	34208154	TSS1500;TSS1500	-777	***CCL5***	***CCL5***
cg27624769	2.68E-05	0.021052446	0.753727802	0.757106411	-0.003378609	FDR0.05	8	116657166	Body;Body;Body;Body	23073	***TRPS1***	***TRPS1***
cg19805160	2.68E-05	0.021052446	0.647527553	0.67762978	-0.030102227	FDR0.05	1	159870731	TSS1500;TSS1500	-825	***CFAP45***	***CFAP45;MIR4259***
cg24780759	2.70E-05	0.021137274	0.694615663	0.759630876	-0.065015212	FDR0.05	6	109712745	3'UTR;3'UTR;3'UTR;Body;3'UTR	-8730	***CD164***	***PPIL6***
cg26977759	2.71E-05	0.021137274	0.7902267	0.834968593	-0.044741893	FDR0.05	8	37518651	Body	-7764	***RNU6-607P***	***RP11-346L1.2***
cg07478100	2.71E-05	0.021137274	0.042432183	0.03928611	0.003146073	FDR0.05	17	5389857	TSS1500;TSS1500;TSS1500;TSS1500;TSS1500;TSS1500;Body;Body;5'UTR;5'UTR	164	***MIS12***	***DERL2;MIS12***
cg10868668	2.71E-05	0.021137274	0.634155794	0.680390679	-0.046234884	FDR0.05	4	38606942		14116	***RP11-617D20.2***	
cg10462803	2.73E-05	0.021224431	0.873278869	0.866205893	0.007072976	FDR0.05	16	85568220	Body	-21352	***GSE1***	***GSE1***
cg05698020	2.75E-05	0.021292944	0.861301362	0.909228693	-0.047927332	FDR0.05	13	111163978	TSS5000;Body	-3452	***COL4A2-AS1***	***COL4A2-AS1;COL4A2***
cg16879936	2.75E-05	0.021292944	0.905479831	0.908707673	-0.003227842	FDR0.05	17	15134351	Body;Body;Body;Body;Body;Body;Body;TES5000	20662	***MIR4731***	***PMP22***
cg13335691	2.76E-05	0.021292944	0.414088497	0.459394265	-0.045305769	FDR0.05	7	56242142		-58049	***NUPR2***	
cg12927772	2.76E-05	0.021292944	0.06206899	0.058481335	0.003587655	FDR0.05	9	86571585	5'UTR;Body	72	***C9orf64***	***C9orf64***
cg22128435	2.76E-05	0.021292944	0.039060519	0.032618329	0.006442191	FDR0.05	17	42766776	Body;Body	389	***CCDC43***	***CCDC43***
cg17971251	2.78E-05	0.021371518	0.713818497	0.743718296	-0.029899799	FDR0.05	1	177907297	Body;Body;Body;Body	31760	***SEC16B***	***SEC16B;CRYZL2P-SEC16B***
cg19232164	2.78E-05	0.021375564	0.769539666	0.831199973	-0.061660307	FDR0.05	11	114320724	3'UTR	10617	***REXO2***	***REXO2***
cg08444427	2.80E-05	0.021438106	0.199610301	0.162061816	0.037548485	FDR0.05	17	41987800	TSS5000;TSS5000;TSS5000;TSS1500;TSS5000;TSS5000;TSS1500	-721	***MPP2***	***MPP2***
cg19836894	2.80E-05	0.021438106	0.053687363	0.039450253	0.01423711	FDR0.05	19	44669244	TES5000;TSS200;5'UTR;TSS200;TES5000;5'UTR;5'UTR;5'UTR;TSS200;TSS200;TSS200	-4	***ZNF226***	***ZNF234;ZNF226***
cg23112950	2.81E-05	0.021447959	0.641855698	0.643770794	-0.001915096	FDR0.05	11	66369765	Body	9076	***CCS***	***CCS***
cg21041701	2.83E-05	0.021610092	0.836280273	0.853677844	-0.01739757	FDR0.05	12	31942768	TES5000	2407	***H3F3C***	***H3F3C***
cg24047689	2.84E-05	0.021644674	0.01701682	0.018515416	-0.001498596	FDR0.05	1	6614587	5'UTR;TSS1500;TSS1500	71	***NOL9***	***NOL9;TAS1R1***
cg20151221	2.86E-05	0.021739131	0.655950596	0.727709393	-0.071758797	FDR0.05	11	325030	TSS5000;TSS5000	-4116	***IFITM3***	***IFITM3***
cg13058453	2.87E-05	0.021777456	0.739968664	0.738635687	0.001332977	FDR0.05	1	85584420	Body;Body	-15056	***MIR4423***	***WDR63***
cg25124739	2.88E-05	0.021800461	0.120761922	0.102525229	0.018236693	FDR0.05	2	46527098	Body	2558	***EPAS1***	***EPAS1***
cg08150742	2.90E-05	0.021933943	0.791348733	0.792532462	-0.001183729	FDR0.05	12	49086916	Body;3'UTR	-10881	***KANSL2***	***CCNT1***
cg20689294	2.91E-05	0.021975349	0.107417254	0.092345959	0.015071295	FDR0.05	10	129846082	Body;5'UTR;Body;Body;Body;Body;Body;Body	270	***PTPRE***	***PTPRE***
cg04595323	2.92E-05	0.021975349	0.074383386	0.064304952	0.010078434	FDR0.05	17	78400892	Body;Body;Body;Body;Body;Body;Body;Body;Body;Body	7675	***MIR4730***	***ENDOV***
cg11832804	2.92E-05	0.021975349	0.940690353	0.950885236	-0.010194883	FDR0.05	5	1279449	Body;Body;Body;Body	15713	***TERT***	***TERT***
cg03788952	2.93E-05	0.021975349	0.811045021	0.83498928	-0.02394426	FDR0.05	18	55474222	TSS5000	-3675	***ATP8B1***	***ATP8B1***
cg02026141	2.93E-05	0.021975349	0.393006604	0.329579541	0.063427062	FDR0.05	10	23492470	3'UTR	11011	***PTF1A***	***C10orf67***
cg02909176	2.93E-05	0.021975349	0.839307039	0.851460698	-0.012153659	FDR0.05	13	113426343	Body;Body	-17298	***ATP11A-AS1***	***ATP11A***
cg11228758	2.94E-05	0.0220178	0.855684072	0.858929117	-0.003245045	FDR0.05	1	65976172	Body;Body;Body	-15199	***LEPR***	***LEPR***
cg02985445	2.96E-05	0.022107893	0.549851446	0.574887584	-0.025036137	FDR0.05	7	97908505	TSS5000;TSS5000	-2473	***BRI3***	***BRI3***
cg07582400	2.97E-05	0.022107893	0.902728374	0.900377053	0.002351322	FDR0.05	2	239010679	Body;TES5000;TES5000	1729	***ESPNL***	***ESPNL;UBE2F-SCLY;SCLY***
cg16900604	2.97E-05	0.022107893	0.783350883	0.805549981	-0.022199098	FDR0.05	2	208260036	Body;Body	-125888	***MIR1302-4***	***AC007879.5***
cg16194544	2.99E-05	0.022205717	0.799782898	0.79291154	0.006871358	FDR0.05	5	1484314	Body	26657	***MIR6075***	***LPCAT1***
cg15684162	3.00E-05	0.022205717	0.849741946	0.86907862	-0.019336675	FDR0.05	17	61712639	Body;Body;Body;Body	12855	***MAP3K3***	***MAP3K3***
cg03742132	3.00E-05	0.022205717	0.854724216	0.869038911	-0.014314695	FDR0.05	12	81227142	Body;Body;Body;TSS1500;Body	-734	***MIR617***	***LIN7A;MIR617***
cg20758791	3.00E-05	0.022205717	0.917303834	0.903560631	0.013743203	FDR0.05	17	21101734	Body;Body	16174	***TMEM11***	***TMEM11***
cg15952725	3.02E-05	0.022255687	0.78981192	0.818508805	-0.028696885	FDR0.05	1	15783190	TSS200	-32	***CELA2A***	***CELA2A***
cg03929043	3.02E-05	0.022255687	0.827844201	0.845121191	-0.01727699	FDR0.05	8	59463761	TSS5000;TSS5000;TSS5000;TSS5000;TSS5000;TSS5000;TSS5000;TSS5000;TSS5000	-1721	***SDCBP***	***SDCBP***
cg04276938	3.04E-05	0.022340513	0.634041937	0.666340999	-0.032299063	FDR0.05	5	40802085	TSS5000;TSS5000;TSS5000;TSS5000;TSS5000;TSS5000;TSS5000;TSS5000	-3788	***PRKAA1***	***PRKAA1***
cg10780002	3.05E-05	0.022340513	0.90323491	0.889016697	0.014218213	FDR0.05	14	103782538	TSS1500	-1082	***LOC105378183***	***LOC105378183***
cg04720865	3.05E-05	0.022340513	0.89576342	0.900263251	-0.004499831	FDR0.05	8	27948927	TSS5000;Body;TSS5000;TSS5000;TSS5000;TSS5000	1180	***ELP3***	***ELP3***
cg26267340	3.05E-05	0.022340513	0.09808967	0.111044996	-0.012955326	FDR0.05	8	31497829	1stExon	562	***NRG1***	***NRG1***
cg11081093	3.06E-05	0.022349519	0.817575916	0.84119102	-0.023615104	FDR0.05	1	117671033		-5824	***TRIM45***	
cg17617946	3.06E-05	0.022349519	0.017182286	0.01589594	0.001286346	FDR0.05	5	114879806	Body	785	***FEM1C***	***FEM1C***
cg27054847	3.10E-05	0.022604894	0.667081002	0.695219002	-0.028138	FDR0.05	6	131386869	TSS5000;TSS5000;TSS5000;TSS5000;TSS5000;TSS5000;TSS5000;TSS5000;TSS5000;TSS5000;TSS5000;TSS5000;TSS5000;TSS5000;TSS5000;TSS5000	-2407	***EPB41L2***	***EPB41L2***
cg24708145	3.11E-05	0.022633048	0.725879336	0.737678454	-0.011799119	FDR0.05	5	134780343	TES5000;3'UTR	2695	***DCANP1***	***TIFAB;DCANP1***
cg17033185	3.12E-05	0.022633048	0.85288961	0.838206391	0.014683219	FDR0.05	5	115294388	TSS5000;TES5000	2121	***LINCADL***	***LVRN;LINCADL***
cg00449210	3.12E-05	0.022641544	0.030656125	0.027172662	0.003483463	FDR0.05	7	107385198	Body;TSS200;TSS1500;TSS1500;TSS1500;Body;Body	-172	***LOC101927974***	***CBLL1;LOC101927974***
cg18975462	3.16E-05	0.022891911	0.031591343	0.028339701	0.003251642	FDR0.05	8	119123269	1stExon	789	***EXT1***	***EXT1***
cg07124719	3.17E-05	0.022891911	0.768093864	0.763876924	0.00421694	FDR0.05	4	80748552	TSS200	-72	***PCAT4***	***PCAT4***

#cpg	p_adj	q_val	Meth.BPD	Meth.nonBPD	dMeth	significance	Chr	pos.hg19	locations	dist2TSS	gene.nearest	gene.Illumina
cg01930441	3.17E-05	0.022891911	0.275514173	0.296502395	-0.020988222	FDR0.05	16	21289268	Body	-22901	***CRYM-AS1***	***CRYM***
cg01589587	3.19E-05	0.022995982	0.697601006	0.756990375	-0.059389369	FDR0.05	14	76002440	Body	13657	***BATF***	***BATF***
cg17116895	3.22E-05	0.023152313	0.824387872	0.814761214	0.009626658	FDR0.05	6	112133054	Body	-52720	***FYN***	***FYN***
cg16077055	3.23E-05	0.023152313	0.715902616	0.782164281	-0.066261664	FDR0.05	2	106428750	Body;Body	-39453	***NCK2***	***NCK2***
cg00478326	3.23E-05	0.023152313	0.559031921	0.515627386	0.043404535	FDR0.05	15	78978136	Body;TES5000	22258	***CHRNB4***	***CHRNB4***
cg17825770	3.24E-05	0.023197624	0.838209715	0.851936722	-0.013727007	FDR0.05	8	116573953	Body;Body;Body;Body	106286	***TRPS1***	***TRPS1***
cg11064255	3.27E-05	0.023379588	0.025031068	0.020721918	0.00430915	FDR0.05	18	5629890	TSS1500	-900	***EPB41L3***	***EPB41L3***
cg24669602	3.29E-05	0.023436084	0.716717606	0.678582153	0.038135453	FDR0.05	7	77426814	TSS1500;TSS1500;Body;TSS1500;TSS1500;TSS1500;TSS1500;TSS1500;TSS1500;TSS1500;TSS1500	933	***TMEM60***	***PHTF2;TMEM60***
cg17336354	3.32E-05	0.023590227	0.816783637	0.844125546	-0.027341909	FDR0.05	3	34298905	Body;Body;Body	54170	***AC123023.1***	***AC018359.1;AC123023.1***
cg11926444	3.32E-05	0.023590227	0.721082835	0.815660971	-0.094578136	FDR0.05	3	119137289	3'UTR	45240	***TMEM39A***	***ARHGAP31***
cg08927631	3.33E-05	0.023590227	0.745715664	0.80797633	-0.062260666	FDR0.05	5	36237922	Body;Body;Body;Body	3571	***NADK2***	***NADK2***
cg02166383	3.34E-05	0.023590227	0.34868683	0.363624041	-0.014937211	FDR0.05	14	107252649		6565	***MIR5195***	
cg19126492	3.35E-05	0.023590227	0.95385693	0.967967529	-0.014110599	FDR0.05	2	11264462	Body	7840	***FLJ33534***	***FLJ33534***
cg12033214	3.35E-05	0.023590227	0.742851859	0.755742076	-0.012890217	FDR0.05	22	27017910	TSS200;TSS5000	-17	***CRYBA4***	***CRYBA4;CRYBB1***
cg01782059	3.35E-05	0.023590227	0.786203174	0.846107352	-0.059904178	FDR0.05	1	44715942	Body;Body;Body;TES5000;Body;Body	2289	***SNORA110***	***ERI3;SNORA110***
cg08012278	3.35E-05	0.023590227	0.048352589	0.035561161	0.012791428	FDR0.05	2	237068350		-6534	***Y_RNA***	
cg26522239	3.36E-05	0.023590227	0.117245325	0.103055229	0.014190096	FDR0.05	18	21199097	Body;Body	-32516	***NPC1***	***ANKRD29***
cg04023434	3.37E-05	0.023590227	0.877561389	0.887358325	-0.009796936	FDR0.05	1	183778265	Body;Body;Body;Body;Body	4051	***RGL1***	***RGL1***
cg20300315	3.37E-05	0.023590227	0.734968915	0.742887228	-0.007918313	FDR0.05	2	65051180		6669	***RN7SL341P***	
cg12213687	3.37E-05	0.023590227	0.889730032	0.880653609	0.009076423	FDR0.05	13	110802749	Body	28701	***LOC105370361***	***COL4A1***
cg18728325	3.38E-05	0.023616584	0.048713171	0.038378397	0.010334775	FDR0.05	19	44598784	Body;Body	303	***ZNF224***	***ZNF224***
cg27550595	3.39E-05	0.023616584	0.842128091	0.85541238	-0.013284289	FDR0.05	8	4976707		9227	***SNORA70***	
cg24998879	3.39E-05	0.023616584	0.898800396	0.901808446	-0.003008051	FDR0.05	6	113971911	TSS1500	-634	***LINC02541***	***LINC02541***
cg20070768	3.41E-05	0.023726913	0.641664687	0.640775168	0.00088952	FDR0.05	15	74285128	TSS1500;Body;Body;TSS5000;TSS5000;TSS5000;TSS1500;TSS1500;TSS1500;TSS1500;Body;TSS1500;TSS1500;TSS1500;TSS5000;TSS5000;TSS5000;TSS1500;TSS5000;TSS5000;TSS5000	-439	***STOML1***	***STOML1;PML***
cg26471058	3.43E-05	0.023786489	0.764897293	0.776996628	-0.012099334	FDR0.05	2	31030279	5'UTR	32	***CAPN13***	***CAPN13***
cg15465990	3.44E-05	0.023786489	0.842281335	0.840245013	0.002036322	FDR0.05	1	204394661	Body	-13716	***PPP1R15B***	***PIK3C2B***
cg08229136	3.44E-05	0.023786489	0.770736783	0.768835445	0.001901338	FDR0.05	17	78410117	3'UTR;3'UTR;Body;Body;Body;Body;Body	16900	***MIR4730***	***ENDOV***
cg04492847	3.45E-05	0.023786489	0.901902301	0.904258865	-0.002356564	FDR0.05	16	28550525	TSS200;TSS200	-30	***NUPR1***	***NUPR1***
cg03150857	3.46E-05	0.023786489	0.115564204	0.088212912	0.027351292	FDR0.05	7	106300577	3'UTR	1057	***CCDC71L***	***CCDC71L***
cg08067346	3.48E-05	0.023786489	0.771435923	0.761979048	0.009456875	FDR0.05	16	25011481	Body;Body	15218	***ARHGAP17***	***ARHGAP17***
cg06882533	3.48E-05	0.023786489	0.803436599	0.819985113	-0.016548514	FDR0.05	13	24625581	Body	71817	***SPATA13***	***SPATA13***
cg12081253	3.49E-05	0.023786489	0.854324049	0.871417341	-0.017093292	FDR0.05	17	71431773	Body	-78048	***LOC101928251***	***SDK2***
cg07870528	3.49E-05	0.023786489	0.730802277	0.736736791	-0.005934514	FDR0.05	3	12913423	TES5000	13287	***RP11-767C1.1***	***CAND2***
cg21252175	3.50E-05	0.023786489	0.712249041	0.710900302	0.001348739	FDR0.05	15	64973903	3'UTR	21577	***OAZ2***	***ZNF609***
cg09934309	3.50E-05	0.023786489	0.772891316	0.796492466	-0.023601149	FDR0.05	16	23461721	Body	2791	***COG7***	***COG7***
cg09500328	3.50E-05	0.023786489	0.075290648	0.068255852	0.007034796	FDR0.05	20	33413629	TSS5000;TSS200	-177	***NCOA6***	***NCOA6***
cg17057501	3.50E-05	0.023786489	0.823301217	0.84742715	-0.024125933	FDR0.05	10	64017641	Body;Body	10981	***RTKN2***	***RTKN2***
cg09430664	3.51E-05	0.023786489	0.663060937	0.637443589	0.025617348	FDR0.05	6	166908758	Body;Body;Body;Body;Body	14163	***MIR1913***	***RPS6KA2***
cg22389209	3.51E-05	0.023786489	0.851545262	0.846856708	0.004688555	FDR0.05	7	155587335		12729	***SHH***	
cg07589991	3.52E-05	0.023786489	0.322152877	0.281550031	0.040602846	FDR0.05	1	9847879	Body;Body;Body	36705	***CLSTN1***	***CLSTN1***
cg15834202	3.52E-05	0.023786489	0.483951039	0.43226088	0.051690159	FDR0.05	5	58865918	Body;Body;Body;Body;Body;Body;Body;Body;Body;Body;Body;Body;Body	16406	***PDE4D***	***PDE4D***
cg10735633	3.52E-05	0.023786489	0.034925991	0.025632766	0.009293225	FDR0.05	14	51562026	5'UTR;5'UTR	396	***TRIM9***	***TRIM9***
cg17803965	3.52E-05	0.023786489	0.592497251	0.602437204	-0.009939953	FDR0.05	1	32281812	TSS200;TSS200;TSS200	-160	***SPOCD1***	***SPOCD1***
cg20840540	3.55E-05	0.023899273	0.670030445	0.690550551	-0.020520107	FDR0.05	6	42363749	Body;Body	56040	***TRERF1***	***TRERF1***
cg06699491	3.55E-05	0.023899273	0.771351236	0.769338798	0.002012437	FDR0.05	2	121560593	Body	5727	***GLI2***	***GLI2***
cg06070229	3.56E-05	0.023928599	0.793050419	0.797099345	-0.004048926	FDR0.05	7	133348826	Body	160815	***LOC101928861***	***EXOC4***
cg11797485	3.58E-05	0.024015045	0.844156736	0.852594304	-0.008437568	FDR0.05	15	69107085	Body	6176	***ANP32A***	***ANP32A***
cg15996284	3.58E-05	0.024015045	0.936626661	0.950014151	-0.01338749	FDR0.05	7	156296906	Body;Body	-36278	***LINC00244***	***LINC01006***
cg15756055	3.59E-05	0.024015045	0.736590235	0.754487389	-0.017897154	FDR0.05	2	39645955	Body;Body	18498	***MAP4K3***	***MAP4K3***
cg15864104	3.60E-05	0.024035314	0.883245421	0.914438879	-0.031193458	FDR0.05	18	55254508	TSS1500;TSS1500	-574	***FECH***	***FECH***
cg12776287	3.61E-05	0.024035314	0.710923541	0.7444704	-0.033546859	FDR0.05	18	24125939	Body;Body;Body;Body;Body;Body	2561	***KCTD1***	***KCTD1***
cg14670380	3.62E-05	0.024035314	0.03751722	0.032737382	0.004779838	FDR0.05	2	86789845	Body;Body;Body;Body;Body	775	***CHMP3***	***CHMP3;RNF103-CHMP3***
cg05647526	3.62E-05	0.024035314	0.033104293	0.029843988	0.003260305	FDR0.05	7	128379677	Body;Body;Body;Body;Body;Body	332	***CALU***	***CALU***
cg16592586	3.62E-05	0.024035314	0.138698698	0.110634468	0.02806423	FDR0.05	4	175751001	TSS1500;TSS5000;TSS1500	-535	***GLRA3***	***GLRA3;LOC101928551***
cg23313445	3.62E-05	0.024053648	0.865802688	0.881459103	-0.015656416	FDR0.05	11	4792272	TSS1500	-1125	***OR51F1***	***OR51F1***
cg26789779	3.64E-05	0.024097566	0.020118545	0.015864585	0.00425396	FDR0.05	5	37839943	TSS5000;TSS5000;TSS200;TSS1500;TSS5000;TSS200	-42	***GDNF-AS1***	***GDNF;GDNF-AS1***
cg00944309	3.64E-05	0.024097566	0.776210199	0.762514431	0.013695768	FDR0.05	5	60142446	TSS5000;TSS5000;TSS5000;TSS5000	-2345	***ELOVL7***	***ELOVL7***

#cpg	p_adj	q_val	Meth.BPD	Meth.nonBPD	dMeth	significance	Chr	pos.hg19	locations	dist2TSS	gene.nearest	gene.Illumina
cg01953591	3.67E-05	0.024267629	0.082574127	0.074377147	0.00819698	FDR0.05	1	169555805	TES5000;TSS200	-36	***F5***	***SELP;F5***
cg06367607	3.70E-05	0.024396364	0.735582304	0.771614698	-0.036032394	FDR0.05	6	106603209	Body;Body	47095	***ATG5***	***ATG5***
cg10790709	3.71E-05	0.024413255	0.707130604	0.748513318	-0.041382714	FDR0.05	12	95698084	TES5000;TES5000;TES5000;TSS5000;TES5000	-4111	***MIR331***	***VEZT;MIR331***
cg08724563	3.71E-05	0.024413255	0.036636386	0.032175031	0.004461355	FDR0.05	18	56940755	TSS200	-130	***RAX***	***RAX***
cg01722297	3.72E-05	0.024413255	0.451616329	0.426251277	0.025365051	FDR0.05	17	46190702	Body;Body;Body	5756	***SNX11***	***SNX11***
cg00031708	3.73E-05	0.024467907	0.736657406	0.736870096	-0.00021269	FDR0.05	7	23051361	Body;Body;Body	2388	***FAM126A***	***FAM126A***
cg00256675	3.75E-05	0.024496736	0.90588461	0.901565999	0.004318611	FDR0.05	4	802962	Body	16983	***CPLX1***	***CPLX1***
cg22491409	3.75E-05	0.024496736	0.130244578	0.103833072	0.026411506	FDR0.05	12	125198295		-11774	***RP11-13J12.2***	
cg00756852	3.75E-05	0.024496736	0.035161991	0.028676691	0.0064853	FDR0.05	1	155197384	TSS200	-59	***GBAP1***	***GBAP1***
cg24691255	3.76E-05	0.024496736	0.869170633	0.868930533	0.000240099	FDR0.05	18	61553966	TSS1500;TSS1500	-972	***SERPINB2***	***SERPINB2***
cg07697465	3.76E-05	0.024497168	0.722123494	0.706875892	0.015247602	FDR0.05	16	1516049	Body;Body	9036	***CLCN7***	***CLCN7***
cg24143196	3.78E-05	0.024586319	0.743912612	0.801636648	-0.057724035	FDR0.05	19	14644474	TES5000;TSS5000;Body;Body;Body;Body	4096	***TECR***	***MIR639;DNAJB1;TECR***
cg26987453	3.79E-05	0.024586319	0.838636284	0.845895855	-0.007259571	FDR0.05	8	8652379	Body	92714	***CLDN23***	***MFHAS1***
cg19829477	3.79E-05	0.024586319	0.854342698	0.874155426	-0.019812728	FDR0.05	7	93775464		-68995	***AC003092.2***	
cg17384769	3.84E-05	0.024847698	0.831375554	0.841546963	-0.010171409	FDR0.05	6	149336332	Body	17465	***LOC105378047***	***UST***
cg11076862	3.86E-05	0.024917095	0.040582608	0.033173638	0.007408971	FDR0.05	22	18256616	Body;Body;5'UTR;Body;Body;TSS5000;Body;5'UTR	192	***BID***	***BID;LINC00528***
cg06822690	3.87E-05	0.024917095	0.224940326	0.252528916	-0.02758859	FDR0.05	1	82040009	Body;Body;Body	-16622	***LOC101927434***	***ADGRL2***
cg07380021	3.87E-05	0.024917095	0.244519015	0.229346127	0.015172887	FDR0.05	1	206755252	Body;Body;Body	24761	***RASSF5***	***RASSF5***
cg17116500	3.88E-05	0.024917095	0.799627604	0.814483578	-0.014855974	FDR0.05	12	56600222	Body;3'UTR;3'UTR;3'UTR;3'UTR	15263	***RNF41***	***RNF41***
cg03797398	3.88E-05	0.024917095	0.059415883	0.054653462	0.004762421	FDR0.05	4	2043053	TSS1500;TSS1500	-666	***C4orf48***	***C4orf48***
cg17486234	3.88E-05	0.024917095	0.853928332	0.868220068	-0.014291736	FDR0.05	8	41140500	Body	-11838	***MIR548AO***	***SFRP1***
cg18196829	3.89E-05	0.024931257	0.926725902	0.942378911	-0.01565301	FDR0.05	6	170494301	Body	397	***RP11-302L19.3***	***RP11-302L19.3***
cg02002164	3.92E-05	0.025055748	0.825867803	0.820791399	0.005076405	FDR0.05	11	19223517	5'UTR	8601	***CSRP3***	***CSRP3***
cg04232816	3.92E-05	0.025055748	0.743578679	0.722313996	0.021264683	FDR0.05	2	96905390	Body	25014	***SNORA112***	***STARD7-AS1***
cg14750144	3.94E-05	0.025130395	0.933305513	0.92636497	0.006940543	FDR0.05	4	166256518	Body;Body	7701	***MSMO1***	***MSMO1***
cg14993430	3.97E-05	0.025225423	0.896480197	0.910109856	-0.013629658	FDR0.05	3	193499482		-40972	***RN7SL447P***	
cg01708202	3.97E-05	0.025225423	0.824782623	0.826149141	-0.001366518	FDR0.05	2	1241690	Body	-175542	***TPO***	***SNTG2***
cg14972564	3.97E-05	0.025225423	0.828332301	0.831545287	-0.003212986	FDR0.05	3	127935096	Body	-62339	***RUVBL1***	***EEFSEC***
cg13053150	3.98E-05	0.025225423	0.767272916	0.778387839	-0.011114923	FDR0.05	6	122929007	Body;Body;TSS5000;Body;TSS5000	-2369	***PKIB***	***PKIB***
cg07921371	3.99E-05	0.025271553	0.051081896	0.043341597	0.007740299	FDR0.05	13	20768536	TSS1500	-1422	***GJB2***	***GJB2***
cg23843022	4.01E-05	0.02532535	0.801574432	0.829934195	-0.028359762	FDR0.05	1	208444590		-26925	***PLXNA2***	
cg11185991	4.01E-05	0.02532535	0.775377456	0.794557606	-0.01918015	FDR0.05	8	98194116	Body	96060	***TSPYL5***	***LOC101927066***
cg08491681	4.01E-05	0.02532535	0.80367235	0.815521903	-0.011849553	FDR0.05	13	33781288	TSS1500;Body;Body;TSS1500	-1101	***STARD13***	***STARD13***
cg17521134	4.04E-05	0.025405041	0.777139578	0.81719805	-0.040058472	FDR0.05	15	88120995	Body	836	***LINC00052***	***LINC00052***
cg15913157	4.04E-05	0.025405041	0.646490547	0.698848594	-0.052358046	FDR0.05	2	206223286	Body;Body;Body;Body	-323937	***NRP2***	***PARD3B***
cg23989635	4.04E-05	0.025405041	0.069203015	0.063304777	0.005898238	FDR0.05	16	68771203	5'UTR;5'UTR;5'UTR;5'UTR	11	***CDH1***	***CDH1***
cg21115691	4.06E-05	0.025492571	0.136863309	0.13437899	0.002484319	FDR0.05	1	207140927	Body;Body;Body	3043	***FCAMR***	***FCAMR***
cg04172345	4.07E-05	0.025495772	0.811164568	0.818897241	-0.007732674	FDR0.05	17	146926	Body;Body;Body;Body	-34069	***LOC100506388***	***RPH3AL***
cg03983213	4.08E-05	0.025522814	0.06742457	0.069868958	-0.002444388	FDR0.05	7	157475737	Body;Body;Body;Body;Body	-108623	***MIR153-2***	***PTPRN2***
cg16377117	4.09E-05	0.025522814	0.710135922	0.742898048	-0.032762126	FDR0.05	12	57526305	Body	4024	***LRP1***	***LRP1***
cg02393907	4.09E-05	0.025522814	0.759576977	0.766152932	-0.006575954	FDR0.05	7	150011063	Body;Body;Body;Body;Body	4476	***LRRC61***	***LRRC61;ACTR3C***
cg02348430	4.10E-05	0.025522814	0.824838721	0.857682189	-0.032843468	FDR0.05	10	60567288	Body	92514	***FAM133CP***	***BICC1***
cg06690548	4.10E-05	0.025526958	0.903129048	0.894493987	0.00863506	FDR0.05	4	139162808	Body	695	***SLC7A11***	***SLC7A11***
cg11142525	4.12E-05	0.025608499	0.90843374	0.90845241	-1.87E-05	FDR0.05	17	36553434	Body	-31285	***ARHGAP23***	***SOCS7***
cg05215304	4.13E-05	0.025634362	0.840441578	0.834571302	0.005870277	FDR0.05	2	101402664		-33948	***NPAS2***	
cg00988227	4.14E-05	0.025634362	0.807670612	0.841350936	-0.033680324	FDR0.05	5	40693569	3'UTR	13538	***PTGER4***	***PTGER4***
cg06193382	4.14E-05	0.025634362	0.072185827	0.063890604	0.008295223	FDR0.05	6	136571195	Body;5'UTR;Body	278	***MTFR2***	***MTFR2***
cg12656272	4.16E-05	0.025678591	0.204482329	0.202812717	0.001669612	FDR0.05	3	100328186	TSS1500	-246	***ADGRG7***	***ADGRG7***
cg06637812	4.16E-05	0.025678591	0.314369828	0.27237222	0.041997608	FDR0.05	1	203293433		-5206	***RNU6-487P***	
cg20506184	4.18E-05	0.025762739	0.044356088	0.039288722	0.005067365	FDR0.05	10	7453329	5'UTR;TSS5000	119	***SFMBT2***	***SFMBT2***
cg16172619	4.20E-05	0.025823865	0.813423421	0.83624562	-0.022822199	FDR0.05	20	19197870	Body	4581	***SLC24A3***	***SLC24A3***
cg04307795	4.21E-05	0.025886984	0.894924187	0.88024211	0.014682077	FDR0.05	10	130281583		-163105	***RP11-264E18.1***	
cg00007239	4.23E-05	0.025925268	0.823355144	0.848943083	-0.025587939	FDR0.05	4	165877045	TSS1500;Body	-1054	***FAM218A***	***FAM218A;TRIM61***
cg18128546	4.23E-05	0.025925268	0.064863279	0.059319089	0.00554419	FDR0.05	1	113162116	5'UTR;TSS1500;TSS200;TSS200;TSS200;TSS200	42	***CAPZA1***	***CAPZA1;ST7L***
cg13696605	4.26E-05	0.026062079	0.767199801	0.790604143	-0.023404342	FDR0.05	9	36169203	TSS200	-185	***CCIN***	***CCIN***

#cpg	p_adj	q_val	Meth.BPD	Meth.nonBPD	dMeth	significance	Chr	pos.hg19	locations	dist2TSS	gene.nearest	gene.Illumina
cg17460285	4.27E-05	0.026062079	0.163281794	0.194328002	-0.031046208	FDR0.05	17	40985453	Body;5'UTR;5'UTR;5'UTR;5'UTR	40	***PSME3***	***PSME3***
cg14580853	4.28E-05	0.026062079	0.666576189	0.70306061	-0.036484421	FDR0.05	6	136680107	Body;Body;Body;Body;Body;Body;Body;Body;Body;Body	-69118	***BCLAF1***	***MAP7***
cg10298741	4.28E-05	0.026062079	0.747279354	0.744857699	0.002421655	FDR0.05	16	73019173	Body;Body	63101	***ZFHX3***	***ZFHX3***
cg20460166	4.29E-05	0.026087006	0.646003652	0.667760715	-0.021757063	FDR0.05	1	54423202	Body;Body	11204	***LRRC42***	***LRRC42***
cg08377819	4.31E-05	0.026099644	0.031354267	0.035550953	-0.004196686	FDR0.05	6	28956268	Body;Body	2644	***HCG15***	***HCG15***
cg25926124	4.31E-05	0.026099644	0.943287782	0.933988513	0.009299269	FDR0.05	17	48445371	3'UTR	5191	***MRPL27***	***MRPL27***
cg06700234	4.32E-05	0.026099644	0.80571618	0.800149778	0.005566402	FDR0.05	8	145642243	TSS1500;TSS5000;5'UTR	36	***SLC39A4***	***SLC39A4***
cg11689994	4.32E-05	0.026099644	0.790379231	0.793304314	-0.002925083	FDR0.05	10	63887754		11781	***AL671972.1***	
cg04993089	4.32E-05	0.026099644	0.039858285	0.036176238	0.003682046	FDR0.05	4	185089919	Body	49195	***ENPP6***	***ENPP6***
cg23013029	4.33E-05	0.026105495	0.233498327	0.189902684	0.043595643	FDR0.05	1	17508341		-7936	***RP11-380J14.1***	
cg03021496	4.33E-05	0.026105495	0.851885397	0.878222688	-0.026337291	FDR0.05	6	71997462	TSS1500;TSS1500	-682	***OGFRL1***	***OGFRL1***
cg10896604	4.35E-05	0.026180293	0.849181563	0.839654408	0.009527155	FDR0.05	11	6449300	TES5000	-8598	***APBB1***	***HPX***
cg02077653	4.38E-05	0.026285322	0.018089	0.016088035	0.002000965	FDR0.05	14	88851838	TSS200;TSS200	-149	***SPATA7***	***SPATA7***
cg13234415	4.39E-05	0.026285322	0.099083411	0.074149987	0.024933424	FDR0.05	12	65175168	Body;Body	196	***TBC1D30***	***TBC1D30***
cg18543142	4.39E-05	0.026285322	0.794241032	0.826818632	-0.0325776	FDR0.05	3	23137484		106540	***AC135966.1***	
cg19959477	4.40E-05	0.026285322	0.752473538	0.803630401	-0.051156863	FDR0.05	12	52610273	Body	5560	***LINC00592***	***LINC00592***
cg06916706	4.40E-05	0.026285322	0.035650607	0.025008317	0.010642289	FDR0.05	16	4465613	Body;Body;Body;Body;Body	321	***CORO7***	***CORO7;CORO7-PAM16***
cg07149267	4.43E-05	0.026428642	0.702085506	0.729337415	-0.027251909	FDR0.05	8	22460373	TES5000;TSS5000;TSS5000;TSS5000;Body;Body;TSS5000;Body	-1771	***CCAR2***	***PDLIM2;CCAR2;C8orf58***
cg02104952	4.47E-05	0.026682202	0.559795003	0.575170178	-0.015375175	FDR0.05	1	201481480	TSS5000;TSS5000;Body	-2896	***CSRP1***	***CSRP1;RP11-134G8.7***
cg06846898	4.48E-05	0.02669407	0.747306071	0.744804887	0.002501184	FDR0.05	9	34729535	5'UTR	0	***FAM205A***	***FAM205A***
cg21382800	4.49E-05	0.02669407	0.869065097	0.871354715	-0.002289618	FDR0.05	22	29661771	TSS5000;TSS5000;Body;Body;TSS5000;Body;TSS5000;TSS5000	2213	***RHBDD3***	***EWSR1;RHBDD3***
cg11388547	4.49E-05	0.026694195	0.723373645	0.730172009	-0.006798364	FDR0.05	8	29778333	TSS1500	-695	***LINC02209***	***LINC02209***
cg18115064	4.51E-05	0.026772353	0.070556327	0.062772341	0.007783985	FDR0.05	14	48143618	Body	539	***MDGA2***	***MDGA2***
cg05484879	4.53E-05	0.026814795	0.038311684	0.033271689	0.005039996	FDR0.05	5	126113670	Body;TSS1500;Body;Body	826	***LMNB1***	***LMNB1;LMNB1-DT***
cg06087185	4.55E-05	0.026885095	0.01978507	0.016965616	0.002819454	FDR0.05	6	29521499	TES5000	6203	***UBD***	***UBD***
cg12307818	4.55E-05	0.026885095	0.846052442	0.857552679	-0.011500236	FDR0.05	20	32848271	1stExon	101	***ASIP***	***ASIP***
cg20410192	4.58E-05	0.027001199	0.053984444	0.04669816	0.007286284	FDR0.05	4	185746558	Body;Body	710	***ACSL1***	***ACSL1***
cg09914933	4.58E-05	0.027001199	0.098478091	0.088950445	0.009527646	FDR0.05	13	27936777		-10707	***RP11-125I23.3***	
cg06321213	4.60E-05	0.027074296	0.813135361	0.81351333	-0.000377969	FDR0.05	16	86009621	Body	-5734	***RP11-542M13.2***	***RP11-542M13.6***
cg00950381	4.70E-05	0.027625672	0.869443334	0.895415849	-0.025972515	FDR0.05	17	74272364	Body;Body;TES5000	11079	***UBALD2***	***QRICH2;UBALD2***
cg27058497	4.74E-05	0.027840919	0.381237225	0.347289198	0.033948026	FDR0.05	1	25291546	TSS200;TSS200	-45	***RUNX3***	***RUNX3***
cg09130535	4.76E-05	0.027872872	0.101098261	0.096872687	0.004225574	FDR0.05	11	67039119	Body	5249	***GRK2***	***GRK2***
cg02972715	4.77E-05	0.027872872	0.896350995	0.930342832	-0.033991837	FDR0.05	14	24039333	TSS5000;TSS5000;TSS5000;Body;TSS5000;TSS5000;TSS5000;Body;TES5000;TSS5000;TSS5000;TSS5000;TSS5000	-2288	***AP1G2***	***AP1G2;JPH4;LOC102724814***
cg05428452	4.77E-05	0.027872872	0.859661845	0.835676229	0.023985617	FDR0.05	6	32712979	TES5000;Body	3817	***HLA-DQA2***	***MIR3135B;HLA-DQA2***
cg05346380	4.77E-05	0.027872872	0.043194204	0.039250806	0.003943398	FDR0.05	19	47363358		-9106	***AP2S1***	
cg18424134	4.78E-05	0.027874223	0.019369238	0.016944179	0.002425058	FDR0.05	6	126068038	TSS5000	-2693	***HEY2***	***HEY2***
cg14802312	4.80E-05	0.02788518	0.216542159	0.178394904	0.038147255	FDR0.05	12	57873704	TSS200;TSS5000;TSS1500;TSS5000;TSS5000;Body	-49	***ARHGAP9***	***ARHGAP9***
cg10749912	4.81E-05	0.02788518	0.082396089	0.070606736	0.011789353	FDR0.05	10	99531934	TSS200	-178	***SFRP5***	***SFRP5***
cg12074707	4.81E-05	0.02788518	0.03294254	0.03053901	0.002403531	FDR0.05	1	27019787	Body;TSS5000;TSS5000	835	***LOC101928728***	***LOC101928728;ARID1A***
cg01964562	4.82E-05	0.02788518	0.737428585	0.745659934	-0.008231349	FDR0.05	20	25130549	TSS1500;TSS5000;TSS1500	-1123	***LOC284798***	***LOC284798***
cg04214629	4.83E-05	0.02788518	0.888719308	0.894533209	-0.005813901	FDR0.05	7	710833	Body;Body;Body;Body;Body;Body	41328	***PRKAR1B***	***PRKAR1B***
cg20519944	4.85E-05	0.02788518	0.658446655	0.671160532	-0.012713877	FDR0.05	7	157412886	Body;Body;Body;Body;Body	-45772	***MIR153-2***	***PTPRN2***
cg03638781	4.85E-05	0.02788518	0.280166291	0.242963666	0.037202625	FDR0.05	16	23880664	Body;Body	33365	***PRKCB***	***PRKCB***
cg04690289	4.85E-05	0.02788518	0.029471398	0.024908479	0.00456292	FDR0.05	3	45017999	TSS1500;TSS1500;TSS1500;Body;TSS1500;TSS1500;Body;TSS1500;TSS1500;TSS1500;TSS1500;TSS1500;TSS1500;TSS1500	259	***EXOSC7***	***ZDHHC3;EXOSC7***
cg24176601	4.85E-05	0.02788518	0.903209903	0.891298418	0.011911484	FDR0.05	10	122670471	TES5000	-59780	***WDR11-AS1***	***WDR11***
cg18931108	4.86E-05	0.02788518	0.039548086	0.035583389	0.003964697	FDR0.05	10	52383877	TSS1500;TSS200	-140	***SGMS1***	***SGMS1-AS1;SGMS1***
cg08942192	4.88E-05	0.02788518	0.913745311	0.870860305	0.042885006	FDR0.05	1	108735334	5'UTR;Body	97	***SLC25A24***	***SLC25A24***
cg26777475	4.88E-05	0.02788518	0.774100288	0.771676145	0.002424144	FDR0.05	7	100199952	Body;TES5000;5'UTR;TES5000;TES5000	71	***PCOLCE***	***PCOLCE-AS1;FBXO24;PCOLCE***
cg21400549	4.88E-05	0.02788518	0.287652896	0.285025502	0.002627393	FDR0.05	1	212588848	TSS1500;TSS1500	-581	***TMEM206***	***TMEM206***
cg00690392	4.89E-05	0.02788518	0.874349652	0.877287065	-0.002937413	FDR0.05	1	225697019	Body;Body	-80462	***LBR***	***ENAH***
cg10692528	4.89E-05	0.02788518	0.749411347	0.766700458	-0.017289111	FDR0.05	12	6605071	Body;TSS5000	1774	***NCAPD2***	***NCAPD2;MRPL51***
cg20815297	4.89E-05	0.02788518	0.102382414	0.09339254	0.008989873	FDR0.05	4	13541663	TES5000	4451	***NKX3-2***	***NKX3-2***
cg23036947	4.89E-05	0.02788518	0.170662705	0.163374734	0.007287971	FDR0.05	1	1294018	5'UTR;Body;Body;TSS200;5'UTR	-91	***MXRA8***	***MXRA8***
cg10402121	4.89E-05	0.02788518	0.624471417	0.637066237	-0.01259482	FDR0.05	10	126487101	TSS5000	-3252	***ABRAXAS2***	***ABRAXAS2***
cg16789995	4.92E-05	0.027905048	0.781250836	0.801284255	-0.02003342	FDR0.05	12	13152456	Body;Body;TSS1500	787	***HEBP1***	***HEBP1;GPRC5D-AS1;HTR7P1***
cg00953201	4.92E-05	0.027905048	0.758430366	0.793722578	-0.035292212	FDR0.05	13	30156486	Body	13339	***SLC7A1***	***SLC7A1***
cg11137709	4.92E-05	0.027905048	0.863679347	0.857368303	0.006311043	FDR0.05	14	21508979	Body;TSS1500	-1405	***RNASE7***	***NDRG2;RNASE7***
cg22379472	4.92E-05	0.027905048	0.724373246	0.777867965	-0.053494719	FDR0.05	7	47413617	Body	90784	***SNORD151***	***TNS3***

#cpg	p_adj	q_val	Meth.BPD	Meth.nonBPD	dMeth	significance	Chr	pos.hg19	locations	dist2TSS	gene.nearest	gene.Illumina
cg02921904	4.94E-05	0.027941496	0.737981203	0.756847575	-0.018866372	FDR0.05	3	49503589	TSS5000;TSS5000;TSS5000;TSS5000;TSS5000;TSS5000;TSS5000;TSS5000;TSS5000;TSS5000;TSS5000;TSS5000;TSS5000	-2546	***DAG1***	***DAG1***
cg02273889	4.95E-05	0.027995398	0.86117194	0.884866612	-0.023694672	FDR0.05	5	123941379		38651	***ZNF608***	
cg14021714	4.96E-05	0.027995398	0.905754521	0.918673236	-0.012918715	FDR0.05	12	3757418	Body;3'UTR	104948	***CRACR2A***	***CRACR2A***
cg24225360	4.97E-05	0.028006683	0.078165462	0.056020992	0.022144471	FDR0.05	4	15471699	Body;Body;Body	211	***CC2D2A***	***CC2D2A***
cg05878786	4.97E-05	0.028008615	0.811699814	0.817942193	-0.006242379	FDR0.05	2	192701329	Body	10677	***CAVIN2***	***CAVIN2***
cg16758037	4.98E-05	0.02801787	0.043698832	0.033365252	0.010333581	FDR0.05	16	19980811		78989	***AC134300.1***	
cg05208056	4.99E-05	0.02801787	0.834561202	0.849940723	-0.015379521	FDR0.05	2	106502915	Body;Body;Body	34712	***NCK2***	***NCK2***
cg02364379	5.00E-05	0.028039926	0.889884665	0.900112456	-0.010227791	FDR0.05	4	159083821	Body;Body;Body	-8082	***FAM198B-AS1***	***FAM198B***
cg17862135	5.01E-05	0.02809137	0.874881368	0.883730031	-0.008848663	FDR0.05	2	151331573	Body;Body	12636	***RND3***	***RND3***
cg03041532	5.03E-05	0.028147002	0.687257271	0.71284578	-0.02558851	FDR0.05	11	71711021	TES5000;TES5000;TES5000;5'UTR;TES5000;5'UTR;5'UTR;TES5000;5'UTR;5'UTR;5'UTR;5'UTR;TES5000;TES5000	49	***IL18BP***	***NUMA1;RNF121;IL18BP***
cg14571125	5.06E-05	0.02827691	0.840900198	0.82612454	0.014775658	FDR0.05	6	32796739	Body;Body;Body	9808	***TAP2***	***TAP2***
cg11337525	5.06E-05	0.02827691	0.044855668	0.040682054	0.004173614	FDR0.05	19	45418417	Body;Body;Body	606	***APOC1***	***APOC1***
cg26662732	5.09E-05	0.028374237	0.715180573	0.750422266	-0.035241694	FDR0.05	1	155236380	TSS5000;Body;Body;Body;TSS5000;Body	-4204	***SCAMP3***	***SCAMP3;CLK2***
cg10546977	5.09E-05	0.028374237	0.79351537	0.776639434	0.016875937	FDR0.05	19	13915689	Body	9416	***ZSWIM4***	***ZSWIM4***
cg02268510	5.10E-05	0.028396386	0.765839773	0.780766933	-0.01492716	FDR0.05	19	38681615	Body	27880	***SNORD152***	***SIPA1L3***
cg25629773	5.15E-05	0.028623696	0.169873808	0.132396781	0.037477026	FDR0.05	6	117199067	Body	692	***RFX6***	***RFX6***
cg16441388	5.16E-05	0.028632309	0.717550153	0.739563938	-0.022013785	FDR0.05	7	92849458	Body;TSS1500;Body;TSS1500;Body	-550	***HEPACAM2***	***HEPACAM2***
cg15169162	5.17E-05	0.028632309	0.811368759	0.827643545	-0.016274785	FDR0.05	6	42690308	5'UTR	50	***PRPH2***	***PRPH2***
cg11035202	5.23E-05	0.028933297	0.792496502	0.785554486	0.006942016	FDR0.05	17	56234001	TES5000;TSS1500	-318	***MSX2P1***	***OR4D1;MSX2P1***
cg19737664	5.24E-05	0.028978818	0.03520034	0.02995632	0.00524402	FDR0.05	11	537435	TSS5000;TSS5000;TSS5000;TSS5000;TSS200	-86	***LRRC56***	***HRAS;LRRC56***
cg09070492	5.27E-05	0.029101927	0.03965378	0.03593164	0.003722139	FDR0.05	10	101492601	TSS200;TSS200;TSS200;TSS200;Body;TSS200	-178	***COX15***	***COX15;CUTC***
cg25201381	5.28E-05	0.029101927	0.83657271	0.873796819	-0.037224109	FDR0.05	3	124732807	Body	41995	***HEG1***	***HEG1***
cg08800893	5.28E-05	0.029101927	0.100823641	0.092751644	0.008071998	FDR0.05	22	36727569	Body	-44616	***MIR6819***	***MYH9***
cg18648917	5.29E-05	0.029101927	0.661953558	0.640712917	0.021240641	FDR0.05	3	23713773		-30690	***RNU6-788P***	
cg16878554	5.30E-05	0.029101927	0.842744824	0.829766502	0.012978323	FDR0.05	6	4255199		-64936	***LOC102724096***	
cg04256834	5.31E-05	0.029118683	0.803308702	0.844801763	-0.04149306	FDR0.05	11	132502809	Body;Body;Body;Body;Body;Body	310723	***OPCML***	***OPCML***
cg06103394	5.35E-05	0.029274885	0.05338095	0.055164416	-0.001783466	FDR0.05	16	3018991	TES5000;TES5000;TES5000;TES5000;TES5000;TES5000;TSS1500;TSS1500;TSS1500;TSS1500;TES5000;TSS1500;TES5000	-254	***PAQR4***	***PKMYT1;KREMEN2;PAQR4***
cg04281738	5.35E-05	0.029274885	0.872734671	0.889558646	-0.016823976	FDR0.05	4	96445830	Body	24531	***UNC5C***	***UNC5C***
cg16668176	5.35E-05	0.029274885	0.800188124	0.798953657	0.001234466	FDR0.05	19	5308893	Body;Body;Body;Body	31921	***PTPRS***	***PTPRS***
cg22232504	5.37E-05	0.029318807	0.849214092	0.862887462	-0.013673371	FDR0.05	12	26349469	Body;Body	964	***SSPN***	***SSPN***
cg14218275	5.41E-05	0.029466844	0.847711581	0.846083277	0.001628304	FDR0.05	10	11047435	Body;Body;Body;Body;Body;TSS200;TSS200;Body;Body;Body;Body;Body;Body;Body;Body;Body;Body;Body;Body;Body;Body;Body	-126	***CELF2***	***CELF2***
cg07450633	5.41E-05	0.029466844	0.566670625	0.570347164	-0.003676539	FDR0.05	5	172175321		-7184	***LOC101928093***	
cg07808509	5.42E-05	0.029466844	0.036977421	0.033463983	0.003513439	FDR0.05	6	52926847	TSS1500;TSS1500;TSS5000;TSS5000	-247	***ICK***	***ICK;FBXO9***
cg06291983	5.42E-05	0.029466844	0.843182638	0.8494101	-0.006227462	FDR0.05	2	102438026	Body;Body;Body;Body;Body	123489	***MAP4K4***	***MAP4K4***
cg18901104	5.44E-05	0.029466844	0.188497141	0.170929702	0.01756744	FDR0.05	19	4916783	Body;Body;Body;Body;Body	6405	***UHRF1***	***UHRF1***
cg19002907	5.44E-05	0.029466844	0.865589777	0.868732234	-0.003142457	FDR0.05	10	115939579	Body;Body	468	***TDRD1***	***TDRD1***
cg23378365	5.44E-05	0.029466844	0.150153798	0.136405883	0.013747915	FDR0.05	5	156696351	Body;Body;5'UTR;Body	0	***CYFIP2***	***CYFIP2***
cg09547025	5.45E-05	0.029466844	0.693055966	0.765529895	-0.072473929	FDR0.05	6	142721804	Body;Body;Body;Body	98749	***ADGRG6***	***ADGRG6***
cg10070260	5.47E-05	0.029522289	0.079354735	0.067647768	0.011706967	FDR0.05	15	74422632	TSS200;Body;TSS1500;Body;Body;TSS1500	43	***ISLR2***	***ISLR2;LOC283731***
cg04108787	5.47E-05	0.029522289	0.818371486	0.842397937	-0.024026451	FDR0.05	3	23650443	TES5000	-196940	***UBE2E1***	***MIR548AC***
cg18253910	5.50E-05	0.029613313	0.76177435	0.806532599	-0.044758249	FDR0.05	22	30230951	Body;Body	3342	***ASCC2***	***ASCC2***
cg23584308	5.51E-05	0.029623388	0.688291656	0.68312015	0.005171505	FDR0.05	14	105352991	Body;Body	21342	***CEP170B***	***CEP170B***
cg20647962	5.52E-05	0.029623388	0.039375074	0.034716315	0.004658759	FDR0.05	15	41136443	TSS200;Body;Body;Body;Body;Body	198	***SPINT1***	***SPINT1;SPINT1-AS1***
cg04804726	5.54E-05	0.029623388	0.787492007	0.789598028	-0.002106021	FDR0.05	10	31065098		52919	***RP11-14C22.6***	
cg25658276	5.54E-05	0.029623388	0.615630764	0.608034827	0.007595937	FDR0.05	5	180038655	Body;Body;Body	-20168	***SCGB3A1***	***FLT4***
cg05070626	5.54E-05	0.029623388	0.031819731	0.025274643	0.006545089	FDR0.05	16	19191386	Body;Body;Body	7708	***SYT17***	***SYT17***
cg04187668	5.54E-05	0.029623388	0.784464889	0.784555722	-9.08E-05	FDR0.05	4	178406078	Body	-27696	***RNA5SP172***	***RP11-130F10.1***
cg16735190	5.55E-05	0.029623388	0.79167221	0.789669824	0.002002386	FDR0.05	4	2303732	Body;Body;Body	-39993	***MXD4***	***ZFYVE28***
cg10905613	5.55E-05	0.029623388	0.037680616	0.03449253	0.003188087	FDR0.05	17	7218247	TES5000;TES5000;Body;TES5000;TES5000;TES5000;TES5000	411	***GPS2***	***EIF5A;NEURL4;GPS2***
cg15226808	5.56E-05	0.029623388	0.705591299	0.737129711	-0.031538413	FDR0.05	6	125286951	Body;Body;TSS5000	-2778	***RNF217-AS1***	***RNF217;RNF217-AS1***
cg13497187	5.57E-05	0.029623388	0.702899447	0.722396734	-0.019497287	FDR0.05	1	155271149	TSS1500;1stExon	80	***PKLR***	***PKLR***
cg25922751	5.58E-05	0.029623388	0.199807832	0.195251553	0.004556279	FDR0.05	12	124950720	Body;Body;Body	101290	***NCOR2***	***NCOR2***
cg07256442	5.59E-05	0.029623388	0.041938149	0.034969723	0.006968425	FDR0.05	6	33169127	TSS1500;1stExon;1stExon;TSS5000;TSS1500;TSS1500;1stExon	-497	***RXRB***	***RXRB;SLC39A7;HSD17B8***
cg19860160	5.59E-05	0.029623388	0.806556707	0.821164347	-0.014607641	FDR0.05	1	1045382	Body;Body;Body	6354	***C1orf159***	***C1orf159***
cg26548834	5.60E-05	0.029623388	0.68393429	0.740125317	-0.056191028	FDR0.05	7	4752951	Body	31022	***FOXK1***	***FOXK1***
cg04221521	5.60E-05	0.029623388	0.066920133	0.054182917	0.012737216	FDR0.05	5	72597715		9953	***RP11-60A8.1***	

#cpg	p_adj	q_val	Meth.BPD	Meth.nonBPD	dMeth	significance	Chr	pos.hg19	locations	dist2TSS	gene.nearest	gene.Illumina
cg09607043	5.60E-05	0.029623388	0.412647922	0.402685292	0.00996263	FDR0.05	17	17295349		46767	***NT5M***	
cg15617545	5.63E-05	0.029685147	0.882364593	0.889407471	-0.007042878	FDR0.05	11	57365018	TSS1500;TSS200	-8	***SERPING1***	***SERPING1***
cg01417432	5.63E-05	0.029685147	0.866722755	0.877105624	-0.010382869	FDR0.05	10	63913731		37758	***AL671972.1***	
cg22086973	5.64E-05	0.029685147	0.058390047	0.055148617	0.00324143	FDR0.05	5	77590574	5'UTR;5'UTR	5	***AP3B1***	***AP3B1***
cg20483016	5.65E-05	0.029694547	0.037194771	0.033855971	0.0033388	FDR0.05	14	20774219	TSS200	-66	***TTC5***	***TTC5***
cg01812571	5.67E-05	0.029694547	0.795251475	0.825944571	-0.030693096	FDR0.05	12	132970724		31070	***RP13-714J12.1***	
cg07179000	5.67E-05	0.029694547	0.799857067	0.712575452	0.087281615	FDR0.05	15	33023586	3'UTR;3'UTR;3'UTR	-12520	***LOC100131315***	***GREM1***
cg00793181	5.67E-05	0.029694547	0.642842158	0.653126823	-0.010284664	FDR0.05	12	52995634	TSS1500;TSS1500;TSS1500	-312	***KRT72***	***KRT72***
cg27144670	5.68E-05	0.029694547	0.684216322	0.786363079	-0.102146757	FDR0.05	15	52161438	Body	39614	***TMOD3***	***TMOD3***
cg24334334	5.68E-05	0.029694547	0.925574404	0.913989782	0.011584622	FDR0.05	6	70494380	Body;Body	11929	***LMBRD1***	***LMBRD1***
cg04466898	5.69E-05	0.029694547	0.2131402	0.211286279	0.001853921	FDR0.05	11	65407734	TES5000;TES5000;Body;5'UTR	143	***SIPA1***	***PCNX3;MIR4690;SIPA1***
cg27230496	5.69E-05	0.029694547	0.020211889	0.022292497	-0.002080608	FDR0.05	1	179262989	5'UTR;5'UTR;Body;5'UTR	141	***SOAT1***	***SOAT1***
cg02517342	5.70E-05	0.029694547	0.826268484	0.83997354	-0.013705056	FDR0.05	8	68470264	Body	188356	***CPA6***	***CPA6***
cg17292100	5.70E-05	0.029694547	0.702658541	0.779701624	-0.077043082	FDR0.05	8	101250595	Body;Body	63826	***RNF19A***	***SPAG1***
cg20370364	5.72E-05	0.029744174	0.902623399	0.900447679	0.00217572	FDR0.05	6	170703943	Body;Body;Body;Body	64095	***MIR4644***	***FAM120B***
cg00164282	5.73E-05	0.029744174	0.268140911	0.215961421	0.052179489	FDR0.05	17	30830732	Body	16628	***CDK5R1***	***MYO1D***
cg21635185	5.73E-05	0.029744174	0.926722244	0.93524691	-0.008524666	FDR0.05	2	27360428	Body;TSS5000;TSS5000;TSS5000;TSS5000;TSS5000;TSS5000	1904	***PRR30***	***PRR30;PREB***
cg04898130	5.74E-05	0.029744174	0.067024929	0.061830252	0.005194676	FDR0.05	11	44810532	Body	24557	***TSPAN18***	***TSPAN18***
cg06858181	5.75E-05	0.029744174	0.89978956	0.903273014	-0.003483454	FDR0.05	6	158818768	Body;Body	74755	***SNORA116***	***TULP4***
cg15739913	5.76E-05	0.029781007	0.900219255	0.896517707	0.003701548	FDR0.05	3	4722338	Body;Body;Body	70936	***EGOT***	***ITPR1***
cg16749930	5.77E-05	0.029784977	0.14962467	0.1454734	0.00415127	FDR0.05	11	71709978	TES5000;TES5000;TES5000;TSS200;TES5000;TSS1500;TSS1500;TES5000;5'UTR;<br>TSS200;5'UTR;5'UTR;TES5000;TES5000	21	***IL18BP***	***NUMA1;RNF121;IL18BP***
cg23784132	5.79E-05	0.02984245	0.75999093	0.756830713	0.003160217	FDR0.05	6	30132471	Body;TSS5000;TSS5000	1489	***TRIM15***	***TRIM15;TRIM10***
cg24999588	5.80E-05	0.029870349	0.132901707	0.126385639	0.006516068	FDR0.05	8	61839426	Body;Body;Body	7887	***AC022182.1***	***AC022182.1***
cg18969004	5.80E-05	0.029870349	0.702007263	0.801563089	-0.099555827	FDR0.05	6	31164743	TSS1500	-793	***HCG27***	***HCG27***
cg09753632	5.81E-05	0.029879955	0.743736914	0.806634206	-0.062897293	FDR0.05	5	95413861	Body;TSS1500	-980	***MIR583***	***LOC101929710;MIR583***
cg01695620	5.82E-05	0.029882963	0.919433653	0.932683427	-0.013249774	FDR0.05	8	58122850	Body;TES5000;Body	-7984	***RP11-513O17.2***	***RP11-513O17.2***
cg07365452	5.84E-05	0.029952707	0.944917311	0.949391498	-0.004474187	FDR0.05	3	42265749	3'UTR;Body;3'UTR;3'UTR;3'UTR;3'UTR;3'UTR	39690	***CCK***	***TRAK1***
cg08763433	5.86E-05	0.030001239	0.040109798	0.036325194	0.003784605	FDR0.05	1	156252438	1stExon;1stExon;TSS5000;1stExon;Body;5'UTR;TSS1500	182	***SMG5***	***SMG5;TMEM79***
cg05624376	5.89E-05	0.030158369	0.590245893	0.667421273	-0.077175381	FDR0.05	2	169939876	Body;Body;Body;Body	2260	***DHRS9***	***DHRS9***
cg23966363	5.92E-05	0.030272778	0.29096227	0.27081073	0.020151541	FDR0.05	19	937630	Body	11594	***ARID3A***	***ARID3A***
cg01358842	5.93E-05	0.030288096	0.0296448	0.026191324	0.003453477	FDR0.05	8	42995659	5'UTR;1stExon;1stExon;1stExon	53	***HGSNAT***	***HGSNAT***
cg07472511	5.94E-05	0.03031101	0.084956949	0.083690394	0.001266556	FDR0.05	20	49127687	Body;Body	830	***PTPN1***	***PTPN1***
cg13409589	5.95E-05	0.03031101	0.035363545	0.031423476	0.003940068	FDR0.05	11	3013777	TSS200	-170	***NAP1L4***	***NAP1L4***
cg00733786	5.97E-05	0.030355418	0.044467609	0.040553194	0.003914415	FDR0.05	14	54795898		-67668	***CDKN3***	
cg06803201	5.98E-05	0.030406334	0.067657547	0.064841899	0.002815648	FDR0.05	22	51021816	TSS5000;TSS5000;TSS5000;TSS5000;Body;TSS5000;TSS1500;TSS1500;Body	362	***CHKB-DT***	***CPT1B;CHKB-DT;CHKB;CHKB-CPT1B***
cg23749856	6.01E-05	0.030496679	0.076993349	0.070144552	0.006848797	FDR0.05	3	142839837	1stExon	1220	***CHST2***	***CHST2***
cg02610486	6.01E-05	0.030496679	0.122884773	0.108060001	0.014824772	FDR0.05	5	3593809	TSS5000	-2358	***IRX1***	***IRX1***
cg14358709	6.03E-05	0.030558067	0.032261233	0.029428832	0.002832402	FDR0.05	8	101321928	Body;Body;TSS200	-179	***RNF19A***	***RNF19A***
cg12891195	6.08E-05	0.030761766	0.079485186	0.069439659	0.010045527	FDR0.05	13	20734717	Body	466	***GJA3***	***GJA3***
cg26305504	6.09E-05	0.030761766	0.722295407	0.756245061	-0.033949654	FDR0.05	19	947612	Body	21576	***ARID3A***	***ARID3A***
cg25518868	6.10E-05	0.030797266	0.381219839	0.375448492	0.005771347	FDR0.05	5	140984057	Body;Body;Body	14565	***DIAPH1***	***DIAPH1***
cg27655512	6.13E-05	0.030903231	0.133859249	0.152015574	-0.018156325	FDR0.05	11	2287421	TES5000	4761	***ASCL2***	***ASCL2***
cg04270982	6.14E-05	0.030903231	0.943018253	0.954148841	-0.011130588	FDR0.05	4	188078789		-44350	***LINC02374***	
cg25289028	6.15E-05	0.030903231	0.775474436	0.750522804	0.024951632	FDR0.05	19	36428519	Body	498	***LRFN3***	***LRFN3***
cg12793123	6.15E-05	0.030903231	0.227600262	0.22991612	-0.002315857	FDR0.05	22	24823554	Body;Body;Body;5'UTR;TSS5000;TSS5000;Body;5'UTR;TES5000	25	***ADORA2A***	***ADORA2A;SPECC1L-ADORA2A;ADORA2A-AS1***
cg01871995	6.16E-05	0.030940804	0.769496385	0.811284536	-0.041788151	FDR0.05	8	17754363	TSS1500;TSS1500;TSS1500;TSS1500	-1316	***FGL1***	***FGL1***
cg14611258	6.17E-05	0.030951475	0.760646877	0.7728158	-0.012168924	FDR0.05	17	17946468	TSS5000;Body	3858	***GID4***	***ATPAF2;GID4***
cg19578751	6.20E-05	0.031034588	0.701098098	0.732595545	-0.031497448	FDR0.05	6	32134011	TES5000;TES5000;Body;TES5000;TES5000;Body;TES5000;1stExon;TES5000	1630	***EGFL8***	***AGPAT1;PPT2-EGFL8;PPT2;EGFL8;MIR6721***
cg22027151	6.21E-05	0.031039285	0.741147204	0.804812445	-0.063665241	FDR0.05	12	41860791	Body;Body	29251	***PDZRN4***	***PDZRN4***
cg11843333	6.21E-05	0.031039285	0.85044107	0.883296705	-0.032855635	FDR0.05	4	15719138	Body	14566	***BST1***	***BST1***
cg03157738	6.22E-05	0.031039285	0.028262532	0.023990593	0.004271939	FDR0.05	19	11308118	TES5000;TSS5000;TSS5000;Body	125	***KANK2***	***DOCK6;KANK2***
cg01360093	6.24E-05	0.031091786	0.049545594	0.039828438	0.009717157	FDR0.05	1	21995967	TSS200;TSS200;TSS200;TSS200;TSS200;TSS200;TSS200;TSS200	-94	***RAP1GAP***	***RAP1GAP***
cg03844838	6.25E-05	0.031091786	0.933033128	0.942307283	-0.009274155	FDR0.05	10	661087	Body	-13416	***LOC101930421***	***DIP2C***
cg21008399	6.25E-05	0.031091786	0.624571397	0.653459459	-0.028888062	FDR0.05	12	56120816	Body;TES5000;Body;TES5000;TES5000;Body;Body;Body;Body;Body;Body	756	***CD63***	***CD63;BLOC1S1-RDH5;RDH5***
cg18337287	6.27E-05	0.031091786	0.847658014	0.827792005	0.019866009	FDR0.05	19	930871	Body	4835	***ARID3A***	***ARID3A***
cg01491360	6.28E-05	0.031091786	0.848608619	0.841291825	0.007316794	FDR0.05	19	2421956	Body;TES5000	5958	***TIMM13***	***TMPRSS9;TIMM13***

#cpg	p_adj	q_val	Meth.BPD	Meth.nonBPD	dMeth	significance	Chr	pos.hg19	locations	dist2TSS	gene.nearest	gene.Illumina
cg19955019	6.28E-05	0.031091786	0.851706893	0.868380868	-0.016673975	FDR0.05	3	143773022		80573	***C3orf58***	
cg18279670	6.28E-05	0.031091786	0.045573129	0.039121506	0.006451623	FDR0.05	22	37172204	TSS200;TSS200;TSS200;TSS200	-27	***IFT27***	***IFT27***
cg10624729	6.30E-05	0.03117044	0.911933088	0.907415962	0.004517126	FDR0.05	1	78298207	Body;Body;Body;Body;Body	52718	***MIGA1***	***MIGA1***
cg26972614	6.31E-05	0.03117044	0.654012168	0.664750591	-0.010738423	FDR0.05	11	71710885	TES5000;TES5000;TES5000;5'UTR;TES5000;TSS200;Body;TES5000;Body;5'UTR;Body;Body;TES5000;TES5000	-87	***IL18BP***	***NUMA1;RNF121;IL18BP***
cg14645972	6.34E-05	0.031311128	0.030591896	0.030171647	0.000420249	FDR0.05	6	2988997	Body	797	***LINC01011***	***LINC01011***
cg24820508	6.40E-05	0.031572958	0.043798091	0.039151752	0.004646339	FDR0.05	3	138048370	5'UTR;5'UTR;5'UTR;5'UTR;5'UTR;Body;5'UTR;Body	358	***NME9***	***NME9***
cg05123067	6.41E-05	0.031578801	0.924913199	0.927849299	-0.0029361	FDR0.05	1	203455873	Body;Body	-7397	***OPTC***	***PRELP***
cg06648780	6.42E-05	0.031578801	0.672785416	0.747462164	-0.074676748	FDR0.05	3	105133817	Body;Body;Body;Body	48261	***ALCAM***	***ALCAM***
cg23621496	6.43E-05	0.031585049	0.800326367	0.795440947	0.004885421	FDR0.05	1	211667487	TSS1500;TSS1500	-1228	***RD3***	***RD3***
cg12679261	6.45E-05	0.031594256	0.804011321	0.83721746	-0.033206139	FDR0.05	5	895511	TSS5000;TSS5000;TSS5000;Body;Body;TSS5000	2543	***TRIP13***	***BRD9;TRIP13***
cg15597480	6.45E-05	0.031594256	0.79658363	0.846318669	-0.049735039	FDR0.05	1	110036205	TES5000;TES5000;TES5000;TSS1500;TES5000;TSS1500;TSS1500;TSS1500;TSS1500	-452	***CYB561D1***	***ATXN7L2;CYB561D1***
cg09212489	6.46E-05	0.031594256	0.831323645	0.84433397	-0.013010326	FDR0.05	4	108539552	Body	101867	***PAPSS1***	***PAPSS1***
cg12893736	6.46E-05	0.031594256	0.698140962	0.724561969	-0.026421007	FDR0.05	17	72429636	Body;Body;Body;TSS200	-199	***GPRC5C***	***GPRC5C***
cg24617992	6.46E-05	0.031594256	0.905274165	0.904950011	0.000324154	FDR0.05	6	33866289	TSS5000;TSS1500	-622	***MIR7159***	***LINC01016;MIR7159***
cg01109219	6.52E-05	0.031820492	0.843254628	0.850846902	-0.007592274	FDR0.05	2	33660754	TSS1500;TSS1500;TSS1500	-661	***RASGRP3***	***RASGRP3***
cg12591289	6.53E-05	0.031864717	0.899355897	0.910865398	-0.011509501	FDR0.05	3	99833051	5'UTR;5'UTR;Body;TSS1500	306	***FILIP1L***	***FILIP1L;CMSS1***
cg06933072	6.56E-05	0.031899331	0.808748773	0.831853835	-0.023105063	FDR0.05	1	167882951	TES5000;Body;TES5000;Body;Body;TES5000	513	***ADCY10***	***MPC2;ADCY10***
cg13236481	6.57E-05	0.031899331	0.817853673	0.820638468	-0.002784795	FDR0.05	1	172395460	Body	5633	***C1orf105***	***C1orf105***
cg25587091	6.57E-05	0.031899331	0.147713328	0.133779215	0.013934113	FDR0.05	15	66125463	Body;Body;Body	-10358	***RNU6-265P***	***RAB11A***
cg20645065	6.58E-05	0.031899331	0.12481307	0.118941543	0.005871527	FDR0.05	1	21836138	Body;Body;Body	288	***ALPL***	***ALPL***
cg26513988	6.59E-05	0.031899331	0.830278954	0.843864781	-0.013585827	FDR0.05	10	17244746	TSS1500;TSS1500;TSS1500;TSS1500;TSS1500;TSS1500;TSS1500;TSS1500	-676	***TRDMT1***	***TRDMT1***
cg02618319	6.59E-05	0.031899331	0.946884908	0.952392799	-0.005507891	FDR0.05	11	119247012	Body;Body	5424	***USP2***	***USP2***
cg09421083	6.59E-05	0.031899331	0.385090391	0.342896101	0.042194291	FDR0.05	10	134358535	Body	7253	***INPP5A***	***INPP5A***
cg23387863	6.60E-05	0.031899331	0.630445585	0.636422067	-0.005976482	FDR0.05	15	77472416	1stExon	45330	***LINC00597***	***PEAK1***
cg01604601	6.61E-05	0.031899331	0.097234555	0.083943635	0.01329092	FDR0.05	2	69846936	Body	-24000	***ANXA4***	***AAK1***
cg24849872	6.62E-05	0.031899331	0.057359597	0.077998603	-0.020639006	FDR0.05	17	16472123	5'UTR;Body;Body;Body;Body;5'UTR	403	***ZNF287***	***ZNF287***
cg21182715	6.62E-05	0.031899331	0.700235508	0.748826227	-0.048590718	FDR0.05	12	95567919	Body	43321	***FGD6***	***FGD6***
cg23702644	6.64E-05	0.031933767	0.818731451	0.803847123	0.014884328	FDR0.05	8	3254875	Body	-668884	***LOC101927815***	***CSMD1***
cg26302178	6.65E-05	0.031933767	0.879326068	0.876518866	0.002807202	FDR0.05	6	29794843	Body;Body	88	***HLA-G***	***HLA-G***
cg19828460	6.67E-05	0.031933767	0.839112462	0.842662656	-0.003550194	FDR0.05	10	118551426	Body	-49341	***HSPA12A***	***HSPA12A***
cg04129911	6.67E-05	0.031933767	0.67728887	0.673855219	0.003433651	FDR0.05	4	79543134		10405	***Y_RNA***	
cg09423651	6.67E-05	0.031933767	0.711583323	0.781968663	-0.07038534	FDR0.05	3	136618442	Body;Body	-30874	***NCK1***	***NCK1***
cg00903950	6.68E-05	0.031933767	0.045753475	0.044239776	0.001513699	FDR0.05	1	155715320	TES5000;TES5000;TES5000;TSS1500	-238	***MSTO2P***	***GON4L;MSTO2P***
cg10020890	6.68E-05	0.031933767	0.724767954	0.769824433	-0.045056479	FDR0.05	16	75025753	Body;Body	5318	***WDR59***	***WDR59***
cg27363558	6.68E-05	0.031933767	0.147902345	0.148760032	-0.000857687	FDR0.05	17	7108792	Body;Body;TSS1500;TSS1500;Body;Body;Body	-305	***DLG4***	***DLG4***
cg01708648	6.70E-05	0.031979799	0.74659671	0.756664141	-0.010067431	FDR0.05	11	64023044	Body;Body;Body	4050	***PLCB3***	***PLCB3***
cg26921187	6.71E-05	0.031983174	0.033952967	0.030327147	0.00362582	FDR0.05	14	89883537	Body;TSS200;TSS200	-83	***FOXN3***	***FOXN3;FOXN3-AS1***
cg14214321	6.75E-05	0.03213532	0.061025868	0.045989869	0.015035999	FDR0.05	12	120935059	TSS1500;Body;Body;Body	1201	***DYNLL1***	***NRAV;DYNLL1***
cg19702771	6.76E-05	0.032172918	0.833431859	0.83839504	-0.004963181	FDR0.05	4	82058885	Body;Body;Body;Body;Body;Body;Body	16426	***PRKG2***	***PRKG2***
cg07671622	6.78E-05	0.032207744	0.617804331	0.619444801	-0.00164047	FDR0.05	16	70759994	Body;Body	-29003	***VAC14-AS1***	***VAC14***
cg04276007	6.79E-05	0.032242592	0.699679311	0.737554516	-0.037875205	FDR0.05	7	107386512	Body;TSS1500;TSS5000;TSS5000;TSS5000;Body;Body	-1486	***LOC101927974***	***CBLL1;LOC101927974***
cg12157788	6.83E-05	0.032329716	0.782202919	0.76649528	0.015707639	FDR0.05	7	4389134		116616	***SDK1***	
cg20678070	6.83E-05	0.032329716	0.883869974	0.90005664	-0.016186666	FDR0.05	6	113468219		175525	***RNU6-1163P***	
cg27468857	6.83E-05	0.032329716	0.95493075	0.961284866	-0.006354116	FDR0.05	8	1783358	Body;Body;Body	11217	***ARHGEF10***	***ARHGEF10***
cg26215982	6.85E-05	0.032345683	0.831723125	0.857447737	-0.025724612	FDR0.05	12	4915878	TSS5000	-2463	***KCNA6***	***KCNA6***
cg17242981	6.85E-05	0.032345683	0.691102584	0.698981415	-0.007878831	FDR0.05	1	244435933		-80003	***C1orf100***	
cg09969806	6.86E-05	0.032345683	0.11620577	0.10114115	0.015064621	FDR0.05	2	241758399	Body;Body;Body;Body;Body	1326	***KIF1A***	***KIF1A***
cg24530610	6.87E-05	0.032355702	0.941552675	0.92605257	0.015500105	FDR0.05	1	2440596	TES5000;TES5000;TES5000;Body	17439	***PANK4***	***PLCH2;PANK4***
cg06543365	6.88E-05	0.032367797	0.899028027	0.892935305	0.006092722	FDR0.05	6	167190149	Body;Body;Body	85890	***RPS6KA2***	***RPS6KA2***
cg18220380	6.88E-05	0.032367797	0.950107657	0.958339745	-0.008232089	FDR0.05	8	142185447	Body;Body;Body;Body	46737	***DENND3***	***DENND3***
cg19951107	6.90E-05	0.032376618	0.824090793	0.841499895	-0.017409101	FDR0.05	3	111760098	Body;Body;TSS5000	1634	***TMPRSS7***	***TMPRSS7***
cg20135306	6.90E-05	0.032376618	0.040333654	0.034956383	0.005377271	FDR0.05	19	5623027	TSS200;TSS200;TSS200;TSS200;TSS200;TSS200;TSS200	-18	***SAFB***	***SAFB;SAFB2***
cg13479358	6.91E-05	0.032376618	0.177471477	0.163497705	0.013973772	FDR0.05	6	28603052		12990	***AL121932.1***	
cg10573403	6.91E-05	0.032376618	0.059143051	0.052997183	0.006145867	FDR0.05	14	36003118	TES5000;TES5000;TES5000;TES5000;TES5000;TES5000;TES5000;TSS200;TES5000;TES5000;TES5000;TES5000;TES5000	-129	***INSM2***	***RALGAPA1;INSM2;RALGAPA1P1***
cg26112170	6.93E-05	0.03240171	0.598003478	0.590049895	0.007953583	FDR0.05	13	114103713	TSS1500;Body	-256	***ADPRHL1***	***ADPRHL1***
cg15143024	6.93E-05	0.03240171	0.898711967	0.896160714	0.002551253	FDR0.05	7	91199642		-13479	***RP11-142A5.1***	
cg11665583	6.95E-05	0.032455804	0.681561929	0.680948258	0.000613671	FDR0.05	22	26893201	Body;Body;Body;Body;Body;Body;Body	13352	***SRRD***	***TFIP11***
cg11429283	6.97E-05	0.03251039	0.851792817	0.876218594	-0.024425777	FDR0.05	11	85469037	5'UTR;5'UTR;Body;5'UTR;5'UTR;5'UTR;Body;5'UTR	75	***SYTL2***	***SYTL2***

#cpg	p_adj	q_val	Meth.BPD	Meth.nonBPD	dMeth	significance	Chr	pos.hg19	locations	dist2TSS	gene.nearest	gene.Illumina
cg05777583	6.98E-05	0.03251039	0.754780831	0.789220304	-0.034439473	FDR0.05	1	157716415	Body;3'UTR;3'UTR	30507	FCRL2	FCRL2
cg11034318	6.99E-05	0.03251039	0.802063725	0.825714723	-0.023650998	FDR0.05	7	150097654	TES5000	-5185	LOC728743	ZNF775
cg02674352	6.99E-05	0.03251039	0.05668795	0.041373649	0.015314301	FDR0.05	11	61275826	TSS1500;TSS1500	-241	MIR4488	MIR4488;LRRC10B
cg03154513	7.00E-05	0.03252199	0.03642768	0.030388105	0.006039576	FDR0.05	20	30193400	1stExon;TSS5000;1stExon	315	ID1	ID1;MIR3193
cg04147272	7.02E-05	0.032574624	0.878870755	0.889830596	-0.010959841	FDR0.05	15	79261496	Body;Body;Body	-9734	LOC100129540	RASGRF1
cg06135765	7.03E-05	0.032574624	0.725157957	0.782188947	-0.057030991	FDR0.05	3	37283665	TSS1500;TSS1500;Body	-1016	GOLGA4	GOLGA4;LOC152048
cg01700504	7.05E-05	0.032595662	0.463907799	0.439831305	0.024076494	FDR0.05	13	20805391	TSS200;Body;Body;Body	-19	GJB6	GJB6
cg14209487	7.05E-05	0.032595662	0.799895363	0.807312791	-0.007417428	FDR0.05	6	152635105	Body;Body	-66559	SYNE1-AS1	SYNE1
cg07780199	7.06E-05	0.032599693	0.827204786	0.829638183	-0.002433397	FDR0.05	19	18830892	Body;Body	36468	CRTC1	CRTC1
cg07953310	7.08E-05	0.032665294	0.037098314	0.03365435	0.003443964	FDR0.05	19	37808882	Body;Body;5'UTR;Body;Body;Body;5'UTR;5'UTR;Body;Body;5'UTR;5'UTR;5'UTR;Body;Body;5'UTR;5'UTR;Body;Body	99	HKR1	HKR1
cg20604456	7.08E-05	0.032665294	0.044464645	0.038197927	0.006266717	FDR0.05	2	211341126	5'UTR;5'UTR;5'UTR;TSS1500	303	LANCL1	LANCL1;CPS1
cg09762120	7.10E-05	0.03268495	0.68701571	0.759889076	-0.072873367	FDR0.05	7	30108301	Body;Body;Body;Body;Body;Body;Body;Body	23601	PLEKHA8	PLEKHA8
cg05621259	7.11E-05	0.032691924	0.759895543	0.745092863	0.014802679	FDR0.05	8	39836007	Body	43534	IDO2	IDO2
cg03370330	7.12E-05	0.032691924	0.831672791	0.843749357	-0.012076566	FDR0.05	2	100984712		-45517	LONRF2	
cg19376664	7.12E-05	0.032691924	0.70466869	0.749360292	-0.044691603	FDR0.05	6	32153575	TSS1500;TSS1500;TSS1500;TSS1500;TSS1500;TSS1500;TSS1500;3'UTR;TSS1500;TES5000;TSS1500;TES5000;TSS1500	-1476	AGER	AGER;PBX2;GPSM3
cg25664813	7.13E-05	0.032691924	0.808977588	0.812866159	-0.003888571	FDR0.05	5	1123123		-10951	SLC12A7	
cg02499214	7.17E-05	0.032841438	0.16766566	0.154664552	0.013001107	FDR0.05	12	122230490	TES5000;Body;TES5000;TES5000;TES5000;TES5000;TES5000;TES5000	1104	RHOF	LINC01089;RHOF
cg02487987	7.19E-05	0.032903547	0.773599181	0.817350324	-0.043751143	FDR0.05	5	52153575	Body	69440	ITGA1	ITGA1
cg16698674	7.20E-05	0.032907716	0.817446409	0.833219045	-0.015772637	FDR0.05	1	230272358	Body;Body	69417	GALNT2	GALNT2
cg02066343	7.23E-05	0.033022768	0.647058156	0.628385322	0.018672834	FDR0.05	6	33590458	Body	1303	ITPR3	ITPR3
cg24886068	7.24E-05	0.03304341	0.837380949	0.854065703	-0.016684754	FDR0.05	6	16391052	Body;Body;Body	152242	GMPR	ATXN1
cg05303901	7.27E-05	0.033085994	0.809544947	0.844910106	-0.035365159	FDR0.05	7	28338625	TSS1500	-314	CREB5	CREB5
cg07460095	7.27E-05	0.033085994	0.864977147	0.88967135	-0.024694204	FDR0.05	3	132373398	TSS5000;Body;Body;Body;TSS5000;Body;5'UTR;Body	109	UBA5	UBA5;ACAD11;NPHP3-ACAD11
cg27636676	7.27E-05	0.033085994	0.35453153	0.294502388	0.060029142	FDR0.05	1	165322606	Body;Body	2872	LMX1A	LMX1A
cg21315992	7.29E-05	0.033128497	0.853341868	0.843140476	0.010201392	FDR0.05	15	101592074	Body	-132586	LOC101927751	LRRK1
cg20046777	7.30E-05	0.033128497	0.842022075	0.852098668	-0.010076593	FDR0.05	12	61961255		259759	FAM19A2	
cg06695286	7.31E-05	0.033128497	0.740046416	0.810756677	-0.070710262	FDR0.05	12	42880445	Body;TSS5000;TSS5000;TSS5000	-2648	PRICKLE1	PRICKLE1
cg13451416	7.34E-05	0.033128497	0.793554124	0.783693152	0.009860972	FDR0.05	2	236671149	Body;Body;Body	-244584	TNRC17	AGAP1
cg07005770	7.35E-05	0.033128497	0.815273992	0.79915469	0.016119302	FDR0.05	7	1530051	Body	13967	INTS1	INTS1
cg05041596	7.35E-05	0.033128497	0.465492309	0.406196971	0.059295338	FDR0.05	11	89867385	TSS1500;TSS1500	-432	NAALAD2	NAALAD2
cg15968932	7.35E-05	0.033128497	0.750316325	0.764075235	-0.013758911	FDR0.05	11	19860035	Body;Body;Body;Body	-78406	MIR4694	NAV2
cg02907425	7.35E-05	0.033128497	0.884418914	0.866413788	0.018005125	FDR0.05	19	4688820	TES5000;Body;Body	9527	DPP9-AS1	DPP9-AS1;DPP9
cg11203990	7.36E-05	0.033128497	0.850081984	0.851014256	-0.000932273	FDR0.05	1	1102417	TSS1500;TSS200;TSS5000	-66	MIR200B	MIR200A;MIR200B;MIR429
cg02378784	7.37E-05	0.033128497	0.849388248	0.868034011	-0.018645763	FDR0.05	2	208656272		-21985	FZD5	
cg17128237	7.37E-05	0.033128497	0.772358495	0.769994154	0.002364341	FDR0.05	11	64792167	TES5000;TSS5000;TES5000;Body;TSS5000	-2712	SNX15	ARL2;SNX15;ARL2-SNX15
cg04603184	7.38E-05	0.033128497	0.035358427	0.03074854	0.004609886	FDR0.05	6	13924929	Body;TSS1500;TSS200;TSS200	-168	RNF182	RNF182
cg05627765	7.38E-05	0.033128497	0.679797646	0.708951958	-0.029154312	FDR0.05	15	70315909		27935	TLE3	
cg25205438	7.39E-05	0.033130384	0.475207974	0.566800473	-0.091592499	FDR0.05	5	140517411	3'UTR	2624	PCDHB5	PCDHB5
cg25351920	7.43E-05	0.033280491	0.924740922	0.913231679	0.011509243	FDR0.05	1	3696960	TES5000;TES5000;3'UTR	7610	SMIM1	SMIM1;LRRC47
cg17174836	7.44E-05	0.033296059	0.915300373	0.913893498	0.001406875	FDR0.05	3	101540493	Body;Body;Body;Body;Body;Body;Body;Body;Body;Body;Body;Body	-6340	NFKBIZ	NXPE3
cg03731727	7.45E-05	0.033303213	0.65462721	0.678944805	-0.024317595	FDR0.05	3	128562141		17228	RP11-723O4.9	
cg11685843	7.46E-05	0.033313343	0.767607401	0.718815676	0.048791725	FDR0.05	4	176349171		-24910	RP11-287F9.2	
cg26139080	7.46E-05	0.033313343	0.354781663	0.338504916	0.016276747	FDR0.05	11	47293733	TES5000;TSS200;Body;Body;Body;TES5000;TES5000;TES5000;Body;TSS200;Body;Body;Body;Body;Body;TES5000;Body;TES5000	-72	LOC101928943	NR1H3;LOC101928943;MADD
cg23007325	7.50E-05	0.033420725	0.72471304	0.694001795	0.030711245	FDR0.05	8	10924633	TSS200;Body	-34	LOC101929269	LOC101929269;XKR6
cg21400802	7.51E-05	0.033420725	0.205821681	0.177487917	0.028333764	FDR0.05	1	153312501	Body	8816	PGLYRP4	PGLYRP4
cg04917511	7.51E-05	0.033420725	0.033845696	0.018771622	0.015074074	FDR0.05	2	26785460	TSS5000;5'UTR;TSS5000;5'UTR	34	C2orf70	OTOF;C2orf70
cg01297383	7.52E-05	0.033420725	0.873128221	0.886378384	-0.013250163	FDR0.05	20	40627194	Body	7415	RP5-1121H13.4	RP5-1121H13.4
cg22595391	7.55E-05	0.033486411	0.221028221	0.189780621	0.0312476	FDR0.05	17	80407031	Body;Body;Body;Body;Body;Body;Body;Body;TES5000;Body;Body;Body	571	CYBC1	CYBC1
cg15552493	7.56E-05	0.033486411	0.428880955	0.429659904	-0.000778949	FDR0.05	2	102387391	Body;Body;Body;Body;Body	72854	MAP4K4	MAP4K4
cg21863888	7.57E-05	0.033486411	0.174073221	0.212811454	-0.038738233	FDR0.05	6	31127173	TES5000;TSS1500;TES5000;5'UTR;TES5000;5'UTR;TES5000;TES5000;5'UTR;TSS5000;TSS5000	871	TCF19	POU5F1;CCHCR1;TCF19
cg17528240	7.57E-05	0.033486411	0.78874483	0.83104447	-0.04229964	FDR0.05	17	79042149	Body;Body;Body;Body	33203	BAIAP2	BAIAP2
cg22615948	7.57E-05	0.033486411	0.849573691	0.8654258	-0.015852109	FDR0.05	1	111851998	Body;Body;Body;Body;Body;Body;Body;Body	18525	CHIA	CHIA
cg01434679	7.59E-05	0.033486411	0.807863353	0.842158694	-0.034295341	FDR0.05	1	9775302	Body;Body;Body	23778	PIK3CD	PIK3CD
cg04461311	7.60E-05	0.033486411	0.804948196	0.823286797	-0.018338601	FDR0.05	17	47926783	TSS1500;TSS1500;TSS1500;TES5000;TSS1500;TSS1500	-1404	TAC4	TAC4;FLJ45513
cg24486037	7.60E-05	0.033486411	0.796751368	0.864112702	-0.067361334	FDR0.05	1	151345181	5'UTR;5'UTR;5'UTR	29	SELENBP1	SELENBP1
cg01066270	7.61E-05	0.033486411	0.032706059	0.028514192	0.004191866	FDR0.05	5	74161556	Body;Body;TSS200	-161	LOC441086	FAM169A;LOC441086

#cpg	p_adj	q_val	Meth.BPD	Meth.nonBPD	dMeth	significance	Chr	pos.hg19	locations	dist2TSS	gene.nearest	gene.Illumina
cg14289863	7.61E-05	0.033486411	0.833855134	0.859366568	-0.025511434	FDR0.05	9	72872391	TSS1500;Body	1399	**SMC5-AS1**	**SMC5;SMC5-AS1**
cg19267248	7.63E-05	0.033486411	0.883427491	0.895886425	-0.012458933	FDR0.05	12	98849532	Body	1391	**SLC9A7P1**	**SLC9A7P1**
cg14458002	7.63E-05	0.033486411	0.699255178	0.752116231	-0.052861052	FDR0.05	6	45489463	Body;Body;Body	99150	**RUNX2**	**RUNX2**
cg15196147	7.63E-05	0.033486411	0.856378608	0.866149352	-0.009770743	FDR0.05	7	111791255	Body;Body	55202	**DOCK4**	**DOCK4**
cg23392804	7.64E-05	0.033486411	0.859338009	0.873851616	-0.014513606	FDR0.05	2	196930573	Body	2963	**DNAH7**	**DNAH7**
cg18259253	7.67E-05	0.03360504	0.874312487	0.890860879	-0.016548391	FDR0.05	5	60745986	Body	117887	**ZSWIM6**	**ZSWIM6**
cg26808583	7.68E-05	0.033611148	0.782414506	0.811013749	-0.028599243	FDR0.05	1	153346525	Body	1550	**S100A12**	**S100A12**
cg12625824	7.70E-05	0.033623202	0.862988717	0.876016467	-0.013027751	FDR0.05	2	113672587	Body;Body;Body;TSS200;Body	-182	**IL37**	**IL37**
cg16087826	7.70E-05	0.033623202	0.755017147	0.775632216	-0.020615069	FDR0.05	5	131654574	Body;Body	24430	**SLC22A4**	**MIR3936HG;SLC22A4**
cg21119032	7.71E-05	0.033623202	0.020100903	0.018312164	0.001788739	FDR0.05	2	211341315	Body;Body;Body;TSS1500	114	**LANCL1**	**LANCL1;CPS1**
cg13909879	7.71E-05	0.033623202	0.025920905	0.022191229	0.003729676	FDR0.05	4	85420999	TSS5000	-1612	**NKX6-1**	**NKX6-1**
cg07552087	7.73E-05	0.033645122	0.783895018	0.791975304	-0.008080286	FDR0.05	16	2258457	Body;TES5000;Body;Body;Body;Body;Body;Body;Body;Body;Body;TES5000;Body	2612	**BRICD5**	**MLST8;PGP;BRICD5**
cg08072356	7.74E-05	0.033673395	0.749139198	0.795168363	-0.046029165	FDR0.05	14	20611779	TSS200	-115	**OR4N5**	**OR4N5**
cg00253681	7.75E-05	0.033673395	0.672595673	0.721617777	-0.049022104	FDR0.05	12	14996583	TSS200;TSS200	-116	**ART4**	**ART4**
cg08127369	7.76E-05	0.033696571	0.84246691	0.868493113	-0.026026203	FDR0.05	19	33164662	TSS5000;Body	1440	**ANKRD27**	**RGS9BP;ANKRD27**
cg07084004	7.77E-05	0.033696571	0.837240865	0.856187212	-0.018946347	FDR0.05	14	57506426	Body	28834	**RP11-108M12.3**	**RP11-1077A2.1**
cg05048043	7.78E-05	0.033696571	0.814293612	0.816162803	-0.001869191	FDR0.05	3	188198457	Body;Body;Body	87997	**LPP-AS1**	**LPP**
cg13633866	7.79E-05	0.03371115	0.792475728	0.807057114	-0.014581386	FDR0.05	6	149454921		43009	**AL603766.1**	
cg23250964	7.81E-05	0.0337527	0.869629439	0.873118236	-0.003488796	FDR0.05	6	116781066	TSS1500;TSS1500	-1466	**CALHM6**	**CALHM6**
cg08790319	7.81E-05	0.0337527	0.696791565	0.675159963	0.021631602	FDR0.05	16	2203008	TSS5000;TES5000;Body;Body;TES5000	2098	**SNORD60**	**TRAF7;SNHG19;RAB26;SNORD60**
cg11072779	7.84E-05	0.033832506	0.865456331	0.859604718	0.005851613	FDR0.05	2	70044977	Body;Body;Body;Body;Body	-11760	**GMCL1**	**ANXA4**
cg21627181	7.87E-05	0.0339288	0.730699992	0.749017512	-0.01831752	FDR0.05	6	25754190	TSS1500;TSS1500	-736	**SLC17A4**	**SLC17A4**
cg15387598	7.93E-05	0.034157537	0.039766498	0.033126328	0.00664017	FDR0.05	1	202311228	TSS200;TSS200	-134	**UBE2T**	**UBE2T**
cg27389684	7.94E-05	0.034157537	0.868300315	0.860352583	0.007947732	FDR0.05	20	55953214	3'UTR;3'UTR	-13239	**RBM38**	**RAE1**
cg10492657	7.94E-05	0.034165756	0.687816917	0.702491572	-0.014674655	FDR0.05	6	4854374	Body;Body;Body	18043	**CDYL**	**CDYL**
cg11646078	7.95E-05	0.034165756	0.799147739	0.808972702	-0.009824963	FDR0.05	10	34519312	Body;Body;Body;Body;Body;Body;Body;Body	-457704	**LINC00838**	**PARD3**
cg25610029	8.00E-05	0.034333218	0.855110369	0.871379011	-0.016268642	FDR0.05	2	68544328	Body;Body	2855	**CNRIP1**	**CNRIP1**
cg03884860	8.02E-05	0.034400267	0.805502918	0.816194875	-0.010691957	FDR0.05	18	76397484		-93296	**Y_RNA**	
cg06972903	8.03E-05	0.034412139	0.835534273	0.862234603	-0.02670033	FDR0.05	1	6580525	TES5000;TSS1500	-404	**PLEKHG5**	**NOL9;PLEKHG5**
cg19553056	8.09E-05	0.034639083	0.037095991	0.034109396	0.002986595	FDR0.05	4	142142166	5'UTR;5'UTR	126	**ZNF330**	**ZNF330**
cg23154059	8.12E-05	0.034712695	0.044636466	0.032070819	0.012565646	FDR0.05	2	219736250	Body	-9004	**WNT10A**	**WNT6**
cg21518261	8.13E-05	0.034727883	0.757384251	0.76208633	-0.004702079	FDR0.05	8	145721667	Body;Body;Body;Body;Body	-7797	**GPT**	**PPP1R16A**
cg24389585	8.15E-05	0.03477993	0.893499904	0.887528975	0.005970929	FDR0.05	7	27184125	TES5000;Body;TSS5000;TSS1500	-838	**HOXA5**	**HOXA6;HOXA-AS3;HOXA5**
cg13883590	8.16E-05	0.034789393	0.948832473	0.957388238	-0.008555765	FDR0.05	4	1952852	Body;TES5000;Body;Body;Body	-23510	**SCARNA22**	**NSD2**
cg03660710	8.18E-05	0.034858333	0.098542189	0.092512956	0.006029233	FDR0.05	22	50631079	Body;Body;Body;Body;Body;Body	2101	**TRABD**	**TRABD**
cg18540871	8.20E-05	0.034887549	0.056483216	0.054917396	0.001565819	FDR0.05	6	143832937	5'UTR	83	**FUCA2**	**FUCA2**
cg03044281	8.23E-05	0.034993427	0.747583787	0.759532234	-0.011948446	FDR0.05	17	1553585	TES5000;TSS5000;TSS5000;TSS5000;TSS5000;TSS200	-193	**RILP**	**PRPF8;SCARF1;RILP**
cg16417118	8.28E-05	0.035167214	0.577132133	0.507173423	0.069958709	FDR0.05	1	2162931	Body	2798	**SKI**	**SKI**
cg01408817	8.29E-05	0.035167214	0.786016053	0.781293215	0.004722838	FDR0.05	19	35606534	TSS200;TSS200;TSS200;TSS200;TSS200;TSS200;TSS200;TSS200	-197	**FXYD3**	**FXYD3**
cg03044444	8.31E-05	0.035230384	0.837294443	0.860621676	-0.023327232	FDR0.05	13	113355356	Body;Body	10714	**ATP11A**	**ATP11A**
cg16784745	8.33E-05	0.035238544	0.757616071	0.805877114	-0.048261043	FDR0.05	12	54731086	Body;Body;Body;Body;Body;Body	87	**MIR148B**	**COPZ1;MIR148B**
cg09251400	8.33E-05	0.035238544	0.811179837	0.843680042	-0.032500205	FDR0.05	11	36317254	TSS1500	-470	**PRR5L**	**PRR5L**
cg08914932	8.34E-05	0.035244584	0.835464311	0.833187212	0.002277099	FDR0.05	4	184930883	Body;Body	104375	**STOX2**	**STOX2**
cg16247817	8.35E-05	0.035249197	0.062558492	0.107182842	-0.04462435	FDR0.05	1	153747686	TSS200;TES5000;TSS200;TES5000;TSS200	-196	**SLC27A3**	**SLC27A3;INTS3**
cg09205190	8.36E-05	0.0352678	0.848621813	0.866341392	-0.017719579	FDR0.05	17	5405260	TSS1500;Body	-941	**LOC728392**	**LOC728392;NLRP1**
cg19396574	8.37E-05	0.0352678	0.830900512	0.827337026	0.003563485	FDR0.05	15	43759126	Body;Body;Body;Body	26243	**TP53BP1**	**TP53BP1**
cg08649013	8.38E-05	0.035290083	0.849156719	0.871595207	-0.022438488	FDR0.05	4	10042198	TSS1500	-326	**SLC2A9**	**SLC2A9**
cg22764782	8.44E-05	0.03551126	0.816211144	0.816138995	7.21E-05	FDR0.05	8	94831797	TES5000;TES5000;TES5000	64726	**TMEM67**	**TMEM67**
cg18315834	8.47E-05	0.035575459	0.723976004	0.778044147	-0.054068143	FDR0.05	14	64909119	Body;Body	-23097	**AKAP5**	**MTHFD1**
cg19491415	8.47E-05	0.035575459	0.128109026	0.145665514	-0.017556489	FDR0.05	10	32217948	TSS200;TSS200;TSS200;TSS200;TSS200	-144	**ARHGAP12**	**ARHGAP12**
cg20713035	8.48E-05	0.035575459	0.931384379	0.928055427	0.003328951	FDR0.05	5	14410284	Body;Body	-53807	**SNORD170**	**TRIO**
cg06072769	8.49E-05	0.035575459	0.753043089	0.771755182	-0.018712093	FDR0.05	10	50146962	Body	-24682	**LRRC18**	**WDFY4**
cg19289461	8.50E-05	0.035589209	0.925462735	0.93133411	-0.005871375	FDR0.05	16	30394812	Body;Body;TSS1500;TSS1500	-676	**SEPT1**	**ZNF48;SEPT1**
cg13283952	8.51E-05	0.035589209	0.1526159	0.129105871	0.023510029	FDR0.05	2	121412005	TES5000	3157	**Y_RNA**	**Y_RNA**
cg20451455	8.51E-05	0.035589209	0.787133748	0.796508365	-0.009374617	FDR0.05	1	3449825	Body	27529	**MIR551A**	**MEGF6**
cg20237264	8.55E-05	0.035692749	0.761599924	0.79187476	-0.030274836	FDR0.05	8	30238735	TSS5000;TES5000;TSS5000;TSS5000;TSS5000	-3281	**RBPMS**	**RBPMS;RBPMS-AS1**
cg03730385	8.56E-05	0.035692749	0.76090167	0.819040694	-0.058139023	FDR0.05	19	6825139	Body;Body;Body	52461	**VAV1**	**VAV1**

#cpg	p_adj	q_val	Meth.BPD	Meth.nonBPD	dMeth	significance	Chr	pos.hg19	locations	dist2TSS	gene.nearest	gene.Illumina
cg02563137	8.56E-05	0.035692749	0.25643093	0.304049447	-0.047618517	FDR0.05	5	140778546	Body;Body;TES5000;Body;1stExon;TSS5000;Body;Body;TSS5000;Body;Body;Body;Body;Body;Body;Body;1stExon	852	***PCDHGB5***	***PCDHGA1;PCDHGA2;PCDHGA8;PCDHGB4;PCDHGB5;PCDHGA9;PCDHGB1;PCDHGA7;PCDHGA6;PCDHGA5;PCDHGA4;PCDHGA3;PCDHGB3;PCDHGB2***
cg12060123	8.58E-05	0.035692749	0.758900228	0.78295672	-0.024056492	FDR0.05	11	5668206	TES5000;TES5000;TES5000	14722	***TRIM34***	***TRIM34;TRIM6-TRIM34***
cg11207839	8.59E-05	0.035692749	0.963189549	0.966014499	-0.00282495	FDR0.05	22	31301895	Body;Body;Body;Body;Body;Body;Body	15871	***LOC107985544***	***OSBP2;LOC107985544***
cg02697979	8.59E-05	0.035692749	0.788500699	0.826240027	-0.037739328	FDR0.05	11	34265361	Body	114194	***ABTB2***	***ABTB2***
cg25840576	8.60E-05	0.035692749	0.062623552	0.050860093	0.011763459	FDR0.05	1	29447971	TES5000;TES5000;TES5000;Body;TES5000;Body;TES5000	1042	***TMEM200B***	***EPB41;TMEM200B***
cg25125231	8.61E-05	0.035692749	0.782022906	0.780646219	0.001376687	FDR0.05	12	630773	Body	61231	***B4GALNT3***	***B4GALNT3***
cg11653271	8.61E-05	0.035692749	0.041869725	0.036465752	0.005403973	FDR0.05	16	103723	TSS200;TES5000;TSS200	-91	***POLR3K***	***SNRNP25;RHBDF1;POLR3K***
cg03404566	8.62E-05	0.035692749	0.282840628	0.297065593	-0.014224965	FDR0.05	17	6899310	Body;TSS200	-73	***ALOX12***	***ALOX12-AS1;ALOX12***
cg14207267	8.65E-05	0.035740578	0.462113043	0.45670082	0.005412223	FDR0.05	12	113655841	TSS5000;TSS5000;Body;TSS5000;Body	3058	***IQCD***	***TPCN1;IQCD***
cg03131767	8.65E-05	0.035740578	0.66970889	0.698823692	-0.029114803	FDR0.05	12	123446272	Body;Body;Body;Body;Body	4784	***ABCB9***	***ABCB9***
cg06799721	8.66E-05	0.035740578	0.844370555	0.849044286	-0.004673731	FDR0.05	5	33446800	Body;Body;Body;Body;Body;Body	5919	***TARS***	***TARS***
cg22110922	8.68E-05	0.035740578	0.859220419	0.87160441	-0.012383992	FDR0.05	18	76485587		71085	***RP11-715C4.1***	
cg01228193	8.68E-05	0.035740578	0.822999839	0.812602447	0.010397393	FDR0.05	5	149983979	Body	3338	***SYNPO***	***SYNPO***
cg03508390	8.68E-05	0.035740578	0.681233076	0.694431094	-0.013198018	FDR0.05	3	124120796	Body;Body;Body;Body;Body;Body;Body	27618	***MIR6083***	***KALRN***
cg00128732	8.69E-05	0.035740578	0.041847286	0.040437392	0.001409893	FDR0.05	5	141258637	TSS1500;TSS1500	-662	***PCDH1***	***PCDH1***
cg12974337	8.69E-05	0.035740578	0.034692162	0.030852622	0.00383954	FDR0.05	10	80734012	Body;Body;Body	93193	***ZMIZ1-AS1***	***ZMIZ1-AS1***
cg21233754	8.74E-05	0.035903875	0.018270384	0.017645564	0.00062482	FDR0.05	2	157292608	Body;TSS1500	-291	***GPD2***	***GPD2***
cg06968155	8.76E-05	0.035961447	0.092192034	0.091705668	0.000486366	FDR0.05	5	131705112	Body;TSS1500;TSS5000;TSS1500	-288	***SLC22A5***	***MIR3936HG;SLC22A5;MIR3936***
cg01301319	8.79E-05	0.03605175	0.62809385	0.609757319	0.018336531	FDR0.05	7	27153580	Body;Body	5634	***HOXA3***	***HOXA3***
cg06130714	8.84E-05	0.036186407	0.113328255	0.097893961	0.015434294	FDR0.05	17	10024297	Body	77571	***GAS7***	***GAS7***
cg27147871	8.87E-05	0.036256885	0.045027568	0.042493305	0.002534263	FDR0.05	22	21400184	TSS200;TSS200;TSS200;TSS5000	-64	***LRRC74B***	***LRRC74B;P2RX6P***
cg03604731	8.88E-05	0.036256885	0.783105963	0.768014406	0.015091557	FDR0.05	2	96824799		-12048	***AC012307.2***	
cg05636681	8.89E-05	0.036256885	0.052825891	0.045388209	0.007437682	FDR0.05	10	96163157	1stExon	972	***TBC1D12***	***TBC1D12***
cg07572052	8.89E-05	0.036256885	0.125671983	0.101082758	0.024589225	FDR0.05	1	149821168	TSS1500;TES5000;TSS1500;TSS5000;TSS5000	1172	***HIST2H2BC***	***HIST2H2AA3;HIST2H2BC;HIST2H2AA4;HIST2H3C;HIST2H3A***
cg27285056	8.90E-05	0.036256885	0.752022996	0.764289451	-0.012266456	FDR0.05	19	50868879	5'UTR	208	***NAPSA***	***NAPSA***
cg22518733	8.90E-05	0.036256885	0.127440646	0.129796682	-0.002356036	FDR0.05	17	34417575	TSS200	-69	***CCL3***	***CCL3***
cg13402847	8.91E-05	0.036256885	0.579807682	0.605858228	-0.026050546	FDR0.05	8	22844827	TSS200	-102	***RHOBTB2***	***RHOBTB2***
cg13869366	8.93E-05	0.036294382	0.729756212	0.748173869	-0.018417657	FDR0.05	11	7869666	TES5000;TSS1500	-931	***OR5E1P***	***LOC283299;OR5E1P***
cg11079354	8.94E-05	0.036297487	0.054330045	0.046604853	0.007725192	FDR0.05	6	27740361		10839	***LOC100131289***	
cg07060913	8.95E-05	0.036331654	0.069986932	0.063589779	0.006397153	FDR0.05	16	86537142	Body;Body	5324	***FENDRR***	***FENDRR***
cg27324619	9.01E-05	0.036547834	0.736141896	0.756813397	-0.020671501	FDR0.05	22	31518860	TSS200;TSS200;TSS200;TSS200;TSS200;TSS200	-48	***INPP5J***	***INPP5J***
cg10704177	9.02E-05	0.036553755	0.775650748	0.761376631	0.014274117	FDR0.05	1	62209607	Body;Body	1470	***PATJ***	***PATJ***
cg11809342	9.05E-05	0.036627107	0.746497826	0.821411928	-0.074914102	FDR0.05	2	160007430	Body;Body;Body;Body;Body;Body	-35915	***MIR6888***	***TANC1***
cg24153548	9.09E-05	0.03666096	0.086693856	0.083591911	0.003101944	FDR0.05	3	100119764	1stExon;TSS1500	-272	***LNP1***	***TOMM70;LNP1***
cg06769082	9.10E-05	0.03666096	0.045330028	0.041349468	0.003980561	FDR0.05	7	99156634	Body;5'UTR;Body;5'UTR;5'UTR;Body;Body;5'UTR;5'UTR;5'UTR;Body	178	***ZNF655***	***ZNF655***
cg26463171	9.10E-05	0.03666096	0.773008209	0.771906045	0.001102164	FDR0.05	7	116105259	Body;Body;Body;Body;Body;Body;Body;Body;Body;Body	71	***AC002066.1***	***AC002066.1;CAV2***
cg12593634	9.10E-05	0.03666096	0.808960658	0.793359275	0.015601384	FDR0.05	11	5143555		10317	***OR52A5***	
cg04943927	9.11E-05	0.03666096	0.844218002	0.849335544	-0.005117542	FDR0.05	12	12937632	Body;TSS1500	-908	***APOLD1***	***APOLD1***
cg20931951	9.12E-05	0.03666096	0.914852779	0.917430817	-0.002578039	FDR0.05	1	12508122	Body;Body	-59177	***SNORA59A***	***VPS13D***
cg11348653	9.13E-05	0.03666096	0.726160941	0.72688773	-0.000726789	FDR0.05	5	137824182		23002	***EGR1***	
cg14278345	9.13E-05	0.03666096	0.741390816	0.736501253	0.004889563	FDR0.05	22	50451054	5'UTR	1	***IL17REL***	***IL17REL***
cg05325887	9.13E-05	0.03666096	0.694134096	0.707808162	-0.013674066	FDR0.05	16	611985	Body	1564	***PRR35***	***PRR35***
cg03625951	9.14E-05	0.036664157	0.062719125	0.0539301	0.008789024	FDR0.05	6	149867442	TSS1500	-204	***PPIL4***	***PPIL4***
cg16257215	9.16E-05	0.036708987	0.732880918	0.720495761	0.012385157	FDR0.05	1	202092296	1stExon	328	***GPR37L1***	***GPR37L1***
cg12614395	9.17E-05	0.036708987	0.801298588	0.81339855	-0.012099962	FDR0.05	1	42085415	Body;Body	-135061	***EDN2***	***HIVEP3***
cg06022107	9.18E-05	0.036708987	0.025471504	0.024030134	0.00144137	FDR0.05	3	185216379	Body	466	***TMEM41A***	***TMEM41A***
cg23143553	9.19E-05	0.036708987	0.052809832	0.047330943	0.005478889	FDR0.05	6	105307836	TSS200;TSS200;TSS1500;TSS200;TSS200;TSS200;TSS200;TSS200;TSS200;TSS200;TSS1500;TSS200;TSS200;TSS200;TSS200;TSS200;TSS200;TSS200	-42	***HACE1***	***HACE1***
cg03023237	9.19E-05	0.036708987	0.724948543	0.72906381	-0.004115267	FDR0.05	16	66953047	TSS200;TSS200;TSS200;TSS200;TES5000;TES5000	-160	***CDH16***	***CDH16;RRAD***
cg18686655	9.21E-05	0.036745897	0.828451591	0.83070422	-0.002252629	FDR0.05	7	130558744	TES5000;TES5000;TES5000	2825	***MIR29A***	***LINC-PINT;MIR29A;MIR29B1***
cg04691540	9.24E-05	0.036835571	0.071685417	0.063058824	0.008626593	FDR0.05	1	179198414	1stExon;1stExon;1stExon;1stExon;1stExon	405	***ABL2***	***ABL2***
cg04773818	9.33E-05	0.037114412	0.035669524	0.025554697	0.010114826	FDR0.05	8	31496966	TSS1500	-301	***NRG1***	***NRG1***
cg10206882	9.33E-05	0.037114412	0.739838107	0.78399651	-0.044158404	FDR0.05	14	25411743	Body;Body;Body;Body;Body;Body;Body	106463	***STXBP6***	***STXBP6***
cg15733792	9.38E-05	0.037272228	0.830437125	0.826808983	0.003628142	FDR0.05	1	155935984	Body;Body;Body;Body;Body;Body	11982	***ARHGEF2***	***ARHGEF2***
cg24145685	9.38E-05	0.037272228	0.715335519	0.698910837	0.016424682	FDR0.05	19	1227852	TES5000;3'UTR	10138	***CBARP***	***CBARP;STK11***

#cpg	p_adj	q_val	Meth.BPD	Meth.nonBPD	dMeth	significance	Chr	pos.hg19	locations	dist2TSS	gene.nearest	gene.Illumina
cg11749059	9.40E-05	0.037287939	0.857268338	0.850375671	0.006892667	FDR0.05	1	19684336	Body;Body;Body;Body	45597	PQLC2	CAPZB
cg25350416	9.47E-05	0.037490405	0.029547357	0.02322032	0.006327037	FDR0.05	2	191746073	1stExon;1stExon	527	GLS	GLS
cg27107076	9.47E-05	0.037490405	0.536770797	0.497287825	0.039482971	FDR0.05	2	25050844	Body;Body	-34593	PTRHD1	ADCY3
cg16588159	9.47E-05	0.037490405	0.719382495	0.808893775	-0.08951128	FDR0.05	4	189895469		27760	RP11-818C3.1	
cg16931664	9.52E-05	0.037632604	0.324037425	0.313296341	0.010741084	FDR0.05	11	11169707	Body	30307	RP11-179A10.1	RP11-179A10.1
cg11894856	9.55E-05	0.037654032	0.866561114	0.88268137	-0.016120256	FDR0.05	12	93327173	TSS5000	-4066	EEA1	EEA1
cg06048154	9.55E-05	0.037654032	0.202163144	0.15471697	0.047446175	FDR0.05	16	1311632	TES5000	5360	TPSD1	TPSD1
cg04791601	9.55E-05	0.037654032	0.661213062	0.682738178	-0.021525116	FDR0.05	1	204159016	TES5000	6603	KISS1	KISS1
cg10950661	9.56E-05	0.037669175	0.060564985	0.051389641	0.009175344	FDR0.05	11	31849156	Body;Body	1043	PAUPAR	PAX6-AS1;PAUPAR
cg22168796	9.57E-05	0.037669175	0.180499952	0.168928111	0.011571841	FDR0.05	12	124938326	Body;Body;Body	113684	NCOR2	NCOR2
cg08309775	9.58E-05	0.037681292	0.917198033	0.901968166	0.015229867	FDR0.05	14	105707747	Body;Body;Body;Body;Body;5'UTR	6475	BRF1	BRF1
cg04321566	9.61E-05	0.037728708	0.045093022	0.032136587	0.012956435	FDR0.05	7	64466846	Body;Body;Body;Body	278	ERV3-1	ERV3-1;ERV3-1-ZNF117
cg15488596	9.61E-05	0.037728708	0.717564689	0.782635829	-0.06507114	FDR0.05	1	185253495	Body;Body	32966	IVNS1ABP	SWT1
cg27468224	9.62E-05	0.037752705	0.773104841	0.817265379	-0.044160538	FDR0.05	4	55031503	Body	-42132	snoU13	RP11-231C18.3
cg26807095	9.67E-05	0.037882064	0.845264711	0.852283803	-0.007019092	FDR0.05	6	1833916	Body;Body	223767	FOXC1	GMDS
cg23349517	9.67E-05	0.037882064	0.620027199	0.643162318	-0.02313512	FDR0.05	2	20064988	TSS5000	-3626	LINC00954	LINC00954
cg21716693	9.69E-05	0.037882064	0.14209117	0.161402954	-0.019311783	FDR0.05	6	36806882	Body;Body	338	CPNE5	CPNE5
cg09839874	9.69E-05	0.037882064	0.706385395	0.751900304	-0.045514908	FDR0.05	16	66604187	TES5000;TES5000;Body;TES5000;Body;Body;Body;Body;Body;Body;Body;Body;Body;Body;TES5000;TES5000	3838	CMTM1	CKLF;CKLF-CMTM1;CMTM1
cg20728514	9.72E-05	0.037971917	0.243919882	0.275222573	-0.031302691	FDR0.05	22	44568423	TSS1500;TES5000;TES5000;TES5000;TES5000	-412	PARVG	PARVG;PARVB
cg16817237	9.77E-05	0.038113185	0.601209463	0.586303835	0.014905628	FDR0.05	11	60793675		9301	CD6	
cg12138067	9.78E-05	0.038141585	0.84464217	0.866303969	-0.021661798	FDR0.05	18	29596358	TSS5000	-2086	RNF125	RNF125
cg02218776	9.79E-05	0.038145003	0.719818422	0.745646876	-0.025828454	FDR0.05	2	100814213		-10502	LINC01104	
cg12115557	9.83E-05	0.038223712	0.905240711	0.914413905	-0.009173194	FDR0.05	10	118544342	Body	-42257	HSPA12A	HSPA12A
cg01910330	9.84E-05	0.038223712	0.846718745	0.855007512	-0.008288767	FDR0.05	14	78428407		38833	ADCK1	
cg18876706	9.86E-05	0.038223712	0.746883602	0.746767341	0.000116261	FDR0.05	10	31107995	TES5000	14321	RP11-330O11.3	ZNF438
cg13203135	9.88E-05	0.038223712	0.5212855	0.465421063	0.055864437	FDR0.05	21	45565328	3'UTR;Body;3'UTR;TES5000;TES5000;3'UTR;3'UTR;3'UTR	11764	GATD3B	GATD3A;GATD3B
cg10089324	9.88E-05	0.038223712	0.040089202	0.035645165	0.004444038	FDR0.05	1	51984752	Body	243	EPS15	EPS15
cg16318349	9.88E-05	0.038223712	0.734903271	0.758007476	-0.023104204	FDR0.05	1	154917307	3'UTR;3'UTR;TSS5000;3'UTR	-2142	PMVK	PBXIP1;PMVK
cg26492916	9.89E-05	0.038223712	0.768382257	0.781386185	-0.013003928	FDR0.05	6	32149401	Body;TES5000;Body;TSS5000;Body;Body;Body;Body;Body;TES5000;TES5000;Body;Body;Body;TES5000	1809	MIR6833	AGER;MIR6833;AGPAT1;RNF5;PBX2;RNF5P1
cg03025283	9.90E-05	0.038223712	0.028494046	0.025485318	0.003008729	FDR0.05	13	27825526	TSS200;TSS200;TSS5000;TSS5000	-165	RPL21	RPL21P28;RPL21;SNORA27;SNORD102
cg07103504	9.90E-05	0.038223712	0.036619713	0.032782898	0.003836815	FDR0.05	10	120514672	5'UTR	86	CACUL1	CACUL1
cg12436631	9.90E-05	0.038223712	0.579166995	0.599787628	-0.020620632	FDR0.05	1	155007014	TSS1500;Body;Body	733	DCST1	DCST2;DCST1
cg02665399	9.91E-05	0.038223712	0.981587947	0.983461608	-0.001873662	FDR0.05	7	611060	Body;Body;Body;Body;Body;Body	9466	LOC101927000	PRKAR1B
cg18410110	9.91E-05	0.038223712	0.744007261	0.765029118	-0.021021857	FDR0.05	5	31963583	Body	-27318	MIR4279	PDZD2
cg11185452	9.94E-05	0.038287073	0.023364062	0.020963724	0.002400338	FDR0.05	14	69865072	TSS200;TSS200;TSS1500;TSS1500;TSS200;TSS1500;TSS1500	-23	SLC39A9	SLC39A9;ERH
cg07017636	9.97E-05	0.03834237	0.035249746	0.031920536	0.00332921	FDR0.05	1	165567312	TSS1500	-387	RP11-306I1.2	RP11-306I1.2
cg21861265	9.98E-05	0.03834237	0.877517333	0.890240669	-0.012723336	FDR0.05	1	31457938	Body;Body	-16854	SNORD103C	PUM1
cg21437596	9.98E-05	0.03834237	0.908867777	0.898641135	0.010226642	FDR0.05	1	44678191	TSS1500;TSS1500;TSS1500	-933	DMAP1	DMAP1
cg01911981	9.99E-05	0.03834237	0.932796211	0.923725028	0.009071183	FDR0.05	8	39380341	Body;Body;Body;Body	167	ADAM3A	ADAM3A
cg27065896	1.00E-04	0.03834237	0.696323346	0.722705972	-0.026382626	FDR0.05	10	97235787	Body;Body;Body;Body	-34850	SORBS1	SORBS1
cg07739604	0.0001001	0.038370487	0.119395681	0.118264071	0.00113161	FDR0.05	12	124950990	Body;Body;Body	101020	NCOR2	NCOR2
cg19763682	0.0001003	0.038421171	0.908015694	0.893968595	0.0140471	FDR0.05	13	112903621		23693	LOC101928730	
cg11851382	0.0001005	0.038445175	0.791930519	0.835279664	-0.043349145	FDR0.05	1	56996910	Body	48347	PLPP3	PLPP3
cg10772290	0.0001006	0.038445175	0.060299498	0.068975734	-0.008676235	FDR0.05	20	48184421	Body	286	PTGIS	PTGIS
cg23044178	0.0001007	0.038445532	0.613814515	0.666392191	-0.052577676	FDR0.05	11	12136405	Body;Body;Body;Body;Body;Body;Body;Body;Body;Body;Body;Body;Body;Body;Body;Body;Body	4283	MICAL2	MICAL2
cg03605361	0.0001008	0.038448806	0.876292083	0.875979046	0.000313037	FDR0.05	5	179715673	Body;Body;Body;Body;Body;Body;Body;Body;Body;Body;Body;Body	2602	MAPK9	MAPK9
cg03748158	0.0001009	0.038448806	0.822427816	0.81192863	0.010499186	FDR0.05	16	85670174	Body;Body;Body	23439	GSE1	GSE1
cg22822395	0.000101	0.038448806	0.329998158	0.304562673	0.025435486	FDR0.05	3	182434233		24403	RP11-646E18.4	
cg10716862	0.0001011	0.038448806	0.752898848	0.776955017	-0.024056169	FDR0.05	1	65674876	Body;Body;Body;Body	-55500	DNAJC6	AK4
cg06968878	0.0001012	0.038448806	0.28947182	0.252297219	0.0371746	FDR0.05	1	205400197	TES5000	-8983	LEMD1	BLACAT1
cg02194398	0.0001012	0.038448806	0.780634363	0.784854234	-0.004219871	FDR0.05	4	142556368	TSS1500;TSS1500;TSS1500	-1380	IL15	IL15
cg16401608	0.0001015	0.038553559	0.842901144	0.855112345	-0.012211201	FDR0.05	2	220523857		14046	RP11-256I23.3	
cg17694851	0.0001017	0.038562296	0.896482829	0.901202482	-0.004719653	FDR0.05	8	1707553	TES5000;TSS5000;TES5000	3947	LOC101927752	LOC101927752;CLN8
cg06494105	0.000102	0.03862471	0.835310712	0.825082386	0.010228326	FDR0.05	19	3961553	Body;Body;TSS200	-43	MIR637	DAPK3;MIR1268A;MIR637
cg05617039	0.0001021	0.03862471	0.056817411	0.050410425	0.006406987	FDR0.05	13	76055293	Body;Body;Body	1011	TBC1D4	TBC1D4
cg03847373	0.0001021	0.03862471	0.037881671	0.032077574	0.005804097	FDR0.05	5	50265767	Body	254	LINC02106	LINC02106
cg16581455	0.0001022	0.038633415	0.673059046	0.75288323	-0.079824184	FDR0.05	8	94857684	Body	1287	RP3-388N13.3	RP3-388N13.3

#cpg	p_adj	q_val	Meth.BPD	Meth.nonBPD	dMeth	significance	Chr	pos.hg19	locations	dist2TSS	gene.nearest	gene.Illumina
cg21225430	0.0001027	0.038751854	0.792127675	0.789497897	0.002629778	FDR0.05	22	39792959	TSS5000;TSS5000	-2799	***TAB1***	***TAB1***
cg05080371	0.0001027	0.038751854	0.662263383	0.668312268	-0.006048886	FDR0.05	16	88387274		-106604	***ZNF469***	
cg06958184	0.0001029	0.038818731	0.785392437	0.794654244	-0.009261806	FDR0.05	12	132963993		24598	***RP13-895J2.11***	
cg13863638	0.000103	0.038818731	0.692038084	0.758796641	-0.066758557	FDR0.05	6	7199401	Body;Body;Body;Body	91316	***RREB1***	***RREB1***
cg14156441	0.0001033	0.038891322	0.067435973	0.053912501	0.013523472	FDR0.05	8	126447096	Body;Body	2646	***TRIB1***	***TRIB1***
cg23702046	0.0001036	0.038958895	0.741877733	0.780957068	-0.039079334	FDR0.05	12	3371110	Body;Body	104178	***LOC100128253***	***TSPAN9***
cg08580308	0.000104	0.039077934	0.750722219	0.760258954	-0.009536735	FDR0.05	2	230839060	Body	-51164	***TRIP12***	***FBXO36***
cg09447105	0.0001041	0.039100516	0.626357267	0.646578988	-0.020221721	FDR0.05	12	15126020	5'UTR	65	***PDE6H***	***PDE6H***
cg07565042	0.0001045	0.039214965	0.651178207	0.688339074	-0.037160867	FDR0.05	11	10543456	Body	-12733	***MTRNR2L8***	***RNF141***
cg25149122	0.0001046	0.039214965	0.763155653	0.78129877	-0.018143117	FDR0.05	18	74201231	TSS5000;Body	-2376	***LOC101927989***	***LOC101927989;ZNF516***
cg23185921	0.0001051	0.039378786	0.022588011	0.017086543	0.005501468	FDR0.05	12	45270799	TSS1500;TSS200;TSS1500;TSS1500;Body	-166	***NELL2***	***NELL2***
cg10242379	0.0001053	0.03938465	0.055913448	0.048956983	0.006956465	FDR0.05	6	137113776	TSS200	-120	***MAP3K5***	***MAP3K5***
cg08120986	0.0001053	0.03938465	0.807909191	0.836555606	-0.028646415	FDR0.05	2	218816280	Body;Body;Body	-7429	***TNS1***	***TNS1***
cg04128563	0.0001056	0.039450434	0.023743006	0.02108593	0.002657076	FDR0.05	6	111195434	TSS1500;TSS1500;TSS1500;TSS1500;Body	-552	***AMD1***	***AMD1***
cg16429499	0.0001058	0.039489386	0.813149897	0.828277353	-0.015127456	FDR0.05	16	3597151	Body;Body	30254	***NLRC3***	***NLRC3***
cg04768501	0.000106	0.039489386	0.087417516	0.113193134	-0.025775617	FDR0.05	3	9956823	TSS5000;TSS5000;TSS5000;Body;Body;Body;Body;TSS5000;TSS5000;Body;TSS5000;TSS5000	-1934	***IL17RC***	***IL17RC;IL17RE***
cg19422019	0.0001061	0.039489386	0.837328767	0.841993045	-0.004664278	FDR0.05	21	17442803	TSS1500;TSS1500;TSS200;TSS200;TSS200	-4	***MIR99AHG***	***MIR99AHG***
cg00764632	0.0001061	0.039489386	0.705001708	0.725646219	-0.020644511	FDR0.05	1	155271211	TSS1500;5'UTR	18	***PKLR***	***PKLR***
cg01249016	0.0001062	0.039489386	0.930781163	0.936771621	-0.005990459	FDR0.05	6	30072468	Body;Body;TSS1500;Body	-548	***TRIM31-AS1***	***TRIM31;TRIM31-AS1***
cg07007165	0.0001063	0.039489386	0.64670143	0.666082178	-0.019380748	FDR0.05	17	3872418	TSS5000;TSS5000;TSS5000;TSS5000;TSS5000;TSS5000;TSS5000	-4660	***ATP2A3***	***ATP2A3***
cg13305951	0.0001064	0.039489386	0.52995132	0.510391611	0.019559709	FDR0.05	17	19314618	5'UTR	128	***RNF112***	***RNF112***
cg15134919	0.0001064	0.039489386	0.833107207	0.834885254	-0.001778046	FDR0.05	1	144942354	Body;Body;Body;Body;Body;Body;Body;Body;Body;Body;Body;Body	-9989	***PDE4DIP***	***NBPF20;PDE4DIP;NBPF9;NBPF19;LOC100996724***
cg16179607	0.0001066	0.039529592	0.668438123	0.685651602	-0.017213479	FDR0.05	2	68476020	Body	3631	***PPP3R1***	***PPP3R1***
cg06284479	0.0001067	0.039529592	0.844390325	0.855981192	-0.011590867	FDR0.05	3	37173546	Body;Body;Body;Body;Body;Body;Body;Body;Body;Body;Body;Body;Body;Body;Body;Body;Body;Body;Body	42550	***LRRFIP2***	***LRRFIP2***
cg14643415	0.0001068	0.039546566	0.791017084	0.803089334	-0.01207225	FDR0.05	3	167293056	Body;Body;Body;Body	78674	***WDR49***	***WDR49***
cg05209584	0.0001069	0.039551074	0.05241639	0.038964687	0.013451703	FDR0.05	5	127873283	1stExon	452	***FBN2***	***FBN2***
cg15868235	0.000107	0.039551074	0.611313683	0.633054176	-0.021740493	FDR0.05	3	12486114		-39816	***TSEN2***	
cg11216832	0.0001075	0.039711265	0.744658146	0.735223218	0.009434928	FDR0.05	10	104218498	TES5000;TES5000;TSS5000	-2671	***MFSD13A***	***RPARP-AS1;MFSD13A***
cg26612230	0.000108	0.039837736	0.853422947	0.87171967	-0.018296723	FDR0.05	4	95508526	Body;Body;Body;Body;Body;3'UTR;3'UTR;Body	135519	***PDLIM5***	***PDLIM5***
cg15523463	0.0001081	0.039872973	0.684920884	0.67601694	0.008903944	FDR0.05	5	150804815		-11737	***SLC36A1***	
cg23598424	0.0001086	0.040011662	0.839871971	0.840184789	-0.000312818	FDR0.05	13	97912570	Body;Body;Body	38021	***MBNL2***	***MBNL2***
cg15239238	0.0001087	0.040011662	0.162022877	0.127466046	0.034556831	FDR0.05	11	16629389	Body;TSS5000;TSS200;Body;Body	-115	***SOX6***	***SOX6***
cg08205396	0.0001089	0.040060331	0.830838064	0.833960849	-0.003122785	FDR0.05	8	142217653	3'UTR;TES5000;3'UTR	21020	***SLC45A4***	***SLC45A4***
cg02869486	0.0001091	0.040060331	0.134827595	0.10070048	0.034127115	FDR0.05	9	98112381	Body	-32390	***FANCC***	***FANCC***
cg20584732	0.0001092	0.040060331	0.37555302	0.358233793	0.017319227	FDR0.05	15	42349694		-6306	***PLA2G4E***	
cg08631890	0.0001093	0.040060331	0.046468655	0.039290185	0.00717847	FDR0.05	19	39902969	TSS1500;TSS1500;TSS1500;TSS5000;TES5000	-780	***PLEKHG2***	***PLEKHG2;MIR4530;ZFP36***
cg24998754	0.0001095	0.040060331	0.878708928	0.879953858	-0.001244931	FDR0.05	5	26669882		80224	***CTD-2533K21.4***	
cg12728097	0.0001095	0.040060331	0.790118309	0.810081934	-0.019963625	FDR0.05	1	28154501	TSS5000;TSS5000;TSS5000;TES5000	-2750	***PPP1R8***	***PPP1R8;STX12***
cg14753691	0.0001095	0.040060331	0.84494203	0.843789137	0.001152894	FDR0.05	22	19554706	TSS1500	-344	***LINC00895***	***LINC00895***
cg11759194	0.0001095	0.040060331	0.723369567	0.779709056	-0.056339489	FDR0.05	10	27447053	Body;Body;Body;TSS5000;Body;TSS5000;Body;Body;TSS5000	3301	***MASTL***	***MASTL;YME1L1***
cg10425885	0.0001097	0.040081115	0.797085633	0.79885348	-0.001767847	FDR0.05	11	2458734		-7179	***KCNQ1***	
cg21518279	0.0001099	0.040097027	0.826528459	0.852024643	-0.025496183	FDR0.05	3	27450401	Body;Body;Body;Body;Body;Body;Body;Body;Body;Body;Body;Body;Body	-39450	***NEK10***	***SLC4A7***
cg14967066	0.0001099	0.040097027	0.24629589	0.229176449	0.017119441	FDR0.05	11	314341	1stExon;TES5000	351	***IFITM1***	***IFITM1;IFITM2***
cg13792581	0.00011	0.040105992	0.681869649	0.704141914	-0.022272265	FDR0.05	20	43590115	TSS1500;TSS5000;TSS5000;TES5000;TES5000;TES5000;TSS5000;TSS5000	-1001	***TOMM34***	***TOMM34;STK4;PABPC1L;STK4-AS1***
cg15151685	0.0001104	0.040208175	0.032147031	0.02737628	0.004770751	FDR0.05	11	69453354	TSS5000	-2518	***CCND1***	***CCND1***
cg14457429	0.0001112	0.040425191	0.802637357	0.802835191	-0.000197834	FDR0.05	9	93953180		8842	***RP11-305L7.6***	
cg24051103	0.0001112	0.040425191	0.841973878	0.847645393	-0.005671515	FDR0.05	12	64211866	Body	4070	***RXYLT1-AS1***	***RXYLT1-AS1***
cg03822255	0.0001113	0.040425191	0.753265004	0.754509485	-0.001244481	FDR0.05	9	98786502	TSS5000	-2409	***LINC00092***	***LINC00092***
cg01441865	0.0001116	0.040493829	0.695207242	0.69677411	-0.001566868	FDR0.05	4	2275880	Body;Body;Body	-12141	***MXD4***	***ZFYVE28***
cg00825047	0.0001117	0.040493829	0.072266632	0.065514485	0.006752147	FDR0.05	15	40226572	Body	248	***EIF2AK4***	***EIF2AK4***
cg23621817	0.0001117	0.040493829	0.843647249	0.875447867	-0.031800618	FDR0.05	4	40337853	Body	508	***CHRNA9***	***CHRNA9***
cg20967028	0.0001119	0.040520135	0.758990577	0.805382681	-0.046392104	FDR0.05	12	14996272	5'UTR;5'UTR	195	***ART4***	***ART4***
cg19211880	0.0001121	0.040549056	0.652048963	0.733172493	-0.08112353	FDR0.05	3	134088357	TSS5000;Body;Body;Body;Body	-1653	***MIR6827***	***MIR6827;AMOTL2***
cg13641993	0.0001123	0.040598037	0.835166733	0.846871371	-0.011704638	FDR0.05	9	37559166	Body	17084	***FBXO10***	***FBXO10***
cg12801917	0.0001124	0.040598037	0.839462834	0.846943729	-0.007480894	FDR0.05	17	15417503	Body;Body;Body	-43353	***CDRT3***	***TVP23C;TVP23C-CDRT4***
cg21208686	0.0001125	0.040598037	0.59378572	0.593574364	0.000211356	FDR0.05	9	137711757	Body;Body;Body	29458	***MIR3689C***	***COL5A1;LOC101448202***

#cpg	p_adj	q_val	Meth.BPD	Meth.nonBPD	dMeth	significance	Chr	pos.hg19	locations	dist2TSS	gene.nearest	gene.Illumina
cg20071670	0.0001126	0.040614876	0.747538225	0.782733655	-0.03519543	FDR0.05	11	73661895	5'UTR	532	***DNAJB13***	***DNAJB13***
cg02235261	0.0001128	0.040641123	0.779706066	0.820855538	-0.041149472	FDR0.05	1	47693873	Body;Body;Body;Body;Body;Body	3514	***TAL1***	***TAL1***
cg12087627	0.0001129	0.040641123	0.724871422	0.718588681	0.006282741	FDR0.05	17	3911415	Body	31019	***LINC01975***	***ZZEF1***
cg14506696	0.0001131	0.040691535	0.131896168	0.111700543	0.020195626	FDR0.05	4	100737830	TSS200;TSS200	-150	***DAPP1***	***DAPP1***
cg24540109	0.0001134	0.040736428	0.034683356	0.032301996	0.002381359	FDR0.05	1	32110960	TSS200;TSS200;TSS1500	-122	***PEF1***	***PEF1***
cg00087368	0.0001134	0.040736428	0.146733445	0.116419048	0.030314398	FDR0.05	6	100896392	Body	15159	***SIM1***	***SIM1***
cg25344142	0.0001136	0.040758427	0.824351542	0.823870292	0.000481249	FDR0.05	1	230882603	TSS1500;TSS1500;TSS1500	-526	***CAPN9***	***CAPN9***
cg18085435	0.0001137	0.040771248	0.821985737	0.837844001	-0.015858264	FDR0.05	18	55400167	Body	70380	***ATP8B1***	***ATP8B1***
cg07616374	0.0001139	0.040824863	0.077073194	0.058412153	0.018661041	FDR0.05	2	45159663	Body;Body	6364	***LINC01833***	***LINC01833***
cg04808632	0.000114	0.04083573	0.82518962	0.842143314	-0.016953695	FDR0.05	13	113537050	3'UTR;3'UTR	85902	***MCF2L-AS1***	***ATP11A***
cg25745452	0.0001144	0.040941351	0.038752793	0.035302741	0.003450052	FDR0.05	10	126847349	5'UTR;5'UTR;Body;Body;5'UTR;Body;Body	338	***CTBP2***	***CTBP2***
cg23552820	0.0001145	0.04094659	0.703130694	0.74807417	-0.044943476	FDR0.05	17	64272071		8211	***RNA5SP444***	
cg11359814	0.0001148	0.040984407	0.641749314	0.607425026	0.034324287	FDR0.05	12	132972940		28854	***RP13-714J12.1***	
cg19557407	0.000115	0.040984407	0.873762714	0.887160578	-0.013397864	FDR0.05	11	129876354	TSS5000;TSS5000;TES5000	-3624	***PRDM10***	***PRDM10;LINC00167***
cg23510527	0.000115	0.040984407	0.909271529	0.915936086	-0.006664557	FDR0.05	2	234601443	Body;Body;TSS200;Body;Body;Body	-68	***UGT1A6***	***UGT1A9;UGT1A10;UGT1A6;UGT1A7;UGT1A8***
cg00068408	0.0001151	0.040984407	0.112119756	0.087293158	0.024826597	FDR0.05	5	24644878	5'UTR;5'UTR;5'UTR;5'UTR	209	***CDH10***	***CDH10***
cg06182923	0.0001152	0.040984407	0.546732105	0.615362446	-0.068630341	FDR0.05	1	33985406	Body;Body	47175	***ZSCAN20***	***CSMD2***
cg21570168	0.0001152	0.040984407	0.082183491	0.072253352	0.009930139	FDR0.05	14	102226945	TSS1500;TSS1500;TSS5000;TSS200;TSS1500	-6	***PPP2R5C***	***PPP2R5C***
cg00130866	0.0001155	0.04104861	0.692783961	0.701526316	-0.008742354	FDR0.05	8	124052565	Body;Body;Body	2098	***DERL1***	***DERL1***
cg01375445	0.0001157	0.041073745	0.043467361	0.03782561	0.005641751	FDR0.05	5	145725171	Body;TES5000;TES5000	6585	***POU4F3***	***CTC-359M8.1;POU4F3***
cg26396815	0.0001158	0.041073745	0.698858859	0.721923377	-0.023064518	FDR0.05	4	102878132	Body;Body;Body	143150	***BANK1***	***BANK1***
cg18890461	0.0001158	0.041073745	0.307347178	0.3029714	0.004375778	FDR0.05	6	31010837		-10389	***HCG22***	
cg02002207	0.000116	0.041073745	0.8403222	0.842086155	-0.001763955	FDR0.05	16	89913618	Body	18712	***SPIRE2***	***SPIRE2***
cg02134606	0.0001161	0.041073745	0.782251299	0.785729833	-0.003478533	FDR0.05	9	138901541	3'UTR	-48315	***UBAC1***	***NACC2***
cg05208050	0.0001162	0.041073745	0.863704659	0.859639264	0.004065395	FDR0.05	2	74690526	TES5000;TES5000;Body;Body	1983	***MOGS***	***WBP1;INO80B-WBP1;MOGS***
cg27490391	0.0001162	0.041073745	0.664621135	0.699625477	-0.035004342	FDR0.05	13	40152187	Body	25169	***LHFPL6***	***LHFPL6***
cg00883854	0.0001164	0.041116818	0.031815432	0.030167031	0.001648401	FDR0.05	14	50053110	Body;5'UTR;5'UTR	24	***RPS29***	***RPS29***
cg00193959	0.0001166	0.041128695	0.79772419	0.801166234	-0.003442045	FDR0.05	10	129232405	Body;Body	-115207	***NPS***	***DOCK1***
cg12504877	0.0001167	0.041128695	0.06617246	0.062731975	0.003440484	FDR0.05	20	35258570	Body;Body	16049	***SLA2***	***SLA2***
cg14913777	0.0001167	0.041128695	0.122339399	0.097768196	0.024571203	FDR0.05	10	43947405	Body;Body;Body;Body;Body;Body;Body	14847	***ZNF487***	***ZNF487***
cg01273802	0.0001168	0.041128695	0.443777298	0.412558553	0.031218744	FDR0.05	8	10871371	Body	21441	***MIR598***	***XKR6***
cg13969146	0.000117	0.041128695	0.653510018	0.663333876	-0.009823858	FDR0.05	8	30892490	TSS5000;TSS5000;TSS5000;TSS1500;Body	-1259	***PURG***	***PURG;WRN***
cg01418388	0.000117	0.041128695	0.887932704	0.89495171	-0.007019006	FDR0.05	8	15452400	Body;Body;Body	54805	***TUSC3***	***TUSC3***
cg19848082	0.0001171	0.041128695	0.902073974	0.892827179	0.009246795	FDR0.05	2	74232673	Body;Body	2855	***TET3***	***TET3***
cg13393195	0.0001174	0.041212353	0.063904034	0.060574573	0.003329461	FDR0.05	12	113659153	TSS200;TSS200;TSS1500;TSS200;TSS1500	-89	***TPCN1***	***TPCN1;IQCD***
cg17281031	0.0001176	0.041252346	0.854173196	0.862830139	-0.008656943	FDR0.05	12	128223216		82512	***RP11-749H20.1***	
cg19438469	0.0001181	0.041356725	0.032308738	0.028441503	0.003867235	FDR0.05	12	56211383	TES5000;Body;Body;TSS1500;Body	157	***SARNP***	***DNAJC14;SARNP;ORMDL2***
cg26466479	0.0001181	0.041356725	0.783284549	0.796692335	-0.013407786	FDR0.05	16	15993454		-10948	***FOPNL***	
cg17110920	0.0001183	0.041400656	0.8818015	0.863208736	0.018592764	FDR0.05	1	27893925	Body	36080	***AHDC1***	***AHDC1***
cg24563850	0.0001188	0.041549157	0.719407019	0.707325063	0.012081957	FDR0.05	10	44198568		37453	***RP11-402L1.11***	
cg07906331	0.000119	0.041557663	0.744310829	0.762799399	-0.01848857	FDR0.05	1	15783158	TSS200	-64	***CELA2A***	***CELA2A***
cg14267525	0.000119	0.041557663	0.806567145	0.840679678	-0.034112533	FDR0.05	6	138742041	TES5000;TES5000	14390	***MIR3145***	***NHSL1***
cg22886275	0.0001195	0.04167025	0.103303976	0.081142749	0.022161228	FDR0.05	7	121940992	TSS5000;TES5000;TES5000	-2719	***FEZF1-AS1***	***FEZF1-AS1;FEZF1***
cg21147829	0.0001195	0.04167025	0.726970865	0.759434455	-0.032463589	FDR0.05	12	66521713	TSS5000;Body	-2817	***LLPH-DT***	***LLPH-DT;LLPH***
cg23125506	0.0001197	0.041678714	0.344254993	0.372387533	-0.02813254	FDR0.05	15	41198534	TES5000;TSS1500	-472	***LOC105370943***	***VPS18;LOC105370943***
cg25346972	0.0001198	0.041680578	0.031680578	0.026872205	0.004808373	FDR0.05	14	70826282	Body;Body;1stExon;Body;1stExon	166	***COX16***	***SYNJ2BP-COX16;COX16***
cg10238917	0.0001199	0.041680578	0.762243886	0.791210106	-0.028966221	FDR0.05	16	30942621	Body;Body	6726	***FBXL19***	***FBXL19***
cg19591902	0.0001199	0.041680578	0.879225144	0.889455354	-0.010230209	FDR0.05	4	77653343	Body	156640	***MIR548AH***	***SHROOM3***
cg07499673	0.0001202	0.041728508	0.708471667	0.716517445	-0.008045778	FDR0.05	16	85181016	Body	10261	***LINC02139***	***LINC02139***
cg02639927	0.0001203	0.041728508	0.024253266	0.022602481	0.001650785	FDR0.05	1	77334748	Body;Body;Body	1656	***ST6GALNAC5***	***ST6GALNAC5***
cg13985979	0.0001206	0.041795368	0.952395845	0.963479208	-0.011083363	FDR0.05	9	140297969	Body;Body;Body;Body	19745	***EXD3***	***EXD3***
cg07352016	0.0001207	0.041802735	0.669301556	0.707667734	-0.038366178	FDR0.05	18	55250568	Body;Body	3366	***FECH***	***FECH***
cg23779992	0.0001208	0.041802735	0.03904194	0.037387119	0.001654821	FDR0.05	15	43213357	TSS1500	-350	***TTBK2***	***TTBK2***
cg00834924	0.0001209	0.041802735	0.123114266	0.110579789	0.012534477	FDR0.05	15	85956922	Body;Body	33076	***AKAP13***	***AKAP13***
cg08958793	0.0001211	0.041802735	0.040292443	0.036166718	0.004125725	FDR0.05	10	76970511	5'UTR;5'UTR;Body;Body;5'UTR;5'UTR;Body	600	***VDAC2***	***VDAC2***
cg18164297	0.0001211	0.041802735	0.893432935	0.88324163	0.010191306	FDR0.05	12	91349001	TSS200;TSS200	-48	***CCER1***	***CCER1***
cg00045607	0.0001212	0.041802735	0.09708442	0.079202438	0.017881982	FDR0.05	1	161039690	TES5000;5'UTR;5'UTR;5'UTR;5'UTR	70	***ARHGAP30***	***NECTIN4;ARHGAP30***
cg19455142	0.0001212	0.041802735	0.047793311	0.043293895	0.004499416	FDR0.05	17	40336161	TES5000;TES5000;3'UTR;TSS5000	1309	***HCRT***	***GHDC;HCRT;KCNH4***
cg00335735	0.0001214	0.041823076	0.636374442	0.725715807	-0.089341366	FDR0.05	2	222355484	Body;Body;Body;Body	81594	***EPHA4***	***EPHA4***

#cpg	p_adj	q_val	Meth.BPD	Meth.nonBPD	dMeth	significance	Chr	pos.hg19	locations	dist2TSS	gene.nearest	gene.Illumina
cg13163896	0.0001216	0.041851725	0.887929873	0.915426527	-0.027496653	FDR0.05	17	75414571	Body;Body;Body;Body;Body;Body;Body	13421	***SEPT9***	***SEPT9***
cg19818271	0.0001217	0.041859367	0.885397	0.894403062	-0.009006062	FDR0.05	7	77468418	TSS1500;Body;TSS1500;Body;TSS1500;Body;Body;Body;Body;Body;Body;Body;Body;TSS1500	-1026	***PHTF2***	***PHTF2***
cg18125735	0.000122	0.041905267	0.027683853	0.025141067	0.002542787	FDR0.05	19	14606804	Body;Body;Body;Body;Body	157	***GIPC1***	***GIPC1***
cg24942343	0.000122	0.041905267	0.033368898	0.028469433	0.004899465	FDR0.05	17	7155773	5'UTR;TSS1500;5'UTR;5'UTR;5'UTR;TSS1500;Body	29	***ELP5***	***ELP5;CTDNEP1***
cg17658546	0.0001221	0.041923881	0.06570479	0.057853347	0.007851443	FDR0.05	4	90032747	TSS1500	-1220	***TIGD2***	***TIGD2***
cg19897113	0.0001223	0.041948337	0.777326001	0.843163548	-0.065837547	FDR0.05	1	26083702	Body;Body	-42964	***SELENON***	***MAN1C1***
cg09533305	0.0001225	0.041986025	0.778959956	0.800641926	-0.02168197	FDR0.05	22	38853600	TSS5000	-2395	***KCNJ4***	***KCNJ4***
cg03734104	0.000123	0.04208853	0.822916323	0.840257122	-0.017340799	FDR0.05	11	32817614	TSS1500	-1410	***CCDC73***	***CCDC73***
cg18512336	0.000123	0.04208853	0.564863428	0.520468351	0.044395077	FDR0.05	8	70981068	Body	2494	***PRDM14***	***PRDM14***
cg03095773	0.0001236	0.042274329	0.699610408	0.682004821	0.017605587	FDR0.05	2	102051598	Body	39567	***RFX8***	***RFX8***
cg18991242	0.000124	0.042344775	0.725529058	0.742148555	-0.016619497	FDR0.05	7	154722142	Body;Body	1916	***PAXIP1-AS2***	***PAXIP1-AS2***
cg01471621	0.000124	0.042344775	0.821067888	0.828711267	-0.007643379	FDR0.05	6	34294716	Body;Body	65741	***NUDT3***	***RPS10-NUDT3;NUDT3***
cg21818383	0.0001242	0.042357233	0.039402351	0.034596629	0.004805721	FDR0.05	15	57179837	Body	-30450	***TCF12***	***LOC145783***
cg20671675	0.0001243	0.042371559	0.1032977	0.092087449	0.011210251	FDR0.05	7	1105420	Body;Body;Body;Body;Body	10510	***GPR146***	***C7orf50***
cg06683078	0.0001244	0.042373824	0.66823917	0.694172646	-0.025933476	FDR0.05	21	46102242	TSS200;Body;Body	-164	***KRTAP12-1***	***KRTAP12-1;TSPEAR***
cg12948524	0.0001245	0.042373824	0.772349469	0.78040128	-0.008051811	FDR0.05	3	17136203	TES5000;TES5000	161516	***MIR3714***	***PLCL2***
cg13583230	0.0001246	0.042386888	0.028850569	0.024308744	0.004541825	FDR0.05	14	24809297	TSS200	-55	***RIPK3***	***RIPK3***
cg02420024	0.0001249	0.042441494	0.851676341	0.863709115	-0.012032774	FDR0.05	15	28197292	Body;Body	147166	***OCA2***	***OCA2***
cg25647089	0.0001255	0.042613895	0.886393157	0.863704378	0.022688779	FDR0.05	13	113408304	Body;Body;Body;Body	741	***ATP11A-AS1***	***ATP11A;ATP11A-AS1***
cg07348591	0.0001258	0.042677076	0.88257657	0.874691144	0.007885426	FDR0.05	2	224902303	Body;Body;Body	1054	***SERPINE2***	***SERPINE2***
cg02956093	0.0001259	0.042677076	0.08284034	0.075059833	0.007780507	FDR0.05	14	105261335	TSS1500;Body;Body	745	***AKT1***	***AKT1***
cg06977987	0.0001261	0.042723741	0.802021442	0.826541636	-0.024520194	FDR0.05	11	4902464	TSS1500	-584	***OR51T1***	***OR51T1***
cg14284469	0.0001264	0.04279861	0.030282654	0.034750007	-0.004467353	FDR0.05	8	80525214	Body;Body	2166	***STMN2***	***STMN2***
cg13369164	0.0001265	0.042811953	0.060039073	0.051622317	0.008416756	FDR0.05	7	100810273	TSS1500;TES5000	-1421	***VGF***	***VGF;NAT16***
cg03528118	0.0001268	0.042857437	0.727772271	0.783811745	-0.056039475	FDR0.05	3	133252013		10297	***RP11-91K8.5***	
cg06176201	0.0001269	0.042857747	0.840442925	0.880121412	-0.039678488	FDR0.05	17	40963033	Body;Body;Body;3'UTR;Body;3'UTR	12129	***CNTD1***	***BECN1;CNTD1***
cg27045356	0.000127	0.042867051	0.036652512	0.033881903	0.002770609	FDR0.05	13	80055241	Body;TSS200;TSS200	-17	***NDFIP2***	***NDFIP2-AS1;NDFIP2***
cg23929834	0.0001271	0.042867051	0.036116348	0.031634341	0.004482007	FDR0.05	6	35772961	TSS200	-109	***LHFPL5***	***LHFPL5***
cg18157738	0.0001275	0.042979383	0.948458337	0.951798458	-0.003340121	FDR0.05	1	201161205	Body	1253	***IGFN1***	***IGFN1***
cg05522046	0.0001281	0.043108623	0.043850062	0.037241237	0.006608825	FDR0.05	3	52931664	TSS200;TSS200	-67	***STIMATE***	***TMEM110-MUSTN1;STIMATE***
cg16016034	0.0001281	0.043108623	0.89320155	0.889449014	0.003752536	FDR0.05	1	205579533	3'UTR	22467	***ELK4***	***ELK4***
cg05209917	0.0001282	0.043108623	0.882983561	0.891646055	-0.008662494	FDR0.05	3	1134730	5'UTR;5'UTR;5'UTR;5'UTR;5'UTR;5'UTR;5'UTR;5'UTR;5'UTR;5'UTR;5'UTR;5'UTR;5'UTR;5'UTR;5'UTR;5'UTR	389	***CNTN6***	***CNTN6***
cg01242052	0.000129	0.043295647	0.968817655	0.977411492	-0.008593837	FDR0.05	17	8732166	Body;Body;Body	-29499	***MFSD6L***	***PIK3R6***
cg08921986	0.000129	0.043295647	0.90586566	0.901954663	0.003910996	FDR0.05	3	77366501	Body;Body;Body;Body;Body	219339	***ROBO2***	***ROBO2***
cg21113776	0.0001294	0.043295647	0.897506908	0.903298169	-0.005791261	FDR0.05	7	2499915		16102	***GRIFIN***	
cg08845123	0.0001295	0.043295647	0.021544434	0.017938229	0.003606205	FDR0.05	1	62784332	Body;Body	751	***KANK4***	***KANK4***
cg04780984	0.0001296	0.043295647	0.718384653	0.782774629	-0.064389976	FDR0.05	17	56454528	Body;Body;Body	-24929	***SUPT4H1***	***RNF43***
cg05559808	0.0001298	0.043295647	0.866521346	0.88305831	-0.016536963	FDR0.05	10	29834201	Body;Body;Body;Body;TSS200	-175	***MIR604***	***SVIL;MIR604***
cg03311606	0.0001299	0.043295647	0.040498996	0.036709906	0.003789091	FDR0.05	2	173292277	Body;TSS200;TSS200;TSS200;TSS200	-5	***ITGA6***	***ITGA6***
cg20470467	0.0001299	0.043295647	0.036442008	0.032087614	0.004354394	FDR0.05	16	30886130	TSS1500;TSS1500;TSS1500;TES5000;Body	514	***MIR4519***	***MIR762HG;MIR4519;BCL7C***
cg15456115	0.0001299	0.043295647	0.791219317	0.800490251	-0.009270934	FDR0.05	19	17908598	Body	2680	***B3GNT3***	***B3GNT3***
cg04219552	0.0001299	0.043295647	0.703070921	0.710300454	-0.007229532	FDR0.05	2	72126504		243709	***CYP26B1***	
cg02368583	0.0001299	0.043295647	0.939085609	0.94462903	-0.005543421	FDR0.05	6	168390888	TES5000	6869	***KIF25-AS1***	***KIF25-AS1***
cg13091538	0.0001299	0.043295647	0.048715711	0.041520003	0.007195708	FDR0.05	2	103059886	Body	11138	***MIR4772***	***IL18RAP***
cg15128334	0.0001301	0.043295647	0.754245837	0.776230569	-0.021984733	FDR0.05	3	185215532	Body	1313	***TMEM41A***	***TMEM41A***
cg05366058	0.0001302	0.043295647	0.835755127	0.842439777	-0.00668465	FDR0.05	8	25313630	Body;TSS5000;TSS5000;TSS5000	2354	***KCTD9***	***KCTD9;CDCA2***
cg14399552	0.0001303	0.043295647	0.807607497	0.79948839	0.008119107	FDR0.05	17	41382237	TSS1500;TSS5000	-1175	***CCDC200***	***CCDC200***
cg01942646	0.0001304	0.043295647	0.651212379	0.694169165	-0.042956785	FDR0.05	1	27240694	TSS200	-127	***NR0B2***	***NR0B2***
cg27054546	0.0001304	0.043295647	0.026572908	0.024687249	0.001885659	FDR0.05	1	23495375	TSS200;TSS200	-24	***LUZP1***	***LUZP1***
cg12184157	0.0001305	0.043295647	0.836870312	0.870000728	-0.033130416	FDR0.05	20	23549495	TSS200	-109	***CST9L***	***CST9L***
cg25105522	0.0001312	0.043451653	0.585145854	0.629056814	-0.04391096	FDR0.05	17	43355089	Body	-15610	***SPATA32***	***MAP3K14***
cg27390130	0.0001312	0.043451653	0.798437778	0.807052308	-0.00861453	FDR0.05	19	45501361	TES5000;TES5000;TES5000;TSS5000	-3345	***RELB***	***CLPTM1;RELB***
cg02237342	0.0001313	0.043451653	0.285343651	0.292517633	-0.007173982	FDR0.05	22	24823519	TSS200;Body;Body;TSS200;TSS5000;TSS5000;TSS200;TSS200;TES5000	-10	***ADORA2A***	***ADORA2A;SPECC1L-ADORA2A;ADORA2A-AS1***
cg26808979	0.0001314	0.043451653	0.017328614	0.024278659	-0.006950045	FDR0.05	11	16759946	TSS1500	-201	***C11orf58***	***C11orf58***
cg03451204	0.0001315	0.043477834	0.831514066	0.818759211	0.012754855	FDR0.05	16	75282783	Body;Body;TSS1500;Body;Body;Body;Body;Body	-559	***BCAR1***	***BCAR1***
cg10055222	0.0001321	0.04362416	0.786048392	0.785842972	0.00020542	FDR0.05	7	1125111	Body;TSS1500;Body;Body;TSS1500;Body;Body;TSS5000	-1331	***GPER1***	***C7orf50;GPER1***
cg07332880	0.0001322	0.04362416	0.680200848	0.740271537	-0.060070689	FDR0.05	11	71790279	TSS1500;Body;TSS1500;TSS1500;TSS1500;TSS1500;TSS1500;TSS1500;TSS1500;Body;TSS1500;TSS1500;Body	-1097	***LRTOMT***	***LRTOMT;NUMA1***

#cpg	p_adj	q_val	Meth.BPD	Meth.nonBPD	dMeth	significance	Chr	pos.hg19	locations	dist2TSS	gene.nearest	gene.Illumina
cg22249281	0.0001325	0.043684019	0.026515291	0.023542214	0.002973077	FDR0.05	19	41082756	5'UTR;TES5000	0	***SHKBP1***	***SHKBP1;SPTBN4***
cg20672059	0.0001326	0.043684019	0.750976526	0.755177608	-0.004201082	FDR0.05	13	33594284	Body	3714	***KL***	***KL***
cg10439283	0.0001326	0.043684019	0.590414913	0.564823465	0.025591447	FDR0.05	2	98808005	Body;Body;Body;Body;Body	42048	***VWA3B***	***VWA3B***
cg00599564	0.0001328	0.043702828	0.060505671	0.056160679	0.004344991	FDR0.05	6	29943209	Body	318	***HCG9***	***HCG9***
cg20253442	0.0001329	0.043712079	0.82894015	0.826654577	0.002285573	FDR0.05	15	93152750	Body	6611	***RP11-386M24.9***	***RP11-386M24.9***
cg01559597	0.0001332	0.043779998	0.737405226	0.758012724	-0.020607497	FDR0.05	6	14716193	Body	69428	***RNU6-793P***	***RP11-146I2.1***
cg08794928	0.0001337	0.043912756	0.054331377	0.04769435	0.006637027	FDR0.05	6	144537264		65602	***STX11***	
cg15165864	0.0001338	0.04391757	0.047712351	0.042657945	0.005054406	FDR0.05	17	73201778	5'UTR;5'UTR;5'UTR	234	***NUP85***	***NUP85***
cg05302915	0.0001343	0.044035638	0.925367421	0.929769448	-0.004402027	FDR0.05	19	19312540	3'UTR;Body;3'UTR;3'UTR;3'UTR;3'UTR	1698	***NR2C2AP***	***RFXANK;NR2C2AP***
cg09757712	0.0001344	0.044035638	0.13972918	0.113803384	0.025925795	FDR0.05	8	85094742	TSS1500;TSS1500;TSS1500;TSS1500;TSS1500;TSS1500;TSS1500;TSS1500;TSS1500;TSS5000;TSS1500;TSS1500;TSS1500;TSS1500;TSS1500;TSS1500;TSS1500;TSS5000;TSS5000;TSS5000;TSS1500;TSS1500;TSS1500;TSS1500	-279	***RALYL***	***RALYL***
cg26373518	0.0001348	0.044125982	0.51504124	0.537502584	-0.022461345	FDR0.05	22	31518942	5'UTR;5'UTR;5'UTR;5'UTR;5'UTR;5'UTR	34	***INPP5J***	***INPP5J***
cg09670127	0.0001352	0.044184324	0.652189857	0.687741313	-0.035551456	FDR0.05	2	60722802	Body;Body;Body;Body;Body	57831	***BCL11A***	***BCL11A***
cg26277197	0.0001352	0.044184324	0.77365949	0.773613242	4.62E-05	FDR0.05	7	55188632	Body;Body;Body;Body;Body;Body;Body;Body;Body	11123	***EGFR***	***EGFR***
cg05568557	0.0001353	0.044184324	0.056759651	0.04829633	0.008463321	FDR0.05	16	81069748	TES5000;TES5000;TES5000;TES5000;1stExon;TSS1500	291	***ATMIN***	***CENPN;ATMIN***
cg16032408	0.0001353	0.044184324	0.140650654	0.161495258	-0.020844604	FDR0.05	3	14466411	Body;Body;Body;Body	22336	***SLC6A6***	***SLC6A6***
cg09496161	0.0001356	0.044228509	0.87497932	0.869855133	0.005124188	FDR0.05	19	54155607	TSS1500	-1331	***RNU6-698P***	***RNU6-698P***
cg23598089	0.0001362	0.044394342	0.509242496	0.489191882	0.020050614	FDR0.05	1	203652079	5'UTR;5'UTR;5'UTR;5'UTR	-46629	***SNORA77***	***ATP2B4***
cg14470398	0.0001363	0.044394342	0.034924268	0.027200436	0.007723832	FDR0.05	4	657536	Body;Body;Body;Body;Body	10571	***PDE6B***	***PDE6B***
cg21572599	0.0001365	0.044399966	0.868992702	0.873368504	-0.004375802	FDR0.05	6	44698734		-179116	***LOC105375075***	
cg27295434	0.0001365	0.044399966	0.789686933	0.785682689	0.004004244	FDR0.05	1	95009677	TSS5000;TSS5000	-2264	***F3***	***F3***
cg19508107	0.0001366	0.04441167	0.87521086	0.880631669	-0.005420809	FDR0.05	22	21335080	Body;Body;Body;TSS1500;Body	-1477	***LZTR1***	***AIFM3;LZTR1***
cg13951007	0.0001369	0.044445512	0.826545908	0.827160115	-0.000614207	FDR0.05	1	146503900	Body;Body	10699	***LOC728989***	***NBPF19;LOC728989***
cg17882660	0.000137	0.044445512	0.063904773	0.05375885	0.010145923	FDR0.05	13	100637258	Body	3249	***ZIC2***	***ZIC2***
cg21811143	0.000137	0.044445512	0.040381855	0.033832716	0.006549139	FDR0.05	2	119599748	3'UTR	6011	***EN1***	***EN1***
cg11993754	0.0001372	0.044461809	0.094425541	0.089572194	0.004853347	FDR0.05	17	37856513	1stExon;Body;1stExon;Body;Body;Body	283	***ERBB2***	***ERBB2***
cg06759993	0.0001373	0.044461809	0.829568882	0.847981391	-0.01841251	FDR0.05	6	144065203	Body;Body;Body;Body	66102	***PHACTR2***	***PHACTR2***
cg13057170	0.0001374	0.044461809	0.030697497	0.027442744	0.003254753	FDR0.05	6	149969774	5'UTR;5'UTR	166	***KATNA1***	***KATNA1***
cg24822529	0.0001376	0.044483985	0.839312677	0.844754668	-0.005441992	FDR0.05	5	134582880	Body;Body	987	***C5orf66-AS2***	***C5orf66;C5orf66-AS2***
cg01518459	0.000138	0.044595462	0.825851291	0.832717677	-0.006866387	FDR0.05	19	30199590	Body;Body;Body;Body;Body;Body;Body	6373	***C19orf12***	***C19orf12***
cg08693997	0.0001381	0.044600483	0.856689872	0.848210413	0.008479459	FDR0.05	15	95713013		-36925	***RP11-255M2.2***	
cg19741167	0.0001382	0.044608939	0.087865768	0.087724359	0.000141409	FDR0.05	2	86263270	Body	70008	***POLR1A***	***POLR1A***
cg01338705	0.0001384	0.04463383	0.924234041	0.923924183	0.000309858	FDR0.05	6	157368215	Body;Body;Body;Body	10660	***ARID1B***	***ARID1B***
cg10267872	0.0001387	0.04465057	0.884455212	0.883046381	0.001408831	FDR0.05	2	240698235	Body	13682	***LOC150935***	***LOC150935***
cg03183618	0.0001387	0.04465057	0.852254445	0.864680503	-0.012426058	FDR0.05	2	134964228	Body;Body;Body	-24537	***RNA5SP104***	***MGAT5***
cg17085238	0.0001391	0.044677446	0.940492203	0.940089893	0.00040231	FDR0.05	12	117187838	Body;Body	11743	***RNFT2***	***RNFT2***
cg15055782	0.0001391	0.044677446	0.699950043	0.714266008	-0.014315965	FDR0.05	11	129310863	Body	64983	***BARX2***	***BARX2***
cg12977318	0.0001392	0.044677446	0.042006228	0.037870952	0.004135276	FDR0.05	11	134094651	5'UTR;TSS1500	153	***VPS26B***	***VPS26B;NCAPD3***
cg27129725	0.0001393	0.044677446	0.819480457	0.809579489	0.009900967	FDR0.05	22	46777707	Body	46410	***TRMU***	***CELSR1***
cg18758585	0.0001395	0.044677446	0.266098801	0.268383099	-0.002284298	FDR0.05	1	235115601	Body	950	***RP11-443B7.3***	***RP11-443B7.3***
cg03263730	0.0001396	0.044677446	0.207047793	0.218667696	-0.011619903	FDR0.05	1	7841722	TSS5000;TES5000;TSS5000;TSS5000;TSS5000;TSS5000	-2766	***PER3***	***PER3;VAMP3***
cg04252204	0.0001397	0.044677446	0.886302931	0.871795838	0.014507094	FDR0.05	17	80401500	Body;Body;3'UTR;3'UTR;TES5000;3'UTR;3'UTR;3'UTR;3'UTR;Body;Body;TES5000;3'UTR	6102	***CYBC1***	***CYBC1;HEXD***
cg08065657	0.0001398	0.044677446	0.091208007	0.089267355	0.001940652	FDR0.05	19	52223099	Body;Body	4146	***HAS1***	***HAS1***
cg01851573	0.0001398	0.044677446	0.77963004	0.800326132	-0.020696092	FDR0.05	8	8652454	Body	92789	***CLDN23***	***MFHAS1***
cg17152981	0.0001398	0.044677446	0.222670649	0.236260397	-0.013589748	FDR0.05	6	110299835	Body;Body	377	***GPR6***	***GPR6***
cg17430228	0.0001399	0.044677446	0.790008658	0.776329182	0.013679476	FDR0.05	11	353919		-15875	***B4GALNT4***	
cg14394939	0.0001404	0.044831811	0.704935594	0.706695675	-0.001760081	FDR0.05	21	18983396	Body;Body;TSS1500	-607	***BTG3-AS1***	***BTG3;BTG3-AS1***
cg03225208	0.0001406	0.044837455	0.677186467	0.700161764	-0.022975297	FDR0.05	9	124412094	Body	82714	***DAB2IP***	***DAB2IP***
cg20688297	0.0001407	0.044856498	0.034379056	0.030628492	0.003750564	FDR0.05	19	9879335	Body;5'UTR;5'UTR;Body;5'UTR	79	***ZNF846***	***ZNF846***
cg27573593	0.000141	0.044893005	0.961911217	0.964916004	-0.003004787	FDR0.05	1	9778887	Body;Body;Body	27363	***PIK3CD***	***PIK3CD***
cg18168751	0.0001412	0.044893005	0.740765354	0.774524361	-0.033759006	FDR0.05	4	974821	Body	-5963	***IDUA***	***SLC26A1***
cg12169865	0.0001413	0.044893005	0.788759704	0.796812324	-0.00805262	FDR0.05	17	7494807	TES5000;TES5000;3'UTR;TSS1500;TES5000	-1319	***SOX15***	***MPDU1;FXR2;SOX15***
cg18664336	0.0001414	0.044893005	0.045925998	0.040399033	0.005526965	FDR0.05	17	57970265	5'UTR;TSS200;TSS200;5'UTR;5'UTR;5'UTR;TSS200;5'UTR;5'UTR;TSS200;TSS200	41	***TUBD1***	***TUBD1;RPS6KB1***
cg14732998	0.0001416	0.044893005	0.045098748	0.040957533	0.004141215	FDR0.05	19	37997831	Body;TSS200	-9	***ZNF793***	***ZNF793-AS1;ZNF793***
cg02745045	0.0001416	0.044893005	0.833371148	0.833255276	0.000115872	FDR0.05	17	63150820	Body;Body;Body	17365	***RGS9***	***RGS9***
cg00654461	0.0001417	0.044893005	0.030824167	0.028812789	0.002011378	FDR0.05	12	111180088	Body;Body	695	***PPP1CC***	***PPP1CC***
cg17451138	0.0001417	0.044893005	0.037910377	0.033181081	0.004729296	FDR0.05	12	124155880	Body;Body	221	***TCTN2***	***TCTN2***
cg16816874	0.0001417	0.044893005	0.694541166	0.642449663	0.052091502	FDR0.05	8	11578850	Body;Body;Body	13486	***GATA4***	***GATA4***

#cpg	p_adj	q_val	Meth.BPD	Meth.nonBPD	dMeth	significance	Chr	pos.hg19	locations	dist2TSS	gene.nearest	gene.Illumina
cg02062816	0.0001421	0.044965896	0.663209864	0.686310554	-0.02310069	FDR0.05	1	163166949	Body;Body;Body;Body;Body	6014	***RGS5***	***LOC101928404;RGS5***
cg22816343	0.0001422	0.044965896	0.724941987	0.79946145	-0.074519463	FDR0.05	8	26243601	TSS5000;Body;TSS5000	3079	***BNIP3L***	***BNIP3L;SDAD1P1***
cg22758697	0.0001428	0.045127026	0.069251772	0.059719989	0.009531783	FDR0.05	10	51572216	Body;Body;Body;Body;TSS200;Body;Body;Body;Body;Body;Body;Body;Body;Body;Body;TSS5000;Body;Body;Body	-147	***NCOA4***	***TIMM23B;TIMM23B-AGAP6;NCOA4***
cg12305753	0.000143	0.045164258	0.747528168	0.752080412	-0.004552244	FDR0.05	21	35883992	TSS1500;TSS1500;TES5000;TES5000;TES5000;TES5000;TES5000;TES5000;TES5000;TSS1500;Body	-355	***KCNE1***	***KCNE1;RCAN1***
cg10817479	0.0001432	0.04518337	0.824091576	0.834056814	-0.009965238	FDR0.05	8	104225713	Body;Body;Body	-41345	***BAALC-AS1***	***BAALC***
cg04442638	0.0001433	0.04518337	0.146832439	0.181211356	-0.034378917	FDR0.05	17	73873823	Body	833	***TRIM47***	***TRIM47***
cg13491462	0.0001435	0.045239288	0.025008084	0.019404894	0.00560319	FDR0.05	6	1384491	Body	810	***RP4-668J24.2***	***RP4-668J24.2***
cg26877715	0.0001438	0.045280356	0.029112777	0.021513054	0.007599723	FDR0.05	20	37353907	Body	803	***SLC32A1***	***SLC32A1***
cg09323247	0.0001439	0.045285819	0.03559184	0.030186788	0.005405052	FDR0.05	10	135208072	Body;TES5000;TES5000;TES5000;TES5000;TES5000;TES5000;TES5000	452	***MTG1***	***MTG1;PAOX***
cg19462712	0.000144	0.045285819	0.883595255	0.874161602	0.009433653	FDR0.05	19	34744703	TSS1500	-752	***KIAA0355***	***KIAA0355***
cg04118884	0.0001445	0.045375079	0.050945976	0.045196455	0.005749521	FDR0.05	2	220407999	TES5000;TSS1500;TES5000;TSS200;1stExon;TSS1500	-180	***CHPF***	***ASIC4;TMEM198;CHPF***
cg05944260	0.0001446	0.045375079	0.741734967	0.756034444	-0.014299477	FDR0.05	1	32264223	Body;Body;Body	17429	***SPOCD1***	***SPOCD1***
cg20645058	0.0001446	0.045375079	0.85982125	0.86256301	-0.00274176	FDR0.05	12	10163532	Body;TES5000;Body;Body;Body;Body	307	***CLEC12B***	***CLEC12B;LOC102724020***
cg22983092	0.0001447	0.045375079	0.781273837	0.736000464	0.045273373	FDR0.05	17	38911538	5'UTR	46	***KRT25***	***KRT25***
cg01842741	0.000145	0.045446728	0.874938901	0.89145693	-0.016518029	FDR0.05	12	113503757	Body	8096	***DTX1***	***DTX1***
cg04963153	0.0001452	0.045484597	0.671208002	0.690924608	-0.019716606	FDR0.05	15	78555966	TSS5000;TSS1500;TSS1500	-520	***DNAJA4***	***DNAJA4***
cg03739480	0.0001456	0.045502791	0.900547373	0.887193659	0.013353715	FDR0.05	12	57821471	Body;Body;Body;Body;Body;Body;Body;Body;Body;Body	3316	***R3HDM2***	***R3HDM2***
cg04915788	0.0001456	0.045502791	0.855641593	0.872135329	-0.016493736	FDR0.05	12	25156888	Body	6393	***LOC645177***	***LOC645177***
cg08203624	0.0001457	0.045502791	0.797462425	0.816368365	-0.01890594	FDR0.05	8	125284043	Body	120	***RP11-383J24.1***	***RP11-383J24.1***
cg07434944	0.0001457	0.045502791	0.739012422	0.723631921	0.015380501	FDR0.05	11	1991814	Body;Body	15008	***MRPL23***	***MRPL23***
cg03184368	0.0001458	0.045518088	0.771719281	0.794088721	-0.02236944	FDR0.05	4	40555722	Body	-37732	***RBM47***	***RBM47***
cg11670060	0.0001461	0.045554226	0.166308332	0.155979117	0.010329215	FDR0.05	5	145316416	Body	291	***SH3RF2***	***SH3RF2***
cg00463957	0.0001462	0.045554226	0.805767871	0.816624659	-0.010856788	FDR0.05	16	14583487	Body;Body;Body	-119364	***LINC02130***	***PARN***
cg20005056	0.0001463	0.045554226	0.713313925	0.694580422	0.018733503	FDR0.05	2	26398994	Body;TSS5000	3035	***GAREM2***	***GAREM2***
cg18930076	0.0001464	0.045555397	0.050881033	0.044038557	0.006842476	FDR0.05	11	64216573	Body	45	***LOC100996455***	***LOC100996455***
cg24274995	0.0001469	0.045672216	0.955597903	0.952123382	0.00347452	FDR0.05	7	39500288	Body;Body	-54343	***POU6F2-AS1***	***POU6F2***
cg15983072	0.000147	0.045672216	0.782148627	0.7860879	-0.003939274	FDR0.05	2	146688047	Body	15390	***AC062031.1***	***AC062031.1***
cg27614215	0.0001471	0.045672216	0.773693515	0.763830547	0.009862969	FDR0.05	2	129251945		-49173	***RNA5SP103***	
cg19937288	0.0001473	0.04571725	0.894059548	0.895100495	-0.001040946	FDR0.05	14	101199873	Body;Body	6672	***DLK1***	***DLK1***
cg21205276	0.0001475	0.04571725	0.022827384	0.024498359	-0.001670975	FDR0.05	9	95056231	TSS1500;TSS200;TES5000;TSS200;TES5000;TSS200;TES5000;TES5000;TSS1500	-193	***IARS***	***MIR3651;IARS;NOL8;SNORA84***
cg08339023	0.0001475	0.04571725	0.130350196	0.111060384	0.019289813	FDR0.05	12	57873732	TSS200;TSS5000;TSS1500;TSS5000;TSS5000;Body	-77	***ARHGAP9***	***ARHGAP9***
cg12991522	0.0001477	0.04571725	0.031217891	0.028231588	0.002986303	FDR0.05	16	4986661	Body	475	***PPL***	***PPL***
cg05526341	0.0001478	0.04571725	0.763065304	0.755483955	0.007581349	FDR0.05	3	66489362	Body	61483	***LRIG1***	***LRIG1***
cg08795752	0.0001479	0.04571725	0.468870051	0.434054754	0.034815297	FDR0.05	9	136293271	Body;Body;Body;Body	6152	***ADAMTS13***	***ADAMTS13***
cg11284482	0.0001479	0.04571725	0.031966238	0.028891771	0.003074467	FDR0.05	4	99388941	TES5000	190871	***TSPAN5***	***TSPAN5***
cg12802537	0.000148	0.045719131	0.895977689	0.901952963	-0.005975275	FDR0.05	1	221870819	TES5000;TES5000;TES5000	39423	***DUSP10***	***DUSP10***
cg11835355	0.0001484	0.045795739	0.773484951	0.799291021	-0.02580607	FDR0.05	22	36561746	TSS5000;5'UTR;5'UTR;TSS5000;TSS5000;TSS5000	479	***APOL3***	***APOL3***
cg07270175	0.0001485	0.045807702	0.069375764	0.057515178	0.011860586	FDR0.05	10	96123049	TSS1500	-316	***NOC3L***	***NOC3L***
cg15769724	0.0001488	0.045872005	0.028815107	0.025569201	0.003245906	FDR0.05	15	89787113	TSS200;TSS200	-80	***FANCI***	***FANCI***
cg09294072	0.0001494	0.046020926	0.450317049	0.49355989	-0.043242842	FDR0.05	14	106366523	TES5000;TES5000;TSS5000;TSS5000;TSS200;TES5000;TES5000;TES5000;TES5000;TES5000	-11	***IGHD1-14***	***AL122127.25;IGHD3-16;IGHD2-15;IGHD1-14;IGHD6-13;IGHD5-12;IGHD4-11;IGHD3-10;IGHD3-9***
cg04794690	0.0001498	0.046114044	0.194215748	0.200668597	-0.006452849	FDR0.05	1	17768059	TSS5000;TSS5000	-1809	***RCC2***	***RCC2***
cg02587120	0.0001502	0.046215015	0.832532313	0.845866663	-0.01333435	FDR0.05	15	50557873	5'UTR;5'UTR	289	***HDC***	***HDC***
cg07980405	0.0001507	0.046315634	0.032986299	0.027639608	0.005346691	FDR0.05	6	31870281	TSS1500;Body;Body;TSS5000;TSS5000	-512	***ZBTB12***	***ZBTB12;C2;EHMT2***
cg22122449	0.000151	0.046365288	0.803407675	0.801942702	0.001464973	FDR0.05	22	51176711	TSS200;1stExon	60	***ACR***	***LOC105373100;ACR***
cg17607368	0.0001511	0.046365288	0.019068072	0.018060712	0.00100736	FDR0.05	13	78272393	TSS200;1stExon;TSS1500;TSS1500	-142	***MIR3665***	***MIR3665;SLAIN1***
cg18100586	0.0001512	0.046365288	0.932942723	0.93933598	-0.006393257	FDR0.05	8	67783733	TSS200;Body;Body	-3	***MCMDC2***	***MCMDC2***
cg23696712	0.0001513	0.046365288	0.678429408	0.693820314	-0.015390906	FDR0.05	12	53228141	TES5000;TSS200;TES5000	-64	***KRT79***	***KRT78;KRT79***
cg20118857	0.0001519	0.04652031	0.032343704	0.029031826	0.003311878	FDR0.05	17	48228624	TSS1500	-746	***PPP1R9B***	***PPP1R9B***
cg12698607	0.0001522	0.04652031	0.862616582	0.857401905	0.005214676	FDR0.05	22	17660636	3'UTR;3'UTR;3'UTR;3'UTR;3'UTR;3'UTR	-14459	***HDHD5***	***ADA2***
cg14430943	0.0001522	0.04652031	0.147749277	0.141686236	0.006063041	FDR0.05	7	155556652	Body	45114	***SHH***	***RBM33***
cg02344373	0.0001522	0.04652031	0.833451921	0.805023828	0.028428092	FDR0.05	12	133201348	TES5000;TES5000;TES5000;TES5000;TES5000;TES5000;TES5000;Body;TES5000	5983	***P2RX2***	***P2RX2;POLE***
cg11441891	0.0001523	0.04652031	0.826055564	0.837610997	-0.011555432	FDR0.05	3	46719625	Body;Body;Body;Body	15521	***ALS2CL***	***ALS2CL***
cg13151006	0.0001526	0.046535986	0.869917688	0.87421294	-0.004295252	FDR0.05	15	72075835	TES5000;TES5000	-27052	***NR2E3***	***THSD4***
cg21225501	0.0001527	0.046535986	0.970871359	0.979254068	-0.008382709	FDR0.05	8	143719601	TSS1500	-347	***LOC101928087***	***LOC101928087***
cg22760304	0.0001528	0.046535986	0.624634267	0.596693746	0.027940521	FDR0.05	19	14607597	TSS1500;TSS1500;TSS1500;TSS1500;TSS1500	-636	***GIPC1***	***GIPC1***

#cpg	p_adj	q_val	Meth.BPD	Meth.nonBPD	dMeth	significance	Chr	pos.hg19	locations	dist2TSS	gene.nearest	gene.Illumina
cg08573315	0.0001529	0.046535986	0.193402017	0.236239894	-0.042837876	FDR0.05	2	62797901		-58872	***TMEM17***	
cg05353481	0.0001529	0.046535986	0.864283478	0.884444636	-0.020161158	FDR0.05	7	143806743	1stExon	68	***OR2A2***	***OR2A2***
cg13808918	0.000153	0.046535986	0.043267317	0.038211819	0.005055498	FDR0.05	2	220041352	Body;Body;TSS5000;Body;Body;Body;Body;TSS5000;TSS5000	363	***CNPPD1***	***CNPPD1;RETREG2***
cg14307157	0.0001533	0.046614068	0.725490963	0.726496802	-0.001005839	FDR0.05	15	101939976	Body;Body;Body;Body;Body;Body;Body	65335	***PCSK6-AS1***	***PCSK6***
cg24051786	0.0001541	0.046833335	0.918917815	0.911314388	0.007603427	FDR0.05	3	47514673	Body;Body	2776	***SCAP***	***SCAP***
cg07685728	0.0001551	0.047099413	0.031456665	0.026457807	0.004998858	FDR0.05	19	58280891	TSS200;TSS200;TSS200	-128	***ZNF586***	***ZNF586***
cg13233042	0.0001556	0.047213499	0.853353665	0.84281131	0.010542355	FDR0.05	11	63432489	Body;Body	6713	***ATL3***	***ATL3***
cg23250750	0.0001558	0.047213499	0.473462167	0.466991032	0.006471135	FDR0.05	2	36891490		-18260	***FEZ2***	
cg19753174	0.0001558	0.047213499	0.883556306	0.89933399	-0.015777684	FDR0.05	1	219351218	Body;Body;Body;Body;Body;TSS5000;Body;Body	2908	***LYPLAL1***	***LYPLAL1;LYPLAL1-DT***
cg03741475	0.000156	0.047218816	0.880605673	0.875702429	0.004903245	FDR0.05	2	127635614		-20825	***AC114783.1***	
cg11689753	0.0001561	0.047218816	0.825810028	0.848849497	-0.023039469	FDR0.05	10	6821048	TSS1500;Body	94	***LINC00706***	***LINC00707;LINC00706***
cg06977285	0.0001561	0.047218816	0.562266586	0.558684022	0.003582563	FDR0.05	7	18127468	Body;Body;Body;Body;Body;Body;Body	904	***HDAC9***	***HDAC9***
cg04884025	0.0001567	0.04729738	0.784378536	0.797641392	-0.013262856	FDR0.05	1	52704337	Body;Body	96572	***ZFYVE9***	***ZFYVE9***
cg06391046	0.0001568	0.04729738	0.084282001	0.091068875	-0.006786874	FDR0.05	16	81478765	TSS200	-9	***CMIP***	***CMIP***
cg05714396	0.0001569	0.04729738	0.814886192	0.833897348	-0.019011156	FDR0.05	5	81048495	TSS1500;TSS1500;TSS1500;TSS1500;TSS1500;TSS1500;TSS1500	-1423	***SSBP2***	***SSBP2***
cg24001728	0.0001569	0.04729738	0.713998808	0.693163684	0.020835123	FDR0.05	11	61058953	Body	3835	***VWCE***	***VWCE***
cg11234017	0.000157	0.04729738	0.052146456	0.046109739	0.006036716	FDR0.05	17	42767172	TSS200;TSS200	-7	***CCDC43***	***CCDC43***
cg18127003	0.0001572	0.04729738	0.881215657	0.905359354	-0.024143697	FDR0.05	8	11614472	TSS5000;Body;Body;Body	-4292	***C8orf49***	***C8orf49;GATA4***
cg01765220	0.0001572	0.04729738	0.015595341	0.017056286	-0.001460945	FDR0.05	16	89160058	TSS200;TSS200;TSS200;TSS200;TSS200;TSS200;TSS200	-158	***ACSF3***	***ACSF3***
cg17582777	0.0001573	0.04729738	0.704036496	0.719609797	-0.015573301	FDR0.05	1	155050100	TSS1500	-1247	***EFNA3***	***EFNA3***
cg24334335	0.0001576	0.047334279	0.075943385	0.079543248	-0.003599863	FDR0.05	1	113050814	TSS1500;Body;Body	-555	***WNT2B***	***WNT2B***
cg01794103	0.0001576	0.047334279	0.090263427	0.075671754	0.014591674	FDR0.05	15	78342494	Body;Body	27500	***TBC1D2B***	***TBC1D2B***
cg27540367	0.0001578	0.047334279	0.321183178	0.309918859	0.011264319	FDR0.05	19	41848134	Body	11654	***TGFB1***	***TGFB1***
cg01812599	0.0001579	0.047334279	0.185139946	0.163753736	0.021386209	FDR0.05	10	103043991		-16226	***SNORD112***	
cg20728639	0.0001579	0.047334279	0.02323028	0.021141837	0.002088443	FDR0.05	11	34379053	1stExon	502	***ABTB2***	***ABTB2***
cg10639428	0.000158	0.047334279	0.750508796	0.755735409	-0.005226614	FDR0.05	2	25138879	Body;Body	4007	***ADCY3***	***ADCY3***
cg24029926	0.0001581	0.047341461	0.035415932	0.027846055	0.007569877	FDR0.05	7	33102655	TSS1500;TSS1500	-222	***NT5C3A***	***NT5C3A***
cg21312144	0.0001587	0.047461958	0.037912322	0.035514223	0.0023981	FDR0.05	2	32235240	Body;Body;Body;Body;Body;Body;TES5000	493	***MEMO1***	***MEMO1;DPY30***
cg18239763	0.0001588	0.047461958	0.034604631	0.030553365	0.004051266	FDR0.05	12	49525542	TSS1500	-238	***TUBA1B***	***TUBA1B***
cg14555350	0.0001589	0.047461958	0.651196433	0.678075157	-0.026878724	FDR0.05	5	142580618	Body;Body;Body;Body	8554	***ARHGAP26-IT1***	***ARHGAP26***
cg14774911	0.0001592	0.047534295	0.035310759	0.029793454	0.005517305	FDR0.05	9	15553181	5'UTR;5'UTR	177	***CCDC171***	***CCDC171***
cg27509510	0.0001594	0.047534295	0.699960655	0.778918247	-0.078957592	FDR0.05	2	26200001	Body	5442	***KIF3C***	***KIF3C***
cg12469755	0.0001594	0.047534295	0.859145815	0.864840397	-0.005694582	FDR0.05	5	110937107	Body	-88812	***STARD4***	***STARD4-AS1***
cg02767539	0.0001596	0.047567826	0.076497527	0.06618443	0.010313097	FDR0.05	21	15399990	Body;Body;Body	219	***AP001347.6***	***AP001347.6***
cg16202615	0.0001601	0.047641593	0.052460083	0.046027617	0.006432466	FDR0.05	2	43454763	TES5000;TES5000;TSS1500;TES5000;TES5000;TES5000;TES5000;TES5000;Body	414	***LINC01126***	***THADA;ZFP36L2;LINC01126***
cg16661234	0.0001601	0.047641593	0.919488978	0.937607231	-0.018118254	FDR0.05	17	10541620	Body	19006	***MYH3***	***MYH3***
cg04569594	0.0001603	0.04766792	0.77406848	0.811961666	-0.037893186	FDR0.05	3	125181706	Body;Body	57352	***SNX4***	***SNX4***
cg23520688	0.0001607	0.047744396	0.774305176	0.793295976	-0.0189908	FDR0.05	14	103294740	Body;Body;Body;Body	50925	***TRAF3***	***TRAF3***
cg08962185	0.0001609	0.047778386	0.351804267	0.367054258	-0.01524999	FDR0.05	8	143336052	Body;Body	56336	***LINC00051***	***TSNARE1***
cg19747876	0.0001611	0.047787682	0.032023221	0.025144349	0.006878873	FDR0.05	17	41467024	TSS1500;TSS1500	-758	***LINC00910***	***LINC00910***
cg25668878	0.0001611	0.047787682	0.74078757	0.785177018	-0.044389448	FDR0.05	3	46256527	Body	-6640	***CCR1***	***CCR3***
cg16429927	0.0001615	0.047865682	0.12422055	0.111675882	0.012544668	FDR0.05	2	172430723		51135	***CYBRD1***	
cg11939940	0.0001616	0.047865682	0.048911044	0.043789557	0.005121488	FDR0.05	10	80828645	TSS200;TSS1500;TSS1500;TSS1500	-146	***ZMIZ1***	***ZMIZ1;ZMIZ1-AS1***
cg12219753	0.0001618	0.047868668	0.027224805	0.023272851	0.003951954	FDR0.05	18	34408901	TSS200;5'UTR;TSS200;TSS200;TSS200;TSS200;5'UTR;5'UTR;5'UTR;5'UTR;5'UTR;<br>5'UTR;5'UTR;TSS200;TSS200;TSS200;5'UTR;5'UTR;TSS200	-167	***KIAA1328***	***KIAA1328;TPGS2***
cg04713108	0.000162	0.047868668	0.821943331	0.844273824	-0.022330493	FDR0.05	5	44807541	Body;Body;Body;TSS1500;Body	1354	***MRPS30-DT***	***MRPS30-DT;MRPS30***
cg20839554	0.000162	0.047868668	0.803640973	0.822751028	-0.019110055	FDR0.05	11	1012937	TES5000;TES5000;TES5000;3'UTR;TES5000	23769	***MUC6***	***AP2A2;MUC6***
cg18428373	0.0001621	0.047868668	0.06350758	0.057333986	0.006173594	FDR0.05	9	37651257	Body	206	***FRMPD1***	***FRMPD1***
cg10329694	0.0001623	0.047868668	0.616799788	0.623092213	-0.006292425	FDR0.05	17	3592947	Body;Body;Body;Body;Body	6751	***P2RX5***	***P2RX5;P2RX5-TAX1BP3***
cg00470277	0.0001624	0.047868668	0.736124998	0.74479584	-0.008670842	FDR0.05	7	2669915	TSS5000	-1687	***TTYH3***	***TTYH3***
cg09835675	0.0001624	0.047868668	0.029621211	0.026879934	0.002741277	FDR0.05	16	30546404	TSS200;TSS200;TSS200;TSS200	-113	***ZNF747***	***ZNF747***
cg24815973	0.0001636	0.048193293	0.803568225	0.80424117	-0.000672945	FDR0.05	6	3140901	Body;Body;Body;Body	16882	***TUBB2A***	***BPHL***
cg02227036	0.0001638	0.048217671	0.95822215	0.962362628	-0.004140478	FDR0.05	16	50425329	Body	3715	***LINC02178***	***LINC02178***
cg12332239	0.0001639	0.048237448	0.502152753	0.498945684	0.003207069	FDR0.05	17	66251609	Body;Body;Body;TSS5000;Body;Body;Body;Body;Body;Body;Body;Body;Body;B<br>ody;Body;Body;Body;Body;Body;Body;Body;Body;Body;Body;Body;Body;TSS50<br>00;TSS5000	-3713	***ARSG***	***AMZ2;ARSG***
cg02721602	0.0001642	0.048275939	0.529546028	0.535627533	-0.006081505	FDR0.05	6	99755198	Body;Body;Body;Body;Body;Body;Body	42333	***FAXC***	***FAXC***
cg01219549	0.0001643	0.048290957	0.898081236	0.901060361	-0.002979125	FDR0.05	9	140683649	Body;Body;TSS1500;TES5000;Body	-791	***LOC651337***	***EHMT1;LOC651337***
cg01296364	0.0001651	0.048462458	0.021820933	0.020581654	0.001239279	FDR0.05	14	31495791	Body;Body;TSS200;Body;Body;Body;TSS200;TSS200	-57	***AP4S1***	***AP4S1;STRN3***

#cpg	p_adj	q_val	Meth.BPD	Meth.nonBPD	dMeth	significance	Chr	pos.hg19	locations	dist2TSS	gene.nearest	gene.Illumina
cg14043702	0.0001651	0.048462458	0.790420731	0.803916575	-0.013495844	FDR0.05	1	101136251		-23691	***LINC01349***	
cg11271546	0.0001656	0.048561557	0.852131252	0.848850516	0.003280736	FDR0.05	15	29269461	Body;Body;Body;Body;Body;Body;Body;Body;Body	-18254	***APBA2***	***APBA2***
cg16140253	0.0001659	0.048613782	0.222618897	0.225459916	-0.002841019	FDR0.05	1	110166563	Body;TSS1500;TSS200;Body;Body;Body	-46	***AMPD2***	***AMPD2***
cg26016267	0.0001662	0.048660138	0.770931308	0.814723663	-0.043792356	FDR0.05	11	1325784	TSS5000;Body;Body;Body;Body	-1764	***TOLLIP***	***TOLLIP***
cg18368297	0.0001662	0.048660138	0.064613782	0.062587541	0.00202624	FDR0.05	13	95364928	TSS200;TSS200;TSS5000;TES5000	-41	***SOX21-AS1***	***SOX21-AS1;SOX21;LOC101927248;LOC112268114***
cg09855692	0.0001665	0.048702845	0.07420404	0.066638526	0.007565514	FDR0.05	3	15643242	5'UTR;Body;TSS200;5'UTR;5'UTR;5'UTR;TSS1500;TSS200;1stExon;TSS200;TSS200	-9	***BTD***	***HACL1;BTD***
cg09920531	0.0001668	0.048756261	0.97295614	0.979486568	-0.006530428	FDR0.05	7	157533168	Body;Body;Body;Body;Body	-114108	***LOC100506585***	***PTPRN2***
cg16560824	0.0001669	0.048756261	0.926217875	0.939548153	-0.013330278	FDR0.05	8	142189535	Body;Body;Body;Body	49138	***SLC45A4***	***DENND3***
cg10011744	0.0001671	0.048795619	0.807679237	0.80938285	-0.001703614	FDR0.05	17	76142756	Body;TES5000	323	***C17orf99***	***C17orf99;TMC8***
cg16340446	0.0001683	0.049114628	0.874390672	0.889861078	-0.015470407	FDR0.05	3	179318612	Body;TSS5000;TSS5000;Body;TSS5000	3822	***MRPL47***	***MRPL47;NDUFB5***
cg10143301	0.0001685	0.049137566	0.0337676	0.031400892	0.002366708	FDR0.05	5	134210503	Body;Body	1044	***TXNDC15***	***TXNDC15***
cg03314158	0.0001687	0.049165515	0.040216433	0.022579621	0.017636812	FDR0.05	16	2653280	TES5000;TES5000;TES5000;TSS200	-104	***LOC652276***	***PDPK1;LOC652276***
cg01623050	0.0001689	0.049169664	0.873627539	0.879986299	-0.00635876	FDR0.05	4	14341676		-50386	***AC006296.2***	
cg25029360	0.000169	0.049177468	0.047172121	0.044639809	0.002532312	FDR0.05	8	141646502	TSS1500;TSS1500	-857	***AGO2***	***AGO2***
cg14011244	0.0001694	0.049198894	0.611860338	0.612041412	-0.000181074	FDR0.05	11	130240665	Body	55778	***LOC646383***	***LOC646383***
cg21146209	0.0001694	0.049198894	0.836195771	0.842196055	-0.006000285	FDR0.05	8	23809250		-96930	***STC1***	
cg15033036	0.0001695	0.049198894	0.029585958	0.026253977	0.003331982	FDR0.05	9	133556841	Body	-12305	***EXOSC2***	***PRDM12***
cg13612274	0.0001695	0.049198894	0.026365847	0.024199913	0.002165934	FDR0.05	14	90085330	Body	164	***FOXN3***	***FOXN3***
cg06808983	0.0001697	0.049219606	0.027645921	0.024661154	0.002984767	FDR0.05	17	42148292	Body;5'UTR;5'UTR;Body;TSS5000	195	***G6PC3***	***G6PC3;LSM12***
cg09550127	0.0001699	0.049237755	0.82865034	0.843836429	-0.015186089	FDR0.05	6	14456911		62355	***RP3-448I9.1***	
cg17101703	0.00017	0.049237755	0.516975192	0.507391613	0.00958358	FDR0.05	2	69793701	Body	-46398	***SNORA36C***	***AAK1***
cg27447219	0.0001702	0.049237755	0.889549987	0.901455857	-0.01190587	FDR0.05	13	99174373	Body;5'UTR;Body	6	***STK24***	***STK24***
cg10357038	0.0001702	0.049237755	0.863412285	0.85710497	0.006307314	FDR0.05	14	89257514	Body	1783	***EML5***	***EML5***
cg08550881	0.0001704	0.049237755	0.854492957	0.844876478	0.009616479	FDR0.05	16	84213572	Body;Body;Body;Body;Body;Body;TES5000;Body;TES5000	7104	***TAF1C***	***TAF1C;DNAAF1***
cg08963122	0.0001704	0.049237755	0.023976995	0.020363449	0.003613546	FDR0.05	2	62081768	TSS1500;TSS1500;TSS1500	-490	***FAM161A***	***FAM161A***
cg11310939	0.0001711	0.049385474	0.733439949	0.748893341	-0.015453392	FDR0.05	5	126257116	Body	109384	***MARCH3***	***MARCH3***
cg09742465	0.0001712	0.049385474	0.325949943	0.316327216	0.009622728	FDR0.05	2	8618154		-94403	***LINC00299***	
cg03195826	0.0001713	0.04938656	0.856254076	0.869409848	-0.013155773	FDR0.05	5	131628641	TSS5000	-1503	***SLC22A4***	***SLC22A4***
cg06943667	0.0001715	0.04942827	0.047070456	0.040994918	0.006075537	FDR0.05	11	12030278	Body;Body;Body;TSS200	-92	***DKK3***	***DKK3***
cg13386217	0.0001722	0.049585998	0.7053523	0.686299257	0.019053043	FDR0.05	8	2019935	Body;TSS5000	-4733	***MIR7160***	***MYOM2;MIR7160***
cg19688772	0.0001723	0.049589389	0.792951573	0.819281394	-0.026329821	FDR0.05	4	102160529	Body;Body;TES5000;Body	1500	***MIR8066***	***PPP3CA;MIR8066***
cg04361556	0.0001727	0.049589389	0.807336717	0.833052813	-0.025716096	FDR0.05	2	85034766	Body	73603	***TRABD2A***	***DNAH6***
cg27175509	0.0001728	0.049589389	0.512209118	0.52785832	-0.015649202	FDR0.05	2	149891051	TSS5000;TSS5000;TSS5000;TSS5000	-3929	***LYPD6B***	***LYPD6B***
cg08972301	0.0001728	0.049589389	0.148384512	0.16421636	-0.015831847	FDR0.05	9	139553914	TSS5000;TSS5000;TSS5000;Body;Body	607	***EGFL7***	***EGFL7;HSPC324***
cg05454635	0.0001728	0.049589389	0.68607884	0.681512434	0.004566406	FDR0.05	15	74657911	TES5000;Body;Body	642	***CYP11A1***	***PPIAP46;CYP11A1***
cg02712553	0.0001729	0.049589389	0.892066567	0.900494715	-0.008428148	FDR0.05	10	64136038	TSS1500;1stExon;1stExon;1stExon	-1152	***LOC283045***	***LOC283045;ZNF365***
cg00345621	0.000173	0.049599717	0.967081953	0.970835708	-0.003753755	FDR0.05	19	1277218	Body;TES5000;TES5000;TES5000;TES5000;TES5000	1699	***C19orf24***	***C19orf24;CIRBP***
cg26147338	0.0001732	0.049612266	0.87109192	0.873961956	-0.002870036	FDR0.05	17	8274875	Body;TSS200	-17	***KRBA2***	***KRBA2***
cg07648215	0.0001736	0.049670812	0.515858888	0.535474206	-0.019615318	FDR0.05	16	46782263	Body;TSS200	-42	***MYLK3***	***MYLK3***
cg09938408	0.0001737	0.049670812	0.89937631	0.90938184	-0.01000553	FDR0.05	15	42211840	TSS5000;Body	-1774	***EHD4-AS1***	***EHD4-AS1;EHD4***
cg25811273	0.0001739	0.049670812	0.878454972	0.864358982	0.01409599	FDR0.05	22	46836187	Body	96880	***CELSR1***	***CELSR1***
cg17528559	0.0001739	0.049670812	0.703848223	0.767045842	-0.063197619	FDR0.05	2	113571025		20127	***IL1B***	
cg23072108	0.000174	0.049670812	0.835386567	0.816767855	0.018618713	FDR0.05	11	1788457	TSS5000	-3235	***CTSD***	***CTSD***
cg13150765	0.000174	0.049670812	0.82284228	0.83432113	-0.01147885	FDR0.05	14	73336176	Body;Body;Body;Body	23181	***DPF3***	***DPF3***
cg02397497	0.0001742	0.049670812	0.049943841	0.043803464	0.006140377	FDR0.05	6	10412073	TSS1500;Body;5'UTR;TES5000;Body	-477	***TFAP2A-AS1***	***TFAP2A-AS1;TFAP2A;TFAP2A-AS2***
cg08383938	0.0001743	0.049670812	0.755348526	0.821795319	-0.066446793	FDR0.05	4	146200144		61427	***LINC02266***	
cg02941852	0.0001747	0.049727141	0.695197297	0.719025321	-0.023828024	FDR0.05	7	105030168	TSS1500;TSS1500;TSS1500;TSS1500;TSS1500;TSS1500;TSS1500	-331	***SRPK2***	***SRPK2***
cg11872231	0.0001748	0.049727141	0.672113678	0.759124882	-0.087011204	FDR0.05	2	144992352	Body;Body;Body;Body;Body;Body;Body;Body;Body;Body;Body;Body;Body;Body;Body;Body;Body;Body	2554	***GTDC1***	***GTDC1***
cg02422997	0.000175	0.049727141	0.876814737	0.879585782	-0.002771045	FDR0.05	15	100277091	TSS5000;TSS5000;TSS5000;TSS5000;TSS5000;TSS5000;TSS5000	-3442	***LYSMD4***	***LYSMD4***
cg13131577	0.000175	0.049727141	0.981480339	0.978919911	0.002560428	FDR0.05	12	132530412	Body	14644	***SNORA49***	***EP400***
cg22250145	0.0001751	0.049727141	0.049745966	0.04712644	0.002619526	FDR0.05	11	72853119	5'UTR	24	***FCHSD2***	***FCHSD2***
cg05361366	0.0001751	0.049727141	0.840142692	0.864582074	-0.024439382	FDR0.05	4	142965608	Body;Body;Body	407860	***IL15***	***INPP4B***
cg16540442	0.0001755	0.049798968	0.883327651	0.87212191	0.01120574	FDR0.05	5	140053369	TES5000;TES5000;TSS200;TES5000;TES5000;TES5000;TES5000;TES5000;TES5000	-198	***DND1***	***HARS;WDR55;DND1***
cg22262168	0.000176	0.049901871	0.066432101	0.062225116	0.004206985	FDR0.05	9	27528999	TES5000;Body	851	***MOB3B***	***IFNK;MOB3B***
cg16397701	0.0001761	0.049901871	0.770793577	0.784922806	-0.014129229	FDR0.05	12	24105160	TSS1500;TSS5000;TSS5000;Body;Body	-1194	***SOX5***	***SOX5***

#cpg	p_adj	q_val	Meth.BPD	Meth.nonBPD	dMeth	significance	Chr	pos.hg19	locations	dist2TSS	gene.nearest	gene.Illumina
cg14159895	0.0001763	0.049948965	0.023574551	0.020968427	0.002606124	FDR0.05	6	31509605	TES5000;Body;Body;Body;TES5000;TSS1500;Body;TSS1500;TES5000;TSS1500	-475	***DDX39B-AS1***	***ATP6V1G2;DDX39B;ATP6V1G2-DDX39B;DDX39B-AS1;SNORD84***
cg03158314	0.0001766	0.049997436	0.858764161	0.858635902	0.000128259	FDR0.05	16	84513159	3'UTR	-12192	***ATP2C2-AS1***	***MEAK7***
cg10851739	0.0001767	0.049998955	0.065132352	0.054984588	0.010147764	FDR0.05	12	131996019		-18523	***RP11-897M7.1***	

Table S5. The number of probes identified by differential methylation analysis.
The input files are methylation (Meth) or winsorized methylation (wins_Meth)

Input	BF	FDR1
**Meth**	**38**	**275**
**wins_Meth**	**45**	**249**
(common)	30	214
(overlap(%))	78.9%	85.9%

Table S6. Association of stochastic epimutation load (EML) with BPD and other covariates

lm = linear regression; bicor = Biweight Midcorrelation

	P(lm)	slope(lm)	P(bicor)	r(bicor)
**BPD**	**4.18E-05**	**1.48415**	**0.000656**	**0.324273**
Sex	0.097927	-0.43193	0.206817	-0.12302
GA	0.074763	-0.10236	0.055672	-0.18556
weight	7.36E-05	-0.00186	0.000856	-0.31764
Ancestry	0.620047	-0.29839	0.965815	0.004192
Maternal_smoking	0.555081	-0.16369	0.382876	-0.08521
Maternal_preeclampsia	0.40496	-0.24041	0.630895	-0.04697
Hospital	0.313718	-0.11835	0.249562	-0.11228
cg05575921	1.34E-05	-8.14556	0.00278	-0.28644
CD8T	2.83E-05	-8.53618	0.056883	-0.18467
CD4T	3.23E-07	-5.45504	0.000157	-0.35747
NK	0.008825	24.903	0.027422	0.213256
Bcell	2.28E-06	-9.78908	0.001181	-0.30945
Mono	0.004492	-8.96091	0.069374	-0.17625
Gran	0.077785	-1.62658	0.026505	-0.21451
nRBC	5.36E-09	2.851882	0.065833	0.178502
percentize_CumulativeDaysO2	0.007094	1.421932	0.001528	0.302729

Table S7A. Biological functions and pathways of the genes annotated to the differentially methylated CpG sites (FDR 5%) associated with bronchopulmonary dysplasia (BPD) risk

Pathways analyzed for 2164 genes annotated to 1581 CpGs (FDR 5%) using Ingenuity Pathway Analysis (IPA), ToppGene Suite (ToppGene), David Functional Annotation (David), and Reactome Pathway Database (Reactome) tools. P value (adjusted) indicates the significance between the number of genes differentially methylated in BPD vs nonBPD and the total number of genes in the enriched ontology, biological functions, associated diseases, or pathways (the lowest P value of similar annotations from pathway analysis tools).

Functions & pathways	*P* adjusted	Selected enriched genes	Analysis tools (*P* resource underlined)
**Cellular morphogenesis, movement and adhesion during development**			
Focal adhesion/homophilic adhesion	2.69E-03	*BAD BCAR1 CAV2 CCND1 CDC42 CDH1 CDH5 CDH10 CDH16 CELSR1 CLSTN1 CNTN6 COL4A1 COL4A2 CTNNA2 CTNND1 DOCK1 DIAPH1 EGFR ERBB2 FLT4 FYN HRAS IGSF11 ITGA1 ITGA6 ITGB1 MAPK9 MYLK3 NECTIN4 NPTN PCHD1 PCHD7 PCDHGB4 PCDHGA8 PCDHB5 PCDHGB5 PCDHGB3 PCDHGB2 PCDHGB1 PCDHGA9 PCDHGA7 PCDHGA6 PCDHGA5 PCDHGA4 PCDHGA3 PCDHGA2 PCDHGA1 PDPK1 PGF PPP1CC PRKCB PTEN RASGRF1 ROBO2 SDK1 SDK2LAMB3 THBS1 THBS4 TNXB VAV1*	<u>ToppGene</u> David IPA
Actin cytoskeleton organization	1.74E-05	*ABL2 AGER AKT1 AKT2 ARHGAP12 ARHGAP17 ARHGEF2 ARRB1 BAIAP2 BCAR1 BST1 CDC42 CDH1 CDK5R1 CELSR1 CORO1A CORO7/CORO7-PAM16 CSRP1 DIAPH1 DIAPH3 DLC1 DOCK2 DPYSL2 EDN1 EFNA5 EGFR ENAH EPB41 EPB41L2 EPHA4 ERBB2 EZR GDNF GRK2 HRAS ITGB1 KALRN LRP1 MGAT5 MICALL2 MLST8 MYH9 NCK1 NCK2 NF1 OOEP PARVB PDPK1 PHACTR1 PIK3CD PLAU PMP22 PPP1R9B PTPN1 RDX RHOF RND3 RNF5 SFRP1 SHROOM3 SIPA1L1 SORBS1 SPIRE2 TGFB1 TMOD3 TRIO UBQLN1 VAV1 WDPCP*	<u>IPA</u> David ToppGene
Cadherin signlaing	1.14E-03	*CDH1 CDH5 CDH10 CDH16 CDHR4 CELSR1 CLSTN1 CTNNA2 CTNND1 EGFR ERBB2 FRK FYN FZD5 PCDHGB4 PCHD1 PCHD7 PCDHGA8 PCDHB5 PTPN1 PCDHGB5 PCDHGB3 PCDHGB2 PCDHGB1 PCDHGA9 PCDHGA7 PCDHGA6 PCDHGA5 PCDHGA4 PCDHGA3 PCDHGA2 PCDHGA1 WNT10A WNT6 WNT2B*	<u>ToppGene</u> David
Wnt signlaing	3.45E-03	*AKT1 AKT2 CCND1 CDH1 CDH5 DKK3 FZD5 KREMEN1 KREMEN2 LRP1 POU5F1 PPP2R5C RUVBL2 SFRP1 SFRP5 SOX15 SOX21 SOX5 SOX6 TAB1 TGFB1 TLE3 UBD WNT10A WNT2B WNT6*	<u>ToppGene</u> David IPA Reactome
Ephrin singliang	9.00E-02	*BCAR1 CDC42 EFNA2 EFNA3 EFNA5 EFHA4 FYN PIK3C2B PIK3CD PIK3R6 VAV1*	<u>David</u> IPA Reactome
Abnormal morphogenesis and embryo growing	1.13E-02	*AGER AGO2 AHSA1 AKAP13 AKT1 AKT2 ALCAM APBA2 ARID1A ARRB1 BRCA1 CCL17 CCND1 CCR1 CDH1 CDH5 CLASP1 CTNND1 CTSH CYP26B1 DBNL EDN1 EEF2K EGFR ERBB2 FBN2 FOLR1 FOXC1 FOXP1 GREM1 HCRT JPH4 LCK MIR429 MYH3 NCOA1 NCOR2 NF1 NKX3-2 PBX2 PDPK1 PIK3CD PKLR PKM PLAU POLE PTEN PTGER4 RARRES2 RB1 RBPJ RNF19A RNU4A SESN2 SHH SKI STK11 TAC THBS1 VDR ZBTB16 ZC3H12C ZFHX3*	<u>IPA</u> ToppGene David
**Lung alveolarization and development**			
*Retinoic acid-retinoid X receptor-vitamin D receptor signaling, androgen receptor signaling	1.70E-02	*ADCY3 ADCY10 AKT1 AKT2 ARID1A ARID5A BRCA1 CCL5 CDC42 COL14A1 CRABP2 CST6 DHRS9 DSTN FHL2 FOXP1 HSD17B3 MAP3K5 MAPK9 NCOA1 NCOA6 NCOR2 NT5C1B NR0B2 PDPK1 PIK3CD PML PRKCB PRKAR1B PTEN RARRES1 RARRES2 RB1 RDH14 RDH5 RELB RUNX2 RXRB TGFB1 VDR ZBTB16*	<u>ToppGene</u> IPA
*Lung surfactant metabolism, alveolar epithelial development, glycosaminoglycan metabolism	2.90E-02	*AGER AHSA1 ARRB1 CAVIN2 CHST14 CTSH FNDC3B FOXP1 HS6ST1 MIR449A MIR326 NCOR2 RB1 RBPJ SPOCK2*	<u>IPA</u>
Quantity of airway cells	2.90E-02	*EPAS1 HOXAS PTEN AP3B1 GLI2 TERT THBS1 STK4 RUNX2*	<u>IPA</u>
**Hematological system function, immune and inflammation**			
*Platelet activation & coagulation	9.91E-03	*AGER AMOTL1 ARRB1 CAVIN2 CDC42 CCL17 DGKA EDN1 GNG2 H3C2 ITGA2B LCK PDK1 PDPK1 PTEN SLC39A5 THBS1*	<u>IPA</u>
Angiogenesis and vascular permeability	1.01E-02	*AKT1 ALCAM ANGPT2 ARRB1 ATP2B4 BCAS3 CAVIN2 CBLL1 CCN1 CDH1 CDH5 CDC42 CD63 COL18A1 COL4A1 CTBP2 CTNND1 DCL1 DGKA DOCK1 DOCK2 DROSHA EDN1 EEF2K EGFR EGR1 EIF4A3 EPAS1 EPS15 ERBB2 F3 FHL2 FLT4 FOXC1GATA4 GDNF GNG2 GREM1 GRIN2B GRK2 HDAC9 HS6ST1 HOXA5 HRH4 HRAS ID1 IL1B IRX1 ITGA2B ITGB1 PATJ LCK LGALS3 LMNB1 MIR4488 MYH9 NCK1 NF1 NRG1 PDK1 PDPK1 PIK3C2B PKM PLCB3 PLAU PLPP3 PLXND1 PML PRKAA1 PTEN PTPN1 RASA3 RASIP1 RGCC RIPK3 RCAN1 ROBO2 ROBO2 RUNX3 SCGB3A1 SELP SLC39A5 SPINT1 STARD13 STK11 SYNPO2 TAL1 TERC TFAP2A TGFB1 THBS4 TFAP2A THBS1 TRIB1 ZMIZ1*	<u>IPA</u> ToppGene David Reactome
Hematopoiesis and leukopoiesis	2.19E-02	*AGER AREB1 ARRB1 BAD BRCA1 CD162 CCL17 CCN1 CDC42 CXCL11 CUL4A CYTH3 DAPP1 DCAF1 DGKA FLOT2 FLT3LG FOXP1 GON4L GNG2 IKZF2 LCK mir-326 NF1 NR0B2 PBXIP1TAL1 PDPK1 PDPK1 PKM PLCB3 PSMB8 PTEN RB1 RBPJ RUNX2 RUNX3 TRPM2 TTC7A UBQLN1 VDR ZBTB16 ZFP36L2*	<u>IPA</u> DAVID
Quantity of blood cells	3.87E-05	*ABTB2 AGER ALPL ANK1 ARRB1 ARID1B BAD BCL11A BNIP3L BST1 BTG1 C13orf46 CD40 CD6 CHD8 CLK1 COL4A1 COL4A2 CPS1 CTSA DLK1 EP400 FAM110B IL18RAP KL MAP3K14 MTHFD1 MTH9 P2RX5 STX11 NPAS2 PARVB PCDH7 PHACTR1 PI3CD PKLR PML PPL PRKAA1 PRKCB RASGRP3 REXO2 RGS5 RHOF RUNX2 RUNX1T1 RUNX3 SERPINB2 SGMS1 TBC1D22A ZDHHC13 ZFP36L2*	<u>ToppGene</u> IPA

Functions & pathways	*P* adjusted	Selected enriched genes	Analysis tools (*P* resource underlined)
T cell receptor signlaing and T cell activation	1.10E-02	*AKT1 AKT2 BAD CCL17 CD6 CDC42 CYP26B1 DOCK2 FYN HLA-DQA2 HRAS IL12RB2 IL15 IL1B IL4 ITGB1 ITPR1 ITPR3 LCK MAPK9 MYH9 PDPK1 PIK3C2B PIK3CD PIK3R6 PPP3CA PPP3R1 RASSF5 RELB RPS6KB1 TGFB1 VAV1*	ToppGene David Reactome IPA
B cell receptor signlaing and B cell viability	8.46E-03	*AKT1 AKT2 BAD CD40 CDC42 CREB3L2 CREB5 DAPP1 EBF1 EGR1 HRAS IKZF2 INPP5J ITPR3 MAP3K14 MAP3K5 MAPK9 MCL1 PDPK1 PIK3CD PIK3C2B PIK3CD PIK3R6 PPP3CA PPP3R1 PRKCB PTEN RASSF5 RELB RIPK3 RPS6KB1 SHH TAC4 TGFB1 TNFSF9 TRAF3 VAV1*	IPA ToppGene
*Enucleation of erythroid precursor cells	1.44E-02	*HIPK2 RB1 MAEA RHAG*	IPA
*Megakaryocyte differentiation	2.09E-02	*AGO2 ALDH16A1 ALDH3A1 CDC42 DOCK2 GNG2 H2AC4 H3C2 ITGA2B KIF1B LCK NCOR2 NEDD8 NR0B2 PSMB8 RB1 THBS1*	
Abnormal morphology of immune cells	2.41E-02	*AGER ALPL ANK1 ARID1B ATP8B1 BAD BCL11A BECH1 BNIP3L BST1 BTG1 B4GALNT3 CD40 CD6 CDH5 CHD8 CLCN7 CLK1 DLK1 DOCK2 EPS15 FADS2MCL1 FAM110B HSPA12A IL18RAP INPP4B KL MAP3K14 MAP4K3 MTH9 MTHFD1 NLRC3 NPAS2 NPC1 P2RX5 PARVB PCDH7 PHACTR1 PPT2 RGS5 RND3 RUNX2 RUNX1T1 RUNX3 SCARF1 SERPINB2 STK24 STX11 TAC4 TRIP13 UHRF1 VDR ZFP36L2*	ToppGene IPA
Inflammatory cell infiltration and chemokine signlaing	1.15E-03	*ADAMTS13 ADCY3 ADORA2A AGER AGTRAP AKT1 AKT2 ANGPT2 ARRB1 ATG5 BCAR1 BAD BECN1 BID C2 CCL17 CCL3 CCL5 CCN1 CCNT1 CCR1 CCR3 CD38 CD40 CD6 CDC42 CFB CHRNA9 CKLF CORO1A CXCL11 CYP26B1 DBNL DLK1 DOCK2 EDN1 END2 EGFL7 EGFR EPAS1 ERBB2 EZR F3 FLT3LG GNG2 GJB2 GPR18 GRK2 GRK5 HARS1 HRAS ID1 IGHE IL15 IL18BP IL1B IL4 ITGA1 ITGB1 KAT8 KL LGALS3 LMNB1 MAP3K5 MAP4K3 NCOA2 NFKBIZ NPC1 NR0B2 NR1H3 PARD3 PDE10A PDK1 PGF PIK3CD PLAU PLCB3 PLTP PPT2 PRKAA1 PRKCB PSMB8 PTEN PTGER4 PTPN1 RARRES2 RB1 RELB RGCC RIPK3 RUNX3 S100A12 SELP SERPINE2 SERPING1 SFRP1 SLC22A5 STC1 STK11 TERC TGFB1 THBS1 TRPM2 VAV1 VDR WRN*	IPA David
TNF signaling	2.40E-01	*AKT1 AKT2 CCL5 DAB2IP CREB3L2 CREB5 EDN1 IL1B IL15 MAPL9 MAP3K14 MAP3K5 PIK3CD RIPK3 TAB1 TRAF2 TNFSF9*	David
**Cell proliferation and differentiation and tissue development**			
Arrest in cell Cycle progression	4.58E-05	*BAD BRCA1 C8orf44-SGK3/SGK3 CDC42 FHL2 FLT3LG HIPK2 KLLN LCK MCPH1 mir-193 mir-20 mir-449 NCOR2 PDPK1 PIWIL1 PTEN RB1 RRAGD SKI THBS1 USP42 ZBTB16 PKMYT1 TRIP12 WEE1*	IPA David
Muscle, bone, and skeletal system development	1.59E-02	*ADAMTS7 AGER AGTRAP AKAP13 AKT1 ALOX12 AMD1 ANGPT2 ARID5A CCL3 CCND1 CHRM5 CLASP1 CREB3L2 CSPR3 ERBB2 EDN1 EGFR EXT1 F3 FHL2 FOXP1 GAS7 GATA4 GLI2 GPER1 GREM1 GRK5 HRAS HEY2 HOXA4 IL15 ITGA6 ITGB1 KALRN KEL LDB3 MAPK9 MIR193B MIR29B1 MYOZ2 NCOA1 NDRG2 NF1 NKX3-2 NRG1 P2RX5 PDE4D PDPK1 PRKG2 PTEN PTPN1 RARRES2 RASGRF1 RB-SKI RB1 RBPJ RIPK3GRK2 RPSKB1 RUNX2 RUNX3 RXRB SCARB1 SHH SOX5 SOX6 SOX15 STC1 STK11 TERC TERT TGFB1 THBS1 TNFSF9 TRIB1 TRPM2 TRPS1 VDR WNT2B ZBTB16 ZNF609*	David IPA
*Epithelial-mesenchymal transition, extracellular matrix organization	3.46E-02	*ADAMTS13 ADAMTS7 ALPL BANP BRCA1 BTBD7 CCN1 CDC42 CLASP1 COL21A1 COL4A1 COL4A2 COL5A1 COL13A1 COL18A1 FBN2 FNDC3B ITGA2B IMPG2 LAMB3 LRIG1MATN2 MFAP3L MIR193B MUC6 NCOA1 OPTC OTOGL PDPK1 PHLDB2 PRELP PTEN RASSES2 RB1 RBPJ SPOCK2 SPON2 SLC25A33 TFIP11 TGFB1 THBS1 THSD4 TNXB UBQLN1 VEPH1 WNT6*	IPA David
Neurogenesis	9.46E-07	*ABL2 AGER ADORA2A AKT1 AKT2 ALCAM APBB1 ARF4 ARHGEF2 ARIDB ATP8B1 ASCL2 BCL11A BECN1 BEND6 CAPZB CCK CD38 CDC42 CDH1 CDK5R1 CPNE5 CREB3L2 CREB5 DBNL DST EGR1 F3 FEZF1 GAS7 GPC2 GPR37L1 GRK2 HRAS HIPK2 ID1 ISLR2 KALRN KIF1A MAP3K14 MAP3K MAP3K5 MAPK9MATN2 MDGA1 NCK1 NCK2 NCOA1 NEUROD4 NF1 NKX6-1 NRBP2 NRP2 OPCML PBX2 PDPK1 PHACTR1 PIK3C2B PIK3CD PIK3R6 PLPP3 RAB11A RELB RPS6KA2 RPS6KB1 RPS6KC1 RRAD RUNX2 RUNX3 SCARF1 SDK1 SERPINE2 SH3RF1 STK24 SPTBN4 TCF12 TMRM67 TRIB1 TRIO UNC5C VGF ZFHX3 ZSWIM6*	David IPA Reactome
Oxygen-induced retinopathy and other retinal disorders	2.89E-02	*CAVIN2 CCN1 CLK EBF1 EBF2 EBF3 FJX1 HIPK2 IGFN1 LCK PTEN RB1 RBPJ PTF1A SDK1 SDK2 TFAP2A*	IPA David
Hearing loss and inner ear development	1.20E-02	*ABCC1 CD164 LHFPL5 LMX1A POU4F3 ARSG CRYM DIAPH1 DIAPH3 EXOSC2 FOXC1 GJB2 GJB6 LRTOMT MYH9 OTOF OTOGL PMP22 KCNE1 KCNQ1 PTPRQ P2RX2 RDX SPTBN4 TRMU TSPEAR TMEM67 SLC12A7 IDUA LRTOMT*	IPA ToppGene David
**Others**			
Oxidative stress and xenobiotic metabolism signlaing	3.59E-02	*ABCC1 AGER AGTRAP AKT1 ALDH16A1 ANGPT2 BRCA1 CHST14 CCL5 CCN1 CDC42 CDH5 CHST2 CUL3 DHRS9 DOCK2 DNAJA4 DNAJC6 DNAJB1 DNAJB12 DNAJB13 DNAJC14 EDN1 HRAS HS6ST1 IL1B LCK MAP3K3 MAP3K5 MAP3K14 MAPK9 NCOA1 NCOR2 PDK1 PIK3C2B PIK3CD PIK3R6 PLCB3 PNKP PPP2R5C PSMB8 PTEN PRKCB RARRES2 RELB RB1 RRBPJ SCARB1 SLC25A33 TOP1 TRPM2 UBQLN1 UGT1A6 UGT1A9 UST VDR*	IPA

Functions & pathways		*P* adjusted	Selected enriched genes	Analysis tools (*P* resource underlined)
	Lipid (steroid, fatty acid, cholesterole) metabolism	8.20E-03	*ACAD11 ACSF3 ACSL1 ACSL6 ALDH3A1 ALOX12 BRCA1 CAP CBR4 CPT1B CYP7B1 CYP11A1 CYP8B1 DHRS9 ELOVL7 FADS2 GCDH HACL1 HMGCR HMGCS2 HSD17B8 LBR MSMO1 NPC1 PRKAA1 PTGIS SOAT1 SPMVKSLC27A3 TECR*	David IPA Reactome
	*Mitochondria function and glucose deprivation	4.35E-02	*AGER AGTRAP AKT1 ALDH3A1 ANGPT2 ARID1A CAV2 CD40 COX10 DNAJB1 EDN1 EEF2K EPAS1 GHSR HIPK2 MAPK9 MDH1 MRPL14 NF1 PDK1 PKLR PKM PPP1R1B PSMB8 PTEN RBPJ RHAG SLC25A26 SLC25A33 TERC TGFB1 THBS1 TOMM7 UBQLN1 ZBTB16*	IPA ToppGene

*Pathways enriched strongly in 386 genes annotaed with 275 CpGs cut at FDR1 %.

Table S7B. GOmeth analysis of Gene Ontology (GO)-enriched pathways for annotated genes in bronchopulmonary dysplasia (BPD) epigenome.

N=number of genes in GO term. DE=number of annotated genes in the deferrentially methyalted CpGs. P.DE=-value for over-representation of the GO term. FDR=false discovery rate.

FDR 1% Gene GO Term	ONTOLOGY	TERM	N	DE	P.DE	FDR
GO:0046965	MF	retinoid X receptor binding	17	4	9.43E-05	1
GO:0035371	CC	microtubule plus-end	23	4	0.000361	1
GO:0042974	MF	retinoic acid receptor binding	26	4	0.000484	1
GO:0044849	BP	estrous cycle	16	3	0.001239	1
GO:1990752	CC	microtubule end	33	4	0.001361	1
GO:0030331	MF	estrogen receptor binding	41	4	0.001467	1
GO:0006903	BP	vesicle targeting	44	4	0.002069	1
GO:0140297	MF	DNA-binding transcription factor binding	373	13	0.00222	1
GO:0016922	MF	nuclear receptor binding	139	7	0.002591	1
GO:0002089	BP	lens morphogenesis in camera-type eye	21	3	0.004538	1
GO:0035254	MF	glutamate receptor binding	44	4	0.004696	1
GO:0035023	BP	regulation of Rho protein signal transduction	79	5	0.005574	1
GO:0007584	BP	response to nutrient	165	7	0.005752	1
GO:0007274	BP	neuromuscular synaptic transmission	25	3	0.005897	1
GO:0035255	MF	ionotropic glutamate receptor binding	24	3	0.00645	1
GO:0099023	CC	vesicle tethering complex	60	4	0.006648	1
GO:0007266	BP	Rho protein signal transduction	127	6	0.008305	1
GO:0000184	BP	nuclear-transcribed mRNA catabolic process, nonsense-mediated decay	35	3	0.009723	1
GO:0031398	BP	positive regulation of protein ubiquitination	109	5	0.010309	1
GO:0042698	BP	ovulation cycle	68	4	0.010447	1
GO:0000956	BP	nuclear-transcribed mRNA catabolic process	109	5	0.010536	1
GO:0045807	BP	positive regulation of endocytosis	96	5	0.010872	1
GO:0003714	MF	transcription corepressor activity	172	7	0.011446	1
GO:0070884	BP	regulation of calcineurin-NFAT signaling cascade	34	3	0.011556	1
GO:0006897	BP	endocytosis	537	14	0.012084	1
GO:0008134	MF	transcription factor binding	550	15	0.012116	1
GO:0106056	BP	regulation of calcineurin-mediated signaling	35	3	0.013087	1
GO:0055072	BP	iron ion homeostasis	76	4	0.013504	1
GO:0050775	BP	positive regulation of dendrite morphogenesis	32	3	0.013545	1
GO:0031670	BP	cellular response to nutrient	43	3	0.014128	1
GO:0097028	BP	dendritic cell differentiation	44	3	0.015885	1
GO:0061629	MF	RNA polymerase II-specific DNA-binding transcription factor binding	288	9	0.016584	1
GO:0007595	BP	lactation	42	3	0.017036	1
GO:0031593	MF	polyubiquitin modification-dependent protein binding	47	3	0.017831	1
GO:0030038	BP	contractile actin filament bundle assembly	103	5	0.018113	1
GO:0043149	BP	stress fiber assembly	103	5	0.018113	1
GO:0071674	BP	mononuclear cell migration	187	6	0.018491	1
GO:1903322	BP	positive regulation of protein modification by small protein conjugation or removal	128	5	0.019862	1
GO:0030100	BP	regulation of endocytosis	204	7	0.02031	1
GO:0031032	BP	actomyosin structure organization	192	7	0.020866	1
GO:0030036	BP	actin cytoskeleton organization	678	17	0.02088	1
GO:0006414	BP	translational elongation	53	3	0.021575	1
GO:0003712	MF	transcription coregulator activity	451	12	0.021978	1
GO:0042982	BP	amyloid precursor protein metabolic process	90	4	0.022125	1
GO:0016567	BP	protein ubiquitination	751	16	0.022593	1
GO:0030863	CC	cortical cytoskeleton	102	5	0.02358	1
GO:0010770	BP	positive regulation of cell morphogenesis involved in differentiation	73	4	0.024196	1
GO:0033173	BP	calcineurin-NFAT signaling cascade	43	3	0.025135	1
GO:1902991	BP	regulation of amyloid precursor protein catabolic process	53	3	0.02624	1
GO:0009988	BP	cell-cell recognition	67	3	0.027023	1
GO:0000122	BP	negative regulation of transcription by RNA polymerase II	831	19	0.029045	1
GO:0051017	BP	actin filament bundle assembly	152	6	0.029479	1
GO:0030864	CC	cortical actin cytoskeleton	75	4	0.029708	1
GO:0007265	BP	Ras protein signal transduction	314	9	0.031445	1
GO:0061572	BP	actin filament bundle organization	155	6	0.032606	1
GO:0097720	BP	calcineurin-mediated signaling	48	3	0.032935	1
GO:0051489	BP	regulation of filopodium assembly	44	3	0.035038	1
GO:0006898	BP	receptor-mediated endocytosis	235	7	0.036588	1
GO:0097435	BP	supramolecular fiber organization	703	16	0.037159	1
GO:0048511	BP	rhythmic process	289	8	0.038668	1
GO:0032446	BP	protein modification by small protein conjugation	805	16	0.039868	1
GO:0016604	CC	nuclear body	782	16	0.040734	1
GO:0051402	BP	neuron apoptotic process	234	7	0.040929	1
GO:0030514	BP	negative regulation of BMP signaling pathway	49	3	0.041161	1
GO:0043388	BP	positive regulation of DNA binding	56	3	0.041592	1
GO:0099072	BP	regulation of postsynaptic membrane neurotransmitter receptor levels	56	3	0.043185	1
GO:0042987	BP	amyloid precursor protein catabolic process	66	3	0.043557	1
GO:0005096	MF	GTPase activator activity	424	11	0.044254	1
GO:0046631	BP	alpha-beta T cell activation	148	5	0.044861	1

FDR 5% Gene GO Term	ONTOLOGY	TERM	N	DE	P.DE	FDR
GO:0019903	MF	protein phosphatase binding	146	26	2.73E-05	0.618695
GO:0030036	BP	actin cytoskeleton organization	678	82	9.63E-05	1
GO:0019902	MF	phosphatase binding	192	29	0.000219	1
GO:0099170	BP	postsynaptic modulation of chemical synaptic transmission	15	6	0.000586	1
GO:0030029	BP	actin filament-based process	760	85	0.000924	1
GO:0097435	BP	supramolecular fiber organization	703	76	0.001285	1
GO:0007015	BP	actin filament organization	419	51	0.00129	1
GO:0051721	MF	protein phosphatase 2A binding	30	8	0.001519	1
GO:0036473	BP	cell death in response to oxidative stress	90	14	0.001864	1
GO:0098685	CC	Schaffer collateral - CA1 synapse	74	15	0.002023	1
GO:0022011	BP	myelination in peripheral nervous system	29	8	0.002213	1
GO:0032292	BP	peripheral nervous system axon ensheathment	29	8	0.002213	1
GO:0032956	BP	regulation of actin cytoskeleton organization	342	41	0.002392	1
GO:0072522	BP	purine-containing compound biosynthetic process	185	23	0.002487	1
GO:0030295	MF	protein kinase activator activity	93	15	0.002872	1
GO:0014044	BP	Schwann cell development	31	8	0.003263	1
GO:0032970	BP	regulation of actin filament-based process	379	44	0.004038	1
GO:0019209	MF	kinase activator activity	99	15	0.004098	1
GO:0007254	BP	JNK cascade	160	22	0.004437	1
GO:0060964	BP	regulation of gene silencing by miRNA	47	10	0.004533	1
GO:0048016	BP	inositol phosphate-mediated signaling	55	11	0.004698	1
GO:0036490	BP	regulation of translation in response to endoplasmic reticulum stress	10	4	0.005476	1
GO:0098945	CC	intrinsic component of presynaptic active zone membrane	16	5	0.006137	1
GO:0060147	BP	regulation of posttranscriptional gene silencing	50	10	0.006325	1
GO:0006164	BP	purine nucleotide biosynthetic process	176	21	0.006415	1
GO:0060966	BP	regulation of gene silencing by RNA	51	10	0.006935	1
GO:0042110	BP	T cell activation	460	45	0.007084	1
GO:0051865	BP	protein autoubiquitination	71	11	0.007104	1
GO:0060965	BP	negative regulation of gene silencing by miRNA	21	6	0.007259	1
GO:0005096	MF	GTPase activator activity	424	48	0.007329	1
GO:0016358	BP	dendrite development	226	31	0.007695	1
GO:0035023	BP	regulation of Rho protein signal transduction	79	13	0.008069	1
GO:0030695	MF	GTPase regulator activity	449	50	0.008168	1
GO:0060589	MF	nucleoside-triphosphatase regulator activity	449	50	0.008168	1
GO:0034599	BP	cellular response to oxidative stress	276	30	0.008175	1
GO:0009895	BP	negative regulation of catabolic process	294	33	0.008313	1
GO:1903799	BP	negative regulation of production of miRNAs involved in gene silencing by miR	15	5	0.008758	1
GO:0005942	CC	phosphatidylinositol 3-kinase complex	26	6	0.00878	1
GO:0035091	MF	phosphatidylinositol binding	259	31	0.009058	1
GO:0098858	CC	actin-based cell projection	212	27	0.00921	1
GO:0046965	MF	retinoid X receptor binding	17	5	0.009444	1
GO:0046328	BP	regulation of JNK cascade	130	18	0.00961	1
GO:0031330	BP	negative regulation of cellular catabolic process	238	27	0.009862	1
GO:0120016	MF	sphingolipid transfer activity	10	3	0.010404	1
GO:0099059	CC	integral component of presynaptic active zone membrane	12	4	0.010451	1
GO:0060149	BP	negative regulation of posttranscriptional gene silencing	23	6	0.010478	1
GO:0060967	BP	negative regulation of gene silencing by RNA	23	6	0.010478	1
GO:0030296	MF	protein tyrosine kinase activator activity	18	5	0.010566	1
GO:0045214	BP	sarcomere organization	42	8	0.010598	1
GO:0048813	BP	dendrite morphogenesis	135	21	0.010747	1
GO:1903201	BP	regulation of oxidative stress-induced cell death	69	10	0.010861	1
GO:0046649	BP	lymphocyte activation	658	60	0.011387	1
GO:0001931	CC	uropod	12	4	0.011899	1
GO:0031254	CC	cell trailing edge	12	4	0.011899	1
GO:1902187	BP	negative regulation of viral release from host cell	13	4	0.012093	1
GO:0050848	BP	regulation of calcium-mediated signaling	71	11	0.0123	1
GO:1902959	BP	regulation of aspartic-type endopeptidase activity involved in amyloid precurso	10	4	0.012329	1
GO:0008154	BP	actin polymerization or depolymerization	206	25	0.012546	1
GO:0098641	MF	cadherin binding involved in cell-cell adhesion	18	5	0.01255	1
GO:1902186	BP	regulation of viral release from host cell	25	6	0.012793	1
GO:0007163	BP	establishment or maintenance of cell polarity	212	27	0.012866	1
GO:0001772	CC	immunological synapse	44	8	0.013312	1
GO:0045296	MF	cadherin binding	325	36	0.013503	1
GO:0051493	BP	regulation of cytoskeleton organization	500	51	0.013615	1
GO:0042974	MF	retinoic acid receptor binding	26	6	0.014049	1
GO:0005938	CC	cell cortex	298	36	0.01415	1
GO:0043087	BP	regulation of GTPase activity	325	37	0.014236	1
GO:0050849	BP	negative regulation of calcium-mediated signaling	21	5	0.014583	1
GO:0014037	BP	Schwann cell differentiation	39	8	0.015014	1

FDR 1% Gene GO Term	ONTOLOGY	TERM	N	DE	P.DE	FDR
GO:0016605	CC	PML body	98	4	0.045297	1
GO:0010769	BP	regulation of cell morphogenesis involved in differentiation	90	4	0.045424	1
GO:0031122	BP	cytoplasmic microtubule organization	56	3	0.045473	1
GO:0070997	BP	neuron death	347	9	0.045639	1
GO:0046579	BP	positive regulation of Ras protein signal transduction	58	3	0.046541	1
GO:0006513	BP	protein monoubiquitination	64	3	0.049028	1
GO:0048016	BP	inositol phosphate-mediated signaling	55	3	0.049861	1

FDR 5% Gene GO Term	ONTOLOGY	TERM	N	DE	P.DE	FDR
GO:0035672	BP	oligopeptide transmembrane transport	14	4	0.015169	1
GO:0016311	BP	dephosphorylation	397	40	0.015206	1
GO:0030041	BP	actin filament polymerization	180	22	0.015564	1
GO:0005858	CC	axonemal dynein complex	23	5	0.015807	1
GO:0051056	BP	regulation of small GTPase mediated signal transduction	281	34	0.015981	1
GO:0030027	CC	lamellipodium	195	25	0.016108	1
GO:1905244	BP	regulation of modification of synaptic structure	12	4	0.016563	1
GO:0050773	BP	regulation of dendrite development	95	15	0.016624	1
GO:0005543	MF	phospholipid binding	440	45	0.017872	1
GO:1903798	BP	regulation of production of miRNAs involved in gene silencing by miRNA	25	6	0.018336	1
GO:0060969	BP	negative regulation of gene silencing	27	6	0.018532	1
GO:1903901	BP	negative regulation of viral life cycle	23	5	0.018726	1
GO:0009152	BP	purine ribonucleotide biosynthetic process	157	18	0.018759	1
GO:0060343	BP	trabecula formation	22	5	0.018842	1
GO:0043525	BP	positive regulation of neuron apoptotic process	55	9	0.019129	1
GO:0033866	BP	nucleoside bisphosphate biosynthetic process	56	9	0.019175	1
GO:0034030	BP	ribonucleoside bisphosphate biosynthetic process	56	9	0.019175	1
GO:0034033	BP	purine nucleoside bisphosphate biosynthetic process	56	9	0.019175	1
GO:0009894	BP	regulation of catabolic process	903	80	0.019307	1
GO:0009165	BP	nucleotide biosynthetic process	235	24	0.019445	1
GO:0070920	BP	regulation of production of small RNA involved in gene silencing by RNA	26	6	0.019498	1
GO:1905245	BP	regulation of aspartic-type peptidase activity	12	4	0.019869	1
GO:0033173	BP	calcineurin-NFAT signaling cascade	43	8	0.020122	1
GO:0035196	BP	production of miRNAs involved in gene silencing by miRNA	51	9	0.020152	1
GO:0005911	CC	cell-cell junction	469	49	0.020192	1
GO:1901293	BP	nucleoside phosphate biosynthetic process	237	24	0.020826	1
GO:0072677	BP	eosinophil migration	23	4	0.021417	1
GO:0071364	BP	cellular response to epidermal growth factor stimulus	44	8	0.021619	1
GO:0004674	MF	protein serine/threonine kinase activity	358	39	0.022174	1
GO:0120014	MF	phospholipid transfer activity	24	5	0.022208	1
GO:0097001	MF	ceramide binding	20	4	0.022223	1
GO:0031050	BP	dsRNA processing	53	9	0.022459	1
GO:0070918	BP	production of small RNA involved in gene silencing by RNA	53	9	0.022459	1
GO:0010975	BP	regulation of neuron projection development	421	48	0.022599	1
GO:0006857	BP	oligopeptide transport	16	4	0.02261	1
GO:0035024	BP	negative regulation of Rho protein signal transduction	22	5	0.022914	1
GO:0003712	MF	transcription coregulator activity	451	46	0.023006	1
GO:0070997	BP	neuron death	347	36	0.024522	1
GO:0031338	BP	regulation of vesicle fusion	23	5	0.024681	1
GO:0034695	BP	response to prostaglandin E	22	5	0.025099	1
GO:0051020	MF	GTPase binding	273	30	0.025614	1
GO:0051959	MF	dynein light intermediate chain binding	27	6	0.026078	1
GO:0007264	BP	small GTPase mediated signal transduction	470	49	0.02626	1
GO:0060996	BP	dendritic spine development	88	14	0.026294	1
GO:1903203	BP	regulation of oxidative stress-induced neuron death	26	5	0.02691	1
GO:0007601	BP	visual perception	200	21	0.026925	1
GO:0047676	MF	arachidonate-CoA ligase activity	10	3	0.027004	1
GO:0051015	MF	actin filament binding	207	26	0.027088	1
GO:0051402	BP	neuron apoptotic process	234	26	0.027249	1
GO:0045123	BP	cellular extravasation	68	9	0.027257	1
GO:0007171	BP	activation of transmembrane receptor protein tyrosine kinase activity	14	4	0.027494	1
GO:0099092	CC	postsynaptic density, intracellular component	13	4	0.027837	1
GO:0006903	BP	vesicle targeting	44	7	0.027923	1
GO:0007229	BP	integrin-mediated signaling pathway	104	14	0.027995	1
GO:0051258	BP	protein polymerization	281	30	0.028137	1
GO:0015645	MF	fatty acid ligase activity	20	4	0.028205	1
GO:0031267	MF	small GTPase binding	242	27	0.028267	1
GO:0071391	BP	cellular response to estrogen stimulus	15	4	0.028785	1
GO:0062197	BP	cellular response to chemical stress	323	32	0.028831	1
GO:0042440	BP	pigment metabolic process	76	9	0.029565	1
GO:0007266	BP	Rho protein signal transduction	127	16	0.029586	1
GO:1903897	BP	regulation of PERK-mediated unfolded protein response	11	3	0.029651	1
GO:0046949	BP	fatty-acyl-CoA biosynthetic process	23	5	0.030091	1
GO:0016567	BP	protein ubiquitination	751	64	0.030249	1
GO:1905898	BP	positive regulation of response to endoplasmic reticulum stress	33	6	0.030568	1
GO:0070849	BP	response to epidermal growth factor	48	8	0.030642	1
GO:1902905	BP	positive regulation of supramolecular fiber organization	204	23	0.030848	1
GO:0030163	BP	protein catabolic process	891	75	0.031111	1
GO:1901570	BP	fatty acid derivative biosynthetic process	43	7	0.031207	1
GO:0099563	BP	modification of synaptic structure	21	5	0.031258	1
GO:0009260	BP	ribonucleotide biosynthetic process	168	18	0.031287	1

FDR 1% Gene GO Term	ONTOLOGY	TERM	N	DE	P.DE	FDR

FDR 5% Gene GO Term	ONTOLOGY	TERM	N	DE	P.DE	FDR
GO:0030217	BP	T cell differentiation	251	26	0.031573	1
GO:0030863	CC	cortical cytoskeleton	102	15	0.031811	1
GO:0051249	BP	regulation of lymphocyte activation	422	38	0.031814	1
GO:0032446	BP	protein modification by small protein conjugation	805	68	0.031988	1
GO:0046330	BP	positive regulation of JNK cascade	86	12	0.032036	1
GO:0046174	BP	polyol catabolic process	25	5	0.032076	1
GO:0045176	BP	apical protein localization	14	4	0.032094	1
GO:0099522	CC	cytosolic region	21	5	0.032225	1
GO:0050953	BP	sensory perception of light stimulus	204	21	0.032403	1
GO:0010976	BP	positive regulation of neuron projection development	154	19	0.032564	1
GO:0071347	BP	cellular response to interleukin-1	109	11	0.032626	1
GO:0030275	MF	LRR domain binding	17	4	0.032667	1
GO:1901216	BP	positive regulation of neuron death	93	13	0.032679	1
GO:0098631	MF	cell adhesion mediator activity	58	9	0.032728	1
GO:0046875	MF	ephrin receptor binding	27	6	0.032839	1
GO:0060997	BP	dendritic spine morphogenesis	55	10	0.032937	1
GO:0097484	BP	dendrite extension	35	7	0.033116	1
GO:0071380	BP	cellular response to prostaglandin E stimulus	14	4	0.033156	1
GO:0044272	BP	sulfur compound biosynthetic process	138	16	0.033195	1
GO:0014075	BP	response to amine	48	7	0.033216	1
GO:0010918	BP	positive regulation of mitochondrial membrane potential	11	3	0.033581	1
GO:0060295	BP	regulation of cilium movement involved in cell motility	24	4	0.03409	1
GO:1902019	BP	regulation of cilium-dependent cell motility	24	4	0.03409	1
GO:0019076	BP	viral release from host cell	32	6	0.034124	1
GO:0035890	BP	exit from host	32	6	0.034124	1
GO:0035891	BP	exit from host cell	32	6	0.034124	1
GO:1902882	BP	regulation of response to oxidative stress	91	11	0.034289	1
GO:0016878	MF	acid-thiol ligase activity	29	5	0.034857	1
GO:0097720	BP	calcineurin-mediated signaling	48	8	0.035262	1
GO:1902903	BP	regulation of supramolecular fiber organization	361	37	0.035302	1
GO:0005158	MF	insulin receptor binding	22	5	0.035335	1
GO:0003779	MF	actin binding	415	45	0.035917	1
GO:1904680	MF	peptide transmembrane transporter activity	15	4	0.036301	1
GO:0030175	CC	filopodium	101	14	0.036806	1
GO:0042176	BP	regulation of protein catabolic process	367	36	0.036889	1
GO:0042177	BP	negative regulation of protein catabolic process	111	14	0.037044	1
GO:0010998	BP	regulation of translational initiation by eIF2 alpha phosphorylation	10	3	0.037221	1
GO:0045505	MF	dynein intermediate chain binding	32	6	0.037336	1
GO:0097386	CC	glial cell projection	32	6	0.038029	1
GO:1900407	BP	regulation of cellular response to oxidative stress	82	10	0.038131	1
GO:0035148	BP	tube formation	146	19	0.038144	1
GO:0046390	BP	ribose phosphate biosynthetic process	173	18	0.038997	1
GO:0046597	BP	negative regulation of viral entry into host cell	19	4	0.039105	1
GO:0098794	CC	postsynapse	597	62	0.039295	1
GO:0019001	MF	guanyl nucleotide binding	364	32	0.039332	1
GO:0032561	MF	guanyl ribonucleotide binding	364	32	0.039332	1
GO:0030239	BP	myofibril assembly	63	9	0.039367	1
GO:0070570	BP	regulation of neuron projection regeneration	30	6	0.039437	1
GO:0035004	MF	phosphatidylinositol 3-kinase activity	12	3	0.039801	1
GO:0046148	BP	pigment biosynthetic process	54	7	0.039865	1
GO:0061158	BP	3'-UTR-mediated mRNA destabilization	17	4	0.040135	1
GO:0022884	MF	macromolecule transmembrane transporter activity	25	4	0.040483	1
GO:0070884	BP	regulation of calcineurin-NFAT signaling cascade	34	6	0.041251	1
GO:0099022	BP	vesicle tethering	10	3	0.041456	1
GO:0048245	BP	eosinophil chemotaxis	19	3	0.041677	1
GO:0043555	BP	regulation of translation in response to stress	18	4	0.04176	1
GO:0016331	BP	morphogenesis of embryonic epithelium	145	19	0.041857	1
GO:0036475	BP	neuron death in response to oxidative stress	30	5	0.042594	1
GO:0031252	CC	cell leading edge	406	44	0.042627	1
GO:0007026	BP	negative regulation of microtubule depolymerization	23	5	0.043114	1
GO:0099010	BP	modification of postsynaptic structure	16	4	0.04326	1
GO:0097061	BP	dendritic spine organization	78	12	0.043261	1
GO:0005912	CC	adherens junction	163	21	0.043413	1
GO:0000049	MF	tRNA binding	69	8	0.043449	1
GO:0035338	BP	long-chain fatty-acyl-CoA biosynthetic process	18	4	0.04352	1
GO:0001539	BP	cilium or flagellum-dependent cell motility	129	13	0.04365	1
GO:0060285	BP	cilium-dependent cell motility	129	13	0.04365	1
GO:0043129	BP	surfactant homeostasis	12	3	0.043864	1
GO:0072337	BP	modified amino acid transport	31	5	0.043889	1
GO:0050870	BP	positive regulation of T cell activation	206	21	0.044154	1
GO:0072175	BP	epithelial tube formation	131	17	0.044433	1

FDR 1% Gene GO Term	ONTOLOGY	TERM	N	DE	P.DE	FDR

FDR 5% Gene GO Term	ONTOLOGY	TERM	N	DE	P.DE	FDR
GO:0055002	BP	striated muscle cell development	64	9	0.044488	1
GO:0008287	CC	protein serine/threonine phosphatase complex	46	7	0.044524	1
GO:1903293	CC	phosphatase complex	46	7	0.044524	1
GO:0001838	BP	embryonic epithelial tube formation	120	16	0.044568	1
GO:0051403	BP	stress-activated MAPK cascade	228	24	0.044636	1
GO:0071545	BP	inositol phosphate catabolic process	18	4	0.044762	1
GO:1905207	BP	regulation of cardiocyte differentiation	34	6	0.045453	1
GO:0016773	MF	phosphotransferase activity, alcohol group as acceptor	635	62	0.045848	1
GO:0061512	BP	protein localization to cilium	63	8	0.046089	1
GO:0060968	BP	regulation of gene silencing	78	10	0.046238	1
GO:0048207	BP	vesicle targeting, rough ER to cis-Golgi	13	3	0.046392	1
GO:0048208	BP	COPII vesicle coating	13	3	0.046392	1
GO:0000164	CC	protein phosphatase type 1 complex	14	3	0.046813	1
GO:0061303	BP	cornea development in camera-type eye	11	3	0.046935	1
GO:0035673	MF	oligopeptide transmembrane transporter activity	11	3	0.046971	1
GO:0140467	BP	integrated stress response signaling	22	4	0.046994	1
GO:0043476	BP	pigment accumulation	11	3	0.047279	1
GO:0043482	BP	cellular pigment accumulation	11	3	0.047279	1
GO:0035627	BP	ceramide transport	15	3	0.047371	1
GO:0097449	CC	astrocyte projection	19	4	0.047626	1
GO:0019904	MF	protein domain specific binding	662	63	0.047649	1
GO:0003714	MF	transcription corepressor activity	172	20	0.04804	1
GO:1990752	CC	microtubule end	33	6	0.048228	1
GO:0042438	BP	melanin biosynthetic process	21	4	0.048311	1
GO:0060170	CC	ciliary membrane	72	9	0.048603	1
GO:0000902	BP	cell morphogenesis	941	94	0.048707	1
GO:1901214	BP	regulation of neuron death	308	31	0.049508	1
GO:0019207	MF	kinase regulator activity	221	23	0.049546	1
GO:0007274	BP	neuromuscular synaptic transmission	25	5	0.049684	1
GO:1990403	BP	embryonic brain development	11	3	0.049721	1
GO:0045321	BP	leukocyte activation	813	66	0.049746	1
GO:0043523	BP	regulation of neuron apoptotic process	203	22	0.049799	1

Table S7C. GOmeth analysis of Kyoto Encyclopedia of Genes and Genomes (KEGG) databases-enriched pathways for annotated genes in bronchopulmonary dysplasia (BPD) epigenome.

N=number of genes in GO term. DE=number of annotated genes in the deferrentially methyalted CpGs. P.DE=-value for over-representation of the GO term. FDR=false discovery rate.

FDR 1% Gene KEGG Terms	Description	N	DE	P.DE	FDR
path:hsa03020	RNA polymerase	32	2	0.039936	1

FDR 5% Gene KEGG Terms	Description	N	DE	P.DE	FDR
path:hsa04510	Focal adhesion	193	26	0.012119	1
path:hsa04015	Rap1 signaling pathway	207	26	0.020342	1
path:hsa04660	T cell receptor signaling pathway	99	13	0.027919	1
path:hsa04931	Insulin resistance	105	14	0.027946	1
path:hsa04668	TNF signaling pathway	108	13	0.034954	1
path:hsa05163	Human cytomegalovirus infection	214	24	0.036027	1
path:hsa04979	Cholesterol metabolism	49	7	0.037606	1
path:hsa04662	B cell receptor signaling pathway	78	10	0.040356	1
path:hsa04520	Adherens junction	70	11	0.040368	1
path:hsa04915	Estrogen signaling pathway	137	16	0.049065	1
path:hsa04062	Chemokine signaling pathway	188	19	0.049337	1

Table 8. cDNA microarray determined differentially expressed genes in cord blood cells of infants with bronchopulmonary dysplasia (BPD).

Illumina Human HT-12 v.40 R2 BeadChip analysis on cord blood cell RNAs from infants with BPD (n=6) and without BPD (n=16).

sig=significance. Bonferroni (BF) correction < 4.10E-06, false discovery rate (FDR) < 0.05.

hg19=Genome Reference Consortium Human Build 37 (GRCh37). Ex=Log2 expression. dEx=Log2 expression difference in BPD vs. nonBPD. dEx_FD=expression difference as fold difference in BPD vs. nonBPD.

Red font-increased DNA methylation in BPD vs nonBPD. Blue font-decreased DNA methyaltion in BPD vs nonBPD.

id	chr	p.start.hg19	p.end.hg19	p_adj	fdr	sig	Ex_BPD.cord	Ex_noBPD.cord	dEx.cord	dEx_FD	symbols	description
ILMN_2379560	9	99262893	99262944	1.13E-09	1.38E-05	BF	6.900563026	7.506975197	-0.61	0.66	CDC14B	cell division cycle 14B
ILMN_2219628	2	170667799	170668194	5.73E-09	3.49E-05	BF	7.506999783	8.04365574	-0.54	0.69	SSB	small RNA binding exonuclease protection factor La
ILMN_2046470	14	59836239	59836290	1.45E-08	5.90E-05	BF	8.760184282	9.33742916	-0.58	0.67	DAAM1	dishevelled associated activator of morphogenesis 1
ILMN_1813955	19	40950124	40950175	1.24E-07	0.00037738	BF	8.995678944	8.745662222	0.25	1.19	SERTAD3	SERTA domain containing 3
ILMN_1767658	8	67342553	67342604	2.92E-07	0.000532345	BF	6.14649506	6.924375533	-0.78	0.58	RRS1	ribosome biogenesis regulator 1 homolog
ILMN_1652160	4	151186499	151186550	3.06E-07	0.000532345	BF	7.454674995	8.057275109	-0.60	0.66	LRBA	LPS responsive beige-like anchor protein
ILMN_1723035	12	10311172	10311223	1.02E-06	0.001556224	BF	8.806131756	7.130458501	1.68	3.19	OLR1	oxidized low density lipoprotein receptor 1
ILMN_1775224	7	150711492	150711543	1.16E-06	0.001571414	BF	6.680579327	6.718645719	-0.04	0.97	NOS3	nitric oxide synthase 3
ILMN_1712291		1474216	1474267	1.45E-06	0.001703186	BF	7.956789236	7.849913886	0.11	1.08	MICALL2	MICAL like 2
ILMN_1784822	X	49143815	49143866	1.54E-06	0.001703186	BF	6.771189005	7.485149089	-0.71	0.61	PPP1R3F	protein phosphatase 1 regulatory subunit 3F
ILMN_1795715	1	97543903	97543954	2.49E-06	0.00252711	BF	7.082691807	8.269004748	-1.19	0.44	DPYD	dihydropyrimidine dehydrogenase
ILMN_1659659	X	55479487	55479538	2.76E-06	0.002584054	BF	6.522884405	7.824869097	-1.30	0.41	MAGEH1	MAGE family member H1
ILMN_1684054	8	17941569	17941620	3.29E-06	0.002805612	BF	9.776503885	10.10369449	-0.33	0.80	ASAH1	N-acylsphingosine amidohydrolase 1
ILMN_1757338	3	145910728	145910779	3.45E-06	0.002805612	BF	6.70997976	7.897394579	-1.19	0.44	PLSCR4	phospholipid scramblase 4
ILMN_2267488	2	173366553	173366604	4.06E-06	0.003093228	BF	6.239718665	6.93366213	-0.69	0.62	ITGA6	integrin subunit alpha 6
ILMN_1698770	5	36193896	36193947	4.78E-06	0.003425861	FDR0.01	6.844167279	8.236697372	-1.39	0.38	NADK2	NAD kinase 2, mitochondrial
ILMN_1796589	5	917890	917941	5.27E-06	0.003564983	FDR0.01	7.661329029	7.94577021	-0.28	0.82	TRIP13	thyroid hormone receptor interactor 13
ILMN_1685156	12	49160214	49160265	6.56E-06	0.00420515	FDR0.01	7.352256749	6.649583153	0.70	1.63	ADCY6	adenylate cyclase 6
ILMN_1774930	1	100985005	100985056	7.42E-06	0.00421177	FDR0.01	6.528380761	7.586871064	-1.06	0.48	CDC14A	cell division cycle 14A
ILMN_1742808	11	64544032	64544083	7.58E-06	0.00421177	FDR0.01	7.642987696	8.501947928	-0.86	0.55	SF1	splicing factor 1
ILMN_2136177	5	180004752	180004803	7.63E-06	0.00421177	FDR0.01	10.71056662	10.88749552	-0.18	0.88	CNOT6	CCR4-NOT transcription complex subunit 6
ILMN_1723124	15	49621667	49621718	7.95E-06	0.00421177	FDR0.01	7.031244198	7.599068746	-0.57	0.67	GALK2	galactokinase 2
ILMN_2062112	2	187373562	187373613	9.52E-06	0.004833247	FDR0.01	8.713498849	9.713543801	-1.00	0.50	ZC3H15	zinc finger CCCH-type containing 15
ILMN_1777322	8	124824884	124824935	1.06E-05	0.005067178	FDR0.01	8.266580549	8.647743755	-0.38	0.77	FAM91A1	family with sequence similarity 91 member A1
ILMN_1752953	19	50176881	50176999	1.08E-05	0.005067178	FDR0.01	9.925076401	9.701298456	0.22	1.17	BCL2L12	BCL2 like 12
ILMN_2322935	12	112330822	112330873	1.28E-05	0.005693758	FDR0.01	7.509270152	8.642977438	-1.13	0.46	MAPKAPK5	MAPK activated protein kinase 5
ILMN_1804735	21	44473356	44473407	1.31E-05	0.005693758	FDR0.01	8.230752253	7.29991514	0.93	1.91	CBS, CBSL	cystathionine beta-synthase|cystathionine beta-synthase like
ILMN_1776552	1	78413920	78413971	1.38E-05	0.005794526	FDR0.01	8.038946198	8.848968768	-0.81	0.57	FUBP1	far upstream element binding protein 1
ILMN_1782954	4	39781063	39781114	1.44E-05	0.005864003	FDR0.01	9.309771276	9.875380093	-0.57	0.68	UBE2K	ubiquitin conjugating enzyme E2 K
ILMN_1688413	14	78184282	78184333	1.54E-05	0.006065846	FDR0.01	6.920776448	7.872137115	-0.95	0.52	SNW1	SNW domain containing 1
ILMN_2092390	X	18181275	18181326	1.65E-05	0.006122639	FDR0.01	6.6824115	7.287112853	-0.60	0.66	BEND2	BEN domain containing 2
ILMN_2346856	1	235507224	235507275	1.66E-05	0.006122639	FDR0.01	9.455635246	10.14402291	-0.69	0.62	GGPS1	geranylgeranyl diphosphate synthase 1
ILMN_1685930	11	31453768	31453819	1.81E-05	0.006322302	FDR0.01	6.556841791	7.130841542	-0.57	0.67	DNAJC24	DnaJ heat shock protein family (Hsp40) member C24
ILMN_1796349	6	123130297	123130348	1.82E-05	0.006322302	FDR0.01	7.06614387	7.442252009	-0.38	0.77	SMPDL3A	sphingomyelin phosphodiesterase acid like 3A
ILMN_1787081	17	35836989	35837040	1.90E-05	0.00643523	FDR0.01	6.764527539	7.199874198	-0.44	0.74	TADA2A	transcriptional adaptor 2A
ILMN_1696311	8	57875592	57875643	1.98E-05	0.006505325	FDR0.01	6.645969379	7.283804402	-0.64	0.64	BPNT2	3'(2'), 5'-bisphosphate nucleotidase 2
ILMN_2233539	4	103183175	103183226	2.06E-05	0.006591968	FDR0.01	8.880297187	9.351226141	-0.47	0.72	SLC39A8	solute carrier family 39 member 8
ILMN_1721729	17	37563023	37563074	2.25E-05	0.00701951	FDR0.01	8.139472691	8.970726246	-0.83	0.56	MED1	mediator complex subunit 1
ILMN_1667791	1	203047700	203047751	2.34E-05	0.007129894	FDR0.01	9.561093343	8.065737492	1.50	2.82	PPFIA4	PTPRF interacting protein alpha 4
ILMN_1780667	3	52109293	52109344	2.58E-05	0.007501785	FDR0.01	8.538917894	7.933176752	0.61	1.52	POC1A	POC1 centriolar protein A
ILMN_1768097	X	38128918	38128969	2.59E-05	0.007501785	FDR0.01	6.822200384	8.255655021	-1.43	0.37	RPGR	retinitis pigmentosa GTPase regulator
ILMN_2128087	14	57877075	57877126	2.75E-05	0.00778798	FDR0.01	5.954102747	7.725554246	-1.77	0.29	NAA30	N-alpha-acetyltransferase 30, NatC catalytic subunit
ILMN_2352293	11	129769983	129770034	2.92E-05	0.008081065	FDR0.01	7.056819585	7.643047394	-0.59	0.67	PRDM10	PR/SET domain 10
ILMN_2257015	6	32150024	32150075	3.02E-05	0.008174435	FDR0.01	7.794011585	7.286169706	0.51	1.42	AGER	advanced glycosylation end-product specific receptor
ILMN_1713935	3	155480495	155480546	3.14E-05	0.008320908	FDR0.01	8.708445803	8.377992335	0.33	1.26	C3orf33	chromosome 3 open reading frame 33
ILMN_1770053	X	16863143	16863194	3.24E-05	0.008405637	FDR0.01	7.543865513	8.005019605	-0.46	0.73	RBBP7	RB binding protein 7, chromatin remodeling factor
ILMN_1749583	7	149128513	149128564	3.31E-05	0.008408348	FDR0.01	6.201960131	7.22061331	-1.02	0.49	ZNF777	zinc finger protein 777
ILMN_1728074	5	125960451	125960502	3.62E-05	0.0090131	FDR0.01	8.700137752	9.100857548	-0.40	0.76	PHAX	phosphorylated adaptor for RNA export
ILMN_2225348	19	57773196	57773247	3.77E-05	0.009196933	FDR0.01	8.045587403	9.407392178	-1.36	0.39	ZNF805	zinc finger protein 805
ILMN_1688565	19	56154559	56154610	3.90E-05	0.009320919	FDR0.01	9.625030421	9.826010827	-0.20	0.87	ZNF580	zinc finger protein 580
ILMN_1690780	9	79000987	79001038	4.09E-05	0.009427484	FDR0.01	6.275816509	7.365885833	-1.09	0.47	RFK	riboflavin kinase
ILMN_1740213	5	60048066	60048117	4.10E-05	0.009427484	FDR0.01	6.719413292	7.591621256	-0.87	0.55	ELOVL7	ELOVL fatty acid elongase 7
ILMN_1725193	2	217528872	217528923	4.25E-05	0.009536463	FDR0.01	6.753666542	7.365825916	-0.61	0.65	IGFBP2	insulin like growth factor binding protein 2
ILMN_2407824	1	169101469	169101520	4.35E-05	0.009536463	FDR0.01	8.221564917	9.272656605	-1.05	0.48	ATP1B1	ATPase Na+/K+ transporting subunit beta 1
ILMN_2362809	14	96137698	96137749	4.38E-05	0.009536463	FDR0.01	9.237385725	7.894875699	1.34	2.54	TCL6	T cell leukemia/lymphoma 6
ILMN_2379675	8	66636502	66636553	4.62E-05	0.009801833	FDR0.01	6.726081092	7.644420782	-0.92	0.53	PDE7A	phosphodiesterase 7A
ILMN_1734596	14	92246782	92246833	4.66E-05	0.009801833	FDR0.01	6.538089215	7.531163384	-0.99	0.50	TC2N	tandem C2 domains, nuclear
ILMN_1800976	16	68225515	68225566	4.85E-05	0.009871617	FDR0.01	7.215816042	7.784072204	-0.57	0.67	NFATC3	nuclear factor of activated T cells 3
ILMN_3239735	1	15884	15935	4.91E-05	0.009871617	FDR0.01	8.443073333	8.275115583	0.17	1.12	WASH7P	WASP family homolog 7, pseudogene
ILMN_1784847	11	85373063	85373114	4.97E-05	0.009871617	FDR0.01	7.000960595	7.942463096	-0.94	0.52	CREBZF	CREB/ATF bZIP transcription factor

id	chr	p.start.hg19	p.end.hg19	p_adj	fdr	sig	Ex_BPD.cord	Ex_noBPD.cord	dEx.cord	dEx_FD	symbols	description
ILMN_1720053	6	38121912	38121963	5.02E-05	0.009871617	FDR0.01	6.338043247	6.761641768	-0.42	0.75	***ZFAND3***	zinc finger AN1-type containing 3
ILMN_1777250		133780990	133781041	5.19E-05	0.010035695	FDR0.05	6.485023761	7.576451068	-1.09	0.47	***ZNF268***	zinc finger protein 268
ILMN_1777519	12	53585348	53585399	5.41E-05	0.010302783	FDR0.05	7.31723372	8.064706176	-0.75	0.60	***ITGB7***	integrin subunit beta 7|zinc finger protein 740
ILMN_1777519	12	53585348	53585399	5.41E-05	0.010302783	FDR0.05	7.31723372	8.064706176	-0.75	0.60	***ZNF740***	integrin subunit beta 7|zinc finger protein 740
ILMN_2137066	8	146068304	146068355	5.81E-05	0.010742531	FDR0.05	7.048091806	8.377385265	-1.33	0.40	***ZNF7***	zinc finger protein 7
ILMN_1747577	9	116148884	116148935	5.82E-05	0.010742531	FDR0.05	7.166276409	8.101895968	-0.94	0.52	***ALAD***	aminolevulinate dehydratase
ILMN_1674297	12	104496991	104497042	6.05E-05	0.011002587	FDR0.05	6.611343842	7.890272285	-1.28	0.41	***HCFC2***	host cell factor C2
ILMN_3236404	17	33900697	33900748	6.18E-05	0.011071248	FDR0.05	8.3395541	8.694301081	-0.35	0.78	***SNORD7***	small nucleolar RNA, C/D box 7
ILMN_1713458	16	204443	204494	6.39E-05	0.011212996	FDR0.05	12.48166149	12.93393975	-0.45	0.73	***HBZ***	hemoglobin subunit zeta
ILMN_1763408	1	89446739	89446790	6.44E-05	0.011212996	FDR0.05	6.232917609	6.906804199	-0.67	0.63	***RBMXL1***	RBMX like 1
ILMN_1691425	22	44888812	44888863	6.68E-05	0.011358691	FDR0.05	6.333033664	7.321938042	-0.99	0.50	***RTL6***	retrotransposon Gag like 6
ILMN_1697906	13	41657748	41657799	6.71E-05	0.011358691	FDR0.05	8.044477462	9.304913933	-1.26	0.42	***WBP4***	WW domain binding protein 4
ILMN_2115553	12	72073973	72074024	6.85E-05	0.011435768	FDR0.05	6.033243932	6.757004694	-0.72	0.61	***THAP2***	THAP domain containing 2
ILMN_2186715	5	68506061	68506112	7.25E-05	0.011935267	FDR0.05	7.959568325	7.393765543	0.57	1.48	***CENPH***	centromere protein H
ILMN_1806710	1	6520907	6520958	7.73E-05	0.012510983	FDR0.05	13.2582078	13.20916308	0.05	1.03	***ESPN***	espin|TNF receptor superfamily member 25
ILMN_1806710	1	6520907	6520958	7.73E-05	0.012510983	FDR0.05	13.2582078	13.20916308	0.05	1.03	***TNFRSF25***	espin|TNF receptor superfamily member 25
ILMN_1770206	17	648004	648055	7.80E-05	0.012510983	FDR0.05	6.458148799	7.135846584	-0.68	0.63	***GEMIN4***	gem nuclear organelle associated protein 4
ILMN_2234229	1	107601432	107601483	8.23E-05	0.012885352	FDR0.05	7.68800063	8.53001515	-0.84	0.56	***PRMT6***	protein arginine methyltransferase 6
ILMN_3245471	17	58308930	58308981	8.25E-05	0.012885352	FDR0.05	11.44544788	11.32760955	0.12	1.09	***SCARNA20***	small Cajal body-specific RNA 20
ILMN_1748116	X	14026480	14026531	8.63E-05	0.013220025	FDR0.05	6.999721645	8.193888778	-1.19	0.44	***GEMIN8***	gem nuclear organelle associated protein 8
ILMN_1814657	16	4307415	4307466	8.91E-05	0.013220025	FDR0.05	5.842453157	6.552706319	-0.71	0.61	***TFAP4***	transcription factor AP-4
ILMN_2153466	6	3851237	3851288	8.94E-05	0.013220025	FDR0.05	7.481839502	8.395607213	-0.91	0.53	***FAM50B***	family with sequence similarity 50 member B
ILMN_1657837	2	112973775	112973826	9.00E-05	0.013220025	FDR0.05	6.590870012	7.482843918	-0.89	0.54	***ZC3H8***	zinc finger CCCH-type containing 8
ILMN_1752665	16	67237450	67237582	9.00E-05	0.013220025	FDR0.05	9.142392027	8.918080794	0.22	1.17	***ELMO3***	engulfment and cell motility 3
ILMN_2103480	19	53379770	53379821	9.13E-05	0.01324669	FDR0.05	6.3668952	7.010396754	-0.64	0.64	***ZNF320***	zinc finger protein 320
ILMN_1722726	11	128838353	128838404	9.33E-05	0.013323631	FDR0.05	6.871672097	7.514531222	-0.64	0.64	***ARHGAP32***	Rho GTPase activating protein 32
ILMN_2223720	16	81080741	81080792	9.40E-05	0.013323631	FDR0.05	5.821543118	7.089233162	-1.27	0.42	***ATMIN***	ATM interactor
ILMN_2261379	1	206637122	206637173	0.00010045	0.01397996	FDR0.05	8.576954878	9.785425937	-1.21	0.43	***SRGAP2***	SLIT-ROBO Rho GTPase activating protein 2
ILMN_1773307	4	89617334	89617385	0.00010173	0.01397996	FDR0.05	8.67719467	10.10504219	-1.43	0.37	***NAP1L5***	nucleosome assembly protein 1 like 5
ILMN_1675674	1	10240977	10241028	0.0001032	0.01397996	FDR0.05	9.241123753	9.550863531	-0.31	0.81	***UBE4B***	ubiquitination factor E4B
ILMN_3238369	7	139784869	139784920	0.00010347	0.01397996	FDR0.05	9.732207022	10.39542975	-0.66	0.63	***KDM7A***	lysine demethylase 7A
ILMN_1774585	11	104839275	104839326	0.00010439	0.01397996	FDR0.05	6.411283154	6.539573345	-0.13	0.91	***CASP4***	caspase 4
ILMN_2373779	2	238006857	238006908	0.00010703	0.014177672	FDR0.05	7.955691909	8.889644108	-0.93	0.52	***COPS8***	COP9 signalosome subunit 8
ILMN_1783142	Y	2733126	2733175	0.00010939	0.014295284	FDR0.05	11.0874152	10.21520309	0.87	1.83	***RPS4Y1***	ribosomal protein S4 Y-linked 1
ILMN_1712522	19	42275747	42275798	0.00011026	0.014295284	FDR0.05	9.995425836	8.604317697	1.39	2.62	***CEACAM6***	CEA cell adhesion molecule 6
ILMN_1695509	7	77268558	77268609	0.00011199	0.014366508	FDR0.05	6.957948274	8.065813674	-1.11	0.46	***PTPN12***	protein tyrosine phosphatase non-receptor type 12
ILMN_1692058	15	23930940	23930991	0.00011493	0.014547216	FDR0.05	7.428595083	7.902309989	-0.47	0.72	***NDN***	necdin, MAGE family member
ILMN_2322458	3	193414617	193414668	0.00011659	0.014547216	FDR0.05	7.590723228	8.947426962	-1.36	0.39	***OPA1***	OPA1 mitochondrial dynamin like GTPase
ILMN_2373177	20	3899367	3899418	0.00011698	0.014547216	FDR0.05	6.332722393	6.761827154	-0.43	0.74	***PANK2***	pantothenate kinase 2
ILMN_1659777	5	179036625	179036676	0.00011857	0.014595855	FDR0.05	7.586265588	7.801995825	-0.22	0.86	***RUFY1***	RUN and FYVE domain containing 1
ILMN_2180582	7	108112180	108112231	0.00012582	0.015281464	FDR0.05	6.740212638	7.845899904	-1.11	0.46	***PNPLA8***	patatin like phospholipase domain containing 8
ILMN_2325347	3	160801781	160801832	0.00012808	0.015281464	FDR0.05	7.765479152	7.469965566	0.30	1.23	***B3GALNT1***	beta-1,3-N-acetylgalactosaminyltransferase 1 (globoside blood group)
ILMN_2143671	19	47978552	47978603	0.00013038	0.015281464	FDR0.05	6.762775008	7.825888826	-1.06	0.48	***KPTN***	kaptin, actin binding protein
ILMN_1718071	9	88162008	88162059	0.00013041	0.015281464	FDR0.05	9.51193363	9.990859032	-0.48	0.72	***AGTPBP1***	ATP/GTP binding carboxypeptidase 1
ILMN_1692834		185260525	185260576	0.00014381	0.016600161	FDR0.05	7.808094436	8.29814606	-0.49	0.71	***SWT1***	SWT1 RNA endoribonuclease homolog
ILMN_1789909	5	179289333	179289384	0.00014513	0.016600161	FDR0.05	7.978677811	8.225685868	-0.25	0.84	***TBC1D9B***	TBC1 domain family member 9B
ILMN_1715814	18	56997394	56997445	0.00014575	0.016600161	FDR0.05	7.279738279	8.055186461	-0.78	0.58	***LMAN1***	lectin, mannose binding 1
ILMN_2116877	13	53626106	53626157	0.0001533	0.017181502	FDR0.05	10.81217611	10.02005122	0.79	1.73	***OLFM4***	olfactomedin 4
ILMN_2378048	9	86583683	86583734	0.00015435	0.017181502	FDR0.05	11.12624916	11.96318817	-0.84	0.56	***HNRNPK***	heterogeneous nuclear ribonucleoprotein K
ILMN_1686235	14	53242065	53242116	0.00015627	0.017181502	FDR0.05	6.442835328	7.223005309	-0.78	0.58	***GNPNAT1***	glucosamine-phosphate N-acetyltransferase 1
ILMN_1671237	17	47283892	47283943	0.00015649	0.017181502	FDR0.05	8.726741811	8.977211087	-0.25	0.84	***GNGT2***	G protein subunit gamma transducin 2
ILMN_1788211	11	130746254	130746305	0.0001652	0.01775231	FDR0.05	7.896451904	8.404531085	-0.51	0.70	***SNX19***	sorting nexin 19
ILMN_1811121	X	103435756	103435807	0.00016595	0.01775231	FDR0.05	6.978149775	8.018721483	-1.04	0.49	***FAM199X***	family with sequence similarity 199, X-linked
ILMN_1810826	9	88633244	88633295	0.00016606	0.01775231	FDR0.05	6.504191212	7.083797542	-0.58	0.67	***NAA35***	N-alpha-acetyltransferase 35, NatC auxiliary subunit
ILMN_1775542	1	207077927	207077978	0.0001702	0.018036831	FDR0.05	10.1283448	10.5251904	-0.40	0.76	***FCMR***	Fc fragment of IgM receptor
ILMN_2372922	9	131910963	131911014	0.00017363	0.018196733	FDR0.05	7.494094901	7.617222137	-0.12	0.92	***PTPA***	protein phosphatase 2 phosphatase activator
ILMN_1652992	1	14108829	14108880	0.0001747	0.018196733	FDR0.05	7.93480023	8.121267588	-0.19	0.88	***PRDM2***	PR/SET domain 2
ILMN_1784540	7	32907874	32907925	0.0001778	0.018362814	FDR0.05	11.64699298	11.46904165	0.18	1.13	***KBTBD2***	kelch repeat and BTB domain containing 2
ILMN_1709882	6	52866669	52866720	0.00018301	0.01874253	FDR0.05	7.999240246	8.78902049	-0.79	0.58	***CILK1***	ciliogenesis associated kinase 1
ILMN_1815308	2	20400667	20400718	0.00019052	0.019348443	FDR0.05	6.62024071	6.436631397	0.18	1.14	***SDC1***	syndecan 1
ILMN_1672405	5	114950104	114950155	0.00019703	0.019400774	FDR0.05	7.58060923	8.478341673	-0.90	0.54	***TMED7***	transmembrane p24 trafficking protein 7
ILMN_2173611	16	56660846	56660897	0.00019899	0.019400774	FDR0.05	8.774533452	8.422372074	0.35	1.28	***MT1E***	metallothionein 1E
ILMN_2070896	2	203432026	203432077	0.00019932	0.019400774	FDR0.05	6.99384845	7.949448528	-0.96	0.52	***BMPR2***	bone morphogenetic protein receptor type 2
ILMN_2323366	1	161334452	161334503	0.00020042	0.019400774	FDR0.05	6.467853677	7.197406421	-0.73	0.60	***SDHC***	succinate dehydrogenase complex subunit C
ILMN_1754538	10	82191853	82191904	0.00020056	0.019400774	FDR0.05	6.996861976	7.504701752	-0.51	0.70	***PRXL2A***	peroxiredoxin like 2A

id	chr	p.start.hg19	p.end.hg19	p_adj	fdr	sig	Ex_BPD.cord	Ex_noBPD.cord	dEx.cord	dEx_FD	symbols	description
ILMN_1668012	7	95749800	95749851	0.00020058	0.019400774	FDR0.05	7.740708111	8.090706603	-0.35	0.78	***SLC25A13***	solute carrier family 25 member 13
ILMN_1758719	6	11184105	11184156	0.00020622	0.019689087	FDR0.05	8.869796163	9.225701787	-0.36	0.78	***NEDD9***	neural precursor cell expressed, developmentally down-regulated 9
ILMN_3241099		92954202	92954253	0.00020685	0.019689087	FDR0.05	6.615230533	7.142976101	-0.53	0.69	***FAM172A***	family with sequence similarity 172 member A
ILMN_2195665	5	167983111	167983162	0.00020841	0.019689087	FDR0.05	6.655805374	7.532832523	-0.88	0.54	***PANK3***	pantothenate kinase 3
ILMN_2394571	5	171289010	171289061	0.00021079	0.019760815	FDR0.05	6.78943623	7.671564625	-0.88	0.54	***FBXW11***	F-box and WD repeat domain containing 11
ILMN_1701195	6	46675851	46675902	0.00021337	0.019850132	FDR0.05	7.164388863	6.520740048	0.64	1.56	***PLA2G7***	phospholipase A2 group VII
ILMN_1738909	1	193054508	193054559	0.0002161	0.019892593	FDR0.05	8.897058311	9.594893149	-0.70	0.62	***RO60***	Ro60, Y RNA binding protein
ILMN_3251620	5	78622484	78622535	0.00021709	0.019892593	FDR0.05	6.269237817	7.703457496	-1.43	0.37	***JMY***	junction mediating and regulatory protein, p53 cofactor
ILMN_1752409	16	24834916	24834967	0.00022152	0.020146521	FDR0.05	8.883077932	8.725376262	0.16	1.12	***TNRC6A***	trinucleotide repeat containing adaptor 6A
ILMN_1654737	9	119462581	119462632	0.00022644	0.020441463	FDR0.05	6.48368077	7.721209726	-1.24	0.42	***TRIM32***	tripartite motif containing 32
ILMN_2219767	2	16086638	16086689	0.00023627	0.02108847	FDR0.05	6.4537146	7.182165064	-0.73	0.60	***MYCN***	MYCN proto-oncogene, bHLH transcription factor
ILMN_1659857	22	21243891	21243942	0.00023707	0.02108847	FDR0.05	9.460383358	9.874589433	-0.41	0.75	***SNAP29***	synaptosome associated protein 29
ILMN_2402629	16	21668039	21668090	0.00023933	0.021136047	FDR0.05	7.897525898	8.585500252	-0.69	0.62	***METTL9***	methyltransferase like 9
ILMN_2285568	4	76836089	76836139	0.0002425	0.021261144	FDR0.05	7.909516263	8.049804295	-0.14	0.91	***NAAA***	N-acylethanolamine acid amidase
ILMN_2311041	14	23304076	23304127	0.00024511	0.021336966	FDR0.05	9.61205273	9.864481896	-0.25	0.84	***MRPL52***	mitochondrial ribosomal protein L52
ILMN_1677843	5	176728551	176728602	0.00025448	0.021995415	FDR0.05	9.352534441	9.136359515	0.22	1.16	***RAB24***	RAB24, member RAS oncogene family
ILMN_1717163	6	6144881	6144932	0.00025928	0.022252194	FDR0.05	10.07846833	10.83393181	-0.76	0.59	***F13A1***	coagulation factor XIII A chain
ILMN_2343563	17	79858135	79858186	0.00026456	0.022475916	FDR0.05	9.891391337	9.299853528	0.59	1.51	***ANAPC11***	anaphase promoting complex subunit 11
ILMN_2368145	15	57998906	57998957	0.00026654	0.022475916	FDR0.05	8.331599517	8.104151726	0.23	1.17	***POLR2M***	RNA polymerase II subunit M
ILMN_1778660	X	41556095	41556147	0.00026742	0.022475916	FDR0.05	7.848390631	8.507669246	-0.66	0.63	***GPR34***	G protein-coupled receptor 34
ILMN_2401822	X	48344375	48344426	0.00027444	0.022789662	FDR0.05	7.668252953	8.035950103	-0.37	0.78	***FTSJ1***	FtsJ RNA 2'-O-methyltransferase 1
ILMN_1678690	22	24922400	24922451	0.00027489	0.022789662	FDR0.05	8.753620883	7.657564385	1.10	2.14	***UPB1***	beta-ureidopropionase 1
ILMN_2093674	14	54941282	54941333	0.00028222	0.023239525	FDR0.05	10.97435569	10.62511906	0.35	1.27	***GMFB***	glia maturation factor beta
ILMN_2204297	14	57756586	57756637	0.00028875	0.023617807	FDR0.05	6.22607825	7.271822857	-1.05	0.48	***AP5M1***	adaptor related protein complex 5 subunit mu 1
ILMN_1731699	14	65412827	65412878	0.00029355	0.023850257	FDR0.05	6.163195579	6.733008771	-0.57	0.67	***RAB15***	RAB15, member RAS oncogene family
ILMN_1806448	9	135117682	135117733	0.00029643	0.023924802	FDR0.05	7.709686529	6.26819825	1.44	2.72	***NTNG2***	netrin G2
ILMN_2360705	2	223806568	223806619	0.00030161	0.024178009	FDR0.05	8.696066089	9.414980895	-0.72	0.61	***ACSL3***	acyl-CoA synthetase long chain family member 3
ILMN_1698739	17	33515786	33515837	0.00030486	0.024178009	FDR0.05	7.334265492	7.726425979	-0.39	0.76	***UNC45B***	unc-45 myosin chaperone B
ILMN_1812616	17	1368546	1368597	0.00030552	0.024178009	FDR0.05	7.488278749	7.700364966	-0.21	0.86	***MYO1C***	myosin IC
ILMN_2049417	19	55738161	55738212	0.0003108	0.024437222	FDR0.05	11.60471281	11.83286768	-0.23	0.85	***TMEM86B***	transmembrane protein 86B
ILMN_1796305	10	11962527	11962578	0.00031404	0.024516091	FDR0.05	9.755349755	10.44725718	-0.69	0.62	***UPF2***	UPF2 regulator of nonsense mediated mRNA decay
ILMN_1756999	16	53524766	53524817	0.00031583	0.024516091	FDR0.05	9.844887357	10.21753135	-0.37	0.77	***RBL2***	RB transcriptional corepressor like 2
ILMN_1755658	17	47300371	47300422	0.00032451	0.024875325	FDR0.05	6.732436967	7.098539304	-0.37	0.78	***ABI3***	ABI family member 3
ILMN_2058512	7	42956645	42956696	0.00032454	0.024875325	FDR0.05	6.445115375	6.971782591	-0.53	0.69	***PSMA2***	proteasome 20S subunit alpha 2
ILMN_1687751	8	104242166	104242217	0.00032949	0.025096746	FDR0.05	7.217164132	6.366795775	0.85	1.80	***BAALC***	BAALC binder of MAP3K1 and KLF4
ILMN_1756417	4	186320132	186320183	0.00033987	0.02572705	FDR0.05	9.380121392	9.433218528	-0.05	0.96	***ANKRD37***	ankyrin repeat domain 37
ILMN_1712530	17	55198108	55198159	0.00034548	0.025989567	FDR0.05	8.168609913	8.902474766	-0.73	0.60	***AKAP1***	A-kinase anchoring protein 1
ILMN_1783226	1	155981602	155981653	0.00036081	0.026976468	FDR0.05	11.99752148	11.85265258	0.14	1.11	***SSR2***	signal sequence receptor subunit 2
ILMN_1695717	X	106312559	106312610	0.00036541	0.027027518	FDR0.05	9.487269193	10.01276563	-0.53	0.69	***RBM41***	RNA binding motif protein 41
ILMN_2196097	5	133532534	133532585	0.00036593	0.027027518	FDR0.05	8.302652774	9.036532722	-0.73	0.60	***PPP2CA***	protein phosphatase 2 catalytic subunit alpha
ILMN_2097858	14	77583222	77583273	0.00036844	0.02704917	FDR0.05	9.155199662	9.513500391	-0.36	0.78	***CIPC***	CLOCK interacting pacemaker
ILMN_1755504	17	46941873	46941924	0.00037233	0.027170869	FDR0.05	9.809814168	10.41067952	-0.60	0.66	***CALCOCO2***	calcium binding and coiled-coil domain 2
ILMN_1733523	13	41932444	41932495	0.00037863	0.02722685	FDR0.05	6.952851279	7.787007685	-0.83	0.56	***NAA16***	N-alpha-acetyltransferase 16, NatA auxiliary subunit
ILMN_1727938	16	30565385	30565436	0.00037952	0.02722685	FDR0.05	8.216169119	7.935573137	0.28	1.21	***ZNF764***	zinc finger protein 764
ILMN_1705032	18	12986377	12986428	0.0003798	0.02722685	FDR0.05	5.891894404	6.693935585	-0.80	0.57	***SEH1L***	SEH1 like nucleoporin
ILMN_1701094	8	1732095	1732146	0.00039058	0.027836512	FDR0.05	7.841138653	8.214412949	-0.37	0.77	***CLN8***	CLN8 transmembrane ER and ERGIC protein
ILMN_1693668	10	88195879	88195930	0.00039394	0.027912218	FDR0.05	8.263287012	9.294991614	-1.03	0.49	***WAPL***	WAPL cohesin release factor
ILMN_2060115	11	121498308	121498359	0.00040328	0.028409121	FDR0.05	9.346608357	8.995803326	0.35	1.28	***SORL1***	sortilin related receptor 1
ILMN_1693597	17	16454781	16454832	0.00040806	0.028580725	FDR0.05	8.104304769	7.760419345	0.34	1.27	***ZNF287***	zinc finger protein 287
ILMN_2407124	20	5975017	5975068	0.00041744	0.029059494	FDR0.05	7.425150931	7.976838684	-0.55	0.68	***MCM8***	minichromosome maintenance 8 homologous recombination repair factor
ILMN_2038778	12	6647058	6647109	0.00042284	0.029113888	FDR0.05	13.36314694	13.08095111	0.28	1.22	***GAPDH***	glyceraldehyde-3-phosphate dehydrogenase
ILMN_2176450	8	86361197	86361248	0.00043501	0.029484496	FDR0.05	8.159541978	8.644481681	-0.48	0.71	***CA3***	carbonic anhydrase 3
ILMN_1768582	8	30643441	30643492	0.00043684	0.029484496	FDR0.05	7.011760841	7.756134735	-0.74	0.60	***PPP2CB***	protein phosphatase 2 catalytic subunit beta
ILMN_1769013	17	7076818	7076869	0.00043742	0.029484496	FDR0.05	6.967328596	7.082760317	-0.12	0.92	***ASGR1***	asialoglycoprotein receptor 1
ILMN_1673181	6	133137957	133138008	0.0004379	0.029484496	FDR0.05	12.11144942	11.97331641	0.14	1.10	***SNORD100***	small nucleolar RNA, C/D box 100
ILMN_1666482	17	46005878	46005929	0.00044313	0.029672601	FDR0.05	8.243947532	9.237412179	-0.99	0.50	***SP2***	Sp2 transcription factor
ILMN_1815527	11	5254174	5254225	0.00046076	0.030432815	FDR0.05	12.00217355	11.79813048	0.20	1.15	***HBD***	hemoglobin subunit delta
ILMN_1701991	21	34001099	34001150	0.00046152	0.030432815	FDR0.05	11.05123749	10.87505417	0.18	1.13	***SYNJ1***	synaptojanin 1
ILMN_3243204	11	22844152	22844203	0.00046197	0.030432815	FDR0.05	6.911546031	7.997740032	-1.09	0.47	***SVIP***	small VCP interacting protein
ILMN_1780306	14	53513197	53513248	0.00046907	0.030510274	FDR0.05	6.03929028	7.144034383	-1.10	0.46	***DDHD1***	DDHD domain containing 1
ILMN_2390472	3	180327746	180327797	0.00047162	0.030510274	FDR0.05	7.681825913	8.818678209	-1.14	0.45	***TTC14***	tetratricopeptide repeat domain 14
ILMN_1676173	22	44420260	44420311	0.00047374	0.030510274	FDR0.05	7.917714565	7.273942424	0.64	1.56	***PARVB***	parvin beta
ILMN_1679438	4	185615394	185615445	0.00047696	0.030510274	FDR0.05	7.362905562	7.919128108	-0.56	0.68	***CENPU***	centromere protein U
ILMN_1682996	11	123994124	123994380	0.00047918	0.030510274	FDR0.05	6.673979542	7.055774636	-0.38	0.77	***VWA5A***	von Willebrand factor A domain containing 5A
ILMN_1797453	3	138015663	138015714	0.00048206	0.030510274	FDR0.05	8.763040426	9.179514157	-0.42	0.75	***ARMC8***	armadillo repeat containing 8

id	chr	p.start.hg19	p.end.hg19	p_adj	fdr	sig	Ex_BPD.cord	Ex_noBPD.cord	dEx.cord	dEx_FD	symbols	description
ILMN_2294751	6	100956807	100956858	0.00048281	0.030510274	FDR0.05	6.952694737	7.080859378	-0.13	0.91	ASCC3	activating signal cointegrator 1 complex subunit 3
ILMN_1740169	15	41871314	41871365	0.00048318	0.030510274	FDR0.05	7.775589442	6.682558182	1.09	2.13	TYRO3	TYRO3 protein tyrosine kinase
ILMN_1705876	12	76441989	76442040	0.00049128	0.030862155	FDR0.05	7.529556546	7.8214465	-0.29	0.82	NAP1L1	nucleosome assembly protein 1 like 1
ILMN_2049021	8	67679820	67679871	0.00051351	0.032093024	FDR0.05	12.00288544	12.07256981	-0.07	0.95	PTTG3P	pituitary tumor-transforming 3, pseudogene
ILMN_2202790	2	47129265	47129316	0.0005244	0.032606586	FDR0.05	6.719567147	7.443380887	-0.72	0.61	MCFD2	multiple coagulation factor deficiency 2, ER cargo receptor complex subunit
ILMN_2149494	1	182798330	182798381	0.00053177	0.032896935	FDR0.05	11.84111139	12.24991502	-0.41	0.75	NPL	N-acetylneuraminate pyruvate lyase
ILMN_1678579	1	53679619	53679670	0.0005483	0.033748103	FDR0.05	7.110132646	7.81860202	-0.71	0.61	CPT2	carnitine palmitoyltransferase 2
ILMN_2213297	11	93495817	93495868	0.00055363	0.033905266	FDR0.05	7.942420091	9.227171411	-1.28	0.41	C11orf54	chromosome 11 open reading frame 54
ILMN_1665943	19	39078379	39078430	0.00055944	0.034089302	FDR0.05	10.03146177	10.18676036	-0.16	0.90	MAP4K1	mitogen-activated protein kinase kinase kinase kinase 1
ILMN_2301955	8	42692168	42692219	0.00057414	0.034811028	FDR0.05	6.575337793	7.752731721	-1.18	0.44	THAP1	THAP domain containing 1
ILMN_2410262	3	9743783	9743834	0.00057999	0.034920529	FDR0.05	10.44950313	10.34646859	0.10	1.07	MTMR14	myotubularin related protein 14
ILMN_1790197	12	118503793	118503844	0.00058349	0.034920529	FDR0.05	8.683544876	7.519971352	1.16	2.24	VSIG10	V-set and immunoglobulin domain containing 10
ILMN_1700660	17	29326658	29326709	0.00058454	0.034920529	FDR0.05	7.093554396	7.000920986	0.09	1.07	RNF135	ring finger protein 135
ILMN_1653337	17	58255878	58255929	0.00059267	0.035233691	FDR0.05	9.712689435	10.15637018	-0.44	0.74	CA4	carbonic anhydrase 4|ubiquitin specific peptidase 32
ILMN_1653337	17	58255878	58255929	0.00059267	0.035233691	FDR0.05	9.712689435	10.15637018	-0.44	0.74	USP32	carbonic anhydrase 4|ubiquitin specific peptidase 32
ILMN_2120022	10	18966786	18966837	0.00060802	0.035721856	FDR0.05	7.858578733	8.36651028	-0.51	0.70	ARL5B	ADP ribosylation factor like GTPase 5B
ILMN_1691927	15	83689447	83689498	0.00061624	0.035721856	FDR0.05	9.304840316	9.55941126	-0.25	0.84	BTBD1	BTB domain containing 1
ILMN_1793915	10	111967699	111967750	0.00061666	0.035721856	FDR0.05	12.09010645	12.48743463	-0.40	0.76	MXI1	MAX interactor 1, dimerization protein
ILMN_2153332	6	16299644	16299695	0.0006169	0.035721856	FDR0.05	7.061936667	7.407065235	-0.35	0.79	ATXN1	ataxin 1
ILMN_2393243	3	112714034	112714085	0.00061773	0.035721856	FDR0.05	8.478263295	8.877254172	-0.40	0.76	GTPBP8	GTP binding protein 8 (putative)
ILMN_1779857	9	110247731	110247782	0.00061847	0.035721856	FDR0.05	6.386395641	6.719063561	-0.33	0.79	KLF4	Kruppel like factor 4
ILMN_1757415	1	53152597	53152648	0.00062511	0.035935249	FDR0.05	8.328671142	7.70840004	0.62	1.54	COA7	cytochrome c oxidase assembly factor 7
ILMN_1663327	11	15992221	15992272	0.00063517	0.036341699	FDR0.05	10.94120623	11.23513215	-0.29	0.82	SOX6	SRY-box transcription factor 6
ILMN_1710408	11	27389449	27389500	0.00064472	0.036715633	FDR0.05	8.332512665	8.418751224	-0.09	0.94	LGR4	leucine rich repeat containing G protein-coupled receptor 4
ILMN_1708991	10	35858362	35858413	0.00065005	0.036719399	FDR0.05	7.530284236	8.376854732	-0.85	0.56	CCNY	cyclin Y
ILMN_1773968	1	67874017	67874068	0.00065089	0.036719399	FDR0.05	8.011951886	8.209198446	-0.20	0.87	SERBP1	SERPINE1 mRNA binding protein 1
ILMN_1789464	5	79407322	79407373	0.00065382	0.036719399	FDR0.05	6.771153001	7.45838055	-0.69	0.62	SERINC5	serine incorporator 5
ILMN_2116516	2	175293336	175293387	0.00066069	0.036829662	FDR0.05	6.231502291	7.098053539	-0.87	0.55	SCRN3	secernin 3
ILMN_1800078	11	33880353	33880403	0.00066565	0.036829662	FDR0.05	6.917467898	7.06129496	-0.14	0.91	LMO2	LIM domain only 2
ILMN_3247325	13	45965523	45965574	0.00066787	0.036829662	FDR0.05	6.41202209	7.361276469	-0.95	0.52	TPT1-AS1	TPT1 antisense RNA 1
ILMN_1788832	18	19450304	19450355	0.00067344	0.036969319	FDR0.05	6.109962348	7.693257799	-1.58	0.33	MIB1	MIB E3 ubiquitin protein ligase 1
ILMN_2113362	16	18803334	18803385	0.00068294	0.037171617	FDR0.05	11.08934941	11.66961911	-0.58	0.67	ARL6IP1	ADP ribosylation factor like GTPase 6 interacting protein 1
ILMN_2063586	1	25170290	25170341	0.00068322	0.037171617	FDR0.05	7.295053056	8.124206453	-0.83	0.56	CLIC4	chloride intracellular channel 4
ILMN_1733560	15	65282111	65282162	0.00071362	0.038652642	FDR0.05	9.857674555	9.282277668	0.58	1.49	SPG21	SPG21 abhydrolase domain containing, maspardin
ILMN_1805247	17	35878383	35878434	0.00072331	0.039004383	FDR0.05	8.898293408	8.99911447	-0.10	0.93	SYNRG	synergin gamma
ILMN_1741711	21	37665748	37665799	0.00074286	0.039882317	FDR0.05	10.10774985	9.649300885	0.46	1.37	DOP1B	DOP1 leucine zipper like protein B
ILMN_2152429	12	110479316	110479367	0.00074837	0.040001876	FDR0.05	9.693266036	10.10055723	-0.41	0.75	C12orf76	chromosome 12 open reading frame 76
ILMN_1664186	3	179110848	179110899	0.00075315	0.040030382	FDR0.05	7.442891225	7.332485717	0.11	1.08	MFN1	mitofusin 1
ILMN_2133100	18	2917403	2917454	0.00075548	0.040030382	FDR0.05	8.005889915	9.297642182	-1.29	0.41	LPIN2	lipin 2
ILMN_2116811	7	6369364	6369415	0.0007598	0.040085358	FDR0.05	7.256623677	8.096642263	-0.84	0.56	FAM220A	family with sequence similarity 220 member A
ILMN_2223836	11	89934793	89934844	0.00076734	0.040308712	FDR0.05	7.788675228	8.943792534	-1.16	0.45	CHORDC1	cysteine and histidine rich domain containing 1
ILMN_1703153	16	4853580	4853632	0.00077074	0.040313154	FDR0.05	8.493322024	9.257969903	-0.76	0.59	GLYR1	glyoxylate reductase 1 homolog
ILMN_2372398	6	24536839	24536890	0.00079557	0.041434436	FDR0.05	10.18638821	10.96060632	-0.77	0.58	ALDH5A1	aldehyde dehydrogenase 5 family member A1
ILMN_2058468	6	90636614	90636665	0.0008176	0.041847788	FDR0.05	6.957760181	8.464762846	-1.51	0.35	BACH2	BTB domain and CNC homolog 2
ILMN_1662964	11	20530555	20530606	0.00081764	0.041847788	FDR0.05	6.700055032	7.737004316	-1.04	0.49	PRMT3	protein arginine methyltransferase 3
ILMN_1762615	10	126525037	126525088	0.00082373	0.041847788	FDR0.05	8.401440445	9.15456546	-0.75	0.59	ABRAXAS2	abraxas 2, BRISC complex subunit
ILMN_1656625	10	79797006	79797057	0.00082471	0.041847788	FDR0.05	12.55718734	12.42563419	0.13	1.10	RPS24	ribosomal protein S24
ILMN_2381753	4	76568425	76568476	0.00082628	0.041847788	FDR0.05	8.945742946	9.609207737	-0.66	0.63	G3BP2	G3BP stress granule assembly factor 2
ILMN_2115591	7	129773520	129773571	0.00082778	0.041847788	FDR0.05	6.691366918	7.961615177	-1.27	0.41	KLHDC10	kelch domain containing 10
ILMN_1739943	12	123782537	123782588	0.00082969	0.041847788	FDR0.05	8.572981631	9.544674737	-0.97	0.51	SBNO1	strawberry notch homolog 1
ILMN_1805826	13	103493551	103493602	0.00083098	0.041847788	FDR0.05	6.323735291	7.097456941	-0.77	0.58	BIVM	basic, immunoglobulin-like variable motif containing
ILMN_1682206	7	127220730	127220781	0.00085289	0.042621078	FDR0.05	6.501275535	7.413187924	-0.91	0.53	GCC1	GRIP and coiled-coil domain containing 1
ILMN_1750896	9	102732361	102732412	0.00085333	0.042621078	FDR0.05	9.208808549	9.500118231	-0.29	0.82	STX17	syntaxin 17
ILMN_2175447	7	149244291	149244342	0.00085846	0.042702452	FDR0.05	10.40062879	10.11585105	0.28	1.22	ZNF767P	zinc finger family member 767, pseudogene
ILMN_1728810	2	206988239	206988290	0.00086733	0.042860971	FDR0.05	8.001121107	8.490917208	-0.49	0.71	NDUFS1	NADH:ubiquinone oxidoreductase core subunit S1
ILMN_1710598	1	2239340	2239391	0.00086868	0.042860971	FDR0.05	9.662989031	9.456530702	0.21	1.15	SKI	SKI proto-oncogene
ILMN_1745447	6	143072768	143072819	0.00087239	0.042870221	FDR0.05	6.751916838	7.463986504	-0.71	0.61	HIVEP2	HIVEP zinc finger 2
ILMN_2128428	5	39372752	39372803	0.00089383	0.043583176	FDR0.05	8.141931323	7.908318425	0.23	1.18	DAB2	DAB adaptor protein 2
ILMN_1694603	3	47627733	47627784	0.00089405	0.043583176	FDR0.05	11.32389633	11.61622	-0.29	0.82	SMARCC1	SWI/SNF related, matrix associated, actin dependent regulator of chromatin subfamily c member 1
ILMN_1752793	13	21722709	21722760	0.00090691	0.043982033	FDR0.05	9.271235021	9.545180756	-0.27	0.83	SAP18	Sin3A associated protein 18
ILMN_1671742	13	115051776	115051827	0.00090945	0.043982033	FDR0.05	7.663613654	8.101925577	-0.44	0.74	UPF3A	UPF3A regulator of nonsense mediated mRNA decay
ILMN_1713731	17	6913665	6913716	0.00091938	0.044286704	FDR0.05	8.027952906	9.031161895	-1.00	0.50	ALOX12	arachidonate 12-lipoxygenase, 12S type
ILMN_1706990	18	32887642	32887693	0.0009625	0.046180955	FDR0.05	10.17734574	10.47757319	-0.30	0.81	ZNF271P	zinc finger protein 271, pseudogene
ILMN_1675797	7	37991200	37991251	0.00096835	0.046243388	FDR0.05	6.113816986	6.547081083	-0.43	0.74	EPDR1	ependymin related 1
ILMN_1696383	19	30106290	30106341	0.00097139	0.046243388	FDR0.05	8.788780977	9.195541696	-0.41	0.75	POP4	POP4 homolog, ribonuclease P/MRP subunit

id	chr	p.start.hg19	p.end.hg19	p_adj	fdr	sig	Ex_BPD.cord	Ex_noBPD.cord	dEx.cord	dEx_FD	symbols	description
ILMN_1730731	10	101910052	101910103	0.00098052	0.046496262	FDR0.05	7.135616914	7.385550735	-0.25	0.84	***ERLIN1***	ER lipid raft associated 1
ILMN_1689142	3	69105009	69105059	0.00098622	0.046505883	FDR0.05	7.281738772	8.343849616	-1.06	0.48	***UBA3***	ubiquitin like modifier activating enzyme 3
ILMN_1778681	5	158125898	158125949	0.00098835	0.046505883	FDR0.05	9.727677194	9.918413751	-0.19	0.88	***EBF1***	EBF transcription factor 1
ILMN_1685467	18	32837922	32837973	0.00100078	0.046909416	FDR0.05	7.728202264	8.547549347	-0.82	0.57	***ZNF397***	zinc finger protein 397
ILMN_2178088	5	43175395	43175446	0.00100542	0.046946527	FDR0.05	7.307011089	8.163909556	-0.86	0.55	***ZNF131***	zinc finger protein 131
ILMN_3237769	1	143767831	143767882	0.001033	0.04801752	FDR0.05	8.978421483	8.165576688	0.81	1.76	***PPIAL4G//PPIAL4H***	peptidylprolyl isomerase A like 4G//peptidylprolyl isomerase A like 4H
ILMN_2169839	3	128888808	128888859	0.00104063	0.04801752	FDR0.05	10.93101647	11.49202106	-0.56	0.68	***CNBP***	CCHC-type zinc finger nucleic acid binding protein
ILMN_1766718	5	89811762	89811813	0.001044	0.04801752	FDR0.05	8.096007253	9.311027835	-1.22	0.43	***LYSMD3***	LysM domain containing 3
ILMN_1782057	1	154323422	154323473	0.00104816	0.04801752	FDR0.05	7.441429773	8.459560674	-1.02	0.49	***ATP8B2***	ATPase phospholipid transporting 8B2
ILMN_1721849	3	44637266	44637317	0.00105154	0.04801752	FDR0.05	6.742815932	6.691870383	0.05	1.04	***ZNF660***	zinc finger protein 660
ILMN_2151488	9	86618767	86618818	0.00105375	0.04801752	FDR0.05	7.761275991	8.497911738	-0.74	0.60	***RMI1***	RecQ mediated genome instability 1
ILMN_1745256	5	139063269	139063320	0.00105594	0.04801752	FDR0.05	7.861664046	8.850571493	-0.99	0.50	***CXXC5***	CXXC finger protein 5
ILMN_1778617	5	68662367	68662418	0.00106866	0.048415439	FDR0.05	6.715406787	8.094406083	-1.38	0.38	***AK6***	adenylate kinase 6|TATA-box binding protein associated factor 9
ILMN_1778617	5	68662367	68662418	0.00106866	0.048415439	FDR0.05	6.715406787	8.094406083	-1.38	0.38	***TAF9***	adenylate kinase 6|TATA-box binding protein associated factor 9
ILMN_2316878	12	15749726	15749777	0.00108565	0.049002976	FDR0.05	5.796271118	6.73565495	-0.94	0.52	***PTPRO***	protein tyrosine phosphatase receptor type O
ILMN_1802615	7	92234657	92234708	0.00108999	0.049017311	FDR0.05	8.71825053	9.300350634	-0.58	0.67	***CDK6***	cyclin dependent kinase 6
ILMN_1708098	1	145498764	145498815	0.00109722	0.049160901	FDR0.05	8.42643328	8.98456172	-0.56	0.68	***LIX1L***	limb and CNS expressed 1 like
ILMN_2198867	3	167217947	167217998	0.00110289	0.049234096	FDR0.05	7.75580714	7.41253885	0.34	1.27	***WDR49***	WD repeat domain 49

Table S9. Differentially methylated CpG sites associated with gestational age (GA) or birth weight (BW).

Illumina HumanMethylation 450 BeadChip analysis on cord blood DNA from nonBPD infants (n=93).

Bonferroni (BF) correction < 1.08E-07, false discovery rate (FDR) < 0.01. M.BPD=mean methlation in BPD samples. M.noBPD=mean methlation in non-BPD samples. dMeth=methylation difference.

Chr=chromosome. Pos.hg19=position on Genome Reference Consortium Human Build 37 (GRCh37, hg19). dist2TSS=distance to transcription start site.

Highlighted in gray-Common CpGs (n=5) beetween EWAS-GA and EWAS-BPD (Shown in Table S4)

Trait	#cpg	p	fdr	significance	chr	pos.hg19	gene.Illumina	gene.description	locations	dist2TSS	gene.nearest	gene.description
**GA**	cg00000714	9.51E-07	0.000647239	FDR0.01	19	54695678	'MBOAT7;TSEN34	lysophospholipid acyltransferase 7;tRNA-splicing endonuclease subunit Sen34	TSS5000;TSS5000;TSS5000;TSS5000;Body;Body;Body;Body	520	'TSEN34	tRNA-splicing endonuclease subunit Sen34
**GA**	cg00016255	7.46E-07	0.000541588	FDR0.01	17	41133928	'PTGES3L-AARSD1;PTGES3L;RUNDC1	PTGES3L-AARSD1 protein isoform 1;putative protein PTGES3L;RUN domain-containing protein 1	TSS5000;TSS1500;TSS1500;TSS1500;TSS1500;Body;Body	1347	'RUNDC1	RUN domain-containing protein 1
**GA**	cg00029284	1.20E-06	0.000768188	FDR0.01	12	111731203	'CUX2	homeobox protein cut-like 2 isoform 2	Body;Body	-10742	'MIR6760	microRNA 6760
**GA**	cg00049664	2.00E-05	0.005597731	FDR0.01	16	66613334	'CKLF-CMTM1;CMTM1;CMTM2	CKLF-CMTM1 protein isoform 3;CKLF-like MARVEL transmembrane domain-containing protein 1;CKLF-like MARVEL transmembrane domain-containing protein 2	TES5000;TES5000;TES5000;TES5000;TES5000;TES5000;TES5000;TES5000;TES5000;TES5000;TES5000;TSS200;TSS200	-31	'CMTM2	CKLF-like MARVEL transmembrane domain-containing protein 2
**GA**	cg00055073	1.13E-05	0.003752139	FDR0.01	2	43398118	'			55627	'ZFP36L2	zinc finger protein 36, C3H1 type-like 2
**GA**	cg00066663	1.37E-10	9.88E-07	BF|FDR0.01	17	7792674	'NAA38;CHD3	N-alpha-acetyltransferase 38, NatC auxiliary subunit isoform 1 precursor;chromodomain-helicase-DNA-binding protein 3 isoform 3	TSS5000;Body;Body;Body	568	'CHD3	chromodomain-helicase-DNA-binding protein 3 isoform 3
**GA**	cg00085493	3.15E-05	0.007442776	FDR0.01	1	208040203	'			-44155	'MIR29B2CHG	MIR29B2 and MIR29C host gene
**GA**	cg00086710	3.25E-05	0.00760174	FDR0.01	4	89204074	'PPM1K;PPM1K-DT	protein phosphatase 1K, mitochondrial;PPM1K divergent transcript	Body;TSS5000;TSS5000;TSS5000	1639	'PPM1K	protein phosphatase 1K, mitochondrial
**GA**	cg00092443	3.70E-05	0.008354942	FDR0.01	20	46578370	'			-33599	'LINC01523	long intergenic non-protein coding RNA 1523
**GA**	cg00093643	3.45E-06	0.001615437	FDR0.01	15	44579510	'CASC4		TSS1500;TSS1500;TSS1500	-1406	'CASC4	
**GA**	cg00102731	5.21E-06	0.002179038	FDR0.01	20	2729245	'EBF4	transcription factor COE4	Body;Body	52028	'CPXM1	probable carboxypeptidase X1 precursor
**GA**	cg00110832	9.63E-06	0.003339791	FDR0.01	6	32121130	'PRRT1;LOC100507547;PPT2;PPT2-EGFL8	proline-rich transmembrane protein 1;uncharacterized LOC100507547;lysosomal thioesterase PPT2 precursor;PPT2-EGFL8 readthrough (NMD candidate)	TSS1500;TSS1500;Body;Body;Body;Body;TSS200;TSS1500;TSS1500;TSS1500	-174	'PPT2	lysosomal thioesterase PPT2 precursor
**GA**	cg00112238	8.36E-06	0.003040491	FDR0.01	1	54719938	'SSBP3	single-stranded DNA-binding protein 3	Body;Body;Body	17823	'SSBP3-AS1	SSBP3 antisense RNA 1
**GA**	cg00115747	2.17E-05	0.005895188	FDR0.01	17	74940026	'MGAT5B	alpha-1,6-mannosylglycoprotein 6-beta-N-acetylglucosaminyltransferase B isoform 1	Body;Body;Body	25255	'LOC105371899	uncharacterized LOC105371899
**GA**	cg00118342	4.96E-06	0.002091295	FDR0.01	2	106777038	'UXS1	UDP-glucuronic acid decarboxylase 1 isoform a	Body;Body;Body	-21701	'UXS1	UDP-glucuronic acid decarboxylase 1 isoform a
**GA**	cg00151922	2.75E-05	0.006861496	FDR0.01	20	10652103	'JAG1	protein jagged-1 precursor	Body	2544	'JAG1	protein jagged-1 precursor
**GA**	cg00153101	2.92E-12	6.22E-08	BF|FDR0.01	1	2431753	'PLCH2	1-phosphatidylinositol 4,5-bisphosphate phosphodiesterase eta-2 isoform 3	Body;Body;Body	24000	'PLCH2	1-phosphatidylinositol 4,5-bisphosphate phosphodiesterase eta-2 isoform 3
**GA**	cg00161791	4.52E-05	0.00940876	FDR0.01	21	45575014	'LINC01678	long intergenic non-protein coding RNA 1678	TES5000	4731	'LINC01678	long intergenic non-protein coding RNA 1678
**GA**	cg00166343	4.22E-10	2.22E-06	BF|FDR0.01	17	29150100	'CRLF3	cytokine receptor-like factor 3	Body;Body	1610	'CRLF3	cytokine receptor-like factor 3
**GA**	cg00171729	1.46E-06	0.000883861	FDR0.01	6	52416856	'TRAM2	translocating chain-associated membrane protein 2	Body	25002	'TRAM2	translocating chain-associated membrane protein 2
**GA**	cg00172603	2.94E-06	0.00144072	FDR0.01	1	54822031	'SSBP3	single-stranded DNA-binding protein 3	Body;Body;Body	50036	'SSBP3	single-stranded DNA-binding protein 3
**GA**	cg00202460	4.43E-07	0.000368854	FDR0.01	15	83680873	'C15orf40;BTBD1	UPF0235 protein C15orf40 isoform c;BTB/POZ domain-containing protein 1	TSS1500;TSS1500;TSS1500;TSS1500;TSS1500;TSS1500;TSS1500;TES5000;TES5000	-482	'C15orf40	UPF0235 protein C15orf40 isoform c
**GA**	cg00232092	4.21E-06	0.001873061	FDR0.01	7	5518887	'FBXL18	F-box/LRR-repeat protein 18	Body;3'UTR;3'UTR;3'UTR	16661	'MIR589	microRNA 589
**GA**	cg00232483	7.66E-06	0.002863133	FDR0.01	16	19849514	'IQCK	IQ domain-containing protein K	Body;Body;Body;Body;Body	46656	'GPRC5B	G-protein coupled receptor family C group 5 member B
**GA**	cg00241281	2.10E-06	0.001142688	FDR0.01	14	105779794	'BRF1;PACS2	transcription factor TFIIIB subunit BRF1;phosphofurin acidic cluster sorting protein 2 isoform 2	Body;Body;Body;TSS1500;TSS1500	-1290	'PACS2	phosphofurin acidic cluster sorting protein 2 isoform 2
**GA**	cg00246451	4.32E-05	0.009131118	FDR0.01	1	155949012	'ARHGEF2	rho guanine nucleotide exchange factor 2	TSS1500;TSS1500;TSS1500;5'UTR;Body;TSS1500	69	'ARHGEF2	rho guanine nucleotide exchange factor 2
**GA**	cg00256046	3.14E-05	0.007428671	FDR0.01	1	217306763	'ESRRG	estrogen-related receptor gamma	Body;Body;Body;Body	4281	'ESRRG	estrogen-related receptor gamma
**GA**	cg00257455	3.41E-07	0.000305698	FDR0.01	1	65530538	'JAK1	tyrosine-protein kinase JAK1	Body;Body;Body	2891	'JAK1	tyrosine-protein kinase JAK1
**GA**	cg00275449	1.19E-06	0.000765013	FDR0.01	3	195162127	'ACAP2	arf-GAP with coiled-coil, ANK repeat and PH domain-containing protein 2	Body	1622	'ACAP2	arf-GAP with coiled-coil, ANK repeat and PH domain-containing protein 2
**GA**	cg00277898	2.95E-05	0.007183669	FDR0.01	6	31745181	'VWA7;VARS1	von Willebrand factor A domain-containing protein 7 precursor;valine--tRNA ligase	TSS200;TES5000	-76	'VWA7	von Willebrand factor A domain-containing protein 7 precursor
**GA**	cg00285620	2.60E-05	0.006646265	FDR0.01	11	102147694	'			-40520	'BIRC3	baculoviral IAP repeat-containing protein 3
**GA**	cg00292107	7.48E-06	0.002812873	FDR0.01	1	32800350	'HDAC1;MARCKSL1	histone deacetylase 1;MARCKS-related protein	TES5000;Body;Body	1484	'MARCKSL1	MARCKS-related protein
**GA**	cg00294109	2.61E-05	0.006646408	FDR0.01	3	3219781	'CRBN	protein cereblon	Body;Body	1620	'CRBN	protein cereblon
**GA**	cg00298065	1.54E-06	0.000925446	FDR0.01	19	58715577	'ZNF274	neurotrophin receptor-interacting factor homolog isoform d	Body;Body;Body;Body	21150	'ZNF274	neurotrophin receptor-interacting factor homolog isoform d
**GA**	cg00306721	4.22E-07	0.000357617	FDR0.01	11	62477480	'BSCL2;HNRNPUL2-BSCL2;GNG3;HNRNPUL2	seipin isoform 1;HNRNPUL2-BSCL2 readthrough (NMD candidate);guanine nucleotide-binding protein G(I)/G(S)/G(O) subunit gamma-3;heterogeneous nuclear ribonucleoprotein U-like protein 2	TSS5000;TSS5000;TSS5000;TSS1500;TSS1500;Body;TES5000;TES5000	-389	'BSCL2	seipin isoform 1
**GA**	cg00310523	8.01E-07	0.000573055	FDR0.01	12	86230176	'RASSF9	ras association domain-containing protein 9	5'UTR	11	'RASSF9	ras association domain-containing protein 9
**GA**	cg00312125	6.25E-11	5.82E-07	BF|FDR0.01	4	84030111	'PLAC8	placenta-specific gene 8 protein	Body;Body;Body	931	'PLAC8	placenta-specific gene 8 protein
**GA**	cg00320288	8.97E-06	0.003177628	FDR0.01	16	85863167	'			29867	'COX4I1	cytochrome c oxidase subunit 4 isoform 1, mitochondrial
**GA**	cg00321478	3.87E-05	0.00858547	FDR0.01	1	197238398	'CRB1	protein crumbs homolog 1 precursor	Body;Body;Body;Body;Body;Body	1017	'CRB1	protein crumbs homolog 1 precursor
**GA**	cg00323916	1.98E-05	0.005541888	FDR0.01	22	38057185	'LOC101927051;PDXP	;pyridoxal phosphate phosphatase	TSS5000;Body	2456	'PDXP	pyridoxal phosphate phosphatase
**GA**	cg00324725	2.23E-05	0.005990582	FDR0.01	16	56581212	'			-17772	'MT4	metallothionein-like protein type 2
**GA**	cg00327947	1.23E-05	0.004006974	FDR0.01	16	85482849	'			-142918	'MIR5093	microRNA 5093
**GA**	cg00328227	3.59E-11	4.22E-07	BF|FDR0.01	1	109204325	'HENMT1	small RNA 2'-O-methyltransferase	TSS200;TSS1500	-177	'HENMT1	small RNA 2'-O-methyltransferase
**GA**	cg00330059	1.21E-05	0.003943536	FDR0.01	4	183637135	'TENM3	teneurin-3	Body	201271	'DCTD	deoxycytidylate deaminase
**GA**	cg00340584	4.68E-05	0.009616031	FDR0.01	1	149512963	'			-40039	'PPIAL4C	peptidyl-prolyl cis-trans isomerase A-like 4A/B/C
**GA**	cg00343092	2.17E-05	0.005892136	FDR0.01	22	43547974	'TSPO	translocator protein	Body;Body;5'UTR;5'UTR	34	'TSPO	translocator protein
**GA**	cg00397764	6.63E-06	0.00259145	FDR0.01	10	115939717	'TDRD1	tudor domain-containing protein 1 isoform 1	Body;Body	606	'TDRD1	tudor domain-containing protein 1 isoform 1
**GA**	cg00411595	6.62E-09	1.64E-05	BF|FDR0.01	9	136339349	'CACFD1;SLC2A6	calcium channel flower homolog;solute carrier family 2, facilitated glucose transporter member 6	TES5000;TES5000;TES5000;TES5000;Body;Body	4872	'SLC2A6	solute carrier family 2, facilitated glucose transporter member 6
**GA**	cg00412294	1.97E-09	6.98E-06	BF|FDR0.01	12	97300582	'NEDD1	protein NEDD1	TSS1500;TSS1500;TSS1500;TSS1500	-452	'NEDD1	protein NEDD1
**GA**	cg00422488	1.44E-05	0.004470366	FDR0.01	9	100747767	'ANP32B	acidic leucine-rich nuclear phosphoprotein 32 family member B	Body	2145	'ANP32B	acidic leucine-rich nuclear phosphoprotein 32 family member B
**GA**	cg00428382	2.96E-05	0.007183669	FDR0.01	4	124427390	'			106696	'SPRY1	protein sprouty homolog 1
**GA**	cg00434573	1.60E-06	0.000951823	FDR0.01	19	2041960	'MKNK2	MAP kinase-interacting serine/threonine-protein kinase 2	Body;Body	9283	'MKNK2	MAP kinase-interacting serine/threonine-protein kinase 2
**GA**	cg00442282	1.23E-05	0.004009397	FDR0.01	17	38471064	'RARA	retinoic acid receptor alpha	Body;TSS5000;TSS5000	-3432	'RARA	retinoic acid receptor alpha
**GA**	cg00446123	1.51E-05	0.004635914	FDR0.01	20	62367888	'ZGPAT;LIME1;SLC2A4RG	zinc finger CCCH-type with G patch domain-containing protein isoform c;lck-interacting transmembrane adapter 1 precursor;SLC2A4 regulator	TES5000;TES5000;TES5000;TES5000;TES5000;Body;TSS200;TSS200;TSS5000	-127	'LIME1	lck-interacting transmembrane adapter 1 precursor
**GA**	cg00466249	8.05E-06	0.002965062	FDR0.01	17	79269884	'SLC38A10	putative sodium-coupled neutral amino acid transporter 10	TSS1500;TSS1500	-777	'SLC38A10	putative sodium-coupled neutral amino acid transporter 10
**GA**	cg00471989	2.21E-05	0.005949657	FDR0.01	16	30380183	'TBC1D10B;MYLPF	TBC1 domain family member 10B;myosin regulatory light chain 2, skeletal muscle isoform	Body;TSS5000;TSS5000	1632	'TBC1D10B	TBC1 domain family member 10B

Trait	#cpg	p	fdr	significance	chr	pos.hg19	gene.Illumina	gene.description	locations	dist2TSS	gene.nearest	gene.description
**GA**	cg00478421	4.11E-06	0.001845388	FDR0.01	2	11515109	'			-18997	'LINC00570	long intergenic non-protein coding RNA 570
**GA**	cg00490097	2.99E-05	0.007229011	FDR0.01	11	1718586	'KRTAP5-6	keratin-associated protein 5-6	1stExon	162	'KRTAP5-6	keratin-associated protein 5-6
**GA**	cg00496272	3.28E-07	0.000296543	FDR0.01	16	67877232	'CENPT;THAP11;NUTF2	centromere protein T;THAP domain-containing protein 11;nuclear transport factor 2	Body;1stExon;TSS5000;TSS5000;TSS5000;TSS5000;TSS5000	1010	'THAP11	THAP domain-containing protein 11
**GA**	cg00499289	7.06E-07	0.000520965	FDR0.01	2	113341849	'POLR1B;CHCHD5	DNA-directed RNA polymerase I subunit RPA2;coiled-coil-helix-coiled-coil-helix domain-containing protein 5 isoform b	TES5000;TES5000;TES5000;TES5000;TES5000;TES5000;TES5000;TES5000;TES5000;TES5000;TSS200;TSS200;TSS1500;TSS1500;TSS1500;TSS1500	-174	'CHCHD5	coiled-coil-helix-coiled-coil-helix domain-containing protein 5 isoform b
**GA**	cg00499707	1.19E-07	0.000138292	FDR0.01	20	34679665	'EPB41L1	band 4.1-like protein 1	Body;TSS1500;TSS1500;TSS1500	330	'EPB41L1	band 4.1-like protein 1
**GA**	cg00499838	2.35E-05	0.006189164	FDR0.01	20	61148684	'MIR1-1HG-AS1;MIR1-1HG;MIR1-1	MIR1-1HG antisense RNA 1;uncharacterized protein MIR1-1HG;microRNA 1-1	Body;Body;Body;TSS5000	84	'MIR1-1HG-AS1	MIR1-1HG antisense RNA 1
**GA**	cg00506049	3.09E-05	0.007368972	FDR0.01	7	75887380	'SRRM3	serine/arginine repetitive matrix protein 3 isoform a	Body;Body	-44609	'HSPB1	heat shock protein beta-1
**GA**	cg00509451	4.80E-05	0.009790278	FDR0.01	11	64811775	'ARL2-SNX15;SNX15;SAC3D1;NAALADL1	ARL2-SNX15 readthrough (NMD candidate);sorting nexin-15;SAC3 domain-containing protein 1;aminopeptidase NAALADL1	TES5000;TES5000;TES5000;Body;3'UTR;Body;Body;Body;3'UTR;Body;TES5000	3094	'SAC3D1	SAC3 domain-containing protein 1
**GA**	cg00510160	4.43E-05	0.009280449	FDR0.01	12	5311273	'			41044	'LINC02443	long intergenic non-protein coding RNA 2443
**GA**	cg00512459	1.93E-05	0.005472556	FDR0.01	15	88266173	'			146014	'LINC00052	long intergenic non-protein coding RNA 52
**GA**	cg00513984	1.25E-05	0.00403741	FDR0.01	12	125039177	'NCOR2	nuclear receptor corepressor 2	Body;Body;Body	12981	'NCOR2	nuclear receptor corepressor 2
**GA**	cg00514628	1.77E-08	3.15E-05	BF|FDR0.01	22	30819337	'SEC14L2;MTFP1	SEC14-like protein 2;mitochondrial fission process protein 1	3'UTR;3'UTR;3'UTR;TSS5000;TSS5000	-2418	'MTFP1	mitochondrial fission process protein 1
**GA**	cg00528616	4.94E-05	0.009970085	FDR0.01	16	75036318	'ZNRF1	E3 ubiquitin-protein ligase ZNRF1	Body	3397	'ZNRF1	E3 ubiquitin-protein ligase ZNRF1
**GA**	cg00533811	2.10E-05	0.005782308	FDR0.01	22	51021411	'CPT1B;CHKB-CPT1B;CHKB;CHKB-DT	carnitine O-palmitoyltransferase 1, muscle isoform;CHKB-CPT1B readthrough (NMD candidate);choline/ethanolamine kinase;CHKB divergent transcript	TSS5000;TSS5000;TSS5000;TSS5000;TSS5000;TSS5000;Body;5'UTR;TSS200;TSS200	17	'CHKB-CPT1B	CHKB-CPT1B readthrough (NMD candidate)
**GA**	cg00537673	4.46E-06	0.001942174	FDR0.01	6	43141823	'SRF	serum response factor	Body;1stExon	1690	'SRF	serum response factor
**GA**	cg00539049	3.05E-05	0.007318277	FDR0.01	2	149933534	'LYPD6B	ly6/PLAUR domain-containing protein 6B precursor	Body;Body;Body;Body;TSS1500	-501	'LYPD6B	ly6/PLAUR domain-containing protein 6B precursor
**GA**	cg00543073	1.68E-08	3.10E-05	BF|FDR0.01	2	60773386	'BCL11A	B-cell lymphoma/leukemia 11A	Body;Body;Body;Body;Body	7247	'BCL11A	B-cell lymphoma/leukemia 11A
**GA**	cg00547530	4.88E-05	0.009911178	FDR0.01	16	3559687	'CLUAP1	clusterin-associated protein 1	Body;Body;TSS1500	-206	'CLUAP1	clusterin-associated protein 1
**GA**	cg00551609	5.88E-06	0.002383578	FDR0.01	2	74214662	'TET3	methylcytosine dioxygenase TET3 isoform 2	Body	2626	'TET3	methylcytosine dioxygenase TET3 isoform 2
**GA**	cg00575744	1.63E-07	0.000171971	FDR0.01	6	32908642	'HLA-DMB	HLA class II histocompatibility antigen, DM beta chain precursor	5'UTR	163	'HLA-DMB	HLA class II histocompatibility antigen, DM beta chain precursor
**GA**	cg00579402	1.26E-06	0.000795355	FDR0.01	19	5838999	'FUT6;FUT3	4-galactosyl-N-acetylglucosaminide 3-alpha-L-fucosyltransferase FUT6 isoform 1;3-galactosyl-N-acetylglucosaminide 4-alpha-L-fucosyltransferase FUT3	TSS200;TSS200;TSS200;5'UTR;5'UTR;TES5000;TES5000;TES5000;TES5000	-196	'FUT6	4-galactosyl-N-acetylglucosaminide 3-alpha-L-fucosyltransferase FUT6 isoform 1
**GA**	cg00583733	5.49E-09	1.44E-05	BF|FDR0.01	16	30075102	'LOC112694756;ALDOA	uncharaterized LOC112694756 isoform 2;fructose-bisphosphate aldolase A	Body;Body;Body;TSS1500;TSS1500;TSS5000;TSS5000	-497	'ALDOA	fructose-bisphosphate aldolase A
**GA**	cg00589617	4.68E-07	0.000384991	FDR0.01	1	230415343	'GALNT2	polypeptide N-acetylgalactosaminyltransferase 2 precursor	3'UTR;3'UTR	146735	'PGBD5	piggyBac transposable element-derived protein 5
**GA**	cg00595243	4.94E-05	0.009971192	FDR0.01	6	133137893	'RPS12;SNORD101;SNORD100;SNORA33	40S ribosomal protein S12;small nucleolar RNA, C/D box 101;small nucleolar RNA, C/D box 100;small nucleolar RNA, H/ACA box 33	Body;TES5000;TSS200;TSS1500	-47	'SNORD100	small nucleolar RNA, C/D box 100
**GA**	cg00601836	1.64E-10	1.11E-06	BF|FDR0.01	6	152130332	'ESR1	estrogen receptor	Body;Body;Body;Body;Body;Body;5'UTR	91	'ESR1	estrogen receptor
**GA**	cg00614157	1.09E-06	0.000714957	FDR0.01	5	112258374	'REEP5	receptor expression-enhancing protein 5	TSS1500	-450	'REEP5	receptor expression-enhancing protein 5
**GA**	cg00654448	1.20E-08	2.43E-05	BF|FDR0.01	8	142365315	'GPR20	G-protein coupled receptor 20	TES5000	12071	'GPR20	G-protein coupled receptor 20
**GA**	cg00682367	3.78E-06	0.001734471	FDR0.01	20	48963280	'			-25401	'LINC01271	long intergenic non-protein coding RNA 1271
**GA**	cg00688810	1.03E-05	0.003507333	FDR0.01	17	7517138	'FXR2;SHBG	fragile X mental retardation syndrome-related protein 2;sex hormone-binding globulin	Body;TSS1500	-243	'SHBG	sex hormone-binding globulin
**GA**	cg00688840	1.12E-09	4.44E-06	BF|FDR0.01	14	106363051	'			-20786	'FAM30A	family with sequence similarity 30 member A
**GA**	cg00695799	1.44E-07	0.000158035	FDR0.01	6	149803087	'ZC3H12D	probable ribonuclease ZC3H12D	Body	3063	'ZC3H12D	probable ribonuclease ZC3H12D
**GA**	cg00697125	3.05E-05	0.007307701	FDR0.01	8	103668147	'KLF10;LOC101927245	Krueppel-like factor 10;uncharacterized LOC101927245	TSS5000;TSS5000;TSS5000;TSS200;TSS1500;TSS1500	-194	'KLF10	Krueppel-like factor 10
**GA**	cg00708789	3.56E-06	0.001646164	FDR0.01	4	16083440	'PROM1	prominin-1 precursor	Body;Body;Body;Body;Body;Body;Body;Body	1519	'PROM1	prominin-1 precursor
**GA**	cg00713294	3.31E-08	5.20E-05	BF|FDR0.01	1	55678649	'USP24;LOC100507634;MIR4422HG	ubiquitin carboxyl-terminal hydrolase 24;uncharacterized LOC100507634;MIR4422 host gene	Body;TSS5000;TSS5000	2388	'USP24	ubiquitin carboxyl-terminal hydrolase 24
**GA**	cg00716470	2.62E-05	0.006669038	FDR0.01	2	176952319	'EVX2	homeobox even-skipped homolog protein 2	TSS5000	-3629	'EVX2	homeobox even-skipped homolog protein 2
**GA**	cg00725635	3.10E-05	0.007375086	FDR0.01	1	193155806	'CDC73;B3GALT2	parafibromin;beta-1,3-galactosyltransferase 2	Body;TSS200	-63	'B3GALT2	beta-1,3-galactosyltransferase 2
**GA**	cg00733683	2.77E-06	0.001377482	FDR0.01	19	1244464	'ATP5F1D;MIDN	ATP synthase subunit delta, mitochondrial;midnolin	Body;3'UTR;TSS5000	2715	'ATP5F1D	ATP synthase subunit delta, mitochondrial
**GA**	cg00739778	2.35E-06	0.001244039	FDR0.01	17	2908023	'RAP1GAP2	rap1 GTPase-activating protein 2 isoform 2	Body;Body;Body	-38834	'LOC101927911	uncharacterized LOC101927911
**GA**	cg00770871	2.53E-06	0.001295341	FDR0.01	12	2999840	'RHNO1;TULP3	RAD9, HUS1, RAD1-interacting nuclear orphan protein 1;tubby-related protein 3	TES5000;TES5000;TES5000;TES5000;TES5000;TES5000;TES5000;TSS1500;TSS1500	-216	'TULP3	tubby-related protein 3
**GA**	cg00792107	2.55E-05	0.006538599	FDR0.01	12	133181600	'LRCOL1	leucine-rich colipase-like protein 1 precursor	Body	5568	'LRCOL1	leucine-rich colipase-like protein 1 precursor
**GA**	cg00792751	9.52E-06	0.003315348	FDR0.01	11	44979029	'			-6172	'TP53I11	tumor protein p53-inducible protein 11
**GA**	cg00793181	1.79E-10	1.16E-06	BF|FDR0.01	12	52995634	'KRT72	keratin, type II cytoskeletal 72 isoform 2	TSS1500;TSS1500;TSS1500	-387	'KRT72	keratin, type II cytoskeletal 72 isoform 2
**GA**	cg00794055	1.94E-08	3.33E-05	BF|FDR0.01	16	2544909	'TBC1D24	TBC1 domain family member 24	Body;Body	-18817	'ATP6V0C	V-type proton ATPase 16 kDa proteolipid subunit
**GA**	cg00796302	4.05E-07	0.000347154	FDR0.01	4	106477599	'ARHGEF38	rho guanine nucleotide exchange factor 38 isoform 2	Body;Body	3823	'ARHGEF38	rho guanine nucleotide exchange factor 38 isoform 2
**GA**	cg00813162	4.92E-05	0.00995413	FDR0.01	14	69443362	'ACTN1;ACTN1-AS1	alpha-actinin-1;	Body;Body;Body;TSS5000	2657	'ACTN1	alpha-actinin-1
**GA**	cg00839584	1.30E-05	0.004161245	FDR0.01	2	113542091	'IL1A	interleukin-1 alpha	TSS200;TSS200	-21	'IL1A	interleukin-1 alpha
**GA**	cg00841988	4.09E-05	0.008847244	FDR0.01	14	100204153	'			53519	'CYP46A1	cholesterol 24-hydroxylase
**GA**	cg00842595	2.92E-05	0.007128596	FDR0.01	17	263193	'LOC105371430;C17orf97	uncharacterized LOC105371430;protein LIAT1	Body;Body	621	'LOC105371430	uncharacterized LOC105371430
**GA**	cg00852033	3.92E-11	4.44E-07	BF|FDR0.01	16	2058572	'ZNF598	E3 ubiquitin-protein ligase ZNF598	Body	1250	'ZNF598	E3 ubiquitin-protein ligase ZNF598
**GA**	cg00862588	3.94E-05	0.008659885	FDR0.01	6	32909397	'HLA-DMB	HLA class II histocompatibility antigen, DM beta chain precursor	TSS1500	-592	'HLA-DMB	HLA class II histocompatibility antigen, DM beta chain precursor
**GA**	cg00866054	6.14E-06	0.002453578	FDR0.01	6	33142284	'COL11A2	collagen alpha-2(XI) chain precursor	Body;Body;Body	17960	'COL11A2	collagen alpha-2(XI) chain precursor
**GA**	cg00866476	1.78E-10	1.16E-06	BF|FDR0.01	2	95740682	'			46620	'MRPS5	mitochondrial 37S ribosomal protein MRPS5
**GA**	cg00869530	3.14E-10	1.81E-06	BF|FDR0.01	6	32155124	'AGER;PBX2;GPSM3	advanced glycosylation end product-specific receptor precursor;pre-B-cell le	TSS5000;TSS5000;TSS5000;TSS5	2855	'PBX2	pre-B-cell leukemia transcription factor 2
**GA**	cg00870279	6.33E-07	0.000481628	FDR0.01	6	33256222	'WDR46;MIR6873;PFDN6;MIR6834;RGL2	WD repeat-containing protein 46;microRNA 6873;prefoldin subunit 6;microR	Body;Body;TSS1500;TSS1500;T	794	'WDR46	WD repeat-containing protein 46
**GA**	cg00889363	1.87E-09	6.71E-06	BF|FDR0.01	2	206255841	'PARD3B	partitioning defective 3 homolog B isoform Par3Lb	Body;Body;Body;Body	-291382	'NRP2	neuropilin-2 precursor
**GA**	cg00895196	7.73E-06	0.002875279	FDR0.01	6	22147182	'CASC15;NBAT1	cancer susceptibility 15;neuroblastoma associated transcript 1	Body;Body	240	'NBAT1	neuroblastoma associated transcript 1
**GA**	cg00903584	1.97E-05	0.005530267	FDR0.01	1	202128682	'PTPN7	tyrosine-protein phosphatase non-receptor type 7	TSS200;5'UTR;Body;Body;Body;	-100	'PTPN7	tyrosine-protein phosphatase non-receptor type 7
**GA**	cg00930606	3.64E-08	5.56E-05	BF|FDR0.01	22	31740944	'PATZ1;LINC01521	POZ-, AT hook-, and zinc finger-containing protein 1;long intergenic non-prot	1stExon;1stExon;1stExon;1stEx	1388	'PATZ1	POZ-, AT hook-, and zinc finger-containing protein 1
**GA**	cg00932063	4.90E-05	0.00994741	FDR0.01	10	126234138	'LHPP	phospholysine phosphohistidine inorganic pyrophosphate phosphatase	Body;Body;Body	83747	'LHPP	phospholysine phosphohistidine inorganic pyrophosphate phosphatase
**GA**	cg00933182	3.62E-05	0.00821909	FDR0.01	17	43298345	'LOC339192;FMNL1	;formin-like protein 1	Body;TSS1500	787	'LOC339192	
**GA**	cg00933411	1.18E-08	2.40E-05	BF|FDR0.01	8	13373090	'DLC1	rho GTPase-activating protein 7 isoform 1	TSS1500;Body;Body;TSS1500	-730	'DLC1	rho GTPase-activating protein 7 isoform 1
**GA**	cg00960580	4.62E-05	0.009555729	FDR0.01	17	16285290	'UBB	polyubiquitin-B	1stExon;1stExon;1stExon;1stEx	512	'UBB	polyubiquitin-B
**GA**	cg00971396	3.18E-05	0.007478357	FDR0.01	3	184081400	'CLCN2;POLR2H	chloride channel protein 2;DNA-directed RNA polymerases I, II, and III subun	TSS5000;TSS5000;TSS5000;TSS5	252	'POLR2H	DNA-directed RNA polymerases I, II, and III subunit RPABC3

Trait	#cpg	p	fdr	significance	chr	pos.hg19	gene.Illumina	gene.description	locations	dist2TSS	gene.nearest	gene.description
**GA**	cg00980649	2.54E-05	0.006528281	FDR0.01	14	106366503	'			-17334	'FAM30A	family with sequence similarity 30 member A
**GA**	cg00988056	4.92E-05	0.009952864	FDR0.01	14	106374554	'			-9283	'FAM30A	family with sequence similarity 30 member A
**GA**	cg00989249	4.06E-05	0.008802751	FDR0.01	5	177548398	'N4BP3	NEDD4-binding protein 3	Body	7866	'N4BP3	NEDD4-binding protein 3
**GA**	cg00991744	1.33E-05	0.004238477	FDR0.01	12	41581484	'PDZRN4	PDZ domain-containing RING finger protein 4 isoform 1	TSS1500	-637	'PDZRN4	PDZ domain-containing RING finger protein 4 isoform 1
**GA**	cg00996315	1.69E-05	0.004994589	FDR0.01	17	42977550	'EFTUD2;CCDC103;GFAP;FAM187A	116 kDa U5 small nuclear ribonucleoprotein component;coiled-coil domain-c	TSS1500;TSS1500;TSS1500;TSS	454	'CCDC103	coiled-coil domain-containing protein 103
**GA**	cg01005605	4.91E-05	0.00994754	FDR0.01	2	60757902	'BCL11A	B-cell lymphoma/leukemia 11A	Body;Body;Body;Body;Body	22731	'BCL11A	B-cell lymphoma/leukemia 11A
**GA**	cg01014262	2.15E-05	0.005862662	FDR0.01	16	48533930	'			110161	'N4BP1	NEDD4-binding protein 1
**GA**	cg01024141	3.30E-05	0.007686286	FDR0.01	22	17090136	'TPTEP1	TPTE pseudogene 1	Body	7336	'TPTEP1	TPTE pseudogene 1
**GA**	cg01047631	6.33E-06	0.002514288	FDR0.01	16	54972573	'IRX5	iroquois-class homeodomain protein IRX-5	TES5000;TES5000	7463	'IRX5	iroquois-class homeodomain protein IRX-5
**GA**	cg01062443	4.23E-15	4.72E-10	BF|FDR0.01	19	1609369	'UQCR11;TCF3	cytochrome b-c1 complex subunit 10;transcription factor E2-alpha	TSS5000;3'UTR;3'UTR;3'UTR;3'U	-3908	'UQCR11	cytochrome b-c1 complex subunit 10
**GA**	cg01076459	1.87E-06	0.00105731	FDR0.01	17	2908230	'RAP1GAP2	rap1 GTPase-activating protein 2 isoform 2	Body;Body;Body	-39041	'LOC101927911	uncharacterized LOC101927911
**GA**	cg01082437	9.09E-08	0.00011354	BF|FDR0.01	21	33945064	'C21orf59-TCP10L;TCP10L	;T-complex protein 10A homolog 1	Body;Body;TES5000;TES5000	12769	'TCP10L	T-complex protein 10A homolog 1
**GA**	cg01091117	2.39E-06	0.001249385	FDR0.01	5	40682333	'PTGER4	prostaglandin E2 receptor EP4 subtype	Body	2317	'PTGER4	prostaglandin E2 receptor EP4 subtype
**GA**	cg01093786	9.88E-06	0.003397911	FDR0.01	11	96047698	'MAML2	mastermind-like protein 2	Body	-26903	'MIR1260B	microRNA 1260b
**GA**	cg01104590	2.87E-05	0.007057248	FDR0.01	11	3073136	'CARS1	cysteine--tRNA ligase, cytoplasmic	Body;Body;Body;Body;Body	5517	'CARS1	cysteine--tRNA ligase, cytoplasmic
**GA**	cg01108370	3.47E-05	0.007952439	FDR0.01	7	151330008	'PRKAG2	5'-AMP-activated protein kinase subunit gamma-2 isoform 1	TSS1500;TSS1500;Body;Body;B	-304	'PRKAG2	5'-AMP-activated protein kinase subunit gamma-2 isoform 1
**GA**	cg01131866	2.79E-05	0.006918106	FDR0.01	5	61601529	'KIF2A	kinesin-like protein KIF2A	TSS1500;TSS1500;TSS1500;TSS	-459	'KIF2A	kinesin-like protein KIF2A
**GA**	cg01132502	2.45E-06	0.001269074	FDR0.01	5	179335607	'TBC1D9B	TBC1 domain family member 9B	TSS1500;TSS1500	-710	'TBC1D9B	TBC1 domain family member 9B
**GA**	cg01154445	1.28E-05	0.004110581	FDR0.01	2	206548171	'NRP2	neuropilin-2 precursor	Body;Body;Body;Body;Body;Bo	947	'NRP2	neuropilin-2 precursor
**GA**	cg01160818	2.82E-05	0.006973091	FDR0.01	5	131747058	'IRF1-AS1	IRF1 antisense RNA 1	Body	438	'IRF1-AS1	IRF1 antisense RNA 1
**GA**	cg01208318	2.77E-05	0.006884049	FDR0.01	14	106329652	'MIR4537	microRNA 4537	TSS5000	-3930	'MIR4537	microRNA 4537
**GA**	cg01226614	1.61E-06	0.000952292	FDR0.01	13	44947593	'SERP2	stress-associated endoplasmic reticulum protein 2	TSS200;TSS1500;TSS1500	-192	'SERP2	stress-associated endoplasmic reticulum protein 2
**GA**	cg01243072	1.55E-05	0.004703133	FDR0.01	2	43398171	'			55574	'ZFP36L2	zinc finger protein 36, C3H1 type-like 2
**GA**	cg01256987	4.28E-05	0.009070784	FDR0.01	12	42539512	'GXYLT1	glucoside xylosyltransferase 1	TSS1500;TSS1500	-836	'GXYLT1	glucoside xylosyltransferase 1
**GA**	cg01266546	1.10E-06	0.000719407	FDR0.01	3	193919979	'LINC02036	long intergenic non-protein coding RNA 2036	TES5000;TES5000	4117	'LINC02036	long intergenic non-protein coding RNA 2036
**GA**	cg01315323	1.43E-05	0.004449935	FDR0.01	2	26569874	'ADGRF3;SELENOI	adhesion G-protein coupled receptor F3 isoform 2 precursor;ethanolaminep	TSS1500;TSS1500;TSS1500;TSS	-217	'ADGRF3	adhesion G-protein coupled receptor F3 isoform 2 precursor
**GA**	cg01329005	2.38E-10	1.46E-06	BF|FDR0.01	19	17516712	'BST2;BISPR;MVB12A	bone marrow stromal antigen 2;BST2 interferon stimulated positive regulato	TSS1500;Body;Body;Body	163	'MVB12A	multivesicular body subunit 12A isoform 2
**GA**	cg01348086	4.25E-05	0.009044808	FDR0.01	5	176785296	'RGS14	regulator of G-protein signaling 14	Body;Body;Body	372	'RGS14	regulator of G-protein signaling 14
**GA**	cg01354879	6.89E-06	0.002657936	FDR0.01	6	84140050	'ME1	NADP-dependent malic enzyme	Body	720	'ME1	NADP-dependent malic enzyme
**GA**	cg01366985	2.67E-05	0.006732359	FDR0.01	6	25167695	'			-29075	'CMAHP	cytidine monophospho-N-acetylneuraminic acid hydroxylase, pseudogene
**GA**	cg01367310	6.75E-06	0.002615681	FDR0.01	4	991593	'SLC26A1;IDUA	sulfate anion transporter 1 isoform b;alpha-L-iduronidase precursor	TSS5000;Body;Body;TSS5000;T	-1904	'IDUA	alpha-L-iduronidase precursor
**GA**	cg01367627	4.19E-07	0.000356459	FDR0.01	7	98477438	'TRRAP;MIR3609;SCARNA28	transformation/transcription domain-associated protein;microRNA 3609;sm	Body;Body;TSS5000;TSS5000	1326	'TRRAP	transformation/transcription domain-associated protein
**GA**	cg01400750	3.28E-05	0.007638953	FDR0.01	4	145956168	'ANAPC10	anaphase-promoting complex subunit 10	Body;Body;Body;Body;Body;Bo	62366	'ANAPC10	anaphase-promoting complex subunit 10
**GA**	cg01401037	4.95E-07	0.000396825	FDR0.01	3	185839955	'			-13077	'ETV5	ETS translocation variant 5
**GA**	cg01405107	1.07E-05	0.003593221	FDR0.01	17	46671635	'HOXB-AS3;HOXB5;HOXB6	HOXB cluster antisense RNA 3;homeobox protein Hox-B5;homeobox protein	Body;Body;Body;TSS1500;Body	-532	'HOXB5	homeobox protein Hox-B5
**GA**	cg01439383	2.90E-05	0.007107425	FDR0.01	7	2728776	'AMZ1	archaemetzincin-1	Body;Body;Body	941	'AMZ1	archaemetzincin-1
**GA**	cg01471232	4.93E-05	0.009970085	FDR0.01	11	415430	'SIGIRR;ANO9	single Ig IL-1-related receptor;anoctamin-9 isoform 2	TSS1500;Body;Body;TES5000;T	-431	'SIGIRR	single Ig IL-1-related receptor
**GA**	cg01511567	8.16E-06	0.002998866	FDR0.01	11	57103631	'SSRP1;P2RX3	FACT complex subunit SSRP1;P2X purinoceptor 3	TSS1500;TSS5000	-265	'SSRP1	FACT complex subunit SSRP1
**GA**	cg01513913	5.74E-06	0.00233725	FDR0.01	14	106329158	'MIR4507;MIR4538;MIR4537	microRNA 4507;microRNA 4538;microRNA 4537	TSS5000;TSS5000;TSS5000	-3436	'MIR4537	microRNA 4537
**GA**	cg01514357	3.75E-05	0.008422514	FDR0.01	1	2105392	'PRKCZ	protein kinase C zeta type	Body;Body;Body;Body;Body;Bo	9922	'PRKCZ-AS1	PRKCZ antisense RNA 1
**GA**	cg01522095	8.19E-08	0.000104375	BF|FDR0.01	16	23522771	'GGA2	phosphatidylinositol 4-phosphate-binding protein	TSS1500	-956	'GGA2	phosphatidylinositol 4-phosphate-binding protein
**GA**	cg01526748	3.14E-07	0.000287045	FDR0.01	3	191930926	'FGF12	fibroblast growth factor 12	Body;Body	-25492	'FGF12-AS1	FGF12 antisense RNA 1
**GA**	cg01542384	5.29E-06	0.002203321	FDR0.01	3	50284305	'GNAI2	guanine nucleotide-binding protein G(i) subunit alpha-2	Body;Body;Body;Body;Body;TS	-36	'GNAI2	guanine nucleotide-binding protein G(i) subunit alpha-2
**GA**	cg01543603	2.20E-05	0.005949657	FDR0.01	16	89469541	'ANKRD11	ankyrin repeat domain-containing protein 11	Body;Body;Body;Body	-27784	'LOC101927817	uncharacterized LOC101927817
**GA**	cg01546248	9.27E-06	0.003252257	FDR0.01	22	31477006	'SMTN	smoothelin	TSS1500;TSS1500;TSS1500;TSS	-297	'SMTN	smoothelin
**GA**	cg01564818	2.65E-05	0.006704434	FDR0.01	3	9464436	'SETD5	histone-lysine N-methyltransferase SETD5	Body;Body;Body	25138	'SETD5	histone-lysine N-methyltransferase SETD5
**GA**	cg01577187	2.08E-09	7.14E-06	BF|FDR0.01	2	106362832	'NCK2	cytoplasmic protein NCK2	Body;Body	1313	'NCK2	cytoplasmic protein NCK2
**GA**	cg01582937	2.44E-06	0.001269028	FDR0.01	8	22411211	'SORBS3	vinexin	Body	1772	'SORBS3	vinexin
**GA**	cg01589860	1.96E-06	0.001080818	FDR0.01	11	72433836	'ARAP1	arf-GAP with Rho-GAP domain, ANK repeat and PH domain-containing prote	TSS1500;TSS1500;TSS1500;Bod	-433	'ARAP1	arf-GAP with Rho-GAP domain, ANK repeat and PH domain-containing protein 1 isoform d
**GA**	cg01597553	2.21E-05	0.005949657	FDR0.01	18	13219225	'LDLRAD4	low-density lipoprotein receptor class A domain-containing protein 4	Body	467	'LDLRAD4	low-density lipoprotein receptor class A domain-containing protein 4
**GA**	cg01621943	5.16E-10	2.56E-06	BF|FDR0.01	17	53531409	'			-32213	'MMD	monocyte to macrophage differentiation factor
**GA**	cg01633363	5.47E-07	0.000429136	FDR0.01	17	79140589	'AATK;PVALEF	serine/threonine-protein kinase LMTK1 isoform 2;parvalbumin-like EF-hand-	TSS1500;5'UTR	-568	'AATK	serine/threonine-protein kinase LMTK1 isoform 2
**GA**	cg01634388	6.38E-08	8.51E-05	BF|FDR0.01	4	129405035	'LINC02615	long intergenic non-protein coding RNA 2615	Body	55865	'LINC02615	long intergenic non-protein coding RNA 2615
**GA**	cg01636650	3.10E-05	0.007375086	FDR0.01	3	38040315	'VILL	villin-like protein	Body;Body;Body;Body;Body	4687	'VILL	villin-like protein
**GA**	cg01642550	1.20E-06	0.000766279	FDR0.01	16	89098327	'			-54712	'CBFA2T3	protein CBFA2T3
**GA**	cg01652514	2.21E-05	0.005949657	FDR0.01	13	114831142	'RASA3	ras GTPase-activating protein 3	Body;Body;Body	12288	'RASA3	ras GTPase-activating protein 3
**GA**	cg01656856	1.08E-06	0.000712673	FDR0.01	2	170551263	'CCDC173;PHOSPHO2;PHOSPHO2-KLHL23	coiled-coil domain-containing protein 173;pyridoxal phosphate phosphatase	TSS1500;Body;Body;Body;Body	285	'PHOSPHO2	pyridoxal phosphate phosphatase PHOSPHO2
**GA**	cg01670771	4.84E-07	0.000392432	FDR0.01	16	31009232	'STX1B	syntaxin1B	Body	12705	'HSD3B7	3 beta-hydroxysteroid dehydrogenase type 7
**GA**	cg01693598	1.84E-08	3.23E-05	BF|FDR0.01	1	47998637	'			94922	'FOXD2	forkhead box protein D2
**GA**	cg01712428	4.09E-05	0.008847096	FDR0.01	7	73132139	'STX1A	syntaxin-1A	Body;Body	1860	'STX1A	syntaxin-1A
**GA**	cg01756756	1.40E-05	0.004373959	FDR0.01	19	30206951	'C19orf12	protein C19orf12 isoform 1	TSS1500;TSS1500;TSS1500;TSS	-255	'C19orf12	protein C19orf12 isoform 1
**GA**	cg01769341	1.35E-05	0.004253717	FDR0.01	1	3526110	'MEGF6	multiple epidermal growth factor-like domains protein 6 precursor	Body	1962	'MEGF6	multiple epidermal growth factor-like domains protein 6 precursor
**GA**	cg01780352	2.10E-06	0.001143571	FDR0.01	6	168818817	'			-23046	'SMOC2	SPARC-related modular calcium-binding protein 2 precursor
**GA**	cg01782059	2.17E-05	0.005895757	FDR0.01	1	44715942	'ERI3;SNORA110	ERI1 exoribonuclease 3 isoform 2;small nucleolar RNA, H/ACA box 110	Body;Body;Body;Body;Body;TE	2289	'SNORA110	small nucleolar RNA, H/ACA box 110
**GA**	cg01787084	8.25E-12	1.42E-07	BF|FDR0.01	16	87371097	'FBXO31	F-box only protein 31	Body;Body	19983	'LOC101928659	
**GA**	cg01792666	2.11E-09	7.19E-06	BF|FDR0.01	16	30710912	'LOC730183;SRCAP	uncharacterized protein LOC730183;helicase SRCAP	TSS1500;TSS1500;Body	421	'SRCAP	helicase SRCAP
**GA**	cg01802545	5.13E-07	0.000406171	FDR0.01	14	93196432	'LGMN	legumain isoform 1 preproprotein	Body;Body;Body;Body	18540	'LGMN	legumain isoform 1 preproprotein
**GA**	cg01839993	9.14E-07	0.000626791	FDR0.01	10	74034644	'DDIT4	DNA damage-inducible transcript 4 protein	Body	963	'DDIT4	DNA damage-inducible transcript 4 protein
**GA**	cg01851573	8.38E-06	0.003043015	FDR0.01	8	8652454	'MFHAS1	malignant fibrous histiocytoma-amplified sequence 1	Body	93008	'CLDN23	claudin-23
**GA**	cg01876130	5.70E-06	0.002334586	FDR0.01	2	97308580	'KANSL3;FER1L5	KAT8 regulatory NSL complex subunit 3;fer-1-like protein 5	TSS5000;TSS5000;TSS5000;TSS	80	'FER1L5	fer-1-like protein 5
**GA**	cg01883777	1.56E-06	0.00093244	FDR0.01	22	38201848	'H1-0;GCAT	histone H1.0;catalase	1stExon;TSS5000;TSS5000	735	'H1-0	histone H1.0
**GA**	cg01910527	1.21E-06	0.000774998	FDR0.01	17	72245578	'TTYH2	protein tweety homolog 2	Body;Body;Body	1074	'TTYH2	protein tweety homolog 2
**GA**	cg01911395	2.99E-05	0.007230021	FDR0.01	8	55014377	'LYPLA1	acyl-protein thioesterase 1 isoform 3	TSS1500;1stExon;1stExon;1stEx	130	'LYPLA1	acyl-protein thioesterase 1 isoform 3
**GA**	cg01917340	3.71E-05	0.008360353	FDR0.01	14	90720130	'PSMC1	26S proteasome regulatory subunit 4	TSS5000;TSS5000	-2766	'PSMC1	26S proteasome regulatory subunit 4
**GA**	cg01921773	9.83E-06	0.003384855	FDR0.01	16	75661691	'ADAT1;KARS1	tRNA-specific adenosine deaminase 1;lysine--tRNA ligase	TSS5000;TSS5000;TSS5000;TSS	-4512	'ADAT1	tRNA-specific adenosine deaminase 1
**GA**	cg01924435	1.96E-05	0.005527866	FDR0.01	4	184679625	'			-39549	'STOX2	storkhead-box protein 2
**GA**	cg01928418	1.27E-07	0.000144384	FDR0.01	22	41810665	'			32140	'TOB2	protein Tob2
**GA**	cg01947769	5.67E-06	0.002325048	FDR0.01	1	2362403	'			-18393	'PEX10	peroxisome biogenesis factor 10
**GA**	cg01948202	6.62E-06	0.00259145	FDR0.01	3	122400474	'PARP14	poly [ADP-ribose] polymerase 14	Body	789	'PARP14	poly [ADP-ribose] polymerase 14
**GA**	cg01959541	4.22E-05	0.009018635	FDR0.01	6	159291228	'C6orf99		Body;Body;Body;Body;Body;Bo	168	'C6orf99	
**GA**	cg01966277	3.24E-06	0.001546985	FDR0.01	7	82435009	'PCLO	protein piccolo isoform 2	Body	357191	'PCLO	protein piccolo isoform 2
**GA**	cg01973920	1.93E-06	0.001076282	FDR0.01	5	980997	'			16458	'LOC100506688	uncharacterized LOC100506688
**GA**	cg01975495	5.27E-06	0.002197866	FDR0.01	7	100779017	'SERPINE1	plasminogen activator inhibitor 1 precursor	Body	8633	'SERPINE1	plasminogen activator inhibitor 1 precursor
**GA**	cg01979157	6.00E-10	2.82E-06	BF|FDR0.01	1	2161013	'SKI	ski oncogene	1stExon	1256	'SKI	ski oncogene
**GA**	cg01983682	3.52E-05	0.008054969	FDR0.01	2	162283979	'TBR1	T-box brain protein 1	TES5000	11172	'TBR1	T-box brain protein 1
**GA**	cg01989203	3.96E-05	0.008676382	FDR0.01	6	49605296	'RHAG	ammonium transporter Rh type A	TSS1500	-744	'RHAG	ammonium transporter Rh type A

Trait	#cpg	p	fdr	significance	chr	pos.hg19	gene.Illumina	gene.description	locations	dist2TSS	gene.nearest	gene.description
GA	cg01991752	1.55E-05	0.004720445	FDR0.01	14	101014148	'BEGAIN	brain-enriched guanylate kinase-associated protein	Body;Body	20259	'BEGAIN	brain-enriched guanylate kinase-associated protein
GA	cg01992512	4.25E-05	0.009044808	FDR0.01	21	46493156	'SSR4P1;ADARB1	signal sequence receptor subunit 4 pseudogene 1;double-stranded RNA-spe	TSS200;TSS1500;TSS1500;TSS1	-30	'SSR4P1	signal sequence receptor subunit 4 pseudogene 1
GA	cg02010738	1.79E-06	0.001025988	FDR0.01	19	2095466	'MOB3A;IZUMO4	MOB kinase activator 3A;izumo sperm-egg fusion protein 4 precursor	Body;TSS1500;TSS1500;TSS150	859	'MOB3A	MOB kinase activator 3A
GA	cg02056098	8.91E-06	0.003163592	FDR0.01	2	167233372	'SCN9A	sodium channel protein type 9 subunit alpha	TSS1500;TSS1500	-875	'SCN9A	sodium channel protein type 9 subunit alpha
GA	cg02066144	2.92E-07	0.00026901	FDR0.01	2	73052126	'EXOC6B	exocyst complex component 6B	Body;Body;Body;Body;Body;Bo	1036	'EXOC6B	exocyst complex component 6B
GA	cg02066331	9.09E-06	0.003210852	FDR0.01	6	17282778	'RBM24	RNA-binding protein 24	Body;Body;TSS200	-153	'RBM24	RNA-binding protein 24
GA	cg02087416	6.63E-06	0.00259145	FDR0.01	1	200182788	'			160132	'LINC00862	long intergenic non-protein coding RNA 862
GA	cg02097429	1.24E-05	0.004012568	FDR0.01	16	27210995	'KDM8	bifunctional peptidase and arginyl-hydroxylase JMJD5 isoform 1	TSS5000;TSS5000	-3851	'KDM8	bifunctional peptidase and arginyl-hydroxylase JMJD5 isoform 1
GA	cg02098619	3.68E-05	0.008324036	FDR0.01	1	8689772	'RERE	arginine-glutamic acid dipeptide repeats protein	Body;Body	-134799	'SNORD128	small nucleolar RNA, C/D box 128
GA	cg02104962	1.67E-05	0.004944599	FDR0.01	6	31548314	'TNF;LTB	tumor necrosis factor;lymphotoxin-beta	TES5000;TES5000;TES5000	1887	'LTB	lymphotoxin-beta
GA	cg02109361	2.95E-05	0.007183669	FDR0.01	11	64782294	'ARL2-SNX15;ARL2;MIR6879	ARL2-SNX15 readthrough (NMD candidate);ADP-ribosylation factor-like prot	Body;Body;Body;TSS5000	663	'ARL2	ADP-ribosylation factor-like protein 2
GA	cg02123534	5.63E-09	1.44E-05	BF|FDR0.01	7	965485	'ADAP1	arf-GAP with dual PH domain-containing protein 1	TSS5000;Body;Body;Body;Body	1367	'ADAP1	arf-GAP with dual PH domain-containing protein 1
GA	cg02149899	1.14E-06	0.000738203	FDR0.01	11	75993521	'			-75975	'WNT11	protein Wnt-11
GA	cg02152631	4.20E-05	0.008991677	FDR0.01	8	29133647	'			-13060	'KIF13B	kinesin-like protein KIF13B
GA	cg02154252	3.89E-05	0.008607033	FDR0.01	1	79472532	'ADGRL4	adhesion G protein-coupled receptor L4 precursor	TSS200	-117	'ADGRL4	adhesion G protein-coupled receptor L4 precursor
GA	cg02165095	3.85E-05	0.008550352	FDR0.01	10	17428870	'ST8SIA6;ST8SIA6-AS1	alpha-2,8-sialyltransferase 8F precursor;ST8SIA6 antisense RNA 1	Body;Body;Body;TSS200	-64	'ST8SIA6-AS1	ST8SIA6 antisense RNA 1
GA	cg02194129	2.68E-05	0.006747113	FDR0.01	14	104171313	'KLC1;XRCC3	kinesin light chain 1;DNA repair protein XRCC3	TES5000;TES5000;Body;Body;B	10475	'XRCC3	DNA repair protein XRCC3
GA	cg02196592	2.90E-06	0.001429017	FDR0.01	16	85209260	'			-32141	'MIR12128	microRNA 12128
GA	cg02197608	4.94E-07	0.000396481	FDR0.01	2	129101883	'			-25441	'HS6ST1	heparan-sulfate 6-O-sulfotransferase 1
GA	cg02205391	5.72E-06	0.002336898	FDR0.01	6	31762409	'VARS1;LSM2	valine--tRNA ligase;U6 snRNA-associated Sm-like protein LSm2	Body;TES5000	1120	'VARS1	valine--tRNA ligase
GA	cg02205445	2.93E-06	0.001437694	FDR0.01	2	144648543	'			-46090	'LOC101928386	uncharacterized LOC101928386
GA	cg02208313	2.48E-05	0.006423894	FDR0.01	17	80085332	'CCDC57	coiled-coil domain-containing protein 57 isoform 1	Body;Body	-29220	'FASN	fatty acid synthase
GA	cg02218324	6.93E-06	0.002664953	FDR0.01	19	46318439	'RSPH6A;SYMPK	radial spoke head protein 6 homolog A;symplekin	5'UTR;TES5000	127	'RSPH6A	radial spoke head protein 6 homolog A
GA	cg02218776	2.14E-05	0.005860161	FDR0.01	2	100814213	'			-10502	'LINC01104	long intergenic non-protein coding RNA 1104
GA	cg02220365	4.13E-05	0.008895517	FDR0.01	11	67211717	'CORO1B	coronin-1B	TSS1500;TSS1500	-425	'CORO1B	coronin-1B
GA	cg02236679	3.61E-05	0.008201409	FDR0.01	16	78782477	'WWOX	WW domain-containing oxidoreductase	Body;Body	648927	'WWOX	WW domain-containing oxidoreductase
GA	cg02244386	4.75E-07	0.000387098	FDR0.01	2	165473994	'GRB14	growth factor receptor-bound protein 14 isoform 2	Body	3998	'GRB14	growth factor receptor-bound protein 14 isoform 2
GA	cg02246725	8.44E-06	0.003053789	FDR0.01	11	2014127	'MRPL23-AS1;LINC01219;HOTS;H19;MIR675	MRPL23 antisense RNA 1;long intergenic non-protein coding RNA 1219;;H19	TSS5000;Body;5'UTR;TES5000;T	490	'HOTS	
GA	cg02247430	1.16E-05	0.003814595	FDR0.01	14	53258644	'GNPNAT1	glucosamine 6-phosphate N-acetyltransferase	TSS1500	-319	'GNPNAT1	glucosamine 6-phosphate N-acetyltransferase
GA	cg02254407	1.60E-05	0.004808641	FDR0.01	11	73357159	'PLEKHB1	pleckstrin homology domain-containing family B member 1	TSS1500;TSS1500;TSS5000;TSS	-466	'PLEKHB1	pleckstrin homology domain-containing family B member 1
GA	cg02255871	2.63E-05	0.00667075	FDR0.01	18	46067528	'CTIF	CBP80/20-dependent translation initiation factor isoform 2	Body;Body	2127	'CTIF	CBP80/20-dependent translation initiation factor isoform 2
GA	cg02303801	6.67E-07	0.000498257	FDR0.01	1	3399318	'ARHGEF16	rho guanine nucleotide exchange factor 16	TES5000	28090	'ARHGEF16	rho guanine nucleotide exchange factor 16
GA	cg02306995	8.56E-06	0.003090506	FDR0.01	3	122635049	'SEMA5B	semaphorin-5B isoform 2	Body;Body;Body;Body;Body	29690	'LINC02035	long intergenic non-protein coding RNA 2035
GA	cg02311364	3.26E-05	0.00760708	FDR0.01	3	13001256	'IQSEC1	IQ motif and SEC7 domain-containing protein 1	Body;Body;Body	7974	'IQSEC1	IQ motif and SEC7 domain-containing protein 1
GA	cg02320474	1.79E-05	0.005227922	FDR0.01	16	1758022	'MAPK8IP3	C-Jun-amino-terminal kinase-interacting protein 3	Body;Body;Body	1827	'MAPK8IP3	C-Jun-amino-terminal kinase-interacting protein 3
GA	cg02324006	2.05E-09	7.12E-06	BF|FDR0.01	19	1080034	'ARHGAP45	rho GTPase-activating protein 45	Body;Body;Body;Body;Body	3402	'ARHGAP45	rho GTPase-activating protein 45
GA	cg02324920	4.80E-05	0.009797842	FDR0.01	10	105253152	'NEURL1-AS1;NEURL1	NEURL1 antisense RNA 1;E3 ubiquitin-protein ligase NEURL1	Body;TSS1500	-309	'NEURL1	E3 ubiquitin-protein ligase NEURL1
GA	cg02369725	2.42E-05	0.006316368	FDR0.01	2	178936951	'PDE11A	dual 3',5'-cyclic-AMP and -GMP phosphodiesterase 11A	1stExon;Body	553	'PDE11A	dual 3',5'-cyclic-AMP and -GMP phosphodiesterase 11A
GA	cg02375166	3.47E-08	5.37E-05	BF|FDR0.01	11	58291656	'LPXN	leupaxin	TES5000;TES5000;TES5000	-16078	'OR5B21	olfactory receptor 5B21
GA	cg02384857	4.58E-05	0.009497988	FDR0.01	7	27197614	'HOXA-AS3;HOXA7;HOXA9;HOXA10-HOXA9	HOXA cluster antisense RNA 3;homeobox protein Hox-A7;homeobox protein	TES5000;TES5000;TSS1500;TES	-1320	'HOXA7	homeobox protein Hox-A7
GA	cg02424569	4.27E-05	0.009067438	FDR0.01	3	14851410	'LINC02011	long intergenic non-protein coding RNA 2011	Body	1511	'LINC02011	long intergenic non-protein coding RNA 2011
GA	cg02430430	1.29E-06	0.000813343	FDR0.01	15	74532450	'CCDC33	coiled-coil domain-containing protein 33 isoform 3	Body	3784	'CCDC33	coiled-coil domain-containing protein 33 isoform 3
GA	cg02431732	9.81E-06	0.003383942	FDR0.01	22	42907591	'SERHL;RRP7A	serine hydrolase-like protein 2;ribosomal RNA-processing protein 7 homolog	Body;3'UTR	8211	'RRP7A	ribosomal RNA-processing protein 7 homolog A
GA	cg02449461	1.58E-06	0.000943112	FDR0.01	11	75061838	'ARRB1	beta-arrestin-1	Body;Body	867	'ARRB1	beta-arrestin-1
GA	cg02468154	1.38E-05	0.004338373	FDR0.01	11	43755634	'HSD17B12	very-long-chain 3-oxoacyl-CoA reductase	Body	53347	'HSD17B12	very-long-chain 3-oxoacyl-CoA reductase
GA	cg02483701	3.92E-05	0.008645923	FDR0.01	7	27215610	'HOXA10-HOXA9;HOXA10-AS;HOXA10	HOXA10-HOXA9 readthrough;HOXA10 antisense RNA;homeobox protein Ho	Body;TES5000;TSS5000;Body	-1671	'HOXA10	homeobox protein Hox-A10 isoform 2
GA	cg02516101	7.10E-07	0.000521103	FDR0.01	22	38711891	'CSNK1E;TPTEP2-CSNK1E	casein kinase I;casein kinase I isoform epsilon	Body;Body;Body	1547	'CSNK1E	casein kinase I
GA	cg02522196	4.29E-11	4.44E-07	BF|FDR0.01	12	44226393	'TMEM117	transmembrane protein 117	TSS5000;TSS5000;TSS5000;TSS	-3270	'TMEM117	transmembrane protein 117
GA	cg02538135	4.56E-05	0.009466284	FDR0.01	19	2200125	'DOT1L	histone-lysine N-methyltransferase, H3 lysine-79 specific	Body	34023	'MIR1227	microRNA 1227
GA	cg02544658	1.26E-06	0.00079771	FDR0.01	21	44876604	'LINC00319	long intergenic non-protein coding RNA 319	TES5000	10124	'LINC00319	long intergenic non-protein coding RNA 319
GA	cg02555807	4.06E-05	0.008802751	FDR0.01	15	40615100	'INAFM2	putative transmembrane protein INAFM2	TSS1500	-792	'INAFM2	putative transmembrane protein INAFM2
GA	cg02557652	1.81E-05	0.005259897	FDR0.01	15	89741016	'ABHD2	monoacylglycerol lipase ABHD2	3'UTR;3'UTR	23794	'RLBP1	retinaldehyde-binding protein 1
GA	cg02558026	6.55E-06	0.002576091	FDR0.01	16	30904573	'BCL7C;MIR762HG;MIR762;CTF1	B-cell CLL/lymphoma 7 protein family member C isoform 2;MIR762 host gen	Body;Body;Body;Body;Body;TS	-650	'MIR762	microRNA 762
GA	cg02567488	5.80E-07	0.000448787	FDR0.01	6	32822565	'TAP1;PSMB9	antigen peptide transporter 1;proteasome subunit beta type-9	TSS5000;TSS1500;Body	597	'PSMB9	proteasome subunit beta type-9
GA	cg02567958	2.03E-12	5.35E-08	BF|FDR0.01	22	37962818	'CDC42EP1;LGALS2	cdc42 effector protein 1;galectin-2	1stExon;TES5000	6332	'CDC42EP1	cdc42 effector protein 1
GA	cg02568531	2.31E-05	0.006126008	FDR0.01	11	820843	'PNPLA2	patatin-like phospholipase domain-containing protein 2	Body	1930	'PNPLA2	patatin-like phospholipase domain-containing protein 2
GA	cg02569458	2.84E-06	0.001408963	FDR0.01	12	86230093	'RASSF9	ras association domain-containing protein 9	5'UTR	94	'RASSF9	ras association domain-containing protein 9
GA	cg02574734	3.70E-05	0.008338881	FDR0.01	1	116369351	'			13973	'NHLH2	helix-loop-helix protein 2
GA	cg02583484	4.21E-08	6.13E-05	BF|FDR0.01	12	54677008	'CBX5;SCAT2;HNRNPA1	chromobox protein homolog 5;S-phase cancer associated transcript 2;hetero	TSS5000;TES5000;Body;Body;B	2499	'HNRNPA1	heterogeneous nuclear ribonucleoprotein A1
GA	cg02584432	4.65E-05	0.009586929	FDR0.01	5	138740366	'SPATA24	spermatogenesis-associated protein 24	TSS1500	-590	'SPATA24	spermatogenesis-associated protein 24
GA	cg02589672	1.40E-05	0.004373959	FDR0.01	22	41763128	'TEF	transcription factor VBP	TSS1500	-208	'TEF	transcription factor VBP
GA	cg02627216	4.97E-07	0.000397526	FDR0.01	17	38599056	'IGFBP4	insulin-like growth factor-binding protein 4 precursor	TSS1500	-645	'IGFBP4	insulin-like growth factor-binding protein 4 precursor
GA	cg02637101	3.26E-06	0.001550582	FDR0.01	15	99548944	'PGPEP1L	pyroglutamyl-peptidase 1-like protein isoform 1	Body;TSS200	-59	'PGPEP1L	pyroglutamyl-peptidase 1-like protein isoform 1
GA	cg02647520	1.09E-06	0.000715501	FDR0.01	6	33173218	'RXRB;SLC39A7;HSD17B8;MIR219A1;RING1	retinoic acid receptor RXR-beta;zinc transporter SLC39A7 precursor;estradiol	TSS5000;TSS5000;TSS5000;TES	787	'HSD17B8	estradiol 17-beta-dehydrogenase 8
GA	cg02647874	1.25E-08	2.51E-05	BF|FDR0.01	11	47380751	'SPI1	Spi1p	Body;Body	-6498	'MYBPC3	myosin-binding protein C, cardiac-type
GA	cg02657830	1.24E-06	0.000786351	FDR0.01	1	27152905	'ZDHHC18	palmitoyltransferase ZDHHC18	TSS1500	-273	'ZDHHC18	palmitoyltransferase ZDHHC18
GA	cg02677609	2.37E-05	0.00622641	FDR0.01	4	154125439	'TRIM2	tripartite motif-containing protein 2	Body;Body;TSS200;TSS200;TSS2	-126	'TRIM2	tripartite motif-containing protein 2
GA	cg02696253	2.15E-05	0.005862662	FDR0.01	6	106964589	'CRYBG1	beta/gamma crystallin domain-containing protein 1 isoform 2	Body;Body	4860	'CRYBG1	beta/gamma crystallin domain-containing protein 1 isoform 2
GA	cg02699829	4.44E-05	0.009287489	FDR0.01	19	13216355	'LYL1;TRMT1	protein lyl-1;tRNA (guanine(26)-N(2))-dimethyltransferase	TSS5000;Body;Body;Body;Body	-2683	'LYL1	protein lyl-1
GA	cg02711886	3.03E-06	0.001470736	FDR0.01	5	177762428	'COL23A1	collagen alpha-1(XXIII) chain	Body	-102605	'PHYKPL	5-phosphohydroxy-L-lysine phospho-lyase
GA	cg02753354	1.63E-08	3.03E-05	BF|FDR0.01	19	1074727	'ARHGAP45	rho GTPase-activating protein 45	Body;Body;Body;Body;TSS5000	-1905	'ARHGAP45	rho GTPase-activating protein 45
GA	cg02796390	5.09E-08	7.07E-05	BF|FDR0.01	20	3726190	'HSPA12B	heat shock 70 kDa protein 12B	Body;Body;Body	12859	'HSPA12B	heat shock 70 kDa protein 12B
GA	cg02798280	3.29E-05	0.00765505	FDR0.01	19	39087135	'MAP4K1;LOC105372397	mitogen-activated protein kinase kinase kinase kinase 1;	Body;Body;Body	149	'LOC105372397	
GA	cg02809401	3.50E-06	0.001628968	FDR0.01	11	65326208	'LTBP3	latent-transforming growth factor beta-binding protein 3 precursor	TSS1500;TSS1500;TSS1500	-509	'LTBP3	latent-transforming growth factor beta-binding protein 3 precursor
GA	cg02825765	2.55E-05	0.006538551	FDR0.01	1	225614261	'LBR	delta(14)-sterol reductase LBR	Body;Body	1479	'LBR	delta(14)-sterol reductase LBR
GA	cg02835421	7.64E-08	9.87E-05	BF|FDR0.01	1	44411569	'IPO13	importin-13	TSS1500	-1052	'IPO13	importin-13
GA	cg02859421	1.67E-07	0.000175532	FDR0.01	19	3465071	'NFIC	nuclear factor 1 C-type	3'UTR;3'UTR;3'UTR;3'UTR;3'UT	15452	'SMIM24	small integral membrane protein 24 precursor
GA	cg02871985	3.94E-05	0.008659885	FDR0.01	2	43398154	'			55591	'ZFP36L2	zinc finger protein 36, C3H1 type-like 2
GA	cg02879960	1.75E-06	0.001008635	FDR0.01	22	50483545	'TTLL8	protein monoglycylase TTLL8	Body	13111	'TTLL8	protein monoglycylase TTLL8
GA	cg02905245	4.39E-05	0.00923681	FDR0.01	3	112359652	'CCDC80	coiled-coil domain-containing protein 80 precursor	5'UTR;Body	338	'CCDC80	coiled-coil domain-containing protein 80 precursor
GA	cg02921623	7.58E-09	1.82E-05	BF|FDR0.01	10	105250537	'NEURL1-AS1;NEURL1	NEURL1 antisense RNA 1;E3 ubiquitin-protein ligase NEURL1	Body;TSS5000	-2924	'NEURL1	E3 ubiquitin-protein ligase NEURL1
GA	cg02932364	4.01E-05	0.00874487	FDR0.01	1	227981671	'			-21746	'PRSS38	serine protease 38 isoform 1 precursor
GA	cg02940661	3.00E-05	0.007230021	FDR0.01	14	106382755	'FAM30A	family with sequence similarity 30 member A	TSS1500	-1082	'FAM30A	family with sequence similarity 30 member A
GA	cg02956248	1.92E-07	0.000195823	FDR0.01	6	32120901	'PRRT1;LOC100507547;PPT2;PPT2-EGFL8	proline-rich transmembrane protein 1;uncharacterized LOC100507547;lysos	TSS200;TSS1500;Body;Body;Bo	-41	'PRRT1	proline-rich transmembrane protein 1
GA	cg02964434	9.38E-06	0.003282895	FDR0.01	15	31617537	'KLF13	Krueppel-like factor 13	TSS1500;TSS1500	-1500	'KLF13	Krueppel-like factor 13
GA	cg02971262	1.08E-06	0.000712673	FDR0.01	9	139925395	'ABCA2;C9orf139;FUT7	ATP-binding cassette sub-family A member 2 isoform b;uncharacterized prot	TSS5000;TSS5000;Body;Body	1145	'FUT7	alpha-(1,3)-fucosyltransferase 7
GA	cg02974085	6.02E-06	0.002426612	FDR0.01	1	222990921	'DISP1	protein dispatched homolog 1	Body;Body	2541	'DISP1	protein dispatched homolog 1
GA	cg02979520	7.73E-06	0.002875279	FDR0.01	3	48471575	'PLXNB1;CCDC51	plexin-B1 precursor;mitochondrial potassium channel isoform 2	TSS1500;TSS200;TES5000;TES5	-80	'PLXNB1	plexin-B1 precursor

Trait	#cpg	p	fdr	significance	chr	pos.hg19	gene.Illumina	gene.description	locations	dist2TSS	gene.nearest	gene.description
**GA**	cg02980249	4.25E-05	0.009044808	FDR0.01	6	31760762	'VARS1;LSM2	valine--tRNA ligase;U6 snRNA-associated Sm-like protein LSm2	Body;TES5000	2767	'VARS1	valine--tRNA ligase
**GA**	cg02988730	6.29E-08	8.43E-05	BF|FDR0.01	1	110167509	'AMPD2	AMP deaminase 2	Body;Body;Body;Body;Body	900	'AMPD2	AMP deaminase 2
**GA**	cg02988947	1.14E-05	0.003766432	FDR0.01	17	61778813	'LIMD2;LOC729683;STRADA	LIM domain-containing protein 2;uncharacterized LOC729683;STE20-related	TSS1500;Body;TES5000;TES500	1116	'LOC729683	uncharacterized LOC729683
**GA**	cg02995045	1.83E-07	0.000189199	FDR0.01	1	6419906	'ACOT7	cytosolic acyl coenzyme A thioester hydrolase	TSS1500;Body;Body;Body	868	'ACOT7	cytosolic acyl coenzyme A thioester hydrolase
**GA**	cg03008387	1.27E-08	2.53E-05	BF|FDR0.01	20	62089152	'KCNQ2	potassium voltage-gated channel subfamily KQT member 2 isoform b	Body;Body;Body;Body;Body	14856	'KCNQ2	potassium voltage-gated channel subfamily KQT member 2 isoform b
**GA**	cg03012289	1.23E-05	0.004009397	FDR0.01	8	110374374	'PKHD1L1	fibrocystin-L precursor	TSS1500	-315	'PKHD1L1	fibrocystin-L precursor
**GA**	cg03027750	1.37E-08	2.69E-05	BF|FDR0.01	13	77899183	'MYCBP2	E3 ubiquitin-protein ligase MYCBP2	Body	2046	'MYCBP2	E3 ubiquitin-protein ligase MYCBP2
**GA**	cg03029127	9.94E-07	0.000672726	FDR0.01	16	57770913	'KATNB1	katanin p80 WD40 repeat-containing subunit B1	5'UTR	1232	'KATNB1	katanin p80 WD40 repeat-containing subunit B1
**GA**	cg03030757	8.23E-06	0.003015634	FDR0.01	6	35420754	'FANCE	Fanconi anemia group E protein	Body	640	'FANCE	Fanconi anemia group E protein
**GA**	cg03035746	1.52E-05	0.004648235	FDR0.01	2	74727286	'LBX2;LBX2-AS1;PCGF1	transcription factor LBX2;LBX2 antisense RNA 1;polycomb group RING finger	TSS1500;Body;TSS5000;TSS500	-556	'LBX2	transcription factor LBX2
**GA**	cg03036210	2.10E-05	0.005782308	FDR0.01	16	89904091	'SPIRE2	protein spire homolog 2	Body	9209	'SPIRE2	protein spire homolog 2
**GA**	cg03047995	1.31E-09	5.09E-06	BF|FDR0.01	14	24601588	'FITM1;PSME1	fat storage-inducing transmembrane protein 1;proteasome activator comple	Body;TSS5000;TSS5000;TSS500	914	'FITM1	fat storage-inducing transmembrane protein 1
**GA**	cg03048432	1.84E-05	0.005308068	FDR0.01	14	51290751	'NIN;LOC105370489	ninein isoform 5;uncharacterized LOC105370489	Body;Body;Body;Body;TES5000	2237	'LOC105370489	uncharacterized LOC105370489
**GA**	cg03050022	4.38E-06	0.00191161	FDR0.01	15	55559759	'RAB27A	ras-related protein Rab-27A	Body;Body;Body;Body	2725	'RAB27A	ras-related protein Rab-27A
**GA**	cg03054026	4.05E-07	0.000347154	FDR0.01	2	242808907	'RTP5	receptor-transporting protein 5	TSS5000	-2973	'RTP5	receptor-transporting protein 5
**GA**	cg03064100	3.46E-07	0.000309606	FDR0.01	2	46720801	'			14098	'TMEM247	transmembrane protein 247
**GA**	cg03074244	4.57E-05	0.009477579	FDR0.01	11	118213414	'CD3D;CD3G	T-cell surface glycoprotein CD3 delta chain precursor;T-cell surface glycoprot	5'UTR;5'UTR;TSS5000	6	'CD3D	T-cell surface glycoprotein CD3 delta chain precursor
**GA**	cg03098721	8.89E-07	0.00061473	FDR0.01	1	84464084	'TTLL7	tubulin polyglutamylase TTLL7	Body;Body;Body	731	'TTLL7	tubulin polyglutamylase TTLL7
**GA**	cg03108070	5.12E-09	1.38E-05	BF|FDR0.01	6	33245490	'RPS18;B3GALT4;WDR46	40S ribosomal protein S18;beta-1,3-galactosyltransferase 4;WD repeat-conta	TES5000;1stExon;TES5000;TES5	591	'B3GALT4	beta-1,3-galactosyltransferase 4
**GA**	cg03144232	7.07E-07	0.000520965	FDR0.01	16	31022351	'STX1B	syntaxin1B	TSS1500	-392	'STX1B	syntaxin1B
**GA**	cg03144619	6.62E-06	0.00259145	FDR0.01	1	230415668	'GALNT2	polypeptide N-acetylgalactosaminyltransferase 2 precursor	3'UTR;3'UTR	146410	'PGBD5	piggyBac transposable element-derived protein 5
**GA**	cg03149565	4.00E-05	0.008741223	FDR0.01	4	109092769	'LEF1;LEF1-AS1	lymphoid enhancer-binding factor 1;LEF1 antisense RNA 1	TSS5000;TSS5000;TSS5000;TSS5	-506	'LEF1-AS1	LEF1 antisense RNA 1
**GA**	cg03189210	3.88E-05	0.008593877	FDR0.01	6	33245474	'RPS18;B3GALT4;WDR46	40S ribosomal protein S18;beta-1,3-galactosyltransferase 4;WD repeat-conta	TES5000;1stExon;TES5000;TES5	575	'B3GALT4	beta-1,3-galactosyltransferase 4
**GA**	cg03217227	5.07E-06	0.002128915	FDR0.01	1	249119553	'SH3BP5L;MIR3124	SH3 domain-binding protein 5-like;microRNA 3124	5'UTR;5'UTR;5'UTR;5'UTR;TSS1	12	'SH3BP5L	SH3 domain-binding protein 5-like
**GA**	cg03220447	3.26E-08	5.15E-05	BF|FDR0.01	11	19745293	'NAV2	neuron navigator 2 isoform 3	Body;Body;Body;Body	-9075	'LOC100126784	
**GA**	cg03227037	1.90E-06	0.001066227	FDR0.01	20	62212228	'			-6636	'HELZ2	helicase with zinc finger domain 2 isoform 2
**GA**	cg03271893	2.64E-07	0.000249773	FDR0.01	10	102760970	'LZTS2	leucine zipper putative tumor suppressor 2	Body;Body;Body;TSS1500	-1283	'LZTS2	leucine zipper putative tumor suppressor 2
**GA**	cg03297901	9.98E-06	0.003420038	FDR0.01	17	76117767	'TMC6	transmembrane channel-like protein 6	Body;Body;Body	7108	'TMC6	transmembrane channel-like protein 6
**GA**	cg03306368	7.67E-06	0.002863133	FDR0.01	6	99396091	'FBXL4	F-box/LRR-repeat protein 4	TSS1500;TSS1500;TSS1500;TSS1	-209	'FBXL4	F-box/LRR-repeat protein 4
**GA**	cg03318940	2.14E-07	0.000212682	FDR0.01	3	182817448	'MCCC1	methylcrotonoyl-CoA carboxylase subunit alpha, mitochondrial	TSS200;TSS200;TSS200;TSS150	-73	'MCCC1	methylcrotonoyl-CoA carboxylase subunit alpha, mitochondrial
**GA**	cg03320827	3.54E-05	0.008103881	FDR0.01	6	10887367	'SYCP2L	synaptonemal complex protein 2-like	1stExon	83	'SYCP2L	synaptonemal complex protein 2-like
**GA**	cg03372852	9.98E-07	0.000674333	FDR0.01	19	55219964	'LILRP2	leukocyte immunoglobulin-like receptor pseudogene 2	Body	265	'LILRP2	leukocyte immunoglobulin-like receptor pseudogene 2
**GA**	cg03390636	4.74E-07	0.000386799	FDR0.01	15	80843452	'ARNT2;LOC101929586	aryl hydrocarbon receptor nuclear translocator 2;uncharacterized LOC10192	Body;TES5000	11833	'LOC101929586	uncharacterized LOC101929586
**GA**	cg03426888	2.87E-06	0.001417448	FDR0.01	6	32156527	'AGER;PBX2;GPSM3	advanced glycosylation end product-specific receptor precursor;pre-B-cell le	TSS5000;TSS5000;TSS5000;TSS5	1452	'PBX2	pre-B-cell leukemia transcription factor 2
**GA**	cg03457485	4.82E-05	0.009828595	FDR0.01	11	63753251	'OTUB1	ubiquitin thioesterase OTUB1	TSS200;TSS200	-73	'OTUB1	ubiquitin thioesterase OTUB1
**GA**	cg03466326	5.34E-08	7.39E-05	BF|FDR0.01	1	205270559	'NUAK2	NUAK family SNF1-like kinase 2	TES5000	20314	'NUAK2	NUAK family SNF1-like kinase 2
**GA**	cg03467001	1.26E-05	0.004064627	FDR0.01	1	183857649	'RGL1	ral guanine nucleotide dissociation stimulator-like 1	Body;Body;Body;Body;Body	83396	'RGL1	ral guanine nucleotide dissociation stimulator-like 1
**GA**	cg03526142	4.70E-06	0.002017103	FDR0.01	17	33864734	'SLFN12L	schlafen family member 12-like	5'UTR	146	'SLFN12L	schlafen family member 12-like
**GA**	cg03536289	3.10E-05	0.007374901	FDR0.01	14	94443120	'ASB2	ankyrin repeat and SOCS box protein 2 isoform 1	TSS1500	-204	'ASB2	ankyrin repeat and SOCS box protein 2 isoform 1
**GA**	cg03544499	1.42E-06	0.000868253	FDR0.01	9	85677016	'RASEF	ras and EF-hand domain-containing protein	Body	1041	'RASEF	ras and EF-hand domain-containing protein
**GA**	cg03559682	2.30E-05	0.006112021	FDR0.01	11	6439864	'APBB1	amyloid-beta A4 precursor protein-binding family B member 1	Body;Body;Body	460	'APBB1	amyloid-beta A4 precursor protein-binding family B member 1
**GA**	cg03559915	2.51E-06	0.001292894	FDR0.01	11	67201998	'RPS6KB2;PTPRCAP;CORO1B	ribosomal protein S6 kinase beta-2;protein tyrosine phosphatase receptor ty	Body;TES5000;TES5000;TES500	3155	'PTPRCAP	protein tyrosine phosphatase receptor type C-associated protein precursor
**GA**	cg03566174	8.00E-09	1.86E-05	BF|FDR0.01	2	95709308	'MAL	myelin and lymphocyte protein	Body;Body;Body;Body	17856	'MAL	myelin and lymphocyte protein
**GA**	cg03597243	5.89E-10	2.82E-06	BF|FDR0.01	6	31628156	'APOM;C6orf47;GPANK1	apolipoprotein M precursor;uncharacterized protein C6orf47;G patch domai	TES5000;TES5000;TES5000;Bod	399	'C6orf47	uncharacterized protein C6orf47
**GA**	cg03605420	3.17E-05	0.007461894	FDR0.01	5	74162809	'FAM169A;LOC441086	soluble lamin-associated protein of 75 kDa;uncharacterized LOC441086	TSS200;TSS1500;Body	-18	'FAM169A	soluble lamin-associated protein of 75 kDa
**GA**	cg03634833	3.06E-05	0.007320937	FDR0.01	7	965534	'ADAP1	arf-GAP with dual PH domain-containing protein 1	Body;Body;Body;Body	1318	'ADAP1	arf-GAP with dual PH domain-containing protein 1
**GA**	cg03639897	1.42E-05	0.004430725	FDR0.01	20	897545	'ANGPT4	angiopoietin-4 precursor	TSS1500;TSS1500	-568	'ANGPT4	angiopoietin-4 precursor
**GA**	cg03640051	3.27E-09	9.56E-06	BF|FDR0.01	4	42657689	'ATP8A1	phospholipid-transporting ATPase IA	Body;Body	1433	'ATP8A1	phospholipid-transporting ATPase IA
**GA**	cg03654783	1.22E-07	0.000140496	FDR0.01	1	156634034	'BCAN;NES	brevican core protein precursor;nestin	TES5000;TES5000	13165	'NES	nestin
**GA**	cg03655701	6.99E-07	0.00051856	FDR0.01	1	24885664	'NCMAP-DT;NCMAP	NCMAP divergent transcript;noncompact myelin-associated protein	TSS5000;Body	3087	'NCMAP	noncompact myelin-associated protein
**GA**	cg03663556	2.35E-06	0.001244039	FDR0.01	7	50900556	'			-39406	'GRB10	growth factor receptor-bound protein 10
**GA**	cg03666441	3.96E-08	5.86E-05	BF|FDR0.01	17	7254671	'ACAP1;KCTD11;TMEM95	arf-GAP with coiled-coil, ANK repeat and PH domain-containing protein 1;BT	Body;TSS1500;TSS1500;TSS500	-536	'KCTD11	BTB/POZ domain-containing protein KCTD11
**GA**	cg03689048	3.25E-06	0.001550582	FDR0.01	6	31619544	'BAG6;APOM	large proline-rich protein BAG6;apolipoprotein M precursor	5'UTR;5'UTR;5'UTR;5'UTR;5'UT	623	'BAG6	large proline-rich protein BAG6
**GA**	cg03699459	2.88E-07	0.000266516	FDR0.01	10	4864636	'AKR1E2	1,5-anhydro-D-fructose reductase	TSS5000;TSS5000;TSS5000;TSS5	-3732	'AKR1E2	1,5-anhydro-D-fructose reductase
**GA**	cg03703887	1.91E-06	0.001070173	FDR0.01	11	34273111	'ABTB2	ankyrin repeat and BTB/POZ domain-containing protein 2	Body	106446	'ABTB2	ankyrin repeat and BTB/POZ domain-containing protein 2
**GA**	cg03706951	7.37E-11	6.46E-07	BF|FDR0.01	17	39465518	'KRTAP16-1	keratin-associated protein 16-1	TSS200	-13	'KRTAP16-1	keratin-associated protein 16-1
**GA**	cg03726147	8.85E-06	0.003154017	FDR0.01	1	44686022	'DMAP1;ERI3;ERI3-IT1	DNA methyltransferase 1-associated protein 1;ERI1 exoribonuclease 3 isofor	Body;Body;Body;TES5000;TES5	6880	'DMAP1	DNA methyltransferase 1-associated protein 1
**GA**	cg03729251	1.06E-06	0.000704611	FDR0.01	4	151501035	'LRBA;LOC729558;MAB21L2	lipopolysaccharide-responsive and beige-like anchor protein isoform 4;uncha	Body;Body;Body;Body;Body;TS	795	'LOC729558	uncharacterized LOC729558
**GA**	cg03731647	2.26E-06	0.001213193	FDR0.01	16	1598953	'IFT140;TMEM204	intraflagellar transport protein 140 homolog;transmembrane protein 204 pr	Body;Body;Body	15287	'TMEM204	transmembrane protein 204 precursor
**GA**	cg03747028	1.08E-06	0.000712673	FDR0.01	1	28970259	'TAF12;LINC01715;RNU11	transcription initiation factor TFIID subunit 12;long intergenic non-protein co	TSS1500;TSS1500;Body;Body;T	492	'LINC01715	long intergenic non-protein coding RNA 1715
**GA**	cg03751906	2.99E-05	0.007229417	FDR0.01	12	129307800	'SLC15A4	solute carrier family 15 member 4	Body	703	'SLC15A4	solute carrier family 15 member 4
**GA**	cg03760457	2.23E-06	0.001205744	FDR0.01	15	70326230	'			45577	'MIR629	microRNA 629
**GA**	cg03773636	3.64E-05	0.008259571	FDR0.01	3	192769633	'			-133988	'MB21D2	protein MB21D2
**GA**	cg03778207	2.36E-05	0.006212516	FDR0.01	11	86230664	'ME3	NADP-dependent malic enzyme, mitochondrial	Body;Body;Body;Body;Body;Bo	144736	'CCDC81	coiled-coil domain-containing protein 81 isoform 2
**GA**	cg03790236	1.08E-08	2.29E-05	BF|FDR0.01	11	67057248	'GRK2;ANKRD13D	beta-adrenergic receptor kinase 1;ankyrin repeat domain-containing protein	TES5000;Body;Body	475	'ANKRD13D	ankyrin repeat domain-containing protein 13D
**GA**	cg03797115	2.18E-07	0.000216209	FDR0.01	4	110625010	'CASP6	caspase-6	TSS1500;TSS1500;TSS1500	-409	'CASP6	caspase-6
**GA**	cg03798942	2.69E-06	0.001343516	FDR0.01	11	128566958	'FLI1;SENCR	Friend leukemia integration 1 transcription factor;smooth muscle and endot	Body;TSS1500;Body;Body;Body	-1040	'SENCR	smooth muscle and endothelial cell enriched migration/differentiation-associated lncRNA
**GA**	cg03822873	4.91E-06	0.002078476	FDR0.01	7	128899554	'AHCYL2	adenosylhomocysteinase 3 isoform d	Body;Body	34684	'AHCYL2	adenosylhomocysteinase 3 isoform d
**GA**	cg03848580	3.84E-08	5.78E-05	BF|FDR0.01	17	76113713	'TMC6	transmembrane channel-like protein 6	Body;Body;Body	-5833	'TNRC6C-AS1	
**GA**	cg03883256	4.51E-06	0.001954883	FDR0.01	11	119235802	'USP2	ubiquitin carboxyl-terminal hydrolase 2	TSS1500;Body;Body	-884	'USP2	ubiquitin carboxyl-terminal hydrolase 2
**GA**	cg03886898	1.87E-05	0.00535964	FDR0.01	1	54359132	'YIPF1;DIO1	protein YIPF1;type I iodothyronine deiodinase precursor	TSS5000;TSS5000;TSS5000;TSS5	-727	'DIO1	type I iodothyronine deiodinase precursor
**GA**	cg03946532	4.48E-05	0.009349123	FDR0.01	16	79344736	'			289898	'MAF	transcription factor Maf
**GA**	cg03961824	1.94E-07	0.000196708	FDR0.01	8	82530967	'IMPA1P1	inositol monophosphatase 1 pseudogene 1	Body	12545	'IMPA1P1	inositol monophosphatase 1 pseudogene 1
**GA**	cg03974286	1.07E-10	8.89E-07	BF|FDR0.01	2	201980638	'CFLAR	CASP8 and FADD-like apoptosis regulator	TSS200;TSS200;TSS1500;TSS15	-188	'CFLAR	CASP8 and FADD-like apoptosis regulator
**GA**	cg03996020	7.96E-06	0.002939583	FDR0.01	7	157180124	'DNAJB6	dnaJ homolog subfamily B member 6	TES5000;Body;Body	50427	'DNAJB6	dnaJ homolog subfamily B member 6
**GA**	cg04004830	1.32E-10	9.68E-07	BF|FDR0.01	2	192711243	'CAVIN2	caveolae-associated protein 2	1stExon	571	'CAVIN2	caveolae-associated protein 2
**GA**	cg04005059	4.48E-05	0.009340808	FDR0.01	2	23719838	'KLHL29	kelch-like protein 29	Body	111789	'KLHL29	kelch-like protein 29
**GA**	cg04006457	1.96E-05	0.005527625	FDR0.01	6	30113825	'TRIM40	tripartite motif-containing protein 40	Body;Body	9316	'TRIM40	tripartite motif-containing protein 40
**GA**	cg04011995	4.65E-05	0.009589947	FDR0.01	1	41326317	'CITED4	cbp/p300-interacting transactivator 4	TES5000	1718	'CITED4	cbp/p300-interacting transactivator 4
**GA**	cg04017102	2.70E-05	0.006771634	FDR0.01	5	111405936	'			-90286	'EPB41L4A-AS1	EPB41L4A antisense RNA 1
**GA**	cg04025970	2.13E-07	0.000212477	FDR0.01	8	8727303	'MFHAS1	malignant fibrous histiocytoma-amplified sequence 1	Body	23837	'MFHAS1	malignant fibrous histiocytoma-amplified sequence 1
**GA**	cg04036329	4.44E-05	0.009294057	FDR0.01	17	19771783	'ULK2	serine/threonine-protein kinase ULK2	TSS1500;TSS1500	-534	'ULK2	serine/threonine-protein kinase ULK2
**GA**	cg04039777	2.86E-05	0.007057248	FDR0.01	11	70289959	'			45325	'CTTN	src substrate cortactin
**GA**	cg04039799	3.32E-05	0.00770305	FDR0.01	11	19745484	'NAV2	neuron navigator 2 isoform 3	Body;Body;Body;Body	-9266	'LOC100126784	
**GA**	cg04052832	4.01E-06	0.001817469	FDR0.01	13	111220199	'			-6130	'RAB20	ras-related protein Rab-20
**GA**	cg04055490	4.49E-05	0.009353738	FDR0.01	17	8059012	'PER1;VAMP2	Per1p;vesicle-associated membrane protein 2	TSS5000;TES5000;TES5000	-3290	'PER1	Per1p
**GA**	cg04063345	1.43E-05	0.004440869	FDR0.01	6	152130058	'ESR1	estrogen receptor	Body;Body;Body;Body;Body;Bo	-183	'ESR1	estrogen receptor

Trait	#cpg	p	fdr	significance	chr	pos.hg19	gene.Illumina	gene.description	locations	dist2TSS	gene.nearest	gene.description
GA	cg04071779	3.57E-11	4.22E-07	BF|FDR0.01	7	30517627	'NOD1	nucleotide-binding oligomerization domain-containing protein 1	Body;Body;Body	773	'NOD1	nucleotide-binding oligomerization domain-containing protein 1
GA	cg04072771	1.71E-07	0.000179867	FDR0.01	15	55574912	'RAB27A	ras-related protein Rab-27A	Body	7099	'RAB27A	ras-related protein Rab-27A
GA	cg04088945	2.20E-05	0.005949657	FDR0.01	7	157090146	'			-39551	'DNAJB6	dnaJ homolog subfamily B member 6
GA	cg04091927	7.55E-07	0.000545316	FDR0.01	8	141519270	'CHRAC1	chromatin accessibility complex protein 1	TSS5000;TSS5000;TSS5000	-2150	'CHRAC1	chromatin accessibility complex protein 1
GA	cg04094640	8.67E-06	0.00311387	FDR0.01	16	27237122	'KDM8;NSMCE1	bifunctional peptidase and arginyl-hydroxylase JMJD5 isoform 1;non-structu	TES5000;TES5000;Body	21827	'KDM8	bifunctional peptidase and arginyl-hydroxylase JMJD5 isoform 1
GA	cg04132891	1.76E-05	0.005164987	FDR0.01	3	112351647	'CCDC80	coiled-coil domain-containing protein 80 precursor	Body;Body	8343	'CCDC80	coiled-coil domain-containing protein 80 precursor
GA	cg04134748	6.07E-06	0.002433109	FDR0.01	1	3052175	'PRDM16	histone-lysine N-methyltransferase PRDM16 isoform 2	Body;Body	7637	'MIR4251	microRNA 4251
GA	cg04140525	2.44E-05	0.006352396	FDR0.01	1	153467760	'			-34633	'S100A7	protein S100-A7
GA	cg04145264	1.69E-05	0.004988083	FDR0.01	11	65190825	'NEAT1	nuclear paraspeckle assembly transcript 1	Body;Body	557	'NEAT1	nuclear paraspeckle assembly transcript 1
GA	cg04155026	3.06E-05	0.007320937	FDR0.01	16	67841269	'RANBP10;TSNAXIP1	ran-binding protein 10;translin-associated factor X-interacting protein 1 isofo	TSS1500;TSS1500;TSS1500;TSS1	478	'TSNAXIP1	translin-associated factor X-interacting protein 1 isoform b
GA	cg04160501	7.97E-16	1.19E-10	BF|FDR0.01	7	4723667	'FOXK1	forkhead box protein K1	Body	1742	'FOXK1	forkhead box protein K1
GA	cg04163847	6.21E-08	8.38E-05	BF|FDR0.01	1	59622594	'			-10115	'HSD52	uncharacterized LOC729467
GA	cg04166500	2.97E-05	0.007199919	FDR0.01	14	100571607	'EVL;MIR151B;MIR342	ena/VASP-like protein;microRNA 151b;microRNA 342	Body;Body;TES5000;TSS5000	4244	'MIR151B	microRNA 151b
GA	cg04173586	1.47E-06	0.00088945	FDR0.01	19	2167496	'DOT1L	histone-lysine N-methyltransferase, H3 lysine-79 specific	Body	3565	'DOT1L	histone-lysine N-methyltransferase, H3 lysine-79 specific
GA	cg04176246	2.20E-05	0.005949657	FDR0.01	17	55434640	'MSI2	RNA-binding protein Musashi homolog 2	Body;Body;Body;Body	100261	'MSI2	RNA-binding protein Musashi homolog 2
GA	cg04213841	2.09E-10	1.32E-06	BF|FDR0.01	13	49792685	'MLNR	motilin receptor	TSS5000	-1788	'MLNR	motilin receptor
GA	cg04229930	3.03E-05	0.007277728	FDR0.01	2	224433836	'			33243	'SCG2	secretogranin-2 precursor
GA	cg04232972	4.06E-06	0.001832219	FDR0.01	16	30671644	'PRR14;FBRS	proline-rich protein 14;probable fibrosin-1	TES5000;TES5000;Body	1893	'FBRS	probable fibrosin-1
GA	cg04234016	1.87E-07	0.000191917	FDR0.01	12	7062109	'PTPN6	tyrosine-protein phosphatase non-receptor type 6	Body;Body;Body	1591	'PTPN6	tyrosine-protein phosphatase non-receptor type 6
GA	cg04257388	2.33E-05	0.006158722	FDR0.01	10	16741464	'RSU1	ras suppressor protein 1	Body;Body	117959	'RSU1	ras suppressor protein 1
GA	cg04264638	3.68E-07	0.000323114	FDR0.01	4	56411138	'CLOCK	circadian locomoter output cycles protein kaput	Body;Body	961	'CLOCK	circadian locomoter output cycles protein kaput
GA	cg04272820	2.99E-06	0.001454315	FDR0.01	17	38228538	'THRA	thyroid hormone receptor alpha	Body;Body;Body;Body	9321	'THRA	thyroid hormone receptor alpha
GA	cg04275695	1.68E-08	3.10E-05	BF|FDR0.01	6	149806273	'ZC3H12D	probable ribonuclease ZC3H12D	TSS200	-123	'ZC3H12D	probable ribonuclease ZC3H12D
GA	cg04310331	7.20E-06	0.002742486	FDR0.01	11	2012332	'MRPL23-AS1;LINC01219;HOTS;H19	MRPL23 antisense RNA 1;long intergenic non-protein coding RNA 1219;;H19	TSS1500;Body;TSS1500;TES500	7	'LINC01219	long intergenic non-protein coding RNA 1219
GA	cg04312620	2.91E-05	0.007120757	FDR0.01	20	50419214	'SALL4	sal-like protein 4	TSS200;TSS200	-154	'SALL4	sal-like protein 4
GA	cg04314978	8.58E-07	0.000598841	FDR0.01	11	36509910	'TRAF6	TNF receptor-associated factor 6	3'UTR;3'UTR	21912	'TRAF6	TNF receptor-associated factor 6
GA	cg04321555	1.05E-05	0.00353656	FDR0.01	19	34285737	'KCTD15	BTB/POZ domain-containing protein KCTD15	TSS5000;TSS5000;TSS5000	-2037	'KCTD15	BTB/POZ domain-containing protein KCTD15
GA	cg04322363	1.78E-06	0.001023455	FDR0.01	3	159757071	'IL12A-AS1	IL12A antisense RNA 1	Body	18636	'LINC01100	long intergenic non-protein coding RNA 1100
GA	cg04326337	2.31E-06	0.001232537	FDR0.01	20	36664969	'TTI1;RPRD1B	TELO2-interacting protein 1 homolog;regulation of nuclear pre-mRNA domai	TSS5000;TSS5000;Body	2822	'RPRD1B	regulation of nuclear pre-mRNA domain-containing protein 1B
GA	cg04338652	1.64E-05	0.004879292	FDR0.01	18	13136597	'			-82161	'LDLRAD4	low-density lipoprotein receptor class A domain-containing protein 4
GA	cg04345034	3.26E-09	9.56E-06	BF|FDR0.01	15	70797389	'LINC02205	long intergenic non-protein coding RNA 2205	Body	878	'LINC02205	long intergenic non-protein coding RNA 2205
GA	cg04347477	2.68E-07	0.00025206	FDR0.01	12	125002007	'NCOR2	nuclear receptor corepressor 2	Body;Body;Body	50151	'NCOR2	nuclear receptor corepressor 2
GA	cg04348265	2.24E-05	0.006014794	FDR0.01	4	186733060	'SORBS2	sorbin and SH3 domain-containing protein 2 isoform 6	TSS1500;TSS1500;5'UTR;5'UTR;	350	'SORBS2	sorbin and SH3 domain-containing protein 2 isoform 6
GA	cg04348643	7.34E-07	0.000534355	FDR0.01	3	153572883	'			-92568	'LINC02006	long intergenic non-protein coding RNA 2006
GA	cg04348689	2.74E-05	0.006842586	FDR0.01	14	93765993	'BTBD7	BTB/POZ domain-containing protein 7 isoform 3	Body;Body;Body	33389	'BTBD7	BTB/POZ domain-containing protein 7 isoform 3
GA	cg04352088	2.62E-05	0.006654375	FDR0.01	22	43609331	'SCUBE1;LOC105373051	signal peptide, CUB and EGF-like domain-containing protein 1 precursor;	Body;Body	652	'LOC105373051	
GA	cg04354689	1.62E-06	0.000957103	FDR0.01	16	2660830	'LOC652276	potassium channel tetramerization domain containing 5 pseudogene	Body	7446	'LOC652276	potassium channel tetramerization domain containing 5 pseudogene
GA	cg04359840	1.44E-05	0.004466242	FDR0.01	16	17563300	'XYLT1	xylosyltransferase 1 precursor	Body	1517	'XYLT1	xylosyltransferase 1 precursor
GA	cg04363228	5.15E-10	2.56E-06	BF|FDR0.01	6	32155141	'AGER;PBX2;GPSM3	advanced glycosylation end product-specific receptor precursor;pre-B-cell le	TSS5000;TSS5000;TSS5000;TSS5	2838	'PBX2	pre-B-cell leukemia transcription factor 2
GA	cg04384031	1.21E-05	0.003952549	FDR0.01	17	19631485	'			-9193	'SLC47A2	multidrug and toxin extrusion protein 2
GA	cg04391186	4.13E-05	0.008895517	FDR0.01	7	24446070	'			122262	'NPY	neuropeptide Y precursor
GA	cg04399988	6.27E-06	0.002497606	FDR0.01	14	24018055	'THTPA;ZFHX2	thiamine-triphosphatase;zinc finger homeobox protein 2	Body;Body;Body	2919	'ZFHX2	zinc finger homeobox protein 2
GA	cg04409030	6.61E-06	0.00259145	FDR0.01	12	93963292	'SOCS2-AS1;SOCS2	SOCS2 antisense RNA 1;suppressor of cytokine signaling 2	Body;TSS1500;TSS1500;TSS150	-452	'SOCS2	suppressor of cytokine signaling 2
GA	cg04411156	4.27E-05	0.009067438	FDR0.01	3	57543029	'PDE12	2',5'-phosphodiesterase 12	1stExon;1stExon;1stExon	1029	'PDE12	2',5'-phosphodiesterase 12
GA	cg04411893	1.07E-06	0.000709212	FDR0.01	3	185300709	'SENP2	sentrin-specific protease 2	TSS5000	-3373	'SENP2	sentrin-specific protease 2
GA	cg04416898	4.88E-07	0.000394693	FDR0.01	5	139040055	'CXXC5	CXXC-type zinc finger protein 5	Body;Body;Body;Body;Body;Bo	916	'CXXC5	CXXC-type zinc finger protein 5
GA	cg04427651	1.82E-06	0.001035833	FDR0.01	1	27152694	'ZDHHC18	palmitoyltransferase ZDHHC18	TSS1500	-484	'ZDHHC18	palmitoyltransferase ZDHHC18
GA	cg04441815	3.71E-05	0.008360353	FDR0.01	8	144448389	'RHPN1-AS1;RHPN1	RHPN1 antisense RNA 1 (head to head);rhophilin-1	TES5000;TSS5000	2416	'RHPN1-AS1	RHPN1 antisense RNA 1 (head to head)
GA	cg04442638	6.85E-06	0.002649337	FDR0.01	17	73873823	'TRIM47	E3 ubiquitin-protein ligase TRIM47	Body	839	'TRIM47	E3 ubiquitin-protein ligase TRIM47
GA	cg04451175	1.34E-05	0.004243425	FDR0.01	8	16860088	'FGF20	fibroblast growth factor 20	TSS1500	-234	'FGF20	fibroblast growth factor 20
GA	cg04452040	1.69E-06	0.000984798	FDR0.01	10	102501358	'PAX2	paired box protein Pax-2	Body;TSS5000;TSS5000;TSS500	-3980	'PAX2	paired box protein Pax-2
GA	cg04494136	5.88E-06	0.002383815	FDR0.01	10	126703576	'CTBP2	C-terminal-binding protein 2	Body;Body;Body;Body;Body;Bo	-8989	'CTBP2	C-terminal-binding protein 2
GA	cg04504205	3.79E-05	0.008481348	FDR0.01	20	45946429	'ZMYND8;LOC100131496	protein kinase C-binding protein 1;uncharacterized LOC100131496	Body;Body;Body;Body;Body;Bo	-816	'LOC100131496	uncharacterized LOC100131496
GA	cg04517263	4.09E-08	6.00E-05	BF|FDR0.01	9	123689193	'TRAF1	TNF receptor-associated factor 1	TSS200;Body	-119	'TRAF1	TNF receptor-associated factor 1
GA	cg04520340	1.58E-07	0.000169426	FDR0.01	1	2005132	'PRKCZ	protein kinase C zeta type	Body;5'UTR;5'UTR;5'UTR;5'UTR	232	'PRKCZ	protein kinase C zeta type
GA	cg04533078	2.57E-06	0.00130441	FDR0.01	16	89382131	'ANKRD11	ankyrin repeat domain-containing protein 11	Body;Body;Body;Body	-5409	'LOC100287036	uncharacterized LOC100287036
GA	cg04549583	4.52E-10	2.30E-06	BF|FDR0.01	2	12146219	'MIR3681HG	MIR3681 host gene	TSS1500;TSS1500;TSS1500	-1022	'MIR3681HG	MIR3681 host gene
GA	cg04554272	3.16E-05	0.007446647	FDR0.01	16	1013990	'LMF1	lipase maturation factor 1	Body;Body;Body;Body;Body;Bo	6994	'LMF1	lipase maturation factor 1
GA	cg04556418	1.03E-05	0.003510259	FDR0.01	4	184367379	'CDKN2AIP	CDKN2A-interacting protein	3'UTR;Body	1591	'CDKN2AIP	CDKN2A-interacting protein
GA	cg04561791	3.88E-05	0.008603768	FDR0.01	16	85478769	'			-138838	'MIR5093	microRNA 5093
GA	cg04573653	1.27E-06	0.000801561	FDR0.01	7	28072450	'JAZF1	juxtaposed with another zinc finger protein 1	Body	-147625	'JAZF1-AS1	JAZF1 antisense RNA 1
GA	cg04574459	1.47E-05	0.00453514	FDR0.01	21	47010768	'LINC01694	long intergenic non-protein coding RNA 1694	TSS5000	-2799	'LINC01694	long intergenic non-protein coding RNA 1694
GA	cg04602755	1.86E-05	0.005349794	FDR0.01	1	154935120	'PYGO2;LOC101928120;SHC1	pygopus homolog 2;uncharacterized protein LOC101928120;SHC-transformi	TSS1500;TES5000;3'UTR;3'UTR;	-862	'PYGO2	pygopus homolog 2
GA	cg04626878	4.31E-05	0.009115326	FDR0.01	20	62168809	'PTK6;SRMS	protein-tyrosine kinase 6;tyrosine-protein kinase Srms	TSS200;TSS200;TES5000	-80	'PTK6	protein-tyrosine kinase 6
GA	cg04636834	1.59E-05	0.004803179	FDR0.01	16	685580	'WFIKKN1;METTL26;LOC100287175	WAP, Kazal, immunoglobulin, Kunitz and NTR domain-containing protein 1 p	TES5000;Body;Body;Body;Body	725	'METTL26	methyltransferase-like 26
GA	cg04659582	3.34E-06	0.001580946	FDR0.01	2	231276073	'SP100	serine protease 100 precursor	TSS5000;TSS5000;TSS5000;TSS5	-4797	'SP100	serine protease 100 precursor
GA	cg04664126	3.56E-05	0.008120945	FDR0.01	6	28554262	'ZBED9	SCAN domain-containing protein 3 isoform 2	1stExon;Body	813	'ZBED9	SCAN domain-containing protein 3 isoform 2
GA	cg04665046	1.86E-05	0.005349794	FDR0.01	9	123691674	'TRAF1	TNF receptor-associated factor 1	TSS5000;TSS1500	-223	'TRAF1	TNF receptor-associated factor 1
GA	cg04685228	3.51E-13	1.43E-08	BF|FDR0.01	5	172462626	'ATP6V0E1	V-type proton ATPase subunit e 1	TES5000	14898	'SNORA74B	small nucleolar RNA, H/ACA box 74B
GA	cg04691264	2.13E-05	0.00584688	FDR0.01	10	29697905	'SVIL-AS1	SVIL antisense RNA 1	TSS1500;TSS1500;TSS1500;TSS1	-557	'SVIL-AS1	SVIL antisense RNA 1
GA	cg04694022	3.60E-07	0.000317496	FDR0.01	6	30582352	'PPP1R10;MRPS18B	serine/threonine-protein phosphatase 1 regulatory subunit 10;28S ribosoma	Body;Body;TSS5000	2668	'PPP1R10	serine/threonine-protein phosphatase 1 regulatory subunit 10
GA	cg04726091	9.25E-10	3.90E-06	BF|FDR0.01	4	140216130	'NDUFC1	NADH dehydrogenase [ubiquinone] 1 subunit C1, mitochondrial	Body;Body;Body;Body;Body;Bo	6234	'NDUFC1	NADH dehydrogenase [ubiquinone] 1 subunit C1, mitochondrial
GA	cg04740205	3.71E-06	0.001712313	FDR0.01	11	82871065	'PCF11	Pcf11p	Body;Body;Body;Body	2893	'PCF11	Pcf11p
GA	cg04740679	2.35E-07	0.000227876	FDR0.01	13	114016140	'GRTP1;GRTP1-AS1	growth hormone-regulated TBC protein 1;GRTP1 antisense RNA 1	Body;Body;Body;Body;Body	2305	'GRTP1	growth hormone-regulated TBC protein 1
GA	cg04749507	1.62E-05	0.004853665	FDR0.01	6	32120203	'PRRT1;LOC100507547;PPT2;PPT2-EGFL8	proline-rich transmembrane protein 1;uncharacterized LOC100507547;lysos	Body;TSS1500;TES5000;TES500	-487	'PRRT1	proline-rich transmembrane protein 1
GA	cg04752680	3.19E-05	0.00749566	FDR0.01	19	14114100	'RFX1	Rfx1p	Body	3032	'RFX1	Rfx1p
GA	cg04777348	3.23E-06	0.001543999	FDR0.01	1	92952897	'GFI1	zinc finger protein Gfi-1	TSS1500;TSS5000;TSS1500	-415	'GFI1	zinc finger protein Gfi-1
GA	cg04781580	1.85E-05	0.005337196	FDR0.01	14	22392573	'			-253341	'OR4E1	olfactory receptor 4E1
GA	cg04793090	3.16E-06	0.001513381	FDR0.01	3	128903277	'CNBP	cellular nucleic acid-binding protein	TSS1500;TSS1500;TSS1500;TSS1	-538	'CNBP	cellular nucleic acid-binding protein
GA	cg04836794	2.06E-05	0.005712275	FDR0.01	12	21827277	'			-16488	'LDHB	L-lactate dehydrogenase B chain
GA	cg04840813	7.58E-06	0.002840256	FDR0.01	3	196363641	'LINC01063;NRROS	long intergenic non-protein coding RNA 1063;transforming growth factor be	TSS5000;TSS5000	-2923	'NRROS	transforming growth factor beta activator LRRC33 precursor
GA	cg04846710	3.74E-05	0.008410914	FDR0.01	1	145478286	'ANKRD34A;LIX1L	ankyrin repeat domain-containing protein 34A;LIX1-like protein	TES5000;Body	1218	'LIX1L	LIX1-like protein
GA	cg04854089	2.62E-06	0.001319172	FDR0.01	11	46355137	'DGKZ	diacylglycerol kinase zeta	Body	662	'DGKZ	diacylglycerol kinase zeta
GA	cg04863713	1.31E-06	0.000820198	FDR0.01	1	150521201	'ADAMTSL4;MIR4257	ADAMTS-like protein 4 precursor;microRNA 4257	TSS1500;TSS1500;TSS1500;TSS1	-643	'ADAMTSL4	ADAMTS-like protein 4 precursor
GA	cg04869854	2.89E-05	0.007103144	FDR0.01	11	67186575	'CARNS1	carnosine synthase 1 isoform 2	1stExon;Body	3427	'CARNS1	carnosine synthase 1 isoform 2

Trait	#cpg	p	fdr	significance	chr	pos.hg19	gene.Illumina	gene.description	locations	dist2TSS	gene.nearest	gene.description
GA	cg04897188	2.09E-05	0.005779049	FDR0.01	1	1363291	'TMEM88B;LINC01770	transmembrane protein 88B;long intergenic non-protein coding RNA 1770	TES5000;TES5000;TES5000;TES	1784	'TMEM88B	transmembrane protein 88B
GA	cg04921068	3.46E-06	0.001615437	FDR0.01	3	160787668	'PPM1L	protein phosphatase 1L isoform 2	3'UTR;3'UTR;3'UTR;Body	34852	'B3GALNT1	UDP-GalNAc:beta-1, 3-N-acetylgalactosaminyltransferase 1
GA	cg04925385	1.63E-10	1.11E-06	BF|FDR0.01	13	52378555	'DHRS12	dehydrogenase/reductase SDR family member 12 isoform 1	TSS1500;TSS1500;TSS1500	-256	'DHRS12	dehydrogenase/reductase SDR family member 12 isoform 1
GA	cg04945076	1.87E-05	0.005356826	FDR0.01	12	131237654	'			-36804	'RIMBP2	RIMS-binding protein 2 isoform g
GA	cg04948284	4.06E-05	0.008802751	FDR0.01	11	65313608	'LTBP3	latent-transforming growth factor beta-binding protein 3 precursor	Body;Body;Body	12091	'LTBP3	latent-transforming growth factor beta-binding protein 3 precursor
GA	cg04952324	2.55E-05	0.006538599	FDR0.01	7	73727108	'CLIP2	CAP-Gly domain-containing linker protein 2	Body;Body	23372	'CLIP2	CAP-Gly domain-containing linker protein 2
GA	cg04953373	7.04E-06	0.002696603	FDR0.01	6	166892762	'RPS6KA2	ribosomal protein S6 kinase alpha-2	Body;Body;Body;Body;Body	-13891	'RPS6KA2-IT1	RPS6KA2 intronic transcript 1
GA	cg04956801	4.73E-06	0.002021722	FDR0.01	10	21798341	'SKIDA1	SKI/DACH domain-containing protein 1	TES5000	-12152	'MIR1915HG	MIR1915 host gene
GA	cg04978343	3.36E-05	0.007778344	FDR0.01	7	38315971	'TARP	TCR gamma alternate reading frame protein isoform 1	TSS5000;TSS5000	-2723	'TARP	TCR gamma alternate reading frame protein isoform 1
GA	cg04980428	6.34E-07	0.000481628	FDR0.01	1	26098214	'MAN1C1	mannosyl-oligosaccharide 1,2-alpha-mannosidase IC isoform 1	Body;Body	-28469	'SELENON	selenoprotein N precursor
GA	cg04984663	4.95E-06	0.002089817	FDR0.01	10	102632048	'			-40671	'SLF2	SMC5-SMC6 complex localization factor protein 2
GA	cg04997045	9.74E-09	2.10E-05	BF|FDR0.01	4	1240068	'CTBP1;CTBP1-DT	C-terminal-binding protein 1;CTBP1 divergent transcript	Body;Body;TSS5000	2850	'CTBP1	C-terminal-binding protein 1
GA	cg04999352	2.77E-08	4.55E-05	BF|FDR0.01	11	63304614	'PLAAT4	phospholipase A and acyltransferase 4	Body	335	'PLAAT4	phospholipase A and acyltransferase 4
GA	cg05006813	6.50E-07	0.000489338	FDR0.01	5	120069936	'			202778	'PRR16	protein Largen isoform 2
GA	cg05007997	1.88E-05	0.00537193	FDR0.01	2	219246985	'SLC11A1	natural resistance-associated macrophage protein 1	TSS200	-10	'SLC11A1	natural resistance-associated macrophage protein 1
GA	cg05012902	1.36E-05	0.004297122	FDR0.01	17	8065517	'VAMP2	vesicle-associated membrane protein 2	Body;Body	677	'VAMP2	vesicle-associated membrane protein 2
GA	cg05017829	3.39E-05	0.007807701	FDR0.01	5	176024970	'CDHR2;GPRIN1	cadherin-related family member 2 precursor;G protein-regulated inducer of	TES5000;TES5000;Body	12187	'GPRIN1	G protein-regulated inducer of neurite outgrowth 1
GA	cg05027597	9.07E-06	0.003206256	FDR0.01	19	47935656	'SLC8A2	sodium/calcium exchanger 2 precursor	Body	-10166	'MEIS3	homeobox protein Meis3
GA	cg05046097	2.30E-06	0.001230661	FDR0.01	10	88717926	'MMRN2;SNCG	multimerin-2 precursor;gamma-synuclein	TSS1500;Body;TSS1500	-429	'SNCG	gamma-synuclein
GA	cg05051976	3.31E-05	0.007698271	FDR0.01	12	69329653	'CPM	carboxypeptidase M precursor	TSS5000;TSS5000;Body	-2674	'CPM	carboxypeptidase M precursor
GA	cg05055821	6.20E-06	0.002471881	FDR0.01	7	2758924	'AMZ1	archaemetzincin-1	TES5000;TES5000;Body	31089	'AMZ1	archaemetzincin-1
GA	cg05087455	1.34E-05	0.004243425	FDR0.01	2	7171869	'RNF144A	E3 ubiquitin-protein ligase RNF144A isoform 4	Body;Body;Body;Body;Body;Bo	46142	'LOC101929452	uncharacterized LOC101929452
GA	cg05091585	4.35E-07	0.00036589	FDR0.01	13	45507332	'			56274	'NUFIP1	nuclear fragile X mental retardation-interacting protein 1
GA	cg05099952	3.79E-05	0.0084904	FDR0.01	16	30367546	'CD2BP2;TBC1D10B	CD2 antigen cytoplasmic tail-binding protein 2;TBC1 domain family member	TSS1500;TSS1500;TES5000	-917	'CD2BP2	CD2 antigen cytoplasmic tail-binding protein 2
GA	cg05138397	9.27E-06	0.003252257	FDR0.01	1	160768549	'LY9	T-lymphocyte surface antigen Ly-9	Body;Body;Body;Body	2586	'LY9	T-lymphocyte surface antigen Ly-9
GA	cg05141059	7.22E-07	0.000528511	FDR0.01	3	195162052	'ACAP2	arf-GAP with coiled-coil, ANK repeat and PH domain-containing protein 2	Body	1697	'ACAP2	arf-GAP with coiled-coil, ANK repeat and PH domain-containing protein 2
GA	cg05164926	1.96E-05	0.005518116	FDR0.01	17	7255624	'ACAP1;KCTD11;TMEM95	arf-GAP with coiled-coil, ANK repeat and PH domain-containing protein 1;BT	TES5000;5'UTR;5'UTR;TSS5000;	144	'KCTD11	BTB/POZ domain-containing protein KCTD11
GA	cg05172887	3.46E-05	0.007952439	FDR0.01	2	147815931	'			471307	'PABPC1P2	poly(A) binding protein cytoplasmic 1 pseudogene 2
GA	cg05175896	1.56E-05	0.004720445	FDR0.01	12	1749013	'WNT5B	protein Wnt-5b precursor	Body;Body	10617	'WNT5B	protein Wnt-5b precursor
GA	cg05177060	2.82E-09	8.70E-06	BF|FDR0.01	7	142511280	'			-41495	'EPHB6	ephrin type-B receptor 6 precursor
GA	cg05190002	1.55E-08	2.92E-05	BF|FDR0.01	2	219262192	'SLC11A1;CTDSP1	natural resistance-associated macrophage protein 1;carboxy-terminal domai	TES5000;TSS1500;TSS5000;TSS	-763	'CTDSP1	carboxy-terminal domain RNA polymerase II polypeptide A small phosphatase 1
GA	cg05205842	1.31E-06	0.000820198	FDR0.01	4	38673130	'KLF3	Krueppel-like factor 3	Body	-6881	'KLF3-AS1	KLF3 antisense RNA 1
GA	cg05208178	1.06E-05	0.003569991	FDR0.01	2	233928423	'INPP5D	phosphatidylinositol 3,4,5-trisphosphate 5-phosphatase 1	Body;Body	3372	'INPP5D	phosphatidylinositol 3,4,5-trisphosphate 5-phosphatase 1
GA	cg05211189	2.29E-11	3.02E-07	BF|FDR0.01	16	2055403	'ZNF598	E3 ubiquitin-protein ligase ZNF598	Body	4419	'ZNF598	E3 ubiquitin-protein ligase ZNF598
GA	cg05220968	1.33E-05	0.004243003	FDR0.01	6	146057943	'EPM2A;LOC100507557	laforin;	TSS1500;TSS1500;TSS1500;TSS	-784	'EPM2A	laforin
GA	cg05225883	4.45E-05	0.009299776	FDR0.01	10	134043755	'STK32C	serine/threonine-protein kinase 32C	Body;Body;Body;Body	18857	'STK32C	serine/threonine-protein kinase 32C
GA	cg05249026	7.00E-06	0.002684478	FDR0.01	16	11031524	'CIITA;DEXI	MHC class II transactivator isoform 2;dexamethasone-induced protein	TES5000;TES5000;Body	4801	'DEXI	dexamethasone-induced protein
GA	cg05251190	3.38E-05	0.007800019	FDR0.01	10	104196206	'CUEDC2;MIR146B	CUE domain-containing protein 2 isoform 1;microRNA 146b	TSS5000;TSS200	-62	'MIR146B	microRNA 146b
GA	cg05253759	1.41E-05	0.00438696	FDR0.01	17	26941260	'SPAG5-AS1;RSKR;KIAA0100	SPAG5 antisense RNA 1;ribosomal protein S6 kinase-related protein;protein	Body;TSS200;TES5000;TES5000	-57	'RSKR	ribosomal protein S6 kinase-related protein
GA	cg05260877	2.64E-06	0.001329339	FDR0.01	11	102068599	'YAP1	DNA-binding transcription factor YAP1	Body;Body;Body;Body;Body;Bo	85421	'YAP1	DNA-binding transcription factor YAP1
GA	cg05276360	3.08E-05	0.007359402	FDR0.01	19	11354401	'DOCK6;ANGPTL8	dedicator of cytokinesis protein 6;angiopoietin-like protein 8 precursor	Body;Body;TES5000	4107	'ANGPTL8	angiopoietin-like protein 8 precursor
GA	cg05298628	2.05E-13	1.15E-08	BF|FDR0.01	12	123379569	'VPS37B	vacuolar protein sorting-associated protein 37B	Body	1102	'VPS37B	vacuolar protein sorting-associated protein 37B
GA	cg05302531	2.03E-05	0.005661075	FDR0.01	7	101936362	'SH2B2	SH2B adapter protein 2 isoform 1	Body;Body;Body;Body;Body	3607	'SH2B2	SH2B adapter protein 2 isoform 1
GA	cg05315334	1.57E-05	0.004752088	FDR0.01	13	113656212	'MCF2L	guanine nucleotide exchange factor DBS	Body;Body;Body;5'UTR;5'UTR;5	185	'MCF2L	guanine nucleotide exchange factor DBS
GA	cg05317714	1.51E-05	0.004635914	FDR0.01	8	145698116	'KIFC2;FOXH1	kinesin-like protein KIFC2;forkhead box protein H1	Body;Body;TES5000	3116	'FOXH1	forkhead box protein H1
GA	cg05323251	1.83E-05	0.005293122	FDR0.01	19	10449942	'ICAM3	intercellular adhesion molecule 3 precursor	Body;Body;Body;Body	358	'ICAM3	intercellular adhesion molecule 3 precursor
GA	cg05324273	3.14E-05	0.007428671	FDR0.01	10	60086738	'LOC112268068	uncharacterized LOC112268068	Body	2701	'LOC112268068	uncharacterized LOC112268068
GA	cg05339037	2.41E-06	0.00125643	FDR0.01	19	13951168	'LOC284454;MIR24-2;MIR27A;MIR23A	;microRNA 24-2;microRNA 27a;microRNA 23a	Body;TSS5000;TSS5000;TSS500	2564	'LOC284454	
GA	cg05352688	3.72E-07	0.000326393	FDR0.01	1	211643515	'			22744	'RD3	protein RD3
GA	cg05368971	2.61E-05	0.006646408	FDR0.01	1	2321364	'MORN1;RER1	MORN repeat-containing protein 1 isoform 2;protein RER1	Body;Body;Body;TSS5000	1629	'MORN1	MORN repeat-containing protein 1 isoform 2
GA	cg05374956	4.47E-05	0.009324362	FDR0.01	19	5838735	'FUT6;FUT3	4-galactosyl-N-acetylglucosaminide 3-alpha-L-fucosyltransferase FUT6 isofor	5'UTR;5'UTR;5'UTR;5'UTR;5'UT	68	'FUT6	4-galactosyl-N-acetylglucosaminide 3-alpha-L-fucosyltransferase FUT6 isoform 1
GA	cg05384139	9.55E-06	0.003319253	FDR0.01	12	123548027	'PITPNM2	membrane-associated phosphatidylinositol transfer protein 2	Body;Body	-17903	'PITPNM2-AS1	PITPNM2 antisense RNA 1
GA	cg05387269	1.39E-07	0.000154328	FDR0.01	1	1306946	'AURKAIP1	aurora kinase A-interacting protein	TES5000;TES5000;TES5000	3641	'AURKAIP1	aurora kinase A-interacting protein
GA	cg05394783	2.28E-05	0.006078006	FDR0.01	10	134043897	'STK32C	serine/threonine-protein kinase 32C	Body;Body;Body;Body	18715	'STK32C	serine/threonine-protein kinase 32C
GA	cg05397514	2.15E-05	0.005860696	FDR0.01	1	155240680	'CLK2	dual specificity protein kinase CLK2	1stExon;1stExon;5'UTR;1stExon	2615	'CLK2	dual specificity protein kinase CLK2
GA	cg05398260	2.81E-05	0.006958299	FDR0.01	7	64363546	'ZNF273	zinc finger protein 273	TSS200;TSS200	-73	'ZNF273	zinc finger protein 273
GA	cg05412531	1.81E-05	0.005259275	FDR0.01	8	125183758	'FER1L6-AS2	FER1L6 antisense RNA 2	Body	5	'FER1L6-AS2	FER1L6 antisense RNA 2
GA	cg05413628	1.08E-07	0.000129809	BF|FDR0.01	16	1521656	'CLCN7	H(+)/Cl(-) exchange transporter 7	Body;Body	3373	'CLCN7	H(+)/Cl(-) exchange transporter 7
GA	cg05433642	1.47E-06	0.00088757	FDR0.01	13	97873992	'MBNL2	muscleblind-like protein 2	TSS1500;TSS1500;TSS1500	-550	'MBNL2	muscleblind-like protein 2
GA	cg05436939	2.59E-05	0.00661421	FDR0.01	4	1202599	'SPON2;LOC100130872;CTBP1-AS;CTBP1	spondin-2 precursor;uncharacterized LOC100130872;CTBP1 antisense RNA;C	5'UTR;Body;TSS1500;TES5000;T	151	'SPON2	spondin-2 precursor
GA	cg05437446	3.65E-05	0.008274484	FDR0.01	13	113622743	'MCF2L-AS1;MCF2L	MCF2L antisense RNA 1;guanine nucleotide exchange factor DBS	Body;TSS1500;TSS1500	209	'MCF2L-AS1	MCF2L antisense RNA 1
GA	cg05447916	1.75E-05	0.005129309	FDR0.01	6	31744831	'VWA7;VARS1	von Willebrand factor A domain-containing protein 7 precursor;valine--tRNA	Body;TES5000	274	'VWA7	von Willebrand factor A domain-containing protein 7 precursor
GA	cg05462570	4.65E-05	0.009586929	FDR0.01	11	64814948	'SAC3D1;NAALADL1	SAC3 domain-containing protein 1;aminopeptidase NAALADL1	TES5000;TES5000;TES5000;TES	6267	'SAC3D1	SAC3 domain-containing protein 1
GA	cg05486872	2.48E-05	0.006417709	FDR0.01	18	3066822	'MYOM1	myomesin-1	3'UTR;3'UTR	-54877	'LPIN2	phosphatidate phosphatase LPIN2
GA	cg05502464	2.44E-06	0.001269028	FDR0.01	15	23086888	'NIPA1	magnesium transporter NIPA1 isoform 2	TSS1500;TSS200	-45	'NIPA1	magnesium transporter NIPA1 isoform 2
GA	cg05503887	2.24E-05	0.005995871	FDR0.01	11	34641925	'EHF	ETS homologous factor	TSS1500;TSS1500	-714	'EHF	ETS homologous factor
GA	cg05506292	3.06E-05	0.007318911	FDR0.01	15	85925235	'MIR7706;AKAP13	microRNA 7706;A-kinase anchor protein 13 isoform 4	TES5000;Body;Body	1402	'AKAP13	A-kinase anchor protein 13 isoform 4
GA	cg05512483	1.61E-05	0.004845574	FDR0.01	11	62621791	'SNHG1;SNORD22;SNORD31;SNORD30;SNORD29;SN	small nucleolar RNA host gene 1;small nucleolar RNA, C/D box 22;small nucle	Body;Body;Body;Body;Body;Bo	-351	'SNORD29	small nucleolar RNA, C/D box 29
GA	cg05512561	2.38E-11	3.04E-07	BF|FDR0.01	13	33925656	'STARD13	stAR-related lipid transfer protein 13	Body	-16253	'LINC02344	long intergenic non-protein coding RNA 2344
GA	cg05531689	4.18E-06	0.001864837	FDR0.01	2	26729139	'OTOF	otoferlin	Body;Body	-28147	'OTOF	otoferlin
GA	cg05541267	1.33E-09	5.14E-06	BF|FDR0.01	3	50647374	'CISH;MAPKAPK3	cytokine-inducible SH2-containing protein;MAP kinase-activated protein kina	Body;Body;TSS5000	1831	'CISH	cytokine-inducible SH2-containing protein
GA	cg05543030	3.95E-05	0.008669706	FDR0.01	2	45005364	'			160663	'LINC01833	long intergenic non-protein coding RNA 1833
GA	cg05546863	1.13E-06	0.000734982	FDR0.01	16	66613220	'CKLF-CMTM1;CMTM1;CMTM2	CKLF-CMTM1 protein isoform 3;CKLF-like MARVEL transmembrane domain-c	TES5000;TES5000;TES5000;TES	-145	'CMTM2	CKLF-like MARVEL transmembrane domain-containing protein 2
GA	cg05567440	2.07E-10	1.32E-06	BF|FDR0.01	11	61772344	'			-37238	'FTH1	ferritin heavy chain
GA	cg05580141	3.37E-06	0.00158873	FDR0.01	12	49071788	'KANSL2	KAT8 regulatory NSL complex subunit 2	Body	4233	'KANSL2	KAT8 regulatory NSL complex subunit 2
GA	cg05592245	3.60E-05	0.008185792	FDR0.01	10	65280473	'JMJD1C;REEP3	probable JmjC domain-containing histone demethylation protein 2C;recepto	Body;Body;TSS1500	-687	'REEP3	receptor expression-enhancing protein 3
GA	cg05600717	4.91E-06	0.002078476	FDR0.01	13	52378744	'DHRS12	dehydrogenase/reductase SDR family member 12 isoform 1	TSS1500;TSS1500;TSS1500	-445	'DHRS12	dehydrogenase/reductase SDR family member 12 isoform 1
GA	cg05603985	1.17E-07	0.000136961	FDR0.01	1	2161049	'SKI	ski oncogene	1stExon	1292	'SKI	ski oncogene
GA	cg05625284	4.75E-05	0.009710744	FDR0.01	3	32509105	'			35239	'CMTM6	CKLF-like MARVEL transmembrane domain-containing protein 6
GA	cg05637296	1.66E-05	0.004938655	FDR0.01	17	76129475	'TMC6;TMC8	transmembrane channel-like protein 6;transmembrane channel-like protein	TSS5000;TSS5000;TSS1500;Bod	-967	'TMC6	transmembrane channel-like protein 6
GA	cg05638787	2.48E-07	0.000237641	FDR0.01	15	41062273	'GCHFR;DNAJC17;C15orf62	GTP cyclohydrolase 1 feedback regulatory protein;dnaJ homolog subfamily C	TES5000;Body;5'UTR	96	'C15orf62	uncharacterized protein C15orf62, mitochondrial precursor
GA	cg05641843	8.35E-06	0.003040491	FDR0.01	17	7477972	'SENP3;SENP3-EIF4A1;EIF4A1;SNORA48;SNORD10;SN	sentrin-specific protease 3;SENP3-EIF4A1 readthrough (NMD candidate);euk	TES5000;Body;Body;Body;TSS2	-58	'SNORA48	small nucleolar RNA, H/ACA box 48
GA	cg05660881	8.28E-07	0.00058792	FDR0.01	13	24798240	'SPATA13	spermatogenesis-associated protein 13 isoform 6	Body;Body;1stExon	30337	'SPATA13-AS1	SPATA13 antisense RNA 1
GA	cg05666458	2.45E-05	0.006374174	FDR0.01	11	787971	'LOC171391;CEND1;SLC25A22	uncharacterized LOC171391;cell cycle exit and neuronal differentiation prote	TES5000;TES5000;3'UTR;TES50	2119	'CEND1	cell cycle exit and neuronal differentiation protein 1

Trait	#cpg	p	fdr	significance	chr	pos.hg19	gene.Illumina	gene.description	locations	dist2TSS	gene.nearest	gene.description
GA	cg05693982	9.68E-07	0.000657283	FDR0.01	12	12940390	'APOLD1	apolipoprotein L domain-containing protein 1	Body;Body	1798	'APOLD1	apolipoprotein L domain-containing protein 1
GA	cg05697274	2.66E-08	4.40E-05	BF|FDR0.01	1	230415377	'GALNT2	polypeptide N-acetylgalactosaminyltransferase 2 precursor	3'UTR;3'UTR	146701	'PGBD5	piggyBac transposable element-derived protein 5
GA	cg05730269	5.67E-08	7.76E-05	BF|FDR0.01	11	118477055	'ARCN1;PHLDB1	coatomer subunit delta;pleckstrin homology-like domain family B member 1	TES5000;TES5000;TSS200;TSS1!	-95	'PHLDB1	pleckstrin homology-like domain family B member 1
GA	cg05746014	3.40E-05	0.007827843	FDR0.01	6	107860875	'SOBP	sine oculis-binding protein homolog	Body	49555	'SOBP	sine oculis-binding protein homolog
GA	cg05776630	2.95E-05	0.007183669	FDR0.01	1	20015027	'TMCO4	transmembrane and coiled-coil domain-containing protein 4 isoform 1	Body;Body;Body;Body	23660	'HTR6	5-hydroxytryptamine receptor 6
GA	cg05779272	5.29E-09	1.40E-05	BF|FDR0.01	9	33168018	'B4GALT1;B4GALT1-AS1	beta-1,4-galactosyltransferase 1;B4GALT1 antisense RNA 1	TSS1500;Body;Body;Body	162	'B4GALT1-AS1	B4GALT1 antisense RNA 1
GA	cg05783290	2.64E-05	0.006679927	FDR0.01	7	150383337	'GIMAP2	GTPase IMAP family member 2	Body	544	'GIMAP2	GTPase IMAP family member 2
GA	cg05787209	3.55E-06	0.001645801	FDR0.01	16	31022521	'STX1B	syntaxin1B	TSS1500	-562	'STX1B	syntaxin1B
GA	cg05787952	4.42E-05	0.009280449	FDR0.01	16	734667	'LINC02867;STUB1;JMJD8;WDR24	;E3 ubiquitin-protein ligase CHIP;jmjC domain-containing protein 8 precursor	TSS5000;TES5000;TES5000;TSS1	-333	'JMJD8	jmjC domain-containing protein 8 precursor
GA	cg05810129	2.20E-05	0.005947158	FDR0.01	10	14597004	'FAM107B	protein FAM107B isoform b	Body;Body;Body;Body;Body;Bo	1175	'FAM107B	protein FAM107B isoform b
GA	cg05812836	1.06E-07	0.000128656	BF|FDR0.01	4	130015087	'SCLT1;C4orf33	sodium channel and clathrin linker 1;UPF0462 protein C4orf33	TSS1500;TSS1500;TSS1500;5'UT	221	'C4orf33	UPF0462 protein C4orf33
GA	cg05837435	2.01E-05	0.005616168	FDR0.01	10	118609965	'HSPA12A;ENO4	heat shock 70 kDa protein 12A isoform 1;enolase 4	TSS1500;Body	-732	'HSPA12A	heat shock 70 kDa protein 12A isoform 1
GA	cg05858126	2.98E-05	0.007219891	FDR0.01	10	104196213	'CUEDC2;MIR146B	CUE domain-containing protein 2 isoform 1;microRNA 146b	TSS5000;TSS200	-55	'MIR146B	microRNA 146b
GA	cg05871207	1.81E-05	0.005259275	FDR0.01	5	676441	'TPPP	tubulin polymerization-promoting protein	Body	17026	'TPPP	tubulin polymerization-promoting protein
GA	cg05871254	4.14E-05	0.008900515	FDR0.01	17	14213225	'HS3ST3B1;MGC12916	heparan sulfate glucosamine 3-O-sulfotransferase 3B1;uncharacterized prote	Body;Body;TES5000	6169	'MGC12916	uncharacterized protein MGC12916
GA	cg05878786	1.61E-06	0.000955343	FDR0.01	2	192701329	'CAVIN2	caveolae-associated protein 2	Body	10485	'CAVIN2	caveolae-associated protein 2
GA	cg05881762	1.56E-05	0.004720445	FDR0.01	15	25684849	'UBE3A	ubiquitin-protein ligase E3A isoform 1	TSS1500;TSS1500;TSS1500;TSS1	-646	'UBE3A	ubiquitin-protein ligase E3A isoform 1
GA	cg05883690	1.98E-05	0.005557935	FDR0.01	11	112837374	'LOC101928847;NCAM1	uncharacterized LOC101928847;neural cell adhesion molecule 1 precursor	TSS5000;Body;Body;Body;Body	-3192	'LOC101928847	uncharacterized LOC101928847
GA	cg05896295	1.52E-05	0.0046475	FDR0.01	19	41812492	'HNRNPUL1;CCDC97	heterogeneous nuclear ribonucleoprotein U-like protein 1;coiled-coil domain	3'UTR;3'UTR;3'UTR;3'UTR;3'UT	-3584	'CCDC97	coiled-coil domain-containing protein 97
GA	cg05903330	8.73E-06	0.003126646	FDR0.01	22	39542136	'CBX7	chromobox protein homolog 7	Body;Body;Body	6549	'CBX7	chromobox protein homolog 7
GA	cg05928218	4.81E-05	0.009816242	FDR0.01	1	76188917	'ACADM	medium-chain specific acyl-CoA dehydrogenase, mitochondrial isoform c	TSS1500;TSS1500;TSS1500;TSS1	-1114	'ACADM	medium-chain specific acyl-CoA dehydrogenase, mitochondrial isoform c
GA	cg05958910	3.86E-08	5.78E-05	BF|FDR0.01	10	21796441	'			-10252	'MIR1915HG	MIR1915 host gene
GA	cg05960182	1.15E-07	0.000134593	FDR0.01	1	16160360	'FLJ37453	uncharacterized LOC729614	TES5000	-13841	'SPEN	msx2-interacting protein
GA	cg06008435	2.77E-05	0.006897045	FDR0.01	6	25027714	'RIPOR2	rho family-interacting cell polarization regulator 2	Body	14802	'RIPOR2	rho family-interacting cell polarization regulator 2
GA	cg06013494	7.66E-08	9.87E-05	BF|FDR0.01	17	72528764	'CD300LB	CMRF35-like molecule 7 precursor	TSS1500	-1150	'CD300LB	CMRF35-like molecule 7 precursor
GA	cg06015525	3.02E-05	0.007272241	FDR0.01	12	57872123	'ARHGAP9	rho GTPase-activating protein 9 isoform 2	TSS1500;TSS1500;TSS1500;TSS1	-473	'ARHGAP9	rho GTPase-activating protein 9 isoform 2
GA	cg06016427	4.91E-07	0.000395985	FDR0.01	4	71862187	'DCK	deoxycytidine kinase	Body	2822	'DCK	deoxycytidine kinase
GA	cg06017559	1.53E-05	0.004658203	FDR0.01	16	1026760	'LMF1	lipase maturation factor 1	Body;Body;Body;Body	4853	'LMF1	lipase maturation factor 1
GA	cg06029308	1.56E-06	0.000931391	FDR0.01	3	57995069	'FLNB	filamin-B	Body;Body;Body;Body	921	'FLNB	filamin-B
GA	cg06029792	2.37E-06	0.001249385	FDR0.01	15	70326224	'			45583	'MIR629	microRNA 629
GA	cg06033254	4.11E-11	4.44E-07	BF|FDR0.01	16	66613355	'CKLF-CMTM1;CMTM1;CMTM2	CKLF-CMTM1 protein isoform 3;CKLF-like MARVEL transmembrane domain-c	TES5000;TES5000;TES5000;TES!	-10	'CMTM2	CKLF-like MARVEL transmembrane domain-containing protein 2
GA	cg06046912	2.58E-06	0.001307649	FDR0.01	2	55747633	'CFAP36	cilia- and flagella-associated protein 36	Body;Body	903	'CFAP36	cilia- and flagella-associated protein 36
GA	cg06055249	1.22E-05	0.003978948	FDR0.01	5	153988192	'			-12560	'MIR3141	microRNA 3141
GA	cg06057569	1.69E-14	1.26E-09	BF|FDR0.01	22	50219754	'BRD1	bromodomain-containing protein 1	TSS5000;TSS5000;TSS5000;TSS1	-206	'BRD1	bromodomain-containing protein 1
GA	cg06067425	3.54E-05	0.008091002	FDR0.01	4	141418804	'MGAT4D	alpha-1,3-mannosyl-glycoprotein 4-beta-N-acetylglucosaminyltransferase-lik	Body	643	'MGAT4D	alpha-1,3-mannosyl-glycoprotein 4-beta-N-acetylglucosaminyltransferase-like protein MGAT4D
GA	cg06097370	3.36E-05	0.0077764	FDR0.01	10	21604082	'			57797	'LINC02643	long intergenic non-protein coding RNA 2643
GA	cg06100166	3.08E-05	0.007359402	FDR0.01	3	49377028	'USP4	ubiquitin carboxyl-terminal hydrolase 4	Body;Body;Body	458	'USP4	ubiquitin carboxyl-terminal hydrolase 4
GA	cg06108383	3.98E-07	0.000342506	FDR0.01	6	32120899	'PRRT1;LOC100507547;PPT2;PPT2-EGFL8	proline-rich transmembrane protein 1;uncharacterized LOC100507547;lysos	TSS200;TSS1500;Body;Body;Bo	-39	'PRRT1	proline-rich transmembrane protein 1
GA	cg06126809	8.20E-06	0.003009876	FDR0.01	17	72767008	'SLC9A3R1;NAT9	Na(+)/H(+) exchange regulatory cofactor NHE-RF1;N-acetyltransferase 9	TES5000;3'UTR;3'UTR;3'UTR;3'U	5476	'NAT9	N-acetyltransferase 9
GA	cg06134471	1.86E-06	0.001049272	FDR0.01	14	51560134	'TRIM9	E3 ubiquitin-protein ligase TRIM9 isoform 1	Body;Body	2288	'TRIM9	E3 ubiquitin-protein ligase TRIM9 isoform 1
GA	cg06143522	4.38E-05	0.009224399	FDR0.01	13	88330200	'SLITRK5	SLIT and NTRK-like protein 5	1stExon	5331	'SLITRK5	SLIT and NTRK-like protein 5
GA	cg06152215	2.99E-09	9.04E-06	BF|FDR0.01	12	124422259	'DNAH10;CCDC92	dynein heavy chain 10, axonemal isoform 1;coiled-coil domain-containing pr	TES5000;TES5000;Body;Body;B	34347	'CCDC92	coiled-coil domain-containing protein 92
GA	cg06161540	8.38E-06	0.003043015	FDR0.01	16	1361806	'UBE2I	SUMO-conjugating enzyme UBC9	Body;Body;Body;Body	2170	'UBE2I	SUMO-conjugating enzyme UBC9
GA	cg06202737	2.98E-10	1.76E-06	BF|FDR0.01	2	128166279	'			-9716	'PROC	vitamin K-dependent protein C precursor
GA	cg06215107	2.31E-05	0.006126008	FDR0.01	4	151504725	'LRBA;LOC729558;MAB21L2	lipopolysaccharide-responsive and beige-like anchor protein isoform 4;uncha	Body;Body;Body;Body;TES5000	1423	'MAB21L2	protein mab-21-like 2
GA	cg06230948	2.97E-06	0.001449697	FDR0.01	6	33280518	'TAPBP;ZBTB22	tapasin precursor;zinc finger and BTB domain-containing protein 22	Body;Body;Body;TES5000;TES5	1337	'TAPBP	tapasin precursor
GA	cg06241374	5.56E-09	1.44E-05	BF|FDR0.01	2	56410071	'LOC100129434;CCDC85A	uncharacterized LOC100129434;coiled-coil domain-containing protein 85A	Body;TSS1500;TSS1500;TSS150	-1053	'CCDC85A	coiled-coil domain-containing protein 85A
GA	cg06277588	1.49E-05	0.004589668	FDR0.01	6	13489246	'GFOD1	glucose-fructose oxidoreductase domain-containing protein 1	TSS1500;TSS5000;TSS1500	-1377	'GFOD1	glucose-fructose oxidoreductase domain-containing protein 1
GA	cg06277849	6.90E-11	6.29E-07	BF|FDR0.01	4	186316674	'ANKRD37;UFSP2	ankyrin repeat domain-containing protein 37;ufm1-specific protease 2	TSS1500;TES5000;TES5000;TES!	-1320	'ANKRD37	ankyrin repeat domain-containing protein 37
GA	cg06285727	1.85E-08	3.23E-05	BF|FDR0.01	11	72524028	'ATG16L2	autophagy-related protein 16-2	TSS1500;TSS1500	-1427	'ATG16L2	autophagy-related protein 16-2
GA	cg06311778	8.18E-06	0.003003206	FDR0.01	12	53441582	'LOC283335;TNS2	uncharacterized LOC283335;tensin-2 isoform 3	Body;Body;TSS1500;TSS5000	808	'TNS2	tensin-2 isoform 3
GA	cg06362282	1.38E-07	0.000153917	FDR0.01	6	33245893	'RPS18;B3GALT4;WDR46	40S ribosomal protein S18;beta-1,3-galactosyltransferase 4;WD repeat-conta	TES5000;1stExon;TES5000;TES5	994	'B3GALT4	beta-1,3-galactosyltransferase 4
GA	cg06362313	2.86E-07	0.000264969	FDR0.01	12	6645287	'NCAPD2;GAPDH;IFFO1	condensin complex subunit 1;glyceraldehyde-3-phosphate dehydrogenase;ir	TES5000;Body;Body;Body;Body	858	'GAPDH	glyceraldehyde-3-phosphate dehydrogenase
GA	cg06375148	1.68E-05	0.004965664	FDR0.01	1	209958343	'TRAF3IP3;C1orf74;IRF6	TRAF3-interacting JNK-activating modulator;UPF0739 protein C1orf74;interfe	TES5000;TES5000;TES5000;TSS1	-439	'C1orf74	UPF0739 protein C1orf74
GA	cg06385087	1.64E-05	0.004880187	FDR0.01	20	57581903	'CTSZ	cathepsin Z precursor	Body	406	'CTSZ	cathepsin Z precursor
GA	cg06388418	3.08E-05	0.007359402	FDR0.01	16	27237190	'KDM8;NSMCE1	bifunctional peptidase and arginyl-hydroxylase JMJD5 isoform 1;non-structu	TES5000;TES5000;Body	21895	'KDM8	bifunctional peptidase and arginyl-hydroxylase JMJD5 isoform 1
GA	cg06396119	8.76E-06	0.003135806	FDR0.01	13	49792767	'MLNR	motilin receptor	TSS5000	-1706	'MLNR	motilin receptor
GA	cg06407111	5.56E-06	0.002285468	FDR0.01	17	73872650	'TRIM47	E3 ubiquitin-protein ligase TRIM47	Body	2012	'TRIM47	E3 ubiquitin-protein ligase TRIM47
GA	cg06438942	1.34E-05	0.004243425	FDR0.01	6	31805329	'SNHG32;SNORD48;SNORD52	small nucleolar RNA host gene 32;small nucleolar RNA, C/D box 48;small nuc	Body;Body;Body;Body;Body;Bo	477	'SNORD52	small nucleolar RNA, C/D box 52
GA	cg06457011	4.21E-05	0.009010672	FDR0.01	20	39767490	'PLCG1-AS1;PLCG1	PLCG1 antisense RNA 1;1-phosphatidylinositol 4,5-bisphosphate phosphodie	TSS1500;Body;Body	-850	'PLCG1-AS1	PLCG1 antisense RNA 1
GA	cg06457408	1.32E-05	0.00421052	FDR0.01	17	79827882	'ARHGDIA	rho GDP-dissociation inhibitor 1	Body;Body;Body;Body;Body;Bo	991	'ARHGDIA	rho GDP-dissociation inhibitor 1
GA	cg06460691	2.34E-05	0.006167008	FDR0.01	5	74162851	'FAM169A;LOC441086	soluble lamin-associated protein of 75 kDa;uncharacterized LOC441086	TSS200;TSS1500;Body	-60	'FAM169A	soluble lamin-associated protein of 75 kDa
GA	cg06469229	3.90E-06	0.001779247	FDR0.01	5	59188213	'PDE4D	cAMP-specific 3',5'-cyclic phosphodiesterase 4D isoform 12	Body;Body;Body;Body;Body;Bo	1340	'PDE4D	cAMP-specific 3',5'-cyclic phosphodiesterase 4D isoform 12
GA	cg06473363	8.23E-09	1.88E-05	BF|FDR0.01	6	31631797	'C6orf47;GPANK1;CSNK2B	uncharacterized protein C6orf47;G patch domain and ankyrin repeat-contain	TSS5000;1stExon;1stExon;1stEx	977	'GPANK1	G patch domain and ankyrin repeat-containing protein 1
GA	cg06484274	1.29E-05	0.0041292	FDR0.01	3	142164840	'XRN1;ATR	chromatin-binding exonuclease XRN1;serine/threonine-protein kinase ATR is	Body;Body;Body;TES5000;TES5	2039	'XRN1	chromatin-binding exonuclease XRN1
GA	cg06485521	7.31E-06	0.002768733	FDR0.01	6	53530600	'KLHL31	kelch-like protein 31	TSS200	-46	'KLHL31	kelch-like protein 31
GA	cg06492744	1.09E-08	2.29E-05	BF|FDR0.01	11	65406254	'PCNX3;MIR4690;SIPA1	pecanex-like protein 3;microRNA 4690;signal-induced proliferation-associate	TES5000;TES5000;Body;TSS150	683	'SIPA1	signal-induced proliferation-associated protein 1
GA	cg06518251	1.90E-07	0.000194151	FDR0.01	11	67776030	'UNC93B1;ALDH3B1	protein unc-93 homolog B1;aldehyde dehydrogenase family 3 member B1	TSS5000;5'UTR;TSS5000;TSS500	14	'ALDH3B1	aldehyde dehydrogenase family 3 member B1
GA	cg06533408	4.35E-07	0.00036589	FDR0.01	12	69139618	'NUP107;SLC35E3	nuclear pore complex protein Nup107;solute carrier family 35 member E3	TES5000;TES5000;TSS1500;TSS1	-337	'SLC35E3	solute carrier family 35 member E3
GA	cg06538684	2.91E-06	0.001430304	FDR0.01	12	12511223	'LOH12CR2;BORCS5	loss of heterozygosity on chromosome 12, region 2;BLOC-1-related complex	TSS1500;Body;Body;Body	1212	'BORCS5	BLOC-1-related complex subunit 5
GA	cg06540458	3.14E-07	0.000287045	FDR0.01	1	226951747	'			-24871	'ITPKB	inositol-trisphosphate 3-kinase B
GA	cg06569117	2.95E-05	0.007183669	FDR0.01	2	217657537	'			67250	'TNP1	spermatid nuclear transition protein 1
GA	cg06605053	2.13E-05	0.00584688	FDR0.01	15	93424933	'LINC01578		TSS1500;TSS1500;TSS5000	-1139	'LINC01578	
GA	cg06623899	2.43E-05	0.006322573	FDR0.01	8	142161840	'DENND3	DENN domain-containing protein 3 isoform 1	Body;Body;Body;Body	23153	'DENND3	DENN domain-containing protein 3 isoform 1
GA	cg06627532	3.12E-05	0.007406807	FDR0.01	16	81491312	'CMIP	C-Maf-inducing protein isoform Tc-Mip	Body	12538	'CMIP	C-Maf-inducing protein isoform Tc-Mip
GA	cg06635797	1.51E-05	0.004635393	FDR0.01	19	4915394	'UHRF1	E3 ubiquitin-protein ligase UHRF1	Body;Body;Body;Body;Body	5016	'UHRF1	E3 ubiquitin-protein ligase UHRF1
GA	cg06647068	4.10E-10	2.21E-06	BF|FDR0.01	12	104853274	'CHST11	carbohydrate sulfotransferase 11	Body;Body	2549	'CHST11	carbohydrate sulfotransferase 11
GA	cg06650246	9.67E-06	0.003346066	FDR0.01	1	206897270	'MAPKAPK2	MAP kinase-activated protein kinase 2 isoform 1	Body;Body	39021	'MAPKAPK2	MAP kinase-activated protein kinase 2 isoform 1
GA	cg06652329	2.59E-06	0.001310889	FDR0.01	12	102874566	'IGF1	insulin-like growth factor I	TSS5000;TSS1500;TSS1500;TSS1	-225	'IGF1	insulin-like growth factor I
GA	cg06664254	1.93E-07	0.000195823	FDR0.01	16	75628226	'ADAT1	tRNA-specific adenosine deaminase 1	TES5000;TES5000;TES5000;TES!	27950	'GABARAPL2	gamma-aminobutyric acid receptor-associated protein-like 2
GA	cg06670599	7.09E-11	6.34E-07	BF|FDR0.01	6	31631801	'C6orf47;GPANK1;CSNK2B	uncharacterized protein C6orf47;G patch domain and ankyrin repeat-contain	TSS5000;1stExon;1stExon;1stEx	973	'GPANK1	G patch domain and ankyrin repeat-containing protein 1
GA	cg06679265	4.57E-05	0.009477579	FDR0.01	12	57040204	'ATP5F1B;SNORD59B;SNORD59A	ATP synthase subunit beta, mitochondrial;small nucleolar RNA, C/D box 59B;	TSS1500;TSS5000;TSS1500	-436	'ATP5F1B	ATP synthase subunit beta, mitochondrial
GA	cg06685437	1.79E-06	0.001026044	FDR0.01	17	17669973	'RAI1;RAI1-AS1	decapping nuclease;RAI1 antisense RNA 1	Body;TSS1500	-357	'RAI1-AS1	RAI1 antisense RNA 1
GA	cg06689372	2.30E-05	0.006110204	FDR0.01	3	58645085	'FAM3D	protein FAM3D precursor	Body	7416	'FAM3D	protein FAM3D precursor
GA	cg06692785	4.55E-05	0.009460437	FDR0.01	1	180882681	'KIAA1614	uncharacterized protein KIAA1614	Body	649	'KIAA1614	uncharacterized protein KIAA1614
GA	cg06713098	4.67E-05	0.009616031	FDR0.01	7	45960367	'IGFBP3	insulin-like growth factor-binding protein 3	1stExon;1stExon	504	'IGFBP3	insulin-like growth factor-binding protein 3

Trait	#cpg	p	fdr	significance	chr	pos.hg19	gene.Illumina	gene.description	locations	dist2TSS	gene.nearest	gene.description
GA	cg06715352	9.17E-06	0.003230315	FDR0.01	16	66729204	'CMTM4	CKLF-like MARVEL transmembrane domain-containing protein 4	Body;Body	1442	'CMTM4	CKLF-like MARVEL transmembrane domain-containing protein 4
GA	cg06720361	5.78E-06	0.002348897	FDR0.01	16	85202391	'			31636	'LINC02139	long intergenic non-protein coding RNA 2139
GA	cg06724402	4.43E-05	0.009280449	FDR0.01	11	101783583	'ANGPTL5;CEP126	angiopoietin-related protein 5 precursor;centrosomal protein of 126 kDa iso	Body;TSS5000;TSS5000	-2157	'CEP126	centrosomal protein of 126 kDa isoform 1
GA	cg06730678	9.19E-06	0.003230859	FDR0.01	12	6166943	'VWF	von Willebrand factor precursor	Body	-17594	'SNORA120	small nucleolar RNA, H/ACA box 120
GA	cg06732666	1.63E-05	0.004862178	FDR0.01	2	208890496	'PLEKHM3	pleckstrin homology domain-containing family M member 3	TSS1500	-245	'PLEKHM3	pleckstrin homology domain-containing family M member 3
GA	cg06740227	8.97E-06	0.003177628	FDR0.01	12	86229804	'RASSF9	ras association domain-containing protein 9	Body	383	'RASSF9	ras association domain-containing protein 9
GA	cg06740296	7.45E-10	3.20E-06	BF|FDR0.01	1	205561401	'MFSD4A	major facilitator superfamily domain-containing protein 4A	Body	-22968	'MFSD4A-AS1	MFSD4A antisense RNA 1
GA	cg06753281	1.28E-11	1.91E-07	BF|FDR0.01	1	6086652	'KCNAB2	voltage-gated potassium channel subunit beta-2 isoform 1	Body;Body;Body	260	'KCNAB2	voltage-gated potassium channel subunit beta-2 isoform 1
GA	cg06759518	3.37E-05	0.00779633	FDR0.01	3	72496799	'RYBP	ring and YY1 binding protein, isoform A	TSS1500	-1025	'RYBP	ring and YY1 binding protein, isoform A
GA	cg06778024	2.91E-06	0.001431542	FDR0.01	16	89408567	'ANKRD11	ankyrin repeat domain-containing protein 11	Body;Body;Body;Body	21027	'LOC100287036	uncharacterized LOC100287036
GA	cg06784563	4.11E-05	0.008870684	FDR0.01	18	77284509	'NFATC1	nuclear factor of activated T-cells, cytoplasmic 1 isoform D	Body;Body;Body;Body;Body;Bo	-54772	'LOC284240	uncharacterized LOC284240
GA	cg06789445	1.57E-05	0.004742789	FDR0.01	3	69942668	'MITF	microphthalmia-associated transcription factor	Body;Body;Body;Body;Body;Bo	27294	'MITF	microphthalmia-associated transcription factor
GA	cg06800565	1.76E-07	0.00018465	FDR0.01	1	161124156	'UFC1	ubiquitin-fold modifier-conjugating enzyme 1	Body	389	'UFC1	ubiquitin-fold modifier-conjugating enzyme 1
GA	cg06813862	3.81E-05	0.00850805	FDR0.01	12	89346059	'			67410	'LINC02458	long intergenic non-protein coding RNA 2458
GA	cg06814287	4.48E-06	0.001950161	FDR0.01	6	32120584	'PRRT1;LOC100507547;PPT2;PPT2-EGFL8	proline-rich transmembrane protein 1;uncharacterized LOC100507547;lysos	Body;TSS1500;Body;Body;Body	276	'PRRT1	proline-rich transmembrane protein 1
GA	cg06815950	8.87E-09	1.99E-05	BF|FDR0.01	12	111619455	'CUX2	homeobox protein cut-like 2 isoform 2	Body;Body	81648	'CUX2	homeobox protein cut-like 2 isoform 2
GA	cg06825448	7.85E-10	3.34E-06	BF|FDR0.01	2	240884925	'NDUFA10;MIR4786	NADH dehydrogenase [ubiquinone] 1 alpha subcomplex subunit 10, mitocho	Body;TSS5000	-2414	'MIR4786	microRNA 4786
GA	cg06832687	4.21E-06	0.001873061	FDR0.01	6	32122753	'PRRT1;LOC100507547;PPT2;PPT2-EGFL8	proline-rich transmembrane protein 1;uncharacterized LOC100507547;lysos	TSS5000;TSS5000;TSS1500;TSS	-611	'LOC100507547	uncharacterized LOC100507547
GA	cg06850526	4.18E-06	0.001864837	FDR0.01	17	79269886	'SLC38A10	putative sodium-coupled neutral amino acid transporter 10	TSS1500;TSS1500	-779	'SLC38A10	putative sodium-coupled neutral amino acid transporter 10
GA	cg06853339	4.34E-05	0.009167231	FDR0.01	17	76117687	'TMC6	transmembrane channel-like protein 6	Body;Body;Body	7188	'TMC6	transmembrane channel-like protein 6
GA	cg06869171	8.22E-07	0.000584641	FDR0.01	1	155949839	'ARHGEF2	rho guanine nucleotide exchange factor 2	TSS5000;TSS5000;TSS5000;TSS	-488	'ARHGEF2	rho guanine nucleotide exchange factor 2
GA	cg06870470	1.88E-07	0.000192766	FDR0.01	19	11315767	'DOCK6;LOC105372273	dedicator of cytokinesis protein 6;uncharacterized LOC105372273	Body;Body	1464	'LOC105372273	uncharacterized LOC105372273
GA	cg06875488	2.49E-05	0.006441228	FDR0.01	4	7657070	'SORCS2	VPS10 domain-containing receptor SorCS2 precursor	Body	-98746	'AFAP1-AS1	AFAP1 antisense RNA 1
GA	cg06888698	6.77E-06	0.002621703	FDR0.01	14	100204149	'			53515	'CYP46A1	cholesterol 24-hydroxylase
GA	cg06893273	2.34E-06	0.001240403	FDR0.01	17	46604774	'HOXB1	homeobox protein Hox-B1	TES5000	3599	'HOXB1	homeobox protein Hox-B1
GA	cg06895640	4.61E-07	0.000382145	FDR0.01	11	93277191	'SMCO4	single-pass membrane and coiled-coil domain-containing protein 4	TSS1500	-634	'SMCO4	single-pass membrane and coiled-coil domain-containing protein 4
GA	cg06896207	2.06E-07	0.000207065	FDR0.01	19	2095364	'MOB3A;IZUMO4	MOB kinase activator 3A;izumo sperm-egg fusion protein 4 precursor	Body;TSS5000;TSS5000;TSS500	961	'MOB3A	MOB kinase activator 3A
GA	cg06897661	1.77E-09	6.44E-06	BF|FDR0.01	16	50322074	'ADCY7;MIR6771	adenylate cyclase type 7;microRNA 6771	5'UTR;5'UTR;TSS5000	-4452	'MIR6771	microRNA 6771
GA	cg06907680	1.16E-05	0.003814595	FDR0.01	14	91580129	'DGLUCY	D-glutamate cyclase, mitochondrial	Body;Body;Body;TSS1500;TSS1	-227	'DGLUCY	D-glutamate cyclase, mitochondrial
GA	cg06912623	3.15E-05	0.007442776	FDR0.01	1	224781135	'CNIH3	protein cornichon homolog 3	Body;Body;Body;Body;Body;Bo	-22883	'CNIH3	protein cornichon homolog 3
GA	cg06912918	2.25E-05	0.006019803	FDR0.01	2	69171302	'GKN2	gastrokine-2 precursor	TES5000	8723	'GKN2	gastrokine-2 precursor
GA	cg06937927	1.31E-07	0.000148266	FDR0.01	8	17979647	'			-37177	'ASAH1	acid ceramidase precursor
GA	cg06960794	2.06E-07	0.000207065	FDR0.01	6	149022017	'			-46148	'UST	uronyl-2-sulfotransferase
GA	cg06964027	1.91E-05	0.005434268	FDR0.01	11	1073496	'MUC2	mucin-2 precursor	TSS1500	-1378	'MUC2	mucin-2 precursor
GA	cg06968912	2.95E-06	0.00144175	FDR0.01	17	40573643	'CAVIN1	caveolae-associated protein 1 isoform 1	Body	1631	'CAVIN1	caveolae-associated protein 1 isoform 1
GA	cg06994420	5.68E-06	0.002326883	FDR0.01	2	66672553	'MEIS1	homeobox protein Meis1	Body	-5091	'MEIS1-AS2	MEIS1 antisense RNA 2
GA	cg07006935	4.21E-06	0.001873061	FDR0.01	7	55620641	'VOPP1	vesicular, overexpressed in cancer, prosurvival protein 1 isoform 1 precursor	Body;Body;Body;TSS200;Body;E	-182	'VOPP1	vesicular, overexpressed in cancer, prosurvival protein 1 isoform 1 precursor
GA	cg07031797	6.72E-06	0.002611536	FDR0.01	22	50483123	'TTLL8	protein monoglycylase TTLL8	Body	13533	'TTLL8	protein monoglycylase TTLL8
GA	cg07040244	1.39E-05	0.004351996	FDR0.01	1	231555522	'EGLN1	egl nine homolog 1	Body	5268	'EGLN1	egl nine homolog 1
GA	cg07053697	1.61E-05	0.004838912	FDR0.01	12	111374835	'LINC01405	long intergenic non-protein coding RNA 1405	Body	430	'LINC01405	long intergenic non-protein coding RNA 1405
GA	cg07063068	1.03E-05	0.003507333	FDR0.01	14	91711033	'GPR68	ovarian cancer G-protein coupled receptor 1	5'UTR;Body;Body	15	'GPR68	ovarian cancer G-protein coupled receptor 1
GA	cg07069090	1.74E-05	0.005123971	FDR0.01	2	170148834	'LRP2	low-density lipoprotein receptor-related protein 2 precursor	Body	70210	'LRP2	low-density lipoprotein receptor-related protein 2 precursor
GA	cg07076751	1.08E-07	0.000129809	BF|FDR0.01	6	30647539	'PPP1R18	phostensin	Body;Body	-6709	'DHX16	pre-mRNA-splicing factor ATP-dependent RNA helicase DHX16
GA	cg07080372	1.31E-05	0.004174786	FDR0.01	11	796607	'SLC25A22;PANO1;PIDD1	mitochondrial glutamate carrier 1;proapoptotic nucleolar protein 1;p53-indu	TSS1500;Body;Body;TSS1500;TI	-369	'SLC25A22	mitochondrial glutamate carrier 1
GA	cg07090356	1.61E-07	0.000171077	FDR0.01	18	23668764	'SS18	protein SSXT	Body;Body;Body	1813	'SS18	protein SSXT
GA	cg07106169	1.47E-09	5.53E-06	BF|FDR0.01	2	95740362	'			46940	'MRPS5	mitochondrial 37S ribosomal protein MRPS5
GA	cg07106927	1.37E-06	0.000846846	FDR0.01	1	160769081	'LY9	T-lymphocyte surface antigen Ly-9	Body;Body;Body;Body	3118	'LY9	T-lymphocyte surface antigen Ly-9
GA	cg07131742	6.23E-07	0.000476672	FDR0.01	11	66112880	'BRMS1;B4GAT1;LOC102724064	breast cancer metastasis-suppressor 1 homolog;beta-1,4-glucuronyltransfera	TSS1500;TSS1500;3'UTR;TSS500	-305	'BRMS1	breast cancer metastasis-suppressor 1 homolog
GA	cg07140459	1.04E-07	0.000127255	BF|FDR0.01	3	142165032	'XRN1;ATR	chromatin-binding exonuclease XRN1;serine/threonine-protein kinase ATR is	Body;Body;Body;TES5000;TES5	1847	'XRN1	chromatin-binding exonuclease XRN1
GA	cg07161369	1.54E-07	0.000166437	FDR0.01	7	38350464	'			-30713	'TRG-AS1	T cell receptor gamma locus antisense RNA 1
GA	cg07163845	1.43E-05	0.004449935	FDR0.01	14	106071651	'			-15801	'MIR8071-1	microRNA 8071-1
GA	cg07167185	6.06E-06	0.002430299	FDR0.01	1	24120017	'LYPLA2;GALE	acyl-protein thioesterase 2;UDP-glucose 4-epimerase	Body;TES5000;TES5000;TES500	2383	'LYPLA2	acyl-protein thioesterase 2
GA	cg07175580	1.92E-06	0.001072185	FDR0.01	1	206971605	'IL19	interleukin-19 isoform 1	Body	-25766	'IL10	interleukin-10 precursor
GA	cg07207490	8.89E-09	1.99E-05	BF|FDR0.01	11	118492463	'PHLDB1	pleckstrin homology-like domain family B member 1	Body;Body;Body	14134	'PHLDB1	pleckstrin homology-like domain family B member 1
GA	cg07207652	1.71E-05	0.005053754	FDR0.01	17	45798257	'			-12344	'TBX21	T-box transcription factor TBX21
GA	cg07217350	8.98E-07	0.000618561	FDR0.01	5	162934022	'MAT2B	methionine adenosyltransferase 2 subunit beta	Body;Body	1398	'MAT2B	methionine adenosyltransferase 2 subunit beta
GA	cg07238066	1.95E-08	3.33E-05	BF|FDR0.01	11	65188862	'NEAT1	nuclear paraspeckle assembly transcript 1	TSS1500;TSS1500	-1406	'NEAT1	nuclear paraspeckle assembly transcript 1
GA	cg07250128	4.43E-05	0.009281622	FDR0.01	3	194833983	'XXYLT1	xyloside xylosyltransferase 1	Body;Body	18667	'XXYLT1-AS1	XXYLT1 antisense RNA 1
GA	cg07250758	3.35E-07	0.000302322	FDR0.01	11	61049251	'VWCE	von Willebrand factor C and EGF domain-containing protein precursor	Body	13537	'VWCE	von Willebrand factor C and EGF domain-containing protein precursor
GA	cg07258838	3.85E-05	0.008554866	FDR0.01	6	116867864	'TRAPPC3L;CALHM4	trafficking protein particle complex subunit 3-like protein;calcium homeosta	TSS1500;Body;Body;Body;Body	-1017	'TRAPPC3L	trafficking protein particle complex subunit 3-like protein
GA	cg07285708	1.31E-05	0.004188076	FDR0.01	3	10291324	'TATDN2	putative deoxyribonuclease TATDN2	Body	1182	'TATDN2	putative deoxyribonuclease TATDN2
GA	cg07288557	3.91E-05	0.008636129	FDR0.01	12	47226301	'			-6521	'SLC38A4	sodium-coupled neutral amino acid transporter 4
GA	cg07306253	3.59E-10	2.03E-06	BF|FDR0.01	15	41062224	'GCHFR;DNAJC17;C15orf62	GTP cyclohydrolase 1 feedback regulatory protein;dnaJ homolog subfamily C	TES5000;Body;5'UTR	47	'C15orf62	uncharacterized protein C15orf62, mitochondrial precursor
GA	cg07313882	1.73E-08	3.13E-05	BF|FDR0.01	17	76121348	'TMC6	transmembrane channel-like protein 6	Body;Body;Body	3527	'TMC6	transmembrane channel-like protein 6
GA	cg07320695	1.15E-07	0.000134593	FDR0.01	4	107423690	'			68910	'LINC02173	long intergenic non-protein coding RNA 2173
GA	cg07324381	1.21E-08	2.43E-05	BF|FDR0.01	9	139222968	'DKFZP434A062;GPSM1	uncharacterized LOC26102;G-protein-signaling modulator 1 isoform c	TSS1500;Body;Body	974	'GPSM1	G-protein-signaling modulator 1 isoform c
GA	cg07342647	4.03E-05	0.008762455	FDR0.01	16	9060697	'USP7	ubiquitin carboxyl-terminal hydrolase 7	TSS5000	-2934	'USP7	ubiquitin carboxyl-terminal hydrolase 7
GA	cg07349815	7.83E-06	0.002904002	FDR0.01	3	123751269	'			-40252	'ROPN1	ropporin-1A
GA	cg07354583	4.84E-06	0.002060624	FDR0.01	12	9911289	'CD69	early activation antigen CD69	Body	2208	'CD69	early activation antigen CD69
GA	cg07363144	4.06E-05	0.008802751	FDR0.01	1	2796691	'			-79258	'TTC34	tetratricopeptide repeat protein 34
GA	cg07367469	4.14E-05	0.008906906	FDR0.01	5	115988245	'			-77726	'SEMA6A	semaphorin-6A isoform 2 precursor
GA	cg07377178	2.14E-05	0.005860696	FDR0.01	6	3025064	'NQO2;HTATSF1P2;LOC101927759	ribosyldihydronicotinamide dehydrogenase [quinone];HIV-1 Tat specific fact	TES5000;TES5000;TES5000;TSS	768	'LOC101927759	uncharacterized LOC101927759
GA	cg07387383	2.17E-05	0.005892124	FDR0.01	3	194722955	'			-92361	'XXYLT1-AS1	XXYLT1 antisense RNA 1
GA	cg07405370	2.71E-06	0.001353796	FDR0.01	20	62528249	'DNAJC5	dnaJ homolog subfamily C member 5	Body	1771	'DNAJC5	dnaJ homolog subfamily C member 5
GA	cg07408552	1.57E-14	1.26E-09	BF|FDR0.01	17	40611995	'ATP6V0A1	V-type proton ATPase 116 kDa subunit a1	Body;Body;Body	1100	'ATP6V0A1	V-type proton ATPase 116 kDa subunit a1
GA	cg07419740	3.46E-06	0.001615726	FDR0.01	5	172410441	'ATP6V0E1	V-type proton ATPase subunit e 1	TSS1500	-332	'ATP6V0E1	V-type proton ATPase subunit e 1
GA	cg07429038	4.42E-05	0.009280449	FDR0.01	7	138482826	'ATP6V0A4;TMEM213	V-type proton ATPase 116 kDa subunit a isoform 4;transmembrane protein 2	5'UTR;5'UTR;5'UTR	88	'TMEM213	transmembrane protein 213 precursor
GA	cg07436701	3.94E-05	0.008659885	FDR0.01	10	88717623	'MMRN2;SNCG	multimerin-2 precursor;gamma-synuclein	TSS200;Body;TSS1500	-198	'MMRN2	multimerin-2 precursor
GA	cg07443287	4.75E-06	0.002028977	FDR0.01	12	1739687	'WNT5B	protein Wnt-5b precursor	Body;Body	1291	'WNT5B	protein Wnt-5b precursor
GA	cg07450210	2.44E-09	7.97E-06	BF|FDR0.01	13	99880947	'UBAC2	ubiquitin-associated domain-containing protein 2	Body;Body;Body	27840	'UBAC2	ubiquitin-associated domain-containing protein 2
GA	cg07450633	9.23E-07	0.000632311	FDR0.01	5	172175321	'			-7184	'LOC101928093	uncharacterized LOC101928093
GA	cg07454620	2.72E-05	0.006810712	FDR0.01	17	27070948	'NEK8;TRAF4	serine/threonine-protein kinase Nek8;TNF receptor-associated factor 4	TES5000;TSS200	-80	'TRAF4	TNF receptor-associated factor 4
GA	cg07470708	2.28E-05	0.006086474	FDR0.01	19	49564932	'NTF4	neurotrophin-4 precursor	Body	2192	'NTF4	neurotrophin-4 precursor
GA	cg07488576	1.42E-07	0.000156287	FDR0.01	2	192711481	'CAVIN2	caveolae-associated protein 2	1stExon	333	'CAVIN2	caveolae-associated protein 2
GA	cg07491676	6.75E-06	0.002615681	FDR0.01	15	76400783	'TMEM266	transmembrane protein 266	Body	48458	'TMEM266	transmembrane protein 266
GA	cg07492045	1.96E-05	0.005516506	FDR0.01	5	180257795	'HEIH;LINC00847	hepatocellular carcinoma up-regulated EZH2-associated long non-coding RN	Body;TSS200;TSS200;TSS200	-161	'LINC00847	long intergenic non-protein coding RNA 847

Trait	#cpg	p	fdr	significance	chr	pos.hg19	gene.Illumina	gene.description	locations	dist2TSS	gene.nearest	gene.description
GA	cg07504977	1.63E-11	2.21E-07	BF|FDR0.01	10	102131012	'OLMALINC	oligodendrocyte maturation-associated long intergenic non-coding RNA	TSS5000	-2320	'OLMALINC	oligodendrocyte maturation-associated long intergenic non-coding RNA
GA	cg07544356	2.48E-05	0.006424098	FDR0.01	19	58716517	'ZNF274	neurotrophin receptor-interacting factor homolog isoform d	Body;Body;Body;Body	22090	'ZNF274	neurotrophin receptor-interacting factor homolog isoform d
GA	cg07565956	2.02E-05	0.005638907	FDR0.01	17	7381288	'ZBTB4;SLC35G6	zinc finger and BTB domain-containing protein 4;solute carrier family 35 mer	Body;Body;TSS5000	1662	'ZBTB4	zinc finger and BTB domain-containing protein 4
GA	cg07577569	4.40E-05	0.009252778	FDR0.01	20	40245965	'CHD6	chromodomain-helicase-DNA-binding protein 6	Body	1051	'CHD6	chromodomain-helicase-DNA-binding protein 6
GA	cg07588779	4.01E-05	0.00874487	FDR0.01	11	64051753	'BAD;GPR137	bcl2-associated agonist of cell death;integral membrane protein GPR137	1stExon;1stExon;TSS200;TSS15	-57	'GPR137	integral membrane protein GPR137
GA	cg07599979	9.27E-09	2.02E-05	BF|FDR0.01	21	38592612	'DSCR9;VPS26C	Down syndrome critical region 9;vacuolar protein sorting-associated protein	Body;TES5000;TES5000;TES500	11809	'DSCR9	Down syndrome critical region 9
GA	cg07639694	8.44E-06	0.003053789	FDR0.01	17	7254909	'ACAP1;KCTD11;TMEM95	arf-GAP with coiled-coil, ANK repeat and PH domain-containing protein 1;BT	TES5000;TSS1500;TSS1500;TSS	-298	'KCTD11	BTB/POZ domain-containing protein KCTD11
GA	cg07656173	5.52E-06	0.002276437	FDR0.01	4	16086023	'PROM1	prominin-1 precursor	TSS1500;TSS1500;TSS1500;TSS	-377	'PROM1	prominin-1 precursor
GA	cg07667560	4.00E-06	0.001816177	FDR0.01	19	42637805	'POU2F2;LOC100505622;MIR4323	POU domain, class 2, transcription factor 2 isoform 1;;microRNA 4323	TSS1500;TSS1500;TSS1500;TSS	-140	'MIR4323	microRNA 4323
GA	cg07675998	3.94E-08	5.85E-05	BF|FDR0.01	11	64268315	'			51787	'LINC02724	long intergenic non-protein coding RNA 2724
GA	cg07706367	1.34E-06	0.000829528	FDR0.01	4	3342725	'RGS12	regulator of G-protein signaling 12 isoform 3	Body;Body	26852	'RGS12	regulator of G-protein signaling 12 isoform 3
GA	cg07712198	7.00E-07	0.00051856	FDR0.01	5	9015582	'			38425	'MIR4636	microRNA 4636
GA	cg07718813	3.27E-05	0.007632873	FDR0.01	1	6640976	'TAS1R1;ZBTB48	taste receptor type 1 member 1 isoform c precursor;telomere zinc finger-ass	TES5000;TES5000;1stExon;1stE	923	'ZBTB48	telomere zinc finger-associated protein
GA	cg07739369	3.88E-08	5.78E-05	BF|FDR0.01	17	80241456	'			9234	'LINC01970	long intergenic non-protein coding RNA 1970
GA	cg07743764	5.49E-13	1.75E-08	BF|FDR0.01	11	62559882	'TMEM179B;MIR6748;TMEM223;NXF1;MIR6514	transmembrane protein 179B precursor;microRNA 6748;transmembrane pro	TES5000;TES5000;TES5000;TES	361	'MIR6514	microRNA 6514
GA	cg07749613	1.70E-06	0.000987446	FDR0.01	2	97073539	'			72036	'NCAPH	condensin complex subunit 2
GA	cg07749664	2.92E-06	0.001432923	FDR0.01	13	73634670	'KLF5	Krueppel-like factor 5	Body;Body	1528	'KLF5	Krueppel-like factor 5
GA	cg07788865	6.48E-19	2.90E-13	BF|FDR0.01	10	30101042	'			-75177	'SVIL	supervillin
GA	cg07794010	2.13E-05	0.00584688	FDR0.01	1	114665617	'SYT6	synaptotagmin-6 isoform b	Body;Body;Body;Body;Body;Bo	29445	'SYT6	synaptotagmin-6 isoform b
GA	cg07799005	8.10E-06	0.00297989	FDR0.01	11	65265684	'MALAT1;TALAM1	metastasis associated lung adenocarcinoma transcript 1;TALAM1 transcript,	Body;Body;Body;TES5000	476	'MALAT1	metastasis associated lung adenocarcinoma transcript 1
GA	cg07807026	1.35E-05	0.004252885	FDR0.01	12	2041581	'LINC00940	long intergenic non-protein coding RNA 940	Body	4161	'LINC00940	long intergenic non-protein coding RNA 940
GA	cg07815836	6.86E-06	0.002650885	FDR0.01	19	58715351	'ZNF274	neurotrophin receptor-interacting factor homolog isoform d	Body;Body;Body;Body	20924	'ZNF274	neurotrophin receptor-interacting factor homolog isoform d
GA	cg07816074	2.27E-06	0.001216182	FDR0.01	4	8201560	'SH3TC1	SH3 domain and tetratricopeptide repeat-containing protein 1	Body;Body;Body	544	'SH3TC1	SH3 domain and tetratricopeptide repeat-containing protein 1
GA	cg07817400	3.18E-05	0.007478357	FDR0.01	6	33172817	'RXRB;SLC39A7;HSD17B8;MIR219A1;RING1	retinoic acid receptor RXR-beta;zinc transporter SLC39A7 precursor;estradio	TSS5000;TSS5000;TSS5000;TES	386	'HSD17B8	estradiol 17-beta-dehydrogenase 8
GA	cg07825294	1.36E-08	2.68E-05	BF|FDR0.01	19	1415757	'DAZAP1	DAZ-associated protein 1	Body;Body;Body;Body	8173	'DAZAP1	DAZ-associated protein 1
GA	cg07835443	3.89E-10	2.12E-06	BF|FDR0.01	16	89734986	'SPATA33	spermatogenesis-associated protein 33 isoform b	Body;Body;Body;Body;Body;Bo	10511	'SPATA33	spermatogenesis-associated protein 33 isoform b
GA	cg07844828	8.80E-06	0.003140419	FDR0.01	4	83680040	'SCD5	stearoyl-CoA desaturase 5	Body;Body	-5457	'MIR575	microRNA 575
GA	cg07850154	1.85E-05	0.005323892	FDR0.01	5	63461232	'RNF180	E3 ubiquitin-protein ligase RNF180	TSS1500;TSS1500;TSS1500;TSS	-437	'RNF180	E3 ubiquitin-protein ligase RNF180
GA	cg07900197	1.06E-06	0.000704611	FDR0.01	2	149873848	'KIF5C	kinesin heavy chain isoform 5C	Body;Body	-21132	'LYPD6B	ly6/PLAUR domain-containing protein 6B precursor
GA	cg07904475	1.45E-08	2.80E-05	BF|FDR0.01	4	151500931	'LRBA;LOC729558;MAB21L2	lipopolysaccharide-responsive and beige-like anchor protein isoform 4;uncha	Body;Body;Body;Body;Body;TS	691	'LOC729558	uncharacterized LOC729558
GA	cg07905444	2.64E-05	0.006679232	FDR0.01	6	17101970	'STMND1	stathmin domain-containing protein 1	TSS1500	-310	'STMND1	stathmin domain-containing protein 1
GA	cg07912689	2.80E-05	0.006932937	FDR0.01	20	56195458	'ZBP1	Z-DNA-binding protein 1	5'UTR;5'UTR;5'UTR;Body;5'UTR	24	'ZBP1	Z-DNA-binding protein 1
GA	cg07932300	6.47E-10	2.92E-06	BF|FDR0.01	6	139454928	'HECA	headcase protein homolog	TSS1500	-1288	'HECA	headcase protein homolog
GA	cg07941983	8.11E-08	0.000103574	BF|FDR0.01	4	36245138	'ARAP2;LOC439933	arf-GAP with Rho-GAP domain, ANK repeat and PH domain-containing prote	Body;Body;Body;TSS1500	423	'ARAP2	arf-GAP with Rho-GAP domain, ANK repeat and PH domain-containing protein 2
GA	cg07946633	7.30E-07	0.000532555	FDR0.01	1	2984245	'PRDM16-DT;PRDM16	PRDM16 divergent transcript;histone-lysine N-methyltransferase PRDM16 is	Body;Body;TSS5000;TSS5000	44	'PRDM16-DT	PRDM16 divergent transcript
GA	cg07952905	6.20E-07	0.000474775	FDR0.01	17	27275352	'PHF12;LOC101927018	PHD finger protein 12;uncharacterized LOC101927018	Body;Body;Body;TES5000	3184	'PHF12	PHD finger protein 12
GA	cg07954706	3.98E-05	0.008713473	FDR0.01	1	110164547	'AMPD2	AMP deaminase 2	Body;Body;Body;Body;TSS5000	784	'AMPD2	AMP deaminase 2
GA	cg07985890	2.45E-06	0.001269028	FDR0.01	11	65190999	'NEAT1	nuclear paraspeckle assembly transcript 1	Body;Body	731	'NEAT1	nuclear paraspeckle assembly transcript 1
GA	cg07988060	4.72E-05	0.009666218	FDR0.01	17	30234789	'			-6062	'UTP6	snoRNA-binding rRNA-processing protein UTP6
GA	cg08002381	7.60E-06	0.002844935	FDR0.01	9	86596124	'HNRNPK;RMI1	heterogeneous nuclear ribonucleoprotein K;recQ-mediated genome instabili	TSS1500;TSS1500;TSS1500;TSS	405	'RMI1	recQ-mediated genome instability protein 1
GA	cg08004014	2.20E-07	0.00021733	FDR0.01	17	4521010	'LINC01996	long intergenic non-protein coding RNA 1996	TES5000	7267	'LINC01996	long intergenic non-protein coding RNA 1996
GA	cg08005460	8.73E-07	0.00060623	FDR0.01	17	38473382	'RARA	retinoic acid receptor alpha	Body;TSS1500;TSS1500	-1114	'RARA	retinoic acid receptor alpha
GA	cg08009275	2.71E-09	8.46E-06	BF|FDR0.01	17	79790191	'MCRIP1;PPP1R27	mapk-regulated corepressor-interacting protein 1;protein phosphatase 1 reg	Body;Body;Body;Body;TES5000	976	'MCRIP1	mapk-regulated corepressor-interacting protein 1
GA	cg08016363	4.24E-06	0.00187778	FDR0.01	17	21118577	'TMEM11	transmembrane protein 11, mitochondrial	TSS1500;TSS1500	-669	'TMEM11	transmembrane protein 11, mitochondrial
GA	cg08024114	2.31E-05	0.006126008	FDR0.01	19	55865076	'COX6B2;FAM71E2	cytochrome c oxidase subunit 6B2;protein FAM71E2	3'UTR;3'UTR;3'UTR;3'UTR;TES5	1011	'COX6B2	cytochrome c oxidase subunit 6B2
GA	cg08025954	3.23E-09	9.56E-06	BF|FDR0.01	22	24823455	'SPECC1L-ADORA2A;ADORA2A;ADORA2A-AS1	SPECC1L-ADORA2A readthrough (NMD candidate);adenosine receptor A2a;A	Body;Body;TSS200;TSS200;TSS2	-111	'ADORA2A	adenosine receptor A2a
GA	cg08039560	1.44E-09	5.46E-06	BF|FDR0.01	21	45575832	'LINC01678	long intergenic non-protein coding RNA 1678	TES5000	3913	'LINC01678	long intergenic non-protein coding RNA 1678
GA	cg08040471	3.16E-09	9.41E-06	BF|FDR0.01	17	80407779	'CYBC1	uncharacterized protein C17orf62 homolog	TSS200;Body;Body;Body;Body;E	-177	'CYBC1	uncharacterized protein C17orf62 homolog
GA	cg08055131	2.56E-08	4.26E-05	BF|FDR0.01	13	28673714	'FLT3	receptor-type tyrosine-protein kinase FLT3 precursor	Body;Body	999	'FLT3	receptor-type tyrosine-protein kinase FLT3 precursor
GA	cg08076462	4.88E-05	0.009909779	FDR0.01	16	87784310	'KLHDC4	kelch domain-containing protein 4	Body;Body;Body;Body;Body;Bo	15282	'KLHDC4	kelch domain-containing protein 4
GA	cg08097581	5.89E-09	1.49E-05	BF|FDR0.01	19	15528589	'AKAP8L;WIZ	A-kinase anchor protein 8-like;protein Wiz isoform 2	Body;Body;Body;TES5000;TES5	1210	'AKAP8L	A-kinase anchor protein 8-like
GA	cg08128577	8.63E-06	0.003107215	FDR0.01	16	27413459	'IL21R	interleukin-21 receptor precursor	TSS200;TSS200	-35	'IL21R	interleukin-21 receptor precursor
GA	cg08133584	1.24E-05	0.004021804	FDR0.01	16	609679	'PRR35	proline-rich protein 35	TSS1500	-714	'PRR35	proline-rich protein 35
GA	cg08145067	1.72E-06	0.000996493	FDR0.01	19	3688176	'PIP5K1C	phosphatidylinositol 4-phosphate 5-kinase type-1 gamma	Body;Body;Body	12290	'PIP5K1C	phosphatidylinositol 4-phosphate 5-kinase type-1 gamma
GA	cg08151809	5.08E-06	0.002132303	FDR0.01	7	151480352	'PRKAG2	5'-AMP-activated protein kinase subunit gamma-2 isoform 1	Body;Body	-23071	'LOC644090	uncharacterized protein LOC644090
GA	cg08155249	4.07E-06	0.001836498	FDR0.01	5	72919936	'ARHGEF28	rho guanine nucleotide exchange factor 28	TSS5000;TSS5000	-2046	'ARHGEF28	rho guanine nucleotide exchange factor 28
GA	cg08155741	9.64E-09	2.09E-05	BF|FDR0.01	11	63994308	'FERMT3;TRPT1;NUDT22;DNAJC4	fermitin family homolog 3 isoform 6;tRNA 2'-phosphotransferase 1 isoform 2	TES5000;TES5000;TSS1500;TSS	563	'NUDT22	uridine diphosphate glucose pyrophosphatase NUDT22
GA	cg08161546	1.10E-05	0.003662185	FDR0.01	6	152011415	'ESR1	estrogen receptor	TSS1500	-215	'ESR1	estrogen receptor
GA	cg08198187	1.88E-05	0.00536597	FDR0.01	12	31882596	'AMN1	protein AMN1 homolog	TSS1500;TSS1500;TSS1500;TSS	-488	'AMN1	protein AMN1 homolog
GA	cg08202399	3.80E-07	0.000330481	FDR0.01	16	14280512	'MRTFB	myocardin-related transcription factor B	Body;Body;Body;TSS200;TSS20	-30	'MRTFB	myocardin-related transcription factor B
GA	cg08234504	2.67E-06	0.001336904	FDR0.01	5	139013317	'			-13566	'CXXC5	CXXC-type zinc finger protein 5
GA	cg08237220	1.07E-06	0.000709212	FDR0.01	3	156332967	'			-59411	'TIPARP	protein mono-ADP-ribosyltransferase TIPARP
GA	cg08248579	2.56E-07	0.000243701	FDR0.01	6	17988937	'KIF13A	kinesin-like protein KIF13A isoform a	TSS1500;TSS1500;TSS1500;TSS	-1071	'KIF13A	kinesin-like protein KIF13A isoform a
GA	cg08257293	3.33E-05	0.007729903	FDR0.01	20	30308440	'BCL2L1;ABALON	bcl-2-like protein 1;apoptotic BCL2L1-antisense long non-coding RNA	Body;Body;Body;Body;Body;Bo	-869	'ABALON	apoptotic BCL2L1-antisense long non-coding RNA
GA	cg08260891	3.18E-06	0.001522567	FDR0.01	20	44518802	'ZSWIM1;SPATA25;NEURL2;CTSA	zinc finger SWIM domain-containing protein 1;spermatogenesis-associated p	TES5000;TSS5000;Body;Body;5	20	'CTSA	lysosomal protective protein precursor
GA	cg08267038	4.01E-08	5.89E-05	BF|FDR0.01	3	113325595	'SIDT1	SID1 transmembrane family member 1 precursor	Body;Body;Body;Body;Body;Bo	11873	'MIR4446	microRNA 4446
GA	cg08278503	6.38E-07	0.000481893	FDR0.01	2	54517167	'ACYP2	acylphosphatase-2	Body;Body;Body;Body;Body	-33801	'TSPYL6	testis-specific Y-encoded-like protein 6
GA	cg08323969	2.62E-05	0.006666096	FDR0.01	17	40770823	'TUBG1	tubulin gamma-1 chain	TES5000	9123	'TUBG1	tubulin gamma-1 chain
GA	cg08326019	7.84E-09	1.84E-05	BF|FDR0.01	19	11472261	'PLPPR2	phospholipid phosphatase-related protein type 2 isoform 2	Body;Body	6154	'PLPPR2	phospholipid phosphatase-related protein type 2 isoform 2
GA	cg08345614	1.76E-05	0.00516726	FDR0.01	20	62586772	'UCKL1;UCKL1-AS1;ZNF512B	uridine-cytidine kinase-like 1;UCKL1 antisense RNA 1;zinc finger protein 512E	TSS5000;TSS5000;TSS5000;Bod	997	'UCKL1	uridine-cytidine kinase-like 1
GA	cg08354216	1.60E-06	0.000948286	FDR0.01	4	146951349	'			-91374	'ZNF827	zinc finger protein 827 isoform 2
GA	cg08373610	3.29E-05	0.007658594	FDR0.01	2	198540621	'RFTN2	raftlin-2	TSS1500	-310	'RFTN2	raftlin-2
GA	cg08379897	3.85E-06	0.001759595	FDR0.01	2	85805621	'VAMP8	vesicle-associated membrane protein 8	Body	913	'VAMP8	vesicle-associated membrane protein 8
GA	cg08386009	3.04E-05	0.007302132	FDR0.01	6	32094845	'ATF6B;FKBPL	cyclic AMP-dependent transcription factor ATF-6 beta;FK506-binding protein	Body;Body;TES5000	1178	'ATF6B	cyclic AMP-dependent transcription factor ATF-6 beta
GA	cg08390172	2.01E-05	0.005602428	FDR0.01	2	192711905	'CAVIN2	caveolae-associated protein 2	TSS200	-91	'CAVIN2	caveolae-associated protein 2
GA	cg08397273	3.43E-06	0.001610324	FDR0.01	19	58740455	'ZNF544	zinc finger protein 544 isoform 10	Body;Body;Body;Body;Body;Bo	176	'ZNF544	zinc finger protein 544 isoform 10
GA	cg08398371	1.93E-06	0.001074503	FDR0.01	19	41814839	'HNRNPUL1;CCDC97	heterogeneous nuclear ribonucleoprotein U-like protein 1;coiled-coil domain	TES5000;TES5000;TES5000;TES	-1237	'CCDC97	coiled-coil domain-containing protein 97
GA	cg08404702	7.25E-10	3.15E-06	BF|FDR0.01	16	2133388	'TSC2	tuberin	Body;Body;Body;Body;Body;Bo	6897	'MIR1225	microRNA 1225
GA	cg08412913	3.95E-07	0.000340863	FDR0.01	16	85429522	'			-89591	'MIR5093	microRNA 5093
GA	cg08416522	3.09E-08	4.94E-05	BF|FDR0.01	21	30451568	'MAP3K7CL	MAP3K7 C-terminal-like protein isoform 1	Body;Body;Body;Body;Body;Bo	2134	'MAP3K7CL	MAP3K7 C-terminal-like protein isoform 1
GA	cg08457421	6.61E-06	0.00259145	FDR0.01	22	31536431	'PLA2G3	group 3 secretory phospholipase A2 precursor	5'UTR	63	'PLA2G3	group 3 secretory phospholipase A2 precursor
GA	cg08471846	3.94E-05	0.008668411	FDR0.01	19	799621	'PTBP1	polypyrimidine tract-binding protein 1	Body;Body;Body	2170	'PTBP1	polypyrimidine tract-binding protein 1
GA	cg08482979	3.76E-05	0.008432891	FDR0.01	16	88858277	'			-6649	'PIEZO1	piezo-type mechanosensitive ion channel component 1
GA	cg08521967	1.22E-05	0.003969164	FDR0.01	1	228345490	'GJC2	gap junction gamma-2 protein	1stExon	7059	'IBA57-DT	IBA57 divergent transcript
GA	cg08528970	2.97E-05	0.007200616	FDR0.01	4	76640579	'			-9126	'USO1	Uso1p
GA	cg08531140	1.96E-06	0.001084302	FDR0.01	11	67143062	'LOC100130987;CLCF1	uncharacterized LOC100130987;cardiotrophin-like cytokine factor 1 isoform	Body;TSS5000;TSS1500	-1414	'CLCF1	cardiotrophin-like cytokine factor 1 isoform 2 precursor
GA	cg08539620	1.61E-08	3.00E-05	BF|FDR0.01	17	40118030	'TTC25;CNP	tetratricopeptide repeat protein 25;2',3'-cyclic-nucleotide 3'-phosphodiester	3'UTR;3'UTR;Body;Body;Body;T	-782	'CNP	2',3'-cyclic-nucleotide 3'-phosphodiesterase
GA	cg08541457	4.53E-05	0.009412742	FDR0.01	3	108606824	'			65192	'TRAT1	T-cell receptor-associated transmembrane adapter 1

Trait	#cpg	p	fdr	significance	chr	pos.hg19	gene.Illumina	gene.description	locations	dist2TSS	gene.nearest	gene.description
GA	cg08544453	4.37E-06	0.00191161	FDR0.01	1	11361900	'UBIAD1	ubiA prenyltransferase domain-containing protein 1	TES5000;TES5000	28646	'UBIAD1	ubiA prenyltransferase domain-containing protein 1
GA	cg08545136	9.57E-06	0.003319253	FDR0.01	17	76775543	'CYTH1	cytohesin-1	Body;Body;Body;Body;Body	2747	'CYTH1	cytohesin-1
GA	cg08548882	7.27E-06	0.002754318	FDR0.01	6	150953085	'PLEKHG1	pleckstrin homology domain-containing family G member 1	Body;Body;Body;Body;Body;Bo	-1014	'PLEKHG1	pleckstrin homology domain-containing family G member 1
GA	cg08549806	3.11E-05	0.007393829	FDR0.01	16	49868530	'ZNF423	zinc finger protein 423	Body	-11881	'ZNF423	zinc finger protein 423
GA	cg08558886	3.44E-05	0.007900136	FDR0.01	2	151469837	'			-15573	'LINC02612	long intergenic non-protein coding RNA 2612
GA	cg08576185	4.02E-11	4.44E-07	BF|FDR0.01	16	2055684	'ZNF598	E3 ubiquitin-protein ligase ZNF598	Body	4138	'ZNF598	E3 ubiquitin-protein ligase ZNF598
GA	cg08591265	6.95E-06	0.002667112	FDR0.01	17	56082867	'SRSF1	serine/arginine-rich splicing factor 1	3'UTR;Body;Body	1740	'SRSF1	serine/arginine-rich splicing factor 1
GA	cg08672999	1.45E-05	0.004492495	FDR0.01	19	52675203	'ZNF836	zinc finger protein 836	TSS1500	-329	'ZNF836	zinc finger protein 836
GA	cg08696931	4.15E-05	0.008928981	FDR0.01	12	123754071	'CDK2AP1	cyclin-dependent kinase 2-associated protein 1	TSS1500;Body;Body;Body;Body	759	'CDK2AP1	cyclin-dependent kinase 2-associated protein 1
GA	cg08700083	2.39E-06	0.001251958	FDR0.01	4	48382815	'SLAIN2	SLAIN motif-containing protein 2	Body	39270	'SLAIN2	SLAIN motif-containing protein 2
GA	cg08708961	1.77E-06	0.001017349	FDR0.01	1	227070630	'PSEN2	presenilin-2	Body;Body	12314	'PSEN2	presenilin-2
GA	cg08716396	1.13E-05	0.003756248	FDR0.01	1	161283845	'MPZ;SDHC	myelin protein P0 isoform MPZ precursor;succinate dehydrogenase cytochro	TSS5000;TSS5000;TSS1500;TSS1	-320	'SDHC	succinate dehydrogenase cytochrome b560 subunit, mitochondrial
GA	cg08716889	2.52E-06	0.001293814	FDR0.01	10	71905226	'TYSND1;SAR1A	peroxisomal leader peptide-processing protease isoform b;GTP-binding prot	1stExon;1stExon;TSS1500;Body	-742	'TYSND1	peroxisomal leader peptide-processing protease isoform b
GA	cg08716982	1.62E-05	0.004858132	FDR0.01	5	179640174	'RASGEF1C	ras-GEF domain-containing family member 1C	TSS5000	-3963	'RASGEF1C	ras-GEF domain-containing family member 1C
GA	cg08722122	1.02E-08	2.18E-05	BF|FDR0.01	8	38326574	'FGFR1	fibroblast growth factor receptor 1 precursor	TSS1500;TSS1500;TSS1500;TSS1	-222	'FGFR1	fibroblast growth factor receptor 1 precursor
GA	cg08726900	8.83E-06	0.003148831	FDR0.01	16	89550474	'ANKRD11	ankyrin repeat domain-containing protein 11	Body;Body;Body;Body	6495	'ANKRD11	ankyrin repeat domain-containing protein 11
GA	cg08729004	1.10E-05	0.00367788	FDR0.01	1	169406242	'CCDC181	coiled-coil domain-containing protein 181	Body	-9530	'CCDC181	coiled-coil domain-containing protein 181
GA	cg08731696	2.88E-05	0.007086753	FDR0.01	1	59280489	'LINC01135	long intergenic non-protein coding RNA 1135	Body;Body;Body	29667	'LINC01135	long intergenic non-protein coding RNA 1135
GA	cg08735211	1.84E-05	0.005313661	FDR0.01	6	32920657	'HLA-DMA	HLA class II histocompatibility antigen, DM alpha chain precursor	Body	217	'HLA-DMA	HLA class II histocompatibility antigen, DM alpha chain precursor
GA	cg08751508	1.61E-09	5.93E-06	BF|FDR0.01	10	134221855	'PWWP2B	PWWP domain-containing protein 2B	Body;Body	11152	'PWWP2B	PWWP domain-containing protein 2B
GA	cg08805037	6.48E-08	8.59E-05	BF|FDR0.01	22	20009275	'ARVCF;TANGO2	armadillo repeat protein deleted in velo-cardio-facial syndrome;transport an	TSS5000;Body;Body;Body;Body	643	'TANGO2	transport and Golgi organization protein 2 homolog
GA	cg08810397	4.94E-07	0.000396481	FDR0.01	16	1521676	'CLCN7	H(+)/Cl(-) exchange transporter 7	Body;Body	3353	'CLCN7	H(+)/Cl(-) exchange transporter 7
GA	cg08810582	1.59E-05	0.004792161	FDR0.01	12	69139650	'NUP107;SLC35E3	nuclear pore complex protein Nup107;solute carrier family 35 member E3	TES5000;TES5000;TSS1500;TSS	-305	'SLC35E3	solute carrier family 35 member E3
GA	cg08813888	7.14E-09	1.74E-05	BF|FDR0.01	2	662396	'TMEM18	transmembrane protein 18 isoform 2	TES5000;TES5000;TES5000	14308	'TMEM18	transmembrane protein 18 isoform 2
GA	cg08817867	4.40E-10	2.26E-06	BF|FDR0.01	17	19656554	'ALDH3A1	aldehyde dehydrogenase, dimeric NADP-preferring isoform 2	TSS5000;TSS5000	-4943	'ALDH3A1	aldehyde dehydrogenase, dimeric NADP-preferring isoform 2
GA	cg08824344	4.42E-05	0.00927102	FDR0.01	3	126108727	'CCDC37-DT	CCDC37 divergent transcript	TES5000;TES5000;TES5000	4914	'CCDC37-DT	CCDC37 divergent transcript
GA	cg08827060	3.62E-08	5.54E-05	BF|FDR0.01	8	102121621	'			57340	'FLJ42969	uncharacterized LOC441374
GA	cg08838842	6.87E-06	0.002651898	FDR0.01	3	111577634	'PHLDB2	pleckstrin homology-like domain family B member 2	Body;TSS1500;TSS1500;TSS150	-392	'PHLDB2	pleckstrin homology-like domain family B member 2
GA	cg08857201	3.37E-06	0.00158873	FDR0.01	15	56536865	'RFX7	DNA-binding protein RFX7	TSS1500;TSS1500;Body	415	'RFX7	DNA-binding protein RFX7
GA	cg08858051	1.67E-05	0.00494544	FDR0.01	1	94313454	'BCAR3;MIR760	breast cancer anti-estrogen resistance protein 3 isoform 3;microRNA 760	TSS1500;TES5000	-679	'BCAR3	breast cancer anti-estrogen resistance protein 3 isoform 3
GA	cg08869700	3.08E-05	0.007359402	FDR0.01	1	151318680	'RFX5;GBAT2	DNA-binding protein RFX5;	1stExon;1stExon;TSS1500	-805	'GBAT2	
GA	cg08873568	3.08E-05	0.007359402	FDR0.01	19	4894976	'ARRDC5	arrestin domain-containing protein 5	Body;Body	7932	'ARRDC5	arrestin domain-containing protein 5
GA	cg08888956	5.65E-07	0.000439683	FDR0.01	12	86267839	'NTS	neurotensin precursor	TSS1500	-233	'NTS	neurotensin precursor
GA	cg08903441	1.82E-08	3.21E-05	BF|FDR0.01	17	80304981	'			-12141	'TEX19	testis-expressed protein 19
GA	cg08909806	2.23E-06	0.001205744	FDR0.01	22	43548696	'TSPO	translocator protein	Body;Body;Body;Body	756	'TSPO	translocator protein
GA	cg08915038	4.67E-06	0.002010069	FDR0.01	1	16562923	'CPLANE2	ciliogenesis and planar polarity effector 2	Body	755	'CPLANE2	ciliogenesis and planar polarity effector 2
GA	cg08942051	1.21E-07	0.000139372	FDR0.01	3	195914092	'			24179	'ZDHHC19	palmitoyltransferase ZDHHC19
GA	cg08962185	3.88E-06	0.001773854	FDR0.01	8	143336052	'TSNARE1	t-SNARE domain-containing protein 1	Body;Body;Body	56336	'LINC00051	long intergenic non-protein coding RNA 51
GA	cg08970648	1.52E-07	0.000165018	FDR0.01	3	50644192	'CISH	cytokine-inducible SH2-containing protein	3'UTR;3'UTR	5013	'CISH	cytokine-inducible SH2-containing protein
GA	cg09023584	2.67E-05	0.006731541	FDR0.01	4	2273606	'ZFYVE28	lateral signaling target protein 2 homolog isoform 1	Body;Body;Body	-9770	'MXD4	max dimerization protein 4
GA	cg09063663	4.46E-05	0.009320659	FDR0.01	1	16484811	'EPHA2	ephrin type-A receptor 2 precursor	TSS5000;TSS5000	-2247	'EPHA2	ephrin type-A receptor 2 precursor
GA	cg09072216	2.38E-06	0.001249385	FDR0.01	13	36919344	'SPART;SPART-AS1	spartin;SPART antisense RNA 1	Body;Body;Body;Body;TSS1500	1187	'SPART	spartin
GA	cg09096031	5.69E-10	2.80E-06	BF|FDR0.01	2	198540569	'RFTN2	raftlin-2	TSS1500	-258	'RFTN2	raftlin-2
GA	cg09109520	1.47E-13	9.40E-09	BF|FDR0.01	16	57673258	'ADGRG1	adhesion G-protein coupled receptor G1 precursor	Body;Body;Body;Body;Body;Bo	-145	'ADGRG1	adhesion G-protein coupled receptor G1 precursor
GA	cg09121543	1.40E-05	0.004373959	FDR0.01	17	61774794	'MAP3K3;LIMD2;LOC729683	mitogen-activated protein kinase kinase kinase 3;LIM domain-containing pro	TES5000;TES5000;TES5000;TES	2691	'LIMD2	LIM domain-containing protein 2
GA	cg09124496	3.78E-07	0.000329993	FDR0.01	7	41735851	'INHBA;INHBA-AS1	inhibin beta A chain precursor;INHBA antisense RNA 1	Body;Body;Body	2335	'INHBA-AS1	INHBA antisense RNA 1
GA	cg09125320	2.71E-05	0.006793468	FDR0.01	1	173685201	'KLHL20	kelch-like protein 20	1stExon	1082	'KLHL20	kelch-like protein 20
GA	cg09143713	1.27E-05	0.004079606	FDR0.01	20	9141615	'PLCB4	1-phosphatidylinositol 4,5-bisphosphate phosphodiesterase beta-4	Body	-56421	'PLCB4	1-phosphatidylinositol 4,5-bisphosphate phosphodiesterase beta-4
GA	cg09163035	4.23E-06	0.001874619	FDR0.01	17	37054726	'LASP1	LIM and SH3 domain protein 1	Body;Body;Body	-16505	'MIR6779	microRNA 6779
GA	cg09168805	1.90E-05	0.005409077	FDR0.01	1	94514548	'ABCA4	retinal-specific phospholipid-transporting ATPase ABCA4	Body	72156	'ABCA4	retinal-specific phospholipid-transporting ATPase ABCA4
GA	cg09175485	2.55E-06	0.001300671	FDR0.01	11	67020555	'KDM2A	lysine-specific demethylase 2A	Body;Body;Body	13071	'KDM2A	lysine-specific demethylase 2A
GA	cg09187007	7.23E-06	0.002751232	FDR0.01	1	3514990	'MEGF6	multiple epidermal growth factor-like domains protein 6 precursor	Body	13082	'MEGF6	multiple epidermal growth factor-like domains protein 6 precursor
GA	cg09188980	3.57E-06	0.001649222	FDR0.01	11	14993378	'CALCA	calcitonin isoform 1 preproprotein	Body;Body;Body;Body	454	'CALCA	calcitonin isoform 1 preproprotein
GA	cg09204187	1.96E-08	3.34E-05	BF|FDR0.01	2	130940668	'SMPD4;MZT2B	sphingomyelin phosphodiesterase 4;mitotic-spindle organizing protein 2B	TSS1500;TSS1500;TSS1500;TSS	834	'MZT2B	mitotic-spindle organizing protein 2B
GA	cg09233395	6.86E-07	0.000510579	FDR0.01	8	116678582	'TRPS1	zinc finger transcription factor Trps1	Body;Body;Body;Body	1658	'TRPS1	zinc finger transcription factor Trps1
GA	cg09238801	5.93E-09	1.49E-05	BF|FDR0.01	14	74179101	'PNMA1;ELMSAN1	paraneoplastic antigen Ma1;	3'UTR;TES5000;TES5000;TES50	1986	'PNMA1	paraneoplastic antigen Ma1
GA	cg09248380	1.63E-05	0.004862893	FDR0.01	4	111397190	'ENPEP	glutamyl aminopeptidase	TSS200	-120	'ENPEP	glutamyl aminopeptidase
GA	cg09253696	3.82E-05	0.008522108	FDR0.01	17	73873529	'TRIM47	E3 ubiquitin-protein ligase TRIM47	Body	1133	'TRIM47	E3 ubiquitin-protein ligase TRIM47
GA	cg09274826	1.08E-07	0.000129809	BF|FDR0.01	1	156718357	'HDGF	hepatoma-derived growth factor isoform d	Body;Body;Body;Body;Body;Bo	2728	'HDGF	hepatoma-derived growth factor isoform d
GA	cg09276655	1.43E-05	0.004447175	FDR0.01	16	87735689	'JPH3	junctophilin-3	TES5000;TES5000	63903	'KLHDC4	kelch domain-containing protein 4
GA	cg09276998	1.08E-08	2.29E-05	BF|FDR0.01	10	29991884	'SVIL	supervillin	Body;Body;Body	32846	'SVIL	supervillin
GA	cg09294072	6.94E-08	9.06E-05	BF|FDR0.01	14	106366523	'			-17314	'FAM30A	family with sequence similarity 30 member A
GA	cg09370806	2.65E-05	0.006704434	FDR0.01	12	105994959	'			-103021	'CASC18	cancer susceptibility 18
GA	cg09384937	5.52E-08	7.57E-05	BF|FDR0.01	12	49359996	'WNT10B	protein Wnt-10b precursor	Body	5522	'WNT10B	protein Wnt-10b precursor
GA	cg09406615	1.16E-07	0.000135795	FDR0.01	3	165555103	'BCHE	cholinesterase precursor	5'UTR;Body;Body	108	'BCHE	cholinesterase precursor
GA	cg09411730	3.26E-07	0.000295783	FDR0.01	11	65190946	'NEAT1	nuclear paraspeckle assembly transcript 1	Body;Body	678	'NEAT1	nuclear paraspeckle assembly transcript 1
GA	cg09414827	1.69E-06	0.000984798	FDR0.01	7	134832671	'CYREN;TMEM140	cell cycle regulator of non-homologous end joining;transmembrane protein	Body;TSS200	-152	'TMEM140	transmembrane protein 140 precursor
GA	cg09419900	2.81E-05	0.006946891	FDR0.01	10	124739466	'PSTK	L-seryl-tRNA(Sec) kinase	TSS1500;TSS1500	-450	'PSTK	L-seryl-tRNA(Sec) kinase
GA	cg09423126	1.31E-06	0.000820198	FDR0.01	8	22735333	'PEBP4;LOC101929237	phosphatidylethanolamine-binding protein 4 precursor;uncharacterized LOC	Body;Body;TSS200	-151	'LOC101929237	uncharacterized LOC101929237
GA	cg09430518	1.16E-08	2.38E-05	BF|FDR0.01	8	118662568	'			129617	'MED30	mediator of RNA polymerase II transcription subunit 30
GA	cg09438075	3.76E-05	0.008432891	FDR0.01	13	44359415	'ENOX1	ecto-NOX disulfide-thiol exchanger 1 isoform b	Body;Body;Body;Body;Body;Bo	1693	'ENOX1	ecto-NOX disulfide-thiol exchanger 1 isoform b
GA	cg09438113	1.23E-07	0.000140828	FDR0.01	8	82545688	'IMPA1P1	inositol monophosphatase 1 pseudogene 1	TSS5000	-2176	'IMPA1P1	inositol monophosphatase 1 pseudogene 1
GA	cg09461851	1.13E-05	0.003748447	FDR0.01	16	921161	'LMF1	lipase maturation factor 1	Body;Body;Body;Body;Body;Bo	-49871	'LMF1-AS1	LMF1 antisense RNA 1
GA	cg09464137	4.53E-06	0.001964364	FDR0.01	2	10444574	'HPCAL1	hippocalcin-like protein 1	Body;Body	749	'HPCAL1	hippocalcin-like protein 1
GA	cg09464990	2.62E-06	0.001319172	FDR0.01	17	70724035	'SLC39A11	zinc transporter ZIP11 isoform 2	Body;Body;Body;Body;Body	-87424	'LINC00511	long intergenic non-protein coding RNA 511
GA	cg09487449	1.34E-05	0.004243425	FDR0.01	19	47983663	'KPTN;NAPA-AS1	KICSTOR complex protein kaptin;NAPA antisense RNA 1	Body;Body;Body;TSS5000	3813	'KPTN	KICSTOR complex protein kaptin
GA	cg09507934	1.41E-05	0.004388462	FDR0.01	7	102072549	'ORAI2	protein orai-2	TSS1500;TSS1500;TSS1500;TSS	-1427	'ORAI2	protein orai-2
GA	cg09512548	1.80E-05	0.005246511	FDR0.01	6	111983184	'FYN	tyrosine-protein kinase Fyn	Body;Body;Body;Body	-55707	'TRAF3IP2	adapter protein CIKS
GA	cg09547314	3.71E-08	5.65E-05	BF|FDR0.01	19	5251120	'PTPRS	receptor-type tyrosine-protein phosphatase S isoform 3 precursor	Body;Body;Body;Body	89703	'PTPRS	receptor-type tyrosine-protein phosphatase S isoform 3 precursor
GA	cg09547692	1.99E-05	0.005576344	FDR0.01	1	91027278	'			-96028	'SNORD3G	small nucleolar RNA, C/D box 3G
GA	cg09550083	4.92E-05	0.009952864	FDR0.01	2	66672337	'MEIS1;MEIS1-AS2	homeobox protein Meis1;MEIS1 antisense RNA 2	Body;TSS5000	-4875	'MEIS1-AS2	MEIS1 antisense RNA 2
GA	cg09564253	3.81E-07	0.0003312	FDR0.01	17	37028105	'LASP1	LIM and SH3 domain protein 1	Body;Body;Body	1795	'LASP1	LIM and SH3 domain protein 1
GA	cg09575312	1.03E-06	0.000689059	FDR0.01	19	8511155	'HNRNPM	heterogeneous nuclear ribonucleoprotein M	Body;Body;Body	1297	'HNRNPM	heterogeneous nuclear ribonucleoprotein M
GA	cg09580393	2.03E-07	0.000204035	FDR0.01	17	4869866	'SPAG7;CAMTA2;MIR6864;MIR6865	sperm-associated antigen 7;calmodulin-binding transcription activator 2;mic	Body;TES5000;TES5000;TES5000	1246	'SPAG7	sperm-associated antigen 7

Trait	#cpg	p	fdr	significance	chr	pos.hg19	gene.Illumina	gene.description	locations	dist2TSS	gene.nearest	gene.description
GA	cg09580592	4.60E-05	0.009517024	FDR0.01	17	907643	'TIMM22;ABR	mitochondrial import inner membrane translocase subunit Tim22;active brea	TES5000;3'UTR;3'UTR;3'UTR;3'U	7275	'TIMM22	mitochondrial import inner membrane translocase subunit Tim22
GA	cg09584358	2.05E-08	3.49E-05	BF|FDR0.01	17	14201680	'HS3ST3B1	heparan sulfate glucosamine 3-O-sulfotransferase 3B1	TSS5000;TSS5000	-2717	'HS3ST3B1	heparan sulfate glucosamine 3-O-sulfotransferase 3B1
GA	cg09591867	8.64E-06	0.003107215	FDR0.01	17	79098558	'AATK;MIR657;MIR3065;MIR338	serine/threonine-protein kinase LMTK1 isoform 2;microRNA 657;microRNA 3	Body;Body;TES5000;TSS1500;TE	615	'MIR657	microRNA 657
GA	cg09608765	6.72E-06	0.002611536	FDR0.01	3	45636137	'LIMD1	LIM domain-containing protein 1	TSS200	-105	'LIMD1	LIM domain-containing protein 1
GA	cg09614389	9.14E-08	0.00011388	BF|FDR0.01	20	25450839	'NINL	ninein-like protein isoform 1	Body;Body	62521	'GINS1	DNA replication complex GINS protein PSF1
GA	cg09674468	2.71E-05	0.006792664	FDR0.01	1	234300299	'SLC35F3	putative thiamine transporter SLC35F3	Body	-49657	'SLC35F3	putative thiamine transporter SLC35F3
GA	cg09696044	1.37E-08	2.69E-05	BF|FDR0.01	20	55968294	'LOC100291105;RBM38	;RNA-binding protein 38	TSS200;Body;Body;Body	-182	'LOC100291105	
GA	cg09716613	3.44E-08	5.34E-05	BF|FDR0.01	13	33000534	'N4BP2L1	NEDD4-binding protein 2-like 1 isoform 2	Body;Body;Body;Body;Body;Bo	1733	'N4BP2L1	NEDD4-binding protein 2-like 1 isoform 2
GA	cg09745987	4.92E-06	0.002078476	FDR0.01	16	17318866	'XYLT1	xylosyltransferase 1 precursor	Body	90506	'LOC102723692	uncharacterized LOC102723692
GA	cg09796457	3.06E-06	0.001481795	FDR0.01	15	68571585	'FEM1B	protein fem-1 homolog B	Body	1503	'FEM1B	protein fem-1 homolog B
GA	cg09803560	4.45E-07	0.000369722	FDR0.01	7	158664058	'WDR60		1stExon;5'UTR;5'UTR;Body;5'UT	14810	'WDR60	
GA	cg09807452	3.57E-05	0.008134104	FDR0.01	4	140099025	'ELF2	ETS-related transcription factor Elf-2 isoform 1	TSS1500;TSS1500;TSS1500;TSS1	-653	'ELF2	ETS-related transcription factor Elf-2 isoform 1
GA	cg09808864	1.93E-05	0.005462927	FDR0.01	6	138482336	'ARFGEF3	brefeldin A-inhibited guanine nucleotide-exchange protein 3	TSS1500	-739	'ARFGEF3	brefeldin A-inhibited guanine nucleotide-exchange protein 3
GA	cg09824706	4.27E-05	0.009067475	FDR0.01	7	6487775	'DAGLB	diacylglycerol lipase-beta	TSS200;TSS200	-132	'DAGLB	diacylglycerol lipase-beta
GA	cg09829983	1.18E-06	0.000759985	FDR0.01	2	7017698	'RSAD2	radical S-adenosyl methionine domain-containing protein 2	TSS1500	-209	'RSAD2	radical S-adenosyl methionine domain-containing protein 2
GA	cg09854734	3.54E-07	0.000313298	FDR0.01	16	66613273	'CKLF-CMTM1;CMTM1;CMTM2	CKLF-CMTM1 protein isoform 3;CKLF-like MARVEL transmembrane domain-c	TES5000;TES5000;TES5000;TES	-92	'CMTM2	CKLF-like MARVEL transmembrane domain-containing protein 2
GA	cg09874605	2.87E-05	0.00706313	FDR0.01	15	68123157	'SKOR1;LOC101929076	SKI family transcriptional corepressor 1;uncharacterized LOC101929076	Body;TES5000	5311	'SKOR1	SKI family transcriptional corepressor 1
GA	cg09879876	4.69E-05	0.009625669	FDR0.01	11	74420989	'CHRDL2	chordin-like protein 2 precursor	Body;Body;Body;Body;Body;Bo	1423	'CHRDL2	chordin-like protein 2 precursor
GA	cg09882118	7.31E-13	2.18E-08	BF|FDR0.01	3	48508272	'ATRIP;ATRIP-TREX1;TREX1;SHISA5	ATR-interacting protein;ATRIP-TREX1 readthrough;three-prime repair exonu	TES5000;3'UTR;3'UTR;Body;3'U	644	'TREX1	three-prime repair exonuclease 1 isoform c
GA	cg09889350	2.50E-07	0.000238464	FDR0.01	16	56995813	'CETP	cholesteryl ester transfer protein precursor	TSS200;TSS200	-48	'CETP	cholesteryl ester transfer protein precursor
GA	cg09899220	2.96E-05	0.007183669	FDR0.01	16	67232281	'E2F4;ELMO3;MIR328	transcription factor E2F4;engulfment and cell motility protein 3;microRNA 32	3'UTR;TSS1500;TES5000	-752	'ELMO3	engulfment and cell motility protein 3
GA	cg09915396	1.12E-09	4.44E-06	BF|FDR0.01	17	2907895	'RAP1GAP2	rap1 GTPase-activating protein 2 isoform 2	Body;Body;Body	-38706	'LOC101927911	uncharacterized LOC101927911
GA	cg09917805	4.59E-05	0.009506349	FDR0.01	4	4763596	'LOC101928279	uncharacterized LOC101928279	Body	110	'LOC101928279	uncharacterized LOC101928279
GA	cg09939079	9.44E-07	0.00064433	FDR0.01	12	69753248	'YEATS4	YEATS domain-containing protein 4	TSS1500;TSS1500	-241	'YEATS4	YEATS domain-containing protein 4
GA	cg09974063	1.41E-06	0.000863461	FDR0.01	11	105806773	'GRIA4	glutamate receptor 4 isoform 2 precursor	Body;Body;Body	86194	'MSANTD4	myb/SANT-like DNA-binding domain-containing protein 4
GA	cg09981830	4.90E-06	0.00207587	FDR0.01	7	55376098	'			-52770	'ELDR	EGFR long non-coding downstream RNA
GA	cg10017843	2.38E-06	0.001249385	FDR0.01	16	67693452	'CARMIL2;ACD;PARD6A;ENKD1	capping protein, Arp2/3 and myosin-I linker protein 2 isoform 2;adrenocortic	TES5000;TES5000;Body;Body;TS	711	'ACD	adrenocortical dysplasia protein homolog isoform 1
GA	cg10020892	3.89E-10	2.12E-06	BF|FDR0.01	1	147016901	'BCL9	B-cell CLL/lymphoma 9 protein	Body	3631	'BCL9	B-cell CLL/lymphoma 9 protein
GA	cg10037005	2.30E-07	0.000223746	FDR0.01	19	49838478	'CD37	leukocyte antigen CD37 isoform B	TSS200;TSS200	-184	'CD37	leukocyte antigen CD37 isoform B
GA	cg10059056	3.31E-05	0.007687503	FDR0.01	11	46355283	'DGKZ	diacylglycerol kinase zeta	Body	808	'DGKZ	diacylglycerol kinase zeta
GA	cg10072464	2.09E-05	0.005779049	FDR0.01	13	114064536	'LOC101928841	uncharacterized protein LOC101928841	Body	4172	'LOC101928841	uncharacterized protein LOC101928841
GA	cg10078415	5.92E-06	0.002390532	FDR0.01	16	20775011	'ACSM3	acyl-coenzyme A synthetase ACSM3, mitochondrial isoform 2	TSS1500;TSS1500	-300	'ACSM3	acyl-coenzyme A synthetase ACSM3, mitochondrial isoform 2
GA	cg10088320	5.14E-06	0.002151363	FDR0.01	6	32805759	'TAP2;PSMB8	antigen peptide transporter 2;proteasome subunit beta type-8	1stExon;1stExon;TES5000;TES5	788	'TAP2	antigen peptide transporter 2
GA	cg10097651	8.27E-06	0.003023415	FDR0.01	14	35802397	'			40810	'PSMA6	proteasome subunit alpha type-6
GA	cg10112854	3.53E-08	5.45E-05	BF|FDR0.01	16	80839183	'CDYL2	chromodomain Y-like protein 2	TSS1500	-688	'CDYL2	chromodomain Y-like protein 2
GA	cg10118545	3.79E-05	0.008488332	FDR0.01	7	4784090	'FOXK1	forkhead box protein K1	Body	-31163	'AP5Z1	AP-5 complex subunit zeta-1
GA	cg10130088	1.09E-07	0.000129809	BF|FDR0.01	11	122138618	'MIR100HG	mir-100-let-7a-2-mir-125b-1 cluster host gene	Body;Body;Body;Body	41577	'MIR100HG	mir-100-let-7a-2-mir-125b-1 cluster host gene
GA	cg10145196	1.85E-06	0.001046205	FDR0.01	6	30647649	'PPP1R18	phostensin	Body;Body	6773	'PPP1R18	phostensin
GA	cg10152449	1.04E-06	0.00069549	FDR0.01	7	2444534	'CHST12	carbohydrate sulfotransferase 12	Body;Body;Body	1289	'CHST12	carbohydrate sulfotransferase 12
GA	cg10156217	3.67E-05	0.008291959	FDR0.01	14	50362354	'ARF6	ADP-ribosylation factor 6	3'UTR	2555	'ARF6	ADP-ribosylation factor 6
GA	cg10162659	7.34E-06	0.002771133	FDR0.01	16	73068480	'ZFHX3	zinc finger homeobox protein 3 isoform B	Body;Body	13547	'ZFHX3	zinc finger homeobox protein 3 isoform B
GA	cg10165952	2.50E-07	0.000238464	FDR0.01	17	58239449	'CA4	carbonic anhydrase 4 precursor	TES5000;Body	12116	'CA4	carbonic anhydrase 4 precursor
GA	cg10179949	9.67E-06	0.00334613	FDR0.01	1	159795589	'SLAMF8	uncharacterized protein LOC768588 precursor	TSS1500;TSS1500	-1073	'SLAMF8	uncharacterized protein LOC768588 precursor
GA	cg10184289	3.19E-08	5.05E-05	BF|FDR0.01	2	64870490	'SERTAD2	SERTA domain-containing protein 2	Body	10557	'SERTAD2	SERTA domain-containing protein 2
GA	cg10185424	1.61E-08	3.00E-05	BF|FDR0.01	5	66478491	'CD180	CD180 antigen precursor	3'UTR	14136	'CD180	CD180 antigen precursor
GA	cg10187674	3.57E-05	0.008134104	FDR0.01	17	67323922	'ABCA5	ATP-binding cassette sub-family A member 5	TSS1500	-648	'ABCA5	ATP-binding cassette sub-family A member 5
GA	cg10189135	1.74E-06	0.001005554	FDR0.01	16	67516162	'ATP6V0D1;AGRP	V-type proton ATPase subunit d 1;agouti-related protein precursor	TSS1500;TES5000	-1102	'ATP6V0D1	V-type proton ATPase subunit d 1
GA	cg10191240	3.91E-05	0.008636129	FDR0.01	8	134230101	'CCN4	CCN family member 4 isoform 4 precursor	Body;Body;Body;Body;Body	26820	'CCN4	CCN family member 4 isoform 4 precursor
GA	cg10192797	6.68E-06	0.002608476	FDR0.01	16	10678655	'EMP2	epithelial membrane protein 2 precursor	TSS5000	-4200	'EMP2	epithelial membrane protein 2 precursor
GA	cg10201400	3.60E-05	0.008185792	FDR0.01	13	44359601	'ENOX1	ecto-NOX disulfide-thiol exchanger 1 isoform b	Body;Body;Body;Body;Body;Bo	1507	'ENOX1	ecto-NOX disulfide-thiol exchanger 1 isoform b
GA	cg10206969	2.76E-05	0.006861496	FDR0.01	7	5518392	'FBXL18	F-box/LRR-repeat protein 18	Body;3'UTR;3'UTR;3'UTR	17156	'MIR589	microRNA 589
GA	cg10212621	2.21E-05	0.005957728	FDR0.01	1	120311649	'HMGCS2	hydroxymethylglutaryl-CoA synthase, mitochondrial	TSS200;TSS200	-94	'HMGCS2	hydroxymethylglutaryl-CoA synthase, mitochondrial
GA	cg10250177	2.14E-05	0.005860161	FDR0.01	1	32739752	'LCK	tyrosine-protein kinase Lck	Body;Body;5'UTR	41	'LCK	tyrosine-protein kinase Lck
GA	cg10255141	3.63E-05	0.008242927	FDR0.01	12	109531108	'ALKBH2;UNG	alpha-ketoglutarate-dependent dioxygenase alkB homolog 2 isoform 1;uracil	5'UTR;5'UTR;Body;Body;5'UTR;T	169	'ALKBH2	alpha-ketoglutarate-dependent dioxygenase alkB homolog 2 isoform 1
GA	cg10278520	2.35E-05	0.006194686	FDR0.01	8	59771002	'TOX	thymocyte selection-associated high mobility group box protein TOX	Body	-198590	'NSMAF	protein FAN precursor
GA	cg10288995	3.65E-05	0.008277727	FDR0.01	3	43332555	'SNRK	SNF-related serine/threonine-protein kinase	Body;Body;Body	4524	'SNRK	SNF-related serine/threonine-protein kinase
GA	cg10298741	1.79E-05	0.005227922	FDR0.01	16	73019173	'ZFHX3	zinc finger homeobox protein 3 isoform B	Body;Body	62854	'ZFHX3	zinc finger homeobox protein 3 isoform B
GA	cg10307662	3.28E-07	0.000296543	FDR0.01	4	4679628	'STX18-AS1	STX18 antisense RNA 1 (head to head)	Body	13576	'SNORD162	small nucleolar RNA, C/D box 162
GA	cg10322794	2.33E-07	0.00022678	FDR0.01	21	47740892	'C21orf58;PCNT	uncharacterized protein C21orf58 isoform 1;pericentrin isoform 2	TSS200;TSS5000;Body;Body;Bo	-153	'C21orf58	uncharacterized protein C21orf58 isoform 1
GA	cg10333333	3.87E-05	0.00858547	FDR0.01	13	42535460	'VWA8;VWA8-AS1	von Willebrand factor A domain-containing protein 8 isoform a precursor;VW	TSS1500;TSS1500;Body	156	'VWA8-AS1	VWA8 antisense RNA 1 (head to head)
GA	cg10343364	1.00E-06	0.000674986	FDR0.01	16	3559568	'CLUAP1	clusterin-associated protein 1	Body;Body;TSS1500	-325	'CLUAP1	clusterin-associated protein 1
GA	cg10351829	2.52E-08	4.20E-05	BF|FDR0.01	10	134661550	'CFAP46	cilia- and flagella-associated protein 46	Body	-61899	'NKX6-2	homeobox protein Nkx-6.2
GA	cg10380221	3.48E-07	0.000310229	FDR0.01	16	31075618	'ZNF668	zinc finger protein 668	Body;1stExon;1stExon;1stExon	791	'ZNF668	zinc finger protein 668
GA	cg10399789	3.69E-05	0.008332894	FDR0.01	1	92945668	'GFI1	zinc finger protein Gfi-1	Body;Body;Body	3843	'GFI1	zinc finger protein Gfi-1
GA	cg10406690	3.12E-05	0.007400248	FDR0.01	14	85995726	'FLRT2	leucine-rich repeat transmembrane protein FLRT2 precursor	TSS1500;TSS1500;TSS1500;TSS1	-642	'FLRT2	leucine-rich repeat transmembrane protein FLRT2 precursor
GA	cg10418289	8.77E-06	0.003135806	FDR0.01	14	23308294	'MRPL52;MMP14	39S ribosomal protein L52, mitochondrial;matrix metalloproteinase 14 precu	TES5000;TES5000;TES5000;TES	2501	'MMP14	matrix metalloproteinase 14 precursor
GA	cg10422709	1.36E-05	0.004282253	FDR0.01	15	53083978	'ONECUT1	hepatocyte nuclear factor 6	TSS1500	-1445	'ONECUT1	hepatocyte nuclear factor 6
GA	cg10432620	4.95E-05	0.009979528	FDR0.01	16	74808963	'FA2H	fatty acid 2-hydroxylase	TSS1500	-245	'FA2H	fatty acid 2-hydroxylase
GA	cg10436833	2.03E-05	0.005640629	FDR0.01	1	43354025	'LOC339539	uncharacterized LOC339539	Body	30733	'LOC339539	uncharacterized LOC339539
GA	cg10447977	4.83E-07	0.000391885	FDR0.01	3	64598405	'ADAMTS9	A disintegrin and metalloproteinase with thrombospondin motifs 9 precurso	Body;Body	51384	'ADAMTS9-AS1	ADAMTS9 antisense RNA 1
GA	cg10464312	3.96E-05	0.008676382	FDR0.01	2	66672688	'MEIS1	homeobox protein Meis1	Body	-5226	'MEIS1-AS2	MEIS1 antisense RNA 2
GA	cg10467022	1.04E-06	0.000690961	FDR0.01	11	14994983	'CALCA	calcitonin isoform 1 preproprotein	TSS1500;TSS1500;TSS1500;TSS1	-1086	'CALCA	calcitonin isoform 1 preproprotein
GA	cg10467098	3.08E-06	0.001485505	FDR0.01	11	65687892	'C11orf68;DRAP1	UPF0696 protein C11orf68 isoform 2;dr1-associated corepressor	TSS1500;TSS1500;Body	996	'DRAP1	dr1-associated corepressor
GA	cg10499832	3.04E-06	0.001472486	FDR0.01	17	41450195	'LINC00910	long intergenic non-protein coding RNA 910	Body;TES5000	16071	'LINC00910	long intergenic non-protein coding RNA 910
GA	cg10517650	3.95E-06	0.001799975	FDR0.01	3	113235015	'SPICE1	spindle and centriole-associated protein 1 isoform b	TSS1500;TSS1500;TSS1500	-1012	'SPICE1	spindle and centriole-associated protein 1 isoform b
GA	cg10535320	2.33E-05	0.006151357	FDR0.01	17	61908869	'FTSJ3;PSMC5;SMARCD2	pre-rRNA 2'-O-ribose RNA methyltransferase FTSJ3;26S proteasome regulato	TSS5000;Body;Body;TES5000;TE	3826	'PSMC5	26S proteasome regulatory subunit 8
GA	cg10536303	4.56E-05	0.009476242	FDR0.01	14	24583056	'NRL;DCAF11	neural retina-specific leucine zipper protein isoform 2;DDB1- and CUL4-assoc	Body;Body;Body;TSS1500;TSS15	-849	'DCAF11	DDB1- and CUL4-associated factor 11
GA	cg10537821	4.30E-05	0.009104529	FDR0.01	15	70105522	'			-22050	'LINC00593	long intergenic non-protein coding RNA 593
GA	cg10540679	2.10E-05	0.005782308	FDR0.01	7	130606499	'LINC00513	long intergenic non-protein coding RNA 513	Body	8277	'LINC00513	long intergenic non-protein coding RNA 513
GA	cg10565187	3.89E-06	0.001775769	FDR0.01	19	1468943	'APC2;C19orf25	anaphase promoting complex subunit 2;UPF0449 protein C19orf25	Body;Body;TES5000	10271	'C19orf25	UPF0449 protein C19orf25
GA	cg10570754	5.33E-06	0.00221439	FDR0.01	12	5589444	'NTF3	neurotrophin-3 precursor	Body	-13853	'NTF3	neurotrophin-3 precursor
GA	cg10577216	2.20E-05	0.005949657	FDR0.01	6	143265045	'HIVEP2	transcription factor HIVEP2	Body	1268	'HIVEP2	transcription factor HIVEP2
GA	cg10585962	1.53E-05	0.004670003	FDR0.01	11	75428198	'MOGAT2	2-acylglycerol O-acyltransferase 2	TSS1500	-684	'MOGAT2	2-acylglycerol O-acyltransferase 2
GA	cg10592521	2.30E-06	0.001230261	FDR0.01	19	1560658	'MEX3D	RNA-binding protein MEX3D isoform 1	Body;Body	7399	'MEX3D	RNA-binding protein MEX3D isoform 1
GA	cg10609310	2.50E-05	0.006452624	FDR0.01	8	38722935	'			-35928	'PLEKHA2	pleckstrin homology domain-containing family A member 2
GA	cg10611580	3.36E-06	0.00158657	FDR0.01	17	75319942	'SEPTIN9	septin-9	Body;Body;Body;Body	4346	'SEPTIN9	septin-9
GA	cg10612237	3.54E-07	0.000313298	FDR0.01	10	80827823	'ZMIZ1-AS1;ZMIZ1	ZMIZ1 antisense RNA 1;zinc finger MIZ domain-containing protein 1	TSS1500;TSS1500;TSS1500;TSS1	-618	'ZMIZ1-AS1	ZMIZ1 antisense RNA 1

Trait	#cpg	p	fdr	significance	chr	pos.hg19	gene.Illumina	gene.description	locations	dist2TSS	gene.nearest	gene.description
GA	cg10628205	3.26E-05	0.00760708	FDR0.01	1	61547131	'NFIA	nuclear factor 1 A-type	Body;TSS1500;TSS1500;TSS150	-279	'NFIA	nuclear factor 1 A-type
GA	cg10658438	2.49E-09	8.07E-06	BF|FDR0.01	3	194117018	'GP5	platelet glycoprotein V precursor	3'UTR	2977	'GP5	platelet glycoprotein V precursor
GA	cg10699871	3.97E-07	0.000342211	FDR0.01	22	19879696	'TXNRD2	thioredoxin reductase 2, mitochondrial	Body;Body;Body;Body;Body;TE	-37299	'RTL10	protein Bop
GA	cg10705800	5.83E-07	0.000449995	FDR0.01	1	41328420	'CITED4	cbp/p300-interacting transactivator 4	TSS1500	-385	'CITED4	cbp/p300-interacting transactivator 4
GA	cg10714284	5.58E-07	0.000435319	FDR0.01	6	32908901	'HLA-DMB	HLA class II histocompatibility antigen, DM beta chain precursor	TSS200	-96	'HLA-DMB	HLA class II histocompatibility antigen, DM beta chain precursor
GA	cg10718608	1.39E-05	0.004351996	FDR0.01	17	19438221	'SLC47A1	multidrug and toxin extrusion protein 1	Body	1055	'SLC47A1	multidrug and toxin extrusion protein 1
GA	cg10742957	2.55E-06	0.001300715	FDR0.01	19	39466716	'FBXO17	F-box only protein 17	TSS1500;TSS1500	-334	'FBXO17	F-box only protein 17
GA	cg10745272	4.28E-05	0.009073464	FDR0.01	4	2819770	'SH3BP2	SH3 domain-binding protein 2	Body;Body;TSS200;TSS1500	-126	'SH3BP2	SH3 domain-binding protein 2
GA	cg10799440	2.78E-05	0.006911162	FDR0.01	8	120685686	'ENPP2	ectonucleotide pyrophosphatase/phosphodiesterase family member 2	TSS200	-56	'ENPP2	ectonucleotide pyrophosphatase/phosphodiesterase family member 2
GA	cg10805254	2.59E-05	0.006622972	FDR0.01	3	72433837	'RYBP	ring and YY1 binding protein, isoform A	Body	61937	'RYBP	ring and YY1 binding protein, isoform A
GA	cg10814005	4.36E-05	0.009207403	FDR0.01	14	91711041	'GPR68	ovarian cancer G-protein coupled receptor 1	5'UTR;Body;Body	7	'GPR68	ovarian cancer G-protein coupled receptor 1
GA	cg10844760	7.63E-09	1.82E-05	BF|FDR0.01	2	7017723	'RSAD2	radical S-adenosyl methionine domain-containing protein 2	TSS200	-184	'RSAD2	radical S-adenosyl methionine domain-containing protein 2
GA	cg10857905	6.67E-06	0.00260535	FDR0.01	10	80026963	'LINC00595	long intergenic non-protein coding RNA 595	TSS200	-135	'LINC00595	long intergenic non-protein coding RNA 595
GA	cg10860819	3.04E-06	0.001475318	FDR0.01	12	40619282	'LRRK2	leucine-rich repeat serine/threonine-protein kinase 2	Body	484	'LRRK2	leucine-rich repeat serine/threonine-protein kinase 2
GA	cg10867751	4.68E-05	0.009616031	FDR0.01	1	25870981	'LDLRAP1	low density lipoprotein receptor adapter protein 1	Body	885	'LDLRAP1	low density lipoprotein receptor adapter protein 1
GA	cg10893437	3.95E-05	0.008669363	FDR0.01	13	115079492	'CHAMP1	chromosome alignment-maintaining phosphoprotein 1	TSS1500;TSS1500;TSS1500	-485	'CHAMP1	chromosome alignment-maintaining phosphoprotein 1
GA	cg10938046	6.32E-07	0.000481557	FDR0.01	6	43969577	'LOC101929705;C6orf223	;chromosome 6 open reading frame 223	Body;Body;Body;Body;Body	1256	'C6orf223	chromosome 6 open reading frame 223
GA	cg10947827	4.64E-06	0.002000994	FDR0.01	2	201980652	'CFLAR	CASP8 and FADD-like apoptosis regulator	TSS200;TSS200;TSS1500;TSS15	-174	'CFLAR	CASP8 and FADD-like apoptosis regulator
GA	cg10951873	1.21E-05	0.003943536	FDR0.01	1	25254746	'RUNX3	runt-related transcription factor 3 isoform 1	Body;Body;Body	2021	'RUNX3	runt-related transcription factor 3 isoform 1
GA	cg10954182	4.22E-06	0.001873061	FDR0.01	12	104532377	'NFYB	nuclear transcription factor Y subunit beta	TSS1500	-389	'NFYB	nuclear transcription factor Y subunit beta
GA	cg10977734	2.67E-07	0.000251907	FDR0.01	6	30582416	'PPP1R10;MRPS18B	serine/threonine-protein phosphatase 1 regulatory subunit 10;28S ribosoma	Body;Body;TSS5000	2604	'PPP1R10	serine/threonine-protein phosphatase 1 regulatory subunit 10
GA	cg10978799	1.11E-06	0.00072688	FDR0.01	1	206945924	'IL10;IL19	interleukin-10 precursor;interleukin-19 isoform 1	TSS200;Body	-85	'IL10	interleukin-10 precursor
GA	cg10980102	2.54E-05	0.006528281	FDR0.01	12	6166874	'VWF	von Willebrand factor precursor	Body	-17525	'SNORA120	small nucleolar RNA, H/ACA box 120
GA	cg11027221	2.56E-06	0.001303031	FDR0.01	11	67770665	'UNC93B1	protein unc-93 homolog B1	Body	902	'UNC93B1	protein unc-93 homolog B1
GA	cg11045814	4.04E-05	0.008776111	FDR0.01	5	150461165	'TNIP1	TNFAIP3-interacting protein 1	TSS1500;TSS1500;TSS1500;TSS1	-520	'TNIP1	TNFAIP3-interacting protein 1
GA	cg11051158	4.09E-07	0.000349502	FDR0.01	2	75938223	'GCFC2	GC-rich sequence DNA-binding factor 2	TSS200;TSS200;TSS200	-182	'GCFC2	GC-rich sequence DNA-binding factor 2
GA	cg11057378	1.71E-08	3.13E-05	BF|FDR0.01	10	81107060	'PPIF	peptidyl-prolyl cis-trans isomerase F, mitochondrial	TSS200	-164	'PPIF	peptidyl-prolyl cis-trans isomerase F, mitochondrial
GA	cg11087049	2.33E-05	0.006158722	FDR0.01	14	51296586	'NIN	ninein isoform 5	Body;Body;Body;Body	1253	'NIN	ninein isoform 5
GA	cg11092416	1.04E-05	0.003513867	FDR0.01	6	29617549	'			-7319	'MOG	myelin-oligodendrocyte glycoprotein precursor
GA	cg11101926	1.36E-05	0.004282253	FDR0.01	17	32483711	'ASIC2	acid-sensing ion channel 2	5'UTR	114	'ASIC2	acid-sensing ion channel 2
GA	cg11105610	4.19E-07	0.000356459	FDR0.01	17	76976352	'LGALS3BP	galectin-3-binding protein precursor	TSS1500	-347	'LGALS3BP	galectin-3-binding protein precursor
GA	cg11118235	4.34E-05	0.009167231	FDR0.01	3	50284010	'GNAI2	guanine nucleotide-binding protein G(i) subunit alpha-2	Body;Body;Body;Body;Body;TS	-331	'GNAI2	guanine nucleotide-binding protein G(i) subunit alpha-2
GA	cg11122944	7.98E-06	0.002943129	FDR0.01	17	9924800	'GAS7	growth arrest-specific protein 7	Body;Body;Body	4802	'GAS7	growth arrest-specific protein 7
GA	cg11124350	3.75E-06	0.001725061	FDR0.01	7	1251125	'			-21382	'UNCX	homeobox protein unc-4 homolog
GA	cg11182874	2.60E-05	0.006642948	FDR0.01	8	102472508	'			-32158	'GRHL2	grainyhead-like protein 2 homolog isoform 2
GA	cg11196848	2.67E-05	0.006739121	FDR0.01	8	16859833	'FGF20	fibroblast growth factor 20	5'UTR	21	'FGF20	fibroblast growth factor 20
GA	cg11216632	1.92E-06	0.001072185	FDR0.01	1	155944655	'ARHGEF2	rho guanine nucleotide exchange factor 2	Body;Body;Body;Body;Body;Bo	3311	'ARHGEF2	rho guanine nucleotide exchange factor 2
GA	cg11216832	9.06E-07	0.000622238	FDR0.01	10	104218498	'RPARP-AS1;MFSD13A	;transmembrane protein 180	TES5000;TES5000;TSS5000	-2653	'MFSD13A	transmembrane protein 180
GA	cg11217218	1.84E-07	0.000189199	FDR0.01	17	79521061	'FAAP100;NPLOC4	Fanconi anemia core complex-associated protein 100;nuclear protein localiza	TSS5000;TSS5000;TES5000;TES	-1682	'FAAP100	Fanconi anemia core complex-associated protein 100
GA	cg11217654	2.82E-06	0.001403768	FDR0.01	11	9283463	'DENND5A	DENN domain-containing protein 5A isoform 4	Body;Body;Body;Body;Body	3410	'DENND5A	DENN domain-containing protein 5A isoform 4
GA	cg11229399	2.25E-07	0.000220764	FDR0.01	1	228689056	'			10933	'BTNL10	butyrophilin-like protein 10
GA	cg11254532	5.25E-06	0.002192938	FDR0.01	1	61520399	'			-22499	'NFIA	nuclear factor 1 A-type
GA	cg11261958	9.17E-09	2.01E-05	BF|FDR0.01	3	118958539	'B4GALT4;B4GALT4-AS1	beta-1,4-galactosyltransferase 4;B4GALT4 antisense RNA 1	Body;Body;TES5000	1186	'B4GALT4	beta-1,4-galactosyltransferase 4
GA	cg11263296	1.59E-05	0.004792161	FDR0.01	12	120428313	'BICDL1	BICD family-like cargo adapter 1 isoform 2	Body;Body;Body;Body;Body	666	'BICDL1	BICD family-like cargo adapter 1 isoform 2
GA	cg11267410	7.33E-06	0.002771133	FDR0.01	15	65360474	'RASL12	ras-like protein family member 12	TSS200;TSS200	-145	'RASL12	ras-like protein family member 12
GA	cg11308211	4.13E-05	0.008899023	FDR0.01	2	172750783	'SLC25A12	calcium-binding mitochondrial carrier protein Aralar1	TSS200;Body	-29	'SLC25A12	calcium-binding mitochondrial carrier protein Aralar1
GA	cg11321035	8.15E-07	0.000581312	FDR0.01	19	13944814	'ZSWIM4;LOC284454;MIR24-2;MIR27A;MIR23A	zinc finger SWIM domain-containing protein 4 isoform 1;;microRNA 24-2;mic	TES5000;TES5000;TES5000;TES	2359	'MIR24-2	microRNA 24-2
GA	cg11328127	4.68E-05	0.009616031	FDR0.01	11	415441	'SIGIRR;ANO9	single Ig IL-1-related receptor;anoctamin-9 isoform 2	TSS1500;Body;Body;TES5000;T	-442	'SIGIRR	single Ig IL-1-related receptor
GA	cg11341610	4.49E-06	0.001950161	FDR0.01	19	13050931	'CALR;MIR6515	calreticulin precursor;microRNA 6515	Body;TSS1500	-366	'MIR6515	microRNA 6515
GA	cg11351908	4.85E-05	0.00987219	FDR0.01	17	75402473	'SEPTIN9	septin-9	Body;Body;Body;Body;Body;Bo	1315	'SEPTIN9	septin-9
GA	cg11352146	7.44E-09	1.81E-05	BF|FDR0.01	11	65192550	'NEAT1	nuclear paraspeckle assembly transcript 1	Body;Body	2282	'NEAT1	nuclear paraspeckle assembly transcript 1
GA	cg11360522	8.13E-06	0.002991402	FDR0.01	13	113379828	'ATP11A	probable phospholipid-transporting ATPase IH	Body;Body	29217	'ATP11A-AS1	ATP11A antisense RNA 1
GA	cg11376341	1.19E-07	0.000138292	FDR0.01	10	21796152	'			-9963	'MIR1915HG	MIR1915 host gene
GA	cg11396509	2.83E-06	0.001406561	FDR0.01	19	39389915	'SIRT2;NFKBIB	NAD-dependent protein deacetylase sirtuin-2;NF-kappa-B inhibitor beta	Body;Body;Body;TSS1500;TSS1	343	'SIRT2	NAD-dependent protein deacetylase sirtuin-2
GA	cg11402363	2.63E-05	0.006675936	FDR0.01	18	908505	'ADCYAP1	pituitary adenylate cyclase-activating polypeptide precursor	Body;Body	3139	'ADCYAP1	pituitary adenylate cyclase-activating polypeptide precursor
GA	cg11405655	7.27E-06	0.002754318	FDR0.01	1	999599	'LOC100288175	uncharacterized LOC100288175	Body	4486	'LOC100288175	uncharacterized LOC100288175
GA	cg11435826	1.13E-07	0.000133409	FDR0.01	12	2792111	'CACNA1C;CACNA1C-AS1	voltage-dependent L-type calcium channel subunit alpha-1C;CACNA1C antise	Body;Body;Body;Body;Body;Bo	8212	'CACNA1C-AS1	CACNA1C antisense RNA 1
GA	cg11436362	1.29E-10	9.68E-07	BF|FDR0.01	11	67053929	'GRK2;ANKRD13D	beta-adrenergic receptor kinase 1;ankyrin repeat domain-containing protein	3'UTR;TSS5000;TSS5000	-2844	'ANKRD13D	ankyrin repeat domain-containing protein 13D
GA	cg11461670	1.57E-06	0.000935437	FDR0.01	8	22454935	'PDLIM2;C8orf58	PDZ and LIM domain protein 2;uncharacterized protein C8orf58 isoform 2	TES5000;TES5000;TES5000;3'U	-2176	'C8orf58	uncharacterized protein C8orf58 isoform 2
GA	cg11466832	6.33E-06	0.002514288	FDR0.01	2	11885560	'LPIN1	phosphatidate phosphatase LPIN1	Body;Body;Body;Body;TSS1500	-1197	'LPIN1	phosphatidate phosphatase LPIN1
GA	cg11469540	1.15E-05	0.00378478	FDR0.01	17	21189254	'MAP2K3	dual specificity mitogen-activated protein kinase kinase 3	Body;Body;TSS5000	1232	'MAP2K3	dual specificity mitogen-activated protein kinase kinase 3
GA	cg11512608	2.03E-09	7.08E-06	BF|FDR0.01	11	73357276	'PLEKHB1	pleckstrin homology domain-containing family B member 1	TSS1500;TSS1500;TSS1500;TSS1	-349	'PLEKHB1	pleckstrin homology domain-containing family B member 1
GA	cg11526198	8.31E-07	0.000588745	FDR0.01	1	23854847	'E2F2;LOC101928163	transcription factor E2F2;uncharacterized LOC101928163	Body;Body	2878	'E2F2	transcription factor E2F2
GA	cg11538810	4.34E-05	0.009167231	FDR0.01	22	46481727	'MIRLET7BHG	MIRLET7B host gene	TSS200;TSS200	-149	'MIRLET7BHG	MIRLET7B host gene
GA	cg11570656	2.18E-05	0.005922042	FDR0.01	13	45149702	'TSC22D1;TSC22D1-AS1	TSC22 domain family protein 1 isoform 3;TSC22D1 antisense RNA 1	1stExon;1stExon;TSS1500	-329	'TSC22D1-AS1	TSC22D1 antisense RNA 1
GA	cg11572844	5.91E-06	0.002390532	FDR0.01	5	129239273	'CHSY3	chondroitin sulfate synthase 3	TSS1500	-884	'CHSY3	chondroitin sulfate synthase 3
GA	cg11579421	2.14E-07	0.000212682	FDR0.01	10	134211857	'PWWP2B	PWWP domain-containing protein 2B	Body;Body	1154	'PWWP2B	PWWP domain-containing protein 2B
GA	cg11594821	3.66E-05	0.008278686	FDR0.01	6	30458601	'HLA-E	HLA class I histocompatibility antigen, alpha chain E precursor	Body	1316	'HLA-E	HLA class I histocompatibility antigen, alpha chain E precursor
GA	cg11621113	8.91E-09	1.99E-05	BF|FDR0.01	19	12776725	'MAN2B1;WDR83;WDR83OS	lysosomal alpha-mannosidase;WD repeat domain-containing protein 83;prot	Body;Body;TSS1500;TSS1500;T	831	'MAN2B1	lysosomal alpha-mannosidase
GA	cg11621911	1.25E-06	0.000795012	FDR0.01	13	102069296	'NALCN	sodium leak channel non-selective protein isoform 1	TSS1500;TSS1500;TSS1500;TSS1	-437	'NALCN	sodium leak channel non-selective protein isoform 1
GA	cg11629955	4.08E-05	0.008825587	FDR0.01	3	126679199	'CHCHD6	MICOS complex subunit MIC25	3'UTR;3'UTR	-28237	'PLXNA1	plexin-A1 precursor
GA	cg11631063	3.52E-07	0.000312548	FDR0.01	4	184679835	'			-39339	'STOX2	storkhead-box protein 2
GA	cg11663144	1.05E-05	0.00353331	FDR0.01	21	46675770	'LINC00334	long intergenic non-protein coding RNA 334	Body	21516	'LINC00334	long intergenic non-protein coding RNA 334
GA	cg11687085	3.18E-05	0.007478357	FDR0.01	18	77724447	'HSBP1L1	heat shock factor-binding protein 1-like protein 1	TSS200	-195	'HSBP1L1	heat shock factor-binding protein 1-like protein 1
GA	cg11697588	2.36E-05	0.006209959	FDR0.01	11	14995231	'CALCA	calcitonin isoform 1 preproprotein	TSS1500;TSS1500;TSS1500;TSS1	-1334	'CALCA	calcitonin isoform 1 preproprotein
GA	cg11698035	4.89E-06	0.002074155	FDR0.01	17	73105356	'SLC16A5;ARMC7	monocarboxylate transporter 6;armadillo repeat-containing protein 7	TES5000;TES5000;TES5000;TSS1	-707	'ARMC7	armadillo repeat-containing protein 7
GA	cg11700584	8.33E-07	0.000588745	FDR0.01	14	50088544	'RPL36AL;MGAT2;DNAAF2	60S ribosomal protein L36a;alpha-1,6-mannosyl-glycoprotein 2-beta-N-acety	TSS1500;1stExon;TES5000;TES5	1028	'MGAT2	alpha-1,6-mannosyl-glycoprotein 2-beta-N-acetylglucosaminyltransferase
GA	cg11751927	5.42E-06	0.002241996	FDR0.01	11	117857137	'IL10RA	interleukin-10 receptor subunit alpha precursor	5'UTR;Body	29	'IL10RA	interleukin-10 receptor subunit alpha precursor
GA	cg11776124	2.88E-05	0.007076105	FDR0.01	3	101395209	'ZBTB11;ZBTB11-AS1;RPL24	zinc finger and BTB domain-containing protein 11;ZBTB11 antisense RNA 1;6	Body;TSS200;TES5000	-64	'ZBTB11-AS1	ZBTB11 antisense RNA 1
GA	cg11789885	3.82E-05	0.008520256	FDR0.01	19	11472731	'PLPPR2	phospholipid phosphatase-related protein type 2 isoform 2	Body;Body	6624	'PLPPR2	phospholipid phosphatase-related protein type 2 isoform 2
GA	cg11791427	2.81E-05	0.006946891	FDR0.01	7	73704194	'CLIP2	CAP-Gly domain-containing linker protein 2	Body;Body	458	'CLIP2	CAP-Gly domain-containing linker protein 2
GA	cg11791710	2.61E-06	0.001319172	FDR0.01	10	129172535	'DOCK1	dedicator of cytokinesis protein 1 isoform 1	Body;Body	-175077	'NPS	neuropeptide S precursor
GA	cg11795753	2.91E-05	0.007120757	FDR0.01	12	133337036	'ANKLE2	ankyrin repeat and LEM domain-containing protein 2	Body	1382	'ANKLE2	ankyrin repeat and LEM domain-containing protein 2
GA	cg11808757	6.75E-06	0.002615681	FDR0.01	22	50683907	'TUBGCP6;HDAC10	gamma-tubulin complex component 6;polyamine deacetylase HDAC10	TSS1500;3'UTR;3'UTR	-455	'TUBGCP6	gamma-tubulin complex component 6
GA	cg11820154	3.84E-10	2.12E-06	BF|FDR0.01	8	29732714	'			-46314	'LINC02209	long intergenic non-protein coding RNA 2209
GA	cg11821039	2.65E-05	0.006704434	FDR0.01	2	241937756	'SNED1	sushi, nidogen and EGF-like domain-containing protein 1 precursor	TSS1500	-278	'SNED1	sushi, nidogen and EGF-like domain-containing protein 1 precursor
GA	cg11821439	5.06E-08	7.07E-05	BF|FDR0.01	7	29833650	'			-12519	'WIPF3	WAS/WASL-interacting protein family member 3

Trait	#cpg	p	fdr	significance	chr	pos.hg19	gene.Illumina	gene.description	locations	dist2TSS	gene.nearest	gene.description
GA	cg11824191	2.26E-05	0.00605623	FDR0.01	1	183274124	'NMNAT2	nicotinamide/nicotinic acid mononucleotide adenylyltransferase 2	TSS200;Body	-115	'NMNAT2	nicotinamide/nicotinic acid mononucleotide adenylyltransferase 2
GA	cg11832281	4.72E-06	0.002019092	FDR0.01	10	11211022	'CELF2	CUGBP Elav-like family member 2	Body;Body;Body;Body;Body;Bo	4030	'CELF2	CUGBP Elav-like family member 2
GA	cg11835355	1.83E-05	0.00528688	FDR0.01	22	36561746	'APOL3	apolipoprotein L3	TSS5000;5'UTR;5'UTR;TSS5000;	479	'APOL3	apolipoprotein L3
GA	cg11839863	4.11E-06	0.001845388	FDR0.01	5	142066121	'FGF1	fibroblast growth factor 1	TSS1500;TSS1500;TSS1500;TSS1	-61	'FGF1	fibroblast growth factor 1
GA	cg11869018	9.57E-06	0.003319253	FDR0.01	5	116378307	'			-280402	'LINC02214	long intergenic non-protein coding RNA 2214
GA	cg11931626	7.48E-08	9.72E-05	BF|FDR0.01	4	184679532	'			-39642	'STOX2	storkhead-box protein 2
GA	cg11934771	1.03E-06	0.000690501	FDR0.01	6	31021796	'HCG22	protein PBMUCL2 precursor	Body;Body	570	'HCG22	protein PBMUCL2 precursor
GA	cg11934832	1.23E-10	9.50E-07	BF|FDR0.01	18	45683841	'ZBTB7C	zinc finger and BTB domain-containing protein 7C	Body;Body;Body;Body;Body;Bo	-20322	'ZBTB7C	zinc finger and BTB domain-containing protein 7C
GA	cg11935248	4.88E-06	0.002071724	FDR0.01	6	4942483	'CDYL	chromodomain Y-like protein	Body;Body;Body;Body;Body;Bo	52210	'CDYL	chromodomain Y-like protein
GA	cg11945297	4.22E-07	0.000357617	FDR0.01	8	102514634	'GRHL2	grainyhead-like protein 2 homolog isoform 2	Body;Body	9968	'GRHL2	grainyhead-like protein 2 homolog isoform 2
GA	cg11947782	1.02E-06	0.000688622	FDR0.01	1	9775985	'PIK3CD	phosphatidylinositol 4,5-bisphosphate 3-kinase catalytic subunit delta isofor	Body;Body;Body	24461	'PIK3CD	phosphatidylinositol 4,5-bisphosphate 3-kinase catalytic subunit delta isoform
GA	cg11950754	4.94E-06	0.002087972	FDR0.01	1	53782077	'LRP8	low-density lipoprotein receptor-related protein 8 precursor	Body;Body;Body;Body	11665	'LRP8	low-density lipoprotein receptor-related protein 8 precursor
GA	cg11952714	1.24E-06	0.000786457	FDR0.01	1	99126623	'SNX7	sorting nexin-7	TSS1500;TSS1500;TSS1500;TSS1	-653	'SNX7	sorting nexin-7
GA	cg11953775	1.74E-08	3.13E-05	BF|FDR0.01	1	207597011	'			-30633	'CR2	complement receptor type 2 precursor
GA	cg11954313	4.17E-05	0.008952664	FDR0.01	17	28921927	'SMURF2P1-LRRC37BP1	SMURF2P1-LRRC37BP1 readthrough transcribed pseudogene	Body	18445	'SMURF2P1-LRRC37BP1	SMURF2P1-LRRC37BP1 readthrough transcribed pseudogene
GA	cg11960677	4.02E-06	0.001820353	FDR0.01	1	32705760	'MTMR9LP	myotubularin related protein 9 like, pseudogene	Body	1551	'MTMR9LP	myotubularin related protein 9 like, pseudogene
GA	cg11969813	4.61E-05	0.009541576	FDR0.01	17	79816559	'P4HB	protein disulfide-isomerase precursor	Body	1852	'P4HB	protein disulfide-isomerase precursor
GA	cg11977443	5.74E-08	7.83E-05	BF|FDR0.01	17	77721007	'ENPP7	ectonucleotide pyrophosphatase/phosphodiesterase family member 7 precu	TES5000	16303	'ENPP7	ectonucleotide pyrophosphatase/phosphodiesterase family member 7 precursor
GA	cg11993506	1.84E-05	0.005308068	FDR0.01	4	1593537	'			92181	'FAM53A	protein FAM53A isoform 1
GA	cg12036924	8.09E-07	0.000578117	FDR0.01	19	5838731	'FUT6;FUT3	4-galactosyl-N-acetylglucosaminide 3-alpha-L-fucosyltransferase FUT6 isofor	5'UTR;5'UTR;5'UTR;5'UTR;5'UT	72	'FUT6	4-galactosyl-N-acetylglucosaminide 3-alpha-L-fucosyltransferase FUT6 isoform 1
GA	cg12051710	1.38E-09	5.28E-06	BF|FDR0.01	12	2989070	'FOXM1;RHNO1	forkhead box protein M1;RAD9, HUS1, RAD1-interacting nuclear orphan prot	TSS5000;TSS5000;TSS5000;TSS5	2682	'RHNO1	RAD9, HUS1, RAD1-interacting nuclear orphan protein 1
GA	cg12064202	3.76E-06	0.001727027	FDR0.01	6	31038842	'			17616	'HCG22	protein PBMUCL2 precursor
GA	cg12071775	3.47E-07	0.000309606	FDR0.01	14	52591786	'			-56009	'NID2	nidogen-2 precursor
GA	cg12074985	2.06E-05	0.00571205	FDR0.01	10	99080756	'FRAT1	proto-oncogene FRAT1	3'UTR	1729	'FRAT1	proto-oncogene FRAT1
GA	cg12121075	1.94E-05	0.005479209	FDR0.01	12	121615201	'P2RX7	P2X purinoceptor 7	Body;Body;Body;Body;Body;Bo	-32730	'P2RX4	P2X purinoceptor 4
GA	cg12127706	4.54E-06	0.001964364	FDR0.01	11	70261959	'CTTN	src substrate cortactin	Body;Body;Body	17325	'CTTN	src substrate cortactin
GA	cg12140144	2.97E-08	4.79E-05	BF|FDR0.01	1	2984275	'PRDM16-DT;PRDM16	PRDM16 divergent transcript;histone-lysine N-methyltransferase PRDM16 is	Body;Body;TSS1500;TSS1500	14	'PRDM16-DT	PRDM16 divergent transcript
GA	cg12160741	1.44E-05	0.004466242	FDR0.01	14	105760556	'BRF1	transcription factor TFIIIB subunit BRF1	Body;Body;Body;Body;Body	-6554	'PACS2	phosphofurin acidic cluster sorting protein 2 isoform 2
GA	cg12167830	2.47E-05	0.00640322	FDR0.01	1	57111108	'LOC101929935;PRKAA2	uncharacterized LOC101929935;5'-AMP-activated protein kinase catalytic su	TSS200;1stExon	-117	'LOC101929935	uncharacterized LOC101929935
GA	cg12185873	1.05E-06	0.000699251	FDR0.01	14	106355821	'			-28016	'FAM30A	family with sequence similarity 30 member A
GA	cg12194745	1.94E-05	0.005488396	FDR0.01	17	79423649	'BAHCC1	BAH and coiled-coil domain-containing protein 1 isoform 1	Body	-5435	'MIR3186	microRNA 3186
GA	cg12229555	2.16E-05	0.005889581	FDR0.01	17	80260329	'			-9639	'LINC01970	long intergenic non-protein coding RNA 1970
GA	cg12245247	1.44E-06	0.000877703	FDR0.01	19	56127147	'ZNF865;ZNF784	zinc finger protein 865;zinc finger protein 784	1stExon;TES5000	2189	'ZNF865	zinc finger protein 865
GA	cg12272369	2.90E-05	0.007106601	FDR0.01	10	95848841	'PLCE1;PLCE1-AS2	1-phosphatidylinositol 4,5-bisphosphate phosphodiesterase epsilon-1;PLCE1	Body;Body;Body;5'UTR	59	'PLCE1	1-phosphatidylinositol 4,5-bisphosphate phosphodiesterase epsilon-1
GA	cg12277998	1.82E-05	0.005279529	FDR0.01	20	33999883	'UQCC1	ubiquinol-cytochrome c reductase complex assembly factor 1	5'UTR;5'UTR;TSS200	62	'UQCC1	ubiquinol-cytochrome c reductase complex assembly factor 1
GA	cg12291829	2.12E-05	0.005840177	FDR0.01	5	42991862	'FLJ32255	uncharacterized LOC643977	Body	1573	'FLJ32255	uncharacterized LOC643977
GA	cg12295918	3.76E-05	0.008435063	FDR0.01	3	46506481	'LTF	lactotransferrin precursor	TSS1500;TSS200;TSS200;Body	-86	'LTF	lactotransferrin precursor
GA	cg12324629	2.69E-05	0.006758436	FDR0.01	17	19771548	'ULK2	serine/threonine-protein kinase ULK2	TSS1500;TSS1500	-299	'ULK2	serine/threonine-protein kinase ULK2
GA	cg12359279	3.12E-05	0.007400248	FDR0.01	21	42797953	'MX1	interferon-induced GTP-binding protein Mx1	Body;TSS200;TSS1500	-4	'MX1	interferon-induced GTP-binding protein Mx1
GA	cg12424598	2.46E-06	0.001269693	FDR0.01	1	151788227	'RORC	nuclear receptor ROR-gamma	Body;Body	-10332	'LINGO4	leucine-rich repeat and immunoglobulin-like domain-containing nogo receptor-interacting protein 4 precursor
GA	cg12431087	1.04E-06	0.000690961	FDR0.01	3	71355122	'FOXP1;FOXP1-AS1	forkhead box protein P1;FOXP1 antisense RNA 1	TSS1500;Body;Body;Body;Body	-1200	'FOXP1	forkhead box protein P1
GA	cg12433486	6.04E-06	0.002426612	FDR0.01	15	72520804	'PKM	pyruvate kinase PKM	Body;Body;Body;Body;Body;Bo	1448	'PKM	pyruvate kinase PKM
GA	cg12434312	2.32E-05	0.006139122	FDR0.01	10	129845509	'PTPRE	receptor-type tyrosine-protein phosphatase epsilon	Body;Body;Body;Body;Body;Bo	-366	'PTPRE	receptor-type tyrosine-protein phosphatase epsilon
GA	cg12434587	8.20E-07	0.000584078	FDR0.01	10	131265209	'MGMT	methylated-DNA--protein-cysteine methyltransferase	TSS1500	-295	'MGMT	methylated-DNA--protein-cysteine methyltransferase
GA	cg12436631	5.51E-06	0.002271833	FDR0.01	1	155007014	'DCST2;DCST1	DC-STAMP domain-containing protein 2;E3 ubiquitin-protein ligase DCST1	TSS1500;Body;Body	733	'DCST1	E3 ubiquitin-protein ligase DCST1
GA	cg12461930	4.42E-07	0.000368854	FDR0.01	1	228345378	'GJC2	gap junction gamma-2 protein	Body	7171	'IBA57-DT	IBA57 divergent transcript
GA	cg12466700	1.32E-06	0.000823497	FDR0.01	3	69789955	'MITF	microphthalmia-associated transcription factor	Body;Body;Body;Body;Body;Bo	1341	'MITF	microphthalmia-associated transcription factor
GA	cg12467404	2.13E-06	0.001153723	FDR0.01	1	207496045	'CD55	complement decay-accelerating factor precursor	Body;Body;Body;Body;Body;Bo	1023	'CD55	complement decay-accelerating factor precursor
GA	cg12478185	1.00E-06	0.000675479	FDR0.01	17	10602001	'SCO1;ADPRM	Cu-binding protein SCO1;manganese-dependent ADP-ribose/CDP-alcohol dip	TSS1500;Body	1091	'ADPRM	manganese-dependent ADP-ribose/CDP-alcohol diphosphatase
GA	cg12490887	9.53E-08	0.000117461	BF|FDR0.01	9	127267658	'NR5A1	steroidogenic factor 1	Body	2020	'NR5A1	steroidogenic factor 1
GA	cg12498580	1.19E-07	0.000138292	FDR0.01	12	7070732	'PTPN6;MIR200CHG;MIR200C;MIR141;PHB2	tyrosine-protein phosphatase non-receptor type 6;MIR200C and MIR141 hos	TES5000;TES5000;TES5000;TSS5	-1675	'MIR200CHG	MIR200C and MIR141 host gene
GA	cg12526197	4.73E-05	0.009680863	FDR0.01	1	230416938	'GALNT2	polypeptide N-acetylgalactosaminyltransferase 2 precursor	3'UTR;3'UTR	145140	'PGBD5	piggyBac transposable element-derived protein 5
GA	cg12537337	1.87E-05	0.005356826	FDR0.01	6	26442290	'BTN3A3	butyrophilin subfamily 3 member A3 precursor	Body;Body;Body	1559	'BTN3A3	butyrophilin subfamily 3 member A3 precursor
GA	cg12547218	2.72E-05	0.006810712	FDR0.01	2	240153820	'HDAC4	histone deacetylase 4, isoform B	Body	-36667	'MGC16025	uncharacterized LOC85009
GA	cg12564453	2.01E-05	0.005602428	FDR0.01	16	56995840	'CETP	cholesteryl ester transfer protein precursor	TSS200;TSS200	-21	'CETP	cholesteryl ester transfer protein precursor
GA	cg12571570	2.34E-06	0.001240922	FDR0.01	2	145267984	'ZEB2	zinc finger E-box-binding homeobox 2	Body;Body;TES5000	-9196	'ZEB2-AS1	ZEB2 antisense RNA 1
GA	cg12577010	4.63E-05	0.009564714	FDR0.01	7	134832544	'CYREN;TMEM140	cell cycle regulator of non-homologous end joining;transmembrane protein 1	Body;TSS1500	-279	'TMEM140	transmembrane protein 140 precursor
GA	cg12584718	2.37E-06	0.001249385	FDR0.01	7	100028402	'ZCWPW1;MEPCE;PPP1R35	zinc finger CW-type PWWP domain protein 1;7SK snRNA methylphosphate ca	TSS5000;TSS5000;Body;Body;5'	949	'MEPCE	7SK snRNA methylphosphate capping enzyme
GA	cg12586150	2.16E-05	0.005889581	FDR0.01	6	2840792	'SERPINB1	leukocyte elastase inhibitor	1stExon;Body;Body	1305	'SERPINB1	leukocyte elastase inhibitor
GA	cg12587615	8.83E-09	1.99E-05	BF|FDR0.01	19	11472397	'PLPPR2	phospholipid phosphatase-related protein type 2 isoform 2	Body;Body	6290	'PLPPR2	phospholipid phosphatase-related protein type 2 isoform 2
GA	cg12614630	3.34E-05	0.007729903	FDR0.01	12	57387318	'GPR182	G-protein coupled receptor 182	TSS1500	-957	'GPR182	G-protein coupled receptor 182
GA	cg12626589	1.94E-06	0.001076846	FDR0.01	6	32120783	'PRRT1;LOC100507547;PPT2;PPT2-EGFL8	proline-rich transmembrane protein 1;uncharacterized LOC100507547;lysos	5'UTR;TSS1500;Body;Body;Body	77	'PRRT1	proline-rich transmembrane protein 1
GA	cg12646820	5.32E-06	0.002211868	FDR0.01	6	13924406	'RNF182	E3 ubiquitin-protein ligase RNF182	TSS1500;TSS1500;TSS1500;TSS1	-270	'RNF182	E3 ubiquitin-protein ligase RNF182
GA	cg12647283	3.03E-06	0.001470736	FDR0.01	10	126791396	'CTBP2	C-terminal-binding protein 2	Body;Body;Body;Body;Body;Bo	55950	'CTBP2	C-terminal-binding protein 2
GA	cg12655416	1.27E-05	0.004079606	FDR0.01	17	38077870	'GSDMB;ORMDL3	gasdermin-B;ORM1-like protein 3	TSS5000;TSS5000;TSS5000;TSS5	-2967	'GSDMB	gasdermin-B
GA	cg12662084	4.05E-05	0.008788927	FDR0.01	6	17809126	'KIF13A	kinesin-like protein KIF13A isoform a	Body;Body;Body;Body	101853	'LOC105374952	
GA	cg12662887	2.01E-05	0.005602428	FDR0.01	10	105343920	'NEURL1	E3 ubiquitin-protein ligase NEURL1	Body	-66721	'NEURL1-AS1	NEURL1 antisense RNA 1
GA	cg12689752	1.26E-05	0.004078461	FDR0.01	1	156495553	'IQGAP3	ras GTPase-activating-like protein IQGAP3	3'UTR	-24986	'MEF2D	myocyte-specific enhancer factor 2D
GA	cg12700271	1.88E-05	0.00535964	FDR0.01	6	30458237	'HLA-E	HLA class I histocompatibility antigen, alpha chain E precursor	Body	952	'HLA-E	HLA class I histocompatibility antigen, alpha chain E precursor
GA	cg12738763	1.02E-05	0.00348157	FDR0.01	10	134898887	'ADGRA1-AS1;ADGRA1	ADGRA1 antisense RNA 1;adhesion G protein-coupled receptor A1 isoform 2	Body;TSS5000	1314	'ADGRA1-AS1	ADGRA1 antisense RNA 1
GA	cg12743470	5.81E-06	0.002361871	FDR0.01	14	74815085	'VRTN	vertnin	TSS200	-88	'VRTN	vertnin
GA	cg12751042	1.76E-08	3.15E-05	BF|FDR0.01	12	12874288	'CDKN1B;APOLD1	cyclin-dependent kinase inhibitor 1B;apolipoprotein L domain-containing pro	3'UTR;TSS5000	4085	'CDKN1B	cyclin-dependent kinase inhibitor 1B
GA	cg12758082	5.43E-06	0.002246257	FDR0.01	13	113407769	'ATP11A;ATP11A-AS1	probable phospholipid-transporting ATPase IH;ATP11A antisense RNA 1	Body;Body;Body;Body	1276	'ATP11A-AS1	ATP11A antisense RNA 1
GA	cg12793711	2.47E-07	0.000237641	FDR0.01	17	79477853	'ACTG1	actin, cytoplasmic 2	Body;Body;Body	1972	'ACTG1	actin, cytoplasmic 2
GA	cg12798040	2.32E-05	0.006148619	FDR0.01	14	104171840	'KLC1;XRCC3	kinesin light chain 1;DNA repair protein XRCC3	TES5000;TES5000;Body;Body;B	9948	'XRCC3	DNA repair protein XRCC3
GA	cg12799029	7.55E-07	0.000545316	FDR0.01	17	984184	'ABR	active breakpoint cluster region-related protein isoform c	TSS5000;Body;Body;Body;Body	-1951	'ABR	active breakpoint cluster region-related protein isoform c
GA	cg12799818	1.51E-07	0.000164394	FDR0.01	17	19450343	'SLC47A1	multidrug and toxin extrusion protein 1	Body	-10529	'SNORA59B	small nucleolar RNA, H/ACA box 59B
GA	cg12819873	2.23E-05	0.005995871	FDR0.01	11	57157632	'PRG2	bone marrow proteoglycan isoform 1 preproprotein	Body;Body;Body;Body	491	'PRG2	bone marrow proteoglycan isoform 1 preproprotein
GA	cg12833872	3.03E-05	0.007286057	FDR0.01	5	501863	'SLC9A3	sodium/hydrogen exchanger 3 precursor	Body;Body	22701	'SLC9A3	sodium/hydrogen exchanger 3 precursor
GA	cg12835981	7.49E-06	0.002813862	FDR0.01	2	30670159	'LCLAT1	lysocardiolipin acyltransferase 1	5'UTR;5'UTR;5'UTR;5'UTR	48	'LCLAT1	lysocardiolipin acyltransferase 1
GA	cg12840248	2.19E-07	0.000216593	FDR0.01	6	32908718	'HLA-DMB	HLA class II histocompatibility antigen, DM beta chain precursor	5'UTR	87	'HLA-DMB	HLA class II histocompatibility antigen, DM beta chain precursor
GA	cg12883279	9.52E-08	0.000117461	BF|FDR0.01	6	32120773	'PRRT1;LOC100507547;PPT2;PPT2-EGFL8	proline-rich transmembrane protein 1;uncharacterized LOC100507547;lysos	5'UTR;TSS1500;Body;Body;Body	87	'PRRT1	proline-rich transmembrane protein 1
GA	cg12884779	3.51E-09	1.01E-05	BF|FDR0.01	5	172198737	'DUSP1	dual specificity protein phosphatase 1	TSS1500	-539	'DUSP1	dual specificity protein phosphatase 1

Trait	#cpg	p	fdr	significance	chr	pos.hg19	gene.Illumina	gene.description	locations	dist2TSS	gene.nearest	gene.description
GA	cg12903529	1.90E-05	0.005406402	FDR0.01	12	65277488	'TBC1D30;LINC02389	TBC1 domain family member 30;long intergenic non-protein coding RNA 238	TES5000;TES5000;TES5000;TES	-65	'LINC02389	long intergenic non-protein coding RNA 2389
GA	cg12949547	8.32E-06	0.00303357	FDR0.01	12	58718950	'			237276	'LINC02403	long intergenic non-protein coding RNA 2403
GA	cg12949616	4.82E-05	0.009827177	FDR0.01	19	18308021	'MPV17L2;RAB3A	mpv17-like protein 2 precursor;ras-related protein Rab-3A	TES5000;3'UTR	3994	'MPV17L2	mpv17-like protein 2 precursor
GA	cg12970171	3.14E-07	0.000287045	FDR0.01	5	6766613	'			-29379	'LINC02236	long intergenic non-protein coding RNA 2236
GA	cg12972233	2.85E-08	4.67E-05	BF|FDR0.01	9	91605893	'C9orf47;S1PR3	uncharacterized protein C9orf47 isoform 1 precursor;sphingosine 1-phospha	5'UTR;5'UTR;TSS1500	116	'C9orf47	uncharacterized protein C9orf47 isoform 1 precursor
GA	cg12978040	1.53E-08	2.92E-05	BF|FDR0.01	19	55768527	'PPP6R1	serine/threonine-protein phosphatase 6 regulatory subunit 1	Body	1858	'PPP6R1	serine/threonine-protein phosphatase 6 regulatory subunit 1
GA	cg12999267	6.56E-10	2.93E-06	BF|FDR0.01	12	94376970	'			-39588	'LOC105369911	uncharacterized LOC105369911
GA	cg13001963	6.35E-10	2.90E-06	BF|FDR0.01	5	10467973	'ROPN1L	ropporin-1-like protein	TES5000;TES5000	-10175	'MIR6131	microRNA 6131
GA	cg13008174	2.47E-05	0.006408289	FDR0.01	8	11640960	'NEIL2	endonuclease 8-like 2	Body;Body;Body;Body;Body;Bo	-12121	'FDFT1	squalene synthase
GA	cg13031210	8.32E-06	0.00303357	FDR0.01	12	45444902	'DBX2	homeobox protein DBX2	TSS200	-20	'DBX2	homeobox protein DBX2
GA	cg13036381	1.83E-06	0.001038268	FDR0.01	3	159942495	'C3orf80;TRIM59-IFT80	uncharacterized membrane protein C3orf80 precursor;TRIM59-IFT80 protein	TSS1500;TES5000	-927	'C3orf80	uncharacterized membrane protein C3orf80 precursor
GA	cg13052625	1.82E-06	0.001035738	FDR0.01	3	47865946	'DHX30	ATP-dependent RNA helicase DHX30 isoform 2	Body;Body;Body	21319	'DHX30	ATP-dependent RNA helicase DHX30 isoform 2
GA	cg13055385	1.81E-05	0.005259708	FDR0.01	13	25743999	'AMER2;LINC00463	APC membrane recruitment protein 2 isoform 1;long intergenic non-protein	Body;Body;TES5000	2306	'AMER2	APC membrane recruitment protein 2 isoform 1
GA	cg13066703	4.04E-05	0.008776111	FDR0.01	1	211526705	'TRAF5	TNF receptor-associated factor 5	1stExon;1stExon;1stExon;1stEx	7000	'TRAF5	TNF receptor-associated factor 5
GA	cg13078421	1.62E-05	0.004858132	FDR0.01	19	2624622	'GNG7	guanine nucleotide-binding protein G(I)/G(S)/G(O) subunit gamma-7	Body	-6090	'MIR7850	microRNA 7850
GA	cg13094313	1.07E-10	8.89E-07	BF|FDR0.01	14	62278847	'SYT16	synaptotagmin-16 isoform 4	TSS200;TSS1500;TSS1500;TSS1	-32	'SYT16	synaptotagmin-16 isoform 4
GA	cg13098318	2.10E-05	0.005792525	FDR0.01	6	34663277	'ILRUN;LOC101929243	protein ILRUN isoform a;	Body;Body;TSS1500;TSS1500	-653	'LOC101929243	
GA	cg13110239	1.88E-05	0.00535964	FDR0.01	22	38711267	'CSNK1E;TPTEP2-CSNK1E	casein kinase I;casein kinase I isoform epsilon	Body;Body;Body	2171	'CSNK1E	casein kinase I
GA	cg13167816	1.27E-05	0.004080207	FDR0.01	14	24601808	'FITM1;PSME1	fat storage-inducing transmembrane protein 1;proteasome activator comple	Body;TSS5000;TSS5000;TSS500	1134	'FITM1	fat storage-inducing transmembrane protein 1
GA	cg13175329	3.17E-06	0.001517244	FDR0.01	6	25035073	'RIPOR2	rho family-interacting cell polarization regulator 2	Body	7443	'RIPOR2	rho family-interacting cell polarization regulator 2
GA	cg13182713	2.91E-05	0.007120757	FDR0.01	17	73780396	'H3-3B;MIR4738;UNK	H3 histone family member 3B;microRNA 4738;RING finger protein unkempt	TSS5000;TES5000;TSS1500;TSS1	292	'MIR4738	microRNA 4738
GA	cg13185188	2.83E-06	0.001407015	FDR0.01	14	100680334	'			-25146	'YY1	transcriptional repressor protein YY1
GA	cg13213009	1.38E-07	0.000153385	FDR0.01	8	74903761	'LY96	lymphocyte antigen 96 precursor	1stExon;1stExon	135	'LY96	lymphocyte antigen 96 precursor
GA	cg13228442	1.07E-05	0.003593221	FDR0.01	22	41763512	'TEF	transcription factor VBP	5'UTR	176	'TEF	transcription factor VBP
GA	cg13251666	2.24E-05	0.005995871	FDR0.01	7	38375485	'			-5692	'TRG-AS1	T cell receptor gamma locus antisense RNA 1
GA	cg13272108	1.69E-05	0.004999317	FDR0.01	20	2634697	'NOP56;MIR1292;SNORD110;SNORA51;SNORD86;SN	nucleolar protein 56;microRNA 1292;small nucleolar RNA, C/D box 110;small	Body;Body;Body;TES5000;TSS2	-160	'SNORD110	small nucleolar RNA, C/D box 110
GA	cg13276502	1.28E-05	0.004099763	FDR0.01	6	21600485	'SOX4	transcription factor SOX-4	TES5000	6504	'SOX4	transcription factor SOX-4
GA	cg13279566	1.29E-05	0.0041368	FDR0.01	3	160823315	'B3GALNT1	UDP-GalNAc:beta-1, 3-N-acetylgalactosaminyltransferase 1	TSS1500;TSS1500;TSS1500;TSS2	-155	'B3GALNT1	UDP-GalNAc:beta-1, 3-N-acetylgalactosaminyltransferase 1
GA	cg13283635	9.99E-06	0.003422684	FDR0.01	20	61450653	'COL9A3	collagen alpha-3(IX) chain precursor	Body	2252	'COL9A3	collagen alpha-3(IX) chain precursor
GA	cg13299776	3.85E-08	5.78E-05	BF|FDR0.01	3	148847007	'HPS3	Hermansky-Pudlak syndrome 3 protein	TSS1500;TSS1500	-417	'HPS3	Hermansky-Pudlak syndrome 3 protein
GA	cg13315706	7.89E-06	0.002920484	FDR0.01	21	46341054	'ITGB2;ITGB2-AS1	integrin beta-2 precursor;ITGB2 antisense RNA 1	TSS1500;TSS1500;Body;Body;B	105	'ITGB2-AS1	ITGB2 antisense RNA 1
GA	cg13327336	4.17E-05	0.008952465	FDR0.01	3	50403312	'CYB561D2;CACNA2D2	cytochrome b561 domain-containing protein 2;voltage-dependent calcium c	Body;Body;Body;Body;Body;Bo	519	'CYB561D2	cytochrome b561 domain-containing protein 2
GA	cg13329242	4.57E-05	0.009477579	FDR0.01	11	117382759	'DSCAML1	Down syndrome cell adhesion molecule-like protein 1 isoform 3	Body;Body	184190	'CEP164	centrosomal protein of 164 kDa isoform 2
GA	cg13353574	3.47E-06	0.001619661	FDR0.01	8	61326925	'LINC01301	long intergenic non-protein coding RNA 1301	TSS1500	-548	'LINC01301	long intergenic non-protein coding RNA 1301
GA	cg13385210	2.33E-06	0.001240187	FDR0.01	17	38599271	'IGFBP4	insulin-like growth factor-binding protein 4 precursor	TSS1500	-430	'IGFBP4	insulin-like growth factor-binding protein 4 precursor
GA	cg13415831	1.62E-05	0.004858132	FDR0.01	9	92137791	'			-24763	'SEMA4D	semaphorin-4D isoform 2 precursor
GA	cg13422817	4.84E-08	6.83E-05	BF|FDR0.01	12	4550927	'FGF6	fibroblast growth factor 6	Body	3853	'FGF6	fibroblast growth factor 6
GA	cg13423759	3.46E-09	9.99E-06	BF|FDR0.01	1	37937403	'LINC01137;ZC3H12A	long intergenic non-protein coding RNA 1137;ribonuclease ZC3H12A	Body;TSS5000;TSS5000;TSS500	2641	'LINC01137	long intergenic non-protein coding RNA 1137
GA	cg13427834	1.77E-07	0.000184919	FDR0.01	2	106362217	'NCK2	cytoplasmic protein NCK2	Body;Body	698	'NCK2	cytoplasmic protein NCK2
GA	cg13428066	3.37E-05	0.00779633	FDR0.01	11	2677768	'KCNQ1;KCNQ1OT1	potassium voltage-gated channel subfamily KQT member 1;KCNQ1 opposite	Body;Body;Body	43460	'KCNQ1OT1	KCNQ1 opposite strand/antisense transcript 1
GA	cg13430225	1.41E-05	0.004406411	FDR0.01	1	53662423	'CPT2	carnitine O-palmitoyltransferase 2, mitochondrial precursor	TSS200;TSS200	-72	'CPT2	carnitine O-palmitoyltransferase 2, mitochondrial precursor
GA	cg13453520	3.92E-05	0.008640599	FDR0.01	4	48485306	'SLC10A4	sodium/bile acid cotransporter 4	TSS200	-53	'SLC10A4	sodium/bile acid cotransporter 4
GA	cg13460643	1.06E-05	0.00355761	FDR0.01	1	209848845	'HSD11B1-AS1;G0S2	HSD11B1 antisense RNA 1;G0/G1 switch protein 2	TSS1500;Body;Body;5'UTR	89	'G0S2	G0/G1 switch protein 2
GA	cg13487983	1.16E-06	0.000748451	FDR0.01	9	137296319	'RXRA	retinoic acid receptor RXR-alpha	Body;Body;TSS5000	-2086	'RXRA	retinoic acid receptor RXR-alpha
GA	cg13495532	2.34E-06	0.001240922	FDR0.01	6	44214738	'HSP90AB1	heat shock protein HSP 90-beta	Body;5'UTR;5'UTR;5'UTR;TSS20	44	'HSP90AB1	heat shock protein HSP 90-beta
GA	cg13503476	1.63E-06	0.000960311	FDR0.01	1	113599742	'LRIG2-DT	LRIG2 divergent transcript	Body	15982	'LRIG2-DT	LRIG2 divergent transcript
GA	cg13503564	1.61E-07	0.000171528	FDR0.01	2	45539374	'			-57294	'LINC01121	long intergenic non-protein coding RNA 1121
GA	cg13511752	1.59E-06	0.000944848	FDR0.01	10	74094697	'DNAJB12	dnaJ homolog subfamily B member 12	3'UTR;Body;Body;Body;Body	19980	'DNAJB12	dnaJ homolog subfamily B member 12
GA	cg13519902	1.33E-12	3.72E-08	BF|FDR0.01	3	50639079	'CISH	cytokine-inducible SH2-containing protein	TES5000;TES5000	10126	'CISH	cytokine-inducible SH2-containing protein
GA	cg13522406	4.25E-05	0.009044808	FDR0.01	6	30581820	'PPP1R10;MRPS18B	serine/threonine-protein phosphatase 1 regulatory subunit 10;28S ribosoma	Body;Body;TSS5000	3200	'PPP1R10	serine/threonine-protein phosphatase 1 regulatory subunit 10
GA	cg13541527	2.15E-05	0.005862662	FDR0.01	6	31804078	'SNHG32;SNORD48;SNORD52	small nucleolar RNA host gene 32;small nucleolar RNA, C/D box 48;small nuc	Body;Body;Body;Body;Body;Bo	-774	'SNORD52	small nucleolar RNA, C/D box 52
GA	cg13543355	6.05E-06	0.002428678	FDR0.01	14	77350013	'			57263	'LRRC74A	leucine-rich repeat-containing protein 74A isoform 1
GA	cg13547237	3.74E-06	0.001722396	FDR0.01	11	65687877	'C11orf68;DRAP1	UPF0696 protein C11orf68 isoform 2;dr1-associated corepressor	TSS1500;TSS1500;Body	981	'DRAP1	dr1-associated corepressor
GA	cg13593323	1.94E-08	3.33E-05	BF|FDR0.01	9	132647349	'USP20;FNBP1	ubiquitin carboxyl-terminal hydrolase 20;formin-binding protein 1	TES5000;TES5000;TES5000;TES	15466	'MIR6855	microRNA 6855
GA	cg13607248	3.97E-08	5.86E-05	BF|FDR0.01	3	183874556	'DVL3	segment polarity protein dishevelled homolog DVL-3	Body	1392	'DVL3	segment polarity protein dishevelled homolog DVL-3
GA	cg13641317	1.36E-11	1.97E-07	BF|FDR0.01	3	127255552	'LINC01471	long intergenic non-protein coding RNA 1471	Body	1071	'LINC01471	long intergenic non-protein coding RNA 1471
GA	cg13675015	7.87E-06	0.002911985	FDR0.01	6	43217925	'TTBK1	tau-tubulin kinase 1	Body	6707	'TTBK1	tau-tubulin kinase 1
GA	cg13687834	3.16E-05	0.00745702	FDR0.01	10	3514783	'LOC105376360;LINC02669	uncharacterized LOC105376360;long intergenic non-protein coding RNA 266	Body;Body	30247	'LINC02669	long intergenic non-protein coding RNA 2669
GA	cg13700939	1.38E-06	0.000851679	FDR0.01	2	128166166	'			-9829	'PROC	vitamin K-dependent protein C precursor
GA	cg13708218	3.36E-05	0.007771133	FDR0.01	11	65601267	'SNX32	sorting nexin-6	TSS200	-166	'SNX32	sorting nexin-6
GA	cg13724780	4.70E-05	0.009635413	FDR0.01	5	180237085	'MGAT1	alpha-1,3-mannosyl-glycoprotein 2-beta-N-acetylglucosaminyltransferase	TSS1500;TSS1500;TSS1500;TSS1	52	'MGAT1	alpha-1,3-mannosyl-glycoprotein 2-beta-N-acetylglucosaminyltransferase
GA	cg13733403	2.60E-07	0.000247246	FDR0.01	19	6528291	'TNFSF9	tumor necrosis factor ligand superfamily member 9	TSS5000	-2745	'TNFSF9	tumor necrosis factor ligand superfamily member 9
GA	cg13765061	2.40E-05	0.006269019	FDR0.01	9	78830510	'PCSK5	proprotein convertase subtilisin/kexin type 5 isoform PC6B preproprotein	Body;Body	178832	'RFK	riboflavin kinase
GA	cg13781956	2.36E-05	0.006209959	FDR0.01	9	117826025	'TNC	tenascin precursor	Body	54411	'TNC	tenascin precursor
GA	cg13784312	4.10E-06	0.001845388	FDR0.01	9	134609065	'RAPGEF1	rap guanine nucleotide exchange factor 1	Body;Body	3860	'RAPGEF1	rap guanine nucleotide exchange factor 1
GA	cg13790353	7.70E-06	0.00287045	FDR0.01	11	85565559	'CCDC83	coiled-coil domain-containing protein 83	TSS1500;TSS1500	-584	'CCDC83	coiled-coil domain-containing protein 83
GA	cg13805537	6.76E-08	8.86E-05	BF|FDR0.01	9	130728012	'FAM102A	protein FAM102A	Body	-15019	'FAM102A	protein FAM102A
GA	cg13813391	2.99E-05	0.007227759	FDR0.01	16	66613407	'CKLF-CMTM1;CMTM1;CMTM2	CKLF-CMTM1 protein isoform 3;CKLF-like MARVEL transmembrane domain-c	TES5000;TES5000;TES5000;TES	42	'CMTM2	CKLF-like MARVEL transmembrane domain-containing protein 2
GA	cg13836518	5.76E-06	0.002342691	FDR0.01	9	100883593	'TRIM14;CORO2A	tripartite motif-containing protein 14;coronin-2A	TSS5000;TSS5000;3'UTR;3'UTR	-2089	'TRIM14	tripartite motif-containing protein 14
GA	cg13840445	8.59E-07	0.000598841	FDR0.01	6	53530628	'KLHL31	kelch-like protein 31	TSS200	-74	'KLHL31	kelch-like protein 31
GA	cg13845961	5.05E-07	0.000402428	FDR0.01	2	3106295	'LINC01250	long intergenic non-protein coding RNA 1250	Body	23503	'LINC01250	long intergenic non-protein coding RNA 1250
GA	cg13847322	7.20E-07	0.000527564	FDR0.01	9	137272766	'RXRA;MIR4669	retinoic acid receptor RXR-alpha;microRNA 4669	Body;Body;TES5000	1510	'MIR4669	microRNA 4669
GA	cg13851767	9.09E-09	2.01E-05	BF|FDR0.01	9	123656764	'PHF19	PHD finger protein 19	Body	410	'PHF19	PHD finger protein 19
GA	cg13856340	2.61E-05	0.006652483	FDR0.01	9	127888818	'SCAI	protein SCAI	Body;Body	16989	'SCAI	protein SCAI
GA	cg13889620	1.40E-05	0.004373959	FDR0.01	12	122092201	'MORN3	MORN repeat-containing protein 3	Body;Body	15359	'MORN3	MORN repeat-containing protein 3
GA	cg13896106	4.62E-07	0.000382145	FDR0.01	17	72754589	'SLC9A3R1	Na(+)/H(+) exchange regulatory cofactor NHE-RF1	Body	9823	'SLC9A3R1	Na(+)/H(+) exchange regulatory cofactor NHE-RF1
GA	cg13904214	4.21E-05	0.009006321	FDR0.01	7	155556729	'RBM33	RNA-binding protein 33	Body	45128	'SHH	sonic hedgehog protein precursor
GA	cg13922488	8.45E-06	0.003053789	FDR0.01	19	14545201	'PKN1	serine/threonine-protein kinase N1 isoform 1	Body	1084	'PKN1	serine/threonine-protein kinase N1 isoform 1
GA	cg13927769	2.33E-09	7.74E-06	BF|FDR0.01	9	101740711	'COL15A1	collagen alpha-1(XV) chain precursor	Body	34523	'COL15A1	collagen alpha-1(XV) chain precursor
GA	cg13928411	3.55E-05	0.008118444	FDR0.01	16	22103791	'VWA3A	von Willebrand factor A domain-containing protein 3A precursor	TSS200	-70	'VWA3A	von Willebrand factor A domain-containing protein 3A precursor
GA	cg13932501	4.75E-05	0.009717544	FDR0.01	9	94060853	'AUH	methylglutaconyl-CoA hydratase, mitochondrial	Body;Body;Body;Body;Body	63353	'AUH	methylglutaconyl-CoA hydratase, mitochondrial
GA	cg13934406	2.45E-07	0.000235758	FDR0.01	6	32120878	'PRRT1;LOC100507547;PPT2;PPT2-EGFL8	proline-rich transmembrane protein 1;uncharacterized LOC100507547;lysos	TSS200;TSS1500;Body;Body;Bo	-18	'PRRT1	proline-rich transmembrane protein 1
GA	cg13985198	1.04E-07	0.000127068	BF|FDR0.01	1	45242073	'RPS8;SNORD55;SNORD46;SNORD38A;SNORD38B	40S ribosomal protein S8;small nucleolar RNA, C/D box 55;small nucleolar RN	Body;TES5000;TSS200;TSS1500	-90	'SNORD46	small nucleolar RNA, C/D box 46
GA	cg13991834	2.31E-05	0.006126008	FDR0.01	1	114430280	'AP4B1-AS1;BCL2L15	AP4B1 antisense RNA 1;bcl-2-like protein 15	Body;Body;TSS200	-77	'BCL2L15	bcl-2-like protein 15
GA	cg14006677	2.22E-05	0.005969076	FDR0.01	11	111808394	'DIXDC1	dixin isoform b	Body;Body	328	'DIXDC1	dixin isoform b
GA	cg14010720	4.78E-06	0.00203829	FDR0.01	20	62168878	'PTK6;SRMS	protein-tyrosine kinase 6;tyrosine-protein kinase Srms	TSS200;TSS200;TES5000	-149	'PTK6	protein-tyrosine kinase 6
GA	cg14018648	1.57E-08	2.95E-05	BF|FDR0.01	17	7083015	'ASGR1	asialoglycoprotein receptor 1	TSS1500;TSS200	-132	'ASGR1	asialoglycoprotein receptor 1

Trait	#cpg	p	fdr	significance	chr	pos.hg19	gene.Illumina	gene.description	locations	dist2TSS	gene.nearest	gene.description
GA	cg14019193	1.87E-05	0.005356826	FDR0.01	11	66278004	'DPP3;BBS1	dipeptidyl peptidase 3;Bardet-Biedl syndrome 1 protein	TES5000;TES5000;TES5000;TSS	-101	'BBS1	Bardet-Biedl syndrome 1 protein
GA	cg14033237	2.96E-05	0.007183669	FDR0.01	3	133506162	'TF;SRPRB	serotransferrin precursor;signal recognition particle receptor subunit beta	3'UTR;3'UTR;3'UTR;Body	3296	'SRPRB	signal recognition particle receptor subunit beta
GA	cg14037346	1.62E-06	0.000958058	FDR0.01	9	131262765	'ODF2;GLE1	outer dense fiber protein 2 precursor;nucleoporin GLE1	3'UTR;3'UTR;3'UTR;3'UTR;3'UT	-4205	'GLE1	nucleoporin GLE1
GA	cg14037769	7.60E-06	0.002844935	FDR0.01	1	9895799	'			-11215	'CLSTN1	calsyntenin-1 precursor
GA	cg14039237	5.12E-07	0.000406171	FDR0.01	9	130833354	'NAIF1;SLC25A25	nuclear apoptosis-inducing factor 1;calcium-binding mitochondrial carrier pr	TSS5000;Body;Body	2876	'SLC25A25	calcium-binding mitochondrial carrier protein SCaMC-2
GA	cg14040358	1.97E-06	0.001087011	FDR0.01	19	4369393	'SH3GL1	endophilin-A2 isoform 2	Body;Body;Body	25870	'MPND	MPN domain-containing protein isoform 2
GA	cg14040796	3.20E-05	0.007501015	FDR0.01	19	49403969	'TULP2;NUCB1	tubby-related protein 2;nucleobindin-1 precursor	TSS5000;Body	401	'NUCB1	nucleobindin-1 precursor
GA	cg14064762	9.02E-06	0.003193007	FDR0.01	9	123688745	'TRAF1	TNF receptor-associated factor 1	Body;Body	329	'TRAF1	TNF receptor-associated factor 1
GA	cg14083015	8.89E-06	0.003159538	FDR0.01	6	25042090	'RIPOR2	rho family-interacting cell polarization regulator 2	1stExon	426	'RIPOR2	rho family-interacting cell polarization regulator 2
GA	cg14093103	1.80E-07	0.000187704	FDR0.01	1	61546214	'NFIA	nuclear factor 1 A-type	Body;TSS1500;TSS5000;TSS500	-1196	'NFIA	nuclear factor 1 A-type
GA	cg14093474	3.51E-05	0.008049824	FDR0.01	1	14052489	'PRDM2	PR domain zinc finger protein 2 isoform c	Body;Body;Body	21140	'PRDM2	PR domain zinc finger protein 2 isoform c
GA	cg14093886	3.87E-05	0.008593877	FDR0.01	7	41745179	'INHBA;INHBA-AS1	inhibin beta A chain precursor;INHBA antisense RNA 1	TSS5000;Body;Body	-2491	'INHBA	inhibin beta A chain precursor
GA	cg14101485	1.97E-07	0.000198616	FDR0.01	19	3369759	'NFIC	nuclear factor 1 C-type	Body;Body;Body;Body;Body	3179	'NFIC	nuclear factor 1 C-type
GA	cg14116908	1.44E-06	0.000878693	FDR0.01	9	95859189	'CARD19	caspase recruitment domain-containing protein 19	Body;Body;Body;Body;Body;Bo	691	'CARD19	caspase recruitment domain-containing protein 19
GA	cg14128068	4.05E-06	0.001830165	FDR0.01	9	96590527	'			8889	'MIR4291	microRNA 4291
GA	cg14134128	7.87E-07	0.000563967	FDR0.01	17	76774832	'CYTH1	cytohesin-1	Body;Body;Body;Body;Body	3458	'CYTH1	cytohesin-1
GA	cg14154859	9.64E-12	1.54E-07	BF|FDR0.01	1	220702826	'MARK1	serine/threonine-protein kinase MARK1 isoform 4	Body;Body;Body;Body;Body	1302	'MARK1	serine/threonine-protein kinase MARK1 isoform 4
GA	cg14155196	3.29E-06	0.001563517	FDR0.01	8	22725904	'PEBP4	phosphatidylethanolamine-binding protein 4 precursor	Body;Body	-9580	'LOC101929237	uncharacterized LOC101929237
GA	cg14171369	2.31E-05	0.006126008	FDR0.01	14	75447688	'			-21935	'EIF2B2	translation initiation factor eIF-2B subunit beta
GA	cg14179389	1.54E-09	5.74E-06	BF|FDR0.01	1	92947961	'GFI1	zinc finger protein Gfi-1	Body;Body;Body	1550	'GFI1	zinc finger protein Gfi-1
GA	cg14186336	3.56E-05	0.008120945	FDR0.01	9	124045139	'GSN;GSN-AS1	gelsolin;GSN antisense RNA 1	Body;Body;Body;Body;Body;Bo	2669	'GSN-AS1	GSN antisense RNA 1
GA	cg14188508	7.34E-06	0.002772129	FDR0.01	19	11685647	'ACP5	tartrate-resistant acid phosphatase type 5 precursor	3'UTR;3'UTR;3'UTR;3'UTR;3'UT	2813	'ACP5	tartrate-resistant acid phosphatase type 5 precursor
GA	cg14191466	2.07E-07	0.000207065	FDR0.01	14	103440432	'CDC42BPB	serine/threonine-protein kinase MRCK beta	Body	51440	'AMN	protein amnionless precursor
GA	cg14198101	4.29E-05	0.009087532	FDR0.01	19	1270276	'CIRBP-AS1;CIRBP	CIRBP antisense RNA 1;cold-inducible RNA-binding protein	TSS200;Body;Body;Body;Body;	-17	'CIRBP-AS1	CIRBP antisense RNA 1
GA	cg14199150	7.04E-10	3.12E-06	BF|FDR0.01	9	136930919	'BRD3	bromodomain-containing protein 3	Body	2229	'BRD3	bromodomain-containing protein 3
GA	cg14206172	5.53E-06	0.002278683	FDR0.01	19	39466757	'FBXO17	F-box only protein 17	TSS1500;TSS1500	-375	'FBXO17	F-box only protein 17
GA	cg14207833	2.06E-05	0.005712275	FDR0.01	19	18945153	'UPF1	regulator of nonsense transcripts 1 isoform 1	Body;Body	2386	'UPF1	regulator of nonsense transcripts 1 isoform 1
GA	cg14213424	2.17E-05	0.005892136	FDR0.01	17	18380159	'LGALS9C	galectin-9C	5'UTR	57	'LGALS9C	galectin-9C
GA	cg14217861	1.82E-06	0.001035833	FDR0.01	9	139426233	'NOTCH1	neurogenic locus notch homolog protein 1	Body	-12155	'MIR4673	microRNA 4673
GA	cg14225168	3.14E-05	0.007436052	FDR0.01	9	139403827	'NOTCH1	neurogenic locus notch homolog protein 1	Body	10251	'MIR4673	microRNA 4673
GA	cg14254562	3.20E-05	0.007503175	FDR0.01	9	84213071	'TLE1	transducin-like enhancer protein 1	Body;Body;Body	91379	'TLE1	transducin-like enhancer protein 1
GA	cg14260530	8.39E-06	0.003044618	FDR0.01	9	117157871	'AKNA	microtubule organization protein AKNA isoform 2	TSS1500;TSS1500	-1186	'AKNA	microtubule organization protein AKNA isoform 2
GA	cg14266527	9.20E-11	7.91E-07	BF|FDR0.01	4	151501298	'LRBA;LOC729558;MAB21L2	lipopolysaccharide-responsive and beige-like anchor protein isoform 4;uncha	Body;Body;Body;Body;Body;TS	1058	'LOC729558	uncharacterized LOC729558
GA	cg14269109	1.34E-06	0.000828715	FDR0.01	17	78513167	'			-5470	'RPTOR	regulatory-associated protein of mTOR
GA	cg14271690	2.08E-05	0.005746234	FDR0.01	20	37209999	'RALGAPB;ADIG	ral GTPase-activating protein subunit beta;adipogenin	TES5000;TES5000;TES5000;1st	160	'ADIG	adipogenin
GA	cg14276125	6.37E-07	0.000481893	FDR0.01	11	67052759	'GRK2;ANKRD13D	beta-adrenergic receptor kinase 1;ankyrin repeat domain-containing protein	Body;TSS5000;TSS5000	-4014	'ANKRD13D	ankyrin repeat domain-containing protein 13D
GA	cg14276557	6.26E-07	0.000477413	FDR0.01	9	139538535	'HSPC324	uncharacterized LOC101928612	TES5000	-14772	'EGFL7	epidermal growth factor-like protein 7 precursor
GA	cg14289429	2.42E-05	0.006316368	FDR0.01	9	134139878	'FAM78A	protein FAM78A	Body	12019	'FAM78A	protein FAM78A
GA	cg14292368	5.00E-07	0.00039958	FDR0.01	19	18793705	'CRTC1	CREB-regulated transcription coactivator 1	TSS1500;TSS1500	-784	'CRTC1	CREB-regulated transcription coactivator 1
GA	cg14310606	8.69E-06	0.003116837	FDR0.01	9	139693852	'CCDC183;CCDC183-AS1	coiled-coil domain-containing protein 183;CCDC183 antisense RNA 1	Body;TES5000	3063	'CCDC183	coiled-coil domain-containing protein 183
GA	cg14316944	9.96E-06	0.003417326	FDR0.01	17	79485529	'			-5704	'ACTG1	actin, cytoplasmic 2
GA	cg14319451	5.75E-07	0.000446034	FDR0.01	11	63685777	'RCOR2	REST corepressor 2	TSS1500;TSS1500	-1141	'RCOR2	REST corepressor 2
GA	cg14348532	9.55E-06	0.003319253	FDR0.01	11	14993562	'CALCA	calcitonin isoform 1 preproprotein	Body;Body;Body;Body	270	'CALCA	calcitonin isoform 1 preproprotein
GA	cg14348967	1.51E-05	0.004632675	FDR0.01	4	10160060	'			-41637	'WDR1	WD repeat-containing protein 1
GA	cg14379939	4.43E-07	0.000368854	FDR0.01	9	100721257	'			-14123	'HEMGN	hemogen
GA	cg14384748	1.54E-05	0.004701738	FDR0.01	12	132859951	'GALNT9;LOC100130238	polypeptide N-acetylgalactosaminyltransferase 9;uncharacterized LOC10013	Body;TES5000	7975	'LOC100130238	uncharacterized LOC100130238
GA	cg14387626	9.24E-06	0.003246098	FDR0.01	14	106331803	'			-6081	'MIR4537	microRNA 4537
GA	cg14410964	2.38E-05	0.006240501	FDR0.01	9	139427106	'NOTCH1	neurogenic locus notch homolog protein 1	Body	-13028	'MIR4673	microRNA 4673
GA	cg14420108	6.53E-06	0.002572219	FDR0.01	9	34647483	'GALT;IL11RA	galactose-1-phosphate uridylyltransferase;interleukin-11 receptor subunit al	Body;Body;TSS5000	812	'GALT	galactose-1-phosphate uridylyltransferase
GA	cg14434062	1.80E-09	6.48E-06	BF|FDR0.01	15	70767419	'			30848	'LINC02205	long intergenic non-protein coding RNA 2205
GA	cg14439572	2.49E-05	0.006441228	FDR0.01	8	102121398	'			57117	'FLJ42969	uncharacterized LOC441374
GA	cg14479136	1.48E-10	1.05E-06	BF|FDR0.01	9	139222701	'DKFZP434A062;GPSM1	uncharacterized LOC26102;G-protein-signaling modulator 1 isoform c	TSS1500;Body;Body	707	'GPSM1	G-protein-signaling modulator 1 isoform c
GA	cg14489570	2.57E-05	0.006582709	FDR0.01	8	144099280	'LY6E-DT;LY6E	LY6E divergent transcript;lymphocyte antigen 6E precursor	Body;TSS1500;TSS1500	527	'LY6E-DT	LY6E divergent transcript
GA	cg14499274	1.14E-05	0.003782764	FDR0.01	9	136705398	'VAV2	guanine nucleotide exchange factor VAV2	Body;Body	-100321	'SARDH	sarcosine dehydrogenase, mitochondrial
GA	cg14517355	3.40E-06	0.001601446	FDR0.01	3	9996954	'PRRT3;PRRT3-AS1	proline-rich transmembrane protein 3 isoform 1 precursor;PRRT3 antisense	TSS5000;TSS5000;TES5000;TSS	-2855	'PRRT3	proline-rich transmembrane protein 3 isoform 1 precursor
GA	cg14519350	4.84E-06	0.002060624	FDR0.01	22	30661949	'OSM	oncostatin-M precursor	5'UTR;Body	280	'OSM	oncostatin-M precursor
GA	cg14521421	1.89E-05	0.005391412	FDR0.01	11	8862019	'DENND2B	DENN domain-containing protein 2B	Body	-29137	'DENND2B	DENN domain-containing protein 2B
GA	cg14535245	7.78E-06	0.002887507	FDR0.01	2	47283713	'TTC7A	tetratricopeptide repeat protein 7A	Body;Body;Body;Body	98734	'STPG4	protein STPG4
GA	cg14539363	5.12E-09	1.38E-05	BF|FDR0.01	19	55766853	'PPP6R1	serine/threonine-protein phosphatase 6 regulatory subunit 1	Body	3532	'PPP6R1	serine/threonine-protein phosphatase 6 regulatory subunit 1
GA	cg14539422	2.57E-06	0.00130441	FDR0.01	9	116343185	'RGS3	regulator of G-protein signaling 3	Body;Body;Body;Body;Body;Bo	-22	'RGS3	regulator of G-protein signaling 3
GA	cg14553895	7.25E-06	0.002754318	FDR0.01	3	178899170	'PIK3CA	phosphatidylinositol 4,5-bisphosphate 3-kinase catalytic subunit alpha isofor	Body	33026	'PIK3CA	phosphatidylinositol 4,5-bisphosphate 3-kinase catalytic subunit alpha isoform
GA	cg14556243	3.43E-06	0.0016095	FDR0.01	9	128107984	'GAPVD1	GTPase-activating protein and VPS9 domain-containing protein 1	Body;Body;Body;Body;Body;Bo	83880	'GAPVD1	GTPase-activating protein and VPS9 domain-containing protein 1
GA	cg14578866	2.06E-05	0.005706124	FDR0.01	2	1680784	'PXDN	peroxidasin homolog precursor	Body	67503	'PXDN	peroxidasin homolog precursor
GA	cg14579997	2.70E-05	0.006772905	FDR0.01	6	89792529	'PNRC1	proline-rich nuclear receptor coactivator 1	Body	2060	'PNRC1	proline-rich nuclear receptor coactivator 1
GA	cg14588779	3.26E-05	0.007618947	FDR0.01	19	15528530	'AKAP8L;WIZ	A-kinase anchor protein 8-like;protein Wiz isoform 2	Body;Body;Body;TES5000;TES5	1269	'AKAP8L	A-kinase anchor protein 8-like
GA	cg14607011	3.16E-05	0.00745702	FDR0.01	10	135171746	'FUOM;ECHS1	fucose mutarotase;enoyl-CoA hydratase, mitochondrial	TSS1500;TSS1500;TSS1500;TSS	-223	'FUOM	fucose mutarotase
GA	cg14613228	4.38E-05	0.009224399	FDR0.01	1	78621066	'			-74216	'MGC27382	uncharacterized MGC27382
GA	cg14624731	5.67E-06	0.002325048	FDR0.01	2	121436004	'			-57439	'GLI2	zinc finger protein GLI2
GA	cg14662750	1.74E-05	0.005123307	FDR0.01	6	15280024	'JARID2	protein Jumonji	Body;Body	30939	'JARID2	protein Jumonji
GA	cg14666404	5.73E-06	0.002336898	FDR0.01	3	183016948	'MCF2L2	probable guanine nucleotide exchange factor MCF2L2	Body	45908	'B3GNT5	lactosylceramide 1,3-N-acetyl-beta-D-glucosaminyltransferase
GA	cg14689532	3.98E-05	0.008711177	FDR0.01	11	820572	'PNPLA2	patatin-like phospholipase domain-containing protein 2	Body	1659	'PNPLA2	patatin-like phospholipase domain-containing protein 2
GA	cg14707834	2.44E-09	7.97E-06	BF|FDR0.01	11	415457	'SIGIRR;ANO9	single Ig IL-1-related receptor;anoctamin-9 isoform 2	TSS1500;Body;Body;TES5000;T	-458	'SIGIRR	single Ig IL-1-related receptor
GA	cg14725580	5.88E-10	2.82E-06	BF|FDR0.01	6	34663155	'ILRUN;LOC101929243	protein ILRUN isoform a;	Body;Body;TSS1500;TSS5000	-775	'LOC101929243	
GA	cg14728380	4.47E-08	6.43E-05	BF|FDR0.01	17	80280330	'CD7;SECTM1	T-cell antigen CD7 precursor;secreted and transmembrane 1 precursor	TSS5000;Body	-4846	'CD7	T-cell antigen CD7 precursor
GA	cg14761252	6.03E-07	0.000462932	FDR0.01	18	71815123	'FBXO15;TIMM21	F-box only protein 15;mitochondrial import inner membrane translocase sub	TSS200;TSS200;TSS1500	-54	'FBXO15	F-box only protein 15
GA	cg14765414	8.40E-08	0.000106023	BF|FDR0.01	13	113652335	'MCF2L	guanine nucleotide exchange factor DBS	Body;Body;Body;TSS5000;TSS5	-3692	'MCF2L	guanine nucleotide exchange factor DBS
GA	cg14778622	1.42E-07	0.000156287	FDR0.01	2	136743698	'DARS1;DARS-AS1	aspartate--tRNA ligase, cytoplasmic;	TSS1500;TSS1500;Body;Body	-444	'DARS1	aspartate--tRNA ligase, cytoplasmic
GA	cg14784664	2.71E-05	0.006793468	FDR0.01	11	123302579	'GRAMD1B	protein Aster-B	Body;Body;Body;Body;Body	1603	'GRAMD1B	protein Aster-B
GA	cg14789828	1.55E-05	0.004720445	FDR0.01	11	62201168	'AHNAK	neuroblast differentiation-associated protein AHNAK isoform 1	3'UTR	14664	'SCGB1A1	uteroglobin precursor
GA	cg14844236	1.40E-06	0.000860807	FDR0.01	12	123753212	'CDK2AP1	cyclin-dependent kinase 2-associated protein 1	TSS1500;Body;Body;Body;Body	-411	'CDK2AP1	cyclin-dependent kinase 2-associated protein 1
GA	cg14847009	7.26E-06	0.002754318	FDR0.01	1	175162515	'KIAA0040	uncharacterized protein KIAA0040 homolog	TSS1500;TSS1500;TSS1500;TSS	-392	'KIAA0040	uncharacterized protein KIAA0040 homolog
GA	cg14857493	3.99E-06	0.00181266	FDR0.01	5	107135281	'			-128643	'EFNA5	ephrin-A5 precursor
GA	cg14864103	1.27E-05	0.004085192	FDR0.01	13	113622738	'MCF2L-AS1;MCF2L	MCF2L antisense RNA 1;guanine nucleotide exchange factor DBS	Body;TSS1500;TSS1500	214	'MCF2L-AS1	MCF2L antisense RNA 1
GA	cg14923289	6.80E-08	8.89E-05	BF|FDR0.01	6	52535675	'LOC730101;TMEM14A	uncharacterized LOC730101;transmembrane protein 14A	TES5000;TES5000;TSS1500	-235	'TMEM14A	transmembrane protein 14A
GA	cg14983728	2.85E-07	0.000264656	FDR0.01	22	35695234	'HMGXB4;TOM1	HMG domain-containing protein 4;target of Myb protein 1	TES5000;TES5000;TES5000;TSS	-33	'TOM1	target of Myb protein 1
GA	cg14989316	6.29E-06	0.002505967	FDR0.01	10	80757927	'ZMIZ1-AS1	ZMIZ1 antisense RNA 1	Body;Body;Body	69278	'ZMIZ1-AS1	ZMIZ1 antisense RNA 1
GA	cg14994180	2.71E-05	0.006793468	FDR0.01	11	85376470	'CREBZF	CREB/ATF bZIP transcription factor	TSS1500;TSS1500;TSS1500;TSS	-297	'CREBZF	CREB/ATF bZIP transcription factor
GA	cg15009198	4.08E-05	0.008825587	FDR0.01	2	97429502	'CNNM4	metal transporter CNNM4 precursor	Body	2864	'CNNM4	metal transporter CNNM4 precursor
GA	cg15028899	6.94E-06	0.002665421	FDR0.01	11	120275443	'ARHGEF12	rho guanine nucleotide exchange factor 12	Body;Body;Body	19408	'ARHGEF12	rho guanine nucleotide exchange factor 12
GA	cg15035133	8.20E-09	1.88E-05	BF|FDR0.01	22	37962637	'CDC42EP1;LGALS2	cdc42 effector protein 1;galectin-2	1stExon;TES5000	6151	'CDC42EP1	cdc42 effector protein 1

Trait	#cpg	p	fdr	significance	chr	pos.hg19	gene.Illumina	gene.description	locations	dist2TSS	gene.nearest	gene.description
GA	cg15066504	4.67E-05	0.009614676	FDR0.01	6	75884800	'COL12A1	collagen alpha-1(XII) chain precursor	Body;Body	30969	'COL12A1	collagen alpha-1(XII) chain precursor
GA	cg15078061	1.13E-06	0.0007356	FDR0.01	6	32117558	'PRRT1;LOC100507547;PPT2;PPT2-EGFL8	proline-rich transmembrane protein 1;uncharacterized LOC100507547;lysos	Body;Body;TES5000;TES5000;T	2158	'PRRT1	proline-rich transmembrane protein 1
GA	cg15090036	1.48E-05	0.004545404	FDR0.01	17	48706075	'CACNA1G	voltage-dependent T-type calcium channel subunit alpha-1G	TES5000;TES5000;TES5000;TES	-6166	'ABCC3	canalicular multispecific organic anion transporter 2
GA	cg15110079	1.02E-05	0.003487773	FDR0.01	7	7974904	'			33479	'GLCCI1-DT	GLCCI1 divergent transcript
GA	cg15142477	4.38E-07	0.000367665	FDR0.01	18	43915662	'RNF165	E3 ubiquitin-protein ligase RNF165 isoform 2	Body;Body;Body	1476	'RNF165	E3 ubiquitin-protein ligase RNF165 isoform 2
GA	cg15143809	1.31E-05	0.004179205	FDR0.01	11	120208324	'TLCD5;ARHGEF12	TLC domain-containing protein 5 isoform e;rho guanine nucleotide exchange	TES5000;TES5000;TES5000;TES	1203	'ARHGEF12	rho guanine nucleotide exchange factor 12
GA	cg15153887	3.41E-07	0.000305698	FDR0.01	6	41123110	'TREML1;TREM2	trem-like transcript 1 protein precursor;triggering receptor expressed on my	TSS1500;TSS1500;TSS1500;TES	-1023	'TREML1	trem-like transcript 1 protein precursor
GA	cg15157360	7.09E-06	0.002709346	FDR0.01	8	26241565	'SDAD1P1;BNIP3L	SDA1 domain containing 1 pseudogene 1;BCL2/adenovirus E1B 19 kDa prote	TSS1500;Body	996	'BNIP3L	BCL2/adenovirus E1B 19 kDa protein-interacting protein 3-like
GA	cg15164472	4.00E-05	0.008741223	FDR0.01	3	195914113	'			24158	'ZDHHC19	palmitoyltransferase ZDHHC19
GA	cg15165154	7.49E-09	1.81E-05	BF|FDR0.01	5	139041014	'CXXC5	CXXC-type zinc finger protein 5	Body;Body;Body;Body;Body;Bo	1875	'CXXC5	CXXC-type zinc finger protein 5
GA	cg15175129	3.37E-07	0.000303571	FDR0.01	12	65672052	'MSRB3	methionine-R-sulfoxide reductase B3	TSS1500;TSS1500;TSS1500;TSS	-410	'MSRB3	methionine-R-sulfoxide reductase B3
GA	cg15176413	4.68E-05	0.009616031	FDR0.01	7	4764443	'FOXK1	forkhead box protein K1	Body	42518	'FOXK1	forkhead box protein K1
GA	cg15192103	5.12E-06	0.00214773	FDR0.01	4	4763372	'LOC101928279	uncharacterized LOC101928279	TSS200	-114	'LOC101928279	uncharacterized LOC101928279
GA	cg15197458	3.89E-07	0.000336935	FDR0.01	19	3464765	'NFIC	nuclear factor 1 C-type	3'UTR;3'UTR;3'UTR;3'UTR;3'UT	15758	'SMIM24	small integral membrane protein 24 precursor
GA	cg15202848	4.02E-05	0.008758136	FDR0.01	11	64618819	'EHD1	EH domain-containing protein 1	TES5000;TES5000;TES5000	-6694	'CDC42BPG	serine/threonine-protein kinase MRCK gamma
GA	cg15204354	3.07E-06	0.001482713	FDR0.01	1	155006911	'DCST2;DCST1	DC-STAMP domain-containing protein 2;E3 ubiquitin-protein ligase DCST1	TSS1500;Body;Body	630	'DCST1	E3 ubiquitin-protein ligase DCST1
GA	cg15233611	1.03E-05	0.003504409	FDR0.01	12	122244660	'LINC01089;SETD1B	long intergenic non-protein coding RNA 1089;histone-lysine N-methyltransfe	TSS5000;TSS5000;TSS5000;TSS	2031	'SETD1B	histone-lysine N-methyltransferase SETD1B
GA	cg15247509	4.23E-05	0.009024042	FDR0.01	10	48416977	'GDF2	growth/differentiation factor 2 precursor	TSS200	-69	'GDF2	growth/differentiation factor 2 precursor
GA	cg15298486	2.54E-05	0.006528281	FDR0.01	10	102046690	'BLOC1S2;PKD2L1	biogenesis of lysosome-related organelles complex 1 subunit 2;polycystic kid	TSS1500;TSS1500;TSS1500;TSS	-221	'BLOC1S2	biogenesis of lysosome-related organelles complex 1 subunit 2
GA	cg15299233	9.77E-06	0.003369931	FDR0.01	15	75162173	'SCAMP2	secretory carrier-associated membrane protein 2	Body;Body	3533	'SCAMP2	secretory carrier-associated membrane protein 2
GA	cg15349304	2.31E-06	0.001232955	FDR0.01	14	106394764	'FAM30A	family with sequence similarity 30 member A	Body	10927	'FAM30A	family with sequence similarity 30 member A
GA	cg15357945	1.90E-05	0.005409077	FDR0.01	11	57158361	'PRG2	bone marrow proteoglycan isoform 1 preproprotein	TSS1500;TSS1500;TSS1500;TSS	-238	'PRG2	bone marrow proteoglycan isoform 1 preproprotein
GA	cg15377871	1.07E-05	0.003585544	FDR0.01	14	23588325	'CEBPE	CCAAT/enhancer-binding protein epsilon	5'UTR	495	'CEBPE	CCAAT/enhancer-binding protein epsilon
GA	cg15387123	5.97E-07	0.000458915	FDR0.01	9	139891058	'PAXX;CLIC3	protein PAXX isoform 1;chloride intracellular channel protein 3	TES5000;TES5000;TES5000;TES	-38	'CLIC3	chloride intracellular channel protein 3
GA	cg15392111	3.25E-12	6.41E-08	BF|FDR0.01	4	140216263	'NDUFC1	NADH dehydrogenase [ubiquinone] 1 subunit C1, mitochondrial	Body;Body;Body;Body;Body;Bo	6101	'NDUFC1	NADH dehydrogenase [ubiquinone] 1 subunit C1, mitochondrial
GA	cg15414132	3.71E-06	0.001712161	FDR0.01	11	65150358	'SLC25A45;FRMD8	solute carrier family 25 member 45;FERM domain-containing protein 8	TSS1500;TSS1500;TSS1500;TSS	-213	'SLC25A45	solute carrier family 25 member 45
GA	cg15417798	9.04E-07	0.000621781	FDR0.01	19	14260651	'LOC100507373;ADGRL1	;adhesion G protein-coupled receptor L1 isoform 2 precursor	Body;3'UTR;3'UTR	12688	'LOC100507373	
GA	cg15440512	8.53E-07	0.000597035	FDR0.01	17	4401718	'SPNS2	protein spinster homolog 2	TSS1500	-457	'SPNS2	protein spinster homolog 2
GA	cg15442907	4.64E-05	0.0095745	FDR0.01	12	2800463	'CACNA1C;CACNA1C-AS1	voltage-dependent L-type calcium channel subunit alpha-1C;CACNA1C antise	3'UTR;3'UTR;3'UTR;3'UTR;3'UT	-140	'CACNA1C-AS1	CACNA1C antisense RNA 1
GA	cg15446939	9.71E-06	0.003354909	FDR0.01	17	38296011	'MSL1;CASC3	MSL1 protein;protein CASC3	TES5000;TES5000;TES5000;TSS	-778	'CASC3	protein CASC3
GA	cg15482893	8.42E-08	0.000106023	BF|FDR0.01	1	32837667	'BSDC1	BSD domain-containing protein 1	Body;Body;Body;Body;Body;Bo	9528	'TSSK3	testis-specific serine/threonine-protein kinase 3
GA	cg15499799	3.22E-05	0.007556076	FDR0.01	22	24823110	'SPECC1L-ADORA2A;ADORA2A;ADORA2A-AS1	SPECC1L-ADORA2A readthrough (NMD candidate);adenosine receptor A2a;A	Body;Body;TSS1500;TSS1500;T	-456	'ADORA2A	adenosine receptor A2a
GA	cg15541609	1.79E-05	0.005227922	FDR0.01	14	100705844	'YY1	transcriptional repressor protein YY1	1stExon	364	'YY1	transcriptional repressor protein YY1
GA	cg15542713	2.62E-06	0.001319172	FDR0.01	1	42385581	'HIVEP3	transcription factor HIVEP3 isoform a	TSS1500;TSS1500;TSS1500;Bod	-988	'HIVEP3	transcription factor HIVEP3 isoform a
GA	cg15544596	1.91E-05	0.005434268	FDR0.01	19	4683273	'DPP9;DPP9-AS1	dipeptidyl peptidase 9 isoform 1;DPP9 antisense RNA 1	Body;Body;Body	3980	'DPP9-AS1	DPP9 antisense RNA 1
GA	cg15546227	3.29E-08	5.18E-05	BF|FDR0.01	1	61546247	'NFIA	nuclear factor 1 A-type	Body;TSS1500;TSS5000;TSS500	-1163	'NFIA	nuclear factor 1 A-type
GA	cg15551881	5.06E-07	0.000402428	FDR0.01	9	123688715	'TRAF1	TNF receptor-associated factor 1	Body;Body	359	'TRAF1	TNF receptor-associated factor 1
GA	cg15555017	4.62E-06	0.00199315	FDR0.01	11	128455958	'ETS1	protein C-ets-1	Body	1495	'ETS1	protein C-ets-1
GA	cg15616915	1.10E-06	0.000718244	FDR0.01	2	219266163	'SLC11A1;CTDSP1;MIR26B	natural resistance-associated macrophage protein 1;carboxy-terminal domai	TES5000;Body;Body;Body;TSS1	-1205	'MIR26B	microRNA 26b
GA	cg15626350	5.44E-08	7.49E-05	BF|FDR0.01	6	152130207	'ESR1	estrogen receptor	Body;Body;Body;Body;Body;Bo	-34	'ESR1	estrogen receptor
GA	cg15630458	3.80E-07	0.000330481	FDR0.01	8	142986649	'			-118975	'MIR1302-7	microRNA 1302-7
GA	cg15677087	5.21E-06	0.002179038	FDR0.01	20	61584850	'SLC17A9	solute carrier family 17 member 9	Body;Body	790	'SLC17A9	solute carrier family 17 member 9
GA	cg15690760	1.30E-06	0.000820198	FDR0.01	5	6729662	'TENT4A	terminal nucleotidyltransferase 4A	Body;Body	16118	'TENT4A	terminal nucleotidyltransferase 4A
GA	cg15694715	2.49E-05	0.006441228	FDR0.01	11	118402280	'KMT2A;LOC101929089;TTC36;TMEM25	histone-lysine N-methyltransferase 2A isoform 2 precursor;;tetratricopeptide	TES5000;TES5000;TSS1500;TSS	374	'TMEM25	transmembrane protein 25 precursor
GA	cg15696058	1.65E-05	0.004914142	FDR0.01	4	15965425	'FGFBP2;PROM1	fibroblast growth factor-binding protein 2 precursor;prominin-1 precursor	TSS1500;TES5000;TES5000;TES	-627	'FGFBP2	fibroblast growth factor-binding protein 2 precursor
GA	cg15700009	6.42E-06	0.002539646	FDR0.01	11	18415711	'LDHA	L-lactate dehydrogenase A chain	TSS1500;TSS1500;TSS1500;TSS	-224	'LDHA	L-lactate dehydrogenase A chain
GA	cg15714321	4.66E-06	0.002004112	FDR0.01	21	38739180	'DYRK1A	dual specificity tyrosine-phosphorylation-regulated kinase 1A	Body;Body;5'UTR;TSS200;TSS1	-55	'DYRK1A	dual specificity tyrosine-phosphorylation-regulated kinase 1A
GA	cg15742605	4.15E-06	0.001856087	FDR0.01	1	879383	'SAMD11;NOC2L	sterile alpha motif domain-containing protein 11;nucleolar complex protein	Body;TES5000	15253	'NOC2L	nucleolar complex protein 2 homolog
GA	cg15750534	4.49E-07	0.000372836	FDR0.01	19	17623398	'LOC100507551;PGLS	;6-phosphogluconolactonase	TSS1500;Body	-700	'LOC100507551	
GA	cg15789095	3.45E-05	0.007922618	FDR0.01	1	32281466	'SPOCD1	SPOC domain-containing protein 1 isoform 1	Body;Body;Body	186	'SPOCD1	SPOC domain-containing protein 1 isoform 1
GA	cg15810415	8.79E-06	0.003140183	FDR0.01	4	111397134	'ENPEP	glutamyl aminopeptidase	TSS200	-176	'ENPEP	glutamyl aminopeptidase
GA	cg15817440	9.14E-09	2.01E-05	BF|FDR0.01	14	58599910	'ARMH4	armadillo-like helical domain-containing protein 4 precursor	Body;Body	19021	'ARMH4	armadillo-like helical domain-containing protein 4 precursor
GA	cg15835620	1.21E-06	0.00077032	FDR0.01	17	26903872	'ALDOC;SPAG5	fructose-bisphosphate aldolase C;sperm-associated antigen 5	5'UTR;TES5000	41	'ALDOC	fructose-bisphosphate aldolase C
GA	cg15849098	3.55E-06	0.001645801	FDR0.01	7	150211761	'GIMAP7	GTPase, IMAP family member 7	TSS200	-198	'GIMAP7	GTPase, IMAP family member 7
GA	cg15865771	3.00E-05	0.007237709	FDR0.01	17	12920709	'ELAC2	zinc phosphodiesterase ELAC protein 2	Body;Body;Body	635	'ELAC2	zinc phosphodiesterase ELAC protein 2
GA	cg15892280	2.94E-09	8.99E-06	BF|FDR0.01	21	40180000	'ETS2	protein C-ets-2	Body;Body	2148	'ETS2	protein C-ets-2
GA	cg15893360	3.29E-05	0.007658594	FDR0.01	12	120700361	'PXN	paxillin	Body;Body;Body	3172	'PXN	paxillin
GA	cg15908709	2.74E-05	0.006842589	FDR0.01	17	46676215	'HOXB-AS3;HOXB6	HOXB cluster antisense RNA 3;homeobox protein Hox-B6	Body;Body;Body;Body;Body;Bo	2896	'HOXB-AS3	HOXB cluster antisense RNA 3
GA	cg15948030	2.24E-09	7.46E-06	BF|FDR0.01	6	31760825	'VARS1;LSM2	valine--tRNA ligase;U6 snRNA-associated Sm-like protein LSm2	Body;TES5000	2704	'VARS1	valine--tRNA ligase
GA	cg15983605	4.54E-08	6.51E-05	BF|FDR0.01	11	7927557	'LOC283299	uncharacterized LOC283299	TSS200	-55	'LOC283299	uncharacterized LOC283299
GA	cg15984516	1.03E-06	0.000688948	FDR0.01	14	100680261	'			-25219	'YY1	transcriptional repressor protein YY1
GA	cg15986847	1.83E-05	0.005301625	FDR0.01	10	126311927	'FAM53B	protein FAM53B	Body	-80669	'FAM53B-AS1	FAM53B antisense RNA 1
GA	cg16001335	3.41E-08	5.33E-05	BF|FDR0.01	22	50219912	'BRD1	bromodomain-containing protein 1	TSS5000;TSS5000;TSS5000;TSS	-364	'BRD1	bromodomain-containing protein 1
GA	cg16014412	1.73E-05	0.005091751	FDR0.01	1	9687471	'			-24317	'PIK3CD	phosphatidylinositol 4,5-bisphosphate 3-kinase catalytic subunit delta isoform
GA	cg16014770	3.45E-06	0.001615284	FDR0.01	12	31882421	'AMN1	protein AMN1 homolog	TSS1500;TSS1500;TSS1500;TSS	-313	'AMN1	protein AMN1 homolog
GA	cg16026647	2.23E-05	0.00598594	FDR0.01	17	15689920	'MEIS3P1	Meis homeobox 3 pseudogene 1	TSS1500	-243	'MEIS3P1	Meis homeobox 3 pseudogene 1
GA	cg16037981	4.34E-10	2.26E-06	BF|FDR0.01	17	2700093	'RAP1GAP2	rap1 GTPase-activating protein 2 isoform 2	Body;Body;Body	362	'RAP1GAP2	rap1 GTPase-activating protein 2 isoform 2
GA	cg16060369	1.65E-06	0.000966724	FDR0.01	10	43698834	'RASGEF1A	ras-GEF domain-containing family member 1A	Body;Body	26353	'RASGEF1A	ras-GEF domain-containing family member 1A
GA	cg16061528	2.90E-05	0.007107775	FDR0.01	2	239420535	'LINC01107	long intergenic non-protein coding RNA 1107	Body	43605	'LINC01107	long intergenic non-protein coding RNA 1107
GA	cg16077055	4.18E-05	0.008952664	FDR0.01	2	106428750	'NCK2	cytoplasmic protein NCK2	Body;Body	-39552	'NCK2	cytoplasmic protein NCK2
GA	cg16090881	2.11E-06	0.001148116	FDR0.01	6	33246043	'RPS18;B3GALT4;WDR46	40S ribosomal protein S18;beta-1,3-galactosyltransferase 4;WD repeat-conta	TES5000;1stExon;TES5000;TES	1144	'B3GALT4	beta-1,3-galactosyltransferase 4
GA	cg16097041	2.42E-06	0.001263756	FDR0.01	1	154965544	'FLAD1;LENEP	FAD synthase;lens epithelial cell protein LEP503	TES5000;3'UTR;3'UTR;3'UTR;TS	-517	'LENEP	lens epithelial cell protein LEP503
GA	cg16104076	3.92E-05	0.008645923	FDR0.01	5	40682069	'PTGER4	prostaglandin E2 receptor EP4 subtype	Body	2053	'PTGER4	prostaglandin E2 receptor EP4 subtype
GA	cg16107105	5.98E-08	8.10E-05	BF|FDR0.01	7	150646704	'KCNH2	potassium voltage-gated channel subfamily H member 2	Body;Body;3'UTR;3'UTR	6188	'KCNH2	potassium voltage-gated channel subfamily H member 2
GA	cg16137564	1.46E-05	0.004498263	FDR0.01	14	89024217	'PTPN21	tyrosine-protein phosphatase non-receptor type 21	TSS5000	-2866	'PTPN21	tyrosine-protein phosphatase non-receptor type 21
GA	cg16140253	7.30E-16	1.19E-10	BF|FDR0.01	1	110166563	'AMPD2	AMP deaminase 2	Body;Body;Body;Body;TSS200	-46	'AMPD2	AMP deaminase 2
GA	cg16145216	4.51E-05	0.009395678	FDR0.01	1	42385662	'HIVEP3	transcription factor HIVEP3 isoform a	TSS1500;TSS1500;TSS1500;Bod	-1069	'HIVEP3	transcription factor HIVEP3 isoform a
GA	cg16223546	1.06E-05	0.003569991	FDR0.01	5	158768976	'LOC285626	uncharacterized LOC285626	Body	10451	'LOC285626	uncharacterized LOC285626
GA	cg16236143	4.27E-05	0.009067438	FDR0.01	20	4795935	'RASSF2	ras association domain-containing protein 2	TSS200;Body	-166	'RASSF2	ras association domain-containing protein 2
GA	cg16239536	2.69E-06	0.001343516	FDR0.01	19	1079617	'ARHGAP45	rho GTPase-activating protein 45	Body;Body;Body;Body;Body	2985	'ARHGAP45	rho GTPase-activating protein 45
GA	cg16253634	9.83E-06	0.003384855	FDR0.01	7	134464123	'CALD1	caldesmon	TSS1500;TSS1500;TSS1500	-261	'CALD1	caldesmon
GA	cg16280667	3.45E-05	0.007922618	FDR0.01	11	118754593	'CXCR5	C-X-C chemokine receptor type 5	TSS200	-7	'CXCR5	C-X-C chemokine receptor type 5
GA	cg16301617	1.62E-05	0.004858132	FDR0.01	17	76128906	'TMC6;TMC8	transmembrane channel-like protein 6;transmembrane channel-like protein	TSS5000;TSS5000;TSS1500;Bod	-398	'TMC6	transmembrane channel-like protein 6
GA	cg16304656	2.74E-05	0.006842589	FDR0.01	7	641337	'PRKAR1B;PRKAR1B-AS1	cAMP-dependent protein kinase type I-beta regulatory subunit;PRKAR1B ant	Body;Body;Body;Body;Body;Bo	-1144	'PRKAR1B-AS1	PRKAR1B antisense RNA 1
GA	cg16306870	5.02E-08	7.04E-05	BF|FDR0.01	3	194868790	'XXYLT1;XXYLT1-AS2	xyloside xylosyltransferase 1;XXYLT1 antisense RNA 2	Body;Body;Body;Body	191	'XXYLT1-AS2	XXYLT1 antisense RNA 2
GA	cg16317457	3.00E-05	0.007230021	FDR0.01	11	123173103	'			-56026	'GRAMD1B	protein Aster-B
GA	cg16330893	8.60E-07	0.000598841	FDR0.01	8	118662804	'			129853	'MED30	mediator of RNA polymerase II transcription subunit 30
GA	cg16344442	1.62E-05	0.004858132	FDR0.01	12	95010017	'TMCC3	transmembrane and coiled-coil domain protein 3	TSS1500;Body	-240	'TMCC3	transmembrane and coiled-coil domain protein 3

Trait	#cpg	p	fdr	significance	chr	pos.hg19	gene.Illumina	gene.description	locations	dist2TSS	gene.nearest	gene.description
GA	cg16348358	2.67E-06	0.001336904	FDR0.01	1	32731477	'LCK	tyrosine-protein kinase Lck	Body;Body	-8234	'LCK	tyrosine-protein kinase Lck
GA	cg16348385	7.84E-06	0.002904606	FDR0.01	16	30106822	'TBX6;YPEL3;LOC101928595	T-box transcription factor TBX6;protein yippee-like 3;	TSS5000;5'UTR;Body;TSS1500	712	'YPEL3	protein yippee-like 3
GA	cg16363586	1.57E-07	0.000169305	FDR0.01	19	17516329	'BST2;BISPR;MVB12A	bone marrow stromal antigen 2;BST2 interferon stimulated positive regulato	1stExon;TSS200;TSS200;TSS150	110	'BST2	bone marrow stromal antigen 2
GA	cg16373471	1.04E-05	0.003515574	FDR0.01	6	158982071	'TMEM181	transmembrane protein 181 isoform 2	Body	24604	'TMEM181	transmembrane protein 181 isoform 2
GA	cg16373526	1.12E-08	2.31E-05	BF|FDR0.01	11	70248709	'CTTN	src substrate cortactin	Body;Body;Body	4075	'CTTN	src substrate cortactin
GA	cg16374343	2.79E-05	0.00692881	FDR0.01	17	1014352	'ABR	active breakpoint cluster region-related protein isoform c	TSS5000;Body;Body;Body;Body	-2010	'ABR	active breakpoint cluster region-related protein isoform c
GA	cg16396284	2.40E-06	0.001251958	FDR0.01	6	33245537	'RPS18;B3GALT4;WDR46	40S ribosomal protein S18;beta-1,3-galactosyltransferase 4;WD repeat-conta	TES5000;1stExon;TES5000;TES5	638	'B3GALT4	beta-1,3-galactosyltransferase 4
GA	cg16398761	2.44E-05	0.006341063	FDR0.01	14	74220238	'ELMSAN1		Body;Body;Body	-5211	'MIR4505	microRNA 4505
GA	cg16398956	1.15E-06	0.000744865	FDR0.01	14	100212958	'			-46791	'EML1	echinoderm microtubule-associated protein-like 1 isoform b
GA	cg16399664	3.67E-07	0.000322828	FDR0.01	12	113417284	'OAS2	2'-5'-oligoadenylate synthase 2	Body;Body;Body	961	'OAS2	2'-5'-oligoadenylate synthase 2
GA	cg16401270	5.97E-10	2.82E-06	BF|FDR0.01	1	32404469	'PTP4A2	protein tyrosine phosphatase type IVA 3	TSS1500;TSS1500;TSS1500;TSS1	-500	'PTP4A2	protein tyrosine phosphatase type IVA 3
GA	cg16429499	2.59E-05	0.006620317	FDR0.01	16	3597151	'NLRC3	NLR family CARD domain-containing protein 3	Body;Body	30253	'NLRC3	NLR family CARD domain-containing protein 3
GA	cg16435539	2.33E-05	0.006151357	FDR0.01	3	39851009	'MYRIP	rab effector MyRIP	Body;TSS200;TSS200;TSS200;TS	-90	'MYRIP	rab effector MyRIP
GA	cg16435910	1.04E-05	0.003510259	FDR0.01	2	174488472	'			268873	'CDCA7	cell division cycle-associated protein 7
GA	cg16437021	1.89E-06	0.001065323	FDR0.01	16	89510331	'ANKRD11;LOC101927817	ankyrin repeat domain-containing protein 11;uncharacterized LOC10192781	Body;Body;Body;Body;Body;Bo	13006	'LOC101927817	uncharacterized LOC101927817
GA	cg16476991	1.37E-06	0.000846993	FDR0.01	13	114831404	'RASA3	ras GTPase-activating protein 3	Body;Body;Body	12026	'RASA3	ras GTPase-activating protein 3
GA	cg16509569	1.25E-07	0.000142459	FDR0.01	12	54891634	'NCKAP1L	nck-associated protein 1-like	1stExon;TSS1500	99	'NCKAP1L	nck-associated protein 1-like
GA	cg16517298	3.98E-05	0.008711177	FDR0.01	1	230413174	'GALNT2	polypeptide N-acetylgalactosaminyltransferase 2 precursor	Body;Body	148904	'PGBD5	piggyBac transposable element-derived protein 5
GA	cg16521454	2.25E-07	0.000220764	FDR0.01	5	150158951	'SMIM3	small integral membrane protein 3	Body	1444	'SMIM3	small integral membrane protein 3
GA	cg16536330	3.12E-06	0.001501075	FDR0.01	8	29957344	'LEPROTL1	leptin receptor overlapping transcript-like 1	Body;Body	4421	'LEPROTL1	leptin receptor overlapping transcript-like 1
GA	cg16541026	1.56E-05	0.004732997	FDR0.01	3	49026934	'ARIH2;P4HTM	E3 ubiquitin-protein ligase ARIH2;transmembrane prolyl 4-hydroxylase isofor	TES5000;TES5000;TES5000;TES	-406	'P4HTM	transmembrane prolyl 4-hydroxylase isoform c
GA	cg16553024	4.16E-06	0.001859025	FDR0.01	7	116138462	'CAV2	caveolin-2	TSS1500;TSS1500;TSS1500;TSS5	-1329	'CAV2	caveolin-2
GA	cg16594273	2.87E-06	0.00141781	FDR0.01	16	58131312	'			31973	'CFAP20	cilia- and flagella-associated protein 20
GA	cg16594502	9.28E-06	0.003252516	FDR0.01	15	40339900	'SRP14-AS1	SRP14 antisense RNA1 (head to head)	Body;Body;Body;Body	8389	'SRP14-AS1	SRP14 antisense RNA1 (head to head)
GA	cg16624069	1.16E-10	9.28E-07	BF|FDR0.01	13	99630210	'DOCK9	dedicator of cytokinesis protein 9	5'UTR;5'UTR;5'UTR;5'UTR;5'UTI	108	'DOCK9	dedicator of cytokinesis protein 9
GA	cg16659026	9.11E-09	2.01E-05	BF|FDR0.01	19	54617827	'TFPT;PRPF31	TCF3 fusion partner;U4/U6 small nuclear ribonucleoprotein Prp31	1stExon;Body;TSS1500	627	'TFPT	TCF3 fusion partner
GA	cg16660091	2.00E-06	0.001099025	FDR0.01	16	56995856	'CETP	cholesteryl ester transfer protein precursor	TSS200;TSS200	-5	'CETP	cholesteryl ester transfer protein precursor
GA	cg16674433	8.44E-06	0.003053789	FDR0.01	17	19314408	'RNF112	RING finger protein 112	TSS200	-136	'RNF112	RING finger protein 112
GA	cg16677144	3.03E-05	0.007286057	FDR0.01	1	1790217	'GNB1	guanine nucleotide-binding protein G(I)/G(S)/G(T) subunit beta-1	Body;Body;Body	32309	'GNB1	guanine nucleotide-binding protein G(I)/G(S)/G(T) subunit beta-1
GA	cg16679650	2.05E-05	0.005689047	FDR0.01	1	24239820	'CNR2	cannabinoid receptor 2	5'UTR	32	'CNR2	cannabinoid receptor 2
GA	cg16701258	1.24E-05	0.004012568	FDR0.01	19	5838729	'FUT6;FUT3	4-galactosyl-N-acetylglucosaminide 3-alpha-L-fucosyltransferase FUT6 isofor	5'UTR;5'UTR;5'UTR;5'UTR;5'UTI	74	'FUT6	4-galactosyl-N-acetylglucosaminide 3-alpha-L-fucosyltransferase FUT6 isoform 1
GA	cg16705546	5.33E-07	0.000419597	FDR0.01	16	85936666	'IRF8	interferon regulatory factor 8	1stExon;Body;5'UTR	46	'IRF8	interferon regulatory factor 8
GA	cg16710042	1.74E-08	3.13E-05	BF|FDR0.01	2	165760725	'SLC38A11	putative sodium-coupled neutral amino acid transporter 11	Body;Body;Body;Body;Body;Bo	51310	'SLC38A11	putative sodium-coupled neutral amino acid transporter 11
GA	cg16725984	7.43E-06	0.002798005	FDR0.01	16	89735184	'SPATA33	spermatogenesis-associated protein 33 isoform b	Body;Body;Body;Body;Body;Bo	10709	'SPATA33	spermatogenesis-associated protein 33 isoform b
GA	cg16729850	3.25E-05	0.007603125	FDR0.01	12	53339813	'KRT8;KRT18	keratin, type II cytoskeletal 8;keratin, type I cytoskeletal 18	Body;Body;TSS5000;TSS5000	-2841	'KRT18	keratin, type I cytoskeletal 18
GA	cg16762386	3.72E-05	0.008373436	FDR0.01	1	27240514	'NR0B2	nuclear receptor subfamily 0 group B member 2	TSS200	-48	'NR0B2	nuclear receptor subfamily 0 group B member 2
GA	cg16764778	1.96E-05	0.0055298	FDR0.01	15	41061410	'GCHFR;DNAJC17;C15orf62	GTP cyclohydrolase 1 feedback regulatory protein;dnaJ homolog subfamily C	TES5000;Body;TSS1500	-767	'C15orf62	uncharacterized protein C15orf62, mitochondrial precursor
GA	cg16774946	2.70E-07	0.000253397	FDR0.01	13	24798195	'SPATA13	spermatogenesis-associated protein 13 isoform 6	Body;Body;1stExon	30382	'SPATA13-AS1	SPATA13 antisense RNA 1
GA	cg16777510	3.23E-07	0.00029416	FDR0.01	17	40440022	'STAT5A	signal transducer and activator of transcription 5A isoform 1	Body;5'UTR;TSS200;TSS200	-51	'STAT5A	signal transducer and activator of transcription 5A isoform 1
GA	cg16794579	8.12E-09	1.88E-05	BF|FDR0.01	16	17562419	'XYLT1	xylosyltransferase 1 precursor	Body	2398	'XYLT1	xylosyltransferase 1 precursor
GA	cg16831185	2.45E-08	4.10E-05	BF|FDR0.01	17	29158530	'ATAD5	ATPase family AAA domain-containing protein 5	TSS1500	-453	'ATAD5	ATPase family AAA domain-containing protein 5
GA	cg16854524	2.13E-05	0.00584688	FDR0.01	4	83931902	'LIN54	protein lin-54 homolog	5'UTR;5'UTR;5'UTR;5'UTR;Body	74	'LIN54	protein lin-54 homolog
GA	cg16872595	1.76E-05	0.005151912	FDR0.01	18	77710649	'SLC66A2	solute carrier family 66 member 2	Body;Body;Body	1004	'SLC66A2	solute carrier family 66 member 2
GA	cg16874095	2.04E-06	0.001118582	FDR0.01	14	34529121	'			-108841	'EGLN3	prolyl hydroxylase EGLN3
GA	cg16882373	1.34E-05	0.004243003	FDR0.01	7	26191458	'NFE2L3	nuclear factor erythroid 2-related factor 3	TSS1500	-359	'NFE2L3	nuclear factor erythroid 2-related factor 3
GA	cg16884222	7.93E-08	0.000101639	BF|FDR0.01	11	65191531	'NEAT1	nuclear paraspeckle assembly transcript 1	Body;Body	1263	'NEAT1	nuclear paraspeckle assembly transcript 1
GA	cg16903510	1.52E-06	0.000913534	FDR0.01	19	49314475	'BCAT2;HSD17B14	branched-chain amino acid aminotransferase;17-beta-hydroxysteroid dehyd	TSS200;TSS200;TSS200;TES500	-189	'BCAT2	branched-chain amino acid aminotransferase
GA	cg16903735	2.12E-05	0.005838448	FDR0.01	11	1903176	'LSP1;MIR7847	lipid-binding protein LSP1;microRNA 7847	Body;Body;Body;Body;Body;Bo	1902	'MIR7847	microRNA 7847
GA	cg16912563	1.33E-06	0.000828715	FDR0.01	10	77157423	'ZNF503;ZNF503-AS2	zinc finger protein 503;ZNF503 antisense RNA 2	Body;TES5000;TSS5000	-3862	'ZNF503-AS2	ZNF503 antisense RNA 2
GA	cg16963852	3.11E-06	0.00149899	FDR0.01	3	150479084	'SIAH2	E3 ubiquitin-protein ligase SIAH2	Body	1872	'SIAH2	E3 ubiquitin-protein ligase SIAH2
GA	cg16990174	1.58E-05	0.00476344	FDR0.01	3	72496875	'RYBP	ring and YY1 binding protein, isoform A	TSS1500	-1101	'RYBP	ring and YY1 binding protein, isoform A
GA	cg17004353	4.34E-05	0.009167231	FDR0.01	11	3073441	'CARS1	cysteine--tRNA ligase, cytoplasmic	Body;Body;Body;Body;Body	5212	'CARS1	cysteine--tRNA ligase, cytoplasmic
GA	cg17009433	1.21E-05	0.003943719	FDR0.01	9	6645686	'GLDC	glycine dehydrogenase (decarboxylating), mitochondrial precursor	5'UTR	6	'GLDC	glycine dehydrogenase (decarboxylating), mitochondrial precursor
GA	cg17018786	8.88E-06	0.003158989	FDR0.01	15	40659816	'DISP2	protein dispatched homolog 2	Body	9397	'DISP2	protein dispatched homolog 2
GA	cg17022038	3.10E-07	0.000284877	FDR0.01	19	18260479	'MAST3;PIK3R2	microtubule-associated serine/threonine-protein kinase 3;phosphatidylinosi	Body;TSS5000;TSS5000;TSS500	-3493	'PIK3R2	phosphatidylinositol 3-kinase regulatory subunit beta
GA	cg17084151	2.87E-06	0.001417448	FDR0.01	8	102803237	'NCALD	neurocalcin-delta	TSS200;Body;Body;Body;Body;B	-40	'NCALD	neurocalcin-delta
GA	cg17103217	1.12E-05	0.003731748	FDR0.01	6	33245721	'RPS18;B3GALT4;WDR46	40S ribosomal protein S18;beta-1,3-galactosyltransferase 4;WD repeat-conta	TES5000;1stExon;TES5000;TES5	822	'B3GALT4	beta-1,3-galactosyltransferase 4
GA	cg17107867	3.38E-05	0.007800019	FDR0.01	1	153522407	'S100A4;LOC101928034;S100A3	protein S100-A4;uncharacterized LOC101928034;protein S100-A3	TSS5000;TSS5000;Body;TSS150	-673	'S100A3	protein S100-A3
GA	cg17113856	1.63E-07	0.000171971	FDR0.01	6	32120895	'PRRT1;LOC100507547;PPT2;PPT2-EGFL8	proline-rich transmembrane protein 1;uncharacterized LOC100507547;lysos	TSS200;TSS1500;Body;Body;Bo	-35	'PRRT1	proline-rich transmembrane protein 1
GA	cg17117459	6.24E-08	8.38E-05	BF|FDR0.01	13	25085405	'PARP4	protein mono-ADP-ribosyltransferase PARP4	Body	1511	'PARP4	protein mono-ADP-ribosyltransferase PARP4
GA	cg17133774	1.64E-07	0.000173203	FDR0.01	1	6198667	'CHD5	chromodomain-helicase-DNA-binding protein 5	Body	41714	'CHD5	chromodomain-helicase-DNA-binding protein 5
GA	cg17139085	2.55E-05	0.006538561	FDR0.01	1	36787356	'SH3D21;EVA1B	SH3 domain-containing protein 21;protein eva-1 homolog B	TES5000;TES5000;TES5000;TES	1890	'EVA1B	protein eva-1 homolog B
GA	cg17158750	3.26E-06	0.001551056	FDR0.01	20	61924382	'ARFGAP1;COL20A1	ADP-ribosylation factor GTPase-activating protein 1;collagen alpha-1(XX) cha	TES5000;TES5000;TES5000;TES	-155	'COL20A1	collagen alpha-1(XX) chain precursor
GA	cg17178761	9.46E-10	3.95E-06	BF|FDR0.01	17	55682851	'MSI2;LOC101927539	RNA-binding protein Musashi homolog 2;uncharacterized LOC101927539	Body;Body;Body;Body;Body	2922	'LOC101927539	uncharacterized LOC101927539
GA	cg17181653	1.99E-05	0.005570512	FDR0.01	6	144417537	'SF3B5	splicing factor 3B subunit 5	TSS1500	-827	'SF3B5	splicing factor 3B subunit 5
GA	cg17192381	4.40E-08	6.35E-05	BF|FDR0.01	18	60792598	'BCL2	apoptosis regulator Bcl-2	3'UTR	194015	'BCL2	apoptosis regulator Bcl-2
GA	cg17264271	5.10E-07	0.000405089	FDR0.01	6	152126938	'ESR1	estrogen receptor	Body;5'UTR;5'UTR;5'UTR;TSS50	131	'ESR1	estrogen receptor
GA	cg17288288	3.19E-05	0.007490615	FDR0.01	13	51418092	'DLEU7;DLEU7-AS1	leukemia-associated protein 7 isoform 2;DLEU7 antisense RNA 1	TSS200;Body;TSS200	-17	'DLEU7	leukemia-associated protein 7 isoform 2
GA	cg17298279	2.43E-10	1.47E-06	BF|FDR0.01	6	158982078	'TMEM181	transmembrane protein 181 isoform 2	Body	24611	'TMEM181	transmembrane protein 181 isoform 2
GA	cg17298925	1.84E-08	3.23E-05	BF|FDR0.01	5	6828054	'LINC02236;MIR4278	long intergenic non-protein coding RNA 2236;microRNA 4278	TES5000;TSS200	-20	'MIR4278	microRNA 4278
GA	cg17331950	6.15E-06	0.002455327	FDR0.01	1	181075666	'			17831	'IER5	immediate early response gene 5 protein
GA	cg17394304	2.27E-05	0.006070677	FDR0.01	3	187453973	'LOC100131635;BCL6	hCG1645011-like;B-cell lymphoma 6 protein	TES5000;TSS1500;5'UTR;Body	316	'BCL6	B-cell lymphoma 6 protein
GA	cg17399545	2.55E-06	0.001299485	FDR0.01	12	88972226	'KITLG	kit ligand precursor	Body;Body	2022	'KITLG	kit ligand precursor
GA	cg17404981	6.22E-08	8.38E-05	BF|FDR0.01	4	1240264	'CTBP1;CTBP1-DT	C-terminal-binding protein 1;CTBP1 divergent transcript	Body;Body;TSS5000	2654	'CTBP1	C-terminal-binding protein 1
GA	cg17431888	1.92E-06	0.001072185	FDR0.01	1	183203863	'LAMC2	laminin subunit gamma-2 precursor	Body;Body	48465	'LAMC2	laminin subunit gamma-2 precursor
GA	cg17452301	1.03E-09	4.24E-06	BF|FDR0.01	10	134211620	'PWWP2B	PWWP domain-containing protein 2B	Body;Body	917	'PWWP2B	PWWP domain-containing protein 2B
GA	cg17474524	7.65E-08	9.87E-05	BF|FDR0.01	20	62168831	'PTK6;SRMS	protein-tyrosine kinase 6;tyrosine-protein kinase Srms	TSS200;TSS200;TES5000	-102	'PTK6	protein-tyrosine kinase 6
GA	cg17514088	2.70E-05	0.006777303	FDR0.01	17	76503074	'DNAH17;DNAH17-AS1	dynein heavy chain 17, axonemal;DNAH17 antisense RNA 1	Body;TES5000	22083	'DNAH17-AS1	DNAH17 antisense RNA 1
GA	cg17593625	2.99E-06	0.001454315	FDR0.01	7	752800	'PRKAR1B	cAMP-dependent protein kinase type I-beta regulatory subunit	TSS1500;TSS1500;5'UTR;Body;E	44	'PRKAR1B	cAMP-dependent protein kinase type I-beta regulatory subunit
GA	cg17621438	7.73E-06	0.002875279	FDR0.01	5	63461216	'RNF180	E3 ubiquitin-protein ligase RNF180	TSS1500;TSS1500;TSS1500;TSS1	-453	'RNF180	E3 ubiquitin-protein ligase RNF180
GA	cg17631451	5.42E-06	0.002241996	FDR0.01	3	48507354	'ATRIP;ATRIP-TREX1;TREX1;SHISA5	ATR-interacting protein;ATRIP-TREX1 readthrough;three-prime repair exonu	TES5000;3'UTR;3'UTR;Body;3'U	126	'TREX1	three-prime repair exonuclease 1 isoform c
GA	cg17700835	1.80E-05	0.005259275	FDR0.01	6	27390890	'ZNF184	zinc finger protein 184 isoform 2	Body;Body	34394	'ZNF391	zinc finger protein 391
GA	cg17714703	7.68E-08	9.87E-05	BF|FDR0.01	19	4912221	'UHRF1	E3 ubiquitin-protein ligase UHRF1	Body;Body;Body;Body;Body	1843	'UHRF1	E3 ubiquitin-protein ligase UHRF1
GA	cg17754782	2.88E-05	0.007086753	FDR0.01	17	72576122	'CD300LD;C17orf77	CMRF35-like molecule 5 precursor;uncharacterized protein C17orf77	3'UTR;TSS5000;TSS5000	-4695	'C17orf77	uncharacterized protein C17orf77
GA	cg17782000	8.37E-06	0.003043015	FDR0.01	19	18303124	'MPV17L2;RAB3A	mpv17-like protein 2 precursor;ras-related protein Rab-3A	TSS1500;TES5000	-903	'MPV17L2	mpv17-like protein 2 precursor
GA	cg17796323	2.38E-06	0.001249385	FDR0.01	5	177547973	'N4BP3	NEDD4-binding protein 3	Body	7441	'N4BP3	NEDD4-binding protein 3
GA	cg17801546	5.23E-07	0.000413359	FDR0.01	1	167090974	'DUSP27	inactive dual specificity phosphatase 27	Body	27663	'DUSP27	inactive dual specificity phosphatase 27

Trait	#cpg	p	fdr	significance	chr	pos.hg19	gene.Illumina	gene.description	locations	dist2TSS	gene.nearest	gene.description
GA	cg17806418	1.53E-08	2.92E-05	BF|FDR0.01	6	29599319	'GABBR1	gamma-aminobutyric acid type B receptor subunit 1 precursor	TSS5000;5'UTR;Body;Body	1593	'GABBR1	gamma-aminobutyric acid type B receptor subunit 1 precursor
GA	cg17819250	2.34E-05	0.006173504	FDR0.01	8	36380237	'			-261654	'KCNU1	potassium channel subfamily U member 1
GA	cg17819732	1.29E-05	0.004130828	FDR0.01	1	203147367	'MYBPH;CHI3L1	myosin-binding protein H;chitinase-3-like protein 1 precursor	TSS5000;TES5000	-2413	'MYBPH	myosin-binding protein H
GA	cg17826530	1.21E-08	2.43E-05	BF|FDR0.01	1	59280290	'LINC01135	long intergenic non-protein coding RNA 1135	Body;Body;Body	29468	'LINC01135	long intergenic non-protein coding RNA 1135
GA	cg17837526	3.82E-05	0.008522108	FDR0.01	5	172410489	'ATP6V0E1	V-type proton ATPase subunit e 1	TSS1500	-284	'ATP6V0E1	V-type proton ATPase subunit e 1
GA	cg17848515	5.48E-07	0.000429246	FDR0.01	2	130940706	'SMPD4;MZT2B	sphingomyelin phosphodiesterase 4;mitotic-spindle organizing protein 2B	TSS1500;TSS1500;TSS1500;TSS5	872	'MZT2B	mitotic-spindle organizing protein 2B
GA	cg17881600	4.90E-08	6.90E-05	BF|FDR0.01	6	17012979	'			-89301	'STMND1	stathmin domain-containing protein 1
GA	cg17888086	9.42E-06	0.003292529	FDR0.01	1	37945057	'ZC3H12A;MIR6732	ribonuclease ZC3H12A;microRNA 6732	Body;Body;Body;TSS1500	-773	'MIR6732	microRNA 6732
GA	cg17898925	1.08E-07	0.000129809	BF|FDR0.01	4	83196047	'			99097	'HNRNPD	heterogeneous nuclear ribonucleoprotein D0
GA	cg17924072	3.91E-13	1.46E-08	BF|FDR0.01	19	1075067	'ARHGAP45	rho GTPase-activating protein 45	Body;Body;Body;Body;TSS5000	-1565	'ARHGAP45	rho GTPase-activating protein 45
GA	cg17936488	8.27E-06	0.003023415	FDR0.01	9	134151519	'FAM78A	protein FAM78A	1stExon	378	'FAM78A	protein FAM78A
GA	cg17942763	5.26E-09	1.40E-05	BF|FDR0.01	1	2163472	'SKI	ski oncogene	Body	3715	'SKI	ski oncogene
GA	cg17950169	3.21E-11	3.99E-07	BF|FDR0.01	1	226067900	'TMEM63A	CSC1-like protein 1	Body	2180	'TMEM63A	CSC1-like protein 1
GA	cg17988187	4.07E-05	0.008820226	FDR0.01	2	74612222	'DCTN1;DCTN1-AS1	dynactin subunit 1 isoform 1;DCTN1 antisense RNA 1	TSS5000;TSS5000;TSS5000;Bod	-407	'DCTN1-AS1	DCTN1 antisense RNA 1
GA	cg18007957	1.12E-08	2.31E-05	BF|FDR0.01	6	152011666	'ESR1	estrogen receptor	5'UTR	36	'ESR1	estrogen receptor
GA	cg18019132	6.55E-06	0.002574857	FDR0.01	11	65191707	'NEAT1	nuclear paraspeckle assembly transcript 1	Body;Body	1439	'NEAT1	nuclear paraspeckle assembly transcript 1
GA	cg18076651	2.63E-09	8.28E-06	BF|FDR0.01	12	53625605	'RARG	retinoic acid receptor gamma	Body;Body;Body	388	'RARG	retinoic acid receptor gamma
GA	cg18082788	1.16E-05	0.003821264	FDR0.01	6	149806339	'ZC3H12D	probable ribonuclease ZC3H12D	TSS200	-189	'ZC3H12D	probable ribonuclease ZC3H12D
GA	cg18090197	6.48E-06	0.002554157	FDR0.01	7	99769602	'GAL3ST4;GPC2	galactose-3-O-sulfotransferase 4;glypican-2 precursor	TSS5000;Body	-3362	'GAL3ST4	galactose-3-O-sulfotransferase 4
GA	cg18115132	1.63E-05	0.004861424	FDR0.01	1	107819805	'NTNG1	netrin-G1 isoform G1c precursor	Body;Body;Body;Body;Body;Bo	135849	'NTNG1	netrin-G1 isoform G1c precursor
GA	cg18115766	3.24E-05	0.007584484	FDR0.01	3	23960290	'NKIRAS1;RPL15	NF-kappa-B inhibitor-interacting Ras-like protein 1;60S ribosomal protein L15	TSS5000;Body;Body;Body;Body	1668	'RPL15	60S ribosomal protein L15
GA	cg18123677	3.63E-05	0.008241298	FDR0.01	3	167454339	'PDCD10;SERPINI1	programmed cell death protein 10;neuroserpin precursor	TSS5000;TSS5000;TSS5000;Bod	829	'SERPINI1	neuroserpin precursor
GA	cg18137244	2.51E-05	0.006472746	FDR0.01	7	2444210	'CHST12	carbohydrate sulfotransferase 12	Body;Body;Body	965	'CHST12	carbohydrate sulfotransferase 12
GA	cg18144742	5.41E-06	0.002241405	FDR0.01	14	107252125	'			7089	'MIR5195	microRNA 5195
GA	cg18157896	1.61E-05	0.004852514	FDR0.01	20	2795593	'C20orf141;TMEM239	uncharacterized protein C20orf141;transmembrane protein 239 isoform 2	TSS200;TSS200;TSS1500;TSS150	-39	'C20orf141	uncharacterized protein C20orf141
GA	cg18164508	8.60E-06	0.003100444	FDR0.01	12	133235007	'POLE	DNA polymerase epsilon catalytic subunit A	Body	28921	'POLE	DNA polymerase epsilon catalytic subunit A
GA	cg18182566	1.97E-05	0.00553265	FDR0.01	14	105888161	'LOC100507437;MTA1	uncharacterized LOC100507437;metastasis-associated protein MTA1	TSS5000;TSS5000;Body;Body	1998	'MTA1	metastasis-associated protein MTA1
GA	cg18188739	3.10E-06	0.001495921	FDR0.01	1	2058941	'PRKCZ	protein kinase C zeta type	Body;Body;Body;Body;Body;Bo	22787	'PRKCZ	protein kinase C zeta type
GA	cg18233411	1.40E-06	0.000859928	FDR0.01	12	74934087	'ATXN7L3B	ataxin-7-like protein 3B	3'UTR	2473	'ATXN7L3B	ataxin-7-like protein 3B
GA	cg18248273	3.93E-05	0.008645923	FDR0.01	17	77896431	'LINC01979;LINC01978	long intergenic non-protein coding RNA 1979;long intergenic non-protein co	Body;Body	3276	'LINC01978	long intergenic non-protein coding RNA 1978
GA	cg18259535	2.15E-06	0.001161911	FDR0.01	11	66092918	'RIN1	ras and Rab interactor 1	TES5000;TES5000;TES5000	-8403	'CD248	endosialin precursor
GA	cg18328933	1.58E-05	0.00476344	FDR0.01	3	52008538	'ABHD14B;ABHD14A;ABHD14A-ACY1	protein ABHD14B;protein ABHD14A precursor;ABHD14A-ACY1 readthrough (	5'UTR;5'UTR;5'UTR;TSS1500;TS	108	'ABHD14B	protein ABHD14B
GA	cg18353563	8.92E-06	0.003165363	FDR0.01	3	127390935	'PODXL2;ABTB1	podocalyxin-like protein 2 precursor;ankyrin repeat and BTB/POZ domain-co	Body;TSS1500;TSS1500;TSS150	-845	'ABTB1	ankyrin repeat and BTB/POZ domain-containing protein 1
GA	cg18358061	2.92E-05	0.007120757	FDR0.01	3	134126698	'			-29970	'MIR4788	microRNA 4788
GA	cg18358136	1.34E-07	0.00015026	FDR0.01	19	46119636	'EML2	echinoderm microtubule-associated protein-like 2 isoform 3	Body;Body;Body;Body;Body;Bo	-14166	'GPR4	G-protein coupled receptor 4
GA	cg18365406	4.50E-06	0.001954883	FDR0.01	19	11472110	'PLPPR2	phospholipid phosphatase-related protein type 2 isoform 2	Body;Body	6003	'PLPPR2	phospholipid phosphatase-related protein type 2 isoform 2
GA	cg18378811	3.08E-06	0.001485505	FDR0.01	4	114900050	'ARSJ	arylsulfatase J	5'UTR;5'UTR;5'UTR	753	'ARSJ	arylsulfatase J
GA	cg18404811	3.54E-07	0.000313298	FDR0.01	17	403288	'			-107557	'RFLNB	refilin-B
GA	cg18405140	1.09E-06	0.000715501	FDR0.01	19	1207644	'STK11	serine/threonine-protein kinase STK11	Body	1868	'STK11	serine/threonine-protein kinase STK11
GA	cg18415791	1.09E-05	0.003661522	FDR0.01	11	3178024	'OSBPL5	oxysterol-binding protein-related protein 5 isoform a	Body;Body;Body	8516	'OSBPL5	oxysterol-binding protein-related protein 5 isoform a
GA	cg18422587	5.48E-09	1.44E-05	BF|FDR0.01	7	3134670	'			-51169	'CARD11	caspase recruitment domain-containing protein 11
GA	cg18444673	2.06E-05	0.005706124	FDR0.01	19	36389500	'NFKBID;HCST	NF-kappa-B inhibitor delta;hematopoietic cell signal transducer precursor	Body;Body;Body;Body;Body;TSS	1139	'NFKBID	NF-kappa-B inhibitor delta
GA	cg18454299	1.57E-07	0.000169305	FDR0.01	15	44959064	'SPG11;PATL2	spatacsin;protein PAT1 homolog 2 isoform 1	TSS5000;TSS5000;Body;Body	-3204	'SPG11	spatacsin
GA	cg18474885	5.56E-06	0.002285468	FDR0.01	21	46292084	'PTTG1IP	pituitary tumor-transforming gene 1 protein-interacting protein precursor	Body;Body;Body	1521	'PTTG1IP	pituitary tumor-transforming gene 1 protein-interacting protein precursor
GA	cg18476766	7.01E-06	0.002687741	FDR0.01	2	240529742	'			-154811	'LOC150935	uncharacterized LOC150935
GA	cg18496212	3.09E-05	0.007364993	FDR0.01	3	69797108	'MITF	microphthalmia-associated transcription factor	Body;Body;Body;Body;Body;Bo	8494	'MITF	microphthalmia-associated transcription factor
GA	cg18520238	7.92E-06	0.002927963	FDR0.01	11	36422557	'PRR5L	proline-rich protein 5-like	5'UTR;Body;5'UTR;5'UTR	11	'PRR5L	proline-rich protein 5-like
GA	cg18537222	4.05E-06	0.001830165	FDR0.01	3	12435731	'PPARG	peroxisome proliferator-activated receptor gamma	Body;Body;Body;Body;Body;Bo	42731	'PPARG	peroxisome proliferator-activated receptor gamma
GA	cg18556455	6.91E-06	0.002661343	FDR0.01	2	45178474	'			-9457	'SIX3-AS1	SIX3 antisense RNA 1
GA	cg18582260	4.15E-08	6.06E-05	BF|FDR0.01	13	25085301	'PARP4	protein mono-ADP-ribosyltransferase PARP4	Body	1615	'PARP4	protein mono-ADP-ribosyltransferase PARP4
GA	cg18586823	3.48E-05	0.007990815	FDR0.01	5	114525702	'			-9459	'TRIM36	E3 ubiquitin-protein ligase TRIM36
GA	cg18592365	2.00E-06	0.001099355	FDR0.01	1	79472536	'ADGRL4	adhesion G protein-coupled receptor L4 precursor	TSS200	-121	'ADGRL4	adhesion G protein-coupled receptor L4 precursor
GA	cg18601167	3.53E-05	0.008069731	FDR0.01	7	752286	'PRKAR1B	cAMP-dependent protein kinase type I-beta regulatory subunit	TSS200;Body;Body;Body;Body;E	-125	'PRKAR1B	cAMP-dependent protein kinase type I-beta regulatory subunit
GA	cg18608055	2.75E-05	0.006845882	FDR0.01	19	1130866	'SBNO2	protein strawberry notch homolog 2	Body;Body	1354	'SBNO2	protein strawberry notch homolog 2
GA	cg18625289	6.44E-06	0.002544626	FDR0.01	19	1591035	'MBD3	methyl-CpG-binding domain protein 3	Body;Body	1829	'MBD3	methyl-CpG-binding domain protein 3
GA	cg18634506	7.46E-06	0.002806465	FDR0.01	7	99007560	'PDAP1;BUD31;MIR12119	28 kDa heat- and acid-stable phosphoprotein;protein BUD31 homolog;micro	TSS1500;Body;Body;Body;TES5(	766	'MIR12119	microRNA 12119
GA	cg18642234	2.65E-06	0.001329339	FDR0.01	3	49394622	'GPX1;RHOA	glutathione peroxidase 1;transforming protein RhoA	3'UTR;3'UTR;3'UTR;3'UTR;3'UTI	1164	'GPX1	glutathione peroxidase 1
GA	cg18651932	1.26E-09	4.94E-06	BF|FDR0.01	4	77226974	'FAM47E-STBD1;STBD1	uncharacterized protein LOC100631383;starch-binding domain-containing pı	Body;TSS1500	-204	'STBD1	starch-binding domain-containing protein 1
GA	cg18666075	1.52E-06	0.000911324	FDR0.01	3	156833184	'LINC00880	long intergenic non-protein coding RNA 880	Body	7607	'LINC00880	long intergenic non-protein coding RNA 880
GA	cg18674487	1.94E-06	0.001076846	FDR0.01	6	125203651	'			-80039	'RNF217	probable E3 ubiquitin-protein ligase RNF217 isoform b
GA	cg18697487	3.38E-05	0.007803092	FDR0.01	19	1502095	'REEP6;ADAMTSL5	receptor expression-enhancing protein 6;ADAMTS-like protein 5 isoform 1 pı	TES5000;TES5000;TES5000;TES	10916	'REEP6	receptor expression-enhancing protein 6
GA	cg18727788	1.18E-10	9.28E-07	BF|FDR0.01	12	31944556	'H3-5	histone H3.3C	3'UTR	676	'H3-5	histone H3.3C
GA	cg18731680	6.63E-07	0.000496565	FDR0.01	22	19953712	'COMT;MIR4761;ARVCF	catechol O-methyltransferase;microRNA 4761;armadillo repeat protein delet	Body;Body;Body;Body;Body;TES	2437	'MIR4761	microRNA 4761
GA	cg18754118	1.39E-06	0.00085407	FDR0.01	19	2588376	'GNG7	guanine nucleotide-binding protein G(I)/G(S)/G(O) subunit gamma-7	Body	-42336	'MIR7850	microRNA 7850
GA	cg18769818	4.12E-06	0.001848217	FDR0.01	11	72433059	'ARAP1	arf-GAP with Rho-GAP domain, ANK repeat and PH domain-containing protei	5'UTR;5'UTR;5'UTR;Body;Body	326	'ARAP1	arf-GAP with Rho-GAP domain, ANK repeat and PH domain-containing protein 1 isoform d
GA	cg18808929	4.72E-05	0.009666218	FDR0.01	8	61825469	'			54838	'LOC100130298	hCG1816373-like
GA	cg18810691	3.09E-05	0.007368105	FDR0.01	7	151130479	'CRYGN;MIR3907	gamma-crystallin N;microRNA 3907	Body;Body;Body;TES5000	246	'MIR3907	microRNA 3907
GA	cg18833928	8.68E-06	0.00311387	FDR0.01	7	38357684	'			-23493	'TRG-AS1	T cell receptor gamma locus antisense RNA 1
GA	cg18841653	2.63E-09	8.28E-06	BF|FDR0.01	10	5046097	'AKR1C2	aldo-keto reductase family 1 member C2	5'UTR;5'UTR;5'UTR;5'UTR	109	'AKR1C2	aldo-keto reductase family 1 member C2
GA	cg18844118	9.46E-07	0.000644763	FDR0.01	7	26191489	'NFE2L3	nuclear factor erythroid 2-related factor 3	TSS1500	-328	'NFE2L3	nuclear factor erythroid 2-related factor 3
GA	cg18876826	2.95E-05	0.007183669	FDR0.01	1	38396334	'INPP5B	type II inositol 1,4,5-trisphosphate 5-phosphatase isoform 2 precursor	Body;Body;Body;Body;Body;Bo	1077	'INPP5B	type II inositol 1,4,5-trisphosphate 5-phosphatase isoform 2 precursor
GA	cg18892239	1.79E-07	0.000186905	FDR0.01	6	31620798	'BAG6;APOM	large proline-rich protein BAG6;apolipoprotein M precursor	TSS1500;TSS1500;TSS1500;TSS1	-321	'BAG6	large proline-rich protein BAG6
GA	cg18900271	4.32E-06	0.001899024	FDR0.01	2	110863886	'MALL	MAL-like protein	Body;Body;Body	9513	'MALL	MAL-like protein
GA	cg18900591	1.03E-06	0.000688948	FDR0.01	20	50419193	'SALL4	sal-like protein 4	TSS200;TSS200	-133	'SALL4	sal-like protein 4
GA	cg18940763	1.92E-06	0.001072185	FDR0.01	22	29197846	'XBP1	X-box-binding protein 1 isoform XBP1(S)	TSS1500;TSS1500	-1286	'XBP1	X-box-binding protein 1 isoform XBP1(S)
GA	cg18966401	1.95E-06	0.001079122	FDR0.01	1	201979478	'RNPEP;ELF3-AS1;ELF3	aminopeptidase B isoform c;ELF3 antisense RNA 1;ETS-related transcription f	TES5000;TES5000;TES5000;TES	3	'ELF3-AS1	ELF3 antisense RNA 1
GA	cg19008371	2.35E-07	0.000227554	FDR0.01	11	126226766	'GSEC;ST3GAL4	G-quadruplex forming sequence containing lncRNA;CMP-N-acetylneuramina	TSS1500;Body;Body;Body;Body	860	'ST3GAL4	CMP-N-acetylneuraminate-beta-galactosamide- alpha-2,3-sialyltransferase 4
GA	cg19020855	1.11E-05	0.003689027	FDR0.01	17	41669266	'			-45558	'ETV4	ETS translocation variant 4 isoform 2
GA	cg19035526	8.46E-06	0.0030552	FDR0.01	2	217107125	'			25514	'LINC01963	long intergenic non-protein coding RNA 1963
GA	cg19049607	9.96E-06	0.003417326	FDR0.01	12	12764333	'CREBL2	cAMP-responsive element-binding protein-like 2	TSS1500	-476	'CREBL2	cAMP-responsive element-binding protein-like 2
GA	cg19056004	6.13E-06	0.002453578	FDR0.01	12	7023262	'LRRC23;ENO2	leucine-rich repeat-containing protein 23 isoform a;gamma-enolase	3'UTR;3'UTR;3'UTR;TSS1500	-481	'ENO2	gamma-enolase
GA	cg19066171	3.98E-05	0.008713473	FDR0.01	4	72061917	'SLC4A4	electrogenic sodium bicarbonate cotransporter 1	Body;Body	8915	'SLC4A4	electrogenic sodium bicarbonate cotransporter 1
GA	cg19103429	1.69E-06	0.000984798	FDR0.01	7	92460784	'CDK6	cyclin-dependent kinase 6	Body;Body	2489	'CDK6	cyclin-dependent kinase 6
GA	cg19103704	4.55E-06	0.001968784	FDR0.01	19	40440963	'FCGBP	IgGFc-binding protein precursor	TSS1500	-430	'FCGBP	IgGFc-binding protein precursor
GA	cg19136673	1.44E-07	0.000157899	FDR0.01	6	32813337	'PSMB8;PSMB8-AS1;TAP1	proteasome subunit beta type-8;PSMB8 antisense RNA 1 (head to head);anti	TSS5000;TSS1500;Body;Body;B	-881	'PSMB8	proteasome subunit beta type-8
GA	cg19169154	2.47E-05	0.006408289	FDR0.01	17	19287978	'MAPK7;MFAP4	mitogen-activated protein kinase 7;microfibril-associated glycoprotein 4 pre	TES5000;TES5000;TES5000;TES	2525	'MFAP4	microfibril-associated glycoprotein 4 precursor

Trait	#cpg	p	fdr	significance	chr	pos.hg19	gene.Illumina	gene.description	locations	dist2TSS	gene.nearest	gene.description
GA	cg19174643	3.74E-05	0.008410914	FDR0.01	18	13136426	'			-82332	'LDLRAD4	low-density lipoprotein receptor class A domain-containing protein 4
GA	cg19217955	1.78E-08	3.16E-05	BF|FDR0.01	17	7123994	'DLG4;ACADVL;MIR324;DVL2	disks large homolog 4;very long-chain specific acyl-CoA dehydrogenase, mito	TSS5000;TSS5000;TSS1500;TSS	-625	'DLG4	disks large homolog 4
GA	cg19220719	8.72E-06	0.003125193	FDR0.01	10	77532329	'LRMDA	leucine-rich melanocyte differentiation-associated protein	Body;Body	-10189	'LRMDA	leucine-rich melanocyte differentiation-associated protein
GA	cg19230709	2.81E-05	0.006947446	FDR0.01	19	4933046	'UHRF1;MIR4747	E3 ubiquitin-protein ligase UHRF1;microRNA 4747	Body;Body;Body;Body;Body;TE	348	'MIR4747	microRNA 4747
GA	cg19233405	1.46E-05	0.004495389	FDR0.01	1	154988721	'ZBTB7B;DCST2	zinc finger and BTB domain-containing protein 7B;DC-STAMP domain-contai	Body;Body;Body;Body;Body;TE	13616	'ZBTB7B	zinc finger and BTB domain-containing protein 7B
GA	cg19241468	2.54E-06	0.001299485	FDR0.01	3	122399344	'PARP14	poly [ADP-ribose] polymerase 14	TSS1500	-341	'PARP14	poly [ADP-ribose] polymerase 14
GA	cg19241689	2.79E-05	0.006920655	FDR0.01	6	33245516	'RPS18;B3GALT4;WDR46	40S ribosomal protein S18;beta-1,3-galactosyltransferase 4;WD repeat-conta	TES5000;1stExon;TES5000;TES	617	'B3GALT4	beta-1,3-galactosyltransferase 4
GA	cg19258882	5.41E-06	0.002241405	FDR0.01	12	56472805	'ERBB3	receptor tyrosine-protein kinase erbB-3 precursor	TSS1500;TSS1500	-1003	'ERBB3	receptor tyrosine-protein kinase erbB-3 precursor
GA	cg19323289	6.39E-06	0.002530226	FDR0.01	11	19463903	'NAV2	neuron navigator 2 isoform 3	Body	64873	'NAV2-AS5	NAV2 antisense RNA 5
GA	cg19327213	2.68E-07	0.000251907	FDR0.01	19	47988783	'KPTN;NAPA-AS1;NAPA	KICSTOR complex protein kaptin;NAPA antisense RNA 1;alpha-soluble NSF at	TSS1500;TSS1500;TSS1500;Bod	1245	'NAPA-AS1	NAPA antisense RNA 1
GA	cg19355069	2.28E-05	0.006086474	FDR0.01	12	125028302	'NCOR2	nuclear receptor corepressor 2	Body;Body;Body	23856	'NCOR2	nuclear receptor corepressor 2
GA	cg19359550	4.92E-07	0.000396481	FDR0.01	12	5137028	'			-16016	'KCNA5	potassium voltage-gated channel subfamily A member 5
GA	cg19381811	9.62E-07	0.000653562	FDR0.01	3	49851713	'UBA7	ubiquitin-like modifier-activating enzyme 7	TSS1500	-327	'UBA7	ubiquitin-like modifier-activating enzyme 7
GA	cg19401033	4.30E-07	0.000363063	FDR0.01	3	107148372	'LINC01990	long intergenic non-protein coding RNA 1990	TSS1500	-1404	'LINC01990	long intergenic non-protein coding RNA 1990
GA	cg19416570	9.51E-06	0.003315348	FDR0.01	19	58715677	'ZNF274	neurotrophin receptor-interacting factor homolog isoform d	Body;Body;Body;Body	21250	'ZNF274	neurotrophin receptor-interacting factor homolog isoform d
GA	cg19426731	2.67E-06	0.001336904	FDR0.01	19	49992527	'FLT3LG;RPL13A;SNORD32A;SNORD33;SNORD34;SN	fms related tyrosine kinase 3 ligand precursor;60S ribosomal protein L13a;sn	TES5000;TES5000;TES5000;TES	-695	'SNORD32A	small nucleolar RNA, C/D box 32A
GA	cg19429405	2.96E-08	4.79E-05	BF|FDR0.01	10	73497609	'CDH23;C10orf105	cadherin-23 precursor;uncharacterized protein C10orf105	Body;TSS200	-2	'C10orf105	uncharacterized protein C10orf105
GA	cg19439123	8.53E-07	0.000597035	FDR0.01	17	17687621	'RAI1;SMCR5	decapping nuclease;Smith-Magenis syndrome chromosome region, candidat	Body;TSS5000	-4778	'SMCR5	Smith-Magenis syndrome chromosome region, candidate 5
GA	cg19442415	1.94E-08	3.33E-05	BF|FDR0.01	11	116567334	'			57196	'LINC02702	long intergenic non-protein coding RNA 2702
GA	cg19448292	1.05E-07	0.000127695	BF|FDR0.01	20	35504064	'TLDC2	TLD domain-containing protein 2	TSS1500;TSS1500	-459	'TLDC2	TLD domain-containing protein 2
GA	cg19449067	1.13E-05	0.003748447	FDR0.01	6	152011103	'ESR1	estrogen receptor	TSS1500	-527	'ESR1	estrogen receptor
GA	cg19483007	7.81E-07	0.000560334	FDR0.01	3	149327651	'WWTR1	WW domain-containing transcription regulator protein 1	Body;Body;Body;Body	48161	'WWTR1	WW domain-containing transcription regulator protein 1
GA	cg19483465	4.01E-05	0.008741223	FDR0.01	12	132896939	'GALNT9	polypeptide N-acetylgalactosaminyltransferase 9	Body	9236	'GALNT9	polypeptide N-acetylgalactosaminyltransferase 9
GA	cg19499884	1.62E-09	5.93E-06	BF|FDR0.01	10	102760724	'LZTS2	leucine zipper putative tumor suppressor 2	Body;Body;Body;TSS5000	1160	'LZTS2	leucine zipper putative tumor suppressor 2
GA	cg19540316	2.39E-05	0.006266982	FDR0.01	13	113622750	'MCF2L-AS1;MCF2L	MCF2L antisense RNA 1;guanine nucleotide exchange factor DBS	Body;TSS1500;TSS1500	202	'MCF2L-AS1	MCF2L antisense RNA 1
GA	cg19548859	3.66E-05	0.008278686	FDR0.01	10	11239482	'CELF2	CUGBP Elav-like family member 2	Body;Body;Body;Body;Body;Bo	32490	'CELF2	CUGBP Elav-like family member 2
GA	cg19576697	6.27E-09	1.56E-05	BF|FDR0.01	5	176837920	'F12	coagulation factor XII precursor	TSS1500	-1343	'F12	coagulation factor XII precursor
GA	cg19577671	4.89E-05	0.009937236	FDR0.01	19	42782524	'CIC	protein capicua homolog isoform CIC-S	Body	-6292	'CIC	protein capicua homolog isoform CIC-S
GA	cg19595760	4.32E-06	0.001899024	FDR0.01	1	25948511	'MAN1C1	mannosyl-oligosaccharide 1,2-alpha-mannosidase IC isoform 1	Body;Body	4553	'MAN1C1	mannosyl-oligosaccharide 1,2-alpha-mannosidase IC isoform 1
GA	cg19607845	6.70E-06	0.002608642	FDR0.01	6	52934457	'FBXO9	F-box only protein 9	Body;Body;TSS1500	-1313	'FBXO9	F-box only protein 9
GA	cg19619003	2.89E-06	0.001427528	FDR0.01	1	41846758	'FOXO6	forkhead box protein O6	Body	-5417	'FOXO6-AS1	FOXO6 antisense RNA 1
GA	cg19619576	8.60E-06	0.003100698	FDR0.01	17	17718525	'RAI1;SREBF1;MIR6777;MIR33B	decapping nuclease;sterol regulatory element-binding protein 1;microRNA 6	TES5000;Body;Body;Body;TSS5	-1280	'MIR33B	microRNA 33b
GA	cg19622662	8.84E-07	0.000612079	FDR0.01	1	167090972	'DUSP27	inactive dual specificity phosphatase 27	Body	27661	'DUSP27	inactive dual specificity phosphatase 27
GA	cg19653589	3.59E-08	5.52E-05	BF|FDR0.01	19	2614177	'GNG7	guanine nucleotide-binding protein G(I)/G(S)/G(O) subunit gamma-7	Body	-16535	'MIR7850	microRNA 7850
GA	cg19687075	1.61E-07	0.000171077	FDR0.01	9	139428384	'NOTCH1	neurogenic locus notch homolog protein 1	Body	12116	'NOTCH1	neurogenic locus notch homolog protein 1
GA	cg19695266	1.04E-05	0.003510259	FDR0.01	1	1241672	'ACAP3;SNORD167;PUSL1	arf-GAP with coiled-coil, ANK repeat and PH domain-containing protein 3;sm	Body;TSS1500;TSS5000;TSS500	-1480	'SNORD167	small nucleolar RNA, C/D box 167
GA	cg19708901	4.64E-05	0.0095745	FDR0.01	1	180882655	'KIAA1614	uncharacterized protein KIAA1614	Body	623	'KIAA1614	uncharacterized protein KIAA1614
GA	cg19713429	6.54E-07	0.000490853	FDR0.01	1	19810690	'CAPZB	F-actin-capping protein subunit beta	1stExon;Body;Body;Body;Body	99	'CAPZB	F-actin-capping protein subunit beta
GA	cg19715094	1.79E-06	0.001025988	FDR0.01	11	118402540	'KMT2A;LOC101929089;TTC36;TMEM25	histone-lysine N-methyltransferase 2A isoform 2 precursor;;tetratricopeptid	TES5000;TSS1500;TSS1500;TSS	634	'TMEM25	transmembrane protein 25 precursor
GA	cg19726321	1.51E-06	0.000908881	FDR0.01	2	37909996	'			-9904	'CDC42EP3	cdc42 effector protein 3
GA	cg19727396	3.33E-05	0.007729903	FDR0.01	1	230415185	'GALNT2	polypeptide N-acetylgalactosaminyltransferase 2 precursor	Body;Body	146893	'PGBD5	piggyBac transposable element-derived protein 5
GA	cg19744173	4.09E-07	0.000349502	FDR0.01	2	112913178	'FBLN7	fibulin-7 precursor	Body;Body	17217	'FBLN7	fibulin-7 precursor
GA	cg19760965	3.16E-10	1.81E-06	BF|FDR0.01	3	194868843	'XXYLT1;XXYLT1-AS2	xyloside xylosyltransferase 1;XXYLT1 antisense RNA 2	Body;Body;Body;Body	244	'XXYLT1-AS2	XXYLT1 antisense RNA 2
GA	cg19776942	1.55E-08	2.92E-05	BF|FDR0.01	1	32714164	'FAM167B;LCK	protein FAM167B;tyrosine-protein kinase Lck	Body;TSS5000;TSS5000	1342	'FAM167B	protein FAM167B
GA	cg19784880	1.11E-09	4.44E-06	BF|FDR0.01	5	178957344	'			-20210	'RUFY1	RUN and FYVE domain-containing protein 1
GA	cg19789466	4.30E-07	0.000363063	FDR0.01	12	113344923	'OAS1	2'-5'-oligoadenylate synthase 1	1stExon;1stExon;1stExon;1stEx	157	'OAS1	2'-5'-oligoadenylate synthase 1
GA	cg19801120	5.36E-08	7.40E-05	BF|FDR0.01	6	53530570	'KLHL31	kelch-like protein 31	TSS200	-16	'KLHL31	kelch-like protein 31
GA	cg19802390	5.61E-09	1.44E-05	BF|FDR0.01	1	2840177	'			-97853	'ACTRT2	actin-related protein T2
GA	cg19848082	9.15E-06	0.003228567	FDR0.01	2	74232673	'TET3	methylcytosine dioxygenase TET3 isoform 2	Body;Body	2855	'TET3	methylcytosine dioxygenase TET3 isoform 2
GA	cg19851816	9.33E-12	1.54E-07	BF|FDR0.01	22	50657907	'SELENOO;TUBGCP6	selenoprotein O precursor;gamma-tubulin complex component 6	TES5000;Body	18468	'SELENOO	selenoprotein O precursor
GA	cg19863740	2.08E-05	0.005754992	FDR0.01	22	44576869	'PARVG	gamma-parvin	Body;5'UTR	65	'PARVG	gamma-parvin
GA	cg19915505	2.68E-05	0.006750105	FDR0.01	3	32147840	'GPD1L	glycerol-3-phosphate dehydrogenase 1-like protein	TSS200	-162	'GPD1L	glycerol-3-phosphate dehydrogenase 1-like protein
GA	cg19917989	1.55E-05	0.004703133	FDR0.01	6	14925093	'			-321206	'JARID2	protein Jumonji
GA	cg19950606	9.01E-08	0.000113076	BF|FDR0.01	17	76121276	'TMC6	transmembrane channel-like protein 6	Body;Body;Body	3599	'TMC6	transmembrane channel-like protein 6
GA	cg19987060	3.01E-05	0.007242909	FDR0.01	6	52530180	'LOC730101	uncharacterized LOC730101	Body;Body	982	'LOC730101	uncharacterized LOC730101
GA	cg20012607	1.22E-06	0.000777858	FDR0.01	13	100085455	'			66105	'LINC01232	long intergenic non-protein coding RNA 1232
GA	cg20025977	1.85E-05	0.005334084	FDR0.01	4	1579814	'			105904	'FAM53A	protein FAM53A isoform 1
GA	cg20038070	1.03E-05	0.003507333	FDR0.01	4	4482826	'STX18;STX18-IT1	syntaxin-18;STX18 intronic transcript 1	Body;Body;Body;Body;Body	601	'STX18-IT1	STX18 intronic transcript 1
GA	cg20072118	4.51E-11	4.48E-07	BF|FDR0.01	12	52653637	'KRT87P	keratin 87 pseudogene	TSS1500	-1345	'KRT87P	keratin 87 pseudogene
GA	cg20090859	1.54E-08	2.92E-05	BF|FDR0.01	8	80590435	'			67084	'STMN2	stathmin-2
GA	cg20100768	3.39E-05	0.007814581	FDR0.01	13	80706924	'			121713	'LINC01080	long intergenic non-protein coding RNA 1080
GA	cg20107668	1.50E-07	0.000163644	FDR0.01	13	113624471	'MCF2L-AS1;MCF2L	MCF2L antisense RNA 1;guanine nucleotide exchange factor DBS	TSS5000;Body;5'UTR	598	'MCF2L	guanine nucleotide exchange factor DBS
GA	cg20117260	4.52E-05	0.009411417	FDR0.01	19	41937434	'B3GNT8;DMAC2	UDP-GlcNAc:betaGal beta-1,3-N-acetylglucosaminyltransferase 8 precursor;	TSS5000;3'UTR;3'UTR;3'UTR;3'	-2799	'B3GNT8	UDP-GlcNAc:betaGal beta-1,3-N-acetylglucosaminyltransferase 8 precursor
GA	cg20122518	2.51E-05	0.00646645	FDR0.01	7	810958	'DNAAF5	dynein assembly factor 5, axonemal	Body;Body	-43645	'PRKAR1B	cAMP-dependent protein kinase type I-beta regulatory subunit
GA	cg20139800	2.18E-05	0.005904872	FDR0.01	6	29599178	'GABBR1	gamma-aminobutyric acid type B receptor subunit 1 precursor	TSS5000;5'UTR;Body;Body	1734	'GABBR1	gamma-aminobutyric acid type B receptor subunit 1 precursor
GA	cg20142309	1.16E-05	0.003821264	FDR0.01	17	27507917	'MYO18A	unconventional myosin-XVIIIa isoform e	TSS5000;TSS1500;TSS1500;TSS	-501	'MYO18A	unconventional myosin-XVIIIa isoform e
GA	cg20149937	4.24E-06	0.00187778	FDR0.01	19	3465709	'NFIC	nuclear factor 1 C-type	3'UTR;3'UTR;3'UTR;3'UTR;3'UT	14814	'SMIM24	small integral membrane protein 24 precursor
GA	cg20156116	1.52E-05	0.004642221	FDR0.01	1	156699437	'ISG20L2;RRNAD1	interferon-stimulated 20 kDa exonuclease-like 2;protein RRNAD1	TSS1500;TSS1500;TSS1500;TSS	-879	'ISG20L2	interferon-stimulated 20 kDa exonuclease-like 2
GA	cg20160197	4.41E-05	0.009269461	FDR0.01	4	1222037	'CTBP1	C-terminal-binding protein 1	Body;Body	18130	'CTBP1-AS	CTBP1 antisense RNA
GA	cg20167216	1.81E-05	0.005259275	FDR0.01	7	142353463	'			-20667	'MTRNR2L6	humanin-like 6
GA	cg20181887	4.18E-05	0.008952664	FDR0.01	12	123753272	'CDK2AP1	cyclin-dependent kinase 2-associated protein 1	TSS1500;Body;Body;Body;Body	-471	'CDK2AP1	cyclin-dependent kinase 2-associated protein 1
GA	cg20229014	1.01E-08	2.16E-05	BF|FDR0.01	8	134529378	'ST3GAL1	CMP-N-acetylneuraminate-beta-galactosamide- alpha-2,3-sialyltransferase 1	Body;Body	54752	'ST3GAL1	CMP-N-acetylneuraminate-beta-galactosamide- alpha-2,3-sialyltransferase 1
GA	cg20239639	1.97E-06	0.001084302	FDR0.01	1	32739493	'LCK	tyrosine-protein kinase Lck	Body;Body;TSS1500	-218	'LCK	tyrosine-protein kinase Lck
GA	cg20248822	1.75E-05	0.005129309	FDR0.01	1	19922867	'LOC105378614;MICOS10;MICOS10-NBL1	uncharacterized LOC105378614;MICOS complex subunit MIC10 isoform c;C1	Body;TSS1500;TSS1500;TSS150	459	'LOC105378614	uncharacterized LOC105378614
GA	cg20249277	1.14E-05	0.003760645	FDR0.01	7	112120877	'IFRD1;LSMEM1	interferon-related developmental regulator 1;leucine-rich single-pass memb	TES5000;TES5000;TES5000;TES	-30	'LSMEM1	leucine-rich single-pass membrane protein 1
GA	cg20253872	3.81E-09	1.07E-05	BF|FDR0.01	1	110166571	'AMPD2	AMP deaminase 2	Body;Body;Body;Body;TSS200	-38	'AMPD2	AMP deaminase 2
GA	cg20272935	1.86E-07	0.000191392	FDR0.01	11	67765720	'UNC93B1	protein unc-93 homolog B1	Body	5847	'UNC93B1	protein unc-93 homolog B1
GA	cg20285026	4.65E-05	0.009589947	FDR0.01	1	1983774	'LOC105378591;PRKCZ	uncharacterized LOC105378591;protein kinase C zeta type	TSS5000;Body	1925	'PRKCZ	protein kinase C zeta type
GA	cg20288630	4.66E-06	0.002004112	FDR0.01	4	120550423	'PDE5A	cGMP-specific 3',5'-cyclic phosphodiesterase	TSS1500;TSS5000;TSS1500	-464	'PDE5A	cGMP-specific 3',5'-cyclic phosphodiesterase
GA	cg20294319	2.49E-06	0.001283701	FDR0.01	13	47253842	'LRCH1	leucine-rich repeat and calponin homology domain-containing protein 1 isof	Body;Body;Body	117454	'ESD	S-formylglutathione hydrolase
GA	cg20301308	1.04E-11	1.60E-07	BF|FDR0.01	1	65534742	'JAK1	tyrosine-protein kinase JAK1	TSS1500;TSS1500;TSS1500	-1313	'JAK1	tyrosine-protein kinase JAK1
GA	cg20309353	3.45E-06	0.001615284	FDR0.01	17	18089940	'ALKBH5	RNA demethylase ALKBH5	Body	2799	'ALKBH5	RNA demethylase ALKBH5
GA	cg20319926	1.82E-07	0.000188165	FDR0.01	16	685686	'WFIKKN1;METTL26;LOC100287175	WAP, Kazal, immunoglobulin, Kunitz and NTR domain-containing protein 1 p	TES5000;Body;Body;Body;Body	619	'METTL26	methyltransferase-like 26
GA	cg20347666	1.38E-05	0.004342185	FDR0.01	7	137505606	'DGKI	diacylglycerol kinase iota isoform 1	Body;Body;Body;Body	25472	'DGKI	diacylglycerol kinase iota isoform 1
GA	cg20360286	1.39E-05	0.004366005	FDR0.01	2	150222839	'LYPD6	ly6/PLAUR domain-containing protein 6 precursor	Body;Body	35739	'LYPD6	ly6/PLAUR domain-containing protein 6 precursor
GA	cg20367304	2.26E-08	3.79E-05	BF|FDR0.01	4	154391944	'TMEM131L	transmembrane protein 131-like isoform 2 precursor	Body;Body	4433	'TMEM131L	transmembrane protein 131-like isoform 2 precursor
GA	cg20392842	4.00E-05	0.008741223	FDR0.01	6	32909003	'HLA-DMB	HLA class II histocompatibility antigen, DM beta chain precursor	TSS200	-198	'HLA-DMB	HLA class II histocompatibility antigen, DM beta chain precursor

Trait	#cpg	p	fdr	significance	chr	pos.hg19	gene.Illumina	gene.description	locations	dist2TSS	gene.nearest	gene.description
GA	cg20429981	1.51E-07	0.000164394	FDR0.01	1	1609972	'SLC35E2B	solute carrier family 35 member E2B	Body;Body	14262	'SLC35E2B	solute carrier family 35 member E2B
GA	cg20457732	2.89E-05	0.007103144	FDR0.01	1	71172486	'LINC01788	long intergenic non-protein coding RNA 1788	Body	351	'LINC01788	long intergenic non-protein coding RNA 1788
GA	cg20460227	3.81E-06	0.001745616	FDR0.01	2	120452632	'TMEM177	transmembrane protein 177	Body	15856	'TMEM177	transmembrane protein 177
GA	cg20460366	4.40E-05	0.009257985	FDR0.01	20	44934032	'CDH22	cadherin-22 precursor	Body	3105	'CDH22	cadherin-22 precursor
GA	cg20462883	4.60E-08	6.55E-05	BF|FDR0.01	12	122461621	'BCL7A	B-cell CLL/lymphoma 7 protein family member A isoform a	Body;Body	1832	'BCL7A	B-cell CLL/lymphoma 7 protein family member A isoform a
GA	cg20519944	2.63E-05	0.006674668	FDR0.01	7	157412886	'PTPRN2	receptor-type tyrosine-protein phosphatase N2 isoform 4 precursor	Body;Body;Body;Body;Body	-45772	'MIR153-2	microRNA 153-2
GA	cg20520115	2.92E-06	0.001432923	FDR0.01	2	75063838	'HK2	hexokinase-2	Body;Body	1542	'HK2	hexokinase-2
GA	cg20534570	7.26E-06	0.002754318	FDR0.01	22	41763093	'TEF	transcription factor VBP	TSS1500	-243	'TEF	transcription factor VBP
GA	cg20556503	1.72E-05	0.005069174	FDR0.01	21	46349095	'ITGB2;ITGB2-AS1;LINC01547	integrin beta-2 precursor;ITGB2 antisense RNA 1;long intergenic non-protein	TSS1500;Body;Body;Body;Body	-365	'ITGB2	integrin beta-2 precursor
GA	cg20556951	4.27E-05	0.009067438	FDR0.01	17	45934112	'SP6	transcription factor Sp6	TSS1500	-1049	'SP6	transcription factor Sp6
GA	cg20566766	1.87E-05	0.00535964	FDR0.01	5	138723816	'SLC23A1;MZB1;PROB1	solute carrier family 23 member 1;marginal zone B- and B1-cell-specific prote	TSS5000;TSS5000;Body;TES500	1786	'MZB1	marginal zone B- and B1-cell-specific protein precursor
GA	cg20575191	4.24E-05	0.009044808	FDR0.01	15	77324607	'PSTPIP1	proline-serine-threonine phosphatase-interacting protein 1	Body;Body;Body;Body;Body	37141	'PSTPIP1	proline-serine-threonine phosphatase-interacting protein 1
GA	cg20591407	5.72E-06	0.002336898	FDR0.01	12	1642663	'LOC107984507	uncharacterized LOC107984507	TSS1500	-1305	'LOC107984507	uncharacterized LOC107984507
GA	cg20600379	9.64E-06	0.003340381	FDR0.01	6	32909282	'HLA-DMB	HLA class II histocompatibility antigen, DM beta chain precursor	TSS1500	-477	'HLA-DMB	HLA class II histocompatibility antigen, DM beta chain precursor
GA	cg20600850	5.84E-07	0.000450038	FDR0.01	12	6712515	'CHD4	chromodomain-helicase-DNA-binding protein 4	Body;Body;Body	3102	'CHD4	chromodomain-helicase-DNA-binding protein 4
GA	cg20630655	2.55E-06	0.001299485	FDR0.01	15	75918700	'SNUPN	snurportin-1	TSS1500;5'UTR;TSS1500	19	'SNUPN	snurportin-1
GA	cg20663831	2.52E-05	0.006504809	FDR0.01	7	150383154	'GIMAP2	GTPase IMAP family member 2	Body	361	'GIMAP2	GTPase IMAP family member 2
GA	cg20670923	2.43E-06	0.001264441	FDR0.01	18	9915771	'VAPA	vesicle-associated membrane protein-associated protein A	Body;Body	1759	'VAPA	vesicle-associated membrane protein-associated protein A
GA	cg20701531	6.51E-07	0.000489438	FDR0.01	2	121583695	'GLI2	zinc finger protein GLI2	Body;Body	33839	'GLI2	zinc finger protein GLI2
GA	cg20716774	3.83E-05	0.00852482	FDR0.01	5	10601638	'ANKRD33B	ankyrin repeat domain-containing protein 33B	Body	37457	'ANKRD33B	ankyrin repeat domain-containing protein 33B
GA	cg20721135	6.36E-07	0.000481893	FDR0.01	13	114861488	'RASA3	ras GTPase-activating protein 3	Body;Body	-18058	'RASA3	ras GTPase-activating protein 3
GA	cg20765408	4.43E-07	0.000368854	FDR0.01	13	25085669	'PARP4	protein mono-ADP-ribosyltransferase PARP4	Body	1247	'PARP4	protein mono-ADP-ribosyltransferase PARP4
GA	cg20773127	3.41E-06	0.001604402	FDR0.01	4	111397155	'ENPEP	glutamyl aminopeptidase	TSS200	-155	'ENPEP	glutamyl aminopeptidase
GA	cg20773320	1.90E-05	0.005409077	FDR0.01	5	179048967	'HNRNPH1	heterogeneous nuclear ribonucleoprotein H	Body;Body;Body;Body;Body;Bo	1435	'HNRNPH1	heterogeneous nuclear ribonucleoprotein H
GA	cg20777390	5.96E-09	1.49E-05	BF|FDR0.01	12	123587316	'PITPNM2	membrane-associated phosphatidylinositol transfer protein 2	Body;Body	7659	'PITPNM2	membrane-associated phosphatidylinositol transfer protein 2
GA	cg20792436	1.63E-05	0.00486214	FDR0.01	17	79972570	'ASPSCR1;CENPX	tether containing UBX domain for GLUT4 isoform 1;centromere protein X	Body;Body;Body;Body;TES5000	8184	'CENPX	centromere protein X
GA	cg20793071	3.51E-12	6.45E-08	BF|FDR0.01	1	54520450	'TMEM59;TCEANC2;MIR4781	transmembrane protein 59 precursor;transcription elongation factor A N-ter	TSS5000;TSS5000;TSS5000;TSS1	699	'MIR4781	microRNA 4781
GA	cg20797674	5.59E-07	0.000435319	FDR0.01	1	35656325	'SFPQ	splicing factor, proline- and glutamine-rich	Body;Body;Body	2421	'SFPQ	splicing factor, proline- and glutamine-rich
GA	cg20803796	1.15E-06	0.000743425	FDR0.01	8	145103842	'SPATC1;SMPD5;OPLAH	speriolin;sphingomyelin phosphodiesterase 5 (pseudogene);5-oxoprolinase	TES5000;TES5000;TSS1500;TES	-456	'SMPD5	sphingomyelin phosphodiesterase 5 (pseudogene)
GA	cg20824977	3.11E-09	9.33E-06	BF|FDR0.01	15	59978600	'BNIP2	BCL2/adenovirus E1B 19 kDa protein-interacting protein 2	Body;Body;Body;Body;Body;Bo	2525	'BNIP2	BCL2/adenovirus E1B 19 kDa protein-interacting protein 2
GA	cg20837626	7.08E-06	0.002707542	FDR0.01	2	54195596	'PSME4;ACYP2	proteasome activator complex subunit 4;acylphosphatase-2	Body;TSS5000;TSS5000	2534	'PSME4	proteasome activator complex subunit 4
GA	cg20850016	2.27E-05	0.006070677	FDR0.01	3	5165254	'ARL8B	ADP-ribosylation factor-like protein 8B	Body	1278	'ARL8B	ADP-ribosylation factor-like protein 8B
GA	cg20855296	3.83E-05	0.00852482	FDR0.01	17	16255560	'CENPV	centromere protein V	Body	1223	'CENPV	centromere protein V
GA	cg20870559	1.11E-05	0.003707138	FDR0.01	12	113416518	'OAS2	2'-5'-oligoadenylate synthase 2	1stExon;1stExon;1stExon	195	'OAS2	2'-5'-oligoadenylate synthase 2
GA	cg20885815	1.77E-06	0.00101961	FDR0.01	2	27478320	'SLC30A3	zinc transporter 3	Body;Body;Body;Body	7595	'SLC30A3	zinc transporter 3
GA	cg20889322	3.67E-09	1.05E-05	BF|FDR0.01	11	65196622	'NEAT1	nuclear paraspeckle assembly transcript 1	TES5000;Body	6354	'NEAT1	nuclear paraspeckle assembly transcript 1
GA	cg20893956	5.90E-07	0.000453856	FDR0.01	6	152126736	'ESR1	estrogen receptor	Body;TSS200;TSS200;TSS200;TS	-71	'ESR1	estrogen receptor
GA	cg20899625	1.20E-05	0.003919522	FDR0.01	3	126721131	'PLXNA1	plexin-A1 precursor	Body	13695	'PLXNA1	plexin-A1 precursor
GA	cg20902783	2.96E-05	0.007183669	FDR0.01	14	21494071	'NDRG2;MIR6717;TPPP2	protein NDRG2;microRNA 6717;tubulin polymerization-promoting protein fa	TSS1500;TSS1500;TSS1500;TSS1	-136	'NDRG2	protein NDRG2
GA	cg20912066	4.12E-05	0.008874072	FDR0.01	11	107436729	'ALKBH8	alkylated DNA repair protein alkB homolog 8 isoform 1	TSS1500;TSS1500	-268	'ALKBH8	alkylated DNA repair protein alkB homolog 8 isoform 1
GA	cg20914572	1.24E-05	0.004023256	FDR0.01	6	32119874	'PRRT1;LOC100507547;PPT2;PPT2-EGFL8	proline-rich transmembrane protein 1;uncharacterized LOC100507547;lysos	1stExon;TSS200;TES5000;TES50	-158	'PRRT1	proline-rich transmembrane protein 1
GA	cg20926021	8.36E-07	0.000589047	FDR0.01	4	154169814	'TRIM2	tripartite motif-containing protein 2	Body;Body;Body;Body;Body;Bo	-8696	'TRIM2	tripartite motif-containing protein 2
GA	cg20930815	1.42E-06	0.000868737	FDR0.01	11	9099393	'SCUBE2	signal peptide, CUB and EGF-like domain-containing protein 2 isoform 1 prec	Body;Body;Body;Body	12533	'MIR5691	microRNA 5691
GA	cg20934105	2.57E-09	8.26E-06	BF|FDR0.01	14	93118717	'RIN3	ras and Rab interactor 3 isoform 2	Body;Body	76139	'RIN3	ras and Rab interactor 3 isoform 2
GA	cg20945261	2.04E-05	0.00567018	FDR0.01	3	13460618	'NUP210	nuclear pore membrane glycoprotein 210 precursor	Body	1204	'NUP210	nuclear pore membrane glycoprotein 210 precursor
GA	cg21002651	2.38E-06	0.001249385	FDR0.01	11	104905889	'CASP1	caspase-1	TSS200;TSS200;TSS200;TSS200;	-5	'CASP1	caspase-1
GA	cg21022247	1.14E-05	0.003769925	FDR0.01	12	51718310	'BIN2;CELA1	bridging integrator 2;chymotrypsin-like elastase family member 1 precursor	TSS1500;TSS1500;TSS1500;TSS1	142	'BIN2	bridging integrator 2
GA	cg21063275	1.11E-08	2.31E-05	BF|FDR0.01	15	22466195	'IGHV1OR15-3	immunoglobulin heavy variable 1/OR15-3 (pseudogene)	Body	352	'IGHV1OR15-3	immunoglobulin heavy variable 1/OR15-3 (pseudogene)
GA	cg21069019	4.06E-05	0.008802751	FDR0.01	2	6636278	'			-120724	'LINC01247	long intergenic non-protein coding RNA 1247
GA	cg21081878	1.63E-06	0.000960311	FDR0.01	21	38334730	'HLCS	biotin--protein ligase isoform 2	TSS200;Body;Body;Body;Body;B	-72	'HLCS	biotin--protein ligase isoform 2
GA	cg21105477	6.88E-07	0.000511349	FDR0.01	5	133560149	'PPP2CA;MIR3661	serine/threonine-protein phosphatase 2A catalytic subunit alpha isoform;mi	Body;Body;Body;TSS1500	1096	'PPP2CA	serine/threonine-protein phosphatase 2A catalytic subunit alpha isoform
GA	cg21105928	5.90E-06	0.002389782	FDR0.01	15	40143956	'GPR176	G-protein coupled receptor 176	Body;Body	-22479	'GPR176	G-protein coupled receptor 176
GA	cg21121843	1.19E-05	0.003902943	FDR0.01	4	3203982	'HTT	huntingtin	Body	-42113	'MSANTD1	myb/SANT-like DNA-binding domain-containing protein 1
GA	cg21132686	2.08E-08	3.51E-05	BF|FDR0.01	20	22795774	'			-129792	'LINC01747	long intergenic non-protein coding RNA 1747
GA	cg21139003	1.40E-05	0.00437772	FDR0.01	2	33173301	'LINC00486;LTBP1	long intergenic non-protein coding RNA 486;latent TGFbeta binding protein p	TES5000;Body	1282	'LTBP1	latent TGFbeta binding protein precursor
GA	cg21139150	8.03E-06	0.002961627	FDR0.01	21	46976175	'SLC19A1	reduced folate transporter	Body	6764	'SLC19A1	reduced folate transporter
GA	cg21142743	2.13E-06	0.001153858	FDR0.01	1	228272495	'ARF1	ADP-ribosylation factor 1	Body;Body;Body;Body	1645	'ARF1	ADP-ribosylation factor 1
GA	cg21146537	3.30E-12	6.41E-08	BF|FDR0.01	22	35695301	'HMGXB4;TOM1	HMG domain-containing protein 4;target of Myb protein 1	TES5000;TES5000;TES5000;5'U	34	'TOM1	target of Myb protein 1
GA	cg21147708	4.22E-05	0.009013544	FDR0.01	6	7611002	'SNRNP48	U11/U12 small nuclear ribonucleoprotein 48 kDa protein	3'UTR	20572	'SNRNP48	U11/U12 small nuclear ribonucleoprotein 48 kDa protein
GA	cg21157690	4.11E-09	1.14E-05	BF|FDR0.01	6	152126895	'ESR1	estrogen receptor	Body;5'UTR;5'UTR;5'UTR;TSS50	88	'ESR1	estrogen receptor
GA	cg21160842	3.72E-05	0.008371958	FDR0.01	11	32112091	'RCN1	reticulocalbin-1 precursor	TSS1500	-528	'RCN1	reticulocalbin-1 precursor
GA	cg21176629	2.91E-08	4.73E-05	BF|FDR0.01	8	67838106	'MCMDC2;SNHG6;SNORD87	minichromosome maintenance domain-containing protein 2 isoform c;small	TES5000;TSS1500;TSS1500;TSS1	-330	'SNHG6	small nucleolar RNA host gene 6
GA	cg21186955	1.12E-08	2.31E-05	BF|FDR0.01	7	100729412	'TRIM56	E3 ubiquitin-protein ligase TRIM56	Body	651	'TRIM56	E3 ubiquitin-protein ligase TRIM56
GA	cg21201657	4.23E-08	6.15E-05	BF|FDR0.01	19	5624574	'SAFB2;SAFB	scaffold attachment factor B2;scaffold attachment factor B1	TSS5000;Body;Body;Body;Body	1481	'SAFB	scaffold attachment factor B1
GA	cg21228270	8.86E-06	0.003154681	FDR0.01	9	133871033	'			-13527	'LAMC3	laminin subunit gamma-3 precursor
GA	cg21239798	4.30E-06	0.00189565	FDR0.01	5	111756360	'EPB41L4A;EPB41L4A-DT	band 4.1-like protein 4A;EPB41L4A divergent transcript	TSS1500;TSS1500;TSS1500;TSS1	1081	'EPB41L4A-DT	EPB41L4A divergent transcript
GA	cg21246872	2.01E-05	0.005613351	FDR0.01	17	38295894	'MSL1;CASC3	MSL1 protein;protein CASC3	TES5000;TES5000;TES5000;TSS1	-895	'CASC3	protein CASC3
GA	cg21251140	1.01E-05	0.003460796	FDR0.01	2	181544642	'			-12188	'SCHLAP1	SWI/SNF complex antagonist associated with prostate cancer 1
GA	cg21281009	2.76E-05	0.006862796	FDR0.01	18	14748298	'ANKRD30B	ankyrin repeat domain-containing protein 30B	5'UTR;Body	128	'ANKRD30B	ankyrin repeat domain-containing protein 30B
GA	cg21308575	7.70E-07	0.000555281	FDR0.01	19	6887544	'ADGRE1	adhesion G protein-coupled receptor E1 precursor	TSS200;TSS200;TSS200;TSS200;	-45	'ADGRE1	adhesion G protein-coupled receptor E1 precursor
GA	cg21321372	1.66E-05	0.004943295	FDR0.01	4	141264456	'SCOC;SCOC-AS1	short coiled-coil protein;SCOC antisense RNA 1	Body;Body;TSS200;TSS200;TSS2	-140	'SCOC	short coiled-coil protein
GA	cg21339822	4.22E-05	0.009017792	FDR0.01	12	101988803	'MYBPC1	myosin-binding protein C, slow-type isoform 5	5'UTR;5'UTR;5'UTR;5'UTR;5'UT	55	'MYBPC1	myosin-binding protein C, slow-type isoform 5
GA	cg21363348	7.41E-06	0.00279012	FDR0.01	7	134832221	'CYREN;TMEM140	cell cycle regulator of non-homologous end joining;transmembrane protein 1	Body;TSS1500	-602	'TMEM140	transmembrane protein 140 precursor
GA	cg21376733	1.31E-10	9.68E-07	BF|FDR0.01	12	54891636	'NCKAP1L	nck-associated protein 1-like	1stExon;TSS1500	101	'NCKAP1L	nck-associated protein 1-like
GA	cg21387281	3.61E-12	6.45E-08	BF|FDR0.01	20	61435439	'MRGBP;OGFR-AS1;OGFR	MRG/MORF4L-binding protein;OGFR antisense RNA 1;opioid growth factor r	TES5000;TES5000;Body;TSS150	-747	'OGFR	opioid growth factor receptor
GA	cg21394729	2.28E-07	0.000223049	FDR0.01	15	31316860	'TRPM1	transient receptor potential cation channel subfamily M member 1 isoform 1	Body;Body;Body	-33029	'MTMR10	myotubularin-related protein 10
GA	cg21404980	1.68E-06	0.000984026	FDR0.01	8	144599027	'ZC3H3	zinc finger CCCH domain-containing protein 3	Body	-10609	'SNORD149	small nucleolar RNA, C/D box 149
GA	cg21415227	1.27E-05	0.004088918	FDR0.01	11	13985911	'SPON1	spondin-1 precursor	Body	1642	'SPON1	spondin-1 precursor
GA	cg21472642	2.23E-09	7.46E-06	BF|FDR0.01	7	29233468	'CPVL;CHN2	probable serine carboxypeptidase CPVL isoform b precursor;beta-chimaerin	TSS5000;TSS5000;TSS5000;Bod	-652	'CHN2	beta-chimaerin
GA	cg21477075	1.35E-07	0.000151586	FDR0.01	6	168433436	'KIF25	kinesin-like protein KIF25 isoform 1	Body;Body	14884	'KIF25	kinesin-like protein KIF25 isoform 1
GA	cg21486510	1.76E-06	0.001016372	FDR0.01	2	7171998	'RNF144A	E3 ubiquitin-protein ligase RNF144A isoform 4	Body;Body;Body;Body;Body;Bo	46013	'LOC101929452	uncharacterized LOC101929452
GA	cg21491967	3.82E-05	0.008520719	FDR0.01	6	156276515	'			8585	'MIR1202	microRNA 1202
GA	cg21498471	4.11E-05	0.008870684	FDR0.01	19	33183713	'NUDT19	nucleoside diphosphate-linked moiety X motif 19	Body	847	'NUDT19	nucleoside diphosphate-linked moiety X motif 19
GA	cg21535931	3.27E-05	0.007618947	FDR0.01	8	145029603	'PLEC	plectin isoform 1a	TSS5000;TSS5000;Body;Body	-1601	'PLEC	plectin isoform 1a
GA	cg21545720	1.10E-08	2.31E-05	BF|FDR0.01	1	32405083	'PTP4A2	protein tyrosine phosphatase type IVA 3	TSS5000;TSS5000;TSS5000;TSS1	-1114	'PTP4A2	protein tyrosine phosphatase type IVA 3
GA	cg21583957	7.56E-06	0.002836307	FDR0.01	3	156782951	'			-16678	'LINC02029	long intergenic non-protein coding RNA 2029
GA	cg21585162	2.84E-07	0.000264593	FDR0.01	11	128566387	'FLI1;SENCR	Friend leukemia integration 1 transcription factor;smooth muscle and endot	Body;TSS1500;Body;Body;Body	-469	'SENCR	smooth muscle and endothelial cell enriched migration/differentiation-associated lncRNA
GA	cg21589273	2.09E-06	0.001138877	FDR0.01	3	113416452	'USF3	basic helix-loop-helix domain-containing protein USF3	TSS1500;TSS1500	-963	'USF3	basic helix-loop-helix domain-containing protein USF3

Trait	#cpg	p	fdr	significance	chr	pos.hg19	gene.Illumina	gene.description	locations	dist2TSS	gene.nearest	gene.description
GA	cg21610439	6.69E-07	0.000498397	FDR0.01	2	129251614	'			-175172	'HS6ST1	heparan-sulfate 6-O-sulfotransferase 1
GA	cg21618521	1.66E-05	0.004923978	FDR0.01	6	33245770	'RPS18;B3GALT4;WDR46	40S ribosomal protein S18;beta-1,3-galactosyltransferase 4;WD repeat-conta	TES5000;1stExon;TES5000;TES5	871	'B3GALT4	beta-1,3-galactosyltransferase 4
GA	cg21644740	3.83E-05	0.00852482	FDR0.01	6	29599248	'GABBR1	gamma-aminobutyric acid type B receptor subunit 1 precursor	TSS5000;5'UTR;Body;Body	1664	'GABBR1	gamma-aminobutyric acid type B receptor subunit 1 precursor
GA	cg21674813	2.45E-05	0.006370954	FDR0.01	2	220425073	'OBSL1	obscurin-like protein 1	Body;Body;TES5000	11188	'OBSL1	obscurin-like protein 1
GA	cg21686577	1.58E-06	0.000943112	FDR0.01	7	75909905	'SRRM3	serine/arginine repetitive matrix protein 3 isoform a	Body;Body	-22084	'HSPB1	heat shock protein beta-1
GA	cg21698310	2.28E-07	0.00022297	FDR0.01	17	48224718	'PPP1R9B	protein phosphatase 1, regulatory (inhibitor) subunit 9B	Body	3320	'PPP1R9B	protein phosphatase 1, regulatory (inhibitor) subunit 9B
GA	cg21710324	1.68E-05	0.004981885	FDR0.01	1	226069189	'TMEM63A;LEFTY1	CSC1-like protein 1;left-right determination factor 1 precursor	Body;TES5000	891	'TMEM63A	CSC1-like protein 1
GA	cg21788755	4.73E-08	6.72E-05	BF|FDR0.01	3	48508391	'ATRIP;ATRIP-TREX1;TREX1;SHISA5	ATR-interacting protein;ATRIP-TREX1 readthrough;three-prime repair exonu	TES5000;3'UTR;3'UTR;Body;3'U	763	'TREX1	three-prime repair exonuclease 1 isoform c
GA	cg21813747	4.24E-06	0.00187778	FDR0.01	1	19969931	'MICOS10-NBL1;NBL1	C1orf151-NBL1 read-through protein isoform a;Nbl1p	Body;Body;Body;Body;5'UTR;TS	29	'NBL1	Nbl1p
GA	cg21829239	3.84E-05	0.008536054	FDR0.01	1	160608184	'SLAMF1	signaling lymphocytic activation molecule precursor	Body;Body;Body;Body;Body	8650	'SLAMF1	signaling lymphocytic activation molecule precursor
GA	cg21890025	9.43E-08	0.000116886	BF|FDR0.01	7	1084594	'C7orf50;GPR146	uncharacterized protein C7orf50 isoform c;probable G-protein coupled recep	Body;Body;Body;Body;Body;Bo	413	'GPR146	probable G-protein coupled receptor 146
GA	cg21892295	3.97E-05	0.008705032	FDR0.01	12	121157589	'UNC119B;MIR4700	protein unc-119 homolog B;microRNA 4700	Body;TSS5000	-3406	'MIR4700	microRNA 4700
GA	cg21892720	3.81E-06	0.001745616	FDR0.01	19	54877827	'LAIR1	leukocyte-associated immunoglobulin-like receptor 1 isoform g	TSS1500;TSS1500;TSS1500;TSS1	-1289	'LAIR1	leukocyte-associated immunoglobulin-like receptor 1 isoform g
GA	cg21932231	9.41E-08	0.000116886	BF|FDR0.01	2	36668864	'CRIM1	cysteine-rich motor neuron 1 protein precursor	Body	85944	'CRIM1	cysteine-rich motor neuron 1 protein precursor
GA	cg21936552	1.22E-07	0.000140508	FDR0.01	17	79386428	'BAHCC1	BAH and coiled-coil domain-containing protein 1 isoform 1	Body	-11850	'MIR4740	microRNA 4740
GA	cg21937377	6.39E-08	8.51E-05	BF|FDR0.01	3	194868750	'XXYLT1;XXYLT1-AS2	xyloside xylosyltransferase 1;XXYLT1 antisense RNA 2	Body;Body;Body;Body	151	'XXYLT1-AS2	XXYLT1 antisense RNA 2
GA	cg21941821	7.75E-06	0.002879728	FDR0.01	22	39712633	'RPL3;SNORD83B;SNORD83A;SNORD139;SNORD43	ribosomal protein L3;small nucleolar RNA, C/D box 83B;small nucleolar RNA,	Body;Body;TSS5000;TSS1500;TI	268	'SNORD139	small nucleolar RNA, C/D box 139
GA	cg21946006	6.45E-06	0.002544626	FDR0.01	2	8817010	'ID2-AS1	ID2 antisense RNA 1	Body;TES5000	4981	'ID2-AS1	ID2 antisense RNA 1
GA	cg21990144	2.78E-07	0.000260226	FDR0.01	2	201726666	'CLK1	dual specificity protein kinase CLK1	Body;Body;Body;Body	2618	'CLK1	dual specificity protein kinase CLK1
GA	cg22000050	3.25E-05	0.00760174	FDR0.01	13	53425961	'PCDH8	protocadherin-8 precursor	TSS5000;TSS5000	-3186	'PCDH8	protocadherin-8 precursor
GA	cg22000727	6.19E-11	5.82E-07	BF|FDR0.01	6	3459199	'SLC22A23;LOC643327	solute carrier family 22 member 23;uncharacterized LOC643327	TSS5000;TSS5000;Body	-2406	'SLC22A23	solute carrier family 22 member 23
GA	cg22001211	6.43E-07	0.000485327	FDR0.01	2	61766532	'XPO1	exportin-1	TSS1500	-1041	'XPO1	exportin-1
GA	cg22026450	4.91E-05	0.00994754	FDR0.01	1	145476687	'ANKRD34A;LIX1L	ankyrin repeat domain-containing protein 34A;LIX1-like protein	TES5000;TSS1500	-381	'LIX1L	LIX1-like protein
GA	cg22062555	2.80E-05	0.006946891	FDR0.01	19	45664251	'NKPD1;TRAPPC6A	NTPase KAP family P-loop domain-containing protein 1;trafficking protein pa	TSS1500;TES5000;TES5000;TES	-843	'NKPD1	NTPase KAP family P-loop domain-containing protein 1
GA	cg22065124	1.31E-06	0.000820198	FDR0.01	13	73649955	'KLF5	Krueppel-like factor 5	Body;Body	16813	'KLF5	Krueppel-like factor 5
GA	cg22068629	5.49E-13	1.75E-08	BF|FDR0.01	19	2446633	'LMNB2	lamin-B2	Body	10324	'LMNB2	lamin-B2
GA	cg22084563	4.19E-05	0.008985584	FDR0.01	17	7255046	'ACAP1;KCTD11;TMEM95	arf-GAP with coiled-coil, ANK repeat and PH domain-containing protein 1;BT	TES5000;TSS200;TSS1500;TSS5	-161	'KCTD11	BTB/POZ domain-containing protein KCTD11
GA	cg22102703	2.43E-05	0.006333662	FDR0.01	21	43346284	'C2CD2	C2 domain-containing protein 2	Body;Body	528	'C2CD2	C2 domain-containing protein 2
GA	cg22123784	4.11E-05	0.008873918	FDR0.01	8	144437507	'TOP1MT	DNA topoisomerase I, mitochondrial isoform 1 precursor	Body;Body	4640	'TOP1MT	DNA topoisomerase I, mitochondrial isoform 1 precursor
GA	cg22124678	3.19E-05	0.007487708	FDR0.01	8	123793780	'ZHX2	zinc fingers and homeoboxes protein 2	TSS200;TSS200	-113	'ZHX2	zinc fingers and homeoboxes protein 2
GA	cg22130205	1.94E-06	0.001077051	FDR0.01	18	77139296	'ATP9B	probable phospholipid-transporting ATPase IIB isoform 2	TES5000;TES5000;TES5000	-16633	'NFATC1	nuclear factor of activated T-cells, cytoplasmic 1 isoform D
GA	cg22140866	8.33E-07	0.000588745	FDR0.01	13	59686676	'			-879425	'LINC00374	long intergenic non-protein coding RNA 374
GA	cg22151768	1.81E-05	0.005259275	FDR0.01	12	1702874	'FBXL14	F-box/LRR-repeat protein 14	1stExon	873	'FBXL14	F-box/LRR-repeat protein 14
GA	cg22160883	1.68E-05	0.004971678	FDR0.01	5	138609222	'SNHG4;MATR3;SNORA74D	small nucleolar RNA host gene 4;matrin-3;small nucleolar RNA, H/ACA box 74	TSS1500;TSS1500;TSS1500;TSS1	-218	'SNHG4	small nucleolar RNA host gene 4
GA	cg22169053	1.72E-08	3.13E-05	BF|FDR0.01	1	32281460	'SPOCD1	SPOC domain-containing protein 1 isoform 1	Body;Body;Body	192	'SPOCD1	SPOC domain-containing protein 1 isoform 1
GA	cg22218709	1.18E-10	9.28E-07	BF|FDR0.01	7	4723740	'FOXK1	forkhead box protein K1	Body	1815	'FOXK1	forkhead box protein K1
GA	cg22225886	1.27E-05	0.004080207	FDR0.01	8	102074434	'FLJ42969	uncharacterized LOC441374	Body	10153	'FLJ42969	uncharacterized LOC441374
GA	cg22238122	1.85E-05	0.005323382	FDR0.01	1	164546143	'PBX1	pre-B-cell leukemia transcription factor 1	Body;Body;Body;Body	17185	'PBX1	pre-B-cell leukemia transcription factor 1
GA	cg22240348	3.35E-06	0.001585151	FDR0.01	21	46975805	'SLC19A1	reduced folate transporter	Body	7134	'SLC19A1	reduced folate transporter
GA	cg22248382	3.88E-05	0.008597948	FDR0.01	17	29758931	'RAB11FIP4	rab11 family-interacting protein 4 isoform 2	Body	40239	'RAB11FIP4	rab11 family-interacting protein 4 isoform 2
GA	cg22277154	6.61E-08	8.75E-05	BF|FDR0.01	13	31020468	'			17996	'HMGB1	high mobility group protein B1
GA	cg22277972	1.09E-07	0.000129826	BF|FDR0.01	12	108960008	'ISCU	iron-sulfur cluster assembly enzyme ISCU, mitochondrial	Body;Body;Body;Body;Body;Bo	4770	'ISCU	iron-sulfur cluster assembly enzyme ISCU, mitochondrial
GA	cg22324029	1.57E-05	0.004752088	FDR0.01	6	32908466	'HLA-DMB	HLA class II histocompatibility antigen, DM beta chain precursor	Body	339	'HLA-DMB	HLA class II histocompatibility antigen, DM beta chain precursor
GA	cg22336993	1.34E-05	0.004243425	FDR0.01	6	31848507	'SLC44A4;EHMT2	choline transporter-like protein 4;histone-lysine N-methyltransferase EHMT2	TSS5000;TSS5000;TSS5000;Bod	-1684	'SLC44A4	choline transporter-like protein 4
GA	cg22349332	1.90E-05	0.00539899	FDR0.01	1	2252794	'MORN1	MORN repeat-containing protein 1 isoform 2	3'UTR	31306	'LOC100129534	small nuclear ribonucleoprotein polypeptide N pseudogene
GA	cg22356527	1.33E-05	0.00421869	FDR0.01	5	374425	'AHRR	aryl hydrocarbon receptor repressor	Body;Body	68850	'EXOC3-AS1	EXOC3 antisense RNA 1
GA	cg22365167	6.09E-10	2.82E-06	BF|FDR0.01	11	62306861	'AHNAK	neuroblast differentiation-associated protein AHNAK isoform 1	Body;Body;Body;Body	6275	'AHNAK	neuroblast differentiation-associated protein AHNAK isoform 1
GA	cg22380007	2.38E-05	0.006244332	FDR0.01	3	59718904	'			-74681	'LOC339902	hCG1813818
GA	cg22407942	2.09E-05	0.005779049	FDR0.01	7	22894828	'SNHG26;SNORD93	small nucleolar RNA host gene 26;small nucleolar RNA, C/D box 93	Body;TSS1500	970	'SNHG26	small nucleolar RNA host gene 26
GA	cg22417398	4.57E-06	0.001973182	FDR0.01	11	65293094	'SCYL1	N-terminal kinase-like protein	Body;Body	541	'SCYL1	N-terminal kinase-like protein
GA	cg22462240	1.76E-05	0.00516726	FDR0.01	17	76962663	'LGALS3BP	galectin-3-binding protein precursor	TES5000	13342	'LGALS3BP	galectin-3-binding protein precursor
GA	cg22480558	1.54E-10	1.07E-06	BF|FDR0.01	5	150461211	'TNIP1	TNFAIP3-interacting protein 1	TSS1500;TSS1500;TSS1500;TSS1	-566	'TNIP1	TNFAIP3-interacting protein 1
GA	cg22498676	4.98E-06	0.002096264	FDR0.01	1	226254585	'H3-3A	histone H3.3	Body	4169	'H3-3A	histone H3.3
GA	cg22499797	3.43E-08	5.34E-05	BF|FDR0.01	17	30585906	'			-6944	'RHBDL3	rhomboid-related protein 3
GA	cg22500132	3.16E-08	5.02E-05	BF|FDR0.01	1	155162752	'MUC1;MIR92B;THBS3	mucin-1 isoform 8 precursor;microRNA 92b;thrombospondin-3 precursor	TSS200;TSS200;TSS200;TSS200	-46	'MUC1	mucin-1 isoform 8 precursor
GA	cg22507023	3.33E-06	0.001579973	FDR0.01	17	55946195	'CUEDC1	CUE domain-containing protein 1	Body;Body	-18790	'MRPS23	28S ribosomal protein S23, mitochondrial
GA	cg22507354	2.08E-05	0.005746234	FDR0.01	17	62493832	'POLG2;DDX5;MIR3064;MIR5047	DNA polymerase subunit gamma-2, mitochondrial;probable ATP-dependent	TSS1500;TES5000;TES5000;TES	-660	'POLG2	DNA polymerase subunit gamma-2, mitochondrial
GA	cg22508782	1.25E-05	0.004054231	FDR0.01	3	49028594	'ARIH2;P4HTM	E3 ubiquitin-protein ligase ARIH2;transmembrane prolyl 4-hydroxylase isofo	TES5000;TES5000;TES5000;TES	921	'P4HTM	transmembrane prolyl 4-hydroxylase isoform c
GA	cg22531371	4.36E-09	1.2E-05	BF|FDR0.01	1	155162756	'MUC1;MIR92B;THBS3	mucin-1 isoform 8 precursor;microRNA 92b;thrombospondin-3 precursor	TSS200;TSS200;TSS200;TSS200	-50	'MUC1	mucin-1 isoform 8 precursor
GA	cg22540532	3.81E-05	0.008507109	FDR0.01	4	140518177	'			-40254	'SETD7	histone-lysine N-methyltransferase SETD7 isoform 2
GA	cg22563987	4.01E-05	0.008741223	FDR0.01	17	76130496	'TMC6;TMC8	transmembrane channel-like protein 6;transmembrane channel-like protein	TSS5000;Body	-1988	'TMC6	transmembrane channel-like protein 6
GA	cg22572362	4.33E-08	6.27E-05	BF|FDR0.01	5	501938	'SLC9A3	sodium/hydrogen exchanger 3 precursor	Body;Body	22626	'SLC9A3	sodium/hydrogen exchanger 3 precursor
GA	cg22606224	4.83E-08	6.83E-05	BF|FDR0.01	19	12266489	'ZNF625-ZNF20;ZNF625	ZNF625-ZNF20 readthrough (NMD candidate);zinc finger protein 625	Body;Body;Body	1057	'ZNF625-ZNF20	ZNF625-ZNF20 readthrough (NMD candidate)
GA	cg22608507	6.75E-08	8.86E-05	BF|FDR0.01	12	123379308	'VPS37B	vacuolar protein sorting-associated protein 37B	Body	1363	'VPS37B	vacuolar protein sorting-associated protein 37B
GA	cg22608655	1.30E-08	2.58E-05	BF|FDR0.01	1	154933512	'PBXIP1;PYGO2;LOC101928120;SHC1	pre-B-cell leukemia transcription factor-interacting protein 1 isoform 3;pygo	TSS5000;TSS5000;TSS5000;Bod	-434	'LOC101928120	uncharacterized protein LOC101928120
GA	cg22618924	3.49E-06	0.001624215	FDR0.01	5	126054151	'			58027	'LMNB1-DT	LMNB1 divergent transcript
GA	cg22622440	3.98E-06	0.001810717	FDR0.01	17	18088359	'ALKBH5	RNA demethylase ALKBH5	Body	1218	'ALKBH5	RNA demethylase ALKBH5
GA	cg22642495	5.14E-09	1.38E-05	BF|FDR0.01	19	10197856	'SHFL	shiftless antiviral inhibitor of ribosomal frameshifting protein	Body;Body	864	'SHFL	shiftless antiviral inhibitor of ribosomal frameshifting protein
GA	cg22670503	8.43E-07	0.00059209	FDR0.01	10	49482695	'FRMPD2	FERM and PDZ domain-containing protein 2 isoform 4	5'UTR	44	'FRMPD2	FERM and PDZ domain-containing protein 2 isoform 4
GA	cg22679406	1.64E-06	0.000962731	FDR0.01	6	29599331	'GABBR1	gamma-aminobutyric acid type B receptor subunit 1 precursor	TSS5000;5'UTR;Body;Body	1581	'GABBR1	gamma-aminobutyric acid type B receptor subunit 1 precursor
GA	cg22681074	1.04E-05	0.003510259	FDR0.01	1	228345324	'GJC2	gap junction gamma-2 protein	Body	7225	'IBA57-DT	IBA57 divergent transcript
GA	cg22685502	7.11E-10	3.12E-06	BF|FDR0.01	3	72496851	'RYBP	ring and YY1 binding protein, isoform A	TSS1500	-1077	'RYBP	ring and YY1 binding protein, isoform A
GA	cg22688566	8.76E-07	0.000607567	FDR0.01	17	27459835	'MYO18A	unconventional myosin-XVIIIa isoform e	Body;Body;Body;Body;Body;Bo	7572	'MYO18A	unconventional myosin-XVIIIa isoform e
GA	cg22720790	1.37E-05	0.004307873	FDR0.01	19	58716032	'ZNF274	neurotrophin receptor-interacting factor homolog isoform d	Body;Body;Body;Body	21605	'ZNF274	neurotrophin receptor-interacting factor homolog isoform d
GA	cg22729539	2.25E-06	0.001211942	FDR0.01	12	122519119	'MLXIP	MLX-interacting protein isoform 2	Body	2458	'MLXIP	MLX-interacting protein isoform 2
GA	cg22734086	1.73E-06	0.001003665	FDR0.01	2	26785367	'OTOF;FAM166C	otoferlin;protein FAM166C	TSS5000;TSS5000;TSS200;TSS20	-87	'FAM166C	protein FAM166C
GA	cg22741244	4.33E-06	0.001899024	FDR0.01	2	239052286	'KLHL30	kelch-like protein 30	Body	4978	'KLHL30	kelch-like protein 30
GA	cg22760304	8.68E-06	0.00311387	FDR0.01	19	14607597	'GIPC1	PDZ domain-containing protein GIPC1	TSS1500;TSS1500;TSS1500;TSS1	-636	'GIPC1	PDZ domain-containing protein GIPC1
GA	cg22761205	6.12E-10	2.82E-06	BF|FDR0.01	11	457256	'PTDSS2	phosphatidylserine synthase 2	Body;Body;Body;Body	6978	'PTDSS2	phosphatidylserine synthase 2
GA	cg22784595	3.65E-05	0.008277727	FDR0.01	10	7455714	'SFMBT2	scm-like with four MBT domains protein 2	TSS5000;TSS5000	-2266	'SFMBT2	scm-like with four MBT domains protein 2
GA	cg22805485	1.38E-06	0.000851679	FDR0.01	11	13983818	'SPON1	spondin-1 precursor	TSS1500	-451	'SPON1	spondin-1 precursor
GA	cg22820364	9.18E-06	0.003230315	FDR0.01	14	81901540	'STON2	stonin-2	Body;Body	1269	'STON2	stonin-2
GA	cg22828383	5.09E-08	7.07E-05	BF|FDR0.01	12	125002191	'NCOR2	nuclear receptor corepressor 2	Body;Body;Body	49967	'NCOR2	nuclear receptor corepressor 2
GA	cg22833809	1.51E-05	0.004632675	FDR0.01	17	76129984	'TMC6;TMC8	transmembrane channel-like protein 6;transmembrane channel-like protein	TSS1500;Body	-1476	'TMC6	transmembrane channel-like protein 6
GA	cg22846809	1.60E-05	0.004818479	FDR0.01	22	22001120	'SDF2L1	stromal cell-derived factor 2-like protein 1 precursor	TES5000	4530	'SDF2L1	stromal cell-derived factor 2-like protein 1 precursor
GA	cg22846826	4.37E-11	4.44E-07	BF|FDR0.01	7	4723927	'FOXK1	forkhead box protein K1	Body	2002	'FOXK1	forkhead box protein K1
GA	cg22871253	2.86E-05	0.007049222	FDR0.01	6	159238744	'EZR;EZR-AS1	ezrin;EZR antisense RNA 1	Body;Body;TSS1500	-266	'EZR-AS1	EZR antisense RNA 1
GA	cg22877452	2.90E-05	0.007107775	FDR0.01	1	61517876	'			-25022	'NFIA	nuclear factor 1 A-type
GA	cg22878489	4.72E-06	0.002019092	FDR0.01	6	33245701	'RPS18;B3GALT4;WDR46	40S ribosomal protein S18;beta-1,3-galactosyltransferase 4;WD repeat-conta	TES5000;1stExon;TES5000;TES5	802	'B3GALT4	beta-1,3-galactosyltransferase 4

Trait	#cpg	p	fdr	significance	chr	pos.hg19	gene.Illumina	gene.description	locations	dist2TSS	gene.nearest	gene.description
GA	cg22879583	3.84E-05	0.008548487	FDR0.01	11	111750412	'FDXACB1;C11orf1	ferredoxin-fold anticodon-binding domain-containing protein 1;UPF0686 pro	TSS1500;TSS1500;Body;Body	148	'C11orf1	UPF0686 protein C11orf1 isoform 2
GA	cg22897141	1.95E-07	0.000197439	FDR0.01	11	64577338	'MEN1	menin	1stExon;1stExon;1stExon;1stEx	685	'MEN1	menin
GA	cg22903402	1.63E-05	0.004861424	FDR0.01	2	128366514	'MYO7B	unconventional myosin-VIIb	Body	-36924	'GPR17	uracil nucleotide/cysteinyl leukotriene receptor
GA	cg22941974	1.34E-05	0.004252885	FDR0.01	2	56410011	'LOC100129434;CCDC85A	uncharacterized LOC100129434;coiled-coil domain-containing protein 85A	Body;TSS1500;TSS1500;TSS150	-1113	'CCDC85A	coiled-coil domain-containing protein 85A
GA	cg23008153	4.74E-07	0.000386799	FDR0.01	5	63461305	'RNF180	E3 ubiquitin-protein ligase RNF180	TSS1500;TSS1500;TSS1500;TSS1	-364	'RNF180	E3 ubiquitin-protein ligase RNF180
GA	cg23009780	3.38E-05	0.007800019	FDR0.01	2	96774492	'ADRA2B	alpha-2B adrenergic receptor	TES5000	7818	'ADRA2B	alpha-2B adrenergic receptor
GA	cg23013850	3.52E-06	0.001633577	FDR0.01	2	225266304	'FAM124B	protein FAM124B	1stExon;1stExon	407	'FAM124B	protein FAM124B
GA	cg23014759	4.38E-06	0.001912728	FDR0.01	1	147059047	'BCL9	B-cell CLL/lymphoma 9 protein	Body	45777	'BCL9	B-cell CLL/lymphoma 9 protein
GA	cg23024102	3.82E-07	0.000331261	FDR0.01	13	45149706	'TSC22D1;TSC22D1-AS1	TSC22 domain family protein 1 isoform 3;TSC22D1 antisense RNA 1	1stExon;1stExon;TSS1500	-325	'TSC22D1-AS1	TSC22D1 antisense RNA 1
GA	cg23028178	1.90E-06	0.001066227	FDR0.01	1	236229267	'NID1	nidogen-1 precursor	TSS1500	-877	'NID1	nidogen-1 precursor
GA	cg23041109	3.14E-06	0.001506851	FDR0.01	13	29157934	'			-75332	'POMP	proteasome maturation protein
GA	cg23042318	3.91E-05	0.008636129	FDR0.01	19	1270330	'CIRBP-AS1;CIRBP	CIRBP antisense RNA 1;cold-inducible RNA-binding protein	TSS200;Body;Body;Body;Body;B	-71	'CIRBP-AS1	CIRBP antisense RNA 1
GA	cg23043544	2.57E-05	0.006574236	FDR0.01	7	2124092	'MAD1L1;SNORA114	mitotic spindle assembly checkpoint protein MAD1;small nucleolar RNA, H/A	Body;Body;Body;Body;Body;TES	4595	'SNORA114	small nucleolar RNA, H/ACA box 114
GA	cg23044005	2.38E-05	0.006244332	FDR0.01	7	22588840	'			-14115	'LOC100506178	uncharacterized LOC100506178
GA	cg23044186	9.10E-06	0.003211722	FDR0.01	5	141228416	'PCDH1	protocadherin-1	TES5000	17351	'PCDH1	protocadherin-1
GA	cg23044884	1.59E-07	0.000170296	FDR0.01	8	30245145	'RBPMS-AS1;RBPMS	RBPMS antisense RNA 1;RNA-binding protein with multiple splicing	TSS5000;Body;Body;Body;Body	-2228	'RBPMS-AS1	RBPMS antisense RNA 1
GA	cg23050300	1.28E-06	0.000807406	FDR0.01	1	3281321	'PRDM16	histone-lysine N-methyltransferase PRDM16 isoform 2	Body;Body	-89907	'ARHGEF16	rho guanine nucleotide exchange factor 16
GA	cg23051299	1.02E-05	0.003484217	FDR0.01	2	75938289	'GCFC2	GC-rich sequence DNA-binding factor 2	TSS1500;TSS1500;TSS1500	-248	'GCFC2	GC-rich sequence DNA-binding factor 2
GA	cg23053723	2.86E-05	0.007057248	FDR0.01	17	63133521	'RGS9	regulator of G-protein signaling 9 isoform 2	5'UTR;5'UTR;5'UTR	34	'RGS9	regulator of G-protein signaling 9 isoform 2
GA	cg23066564	6.33E-06	0.002514288	FDR0.01	22	46481734	'MIRLET7BHG	MIRLET7B host gene	TSS200;TSS200	-142	'MIRLET7BHG	MIRLET7B host gene
GA	cg23068939	2.74E-05	0.006842589	FDR0.01	1	112016586	'C1orf162	transmembrane protein C1orf162 isoform 3	TSS200;TSS200;TSS200	-19	'C1orf162	transmembrane protein C1orf162 isoform 3
GA	cg23091302	7.75E-07	0.000557499	FDR0.01	3	19389409	'KCNH8	potassium voltage-gated channel subfamily H member 8	Body	-32986	'MIR4791	microRNA 4791
GA	cg23098018	2.42E-05	0.006316368	FDR0.01	1	9775755	'PIK3CD	phosphatidylinositol 4,5-bisphosphate 3-kinase catalytic subunit delta isofor	Body;Body;Body	24231	'PIK3CD	phosphatidylinositol 4,5-bisphosphate 3-kinase catalytic subunit delta isoform
GA	cg23100720	2.61E-05	0.006648139	FDR0.01	6	155770386	'NOX3	NADPH oxidase 3	Body	6587	'NOX3	NADPH oxidase 3
GA	cg23141902	1.83E-06	0.001038268	FDR0.01	22	39712694	'RPL3;SNORD83B;SNORD83A;SNORD139;SNORD43	ribosomal protein L3;small nucleolar RNA, C/D box 83B;small nucleolar RNA,	Body;Body;TSS5000;TSS1500;TE	207	'SNORD139	small nucleolar RNA, C/D box 139
GA	cg23152055	4.39E-05	0.009244415	FDR0.01	15	75661059	'MAN2C1;SIN3A	alpha-mannosidase 2C1;paired amphipathic helix protein Sin3a	TSS200;TSS200;TSS200;TSS200;	-91	'MAN2C1	alpha-mannosidase 2C1
GA	cg23157190	1.68E-05	0.004981885	FDR0.01	2	75060880	'HK2	hexokinase-2	TSS1500;TSS1500	-373	'HK2	hexokinase-2
GA	cg23166970	3.91E-05	0.008636129	FDR0.01	3	182817578	'MCCC1	methylcrotonoyl-CoA carboxylase subunit alpha, mitochondrial	TSS1500;TSS1500;TSS1500;TSS1	-203	'MCCC1	methylcrotonoyl-CoA carboxylase subunit alpha, mitochondrial
GA	cg23180593	4.16E-05	0.00894807	FDR0.01	13	111301487	'CARS2	probable cysteine--tRNA ligase, mitochondrial isoform 3	Body;Body;Body;Body	33513	'NAXD	ATP-dependent (S)-NAD(P)H-hydrate dehydratase isoform 1
GA	cg23181573	1.37E-05	0.004317275	FDR0.01	17	3863976	'ATP2A3	sarcoplasmic/endoplasmic reticulum calcium ATPase 3	Body;Body;Body;Body;Body;Bo	3755	'ATP2A3	sarcoplasmic/endoplasmic reticulum calcium ATPase 3
GA	cg23186098	1.15E-05	0.003783424	FDR0.01	6	32056119	'TNXB	tenascin-X precursor	Body;Body	20996	'TNXB	tenascin-X precursor
GA	cg23188579	2.54E-06	0.001299485	FDR0.01	4	141264558	'SCOC;SCOC-AS1	short coiled-coil protein;SCOC antisense RNA 1	Body;Body;TSS200;TSS200;TSS2	-38	'SCOC	short coiled-coil protein
GA	cg23210521	1.82E-06	0.001035738	FDR0.01	4	95680414	'BMPR1B-DT;BMPR1B	BMPR1B divergent transcript;bone morphogenetic protein receptor type-1B	TSS5000;Body	1309	'BMPR1B	bone morphogenetic protein receptor type-1B
GA	cg23225748	2.31E-05	0.006126008	FDR0.01	10	80062665	'			35567	'LINC00595	long intergenic non-protein coding RNA 595
GA	cg23229770	4.05E-05	0.008788927	FDR0.01	2	129491004	'			-131169	'LOC101927881	uncharacterized LOC101927881
GA	cg23238677	1.93E-05	0.005459259	FDR0.01	2	170157334	'LRP2	low-density lipoprotein receptor-related protein 2 precursor	Body	61710	'LRP2	low-density lipoprotein receptor-related protein 2 precursor
GA	cg23239828	1.17E-05	0.003854291	FDR0.01	5	442821	'AHRR;EXOC3-AS1;EXOC3	aryl hydrocarbon receptor repressor;EXOC3 antisense RNA 1;exocyst comple	TES5000;TES5000;Body;TSS150	454	'EXOC3-AS1	EXOC3 antisense RNA 1
GA	cg23254047	1.24E-05	0.004021037	FDR0.01	10	38264940	'ZNF25	zinc finger protein 25	TSS200;Body;Body;Body;Body;B	-32	'ZNF25	zinc finger protein 25
GA	cg23255283	2.24E-06	0.001209617	FDR0.01	18	74240582	'LINC00908	long intergenic non-protein coding RNA 908	TSS200	-29	'LINC00908	long intergenic non-protein coding RNA 908
GA	cg23263226	4.37E-06	0.00191161	FDR0.01	17	78226839	'SLC26A11	sodium-independent sulfate anion transporter	3'UTR;3'UTR;3'UTR;3'UTR	-7811	'RNF213	E3 ubiquitin-protein ligase RNF213 isoform 2
GA	cg23291039	1.13E-05	0.003756248	FDR0.01	4	25808901	'SEL1L3	protein sel-1 homolog 3	Body;Body;Body	55709	'SEL1L3	protein sel-1 homolog 3
GA	cg23314506	3.89E-05	0.008614143	FDR0.01	13	46626917	'ZC3H13;CPB2-AS1;CPB2	zinc finger CCCH domain-containing protein 13 isoform 1;CPB2 antisense RN	TSS200;TSS200;TSS200;TSS200;	-36	'ZC3H13	zinc finger CCCH domain-containing protein 13 isoform 1
GA	cg23329272	2.49E-05	0.006436041	FDR0.01	5	180085924	'			-9324	'FLT4	vascular endothelial growth factor receptor 3 precursor
GA	cg23334662	3.83E-06	0.001752342	FDR0.01	2	198540518	'RFTN2	raftlin-2	TSS1500	-207	'RFTN2	raftlin-2
GA	cg23348081	1.48E-06	0.000892211	FDR0.01	12	14413690	'			-104875	'ATF7IP	activating transcription factor 7-interacting protein 1
GA	cg23359665	2.51E-06	0.001291469	FDR0.01	6	32120907	'PRRT1;LOC100507547;PPT2;PPT2-EGFL8	proline-rich transmembrane protein 1;uncharacterized LOC100507547;lysos	TSS200;TSS1500;Body;Body;Bo	-47	'PRRT1	proline-rich transmembrane protein 1
GA	cg23372544	2.97E-06	0.001449697	FDR0.01	19	23387068	'			46128	'ZNF724	zinc finger protein 724 isoform 1
GA	cg23378033	1.39E-08	2.71E-05	BF|FDR0.01	12	65672031	'MSRB3	methionine-R-sulfoxide reductase B3	TSS1500;TSS1500;TSS1500;TSS1	-431	'MSRB3	methionine-R-sulfoxide reductase B3
GA	cg23384027	4.73E-07	0.000386799	FDR0.01	12	54688344	'NFE2	transcription factor NF-E2 45 kDa subunit	Body;Body;Body	1219	'NFE2	transcription factor NF-E2 45 kDa subunit
GA	cg23391324	1.52E-05	0.00464419	FDR0.01	15	73344290	'NEO1	neogenin	TSS1500;TSS1500;TSS1500	-513	'NEO1	neogenin
GA	cg23462152	2.97E-05	0.00719646	FDR0.01	14	91723888	'GPR68	ovarian cancer G-protein coupled receptor 1	TSS5000;TSS5000	-3664	'GPR68	ovarian cancer G-protein coupled receptor 1
GA	cg23463310	1.16E-06	0.000747427	FDR0.01	11	65188784	'NEAT1	nuclear paraspeckle assembly transcript 1	TSS1500;TSS1500	-1484	'NEAT1	nuclear paraspeckle assembly transcript 1
GA	cg23471393	1.19E-06	0.000765289	FDR0.01	17	79797038	'PPP1R27;P4HB	protein phosphatase 1 regulatory subunit 27;protein disulfide-isomerase pre	TSS5000;TES5000	-4112	'PPP1R27	protein phosphatase 1 regulatory subunit 27
GA	cg23474211	1.84E-05	0.005308068	FDR0.01	7	88388646	'ZNF804B	zinc finger protein 804B	TSS1500	-367	'ZNF804B	zinc finger protein 804B
GA	cg23489038	2.42E-05	0.006311797	FDR0.01	12	111619190	'CUX2	homeobox protein cut-like 2 isoform 2	Body;Body	81383	'CUX2	homeobox protein cut-like 2 isoform 2
GA	cg23490300	9.84E-06	0.003386846	FDR0.01	2	41461406	'			-643288	'LINC01913	long intergenic non-protein coding RNA 1913
GA	cg23513600	1.42E-05	0.004423253	FDR0.01	3	171171401	'TNIK	TRAF2 and NCK-interacting protein kinase	Body;Body;Body;Body;Body;Bo	6793	'TNIK	TRAF2 and NCK-interacting protein kinase
GA	cg23516002	1.41E-06	0.000864695	FDR0.01	7	128099617	'HILPDA	hypoxia-inducible lipid droplet-associated protein precursor	TES5000;TES5000	3686	'HILPDA	hypoxia-inducible lipid droplet-associated protein precursor
GA	cg23523668	3.02E-05	0.007272241	FDR0.01	11	65194989	'NEAT1	nuclear paraspeckle assembly transcript 1	TES5000;Body	4721	'NEAT1	nuclear paraspeckle assembly transcript 1
GA	cg23532980	3.00E-05	0.007230021	FDR0.01	1	35250735	'GJB3	gap junction beta-3 protein	1stExon;1stExon	2867	'GJB3	gap junction beta-3 protein
GA	cg23548163	9.18E-06	0.003230315	FDR0.01	19	46807119	'HIF3A	hypoxia-inducible factor 3-alpha	Body;Body;Body;Body	244	'HIF3A	hypoxia-inducible factor 3-alpha
GA	cg23560159	4.68E-05	0.009616031	FDR0.01	6	32805748	'TAP2;PSMB8	antigen peptide transporter 2;proteasome subunit beta type-8	1stExon;1stExon;TES5000;TES5	799	'TAP2	antigen peptide transporter 2
GA	cg23583454	1.24E-07	0.000141653	FDR0.01	6	30582333	'PPP1R10;MRPS18B	serine/threonine-protein phosphatase 1 regulatory subunit 10;28S ribosoma	Body;Body;TSS5000	2687	'PPP1R10	serine/threonine-protein phosphatase 1 regulatory subunit 10
GA	cg23584647	3.93E-05	0.008645923	FDR0.01	11	61717191	'BEST1	bestrophin-1	TSS200;TSS1500;TSS1500;TSS15	-101	'BEST1	bestrophin-1
GA	cg23588384	3.07E-08	4.92E-05	BF|FDR0.01	8	144684634	'EEF1D;TIGD5;PYCR3	elongation factor 1-delta;tigger transposable element-derived protein 5;pyrr	TSS5000;3'UTR;TES5000;TES50	4634	'TIGD5	tigger transposable element-derived protein 5
GA	cg23590660	2.23E-05	0.005986109	FDR0.01	12	8205602	'FOXJ2;C3AR1	forkhead box protein J2;C3a anaphylatoxin chemotactic receptor	3'UTR;TES5000;TES5000;TES50	13353	'C3AR1	C3a anaphylatoxin chemotactic receptor
GA	cg23592421	9.49E-06	0.003315348	FDR0.01	14	105147461	'MIR4710	microRNA 4710	TSS5000	-3375	'MIR4710	microRNA 4710
GA	cg23599716	2.28E-05	0.006079612	FDR0.01	1	44686794	'DMAP1;ERI3;ERI3-IT1	DNA methyltransferase 1-associated protein 1;ERI1 exoribonuclease 3 isofor	TES5000;TES5000;TES5000;3'UT	7652	'DMAP1	DNA methyltransferase 1-associated protein 1
GA	cg23609982	4.22E-05	0.009017792	FDR0.01	1	154377403	'IL6R-AS1;IL6R	IL6R antisense RNA 1;interleukin-6 receptor subunit alpha precursor	Body;TSS1500;TSS1500;TSS150	-265	'IL6R	interleukin-6 receptor subunit alpha precursor
GA	cg23612220	2.04E-05	0.005667899	FDR0.01	1	151129298	'TNFAIP8L2;TNFAIP8L2-SCNM1;LYSMD1	tumor necrosis factor alpha-induced protein 8-like protein 2;TNFAIP8L2-SCN	Body;Body;Body;TES5000;TES5	174	'TNFAIP8L2-SCNM1	TNFAIP8L2-SCNM1 protein
GA	cg23618477	3.02E-05	0.007272241	FDR0.01	6	169334872	'LOC105378146	uncharacterized LOC105378146	Body	29605	'LOC105378146	uncharacterized LOC105378146
GA	cg23640701	4.70E-06	0.002017305	FDR0.01	12	52301634	'ACVRL1	serine/threonine-protein kinase receptor R3 precursor	Body;TSS5000	347	'ACVRL1	serine/threonine-protein kinase receptor R3 precursor
GA	cg23648809	1.97E-05	0.005530267	FDR0.01	1	2106400	'PRKCZ	protein kinase C zeta type	Body;Body;Body;Body;Body;Bo	8914	'PRKCZ-AS1	PRKCZ antisense RNA 1
GA	cg23667127	3.15E-06	0.001512038	FDR0.01	12	1739917	'WNT5B	protein Wnt-5b precursor	Body;Body	1521	'WNT5B	protein Wnt-5b precursor
GA	cg23668631	3.25E-05	0.007605243	FDR0.01	17	3796936	'CAMKK1;P2RX1	calcium/calmodulin-dependent protein kinase kinase 1;P2X purinoceptor 1	TSS5000;TSS1500;TSS1500;TES5	-589	'CAMKK1	calcium/calmodulin-dependent protein kinase kinase 1
GA	cg23674358	2.68E-05	0.006747113	FDR0.01	10	22726885	'LOC100499489	uncharacterized LOC100499489	TSS200	-27	'LOC100499489	uncharacterized LOC100499489
GA	cg23696891	2.33E-05	0.006153655	FDR0.01	6	138187872	'WAKMAR2;TNFAIP3	wound and keratinocyte migration associated lncRNA 2;tumor necrosis facto	Body;TSS1500;TSS1500;TSS150	-523	'TNFAIP3	tumor necrosis factor alpha-induced protein 3
GA	cg23698978	1.02E-05	0.00348157	FDR0.01	7	20838631	'			-12126	'SP8	transcription factor Sp8 isoform 2
GA	cg23707540	2.29E-10	1.42E-06	BF|FDR0.01	10	126836038	'CTBP2	C-terminal-binding protein 2	Body;Body;Body;Body;Body;Bo	11308	'CTBP2	C-terminal-binding protein 2
GA	cg23730027	1.45E-08	2.80E-05	BF|FDR0.01	3	57995180	'FLNB	filamin-B	Body;Body;Body;Body	1032	'FLNB	filamin-B
GA	cg23738770	1.71E-05	0.005041311	FDR0.01	10	104154605	'NFKB2	nuclear factor NF-kappa-B p100 subunit	Body;Body;Body;TSS1500;TSS15	271	'NFKB2	nuclear factor NF-kappa-B p100 subunit
GA	cg23741330	5.35E-06	0.002220576	FDR0.01	6	116832940	'TRAPPC3L;CALHM5	trafficking protein particle complex subunit 3-like protein;calcium homeostas	Body;1stExon	139	'CALHM5	calcium homeostasis modulator protein 5
GA	cg23754918	6.64E-07	0.000496565	FDR0.01	7	2653733	'IQCE	IQ domain-containing protein E isoform 6	3'UTR;3'UTR	-17886	'TTYH3	protein tweety homolog 3
GA	cg23762465	6.14E-06	0.002453578	FDR0.01	22	50360628	'PIM3;MIR6821	serine/threonine-protein kinase pim-3;microRNA 6821	TES5000;TES5000	4115	'MIR6821	microRNA 6821
GA	cg23791626	2.66E-05	0.00672211	FDR0.01	17	65542892	'PITPNC1	cytoplasmic phosphatidylinositol transfer protein 1	Body;Body	-75191	'MIR548D2	microRNA 548d-2
GA	cg23807570	5.27E-06	0.002197866	FDR0.01	12	110787107	'ATP2A2	sarcoplasmic/endoplasmic reticulum calcium ATPase 2	Body;3'UTR	54428	'ANAPC7	anaphase-promoting complex subunit 7 isoform b
GA	cg23839159	2.37E-06	0.001249385	FDR0.01	1	43281794	'SVBP;ERMAP	small vasohibin-binding protein;erythroid membrane-associated protein pre	Body;TSS1500	-998	'ERMAP	erythroid membrane-associated protein precursor
GA	cg23878241	7.36E-06	0.002774748	FDR0.01	12	111019734	'PPTC7	protein phosphatase PTC7 homolog	Body;Body	1389	'PPTC7	protein phosphatase PTC7 homolog
GA	cg23895076	9.51E-06	0.003315348	FDR0.01	17	8578385	'			-44306	'MYH10	myosin-10

Trait	#cpg	p	fdr	significance	chr	pos.hg19	gene.Illumina	gene.description	locations	dist2TSS	gene.nearest	gene.description
GA	cg23915792	1.31E-06	0.000820198	FDR0.01	22	41763255	'TEF	transcription factor VBP	TSS200	-81	'TEF	transcription factor VBP
GA	cg23934295	6.53E-06	0.002572219	FDR0.01	14	85995655	'FLRT2	leucine-rich repeat transmembrane protein FLRT2 precursor	TSS1500;TSS1500;TSS1500;TSS1	-713	'FLRT2	leucine-rich repeat transmembrane protein FLRT2 precursor
GA	cg23950233	1.97E-05	0.005530267	FDR0.01	6	33245739	'RPS18;B3GALT4;WDR46	40S ribosomal protein S18;beta-1,3-galactosyltransferase 4;WD repeat-conta	TSS5000;1stExon;TES5000;TES5	840	'B3GALT4	beta-1,3-galactosyltransferase 4
GA	cg23987137	5.29E-07	0.000417312	FDR0.01	5	58653624	'PDE4D	cAMP-specific 3',5'-cyclic phosphodiesterase 4D isoform 12	TSS1500;Body;Body;Body;Body	-828	'PDE4D	cAMP-specific 3',5'-cyclic phosphodiesterase 4D isoform 12
GA	cg24003955	2.54E-05	0.006528281	FDR0.01	1	9912508	'CTNNBIP1	beta-catenin-interacting protein 1	Body;Body	-27924	'CLSTN1	calsyntenin-1 precursor
GA	cg24024661	5.56E-07	0.000434678	FDR0.01	19	1074425	'ARHGAP45	rho GTPase-activating protein 45	Body;Body;Body;Body;TSS5000	-2207	'ARHGAP45	rho GTPase-activating protein 45
GA	cg24054871	4.41E-06	0.00192202	FDR0.01	22	45073148	'PRR5	proline-rich protein 5	Body;Body;Body;Body;Body	184	'PRR5	proline-rich protein 5
GA	cg24060890	7.11E-06	0.002711257	FDR0.01	4	93229269	'GRID2	glutamate receptor ionotropic, delta-2 precursor	Body;Body	3720	'GRID2	glutamate receptor ionotropic, delta-2 precursor
GA	cg24079727	3.76E-05	0.008435063	FDR0.01	15	57317980	'TCF12	transcription factor 12	Body;Body;Body;Body;Body;Bo	106338	'TCF12	transcription factor 12
GA	cg24085039	3.76E-07	0.000328784	FDR0.01	10	134211908	'PWWP2B	PWWP domain-containing protein 2B	Body;Body	1205	'PWWP2B	PWWP domain-containing protein 2B
GA	cg24112733	7.69E-06	0.002869069	FDR0.01	7	38255578	'STARD3NL	STARD3 N-terminal-like protein	Body;Body;Body;Body;Body;Bo	37732	'STARD3NL	STARD3 N-terminal-like protein
GA	cg24124954	2.74E-05	0.006842589	FDR0.01	6	31508106	'ATP6V1G2-DDX39B;DDX39B;SNORD117;SNORD84;D	ATP6V1G2-DDX39B readthrough (NMD candidate);spliceosome RNA helicase	Body;1stExon;1stExon;Body;TS	849	'SNORD84	small nucleolar RNA, C/D box 84
GA	cg24129356	8.25E-06	0.003019726	FDR0.01	6	32920735	'HLA-DMA	HLA class II histocompatibility antigen, DM alpha chain precursor	1stExon	139	'HLA-DMA	HLA class II histocompatibility antigen, DM alpha chain precursor
GA	cg24135615	4.30E-05	0.009107607	FDR0.01	17	37042201	'LASP1	LIM and SH3 domain protein 1	Body;Body;Body	15891	'LASP1	LIM and SH3 domain protein 1
GA	cg24142464	7.07E-09	1.74E-05	BF|FDR0.01	1	40040489	'PABPC4;PABPC4-AS1	polyadenylate-binding protein 4;PABPC4 antisense RNA 1	Body;Body;Body;TES5000	1973	'PABPC4	polyadenylate-binding protein 4
GA	cg24144853	3.90E-09	1.09E-05	BF|FDR0.01	3	194258370	'			-46626	'TMEM44-AS1	TMEM44 antisense RNA 1
GA	cg24149590	5.84E-08	7.94E-05	BF|FDR0.01	14	75867231	'			-27195	'JDP2	jun dimerization protein 2 isoform b
GA	cg24160569	7.50E-07	0.000543721	FDR0.01	10	62011445	'ANK3	ankyrin-3	Body;Body;Body;Body	-110671	'ANK3	ankyrin-3
GA	cg24162251	8.43E-07	0.00059209	FDR0.01	14	32420205	'LINC02313	long intergenic non-protein coding RNA 2313	TES5000	6147	'LINC02313	long intergenic non-protein coding RNA 2313
GA	cg24162603	1.37E-07	0.000152926	FDR0.01	3	127945858	'EEFSEC	selenocysteine-specific elongation factor	Body	-73101	'RUVBL1	ruvB-like 1
GA	cg24162801	4.12E-06	0.001846952	FDR0.01	1	184504284	'C1orf21	uncharacterized protein C1orf21	Body	148122	'C1orf21	uncharacterized protein C1orf21
GA	cg24165638	4.09E-06	0.001840008	FDR0.01	19	831456	'AZU1	azurocidin preproprotein	Body	3620	'AZU1	azurocidin preproprotein
GA	cg24166457	2.58E-13	1.19E-08	BF|FDR0.01	19	45590882	'GEMIN7;GEMIN7-AS1	gem-associated protein 7;GEMIN7 antisense RNA 1	Body;Body;Body;Body;Body;Bo	2767	'GEMIN7-AS1	GEMIN7 antisense RNA 1
GA	cg24167314	3.80E-05	0.008505345	FDR0.01	12	103311500	'PAH	phenylalanine hydroxylase	TSS1500;Body	-478	'PAH	phenylalanine hydroxylase
GA	cg24173629	1.32E-07	0.000149153	FDR0.01	22	25575559	'KIAA1671	uncharacterized protein KIAA1671	Body	-20265	'CRYBB3	beta-crystallin B3
GA	cg24185397	2.54E-05	0.006528281	FDR0.01	17	25659609	'			38470	'WSB1	WD repeat and SOCS box-containing protein 1
GA	cg24185852	6.44E-06	0.002544626	FDR0.01	5	168690818	'SLIT3;MIR585	slit homolog 3 protein precursor;microRNA 585	Body;Body;TSS200	-120	'MIR585	microRNA 585
GA	cg24193659	9.53E-06	0.003316848	FDR0.01	11	788406	'LOC171391;CEND1;SLC25A22	uncharacterized LOC171391;cell cycle exit and neuronal differentiation prote	TES5000;TSS5000;Body;TES500	1684	'CEND1	cell cycle exit and neuronal differentiation protein 1
GA	cg24202772	4.19E-05	0.008977311	FDR0.01	10	124402857	'DMBT1	deleted in malignant brain tumors 1 protein isoform b precursor	Body;Body;Body;Body	-29508	'LOC112577516	carbohydrate-binding protein AQN-1-like precursor
GA	cg24247537	1.56E-11	2.19E-07	BF|FDR0.01	11	457278	'PTDSS2	phosphatidylserine synthase 2	Body;Body;Body;Body;Body	7000	'PTDSS2	phosphatidylserine synthase 2
GA	cg24250902	1.75E-06	0.001008635	FDR0.01	1	230415547	'GALNT2	polypeptide N-acetylgalactosaminyltransferase 2 precursor	3'UTR;3'UTR	146531	'PGBD5	piggyBac transposable element-derived protein 5
GA	cg24251439	3.80E-09	1.07E-05	BF|FDR0.01	19	1112327	'SBNO2	protein strawberry notch homolog 2	Body;Body	7679	'GPX4	phospholipid hydroperoxide glutathione peroxidase precursor
GA	cg24261333	6.93E-06	0.002664953	FDR0.01	11	73357019	'PLEKHB1	pleckstrin homology domain-containing family B member 1	TSS1500;TSS1500;TSS5000;TSS	-606	'PLEKHB1	pleckstrin homology domain-containing family B member 1
GA	cg24292665	1.04E-05	0.003513867	FDR0.01	11	69065779	'MYEOV	myeloma-overexpressed gene protein isoform 1	TES5000;TES5000;TES5000;TES	4158	'MYEOV	myeloma-overexpressed gene protein isoform 1
GA	cg24301247	1.05E-05	0.003538386	FDR0.01	12	129295649	'SLC15A4	solute carrier family 15 member 4	Body	12854	'SLC15A4	solute carrier family 15 member 4
GA	cg24323958	4.84E-05	0.009850766	FDR0.01	1	108741884	'SLC25A24	calcium-binding mitochondrial carrier protein SCaMC-1	Body	1081	'SLC25A24	calcium-binding mitochondrial carrier protein SCaMC-1
GA	cg24334983	6.39E-06	0.002530226	FDR0.01	10	81965683	'ANXA11;LINC00857	annexin A11;long intergenic non-protein coding RNA 857	TSS1500;TSS1500;TSS1500;TSS1	-119	'ANXA11	annexin A11
GA	cg24373369	2.88E-08	4.71E-05	BF|FDR0.01	15	65320737	'MTFMT	methionyl-tRNA formyltransferase, mitochondrial	Body	1240	'MTFMT	methionyl-tRNA formyltransferase, mitochondrial
GA	cg24376214	1.99E-05	0.005565589	FDR0.01	19	38918135	'RASGRP4	RAS guanyl-releasing protein 4	TSS1500;TSS1500;TSS1500;TSS1	-1190	'RASGRP4	RAS guanyl-releasing protein 4
GA	cg24391122	3.85E-08	5.78E-05	BF|FDR0.01	19	7986137	'TGFBR3L;SNAPC2;CTXN1	transforming growth factor-beta receptor type 3-like protein;snRNA-activati	TES5000;Body;Body;TES5000	238	'SNAPC2	snRNA-activating protein complex subunit 2
GA	cg24397007	4.79E-05	0.009788241	FDR0.01	2	28619094	'FLJ31356;FOSL2	uncharacterized protein FLJ31356;fos-related antigen 2	TSS5000;Body	-1555	'FLJ31356	uncharacterized protein FLJ31356
GA	cg24401487	3.29E-09	9.57E-06	BF|FDR0.01	1	230416880	'GALNT2	polypeptide N-acetylgalactosaminyltransferase 2 precursor	3'UTR;3'UTR	145198	'PGBD5	piggyBac transposable element-derived protein 5
GA	cg24401737	4.72E-05	0.009666218	FDR0.01	2	202047473	'CASP10	caspase-10	TSS200;TSS1500;TSS1500;TSS1	-147	'CASP10	caspase-10
GA	cg24406240	3.69E-05	0.008338881	FDR0.01	11	88153520	'			-82616	'CTSC	dipeptidyl peptidase 1 precursor
GA	cg24407596	4.07E-05	0.008808141	FDR0.01	7	98718683	'SMURF1	E3 ubiquitin-protein ligase SMURF1	Body;Body;Body	23048	'SMURF1	E3 ubiquitin-protein ligase SMURF1
GA	cg24413391	4.43E-05	0.009280449	FDR0.01	2	27886079	'SUPT7L;SLC4A1AP	STAGA complex 65 subunit gamma;kanadaptin	Body;Body;Body;Body;Body;TS	-258	'SLC4A1AP	kanadaptin
GA	cg24420089	1.86E-06	0.001049272	FDR0.01	11	457304	'PTDSS2	phosphatidylserine synthase 2	Body;Body;Body;Body;Body	7026	'PTDSS2	phosphatidylserine synthase 2
GA	cg24421410	4.63E-05	0.009566758	FDR0.01	6	32920220	'HLA-DMA	HLA class II histocompatibility antigen, DM alpha chain precursor	Body	654	'HLA-DMA	HLA class II histocompatibility antigen, DM alpha chain precursor
GA	cg24437408	1.29E-05	0.0041292	FDR0.01	10	134661502	'CFAP46	cilia- and flagella-associated protein 46	Body	-61851	'NKX6-2	homeobox protein Nkx-6.2
GA	cg24468890	2.90E-05	0.007107775	FDR0.01	1	32757294	'HDAC1	histone deacetylase 1	TSS1500	-389	'HDAC1	histone deacetylase 1
GA	cg24468934	1.49E-06	0.000900387	FDR0.01	1	26880767	'RPS6KA1;MIR1976	ribosomal protein S6 kinase alpha-1;microRNA 1976	Body;Body;Body;TSS1500	-265	'MIR1976	microRNA 1976
GA	cg24487361	4.30E-05	0.009100322	FDR0.01	1	231473529	'EXOC8;SPRTN	exocyst complex component 8;sprT-like domain-containing protein Spartan	Body;TSS1500;TSS1500;TSS150	69	'EXOC8	exocyst complex component 8
GA	cg24503003	2.94E-10	1.75E-06	BF|FDR0.01	12	110682837	'			-36214	'ATP2A2	sarcoplasmic/endoplasmic reticulum calcium ATPase 2
GA	cg24504423	3.59E-05	0.008175615	FDR0.01	1	94374268	'GCLM	glutamate--cysteine ligase regulatory subunit	Body;Body	718	'GCLM	glutamate--cysteine ligase regulatory subunit
GA	cg24565747	4.38E-05	0.009224399	FDR0.01	2	240615835	'			-68718	'LOC150935	uncharacterized LOC150935
GA	cg24577594	1.81E-06	0.001034873	FDR0.01	1	230240631	'GALNT2	polypeptide N-acetylgalactosaminyltransferase 2 precursor	Body;Body	37647	'GALNT2	polypeptide N-acetylgalactosaminyltransferase 2 precursor
GA	cg24585377	2.96E-09	8.99E-06	BF|FDR0.01	1	26857774	'RPS6KA1	ribosomal protein S6 kinase alpha-1	Body	1523	'RPS6KA1	ribosomal protein S6 kinase alpha-1
GA	cg24594459	1.23E-07	0.000140828	FDR0.01	2	27478146	'SLC30A3	zinc transporter 3	3'UTR;3'UTR;3'UTR;3'UTR	7769	'SLC30A3	zinc transporter 3
GA	cg24601030	2.50E-05	0.006452624	FDR0.01	11	111847747	'DIXDC1	dixin isoform b	TES5000;Body;TSS1500	-285	'DIXDC1	dixin isoform b
GA	cg24605046	2.74E-08	4.52E-05	BF|FDR0.01	6	33245895	'RPS18;B3GALT4;WDR46	40S ribosomal protein S18;beta-1,3-galactosyltransferase 4;WD repeat-conta	TES5000;1stExon;TES5000;TES5	996	'B3GALT4	beta-1,3-galactosyltransferase 4
GA	cg24618514	8.93E-06	0.003165753	FDR0.01	3	194868507	'XXYLT1;XXYLT1-AS2	xyloside xylosyltransferase 1;XXYLT1 antisense RNA 2	Body;Body;TSS200;TSS200	-92	'XXYLT1-AS2	XXYLT1 antisense RNA 2
GA	cg24647428	1.95E-05	0.005503156	FDR0.01	3	100789257	'			-76923	'ABI3BP	target of Nesh-SH3 isoform 2 precursor
GA	cg24660675	7.37E-07	0.000536066	FDR0.01	10	135168850	'PRAP1;FUOM	proline-rich acidic protein 1 isoform 2 precursor;fucose mutarotase	TES5000;TES5000;3'UTR;3'UTR;	2673	'FUOM	fucose mutarotase
GA	cg24699699	2.20E-05	0.005949657	FDR0.01	1	93258316	'EVI5	ecotropic viral integration site 5 protein homolog isoform 1	TSS1500;TSS1500	-349	'EVI5	ecotropic viral integration site 5 protein homolog isoform 1
GA	cg24704287	2.64E-12	5.91E-08	BF|FDR0.01	19	13951481	'LOC284454;MIR24-2;MIR27A;MIR23A	;microRNA 24-2;microRNA 27a;microRNA 23a	Body;TSS5000;TSS5000;TSS500	2251	'LOC284454	
GA	cg24705939	1.95E-05	0.005503156	FDR0.01	8	30413864	'RBPMS	RNA-binding protein with multiple splicing	Body;Body;Body	-82232	'SMIM18	small integral membrane protein 18
GA	cg24706505	9.33E-06	0.003269095	FDR0.01	19	13211261	'NFIX;LYL1;TRMT1	nuclear factor 1 X-type;protein lyl-1;tRNA (guanine(26)-N(2))-dimethyltransf	TES5000;TES5000;TES5000;TES	2411	'LYL1	protein lyl-1
GA	cg24713122	4.17E-05	0.008952664	FDR0.01	11	46389164	'DGKZ	diacylglycerol kinase zeta	Body;Body;Body;Body;Body;Bo	6009	'DGKZ	diacylglycerol kinase zeta
GA	cg24716343	2.84E-06	0.001408963	FDR0.01	1	233431953	'PCNX2	pecanex-like protein 2 isoform 2	TSS1500;TSS1500	-482	'PCNX2	pecanex-like protein 2 isoform 2
GA	cg24717799	2.61E-06	0.001319172	FDR0.01	15	83680832	'C15orf40;BTBD1	UPF0235 protein C15orf40 isoform c;BTB/POZ domain-containing protein 1	TSS1500;TSS1500;TSS1500;TSS1	-441	'C15orf40	UPF0235 protein C15orf40 isoform c
GA	cg24718197	9.94E-10	4.11E-06	BF|FDR0.01	17	79485709	'			-5884	'ACTG1	actin, cytoplasmic 2
GA	cg24729928	7.28E-07	0.000532021	FDR0.01	12	31480184	'SINHCAF;FLJ13224	SIN3-HDAC complex-associated factor;uncharacterized LOC79857	TSS1500;TSS1500;TSS1500;TES	-1063	'SINHCAF	SIN3-HDAC complex-associated factor
GA	cg24737324	4.79E-05	0.009790278	FDR0.01	20	19867143	'RIN2	ras and Rab interactor 2	TSS200;TSS5000	-21	'RIN2	ras and Rab interactor 2
GA	cg24741873	7.09E-07	0.000521103	FDR0.01	10	82223998	'TSPAN14	tetraspanin-14	Body;Body;Body;Body;Body;Bo	-4020	'TSPAN14	tetraspanin-14
GA	cg24756378	9.93E-06	0.003412101	FDR0.01	14	33401638	'			-6446	'NPAS3	neuronal PAS domain-containing protein 3 isoform 3
GA	cg24760467	6.69E-06	0.002608642	FDR0.01	10	102760784	'LZTS2	leucine zipper putative tumor suppressor 2	Body;Body;Body;TSS1500	1220	'LZTS2	leucine zipper putative tumor suppressor 2
GA	cg24761525	3.89E-05	0.008614143	FDR0.01	17	46651745	'HOXB3;HOXB4	homeobox protein Hox-B3;homeobox protein Hox-B4	5'UTR;Body;Body;TES5000	65	'HOXB3	homeobox protein Hox-B3
GA	cg24764793	1.69E-06	0.000984798	FDR0.01	6	152126745	'ESR1	estrogen receptor	Body;TSS200;TSS200;TSS200;TS	-62	'ESR1	estrogen receptor
GA	cg24772162	2.14E-05	0.005860161	FDR0.01	11	67059497	'ANKRD13D	ankyrin repeat domain-containing protein 13D	Body;Body	2724	'ANKRD13D	ankyrin repeat domain-containing protein 13D
GA	cg24779381	3.10E-05	0.007375086	FDR0.01	15	74466794	'ISLR	immunoglobulin superfamily containing leucine-rich repeat protein precurso	Body;TSS200	-93	'ISLR	immunoglobulin superfamily containing leucine-rich repeat protein precursor
GA	cg24783356	2.85E-05	0.007028221	FDR0.01	11	98891211	'CNTN5	contactin-5 isoform 2 precursor	TSS1500;TSS1500;TSS1500	-467	'CNTN5	contactin-5 isoform 2 precursor
GA	cg24784730	1.09E-07	0.000129826	BF|FDR0.01	12	125225655	'			98562	'SCARB1	scavenger receptor class B member 1
GA	cg24803255	5.71E-06	0.002334586	FDR0.01	13	114807060	'RASA3	ras GTPase-activating protein 3	Body;Body;Body	36370	'RASA3	ras GTPase-activating protein 3
GA	cg24809544	8.93E-07	0.000616659	FDR0.01	19	51727921	'CD33	myeloid cell surface antigen CD33 isoform 2	TSS1500;TSS1500;TSS1500	-414	'CD33	myeloid cell surface antigen CD33 isoform 2
GA	cg24847254	1.45E-05	0.004486197	FDR0.01	5	101297137	'			334851	'SLCO4C1	solute carrier organic anion transporter family member 4C1
GA	cg24880818	4.68E-07	0.000384991	FDR0.01	1	154167100	'TPM3;MIR190B;C1orf189	tropomyosin alpha-3 chain;microRNA 190b;uncharacterized protein C1orf18	TSS5000;TSS5000;TSS5000;TSS	-881	'MIR190B	microRNA 190b
GA	cg24908802	3.33E-05	0.007721633	FDR0.01	5	144726601	'			488304	'PRELID2	PRELI domain-containing protein 2 isoform c

Trait	#cpg	p	fdr	significance	chr	pos.hg19	gene.Illumina	gene.description	locations	dist2TSS	gene.nearest	gene.description
**GA**	cg24924577	1.87E-05	0.005356826	FDR0.01	15	90735148	'SEMA4B	semaphorin-4B isoform 1 precursor	Body;Body;Body;TSS200	-4	'SEMA4B	semaphorin-4B isoform 1 precursor
**GA**	cg24951886	2.39E-12	5.86E-08	BF|FDR0.01	10	13526236	'BEND7	BEN domain-containing protein 7	Body;Body;Body;Body	18740	'BEND7	BEN domain-containing protein 7
**GA**	cg24956866	5.07E-06	0.002128915	FDR0.01	7	134464129	'CALD1	caldesmon	TSS1500;TSS1500;TSS1500	-255	'CALD1	caldesmon
**GA**	cg25009965	2.49E-12	5.86E-08	BF|FDR0.01	11	114031120	'ZBTB16	zinc finger and BTB domain-containing protein 16	Body;Body;Body;Body;Body	97188	'ZBTB16	zinc finger and BTB domain-containing protein 16
**GA**	cg25027788	1.66E-06	0.000974714	FDR0.01	12	6167002	'VWF	von Willebrand factor precursor	Body	-17653	'SNORA120	small nucleolar RNA, H/ACA box 120
**GA**	cg25030888	2.63E-05	0.006674668	FDR0.01	1	67156909	'SGIP1	SH3-containing GRB2-like protein 3-interacting protein 1	Body;Body;Body;Body	-61232	'TCTEX1D1	tctex1 domain-containing protein 1
**GA**	cg25045219	7.76E-09	1.83E-05	BF|FDR0.01	17	263096	'LOC105371430;C17orf97	uncharacterized LOC105371430;protein LIAT1	Body;Body	718	'LOC105371430	uncharacterized LOC105371430
**GA**	cg25053142	4.92E-05	0.009952864	FDR0.01	10	775353	'			-39745	'DIP2C	disco-interacting protein 2 homolog C
**GA**	cg25061349	7.74E-07	0.000557305	FDR0.01	6	43337791	'ZNF318	zinc finger protein 318	TSS1500	-406	'ZNF318	zinc finger protein 318
**GA**	cg25072666	2.41E-07	0.000232951	FDR0.01	17	79533843	'NPLOC4	nuclear protein localization protein 4 homolog	Body;Body;Body;TSS1500	-215	'NPLOC4	nuclear protein localization protein 4 homolog
**GA**	cg25095518	3.14E-05	0.00742844	FDR0.01	2	98329337	'ZAP70	tyrosine-protein kinase ZAP-70	TSS1500	-693	'ZAP70	tyrosine-protein kinase ZAP-70
**GA**	cg25096368	9.03E-08	0.000113076	BF|FDR0.01	12	54150616	'CISTR	chondrogenesis-associated transcript	TSS5000;Body	202	'CISTR	chondrogenesis-associated transcript
**GA**	cg25097610	4.37E-06	0.00191161	FDR0.01	4	4763360	'LOC101928279	uncharacterized LOC101928279	TSS200	-126	'LOC101928279	uncharacterized LOC101928279
**GA**	cg25117875	4.94E-05	0.009970378	FDR0.01	12	14413581	'			-104984	'ATF7IP	activating transcription factor 7-interacting protein 1
**GA**	cg25124966	3.26E-06	0.001550582	FDR0.01	11	129723987	'TMEM45B	transmembrane protein 45B	Body;Body;Body;Body	38245	'TMEM45B	transmembrane protein 45B
**GA**	cg25144135	1.90E-06	0.001066227	FDR0.01	1	1144012	'TNFRSF18;TNFRSF4	tumor necrosis factor receptor superfamily member 18 precursor;tumor nec	TSS5000;TSS5000;TSS5000;TES	-2040	'TNFRSF18	tumor necrosis factor receptor superfamily member 18 precursor
**GA**	cg25145687	1.80E-05	0.005244066	FDR0.01	11	1606664	'KRTAP5-AS1;KRTAP5-1	KRTAP5-1/KRTAP5-2 antisense RNA 1;keratin-associated protein 5-1	Body;TSS200	-151	'KRTAP5-1	keratin-associated protein 5-1
**GA**	cg25150953	1.38E-05	0.004333856	FDR0.01	4	41540229	'LIMCH1	LIM and calponin homology domains-containing protein 1 isoform j	Body;Body;Body;Body;Body;Bo	56	'LIMCH1	LIM and calponin homology domains-containing protein 1 isoform j
**GA**	cg25162533	5.82E-06	0.002361871	FDR0.01	17	81032067	'			-5417	'METRNL	meteorin-like protein precursor
**GA**	cg25165932	1.19E-05	0.003911308	FDR0.01	12	109025846	'SELPLG;MIR4496	P-selectin glycoprotein ligand 1 precursor;microRNA 4496	5'UTR;Body;TSS5000	11	'SELPLG	P-selectin glycoprotein ligand 1 precursor
**GA**	cg25174412	4.15E-10	2.21E-06	BF|FDR0.01	12	105803653	'			79185	'C12orf75	overexpressed in colon carcinoma 1 protein
**GA**	cg25202280	2.12E-06	0.00114986	FDR0.01	17	79521089	'FAAP100;NPLOC4	Fanconi anemia core complex-associated protein 100;nuclear protein localiza	TSS5000;TSS5000;TES5000;TES	-1710	'FAAP100	Fanconi anemia core complex-associated protein 100
**GA**	cg25207952	1.65E-05	0.004914142	FDR0.01	15	75499952	'C15orf39	uncharacterized protein C15orf39	1stExon	5730	'C15orf39	uncharacterized protein C15orf39
**GA**	cg25211403	1.12E-05	0.003715647	FDR0.01	21	46975897	'SLC19A1	reduced folate transporter	Body	7042	'SLC19A1	reduced folate transporter
**GA**	cg25236791	4.92E-05	0.009954702	FDR0.01	3	52302252	'WDR82;MIRLET7G	WD repeat-containing protein 82;microRNA let-7g	Body;TES5000	125	'MIRLET7G	microRNA let-7g
**GA**	cg25247351	2.02E-06	0.001107518	FDR0.01	6	32908794	'HLA-DMB	HLA class II histocompatibility antigen, DM beta chain precursor	5'UTR	11	'HLA-DMB	HLA class II histocompatibility antigen, DM beta chain precursor
**GA**	cg25254074	1.04E-07	0.000127281	BF|FDR0.01	6	41607326	'MDFI	myoD family inhibitor	Body;Body;Body;Body	1121	'MDFI	myoD family inhibitor
**GA**	cg25254170	2.90E-07	0.00026785	FDR0.01	6	36954695	'MTCH1	mitochondrial carrier homolog 1 isoform 1	TSS1500;TSS1500;TSS1500	-368	'MTCH1	mitochondrial carrier homolog 1 isoform 1
**GA**	cg25266281	5.99E-06	0.00241807	FDR0.01	2	242904768	'LINC01237	long intergenic non-protein coding RNA 1237	Body	-8065	'LINC01238	long intergenic non-protein coding RNA 1238
**GA**	cg25303150	1.33E-06	0.000824422	FDR0.01	10	134211874	'PWWP2B	PWWP domain-containing protein 2B	Body;Body	1171	'PWWP2B	PWWP domain-containing protein 2B
**GA**	cg25325512	3.76E-05	0.008433829	FDR0.01	6	37142220	'PIM1	serine/threonine-protein kinase pim-1	3'UTR;3'UTR	4293	'PIM1	serine/threonine-protein kinase pim-1
**GA**	cg25363679	3.04E-05	0.007290638	FDR0.01	12	71031202	'PTPRB;PTPRR	receptor-type tyrosine-protein phosphatase beta;receptor-type tyrosine-pro	5'UTR;5'UTR;TES5000;TES5000;	7	'PTPRB	receptor-type tyrosine-protein phosphatase beta
**GA**	cg25370658	2.32E-06	0.001232955	FDR0.01	6	7673306	'			-53025	'BMP6	bone morphogenetic protein 6 precursor
**GA**	cg25426302	1.86E-05	0.005354008	FDR0.01	6	32120826	'PRRT1;LOC100507547;PPT2;PPT2-EGFL8	proline-rich transmembrane protein 1;uncharacterized LOC100507547;lysos	5'UTR;TSS1500;Body;Body;Body	34	'PRRT1	proline-rich transmembrane protein 1
**GA**	cg25433552	1.15E-05	0.003800698	FDR0.01	5	159560548	'			-14091	'PWWP2A	PWWP domain-containing protein 2A isoform f
**GA**	cg25456477	1.82E-07	0.000188165	FDR0.01	12	86230367	'RASSF9	ras association domain-containing protein 9	TSS200	-180	'RASSF9	ras association domain-containing protein 9
**GA**	cg25461186	8.20E-09	1.88E-05	BF|FDR0.01	12	122518089	'MLXIP	MLX-interacting protein isoform 2	Body	1428	'MLXIP	MLX-interacting protein isoform 2
**GA**	cg25461290	4.02E-05	0.008750459	FDR0.01	8	142276422	'			-11694	'SLC45A4	solute carrier family 45 member 4 isoform 3
**GA**	cg25472804	2.22E-07	0.000218344	FDR0.01	8	128219418	'CCAT1	colon cancer associated transcript 1	TES5000	-9546	'CASC19	cancer susceptibility 19
**GA**	cg25484139	1.82E-05	0.0052859	FDR0.01	3	13008273	'IQSEC1	IQ motif and SEC7 domain-containing protein 1	Body;Body;Body	957	'IQSEC1	IQ motif and SEC7 domain-containing protein 1
**GA**	cg25486824	2.31E-05	0.006126008	FDR0.01	11	13983790	'SPON1	spondin-1 precursor	TSS1500	-479	'SPON1	spondin-1 precursor
**GA**	cg25494227	4.73E-07	0.000386799	FDR0.01	12	10331614	'TMEM52B	transmembrane protein 52B	Body;TSS200	-16	'TMEM52B	transmembrane protein 52B
**GA**	cg25509184	2.73E-06	0.001362799	FDR0.01	7	117119382	'CFTR	cystic fibrosis transmembrane conductance regulator	TSS1500	-696	'CFTR	cystic fibrosis transmembrane conductance regulator
**GA**	cg25552317	3.09E-05	0.007364993	FDR0.01	12	92379065	'LINC01619	long intergenic non-protein coding RNA 1619	Body;Body;Body;Body;Body	156424	'LINC01619	long intergenic non-protein coding RNA 1619
**GA**	cg25592206	8.36E-07	0.000589047	FDR0.01	1	51439348	'CDKN2C	cyclin-dependent kinase 4 inhibitor C	Body;Body	3432	'CDKN2C	cyclin-dependent kinase 4 inhibitor C
**GA**	cg25599242	2.75E-09	8.53E-06	BF|FDR0.01	11	63304337	'PLAAT4	phospholipase A and acyltransferase 4	5'UTR	58	'PLAAT4	phospholipase A and acyltransferase 4
**GA**	cg25611723	8.36E-08	0.000106023	BF|FDR0.01	18	10453624	'APCDD1	protein APCDD1 precursor	TSS1500	-1007	'APCDD1	protein APCDD1 precursor
**GA**	cg25630910	2.48E-06	0.001280311	FDR0.01	12	123753186	'CDK2AP1	cyclin-dependent kinase 2-associated protein 1	TSS1500;Body;Body;Body;Body	-385	'CDK2AP1	cyclin-dependent kinase 2-associated protein 1
**GA**	cg25636159	2.67E-07	0.000251907	FDR0.01	22	40743859	'ADSL	adenylosuccinate lyase	Body;Body;Body;Body;Body	1356	'ADSL	adenylosuccinate lyase
**GA**	cg25663304	3.59E-05	0.008185792	FDR0.01	5	176172918	'LINC01574	long intergenic non-protein coding RNA 1574	TES5000	2713	'LINC01574	long intergenic non-protein coding RNA 1574
**GA**	cg25671716	1.51E-05	0.004635914	FDR0.01	12	96441714	'LTA4H	leukotriene A-4 hydrolase	TSS5000;TSS5000	-4416	'LTA4H	leukotriene A-4 hydrolase
**GA**	cg25673591	3.66E-05	0.008278686	FDR0.01	17	39945814	'JUP	junction plakoglobin	TSS5000;TSS5000;TSS5000;TSS	-2794	'JUP	junction plakoglobin
**GA**	cg25673593	1.92E-05	0.005452597	FDR0.01	17	37778125	'			-5079	'PPP1R1B	protein phosphatase 1 regulatory subunit 1B
**GA**	cg25718383	2.93E-05	0.007138082	FDR0.01	7	130020096	'CPA1	carbamoyl-phosphate synthase (glutamine-hydrolyzing) CPA1	TSS1500	-238	'CPA1	carbamoyl-phosphate synthase (glutamine-hydrolyzing) CPA1
**GA**	cg25790850	2.05E-06	0.001119665	FDR0.01	22	39541983	'CBX7	chromobox protein homolog 7	Body;Body;Body	6702	'CBX7	chromobox protein homolog 7
**GA**	cg25823926	1.45E-06	0.000880223	FDR0.01	12	94153360	'CRADD	death domain-containing protein CRADD	Body;Body;Body;Body	-21761	'LOC101928731	uncharacterized LOC101928731
**GA**	cg25827710	5.03E-06	0.002118166	FDR0.01	13	46742802	'LCP1	plastin-2	Body	13510	'LCP1	plastin-2
**GA**	cg25838150	2.37E-05	0.006217736	FDR0.01	11	77259596	'			-22537	'LOC646029	uncharacterized LOC646029
**GA**	cg25898146	1.45E-06	0.000879909	FDR0.01	8	1992746	'MYOM2	M-protein, striated muscle	TSS1500	-414	'MYOM2	M-protein, striated muscle
**GA**	cg25903143	3.26E-07	0.000295783	FDR0.01	11	68920466	'LOC338694;SMIM38	uncharacterized LOC338694;small integral membrane protein 38	Body;TSS5000;TSS5000	-2936	'SMIM38	small integral membrane protein 38
**GA**	cg25913882	2.28E-05	0.006078006	FDR0.01	10	17067842	'CUBN	cubilin precursor	Body	103968	'CUBN	cubilin precursor
**GA**	cg25915539	2.81E-07	0.000261963	FDR0.01	4	114214080	'ANK2	ankyrin-2 isoform 38	Body;Body;Body;Body;Body;Bo	86	'ANK2	ankyrin-2 isoform 38
**GA**	cg25934954	2.06E-08	3.50E-05	BF|FDR0.01	11	64567121	'MAP4K2;MEN1	mitogen-activated protein kinase kinase kinase kinase 2;menin	Body;Body;TES5000;TES5000;T	3565	'MAP4K2	mitogen-activated protein kinase kinase kinase kinase 2
**GA**	cg25936876	2.82E-05	0.006964458	FDR0.01	8	6484687	'MCPH1;MCPH1-AS1;MIR8055	microcephalin;MCPH1 antisense RNA 1;microRNA 8055	Body;Body;Body;Body;Body;TS	-4946	'MIR8055	microRNA 8055
**GA**	cg25940447	1.49E-05	0.004585527	FDR0.01	5	141391533	'GNPDA1	glucosamine-6-phosphate isomerase 1	1stExon	1059	'GNPDA1	glucosamine-6-phosphate isomerase 1
**GA**	cg25960854	4.32E-06	0.001899024	FDR0.01	19	1160184	'SBNO2	protein strawberry notch homolog 2	Body	14083	'SBNO2	protein strawberry notch homolog 2
**GA**	cg25983531	3.80E-05	0.008501655	FDR0.01	7	36765142	'AOAH	acyloxyacyl hydrolase precursor	TSS1500;TSS1500;TSS1500	-988	'AOAH	acyloxyacyl hydrolase precursor
**GA**	cg26005232	1.08E-05	0.003613193	FDR0.01	1	27247829	'NUDC	nuclear migration protein nudC	TSS1500	-404	'NUDC	nuclear migration protein nudC
**GA**	cg26027170	1.40E-07	0.000155104	FDR0.01	17	37394705	'LOC101929578	transcription factor CP2-like	Body	683	'LOC101929578	transcription factor CP2-like
**GA**	cg26077811	2.25E-06	0.001210464	FDR0.01	11	119232263	'USP2	ubiquitin carboxyl-terminal hydrolase 2	Body;Body;Body	2655	'USP2	ubiquitin carboxyl-terminal hydrolase 2
**GA**	cg26133399	7.02E-07	0.000518986	FDR0.01	12	62583974	'TAFA2	chemokine-like protein TAFA-2 precursor	Body	2646	'TAFA2	chemokine-like protein TAFA-2 precursor
**GA**	cg26152923	2.27E-06	0.001217899	FDR0.01	19	18284120	'PIK3R2;IFI30	phosphatidylinositol 3-kinase regulatory subunit beta;gamma-interferon-ind	TES5000;TES5000;TES5000;TSS	-502	'IFI30	gamma-interferon-inducible lysosomal thiol reductase preproprotein
**GA**	cg26160218	1.88E-05	0.00535964	FDR0.01	3	24538388	'THRB;THRB-AS1	thyroid hormone receptor beta;THRB antisense RNA 1	TSS5000;TSS5000;TSS5000;TSS	-1189	'THRB	thyroid hormone receptor beta
**GA**	cg26161820	4.16E-06	0.001859025	FDR0.01	17	37792777	'PPP1R1B;STARD3	protein phosphatase 1 regulatory subunit 1B;stAR-related lipid transfer prote	3'UTR;3'UTR;3'UTR;TSS1500;TS	-555	'STARD3	stAR-related lipid transfer protein 3
**GA**	cg26189283	6.65E-08	8.77E-05	BF|FDR0.01	1	155109378	'EFNA1;SLC50A1;DPM3	ephrin-A1 isoform b precursor;sugar transporter SWEET1;dolichol-phosphate	TES5000;TES5000;Body;Body;B	1051	'SLC50A1	sugar transporter SWEET1
**GA**	cg26191447	5.67E-07	0.000439896	FDR0.01	2	191656418	'			-89128	'GLS	glutaminase kidney isoform, mitochondrial
**GA**	cg26201213	6.03E-06	0.002426612	FDR0.01	10	131265796	'MGMT	methylated-DNA--protein-cysteine methyltransferase	Body	292	'MGMT	methylated-DNA--protein-cysteine methyltransferase
**GA**	cg26202340	1.04E-05	0.003510259	FDR0.01	11	126152357	'FOXRED1;TIRAP	FAD-dependent oxidoreductase domain-containing protein 1;toll/interleukin	TES5000;TES5000;TES5000;TSS	-624	'TIRAP	toll/interleukin-1 receptor domain-containing adapter protein
**GA**	cg26207995	4.71E-05	0.009666218	FDR0.01	17	58045920	'RNFT1;TBC1D3P1-DHX40P1;RNFT1-DT	E3 ubiquitin-protein ligase RNFT1;TBC1D3P1-DHX40P1 readthrough, transcri	TSS5000;Body;Body;Body	3464	'RNFT1-DT	RNFT1 divergent transcript
**GA**	cg26215727	4.44E-05	0.009294057	FDR0.01	12	6485537	'SCNN1A;LTBR	amiloride-sensitive sodium channel subunit alpha;tumor necrosis factor rece	Body;TSS1500;TSS1500;Body	-808	'SCNN1A	amiloride-sensitive sodium channel subunit alpha
**GA**	cg26219092	8.41E-08	0.000106023	BF|FDR0.01	8	134388022	'			-78475	'NDRG1	protein NDRG1
**GA**	cg26233209	2.14E-06	0.001158655	FDR0.01	5	115178764	'ATG12;AP3S1	ubiquitin-like protein ATG12;AP-3 complex subunit sigma-1	TSS5000;TSS5000;TSS5000;TSS	581	'AP3S1	AP-3 complex subunit sigma-1
**GA**	cg26265787	4.72E-05	0.009666218	FDR0.01	19	10491889	'TYK2	non-receptor tyrosine-protein kinase TYK2	TSS1500	-641	'TYK2	non-receptor tyrosine-protein kinase TYK2
**GA**	cg26283879	4.87E-05	0.009902713	FDR0.01	8	142255356	'SLC45A4	solute carrier family 45 member 4 isoform 3	Body	-9309	'LOC105375787	uncharacterized LOC105375787
**GA**	cg26297819	1.00E-05	0.003428544	FDR0.01	22	41763280	'TEF	transcription factor VBP	TSS200	-56	'TEF	transcription factor VBP
**GA**	cg26303870	2.28E-05	0.006078006	FDR0.01	14	69443683	'ACTN1;ACTN1-AS1	alpha-actinin-1;	Body;Body;Body;TSS5000	2336	'ACTN1	alpha-actinin-1
**GA**	cg26360695	4.51E-05	0.009396605	FDR0.01	6	39197537	'KCNK5	potassium channel subfamily K member 5	TSS1500	-286	'KCNK5	potassium channel subfamily K member 5

Trait	#cpg	p	fdr	significance	chr	pos.hg19	gene.Illumina	gene.description	locations	dist2TSS	gene.nearest	gene.description
GA	cg26445608	3.36E-05	0.007778344	FDR0.01	20	2780114	'CPXM1	probable carboxypeptidase X1 precursor	Body;Body	1159	'CPXM1	probable carboxypeptidase X1 precursor
GA	cg26451188	2.21E-05	0.005949657	FDR0.01	17	8295971	'RNF222	RING finger protein 222	3'UTR	5174	'RNF222	RING finger protein 222
GA	cg26463621	2.62E-09	8.28E-06	BF|FDR0.01	18	13219222	'LDLRAD4	low-density lipoprotein receptor class A domain-containing protein 4	Body	464	'LDLRAD4	low-density lipoprotein receptor class A domain-containing protein 4
GA	cg26467528	3.95E-05	0.008669363	FDR0.01	6	31632248	'C6orf47;GPANK1;CSNK2B	uncharacterized protein C6orf47;G patch domain and ankyrin repeat-contair	TSS5000;1stExon;1stExon;1stEx	526	'GPANK1	G patch domain and ankyrin repeat-containing protein 1
GA	cg26472802	2.69E-05	0.006763213	FDR0.01	21	45713719	'AIRE	autoimmune regulator	Body	-6216	'PFKL	ATP-dependent 6-phosphofructokinase, liver type
GA	cg26472910	5.02E-11	4.88E-07	BF|FDR0.01	13	28673762	'FLT3	receptor-type tyrosine-protein kinase FLT3 precursor	Body;Body	951	'FLT3	receptor-type tyrosine-protein kinase FLT3 precursor
GA	cg26482939	4.26E-05	0.009067438	FDR0.01	19	3136710	'GNA15;LOC100996351	guanine nucleotide-binding protein subunit alpha-15;	Body;TES5000	680	'GNA15	guanine nucleotide-binding protein subunit alpha-15
GA	cg26500033	3.39E-05	0.007807701	FDR0.01	6	166585954	'TBXT	T-box transcription factor T	TSS5000;TSS5000;TSS5000;TSS	-3797	'TBXT	T-box transcription factor T
GA	cg26518660	7.67E-06	0.002863133	FDR0.01	1	79728608	'			-256193	'ADGRL4	adhesion G protein-coupled receptor L4 precursor
GA	cg26532627	4.86E-05	0.009890677	FDR0.01	17	43339744	'MAP3K14-AS1;SPATA32;MAP3K14	MAP3K14 antisense RNA 1;spermatogenesis-associated protein 32;mitogen-	Body;Body;Body;TSS1500;Body	-283	'SPATA32	spermatogenesis-associated protein 32
GA	cg26542567	1.89E-08	3.29E-05	BF|FDR0.01	8	142986670	'			-118996	'MIR1302-7	microRNA 1302-7
GA	cg26596719	4.58E-09	1.25E-05	BF|FDR0.01	7	142986429	'LOC105375545;TMEM139;CASP2	;transmembrane protein 139;caspase-2	TSS5000;TES5000;TES5000;TES	-251	'CASP2	caspase-2
GA	cg26606789	1.11E-05	0.003697543	FDR0.01	21	44253219	'			46166	'WDR4	tRNA (guanine-N(7)-)-methyltransferase non-catalytic subunit WDR4 isoform 2
GA	cg26615176	4.37E-11	4.44E-07	BF|FDR0.01	11	65150416	'SLC25A45;FRMD8	solute carrier family 25 member 45;FERM domain-containing protein 8	TSS1500;TSS1500;TSS1500;TSS	-271	'SLC25A45	solute carrier family 25 member 45
GA	cg26619671	4.53E-09	1.24E-05	BF|FDR0.01	16	89638625	'CPNE7	copine-7	TSS5000;TSS5000	-3540	'CPNE7	copine-7
GA	cg26647303	4.46E-05	0.009318364	FDR0.01	19	2041955	'MKNK2	MAP kinase-interacting serine/threonine-protein kinase 2	Body;Body	9288	'MKNK2	MAP kinase-interacting serine/threonine-protein kinase 2
GA	cg26682407	1.24E-05	0.004023256	FDR0.01	13	113660246	'MCF2L	guanine nucleotide exchange factor DBS	Body;Body;Body;Body;Body;Bo	4219	'MCF2L	guanine nucleotide exchange factor DBS
GA	cg26700215	4.06E-06	0.001832219	FDR0.01	8	67070006	'TRIM55	tripartite motif-containing protein 55	Body;Body;Body;Body	20693	'CRH	corticoliberin precursor
GA	cg26706803	2.66E-13	1.19E-08	BF|FDR0.01	16	50293530	'			-6931	'ADCY7	adenylate cyclase type 7
GA	cg26723847	8.23E-06	0.003015738	FDR0.01	11	134095652	'NCAPD3;VPS26B	condensin-2 complex subunit D3;vacuolar protein sorting-associated protein	TSS5000;TSS5000;TSS5000;TSS	-297	'NCAPD3	condensin-2 complex subunit D3
GA	cg26749414	1.98E-06	0.001088412	FDR0.01	19	46807272	'HIF3A	hypoxia-inducible factor 3-alpha	Body;1stExon;1stExon;Body	397	'HIF3A	hypoxia-inducible factor 3-alpha
GA	cg26796095	2.16E-09	7.31E-06	BF|FDR0.01	19	5625046	'SAFB2;SAFB	scaffold attachment factor B2;scaffold attachment factor B1	TSS5000;Body;Body;Body;Body	1953	'SAFB	scaffold attachment factor B1
GA	cg26796518	7.11E-06	0.002711257	FDR0.01	12	6184516	'VWF	von Willebrand factor precursor	Body	-35167	'SNORA120	small nucleolar RNA, H/ACA box 120
GA	cg26799474	1.09E-09	4.42E-06	BF|FDR0.01	2	202098951	'CASP10;CASP8	caspase-10;caspase-8	TES5000;TES5000;Body;Body;B	766	'CASP8	caspase-8
GA	cg26800893	1.41E-08	2.74E-05	BF|FDR0.01	11	67184596	'CARNS1	carnosine synthase 1 isoform 2	Body;Body	1448	'CARNS1	carnosine synthase 1 isoform 2
GA	cg26809643	4.90E-05	0.009942248	FDR0.01	7	64023824	'ZNF680	zinc finger protein 680 isoform 2	TSS1500;TSS1500	-319	'ZNF680	zinc finger protein 680 isoform 2
GA	cg26818820	5.68E-09	1.44E-05	BF|FDR0.01	5	135468000	'SMAD5-AS1;SMAD5	SMAD5 antisense RNA 1;mothers against decapentaplegic homolog 5	Body;TSS1500;TSS1500;TSS150	-533	'SMAD5	mothers against decapentaplegic homolog 5
GA	cg26828842	4.26E-06	0.001878844	FDR0.01	18	67952086	'SOCS6	suppressor of cytokine signaling 6	TSS5000	-4194	'SOCS6	suppressor of cytokine signaling 6
GA	cg26833169	3.19E-05	0.007487708	FDR0.01	11	14994045	'CALCA	calcitonin isoform 1 preproprotein	TSS200;TSS1500;TSS1500;TSS2	-148	'CALCA	calcitonin isoform 1 preproprotein
GA	cg26836479	6.71E-06	0.002611536	FDR0.01	19	42706353	'DEDD2	DNA-binding death effector domain-containing protein 2	Body;Body;Body;Body;Body;Bo	15476	'DEDD2	DNA-binding death effector domain-containing protein 2
GA	cg26851141	4.79E-06	0.002042069	FDR0.01	16	58426274	'GINS3	DNA replication complex GINS protein PSF3	TSS200;TSS200;TSS200	-100	'GINS3	DNA replication complex GINS protein PSF3
GA	cg26861960	3.57E-05	0.008134104	FDR0.01	12	104659097	'TXNRD1	thioredoxin reductase 1, cytoplasmic	Body	-21712	'TXNRD1	thioredoxin reductase 1, cytoplasmic
GA	cg26878655	1.15E-06	0.000743425	FDR0.01	17	38481877	'RARA	retinoic acid receptor alpha	Body;Body;Body	7381	'RARA	retinoic acid receptor alpha
GA	cg26908825	2.13E-05	0.00584688	FDR0.01	6	1635818	'GMDS	GDP-mannose 4,6 dehydratase	Body;Body	25669	'FOXC1	forkhead box protein C1
GA	cg26916780	1.28E-05	0.004110581	FDR0.01	15	64889554	'ZNF609	zinc finger protein 609	Body	105926	'OAZ2	ornithine decarboxylase antizyme 2
GA	cg26925644	4.30E-06	0.00189565	FDR0.01	11	118476517	'ARCN1;PHLDB1	coatomer subunit delta;pleckstrin homology-like domain family B member 1	TES5000;TES5000;TSS1500;TSS	-633	'PHLDB1	pleckstrin homology-like domain family B member 1
GA	cg26929272	1.04E-05	0.003510259	FDR0.01	10	88295210	'			-13661	'WAPL	wings apart-like protein homolog isoform 2
GA	cg26933683	9.42E-07	0.000643996	FDR0.01	17	61918738	'SMARCD2	SWI/SNF-related matrix-associated actin-dependent regulator of chromatin	TSS5000;Body;Body	1088	'SMARCD2	SWI/SNF-related matrix-associated actin-dependent regulator of chromatin subfamily D member 2
GA	cg26942169	3.55E-06	0.001645448	FDR0.01	6	36566714	'SRSF3	serine/arginine-rich splicing factor 3	Body;Body	4576	'SRSF3	serine/arginine-rich splicing factor 3
GA	cg26956245	1.09E-07	0.000129873	BF|FDR0.01	7	30518972	'NOD1	nucleotide-binding oligomerization domain-containing protein 1	TSS1500;TSS1500;TSS1500	-490	'NOD1	nucleotide-binding oligomerization domain-containing protein 1
GA	cg26980034	7.34E-06	0.002771133	FDR0.01	6	53530624	'KLHL31	kelch-like protein 31	TSS200	-70	'KLHL31	kelch-like protein 31
GA	cg26981076	3.15E-05	0.007443746	FDR0.01	11	59824116	'MS4A3	membrane-spanning 4-domains subfamily A member 3 isoform c	5'UTR;TSS200;TSS200	16	'MS4A3	membrane-spanning 4-domains subfamily A member 3 isoform c
GA	cg27022558	1.77E-05	0.005185963	FDR0.01	11	64141041	'RPS6KA4;MIR1237	ribosomal protein S6 kinase alpha-4;microRNA 1237	TES5000;TES5000;TES5000;TES	4968	'MIR1237	microRNA 1237
GA	cg27024992	3.38E-06	0.001593362	FDR0.01	2	48094603	'FBXO11	F-box only protein 11	Body;Body	21255	'FBXO11	F-box only protein 11
GA	cg27059598	1.38E-05	0.004342185	FDR0.01	10	21797661	'SKIDA1	SKI/DACH domain-containing protein 1	TES5000	-11472	'MIR1915HG	MIR1915 host gene
GA	cg27066989	3.07E-08	4.92E-05	BF|FDR0.01	13	51640362	'GUCY1B2	guanylate cyclase 1 soluble subunit beta 2 (pseudogene)	TSS200	-69	'GUCY1B2	guanylate cyclase 1 soluble subunit beta 2 (pseudogene)
GA	cg27083484	9.52E-06	0.003315348	FDR0.01	5	122506787	'PRDM6	putative histone-lysine N-methyltransferase PRDM6	Body	-80776	'LOC105379152	uncharacterized LOC105379152
GA	cg27107685	9.83E-08	0.000120778	BF|FDR0.01	7	30590918	'GARS-DT		Body	26477	'GARS-DT	
GA	cg27110277	1.74E-06	0.001005319	FDR0.01	8	16860121	'FGF20	fibroblast growth factor 20	TSS1500	-267	'FGF20	fibroblast growth factor 20
GA	cg27113548	5.47E-06	0.002260072	FDR0.01	14	106354912	'			-28925	'FAM30A	family with sequence similarity 30 member A
GA	cg27132471	7.74E-09	1.83E-05	BF|FDR0.01	15	92613280	'SLCO3A1	solute carrier organic anion transporter family member 3A1	Body;Body;Body	215747	'SLCO3A1	solute carrier organic anion transporter family member 3A1
GA	cg27143082	4.86E-06	0.002065741	FDR0.01	6	31744628	'VWA7;VARS1	von Willebrand factor A domain-containing protein 7 precursor;valine--tRNA	Body;TES5000	477	'VWA7	von Willebrand factor A domain-containing protein 7 precursor
GA	cg27143204	1.51E-07	0.000164206	FDR0.01	5	54530375	'CCNO	cyclin-O	TSS1500;TSS5000;TSS1500;TSS	-830	'CCNO	cyclin-O
GA	cg27145034	4.59E-08	6.55E-05	BF|FDR0.01	16	19179928	'SYT17	synaptotagmin-17	Body;5'UTR;TSS5000	367	'SYT17	synaptotagmin-17
GA	cg27145042	3.83E-08	5.78E-05	BF|FDR0.01	16	25026956	'ARHGAP17	rho GTPase-activating protein 17 isoform 2	TSS1500;TSS1500	-266	'ARHGAP17	rho GTPase-activating protein 17 isoform 2
GA	cg27151174	1.16E-05	0.003814687	FDR0.01	16	30367227	'CD2BP2;TBC1D10B	CD2 antigen cytoplasmic tail-binding protein 2;TBC1 domain family member	TSS1500;TSS1500;TES5000	-598	'CD2BP2	CD2 antigen cytoplasmic tail-binding protein 2
GA	cg27158340	6.03E-06	0.002426612	FDR0.01	14	105603389	'JAG2	protein jagged-2 isoform b precursor	TES5000;TES5000	13812	'MIR6765	microRNA 6765
GA	cg27165456	1.74E-08	3.13E-05	BF|FDR0.01	7	1782577	'ELFN1;ELFN1-AS1	protein ELFN1 precursor;ELFN1 antisense RNA 1	Body;TSS1500;TSS1500;TSS150	-631	'ELFN1-AS1	ELFN1 antisense RNA 1
GA	cg27183791	1.45E-05	0.004486197	FDR0.01	16	89381904	'ANKRD11	ankyrin repeat domain-containing protein 11	Body;Body;Body;Body	-5636	'LOC100287036	uncharacterized LOC100287036
GA	cg27194211	5.73E-06	0.002336898	FDR0.01	17	4503171	'SMTNL2	smoothelin-like protein 2 isoform 2	Body;Body	-10572	'LINC01996	long intergenic non-protein coding RNA 1996
GA	cg27207079	3.52E-05	0.008054969	FDR0.01	9	123656751	'PHF19	PHD finger protein 19	Body	423	'PHF19	PHD finger protein 19
GA	cg27218993	1.04E-05	0.003515574	FDR0.01	6	73263351	'			-68219	'KCNQ5	potassium voltage-gated channel subfamily KQT member 5
GA	cg27237537	2.50E-07	0.000238464	FDR0.01	21	45683513	'DNMT3L	DNA (cytosine-5)-methyltransferase 3-like isoform 1	TSS5000;TSS5000	-1733	'DNMT3L	DNA (cytosine-5)-methyltransferase 3-like isoform 1
GA	cg27239243	5.56E-09	1.44E-05	BF|FDR0.01	2	101945867	'			19956	'MIR5696	microRNA 5696
GA	cg27268279	9.85E-07	0.000667688	FDR0.01	10	71672083	'COL13A1	collagen alpha-1(XIII) chain	Body;Body;Body;Body;Body;Bo	110422	'COL13A1	collagen alpha-1(XIII) chain
GA	cg27275941	6.34E-06	0.002516915	FDR0.01	3	15751015	'BTD;ANKRD28	biotinidase precursor;serine/threonine-protein phosphatase 6 regulatory an	Body;Body;Body;Body;Body;Bo	-12137	'MIR3134	microRNA 3134
GA	cg27277978	1.31E-06	0.000820198	FDR0.01	16	30457458	'SEPHS2	selenide, water dikinase 2	TSS1500	-263	'SEPHS2	selenide, water dikinase 2
GA	cg27281690	6.85E-06	0.002649337	FDR0.01	2	7171963	'RNF144A	E3 ubiquitin-protein ligase RNF144A isoform 4	Body;Body;Body;Body;Body;Bo	46048	'LOC101929452	uncharacterized LOC101929452
GA	cg27291385	1.82E-05	0.005277474	FDR0.01	2	219266135	'SLC11A1;CTDSP1;MIR26B	natural resistance-associated macrophage protein 1;carboxy-terminal domai	TES5000;Body;Body;Body;TSS1	-1233	'MIR26B	microRNA 26b
GA	cg27293155	4.25E-06	0.001878223	FDR0.01	2	38044012	'			12842	'LINC00211	long intergenic non-protein coding RNA 211
GA	cg27335649	2.50E-06	0.001286589	FDR0.01	10	10953578	'CELF2	CUGBP Elav-like family member 2	Body;Body;Body;Body;Body;Bo	40548	'LINC00710	long intergenic non-protein coding RNA 710
GA	cg27340749	1.11E-07	0.000130831	BF|FDR0.01	3	48507385	'ATRIP;ATRIP-TREX1;TREX1;SHISA5	ATR-interacting protein;ATRIP-TREX1 readthrough;three-prime repair exonu	TES5000;3'UTR;3'UTR;Body;3'U	157	'TREX1	three-prime repair exonuclease 1 isoform c
GA	cg27347434	4.69E-06	0.002016117	FDR0.01	3	32726203	'CNOT10	CCR4-NOT transcription complex subunit 10 isoform 2	TSS1500;TSS1500;TSS1500	-476	'CNOT10	CCR4-NOT transcription complex subunit 10 isoform 2
GA	cg27366007	4.35E-06	0.001907752	FDR0.01	15	91479184	'HDDC3;UNC45A	guanosine-3',5'-bis(diphosphate) 3'-pyrophosphohydrolase MESH1;protein u	TSS5000;TSS5000;Body;TSS500	670	'UNC45A	protein unc-45 homolog A
GA	cg27372422	3.13E-05	0.007422032	FDR0.01	19	41086247	'SPTBN4;SHKBP1	spectrin beta chain, non-erythrocytic 4;SH3KBP1-binding protein 1	TES5000;Body	3469	'SHKBP1	SH3KBP1-binding protein 1
GA	cg27381584	2.00E-09	7.04E-06	BF|FDR0.01	17	80407749	'CYBC1	uncharacterized protein C17orf62 homolog	TSS200;Body;Body;Body;Body;	-147	'CYBC1	uncharacterized protein C17orf62 homolog
GA	cg27391816	2.46E-06	0.001269693	FDR0.01	1	1672644	'SLC35E2A	solute carrier family 35 member E2A isoform 1	Body;Body	4794	'SLC35E2A	solute carrier family 35 member E2A isoform 1
GA	cg27409771	2.69E-05	0.006763213	FDR0.01	16	10711296	'			-36841	'EMP2	epithelial membrane protein 2 precursor
GA	cg27410601	2.40E-05	0.006287683	FDR0.01	17	76121564	'TMC6	transmembrane channel-like protein 6	Body;Body;Body	3311	'TMC6	transmembrane channel-like protein 6
GA	cg27429749	7.63E-09	1.82E-05	BF|FDR0.01	11	57333612	'UBE2L6	ubiquitin/ISG15-conjugating enzyme E2 L6	Body;Body	1533	'UBE2L6	ubiquitin/ISG15-conjugating enzyme E2 L6
GA	cg27438980	5.43E-07	0.000427022	FDR0.01	15	99195143	'IRAIN;IGF1R	IGF1R antisense imprinted non-protein coding RNA;insulin-like growth factor	TSS1500;Body;Body	-693	'IRAIN	IGF1R antisense imprinted non-protein coding RNA
GA	cg27440283	4.78E-07	0.000388588	FDR0.01	1	1242757	'ACAP3;SNORD167;PUSL1;INTS11	arf-GAP with coiled-coil, ANK repeat and PH domain-containing protein 3;sm	Body;TSS5000;TSS1500;TSS150	553	'ACAP3	arf-GAP with coiled-coil, ANK repeat and PH domain-containing protein 3
GA	cg27448161	1.72E-10	1.15E-06	BF|FDR0.01	12	53472792	'SPRYD3	SPRY domain-containing protein 3	Body	382	'SPRYD3	SPRY domain-containing protein 3
GA	cg27466845	3.50E-06	0.001628968	FDR0.01	11	64397734	'NRXN2	neurexin-2-beta	Body;Body;Body	13283	'NRXN2	neurexin-2-beta

Trait	#cpg	p	fdr	significance	chr	pos.hg19	gene.Illumina	gene.description	locations	dist2TSS	gene.nearest	gene.description
**GA**	cg27475522	2.94E-05	0.00717409	FDR0.01	10	17496930	'ST8SIA6	alpha-2,8-sialyltransferase 8F precursor	TSS1500;TSS1500;TSS1500	-336	'ST8SIA6	alpha-2,8-sialyltransferase 8F precursor
**GA**	cg27478224	1.15E-05	0.003792405	FDR0.01	10	91061115	'IFIT2	interferon-induced protein with tetratricopeptide repeats 2	TSS1500	-687	'IFIT2	interferon-induced protein with tetratricopeptide repeats 2
**GA**	cg27478579	1.33E-07	0.000149935	FDR0.01	2	55497012	'MTIF2	translation initiation factor IF-2, mitochondrial isoform b	TSS1500;TSS1500;TSS1500;TSS1	-628	'MTIF2	translation initiation factor IF-2, mitochondrial isoform b
**GA**	cg27479634	4.66E-05	0.009597224	FDR0.01	12	54070545	'ATP5MC2	ATP synthase F(0) complex subunit C2, mitochondrial precursor	TSS1500;TSS1500;TSS1500;TSS1	-431	'ATP5MC2	ATP synthase F(0) complex subunit C2, mitochondrial precursor
**GA**	cg27488648	2.15E-05	0.005864506	FDR0.01	11	118477034	'ARCN1;PHLDB1	coatomer subunit delta;pleckstrin homology-like domain family B member 1	TES5000;TES5000;TSS200;TSS1	-116	'PHLDB1	pleckstrin homology-like domain family B member 1
**GA**	cg27494111	9.71E-06	0.003354909	FDR0.01	19	12369481	'			35946	'ZNF44	zinc finger protein 44 isoform 2
**GA**	cg27508055	8.28E-06	0.003024832	FDR0.01	2	19286187	'			-59461	'LINC01376	long intergenic non-protein coding RNA 1376
**GA**	cg27513684	6.04E-06	0.002426612	FDR0.01	1	27729053	'WASF2	wiskott-Aldrich syndrome protein family member 2	TES5000;TES5000	9928	'GPR3	G-protein coupled receptor 3
**GA**	cg27514777	3.19E-07	0.000291111	FDR0.01	16	14377664	'			-18480	'MIR193BHG	MIR193b-365a host gene
**GA**	cg27518892	4.83E-05	0.009828595	FDR0.01	16	57566936	'CCDC102A	coiled-coil domain-containing protein 102A	Body	3547	'CCDC102A	coiled-coil domain-containing protein 102A
**GA**	cg27536187	4.39E-05	0.009244415	FDR0.01	1	2159365	'SKI	ski oncogene	TSS1500	-392	'SKI	ski oncogene
**GA**	cg27539234	4.67E-07	0.000384991	FDR0.01	17	7218945	'EIF5A;GPS2;NEURL4	eukaryotic translation initiation factor 5A-1 isoform A;G protein pathway sup	TES5000;TES5000;TES5000;TES	-266	'GPS2	G protein pathway suppressor 2
**GA**	cg27558541	1.48E-05	0.004545404	FDR0.01	1	110350324	'			-43779	'EPS8L3	epidermal growth factor receptor kinase substrate 8-like protein 3 isoform c
**GA**	cg27565899	7.09E-06	0.002709346	FDR0.01	1	110166664	'AMPD2	AMP deaminase 2	Body;Body;Body;Body;5'UTR	55	'AMPD2	AMP deaminase 2
**GA**	cg27574980	8.69E-07	0.00060416	FDR0.01	6	31744636	'VWA7;VARS1	von Willebrand factor A domain-containing protein 7 precursor;valine--tRNA	Body;TES5000	469	'VWA7	von Willebrand factor A domain-containing protein 7 precursor
**GA**	cg27615366	2.42E-05	0.006311797	FDR0.01	5	131592974	'PDLIM4	PDZ and LIM domain protein 4	TSS1500;TSS1500	-376	'PDLIM4	PDZ and LIM domain protein 4
**GA**	cg27640064	2.61E-05	0.006648876	FDR0.01	7	4752983	'FOXK1	forkhead box protein K1	Body	31058	'FOXK1	forkhead box protein K1
**GA**	cg27659768	1.25E-05	0.00405281	FDR0.01	16	71523436	'ZNF19	zinc finger protein 19	TSS1500	-209	'ZNF19	zinc finger protein 19
**BW**	cg24399376	3.14E-09	0.001405879	BF|FDR0.01	11	2005497	'MRPL23-AS1	MRPL23 antisense RNA 1	Body	5653	'MRPL23-AS1	MRPL23 antisense RNA 1
**BW**	cg26866325	8.74E-08	0.013540645	BF|FDR0.01	4	74920559	'PPBPP2	pro-platelet basic protein pseudogene 2	Body	557	'PPBPP2	pro-platelet basic protein pseudogene 2
**BW**	cg08821669	9.08E-08	0.013540645	BF|FDR0.01	12	120875978	'COX6A1	cytochrome c oxidase subunit 6A1, mitochondrial	1stExon	63	'COX6A1	cytochrome c oxidase subunit 6A1, mitochondrial
