# Supplementary material for: Epigenome-wide association study of bronchopulmonary dysplasia in preterm infants: results from the discovery-BPD program
Source: Clin Epigenetics. 2022 Apr 28;14:57. doi: 10.1186/s13148-022-01272-0 (PMC9052529; doi:10.1186/s13148-022-01272-0)
Supplement: Supplementary file 2 — Additional file 2. Figure S1. DNA methylation of aryl-hydrocarbon receptor repressor (AHRR) CpG (cg23953254) shows minimal association NRBC or smoking. Figure S2. Epigenetic estimation of gestational age (GA) and GA acceleration in bronchopulmonary dysplasia (BPD). Figure S3. Expression of bronchopulmonary dysplasia (BPD) epigenome-associated genes in mouse lung tissues. [file 13148_2022_1272_MOESM2_ESM.pdf]

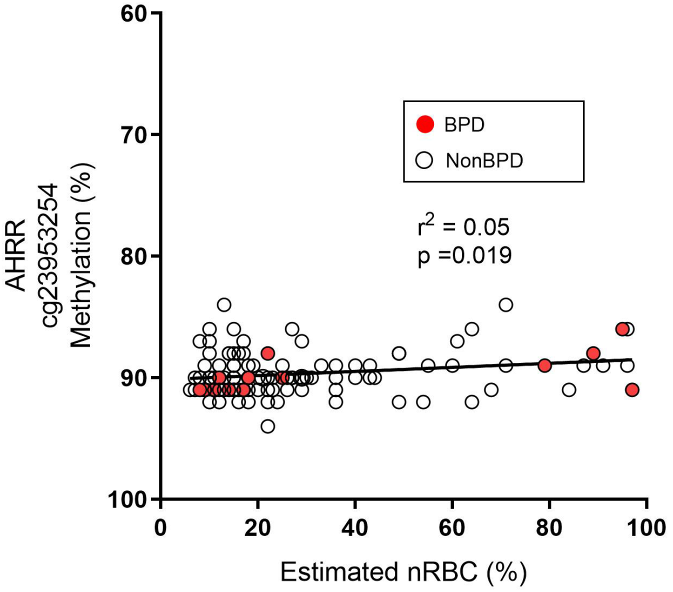

Figure S1. DNA methylation of aryl hydrocarbon receptor repressor (*AHRR*) CpG (cg23953254) not associated with smoking. This CpG shows only slight association with estimated nucleated red blood cell (NRBC) contents in preterm infants with or without bronchopulmonary dysplasia (BPD) indicating smoking effect (shown in Figure 2E) is independent of demethylation of NRBC genomes**.**

Figure S2

A

	GA Recorded	EGA Bohlin Method	EGA Knight Method
**BPD (Weeks)**	27.6 ± 2.5	33.7 ± 3.4	31.4 ± 5.8
**nonBPD (Weeks)**	30.2 ± 2.0	34.2 ± 1.7	32.3 ± 2.3

B

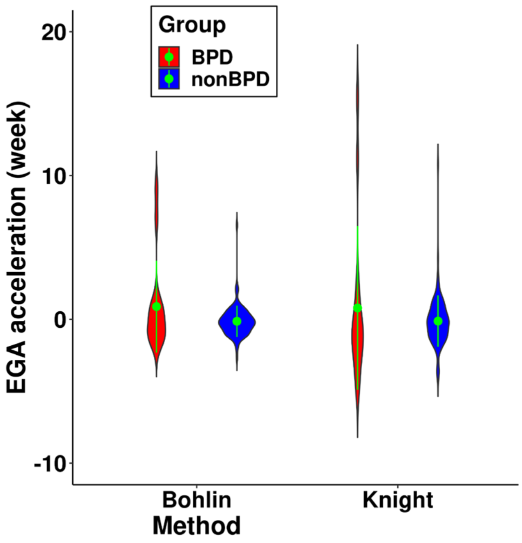

	Bohlin	Knight
**BPD**	0.89 ± 3.20	0.80 ± 5.69
**nonBPD**	-0.13 ± 1.08	-0.12 ± 1.79
p	0.020	0.22
***p* adjusted**	0.033	0.17

Figure S2. Epigenetic estimation of gestational age (GA) and GA acceleration in bronchopulmonary dysplasia (BPD). (A) Recorded gestational age (GA) and predicted epigenetic GA (EGA) in preterm infants with or without BPD using published methods [26, 28]. (B) EGA acceleration in preterm infants with or without BPD. In violin-plot, green dot and bar show mean and standard deviation. Higher EGA acceleration (estimated by linear regression EGA on GA) in BPD samples than in nonBPD samples with Bohlin model [26] but not Knight [28] with or without adjustment for epigenome-wide association study (EWAS) covariates.

Figure S3

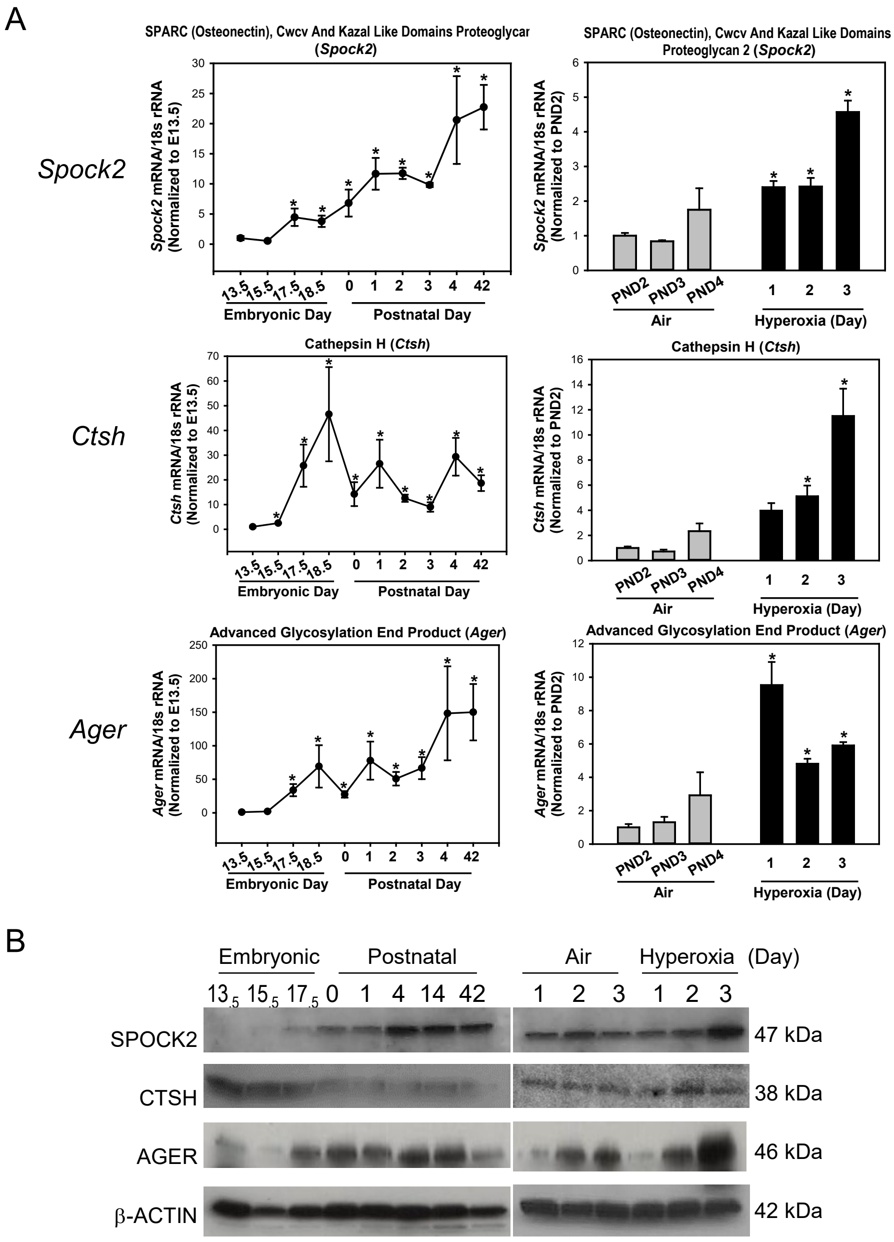

Figure S3. Expression of bronchopulmonary dysplasia (BPD) epigenome-associated genes in mouse lung tissues. qRT-PCR and Western blot analyses determined mRNA (A) and protein (B) expression of SPARC (Osteonectin), Cwcv and Kazal like domains proteoglycan 2 (SPOCK2), cathepsin H (CTSH), and advanced glycosylation end-product specific receptor (AGER) associated with BPD epigenome in mouse lungs during saccular-to-alveolar lung development stages and in hyperoxia model of murine BPD. qRT-PCR Data presented as group mean ± S.E.M. (n = 3/group). *, vs embryonic day 13.5 or time-matched air controls (p < 0.05). Representative blot images from duplicate or triplicate blot analyses presented. kDa = kilodalton.
