# Supplementary material for: Epigenome-wide association study of bronchopulmonary dysplasia in preterm infants: results from the discovery-BPD program
Source: Clin Epigenetics. 2022 Apr 28;14:57. doi: 10.1186/s13148-022-01272-0 (PMC9052529; doi:10.1186/s13148-022-01272-0)
Supplement: Supplementary file 3 — Additional file 3. Western Blot Raw Images [file 13148_2022_1272_MOESM3_ESM.pdf]

Figure S3B: SPOCK2 Western blotting

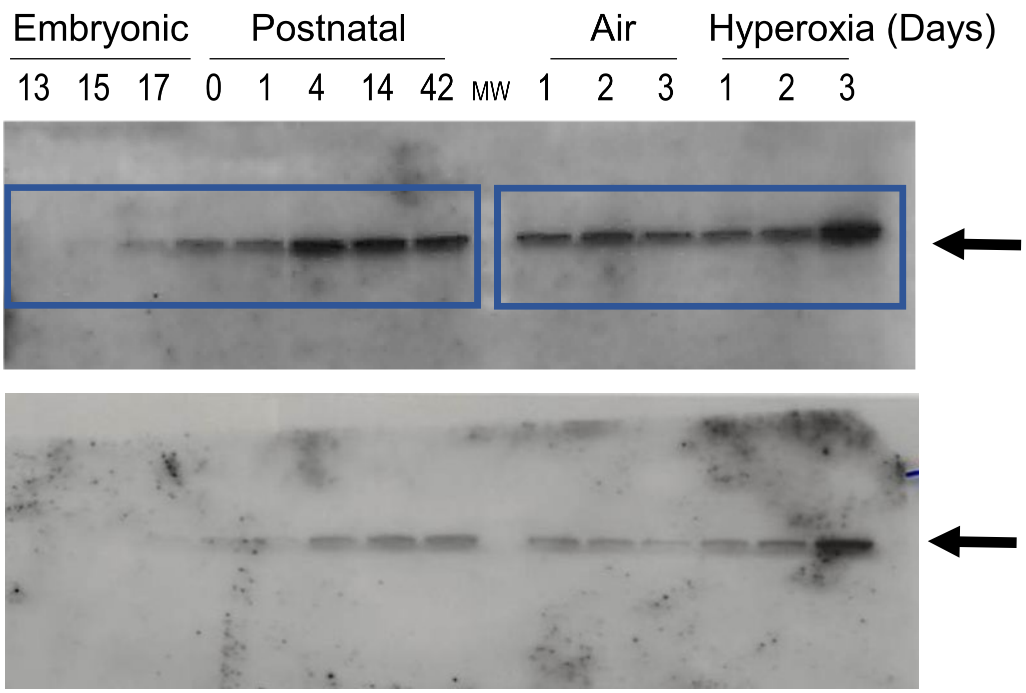

Images in manuscript

Antibody- R&D Systems AF2328

1:200 dilution (overnight at 4°C, in 5% milk)

1:1000 secondary anti-goat HRP (1 hr at room temp. in 5% milk)

Detected - approximately at 47 kDa

Expected size: 60-140 kDa (splice variants)

Figure S3B: CTSH Western blotting

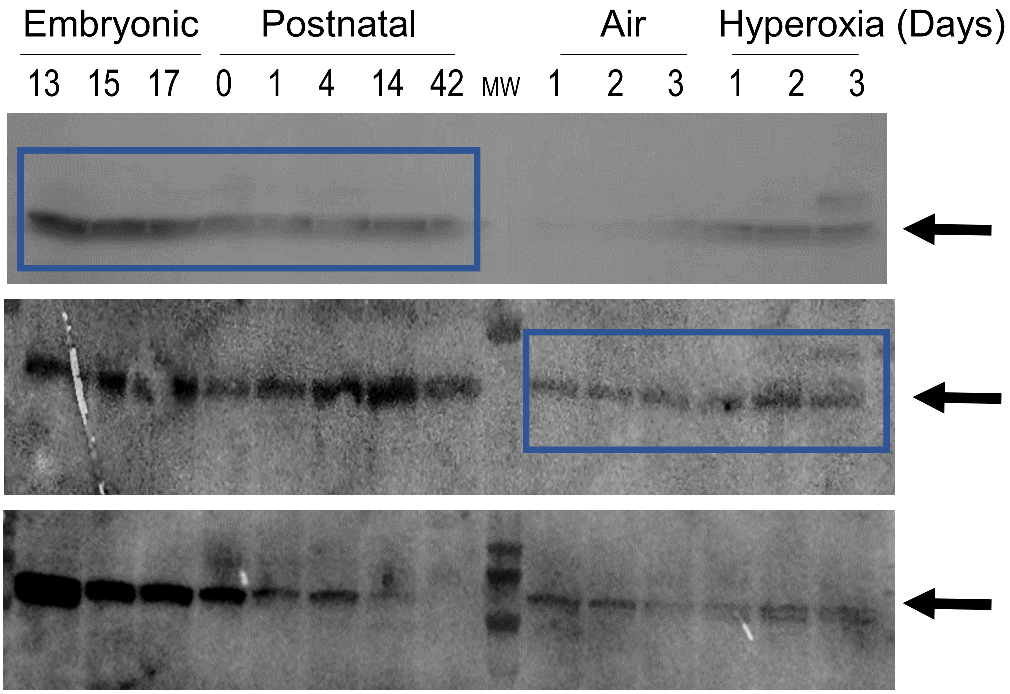

Images in manuscript

Antibody - LS Bio LS-C353958

1:400 (overnight at 4°C, in 5% milk)

1:2000 secondary anti-rabbit HRP (1 hr at room temp. in 5% milk)

Detected - approximately at 38 kDa

Expected size: 37-50 kDa (splice variants)

Figure S3B: AGER Western blotting

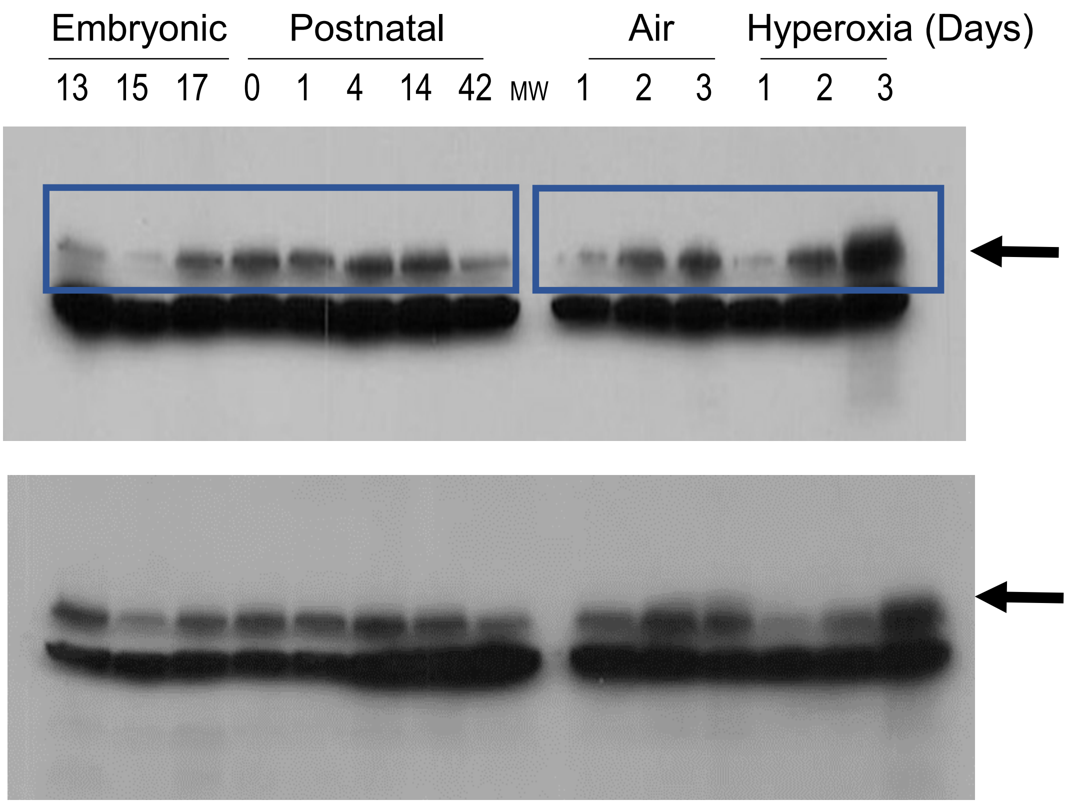

Images in manuscript

Antibody- Santa Cruz (sc-365154)

1:500 (overnight at 4°C, in 5% milk)

1:2000 secondary anti-mouse G1 HRP (1 hr at room temp. in 5% milk)

Detected - approximately at 46 kDa

Expected size: 46 kDa

Figure S3B: β-Actin Western blotting

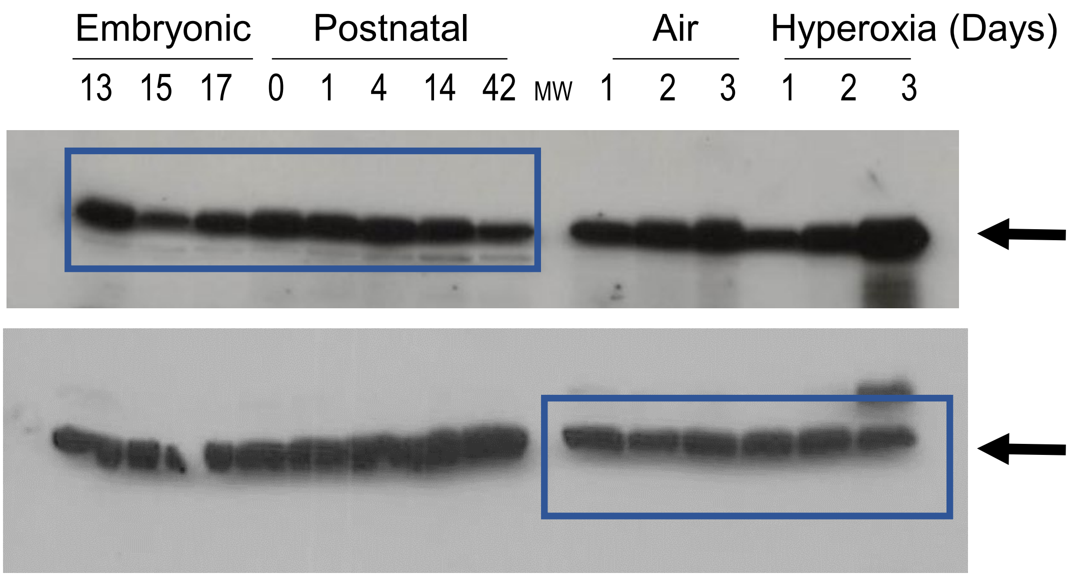

Images in manuscript

Antibody- Santa Cruz (sc-47778)
1:500 (overnight at 4°C, in 5% milk)
1:1500 secondary anti-mouse G1 HRP (1 hr at room temp. in 5% milk)

Detected - approximately at 42 kDa

Expected size: 42 kDa
